# HIV-associated gut microbial alterations are dependent on host and geographic context

**DOI:** 10.1038/s41467-023-44566-4

**Published:** 2024-02-05

**Authors:** Muntsa Rocafort, David B. Gootenberg, Jesús M. Luévano, Jeffrey M. Paer, Matthew R. Hayward, Juliet T. Bramante, Musie S. Ghebremichael, Jiawu Xu, Zoe H. Rogers, Alexander R. Munoz, Samson Okello, June-Ho Kim, Ruth Sentongo, Robert Wagubi, Alex Lankowski, Segametsi Maruapula, Guoyan Zhao, Scott A. Handley, Mosepele Mosepele, Mark J. Siedner, Douglas S. Kwon

**Affiliations:** 1 Ragon Institute of MGH, MIT, and Harvard, Cambridge, MA 02139 USA; 2 Harvard Medical School, Boston, MA 02114 USA; 3 Department of Medicine, Mbarara University of Science and Technology, 1956 Mbarara, Uganda; 4 Medical Practice Evaluation Center, Massachusetts General Hospital, Boston, MA 02114 USA; 5 Department of Family & Consumer Sciences, University of Botswana, 0022 Gaborone, Botswana; 6 Department of Pathology and Immunology, Washington University School of Medicine, St. Louis, MO 63110 USA; 7 Faculty of Medicine, University of Botswana, 0022 Gaborone, Botswana; 8 Division of Infectious Diseases, Massachusetts General Hospital, Boston, MA 02114 USA

**Keywords:** HIV infections, Metagenomics

## Abstract

HIV-associated changes in intestinal microbiota are believed to be important drivers of disease progression. However, the majority of studies have focused on populations in high-income countries rather than in developing regions where HIV burden is greatest. To better understand the impact of HIV on fecal microbiota globally, we compare the fecal microbial community of individuals in the U.S., Uganda, and Botswana. We identify significant bacterial taxa alterations with both treated and untreated HIV infection with a high degree of uniqueness in each cohort. HIV-associated taxa alterations are also significantly different between populations that report men who have sex with men (MSM) behavior and non-MSM populations. Additionally, while we find that HIV infection is consistently associated with higher soluble markers of immune activation, most specific bacterial taxa associated with these markers in each region are not shared and none are shared across all three geographic locations in our study. Our findings demonstrate that HIV-associated changes in fecal microbiota are overall distinct among geographical locations and sexual behavior groups, although a small number of taxa shared between pairs of geographic locations warrant further investigation, highlighting the importance of considering host context to fully assess the impact of the gut microbiome on human health and disease.

## Abstract (web summary)

Here, the authors compare the fecal microbial community of individuals in the U.S., Uganda, and Botswana, and identify significant bacterial taxa alterations with both treated and untreated HIV infection although with a high degree of uniqueness in each cohort, and also significant differences between populations that report men who have sex with men (MSM) behavior and non-MSM populations.

## Introduction

Intestinal microbiota are known to be crucial for gut homeostasis and are disrupted in a number of human diseases, including human immunodeficiency virus type 1 (HIV) infection^1–5^. While the introduction of combination antiretroviral treatment (ART) has improved the life expectancy of people living with HIV, there continues to be significant morbidity and mortality due to inflammation-driven diseases such as stroke, long bone fractures, and cardiovascular disease^6,7^. Although systemic immune activation declines after initiation of ART, it remains persistently elevated even after years of effective treatment^8,9^. The factors contributing to this residual immune activation are not fully understood, but it has been suggested that alterations in intestinal microbiota and impaired local tissue barrier function may play a crucial role^10,11^.

A hallmark of HIV infection is intestinal inflammation, which has been shown to result in epithelial barrier disruption and translocation of luminal microbial products into the systemic circulation^12,13^. This microbial translocation is thought to drive chronic immune activation, eventually resulting in T cell exhaustion and HIV disease progression^14–16^. Several studies of HIV-associated alterations in the fecal microbiome have reported a significant reduction in bacterial richness and diversity and increased relative abundance of Proteobacteria, which is believed to be more inflammatory and thus a significant contributor to systemic inflammation^4,17^. However, these studies have predominantly been performed with populations in high-income countries with relatively low HIV prevalence, as opposed to low-income countries in sub-Saharan Africa, where the HIV burden is greatest. In addition, the potential confounding by the known impact of men who have sex with men (MSM) behavior on fecal microbial community structure has not been considered in a number of studies.

Here we examine the effect of HIV infection on fecal bacterial microbiota in three distinct geographic locations, including a cohort in the U.S. with low HIV prevalence and two in sub-Saharan Africa with high HIV prevalence. We also include a cohort of individuals in the U.S. reporting active MSM sexual behavior. Our study confirms prior results showing baseline differences in fecal microbial communities of HIV-uninfected individuals in each region. We further show significant microbial changes associated with both treated and untreated HIV infection in each region with a high degree of uniqueness and few shared disease-associated taxa between cohorts. Despite the unique microbial signatures associated with HIV infection in each location, we find that all individuals with HIV had increased markers of immune activation often associated with microbial translocation. The specific bacterial taxa associated with these markers in each region were generally not shared, with no taxa shared between all three geographic cohorts. We also demonstrate that in our U.S. cohort, HIV-associated microbial differences, including significant increases in *Prevotella* species, are greater in magnitude in MSM than non-MSM populations. Overall, our findings demonstrate that HIV-associated changes in fecal microbiota are distinct when comparing those in the U.S. and sub-Saharan Africa, as well as individuals who report MSM behavior. These results highlight the need to consider host and geographical context more broadly and emphasize the importance of studying different populations to fully appreciate the impact of gut microbiota on human health and disease.

## Results

### HIV-uninfected non-MSM individuals have distinct fecal microbiota baselines in the U.S. and sub-Saharan Africa

Geography is associated with significant variation in gut microbial community structure^18–20^. Recent findings indicate that income and urbanization are significant factors underlying these geographic differences^21,22^. We therefore sought to understand the impact of HIV infection on fecal microbiota in distinct regions. We collected samples from three cohorts spanning two continents with diverse demographic and socioeconomic characteristics, including areas that are urban, high income, with low HIV prevalence (Boston, MA, U.S., *n* = 233); rural, low income, with high HIV prevalence (Mbarara District, Uganda, *n* = 170)^23,24^; and mixed urban-rural, low income, with high HIV prevalence (Gaborone, Botswana, *n* = 194)^25^ (Table 1). We characterized the fecal microbiota of these subjects using high-throughput sequencing of the V4 region of the 16S bacterial rRNA gene.

**Table 1:** Demographic characteristics of individuals in cohorts from the U.S., Botswana and Uganda

Demographic	Boston, US (*n* = 233)	Gaborone, Botswana (*n* = 194)	Mbarara, Uganda (*n* = 170)	*p* value
Geographical demographics				
Population density per km^2^ Gross National Income in $USD HIV prevalence in adults aged 15–45 per 100,000 population (IQR)	5538 $52,620 0.4 (0.3–0.5)	1400 $14,630 23.2 (20.9–24.8)	314 $1320 6.8 (6.5–7.1)	– – –
Sex, female/male (male MSM/male non-MSM [Boston])	53/180 (118/62)	96/98	85/85	<0.01
Race (Black or African-American/White/American Indian or Alaskan Native/Asian/Multiple/Unknown)	49/161/2/7/12/2	194/0/0/0/0/0	170/0/0/0/0/0	<0.01
Ethnicity (non-Hispanic or Latino/Hispanic or Latino)	209/24	194/0	170/0	<0.01
Age in years (IQR)	51 (37, 55)	38 (34, 41.75)	49 (46, 52)	<0.01
HIV-1 infection status (female/male MSM/male non-MSM/male [MSM unknown])				
Negative On ART ART-naïve	117 (50.2%) (37/32/48/0) 61 (26.2%) (9/42/10/0) 55 (23.6%) (7/44/4/0)	80 (41.3%) (36/0/0/44) 73 (37.6%) (38/0/0/35) 41 (21.1%) (22/0/0/19)	80 (47%) (39/0/0/41) 90 (53%) (46/0/0/44) –	<0.01
Time on ART (years, IQR)	8.87 (4.20, 14.00)	8.98 (6.92, 10.81)	6.99 (6.38, 7.64)	<0.01
ART regimen (NNRTI/PI/ISTI/2_agent)	19/14/25/3	52/18/0/3	81/9/0/0	<0.01
Weight in kg (IQR) (n by negative/ART/ART-naïve)	82 (72, 92) *n* = 208 (43/108/57)	65 (57, 77)	59 (53, 66)	<0.01
Height in cm (IQR) (n by negative/ART/ART-naïve)	175 (170, 180) *n* = 206 (42/107/57)	166 (161, 173)	162 (157, 169)	<0.01
Body mass index (BMI) in kg/cm^2^ (IQR) (n by negative/ART/ART-naïve)	26.6 (23.5, 30) *n* = 205 (42/106/57)	23.1 (20.3, 27.7)	21.8 (19.7, 24.8)	<0.01
Taking TMP-SMX (yes/no)	2 (both ART-naive, MSM)/231	0/194	81 (all ART)/89 (including 9 on ART)	<0.01
History or diagnosis of comorbidity (*n*, %) (n by negative/ART/ART-naïve, total *n* = 200 for Boston)				
Hyperlipidemia Hypertension Cardiovascular disease Diabetes	32 (16) (8/18/6) 37 (18.5) (8/19/10) 8 (4) (4/3/1) 12 (6) (6/4/2)	56 (28.9) (22/28/6) 21 (10.8) (9/11/1) 10 (5.2) (3/3/4) 8 (4.1) (4/1/3)	25 (14.7) (14/11/0) 37 (21.8) (22/15/0) 49 (28.8) (25/24/0) 12 (7.1) (7/5/0)	<0.01 0.015 <0.01 0.4691
Current CD4 T-cell counts in cells/mL (IQR)				
On ART (Negative/ART/ART-naïve) ART-naïve (Negative/ART/ART-naïve)	722 (507.2, 918) *n* = 60 (9/41/10) 548 (440.8, 775) *n* = 54 (7/43/4)	493 (389, 694) 361 (158, 531)	430 (336, 552.8) –	<0.01 <0.01
Nadir CD4 T-cell counts in cells/mL (IQR)				
On ART (Negative/ART/ART-naïve) ART-naïve (Negative/ART/ART-naïve)	361.5 (276.5, 583) *n* = 52 (7/39/6) 402 (347.5, 557) *n* = 50 (6/41/3)	127.5 (73.3, 180) –	123 (85.3, 173.3) –	<0.01 –

For numeric variables, median and IQR (interquartile range) values are shown. *P* values for continuous and discrete variables are calculated with ANOVA and *χ*^2^ test, respectively. For the geographical demographic information, population density is calculated by city and gross national income and HIV prevalence in adults by country.

*MSM* men who have sex with men, *NNRTI* ART regimen containing 2 Nucleoside Reverse Transcriptase Inhibitors (NRTI) and a Non-Nucleoside Reverse Transcriptase Inhibitors (NNRTI), *PI* ART regimen containing 2 NRTI and a Protease Inhibitor (PI), *ISTI* ART regimen containing 2 NRTI and an Integrase Strand Transfer Inhibitor, *2_agent* ART regimen containing only 2 agents (either 2 NRTI, an NRTI and an NNRTI, or an NRTI and a PI), *TMP-SMX* trimethoprim-sulfamethoxazole.

**Fig. 1:**
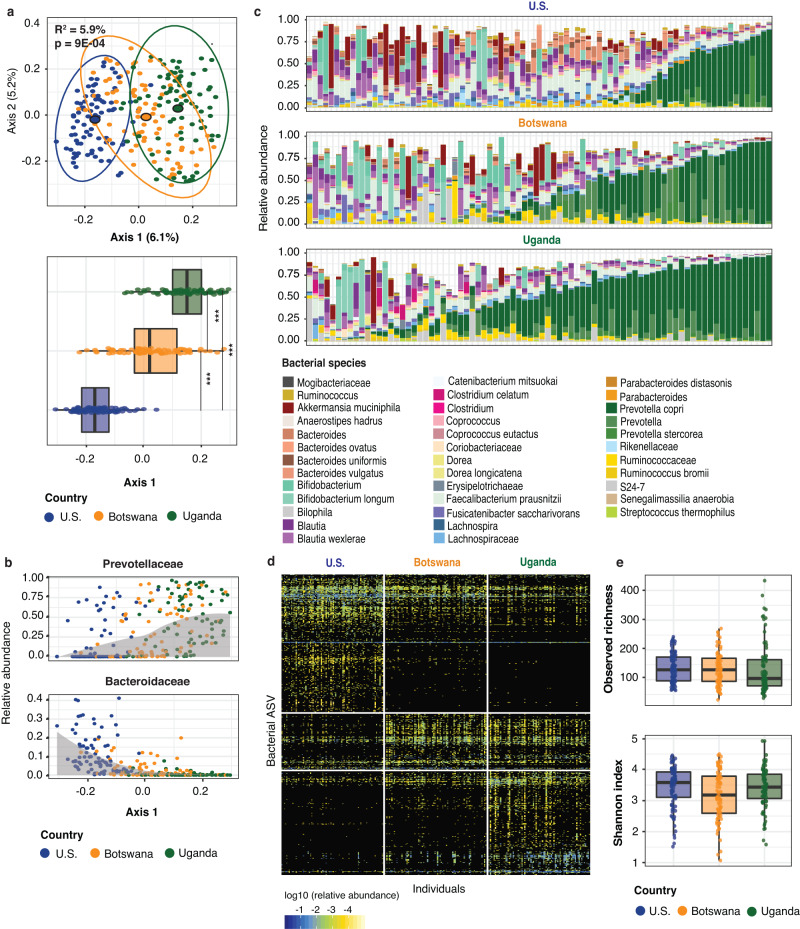
HIV-uninfected non-MSM individuals have a distinct fecal microbiota composition in the U.S., Botswana and Uganda. **a** PCoA showing unweighted UniFrac distances calculated at the ASV (amplicon sequence variant) level among all non-MSM (men who have sex with men) HIV-uninfected individuals. The boxplot represents the distribution of coordinates in Axis 1 of the PCoA above for the three cohorts. Boxplots are displayed with the median as the center value with the box as IQR and whiskers as minima and maxima. For the ordination plot, *R*^2^ and *p* value were obtained from a PERMANOVA analysis accounting for geographical location (*n* = 245 biologically independent samples, *F*(df = 2) = 7.662, *p* = 0.0009, *R*^2^ = 5.9%), and for the boxplot below, from a Kruskal–Wallis test with Bonferroni correction (***p value < 0.001); *χ*^2^(df = 2) = 234.0, *p* < 0.0001; pairwise comparisons: Botswana – U.S. *Z* = 9.53 (*p* < 0.0001), Uganda – U.S. *Z* = 12.94 (*p* < 0.0001), Botswana – Uganda *Z* = 8.13 (*p* < 0.0001). **b** Relative abundance of ASVs in the families Prevotellaceae and Bacteroidaceae. Each dot represents the relative abundance of a given sample and samples are plotted from left to right based on their position on Axis 1 from the PCoA in subfigure (**a**). A local regression (gray) shows the relation between the bacterial families’ relative abundance and the samples’ distribution on Axis 1. These families were significantly different in relative abundance amongst all cohorts as measured by Kruskal–Wallis test with Bonferroni correction (Prevotellaceae: *χ*^2^(df = 2) = 52.0, *p* < 0.001; Bacteroidaceae: *χ*^2^(df = 2) = 131.24, *p* < 0.001). **c** Relative abundance of the bacterial species with at least 1 count in 50% of the samples that show significant differential abundances in HIV-uninfected individuals between cohorts based on a Kruskal–Wallis test with Bonferroni multiple comparison correction (threshold *p* value < 0.01). Bacterial taxa are identified to the most precise taxonomic resolution. **d** Heatmap showing differentially abundant bacterial ASVs between the three cohorts (Kruskal–Wallis test, threshold *p* value < 0.01). Color gradient represents the relative abundance of each taxon in each individual. ASV in the *y*-axis and individuals in the *x*-axis are ordered based on an average clustering using Bray Curtis distance. ASVs are additionally organized and split based on the abundance in the U.S., Botswana or Uganda. **e** Boxplots showing the observed richness and Shannon diversity metrics for the HIV-uninfected individuals (*n* = 245 biologically independent samples) in the three countries. Boxplots are displayed with the median as the center value with the box as IQR and whiskers as minima and maxima.

To establish baseline geographically associated microbiota differences without the additional influence of HIV infection or MSM behavior, we compared the fecal microbiota of 245 HIV-uninfected subjects, excluding subjects who reported MSM behavior, across the three locations. As expected from the differences in urbanization and income, fecal microbiota of HIV-uninfected non-MSM individuals was distinct in each location (Fig. 1a) with a significant effect of geographic location on microbiota composition (*F*(df = 2) = 7.662, *p* = 0.0009, *R*^2^ = 5.9%). When controlling for metadata available for all subjects (*n* = 245; race, ethnicity, age, and sex), this analysis was still significant (*F*(df = 2) = 3.303, *p* = 0.0009, *R*^2^ = 2.6%). The finding was robust with a similar effect size when controlling for metadata available for a subset of subjects (*n* = 231; BMI, history or diagnosis of diabetes, hyperlipidemia, cardiovascular disease, or hypertension; *F*(df = 2) = 2.709, *p* = 0.0009, *R*^2^ = 2.2%) (Supplementary Table 1). The U.S. and Ugandan cohorts had the most dissimilar fecal communities, while the Botswanan cohort overlapped with both other cohorts.

Two bacterial families, Prevotellaceae and Bacteroidaceae, were found to be consistently and significantly different in relative abundance (Prevotellaceae: *χ*^2^(df = 2) = 52.0, *p* < 0.001; Bacteroidaceae: *χ*^2^(df = 2) = 131.24, *p* < 0.001) amongst all cohorts, with an increasing proportion of Prevotellaceae in more rural communities and greater abundance of Bacteroidaceae in urban regions (Fig. 1b). This finding was still observed when controlling for the aforementioned metadata variables, except in the comparison of Prevotellaceae abundance between Botswana and Boston. Single-sex subset analyses, performed to remove potential confounding from incomplete data regarding sexual behavior in some cohorts, recapitulated findings, including that HIV-infected/non-MSM individuals cluster significantly by geography with an *R*^2^ of approximately 6% (female-only *F*(df = 2) = 4.00, *p* = 0.0009, *R*^2^ = 6.8%; male-only *F*(df = 2) = 4.722, *p* = 0.0009, *R*^2^ = 6.7%). These findings remain significant after controlling for available metadata (Supplementary Figs. 1a, b and 2a, b and Supplementary Tables 2 and 3). The Prevotellaceae vs Bacteroidaceae continuum was also observed between geographic locations in both male-only and female-only analyses (Supplementary Figs. 1b and 2b) with significantly different proportions between geographic locations (female-only *χ*^2^(df = 2) = 76.0, *p* < 0.0001; male-only *χ*^2^(df = 2) = 134.2, *p* < 0.0001). We further identified 37 bacterial species with a significant differential abundance in HIV-uninfected non-MSM individuals between cohorts (Fig. 1c). In all cohorts, *Prevotella copri* (dark green) remained the most abundant bacterial species and was higher in Uganda (37.3%, mean relative abundance) and Botswana (19.2%), than in the U.S. (14.3%) (Supplementary Table 4).

To further characterize microbial community differences, we identified 638 amplicon sequence variants (ASVs) that were differentially abundant between the cohorts (21% of the 3035 ASVs identified in all HIV-uninfected non-MSM individuals; Fig. 1d). ASVs each represent unique 16S gene amplicons and can detect dissimilarity in microbial composition between populations that may not be recognized when examining microbial abundance at the species or higher taxonomic ranks^26^. Of these ASVs, 568 (89%) were different between the U.S. and Ugandan cohorts, 277 (43.4%) between Botswanan and Ugandan cohorts, and 385 (60.3%) between the U.S. and Botswanan cohorts, consistent with subjects in U.S. and Uganda having the most dissimilar fecal microbial communities. There was no difference for observed richness or Shannon diversity index between geographical locations (Fig. 1e).

Overall, these findings demonstrate significant geographic differences in the baseline fecal microbiota of HIV-uninfected non-MSM individuals that remain when controlling for metadata representing potential confounding variables. These findings also support the importance of *Prevotella* species, particularly in those living in rural/low-income countries^18,19^. In addition, we demonstrate that the U.S. (high income, urban) and Ugandan (low income, rural) cohorts show the greatest differences in fecal microbiota composition in people without HIV, and that the Botswanan (low income, mixed urban/rural) cohort occupies an intermediate position between these cohorts.

### HIV infection is associated with unique fecal microbiota alterations in the U.S. and sub-Saharan Africa

Having characterized the fecal microbiota from HIV-uninfected non-MSM individuals in each of our cohorts, we then evaluated differences in microbiota associated with HIV infection. Previous studies indicate that MSM behavior is associated with distinct gut bacterial communities^27,28^. We therefore initially considered the impact of HIV infection independent of MSM behavior in each geographic cohort by excluding MSM-reporting subjects.

**Fig. 2:**
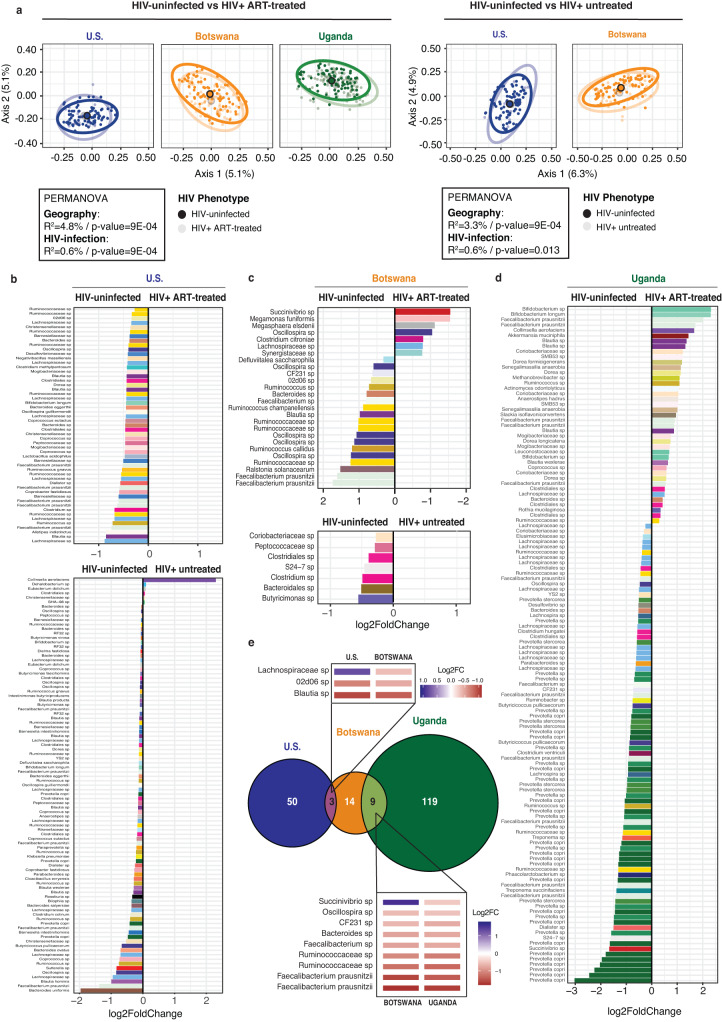
Geography-specific HIV-associated fecal microbial alterations. **a** PCoA ordination plots using unweighted UniFrac distance at the ASV (amplicon sequence variant) level among all non-MSM (men who have sex with men) HIV-uninfected and ART (antiretroviral)-treated HIV-infected subjects (left) and HIV-uninfected and untreated HIV-infected subjects (right). *R*^2^ and *p* value were obtained from a PERMANOVA analysis accounting for either geographical location or HIV infection status. HIV-uninfected vs HIV + ART-treated (*n* = 427 biologically independent samples): geography *F*(df = 2) = 10.78, *p* = 0.0009, *R*^2^ = 4.8%; and HIV infection *F*(df = 2) = 2.94, *p* = 0.0009, *R*^2^ = 0.6%. HIV-uninfected vs HIV+ untreated (*n* = 217 biologically independent samples): geography *F*(df = 2) = 7.56, *p* = 0.0009, *R*^2^ = 3.3%; and HIV infection *F*(df = 2) = 1.44, *p* = 0.007, *R*^2^ = 0.6%. **b** Results from an ANCOM analysis testing for differences of both ART-treated and -untreated HIV infection compared to the HIV-uninfected controls in the U.S. Only ASVs present in at least 5% of the samples in each HIV infection group were considered. The Log2FC values are represented on the *x*-axis for all those single ASVs with significantly different abundance in each of the tested groups (threshold *p* value < 0.05). Color represents taxonomic classification and ASVs are identified to the taxonomic level of greatest resolution. Multiple ASVs can be assigned to the same taxonomy and therefore the same taxonomic name and coloring may appear more than once in the analysis. The same analyses were conducted for the Botswanan (**c**) and Ugandan cohorts (**d**). **e** Venn diagram showing the proportion of unique and shared differentially abundant ASVs between ART-treated HIV-infected and HIV-uninfected individuals among the cohorts. The heatmap indicates the 9 shared ASVs among Botswana and Uganda as well as the 3 shared ASVs among Botswana and the U.S. and their Log2FC in abundance calculated from ANCOM analysis.

When considering all geographic cohorts together, we observed that HIV infection had a significant impact on the fecal microbiota composition, but that geographical location had a greater effect than HIV infection status, regardless of whether the HIV-infected subjects were on ART (Fig. 2a; PERMANOVA; geography *F*(df = 2) = 10.78, *p* = 0.0009, *R*^2^ = 4.8%; and HIV infection *F*(df = 2) = 2.94, *p* = 0.0009, *R*^2^ = 0.6% for ART-treated subjects; geography *F*(df = 2) = 7.56, *p* = 0.0009, *R*^2^ = 3.3% and HIV infection *F*(df = 2) = 1.44, *p* = 0.007, *R*^2^ = 0.6% for untreated subjects). These findings were replicated in single-sex analysis with HIV infection status significantly associated with fecal microbiota composition in all cases except for the comparison of HIV-uninfected and HIV-infected untreated female subjects, although these single-sex analysis subgroups were small (Supplementary Figs. 1c and 2c). The significant impact of HIV infection was robust to controlling with all available metadata in the comparison between the HIV-uninfected and HIV-infected ART-treated populations in both the combined and single-sex analysis, and was also robust to controlling with metadata available for all subjects in the comparison between HIV-uninfected and HIV-infected ART-untreated populations in the combined analysis (Fig. 2a, Supplementary Figs. 1c and 2c and Supplementary Tables 5–7).

We next tested the effect of HIV infection in each cohort separately. When directly comparing groups of different HIV infection status within the same geography, we detected significant differences in overall composition between ART-treated HIV-infected individuals and HIV-uninfected subjects in Botswana and Uganda, and these significant differences remained after controlling for all available metadata (Supplementary Fig. 3a and Supplementary Tables 8–13). Shannon diversity index was significantly reduced with HIV infection in the Ugandan cohort (Supplementary Fig. 3b), and this reduction as well as a significant reduction in observed richness was observed in the female-only subset analysis of the Ugandan cohort (Supplementary Fig. 1d). In all other cohorts and single-sex subset analyses, there were no significant differences in observed richness and Shannon diversity index with HIV infection (Supplementary Figs. 3b, 1d, and 2d). The significant differences in microbiota composition were present despite almost no significant differences in dietary intake within each geographic location, as measured by differences in macro- and micronutrient content of food from a 24-h recall questionnaire (Supplementary Fig. 4).

We next sought to identify specific bacterial ASVs that were differentially abundant in HIV-infected individuals compared to the corresponding HIV-uninfected controls in each cohort using analysis of the composition of microbiomes (ANCOM), which is well-suited to the compositional nature of microbial count data^29,30^. In the U.S. cohort, we identified 53 ASVs that were decreased with ART-treated HIV infection compared to the uninfected controls, and 6 and 87 ASVs that were increased and decreased respectively with untreated HIV infection (Fig. 2b). From the 50 less abundant ASVs with ART-treated HIV infection, 24 were also found to be less abundant in untreated individuals with similar magnitude of differential abundance (Supplementary Fig. 5a). A comparison between both HIV-infected groups in the U.S. identified 132 differentially abundant ASVs, indicating that ART-treated infection is associated with multiple unique taxa relative to untreated HIV infection (Supplementary Fig. 5b).

In Botswana, HIV infection was associated with 26 differentially abundant ASVs in ART-treated HIV infection, and 7 in untreated infection (Fig. 2c). We did not identify shared ASVs with lower abundance in both ART-treated and -untreated HIV infection compared to the HIV-uninfected controls. A total of 13 ASVs were identified with a differential relative abundance between the ART-treated and -untreated HIV-infected groups in this cohort (Supplementary Fig. 5c).

A comparison between ART-treated HIV-infected and HIV-uninfected subjects in Uganda showed the largest number of differentially abundant ASVs, with 128 (Fig. 2d). Of note, multiple taxa were in lower abundance in ART-treated HIV-infected individuals compared to the HIV-uninfected subjects, with 47% (41 of 87) of the ASVs matching taxa within genus *Prevotella*. Thus, *Prevotella* species were not only a major component of the Ugandan HIV-uninfected microbiota but their abundance was also significantly reduced with treated HIV infection.

Overall, we observed a relative uniqueness in the HIV-associated microbial changes in each geographical location, with few shared taxa associated with treated HIV infection between the cohorts and no taxa shared among all three cohorts (Fig. 2e). Botswana and Uganda shared 9 ASVs with overall agreement in the direction and magnitude of differences in these ASVs except for an ASV matching genus *Succinivibrio* that was more abundant with treated HIV in Botswana and less abundant in Uganda. The U.S. and Botswanan cohorts shared 3 ASVs, of which 2 ASVs had agreement in the direction and magnitude of differences and a single ASV matching genus *Lachnospiraceae* which was less abundant with treated HIV in the U.S. and more abundant in Botswana. The observed region-specific responses remained when collapsing at the species and genus levels (Supplementary Fig. 6), as well as for ART-untreated HIV infection and in the comparison between ART-treated and -untreated HIV infection (Supplementary Fig. 5). These findings demonstrate significant changes in intestinal microbiota with both ART-treated and -untreated HIV infection which are unique to each geographic location with few shared HIV-associated taxa between geographic locations and no shared taxa among all three locations.

### HIV-associated microbial changes are more pronounced in those who report MSM behavior

**Fig. 3:**
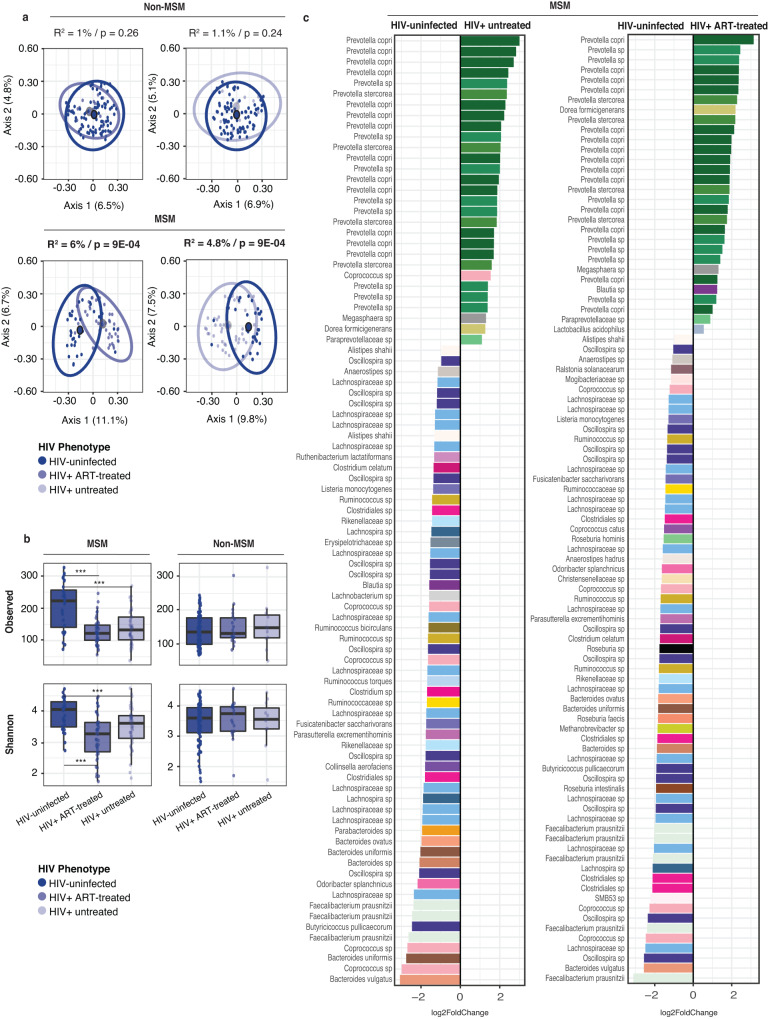
HIV infection impacts fecal microbiota composition differently in MSM and non-MSM populations. **a** PCoA ordination plots using unweighted UniFrac distances at the ASV (amplicon sequence variant) level comparing both ART (antiretroviral)-treated and -untreated HIV infection to the corresponding HIV-uninfected controls for MSM (men who have sex with men) and non-MSM individuals in the U.S. For the ordination plots, *R*^2^ and *p* values were obtained from a PERMANOVA analysis accounting for HIV infection status. HIV-uninfected vs ART-treated HIV infection: *F*(df = 1) = 1.06, *p* = 0.26, *R*^2^ = 1% non-MSM (*n* = 104 biologically independent samples) vs *F*(df = 1) = 4.66, *p* = 0.0009, *R*^2^ = 6% MSM (*n* = 74 biologically independent samples). HIV-uninfected vs untreated HIV infection: (*F*(df = 1) = 1.08, *p* = 0.25, *R*^2^ = 1.1% non-MSM (*n* = 96 biologically independent samples) vs *F*(df = 1) = 3.77, *p* = 0.0009, *R*^2^ = 4.8% MSM (*n* = 76 biologically independent samples). **b** Boxplots showing the values for the observed richness and Shannon diversity metrics for the three HIV groups in MSM (*n* = 236 biologically independent samples) and non-MSM (*n* = 230 biologically independent samples) individuals (***p value ≤ 0.01). Boxplots are displayed with the median as the center value with the box as IQR and whiskers as minima and maxima. Observed (MSM): Kruskal–Wallis with Bonferroni correction *χ*^2^(df = 2) = 24.2, *p* < 0.001; pairwise comparisons: HIV-uninfected vs ART-treated *Z* > 4 (*p* < 0.0001), HIV-uninfected vs untreated HIV infection *Z* > 4 (*p* < 0.0001), ART-treated vs untreated HIV infection *Z* = 0.01 (*p* = 0.99). Shannon (MSM): Kruskal–Wallis with Bonferroni correction *χ*^2^(df = 2) = 17.7, *p* < 0.001; pairwise comparisons: HIV-uninfected vs ART-treated *Z* > 4 (*p* < 0.0001), HIV-uninfected vs untreated HIV infection *Z* = 2.35 (*p* = 0.019), ART-treated vs untreated HIV infection *Z* = 1.37 (*p* = 0.17). **c** Results from an ANCOM analysis testing for differences of both ART-treated and -untreated HIV infection compared to the HIV-uninfected controls in the MSM group in the U.S. Only ASVs present in at least 5% of the samples in each HIV infection group were considered. The Log2FC values are represented on the *x*-axis for all those single ASVs with significantly more or less abundance in each of the tested groups. Color code represents taxonomic classification at the species level and ASVs are identified up to most resolutive taxonomic level.

In the U.S., those reporting MSM behavior comprise a large proportion of the HIV-infected population and account for 69% of new U.S. infections^31^. We therefore examined the impact of HIV infection on the fecal microbiota in subjects in the U.S. self-reporting MSM and heterosexual practices. Interestingly, the variability of the microbiota composition explained by HIV infection was only significant within the MSM population but not the non-MSM subset for both ART-treated HIV infection (Fig. 3a, PERMANOVA; *F*(df = 1) = 1.06, *p* = 0.26, *R*^2^ = 1% non-MSM vs *F*(df = 1) = 4.66, *p* = 0.0009, *R*^2^ = 6% MSM) untreated HIV infection (*F*(df = 1) = 1.08, *p* = 0.25, *R*^2^ = 1.1% non-MSM vs *F*(df = 1) = 3.77, *p* = 0.0009, *R*^2^ = 4.8% MSM), and the significant associations were robust to controlling with available metadata (Supplementary Table 14). While we did not find significant differences in observed richness and Shannon diversity index between HIV-infected and -uninfected individuals in the non-MSM group, both ART-treated and -untreated HIV infection showed significantly lower richness and diversity compared to HIV-uninfected individuals in the MSM group (Fig. 3b).

We used ANCOM to identify specific bacterial ASVs that were differentially abundant with both ART-treated and -untreated HIV infection and found more microbial differences in the MSM group in comparison to ART-treated subjects (Fig. 3c; 95 and 89 ASVs for ART-treated and -untreated HIV infection, respectively) than in the non-MSM group (Fig. 2b; 53 and 117, respectively), but a similar number of microbial differences in the comparison to ART-untreated subjects. Unlike in the non-MSM group, HIV infection in the MSM group was strongly associated with a higher abundance of *Prevotella* ASVs independent of ART (Fig. 3c). We show that in MSM individuals, there were more significant differences in fecal microbiota composition with HIV infection than in those who do not report MSM behavior.

### Immune activation is associated with unique microbial alterations in each region

**Fig. 4:**
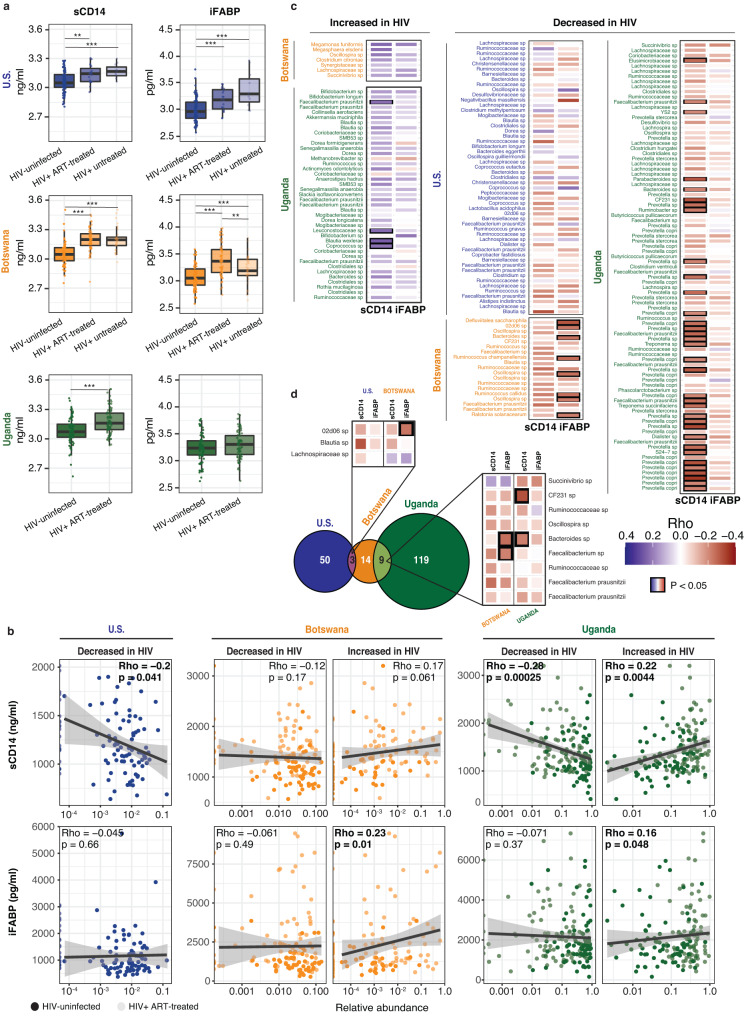
Markers of immune activation are associated with unique microbial alterations in each region. **a** Boxplots showing sCD14 and iFABP plasma measurements in non-MSM (men who have sex with men) HIV-infected and -uninfected individuals in each of the cohorts (***p value < 0.01, **p value < 0.05). *n* = 115(U.S.)/194(Botswana)/170(Uganda) biologically independent samples. Boxplots are displayed with the median as the center value with the box as IQR and whiskers as minima and maxima. Significance determined by Kruskal–Wallis with Bonferroni correction (three groups) or Wilcoxon (two groups) testing. U.S./sCD14: *χ*^2^(df = 2) = 14.4, *p* < 0.001; pairwise comparisons: HIV-uninfected vs ART (antiretroviral)-treated *Z* = 2.68 (*p* = 0.007), HIV-uninfected vs untreated HIV infection *Z* = 3.29 (*p* = 0.001), ART-treated vs untreated HIV infection *Z* = 0.96 (*p* = 0.34). U.S./iFABP: *χ*^2^(df = 2) = 21.4, *p* < 0.001; pairwise comparisons: HIV-uninfected vs ART-treated *Z* = 3.43 (*p* = 0.0006), HIV-uninfected vs untreated HIV infection *Z* = 3.89 (*p* = 0.0001), ART-treated vs untreated HIV infection *Z* = 1.15 (*p* = 0.25). Botswana/sCD14: *χ*^2^(df = 2) = 52.5, *p* < 0.001; pairwise comparisons: HIV-uninfected vs ART-treated *Z* > 4 (*p* < 0.0001), HIV-uninfected vs untreated HIV infection *Z* > 4 (*p* < 0.0001), ART-treated vs untreated HIV infection *Z* = 0.34 (*p* = 0.73). Botswana/iFABP: *χ*^2^(df = 2) = 35.2, *p* < 0.001; pairwise comparisons: HIV-uninfected vs ART-treated *Z* > 4 (*p* < 0.0001), HIV-uninfected vs untreated HIV infection *Z* = 3.09 (*p* = 0.002), ART-treated vs untreated HIV infection *Z* = 1.51 (*p* = 0.13). Uganda/sCD14: Wilcoxon W = 2040, *p* < 0.0001. Uganda/iFABP: Wilcoxon W = 2758, *p* = 0.11. **b** Association between each plasma marker and the total relative abundance of all the ASVs with significantly different abundance between ART-treated HIV-infected and -uninfected individuals in each of the cohorts. Each dot is a sample and the color represents whether they are ART-treated HIV-infected (light) or HIV-uninfected individuals (dark). Rho and *p* values were determined via two-sided Pearson’s correlation. Trendline fit using linear regression and error band denotes 95% confidence interval. U.S./decreased/sCD14: *t*(df = 100) = −2.07, *p* = 0.041, Rho = −0.20 [−0.38, −0.01]; U.S./decreased/iFABP: *t*(df = 100) = −0.45, *p* = 0.66, Rho = −0.045 [−0.23, 0.15]; Botswana/decreased/sCD14: *t*(df = 125) = −1.37, *p* = 0.17, Rho = −0.12 [−0.29, 0.05]; Botswana/increased/sCD14: *t*(df = 125) = 1.89, *p* = 0.061, Rho = 0.17 [−0.01, 0.33]; Botswana/decreased/iFABP: *t*(df = 125) = −0.69, *p* = 0.49, Rho = −0.061 [−0.23, 0.11]; Botswana/increased/iFABP: *t*(df = 125) = 2.62, *p* = 0.01, Rho = 0.23 [0.06, 0.39]; Uganda/decreased/sCD14: *t*(df = 159) = −3.74, *p* = 0.00025, Rho = −0.28 [−0.42, −0.14]; Uganda/increased/sCD14: *t*(df = 159) = 2.89, *p* = 0.0044, Rho = 0.22 [0.07, 0.37]; Uganda/decreased/iFABP: *t*(df = 159) = −0.89, *p* = 0.37, Rho = −0.071 [−0.22, 0.09]; Uganda/increased/iFABP *t*(df = 159) = 1.99, *p* = 0.048, Rho = 0.16 [0.001, 0.30]. **c** Spearman correlation between the relative abundance of each individual ASV detected in the ANCOM comparison between ART-treated HIV-infected and -uninfected subjects in each cohort, and either sCD14 or iFABP. Color code represents the Rho value. Statistically significant correlations after adjusting with FDR are highlighted in a black square (threshold set to 0.05). For each geographic location, ASVs are ordered from highest to lowest Log2FC in the ANCOM comparison (Fig. 2b–d). **d** Zoom out from panel (**c**) highlighting those ASVs that are shared among at least 2 of the geographical locations.

HIV infection leads to disruption of gut epithelial barrier function, which can permit translocation of luminal bacterial products into the systemic circulation and chronic immune activation, ultimately contributing to HIV disease progression^14^. Intestinal fatty-acid binding protein (iFABP), an intracellular protein released by epithelial cells upon mucosal damage, and soluble CD14 (sCD14), a marker of monocyte and macrophage activation by many inflammatory stimuli including bacterial lipopolysaccharide (LPS), which is associated with HIV mortality^32^, have both been reported to be increased in the circulation during HIV infection^33,34^. We measured plasma levels of these markers and found that among HIV-infected individuals, sCD14 was elevated in all three cohorts, and iFABP was elevated in HIV-infected individuals in the U.S. and Botswana, regardless of ART administration (Fig. 4a).

We observed an association between the relative abundance of the specific ASVs differentially abundant between ART-treated HIV-infected individuals and HIV-uninfected controls and sCD14 and/or iFABP in all cohorts (Fig. 4b). In the U.S. cohort, we found a significant negative correlation between the overall abundance of those ASVs decreased in HIV and plasma levels of sCD14 (Fig. 4b). In Botswana, we found a significant positive correlation between the overall abundance of those ASVs increased in HIV and plasma levels of iFABP. In the Ugandan cohort, we observed a significant negative correlation between sCD14 and the overall abundance of those ASVs decreased with HIV, and a positive correlation of those increased with HIV and sCD14 and iFABP. At the single ASV level, we found multiple significant correlations with sCD14 in the Ugandan cohort (Fig. 4c). Interestingly, of the 12 HIV-associated ASVs shared between any of the cohorts (Fig. 1e showing the 12 shared ASVs), only 4 taxa showed any significant association with either inflammatory marker (sCD14 or iFABP) and these significant associations were not shared between geographic locations, although one ASV in the genus *Bacteroides* was associated with increased sCD14 in Uganda and iFABP in Botswana (Fig. 4d). These findings indicate that HIV infection was associated with increased markers of immune activation regardless of geography, but the specific associations between bacterial taxa and these markers were not shared between HIV-infected individuals in each region.

## Discussion

### HIV-associated changes in fecal microbiota differ by geographic location

Previous studies have shown that HIV infection is associated with decreased bacterial richness and diversity and an increase in the abundance of Proteobacteria^4,17^. However, these studies were primarily performed with small cohorts in high-income countries with low overall HIV prevalence such as the U.S. and Western European nations. Few studies of HIV-associated changes in fecal microbiota have been conducted in sub-Saharan Africa, where almost 70% of HIV-infected people live^35^. By directly comparing large cohorts from the U.S., Botswana, and Uganda, we found few common bacterial changes with HIV infection between individuals in these regions, and no HIV-associated bacterial changes were shared between all three geographic cohorts. Moreover, specific bacterial changes associated with markers of monocyte and macrophage activation linked to bacterial translocation differed between each of the cohorts. However, it is interesting to note that even though no taxa were shared between all three geographic locations, the subset of taxa shared between two locations did include members of the families *Faecalibacterium* and *Blautia*, families which have been previously linked with health-associated factors such as reduced inflammation, although these relationships have been noted to be context-dependent^36,37^. Furthermore, one *Faecalibacterium* species shared between Botswana and Uganda was inversely associated with the inflammatory iFABP in the Botswanan cohort, although it was not significantly associated with any inflammatory markers in the Ugandan cohort. Even though these taxa were not shared across all three geographic locations, their occurrence as a common signature of HIV infection does suggest that further investigation into the mechanism of this association and their association with inflammatory modulation is warranted.

The differences in HIV- and inflammation-associated bacterial taxa are likely affected by many factors including diet, environment, and socioeconomic factors that have been shown to affect the microbiota^18,38,39^ and are known to differ between the geographic locations studied here. Our findings indicate that HIV-associated alterations in fecal bacterial communities are strongly influenced by geographic context (which is comprised of multiple different interacting factors), as microbial differences associated with HIV infection in high-income countries with low HIV prevalence have few conserved features with those living in low-income countries in sub-Saharan Africa with high HIV prevalence. An analysis of predicted function extrapolated from 16S rRNA gene sequencing data also suggested limited overlap between geographical cohorts in comparisons with either ART-treated or -untreated HIV infection, with the only overlapping predicted gene function between U.S. and Ugandan cohorts having inverse fold changes (Supplementary Fig. 7). However, performing metagenomic analysis would be a more comprehensive way to address functional contributions to the taxonomic observations reported here. Overall, these findings suggest that associations between fecal microbiota and human disease may vary significantly between groups, highlighting the importance of studying diverse populations.

### Impact of HIV infection on fecal microbiota is more pronounced in MSM than non-MSM populations in the U.S

While geography is associated with unique differences in fecal microbiota with HIV infection, we found more pronounced alterations in MSM compared to non-MSM populations in the U.S., with significant decreases in microbial richness and diversity and enrichment of *Prevotella* species in the MSM population with HIV infection. Interestingly, we observed a significant decrease in *Prevotella* species in those with HIV in Uganda, again highlighting how different the HIV-associated taxonomic differences are within populations. Prior studies (although predominantly in high-income countries) have described intestinal microbial differences in MSM compared to non-MSM populations, notably *Prevotella* species, which also emerged in our analysis of our U.S. (high-income country) MSM population, so these MSM-associated microbial differences have great potential to have confounded studies of HIV-associated differences in fecal microbial communities that did not take MSM behavior into account^28,40–42^. We show that in addition to geography, host sexual behavior also impacts HIV-associated microbial changes.

### Bacterial taxonomic identification is important in HIV-associated dysbiosis

Across the many comparisons in this study, we report different ASVs matching *Prevotella* species as being both increased and decreased with HIV infection in different cohorts. *Prevotella* increased with HIV infection in the MSM population in the U.S. and strongly decreased with ART-treated infection in Uganda, where they also showed a consistent negative correlation to plasma markers of gut barrier integrity and monocyte activation associated with HIV disease severity. This suggests that *Prevotella* species, such as *Prevotella copri*, may have significant intra-species variation and that they may not be uniformly pro-inflammatory in vivo, even though a number of studies performed in high-income countries have shown correlations between increased *Prevotella* and inflammation in a number of disease contexts (reviewed in ref. ^43^). The factors that lead to differing associations between *Prevotella* species and health outcomes suggest that these associations may not be consistent in all populations and may also depend on strain variants that differ by geography as well as potential host genetic differences.

### Limitations to this study

For the African cohorts, we were unable to analyze the impact of MSM behavior due to insufficient numbers of MSM-identifying individuals. Sexual behavior data for subjects in Uganda or HIV-uninfected subjects in Botswana was not available. MSM sexual behavior and the practice of receptive anal intercourse do have the potential to affect the composition of the microbiome, especially with regard to *Prevotella* species^42,44^, which were abundant in our cohorts. An extrapolation of sexual behavior data for HIV-infected subjects in Botswana based on reported HIV risk factor did not show any MSM-identifying individuals: 109 of 114 total HIV-infected subjects listed heterosexual sexual contact as their HIV risk factor, 5 were classified as an unknown risk factor, and no subjects reported risk factors of MSM behavior or intravenous substance use. This lack of data could result in MSM-associated confounding to our findings, although the available data from the general population in Sub-Saharan Africa has shown an overall low prevalence of MSM behavior^45^. As one method for controlling for the influence of MSM behavior, we performed both female-only and male-only analysis (Supplementary Figs. 1 and 2). While we were able to collect data with regard to MSM-identification in our Boston cohort, detailed sexual behavior information was available for only 83 individuals. We found a notable prevalence of receptive anal intercourse behavior, with approximately 20% of the surveyed subjects reporting having had receptive anal intercourse in the last 30 days (Supplementary Table 15), creating the possibility that this practice would represent a confounding factor in our analysis. Therefore, we could not completely assess the effect of specific sexual behaviors such as receptive anal intercourse on fecal microbiota composition, and we could not rule out the role of sexual behavior in *Prevotella*-associated observations. We note that the presence of sex workers in this female population could also contribute confounding effects, although data on occupation collected from our Uganda cohort (in which sex worker was a selectable category in the survey tool) showed no subject reporting sex worker as their occupation (Supplementary Data 1). Given the demonstrated influence of sexual behavior on gut microbiota composition, and the relative lack of this data collected in the African cohort, future studies would benefit from examining the effects of sexual behavior on the gut microbiota of individuals in Africa.

Diet is known to have a profound effect on the fecal microbiota^46^ and in our study dietary information was only available in the U.S. and Botswana, and in the form of a 24-h food recall questionnaire, which can be a limited tool with regard to the ability to fully assess dietary habits and nutrient intake. While in these cohorts we did not find any major dietary differences between HIV-infection groups (Supplementary Fig. 4), we could not assess the dietary habits in Uganda. We did make substantial efforts to control for potential confounders using available subject metadata (Supplementary Tables 1–3, 5–10, and 14), but acknowledge that for many subjects, data were not available for other potential confounders such as hepatitis B or C coinfection, socioeconomic factors, or current and nadir CD4 T cell counts. In addition, while we did control for BMI, ART regimen, and multiple comorbidities including hyperlipidemia, hypertension, and cardiovascular disease, these data were significantly different between each of the geographic cohorts and could still represent potential confounders despite our efforts to control for them. In the U.S. and Botswana cohorts, antibiotic usage within the 30 days prior to sample collection was an active exclusion criterion, up to 95% of HIV-infected individuals in Uganda were on prophylactic cotrimoxazole (trimethoprim-sulfamethoxazole) based on the national clinical standard of care at the time of sample collection, which is another confounding factor in our analysis. Lastly, all of our three cohorts were cross-sectional with no longitudinal time points nor pre- or post-HIV infection assessment.

### Caution in extrapolating microbiome findings between distinct human populations

Here we report the first study to directly compare the impact of HIV infection on fecal microbiota in large cohorts from three diverse geographic regions in the U.S. and sub-Saharan Africa. Our findings show that both geography and MSM behavior are associated with characteristic patterns of HIV-associated fecal microbiota changes with few disease-associated taxa shared between those in the U.S. and sub-Saharan Africa, where the greatest burden of HIV disease exists. In some groups, HIV-associated taxonomic changes were almost inverse, with significant increases in multiple *Prevotella* taxa in U.S. MSM and decreases in those in Uganda. In addition, we demonstrate that HIV infection is consistently associated with higher soluble makers of immune activation linked to microbial translocation across cohorts but that the specific bacterial taxa associated with these markers in each region were not shared. Overall, these findings dispute the idea of a specific HIV-associated dysbiotic community and more broadly support the importance of studying relevant and diverse populations to better understand the impact of the fecal microbiome on human health and disease.

## Methods

### Ethics approval and consent to participate

Samples were collected following informed consent from all participants. Those in the U.S. consented under a protocol reviewed by the Massachusetts General Hospital (MGH) Institutional Review Board (Protocol #2011/P001707). The study in Uganda was approved by the institutional ethics review board of Mbarara University of Science and Technology (MUST-REC) and also received clearance from the Uganda National Council of Science and Technology (UNCST) and the Research Secretariat in the Office of the President of Uganda, in accordance with the national guidelines. The study also received approval from the MGH IRB (Protocol #2014P001928). For subjects in Botswana, ethics clearance was obtained from the Ministry of Health and University of Botswana and the MGH IRB (Protocol #2014P001388).

### Study cohorts

#### Boston, Massachusetts, U.S

Matched, de-identified stool, serum and plasma samples, as well as demographics and clinical data including 24-h food recall surveys, were collected from 233 subjects enrolled from The Ragon Institute of MGH, MIT and Harvard in Boston, MA, as approved by the Institutional Review Boards at Partners Healthcare. Recruitment and sample collection were conducted between November 2010 and April 2019. Sex was self-reported. All participants gave written informed consent. This cohort was comprised of 117 HIV-uninfected subjects, 61 HIV-infected subjects on long-term ART therapy (>4 years), and 55 HIV-infected subjects who had not received ART therapy (ART-untreated) (Supplementary Data 1). The inclusion criteria were that subjects were between 18-75 years of age and had laboratory values within 28 days prior to enrollment and had hemoglobin >9.0 g/dL. Exclusion criteria for enrollment included current use of high-dose commercial probiotics (≥10^8^ CFU per organism/day); current use of systemic steroids, interleukins, or interferon (local use permitted); active oral candidiasis, any unusual changes in skin, or being on active chemotherapy at the time of enrollment; experiencing an episode of acute diarrhea or use of systemic antibiotics within 7 days prior to sampling; use of immunomodulatory agents, sexually transmitted infection (STI) treatment or an active STI within 30 days prior to sampling; clinically significant gastrointestinal disease including but not limited to ulcerative colitis, Crohn’s disease, or *C. difficile* infection within 3 months of sampling; recurrent body rashes within 6 months prior to sampling; any history of psoriasis, recurrent eczema, chronic dry mouth, untreated cavitated dental caries or oral abscesses, urinary incontinence necessitating use of protection garments, or receiving an HIV vaccine. In addition, female subjects were excluded if they were currently pregnant, had a present intention of becoming pregnant, were less than 8 weeks post-partum, or had had a hysterectomy. Subjects with other comorbidities were not excluded.

#### Gaborone, Botswana

Matched, de-identified stool plasma samples were collected from 194 subjects enrolled from the Princess Marina Infectious Disease Clinic and Gaborone Voluntary HIV testing Centre in Botswana as approved by the Institutional Review Boards at Princess Marina Hospital, the Botswanan Ministry of Health Research Division, and Partners Healthcare. Recruitment and sample collection were conducted between January 2014 and December 2015. The cohort was comprised of 80 HIV-infected subjects on suppressive ART, 40 HIV-infected ART-naïve subjects, and 80 HIV-uninfected controls aged between 30 and 50 years inclusive. Participants provided detailed cardiovascular risk history, completed 24-h food recall interview, had physical measurements and bilateral carotid ultrasound scanning, and provided stool and blood samples (Supplementary Data 1). Sex was self-reported. For all HIV-infected participants, medical records were reviewed to obtain relevant HIV disease and treatment history-related data. All procedures were completed during one study visit.

#### Mbarara, Uganda

Matched, de-identified stool, serum, and plasma samples were collected from 170 subjects enrolled from the Mbarara Regional Referral Hospital in Uganda as approved by the Institutional Review Boards at the Mbarara University of Science and Technology, Ugandan National Council of Science and Technology, and Partners Healthcare. All participants gave written informed consent. Recruitment and sample collection were conducted between May 2013 and December 2014. This cohort was comprised of 80 HIV-uninfected subjects and 90 location-matched samples from subjects on long-term ART therapy (>5 years). Subjects with other non-HIV comorbidities were not excluded. Sex was self-reported. Data collected included demographics, vital signs, medication history including antibiotic use, clinical symptoms, HIV RNA viral load, CD4 T cell counts at stool collection and at ART initiation, water source, food security, farming, and other laboratory results (Supplementary Data 1).

### Sample collection and processing

De-identified stool and plasma samples were collected from 597 subjects enrolled from the Mbarara Regional Referral Hospital in Uganda, Princess Marina Hospital in Gaborone, Botswana, and Massachusetts General Hospital, the U.S. All stool samples from Uganda and Botswana were collected without preservative, stored at −80 °C after collection, and shipped on dry ice in compliance with applicable Material Transfer Agreements. Stool samples from the U.S. were homogenized in phosphate-buffered saline, frozen in liquid nitrogen, and stored at −80 °C. Plasma samples were collected in either acid citrate dextrose tubes (Uganda and the U.S.) or EDTA tubes (Botswana) and frozen at −80 °C.

#### Stool total nucleic acid (TNA) extraction

Stool TNA was extracted by using 200 µL of 1 mm diameter zirconia/silica beads (Biospec) added to individual pulverized stool aliquots (~200 mg). 500 μL of phenol:chloroform:isoamyl alcohol (Invitrogen, 25:24:1, buffered to pH 7.9), 500 μL of 0.2 µm-filtered 2× Buffer A (200 mM NaCl, 200 mM Tris, 20 mM EDTA), and 210 μL of 20% SDS. Samples were chilled on ice and homogenized using the highest setting on a BioSpec Mini-Beadbeater for 2 min at 4 °C. The homogenized samples were then centrifuged at 7000 × g for 3 min at 4 °C, and the aqueous phase was transferred to a clean tube. An equal volume of phenol:chloroform:isoamyl alcohol was added and mixed by vortexing. Samples were then centrifuged at 16,000 × g for 5 min at room temperature and the aqueous phase transferred to a clean tube. Nucleic acid was precipitated with isopropanol and 3 M sodium acetate at −80 °C for 20 min, then spun at maximum speed at 4 °C for 30 min. The pellet was washed with 500 µL 100% ethanol, centrifuged at 16,000 × g for 15 min at 4 °C, dried, and resuspended in 200 µL of molecular grade Tris-EDTA buffer (Teknova, Cat # T0226). RNA and DNA fractions were separated from TNA using an AllPrep DNA/RNA Mini Kit (Qiagen, Cat # 80204) per the manufacturer’s protocol including the addition of an optional DNAse treatment (Qiagen, Cat # 79254).

#### 16S rRNA gene amplification and sequencing

The V4 region of the 16S rRNA gene was amplified following the method described previously^47^. Briefly, each sample was amplified in triplicate, pooled, and confirmed by gel electrophoresis. PCR reactions contained 5 μL 5X Q5 Reaction Buffer (New England Biolabs, Cat # B9027S), 16.25 μL RNase/DNase-free water, 0.5 μL 10 mM dNTPs, 0.5 μL each of the forward and reverse Golay-barcoded primers specific for the V4 region of the 16S rRNA gene (515F/806R, 10 μM final concentration^48^), 0.25 μL Q5 Polymerase (New England Biolabs, Cat # M0491S) and 2 μL TNA. Reactions were held at 98 °C for 30 s to denature the DNA, with amplification for 30 cycles of 98 °C for 15 s, 60 °C for 30 s, and 72 °C for 20 s, and a final extension of 2 min at 72 °C (to ensure complete amplification). Amplicons were pooled and the final pooled library was sequenced on the Illumina MiSeq platform.

### Bioinformatic analysis

Output single-end sequences were demultiplexed with QIIME v1.91^47^. Amplicon sequences were binned and quantified using the DADA2 v1.26.0 package in R^26^ using the GreenGenes 13.8 taxonomic reference database^49^. Resulting amplicon sequence variants (ASVs) were filtered when they were not assigned to the kingdom Bacteria or were assigned to the Family Mitochondria, the Class of Chloroplast or the Phylum of Cyanobacteria/Chloroplast. An average of 55824 sequences per sample were obtained (SD ± 44,408). Negative controls were included in DNA extraction and 16S rRNA gene amplification protocols. All controls had less than 10,000 reads/sample (average 3677 SD ± 3183) and were excluded from further analysis. ZymoBIOMICS Microbial Community Standards (Zymo Biomics, Cat# 6300) were used as positive controls and an average number of 34024 reads were obtained (SD ± 10,793).

#### Alpha and beta diversity analysis

To characterize alpha diversity, observed richness and Shannon diversity index were calculated using Vegan^50^ v2.6-4 and BiodiversityR^51^ v2.15-1R packages. To compare alpha diversity metrics among samples, a subset of 8000 counts was randomly selected for each sample using function rrarefy from the R Vegan v2.6-4 package^50^, as representative of the entire dataset.

To test for overall fecal microbiota differences among samples, ordination analyses were performed using Principal Coordinates Analysis (PCoA) in a two-dimensional space based on unweighted UniFrac ecological distances calculated on the ASVs composition matrices using the R Phyloseq v1.42.0 package^52^.

Bacterial ASVs were collapsed at the family, genus and species taxonomic ranks using the tax_glom function from the R Phyloseq v1.42.0 package^52^ and their relative abundances were plotted using the R ggplot2 v3.4.1 and gridExtra v2.3 packages^53^. Differential abundance analysis between two HIV infection groups was tested using ANCOM analysis from R ANCOMBC v2.0.2 package^30^ after filtering ASVs by being present in at least 5% of the samples in each HIV infection group in each cohort. Data were processed as necessary prior to and following analysis using the R packages dplyr v1.1.0, knitr v1.42, and reshape v0.8.9.

#### Statistics and reproducibility

For two group comparisons, the non-parametric Wilcoxon rank-sum test was used to test for statistical differences. For three group comparisons, a non-parametric Kruskal–Wallis Test from the R package agricolae v1.3-5 was used if metadata adjustment was not performed. When including metadata adjustment, a non-parametric ordinal regression on ranked abundances was used. If appropriate, Bonferroni multiple comparison correction on *p* values was applied. For correlations, Pearson or Spearman correlations were used, as appropriate and noted in the text.

A PERMANOVA analysis implemented in the adonis function in the R Vegan v2.6-4 package^50^ was used to test the variability explained by different metadata variables on the microbiota composition similarity measurements.

No statistical method was used to predetermine the sample size. Sex and/or gender was not considered in the initial study design, single-sex analyses were performed to control for confounding by sexual behavior. Other than that described in the main text (the exclusion of MSM or non-MSM subjects), no data were excluded from the main analyses. The experiments were not randomized.

### Plasma markers measurement

Biomarker quantification provided by the University of Vermont (UVM) Laboratory for Clinical Biochemistry Research included sCD14, and iFABP by sandwich ELISA (R&D Systems; Cat # DC140, DC1630, DFBP20). iFABP measurements were performed in duplicate. Dilutions of plasma were 1:200 for sCD14 and 1:5 for iFABP.

### Reporting summary

Further information on research design is available in the Nature Portfolio Reporting Summary linked to this article.

### Supplementary information

Supplementary Information (Figures and Tables)

Supplementary Data and Figures Description

Supplementary Dataset 1

Supplementary Dataset 2

Supplementary Dataset 3

Reporting Summary

Supplementary Table 15

Figure1ANCOM_Rocafort-Gootenberg_2022_12_16

Figure2ANCOM_Rocafort-Gootenberg_2023_03_08

Figure3ANCOM_Rocafort-Gootenberg_2023_03_02

Figure4ANCOM_Rocafort-Gootenberg_2023_04_05

SupplementaryFigure1_Rocafort-Gootenberg_2022_03_20

SupplementaryFigure2_Rocafort-Gootenberg_2022_03_20

SupplementaryFigure3_Rocafort-Gootenberg_2023_03_20

SupplementaryFigure5_Rocafort-Gootenberg_2023_02_07

SupplementaryFigure6_Rocafort-Gootenberg_2023_03_01

SupplementaryFigure7_Rocafort-Gootenberg_2023_04_19

## Data Availability

The metadata and raw 16S rRNA sequences generated in this study have been deposited in the European Nucleotide Archive and are publicly available under the BioProject accession code PRJEB41345. The GreenGenes 13.8 taxonomic reference database^49^ is publicly available at http://greengenes.microbio.me/greengenes_release/gg_13_8_otus/. The clinical metadata generated in this study are provided as Supplementary Data 1 and 3. ASVs generated by DADA2 v1.26.0^26^ are provided as Supplementary Data 2.

## Code availability

The code used in the analysis and figure generation in this study is provided as Supplementary Information
