# Supplementary material for: HIV-associated gut microbial alterations are dependent on host and geographic context
Source: Nat Commun. 2024 Feb 5;15:1055. doi: 10.1038/s41467-023-44566-4 (PMC10844288; doi:10.1038/s41467-023-44566-4)
Supplement: Supplementary file 1 — Supplementary Information (Figures and Tables) [file 41467_2023_44566_MOESM1_ESM.pdf]

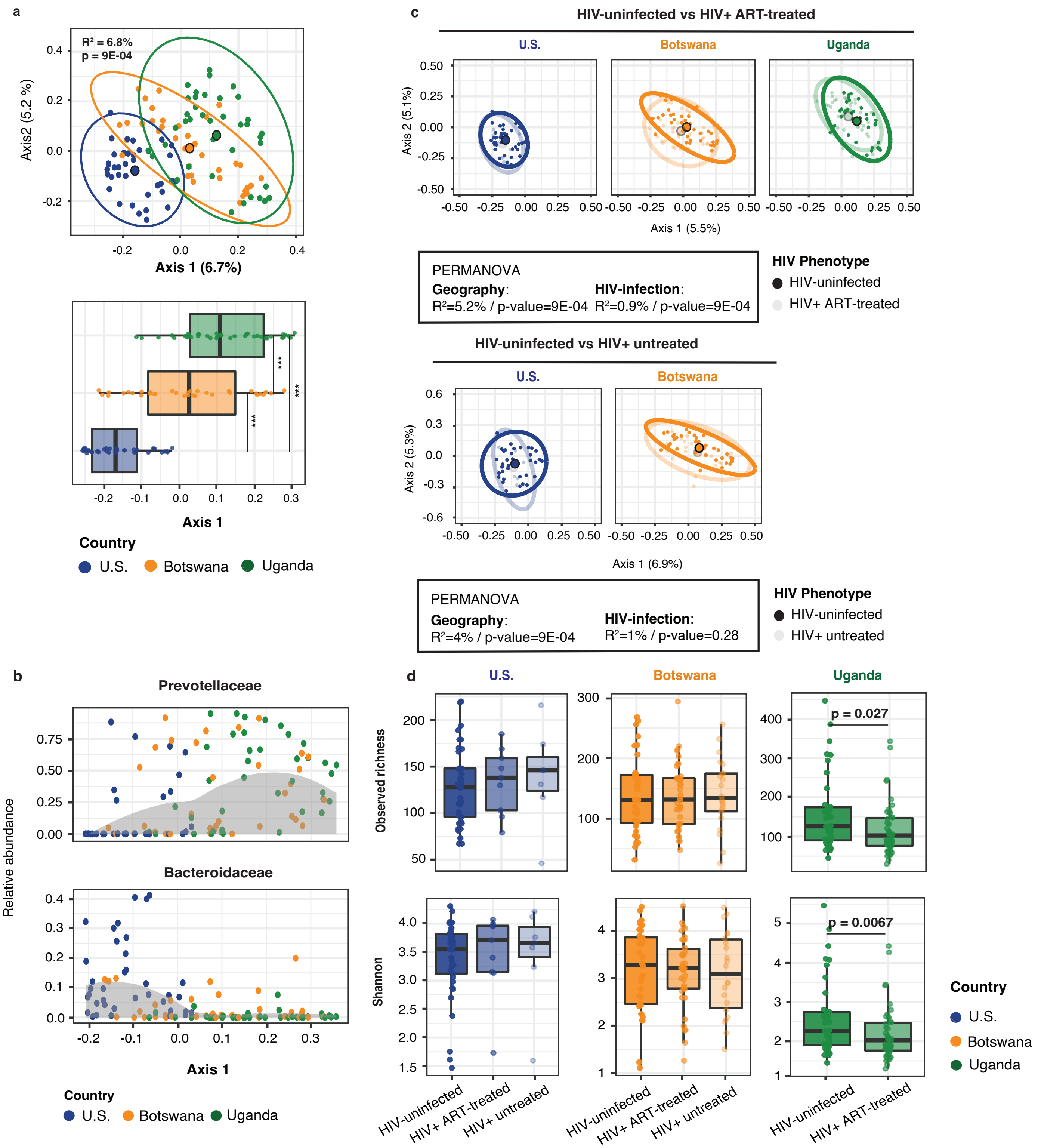
a
R² = 6.8%
p = 9E-04
Axis2 (5.2 %)
Axis 1 (6.7%)
***
***
***
Axis 1
Country
U.S.
Botswana
Uganda
c
HIV-uninfected vs HIV+ ART-treated
U.S.
Botswana
Uganda
Axis 2 (5.1%)
Axis 1 (5.5%)
PERMANOVA
Geography:
R²=5.2% / p-value=9E-04
HIV-infection:
R²=0.9% / p-value=9E-04
HIV Phenotype
HIV-uninfected
HIV+ ART-treated
HIV-uninfected vs HIV+ untreated
U.S.
Botswana
Axis 2 (5.3%)
Axis 1 (6.9%)
PERMANOVA
Geography:
R²=4% / p-value=9E-04
HIV-infection:
R²=1% / p-value=0.28
HIV Phenotype
HIV-uninfected
HIV+ untreated
b
Prevotellaceae
Bacteroidaceae
Relative abundance
Axis 1
Country
U.S.
Botswana
Uganda
d
U.S.
Botswana
Uganda
Observed richness
p = 0.027
Shannon
p = 0.0067
HIV-uninfected
HIV+ ART-treated
HIV+ untreated
Country
U.S.
Botswana
Uganda

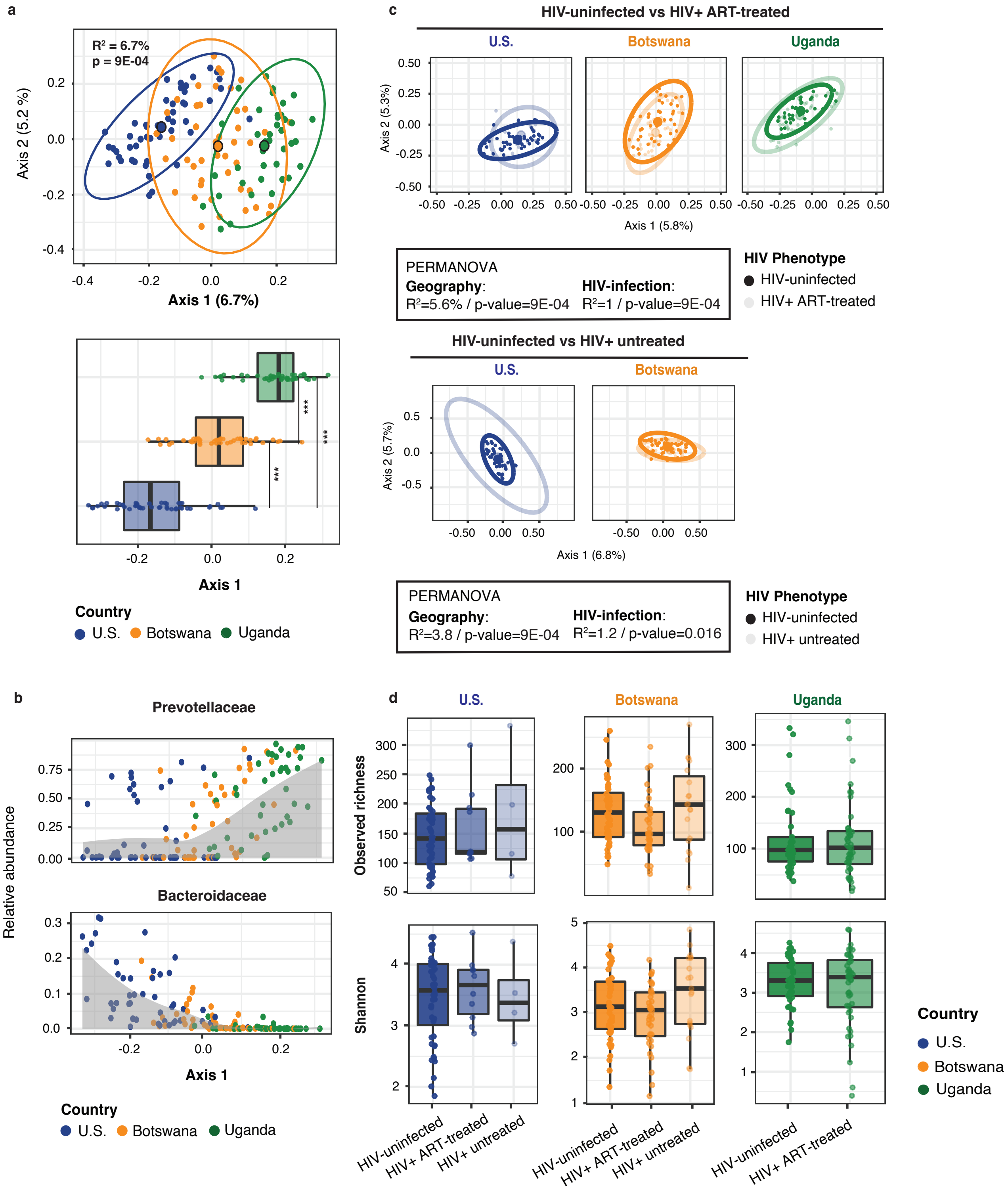
a
R² = 6.7%
p = 9E-04
Axis 2 (5.2 %)
Axis 1 (6.7%)
Axis 1
***
Country
U.S.
Botswana
Uganda
c
HIV-uninfected vs HIV+ ART-treated
U.S.
Botswana
Uganda
Axis 2 (5.3%)
Axis 1 (5.8%)
PERMANOVA
Geography: R²=5.6% / p-value=9E-04
HIV-infection: R²=1 / p-value=9E-04
HIV Phenotype
HIV-uninfected
HIV+ ART-treated
HIV-uninfected vs HIV+ untreated
U.S.
Botswana
Axis 2 (5.7%)
Axis 1 (6.8%)
PERMANOVA
Geography: R²=3.8 / p-value=9E-04
HIV-infection: R²=1.2 / p-value=0.016
HIV Phenotype
HIV-uninfected
HIV+ untreated
b
Prevotellaceae
Bacteroidaceae
Relative abundance
Axis 1
Country
U.S.
Botswana
Uganda
d
U.S.
Botswana
Uganda
Observed richness
Shannon
HIV-uninfected
HIV+ ART-treated
HIV+ untreated
Country
U.S.
Botswana
Uganda

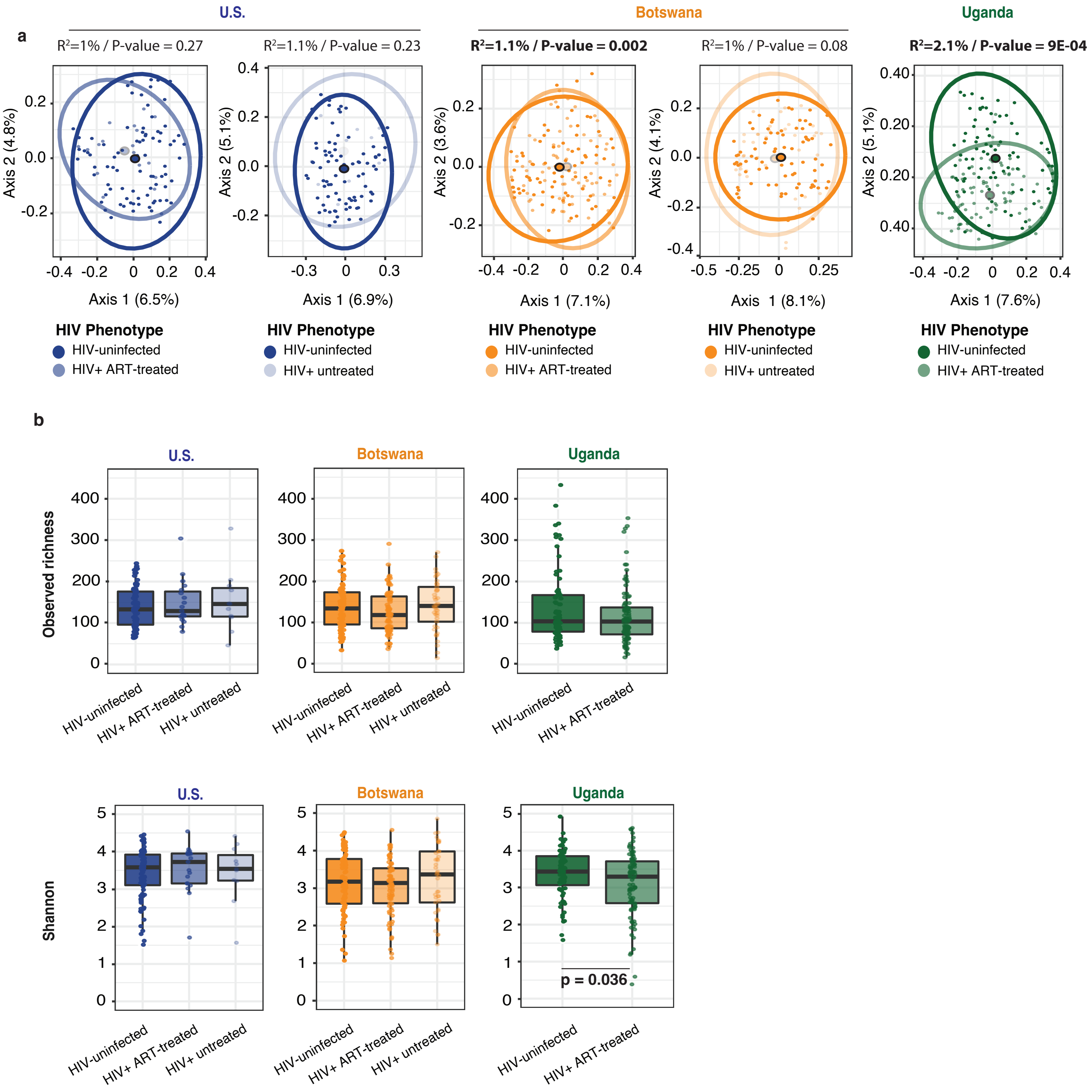
a
U.S.
Botswana
Uganda
R²=1% / P-value = 0.27
R²=1.1% / P-value = 0.23
R²=1.1% / P-value = 0.002
R²=1% / P-value = 0.08
R²=2.1% / P-value = 9E-04
Axis 2 (4.8%)
Axis 2 (5.1%)
Axis 2 (3.6%)
Axis 2 (4.1%)
Axis 2 (5.1%)
Axis 1 (6.5%)
Axis 1 (6.9%)
Axis 1 (7.1%)
Axis 1 (8.1%)
Axis 1 (7.6%)
HIV Phenotype
HIV-uninfected
HIV+ ART-treated
HIV+ untreated
b
Observed richness
Shannon
HIV-uninfected
HIV+ ART-treated
HIV+ untreated
p = 0.036

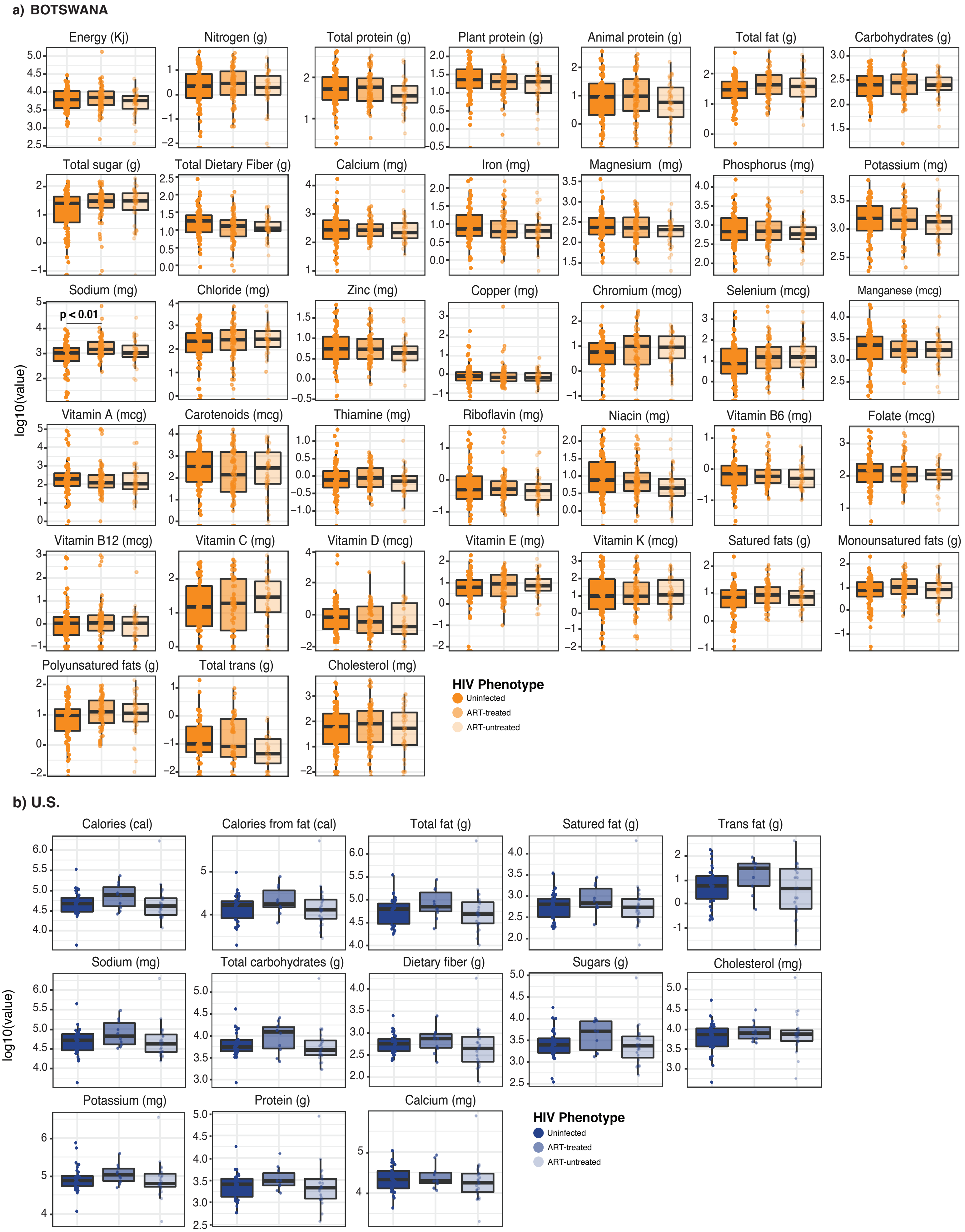

a) BOTSWANA
Energy (Kj)
Nitrogen (g)
Total protein (g)
Plant protein (g)
Animal protein (g)
Total fat (g)
Carbohydrates (g)
Total sugar (g)
Total Dietary Fiber (g)
Calcium (mg)
Iron (mg)
Magnesium (mg)
Phosphorus (mg)
Potassium (mg)
Sodium (mg)
p < 0.01
Chloride (mg)
Zinc (mg)
Copper (mg)
Chromium (mcg)
Selenium (mcg)
Manganese (mcg)
Vitamin A (mcg)
Carotenoids (mcg)
Thiamine (mg)
Riboflavin (mg)
Niacin (mg)
Vitamin B6 (mg)
Folate (mcg)
Vitamin B12 (mcg)
Vitamin C (mg)
Vitamin D (mcg)
Vitamin E (mg)
Vitamin K (mcg)
Satured fats (g)
Monounsatured fats (g)
Polyunsatured fats (g)
Total trans (g)
Cholesterol (mg)
log10(value)
HIV Phenotype
Uninfected
ART-treated
ART-untreated
b) U.S.
Calories (cal)
Calories from fat (cal)
Total fat (g)
Satured fat (g)
Trans fat (g)
Sodium (mg)
Total carbohydrates (g)
Dietary fiber (g)
Sugars (g)
Cholesterol (mg)
Potassium (mg)
Protein (g)
Calcium (mg)
log10(value)
HIV Phenotype
Uninfected
ART-treated
ART-untreated

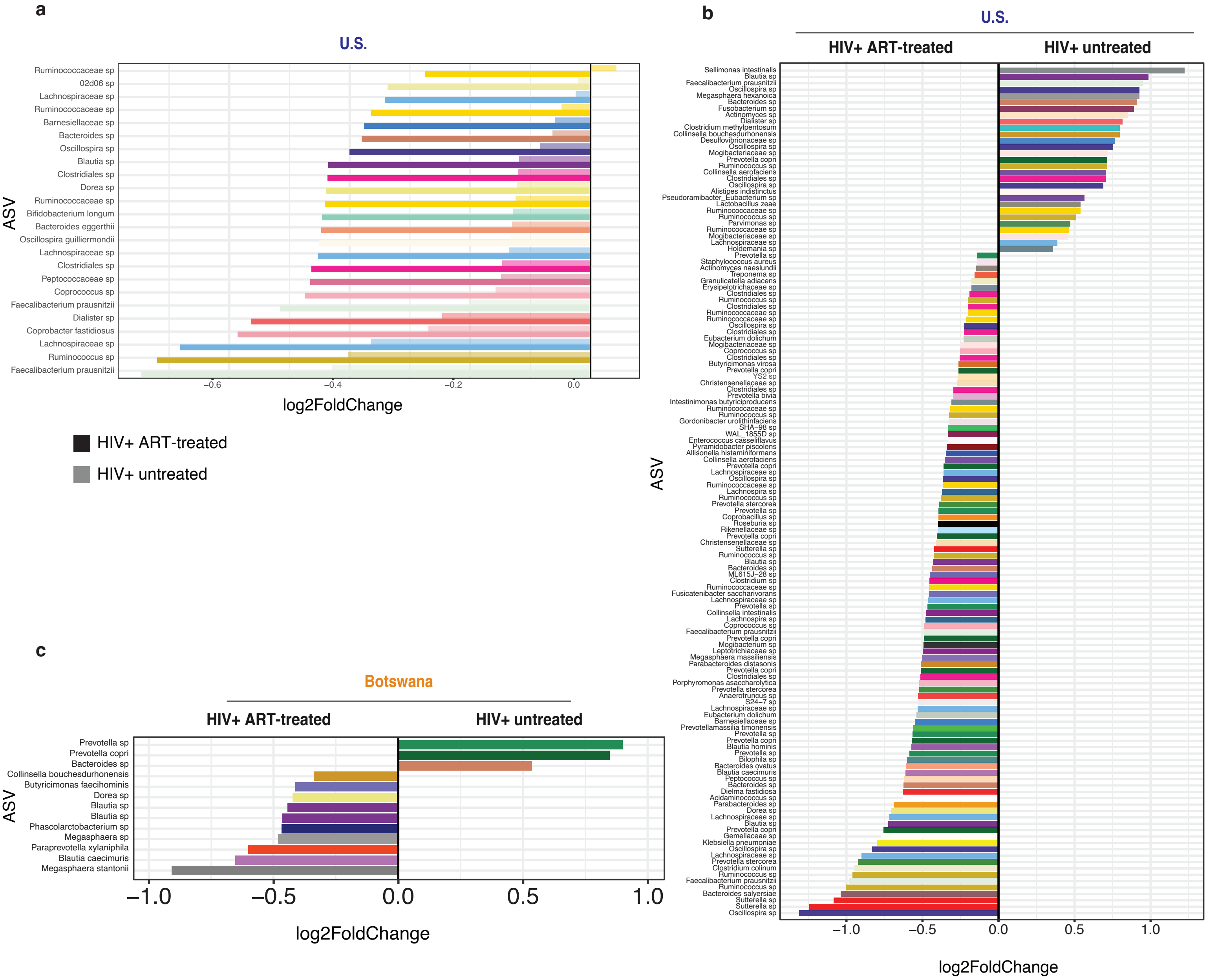
a
U.S.
Ruminococcaceae sp
02d06 sp
Lachnospiraceae sp
Ruminococcaceae sp
Barnesiellaceae sp
Bacteroides sp
Oscillospira sp
Blautia sp
Clostridiales sp
Dorea sp
Ruminococcaceae sp
Bifidobacterium longum
Bacteroides eggerthii
Oscillospira guilliermondii
Lachnospiraceae sp
Clostridiales sp
Peptococcaceae sp
Coprococcus sp
Faecalibacterium prausnitzii
Dialister sp
Coprobacter fastidiosus
Lachnospiraceae sp
Ruminococcus sp
Faecalibacterium prausnitzii
ASV
−0.6
−0.4
−0.2
0.0
log2FoldChange
HIV+ ART-treated
HIV+ untreated
b
U.S.
HIV+ ART-treated
HIV+ untreated
Sellimonas intestinalis
Blautia sp
Faecalibacterium prausnitzii
Oscillospira sp
Megasphaera hexanoica
Bacteroides sp
Fusobacterium sp
Actinomyces sp
Dialister sp
Clostridium methylpentosum
Collinsella bouchesdurhonensis
Desulfovibrionaceae sp
Oscillospira sp
Mogibacteriaceae sp
Prevotella copri
Ruminococcus sp
Collinsella aerofaciens
Clostridiales sp
Oscillospira sp
Alistipes indistinctus
Pseudoramibacter_Eubacterium sp
Lactobacillus zeae
Ruminococcaceae sp
Ruminococcus sp
Parvimonas sp
Ruminococcaceae sp
Mogibacteriaceae sp
Lachnospiraceae sp
Holdemania sp
Prevotella sp
Staphylococcus aureus
Actinomyces naeslundii
Treponema sp
Granulicatella adiacens
Erysipelotrichaceae sp
Clostridiales sp
Ruminococcus sp
Clostridiales sp
Ruminococcaceae sp
Ruminococcaceae sp
Oscillospira sp
Clostridiales sp
Eubacterium dolichum
Mogibacteriaceae sp
Coprococcus sp
Clostridiales sp
Butyricimonas virosa
Prevotella copri
YS2 sp
Christensenellaceae sp
Clostridiales sp
Prevotella bivia
Intestinimonas butyriciproducens
Ruminococcaceae sp
Ruminococcus sp
Gordonibacter urolithinfaciens
SHA−98 sp
WAL_1855D sp
Enterococcus casseliflavus
Pyramidobacter piscolens
Allisonella histaminiformans
Collinsella aerofaciens
Prevotella copri
Lachnospiraceae sp
Oscillospira sp
Ruminococcaceae sp
Lachnospira sp
Ruminococcus sp
Prevotella stercorea
Prevotella sp
Coprobacillus sp
Roseburia sp
Rikenellaceae sp
Prevotella copri
Christensenellaceae sp
Sutterella sp
Ruminococcus sp
Blautia sp
Bacteroides sp
ML615J−28 sp
Clostridium sp
Ruminococcaceae sp
Fusicatenibacter saccharivorans
Lachnospiraceae sp
Prevotella sp
Collinsella intestinalis
Lachnospira sp
Coprococcus sp
Faecalibacterium prausnitzii
Prevotella copri
Mogibacterium sp
Leptotrichiaceae sp
Megasphaera massiliensis
Parabacteroides distasonis
Prevotella copri
Clostridiales sp
Porphyromonas asaccharolytica
Prevotella stercorea
Anaerotruncus sp
S24−7 sp
Lachnospiraceae sp
Eubacterium dolichum
Barnesiellaceae sp
Prevotellamassilia timonensis
Prevotella sp
Prevotella copri
Blautia hominis
Prevotella sp
Bilophila sp
Bacteroides ovatus
Blautia caecimuris
Peptococcus sp
Bacteroides sp
Dielma fastidiosa
Acidaminococcus sp
Parabacteroides sp
Dorea sp
Lachnospiraceae sp
Blautia sp
Prevotella copri
Gemellaceae sp
Klebsiella pneumoniae
Oscillospira sp
Lachnospiraceae sp
Prevotella stercorea
Clostridium colinum
Ruminococcus sp
Faecalibacterium prausnitzii
Ruminococcus sp
Bacteroides salyersiae
Sutterella sp
Sutterella sp
Oscillospira sp
ASV
−1.0
−0.5
0.0
0.5
1.0
log2FoldChange
c
Botswana
HIV+ ART-treated
HIV+ untreated
Prevotella sp
Prevotella copri
Bacteroides sp
Collinsella bouchesdurhonensis
Butyricimonas faecihominis
Dorea sp
Blautia sp
Blautia sp
Phascolarctobacterium sp
Megasphaera sp
Paraprevotella xylaniphila
Blautia caecimuris
Megasphaera stantonii
ASV
−1.0
−0.5
0.0
0.5
1.0
log2FoldChange

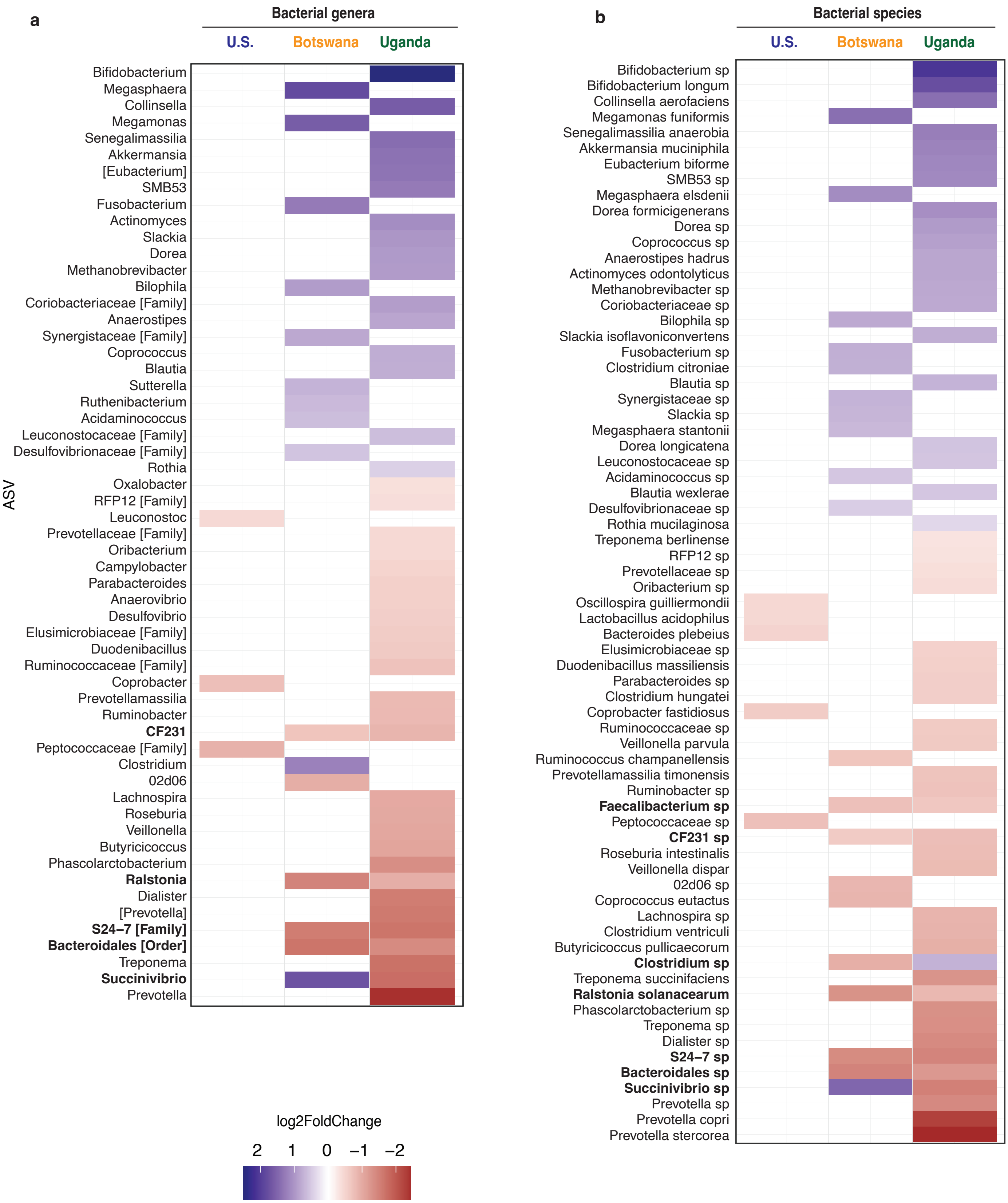
a
Bacterial genera
U.S.
Botswana
Uganda
ASV
Bifidobacterium
Megasphaera
Collinsella
Megamonas
Senegalimassilia
Akkermansia
[Eubacterium]
SMB53
Fusobacterium
Actinomyces
Slackia
Dorea
Methanobrevibacter
Bilophila
Coriobacteriaceae [Family]
Anaerostipes
Synergistaceae [Family]
Coprococcus
Blautia
Sutterella
Ruthenibacterium
Acidaminococcus
Leuconostocaceae [Family]
Desulfovibrionaceae [Family]
Rothia
Oxalobacter
RFP12 [Family]
Leuconostoc
Prevotellaceae [Family]
Oribacterium
Campylobacter
Parabacteroides
Anaerovibrio
Desulfovibrio
Elusimicrobiaceae [Family]
Duodenibacillus
Ruminococcaceae [Family]
Coprobacter
Prevotellamassilia
Ruminobacter
CF231
Peptococcaceae [Family]
Clostridium
02d06
Lachnospira
Roseburia
Veillonella
Butyricicoccus
Phascolarctobacterium
Ralstonia
Dialister
[Prevotella]
S24–7 [Family]
Bacteroidales [Order]
Treponema
Succinivibrio
Prevotella
log2FoldChange
2 1 0 −1 −2
b
Bacterial species
U.S.
Botswana
Uganda
Bifidobacterium sp
Bifidobacterium longum
Collinsella aerofaciens
Megamonas funiformis
Senegalimassilia anaerobia
Akkermansia muciniphila
Eubacterium biforme
SMB53 sp
Megasphaera elsdenii
Dorea formicigenerans
Dorea sp
Coprococcus sp
Anaerostipes hadrus
Actinomyces odontolyticus
Methanobrevibacter sp
Coriobacteriaceae sp
Bilophila sp
Slackia isoflavoniconvertens
Fusobacterium sp
Clostridium citroniae
Blautia sp
Synergistaceae sp
Slackia sp
Megasphaera stantonii
Dorea longicatena
Leuconostocaceae sp
Acidaminococcus sp
Blautia wexlerae
Desulfovibrionaceae sp
Rothia mucilaginosa
Treponema berlinense
RFP12 sp
Prevotellaceae sp
Oribacterium sp
Oscillospira guilliermondii
Lactobacillus acidophilus
Bacteroides plebeius
Elusimicrobiaceae sp
Duodenibacillus massiliensis
Parabacteroides sp
Clostridium hungatei
Coprobacter fastidiosus
Ruminococcaceae sp
Veillonella parvula
Ruminococcus champanellensis
Prevotellamassilia timonensis
Ruminobacter sp
Faecalibacterium sp
Peptococcaceae sp
CF231 sp
Roseburia intestinalis
Veillonella dispar
02d06 sp
Coprococcus eutactus
Lachnospira sp
Clostridium ventriculi
Butyricicoccus pullicaecorum
Clostridium sp
Treponema succinifaciens
Ralstonia solanacearum
Phascolarctobacterium sp
Treponema sp
Dialister sp
S24–7 sp
Bacteroidales sp
Succinivibrio sp
Prevotella sp
Prevotella copri
Prevotella stercorea

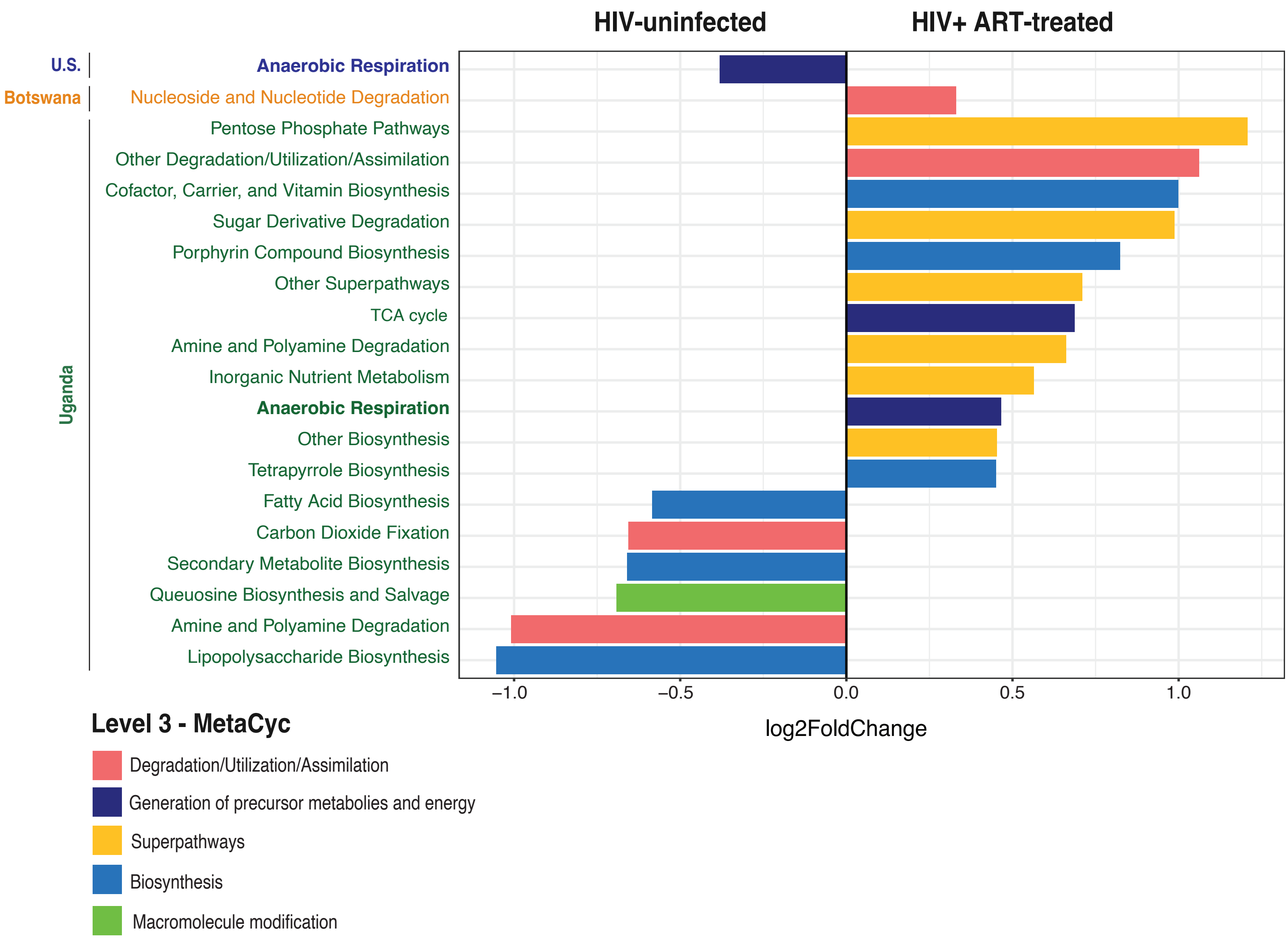

HIV-uninfected
HIV+ ART-treated
U.S.
Botswana
Uganda
Anaerobic Respiration
Nucleoside and Nucleotide Degradation
Pentose Phosphate Pathways
Other Degradation/Utilization/Assimilation
Cofactor, Carrier, and Vitamin Biosynthesis
Sugar Derivative Degradation
Porphyrin Compound Biosynthesis
Other Superpathways
TCA cycle
Amine and Polyamine Degradation
Inorganic Nutrient Metabolism
Anaerobic Respiration
Other Biosynthesis
Tetrapyrrole Biosynthesis
Fatty Acid Biosynthesis
Carbon Dioxide Fixation
Secondary Metabolite Biosynthesis
Queuosine Biosynthesis and Salvage
Amine and Polyamine Degradation
Lipopolysaccharide Biosynthesis
−1.0
−0.5
0.0
0.5
1.0
log2FoldChange
Level 3 - MetaCyc
Degradation/Utilization/Assimilation
Generation of precursor metabolies and energy
Superpathways
Biosynthesis
Macromolecule modification

Supplementary Table 1. Additional metadata correlation (HIV-uninfected)

	HIV-uninfected			
	All metadata available (n = 245)		All expanded metadata available (n = 231)	
	R^2	P	R^2	P
Geography	2.55%	0.0009***	2.22%	0.0009***
Sex	0.58%	0.004**	0.50%	0.0009***
Age	1.07%	0.0009***	0.75%	0.0009***
Race	3.06%	0.0009***	2.28%	0.0009***
Ethnicity	0.46%	0.09	0.31%	0.025*
BMI	-	-	0.75%	0.0009***
History of CVD (self-reported)	-	-	0.42%	0.34
History of CVD (self-reported or determined from available data)	-	-	0.68%	0.002**
History of HLD (self-reported)	-	-	0.43%	0.30
History of HLD (self-reported or determined from available data)	-	-	0.64%	0.006**
History of hypertension (self-reported)	-	-	0.80%	0.0009***
History of Type 2 DM (self-reported or determined from available data)	-	-	0.51%	0.06

Controlling for available metadata from Figure 1A (HIV-uninfected, all geographic cohorts) performed on unweighted UniFrac distances calculated at the ASV level. R^2 and p-value were obtained for each category of metadata available using a multivariate PERMANOVA analysis. When adding in metadata available for all subjects and metadata available for a substantial fraction of subjects, geography remained significantly associated with fecal microbial community. Metadata coding is defined in Supplementary Data 1. ***: $p < 0.001$, **: $p < 0.01$, *: $p < 0.05$

Supplementary Table 2. Additional metadata correlation (female-only, HIV-uninfected)

	HIV-uninfected			
	All metadata available (n = 112)		All expanded metadata available (n = 103)	
	R^2	P	R^2	P
Geography	3.69%	0.0009***	3.17%	0.0009***
Age	1.99%	0.0009***	1.57%	0.002**
Race	4.82%	0.0009***	4.14%	0.0009***
Ethnicity	0.97%	0.12	1.10%	0.094
BMI	-	-	0.98%	0.29
History of CVD (self-reported)	-	-	1.04%	0.16
History of CVD (self-reported or determined from available data)	-	-	1.45%	0.004**
History of HLD (self-reported)	-	-	1.11%	0.078
History of HLD (self-reported or determined from available data)	-	-	1.34%	0.008**
History of hypertension (self-reported)	-	-	1.72%	0.0009***
History of Type 2 DM (self-reported or determined from available data)	-	-	1.22%	0.023*

Controlling for available metadata from Supplementary Figure 1A (female-only, HIV-uninfected subjects) performed on unweighted UniFrac distances calculated at the ASV level. R^2 and p-value were obtained for each category of metadata available using a multivariate PERMANOVA analysis. When adding in metadata available for all subjects and metadata available for a substantial fraction of subjects, geography remained significantly associated with microbiome structure. Metadata coding is defined in Supplementary Data 1. CVD (cardiovascular disease), HLD (hyperlipidemia), DM (diabetes mellitus). ***: $p < 0.001$, **: $p < 0.01$, *: $p < 0.05$

Supplementary Table 3. Additional metadata correlation (male-only analysis, HIV-uninfected)

	HIV-uninfected			
	All metadata available (n = 133)		All expanded metadata available (n = 128)	
	R^2	P	R^2	P
Geography	3.09%	0.0009***	2.93%	0.0009***
Age	1.30%	0.0009***	1.33%	0.0009***
Race	5.63%	0.0009***	3.90%	0.0009***
Ethnicity	0.80%	0.18	0.74%	0.40
BMI	-	-	2.20%	0.0009***
History of CVD (self-reported)	-	-	0.81%	0.19
History of CVD (self-reported or determined from available data)	-	-	1.30%	0.002**
History of HLD (self-reported)	-	-	0.74%	0.45
History of HLD (self-reported or determined from available data)	-	-	0.95%	0.035*
History of hypertension (self-reported)	-	-	0.80%	0.24
History of Type 2 DM (self-reported or determined from available data)	-	-	0.85%	0.11

Controlling for available metadata from Supplementary Figure 2A (male-only, HIV-uninfected subjects) performed on unweighted UniFrac distances calculated at the ASV level. R^2 and p-value were obtained for each category of metadata available using a multivariate PERMANOVA analysis. When adding in metadata available for all subjects and metadata available for a substantial fraction of subjects, geography remained significantly associated with microbiome structure. Metadata coding is defined in Supplementary Data 1. CVD (cardiovascular disease), HLD (hyperlipidemia), DM (diabetes mellitus). ***: $p < 0.001$, **: $p < 0.01$, *: $p < 0.05$

Supplementary Table 4. Differentially abundant bacterial species between HIV-uninfected individuals in the U.S., Botswana, and Uganda.

Species name	RA (mean ± sd)	Species name	RA (mean ± sd)	Species name	RA (mean ± sd)
Prevotella copri	0.143 ± 0.249	*Prevotella copri*	0.192 ± 0.286	*Prevotella copri*	0.373 ± 0.293
Faecalibacterium prausnitzii	0.125 ± 0.088	*Prevotella unclassified*	0.082 ± 0.159	*Faecalibacterium prausnitzii*	0.073 ± 0.053
Blautia wexlerae	0.071 ± 0.066	*Bifidobacterium longum*	0.081 ± 0.146	*Prevotella unclassified*	0.062 ± 0.079
Akkermansia muciniphila	0.070 ± 0.162	*Faecalibacterium prausnitzii*	0.074 ± 0.068	*Bifidobacterium longum*	0.048 ± 0.139
Blautia unclassified	0.065 ± 0.054	*Blautia wexlerae*	0.063 ± 0.091	*Blautia wexlerae*	0.040 ± 0.050
Bifidobacterium longum	0.049 ± 0.102	*Bifidobacterium unclassified*	0.056 ± 0.117	*S24-7 unclassified*	0.037 ± 0.072
Bacteroides vulgatus	0.036 ± 0.053	*Akkermansia muciniphila*	0.040 ± 0.105	*Blautia unclassified*	0.034 ± 0.046
Bifidobacterium unclassified	0.033 ± 0.109	*Blautia unclassified*	0.034 ± 0.032	*Ruminococcaceae unclassified*	0.026 ± 0.031
Lachnospiraceae unclassified	0.027 ± 0.022	*S24-7 unclassified*	0.033 ± 0.079	*Akkermansia muciniphila*	0.021 ± 0.092
Fusicatenibacter saccharivorans	0.024 ± 0.028	*Ruminococcaceae unclassified*	0.030 ± 0.051	*Prevotella stercorea*	0.017 ± 0.020
Bacteroides unclassified	0.019 ± 0.028	*Prevotella stercorea*	0.022 ± 0.068	*Lachnospiraceae unclassified*	0.017 ± 0.025
Coprococcus unclassified	0.016 ± 0.014	*Lachnospiraceae unclassified*	0.019 ± 0.019	*Bifidobacterium unclassified*	0.015 ± 0.045
Prevotella stercorea	0.016 ± 0.069	*Fusicatenibacter saccharivorans*	0.017 ± 0.036	*Clostridium celatum*	0.012 ± 0.0037
Bacteroides uniformis	0.015 ± 0.023	*Ruminococcus bromii*	0.016 ± 0.026	*Coriobacteriaceae unclassified*	0.011 ± 0.020
Ruminococcaceae unclassified	0.011 ± 0.012	*[Ruminococcus] unclassified*	0.008 ± 0.011	*Coprococcus unclassified*	0.008 ± 0.010
[Ruminococcus] unclassified	0.010 ± 0.012	*Coprococcus unclassified*	0.005 ± 0.007	*Ruminococcus bromii*	0.007 ± 0.012
Bacteroides ovatus	0.010 ± 0.014	*Bacteroides unclassified*	0.005 ± 0.012	*Fusicatenibacter saccharivorans*	0.007 ± 0.012
Rikenellaceae unclassified	0.008 ± 0.017	*Bacteroides vulgatus*	0.004 ± 0.010	*[Ruminococcus] unclassified*	0.006 ± 0.009
Prevotella unclassified	0.008 ± 0.033	*Dorea longicatena*	0.004 ± 0.005	*Dorea longicatena*	0.004 ± 0.006
Ruminococcus bromii	0.008 ± 0.015	*Bacteroides uniformis*	0.004 ± 0.008	*Senegalimassilia anaerobia*	0.004 ± 0.014
Anaerostipes hadrus	0.007 ± 0.008	*Dorea unclassified*	0.004 ± 0.007	*Dorea unclassified*	0.003 ± 0.007
Dorea unclassified	0.007 ± 0.008	*Erysipelotrichaceae unclassified*	0.003 ± 0.017	*Streptococcus thermophilus*	0.002 ± 0.006
Dorea longicatena	0.007 ± 0.007	*Lachnospira unclassified*	0.003 ± 0.008	*Erysipelotrichaceae unclassified*	0.002 ± 0.014
Lachnospira unclassified	0.007 ± 0.010	*Parabacteroides unclassified*	0.002 ± 0.006	*Anaerostipes hadrus*	0.002 ± 0.006
Parabacteroides distasonis	0.005 ± 0.008	*Bacteroides ovatus*	0.002 ± 0.004	*Lachnospira unclassified*	0.002 ± 0.003
Parabacteroides unclassified	0.004 ± 0.008	*Streptococcus thermophilus*	0.002 ± 0.007	*Catenibacterium mitsuokai*	0.002 ± 0.005
S24-7 unclassified	0.003 ± 0.010	*Anaerostipes hadrus*	0.001 ± 0.002	*[Mogibacteriaceae] unclassified*	0.001 ± 0.003
Streptococcus thermophilus	0.003 ± 0.010	*Coprococcus eutactus*	0.001 ± 0.002	*Coprococcus eutactus*	0.001 ± 0.003
Bilophila unclassified	0.003 ± 0.004	*[Mogibacteriaceae] unclassified*	0.001 ± 0.001	*Clostridium unclassified*	0.001 ± 0.004
Erysipelotrichaceae unclassified	0.002 ± 0.005	*Catenibacterium mitsuokai*	0.001 ± 0.003	*Bacteroides unclassified*	0.001 ± 0.004
Clostridium unclassified	0.002 ± 0.004	*Clostridium unclassified*	0.001 ± 0.002	Parabacteroides unclassified	0.001 ± 0.002
Coprococcus eutactus	0.002 ± 0.006	*Clostridium celatum*	0.001 ± 0.003	*Bacteroides vulgatus*	0.000 ± 0.003
[Mogibacteriaceae] unclassified	0.001 ± 0.002	*Coriobacteriaceae unclassified*	0.001 ± 0.001	*Bacteroides ovatus*	0.000 ± 0.001
Coriobacteriaceae unclassified	0.001 ± 0.002	*Bilophila unclassified*	0.000 ± 0.001	*Rikenellaceae unclassified*	0.000 ± 0.000
Catenibacterium mitsuokai	0.001 ± 0.003	*Senegalimassilia anaerobia*	0.000 ± 0.000	*Bacteroides uniformis*	0.000 ± 0.000
Senegalimassilia anaerobia	0.00 ± 0.002	*Parabacteroides distasonis*	0.000 ± 0.001	*Parabacteroides distasonis*	0.000 ± 0.000
Clostridium celatum	0.00 ± 0.001	*Rikenellaceae unclassified*	0.001 ± 0.001	*Bilophila unclassified*	0.000 ± 0.000

RA = Relative Abundance

Relative abundance of the bacterial species with a differential abundance in HIV-uninfected individuals between cohorts (Kruskal-Wallis test with Bonferroni multiple comparison correction p-value<0.05) (Figure 1C). In each cohort, bacterial species are ordered from most to least abundant and color-code highlights the top 5 most abundant species in all three cohorts.

Supplementary Table 5. Additional metadata correlation (non-MSM, all geographic cohorts)

	HIV-uninfected vs HIV+ ART-treated				HIV-uninfected vs HIV+ untreated			
	All metadata available (n = 427)		All expanded metadata available (n = 405)		All metadata available (n = 217)		All expanded metadata available (n = 201)	
	R^2	P	R^2	P	R^2	P	R^2	P
Geography	1.73%	0.0009***	1.45%	0.0009***	1.03%	0.0009***	0.87%	0.002**
HIV-Infection	0.63%	0.0009***	0.46%	0.0009***	0.57%	0.041*	0.56%	0.12
Sex	0.40%	0.0009***	0.50%	0.0009***	0.54%	0.096	0.59%	0.062
Age	0.86%	0.0009***	0.75%	0.0009***	0.73%	0.003**	1.16%	0.0009***
Race	3.06%	0.0009***	2.28%	0.0009***	2.07%	0.022*	3.55%	0.0009***
Ethnicity	0.29%	0.024*	0.31%	0.025*	0.41%	0.68	0.54%	0.14
Current ART (by class)	0.89%	0.0009***	0.98%	0.0009***	-	-	-	-
On TMP-SMX Prophylaxis	0.94%	0.0009***	0.64%	0.0009***	-	-	-	-
BMI	-	-	0.62%	0.0009***	-	-	0.71%	0.007**
Time on ART (years)	-	-	0.59%	0.0009***	-	-	-	-
History of CVD (self-reported)	-	-	0.39%	0.0009***	-	-	0.66%	0.016*
History of CVD (self-reported or determined from available data)	-	-	0.43%	0.0009***	-	-	0.67%	0.015*
History of HLD (self-reported)	-	-	0.29%	0.058	-	-	0.57%	0.12
History of HLD (self-reported or determined from available data)	-	-	0.34%	0.008**	-	-	0.79%	0.0009***
History of hypertension (self-reported)	-	-	0.33%	0.009**	-	-	0.47%	0.48
History of Type 2 DM (self-reported or determined from available data)	-	-	0.28%	0.09	-	-	0.52%	0.21

Controlling for available metadata from Figure 2A (non-MSM subjects, all geographic cohorts) performed on unweighted UniFrac distances calculated at the ASV level. R^2 and p-value were obtained for each category of metadata available using a multivariate PERMANOVA analysis. When adding in metadata available for all subjects and metadata available for a substantial fraction of subjects, both geography and HIV-infection status remained significant in the comparison between HIV-uninfected and HIV-infected ART-treated subjects, while HIV-infection status was no longer significant in the comparison between HIV-uninfected and HIV-infected ART-untreated subjects. Metadata coding is defined in Supplementary Data 1. ART (anti-retroviral therapy), TMP-SMX (trimethoprim-sulfamethoxazole), CVD (cardiovascular disease), HLD (hyperlipidemia), DM (diabetes mellitus). ***: $p < 0.001$, **: $p < 0.01$, *: $p < 0.05$

Supplementary Table 6. Additional metadata correlation (female-only analysis)

	HIV-uninfected vs HIV+ ART-treated				HIV-uninfected vs HIV+ untreated			
	All metadata available (n = 205)		All expanded metadata available (n = 190)		All metadata available (n = 102)		All expanded metadata available (n = 91)	
	R^2	P	R^2	P	R^2	P	R^2	P
Geography	2.29%	0.0009***	1.90%	0.0009***	2.03%	0.0009***	1.64%	0.005**
HIV-Infection	0.86%	0.0009***	0.88%	0.0009***	0.97%	0.39	1.04%	0.51
Age	1.24%	0.0009***	1.19%	0.0009***	1.36%	0.013*	1.35%	0.049*
Race	3.79%	0.0009***	2.97%	0.0009***	5.78%	0.0009***	5.86%	0.0009***
Ethnicity	0.59%	0.034*	0.60%	0.068	0.98%	0.37	1.04%	0.52
Current ART (by class)	1.45%	0.31	1.46%	0.62	-	-	-	-
On TMP-SMX Prophylaxis	1.16%	0.0009***	0.84%	0.002**	-	-	-	-
BMI	-	-	0.61%	0.055	-	-	0.98%	0.72
Time on ART (years)	-	-	0.84%	0.002**	-	-	-	-
History of CVD (self-reported)	-	-	0.65%	0.034*	-	-	1.51%	0.015*
History of CVD (self-reported or determined from available data)	-	-	0.83%	0.0009***	-	-	1.09%	0.37
History of HLD (self-reported)	-	-	0.68%	0.026*	-	-	1.03%	0.52
History of HLD (self-reported or determined from available data)	-	-	0.78%	0.002**	-	-	1.66%	0.0009***
History of hypertension (self-reported)	-	-	0.82%	0.003**	-	-	1.20%	0.17
History of Type 2 DM (self-reported or determined from available data)	-	-	0.63%	0.047*	-	-	1.26%	0.09

Controlling for available metadata from Supplementary Figure 1C (female-only, all subjects) performed on unweighted UniFrac distances calculated at the ASV level. R^2 and p-value were obtained for each category of metadata available using a multivariate PERMANOVA analysis. When adding in metadata available for all subjects and metadata available for a substantial fraction of subjects, both geography and HIV-infection status remained significant in the comparison between HIV-uninfected and HIV-infected ART-treated subjects. In the comparison between HIV-uninfected and HIV-infected ART-untreated subjects, geography remained significant and HIV-infection status remained non-significant. Metadata coding is defined in Supplementary Data 1. ART (anti-retroviral therapy), TMP-SMX (trimethoprim-sulfamethoxazole), CVD (cardiovascular disease), HLD (hyperlipidemia), DM (diabetes mellitus). ***: $p < 0.001$, **: $p < 0.01$, *: $p < 0.05$

Supplementary Table 7. Additional metadata correlation (male-only analysis)

	HIV-uninfected vs HIV+ ART-treated				HIV-uninfected vs HIV+ untreated			
	All metadata available (n = 222)		All expanded metadata available (n = 215)		All metadata available (n = 115)		All expanded metadata available (n = 110)	
	R^2	P	R^2	P	R^2	P	R^2	P
Geography	2.00%	0.0009***	1.89%	0.0009***	1.12%	0.037*	1.16%	0.03*
HIV-Infection	0.91%	0.0009***	0.56%	0.03*	1.05%	0.058	1.03%	0.11
Age	1.10%	0.0009***	0.93%	0.0009***	2.01%	0.0009***	1.57%	0.002**
Race	3.56%	0.0009***	2.57%	0.0009***	4.70%	0.0009***	4.38%	0.0009***
Ethnicity	0.46%	0.25	0.43%	0.50	0.96%	0.19	0.90%	0.37
Current ART (by class)	1.63%	0.003**	1.70%	0.0009***	-	-	-	-
On TMP-SMX Prophylaxis	1.16%	0.0009***	0.90%	0.0009***	-	-	-	-
BMI	-	-	1.57%	0.0009***	-	-	1.54%	0.002**
Time on ART (years)	-	-	0.87%	0.0009***	-	-	-	-
History of CVD (self-reported)	-	-	0.56%	0.034*	-	-	1.06%	0.068
History of CVD (self-reported or determined from available data)	-	-	0.85%	0.0009***	-	-	1.03%	0.11
History of HLD (self-reported)	-	-	0.45%	0.33	-	-	0.84%	0.56
History of HLD (self-reported or determined from available data)	-	-	0.63%	0.009**	-	-	1.20%	0.024*
History of hypertension (self-reported)	-	-	0.42%	0.52	-	-	0.79%	0.73
History of Type 2 DM (self-reported or determined from available data)	-	-	0.50%	0.14	-	-	1.09%	0.065

Controlling for available metadata from Supplementary Figure 2C (male-only, all subjects) performed on unweighted UniFrac distances calculated at the ASV level. R^2 and p-value were obtained for each category of metadata available using a multivariate PERMANOVA analysis. When adding in metadata available for all subjects and metadata available for a substantial fraction of subjects, both geography and HIV-infection status remained significant in the comparison between HIV-uninfected and HIV-infected ART-treated subjects. In the comparison between HIV-uninfected and HIV-infected ART-untreated subjects, geography remained significant while HIV-infection status was no longer significant. Metadata coding is defined in Supplementary Data 1. ART (anti-retroviral therapy), TMP-SMX (trimethoprim-sulfamethoxazole), CVD (cardiovascular disease), HLD (hyperlipidemia), DM (diabetes mellitus). ***: $p < 0.001$, **: $p < 0.01$, *: $p < 0.05$

Supplementary Table 8. Additional metadata correlation (Boston)

	HIV-uninfected vs HIV+ ART-treated		HIV-uninfected vs HIV+ untreated	
	All metadata available (n = 104)		All metadata available (n = 96)	
	R^2	P	R^2	P
HIV-Infection	0.86%	0.79	0.98%	0.61
Sex	1.20%	0.043*	1.25%	0.08
Age	1.96%	0.0009***	1.92%	0.0009***
Race	4.89%	0.32	4.88%	0.019*
Ethnicity	1.13%	0.075	1.05%	0.39
Current ART (by class)	3.25%	0.058	-	-

Controlling for available from Supplementary Figure 3A (non-MSM subjects, Boston) performed on unweighted UniFrac distances calculated at the ASV level. R^2 and p-value were obtained for each category of metadata available using a multivariate PERMANOVA analysis. When adding in metadata available for all subjects HIV-infection status remained non-significant in both the comparison between HIV-uninfected and HIV-infected ART-treated subjects, and in the comparison between HIV-uninfected and HIV-infected ART-untreated subjects. Metadata coding is defined in Supplementary Data 1. ART (anti-retroviral therapy). ***: $p < 0.001$, **: $p < 0.01$, *: $p < 0.05$

Supplementary Table 9. Additional metadata correlation (Botswana)

	HIV-uninfected vs HIV+ ART-treated				HIV-uninfected vs HIV+ untreated			
	All metadata available (n = 153)		All expanded metadata available (n = 140)		All metadata available (n = 121)		All expanded metadata available (n = 118)	
	R^2	P	R^2	P	R^2	P	R^2	P
HIV-Infection	1.17%	0.002**	1.40%	0.0009***	0.98%	0.11	0.99%	0.13
Sex	1.01%	0.004**	0.90%	0.038*	0.96%	0.12	0.79%	0.63
Age	0.83%	0.046*	0.90%	0.043*	1.06%	0.04*	1.35%	0.004**
Race	- Uniform (All Black African) -							
Ethnicity	- Uniform (All Non-Hispanic or Latino) -							
Current ART (by class)	1.56%	0.04*	1.71%	0.03*	-	-	-	-
On TMP-SMX Prophylaxis	- Uniform (None) -							
BMI	-	-	0.78%	0.18	0.91%	0.21	0.88%	0.35
Time on ART (years)	0.64%	0.51	0.71%	0.44	-	-	-	-
History of CVD (self-reported)	0.73%	0.18	0.84%	0.09	0.89%	0.25	0.76%	0.76
History of HLD (self-reported)	0.72%	0.17	0.70%	0.50	0.69%	0.90	0.75%	0.77
History of HLD (self-reported or determined from available data)	0.61%	0.68	0.58%	0.95	0.74%	0.77	0.80%	0.59
History of hypertension (self-reported)	0.62%	0.60	0.64%	0.74	0.76%	0.72	0.75%	0.80
History of Type 2 DM (self-reported or determined from available data)	0.58%	0.81	0.66%	0.72	0.78%	0.67	0.88%	0.34
History of chronic kidney disease (self-reported)	0.64%	0.51	0.69%	0.56	- Uniform (None) -			
Smoking status	1.35%	0.31	1.33%	0.75	2.12%	0.009**	1.67%	0.50
Mean Carotid Intima-Media Thickness (mm)	0.58%	0.80	0.69%	0.58	0.77%	0.66	0.89%	0.27
Presence of carotid plaque	0.60%	0.71	0.65%	0.75	0.77%	0.67	0.83%	0.53
Number of carotid plaques	0.60%	0.70	0.65%	0.73	0.71%	0.87	0.74%	0.82
Duration of smoking (years)	-	-	0.99%	0.01**	-	-	1.36%	0.003**
Framingham Risk Score (without laboratory component)	-	-	0.68%	0.60	-	-	0.84%	0.49
Framingham Risk Score (with laboratory component)	-	-	0.74%	0.33	-	-	0.83%	0.50

Controlling for available metadata from Supplementary Figure 3A (Botswana) performed on unweighted UniFrac distances calculated at the ASV level. R^2 and p-value were obtained for each category of metadata available using a multivariate PERMANOVA analysis. When adding in metadata available for all subjects and metadata available for a substantial fraction of subjects, HIV-infection status remained significant in the comparison between HIV-uninfected and HIV-infected ART-treated subjects. HIV-infection status remained non-significant in the comparison between HIV-uninfected and HIV-infected ART-untreated subjects. Metadata coding is defined in Supplementary Data 1. ART (anti-retroviral therapy), TMP-SMX (trimethoprim-sulfamethoxazole), CVD (cardiovascular disease), HLD (hyperlipidemia), DM (diabetes mellitus). ***: $p < 0.001$, **: $p < 0.01$, *: $p < 0.05$

Supplementary Table 10. Additional metadata correlation (Uganda)

	HIV-uninfected vs HIV+ ART-treated			
	All metadata available (n = 170)		All expanded metadata available (n = 150)	
	R^2	P	R^2	P
HIV-Infection	2.13%	0.0009***	1.55%	0.0009***
Sex	0.77%	0.039*	0.79%	0.10
Age	0.71%	0.077	0.60%	0.67
Race	- Uniform (All Black African) -			
Ethnicity	- Uniform (All Non-Hispanic or Latino) -			
Current ART (by class)	0.77%	0.04*	0.99%	0.006**
On TMP-SMX Prophylaxis	0.47%	0.93	0.52%	0.96
BMI	0.62%	0.28	0.77%	0.11
Time on ART (years)	0.68%	0.13	0.73%	0.19
History of CVD (self-reported)	0.49%	0.87	0.53%	0.93
History of CVD (self-reported or determined from available data)	0.56%	0.50	0.78%	0.09
History of HLD (self-reported)	0.76%	0.04*	0.78%	0.12
History of HLD (self-reported or determined from available data)	0.53%	0.69	0.62%	0.63
History of hypertension (self-reported or determined from available data)	0.54%	0.61	0.67%	0.39
History of Type 2 DM (self-reported or determined from available data)	0.76%	0.036*	0.96%	0.013*
History of chronic kidney disease (self-reported)	0.52%	0.77	0.60%	0.69
Smoking status	1.15%	0.47	1.29%	0.55
Mean Carotid Intima-Media Thickness (mm)	0.50%	0.82	0.51%	0.97
Presence of carotid plaque	0.55%	0.59	0.66%	0.43
Number of carotid plaques	0.69%	0.11	0.57%	0.81
Duration of smoking (years)	0.58%	0.44	0.52%	0.95
Framingham Risk Score (without laboratory component)	-	-	0.74%	0.19
Framingham Risk Score (with laboratory component)	-	-	0.68%	0.35
Level of schooling	-	-	4.20%	0.13
Monthly income	-	-	0.79%	0.09
Food security	-	-	2.50%	0.74
Electricity at home	-	-	0.78%	0.11
Toilet facilities at home	-	-	2.70%	0.32
Home wall material	-	-	2.17%	0.12
Home floor material	-	-	2.16%	0.14

Controlling for available metadata from Supplementary Figure 3A (Uganda) performed on unweighted UniFrac distances calculated at the ASV level. R^2 and p-value were obtained for each category of metadata available using a multivariate PERMANOVA analysis. When adding in metadata available for all subjects and metadata available for a substantial fraction of subjects, HIV-infection status remained significant in the comparison between HIV-uninfected and HIV-infected ART-treated subjects. Metadata coding is defined in Supplementary Table 3. ART (anti-retroviral therapy), TMP-SMX (trimethoprim-sulfamethoxazole), CVD (cardiovascular disease), HLD (hyperlipidemia), DM (diabetes mellitus). ***: $p < 0.001$, **: $p < 0.01$, *: $p < 0.05$

Supplementary Table 11. Demographic characteristics of subjects in U.S. cohorts

		HIV-uninfected (n=117)	HIV-infected ART-treated (n=61)	HIV-infected ART-untreated (n=55)	P value
Gender (Female/Male MSM/Male non-MSM)		37/32/48	9/42/10	7/44/4	<0.00001
Race (Black or African-American/White/ AmericanIndian_Alaska/ Asian/Multiple/Unknown)		16/89/1/5/4/2	22/35/1/0/3/0	11/37/0/2/5/0	0.035
Ethnicity (Non-Hispanic or Latino/ Hispanic or Latino)		107/10	54/7	48/7	0.66
Age in years (IQR)		48 (31, 55)	54 (47, 58)	52 (38.5, 54)	0.028
Weight in kg (IQR)		80 (68.5, 89.8)<br>n = 106	86 (77, 95)<br>n = 57	83 (74, 99)<br>n = 45	0.04
Height in cm (IQR)		175 (170, 178)<br>n = 106	178 (170, 183)<br>n = 57	175 (168, 178)<br>n = 43	0.08
Body Mass Index (BMI) in kg/cm^2 (IQR)		25.5 (23, 28.4)<br>n = 106	27.3 (24.3, 30.1)<br>n = 57	27.7 (25.2, 31.6)<br>n = 42	0.015
History or Diagnosis of Comorbidity (n, %)<br><br>(Total n = 103/54/43 for Negative/ART/ART-naïve, respectively)	Hyperlipidemia	8 (7.8)	18 (33.3)	6 (14)	0.0002
	Hypertension	8 (7.8)	19 (35.2)	10 (23.3)	0.0001
	Cardiovascular Disease	4 (3.9)	3 (5.6)	1 (2.3)	0.72
	Diabetes	6 (5.8)	4 (7.4)	2 (4.7)	0.85
Current CD4 T-cell counts in cells/ml (IQR)		946 (625, 1194)<br>n = 25	722.5 (507.2, 918)<br>n = 60	548 (440.8, 775)<br>n = 54	0.0001
Nadir CD4 T-cell counts in cells/ml (IQR)		933 (559, 1148)<br>n = 25	361.5 (276.5, 583)<br>n = 52	402 (347.5, 557)<br>n = 50	<0.00001

For numeric variables, median and IQR values are shown. P-values for continuous and discrete variables are calculated with Kruskal-Wallis and Chi-square test, respectively. Some data were collected from less than the complete cohort, these are notated by n of the number of subjects from which they were collected.

Supplementary Table 12. Demographic characteristics of subjects in Botswana cohorts

		HIV-uninfected (n=80)	HIV-infected ART-treated (n=73)	HIV-infected ART-untreated (n=41)	P value
Gender (Female/Male)		36/44	38/35	22/19	0.57
Race		- All Black African -			
Ethnicity (Non-Hispanic or Latino/ Hispanic or Latino)		- All Non-Hispanic or Latino -			
Age in years (IQR)		38 (33.8, 42)	39 (36, 41)	37 (34, 42)	0.23
Weight in kg (IQR)		66.5 (56, 78.2)	68 (58.5, 76.2)	61 (55, 72)	0.20
Height in cm (IQR)		167 (161.8, 173)	166 (160.8, 174)	165 (160, 172)	0.70
Body Mass Index (BMI) in kg/cm^2 (IQR)		23.3 (20.5, 28)	23.9 (20.6, 27.8)	22.1 (19.8, 26)	0.38
History or Diagnosis of Comorbidity (n, %)	Hyperlipidemia	22 (27.5)	28 (38.4)	6 (14.6)	0.026
	Hypertension	9 (11.2)	11 (15.1)	1 (2.4)	0.11
	Cardiovascular Disease	3 (3.8)	3 (4.1)	4 (9.8)	0.32
	Diabetes	4 (5)	1 (1.4)	3 (7.3)	0.27
Current CD4 T-cell counts in cells/ml (IQR)		NA	493 (389, 694)	361 (158, 531)	0.0002
Nadir CD4 T-cell counts in cells/ml (IQR)		NA	117 (73, 180)	NA	-

For numeric variables, median and IQR values are shown. P-values for continuous and discrete variables are calculated with Kruskal-Wallis and Chi-square test, respectively.

Supplementary Table 13. Demographic characteristics of subjects in Uganda cohorts

		HIV-uninfected (n=80)	HIV-infected ART-treated (n=90)	P value
Gender (Female/Male)		39/41	46/44	0.88
Race		- All Black African -		
Ethnicity (Non-Hispanic or Latino/ Hispanic or Latino)		- All Non-Hispanic or Latino -		
Age in years (IQR)		50 (46, 54)	49 (45, 51)	0.10
Weight in kg (IQR)		58 (52.8, 69)	60 (54, 64.8)	0.96
Height in cm (IQR)		161.5 (156.8, 169)	162 (158, 169.8)	0.39
Body Mass Index (BMI) in kg/cm^2 (IQR)		22.2 (19.5, 25)	21.6 (19.9, 24.4)	0.58
History or Diagnosis of Comorbidity (n, %)	Hyperlipidemia	14 (17.5)	11 (12.2)	0.45
	Hypertension	22 (27.5)	15 (16.7)	0.13
	Cardiovascular Disease	25 (31.2)	24 (26.7)	0.62
	Diabetes	7 (8.8)	5 (5.6)	0.61
Current CD4 T-cell counts in cells/ml (IQR)		NA	430 (336, 552.8)	-
Nadir CD4 T-cell counts in cells/ml (IQR)		NA	123 (85.2, 173.2)	-

For numeric variables, median and IQR values are shown. P-values for continuous and discrete variables are calculated with Kruskal-Wallis and Chi-square test, respectively.

Supplementary Table 14. Additional metadata correlation (MSM)

	HIV-uninfected vs HIV+ ART-treated				HIV-uninfected vs HIV+ untreated			
	All metadata available (n = 74)		All expanded metadata available (n = 62)		All metadata available (n = 76)		All expanded metadata available (n = 65)	
	R^2	P	R^2	P	R^2	P	R^2	P
HIV-Infection	4.93%	0.0009***	3.28%	0.0009***	4.34%	0.0009***	4.53%	0.0009***
Age	1.35%	0.31	1.73%	0.19	1.69%	0.065	1.96%	0.046*
Race	7.04%	0.0009***	6.85%	0.098	6.09%	0.026*	7.18%	0.008**
Ethnicity	1.39%	0.26	1.39%	0.63	1.21%	0.57	1.12%	0.97
Current ART (by class)	-	-	3.18%	0.31	-	-	-	-
BMI	-	-	1.91%	0.107	-	-	1.88%	0.068
Time on ART (years)	-	-	3.97%	0.0009***	-	-	-	-
History of CVD (self-reported or determined from available data)	-	-	1.69%	0.22	-	-	2.01%	0.037*
History of HLD (self-reported or determined from available data)	-	-	2.14%	0.042*	-	-	1.71%	0.15
History of hypertension (self-reported)	-	-	1.96%	0.09	-	-	1.52%	0.37
History of Type 2 DM (self-reported or determined from available data)	-	-	1.78%	0.18	-	-	1.49%	0.37

Controlling for available metadata from Figure 3A (MSM subjects) performed on unweighted UniFrac distances calculated at the ASV level. R^2 and p-value were obtained for each category of metadata available using a multivariate PERMANOVA analysis. When adding in metadata available for all subjects and metadata available for a substantial fraction of subjects, HIV-infection status remained significant in the comparison between MSM HIV-uninfected and HIV-infected ART-treated subjects as well as between MSM HIV-uninfected and HIV-infected ART-untreated subjects. Metadata coding is defined in Supplementary Data 1. ART (anti-retroviral therapy), TMP-SMX (trimethoprim-sulfamethoxazole), CVD (cardiovascular disease), HLD (hyperlipidemia), DM (diabetes mellitus). ***: $p < 0.001$, **: $p < 0.01$, *: $p < 0.05$
