# Supplementary material for: HIV-associated gut microbial alterations are dependent on host and geographic context
Source: Nat Commun. 2024 Feb 5;15:1055. doi: 10.1038/s41467-023-44566-4 (PMC10844288; doi:10.1038/s41467-023-44566-4)
Supplement: Supplementary file 2 — Supplementary Data and Figures Description [file 41467_2023_44566_MOESM2_ESM.docx]

**Description of Additional Supplementary Files**

**File Name: Supplementary Figure 1. Female-only microbiota analysis**

**Description:** Recapitulation of the analyses performed in Figures 1A, 1B, 2A, and Supplemental Figure 3B including only female subjects in order to control for confounding due to sexual behavior. A) Re-analysis of Figure 1A: PCoA of unweighted UniFrac distances calculated at the ASV (amplicon sequence variant) level, clustered by geography. n = 112 biologically independent samples. Boxplots are displayed with median as center value with box as IQR and whiskers as minima and maxima. PCoA significance calculated by PERMANOVA; F(df = 2) = 4.00, p = 0.0009, R2 = 6.8%. Boxplot significance calculated by Kruskal-Wallis test with Bonferroni correction (*** denotes p-value < 0.001); chi-squared(df = 2) = 75.6, p < 0.0001; pairwise comparisons: Botswana - U.S. Z = 5.72 (p < 0.0001), Uganda - U.S. Z = 7.75 (p < 0.0001), Botswana - Uganda Z = 8.31 (p < 0.0001). These findings remain significant when corrected for metadata available (see Supplementary Table 2 for complete results). B) Re-analysis of Figure 1B: Relative abundance of ASVs in the families Prevotellaceae and Bacteroidaceae plotted by Axis 1 position in subfigure A, colored by geography. These families were significantly different in relative abundance amongst all cohorts as measured by Kruskal-Wallis test with Bonferroni correction (Prevotellaceae: chi-squared(df = 2) = 30.9, p < 0.001; Bacteroidaceae: chi-squared(df = 2) = 67.2, p < 0.001). C) Re-analysis of Figure 2A: PCoA of unweighted UniFrac distances calculated at the ASV level, clustered by geography (separate panels) and HIV phenotype for both HIV-uninfected and ART-treated HIV-infected subjects (left) and HIV-uninfected and untreated HIV-infected subjects (right). PCoA significance calculated by PERMANOVA; HIV-uninfected vs HIV+ ART-treated (n = 205 biologically independent samples): geography F(df = 2) = 5.55, p = 0.0009, R2=5.2%; and HIV infection F(df = 1) = 1.84, p = 0.0009, R2=0.9%. HIV-uninfected vs HIV+ untreated (n = 102 biologically independent samples): geography F(df = 1) = 4.19, p = 0.0009, R2=4.0%; and HIV infection F(df = 1) = 1.06, p = 0.28, R2=1.0%. For the comparison between HIV-uninfected and HIV-infected ART-treated subjects, the associations for both factors remain significant when controlling for metadata available. For the comparison between HIV-uninfected and HIV-infected ART-untreated subjects, the association for geography remained significant while HIV remained not significant when controlling for metadata available (see Supplementary Table 6 for complete results). D) Re-analysis of the data presented in Supplemental Figure 3B: Comparing observed richness and Shannon diversity index for the three HIV infection groups in each of the cohorts. n = 234 biologically independent samples. Boxplots are displayed with median as center value with box as IQR and whiskers as minima and maxima. Boxplot significance Kruskal-Wallis with Bonferroni correction (three groups) or Wilcoxon (two groups) testing; Uganda/Observed Wilcoxon W = 1148, p = 0.027; Uganda/Shannon Wilcoxon W = 1203, p = 0.0067.

**File Name: Supplementary Figure 2. Male-only microbiota analysis**

**Description:** Recapitulation of the analyses performed in Figures 1A, 1B, 2A, and Supplemental Figure 3B including only male subjects in order to control for confounding due to sexual behavior. A) Re-analysis of Figure 1A: PCoA of unweighted UniFrac distances calculated at the ASV (amplicon sequence variant) level, clustered by geography. n = 133 biologically independent samples. Boxplots are displayed with median as center value with box as IQR and whiskers as minima and maxima. PCoA significance calculated by PERMANOVA; F(df = 2) = 4.72, p = 0.0009, R2 = 6.7%. Boxplot significance calculated by Kruskal-Wallis test with Bonferroni correction (*** denotes p-value < 0.001); chi-squared(df = 2) = 127.7, p < 0.0001; pairwise comparisons: Botswana - U.S. Z = 6.46 (p < 0.0001), Uganda - U.S. Z = 9.69 (p < 0.0001), Botswana - Uganda Z = 7.31 (p < 0.0001). These findings remain significant when corrected for metadata available (see Supplementary Table 3 for complete results). B) Re-analysis of Figure 1B: Relative abundance of ASVs in the families Prevotellaceae and Bacteroidaceae plotted by Axis 1 position in subfigure A, colored by geography. These families were significantly different in relative abundance amongst all cohorts as measured by Kruskal-Wallis test with Bonferroni correction (Prevotellaceae: chi-squared(df = 2) = 30.4, p < 0.001; Bacteroidaceae: chi-squared(df = 2) = 99.1, p < 0.001). C) Re-analysis of Figure 2A: PCoA of unweighted UniFrac distances calculated at the ASV level, clustered by geography (separate panels) and HIV phenotype for both HIV-uninfected and ART-treated HIV-infected subjects (left) and HIV-uninfected and untreated HIV-infected subjects (right). PCoA significance calculated by PERMANOVA; HIV-uninfected vs HIV+ ART-treated (n = 222 biologically independent samples): geography F(df = 2) = 6.44, p = 0.0009, R2=5.6%; and HIV infection F(df = 1) = 2.20, p = 0.0009, R2=1.0%. HIV-uninfected vs HIV+ untreated (n = 115 biologically independent samples): geography F(df = 1) = 4.44, p = 0.0009, R2=3.8%; and HIV infection F(df = 1) = 1.42, p = 0.016, R2=1.2%. For the comparison between HIV-uninfected and HIV-infected ART-treated subjects, the associations for both factors remain significant when controlling for metadata available. For the comparison between HIV-uninfected and HIV-infected ART-untreated subjects, the association for geography remained significant while HIV was no longer significant when both controlling for metadata available (see Supplementary Table 7 for complete results). D) Re-analysis of the data presented in Supplemental Figure 3B: Comparing observed richness and Shannon diversity index for the three HIV infection groups in each of the cohorts. n = 245 biologically independent samples. Boxplots are displayed with median as center value with box as IQR and whiskers as minima and maxima.

**File Name: Supplementary Figure 3. Each geographic location has a unique HIV infection-associated gut microbial composition**

**Description:** A) PCoA ordination plots using unweighted UniFrac distances at the ASV (amplicon sequence variant) level comparing both ART (antiretroviral)-treated and -untreated HIV infection to the corresponding HIV-uninfected controls for each cohort. PCoA significance calculated by PERMANOVA, U.S./ART-treated(n = 104 biologically independent samples): F(df = 1) = 1.06, p = 0.27, R2 = 1.0%; U.S./untreated(n = 96 biologically independent samples): F(df = 1) = 1.08, p = 0.23, R2 = 1.1%; Botswana/ART-treated(n = 153 biologically independent samples): F(df = 1) = 1.72, p = 0.002, R2 = 1.1%; Botswana/untreated(n = 121 biologically independent samples): F(df = 1) = 1.21, p = 0.08, R2 = 1.1%; Uganda(n = 170 biologically independent samples): F(df = 1) = 3.68, p = 0.0009, R2 = 2.1%. The significant observed microbiota differences between ART-treated HIV-infected individuals and HIV-uninfected subjects in both Botswana and Uganda were robust to controlling for metadata available. The findings in the other cohorts were unchanged when controlling for available metadata (see Supplementary Tables 8, 9, and 10 for complete results). B) Boxplots showing the observed richness and Shannon diversity index for the three HIV infection groups in each of the cohorts. n = 479 biologically independent samples. Boxplots are displayed with median as center value with box as IQR and whiskers as minima and maxima. Boxplot significance calculated by Kruskal-Wallis with Bonferroni correction (three groups) or Wilcoxon (two groups) testing; Uganda/Shannon Wilcoxon W = 4263, p = 0.036.

**File Name: Supplementary Figure 4. Dietary data for HIV-infected and uninfected individuals in the U.S. and Botswana**

**Description:** Dietary content of macro- and micronutrients calculated from foods reported on a 24-hour recall questionnaire from subjects in A) Botswana (n = 194 biologically independent samples) and B) U.S. (n = 64 biologically independent samples), categorized by HIV infection status. Boxplots are displayed with median as center value with box as IQR and whiskers as minima and maxima. Significance was calculated by Kruskal-Wallis test with Bonferroni correction, with only one comparison (sodium consumption in HIV-uninfected and HIV-infected, ART-treated) reaching significance.

**File Name: Supplementary Figure 5. Differences between ART-treated and -untreated HIV infection differ by location**

**Description:** A) Shared ASVs detected by ANCOM as differentially abundant between ART-treated HIV-infected subjects and HIV-uninfected controls and untreated HIV-infected subjects and HIV-uninfected controls in the U.S. The Log2FC values are represented on x axis and color code represents taxonomic classification. ASVs are identified up to most resolutive taxonomic level. Color brightness relates to the HIV-infected group (dark for ART-treated and light for untreated) There were no shared ASVs in the Botswanan cohort. B) Results from an ANCOM analysis testing for differences between ART-treated and untreated HIV-infected groups in the U.S. The Log2FC values are represented on the x axis for all single ASVs significantly more or less abundant in each of the tested groups. Only ASVs present in at least 5% of the samples in each HIV infection group were considered. Color code represents taxonomic classification at the species level and ASVs are identified up to most resolutive taxonomic level. C) Analyses as in B) but for the Botswanan cohort.

**File Name: Supplementary Figure 6. HIV-associated changes in gut microbiota composition at higher taxonomic ranks are unique**

**Description:** Heatmaps showing on the y axis the bacterial genera (A) and bacterial species (B) with significantly different abundance between ART-treated HIV-infected subjects and uninfected controls in each of the geographical cohorts detected by the ANCOM comparison. Only ASVs present in at least 5% of the samples in each HIV infection group were considered. Color gradient represents the Log2FC value for each ASVs. Bolded ASVs are present in more than one cohort.

**File Name: Supplementary Figure 7. Functional Analysis over PICRUST2 functional inference.**

**Description:** Results from ANCOM analysis testing for differences in functional capability of ART-treated HIV infection compared to the HIV-uninfected controls. The Log2FC values are represented on the x axis for all those functional categories with significantly more or less abundance in each of the tested groups. Pathways with significant differences in more than one cohort are bolded. Color code represents functional classification at level 3 of MetaCyc database. No pathways were significantly different when comparing subjects with untreated HIV infection to those who were HIV-uninfected.

**File Name: Supplementary Data 1.**

**Description:** Full collection of metadata for subjects enrolled in the included studies provided in xlsx format due to multiple tabs. “total” tab is metadata table not including diet data. Boolean values stored as 0 or 1. “headings” tab with human-readable text describing column names, similar to a codebook. “art_coding” tab with explanation of abbreviations used to denote anti-retroviral therapy. “boston_diet” and “botswana_diet” tabs containing processed diet data used in analysis of diet of US and Botswana subjects, respectively.

**File Name: Supplementary Data 2.**

**Description:** Amplicon sequence variant (ASV) table derived from R package DADA2 analysis of 16S V4 fastq files. Contains counts of each ASV present in each sample. ASVs identified by 7 assigned taxonomic levels: domain, phylum, class, order, family, genus, species. Stored in format compatible with R package phyloseq. Provided to allow reproduction of analysis using code provided separately as supplemental material.

**File Name: Supplementary Data 3.**

**Description:** Processed metadata table derived from Supplementary Data 1. Column descriptions found in Supplementary Data 1. Stored in format compatible with R package phyloseq. Provided to allow reproduction of analysis using code provided separately as supplemental material.
