# Supplementary material for: HIV-associated gut microbial alterations are dependent on host and geographic context
Source: Nat Commun. 2024 Feb 5;15:1055. doi: 10.1038/s41467-023-44566-4 (PMC10844288; doi:10.1038/s41467-023-44566-4)
Supplement: Supplementary file 6 — Reporting Summary [file 41467_2023_44566_MOESM6_ESM.pdf]

nature portfolio

Corresponding author(s): Douglas S. Kwon
Muntsa Rocafort

Last updated by author(s): Dec 1, 2023

Reporting Summary

Nature Portfolio wishes to improve the reproducibility of the work that we publish. This form provides structure for consistency and transparency in reporting. For further information on Nature Portfolio policies, see our Editorial Policies and the Editorial Policy Checklist.

Statistics

For all statistical analyses, confirm that the following items are present in the figure legend, table legend, main text, or Methods section.

n/a	Confirmed	
☐	☒	The exact sample size (*n*) for each experimental group/condition, given as a discrete number and unit of measurement
☐	☒	A statement on whether measurements were taken from distinct samples or whether the same sample was measured repeatedly
☐	☒	The statistical test(s) used AND whether they are one- or two-sided<br>*Only common tests should be described solely by name; describe more complex techniques in the Methods section.*
☐	☒	A description of all covariates tested
☐	☒	A description of any assumptions or corrections, such as tests of normality and adjustment for multiple comparisons
☐	☒	A full description of the statistical parameters including central tendency (e.g. means) or other basic estimates (e.g. regression coefficient) AND variation (e.g. standard deviation) or associated estimates of uncertainty (e.g. confidence intervals)
☐	☒	For null hypothesis testing, the test statistic (e.g. *F*, *t*, *r*) with confidence intervals, effect sizes, degrees of freedom and *P* value noted<br>*Give P values as exact values whenever suitable.*
☒	☐	For Bayesian analysis, information on the choice of priors and Markov chain Monte Carlo settings
☒	☐	For hierarchical and complex designs, identification of the appropriate level for tests and full reporting of outcomes
☐	☒	Estimates of effect sizes (e.g. Cohen's *d*, Pearson's *r*), indicating how they were calculated

Our web collection on statistics for biologists contains articles on many of the points above.

Software and code

Policy information about availability of computer code

Data collection	No software was used
Data analysis	DADA2 R/package v1.26.0 was used to process the raw fastq files and obtain the corresponding ASV table. Several other R/ packages were used for 16S rRNA gene data analysis and plotting, including: QIIME v1.91, Phyloseq v1.42.0, ggplot2 v3.4.1, gridExtra v2.3, dplyr v1.1.0, knitr v1.42, reshape v0.8.9, agricolae v1.3-5, Vegan v2.6-4, BiodiversityR v2.15-1 and ANCOMBC v2.0.2.

For manuscripts utilizing custom algorithms or software that are central to the research but not yet described in published literature, software must be made available to editors and reviewers. We strongly encourage code deposition in a community repository (e.g. GitHub). See the Nature Portfolio guidelines for submitting code & software for further information.

Data

Policy information about availability of data

All manuscripts must include a data availability statement. This statement should provide the following information, where applicable:

- Accession codes, unique identifiers, or web links for publicly available datasets
- A description of any restrictions on data availability
- For clinical datasets or third party data, please ensure that the statement adheres to our policy

The metadata and raw 16S rRNA sequences generated in this study have been deposited in the European Nucleotide Archive and are publicly available under the BioProject accession code PRJEB41345 [https://www.ebi.ac.uk/ena/browser/view/PRJEB41345].

The GreenGenes 13.8 taxonomic reference database is publicly available at [http://greengenes.microbio.me/greengenes_release/gg_13_8_otus/].

Research involving human participants, their data, or biological material

Policy information about studies with human participants or human data. See also policy information about sex, gender (identity/presentation), and sexual orientation and race, ethnicity and racism.

Reporting on sex and gender	Single-sex subgroup analysis performed to reduce confounding effect of sexual behavior, included in Supplementary Figures 1 and 2. Significant subgroup analysis was performed on men who reported men who have sex with men behavior (MSM) and is a focus of the manuscript as well as additional material including Figure 3. No significant findings differed by sex or gender. Sex and gender were not considered in the original study design. Sex was self-reported. Disaggregated sex and gender data are available in the provided metadata.
Reporting on race, ethnicity, or other socially relevant groupings	Our manuscript included the categorization variable of subjects who reported men who have sex with men behavior (MSM) which has been shown to be associated with gut microbiota differences and could affect findings in HIV-infected individuals (Noguera-Julian, M. et al. EBioMedicine, 2016). MSM status was self-reported by subjects. We performed separate analysis both including and excluding MSM-reporting individuals in order to control for confounding variables.
Population characteristics	This study includes three large cohorts of HIV-infected and -uninfected individuals spanning two continents and diverse demographic and socioeconomic characteristics: Boston (U.S., n=233), Gaborone (Botswana, n=194) and Mbarara (Uganda, n=170). The cohort in Boston includes 117 HIV-uninfected controls, 61 HIV-infected individuals on suppressive-ART and 55 HIV-infected untreated individuals. This cohort also includes a subgroup of men who engage in sexual relations with other men to study the confounding effect of men-who-have-sex-with-men (MSM) practices on the gut microbiota - HIV infection interaction. The cohort in Botswana includes 80 HIV-uninfected controls, 73 HIV-infected subjects on suppressive-ART and 41 untreated HIV-infected subjects. The cohort based in Uganda comprises 80 HIV-uninfected controls and 90 HIV-infected ART-suppressed individuals. Population density, Gross Regional Income and HIV prevalence were used as the demographic discriminatory variables amongst geographical cohorts. Other clinical variables recruited included sex (self-reported), age, BMI, CD4+ T-cell counts (actual and nadir), time on ART and dietary information. Covariate-relevant population characteristics are described in full in Table 1: Demographic characteristics of individuals in cohorts from the U.S., Botswana and Uganda and Supplementary Tables 11, 12, and 13: Demographic characteristics of subjects in U.S./Botswana/Uganda cohorts.
Recruitment	The cohort in Boston, U.S., enrolled 233 subjects from the Ragon Institute of MGH, MIT and Harvard in Boston, MA, as approved by the Institutional Review Boards at Partners Healthcare. For the cohort in Gaborone, Botswana, 194 subjects were enrolled from the Princess Marina Infectious Disease Clinic and Gaborone Voluntary HIV testing Centre in Botswana as approved by the Institutional Review Boards at Princess Marina Hospital, the Botswana Ministry of Health Research Division, and Partners Healthcare. For the cohort in Uganda, 170 subjects were enrolled from the Mbarara Regional Referral Hospital in Uganda as approved by the Institutional Review Boards at the Mbarara University of Science and Technology, Ugandan National Council of Science and Technology and Partners Healthcare.
Ethics oversight	Samples were collected following informed consent from all participants. Those in the U.S. were consented under protocol reviewed by the Massachusetts General Hospital (MGH) Institutional Review Board (Protocol #2011/P001707). The study in Uganda was approved by the institutional ethics review board of Mbarara University of Science and Technology (MUST-REC) and also received clearance from the Uganda National Council of Science and Technology (UNCST) and the Research Secretariat in the Office of the President of Uganda, in accordance with the national guidelines. The study also received approval from the MGH IRB (Protocol #2014P001928). For subjects in Botswana, ethics clearance was obtained from the Ministry of Health and University of Botswana and the MGH IRB (Protocol #2014P001388)

Note that full information on the approval of the study protocol must also be provided in the manuscript.

Field-specific reporting

Please select the one below that is the best fit for your research. If you are not sure, read the appropriate sections before making your selection.

[x] Life sciences   [ ] Behavioural & social sciences   [ ] Ecological, evolutionary & environmental sciences

For a reference copy of the document with all sections, see nature.com/documents/nr-reporting-summary-flat.pdf

Life sciences study design

All studies must disclose on these points even when the disclosure is negative.

Sample size	Sample size estimates were calculated based upon effect size reported in published studies of microbial community change observed in cohorts of HIV infected persons in the developed world. Overall study size of 597 subjects was several-fold larger than prior studies in HIV fecal microbiome. Each of the three geographic cohorts was on its own notably larger than most prior studies in the field (Gootenberg, et al. Current Opinion in Infectious Diseases, 2017)
Data exclusions	No data was excluded from the analysis.
Replication	A subset of identical samples were processed and sequenced independently to confirm reproducibility of the pipeline. Results were found to be identical. Analysis of 16S data including multiple bioinformatic approaches and comparison of different statistical tests were performed by both first authors and found to produce consistent results. All measures of soluble inflammatory markers were performed in duplicate at the

	University of Vermont (UVM) Laboratory for Clinical Biochemistry Research and values were averaged. All attempts at replication were successful.
Randomization	All samples were randomized prior to processing, sequencing, and measurement of soluble markers. After generation of data for analysis, subjects were grouped based on their HIV-infection status within each geographical cohort (U.S., Botswana and Uganda). Within each geographical cohort subjects were grouped into: HIV-uninfected, HIV-infected on antiretroviral treatment (ART) and HIV-infected untreated.
Blinding	Data was generated using samples that were randomized and blinded prior to processing and measurement.

Reporting for specific materials, systems and methods

We require information from authors about some types of materials, experimental systems and methods used in many studies. Here, indicate whether each material, system or method listed is relevant to your study. If you are not sure if a list item applies to your research, read the appropriate section before selecting a response.

Materials & experimental systems

n/a	Involved in the study
☒	☐ Antibodies
☒	☐ Eukaryotic cell lines
☒	☐ Palaeontology and archaeology
☒	☐ Animals and other organisms
☒	☐ Clinical data
☒	☐ Dual use research of concern
☒	☐ Plants

Methods

n/a	Involved in the study
☒	☐ ChIP-seq
☒	☐ Flow cytometry
☒	☐ MRI-based neuroimaging

Plants

Seed stocks	N/A
Novel plant genotypes	N/A
Authentication	N/A
