# Supplementary material for: HIV-associated gut microbial alterations are dependent on host and geographic context
Source: Nat Commun. 2024 Feb 5;15:1055. doi: 10.1038/s41467-023-44566-4 (PMC10844288; doi:10.1038/s41467-023-44566-4)
Supplement: Supplementary file 7 — Supplementary Table 15 [file 41467_2023_44566_MOESM7_ESM.pdf]

**Supplementary Table 15. Sexual behavior characteristics of individuals in cohorts from the U.S.**

| | HIV Uninfected (n=51) | HIV Infected ART-treated (n=20) | HIV Infected ART-untreated (n=12) |
|---|---|---|---|
| Gender (Female/Male MSM/Male non-MSM) | 6/25/20 | 11/3/6 | 7/2/3 |
| Partner Gender (Men/Women/Both/No Answer) | 22/1/2/26 | 3/4/0/13 | 2/1/1/8 |
| Receptive anal intercourse in the past 2 days (all without a condom, all Male MSM) n, (%) | 3, (6%) | 0 | 0 |
| Receptive anal intercourse in the past 30 days (all Male MSM) n, (%) (Protected/Unprotected/Both/Unknown) | 11, (22%) (2/6/3/0) | 3, (15%) (1/2/0/0) | 3, (25%) (0/1/1/1) |
| Rectal insertion in the past 2 days n, (%) (Female/Male MSM/Male non-MSM) | 6, (12%) (0/6/0) | 2, (10%) (1/1/0) | 1, (8%) (0/1/0) |
| Rectal insertion in the past 30 days n, (%) (Female/Male MSM/Male non-MSM) | 10, (20%) (0/10/0) | 3, (15%) (1/2/0) | 3, (25%) (0/3/0) |
| How often do you use a condom? (always/sometimes/never) | 15/13/23 | 9/9/2 | 3/8/1 |

Results from a survey collected on a subset (n = 83) of the Boston Cohort characterizing sexual behavior, divided by HIV phenotype. All responses are subsetted further by preferred gender and whether the subject self-identifies as MSM (men-who-have-sex-with-men). Additional information pertaining to the responses are contained in corresponding parentheticals.
