# Supplementary material for: HIV-associated gut microbial alterations are dependent on host and geographic context
Source: Nat Commun. 2024 Feb 5;15:1055. doi: 10.1038/s41467-023-44566-4 (PMC10844288; doi:10.1038/s41467-023-44566-4)
Supplement: Supplementary file 8 — Figure1ANCOM_Rocafort-Gootenberg_2022_12_16 [file 41467_2023_44566_MOESM8_ESM.html]

Rocafort-Gootenberg\_Figure1


# Rocafort-Gootenberg\_Figure1

#Load needed R packages

```
library("phyloseq")
library("tidyverse")
```

```
## ── Attaching packages ─────────────────────────────────────── tidyverse 1.3.2 ──
## ✔ ggplot2 3.4.1     ✔ purrr   1.0.1
## ✔ tibble  3.1.8     ✔ dplyr   1.1.0
## ✔ tidyr   1.3.0     ✔ stringr 1.5.0
## ✔ readr   2.1.4     ✔ forcats 1.0.0
## ── Conflicts ────────────────────────────────────────── tidyverse_conflicts() ──
## ✖ dplyr::filter() masks stats::filter()
## ✖ dplyr::lag()    masks stats::lag()
```

```
library("stringr")
library("gridExtra")
```

```
## 
## Attaching package: 'gridExtra'
## 
## The following object is masked from 'package:dplyr':
## 
##     combine
```

```
library("vegan")
```

```
## Loading required package: permute
## Loading required package: lattice
## This is vegan 2.6-4
```

```
library("agricolae")
```

```
## Registered S3 methods overwritten by 'klaR':
##   method      from 
##   predict.rda vegan
##   print.rda   vegan
##   plot.rda    vegan
```

```
library("knitr") 
library("BiodiversityR")
```

```
## Loading required package: tcltk
## BiodiversityR 2.15-1: Use command BiodiversityRGUI() to launch the Graphical User Interface; 
## to see changes use BiodiversityRGUI(changeLog=TRUE, backward.compatibility.messages=TRUE)
```

```
library("reshape")
```

```
## 
## Attaching package: 'reshape'
## 
## The following object is masked from 'package:dplyr':
## 
##     rename
## 
## The following objects are masked from 'package:tidyr':
## 
##     expand, smiths
```

```
library("usedist")
library("rms")
```

```
## Loading required package: Hmisc
## Loading required package: survival
## Loading required package: Formula
## 
## Attaching package: 'Hmisc'
## 
## The following objects are masked from 'package:dplyr':
## 
##     src, summarize
## 
## The following objects are masked from 'package:base':
## 
##     format.pval, units
## 
## 
## Attaching package: 'rms'
## 
## The following object is masked from 'package:vegan':
## 
##     calibrate
```

#Load original phyloseq object output from DADA2 pipeline and put in
new metadata

```
ps_gg_fp_f_prevalence_filter_2019_05_26<-readRDS("ps_gg_fp_f_prevalence_filter_2019_05_26")
readr::read_csv(
  "Metadata_formatted_nat_comm_add_2021_10_24.csv",
  col_names = TRUE,
  col_types = NULL,
  col_select = NULL,
  id = NULL,
  locale = default_locale(),
  na = c("", "NA", "empty", "EMPTY"),
  quote = "\"",
  comment = "",
  trim_ws = TRUE,
  skip = 0,
  name_repair = "unique",
  num_threads = readr_threads(),
  progress = show_progress(),
  show_col_types = should_show_types(),
  skip_empty_rows = TRUE,
  lazy = TRUE
) -> new_metadata
```

```
## Rows: 597 Columns: 88
## ── Column specification ────────────────────────────────────────────────────────
## Delimiter: ","
## chr (26): X, SampleID, subject_id, Race, Ethnicity, unique_id, sequencing_da...
## dbl (62): primer_used, read_count, age, height_cm, height_in, weight_kg, wei...
## 
## ℹ Use `spec()` to retrieve the full column specification for this data.
## ℹ Specify the column types or set `show_col_types = FALSE` to quiet this message.
```

```
### add {SampleID} as rownames
new_metadata_as_sample_data <- phyloseq::sample_data(new_metadata)
phyloseq::sample_names(new_metadata_as_sample_data) <- dplyr::pull(new_metadata, 1)
phyloseq::sample_data(ps_gg_fp_f_prevalence_filter_2019_05_26) <- new_metadata_as_sample_data

#Fix randomness
set.seed(1)
```

```
#FIGURE 1A
#--------------------------------------------------------------------------------------------------------------
#Transform count data in the phyloseq object
ps_gg_fp_f_prevalence_filter_2019_05_26_proportion<-phyloseq::transform_sample_counts(ps_gg_fp_f_prevalence_filter_2019_05_26, function(x)(x/sum(x)))

#Select samples of interest and update phyloseq object
metadata<-as.data.frame(phyloseq::sample_data(ps_gg_fp_f_prevalence_filter_2019_05_26_proportion))
metadata<-metadata[metadata$hiv_phenotype=="1_hiv_negative",,drop=FALSE]
metadata<-as.data.frame(as.matrix(metadata[metadata$sexual_orientation!="MSM",,drop=FALSE]))
phyloseq::sample_data(ps_gg_fp_f_prevalence_filter_2019_05_26_proportion)<-metadata

#Run PCoA on the phyloseq object
ordination<-phyloseq::ordinate(ps_gg_fp_f_prevalence_filter_2019_05_26_proportion, "PCoA", "unifrac")
```

```
## Warning in matrix(tree$edge[order(tree$edge[, 1]), ][, 2], byrow = TRUE, : data
## length [8987] is not a sub-multiple or multiple of the number of rows [4494]
```

```
ordination$values[1:2,]
```

```
##   Eigenvalues Relative_eig Broken_stick  Cumul_eig Cumul_br_stick
## 1    5.865119   0.06123524   0.02490341 0.06123524     0.02490341
## 2    4.950507   0.05168615   0.02080505 0.11292139     0.04570846
```

```
metadata<-as.data.frame(phyloseq::sample_data(ps_gg_fp_f_prevalence_filter_2019_05_26_proportion))
metadata<-metadata[row.names(ordination$vectors),,drop=FALSE]
all.equal(row.names(metadata), row.names(ordination$vectors))
```

```
## [1] TRUE
```

```
metadata$Unifrac1<-ordination$vectors[,1]
metadata$Unifrac2<-ordination$vectors[,2]

plot1<-ggplot2::ggplot(data=metadata, aes(x=Unifrac1, y=Unifrac2))+geom_point(aes(color=sample_cohort), size=2)+theme_bw()+stat_ellipse(aes(color=sample_cohort))+
  geom_point(data=metadata %>% dplyr::group_by(sample_cohort) %>% dplyr::summarise_at(vars(matches("Unifrac")), mean),size=5, aes(color=sample_cohort))+
  scale_color_manual(values=c("royalblue4","darkorange", "forestgreen"))+ggtitle("Figure 1A")

plot1_axis<-ggplot2::ggplot(data=metadata, aes(x=sample_cohort, y=Unifrac1))+coord_flip()+geom_boxplot(aes(fill=sample_cohort), alpha=0.5, outlier.color="white")+theme_bw()+
  scale_fill_manual(values=c("royalblue4","darkorange","forestgreen"))+geom_point(aes(color=sample_cohort), position=position_jitterdodge(jitter.width=0.25),alpha=0.75, size=2)+
  scale_color_manual(values=c("royalblue4","darkorange","forestgreen"))

#Run Adonis (n=245) and run orm to compare axis position along Axis 1
ASV_table<-as.data.frame(phyloseq::otu_table(ps_gg_fp_f_prevalence_filter_2019_05_26_proportion))
all.equal(row.names(ASV_table), row.names(metadata))
```

```
## [1] TRUE
```

```
unifrac.distance<-unname(phyloseq::UniFrac(ps_gg_fp_f_prevalence_filter_2019_05_26_proportion, weighted = FALSE)) ### unname fixes error introduced by Desctools see https://github.com/joey711/phyloseq/issues/1457
```

```
## Warning in matrix(tree$edge[order(tree$edge[, 1]), ][, 2], byrow = TRUE, : data
## length [8987] is not a sub-multiple or multiple of the number of rows [4494]
```

```
attributes(unifrac.distance)$Labels <- phyloseq::sample_names(ps_gg_fp_f_prevalence_filter_2019_05_26_proportion)
print(vegan::adonis2(unifrac.distance~metadata$sample_cohort, data=ASV_table, permutations=1000)) -> adon_univar
```

```
## Permutation test for adonis under reduced model
## Terms added sequentially (first to last)
## Permutation: free
## Number of permutations: 1000
## 
## vegan::adonis2(formula = unifrac.distance ~ metadata$sample_cohort, data = ASV_table, permutations = 1000)
##                         Df SumOfSqs      R2     F   Pr(>F)    
## metadata$sample_cohort   2    5.704 0.05955 7.662 0.000999 ***
## Residual               242   90.076 0.94045                   
## Total                  244   95.780 1.00000                   
## ---
## Signif. codes:  0 '***' 0.001 '**' 0.01 '*' 0.05 '.' 0.1 ' ' 1
```

```
###*** sample_cohort r2 = 0.05955 p = 0.000999

# Kruskal and orm to test boxplot panel
print(agricolae::kruskal(metadata$Unifrac1, metadata$sample_cohort, group=F, p.adj = "bonferroni")) -> kruskal_univar
```

```
## $statistics
##     Chisq Df p.chisq
##   165.167  2       0
## 
## $parameters
##             test  p.ajusted                 name.t ntr alpha
##   Kruskal-Wallis bonferroni metadata$sample_cohort   3  0.05
## 
## $means
##          metadata.Unifrac1      rank        std  r         Min        Max
## boston         -0.16257080  48.92941 0.06564368 85 -0.30601250 0.04460428
## botswana        0.02719064 135.48750 0.10957614 80 -0.22309323 0.27899413
## uganda_2        0.14554084 189.21250 0.08377209 80 -0.06504402 0.29462462
##                  Q25         Q50        Q75
## boston   -0.21511440 -0.16961120 -0.1207705
## botswana -0.03195126  0.02058396  0.1154107
## uganda_2  0.10042632  0.15047450  0.2007123
## 
## $comparison
##                     Difference pvalue Signif.        LCL        UCL
## boston - botswana    -86.55809      0     *** -101.74760  -71.36857
## boston - uganda_2   -140.28309      0     *** -155.47260 -125.09357
## botswana - uganda_2  -53.72500      0     ***  -69.14294  -38.30706
## 
## $groups
## NULL
## 
## attr(,"class")
## [1] "group"
```

```
rms::orm(formula =  Unifrac1~sample_cohort, data = as_tibble(metadata)) -> orm_univar
print(orm_univar)
```

```
## Logistic (Proportional Odds) Ordinal Regression Model
## 
## rms::orm(formula = Unifrac1 ~ sample_cohort, data = as_tibble(metadata))
## 
##                              Model Likelihood               Discrimination    Rank Discrim.    
##                                    Ratio Test                      Indexes          Indexes    
## Obs                 245    LR chi2     248.67    R2                  0.638    rho     0.816    
## Distinct Y          245    d.f.             2    R2(2,245)           0.635                     
## Median Y    0.001016718    Pr(> chi2) <0.0001    R2(2,245)           0.635                     
## max |deriv|       1e-05    Score chi2  234.28    |Pr(Y>=median)-0.5| 0.328                     
##                            Pr(> chi2) <0.0001                                                  
## 
##                        Coef   S.E.   Wald Z Pr(>|Z|)
## sample_cohort=botswana 3.7734 0.3959  9.53  <0.0001 
## sample_cohort=uganda_2 5.8486 0.4519 12.94  <0.0001
```

```
###*** botswana-boston p = <0.0001 uganda-boston p = <0.0001 uganda-bostwana p = <0.0001

# Logistic (Proportional Odds) Ordinal Regression Model
# 
# rms::orm(formula = Unifrac1 ~ sample_cohort, data = as_tibble(metadata))
# 
#                              Model Likelihood               Discrimination    Rank Discrim.    
#                                    Ratio Test                      Indexes          Indexes    
# Obs                 245    LR chi2     248.67    R2                  0.638    rho     0.816    
# Distinct Y          245    d.f.             2    R2(2,245)           0.635                     
# Median Y    0.001016718    Pr(> chi2) <0.0001    R2(2,245)           0.635                     
# max |deriv|       1e-05    Score chi2  234.28    |Pr(Y>=median)-0.5| 0.328                     
#                            Pr(> chi2) <0.0001                                                  
# 
#                        Coef   S.E.   Wald Z Pr(>|Z|)
# sample_cohort=botswana 3.7734 0.3959  9.53  <0.0001 
# sample_cohort=uganda_2 5.8486 0.4519 12.94  <0.0001 


### Extra metadata to add that have full n: Race, Ethnicity, age, sex
### Extra metadata to add that have <n:BMI, comorbidities (dm2_hx, hld_hx, htn_hx, cvd_hx, ckd_hx, dm2hx_dx, hldhx_dx, htnhx_dx, cvdhx_dx)
metadata$age <- as.numeric(metadata$age)
metadata$BMI <- as.numeric(metadata$BMI)

### control for metadata with full n
covars_full_n <- c("Ethnicity", "age", "Race", "sex", "sample_cohort")
print(vegan::adonis2(as.formula(paste("unifrac.distance~metadata$", paste(covars_full_n, collapse = "+metadata$"), sep = "")), data=ASV_table, permutations=1000)) -> adon_full_n
```

```
## Permutation test for adonis under reduced model
## Terms added sequentially (first to last)
## Permutation: free
## Number of permutations: 1000
## 
## vegan::adonis2(formula = as.formula(paste("unifrac.distance~metadata$", paste(covars_full_n, collapse = "+metadata$"), sep = "")), data = ASV_table, permutations = 1000)
##                         Df SumOfSqs      R2      F   Pr(>F)    
## metadata$Ethnicity       1    0.440 0.00460 1.1913 0.092907 .  
## metadata$age             1    1.028 0.01073 2.7806 0.000999 ***
## metadata$Race            4    4.437 0.04632 3.0004 0.000999 ***
## metadata$sex             1    0.552 0.00576 1.4929 0.003996 ** 
## metadata$sample_cohort   2    2.442 0.02550 3.3033 0.000999 ***
## Residual               235   86.880 0.90708                    
## Total                  244   95.780 1.00000                    
## ---
## Signif. codes:  0 '***' 0.001 '**' 0.01 '*' 0.05 '.' 0.1 ' ' 1
```

```
###*** sample_cohort r2 = 0.02550 p = 0.000999 

#                         Df SumOfSqs      R2      F   Pr(>F)    
# metadata$Ethnicity       1    0.440 0.00460 1.1913 0.092907 .  
# metadata$age             1    1.028 0.01073 2.7806 0.000999 ***
# metadata$Race            4    4.437 0.04632 3.0004 0.000999 ***
# metadata$sex             1    0.552 0.00576 1.4929 0.003996 ** 
# metadata$sample_cohort   2    2.442 0.02550 3.3033 0.000999 ***
# Residual               235   86.880 0.90708                    
# Total                  244   95.780 1.00000                    

# orm to test boxplot panel
rms::orm(formula =  as.formula(paste("Unifrac1~", paste(covars_full_n, collapse = "+"), sep = "")), data = as_tibble(metadata)) -> orm_full_n
print(orm_full_n)
```

```
## Logistic (Proportional Odds) Ordinal Regression Model
## 
## rms::orm(formula = as.formula(paste("Unifrac1~", paste(covars_full_n, 
##     collapse = "+"), sep = "")), data = as_tibble(metadata))
## 
##                              Model Likelihood               Discrimination    Rank Discrim.    
##                                    Ratio Test                      Indexes          Indexes    
## Obs                 245    LR chi2     269.09    R2                  0.667    rho     0.803    
## Distinct Y          245    d.f.             9    R2(9,245)           0.654                     
## Median Y    0.001016718    Pr(> chi2) <0.0001    R2(9,245)           0.654                     
## max |deriv|       2e-05    Score chi2  258.33    |Pr(Y>=median)-0.5| 0.326                     
##                            Pr(> chi2) <0.0001                                                  
## 
##                               Coef    S.E.   Wald Z Pr(>|Z|)
## Ethnicity=Not_Hispanic_Latino -1.2599 0.7363 -1.71  0.0871  
## age                            0.0066 0.0121  0.54  0.5859  
## Race=Black_AA                  0.3557 0.9621  0.37  0.7116  
## Race=Unknown                   1.1325 1.3703  0.83  0.4085  
## Race=Varied                    0.1514 1.4338  0.11  0.9159  
## Race=White                    -1.1523 0.8543 -1.35  0.1774  
## sex=male                      -0.2776 0.2303 -1.21  0.2280  
## sample_cohort=botswana         3.0494 0.5878  5.19  <0.0001 
## sample_cohort=uganda_2         5.0662 0.6223  8.14  <0.0001
```

```
###*** botswana-boston p = <0.0001 uganda-boston p = <0.0001 uganda-bostwana p = <0.0001

# Logistic (Proportional Odds) Ordinal Regression Model
# 
# rms::orm(formula = as.formula(paste("Unifrac1~", paste(covars_full_n, 
#     collapse = "+"), sep = "")), data = as_tibble(metadata))
# 
#                              Model Likelihood               Discrimination    Rank Discrim.    
#                                    Ratio Test                      Indexes          Indexes    
# Obs                 245    LR chi2     269.09    R2                  0.667    rho     0.803    
# Distinct Y          245    d.f.             9    R2(9,245)           0.654                     
# Median Y    0.001016718    Pr(> chi2) <0.0001    R2(9,245)           0.654                     
# max |deriv|       2e-05    Score chi2  258.33    |Pr(Y>=median)-0.5| 0.326                     
#                            Pr(> chi2) <0.0001                                                  
# 
#                               Coef    S.E.   Wald Z Pr(>|Z|)
# Ethnicity=Not_Hispanic_Latino -1.2599 0.7363 -1.71  0.0871  
# age                            0.0066 0.0121  0.54  0.5859  
# Race=Black_AA                  0.3557 0.9621  0.37  0.7116  
# Race=Unknown                   1.1325 1.3703  0.83  0.4085  
# Race=Varied                    0.1514 1.4338  0.11  0.9159  
# Race=White                    -1.1523 0.8543 -1.35  0.1774  
# sex=male                      -0.2776 0.2303 -1.21  0.2280  
# sample_cohort=botswana         3.0494 0.5878  5.19  <0.0001 
# sample_cohort=uganda_2         5.0662 0.6223  8.14  <0.0001 


### remove samples with BMI/comorbidities is NA (n=245 -> n=231)
ps_gg_fp_f_prevalence_filter_2019_05_26_proportion_bc <- ps_gg_fp_f_prevalence_filter_2019_05_26_proportion
metadata_bc<-metadata[!is.na(metadata$BMI) & !is.na(metadata$dm2_hx),,drop=FALSE]
sample_data(ps_gg_fp_f_prevalence_filter_2019_05_26_proportion_bc) <- metadata_bc

### subset ASV table and distance matrix
ASV_table_bc<-as.data.frame(otu_table(ps_gg_fp_f_prevalence_filter_2019_05_26_proportion_bc))
all.equal(row.names(ASV_table_bc), row.names(metadata_bc))
```

```
## [1] TRUE
```

```
unifrac.distance_bc <- unname(usedist::dist_subset(unifrac.distance, sample_names(ps_gg_fp_f_prevalence_filter_2019_05_26_proportion_bc))) ### unname fixes error introduced by Desctools see https://github.com/joey711/phyloseq/issues/1457

### run adonis
covars_bc <- c("BMI", "dm2_hx", "hld_hx", "htn_hx", "cvd_hx", "dm2hx_dx", "hldhx_dx", "htnhx_dx", "cvdhx_dx", covars_full_n)
print(vegan::adonis2(as.formula(paste("unifrac.distance_bc~metadata_bc$", paste(covars_bc, collapse = "+metadata_bc$"), sep = "")), data=ASV_table_bc, permutations=1000)) -> adon_bc
```

```
## Permutation test for adonis under reduced model
## Terms added sequentially (first to last)
## Permutation: free
## Number of permutations: 1000
## 
## vegan::adonis2(formula = as.formula(paste("unifrac.distance_bc~metadata_bc$", paste(covars_bc, collapse = "+metadata_bc$"), sep = "")), data = ASV_table_bc, permutations = 1000)
##                            Df SumOfSqs      R2      F   Pr(>F)    
## metadata_bc$BMI             1    0.675 0.00750 1.8301 0.000999 ***
## metadata_bc$dm2_hx          1    0.416 0.00462 1.1273 0.151848    
## metadata_bc$hld_hx          1    0.386 0.00429 1.0454 0.301698    
## metadata_bc$htn_hx          1    0.715 0.00795 1.9383 0.000999 ***
## metadata_bc$cvd_hx          1    0.381 0.00424 1.0335 0.341658    
## metadata_bc$dm2hx_dx        1    0.455 0.00506 1.2333 0.059940 .  
## metadata_bc$hldhx_dx        1    0.572 0.00636 1.5511 0.005994 ** 
## metadata_bc$cvdhx_dx        1    0.609 0.00676 1.6495 0.001998 ** 
## metadata_bc$Ethnicity       1    0.481 0.00535 1.3038 0.026973 *  
## metadata_bc$age             1    0.879 0.00977 2.3829 0.000999 ***
## metadata_bc$Race            4    3.240 0.03600 2.1947 0.000999 ***
## metadata_bc$sex             1    0.580 0.00645 1.5717 0.001998 ** 
## metadata_bc$sample_cohort   2    2.000 0.02222 2.7091 0.000999 ***
## Residual                  213   78.610 0.87344                    
## Total                     230   90.001 1.00000                    
## ---
## Signif. codes:  0 '***' 0.001 '**' 0.01 '*' 0.05 '.' 0.1 ' ' 1
```

```
###*** sample_cohort r2 = 0.02222 p = 0.000999 

#                            Df SumOfSqs      R2      F   Pr(>F)    
# metadata_bc$BMI             1    0.675 0.00750 1.8301 0.000999 ***
# metadata_bc$dm2_hx          1    0.416 0.00462 1.1273 0.151848    
# metadata_bc$hld_hx          1    0.386 0.00429 1.0454 0.301698    
# metadata_bc$htn_hx          1    0.715 0.00795 1.9383 0.000999 ***
# metadata_bc$cvd_hx          1    0.381 0.00424 1.0335 0.341658    
# metadata_bc$dm2hx_dx        1    0.455 0.00506 1.2333 0.059940 .  
# metadata_bc$hldhx_dx        1    0.572 0.00636 1.5511 0.005994 ** 
# metadata_bc$cvdhx_dx        1    0.609 0.00676 1.6495 0.001998 ** 
# metadata_bc$Ethnicity       1    0.481 0.00535 1.3038 0.026973 *  
# metadata_bc$age             1    0.879 0.00977 2.3829 0.000999 ***
# metadata_bc$Race            4    3.240 0.03600 2.1947 0.000999 ***
# metadata_bc$sex             1    0.580 0.00645 1.5717 0.001998 ** 
# metadata_bc$sample_cohort   2    2.000 0.02222 2.7091 0.000999 ***
# Residual                  213   78.610 0.87344                    
# Total                     230   90.001 1.00000                   

# orm to test boxplot panel
rms::orm(formula =  as.formula(paste("Unifrac1~", paste(covars_bc[!covars_bc %in% c("htn_hx")], collapse = "+"), sep = "")), data = as_tibble(metadata)) -> orm_bc
print(orm_bc)
```

```
## Frequencies of Missing Values Due to Each Variable
##      Unifrac1           BMI        dm2_hx        hld_hx        cvd_hx 
##             0            11            14            14            14 
##      dm2hx_dx      hldhx_dx      htnhx_dx      cvdhx_dx     Ethnicity 
##            14            14            14            14             0 
##           age          Race           sex sample_cohort 
##             0             0             0             0 
## 
## Logistic (Proportional Odds) Ordinal Regression Model
## 
## rms::orm(formula = as.formula(paste("Unifrac1~", paste(covars_bc[!covars_bc %in% 
##     c("htn_hx")], collapse = "+"), sep = "")), data = as_tibble(metadata))
## 
## 
##                            Model Likelihood               Discrimination    Rank Discrim.    
##                                  Ratio Test                      Indexes          Indexes    
## Obs               231    LR chi2     253.10    R2                  0.666    rho     0.799    
## Distinct Y        231    d.f.            17    R2(17,231)          0.640                     
## Median Y    0.0156412    Pr(> chi2) <0.0001    R2(17,231)          0.640                     
## max |deriv|     2e-05    Score chi2  250.50    |Pr(Y>=median)-0.5| 0.310                     
##                          Pr(> chi2) <0.0001                                                  
## 
##                               Coef    S.E.   Wald Z Pr(>|Z|)
## BMI                           -0.0589 0.0274 -2.15  0.0319  
## dm2_hx= 1                      0.6232 1.0241  0.61  0.5428  
## hld_hx= 1                     -0.5884 0.6698 -0.88  0.3797  
## cvd_hx= 1                     -0.6453 0.6619 -0.97  0.3296  
## dm2hx_dx= 1                   -0.2842 0.7791 -0.36  0.7153  
## hldhx_dx= 1                    0.6169 0.4487  1.37  0.1692  
## htnhx_dx= 1                    0.6652 0.4043  1.65  0.0999  
## cvdhx_dx= 1                    0.3729 0.4482  0.83  0.4053  
## Ethnicity=Not_Hispanic_Latino -1.6820 0.9798 -1.72  0.0861  
## age                            0.0049 0.0135  0.36  0.7193  
## Race=Black_AA                  0.4991 0.9873  0.51  0.6132  
## Race=Unknown                   0.2096 1.8985  0.11  0.9121  
## Race=Varied                    1.3306 1.6457  0.81  0.4188  
## Race=White                    -1.1478 0.8415 -1.36  0.1725  
## sex=male                      -0.4740 0.2529 -1.87  0.0609  
## sample_cohort=botswana         2.6263 0.6742  3.90  <0.0001 
## sample_cohort=uganda_2         4.5874 0.7020  6.54  <0.0001
```

```
###*** botswana-boston p = <0.0001 uganda-boston p = <0.0001 uganda-bostwana p = <0.0001

#--------------------------------------------------------------------------------------------------------------

#FIGURE 1B
#--------------------------------------------------------------------------------------------------------------
#Tax glom at the Family level
ps_gg_fp_f_prevalence_filter_2019_05_26_agglomerate<-phyloseq::tax_glom(ps_gg_fp_f_prevalence_filter_2019_05_26, taxrank="Family")

#Apply filters: min 5000 counts in 50% of the samples
phyloseq::otu_table(ps_gg_fp_f_prevalence_filter_2019_05_26_agglomerate)<-t(phyloseq::otu_table(ps_gg_fp_f_prevalence_filter_2019_05_26_agglomerate))
filter_conditions<-phyloseq::filterfun_sample(function(x) x>=5000)
filtered <- phyloseq::genefilter_sample(phyloseq::otu_table(ps_gg_fp_f_prevalence_filter_2019_05_26_agglomerate), filter_conditions, A=(0.5*nrow(phyloseq::otu_table(ps_gg_fp_f_prevalence_filter_2019_05_26_agglomerate))))
ps_gg_fp_f_prevalence_filter_2019_05_26_agglomerate_filtered<-phyloseq::prune_taxa(filtered, ps_gg_fp_f_prevalence_filter_2019_05_26_agglomerate)

#Select samples of interest (the metadata dataframe has been already been subset before)
phyloseq::sample_data(ps_gg_fp_f_prevalence_filter_2019_05_26_agglomerate_filtered) <- metadata

#Calculate relative abundance
ps_gg_fp_f_prevalence_filter_2019_05_26_agglomerate_filtered_proportion<-phyloseq::transform_sample_counts(ps_gg_fp_f_prevalence_filter_2019_05_26_agglomerate_filtered, function(x)(x/sum(x)))

#Get data out from phyloseq object to plot with ggplot2:
ASV_table<-as.data.frame(t(phyloseq::otu_table(ps_gg_fp_f_prevalence_filter_2019_05_26_agglomerate_filtered_proportion)))
TAX_table<-as.data.frame(as.matrix(ps_gg_fp_f_prevalence_filter_2019_05_26_agglomerate_filtered_proportion@tax_table@.Data))

dim(TAX_table)
```

```
## [1] 7 7
```

```
TAX_table$Name<-paste(TAX_table$Kingdom, TAX_table$Phylum, TAX_table$Class, TAX_table$Order, TAX_table$Family)
all.equal(colnames(ASV_table), row.names(TAX_table))
```

```
## [1] TRUE
```

```
colnames(ASV_table) <- TAX_table$Name

all.equal(row.names(ASV_table), row.names(metadata))
```

```
## [1] TRUE
```

```
ASV_table$sample_cohort<-metadata$sample_cohort
ASV_table$SampleID<-row.names(ASV_table)
ASV_table$hiv_phenotype<-metadata$hiv_phenotype
ASV_table$Unifrac1<-metadata$Unifrac1

#Sort samples by position in Axis1 from Figure 1A
ASV_table<-ASV_table[order(ASV_table$Unifrac1),,drop=FALSE]
ASV_table$SampleID<-factor(ASV_table$SampleID, levels=ASV_table$SampleID)
ASV_table$Unifrac1<-as.factor(ASV_table$Unifrac1)
ASV_table_melt<-reshape::melt(ASV_table)
```

```
## Using sample_cohort, SampleID, hiv_phenotype, Unifrac1 as id variables
```

```
ASV_table_melt$variable<-as.character(ASV_table_melt$variable)
ASV_table_melt$Family<-stringr::str_split_fixed(ASV_table_melt$variable,"_",4)[,1]
ASV_table_melt$Genus<-stringr::str_split_fixed(ASV_table_melt$variable,"_",4)[,2]
ASV_table_melt$Species<-paste(stringr::str_split_fixed(ASV_table_melt$variable,"_",4)[,2],stringr::str_split_fixed(ASV_table_melt$variable,"_",4)[,3])

#Kruskal Wallis test for differential abundance between cohorts
family<-c()
all_sig<-c() #We are interested in those families have all pairwise comparisons statistically significant:
for (i in colnames(ASV_table)[1:(dim(ASV_table)[2]-5)]){
  result<-kruskal.test(ASV_table[[i]],ASV_table[["sample_cohort"]])
  if (result$p.value<=0.01){
    print(i)
    print(result)
    result2<-agricolae::kruskal(ASV_table[[i]],ASV_table[["sample_cohort"]],group=FALSE, p.adj ="BH")
    if (result2$comparison$pvalue[1]<0.1 & result2$comparison$pvalue[2]<0.1 & result2$comparison$pvalue[3]<0.1){
      all_sig<-c(all_sig, i)
    }
    family<-c(family, i)
  }
}
```

```
## [1] "Bacteria Actinobacteria Actinobacteria Bifidobacteriales Bifidobacteriaceae"
## 
##  Kruskal-Wallis rank sum test
## 
## data:  ASV_table[[i]] and ASV_table[["sample_cohort"]]
## Kruskal-Wallis chi-squared = 14.758, df = 2, p-value = 0.0006243
## 
## [1] "Bacteria Verrucomicrobia Verrucomicrobiae Verrucomicrobiales Verrucomicrobiaceae"
## 
##  Kruskal-Wallis rank sum test
## 
## data:  ASV_table[[i]] and ASV_table[["sample_cohort"]]
## Kruskal-Wallis chi-squared = 16.112, df = 2, p-value = 0.0003172
## 
## [1] "Bacteria Bacteroidetes Bacteroidia Bacteroidales Bacteroidaceae"
## 
##  Kruskal-Wallis rank sum test
## 
## data:  ASV_table[[i]] and ASV_table[["sample_cohort"]]
## Kruskal-Wallis chi-squared = 131.24, df = 2, p-value < 2.2e-16
## 
## [1] "Bacteria Bacteroidetes Bacteroidia Bacteroidales Prevotellaceae"
## 
##  Kruskal-Wallis rank sum test
## 
## data:  ASV_table[[i]] and ASV_table[["sample_cohort"]]
## Kruskal-Wallis chi-squared = 51.999, df = 2, p-value = 5.111e-12
## 
## [1] "Bacteria Firmicutes Clostridia Clostridiales Veillonellaceae"
## 
##  Kruskal-Wallis rank sum test
## 
## data:  ASV_table[[i]] and ASV_table[["sample_cohort"]]
## Kruskal-Wallis chi-squared = 23.239, df = 2, p-value = 8.99e-06
```

```
# [1] "Bacteria Bacteroidetes Bacteroidia Bacteroidales Bacteroidaceae"
# 
#   Kruskal-Wallis rank sum test
# 
# data:  ASV_table[[i]] and ASV_table[["sample_cohort"]]
# Kruskal-Wallis chi-squared = 131.24, df = 2, p-value < 2.2e-16
# 
# [1] "Bacteria Bacteroidetes Bacteroidia Bacteroidales Prevotellaceae"
# 
#   Kruskal-Wallis rank sum test
# 
# data:  ASV_table[[i]] and ASV_table[["sample_cohort"]]
# Kruskal-Wallis chi-squared = 51.999, df = 2, p-value = 5.111e-12


# Add all metadata to the table for multivariate testing of abundance differences
metadata$age <- as.numeric(metadata$age)
metadata$BMI <- as.numeric(metadata$BMI)
dplyr::left_join(ASV_table[colnames(ASV_table) %in% c("SampleID", "Unifrac1") | !colnames(ASV_table) %in% colnames(metadata)], metadata[, !colnames(metadata) %in% c("Unifrac1")], by = "SampleID") -> ASV_table_full_metadata

# Run orm (n=245) and compare family abundances between sample_cohort
rms::orm(formula =  as.formula(paste("ASV_table_full_metadata[[", which(stringr::str_detect(colnames(ASV_table_full_metadata), "Bacteroidaceae")), "]]~", "sample_cohort", sep = "")), data = ASV_table_full_metadata) -> orm_abund_bact
print(orm_abund_bact)
```

```
## Logistic (Proportional Odds) Ordinal Regression Model
## 
## rms::orm(formula = as.formula(paste("ASV_table_full_metadata[[", 
##     which(stringr::str_detect(colnames(ASV_table_full_metadata), 
##         "Bacteroidaceae")), "]]~", "sample_cohort", sep = "")), 
##     data = ASV_table_full_metadata)
## 
##                              Model Likelihood               Discrimination    Rank Discrim.    
##                                    Ratio Test                      Indexes          Indexes    
## Obs                 245    LR chi2     168.18    R2                  0.497    rho     0.733    
## Distinct Y          204    d.f.             2    R2(2,245)           0.493                     
## Median Y    0.009656538    Pr(> chi2) <0.0001    R2(2,243.8)         0.494                     
## max |deriv|       3e-05    Score chi2  164.58    |Pr(Y>=median)-0.5| 0.275                     
##                            Pr(> chi2) <0.0001                                                  
## 
##                        Coef    S.E.   Wald Z Pr(>|Z|)
## sample_cohort=botswana -2.1607 0.3064  -7.05 <0.0001 
## sample_cohort=uganda_2 -4.2307 0.3637 -11.63 <0.0001
```

```
###*** botswana-boston p = <0.0001 uganda-boston p = <0.0001 uganda-bostwana p = <0.0001

# Logistic (Proportional Odds) Ordinal Regression Model
# 
# rms::orm(formula = as.formula(paste("ASV_table_full_metadata[[", 
#     which(stringr::str_detect(colnames(ASV_table_full_metadata), 
#         "Bacteroidaceae")), "]]~", "sample_cohort", sep = "")), 
#     data = ASV_table_full_metadata)
# 
#                              Model Likelihood               Discrimination    Rank Discrim.    
#                                    Ratio Test                      Indexes          Indexes    
# Obs                 245    LR chi2     168.18    R2                  0.497    rho     0.733    
# Distinct Y          204    d.f.             2    R2(2,245)           0.493                     
# Median Y    0.009656538    Pr(> chi2) <0.0001    R2(2,243.8)         0.494                     
# max |deriv|       3e-05    Score chi2  164.58    |Pr(Y>=median)-0.5| 0.275                     
#                            Pr(> chi2) <0.0001                                                  
# 
#                        Coef    S.E.   Wald Z Pr(>|Z|)
# sample_cohort=botswana -2.1607 0.3064  -7.05 <0.0001 
# sample_cohort=uganda_2 -4.2307 0.3637 -11.63 <0.0001 

rms::orm(formula =  as.formula(paste("ASV_table_full_metadata[[", which(stringr::str_detect(colnames(ASV_table_full_metadata), "Prevotellaceae")), "]]~", "sample_cohort", sep = "")), data = ASV_table_full_metadata) -> orm_abund_prev
print(orm_abund_prev)
```

```
## Logistic (Proportional Odds) Ordinal Regression Model
## 
## rms::orm(formula = as.formula(paste("ASV_table_full_metadata[[", 
##     which(stringr::str_detect(colnames(ASV_table_full_metadata), 
##         "Prevotellaceae")), "]]~", "sample_cohort", sep = "")), 
##     data = ASV_table_full_metadata)
## 
##                            Model Likelihood               Discrimination    Rank Discrim.    
##                                  Ratio Test                      Indexes          Indexes    
## Obs               245    LR chi2      58.14    R2                  0.211    rho     0.461    
## Distinct Y        230    d.f.             2    R2(2,245)           0.205                     
## Median Y    0.1757825    Pr(> chi2) <0.0001    R2(2,244.9)         0.205                     
## max |deriv|    0.0003    Score chi2   57.49    |Pr(Y>=median)-0.5| 0.172                     
##                          Pr(> chi2) <0.0001                                                  
## 
##                        Coef   S.E.   Wald Z Pr(>|Z|)
## sample_cohort=botswana 1.2680 0.2876 4.41   <0.0001 
## sample_cohort=uganda_2 2.2106 0.3012 7.34   <0.0001
```

```
###*** botswana-boston p = <0.0001 uganda-boston p = <0.0001 uganda-bostwana p = <0.0001

# Logistic (Proportional Odds) Ordinal Regression Model
# 
# rms::orm(formula = as.formula(paste("ASV_table_full_metadata[[", 
#     which(stringr::str_detect(colnames(ASV_table_full_metadata), 
#         "Prevotellaceae")), "]]~", "sample_cohort", sep = "")), 
#     data = ASV_table_full_metadata)
# 
#                            Model Likelihood               Discrimination    Rank Discrim.    
#                                  Ratio Test                      Indexes          Indexes    
# Obs               245    LR chi2      58.14    R2                  0.211    rho     0.461    
# Distinct Y        230    d.f.             2    R2(2,245)           0.205                     
# Median Y    0.1757825    Pr(> chi2) <0.0001    R2(2,244.9)         0.205                     
# max |deriv|    0.0003    Score chi2   57.49    |Pr(Y>=median)-0.5| 0.172                     
#                          Pr(> chi2) <0.0001                                                  
# 
#                        Coef   S.E.   Wald Z Pr(>|Z|)
# sample_cohort=botswana 1.2680 0.2876 4.41   <0.0001 
# sample_cohort=uganda_2 2.2106 0.3012 7.34   <0.0001 

### Extra metadata to add that have full n: Race, Ethnicity, age, sex
### Extra metadata to add that have <n:BMI, comorbidities (dm2_hx, hld_hx, htn_hx, cvd_hx, ckd_hx, dm2hx_dx, hldhx_dx, htnhx_dx, cvdhx_dx)
### control for metadata with full n
covars_full_n <- c("Ethnicity", "age", "Race", "sex", "sample_cohort")
rms::orm(formula =  as.formula(paste("ASV_table_full_metadata[[", which(stringr::str_detect(colnames(ASV_table_full_metadata), "Bacteroidaceae")), "]]~", paste(covars_full_n, collapse = "+"), sep = "")), data = ASV_table_full_metadata) -> orm_abund_bact_full_n
print(orm_abund_bact_full_n)
```

```
## Logistic (Proportional Odds) Ordinal Regression Model
## 
## rms::orm(formula = as.formula(paste("ASV_table_full_metadata[[", 
##     which(stringr::str_detect(colnames(ASV_table_full_metadata), 
##         "Bacteroidaceae")), "]]~", paste(covars_full_n, collapse = "+"), 
##     sep = "")), data = ASV_table_full_metadata)
## 
##                              Model Likelihood               Discrimination    Rank Discrim.    
##                                    Ratio Test                      Indexes          Indexes    
## Obs                 245    LR chi2     175.80    R2                  0.512    rho     0.717    
## Distinct Y          204    d.f.             9    R2(9,245)           0.494                     
## Median Y    0.009656538    Pr(> chi2) <0.0001    R2(9,243.8)         0.496                     
## max |deriv|      0.0001    Score chi2  173.01    |Pr(Y>=median)-0.5| 0.279                     
##                            Pr(> chi2) <0.0001                                                  
## 
##                               Coef    S.E.   Wald Z Pr(>|Z|)
## Ethnicity=Not_Hispanic_Latino -0.7588 0.9075 -0.84  0.4031  
## age                            0.0066 0.0123  0.53  0.5934  
## Race=Black_AA                  0.1858 1.1816  0.16  0.8750  
## Race=Unknown                  -2.3522 1.6335 -1.44  0.1499  
## Race=Varied                   -1.0189 1.5470 -0.66  0.5101  
## Race=White                     0.2106 1.1029  0.19  0.8486  
## sex=male                      -0.3316 0.2371 -1.40  0.1620  
## sample_cohort=botswana        -2.2273 0.5392 -4.13  <0.0001 
## sample_cohort=uganda_2        -4.4163 0.5765 -7.66  <0.0001
```

```
###*** botswana-boston p = <0.0001 uganda-boston p = <0.0001 uganda-bostwana p = <0.0001
###* Bacteroidaceae: Z = -4.13, -7.66, *;  p = <0.0001 , <0.0001 , <0.0001 

# Logistic (Proportional Odds) Ordinal Regression Model
# 
# rms::orm(formula = as.formula(paste("ASV_table_full_metadata[[", 
#     which(stringr::str_detect(colnames(ASV_table_full_metadata), 
#         "Bacteroidaceae")), "]]~", paste(covars_full_n, collapse = "+"), 
#     sep = "")), data = ASV_table_full_metadata)
# 
#                              Model Likelihood               Discrimination    Rank Discrim.    
#                                    Ratio Test                      Indexes          Indexes    
# Obs                 245    LR chi2     175.80    R2                  0.512    rho     0.717    
# Distinct Y          204    d.f.             9    R2(9,245)           0.494                     
# Median Y    0.009656538    Pr(> chi2) <0.0001    R2(9,243.8)         0.496                     
# max |deriv|      0.0001    Score chi2  173.01    |Pr(Y>=median)-0.5| 0.279                     
#                            Pr(> chi2) <0.0001                                                  
# 
#                               Coef    S.E.   Wald Z Pr(>|Z|)
# Ethnicity=Not_Hispanic_Latino -0.7588 0.9075 -0.84  0.4031  
# age                            0.0066 0.0123  0.53  0.5934  
# Race=Black_AA                  0.1858 1.1816  0.16  0.8750  
# Race=Unknown                  -2.3522 1.6335 -1.44  0.1499  
# Race=Varied                   -1.0189 1.5470 -0.66  0.5101  
# Race=White                     0.2106 1.1029  0.19  0.8486  
# sex=male                      -0.3316 0.2371 -1.40  0.1620  
# sample_cohort=botswana        -2.2273 0.5392 -4.13  <0.0001 
# sample_cohort=uganda_2        -4.4163 0.5765 -7.66  <0.0001 


rms::orm(formula =  as.formula(paste("ASV_table_full_metadata[[", which(stringr::str_detect(colnames(ASV_table_full_metadata), "Prevotellaceae")), "]]~", paste(covars_full_n, collapse = "+"), sep = "")), data = ASV_table_full_metadata) -> orm_abund_prev_full_n
print(orm_abund_prev_full_n)
```

```
## Logistic (Proportional Odds) Ordinal Regression Model
## 
## rms::orm(formula = as.formula(paste("ASV_table_full_metadata[[", 
##     which(stringr::str_detect(colnames(ASV_table_full_metadata), 
##         "Prevotellaceae")), "]]~", paste(covars_full_n, collapse = "+"), 
##     sep = "")), data = ASV_table_full_metadata)
## 
##                            Model Likelihood               Discrimination    Rank Discrim.    
##                                  Ratio Test                      Indexes          Indexes    
## Obs               245    LR chi2      68.22    R2                  0.243    rho     0.474    
## Distinct Y        230    d.f.             9    R2(9,245)           0.215                     
## Median Y    0.1757825    Pr(> chi2) <0.0001    R2(9,244.9)         0.215                     
## max |deriv|     0.005    Score chi2   68.81    |Pr(Y>=median)-0.5| 0.185                     
##                          Pr(> chi2) <0.0001                                                  
## 
##                               Coef    S.E.   Wald Z Pr(>|Z|)
## Ethnicity=Not_Hispanic_Latino  0.1458 0.8208  0.18  0.8590  
## age                            0.0055 0.0122  0.45  0.6492  
## Race=Black_AA                  0.9834 1.1300  0.87  0.3842  
## Race=Unknown                   2.1207 1.6323  1.30  0.1939  
## Race=Varied                    0.1674 1.5555  0.11  0.9143  
## Race=White                    -0.2861 1.0406 -0.27  0.7834  
## sex=male                       0.4152 0.2291  1.81  0.0700  
## sample_cohort=botswana         0.3416 0.5353  0.64  0.5234  
## sample_cohort=uganda_2         1.2380 0.5413  2.29  0.0222
```

```
###*** botswana-boston p = 0.5234 uganda-boston p = 0.0222 uganda-bostwana p = 0.0051
###* Prevotellaceae: Z = 0.64, 2.29, 2.80 ;  p = 0.5234, 0.0222, 0.0051

# Logistic (Proportional Odds) Ordinal Regression Model
# 
# rms::orm(formula = as.formula(paste("ASV_table_full_metadata[[", 
#     which(stringr::str_detect(colnames(ASV_table_full_metadata), 
#         "Prevotellaceae")), "]]~", paste(covars_full_n, collapse = "+"), 
#     sep = "")), data = ASV_table_full_metadata)
# 
#                            Model Likelihood               Discrimination    Rank Discrim.    
#                                  Ratio Test                      Indexes          Indexes    
# Obs               245    LR chi2      68.22    R2                  0.243    rho     0.474    
# Distinct Y        230    d.f.             9    R2(9,245)           0.215                     
# Median Y    0.1757825    Pr(> chi2) <0.0001    R2(9,244.9)         0.215                     
# max |deriv|     0.005    Score chi2   68.81    |Pr(Y>=median)-0.5| 0.185                     
#                          Pr(> chi2) <0.0001                                                  
# 
#                               Coef    S.E.   Wald Z Pr(>|Z|)
# Ethnicity=Not_Hispanic_Latino  0.1458 0.8208  0.18  0.8590  
# age                            0.0055 0.0122  0.45  0.6492  
# Race=Black_AA                  0.9834 1.1300  0.87  0.3842  
# Race=Unknown                   2.1207 1.6323  1.30  0.1939  
# Race=Varied                    0.1674 1.5555  0.11  0.9143  
# Race=White                    -0.2861 1.0406 -0.27  0.7834  
# sex=male                       0.4152 0.2291  1.81  0.0700  
# sample_cohort=botswana         0.3416 0.5353  0.64  0.5234  
# sample_cohort=uganda_2         1.2380 0.5413  2.29  0.0222  

ASV_table_melt_significant<-ASV_table_melt[ASV_table_melt$variable%in%all_sig,,drop=FALSE]
unique(ASV_table_melt_significant$variable)
```

```
## [1] "Bacteria Bacteroidetes Bacteroidia Bacteroidales Bacteroidaceae"
## [2] "Bacteria Bacteroidetes Bacteroidia Bacteroidales Prevotellaceae"
```

```
ordered_names<-c(names(sort(colSums(ASV_table[1:(dim(ASV_table)[2]-5)]), decreasing=FALSE)))

ASV_table_melt_significant$variable<-factor(ASV_table_melt_significant$variable, levels=ordered_names)
ASV_table_melt_significant$Unifrac1<-as.numeric(as.character(ASV_table_melt_significant$Unifrac1))

ggsave("Figure1B.pdf", ggplot(data=ASV_table_melt_significant, aes(x=Unifrac1, y=value))+geom_point(aes(color=sample_cohort), size=2.5)+
  stat_smooth(geom = "area", method = "loess", alpha = 0.5, size = 1,fill="grey60")+theme_bw()+
  facet_wrap(~variable, scales="free_y", ncol=1)+
  theme(legend.position = "left", axis.text.x = element_blank(), panel.grid.major.x=element_blank())+
  scale_colour_manual(values=c("royalblue4","darkorange","forestgreen")), width=15, height=10)
```

```
## Warning: Using `size` aesthetic for lines was deprecated in ggplot2 3.4.0.
## ℹ Please use `linewidth` instead.
```

```
## `geom_smooth()` using formula = 'y ~ x'
```

```
#--------------------------------------------------------------------------------------------------------------

#FIGURE 1C
#--------------------------------------------------------------------------------------------------------------
#Tax glom at the Species level
ps_gg_fp_f_prevalence_filter_2019_05_26_agglomerate<-phyloseq::tax_glom(ps_gg_fp_f_prevalence_filter_2019_05_26, taxrank="Species")

#Apply filters (minimum 1 counts in 50% of the samples)
phyloseq::otu_table(ps_gg_fp_f_prevalence_filter_2019_05_26_agglomerate)<-t(phyloseq::otu_table(ps_gg_fp_f_prevalence_filter_2019_05_26_agglomerate))
filter_conditions<-phyloseq::filterfun_sample(function(x) x>=1)
filtered<-phyloseq::genefilter_sample(phyloseq::otu_table(ps_gg_fp_f_prevalence_filter_2019_05_26_agglomerate), filter_conditions, A=(0.5*nrow(phyloseq::otu_table(ps_gg_fp_f_prevalence_filter_2019_05_26_agglomerate))))
ps_gg_fp_f_prevalence_filter_2019_05_26_agglomerate_filtered<-phyloseq::prune_taxa(filtered, ps_gg_fp_f_prevalence_filter_2019_05_26_agglomerate)

#Calculate relative abundance
ps_gg_fp_f_prevalence_filter_2019_05_26_agglomerate_filtered_proportion<-phyloseq::transform_sample_counts(ps_gg_fp_f_prevalence_filter_2019_05_26_agglomerate_filtered, function(x)(x/sum(x)))

#Select samples of interest (the metadata dataframe has been already been subset before)
phyloseq::sample_data(ps_gg_fp_f_prevalence_filter_2019_05_26_agglomerate_filtered_proportion)<-metadata

#Get data out from phyloseq object to plot with ggplot2:
ASV_table<-as.data.frame(t(phyloseq::otu_table(ps_gg_fp_f_prevalence_filter_2019_05_26_agglomerate_filtered_proportion)))
TAX_table<-as.data.frame(phyloseq::tax_table(ps_gg_fp_f_prevalence_filter_2019_05_26_agglomerate_filtered_proportion))
metadata<-as.data.frame(phyloseq::sample_data(ps_gg_fp_f_prevalence_filter_2019_05_26_agglomerate_filtered_proportion))

dim(TAX_table)
```

```
## [1] 53  7
```

```
TAX_table$Name<-paste(TAX_table$Genus, TAX_table$Species,1:dim(TAX_table)[2],sep="_") 
all.equal(colnames(ASV_table), row.names(TAX_table))
```

```
## [1] TRUE
```

```
colnames(ASV_table)<- TAX_table$Name

all.equal(row.names(ASV_table), row.names(metadata))
```

```
## [1] TRUE
```

```
ASV_table$sample_cohort<-metadata$sample_cohort
ASV_table$SampleID<-row.names(ASV_table)

#Sort samples by overall Prevotella genus abundance
Prev<-ASV_table[,grepl("Prevo", colnames(ASV_table)),drop=F]
Prev_names<-row.names(as.data.frame(sort(rowSums(Prev))))

ASV_table<-ASV_table[Prev_names,,drop=FALSE]
ASV_table$Position<-factor(c(1:dim(ASV_table)[1]))
ASV_table$SampleID<-factor(ASV_table$SampleID, levels=ASV_table$SampleID)

ASV_table_melt<-reshape2::melt(ASV_table)
```

```
## Using sample_cohort, SampleID, Position as id variables
```

```
ASV_table_melt$variable<-as.character(ASV_table_melt$variable)
ASV_table_melt$Family<-stringr::str_split_fixed(ASV_table_melt$variable,"_",4)[,1]
ASV_table_melt$Genus<-stringr::str_split_fixed(ASV_table_melt$variable,"_",4)[,2]
ASV_table_melt$Species<-paste(stringr::str_split_fixed(ASV_table_melt$variable,"_",4)[,2],stringr::str_split_fixed(ASV_table_melt$variable,"_",4)[,3])

#Only Kruskal significant taxa
species<-c()
for (i in colnames(ASV_table)[1:(dim(ASV_table)[2]-3)]){
  result<-kruskal.test(ASV_table[[i]],ASV_table[["sample_cohort"]])
  if (result$p.value<=0.01){
    print(i)
    # print(agricolae::kruskal(ASV_table[[i]],ASV_table[["sample_cohort"]],group=FALSE, p.adj ="bonferroni"))
    species<-c(species, i)
  }
}
```

```
## [1] "Coprococcus_Coprococcus [Genus]_2"
## [1] "Clostridium_Clostridium [Genus]_3"
## [1] "Ruminococcaceae [Family]_Ruminococcaceae [Family]_5"
## [1] "Ruminococcus_bromii_7"
## [1] "Faecalibacterium_prausnitzii_1"
## [1] "Bilophila_Bilophila [Genus]_5"
## [1] "Bifidobacterium_longum_6"
## [1] "Bifidobacterium_Bifidobacterium [Genus]_7"
## [1] "Akkermansia_muciniphila_2"
## [1] "Bacteroides_vulgatus_3"
## [1] "Bacteroides_Bacteroides [Genus]_4"
## [1] "Bacteroides_ovatus_5"
## [1] "Bacteroides_uniformis_6"
## [1] "Prevotella_copri_7"
## [1] "Prevotella_Prevotella [Genus]_1"
## [1] "Prevotella_stercorea_2"
## [1] "Parabacteroides_Parabacteroides [Genus]_3"
## [1] "Parabacteroides_distasonis_4"
## [1] "Rikenellaceae [Family]_Rikenellaceae [Family]_5"
## [1] "S24-7 [Family]_S24-7 [Family]_6"
## [1] "Coriobacteriaceae [Family]_Coriobacteriaceae [Family]_1"
## [1] "Senegalimassilia_anaerobia_3"
## [1] "[Mogibacteriaceae] [Family]_[Mogibacteriaceae] [Family]_5"
## [1] "Streptococcus_thermophilus_6"
## [1] "Erysipelotrichaceae [Family]_Erysipelotrichaceae [Family]_1"
## [1] "Catenibacterium_mitsuokai_2"
## [1] "Clostridium_celatum_3"
## [1] "Blautia_wexlerae_5"
## [1] "Blautia_Blautia [Genus]_7"
## [1] "[Ruminococcus]_[Ruminococcus] [Genus]_2"
## [1] "Fusicatenibacter_saccharivorans_3"
## [1] "Dorea_Dorea [Genus]_4"
## [1] "Lachnospiraceae [Family]_Lachnospiraceae [Family]_6"
## [1] "Coprococcus_eutactus_7"
## [1] "Dorea_longicatena_1"
## [1] "Anaerostipes_hadrus_3"
## [1] "Lachnospira_Lachnospira [Genus]_4"
```

```
ASV_table_melt_significant<-ASV_table_melt[ASV_table_melt$variable%in%species,,drop=FALSE]
unique(ASV_table_melt_significant$variable)
```

```
##  [1] "Coprococcus_Coprococcus [Genus]_2"                          
##  [2] "Clostridium_Clostridium [Genus]_3"                          
##  [3] "Ruminococcaceae [Family]_Ruminococcaceae [Family]_5"        
##  [4] "Ruminococcus_bromii_7"                                      
##  [5] "Faecalibacterium_prausnitzii_1"                             
##  [6] "Bilophila_Bilophila [Genus]_5"                              
##  [7] "Bifidobacterium_longum_6"                                   
##  [8] "Bifidobacterium_Bifidobacterium [Genus]_7"                  
##  [9] "Akkermansia_muciniphila_2"                                  
## [10] "Bacteroides_vulgatus_3"                                     
## [11] "Bacteroides_Bacteroides [Genus]_4"                          
## [12] "Bacteroides_ovatus_5"                                       
## [13] "Bacteroides_uniformis_6"                                    
## [14] "Prevotella_copri_7"                                         
## [15] "Prevotella_Prevotella [Genus]_1"                            
## [16] "Prevotella_stercorea_2"                                     
## [17] "Parabacteroides_Parabacteroides [Genus]_3"                  
## [18] "Parabacteroides_distasonis_4"                               
## [19] "Rikenellaceae [Family]_Rikenellaceae [Family]_5"            
## [20] "S24-7 [Family]_S24-7 [Family]_6"                            
## [21] "Coriobacteriaceae [Family]_Coriobacteriaceae [Family]_1"    
## [22] "Senegalimassilia_anaerobia_3"                               
## [23] "[Mogibacteriaceae] [Family]_[Mogibacteriaceae] [Family]_5"  
## [24] "Streptococcus_thermophilus_6"                               
## [25] "Erysipelotrichaceae [Family]_Erysipelotrichaceae [Family]_1"
## [26] "Catenibacterium_mitsuokai_2"                                
## [27] "Clostridium_celatum_3"                                      
## [28] "Blautia_wexlerae_5"                                         
## [29] "Blautia_Blautia [Genus]_7"                                  
## [30] "[Ruminococcus]_[Ruminococcus] [Genus]_2"                    
## [31] "Fusicatenibacter_saccharivorans_3"                          
## [32] "Dorea_Dorea [Genus]_4"                                      
## [33] "Lachnospiraceae [Family]_Lachnospiraceae [Family]_6"        
## [34] "Coprococcus_eutactus_7"                                     
## [35] "Dorea_longicatena_1"                                        
## [36] "Anaerostipes_hadrus_3"                                      
## [37] "Lachnospira_Lachnospira [Genus]_4"
```

```
color_selection<-c("grey30", "gold3", "firebrick4", "seashell2", "lightsalmon3",
                   "lightsalmon1", "lightsalmon4", "darksalmon", "aquamarine3", "aquamarine2",
                   "grey80", "mediumorchid4", "mediumorchid2", "aliceblue", "deeppink3",
                   "deeppink2", "lightpink1", "lightpink3", "bisque", "khaki2", "khaki", 
                   "lightslategray ", "honeydew2", "lightslateblue", "steelblue4", "steelblue1", 
                   "orange3", "orange2", "darkgreen", "palegreen4", "chartreuse4", "lightskyblue1",
                   "gold1", "gold2", "grey80", "tan", "olivedrab2")

ggsave("Figure1C.pdf", ggplot(data=ASV_table_melt_significant, aes(x=SampleID, y=value))+geom_bar(aes(fill=variable),stat="identity")+theme_bw()+
  theme(legend.position = "bottom", axis.text.x = element_blank())+scale_fill_manual(values=color_selection)+
  facet_wrap(~sample_cohort, nrow=3, scales="free_x"), width=10, height=15)

#Prepare the tables with the mean and SD for those significant ones
ASV_table_sig<-ASV_table[,colnames(ASV_table)%in%c(species, "sample_cohort"),drop=F]

USA<-ASV_table_sig[ASV_table_sig$sample_cohort=="boston",,drop=F]
Botswana<-ASV_table_sig[ASV_table_sig$sample_cohort=="botswana",,drop=F]
Uganda<-ASV_table_sig[ASV_table_sig$sample_cohort=="uganda_2",,drop=F]

USA_means<-as.data.frame(colMeans(USA[1:37]))
USA_means<-USA_means[order(USA_means$`colMeans(USA[1:37])`, decreasing = TRUE),,drop=F]
              
Botswana_means<-as.data.frame(colMeans(Botswana[1:37]))
Botswana_means<-Botswana_means[order(Botswana_means$`colMeans(Botswana[1:37])`, decreasing = TRUE),,drop=F]

Uganda_means<-as.data.frame(colMeans(Uganda[1:37]))
Uganda_means<-Uganda_means[order(Uganda_means$`colMeans(Uganda[1:37])`, decreasing = TRUE),,drop=F]
#--------------------------------------------------------------------------------------------------------------

#FIGURE 1D
#--------------------------------------------------------------------------------------------------------------
#Create duplicated phyloseq object
ps_gg_fp_f_prevalence_filter_2019_05_26_t <- ps_gg_fp_f_prevalence_filter_2019_05_26

#Keep only those ASVs with at least 1 count in at least 2 samples
otu_table(ps_gg_fp_f_prevalence_filter_2019_05_26_t)<-t(otu_table(ps_gg_fp_f_prevalence_filter_2019_05_26_t))
filter_conditions<-filterfun_sample(function(x) x>=1)
filtered<-genefilter_sample(otu_table(ps_gg_fp_f_prevalence_filter_2019_05_26_t), filter_conditions, A=2)
ps_gg_fp_f_prevalence_filter_2019_05_26_t_filtered<-prune_taxa(filtered, ps_gg_fp_f_prevalence_filter_2019_05_26_t)

#Calculate relative abundance
ps_gg_fp_f_prevalence_filter_2019_05_26_t_filtered_proportion<-transform_sample_counts(ps_gg_fp_f_prevalence_filter_2019_05_26_t_filtered, function(x)(x/sum(x)))

# #Transform count data in the phyloseq object
# ps_gg_fp_f_prevalence_filter_2019_05_26_proportion<-phyloseq::transform_sample_counts(ps_gg_fp_f_prevalence_filter_2019_05_26, function(x)(x/sum(x)))
# 
# #Select samples of interest and update phyloseq object
# metadata<-as.data.frame(phyloseq::sample_data(ps_gg_fp_f_prevalence_filter_2019_05_26_proportion))
# metadata<-metadata[metadata$hiv_phenotype=="1_hiv_negative",,drop=FALSE]
# metadata<-as.data.frame(as.matrix(metadata[metadata$sexual_orientation!="MSM",,drop=FALSE]))
# phyloseq::sample_data(ps_gg_fp_f_prevalence_filter_2019_05_26_proportion)<-metadata

#Select samples of interest and update phyloseq object
sample_data(ps_gg_fp_f_prevalence_filter_2019_05_26_t_filtered_proportion)<-metadata

#Remove taxa with total sum=0
ps_gg_fp_f_prevalence_filter_2019_05_26_t_filtered_proportion<-prune_taxa(taxa_sums(ps_gg_fp_f_prevalence_filter_2019_05_26_t_filtered_proportion)>0, ps_gg_fp_f_prevalence_filter_2019_05_26_t_filtered_proportion)

#Get data out from phyloseq object to plot with ggplot2:
ASV_table<-as.data.frame(t(otu_table(ps_gg_fp_f_prevalence_filter_2019_05_26_t_filtered_proportion)))
TAX_table<-as.data.frame(tax_table(ps_gg_fp_f_prevalence_filter_2019_05_26_t_filtered_proportion))

dim(TAX_table)
```

```
## [1] 2998    7
```

```
TAX_table$Name<-paste(TAX_table$Family, TAX_table$Genus, TAX_table$Species,1:dim(TAX_table)[1],sep="_") 
all.equal(colnames(ASV_table),row.names(TAX_table))
```

```
## [1] TRUE
```

```
colnames(ASV_table)<- TAX_table$Name

all.equal(row.names(ASV_table), row.names(metadata))
```

```
## [1] TRUE
```

```
ASV_table$Sample_cohort<-metadata$sample_cohort

#Let's see what ASVs are differentially abundant between pairs of cohorts:
bos_bot<-c()
bos_ug<-c()
bot_ug<-c()
asv<-c()
for (i in colnames(ASV_table)[1:(dim(ASV_table)[2]-1)]){
  result<-kruskal.test(ASV_table[[i]],ASV_table[["Sample_cohort"]])
  if (result$p.value<=0.01){
#    print(i)
    result2<-kruskal(ASV_table[[i]],ASV_table[["Sample_cohort"]],group=FALSE, p.adj ="BH")
    if (result2$comparison$pvalue[1]<0.05){
      bos_bot<-c(bos_bot, i)
    }
    if (result2$comparison$pvalue[2]<0.05){
      bos_ug<-c(bos_ug, i)
    }
    if (result2$comparison$pvalue[3]<0.05){
      bot_ug<-c(bot_ug, i)
    }
    asv<-c(asv, i)
  }
}

#Just keep the significant ones:
ASV_table$Sample_cohort<-NULL
ASV_table2<-ASV_table[,colnames(ASV_table)%in%asv,drop=F]

#Set order for samples and reorder dataframe:
samples<-hclust(vegdist(ASV_table2, method = "bray"), "aver")
ASV_table_ordered<-ASV_table2[samples$order,,drop=FALSE]

#Set order for ASVs and reorder dataframe:
ASV_table2<-t(ASV_table2)
species_dist<-vegdist(ASV_table2, method = "bray")
species_dist[is.na(species_dist)] <- 0
species<-hclust(species_dist, "aver")
ASV_table_ordered<-ASV_table_ordered[,species$order,drop=FALSE]

#Add metadata information
metadata<-metadata[row.names(ASV_table_ordered),,drop=FALSE]
all.equal(row.names(ASV_table_ordered), row.names(metadata))
```

```
## [1] TRUE
```

```
ASV_table_ordered$sample_cohort<-metadata$sample_cohort
ASV_table_ordered$SampleID<-row.names(ASV_table)
ASV_table_melt<-melt(ASV_table_ordered)
```

```
## Using sample_cohort, SampleID as id variables
```

```
#Additionally to the clustering, order rows by sample cohort signature (i.e. more abundant in each of the cohorts compared to the other two):
boston<-ASV_table_ordered[ASV_table_ordered$sample_cohort=="boston",,drop=F]
botswana<-ASV_table_ordered[ASV_table_ordered$sample_cohort=="botswana",,drop=F]
uganda<-ASV_table_ordered[ASV_table_ordered$sample_cohort=="uganda_2",,drop=F]

boston_top<-c()
botswana_top<-c()
uganda_top<-c()
for (i in colnames(ASV_table_ordered)[1:(length(colnames(ASV_table_ordered))-3)]){
  boston_bacteria<-mean(boston[[i]])
  botswana_bacteria<-mean(botswana[[i]])
  uganda_bacteria<-mean(uganda[[i]])
  if (boston_bacteria > botswana_bacteria & botswana_bacteria > uganda_bacteria){
    boston_top <- c(boston_top, i)
  } else if (boston_bacteria < botswana_bacteria & botswana_bacteria > uganda_bacteria){
    botswana_top <- c(botswana_top, i)
  } else if (boston_bacteria < botswana_bacteria & botswana_bacteria < uganda_bacteria){
    uganda_top <- c(uganda_top, i)
  }
}

length(boston_top)
```

```
## [1] 154
```

```
length(botswana_top)
```

```
## [1] 121
```

```
length(uganda_top)
```

```
## [1] 143
```

```
asv_ordered_top<-c(uganda_top, botswana_top, boston_top)

ASV_table_ordered2<-ASV_table_ordered
ASV_table_ordered2<-ASV_table_ordered[,c(asv_ordered_top,"sample_cohort", "SampleID"),drop=F]
ASV_table_ordered2_melt<-melt(ASV_table_ordered2)
```

```
## Using sample_cohort, SampleID as id variables
```

```
ggsave("Figure1DANCOM.pdf", ggplot(ASV_table_ordered2_melt, aes(x=SampleID, y=variable))+geom_tile(aes(fill=log10(value)), na.rm = T)+geom_rug(aes(color=sample_cohort), sides = "b", size=0.5, outside=T)+
         theme_bw()+theme(axis.text.x = element_blank(), axis.text.y=element_text(angle=0, size=0), axis.title.x=element_blank(), axis.ticks = element_blank())+
         scale_fill_gradientn(colors=c("lemonchiffon", "gold","darkolivegreen3", "dodgerblue2", "darkblue"), na.value = "black")+scale_color_manual(values=c("coral", "lightpink4", "darkgreen"))+
         coord_cartesian(clip = "off")+facet_wrap(~sample_cohort, scales="free_x"), width=15, height=10)
#--------------------------------------------------------------------------------------------------------------

#FIGURE 1E
#--------------------------------------------------------------------------------------------------------------
#Prepare OTU table and taxonomy files out from the phyloseq object:
OTU_table<-as.data.frame(phyloseq::otu_table(ps_gg_fp_f_prevalence_filter_2019_05_26))
Taxonomy<-as.data.frame(phyloseq::tax_table(ps_gg_fp_f_prevalence_filter_2019_05_26))
metadata<-as.data.frame(phyloseq::sample_data(ps_gg_fp_f_prevalence_filter_2019_05_26))

#Find the sample with the smallest number of reads. It will be the value used to normalize all data so all samples will be comparable among them:
rowSums(OTU_table)
```

```
## 105574.boston1.0139.2014.12.08 108777.boston1.0140.2014.12.08 
##                          72609                          64328 
## 112993.boston1.0141.2014.12.08 123656.boston1.0005.2014.12.08 
##                          79920                          36479 
## 143200.boston1.0006.2014.12.08 153724.boston1.0007.2014.12.08 
##                          81427                          85980 
## 165642.boston1.0008.2014.12.08 194317.boston1.0010.2014.12.08 
##                          98119                          53263 
## 196203.boston1.0011.2014.12.08 205120.boston1.0013.2014.12.08 
##                          36651                          59443 
## 211774.boston1.0014.2014.12.08 228437.boston1.0017.2014.12.08 
##                          57586                          52936 
## 229969.boston1.0018.2014.12.08 237983.boston1.0019.2014.12.08 
##                          62357                          37574 
## 258085.boston1.0142.2014.12.08 273479.boston1.0143.2014.12.08 
##                          59728                         102691 
## 315504.boston1.0028.2014.12.08 330183.boston1.0144.2014.12.08 
##                          21399                          68760 
## 337016.boston1.0030.2014.12.08 365685.boston1.0032.2014.12.08 
##                          43655                          41183 
## 386576.boston1.0035.2014.12.08 389876.boston1.0036.2014.12.08 
##                          36454                          66739 
## 410644.boston1.0125.2014.12.08 410932.boston1.0039.2014.12.08 
##                          73269                          27289 
## 413736.boston1.0126.2014.12.08 427838.boston1.0127.2014.12.08 
##                          39912                          66847 
## 453548.boston1.0045.2014.12.08 460380.boston1.0046.2014.12.08 
##                          34382                          31329 
## 473516.boston1.0047.2014.12.08 479693.boston1.0048.2014.12.08 
##                          46039                          18765 
## 485548.boston1.0049.2014.12.08 498553.boston1.0050.2014.12.08 
##                          35582                          56405 
## 505402.boston1.0051.2014.12.08 516980.boston1.0130.2014.12.08 
##                          59815                          14678 
## 522458.boston1.0132.2014.12.08 526318.boston1.0133.2014.12.08 
##                          68609                          27811 
## 527968.boston1.0057.2014.12.08 529516.boston1.0136.2014.12.08 
##                          26204                          20681 
## 533586.boston1.0059.2014.12.08 534694.boston1.0060.2014.12.08 
##                          57983                          51661 
## 604772.boston1.0071.2014.12.08 614225.boston1.0073.2014.12.08 
##                          52199                          47445 
## 615167.boston1.0074.2014.12.08 616147.boston1.0075.2014.12.08 
##                          37085                          50711 
## 653425.boston1.0077.2014.12.08 666207.boston1.0079.2014.12.08 
##                          42115                          70102 
## 694413.boston1.0080.2014.12.08 708968.boston1.0083.2014.12.08 
##                          44825                          28721 
## 734962.boston1.0085.2014.12.08 745577.boston1.0086.2014.12.08 
##                          43153                          75694 
## 758572.boston1.0088.2014.12.08 775609.boston1.0091.2014.12.08 
##                          63237                          64948 
## 813341.boston1.0095.2014.12.08 819622.boston1.0096.2014.12.08 
##                          74295                          34279 
## 842279.boston1.0097.2014.12.08 847041.boston1.0098.2014.12.08 
##                          61932                          59051 
## 862898.boston1.0103.2014.12.08 874612.boston1.0105.2014.12.08 
##                          38913                          21417 
## 880160.boston1.0106.2014.12.08 899025.boston1.0107.2014.12.08 
##                          61338                          51550 
## 900158.boston1.0108.2014.12.08 911594.boston1.0109.2014.12.08 
##                          18175                          54168 
## 923358.boston1.0111.2014.12.08 950965.boston1.0116.2014.12.08 
##                          34979                          66238 
## 953586.boston1.0117.2014.12.08 958793.boston1.0118.2014.12.08 
##                          54430                          66440 
## 966971.boston1.0120.2014.12.08 970489.boston1.0121.2014.12.08 
##                          44109                          48095 
## 995725.boston1.0123.2014.12.08  529863.boston.0165.2017.04.06 
##                          58954                          42388 
##  608647.boston.0072.2017.04.06    686039.0040.0323.2017.04.06 
##                          31028                         374932 
##   WT24922.0093.0468.2017.04.06    102438.0086.0363.2017.03.15 
##                         107819                          25113 
##    106085.0054.0333.2017.03.15    122897.0017.0307.2017.03.15 
##                          19135                          80414 
##    129226.0089.0367.2017.03.15    136376.0013.0302.2017.03.15 
##                          33236                          27232 
##    148342.0027.0314.2017.03.15    157072.0043.0244.2017.03.15 
##                          45438                          22883 
##    175067.0033.0231.2017.03.15    181090.0091.0369.2017.03.15 
##                          15936                          22823 
##    189326.0070.0343.2017.03.15    191447.0008.0296.2017.03.15 
##                          61451                          62556 
##    207295.0010.0298.2017.03.15    211578.0032.0230.2017.03.15 
##                          30235                          23193 
##    228516.0076.0350.2017.03.15    236532.0078.0379.2017.03.15 
##                          39086                          66657 
##    238426.0046.0247.2017.03.15    249768.0083.0359.2017.03.15 
##                          15578                          65769 
##    251073.0025.0221.2017.03.15    285803.0064.0338.2017.03.15 
##                          27143                          63788 
##    293340.0035.0233.2017.03.15    305385.0051.0330.2017.03.15 
##                          24885                          20588 
##    310817.0006.0293.2017.03.15    331904.0098.0430.2017.03.15 
##                          32927                          35529 
##    347964.0061.0336.2017.03.15    350103.0012.0301.2017.03.15 
##                          66932                          34897 
##    380272.0044.0245.2017.03.15    387879.0030.0228.2017.03.15 
##                          55368                          51686 
##    400609.0058.0262.2017.03.15    408044.0052.0331.2017.03.15 
##                          24415                          57931 
##    419034.0081.0382.2017.03.15    432158.0045.0246.2017.03.15 
##                          57230                          37797 
##    442916.0037.0319.2017.03.15    444991.0047.0327.2017.03.15 
##                          24253                          17042 
##    460929.0041.0324.2017.03.15    466105.0018.0377.2017.03.15 
##                          18796                          48350 
##    470588.0066.0340.2017.03.15  481066.boston.0168.2017.03.15 
##                          52619                          14233 
##    487268.0057.0261.2017.03.15    498229.0036.0318.2017.03.15 
##                          17799                          38925 
##    498554.0062.0337.2017.03.15    502743.0038.0320.2017.03.15 
##                          47910                          29154 
##    515591.0056.0334.2017.03.15    516035.0020.0310.2017.03.15 
##                          16026                          41843 
##    521471.0067.0341.2017.03.15    524541.0024.0313.2017.03.15 
##                          37753                          34986 
##    560575.0080.0381.2017.03.15    564855.0053.0332.2017.03.15 
##                          76936                          32030 
##    565723.0005.0292.2017.03.15    588311.0072.0346.2017.03.15 
##                          33565                          61200 
##    596527.0095.0374.2017.03.15    614631.0065.0339.2017.03.15 
##                          49031                          23279 
##    629358.0009.0297.2017.03.15    637837.0021.0312.2017.03.15 
##                          63706                          33770 
##    651433.0099.0431.2017.03.15    658217.0055.0258.2017.03.15 
##                          27060                          34249 
##    711750.0082.0383.2017.03.15    721154.0060.0335.2017.03.15 
##                          83786                          16367 
##    721729.0088.0366.2017.03.15    722188.0063.0268.2017.03.15 
##                          23928                          26981 
##    725896.0022.0218.2017.03.15    732425.0039.0378.2017.03.15 
##                          18124                          48694 
##    735345.0071.0345.2017.03.15    768392.0096.0375.2017.03.15 
##                          46984                          41304 
##    805457.0094.0373.2017.03.15    805641.0015.0305.2017.03.15 
##                          65857                          38997 
##    839338.0011.0300.2017.03.15    848334.0077.0351.2017.03.15 
##                          70395                          45711 
##    885614.0048.0328.2017.03.15    888751.0019.0309.2017.03.15 
##                          18203                          47832 
##    893231.0074.0348.2017.03.15    902901.0029.0226.2017.03.15 
##                          28255                          18740 
##    905350.0085.0362.2017.03.15    908782.0092.0371.2017.03.15 
##                          42388                          28907 
##  909824.boston.0174.2017.03.15    910641.0031.0316.2017.03.15 
##                          12820                          38990 
##    916034.0034.0232.2017.03.15    919901.0026.0222.2017.03.15 
##                          21405                          19714 
##    940622.0075.0349.2017.03.15    959714.0004.0291.2017.03.15 
##                          42914                          30169 
##    959734.0090.0368.2017.03.15    968359.0087.0365.2017.03.15 
##                          37955                          33934 
##    968902.0073.0347.2017.03.15    972684.0028.0224.2017.03.15 
##                          34542                          22469 
##    975240.0016.0306.2017.03.15    976183.0050.0329.2017.03.15 
##                          30867                          31908 
##    979196.0003.0290.2017.03.15    989517.0068.0274.2017.03.15 
##                          34682                          45375 
##   WT09782.0159.0121.2017.02.01   WT15101.0174.0106.2017.02.01 
##                          52918                          17604 
##   WT42336.0160.0122.2017.02.01   WT44778.0158.0120.2017.02.01 
##                          17747                          80361 
##   XE17833.0092.0100.2017.02.01   XE22903.0043.0125.2017.02.01 
##                          10751                          92243 
##   XE28163.0194.0127.2017.02.01   WM26348.0100.0113.2017.01.11 
##                          77437                          52751 
##   WM26354.0139.0315.2017.01.11   WQ64001.0145.0143.2017.01.11 
##                          45678                          39753 
##   WS20813.0155.0153.2017.01.11   WS21384.0094.0102.2017.01.11 
##                          29351                          12873 
##   WS21401.0047.0283.2017.01.11   WS21556.0141.0317.2017.01.11 
##                          65243                          57521 
##   WS21562.0055.0438.2017.01.11   WS21578.0125.0134.2017.01.11 
##                          13616                          46331 
##   WS21584.0066.0321.2017.01.11   WS22205.0097.0110.2017.01.11 
##                          28249                          65967 
##   WS74808.0165.0347.2017.01.11   WS74858.0098.0111.2017.01.11 
##                          32886                          18675 
##   WS75418.0032.0279.2017.01.11   WS76117.0102.0115.2017.01.11 
##                          66488                          78753 
##   WS76840.0117.0126.2017.01.11   WS77050.0026.0276.2017.01.11 
##                          39459                          15102 
##   WT02695.0166.0104.2017.01.11   WT02712.0078.0323.2017.01.11 
##                          20141                          38952 
##   WT05558.0190.0378.2017.01.11   WT05564.0116.0125.2017.01.11 
##                          17973                          33463 
##   WT06263.0127.0135.2017.01.11   WT07439.0058.0440.2017.01.11 
##                          35147                          43263 
##   WT08061.0090.0098.2017.01.11   WT09760.0053.0437.2017.01.11 
##                          28736                          34666 
##   WT09798.0057.0285.2017.01.11   WT10373.0080.0325.2017.01.11 
##                          78499                          37600 
##   WT10389.0036.0270.2017.01.11   WT10395.0035.0269.2017.01.11 
##                          29095                          43853 
##   WT12565.0189.0377.2017.01.11   WT14129.0153.0151.2017.01.11 
##                          26219                          58444 
##   WT14135.0123.0132.2017.01.11   WT14818.0111.0120.2017.01.11 
##                          42345                          33448 
##   WT15084.0046.0282.2017.01.11   WT15117.0157.0155.2017.01.11 
##                         122971                          41809 
##   WT15123.0164.0346.2017.01.11   WT23223.0144.0320.2017.01.11 
##                          37517                          39399 
##   WT23273.0101.0114.2017.01.11   WT24900.0152.0150.2017.01.11 
##                          32170                          25802 
##   WT24916.0044.0281.2017.01.11   WT24944.0124.0159.2017.01.11 
##                          74046                          36504 
##   WT24950.0033.0267.2017.01.11   WT24966.0142.0318.2017.01.11 
##                          54801                          37339 
##   WT27396.0121.0130.2017.01.11   WT27407.0156.0154.2017.01.11 
##                          30349                          27423 
##   WT27441.0129.0137.2017.01.11   WT30335.0134.0140.2017.01.11 
##                          25366                          76057 
##   WT30357.0049.0274.2017.01.11   WT30818.0107.0118.2017.01.11 
##                          35457                          26163 
##   WT30824.0147.0145.2017.01.11   WT30830.0052.0275.2017.01.11 
##                          45066                          41492 
##   WT34353.0120.0129.2017.01.11   WT40033.0133.0139.2017.01.11 
##                          40414                          22514 
##   WT40049.0148.0146.2017.01.11   WT40083.0038.0271.2017.01.11 
##                          60368                          23791 
##   WT40516.0122.0131.2017.01.11   WT41865.0089.0097.2017.01.11 
##                          69537                          24316 
##   WT41910.0095.0108.2017.01.11   WT42069.0154.0152.2017.01.11 
##                          43202                          25172 
##   WT43352.0087.0447.2017.01.11   WT43368.0118.0127.2017.01.11 
##                          15820                          50208 
##   WT43374.0048.0273.2017.01.11   WT44245.0081.0337.2017.01.11 
##                          57836                          54390 
##   WT44601.0082.0445.2017.01.11   WT44762.0130.0138.2017.01.11 
##                          41145                         118478 
##   WT47297.0173.0105.2017.01.11   WT48435.0099.0112.2017.01.11 
##                          30740                          39063 
##   WT48441.0112.0121.2017.01.11   WT48457.0060.0441.2017.01.11 
##                          31526                          41539 
##   WT48491.0146.0144.2017.01.11   WT48502.0106.0117.2017.01.11 
##                          28055                          63937 
##   WT50842.0187.0376.2017.01.11   WT50858.0128.0136.2017.01.11 
##                          20957                          36154 
##   WY74094.0079.0324.2017.01.11   WY74105.0151.0149.2017.01.11 
##                          55072                          67120 
##   WY74777.0073.0444.2017.01.11   WY74799.0176.0369.2017.01.11 
##                          45932                          27435 
##   WY75915.0114.0123.2017.01.11   WY75959.0191.0379.2017.01.11 
##                          27184                          28579 
##   WY75971.0062.0443.2017.01.11   WY76486.0162.0344.2017.01.11 
##                          49071                          52240 
##   WY78078.0161.0343.2017.01.11   WY78084.0061.0442.2017.01.11 
##                          29088                          15433 
##   WY78567.0119.0128.2017.01.11   WY79216.0040.0272.2017.01.11 
##                          21515                          42024 
##   WY79222.0056.0439.2017.01.11   WY79266.0113.0157.2017.01.11 
##                          27261                          55014 
##   WY79272.0177.0370.2017.01.11   WY80257.0027.0277.2017.01.11 
##                          31091                          65700 
##   WY80324.0137.0287.2017.01.11   WY81156.0104.0116.2017.01.11 
##                          37886                          41525 
##   WY81162.0169.0362.2017.01.11   WY81184.0045.0436.2017.01.11 
##                          65619                          23675 
##   XE13926.0025.0214.2017.01.11   XE13948.0192.0380.2017.01.11 
##                          25666                          43145 
##   XE13960.0150.0148.2017.01.11   XE15596.0149.0147.2017.01.11 
##                          34682                          51049 
##   XE17300.0083.0339.2017.01.11   XE21060.0015.0266.2017.01.11 
##                          60567                          39820 
##   XE21076.0182.0372.2017.01.11   XE21082.0186.0375.2017.01.11 
##                          37296                          20236 
##   XE21098.0115.0158.2017.01.11   XE22892.0171.0364.2017.01.11 
##                          42113                          50713 
##   XE22953.0096.0109.2017.01.11   XE27236.0054.0284.2017.01.11 
##                          52070                         135285 
##   XE27414.0059.0286.2017.01.11   XE28202.0028.0278.2017.01.11 
##                          51840                          44456 
##   XE29167.0135.0141.2017.01.11   XE30225.0020.0212.2017.01.11 
##                          64259                          24576 
##   XE31552.0011.0211.2017.01.11   XE33411.0143.0319.2017.01.11 
##                          20444                          38078 
##   XE36257.0175.0156.2017.01.11   XE36952.0091.0099.2017.01.11 
##                          48972                          11715 
##   XE36996.0009.0210.2017.01.11   XE38944.0167.0360.2017.01.11 
##                          38916                          26375 
##   XE38950.0140.0316.2017.01.11   XE38966.0034.0268.2017.01.11 
##                          38755                          55702 
##   XE38972.0178.0371.2017.01.11   XE38988.0110.0119.2017.01.11 
##                          35384                          32939 
##   XE38994.0185.0374.2017.01.11   XE40684.0024.0213.2017.01.11 
##                          21707                          25565 
##   XE40690.0172.0365.2017.01.11   XE40701.0163.0103.2017.01.11 
##                          46970                          46782 
##   XE40717.0010.0265.2017.01.11   XE40745.0006.0264.2017.01.11 
##                          44631                          59807 
##   XE40751.0088.0096.2017.01.11   XE41305.0193.0381.2017.01.11 
##                          17438                          27692 
##   XE41311.0138.0314.2017.01.11   XE41327.0085.0446.2017.01.11 
##                          63493                          13408 
##   XE41333.0136.0142.2017.01.11   XE41349.0168.0361.2017.01.11 
##                          44701                          26835 
##   XE41561.0037.0280.2017.01.11   XE41577.0170.0363.2017.01.11 
##                          44727                          31898 
##   XE41583.0183.0373.2017.01.11   WS19294.0064.0471.2016.11.13 
##                          26328                          58457 
##   WS20829.0126.0349.2016.11.13   WS20835.0007.0390.2016.11.13 
##                         136145                          35250 
##   WS21390.0076.0299.2016.11.13   WS21540.0031.0414.2016.11.13 
##                         144074                          52735 
##   WT02689.0077.0300.2016.11.13   WT02728.0109.0332.2016.11.13 
##                          70581                          83182 
##   WT04693.0016.0246.2016.11.13   WT04704.0071.0294.2016.11.13 
##                          60837                         133384 
##   WT07417.0184.0222.2016.11.13   WT08055.0075.0298.2016.11.13 
##                         122883                         116845 
##   WT09332.0070.0293.2016.11.13   WT09776.0074.0297.2016.11.13 
##                         159041                         156778 
##   WT10406.0003.0386.2016.11.13   WT11111.0023.0406.2016.11.13 
##                          24724                         196412 
##   WT12559.0072.0295.2016.11.13   WT14141.0002.0385.2016.11.13 
##                         192888                          57645 
##   WT15090.0180.0218.2016.11.13   WT23295.0022.0251.2016.11.13 
##                         106854                          68044 
##   WT27518.0050.0457.2016.11.13   WT27607.0039.0422.2016.11.13 
##                          32576                          90830 
##   WT30341.0012.0243.2016.11.13   WT30868.0042.0449.2016.11.13 
##                          55241                          74533 
##   WT30880.0069.0292.2016.11.13   WT34347.0181.0219.2016.11.13 
##                          81481                         103299 
##   WT37711.0068.0291.2016.11.13   WT42192.0131.0354.2016.11.13 
##                         146323                          52000 
##   WT47308.0004.0387.2016.11.13   WY76492.0067.0290.2016.11.13 
##                          54624                         132789 
##   WY78062.0019.0248.2016.11.13   WY79238.0108.0331.2016.11.13 
##                          67007                          37816 
##   WY79244.0105.0328.2016.11.13   WY80318.0103.0326.2016.11.13 
##                          16417                          53131 
##   WY82116.0188.0226.2016.11.13   XE15574.0065.0288.2016.11.13 
##                          79589                         203487 
##   XE17922.0179.0217.2016.11.13   XE17938.0014.0245.2016.11.13 
##                          93388                          67436 
##   XE18326.0084.0307.2016.11.13   XE21109.0018.0247.2016.11.13 
##                          81864                         105606 
##   XE28157.0013.0244.2016.11.13   XE28274.0051.0458.2016.11.13 
##                          94532                          24318 
##   XE29173.0008.0240.2016.11.13   XE29812.0132.0355.2016.11.13 
##                          82688                         143895 
##   XE33372.0021.0427.2016.11.13   XE36174.0063.0470.2016.11.13 
##                          74927                          92285 
##   XE37001.0030.0413.2016.11.13   XE39009.0029.0412.2016.11.13 
##                          80217                          48882 
##   XE39532.0017.0400.2016.11.13   XE39554.0041.0448.2016.11.13 
##                         205766                          65169 
##   XE40723.0001.0384.2016.11.13   XE40739.0086.0309.2016.11.13 
##                          61118                          34672 
##   XE41599.0005.0388.2016.11.13   MBA4060.0077.0266.2016.03.20 
##                          56531                          11517 
##   MBA1007.0092.0281.2016.03.11   MBA1037.0129.0437.2016.03.11 
##                          16176                          14385 
##   MBA1083.0088.0367.2016.03.11   MBA1141.0108.0297.2016.03.11 
##                          11423                          11454 
##   MBA1166.0047.0236.2016.03.11   MBA1172.0017.0444.2016.03.11 
##                          11848                          11809 
##   MBA1261.0125.0433.2016.03.11   MBA1327.0122.0368.2016.03.11 
##                          13925                          20343 
##   MBA1447.0019.0469.2016.03.11   MBA4044.0024.0470.2016.03.11 
##                          45329                          42889 
##   MBA4049.0043.0416.2016.03.11   MBA4051.0001.0446.2016.03.11 
##                          13037                          12351 
##   MBA4056.0119.0308.2016.03.11   MBA4065.0152.0341.2016.03.11 
##                          12105                          12002 
##   MBA4077.0040.0378.2016.03.11   MBA4085.0075.0264.2016.03.11 
##                          27897                          14923 
##   MBA4088.0082.0271.2016.03.11   MBA4091.0085.0429.2016.03.11 
##                           9947                          14136 
##   MBA4120.0076.0265.2016.03.11   MBA4121.0046.0235.2016.03.11 
##                          13922                          12858 
##   MBA4129.0087.0468.2016.03.11   MBA4130.0148.0337.2016.03.11 
##                          40274                          16263 
##   MBA4134.0120.0363.2016.03.11   MBA4139.0164.0353.2016.03.11 
##                          25631                          11278 
##  233202.Boston.0164.2016.02.14  930024.Boston.0114.2016.02.14 
##                          24841                          27023 
##   MBA1003.0131.0320.2016.02.14   MBA1021.0035.0460.2016.02.14 
##                          49506                          21557 
##   MBA1030.0014.0205.2016.02.14   MBA1033.0055.0244.2016.02.14 
##                          60325                          23191 
##   MBA1035.0102.0426.2016.02.14   MBA1041.0053.0242.2016.02.14 
##                          40213                          14230 
##   MBA1052.0009.0451.2016.02.14   MBA1071.0011.0202.2016.02.14 
##                          14859                          82865 
##   MBA1074.0058.0247.2016.02.14   MBA1082.0023.0463.2016.02.14 
##                          13002                          28556 
##   MBA1084.0114.0303.2016.02.14   MBA1090.0003.0194.2016.02.14 
##                          11543                         133259 
##   MBA1095.0090.0279.2016.02.14   MBA1096.0060.0249.2016.02.14 
##                          23430                          19672 
##   MBA1099.0066.0255.2016.02.14   MBA1100.0027.0386.2016.02.14 
##                          19574                          26058 
##   MBA1101.0149.0338.2016.02.14   MBA1103.0026.0385.2016.02.14 
##                          19728                          24858 
##   MBA1111.0139.0328.2016.02.14   MBA1133.0041.0453.2016.02.14 
##                          17707                          23140 
##   MBA1135.0140.0329.2016.02.14   MBA1139.0018.0445.2016.02.14 
##                           9599                          13882 
##   MBA1143.0105.0294.2016.02.14   MBA1151.0107.0296.2016.02.14 
##                          19881                          13159 
##   MBA1159.0165.0354.2016.02.14   MBA1163.0167.0356.2016.02.14 
##                          30857                          33492 
##   MBA1181.0126.0315.2016.02.14   MBA1187.0062.0251.2016.02.14 
##                          43998                          15695 
##   MBA1190.0048.0237.2016.02.14   MBA1193.0132.0321.2016.02.14 
##                          53414                          15292 
##   MBA1199.0069.0258.2016.02.14   MBA1202.0094.0456.2016.02.14 
##                          21096                          14588 
##   MBA1209.0101.0290.2016.02.14   MBA1211.0136.0325.2016.02.14 
##                          10734                          20052 
##   MBA1218.0030.0425.2016.02.14   MBA1230.0093.0282.2016.02.14 
##                          19204                          19109 
##   MBA1237.0170.0359.2016.02.14   MBA1241.0022.0419.2016.02.14 
##                          25519                          30836 
##   MBA1248.0070.0259.2016.02.14   MBA1251.0033.0467.2016.02.14 
##                          16468                          26599 
##   MBA1260.0158.0347.2016.02.14   MBA1267.0038.0227.2016.02.14 
##                          39840                          17977 
##   MBA1270.0042.0231.2016.02.14   MBA1271.0116.0462.2016.02.14 
##                          16729                          22420 
##   MBA1279.0162.0461.2016.02.14   MBA1307.0029.0372.2016.02.14 
##                          48196                          13686 
##   MBA1312.0074.0263.2016.02.14   MBA1317.0067.0256.2016.02.14 
##                          16407                          16233 
##   MBA1330.0050.0239.2016.02.14   MBA1341.0160.0349.2016.02.14 
##                          12379                          21517 
##   MBA1344.0169.0358.2016.02.14   MBA1355.0037.0458.2016.02.14 
##                          38110                          38263 
##   MBA1363.0123.0312.2016.02.14   MBA1365.0171.0360.2016.02.14 
##                          11750                          19399 
##   MBA1370.0034.0465.2016.02.14   MBA1375.0061.0250.2016.02.14 
##                          32525                          24576 
##   MBA1382.0159.0348.2016.02.14   MBA1385.0124.0313.2016.02.14 
##                          23025                          37460 
##   MBA1392.0134.0323.2016.02.14   MBA1399.0147.0336.2016.02.14 
##                          41620                          26657 
##   MBA1410.0130.0379.2016.02.14   MBA1437.0063.0252.2016.02.14 
##                          19712                          13941 
##   MBA1460.0106.0295.2016.02.14   MBA1480.0166.0355.2016.02.14 
##                           9819                          34300 
##   MBA1486.0115.0304.2016.02.14   MBA1488.0072.0452.2016.02.14 
##                          22751                          13626 
##   MBA1511.0163.0352.2016.02.14   MBA1515.0153.0342.2016.02.14 
##                          21490                          24838 
##   MBA4041.0044.0449.2016.02.14   MBA4043.0059.0248.2016.02.14 
##                          10254                          13060 
##   MBA4045.0157.0346.2016.02.14   MBA4047.0012.0203.2016.02.14 
##                          33741                          48792 
##   MBA4050.0028.0434.2016.02.14   MBA4052.0118.0457.2016.02.14 
##                          14519                          35830 
##   MBA4053.0057.0246.2016.02.14   MBA4057.0133.0322.2016.02.14 
##                          25496                           9954 
##   MBA4061.0010.0201.2016.02.14   MBA4062.0079.0393.2016.02.14 
##                         104643                          11876 
##   MBA4063.0100.0289.2016.02.14   MBA4066.0142.0430.2016.02.14 
##                          16644                          12023 
##   MBA4067.0104.0293.2016.02.14   MBA4068.0007.0198.2016.02.14 
##                          22257                          49188 
##   MBA4069.0004.0195.2016.02.14   MBA4070.0086.0478.2016.02.14 
##                          90587                          41192 
##   MBA4072.0161.0350.2016.02.14   MBA4074.0078.0267.2016.02.14 
##                          27345                          13679 
##   MBA4076.0016.0207.2016.02.14   MBA4078.0151.0340.2016.02.14 
##                          47724                          35591 
##   MBA4080.0065.0254.2016.02.14   MBA4081.0052.0241.2016.02.14 
##                          23350                          20586 
##   MBA4082.0032.0221.2016.02.14   MBA4086.0110.0299.2016.02.14 
##                          15687                          18709 
##   MBA4087.0155.0344.2016.02.14   MBA4089.0141.0330.2016.02.14 
##                          35300                          21650 
##   MBA4092.0008.0199.2016.02.14   MBA4095.0002.0193.2016.02.14 
##                          69046                         138863 
##   MBA4096.0145.0334.2016.02.14   MBA4102.0154.0343.2016.02.14 
##                          18861                          23941 
##   MBA4103.0128.0317.2016.02.14   MBA4106.0168.0357.2016.02.14 
##                          13480                          33202 
##   MBA4107.0138.0327.2016.02.14   MBA4108.0099.0288.2016.02.14 
##                          12705                          14653 
##   MBA4109.0073.0262.2016.02.14   MBA4111.0036.0459.2016.02.14 
##                          10720                          38086 
##   MBA4112.0005.0196.2016.02.14   MBA4113.0137.0396.2016.02.14 
##                          59252                          13144 
##   MBA4114.0064.0253.2016.02.14   MBA4115.0143.0332.2016.02.14 
##                          13795                           9242 
##   MBA4118.0031.0466.2016.02.14   MBA4119.0127.0316.2016.02.14 
##                          42048                          13338 
##   MBA4122.0112.0301.2016.02.14   MBA4123.0056.0245.2016.02.14 
##                          28583                          21135 
##   MBA4126.0150.0339.2016.02.14   MBA4127.0021.0417.2016.02.14 
##                           8596                          18287 
##   MBA4131.0006.0197.2016.02.14   MBA4132.0051.0240.2016.02.14 
##                         121717                          12687 
##   MBA4133.0135.0324.2016.02.14   MBA4136.0020.0464.2016.02.14 
##                          18548                          26125 
##   MBA4138.0068.0257.2016.02.14   MBA4140.0015.0206.2016.02.14 
##                          21282                          32717 
##  226855.boston.0016.2015.11.25  229075.boston.0178.2015.11.25 
##                          12316                          21754 
##  447537.boston.0170.2015.11.25  503564.boston.0169.2015.11.25 
##                          11055                          16113 
##  561130.boston.0065.2015.11.25  588800.boston.0068.2015.11.25 
##                          64533                          54160 
##  629356.boston.0076.2015.11.25  765828.boston.0089.2015.11.25 
##                          17564                          18760 
##  772512.boston.0090.2015.11.25  826391.boston.0177.2015.11.25 
##                          28599                          29717 
##  849016.boston.0176.2015.11.25  872569.boston.0179.2015.11.25 
##                          27270                          22343 
##     136109.048.0334.2018.12.15     137787.006.0293.2018.12.15 
##                         142042                         110949 
##     186400.027.0314.2018.12.15     207722.029.0316.2018.12.15 
##                         130465                         140162 
##     221355.060.0345.2018.12.15     225794.001.0288.2018.12.15 
##                         131800                         169725 
##     235185.033.0320.2018.12.15     236512.015.0302.2018.12.15 
##                         146986                         136583 
##     240884.013.0300.2018.12.15     241367.042.0329.2018.12.15 
##                         134087                         130533 
##     243736.036.0323.2018.12.15     276693.051.0337.2018.12.15 
##                         125215                         143542 
##     282036.026.0313.2018.12.15     298053.032.0319.2018.12.15 
##                         140377                         139377 
##     319820.035.0322.2018.12.15     354109.012.0299.2018.12.15 
##                         156031                         130651 
##     356403.053.0339.2018.12.15     364485.002.0289.2018.12.15 
##                         163928                         138715 
##     373167.021.0308.2018.12.15     395379.010.0297.2018.12.15 
##                          91803                         125471 
##     402235.052.0338.2018.12.15     411794.028.0315.2018.12.15 
##                         103407                         125418 
##     416151.055.0340.2018.12.15     420641.004.0291.2018.12.15 
##                         147234                         137640 
##     423443.005.0292.2018.12.15     445474.025.0312.2018.12.15 
##                         153787                         162503 
##     453551.040.0327.2018.12.15     459452.044.0331.2018.12.15 
##                         149435                         105418 
##     467375.007.0294.2018.12.15     471179.003.0290.2018.12.15 
##                         148513                         139922 
##     492275.058.0343.2018.12.15     570298.041.0328.2018.12.15 
##                         165014                         135343 
##     574541.045.0332.2018.12.15     576662.030.0317.2018.12.15 
##                         124317                         151416 
##     578598.017.0304.2018.12.15     580176.038.0325.2018.12.15 
##                         121232                         141927 
##     588443.031.0318.2018.12.15     588873.064.0349.2018.12.15 
##                          94483                         109394 
##     612872.019.0306.2018.12.15     667045.043.0330.2018.12.15 
##                         139365                         116480 
##     675294.009.0296.2018.12.15     684908.056.0341.2018.12.15 
##                         138860                         122542 
##     714983.057.0342.2018.12.15     720750.034.0321.2018.12.15 
##                         145724                         121774 
##     774051.062.0347.2018.12.15     821034.039.0326.2018.12.15 
##                         147680                         119747 
##     822655.037.0324.2018.12.15     823946.047.0333.2018.12.15 
##                          93713                         121833 
##     850240.022.0309.2018.12.15     851204.014.0301.2018.12.15 
##                         107960                         145594 
##     853781.063.0348.2018.12.15     857829.018.0305.2018.12.15 
##                         130937                         136894 
##     883092.049.0335.2018.12.15     909074.024.0311.2018.12.15 
##                         126602                          83194 
##     918320.061.0346.2018.12.15     938150.059.0344.2018.12.15 
##                         150659                         159642 
##     945496.008.0295.2018.12.15     948983.050.0336.2018.12.15 
##                         130406                         160584 
##     982757.020.0307.2018.12.15     985170.011.0298.2018.12.15 
##                         135822                         132462 
##    MBA1008.062.0371.2018.12.15    MBA1073.103.0380.2018.12.15 
##                          97776                         121454 
##    MBA1144.144.0244.2018.12.15    MBA1215.004.0359.2018.12.15 
##                          58436                         141749 
##    MBA1226.010.0360.2018.12.15    MBA1240.156.0246.2018.12.15 
##                         118025                         123306 
##    MBA1284.025.0362.2018.12.15    MBA1302.089.0375.2018.12.15 
##                         106064                         122557 
##    MBA1315.035.0365.2018.12.15    MBA1398.080.0374.2018.12.15 
##                         122382                         124340 
##    MBA1491.070.0372.2018.12.15    MBA1509.039.0366.2018.12.15 
##                         141649                         123099 
##    MBA1519.096.0377.2018.12.15    MBA4042.011.0361.2018.12.15 
##                         105745                         135959 
##    MBA4046.032.0363.2018.12.15    MBA4048.146.0245.2018.12.15 
##                         121271                         104141 
##    MBA4058.049.0368.2018.12.15    MBA4059.113.0242.2018.12.15 
##                         163357                         154949 
##    MBA4064.117.0243.2018.12.15    MBA4071.098.0379.2018.12.15 
##                          99265                         173599 
##    MBA4083.097.0378.2018.12.15    MBA4093.033.0364.2018.12.15 
##                         131209                         131778 
##    MBA4099.045.0367.2018.12.15    MBA4104.091.0376.2018.12.15 
##                         140969                         128881 
##    MBA4128.059.0369.2018.12.15 
##                         142304
```

```
min(rowSums(OTU_table))
```

```
## [1] 8596
```

```
Total_counts<-as.data.frame(rowSums(OTU_table))
colnames(Total_counts)<-c("Counts")
ggplot2::ggplot(Total_counts, aes(x=Counts))+geom_histogram(binwidth=100)+geom_vline(aes(xintercept=mean(Counts, na.rm=T)), color="red", linetype="dashed", size=1)+theme_bw()+
  ggtitle("Total count per sample distribution")+theme(plot.title=element_text(lineheight=10, size=15))+
  xlab("Counts")+ylab("Number of samples")+theme(axis.text=element_text(size=15), axis.title=element_text(size=15))
```

```
#Remove all those samples that do not reach a minimum threshold of number of reads:
counts<-as.data.frame(rowSums(OTU_table))
colnames(counts)<-"counts"
counts$Sample<-row.names(counts)
subset_8000<-counts[counts$counts>=8000,]
subset_8000$Sample<-NULL
OTU_table<-OTU_table[row.names(OTU_table)%in%as.vector(row.names(subset_8000)),]

#Rarefy to same sequencing depth:
set.seed(1)
OTU_table_8000<-vegan::rrarefy(OTU_table, 8000)
```

```
## Warning in vegan::rrarefy(OTU_table, 8000): function should be used for
## observed counts, but smallest count is 2
```

```
#Estimate richness:
richness_8000<-vegan::estimateR(OTU_table_8000)
richness_8000<-t(richness_8000)
richness_8000<-as.data.frame(richness_8000)
richness_8000$se.chao1<-NULL
richness_8000$se.ACE<-NULL

#Estimate evenness:
shannon<-BiodiversityR::diversityresult(x=OTU_table_8000, method="each site", index="Shannon")
diversity_8000<-cbind(shannon)

#Join data from richness and evenness calculations:
ecology_8000<-cbind(richness_8000, diversity_8000)
colnames(ecology_8000)<-c("Observed", "Chao1", "ACE", "Shannon")

#Subset metadata
metadata<-metadata[row.names(metadata)%in%row.names(ecology_8000),,drop=FALSE]

all.equal(row.names(metadata),row.names(ecology_8000))
```

```
## [1] TRUE
```

```
ecology_8000$SampleID<-metadata$SampleID
ecology_8000$Cohort<-metadata$sample_cohort
ecology_8000$hiv_phenotype<-metadata$hiv_phenotype
ecology_8000$sexual_orientation<-metadata$sexual_orientation

#Subset indivudals for this comparison
ecology_8000_nomsm<-ecology_8000[ecology_8000$sexual_orientation!="MSM",,drop=FALSE]
ecology_8000_nomsm_neg<-ecology_8000_nomsm[ecology_8000_nomsm$hiv_phenotype=="1_hiv_negative",,drop=F]
ecology_8000_nomsm_neg_melt<-reshape2::melt(ecology_8000_nomsm_neg)
```

```
## Using SampleID, Cohort, hiv_phenotype, sexual_orientation as id variables
```

```
ggplot2::ggsave("Figure1E.pdf", ggplot(data=ecology_8000_nomsm_neg_melt, aes(x=Cohort,y=value))+geom_boxplot(aes(fill=Cohort), alpha=0.5, outlier.color="white")+theme_bw()+
  scale_fill_manual(values=c("royalblue4","darkorange","forestgreen"))+geom_point(aes(color=Cohort), position=position_jitterdodge(jitter.width=0.25),alpha=0.75, size=1)+
  facet_wrap(~variable, scales="free_y", nrow=1)+scale_color_manual(values=c("royalblue4","darkorange","forestgreen"))+
  theme(axis.text.x = element_text(angle=90))+ggtitle("NEG"), width=10, height=15)

for (i in unique(colnames(ecology_8000_nomsm_neg[1:4]))){
  print(i)
  print(agricolae::kruskal(ecology_8000_nomsm_neg[i], ecology_8000_nomsm_neg["Cohort"],group=F,p.adj = "bonferroni"))
}
```

```
## [1] "Observed"
## $statistics
##       Chisq Df    p.chisq
##   3.7586679  2 0.15269178
## 
## $parameters
##             test  p.ajusted                           name.t ntr alpha
##   Kruskal-Wallis bonferroni ecology_8000_nomsm_neg["Cohort"]   3  0.05
## 
## $means
##          ecology_8000_nomsm_neg.i.      rank       std  r Min Max   Q25   Q50
## boston                   138.62353 131.57647 48.321093 85  61 249 96.00 137.0
## botswana                 136.41250 126.06875 55.522306 80  32 268 93.00 131.0
## uganda_2                 137.65000 110.81875 86.018558 80  38 446 81.25 105.5
##             Q75
## boston   175.00
## botswana 171.25
## uganda_2 170.00
## 
## $comparison
##                     Difference pvalue Signif.         LCL       UCL
## boston - botswana    5.5077206 1.0000         -21.0074575 32.022899
## boston - uganda_2   20.7577206 0.1810          -5.7574575 47.272899
## botswana - uganda_2 15.2500000 0.5196         -11.6639250 42.163925
## 
## $groups
## NULL
## 
## attr(,"class")
## [1] "group"
## [1] "Chao1"
## $statistics
##       Chisq Df    p.chisq
##   4.2493326  2 0.11947283
## 
## $parameters
##             test  p.ajusted                           name.t ntr alpha
##   Kruskal-Wallis bonferroni ecology_8000_nomsm_neg["Cohort"]   3  0.05
## 
## $means
##          ecology_8000_nomsm_neg.i.      rank       std  r   Min       Max
## boston                   145.50557 132.41176 55.745381 85 61.25 287.04167
## botswana                 140.79680 125.83125 59.032139 80 32.00 305.04348
## uganda_2                 143.70496 110.16875 96.446000 80 38.00 499.62500
##                Q25       Q50       Q75
## boston   97.000000 137.33333 180.04762
## botswana 94.857143 136.81250 174.05000
## uganda_2 81.375000 106.67143 174.57143
## 
## $comparison
##                     Difference pvalue Signif.         LCL       UCL
## boston - botswana    6.5805147 1.0000         -19.9085452 33.069575
## boston - uganda_2   22.2430147 0.1321          -4.2460452 48.732075
## botswana - uganda_2 15.6625000 0.4845         -11.2249140 42.549914
## 
## $groups
## NULL
## 
## attr(,"class")
## [1] "group"
## [1] "ACE"
## $statistics
##       Chisq Df    p.chisq
##   4.1911381  2 0.12300023
## 
## $parameters
##             test  p.ajusted                           name.t ntr alpha
##   Kruskal-Wallis bonferroni ecology_8000_nomsm_neg["Cohort"]   3  0.05
## 
## $means
##          ecology_8000_nomsm_neg.i.      rank       std  r       Min       Max
## boston                   144.85963 132.31765 54.678722 85 61.655263 276.58237
## botswana                 140.37235 125.85625 58.569433 80 32.000000 291.77820
## uganda_2                 143.75177 110.24375 96.453998 80 38.000000 494.32519
##                Q25       Q50       Q75
## boston   97.198442 137.38925 182.67036
## botswana 95.133580 135.42375 173.76443
## uganda_2 81.694465 106.82082 174.51065
## 
## $comparison
##                     Difference pvalue Signif.         LCL       UCL
## boston - botswana    6.4613971 1.0000         -20.0310612 32.953855
## boston - uganda_2   22.0738971 0.1371          -4.4185612 48.566355
## botswana - uganda_2 15.6125000 0.4887         -11.2783635 42.503363
## 
## $groups
## NULL
## 
## attr(,"class")
## [1] "group"
## [1] "Shannon"
## $statistics
##       Chisq Df    p.chisq
##   4.4679718  2 0.10710069
## 
## $parameters
##             test  p.ajusted                           name.t ntr alpha
##   Kruskal-Wallis bonferroni ecology_8000_nomsm_neg["Cohort"]   3  0.05
## 
## $means
##          ecology_8000_nomsm_neg.i.      rank        std  r       Min       Max
## boston                   3.4220815 132.55294 0.68648328 85 1.4674836 4.4513155
## botswana                 3.1739522 109.80000 0.81095019 80 1.1161733 4.5147266
## uganda_2                 3.4005418 126.05000 0.66001093 80 1.6025618 4.9432966
##                Q25       Q50       Q75
## boston   3.0708710 3.5533268 3.9279747
## botswana 2.5829754 3.1852682 3.7863951
## uganda_2 3.0940477 3.4318150 3.8541692
## 
## $comparison
##                      Difference pvalue Signif.         LCL       UCL
## boston - botswana    22.7529412 0.1181          -3.7242268 49.230109
## boston - uganda_2     6.5029412 1.0000         -19.9742268 32.980109
## botswana - uganda_2 -16.2500000 0.4387         -43.1253432 10.625343
## 
## $groups
## NULL
## 
## attr(,"class")
## [1] "group"
```

```
#--------------------------------------------------------------------------------------------------------------
```
