# Supplementary material for: HIV-associated gut microbial alterations are dependent on host and geographic context
Source: Nat Commun. 2024 Feb 5;15:1055. doi: 10.1038/s41467-023-44566-4 (PMC10844288; doi:10.1038/s41467-023-44566-4)
Supplement: Supplementary file 9 — Figure2ANCOM_Rocafort-Gootenberg_2023_03_08 [file 41467_2023_44566_MOESM9_ESM.html]

Rocafort-Gootenberg\_Figure2ANCOM


# Rocafort-Gootenberg\_Figure2ANCOM

#Load needed R packages

```
library("phyloseq")
library("tidyverse")
```

```
## ── Attaching packages ─────────────────────────────────────── tidyverse 1.3.2 ──
## ✔ ggplot2 3.4.1     ✔ purrr   1.0.1
## ✔ tibble  3.1.8     ✔ dplyr   1.1.0
## ✔ tidyr   1.3.0     ✔ stringr 1.5.0
## ✔ readr   2.1.4     ✔ forcats 1.0.0
## ── Conflicts ────────────────────────────────────────── tidyverse_conflicts() ──
## ✖ dplyr::filter() masks stats::filter()
## ✖ dplyr::lag()    masks stats::lag()
```

```
library("ggplot2")
library("dplyr")
library("gridExtra")
```

```
## 
## Attaching package: 'gridExtra'
## 
## The following object is masked from 'package:dplyr':
## 
##     combine
```

```
library("vegan")
```

```
## Loading required package: permute
## Loading required package: lattice
## This is vegan 2.6-4
```

```
library("knitr") 
library("reshape")
```

```
## 
## Attaching package: 'reshape'
## 
## The following object is masked from 'package:dplyr':
## 
##     rename
## 
## The following objects are masked from 'package:tidyr':
## 
##     expand, smiths
```

```
library("DESeq2")
```

```
## Loading required package: S4Vectors
## Loading required package: stats4
## Loading required package: BiocGenerics
## 
## Attaching package: 'BiocGenerics'
## 
## The following object is masked from 'package:gridExtra':
## 
##     combine
## 
## The following objects are masked from 'package:dplyr':
## 
##     combine, intersect, setdiff, union
## 
## The following objects are masked from 'package:stats':
## 
##     IQR, mad, sd, var, xtabs
## 
## The following objects are masked from 'package:base':
## 
##     anyDuplicated, aperm, append, as.data.frame, basename, cbind,
##     colnames, dirname, do.call, duplicated, eval, evalq, Filter, Find,
##     get, grep, grepl, intersect, is.unsorted, lapply, Map, mapply,
##     match, mget, order, paste, pmax, pmax.int, pmin, pmin.int,
##     Position, rank, rbind, Reduce, rownames, sapply, setdiff, sort,
##     table, tapply, union, unique, unsplit, which.max, which.min
## 
## 
## Attaching package: 'S4Vectors'
## 
## The following objects are masked from 'package:reshape':
## 
##     expand, rename
## 
## The following objects are masked from 'package:dplyr':
## 
##     first, rename
## 
## The following object is masked from 'package:tidyr':
## 
##     expand
## 
## The following objects are masked from 'package:base':
## 
##     expand.grid, I, unname
## 
## Loading required package: IRanges
## 
## Attaching package: 'IRanges'
## 
## The following objects are masked from 'package:dplyr':
## 
##     collapse, desc, slice
## 
## The following object is masked from 'package:purrr':
## 
##     reduce
## 
## The following object is masked from 'package:phyloseq':
## 
##     distance
## 
## Loading required package: GenomicRanges
## Loading required package: GenomeInfoDb
## Loading required package: SummarizedExperiment
## Loading required package: MatrixGenerics
## Loading required package: matrixStats
## 
## Attaching package: 'matrixStats'
## 
## The following object is masked from 'package:dplyr':
## 
##     count
## 
## 
## Attaching package: 'MatrixGenerics'
## 
## The following objects are masked from 'package:matrixStats':
## 
##     colAlls, colAnyNAs, colAnys, colAvgsPerRowSet, colCollapse,
##     colCounts, colCummaxs, colCummins, colCumprods, colCumsums,
##     colDiffs, colIQRDiffs, colIQRs, colLogSumExps, colMadDiffs,
##     colMads, colMaxs, colMeans2, colMedians, colMins, colOrderStats,
##     colProds, colQuantiles, colRanges, colRanks, colSdDiffs, colSds,
##     colSums2, colTabulates, colVarDiffs, colVars, colWeightedMads,
##     colWeightedMeans, colWeightedMedians, colWeightedSds,
##     colWeightedVars, rowAlls, rowAnyNAs, rowAnys, rowAvgsPerColSet,
##     rowCollapse, rowCounts, rowCummaxs, rowCummins, rowCumprods,
##     rowCumsums, rowDiffs, rowIQRDiffs, rowIQRs, rowLogSumExps,
##     rowMadDiffs, rowMads, rowMaxs, rowMeans2, rowMedians, rowMins,
##     rowOrderStats, rowProds, rowQuantiles, rowRanges, rowRanks,
##     rowSdDiffs, rowSds, rowSums2, rowTabulates, rowVarDiffs, rowVars,
##     rowWeightedMads, rowWeightedMeans, rowWeightedMedians,
##     rowWeightedSds, rowWeightedVars
## 
## Loading required package: Biobase
## Welcome to Bioconductor
## 
##     Vignettes contain introductory material; view with
##     'browseVignettes()'. To cite Bioconductor, see
##     'citation("Biobase")', and for packages 'citation("pkgname")'.
## 
## 
## Attaching package: 'Biobase'
## 
## The following object is masked from 'package:MatrixGenerics':
## 
##     rowMedians
## 
## The following objects are masked from 'package:matrixStats':
## 
##     anyMissing, rowMedians
## 
## The following object is masked from 'package:phyloseq':
## 
##     sampleNames
```

```
library("ANCOMBC")
library("ComplexHeatmap")
```

```
## Loading required package: grid
## ========================================
## ComplexHeatmap version 2.14.0
## Bioconductor page: http://bioconductor.org/packages/ComplexHeatmap/
## Github page: https://github.com/jokergoo/ComplexHeatmap
## Documentation: http://jokergoo.github.io/ComplexHeatmap-reference
## 
## If you use it in published research, please cite either one:
## - Gu, Z. Complex Heatmap Visualization. iMeta 2022.
## - Gu, Z. Complex heatmaps reveal patterns and correlations in multidimensional 
##     genomic data. Bioinformatics 2016.
## 
## 
## The new InteractiveComplexHeatmap package can directly export static 
## complex heatmaps into an interactive Shiny app with zero effort. Have a try!
## 
## This message can be suppressed by:
##   suppressPackageStartupMessages(library(ComplexHeatmap))
## ========================================
```

```
library("usedist")
library("gplots")
```

```
## Registered S3 method overwritten by 'gplots':
##   method         from     
##   reorder.factor DescTools
## 
## Attaching package: 'gplots'
## 
## The following object is masked from 'package:IRanges':
## 
##     space
## 
## The following object is masked from 'package:S4Vectors':
## 
##     space
## 
## The following object is masked from 'package:stats':
## 
##     lowess
```

```
library("ggpattern")
library("BioVenn")
library("eulerr")
```

```
## Registered S3 method overwritten by 'eulerr':
##   method    from  
##   plot.venn gplots
## 
## Attaching package: 'eulerr'
## 
## The following object is masked from 'package:gplots':
## 
##     venn
```

```
library("ggplotify")
```

#Load original phyloseq object output from DADA2 pipeline and pull in
new metadata

```
ps_gg_fp_f_prevalence_filter_2019_05_26<-readRDS("ps_gg_fp_f_prevalence_filter_2019_05_26")
readr::read_csv(
  "Metadata_formatted_nat_comm_add_2021_10_24.csv",
  col_names = TRUE,
  col_types = NULL,
  col_select = NULL,
  id = NULL,
  locale = default_locale(),
  na = c("", "NA", "empty", "EMPTY"),
  quote = "\"",
  comment = "",
  trim_ws = TRUE,
  skip = 0,
  name_repair = "unique",
  num_threads = readr_threads(),
  progress = show_progress(),
  show_col_types = should_show_types(),
  skip_empty_rows = TRUE,
  lazy = TRUE
) -> new_metadata
```

```
## Rows: 597 Columns: 88
## ── Column specification ────────────────────────────────────────────────────────
## Delimiter: ","
## chr (26): X, SampleID, subject_id, Race, Ethnicity, unique_id, sequencing_da...
## dbl (62): primer_used, read_count, age, height_cm, height_in, weight_kg, wei...
## 
## ℹ Use `spec()` to retrieve the full column specification for this data.
## ℹ Specify the column types or set `show_col_types = FALSE` to quiet this message.
```

```
### add {SampleID} as rownames
new_metadata_as_sample_data <- phyloseq::sample_data(new_metadata)
phyloseq::sample_names(new_metadata_as_sample_data) <- dplyr::pull(new_metadata, 1)
phyloseq::sample_data(ps_gg_fp_f_prevalence_filter_2019_05_26) <- new_metadata_as_sample_data
```

```
## Found more than one class "phylo" in cache; using the first, from namespace 'phyloseq'
## Also defined by 'tidytree'
## Found more than one class "phylo" in cache; using the first, from namespace 'phyloseq'
## Also defined by 'tidytree'
## Found more than one class "phylo" in cache; using the first, from namespace 'phyloseq'
## Also defined by 'tidytree'
## Found more than one class "phylo" in cache; using the first, from namespace 'phyloseq'
## Also defined by 'tidytree'
## Found more than one class "phylo" in cache; using the first, from namespace 'phyloseq'
## Also defined by 'tidytree'
## Found more than one class "phylo" in cache; using the first, from namespace 'phyloseq'
## Also defined by 'tidytree'
## Found more than one class "phylo" in cache; using the first, from namespace 'phyloseq'
## Also defined by 'tidytree'
```

```
#Fix randomness
set.seed(1)
```

#Figure 2A

```
#FIGURE 2A
#--------------------------------------------------------------------------------------------------------------
#COMPARISON 1: NEG vs ART
#Transform count data in the phyloseq object
ps_gg_fp_f_prevalence_filter_2019_05_26_proportion<-phyloseq::transform_sample_counts(ps_gg_fp_f_prevalence_filter_2019_05_26, function(x)(x/sum(x)))
```

```
## Found more than one class "phylo" in cache; using the first, from namespace 'phyloseq'
```

```
## Also defined by 'tidytree'
```

```
## Found more than one class "phylo" in cache; using the first, from namespace 'phyloseq'
```

```
## Also defined by 'tidytree'
```

```
## Found more than one class "phylo" in cache; using the first, from namespace 'phyloseq'
```

```
## Also defined by 'tidytree'
```

```
## Found more than one class "phylo" in cache; using the first, from namespace 'phyloseq'
```

```
## Also defined by 'tidytree'
```

```
#Select samples of interest and update phyloseq object 
metadata<-phyloseq::sample_data(ps_gg_fp_f_prevalence_filter_2019_05_26)
metadata<-metadata[metadata$hiv_phenotype %in% c("1_hiv_negative","2_suppressed"),,drop=F]
metadata<-as.data.frame(as.matrix(metadata[metadata$sexual_orientation != "MSM",,drop=FALSE])) ### swap to dropping "MSM" 
phyloseq::sample_data(ps_gg_fp_f_prevalence_filter_2019_05_26_proportion)<-metadata
```

```
## Found more than one class "phylo" in cache; using the first, from namespace 'phyloseq'
## Also defined by 'tidytree'
```

```
## Found more than one class "phylo" in cache; using the first, from namespace 'phyloseq'
```

```
## Also defined by 'tidytree'
```

```
## Found more than one class "phylo" in cache; using the first, from namespace 'phyloseq'
```

```
## Also defined by 'tidytree'
```

```
## Found more than one class "phylo" in cache; using the first, from namespace 'phyloseq'
```

```
## Also defined by 'tidytree'
```

```
#Run PCoA on the phyloseq object
ordination<-phyloseq::ordinate(ps_gg_fp_f_prevalence_filter_2019_05_26_proportion, "PCoA", "unifrac")
```

```
## Warning in matrix(tree$edge[order(tree$edge[, 1]), ][, 2], byrow = TRUE, : data
## length [8987] is not a sub-multiple or multiple of the number of rows [4494]
```

```
ordination$values[1:2,]
```

```
##   Eigenvalues Relative_eig Rel_corr_eig Broken_stick Cum_corr_eig
## 1    8.666120   0.05178965   0.05085708   0.01560113   0.05085708
## 2    8.650009   0.05169337   0.05076261   0.01324819   0.10161970
##   Cumul_br_stick
## 1     0.01560113
## 2     0.02884932
```

```
metadata_ordered<-metadata[row.names(ordination$vectors),,drop=FALSE]

all.equal(row.names(metadata_ordered), row.names(ordination$vectors))
```

```
## [1] TRUE
```

```
metadata_ordered$Unifrac1 <- ordination$vectors[,1]
metadata_ordered$Unifrac2 <- ordination$vectors[,2]

comparison1 <- metadata_ordered
comparison1$Grouping <- as.factor(paste(comparison1$hiv_phenotype, comparison1$sample_cohort, sep="_"))

#Let's plot the data
us<-comparison1[comparison1$sample_cohort == "boston",,drop=F]
botswana<-comparison1[comparison1$sample_cohort == "botswana",,drop=F]
uganda<-comparison1[comparison1$sample_cohort == "uganda_2",,drop=F]

plot_us_comparison<-ggplot2::ggplot(data=us, aes(x=Unifrac1, y=Unifrac2))+geom_point(color="royalblue4", aes(alpha=hiv_phenotype), size=2, shape=16)+
  theme_bw()+stat_ellipse(color="royalblue4", aes(alpha=hiv_phenotype), size=1)+scale_alpha_manual(values=c(1,0.3))+ylim(c(-0.4, 0.4))+xlim(c(-0.4,0.5))+
  ggtitle("neg-art boston")+geom_point(data=us %>% group_by(hiv_phenotype) %>% summarise_at(vars(matches("UniFrac")), mean),size=5, color="royalblue4", aes(alpha=hiv_phenotype))
```

```
## Warning: Using `size` aesthetic for lines was deprecated in ggplot2 3.4.0.
## ℹ Please use `linewidth` instead.
```

```
plot_botswana_comparison<-ggplot(data=botswana, aes(x=Unifrac1, y=Unifrac2))+geom_point(color="darkorange", aes(alpha=hiv_phenotype), size=2, shape=16)+
  theme_bw()+stat_ellipse(color="darkorange", aes(alpha=hiv_phenotype), size=1)+scale_alpha_manual(values=c(1,0.3))+ylim(c(-0.4, 0.4))+xlim(c(-0.4,0.5))+
  ggtitle("neg-art botswana")+geom_point(data=botswana %>% group_by(hiv_phenotype) %>% summarise_at(vars(matches("UniFrac")), mean),size=5, color="darkorange", aes(alpha=hiv_phenotype))
plot_uganda_comparison<-ggplot(data=uganda, aes(x=Unifrac1, y=Unifrac2))+geom_point(color="forestgreen", aes(alpha=hiv_phenotype), size=2, shape=16)+
  theme_bw()+stat_ellipse(color="forestgreen", aes(alpha=hiv_phenotype), size=1)+scale_alpha_manual(values=c(1,0.3))+ylim(c(-0.4, 0.4))+xlim(c(-0.4,0.5))+
  ggtitle("neg-art uganda")+geom_point(data=uganda %>% group_by(hiv_phenotype) %>% summarise_at(vars(matches("UniFrac")), mean),size=5, color="forestgreen", aes(alpha=hiv_phenotype))

ggsave("Figure2A_1v3.pdf", grid.arrange(plot_us_comparison,plot_botswana_comparison,plot_uganda_comparison, nrow=3, ncol=3), width=15, height=10)
```

```
#Adonis (n = 427)
ASV_table <- as.data.frame(phyloseq::otu_table(ps_gg_fp_f_prevalence_filter_2019_05_26_proportion))
all.equal(row.names(ASV_table), row.names(metadata_ordered))
```

```
## [1] TRUE
```

```
unifrac.distance <- unname(phyloseq::UniFrac(ps_gg_fp_f_prevalence_filter_2019_05_26_proportion, weighted = FALSE)) ### unname fixes error introduced by Desctools see https://github.com/joey711/phyloseq/issues/1457
```

```
## Warning in matrix(tree$edge[order(tree$edge[, 1]), ][, 2], byrow = TRUE, : data
## length [8987] is not a sub-multiple or multiple of the number of rows [4494]
```

```
attributes(unifrac.distance)$Labels <- phyloseq::sample_names(ps_gg_fp_f_prevalence_filter_2019_05_26_proportion)
print(vegan::adonis2(unifrac.distance ~ metadata_ordered$hiv_phenotype, data = ASV_table, permutations = 1000)) -> adon_hiv_phenotype
```

```
## Permutation test for adonis under reduced model
## Terms added sequentially (first to last)
## Permutation: free
## Number of permutations: 1000
## 
## vegan::adonis2(formula = unifrac.distance ~ metadata_ordered$hiv_phenotype, data = ASV_table, permutations = 1000)
##                                 Df SumOfSqs      R2      F   Pr(>F)    
## metadata_ordered$hiv_phenotype   1    1.148 0.00686 2.9357 0.000999 ***
## Residual                       425  166.185 0.99314                    
## Total                          426  167.333 1.00000                    
## ---
## Signif. codes:  0 '***' 0.001 '**' 0.01 '*' 0.05 '.' 0.1 ' ' 1
```

```
###*** hiv_phenotype r2 = 0.00686 p = 0.000999
print(vegan::adonis2(unifrac.distance ~ metadata_ordered$sample_cohort, data = ASV_table, permutations = 1000)) -> adon_cohort
```

```
## Permutation test for adonis under reduced model
## Terms added sequentially (first to last)
## Permutation: free
## Number of permutations: 1000
## 
## vegan::adonis2(formula = unifrac.distance ~ metadata_ordered$sample_cohort, data = ASV_table, permutations = 1000)
##                                 Df SumOfSqs      R2      F   Pr(>F)    
## metadata_ordered$sample_cohort   2    8.094 0.04837 10.776 0.000999 ***
## Residual                       424  159.239 0.95163                    
## Total                          426  167.333 1.00000                    
## ---
## Signif. codes:  0 '***' 0.001 '**' 0.01 '*' 0.05 '.' 0.1 ' ' 1
```

```
###*** sample_cohort r2 = 0.04837 p = 0.000999
print(vegan::adonis2(unifrac.distance ~ metadata_ordered$hiv_phenotype + metadata_ordered$sample_cohort, data = ASV_table, permutations = 1000)) -> adon_cohort_phenotype
```

```
## Permutation test for adonis under reduced model
## Terms added sequentially (first to last)
## Permutation: free
## Number of permutations: 1000
## 
## vegan::adonis2(formula = unifrac.distance ~ metadata_ordered$hiv_phenotype + metadata_ordered$sample_cohort, data = ASV_table, permutations = 1000)
##                                 Df SumOfSqs      R2       F   Pr(>F)    
## metadata_ordered$hiv_phenotype   1    1.148 0.00686  3.0702 0.000999 ***
## metadata_ordered$sample_cohort   2    8.026 0.04797 10.7332 0.000999 ***
## Residual                       423  158.159 0.94517                     
## Total                          426  167.333 1.00000                     
## ---
## Signif. codes:  0 '***' 0.001 '**' 0.01 '*' 0.05 '.' 0.1 ' ' 1
```

```
###*** sample_cohort r2 = 0.04797 p = 0.000999, hiv_phenotype r2 = 0.00686 p = 0.000999


#--------------------------------------------------------------------------------------------------------------

### COMPARISON 1: Adonis: controlling for additional metadata
#--------------------------------------------------------------------------------------------------------------
### Extra metadata that have full n: Race, Ethnicity, age, sex, current_art_class_consolid2, tmp_smx_active
### Extra metadata that have <n: BMI, comorbidities (hld_hx, htn_hx, cvd_hx, ckd_hx, cvd_dx [missing boston], dm2hx_dx, hldhx_dx, htnhx_dx, cvdhx_dx, ever_smoke, current_smoke, smoke_cat), school_level [uganda2 only], monthly_income[uganda2 only], current_art_class_consolid2, tmp_smx_active, days_on_art, sexual_orientation
### additional: smoking_years, fram_10yr_risk_lab, fram_10yr_risk_nonlab, mean_imt, total_plaques, any_plaques
metadata_ordered$age <- as.numeric(metadata_ordered$age)
metadata_ordered$BMI <- as.numeric(metadata_ordered$BMI)
metadata_ordered$days_on_art <- as.numeric(metadata_ordered$days_on_art)

### control for metadata with full n
covars_full_n <- c("Ethnicity", "age", "sex", "Race", "hiv_phenotype", "tmp_smx_active", "current_art_class_consolid2", "sample_cohort")
print(vegan::adonis2(as.formula(paste("unifrac.distance~metadata_ordered$", paste(covars_full_n, collapse = "+metadata_ordered$"), sep = "")), data=ASV_table, permutations=1000)) -> adon_full_n
```

```
## Permutation test for adonis under reduced model
## Terms added sequentially (first to last)
## Permutation: free
## Number of permutations: 1000
## 
## vegan::adonis2(formula = as.formula(paste("unifrac.distance~metadata_ordered$", paste(covars_full_n, collapse = "+metadata_ordered$"), sep = "")), data = ASV_table, permutations = 1000)
##                                               Df SumOfSqs      R2      F
## metadata_ordered$Ethnicity                     1    0.489 0.00292 1.3177
## metadata_ordered$age                           1    1.448 0.00865 3.9011
## metadata_ordered$sex                           1    0.682 0.00408 1.8372
## metadata_ordered$Race                          5    5.128 0.03064 2.7626
## metadata_ordered$hiv_phenotype                 1    1.064 0.00636 2.8659
## metadata_ordered$tmp_smx_active                1    1.568 0.00937 4.2230
## metadata_ordered$current_art_class_consolid2   3    1.486 0.00888 1.3346
## metadata_ordered$sample_cohort                 2    2.898 0.01732 3.9032
## Residual                                     411  152.570 0.91178       
## Total                                        426  167.333 1.00000       
##                                                Pr(>F)    
## metadata_ordered$Ethnicity                   0.026973 *  
## metadata_ordered$age                         0.000999 ***
## metadata_ordered$sex                         0.000999 ***
## metadata_ordered$Race                        0.000999 ***
## metadata_ordered$hiv_phenotype               0.000999 ***
## metadata_ordered$tmp_smx_active              0.000999 ***
## metadata_ordered$current_art_class_consolid2 0.001998 ** 
## metadata_ordered$sample_cohort               0.000999 ***
## Residual                                                 
## Total                                                    
## ---
## Signif. codes:  0 '***' 0.001 '**' 0.01 '*' 0.05 '.' 0.1 ' ' 1
```

```
###*** sample_cohort r2 = 0.01732 p = 0.000999, hiv_phenotype r2 = 0.00636 p = 0.000999

#                                               Df SumOfSqs      R2      F   Pr(>F)    
# metadata_ordered$Ethnicity                     1    0.489 0.00292 1.3177 0.023976 *  
# metadata_ordered$age                           1    1.448 0.00865 3.9011 0.000999 ***
# metadata_ordered$sex                           1    0.682 0.00408 1.8372 0.000999 ***
# metadata_ordered$Race                          5    5.128 0.03064 2.7626 0.000999 ***
# metadata_ordered$hiv_phenotype                 1    1.064 0.00636 2.8659 0.000999 ***
# metadata_ordered$tmp_smx_active                1    1.568 0.00937 4.2230 0.000999 ***
# metadata_ordered$current_art_class_consolid2   3    1.486 0.00888 1.3346 0.000999 ***
# metadata_ordered$sample_cohort                 2    2.898 0.01732 3.9032 0.000999 ***
# Residual                                     411  152.570 0.91178                    
# Total                                        426  167.333 1.00000                    


#--------------------------------------------------------------------------------------------------------------

### COMPARISON 1: Adonis: controlling for additional metadata - removing samples w/o values - BMI/comorbidities/days_on_art
#--------------------------------------------------------------------------------------------------------------

### remove samples with BMI/comorbidities/days_on_art is NA (n = 427 -> n = 405)
ps_gg_fp_f_prevalence_filter_2019_05_26_proportion_bcd <- ps_gg_fp_f_prevalence_filter_2019_05_26_proportion
metadata_ordered_bcd <- metadata_ordered[!is.na(metadata_ordered$hld_hx) & !is.na(metadata_ordered$days_on_art) & !is.na(metadata_ordered$BMI), , drop = FALSE]
sample_data(ps_gg_fp_f_prevalence_filter_2019_05_26_proportion_bcd) <- metadata_ordered_bcd
```

```
## Found more than one class "phylo" in cache; using the first, from namespace 'phyloseq'
## Also defined by 'tidytree'
```

```
## Found more than one class "phylo" in cache; using the first, from namespace 'phyloseq'
```

```
## Also defined by 'tidytree'
```

```
## Found more than one class "phylo" in cache; using the first, from namespace 'phyloseq'
```

```
## Also defined by 'tidytree'
```

```
## Found more than one class "phylo" in cache; using the first, from namespace 'phyloseq'
```

```
## Also defined by 'tidytree'
```

```
### subset ASV table and distance matrix
ASV_table_bcd <- as.data.frame(otu_table(ps_gg_fp_f_prevalence_filter_2019_05_26_proportion_bcd))
all.equal(row.names(ASV_table_bcd), row.names(metadata_ordered_bcd))
```

```
## [1] TRUE
```

```
unifrac.distance_bcd <- unname(usedist::dist_subset(unifrac.distance, sample_names(ps_gg_fp_f_prevalence_filter_2019_05_26_proportion_bcd))) ### unname fixes error introduced by Desctools see https://github.com/joey711/phyloseq/issues/1457
attributes(unifrac.distance_bcd)$Labels <- phyloseq::sample_names(ps_gg_fp_f_prevalence_filter_2019_05_26_proportion_bcd)

### run adonis
covars_bcd <- c("BMI", "hld_hx", "htn_hx", "cvd_hx", "dm2hx_dx", "hldhx_dx", "cvdhx_dx", "days_on_art", covars_full_n)
print(vegan::adonis2(as.formula(paste("unifrac.distance_bcd~metadata_ordered_bcd$", paste(covars_bcd, collapse = "+metadata_ordered_bcd$"), sep = "")), data = ASV_table_bcd, permutations=1000)) -> adon_full_n_bcd
```

```
## Permutation test for adonis under reduced model
## Terms added sequentially (first to last)
## Permutation: free
## Number of permutations: 1000
## 
## vegan::adonis2(formula = as.formula(paste("unifrac.distance_bcd~metadata_ordered_bcd$", paste(covars_bcd, collapse = "+metadata_ordered_bcd$"), sep = "")), data = ASV_table_bcd, permutations = 1000)
##                                                   Df SumOfSqs      R2      F
## metadata_ordered_bcd$BMI                           1    0.983 0.00621 2.6552
## metadata_ordered_bcd$hld_hx                        1    0.457 0.00289 1.2361
## metadata_ordered_bcd$htn_hx                        1    0.515 0.00326 1.3917
## metadata_ordered_bcd$cvd_hx                        1    0.622 0.00393 1.6796
## metadata_ordered_bcd$dm2hx_dx                      1    0.443 0.00280 1.1978
## metadata_ordered_bcd$hldhx_dx                      1    0.534 0.00338 1.4439
## metadata_ordered_bcd$cvdhx_dx                      1    0.684 0.00433 1.8494
## metadata_ordered_bcd$days_on_art                   1    0.928 0.00587 2.5080
## metadata_ordered_bcd$Ethnicity                     1    0.487 0.00308 1.3146
## metadata_ordered_bcd$age                           1    1.184 0.00749 3.1999
## metadata_ordered_bcd$sex                           1    0.798 0.00504 2.1563
## metadata_ordered_bcd$Race                          4    3.610 0.02282 2.4388
## metadata_ordered_bcd$hiv_phenotype                 1    0.731 0.00462 1.9738
## metadata_ordered_bcd$tmp_smx_active                1    1.008 0.00637 2.7248
## metadata_ordered_bcd$current_art_class_consolid2   3    1.554 0.00982 1.3996
## metadata_ordered_bcd$sample_cohort                 2    2.292 0.01449 3.0969
## Residual                                         382  141.381 0.89361       
## Total                                            404  158.214 1.00000       
##                                                    Pr(>F)    
## metadata_ordered_bcd$BMI                         0.000999 ***
## metadata_ordered_bcd$hld_hx                      0.068931 .  
## metadata_ordered_bcd$htn_hx                      0.013986 *  
## metadata_ordered_bcd$cvd_hx                      0.000999 ***
## metadata_ordered_bcd$dm2hx_dx                    0.058941 .  
## metadata_ordered_bcd$hldhx_dx                    0.005994 ** 
## metadata_ordered_bcd$cvdhx_dx                    0.000999 ***
## metadata_ordered_bcd$days_on_art                 0.000999 ***
## metadata_ordered_bcd$Ethnicity                   0.030969 *  
## metadata_ordered_bcd$age                         0.000999 ***
## metadata_ordered_bcd$sex                         0.000999 ***
## metadata_ordered_bcd$Race                        0.000999 ***
## metadata_ordered_bcd$hiv_phenotype               0.000999 ***
## metadata_ordered_bcd$tmp_smx_active              0.000999 ***
## metadata_ordered_bcd$current_art_class_consolid2 0.000999 ***
## metadata_ordered_bcd$sample_cohort               0.000999 ***
## Residual                                                     
## Total                                                        
## ---
## Signif. codes:  0 '***' 0.001 '**' 0.01 '*' 0.05 '.' 0.1 ' ' 1
```

```
###*** sample_cohort r2 = 0.01449 p = 0.000999, hiv_phenotype r2 = 0.00462 p = 0.000999


#                                                   Df SumOfSqs      R2      F   Pr(>F)    
# metadata_ordered_bcd$BMI                           1    0.983 0.00621 2.6552 0.000999 ***
# metadata_ordered_bcd$hld_hx                        1    0.457 0.00289 1.2361 0.057942 .  
# metadata_ordered_bcd$htn_hx                        1    0.515 0.00326 1.3917 0.008991 ** 
# metadata_ordered_bcd$cvd_hx                        1    0.622 0.00393 1.6796 0.000999 ***
# metadata_ordered_bcd$dm2hx_dx                      1    0.443 0.00280 1.1978 0.089910 .  
# metadata_ordered_bcd$hldhx_dx                      1    0.534 0.00338 1.4439 0.007992 ** 
# metadata_ordered_bcd$cvdhx_dx                      1    0.684 0.00433 1.8494 0.000999 ***
# metadata_ordered_bcd$days_on_art                   1    0.928 0.00587 2.5080 0.000999 ***
# metadata_ordered_bcd$Ethnicity                     1    0.487 0.00308 1.3146 0.024975 *  
# metadata_ordered_bcd$age                           1    1.184 0.00749 3.1999 0.000999 ***
# metadata_ordered_bcd$sex                           1    0.798 0.00504 2.1563 0.000999 ***
# metadata_ordered_bcd$Race                          4    3.610 0.02282 2.4388 0.000999 ***
# metadata_ordered_bcd$hiv_phenotype                 1    0.731 0.00462 1.9738 0.000999 ***
# metadata_ordered_bcd$tmp_smx_active                1    1.008 0.00637 2.7248 0.000999 ***
# metadata_ordered_bcd$current_art_class_consolid2   3    1.554 0.00982 1.3996 0.000999 ***
# metadata_ordered_bcd$sample_cohort                 2    2.292 0.01449 3.0969 0.000999 ***
# Residual                                         382  141.381 0.89361                    
# Total                                            404  158.214 1.00000   

#--------------------------------------------------------------------------------------------------------------


#--------------------------------------------------------------------------------------------------------------
#COMPARISON 2: NEG vs UNTREATED
#Transform count data in the phyloseq object
ps_gg_fp_f_prevalence_filter_2019_05_26_proportion <- transform_sample_counts(ps_gg_fp_f_prevalence_filter_2019_05_26, function(x)(x/sum(x)))
```

```
## Found more than one class "phylo" in cache; using the first, from namespace 'phyloseq'
## Also defined by 'tidytree'
```

```
## Found more than one class "phylo" in cache; using the first, from namespace 'phyloseq'
```

```
## Also defined by 'tidytree'
```

```
## Found more than one class "phylo" in cache; using the first, from namespace 'phyloseq'
```

```
## Also defined by 'tidytree'
```

```
## Found more than one class "phylo" in cache; using the first, from namespace 'phyloseq'
```

```
## Also defined by 'tidytree'
```

```
#Select samples of interest and update phyloseq object 
metadata <- phyloseq::sample_data(ps_gg_fp_f_prevalence_filter_2019_05_26)
metadata <- metadata[metadata$hiv_phenotype%in%c("1_hiv_negative","4_unsuppressed"),,drop=F]
metadata <- as.data.frame(as.matrix(metadata[metadata$sexual_orientation!="MSM",,drop=F]))
metadata <- metadata[metadata$sample_cohort%in%c("botswana", "boston"),,drop=F]
phyloseq::sample_data(ps_gg_fp_f_prevalence_filter_2019_05_26_proportion)<-metadata
```

```
## Found more than one class "phylo" in cache; using the first, from namespace 'phyloseq'
## Also defined by 'tidytree'
```

```
## Found more than one class "phylo" in cache; using the first, from namespace 'phyloseq'
```

```
## Also defined by 'tidytree'
```

```
## Found more than one class "phylo" in cache; using the first, from namespace 'phyloseq'
```

```
## Also defined by 'tidytree'
```

```
## Found more than one class "phylo" in cache; using the first, from namespace 'phyloseq'
```

```
## Also defined by 'tidytree'
```

```
#Run PCoA on the phyloseq object
ordination <- phyloseq::ordinate(ps_gg_fp_f_prevalence_filter_2019_05_26_proportion, "PCoA", "unifrac")
```

```
## Warning in matrix(tree$edge[order(tree$edge[, 1]), ][, 2], byrow = TRUE, : data
## length [8987] is not a sub-multiple or multiple of the number of rows [4494]
```

```
ordination$values[1:2,]
```

```
##   Eigenvalues Relative_eig Broken_stick  Cumul_eig Cumul_br_stick
## 1    5.257069   0.06368984   0.02756855 0.06368984     0.02756855
## 2    4.098253   0.04965069   0.02293892 0.11334053     0.05050747
```

```
metadata_ordered<-metadata[row.names(ordination$vectors),,drop=FALSE]

all.equal(row.names(metadata_ordered), row.names(ordination$vectors))
```

```
## [1] TRUE
```

```
metadata_ordered$Unifrac1 <- ordination$vectors[,1]
metadata_ordered$Unifrac2 <- ordination$vectors[,2]

comparison2 <- metadata_ordered
comparison2$Grouping <- as.factor(paste(comparison2$hiv_phenotype, comparison2$sample_cohort, sep="_"))

#Let's plot the data
us<-comparison2[comparison2$sample_cohort=="boston",,drop=F]
botswana<-comparison2[comparison2$sample_cohort=="botswana",,drop=F]

plot_us_comparison<-ggplot2::ggplot(data=us, aes(x=Unifrac1, y=Unifrac2))+geom_point(color="royalblue4", aes(alpha=hiv_phenotype), size=2, shape=16)+
  theme_bw()+stat_ellipse(color="royalblue4", aes(alpha=hiv_phenotype), size=1)+scale_alpha_manual(values=c(1,0.3))+ylim(c(-0.5, 0.5))+xlim(c(-0.5,0.5))+
  ggtitle("neg-naive us")+geom_point(data=us %>% group_by(hiv_phenotype) %>% summarise_at(vars(matches("UniFrac")), mean),size=5, color="royalblue4", aes(alpha=hiv_phenotype))
plot_botswana_comparison<-ggplot(data=botswana, aes(x=Unifrac1, y=Unifrac2))+geom_point(color="darkorange", aes(alpha=hiv_phenotype), size=2, shape=16)+
  theme_bw()+stat_ellipse(color="darkorange", aes(alpha=hiv_phenotype), size=1)+scale_alpha_manual(values=c(1,0.3))+ylim(c(-0.5, 0.5))+xlim(c(-0.5,0.5))+
  ggtitle("neg-naive botswana")+geom_point(data=botswana %>% group_by(hiv_phenotype) %>% summarise_at(vars(matches("UniFrac")), mean),size=5, color="darkorange", aes(alpha=hiv_phenotype))

ggsave("Figure2A_2v3.pdf", grid.arrange(plot_us_comparison,plot_botswana_comparison, nrow=3, ncol=3), width=15, height=10)
```

```
#Adonis (n = 217)
ASV_table <- as.data.frame(phyloseq::otu_table(ps_gg_fp_f_prevalence_filter_2019_05_26_proportion))
all.equal(row.names(ASV_table), row.names(metadata_ordered))
```

```
## [1] TRUE
```

```
unifrac.distance <- unname(phyloseq::UniFrac(ps_gg_fp_f_prevalence_filter_2019_05_26_proportion, weighted = FALSE)) ### unname fixes error introduced by Desctools see https://github.com/joey711/phyloseq/issues/1457
```

```
## Warning in matrix(tree$edge[order(tree$edge[, 1]), ][, 2], byrow = TRUE, : data
## length [8987] is not a sub-multiple or multiple of the number of rows [4494]
```

```
attributes(unifrac.distance)$Labels <- phyloseq::sample_names(ps_gg_fp_f_prevalence_filter_2019_05_26_proportion)
print(vegan::adonis2(unifrac.distance ~ metadata_ordered$hiv_phenotype, data = ASV_table, permutations = 1000)) -> adon_hiv_phenotype
```

```
## Permutation test for adonis under reduced model
## Terms added sequentially (first to last)
## Permutation: free
## Number of permutations: 1000
## 
## vegan::adonis2(formula = unifrac.distance ~ metadata_ordered$hiv_phenotype, data = ASV_table, permutations = 1000)
##                                 Df SumOfSqs      R2      F   Pr(>F)   
## metadata_ordered$hiv_phenotype   1    0.550 0.00666 1.4412 0.008991 **
## Residual                       215   81.992 0.99334                   
## Total                          216   82.542 1.00000                   
## ---
## Signif. codes:  0 '***' 0.001 '**' 0.01 '*' 0.05 '.' 0.1 ' ' 1
```

```
###*** r2 = 0.00666 p = 0.005994
print(vegan::adonis2(unifrac.distance ~ metadata_ordered$sample_cohort, data = ASV_table, permutations = 1000)) -> adon_cohort
```

```
## Permutation test for adonis under reduced model
## Terms added sequentially (first to last)
## Permutation: free
## Number of permutations: 1000
## 
## vegan::adonis2(formula = unifrac.distance ~ metadata_ordered$sample_cohort, data = ASV_table, permutations = 1000)
##                                 Df SumOfSqs      R2     F   Pr(>F)    
## metadata_ordered$sample_cohort   1    2.805 0.03398 7.563 0.000999 ***
## Residual                       215   79.737 0.96602                   
## Total                          216   82.542 1.00000                   
## ---
## Signif. codes:  0 '***' 0.001 '**' 0.01 '*' 0.05 '.' 0.1 ' ' 1
```

```
###*** r2 = 0.03398 p = 0.000999
print(vegan::adonis2(unifrac.distance ~ metadata_ordered$hiv_phenotype + metadata_ordered$sample_cohort, data = ASV_table, permutations = 1000)) -> adon_cohort_phenotype
```

```
## Permutation test for adonis under reduced model
## Terms added sequentially (first to last)
## Permutation: free
## Number of permutations: 1000
## 
## vegan::adonis2(formula = unifrac.distance ~ metadata_ordered$hiv_phenotype + metadata_ordered$sample_cohort, data = ASV_table, permutations = 1000)
##                                 Df SumOfSqs      R2      F   Pr(>F)    
## metadata_ordered$hiv_phenotype   1    0.550 0.00666 1.4840 0.007992 ** 
## metadata_ordered$sample_cohort   1    2.736 0.03315 7.3888 0.000999 ***
## Residual                       214   79.256 0.96019                    
## Total                          216   82.542 1.00000                    
## ---
## Signif. codes:  0 '***' 0.001 '**' 0.01 '*' 0.05 '.' 0.1 ' ' 1
```

```
###*** sample_cohort r2 = 0.03315 p = 0.000999, hiv_phenotype r2 = 0.00666 p = 0.008991

#--------------------------------------------------------------------------------------------------------------


### COMPARISON 2: Adonis: controlling for additional metadata
#--------------------------------------------------------------------------------------------------------------
### Extra metadata to add: (in addition to sample_cohort, hiv_phenotype)
### that have full n: Race, Ethnicity, age, sex
metadata_ordered$age <- as.numeric(metadata_ordered$age)
metadata_ordered$BMI <- as.numeric(metadata_ordered$BMI)
### metadata not collected in Boston:
metadata_ordered$monthly_income <- as.numeric(metadata_ordered$monthly_income)
metadata_ordered$smoking_years <- as.numeric(metadata_ordered$smoking_years)
metadata_ordered$fram_10yr_risk_lab <- as.numeric(metadata_ordered$fram_10yr_risk_lab)
metadata_ordered$fram_10yr_risk_nonlab <- as.numeric(metadata_ordered$fram_10yr_risk_nonlab)
metadata_ordered$mean_imt <- as.numeric(metadata_ordered$mean_imt)
metadata_ordered$total_plaques <- as.numeric(metadata_ordered$total_plaques)
metadata_ordered$any_plaques <- as.numeric(metadata_ordered$any_plaques)

### control for metadata with full n
covars_full_n_untreat <- covars_full_n[!covars_full_n %in% c("tmp_smx_active", "current_art_class_consolid2")]
print(vegan::adonis2(as.formula(paste("unifrac.distance~metadata_ordered$", paste(covars_full_n_untreat, collapse = "+metadata_ordered$"), sep = "")), data=ASV_table, permutations=1000, by = "margin")) -> adon_full_n_marg
```

```
## Permutation test for adonis under reduced model
## Marginal effects of terms
## Permutation: free
## Number of permutations: 1000
## 
## vegan::adonis2(formula = as.formula(paste("unifrac.distance~metadata_ordered$", paste(covars_full_n_untreat, collapse = "+metadata_ordered$"), sep = "")), data = ASV_table, permutations = 1000, by = "margin")
##                                 Df SumOfSqs      R2      F   Pr(>F)    
## metadata_ordered$Ethnicity       1    0.342 0.00414 0.9289 0.668332    
## metadata_ordered$age             1    0.604 0.00732 1.6423 0.003996 ** 
## metadata_ordered$sex             1    0.442 0.00536 1.2025 0.073926 .  
## metadata_ordered$Race            4    1.708 0.02069 1.1615 0.018981 *  
## metadata_ordered$hiv_phenotype   1    0.471 0.00571 1.2816 0.041958 *  
## metadata_ordered$sample_cohort   1    0.848 0.01027 2.3059 0.000999 ***
## Residual                       207   76.105 0.92202                    
## Total                          216   82.542 1.00000                    
## ---
## Signif. codes:  0 '***' 0.001 '**' 0.01 '*' 0.05 '.' 0.1 ' ' 1
```

```
###*** sample_cohort r2 = 0.01027 p = 0.000999, hiv_phenotype r2 = 0.00571 p = 0.040959

#                                 Df SumOfSqs      R2      F   Pr(>F)    
# metadata_ordered$Ethnicity       1    0.342 0.00414 0.9289 0.683317    
# metadata_ordered$age             1    0.604 0.00732 1.6423 0.002997 ** 
# metadata_ordered$sex             1    0.442 0.00536 1.2025 0.095904 .  
# metadata_ordered$Race            4    1.708 0.02069 1.1615 0.021978 *  
# metadata_ordered$hiv_phenotype   1    0.471 0.00571 1.2816 0.040959 *  
# metadata_ordered$sample_cohort   1    0.848 0.01027 2.3059 0.000999 ***
# Residual                       207   76.105 0.92202                    
# Total                          216   82.542 1.00000                   

#--------------------------------------------------------------------------------------------------------------

### COMPARISON 2: Adonis: controlling for additional metadata - removing samples w/o values - BMI/comorbidities
#--------------------------------------------------------------------------------------------------------------
### remove samples with BMI/comorbidities is NA (n=217 -> n=201)
ps_gg_fp_f_prevalence_filter_2019_05_26_proportion_bc <- ps_gg_fp_f_prevalence_filter_2019_05_26_proportion
metadata_ordered_bc <- metadata_ordered[!is.na(metadata_ordered$hld_hx) & !is.na(metadata_ordered$BMI), , drop = FALSE]
sample_data(ps_gg_fp_f_prevalence_filter_2019_05_26_proportion_bc) <- metadata_ordered_bc
```

```
## Found more than one class "phylo" in cache; using the first, from namespace 'phyloseq'
## Also defined by 'tidytree'
```

```
## Found more than one class "phylo" in cache; using the first, from namespace 'phyloseq'
```

```
## Also defined by 'tidytree'
```

```
## Found more than one class "phylo" in cache; using the first, from namespace 'phyloseq'
```

```
## Also defined by 'tidytree'
```

```
## Found more than one class "phylo" in cache; using the first, from namespace 'phyloseq'
```

```
## Also defined by 'tidytree'
```

```
### subset ASV table and distance matrix
ASV_table_bc<-as.data.frame(otu_table(ps_gg_fp_f_prevalence_filter_2019_05_26_proportion_bc))
all.equal(row.names(ASV_table_bc), row.names(metadata_ordered_bc))
```

```
## [1] TRUE
```

```
unifrac.distance_bc <- unname(usedist::dist_subset(unifrac.distance, sample_names(ps_gg_fp_f_prevalence_filter_2019_05_26_proportion_bc))) ### unname fixes error introduced by Desctools see https://github.com/joey711/phyloseq/issues/1457
attributes(unifrac.distance_bc)$Labels <- phyloseq::sample_names(ps_gg_fp_f_prevalence_filter_2019_05_26_proportion_bc)

### run adonis
covars_bc <- c("BMI", "hld_hx", "htn_hx", "cvd_hx", "dm2hx_dx", "hldhx_dx", "cvdhx_dx", covars_full_n_untreat)
print(vegan::adonis2(as.formula(paste("unifrac.distance_bc~metadata_ordered_bc$", paste(covars_bc, collapse = "+metadata_ordered_bc$"), sep = "")), data=ASV_table_bc, permutations=1000)) -> adon_full_n_bc
```

```
## Permutation test for adonis under reduced model
## Terms added sequentially (first to last)
## Permutation: free
## Number of permutations: 1000
## 
## vegan::adonis2(formula = as.formula(paste("unifrac.distance_bc~metadata_ordered_bc$", paste(covars_bc, collapse = "+metadata_ordered_bc$"), sep = "")), data = ASV_table_bc, permutations = 1000)
##                                    Df SumOfSqs      R2      F   Pr(>F)    
## metadata_ordered_bc$BMI             1    0.542 0.00713 1.4843 0.010989 *  
## metadata_ordered_bc$hld_hx          1    0.432 0.00568 1.1830 0.109890    
## metadata_ordered_bc$htn_hx          1    0.360 0.00474 0.9874 0.456543    
## metadata_ordered_bc$cvd_hx          1    0.502 0.00660 1.3755 0.010989 *  
## metadata_ordered_bc$dm2hx_dx        1    0.397 0.00522 1.0880 0.204795    
## metadata_ordered_bc$hldhx_dx        1    0.597 0.00785 1.6350 0.001998 ** 
## metadata_ordered_bc$cvdhx_dx        1    0.511 0.00672 1.4002 0.010989 *  
## metadata_ordered_bc$Ethnicity       1    0.413 0.00543 1.1309 0.164835    
## metadata_ordered_bc$age             1    0.879 0.01156 2.4075 0.000999 ***
## metadata_ordered_bc$sex             1    0.451 0.00593 1.2354 0.066933 .  
## metadata_ordered_bc$Race            4    2.699 0.03550 1.8488 0.000999 ***
## metadata_ordered_bc$hiv_phenotype   1    0.423 0.00557 1.1599 0.104895    
## metadata_ordered_bc$sample_cohort   1    0.660 0.00869 1.8091 0.000999 ***
## Residual                          184   67.155 0.88338                    
## Total                             200   76.020 1.00000                    
## ---
## Signif. codes:  0 '***' 0.001 '**' 0.01 '*' 0.05 '.' 0.1 ' ' 1
```

```
###*** sample_cohort r2 = 0.00854 p = 0.000999, hiv_phenotype r2 = 0.00557 p = 0.135864

#                                    Df SumOfSqs      R2      F   Pr(>F)    
# metadata_ordered_bc$BMI             1    0.542 0.00713 1.4843 0.006993 ** 
# metadata_ordered_bc$hld_hx          1    0.432 0.00568 1.1830 0.118881    
# metadata_ordered_bc$htn_hx          1    0.360 0.00474 0.9874 0.481518    
# metadata_ordered_bc$cvd_hx          1    0.502 0.00660 1.3755 0.015984 *  
# metadata_ordered_bc$dm2hx_dx        1    0.397 0.00522 1.0880 0.210789    
# metadata_ordered_bc$hldhx_dx        1    0.597 0.00785 1.6350 0.000999 ***
# metadata_ordered_bc$cvdhx_dx        1    0.511 0.00672 1.4002 0.014985 *  
# metadata_ordered_bc$Ethnicity       1    0.413 0.00543 1.1309 0.135864    
# metadata_ordered_bc$age             1    0.879 0.01156 2.4075 0.000999 ***
# metadata_ordered_bc$sex             1    0.451 0.00593 1.2354 0.061938 .  
# metadata_ordered_bc$Race            4    2.699 0.03550 1.8488 0.000999 ***
# metadata_ordered_bc$hiv_phenotype   1    0.423 0.00557 1.1599 0.119880    
# metadata_ordered_bc$sample_cohort   1    0.660 0.00869 1.8091 0.001998 ** 
# Residual                          184   67.155 0.88338                    
# Total                             200   76.020 1.00000                   

#--------------------------------------------------------------------------------------------------------------
```

#Figure 2BCDE

```
#FIGURE 2B, C, D: ANCOM NEG-ART & FIGURE 2E
#--------------------------------------------------------------------------------------------------------------
#Transform count data in the phyloseq object
ps_gg_fp_f_prevalence_filter_2019_05_26_proportion<-transform_sample_counts(ps_gg_fp_f_prevalence_filter_2019_05_26, function(x)(x/sum(x)))
```

```
## Found more than one class "phylo" in cache; using the first, from namespace 'phyloseq'
```

```
## Also defined by 'tidytree'
```

```
## Found more than one class "phylo" in cache; using the first, from namespace 'phyloseq'
```

```
## Also defined by 'tidytree'
```

```
## Found more than one class "phylo" in cache; using the first, from namespace 'phyloseq'
```

```
## Also defined by 'tidytree'
```

```
## Found more than one class "phylo" in cache; using the first, from namespace 'phyloseq'
```

```
## Also defined by 'tidytree'
```

```
###RUN ANCOM###

#US 
dataset<-ps_gg_fp_f_prevalence_filter_2019_05_26
metadata<-as.data.frame(sample_data(ps_gg_fp_f_prevalence_filter_2019_05_26))
metadata<-metadata[metadata$hiv_phenotype %in% c("1_hiv_negative", "2_suppressed"), , drop=F]
metadata<-as.data.frame(as.matrix(metadata[metadata$sexual_orientation != "MSM" | is.na(metadata$sexual_orientation), , drop=F]))
metadata_boston<-metadata[metadata$sample_cohort == "boston", , drop=F]
sample_data(dataset)<-metadata_boston
```

```
## Found more than one class "phylo" in cache; using the first, from namespace 'phyloseq'
## Also defined by 'tidytree'
```

```
## Found more than one class "phylo" in cache; using the first, from namespace 'phyloseq'
```

```
## Also defined by 'tidytree'
```

```
## Found more than one class "phylo" in cache; using the first, from namespace 'phyloseq'
```

```
## Also defined by 'tidytree'
```

```
## Found more than one class "phylo" in cache; using the first, from namespace 'phyloseq'
```

```
## Also defined by 'tidytree'
```

```
ps.taxa.sub <- phyloseq::prune_taxa(taxa_sums(dataset) > 0, dataset)
```

```
## Found more than one class "phylo" in cache; using the first, from namespace 'phyloseq'
## Also defined by 'tidytree'
```

```
out <- ANCOMBC::ancombc(data = ps.taxa.sub, formula = "hiv_phenotype", tax_level = NULL,
              p_adj_method = "BH", prv_cut = 0.05, lib_cut = 1000, 
              group = "hiv_phenotype", struc_zero = TRUE, neg_lb = FALSE, tol = 1e-5, 
              max_iter = 100, conserve = FALSE, alpha = 0.05, global = FALSE, n_cl = 6)
```

```
## 'ancombc' is deprecated 
## Use 'ancombc2' instead
```

```
## Found more than one class "phylo" in cache; using the first, from namespace 'phyloseq'
```

```
## Also defined by 'tidytree'
```

```
## Found more than one class "phylo" in cache; using the first, from namespace 'phyloseq'
```

```
## Also defined by 'tidytree'
```

```
## `tax_level` is not speficified 
## No agglomeration will be performed
## Otherwise, please speficy `tax_level` by one of the following: 
## Kingdom, Phylum, Class, Order, Family, Genus, Species
```

```
## Found more than one class "phylo" in cache; using the first, from namespace 'phyloseq'
```

```
## Also defined by 'tidytree'
```

```
## Found more than one class "phylo" in cache; using the first, from namespace 'phyloseq'
```

```
## Also defined by 'tidytree'
```

```
## Found more than one class "phylo" in cache; using the first, from namespace 'phyloseq'
```

```
## Also defined by 'tidytree'
```

```
## Found more than one class "phylo" in cache; using the first, from namespace 'phyloseq'
```

```
## Also defined by 'tidytree'
```

```
## Warning: The group variable has < 3 categories 
## The multi-group comparisons (global/pairwise/dunnet/trend) will be deactivated
```

```
## Found more than one class "phylo" in cache; using the first, from namespace 'phyloseq'
## Also defined by 'tidytree'
```

```
## Found more than one class "phylo" in cache; using the first, from namespace 'phyloseq'
```

```
## Also defined by 'tidytree'
```

```
res <- out$res
res_rn <- purrr::imap(res, function(x, y) dplyr::rename(x, !!y := hiv_phenotype2_suppressed))
res_df <- purrr::reduce(res_rn, dplyr::left_join, by = "taxon")
res_df <- dplyr::select(res_df, !starts_with("(Int"))
res_df_taxa <- dplyr::left_join(res_df, tibble::rownames_to_column(as.data.frame(phyloseq::tax_table(ps.taxa.sub))), by = c("taxon" = "rowname"))
res_df_taxa[["index_num"]] <- 1:nrow(res_df_taxa)
res_df_taxa[["cohort"]] <- "boston"
res_df_taxa[["method"]] <- "ancom"
res_df_taxa <- tidyr::unite(res_df_taxa, col =  "Genus_Species", Genus, Species, index_num, remove = FALSE)
alpha = 0.05
taxa_sig <- dplyr::filter(res_df_taxa, q_val < 0.05)
```

```
## Warning: Using one column matrices in `filter()` was deprecated in dplyr 1.1.0.
## ℹ Please use one dimensional logical vectors instead.
## ℹ The deprecated feature was likely used in the dplyr package.
##   Please report the issue at <]8;;https://github.com/tidyverse/dplyr/issueshttps://github.com/tidyverse/dplyr/issues]8;;>.
```

```
taxa_sig$Genus_Species <- forcats::fct_reorder(taxa_sig$Genus_Species, taxa_sig$lfc, min)
taxa_sig$taxon_short <- stringr::str_sub(taxa_sig$taxon, 1, 4)
ps.taxa.rel.sig <- phyloseq::prune_taxa(taxa_sig[["taxon"]], ps_gg_fp_f_prevalence_filter_2019_05_26_proportion)
```

```
## Found more than one class "phylo" in cache; using the first, from namespace 'phyloseq'
## Also defined by 'tidytree'
```

```
# Only keep filtered samples 
ps.taxa.rel.sig <- phyloseq::prune_samples(rownames(phyloseq::otu_table(ps.taxa.sub)), ps.taxa.rel.sig)
sigtab_dataset_us <- taxa_sig
write.csv(sigtab_dataset_us, "ANCOM_US_NEG_ART_Filtered.csv")


#BOTSWANA
dataset<-ps_gg_fp_f_prevalence_filter_2019_05_26
metadata<-as.data.frame(sample_data(ps_gg_fp_f_prevalence_filter_2019_05_26))
metadata<-metadata[metadata$hiv_phenotype %in% c("1_hiv_negative", "2_suppressed"), , drop=F]
metadata<-as.data.frame(as.matrix(metadata[metadata$sexual_orientation != "MSM" | is.na(metadata$sexual_orientation), , drop=F]))
metadata_botswana<-metadata[metadata$sample_cohort == "botswana", , drop=F]
sample_data(dataset)<-metadata_botswana
```

```
## Found more than one class "phylo" in cache; using the first, from namespace 'phyloseq'
## Also defined by 'tidytree'
```

```
## Found more than one class "phylo" in cache; using the first, from namespace 'phyloseq'
```

```
## Also defined by 'tidytree'
```

```
## Found more than one class "phylo" in cache; using the first, from namespace 'phyloseq'
```

```
## Also defined by 'tidytree'
```

```
## Found more than one class "phylo" in cache; using the first, from namespace 'phyloseq'
```

```
## Also defined by 'tidytree'
```

```
ps.taxa.sub <- phyloseq::prune_taxa(taxa_sums(dataset) > 0, dataset)
```

```
## Found more than one class "phylo" in cache; using the first, from namespace 'phyloseq'
## Also defined by 'tidytree'
```

```
out <- ANCOMBC::ancombc(data = ps.taxa.sub, formula = "hiv_phenotype", tax_level = NULL,
              p_adj_method = "BH", prv_cut = 0.05, lib_cut = 1000, 
              group = "hiv_phenotype", struc_zero = TRUE, neg_lb = FALSE, tol = 1e-5, 
              max_iter = 100, conserve = FALSE, alpha = 0.05, global = FALSE, n_cl = 6)
```

```
## 'ancombc' is deprecated 
## Use 'ancombc2' instead
```

```
## Found more than one class "phylo" in cache; using the first, from namespace 'phyloseq'
```

```
## Also defined by 'tidytree'
```

```
## Found more than one class "phylo" in cache; using the first, from namespace 'phyloseq'
```

```
## Also defined by 'tidytree'
```

```
## `tax_level` is not speficified 
## No agglomeration will be performed
## Otherwise, please speficy `tax_level` by one of the following: 
## Kingdom, Phylum, Class, Order, Family, Genus, Species
```

```
## Found more than one class "phylo" in cache; using the first, from namespace 'phyloseq'
```

```
## Also defined by 'tidytree'
```

```
## Found more than one class "phylo" in cache; using the first, from namespace 'phyloseq'
```

```
## Also defined by 'tidytree'
```

```
## Found more than one class "phylo" in cache; using the first, from namespace 'phyloseq'
```

```
## Also defined by 'tidytree'
```

```
## Found more than one class "phylo" in cache; using the first, from namespace 'phyloseq'
```

```
## Also defined by 'tidytree'
```

```
## Warning: The group variable has < 3 categories 
## The multi-group comparisons (global/pairwise/dunnet/trend) will be deactivated
```

```
## Found more than one class "phylo" in cache; using the first, from namespace 'phyloseq'
## Also defined by 'tidytree'
```

```
## Found more than one class "phylo" in cache; using the first, from namespace 'phyloseq'
```

```
## Also defined by 'tidytree'
```

```
res <- out$res
res_rn <- purrr::imap(res, function(x, y) dplyr::rename(x, !!y := hiv_phenotype2_suppressed))
res_df <- purrr::reduce(res_rn, dplyr::left_join, by = "taxon")
res_df <- dplyr::select(res_df, !starts_with("(Int"))
res_df_taxa <- dplyr::left_join(res_df, tibble::rownames_to_column(as.data.frame(phyloseq::tax_table(ps.taxa.sub))), by = c("taxon" = "rowname"))
res_df_taxa[["index_num"]] <- 1:nrow(res_df_taxa)
res_df_taxa[["cohort"]] <- "botswana"
res_df_taxa[["method"]] <- "ancom"
res_df_taxa <- tidyr::unite(res_df_taxa, col =  "Genus_Species", Genus, Species, index_num, remove = FALSE)
alpha = 0.05
taxa_sig <- dplyr::filter(res_df_taxa, q_val < 0.05)
taxa_sig$Genus_Species <- forcats::fct_reorder(taxa_sig$Genus_Species, taxa_sig$lfc, min)
taxa_sig$taxon_short <- stringr::str_sub(taxa_sig$taxon, 1, 4)
ps.taxa.rel.sig <- phyloseq::prune_taxa(taxa_sig[["taxon"]], ps_gg_fp_f_prevalence_filter_2019_05_26_proportion)
```

```
## Found more than one class "phylo" in cache; using the first, from namespace 'phyloseq'
## Also defined by 'tidytree'
```

```
# Only keep filtered samples 
ps.taxa.rel.sig <- phyloseq::prune_samples(rownames(phyloseq::otu_table(ps.taxa.sub)), ps.taxa.rel.sig)
sigtab_dataset_botswana <- taxa_sig
write.csv(sigtab_dataset_botswana, "ANCOM_BOTS_NEG_ART_Filtered.csv")


#UGANDA
dataset<-ps_gg_fp_f_prevalence_filter_2019_05_26
metadata<-as.data.frame(sample_data(ps_gg_fp_f_prevalence_filter_2019_05_26))
metadata<-metadata[metadata$hiv_phenotype %in% c("1_hiv_negative", "2_suppressed"), , drop=F]
metadata<-as.data.frame(as.matrix(metadata[metadata$sexual_orientation != "MSM" | is.na(metadata$sexual_orientation), , drop=F]))
metadata_uganda<-metadata[metadata$sample_cohort == "uganda_2", , drop=F]
sample_data(dataset)<-metadata_uganda
```

```
## Found more than one class "phylo" in cache; using the first, from namespace 'phyloseq'
## Also defined by 'tidytree'
```

```
## Found more than one class "phylo" in cache; using the first, from namespace 'phyloseq'
```

```
## Also defined by 'tidytree'
```

```
## Found more than one class "phylo" in cache; using the first, from namespace 'phyloseq'
```

```
## Also defined by 'tidytree'
```

```
## Found more than one class "phylo" in cache; using the first, from namespace 'phyloseq'
```

```
## Also defined by 'tidytree'
```

```
ps.taxa.sub <- phyloseq::prune_taxa(taxa_sums(dataset) > 0, dataset)
```

```
## Found more than one class "phylo" in cache; using the first, from namespace 'phyloseq'
## Also defined by 'tidytree'
```

```
out <- ANCOMBC::ancombc(data = ps.taxa.sub, formula = "hiv_phenotype", tax_level = NULL,
              p_adj_method = "BH", prv_cut = 0.05, lib_cut = 1000, 
              group = "hiv_phenotype", struc_zero = TRUE, neg_lb = FALSE, tol = 1e-5, 
              max_iter = 100, conserve = FALSE, alpha = 0.05, global = FALSE, n_cl = 6)
```

```
## 'ancombc' is deprecated 
## Use 'ancombc2' instead
```

```
## Found more than one class "phylo" in cache; using the first, from namespace 'phyloseq'
```

```
## Also defined by 'tidytree'
```

```
## Found more than one class "phylo" in cache; using the first, from namespace 'phyloseq'
```

```
## Also defined by 'tidytree'
```

```
## `tax_level` is not speficified 
## No agglomeration will be performed
## Otherwise, please speficy `tax_level` by one of the following: 
## Kingdom, Phylum, Class, Order, Family, Genus, Species
```

```
## Found more than one class "phylo" in cache; using the first, from namespace 'phyloseq'
```

```
## Also defined by 'tidytree'
```

```
## Found more than one class "phylo" in cache; using the first, from namespace 'phyloseq'
```

```
## Also defined by 'tidytree'
```

```
## Found more than one class "phylo" in cache; using the first, from namespace 'phyloseq'
```

```
## Also defined by 'tidytree'
```

```
## Found more than one class "phylo" in cache; using the first, from namespace 'phyloseq'
```

```
## Also defined by 'tidytree'
```

```
## Warning: The group variable has < 3 categories 
## The multi-group comparisons (global/pairwise/dunnet/trend) will be deactivated
```

```
## Found more than one class "phylo" in cache; using the first, from namespace 'phyloseq'
## Also defined by 'tidytree'
```

```
## Found more than one class "phylo" in cache; using the first, from namespace 'phyloseq'
```

```
## Also defined by 'tidytree'
```

```
res <- out$res
res_rn <- purrr::imap(res, function(x, y) dplyr::rename(x, !!y := hiv_phenotype2_suppressed))
res_df <- purrr::reduce(res_rn, dplyr::left_join, by = "taxon")
res_df <- dplyr::select(res_df, !starts_with("(Int"))
res_df_taxa <- dplyr::left_join(res_df, tibble::rownames_to_column(as.data.frame(phyloseq::tax_table(ps.taxa.sub))), by = c("taxon" = "rowname"))
res_df_taxa[["index_num"]] <- 1:nrow(res_df_taxa)
res_df_taxa[["cohort"]] <- "uganda_2"
res_df_taxa[["method"]] <- "ancom"
res_df_taxa <- tidyr::unite(res_df_taxa, col =  "Genus_Species", Genus, Species, index_num, remove = FALSE)
alpha = 0.05
taxa_sig <- dplyr::filter(res_df_taxa, q_val < 0.05)
taxa_sig$Genus_Species <- forcats::fct_reorder(taxa_sig$Genus_Species, taxa_sig$lfc, min)
taxa_sig$taxon_short <- stringr::str_sub(taxa_sig$taxon, 1, 4)
ps.taxa.rel.sig <- phyloseq::prune_taxa(taxa_sig[["taxon"]], ps_gg_fp_f_prevalence_filter_2019_05_26_proportion)
```

```
## Found more than one class "phylo" in cache; using the first, from namespace 'phyloseq'
## Also defined by 'tidytree'
```

```
# Only keep filtered samples 
ps.taxa.rel.sig <- phyloseq::prune_samples(rownames(phyloseq::otu_table(ps.taxa.sub)), ps.taxa.rel.sig)
sigtab_dataset_uganda <- taxa_sig
write.csv(sigtab_dataset_uganda, "ANCOM_UGANDA_NEG_ART_Filtered.csv")


# Save plots of individual ANCOM fold-change
ggsave("Figure2BCD_NEG_ART_ANCOM_US_v7.pdf", ggplot(data = sigtab_dataset_us, aes(x = Genus_Species, y = lfc)) + theme_bw() + coord_flip() + geom_bar(stat="identity", aes(fill = Genus)) + ggtitle("NEG-ART US") + theme(legend.position = "bottom"), width = 10, height = 10, units = "in", dpi = 300)

ggsave("Figure2BCD_NEG_ART_ANCOM_BOTS_v7.pdf", ggplot(data = sigtab_dataset_botswana, aes(x = Genus_Species, y = lfc)) + theme_bw() + coord_flip() + geom_bar(stat="identity", aes(fill = Genus)) + ggtitle("NEG-ART BOTSWANA") + theme(legend.position = "bottom"), width = 10, height = 10, units = "in", dpi = 300)

ggsave("Figure2BCD_NEG_ART_ANCOM_UGANDA_v7.pdf", ggplot(data = sigtab_dataset_uganda, aes(x = Genus_Species, y = lfc)) + theme_bw() + coord_flip() + geom_bar(stat="identity", aes(fill = Genus)) + ggtitle("NEG-ART UGANDA") + theme(legend.position = "bottom"), width = 10, height = 10, units = "in", dpi = 300)

#Merge results from all three sample cohorts:
sigtab_dataset_art<-rbind(sigtab_dataset_us, sigtab_dataset_botswana, sigtab_dataset_uganda)
sigtab_dataset_art[["index_num_all"]] <- 1:nrow(sigtab_dataset_art)
sigtab_dataset_art <- tidyr::unite(sigtab_dataset_art, col =  "Genus_Species_all", Genus, Species, index_num_all, sep = "_", remove = FALSE)
sigtab_dataset_art <- tidyr::unite(sigtab_dataset_art, col =  "Genus_Species_all_for_color", Genus, Species, sep = " ", remove = FALSE)

# Remove extra brackets around taxonomic name for labels
sigtab_dataset_art$Genus_label <- stringr::str_replace(sigtab_dataset_art$Genus, "^\\[([^\\]]+)\\]", "\\1")
sigtab_dataset_art$Species_label <- stringr::str_replace(sigtab_dataset_art$Species, "^\\[([^\\]]+)\\]", "\\1")
sigtab_dataset_art %>% dplyr::mutate(Genus_species_label =
                dplyr::case_when(is.na(stringr::str_extract(Genus_label, "\\[")) & is.na(stringr::str_extract(Species_label, "\\[")) ~ paste(Genus_label,Species_label),
                                 TRUE ~ paste(Genus_label,"sp"))) -> sigtab_dataset_art
sigtab_dataset_art$Genus_species_label <- stringr::str_replace(sigtab_dataset_art$Genus_species_label, "\\[([^\\]]+)\\]", "")
sigtab_dataset_art$Genus_species_label <- stringr::str_replace(sigtab_dataset_art$Genus_species_label, "\\ +", " ")
sigtab_dataset_art <- dplyr::arrange(sigtab_dataset_art, cohort, lfc)
sigtab_dataset_art$Genus_Species_all<-factor(sigtab_dataset_art$Genus_Species_all, levels = c(sigtab_dataset_art$Genus_Species_all))

# Make lookup table for Genus_species_label
Genus_species_label_lookup <- dplyr::distinct(sigtab_dataset_art, Genus_Species_all, .keep_all = TRUE)$Genus_species_label
names(Genus_species_label_lookup) <- dplyr::distinct(sigtab_dataset_art, Genus_Species_all, .keep_all = TRUE)$Genus_Species_all

#Load color dictionary and construct figure:
dictionary_unique <- read.csv("COLOR_DICTIONARY3.csv", sep=";")
sigtab_dataset_art$Genus_species_label <- factor(sigtab_dataset_art$Genus_species_label, levels = unique(sigtab_dataset_art$Genus_species_label))
dictionary_plot <- dictionary_unique[dictionary_unique$Genus_species_label%in%sigtab_dataset_art$Genus_species_label,,drop=F]
rownames(dictionary_plot)<-dictionary_plot$Genus_species_label
dictionary_plot<-dictionary_plot[as.character(unique(sigtab_dataset_art$Genus_species_label)),,drop=F]
sigtab_dataset_art$cohort <- fct_relevel(as.factor(sigtab_dataset_art$cohort), "boston", "botswana", "uganda_2")
header_namer <- as_labeller(c(`boston` = "U.S.\nHIV-uninfected     HIV+ ART-treated", `botswana` = "Botswana\nHIV-uninfected     HIV+ ART-treated", `uganda_2` = "Uganda\nHIV-uninfected     HIV+ ART-treated"))


ggsave("Figure2BCD_NEG_ART_ANCOM_ALL_v9.pdf", 
       ggplot(data = sigtab_dataset_art, aes(x = Genus_Species_all, y = lfc)) +
         geom_bar(stat = "identity", aes(fill = Genus_species_label)) +
         scale_fill_manual(values = as.character(dictionary_plot$Color)) +
         coord_flip() + scale_x_discrete(label = as_labeller(Genus_species_label_lookup)) + theme_bw() +
         theme(legend.position = "bottom", axis.text.x = element_text(size = 16), axis.text.y = element_text(size = 6, face = "plain"), axis.ticks.y = element_blank(), 
               panel.border = element_rect(linetype = "solid", fill = NA, linewidth = 1), axis.title.y = element_blank(),
               strip.background = element_rect(colour = "white", fill = "white"), strip.text = element_text(colour = "black", face = "bold", size = rel(1.2))) + 
         geom_hline(yintercept = 0) + ylab("log2FoldChange") +
         facet_wrap(vars(cohort), scales = "fixed", ncol = 3, labeller = labeller(cohort = header_namer)), width = 15, height = 20, units = "in", dpi = 300)


#FIGURE 2E
#--------------------------------------------------------------------------------------------------------------

# HEATMAPS
us <- dplyr::filter(.data = sigtab_dataset_art, cohort == "boston")
botswana <- dplyr::filter(.data = sigtab_dataset_art, cohort == "botswana")
uganda <- dplyr::filter(.data = sigtab_dataset_art, cohort == "uganda_2")

length(intersect(us$taxon, botswana$taxon)) -> us_botswana_overlap #3
length(intersect(us$taxon, uganda$taxon)) -> us_uganda_overlap #0
length(intersect(botswana$taxon, uganda$taxon)) -> botswana_uganda_overlap #9

# US-BOTSWANA Overlap
common_us_bots <- c(intersect(us$taxon, botswana$taxon))
botswana_common <- botswana[botswana$taxon%in%common_us_bots,,drop=F]
us_common <- us[us$taxon%in%common_us_bots,,drop=F]
all.equal(botswana_common$taxon, us_common$taxon)
```

```
## [1] "2 string mismatches"
```

```
data1 <- cbind(botswana_common$lfc, us_common$lfc)
colnames(data1) <- c("Botswana", "US")
row.names(data1) <- botswana_common$taxon
data1 <- as.data.frame(data1)
data1$Genus_Species_all <- botswana_common$Genus_Species_all
data_melt1 <- melt(data1)
```

```
## Using Genus_Species_all as id variables
```

```
ggsave("Figure2E_heat_US_BOTSWANA_v4.pdf", 
       ggplot(data = data_melt1, aes(x = variable, y = Genus_Species_all)) + geom_tile(aes(fill = value), color = "white", size = 2) +
         scale_fill_gradient2(low = "brown", mid = "white", high = "darkblue", limits = c(-1.1,1.1)) + theme_bw() +
         scale_y_discrete(label = as_labeller(Genus_species_label_lookup)) +
         theme(axis.text.x = element_text(size = 16), axis.text.y = element_text(size = 16, face = "plain"),
               axis.ticks = element_blank(), axis.title = element_blank(),
               panel.grid = element_line(linewidth = 0.1), panel.background = element_rect(fill = "white")), width = 15, height = 10)


# UGANDA-BOTSWANA Overlap
common_ug_bots <- c(intersect(botswana$taxon, uganda$taxon))
botswana_common <- botswana[botswana$taxon%in%common_ug_bots,,drop=F]
uganda_common <- uganda[uganda$taxon%in%common_ug_bots,,drop=F]
all.equal(botswana_common$taxon, uganda_common$taxon)
```

```
## [1] "8 string mismatches"
```

```
data2 <- cbind(botswana_common$lfc, uganda_common$lfc)
colnames(data2) <- c("Botswana", "Uganda")
row.names(data2) <- botswana_common$taxon
data2 <- as.data.frame(data2)
data2$Genus_Species_all <- botswana_common$Genus_Species_all
data_melt2 <- melt(data2)
```

```
## Using Genus_Species_all as id variables
```

```
ggsave("Figure2E_heat_UGANDA_BOTSWANA_v2.pdf", 
       ggplot(data = data_melt2, aes(x = variable, y = Genus_Species_all)) + geom_tile(aes(fill = value), color = "white", size = 2) +
         scale_fill_gradient2(low = "brown", mid = "white", high = "darkblue", limits = c(-1.8, 1.8)) + theme_bw() +
         scale_y_discrete(label = as_labeller(Genus_species_label_lookup)) +
         theme(axis.text.x = element_text(size = 16), axis.text.y = element_text(size = 16, face = "plain"),
               axis.ticks = element_blank(), axis.title = element_blank(),
               panel.grid = element_line(linewidth = 0.1), panel.background = element_rect(fill = "white")), width = 15, height = 10)

#VENN-EULER DIAGRAMS
BioVenn::draw.venn(us$taxon, botswana$taxon, uganda$taxon, title = NULL, subtitle = NULL, 
                   xtitle = "U.S.", x_c = "royalblue4",
                   ytitle = "Botswana", y_c = "darkorange",
                   ztitle = "Uganda", z_c = "forestgreen",
                   nrtype = "abs", 
                   output = "pdf", filename = "Figure2E_venn_v2.pdf")
```

```
## [1] "x total: 53"
## [1] "y total: 26"
## [1] "z total: 128"
## [1] "x only: 50"
## [1] "y only: 14"
## [1] "z only: 119"
## [1] "x-y total overlap: 3"
## [1] "x-z total overlap: 0"
## [1] "y-z total overlap: 9"
## [1] "x-y only overlap: 3"
## [1] "x-z only overlap: 0"
## [1] "y-z only overlap: 9"
## [1] "x-y-z overlap: 0"
```

```
## $x
##  [1] "GCAAGCGTTATCCGGATTTACTGGGTGTAAAGGGAGCGTAGACGGCATGGCAAGTCTGAAGTGAAATGCGGGGGCTCAACCCCTGAACTGCTTTGGAAACTGTCAGGCTGGAGTGCAGGAGAGGTAAGTGGAATTCCTAGTGTAGCGGTGAAATGCGTAGATATTAGGAGGAACACCAGTGGCGAAGGCGGCTTACTGGACTGTAACTGACGTTGAGGCTCGAAAGCGTGGGGAGCAAAC"
##  [2] "GCAAGCGTTATCCGGATTTACTGGGTGTAAAGGGAGCGTAGACGGCCGTGCAAGTCTGATGTGAAAGGCTGGGGCTCAACCCCGGGACTGCATTGGAAACTGTATGGCTGGAGTGCCGGAGAGGTAAGCGGAATTCCTAGTGTAGCGGTGAAATGCGTAGATATTAGGAGGAACACCAGTGGCGAAGGCGGCTTACTGGACGGTAACTGACGTTGAGGCTCGAAAGCGTGGGGAGCAAAC"
##  [3] "CCGAGCGTTATCCGGATTTATTGGGTTTAAAGGGTGCGTAGGCTGTTTTTTAAGTTAGAGGTGAAAGCTCGACGCTCAACGTCGAAATTGCCTCTGATACTGAGAGACTAGAGTGTAGTTGCGGAAGGCGGAATGTGTGGTGTAGCGGTGAAATGCTTAGATATCACACAGAACACCGATTGCGAAGGCAGCTTTCCAAGCTATTACTGACGCTGAGGCACGAAAGCGTGGGGAGCGAAC"
##  [4] "ACAAGCGTTGTCCGGAATTACTGGGTGTAAAGGGAGCGCAGGCGGGAAGACAAGTTGGAAGTGAAATCTATGGGCTCAACCCATAAACTGCTTTCAAAACTGCTGGCCTTGAGTAGTGCAGAGGTAGGTGGAATTCCCGGTGTAGCGGTGGAATGCGTAGATATCGGGAGGAACACCAGTGGCGAAGGCGGCCTACTGGGCACCAACTGACGCTGAGGCTCGAAAGTGTGGGTAGCAAAC"
##  [5] "GCGAGCGTTGTCCGGAATTACTGGGTGTAAAGGGAGTGTAGGCGGGAAGGCAAGTCAGAAGTGAAAATTATGGGCTTAACCCATAACCTGCTTTTGAAACTGTTTTTCTTGAGTGAGGCAGAGGCAAGCGGAATTCCTAGTGTAGCGGTGAAATGCGTAGATATTAGGAGGAACACCAGTGGCGAAGGCGGCTTGCTGGGCCTTTACTGACGCTGAGGCTCGAAAGCGTGGGGAGCAAAC"
##  [6] "GCAAGCGTTATCCGGATTTACTGGGTGTAAAGGGAGCGTAGGCGGTCCTGCAAGTCTGATGTGAAAACCCGGGGCTCAACCCCGGGACTGCATTGGAAACTGTAGGACTAGAGTGTCGGAGGGGTAAGTGGAATTCCTAGTGTAGCGGTGAAATGCGTAGATATTAGGAGGAACACCAGTGGCGAAGGCGGCTTACTGGACGACCACTGACGCTGAGGCTCGAAAGCGTGGGGAGCAAAC"
##  [7] "GCAAGCGTTGTCCGGAATTACTGGGTGTAAAGGGAGCGTAGGCGGGATGCCAAGTCAGCTGTGAAAACTATGGGCTTAACCTGTAGACTGCAGTTGAAACTGGTATTCTTGAGTGAAGTAGAGGTTGGCGGAATTCCGAGTGTAGCGGTGAAATGCGTAGATATTCGGAGGAACACCGGTGGCGAAGGCGGCCAACTGGGCTTTAACTGACGCTGAGGCTCGAAAGTGTGGGGAGCAAAC"
##  [8] "ACAAGCGTTGTCCGGATTTACTGGGTGTAAAGGGCGCGTAGGCGGACTGTCAAGTCAGTCGTGAAATACCGGGGCTTAACCCCGGGGCTGCGATTGAAACTGACAGCCTTGAGTATCGGAGAGGAAAGCGGAATTCCTAGTGTAGCGGTGAAATGCGTAGATATTAGGAGGAACACCAGTGGCGAAGGCGGCTTACTGGACGGTAACTGACGTTGAGGCTCGAAAGCGTGGGGAGCAAAC"
##  [9] "ACAAGCGTTGTCCGGAATTACTGGGTGTAAAGGGAGCGCAGGCGGGAAGACAAGTTGGAAGTGAAATCTATGGGCTCAACCCATAAACTGCTTTCAAAACTGTTTTTCTTGAGTAGTGCAGAGGTAGGCGGAATTCCCGGTGTAGCGGTGGAATGCGTAGATATCGGGAGGAACACCAGTGGCGAAGGCGGCCTACTGGGCACCAACTGACGCTGAGGCTCGGAAGTGTGGGTAGCAAAC"
## [10] "ACAAGCGTTGTCCGGAACTACTGGGTGTAAAGGGAGCGCAGGCGGGAAGACAAGTTGGAAGTGAAATCTATGGGCTCAACCCATAAACTGCTTTCAAAACTGTTTTTCTTGAGTAGTGCAGAGGTAGGCGGAATTCCCGGTGTAGCGGTGGAATGCGTAGATATCGGGAGGAACACCAGTGGCGAAGGCGGCCTACTGGGCACCAACTGACGCTGAGGCTCGAAAGTGTGGGTAGCAAAC"
## [11] "GCGAGCGTTATCCGGATTTATTGGGTTTAAAGGGTGCGTAGGCGGCACGCCAAGTCAGCGGTGAAATTTTCGGGCTCAACCCGGACTGTGCCGTTGAAACTGGCGAGCTAGAGTGCACAAGAGGCAGGCGGAATGCGTGGTGTAGCGGTGAAATGCATAGATATCACGCAGAACCCCGATTGCGAAGGCAGCCTGCTAGGGTGCGACAGACGCTGAGGCACGAAAGCGTGGGTATCGAAC"
## [12] "GCGAGCGTTATCCGGATTTATTGGGTTTAAAGGGTGCGTAGGCGGAAGAATAAGTCAGCGGTGAAATGCTTCAGCTCAACTGGAGAATTGCCGATGAAACTGTTTTTCTAGAGTATAAAAGAGGTATGCGGAATGCGTGGTGTAGCGGTGAAATGCATAGATATCACGCAGAACCCCGATTGCGAAGGCAGCATACTGGGCTATAACTGACGCTGAAGCACGAAAGCGTGGGTATCGAAC"
## [13] "ACAAGCGTTGTCCGGAATTACTGGGTGTAAAGGGAGCGCAGGCGGGCGATCAAGTTGGAAGTGAAATCCATGGGCTCAACCCATGAACTGCTTTCAAAACTGATTGTCTTGAGTAGTGCAGAGGTAGGCGGAATTCCCGGTGTAGCGGTGGAATGCGTAGATATCGGGAGGAACACCAGTGGCGAAGGCGGCCTACTGGGCACCAACTGACGCTGAGGCTCGAAAGTGTGGGTAGCAAAC"
## [14] "GCAAGCGTTGTCCGGAATTATTGGGCGTAAAGCGCGCGCAGGCGGCTTCTTAAGTCCATCTTAAAAGTGCGGGGCTTAACCCCGTGATGGGATGGAAACTGGGAGGCTGGAGTATCGGAGAGGAAAGTGGAATTCCTAGTGTAGCGGTGAAATGCGTAGAGATTAGGAAGAACACCGGTGGCGAAGGCGACTTTCTGGACGACAACTGACGCTGAGGCGCGAAAGCGTGGGGAGCAAACA"
## [15] "GCAAGCGTTATCCGGATTTACTGGGTGTAAAGGGAGCGCAGGCGGCATGATAAGTCTGATGTGAAAACCCAAGGCTCAACCATGGGACTGCATTGGAAACTGTCGTGCTGGAGTGTCGGAGAGGTAAGCGGAATTCCTAGTGTAGCGGTGAAATGCGTAGATATTAGGAGGAACACCAGTGGCGAAGGCGGCTTACTGGACGGTAACTGACGTTGAGGCTCGAAAGCGTGGGGAGCAAAC"
## [16] "GCGAGCGTTATCCGGAATTACTGGGTGTAAAGGGTGTGTAGGCGGGGTGTCAAGTCAGATGTGAAAACTGTGGGCTCAACCCACAAACTGCATTTGAAACTGATACTCTTGAGAGTGGGAGAGGTAAACGGAATTCCTGGTGTAGTAGTGAAATGCGTAGATATCAGGAGGAACACCGGTGGCGAAGGCGGTTTACTGGACCACAACTGACGCTGAGACACGAAAGCGTGGGGAGCAAAC"
## [17] "GCAAGCGTTATCCGGATTTACTGGGTGTAAAGGGAGCGTAGACGGCATGGCAAGCCAGATGTGAAAGCCCGGGGCTCAACCCCGGGACTGCATTTGGAACTGTCAGGCTAGAGTGTCGGAGAGGAAAGCGGAATTCCTAGTGTAGCGGTGAAATGCGTAGATATTAGGAGGAACACCAGTGGCGAAGGCGGCTTACTGGACGGTAACTGACGTTGAGGCTCGAAAGCGTGGGGAGCAAAC"
## [18] "ACAAGCGTTGTCCGGAATTACTGGGTGTAAAGGGAGCGCAGGCGGGAAGACAAGTTGGGAGTGAAATCTATGGGCTCAACCCATAAACTGCTTTCAAAACTGTTTTTCTTGAGTAGTGCAGAGGTAGGCGGAATTCCCGGTGTAGCGGTGGAATGCGTAGATATCGGGAGGAACACCAGTGGCGAAGGCGGCCTACTGGGCACCAACTGACGCTGAGGCTCGAAAGTGTGGGTAGCAAAC"
## [19] "GCGAGCGTTATCCGGATTTATTGGGTTTAAAGGGTGCGTAGGCGGGTGATTAAGTCAGCGGTGAAATGCGTCAGCTTAACTGGCGAACTGCCATTGAAACTGGTTACCTTGAGTGTAGCGGAAGTATGCGGAATGCGTGGTGTAGCGGTGAAATGCATAGATATCACGCAGAACTCCGATTGCGAAGGCAGCATACCATACTATAACTGACGCTGAAGCACGAAAGCGTGGGTATCGAAC"
## [20] "GCAAGCGTTGTCCGGATTTATTGGGCGTAAAGCGAGCGCAGGCGGAAGAATAAGTCTGATGTGAAAGCCCTCGGCTTAACCGAGGAACTGCATCGGAAACTGTTTTTCTTGAGTGCAGAAGAGGAGAGTGGAACTCCATGTGTAGCGGTGGAATGCGTAGATATATGGAAGAACACCAGTGGCGAAGGCGGCTCTCTGGTCTGCAACTGACGCTGAGGCTCGAAAGCATGGGTAGCGAAC"
## [21] "GCAAGCGTTATCCGGATTTACTGGGTGTAAAGGGTGCGTAGGTGGCAGTGCAAGTCAGATGTGAAAGGCCGGGGCTCAACCCCGGAGCTGCATTTGAAACTGCGCGGCTAGAGTACAGGAGAGGCAGGCGGAATTCCTAGTGTAGCGGTGAAATGCGTAGATATTAGGAGGAACACCAGTGGCGAAGGCGGCCTGCTGGACTGTTACTGACACTGAGGCACGAAAGCGTGGGGAGCAAAC"
## [22] "GCGAGCGTTGTCCGGAATTATTGGGCGTAAAGAGTACGTAGGCGGTTTGCTAAGCGCAAGGTGAAAGGCAGTGGCTTAACCATTGTAAGCCTTGCGAACTGGCAGACTTGAGTGCAGGAGAGGAAAGCGGAATTCCTAGTGTAGCGGTGAAATGCGTAGATATTAGGAGGAACACCGGTGGCGAAGGCGGCTTTCTGGACTGTAACTGACGCTGAGGTACGAAAGCGTGGGGAGCAAACA"
## [23] "GCAAGCGTTGTCCGGAATCACTGGGCGTAAAGGGCGCGTAGGCGGCAATATAAGTCAGATGTGAAAGGTGAGGGCTCAACCCTTAGACTGCATCTGATACTGTATAGCTTGAGTGTGAGAGAGGAAAGCGGAATTCCTAGTGTAGCGGTGAAATGCGTAGATATTAGGAGGAACACCAGTGGCGAAGGCGGCTTTCTGGCTCATAACTGACGCTGAGGCGCGAAAGCGTGGGGAGCAAAC"
## [24] "GCAAGCGTTATCCGGATTTACTGGGTGTAAAGGGAGCGTAGGCGGCGATGCAAGTCAGAAGTGAAAGCCCAGGGCTTAACCGTGGGACTGCTTTTGAAACTGTGTTGCTGGATTGCCGGAGAGGTAAGTGGAATTCCTAGTGTAGCGGTGAAATGCGTAGATATTAGGAGGAACACCAGTGGCGAAGGCGGCTTACTGGACGGTGAATGACGCTGAGGCTCGAAAGCGTGGGGAGCAAAC"
## [25] "GCGAGCGTTGTCCGGAATTACTGGGCGTAAAGGGAGCGTAGGCGGTCTGATAAGTTGGATGTGAAATACCCGGGCTTAACTTGGGGGGTGCATCCAATACTGTTGGACTAGAGTACAGGAGAGGAAAGCGGAATTCCTAGTGTAGCGGTGAAATGCATAGATATTAGGAGGAACATCGGTGGCGAAGGCGGCTTTCTGGACTGCAACTGACGCTGAGGCTCGAAAGCGTGGGGAGCAAAC"
## [26] "GCAAGCGTTATCCGGATTTATTGGGTGTAAAGGGTGCGTAGACGGGAAGGTAAGTTAGTTGTGAAATCCCTCGGCTCAACTGAGGAACTGCGACTAAAACTGCTTTTCTTGAGTGCTGGAGAGGAAAGTGGAATTCCTAGTGTAGCGGTGAAATGCGTAGATATTAGGAGGAACACCAGTGGCGAAGGCGACTTTCTGGACAGCAACTGACGTTGAGGCACGAAAGTGTGGGGAGCAAAC"
## [27] "CCGAGCGTTATCCGGATTTATTGGGTTTAAAGGGAGCGTAGGTGGACAGTTAAGTCAGTTGTGAAAGTTTGCGGCTCAACCGTAAAATTGCAGTTGATACTGGCTGTCTTGAGTACAGTAGAGGTGGGCGGAATTCGTGGTGTAGCGGTGAAATGCTTAGATATCACGAAGAACTCCGATTGCGAAGGCAGCTCACTGGAGCGCAACTGACGCTGAAGCTCGAAAGTGCGGGTATCGAAC"
## [28] "GCAAGCGTTATCCGGATTTACTGGGTGTAAAGGGTGCGTAGGTGGTGAGACAAGTCTGAAGTGAAAATCCGGGGCTTAACCCCGGAACTGCTTTGGAAACTGCCTGACTAGAGTACAGGAGAGGTAAGTGGAATTCCTAGTGTAGCGGTGAAATGCGTAGATATTAGGAGGAACACCAGTGGCGAAGGCGACTTACTGGACTGCTACTGACACTGAGGCACGAAAGCGTGGGGAGCAAAC"
## [29] "GCAAGCGTTATCCGGATTTACTGGGTGTAAAGGGAGCGTAGGTGGCAAGGCAAGCCAGAAGTGAAAACCCGGGGCTCAACCGCGGGATTGCTTTTGGAACTGTCATGCTAGAGTGCAGGAGGGGTGAGCGGAATTCCTAGTGTAGCGGTGAAATGCGTAGATATTAGGAGGAACACCAGTGGCGAAGGCGGCTTACTGGACGATAACTGACGCTGAGGCTCGAAAGCGTGGGGAGCAAAC"
## [30] "GCAAGCGTTGTCCGGATTTACTGGGTGTAAAGGGCGTGCAGCCGGGCCGGCAAGTCAGATGTGAAATCCACGGGCTTAACCCGTGAACTGCATTTGAAACTGTTGGTCTTGAGTATCGGAGAGGTAATCGGAATTCCTTGTGTAGCGGTGAAATGCGTAGATATAAGGAAGAACACCAGTGGCGAAGGCGGATTACTGGACGACAACTGACGGTGAGGCGCGAAAGCGTGGGGAGCAAAC"
## [31] "CCGAGCGTTATCCGGATTTATTGGGTTTAAAGGGAGCGTAGGCGGATTATTAAGTCAGTTGTGAAAGTTTGCGGCTCAACCGTAAAATTGCAGTTGATACTGGTAGTCTTGAGTGCAGCAGAGGTAGGCGGAATTCGTGGTGTAGCGGTGAAATGCTTAGATATCACGAAGAACTCCGATTGCGAAGGCAGCTTACTGGACTGTAACTGACGCTGATGCTCGAAAGTGTGGGTATCAAAC"
## [32] "GCAAGCGTTATCCGGAATTATTGGGCGTAAAGGGCTCGTAGGCGGTTCGTCGCGTCCGGTGTGAAAGTTCATCGCTTAACGGTGGATCCGCGCCGGGTACGGGCGGGCTTGAGTGCGGTAGGGGAGACTGGAATTCCCGGTGTAACGGTGGAATGTGTAGATATCGGGAAGAACACCAATGGCGAAGGCAGGTCTCTGGGCCGTTACTGACGCTGAGGAGCGAAAGCGTGGGGAGCGAAC"
## [33] "GCAAGCGTTATCCGGATTTACTGGGTGTAAAGGGAGCGCAGACGGCACTGCAAGTCTGAAGTGAAAGCCCGGGGCTCAACCCCGGGACTGCTTTGGAAACTGTAGAGCTAGAGTGCTGGAGAGGCAAGCGGAATTCCTAGTGTAGCGGTGAAATGCGTAGATATTAGGAGGAACACCAGTGGCGAAGGCGGCTTACTGGACGGTAACTGACGTTGAGGCTCGAAAGCGTGGGGAGCAAAC"
## [34] "GCAAGCGTTGTCCGGAATTACTGGGTGTAAAGGGAGCGTAGGCGGGATGCCAAGTCAGCTGTGAAAACTATGGGCTTAACTTGTAGACTGCAGTTGAAACTGGTATTCTTGAGTGAAGTAGAGGTTGGCGGAATTCCGAGTGTAGCGGTGAAATGCGTAGATATTCGGAGGAACACCGGTGGCGAAGGCGGCCAACTGGGCTTTAACTGACGCTGAGGCTCGAAAGTGTGGGGAGCAAAC"
## [35] "GCAAGCGTTATCCGGATCTACTGGGTGTAAAGGGAGCGTAGACGGATGGACAAGTCTGATGTGAAAGGCTGGGGCTCAACCCCGGGACTGCATTGGAAACTGCCCGTCTTGAGTGCCGGAGAGGTAAGCGGAATTCCTAGTGTAGCGGTGAAATGCGTAGATATTAGGAGGAACACCAGTGGCGAAGGCGGCTTACTGGACGGTAACTGACGTTGAGGCTCGAAAGCGTGGGGAGCAAAC"
## [36] "GCAAGCGTTATCCGGATTTACTGGGTGTAAAGGGAGCGTAGACGGTATGGCAAGTCTGATGTGAAAGGCCAGGGCTCAACCCTGGGACTGCATTGGAAACTGTCGAACTAGAGTGTCGGAGAGGCAAGTGGAATTCCTAGTGTAGCGGTGAAATGCGTAGATATTAGGAGGAACACCAGTGGCGAAGGCGGCTTACTGGACGGTAACTGACGTTGAGGCTCGAAAGCGTGGGGAGCAAAC"
## [37] "GCAAGCGTTGTCCGGAATGATTGGGCGTAAAGGGCGCGTAGGCGGCCAACTAAGTCTGGAGTGAAAGTCCTGCTTTTAAGGTGGGAATTGCTTTGGAAACTGGATGGCTTGAGTGCAGGAGAGGTAAGCGGAATTCCCGGTGTAGCGGTGAAATGCGTAGAGATCGGGAGGAACACCAGTGGCGAAGGCGGCTTACTGGACTGTAACTGACGCTGAGGCGCGAAAGTGTGGGGAGCAAAC"
## [38] "GCAAGCGTTATCCGGATTTACTGGGTGTAAAGGGAGCGTAGACGGATGGACAAGTCTGATGTGAAAGGCTGGGGCTCAACCCCGGGACTGCATTGGAAACTGCCCGTCTTGAGTGCCGGAGAGGTAAGCGGAATTCCTAGTGTAGCGGTGAAATGCGTAGATATACGGAGGAACACCAGTGGCGAAGGCGGCCTGCTGGACATTAACTGACGCTGAGGCGCGAAAGCGTGGGGAGCAAAC"
## [39] "GCAAGCGTTGTCCGGAATTATTGGGCGTAAAGAGTACGTAGGCGGTCTGGTAAGCGCAAGGTGAAAGGCATAGGCTCAACCAATGTCAGCCTTGCGAACTGTCAGACTTGAGTGCAGGAGGGGAAAGTGGAATTCCTAGTGTAGCGGTGAAATGCGTAGATATTAGGAGGAACACCAGTGGCGAAGGCGACTTTCTGGACTGTAACTGACGCTGAGGTACGAAAGCGTGGGGAGCAAACA"
## [40] "GCGAGCGTTGTCCGGAATTACTGGGTGTAAAGGGAGCGTAGGCGGGACAGCAAGTTGAATGTGAAATCTATGGGCTCAACCCATAAACTGCGTTCAAAACTGTTGTTCTTGAGTGAAGTAGAGGTAGGCGGAATTCCTAGTGTAGCGGTGAAATGCGTAGATATTAGGAGGAACACCAGTGGCGAAGGCGGCCTACTGGGCTTTAACTGACGCTGAGGCTCGAAAGCGTGGGTAGCAAAC"
## [41] "GCAAGCGTTATCCGGATTTACTGGGTGTAAAGGGAGCGTAGGCGGTCCTGCAAGTCTGATGTGAAAGGCCGGGGCTCAACCCCGGGACTGCATTGGAAACTGTAGGACTAGAGTGTCGGAGGGGTAAGTGGAATTCCTAGTGTAGCGGTGAAATGCGTAGATATTAGGAGGAACACCAGTGGCGAAGGCGGCTTACTGGACGGTAACTGACGTTGAGGCTCGAAAGCGTGGGGAGCAAAC"
## [42] "GCAAGCGTTGTCCGGAATTACTGGGTGTAAAGGGAGCGTAGGCGGGGAGACAAGTTGAATGTTTAAACTATCGGCTCAACTGATAGTCGCGTTCAAAACTATCACTCTTGAGTGCAGTAGAGGTAGGCGGAATTCCTAGTGTAGCGGTGAAATGCGTAGATATTAGGAGGAACACCAGTGGCGAAGGCGGCCTACTGGGCTGTAACTGACGCTGAGGCTCGAAAGCGTGGGTAGCAAACA"
## [43] "GCGAGCGTTAATCGGAATTACTGGGCGTAAAGCGCACGTAGGCTGTATGTCAAGTCAAGGGTGAAATCCCACGGCTCAACCGTGGAACTGCCTTTGAAACTGGCAAACTGGAGTATGTGAGAGGGCGGCGGAATTCCTGGTGTAGGAGTGAAATCCGTAGATATCAGGAGGAACATCAGTGGCGAAGGCGGCCGCCTGGCACAAAACTGACGCTGAGGTGCGAAAGCGTGGGTAGCAAAC"
## [44] "GCAAGCGTTATCCGGATTTACTGGGTGTAAAGGGCGTGTAGGCGGGACTGCAAGTCAGGTGTGAAAACCAGGGGCTCAACCTCTGGCCTGCATTTGAAACTGTAGTTCTTGAGTGCTGGAGAGGCAATCGGAATTCCGTGTGTAGCGGTGAAATGCGTAGATATACGGAGGAACACCAGTGGCGAAGGCGGATTGCTGGACAGTAACTGACGCTGAGGCGCGAAAGCGTGGGGAGCAAAC"
## [45] "GCAAGCGTTATCCGGATTTATTGGGTGTAAAGGGTGTGTAGGCGGGACTGCAAGTCAGACGTGAAAATCATGGGCTCAACCCATGACTTGCGTTTGAAACTGCGGTTCTTGAGAGTGGGAGAGGTAAACGGAATTCCTGGTGTAGCGGTGAAATGCGTAGATATCAGGAGGAACACCGGTGGCGAAGGCGGTTTACTGGACCACAACTGACGCTGAGACACGAAAGCGTGGGGAGCAAAC"
## [46] "CCGAGCGTTATCCGGATTTATTGGGTTTAAAGGGAGCGTAGATGGATGTTTAAGTCAGTTGTGAAAGTTTGCGGCTCAACCGTAAAATTGCAGTTGATACTGGCAGTCTTGAGTGCAGTAGAGGTGGGCGGAATTCGTGGTGTAGCGGTGAAATGCTTAGATATCACGAAGAACTCCGATTGCGAAGGCAGCCTGCTAAGCTGCAACTGACATTGAGGCTCGAAAGTGTGGGTATCAAAC"
## [47] "GCGAGCGTTATCCGGATTTATTGGGTTTAAAGGGTGCGTAGGCGGCCCTATAAGTCAGCGGTGAAATGTTCCGGCTCAACCGGGAAACTGCCGTTGAAACTGTAGAGCTAGAGTCCACAAGAGGTATGCGGAATGCGTGGTGTAGCGGTGAAATGCATAGATATCACGCAGAACCCCGATTGCGAAGGCAGCATACTGGGGTGAAACAGACGCTGAAGCACGAAAGCGTGGGTATCGAAC"
## [48] "GCAAGCGTTGTCCGGAATTACTGGGTGTAAAGGGCGTGTAGGCGGAGCTGCAAGTCAGATGTGAAATCCCGGGGCTCAACCCCGGAACTGCATTTGAAACTGTAGCCCTTGAGTATCGGAGAGGCAAGCGGAATTCCTAGTGTAGCGGTGAAATGCGTAGATATTAGGAGGAACACCAGTGGCGAAGGCGGCTTGCTGGACGACAACTGACGCTGAGGCGCGAAAGCGTGGGGAGCAAAC"
## [49] "GCGAGCGTTGTCCGGAATTACTGGGCGTAAAGGGCGCGTAGGCGGCCAATTAAGTTAGATGTGAAATCCCCGGGCTTAACCTGGGTGTTGCATTTAAAACTGATAGGCTTGAGTGCAGGAGAGGGAAGCGGAATTCCTAGTGTAGCGGTGGAATGCGTAGATATTAGGAGGAACACCAGTGGCGAAGGCGGCTTTCTGGACTGTAACTGACGCTGAGGCGCGAGAGCGTGGGGAGCAAAC"
## [50] "GCAAGCGTTATCCGGAATTACTGGGTGTAAAGGGTGAGTAGGCGGCACGGCAAGTAAGATGTGAAAGCCCGAGGCTTAACCTCGGGATTGCATTTTAAACTGCTGAGCTAGAGTACAGGAGAGGAAAGCGGAATTCCTAGTGTAGCGGTGAAATGCGTAGATATTAGGAAGAACACCAGTGGCGAAGGCGGCTTTCTGGACTGAAACTGACGCTGAGGCACGAAAGCGTGGGGAGCGAAC"
## [51] "GCAAGCGTTATCCGGATTTACTGGGTGTAAAGGGCGCGTAGGCGGGGATGCAAGTCAGATGTGAAATCTATGGGCTTAACCCATAAACTGCATTTGAAACTGTATCTCTTGAGTGCTGGAGAGGTAGACGGAATTCCTTGTGTAGCGGTGAAATGCGTAGATATAAGGAAGAACACCAGTGGCGAAGGCGGTCTACTGGACAGTAACTGACGCTGAGGCGCGAGAGCGTGGGGAGCAAAC"
## [52] "GCAAGCGTTGTCCGGATTTACTGGGTGTAAAGGGCGTGTAGGCGGAGCAGCAAGTCAGAAGTGAAATCTCTGGGCTCAACCCAGAAACTGCTTTTGAAACTGTTGCCCTTGAGTATCGGAGAGGCAGGCGGAATTCCTAGTGTAGCGGTGAAATGCGTAGATATTAGGAGGAACACCAGTGGCGAAGGCGGCCTGCTGGACGACAACTGACGCTGAGGCGCGAAAGCGTGGGGAGCAAAC"
## [53] "GCGAGCGTTGTCCGGATTTACTGGGTGTAAAGGGTGCGTAGGCGGCTAGACAAGTCAGGTGTGAAATACCGCAGCTCAACTGCGGGGCTGCACTTGAAACTGTAGAGCTTGAGTGATGGAGAGGTAAGCGGAATTCCTAGTGTAGCGGTAAAATGCGTAGATATTAGGAGGAACACCAGTGGCGAAGGCGGCTTACTGGACATTAACTGACGCTGAGGCACGAAAGCGTGGGGAGCAAAC"
## 
## $y
##  [1] "ACAAGCGTTGTCCGGAATTACTGGGTGTAAAGGGAGCGCAGGCGGGCGATCAAGTTGGAAGTGAAATCCATGGGCTCAACCCATGAACTGCTTTCAAAACTGGTCGTCTTGAGTAGTGCAGAGGTAGGCGGAATTCCCGGTGTAGCGGTGGAATGCGTAGATATCGGGAGGAACACCAGTGGCGAAGGCGGCCTACTGGGCACCAACTGACGCTGAGGCTCGAAAGTGTGGGTAGCAAAC"
##  [2] "ACAAGCGTTGTCCGGAATTACTGGGTGTAAAGGGAGCGCAGGCGGGAAGACAAGTTGGAAGTGAAATCCATGGGCTCAACCCATGAACTGCTTTCAAAACTGTTTTTCTTGAGTAGTGCAGAGGTAGGCGGAATTCCCGGTGTAGCGGTGGAATGCGTAGATATCGGGAGGAACACCAGTGGCGAAGGCGGCCTACTGGGCACCAACTGACGCTGAGGCTCGAAAGTGTGGGTAGCAAAC"
##  [3] "GCGAGCGTTGTCCGGATTTACTGGGCGTAAAGGGAGCGTAGGCGGATTTTTAAGTGAGATGTGAAATACTCGGGCTTAACCTGAGTGCTGCATTTCAAACTGGAAGTCTAGAGTGCAGGAGAGGAGAAGGGAATTCCTAGTGTAGCGGTGAAATGCGTAGAGATTAGGAAGAACACCAGTGGCGAAGGCGCTTCTCTGGACTGTAACTGACGCTGAGGCTCGAAAGCGTGGGGAGCAAAC"
##  [4] "GCGAGCGTTGTCCGGATTTACTGGGTGTAAAGGGCGTGTAGGCGGAGATGCAAGTTGGGAGTGAAATCCATGGGCTCAACCCATGAACTGCTTCCAAAACTGTATCCCTTGAGTATCGGAGAGGCAAGCGGAATTCCTAGTGTAGCGGTGAAATGCGTAGATATTAGGAGGAACACCAGTGGCGAAGGCGGCTTGCTGGACGACAACTGACGCTGAGGCGCGAAAGCGTGGGGAGCAAAC"
##  [5] "GCAAGCGTTGTCCGGATTTACTGGGTGTAAAGGGCGTGCAGCCGGGCATGCAAGTCAGATGTGAAATCTCAGGGCTTAACCCTGAAACTGCATTTGAAACTGTATGTCTTGAGTGCCGGAGAGGTAATCGGAATTCCTTGTGTAGCGGTGAAATGCGTAGATATAAGGAAGAACACCAGTGGCGAAGGCGGATTACTGGACGGTAACTGACGGTGAGGCGCGAAAGCGTGGGGAGCGAAC"
##  [6] "GCGAGCGTTGTCCGGAATTACTGGGTGTAAAGGGAGCGTAGGCGGGATGGCAAGTCAGATGTGAAAACTATGGGCTCAACCCATAGACTGCATTTGAAACTGTTGTTCTTGAGTGAGGTAGAGGTAAGCGGAATTCCTGGTGTAGCGGTGAAATGCGTAGAGATCAGGAGGAACATCGGTGGCGAAGGCGGCTTACTGGGCCTTTACTGACGCTGAGGCTCGAAAGCGTGGGGAGCAAAC"
##  [7] "GCAAGCGTTGTCCGGATTTACTGGGTGTAAAGGGCGTGCAGCCGGGTCTGCAAGTCAGATGTGAAATCCATGGGCTCAACCCATGAACTGCATTTGAAACTGTAGATCTTGAGTGTCGGAGGGGCAATCGGAATTCCTAGTGTAGCGGTGAAATGCGTAGATATTAGGAGGAACACCAGTGGCGAAGGCGGATTGCTGGACGATAACTGACGGTGAGGCGCGAAAGTGTGGGGAGCAAAC"
##  [8] "GCAAGCGTTGTCCGGATTTACTGGGTGTAAAGGGCGTGCAGCCGGGAATGCAAGTCAGATGTGAAATCCATGGGCTTAACCCATGAACTGCATTTGAAACTGTATTTCTTGAGTACTGGAGAGGCAATCGGAATTCCTAGTGTAGCGGTGAAATGCGTAGATATTAGGAGGAACACCAGTGGCGAAGGCGGATTGCTGGACAGCAACTGACGGTGAGGCGCGAAAGTGTGGGGAGCAAAC"
##  [9] "GCAAGCGTTGTCCGGATTTACTGGGTGTAAAGGGCGTGTAGGCGGAGATGCAAGTCGGGAGTGAAATCCATGGGCTCAACCCATGAACTGCTTTCGAAACTGTATCCCTTGAGTATCGGAGAGGCAAGCGGAATTCCTAGTGTAGCGGTGAAATGCGTAGATATTAGGAGGAACACCAGTGGCGAAGGCGGCTTGCTGGACGACAACTGACGCTGAGGCGCGAAAGCGTGGGGAGCAAAC"
## [10] "GCAAGCGTTGTCCGGATTTACTGGGTGTAAAGGGCGTGTAGGCGGAGATGCAAGTTAGGAGTGAAATCTATGGGCTCAACCCATAAACTGCTTCTAAAACTGTATCCCTTGAGTATCGGAGAGGCAAGCGGAATTCCTAGTGTAGCGGTGAAATGCGTAGATATTAGGAGGAACACCAGTGGCGAAGGCGGCTTGCTGGACGACAACTGACGCTGAGGCGCGAAAGCGTGGGGAGCAAAC"
## [11] "GCAAGCGTTATCCGGATTTACTGGGTGTAAAGGGAGCGTAGACGGCCGTGCAAGTCTGATGTGAAAGGCTGGGGCTCAACCCCGGGACTGCATTGGAAACTGTATGGCTGGAGTGCCGGAGAGGTAAGCGGAATTCCTAGTGTAGCGGTGAAATGCGTAGATATTAGGAGGAACACCAGTGGCGAAGGCGGCTTACTGGACGGTAACTGACGTTGAGGCTCGAAAGCGTGGGGAGCAAAC"
## [12] "GCGAGCGTTGTCCGGAATTACTGGGTGTAAAGGGAGCGTAGGCGGGATTGCAAGTCAGATGTGAAAACTATGGGCTTAACCCATAGACTGCATTTGAAACTGTAGTTCTTGAGTGAAGTAGAGGTAAGCGGAATTCCTAGTGTAGCGGTGAAATGCGTAGATATTAGGAGGAACATCGGTGGCGAAGGCGGCTTACTGGGCTTTTACTGACGCTGAGGCTCGAAAGCGTGGGGAGCAAAC"
## [13] "GCAAGCGTTGTCCGGAATTACTGGGTGTAAAGGGAGCGCAGGCGGAAGGACAAGTTGGAAGTGAAACCCACGGGCTCAACCCGTGAACTGCTTTCAAAACTGTTTTTCTTGAGTGGTGTAGAGGTAGGCGGAATTCCCGGTGTAGCGGTGGAATGCGTAGATATCGGGAGGAACACCAGTGGCGAAGGCGGCCTACTGGGCACTAACTGACGCTGAGGCTCGAAAGCATGGGTAGCAAAC"
## [14] "GCGAGCGTTATCCGGATTTATTGGGTTTAAAGGGAGCGCAGACGGGACTTTAAGTCAGCTGTGAAATTTTCCGGCTCAACCGGGAAACTGCAGTTGATACTGGCGTCCTTGAGTACGGTCGAGGCAGGCGGAATTCGTGGTGTAGCGGTGAAATGCTTAGATATCACGAAGAACCCCGATTGCGAAGGCAGCCTGCCAGACCGCAACTGACGTTCATGCTCGAAAGTGCGGGTATCAAAC"
## [15] "GCGAGCGTTGTCCGGAATTACTGGGTGTAAAGGGAGCGTAGGCGGGATCTTAAGTCAGGTGTGAAAACTATGGGCTCAACCCATAGACTGCACTTGAAACTGAGGTTCTTGAGTGAAGTAGAGGCAGGCGGAATTCCTAGTGTAGCGGTGAAATGCGTAGATATTAGGAGGAACATCAGTGGCGAAGGCGGCCTGCTGGGCTTTTACTGACGCTGAGGCTCGAAAGCGTGGGGAGCAAAC"
## [16] "GCAAGCGTTATCCGGATTTACTGGGTGTAAAGGGCGCGTAGGCGGGGATGCAAGTCAGATGTGAAATCTATGGGCTTAACCCATAAACTGCATTTGAAACTGTATCTCTTGAGTGCTGGAGAGGTAGACGGAATTCCTTGTGTAGCGGTGAAATGCGTAGATATAAGGAAGAACACCAGTGGCGAAGGCGGTCTACTGGACAGTAACTGACGCTGAGGCGCGAGAGCGTGGGGAGCAAAC"
## [17] "GCGAGCGTTATCCGGATTTATTGGGTTTAAAGGGAGCGTAGGCGGGCTGTTAAGTCAGCGGTCAAATGTCAGGGCCCAACCTTGGCATGCCGTTGATACTGGCGGCCTTGAGTTCACACAAGGAAGGTGGAATTCGTCGTGTAGCGGTGAAATGCTTAGATATGACGAAGAACTCCGATTGCGAAGGCAGCCTTCTGGGGTGTTACTGACGCTGAGGCTCGAAAGTGCGGGAATCAAACA"
## [18] "GCAAGCGTTATCCGGATTTACTGGGTGTAAAGGGCGTGTAGGCGGGAAAGCAAGTCAGATGTGAAAACTGTGGGCTCAACCCACAGCCTGCATTTGAAACTGTTTTTCTTGAGTACTGGAGAGGCAGATGGAATTCCTAGTGTAGCGGTGAAATGCGTAGATATTAGGAGGAACACCAGTGGCGAAGGCGATCTGCTGGACAGCAACTGACGCTGAGGCGCGAAAGCGTGGGGAGCAAAC"
## [19] "GCAAGCGTTATCCGGAATTACTGGGTGTAAAGGGTGCGTAGGCGGTATGGCAAGTTTGATGTGAAACCCACAGGCTTAACCTGTGGCTTGCATCGAAAACTACTGAACTAGAGTGCAGGAGAGGAAAGCGGAATTCCTAGTGTAGCGGTGAAATGCGTAGATATTAGGAAGAACACCAGTGGCGAAGGCGGCTTTCTGGACTGCAACTGACGCTGAGGCACGAAAGCGTGGGGAGCAAAC"
## [20] "GCGAGCGTTGTTCGGAATTACTGGGCGTAAAGCGCACGCAGGCGGTTCGTTAAGTCTGCTGTCAAAGGCTGAGGCTCAACCTCAGTTCTACAACAGATACTGGCGGACTAGAGTATGTGAGAGGGAAGTGGAATTCCCGGTGTAGCGGTGAAATGCGTAGATATCGGGAGGAACACCAGTGGCGAAGGCGGCTTCCTGGCACACTACTGACGCTCATGTGCGAAAGCCAGGGCAGCGAAC"
## [21] "GCAAGCGTTATCCGGATTTACTGGGTGTAAAGGGAGCGCAGACGGCACTGCAAGTCTGAAGTGAAAGCCCGGGGCTCAACCCCGGGACTGCTTTGGAAACTGTAGAGCTAGAGTGCTGGAGAGGCAAGCGGAATTCCTAGTGTAGCGGTGAAATGCGTAGATATTAGGAGGAACACCAGTGGCGAAGGCGGCTTACTGGACGGTAACTGACGTTGAGGCTCGAAAGCGTGGGGAGCAAAC"
## [22] "GCAAGCGTTATCCGGATTTACTGGGTGTAAAGGGAGCGTAGACGGCGAAGCAAGTCTGAAGTGAAAACCCAGGGCTCAACCCTGGGACTGCTTTGGAAACTGTTTTGCTAGAGTGTCGGAGAGGTAAGTGGAATTCCTAGTGTAGCGGTGAAATGCGTAGATATTAGGAGGAACACCAGTGGCGAAGGCGGCTTACTGGACGATAACTGACGTTGAGGCTCGAAAGCGTGGGGAGCAAAC"
## [23] "GCAAGCGTTGTCCGGATTTACTGGGTGTAAAGGGCGTGCAGCCGGGCCGGCAAGTCAGATGTGAAATCTGGAGGCTTAACCTCCAAACTGCATTTGAAACTGTAGGTCTTGAGTACCGGAGAGGTTATCGGAATTCCTTGTGTAGCGGTGAAATGCGTAGATATAAGGAAGAACACCAGTGGCGAAGGCGGATAACTGGACGGCAACTGACGGTGAGGCGCGAAAGCGTGGGGAGCAAAC"
## [24] "GCAAGCGTTGTCCGGAATTATTGGGCGTAAAGGGCGCGCAGGCGGCGTCGTAAGTCGGTCTTAAAAGTGCGGGGCTTAACCCCGTGAGGGGACCGAAACTGCGATGCTAGAGTATCGGAGAGGAAAGCGGAATTCCTAGTGTAGCGGTGAAATGCGTAGATATTAGGAGGAACACCAGTGGCGAAAGCGGCTTTCTGGACGACAACTGACGCTGAGGCGCGAAAGCCAGGGGAGCAAACG"
## [25] "GCAAGCGTTGTCCGGAATTATTGGGCGTAAAGGGAGCGCAGGCGGGAAACTAAGCGGATCTTAAAAGTGCGGGGCTCAACCCCGTGATGGGGTCCGAACTGGTTTTCTTGAGTGCAGGAGAGGAAAGCGGAATTCCCAGTGTAGCGGTGAAATGCGTAGATATTGGGAAGAACACCAGTGGCGAAGGCGGCTTTCTGGACTGTAACTGACGCTGAGGCTCGAAAGCTAGGGTAGCGAACG"
## [26] "GCAAGCGTTAATCGGAATAACTGGGCGTAAAGGGCATGCAGGCGGTTCATCAAGTAGGATGTGAAATCCCCGGGCTCAACCTGGGAACAGCATACTAAACTGGTGGACTAGAGTATTGCAGGGGGAGACGGAATTCCAGGTGTAGCGGTGGAATGCGTAGATATCTGGAAGAACACCAAAGGCGAAGGCAGTCTCCTGGGCAAATACTGACGCTCATATGCGAAAGCGTGGGTAGCAAAC"
## 
## $z
##   [1] "CCGGGCGTTATCCGGATTTATTGGGTTTAAAGGGAGCGTAGGCCGGAGATTAAGCGTGTTGTGAAATGTAGATGCTCAACATCTGCACTGCAGCGCGAACTGGTTTCCTTGAGTACGCACAAAGTGGGCGGAATTCGTGGTGTAGCGGTGAAATGCTTAGATATCACGAAGAACTCCGATTGCGAAGGCAGCTCACTGGAGCGCAACTGACGCTGAAGCTCGAAAGTGCGGGTATCGAAC"
##   [2] "CCGGGCGTTATCCGGATTTATTGGGTTTAAAGGGAGCGTAGGCCGGAGATTAAGCGTGTTGTGAAATGTAGACGCTCAACGTCTGCACTGCAGCGCGAACTGGTTTCCTTGAGTACGCACAAAGTGGGCGGAATTCGTGGTGTAGCGGTGAAATGCTTAGATATCACGAAGAACTCCGATTGCGAAGGCAGCTCACTGGAGCGCAACTGACGCTGAAGCTCGAAAGTGCGGGTATCGAAC"
##   [3] "CCGGGCGTTATCCGGATTTATTGGGTTTAAAGGGAGCGTAGGCCGCAGGTTAAGCGTGTTGTGAAATGTAGGGGCTCAACCTCTGCACTGCAGCGCGAACTGGCTTGCTTGAGTACGCACAACGTGGGCGGAATTCGTGGTGTAGCGGTGAAATGCTTAGATATCACGAAGAACTCCGATTGCGAAGGCAGCTCACGGGAGCGCAACTGACGCTGAAGCTCGAAAGTGCGGGTATCGAAC"
##   [4] "CCGGGCGTTATCCGGATTTATTGGGTTTAAAGGGAGCGTAGGCCGGAGATTAAGCGTGTTGTGAAATGTAGAGGCTCAACCTCTGCACTGCAGCGCGAACTGGTCTTCTTGAGTACGCACAACGTGGGCGGAATTCGTGGTGTAGCGGTGAAATGCTTAGATATCACGAAGAACTCCGATTGCGAAGGCAGCTCACGGGAGCGCAACTGACGCTGAAGCTCGAAAGTGCGGGTATCGAAC"
##   [5] "CCGGGCGTTATCCGGATTTATTGGGTTTAAAGGGAGCGTAGGCCGGAGATTAAGCGTGTTGTGAAATGTAGACGCTCAACGTCTGCACTGCAGCGCGAACTGGTTTCCTTGAGTACGCACAAAGTGGGCGGAATTCGTGGTGTAGCGGTGAAATGCTTAGATATCACGAAGAACTCCGATTGCGAAGGCAGCTCACGGGAGCGCAACTGACGCTGAAGCTCGAAAGTGCGGGTATCGAAC"
##   [6] "CCGGGCGTTATCCGGATTTATTGGGTTTAAAGGGAGCGTAGGCCGGAGATTAAGCGTGTTGTGAAATGTAGATGCTCAACATCTGCACTGCAGCGCGAACTGGTTTCCTTGAGTACGCATAAAGTGGGCGGAATTCGTGGTGTAGCGGTGAAATGCTTAGATATCACGAAGAACTCCGATTGCGAAGGCAGCTCACTGGGGCGCAACTGACGCTGAAGCTCGAAAGCGCGGGTATCGAAC"
##   [7] "GCAAGCGTTAATCGGAATAACTGGGCGTAAAGGGCATGCAGGCGGTTCATCAAGTAGGATGTGAAATCCCCGGGCTCAACCTGGGAACAGCATACTAAACTGGTGGACTAGAGTATTGCAGGGGGAGACGGAATTCCAGGTGTAGCGGTGGAATGCGTAGATATCTGGAAGAACACCAAAGGCGAAGGCAGTCTCCTGGGCAAATACTGACGCTCATATGCGAAAGCGTGGGTAGCAAAC"
##   [8] "CCGGGCGTTATCCGGATTTATTGGGTTTAAAGGGAGCGTAGGCCGGAGATTAAGCGTGTTGTGAAATGTAGAGGCTCAACCTCTGCACTGCAGCGCGAACTGGTCTTCTTGAGTACGCACAACGTGGGCGGAATTCGTGGTGTAGCGGTGAAATGCTTAGATATCACGAAGAACTCCGATTGCGAAGGCAGCTCACTGGAGCGCAACTGACGCTGAAGCTCGAAAGTGCGGGTATCGAAC"
##   [9] "GCGAGCGTTATCCGGATTTATTGGGTTTAAAGGGTGCGCAGGCGGCGCGCCAAGTCAGCGGTCAAAGTTCCGGGCTCAACCCGGTGTCGCCGTTGAAACTGGCGTGCTCGAGTGCGTGCGAGGAAGGCGGAATGCGTTGTGTAGCGGTGAAATGCATAGATATGACGCAGAACTCCGATTGCGAAGGCAGCTTTCCAGCGCGCTACTGACGCTGAGGCACGAAAGCGTGGGGATCGAACA"
##  [10] "CCGGGCGTTATCCGGATTTATTGGGTTTAAAGGGAGCGTAGGCCGCCCCTTAAGCGTGTTGTGAAATGCCGCGGCTCAACCGTGGCACTGCAGCGCGAACTGGGGGGCTTGAGTGCACGCAACGCAGGCGGAATTCGTGGTGTAGCGGTGAAATGCTTAGATATCACGAAGAACTCCGATTGCGAAGGCAGCTTGCGGGAGTGCGACTGACGCTGAAGCTCGAAGGTGCGGGTATCGAAC"
##  [11] "GCAAGCGTTGTCCGGAATTATTGGGCGTAAAGCGCGCGCAGGCGGCTTCTTAAGTCCATCTTAAAAGTGCGGGGCTTAACCCCGTGATGGGATGGAAACTGAGAGGCTGGAGTATCGGAGAGGAAAGTGGAATTCCTAGTGTAGCGGTGAAATGCGTAGAGATTAGGAAGAACACCGGTGGCGAAGGCGACTTTCTGGACGACAACTGACGCTGAGGCGCGAAAGCGTGGGGAGCAAACA"
##  [12] "CCGGGCGTTATCCGGATTTATTGGGTTTAAAGGGAGCGTAGGCCGGAGATTAAGCGTGTTGTGAAATGTAGACGCTCAACGTCTGCACTGCAGCGCGAACTGGTTTCCTTGAGTACGCACAAAGTGGGCGGAATTCGTGGTGTAGCGGTGAAATGCTTAGATATCACGAAGAACTCCGATTGCGAAGGCAGCTCACTGGGGCGCAACTGACGCTGAAGCTCGAAAGCGCGGGTATCGAAC"
##  [13] "CCGGGCGTTATCCGGATTTATTGGGTTTAAAGGGAGCGTAGGCCGTGAGGTAAGCGTGTTGTGAAATGTAGGCGCCCAACGTCTGCACTGCAGCGCGAACTGCCCCACTTGAGTGCGCGCAACGCCGGCGGAACTCGTCGTGTAGCGGTGAAATGCTTAGATATGACGAAGAACCCCGATTGCGAAGGCAGCTGGCGGGAGCGTAACTGACGCTGAAGCTCGAAAGCGCGGGTATCGAAC"
##  [14] "CCGGGCGTTATCCGGATTTATTGGGTTTAAAGGGAGCGTAGGCCGGAGATTAAGCGTGTTGTGAAATGTAGTGGCTCAACCTCTGCACTGCAGCGCGAACTGGTCTTCTTGAGTACGCACAACGTGGGCGGAATTCGTGGTGTAGCGGTGAAATGCTTAGATATCACGAAGAACTCCGATTGCGAAGGCAGCTCACGGGAGCGCAACTGACGCTGAAGCTCGAAAGTGCGGGTATCGAAC"
##  [15] "CCGGGCGTTATCCGGATTTATTGGGTTTAAAGGGAGCGTAGGCCGTCTTTTAAGCGTGTTGTGAAATACTGTCGCTCAACGACAGAGGTGCAGCGCGAACTGGGAGACTTGAGTGCGCGGAATGCAGGCGGAATTCGTCGTGTAGCGGTGAAATGCTTAGATATGACGAAGAACTCCGATTGCGAAGGCAGCTTGCAGTAGCGTAACTGACGCTGAAGCTCGAAAGTGCGGGTATCGAAC"
##  [16] "CCGGGCGTTATCCGGATTTATTGGGTTTAAAGGGAGCGTAGGCCGTCTTATAAGCGTGTTGTGAAATGTCGGGGCTCAACCTGGGCATTGCAGCGCGAACTGTGAGACTTGAGTGCGCAGGAAGTAGGCGGAATTCGTCGTGTAGCGGTGAAATGCTTAGATATGACGAAGAACTCCGATTGCGAAGGCAGCCTGCTGTAGCGCAACTGACGCTGAAGCTCGAAAGCGTGGGTATCGAAC"
##  [17] "ACAAGCGTTGTCCGGAATTACTGGGTGTAAAGGGAGCGCAGGCGGGAAGACAAGTTGGAAGTGAAATCCATGGGCTCAACCCATGAACTGCTTTCAAAACTGTTTTTCTTGAGTAGTGCAGAGGTAGGCGGAATTCCCGGTGTAGCGGTGGAATGCGTAGATATCGGGAGGAACACCAGTGGCGAAGGCGGCCTACTGGGCACCAACTGACGCTGAGGCTCGAAAGTGTGGGTAGCAAAC"
##  [18] "GCGAGCGTTGTTCGGAATCATTGGGCGTAAAGGGCGTGTAGGCGGCCCTGCAAGCCTGGCGTGAAATCCCGGGGCCCAACCCCGGAACCGCGCTGGGAACTGCTGGGCTTGAGCCGCTGTGGCGCAGCCGGAATTCCAGGTGTAGGGGTGAAATCTGTAGATATCTGGAAGAACACCGATGGCGAAGGCAGGCTGCGAGCGGACGGCTGACGCTGAGGCGCGAAGGCGCGGGGAGCGAAC"
##  [19] "ACAAGCGTTGTCCGGAATTACTGGGTGTAAAGGGAGCGCAGGCGGGAAAGCAAGTTGGAAGTGAAATCCATGGGCTCAACCCATGAACTGCTTTCAAAACTGTTTTTCTTGAGTAGTGCAGAGGTAGGCGGAATTCCCGGTGTAGCGGTGGAATGCGTAGATATCGGGAGGAACACCAGTGGCGAAGGCGGCCTACTGGGCACCAACTGACGCTGAGGCTCGAAAGTGTGGGTAGCAAAC"
##  [20] "CCGGGCGTTATCCGGATTTATTGGGTTTAAAGGGAGCGTAGGCCGCAGGTTAAGCGTGTTGTGAAATGTAGGGGCTCAACCTCTGCACTGCAGCGCGAACTGGCTTGCTTGAGTACGCACAACGTGGGCGGAATTCGTGGTGTAGCGGTGAAATGCTTAGATATCACGAAGAACTCCGATTGCGAAGGCAGCTCACTGGAGCGCAACTGACGCTGAAGCTCGAAAGTGCGGGTATCGAAC"
##  [21] "GCGAGCGTTGTCCGGAATTATTGGGCGTAAAGAGCATGTAGGCGGTTTTTTAAGTCTGGAGTGAAAATGCGGGGCTCAACCCCGTATGGCTCTGGATACTGGAAGACTTGAGTGCAGGAGAGGAAAGGGGAATTCCCAGTGTAGCGGTGAAATGCGTAGATATTGGGAGGAACACCAGTGGCGAAGGCGCCTTTCTGGACTGTGTCTGACGCTGAGATGCGAAAGCCAGGGTAGCGAACG"
##  [22] "GCAAGCGTTGTCCGGATTTACTGGGTGTAAAGGGCGTGTAGGCGGAGATGCAAGTCGGGAGTGAAATCCATGGGCTCAACCCATGAACTGCTTTCGAAACTGTATCCCTTGAGTATCGGAGAGGCAAGCGGAATTCCTAGTGTAGCGGTGAAATGCGTAGATATTAGGAGGAACACCAGTGGCGAAGGCGGCTTGCTGGACGACAACTGACGCTGAGGCGCGAAAGCGTGGGGAGCAAAC"
##  [23] "CCGGGCGTTATCCGGATTTATTGGGTTTAAAGGGAGCGTAGGCCGGAGATTAAGCGTGTTGTGAAATGTAGATGCTCAACATCTGCACTGCAGCGCGAACTGGTTTCCTTGAGTACGCATAAAGTGGGCGGAATTCGTGGTGTAGCGGTGAAATGCTTAGATATCACGAAGAACTCCGATTGCGAAGGCAGCTCACTGGAGCGCAACTGACGCTGAAGCTCGAAAGTGCGGGTATCGAAC"
##  [24] "CCGGGCGTTATCCGGATTTATTGGGTTTAAAGGGAGCGTAGGCCGGAGATTAAGCGTGTTGTGAAATGTAGATGCTCAACATCTGAACTGCAGCGCGAACTGGTTTCCTTGAGTACGCACAAAGTGGGCGGAATTCGTGGTGTAGCGGTGAAATGCTTAGATATCACGAAGAACTCCGATTGCGAAGGCAGCTCACTGGAGCGCAACTGACGCTGAAGCTCGAAAGTGCGGGTATCGAAC"
##  [25] "CCGGGCGTTATCCGGATTTATTGGGTTTAAAGGGAGCGTAGGCCGGAGATTAAGCGTGTTGTGAAATGTAGTGGCTCAACCTCTGCACTGCAGCGCGAACTGGTCTTCTTGAGTACGCACAACGTGGGCGGAATTCGTGGTGTAGCGGTGAAATGCTTAGATATCACGAAGAACTCCGATTGCGAAGGCAGCTCACTGGAGCGCAACTGACGCTGAAGCTCGAAAGTGCGGGTATCGAAC"
##  [26] "CCGGGCGTTATCCGGATTTATTGGGTTTAAAGGGAGCGCAGGCCGTGGGCTAAGCGTGCCGTGAAATTCTGTCGCTCAACGGCAGACGTGCGGCGCGAACTGGTCCACTTGAGTACGCGGGACGTTGGCGGAATTCGTGGTGTAGCGGTGAAATGCTTAGATATCACGAAGAACTCCGATTGCGAAGGCAGCTGACGGTAGCGCAACTGACGCTGAGGCTCGAAAGCGCGGGTATCGAAC"
##  [27] "CCAGGCGTTATCCGGATTCATTGGGTTTAAAGGGAGCGTAGGCCGCCTTTTAAGCGTGTTGTGAAATGTAGGGGCTCAACCCCTGCACTGCAGCGCGAACTGGAGGGCTTGAGTACACACAAAGTAGGCGGAATTCATGGTGTAGCGGTGAAATGCTTAGATATCATGAAGAACCCCGATTGCGAAGGCAGCTTACTGGAGTGTAACTGACGCTGAAGCTCGAAAGCGCGGGTATCGAAC"
##  [28] "GCGAGCGTTGTTCGGAATCATTGGGCGTAAAGGGCATGTAGGCGGTTACGCAAGCCTGGTGTGAAAGCCCGAGGCTCAACCTCGGGATGCGCCGGGAACTGTGCAACTAGAGTAGCTGAGGGGCAGCCGGAATTCCAGGTGTAGGGGTGAAATCTGTAGATATCTGGAAGAACACCGATGGCGAAGGCAGGCTGCCAGCAGATTACTGACGCTGAGGTGCGAAGGTGCGGGGAGCGAACA"
##  [29] "GCGAGCGTTGTCCGGATTTACTGGGTGTAAAGGGCGTGTAGGCGGAGATGCAAGTTGGGAGTGAAATCCATGGGCTCAACCCATGAACTGCTTCCAAAACTGTATCCCTTGAGTATCGGAGAGGCAAGCGGAATTCCTAGTGTAGCGGTGAAATGCGTAGATATTAGGAGGAACACCAGTGGCGAAGGCGGCTTGCTGGACGACAACTGACGCTGAGGCGCGAAAGCGTGGGGAGCAAAC"
##  [30] "CCAGGCGTTATCCGGATTTATTGGGTTTAAAGGGAGCGCAGGCGGACCTTTAAGTCAGCTGTGAAATACGGCGGCTCAACCGTCGAACTGCAGTTGATACTGGAGGTCTTGAGTGCACACAGGGATACTGGAATTCATGGTGTAGCGGTGAAATGCTCAGATATCATGAAGAACTCCAATCGCGAAGGCAGGTATCCGGGGTGCAACTGACGCTGAGGCTCGAAAGTGCGGGTATCAAAC"
##  [31] "ACAAGCGTTGTCCGGAATTACTGGGTGTAAAGGGAGCGCAGGCGGGAAGGCAAGTTGGAAGTGAAATCCATGGGCTCAACCCATGAACTGCTTTCAAAACTGTTTTTCTTGAGTAGTGCAGAGGTAGGCGGAATTCCCGGTGTAGCGGTGGAATGCGTAGATATCGGGAGGAACACCAGTGGCGAAGGCGGCCTACTGGGCACCAACTGACGCTGAGGCTCGAAAGTGTGGGTAGCAAAC"
##  [32] "CCAGGCGTTATCCGGATTTATTGGGTTTAAAGGGAGCGTAGGCCGCCCCTTAAGCGTGTTGTGAAATGCCGCGGCTCAACCGTGGCACTGCAGCGCGAACTGGGGGGCTTGAGTGCACGCAACGCAGGCGGAATTCGTGGTGTAGCGGTGAAATGCTTAGATATCACGAAGAACTCCGATTGCGAAGGCAGCTTGCGGGAGTGCGACTGACGCTGAAGCTCGAAGGTGCGGGTATCGAAC"
##  [33] "CCGGGCGTTATCCGGATTTATTGGGTTTAAAGGGAGCGTAGGCCGGAGATTAAGCGTGTTGTGAAATGTAGATGCTCAACATCTGCACTGCAGCGCGAACTGGTCTTCTTGAGTACGCACAACGTGGGCGGAATTCGTGGTGTAGCGGTGAAATGCTTAGATATCACGAAGAACTCCGATTGCGAAGGCAGCTCACGGGAGCGCAACTGACGCTGAAGCTCGAAAGTGCGGGTATCGAAC"
##  [34] "GCGAGCGTTGTCCGGAATGACTGGGTGTAAAGGGAGCGTAGGCGGGATGGCAAGTCAGATGTGAAACCTGAGGGCTCAACCTTCAGACTGCATTTGAAACTGCTGTTCTTGAGTGAAGTAGAGGTAAGCGGAATTCCTGGTGTAGCGGTGAAATGCGTAGAGATCAGGAGGAACATCGGTGGCGAAGGCGGCTTACTGGGCTTTTACTGACGCTGAGGCTCGAAAGCGTGGGGAGCAAAC"
##  [35] "CCGGGCGTTATCCGGATTTATTGGGTTTAAAGGGAGCGTAGGCCGGAGATTAAGCGTGTTGTGAAATGTAGACGCTCAACGTCTGCACTGCAGCGCGAACTGGTTTCCTTGAGTACGCACAAAGTGGGCGGAATTCGTGGTGTAGCGGTGAAATGCTTAGATATCACGAAGAACTCCGATTGCGAAGGCAGCTTGCGGGAGTGCGACTGACGCTGAAGCTCGAAGGTGCGGGTATCGAAC"
##  [36] "CCGGGCGTTATCCGGATTTATTGGGTTTAAAGGGAGCGCAGGCCGTGGGTTAAGCGTGTCGTGAAATTCCGTCGCTCAACGGCGGACGTGCGGCGCGAACTGGTCCACTTGAGTACGCGGGACGTTGGCGGAATTCGTGGTGTAGCGGTGAAATGCTTAGATATCACGAAGAACTCCGATTGCGAAGGCAGCTGACGGTAGCGCAACTGACGCTGAGGCTCGAAAGTGCGGGTATCGAAC"
##  [37] "CCGGGCGTTATCCGGATTTATTGGGTTTAAAGGGAGCGTAGGCCGTTTGGTAAGCGTGTTGTGAAATGTCGGGGCTCAACCTGGGCATTGCAGCGCGAACTGCCAGACTTGAGTGCGCAGGAAGTAGGCGGAATTCGTCGTGTAGCGGTGAAATGCTTAGATATGACGAAGAACTCCGATTGCGAAGGCAGCCTGCTGTAGCGCAACTGACGCTGAAGCTCGAAAGCGTGGGTATCGAAC"
##  [38] "CCGGGCGTTATCCGGATTTATTGGGTTTAAAGGGAGCGTAGGCCGTCTGGTAAGCGTGTTGTGAAATGTCGGGGCTCAACCTGGGCATTGCAGCGCGAACTGTCAGACTTGAGTGCGCGGGAAGTAGGCGGAATTCGTCGTGTAGCGGTGAAATGCTTAGATATGACGAAGAACTCCGATTGCGAAGGCAGCCTGCTGTAGCGCAACTGACGCTGAAGCTCGAAAGCGTGGGTATCGAAC"
##  [39] "CCGGGCGTTATCCGGATTTATTGGGTTTAAAGGGAGCGTAGGCCGCCCCTTAAGCGTGTTGTGAAATGCCGCGGCTCAACCGTGGCACTGCAGCGCGAACTGGGGGGCTTGAGTGCACGCAACGCAGGCGGAATTCGTGGTGTAGCGGTGAAATGCTTAGATATCACGAAGAACTCCGATTGCGAAGGCAGCTCACTGGAGCGCAACTGACGCTGAAGCTCGAAAGTGCGGGTATCGAAC"
##  [40] "GCAAGCGTTATCCGGATTTACTGGGTGTAAAGGGAGTGTAGGTGGCCATGCAAGTCAGAAGTGAAAATCCGGGGCTCAACCCCGGAACTGCTTTTGAAACTGTAAGGCTGGAGTGCAGGAGGGGTGAGTGGAATTCCTAGTGTAGCGGTGAAATGCGTAGATATTAGGAGGAACACCAGTGGCGAAGGCGGCTCACTGGACTGTAACTGACACTGAGGCTCGAAAGCGTGGGGAGCAAAC"
##  [41] "CCGGGCGTTATCCGGATTTATTGGGTTTAAAGGGAGCGTAGGCCGGAGATTAAGCGTGTTGTGAAATGTAGTTGCTCAACATCTGCACTGCAGCGCGAACTGGTTTCCTTGAGTACGCACAAAGTGGGCGGAATTCGTGGTGTAGCGGTGAAATGCTTAGATATCACGAAGAACTCCGATTGCGAAGGCAGCTCACTGGAGCGCAACTGACGCTGAAGCTCGAAAGTGCGGGTATCGAAC"
##  [42] "CCGGGCGTTATCCGGATTTATTGGGTTTAAAGGGAGCGTAGGCTGTCCTTTAAGCGTGTTGTGAAATGTAGGCGCTCAACGCCTGCACTGCAGCGCGAACTGAAGGACTTGAGTGTGCACGACGCTGGCGGAATTCGTGGTGTAGCGGTGAAATGCTTAGATATCACGAAGAACTCCGATTGCGAAGGCAGCTGGCGAGAGCACAACTGACGCTGAAGCTCGAAAGTGCGGGTATCGAAC"
##  [43] "ACAAGCGTTGTCCGGAATTACTGGGTGTAAAGGGAGCGCAGGCGGGAGAACAAGTTGGAAGTGAAATCCATGGGCTCAACCCATGAACTGCTTTCAAAACTGTTTTTCTTGAGTAGTGCAGAGGTAGGCGGAATTCCCGGTGTAGCGGTGGAATGCGTAGATATCGGGAGGAACACCAGTGGCGAAGGCGGCCTACTGGGCACCAACTGACGCTGAGGCTCGAAAGTGTGGGTAGCAAAC"
##  [44] "GCAAGCGTTATCCGGATTTACTGGGCGTAAAGGGAGCGTAGGCGGATATTTAAGTGGGATGTGAAATACCCGAGCTTAACTTGGGAGCTGCATTCCAAACTGGATATCTAGAGTGCAGGAGAGGAGAATGGAATTCCTAGTGTAGCGGTGAAATGCGTAGAGATTAGGAAGAACACCAGTGGCGAAGGCGATTCTCTGGACTGTAACTGACGCTGAGGCTCGAAAGCGTGGGGAGCAAAC"
##  [45] "CCGGGCGTTATCCGGATTTATTGGGTTTAAAGGGAGCGCAGGCCGTGGGCTAAGCGTGCCGTGAAATTCTGTCGCTCAACGGCAGACGTGCGGCGCGAACTGGTCCACTTGAGTACGCGGGACGTTGGCGGAATTCGTGGTGTAGCGGTGAAATGCTTAGATATCACGAAGAACTCCGATTGCGAAGGCAGCTCACTGGAGCGCAACTGACGCTGAAGCTCGAAAGTGCGGGTATCGAAC"
##  [46] "GCAAGCGTTATCCGGATTTACTGGGTGTAAAGGGCGCGCAGGCGGGCCGGCAAGTTGGAAGTGAAATCCGGGGGCTTAACCCCCGAACTGCTTTCAAAACTGCTGGTCTTGAGTGATGGAGAGGCAGGCGGAATTCCGTGTGTAGCGGTGAAATGCGTAGATATACGGAGGAACACCAGTGGCGAAGGCGGCCTGCTGGACATTAACTGACGCTGAGGCGCGAAAGCGTGGGGAGCAAAC"
##  [47] "CCGGGCGTTATCCGGATTTATTGGGTTTAAAGGGAGCGTAGGCCGGAGATTAAGCGTGTTGTGAAATGTAGACGCTCAACGTCTGCACTGCAGCGCGAACTGGTTTCCTTGAGTACGCATAAAGTGGGCGGAATTCGTGGTGTAGCGGTGAAATGCTTAGATATCACGAAGAACTCCGATTGCGAAGGCAGCTCACTGGAGCGCAACTGACGCTGAAGCTCGAAAGTGCGGGTATCGAAC"
##  [48] "CCGGGCGTTATCCGGATTTATTGGGTTTAAAGGGAGCGTAGGCCGGAGATTAAGCGTGTTGTGAAATGTAGACGCTCAACGTCTGCACTGCAGCGCGAACTGGTTTCCTTGAGTACGCACAAAGTGGGCGGAATTCGTGGTGTAGCGGTGAAATGCTTAGATATCACGAAGAACTCCGATTGCGAAGGCAGCTGACGGTAGCGCAACTGACGCTGAGGCTCGAAAGCGCGGGTATCGAAC"
##  [49] "CCGGGCGTTATCCGGATTTATTGGGTTTAAAGGGAGCGTAGGCCGTCTTATAAGCGTGTTGTGAAATGTCGGGGCTCAACCTGGGCATTGCAGCGCGAACTGTGAGACTTGAGTGCGCAGGAAGTAGGCGGAATTCGTCGTGTAGCGGTGAAATGCTTAGATATCACGAAGAACTCCGATTGCGAAGGCAGCTCACTGGAGCGCAACTGACGCTGAAGCTCGAAAGTGCGGGTATCGAAC"
##  [50] "CCGGGCGTTATCCGGATTTATTGGGTTTAAAGGGAGCGTAGGCCGTCTGTTAAGCGTGTTGTGAAATGTCGGGGCTCAACCTGGGCATTGCAGCGCGAACTGGCAGACTTGAGTGCGCAGGAAGTAGGCGGAATTCGTCGTGTAGCGGTGAAATGCTTAGATATGACGAAGAACTCCGATTGCGAAGGCAGCCTGCTGTAGCGTAACTGACGCTGAAGCTCGAAAGCGTGGGTATCGAAC"
##  [51] "CCGGGCGTTATCCGGATTTATTGGGTTTAAAGGGAGCGTAGGCCGGAGATTAAGCGTGTTGTGAAATGTAGATGCTCAACATCTGAACTGCAGCGCGAACTGGTTTCCTTGAGTACGCATAAAGTGGGCGGAATTCGTGGTGTAGCGGTGAAATGCTTAGATATCACGAAGAACTCCGATTGCGAAGGCAGCTCACTGGAGCGCAACTGACGCTGAAGCTCGAAAGTGCGGGTATCGAAC"
##  [52] "CCAGGCGTTATCCGGATTTATTGGGTTTAAAGGGAGCGTAGGCCGCCCCTTAAGCGTGTTGTGAAATGCCGCGGCTCAACCGTGGCACTGCAGCGCGAACTGGGGGGCTTGAGTGCACGCAACGCAGGCGGAATTCGTGGTGTAGCGGTGAAATGCTTAGATATCACGAAGAACTCCGATTGCGAAGGCAGCTCACTGGAGCGCAACTGACGCTGAAGCTCGAAAGTGCGGGTATCGAAC"
##  [53] "GCAAGCGTTATCCGGATTTACTGGGTGTAAAGGGCGAGTAGGCGGATTGGCAAGTTGGGAGTGAAATGTCGGGGCTTAACCCCGGAACTGCTTCCAAAACTGTTGATCTTGAGTGATGGAGAGGCAGGCGGAATTCCCAGTGTAGCGGTGAAATGCGTAGATATTGGGAGGAACACCAGTGGCGAAGGCGGCCTGCTGGACATTAACTGACGCTGAGGAGCGAAAGCGTGGGGAGCAAAC"
##  [54] "GCAAGCGTTAATCGGAATAACTGGGCGTAAAGCGCACGTAGGTGGTTCGACAAGTCAGATGTGAAAGCCCCGGGCTTAACCTGGGATGTGCATTTGAAACTGTTGGACTCGAGTACTGTAGAGGGAGGTAGAATTCCAGGTGTAGCGGTGAAATGCGTAGATATCTGGAGGAATACCAGTGGCGAAGGCGGCCTCCTGGACAGACACTGACACTGAGGTGCGAAAGCGTGGGGAGCAAAC"
##  [55] "ACAAGCGTTGTCCGGAATTACTGGGTGTAAAGGGAGCGCAGGCGGGCGATCAAGTTGGAAGTGAAATCCATGGGCTCAACCCATGAACTGCTTTCAAAACTGGTCGTCTTGAGTAGTGCAGAGGTAGGCGGAATTCCCGGTGTAGCGGTGGAATGCGTAGATATCGGGAGGAACACCAGTGGCGAAGGCGGCCTACTGGGCACCAACTGACGCTGAGGCTCGAAAGTGTGGGTAGCAAAC"
##  [56] "GCGAGCGTTATCCGGATTTATTGGGTTTAAAGGGAGCGTAGGCGGGCTGTTAAGTCAGCGGTCAAATGTCAGGGCCCAACCTTGGCATGCCGTTGATACTGGCGGCCTTGAGTTCACACAAGGAAGGTGGAATTCGTCGTGTAGCGGTGAAATGCTTAGATATGACGAAGAACTCCGATTGCGAAGGCAGCCTTCTGGGGTGTTACTGACGCTGAGGCTCGAAAGTGCGGGAATCAAACA"
##  [57] "GCAAGCGTTGTCCGGAATTACTGGGTGTAAAGGGAGCGCAGGCGGAAGGACAAGTTGGAAGTGAAACCCACGGGCTCAACCCGTGAACTGCTTTCAAAACTGTTTTTCTTGAGTGGTGTAGAGGTAGGCGGAATTCCCGGTGTAGCGGTGGAATGCGTAGATATCGGGAGGAACACCAGTGGCGAAGGCGGCCTACTGGGCACTAACTGACGCTGAGGCTCGAAAGCATGGGTAGCAAAC"
##  [58] "CCGGGCGTTATCCGGATTTATTGGGTTTAAAGGGAGCGTAGGCTGTCCTTTAAGCGTGTTGTGAAATGTAGGCGCTCAACGCCTGCACTGCAGCGCGAACTGAAGGACTTGAGTGTGCACGACGCTGGCGGAATTCGTGGTGTAGCGGTGAAATGCTTAGATATCACGAAGAACTCCGATTGCGAAGGCAGCTCACTGGAGCGCAACTGACGCTGAAGCTCGAAAGTGCGGGTATCGAAC"
##  [59] "CCGGGCGTTATCCGGATTTATTGGGTTTAAAGGGAGCGCAGGCCGTGGGTTAAGCGTGTCGTGAAATTCCGTCGCTCAACGGCGGACGTGCGGCGCGAACTGGTCCACTTGAGTACGCGGGACGTTGGCGGAATTCGTGGTGTAGCGGTGAAATGCTTAGATATCACGAAGAACTCCGATTGCGAAGGCAGCTCACTGGAGCGCAACTGACGCTGAAGCTCGAAAGTGCGGGTATCGAAC"
##  [60] "GCAAGCGTTATCCGGATTTACTGGGTGTAAAGGGAGCGTAGACGGCGAGACAAGTCTGAAGTGAAAGCCCGGGGCTCAACCCCGGGACTGCTTTGGAAACTGCCTTGCTAGAGTGCTGGAGAGGTAAGTGGAATTCCTAGTGTAGCGGTGAAATGCGTAGATATTAGGAGGAACACCAGTGGCGAAGGCGGCTTACTGGACAGTAACTGACGTTGAGGCTCGAAAGCGTGGGGAGCAAAC"
##  [61] "GCGAGCGTTATCCGGATTTATTGGGTTTAAAGGGTGCGTAGGTCGCCGATTAAGTCAGCGGTGAAATCCAGTGGCTCAACCATCGGACTGCCGTTGAAACTGGCCGGCTTGAGTATGATTGAGGCAGGCGGAATGCGTGGTGTAGCGGTGAAATGCATAGATATCACGCAGAACCCCGATTGCGAAGGCAGCTTGCCAAGCCATGACTGACACTGAAGCACGAAAGCGTGGGTATCAAAC"
##  [62] "GCAAGCGTTATCCGGATTTACTGGGTGTAAAGGGAGCGTAGACGGCACAGCAAGTCTGATGTGAAAGCCCGGGGCCCAACCCCGGAACTGCATTGGAAACTGCTGGGCTTGAGTGCAGGAGAGGTAAGCGGAATTCCTAGTGTAGCGGTGAAATGCGTAGATATTAGGAGGAACACCAGTGGCGAAGGCGGCTTACTGGACTGTAACTGACGTTGAGGCTCGAAAGCGTGGGGAGCAAAC"
##  [63] "GCAAGCGTTATCCGGATTTACTGGGTGTAAAGGGAGCGTAGACGGCATGGCAAGTCTGATGTGAAAATCCCGGGCTCAACCCGGGAACTGCATTGGAAACTGTTAAGCTAGAGTGCAGGAGAGGTAAGTGGAATTCCTAGTGTAGCGGTGAAATGCGTAGATATTAGGAGGAACACCAGTGGCGAAGGCGGCTTACTGGACTGTAACTGACGTTGAGGCTCGAAAGCGTGGGGAGCAAAC"
##  [64] "GCAAGCGTTATCCGGATTTACTGGGTGTAAAGGGAGCGTAGACGGTAAAGCAAGTCTGAAGTGAAAGCCCGGGGCTCAACCGCGGGACTGCTTTGGAAACTGTTTAACTAGAGTGCTGGAGAGGTAAGCGGAATTCCTAGTGTAGCGGTGAAATGCGTAGATATTAGGAGGAACACCAGTGGCGAAGGCGGCTTACTGGACAGTAACTGACGTTGAGGCTCGAAAGCGTGGGGAGCAAAC"
##  [65] "CCGGGCGTTATCCGGATTTATTGGGTTTAAAGGGAGCGTAGGCCGTCTGTTAAGCGTGTTGTGAAATGTCGTGGCTCAACCGGGGCACTGCAGCGCGAACTGGCAGACTTGAGTGCACGGTAGGAAGGCGGAATTCGTCGTGTAGCGGTGAAATGCTTAGATATGACGAAGAACTCCGATTGCGAAGGCAGCTTTCCGTAGTGTAACTGACGCTGAAGCTCGAAAGCGTGGGTATCGAAC"
##  [66] "GCGAGCGTTGTCCGGAATGACTGGGCGTAAAGGGCGTGTAGGCGGCAGTATAAGTCCGGAGTGAAAGTCCTGCTTTCAAGGTGGGAATTGCTTTGGAGACTGTACAGCTTGAGTGCGGAAGAGGTAAGTGGAATTCCCAGTGTAGCGGTGAAATGCGTAGAGATTGGGAGGAACACCAGTGGCGAAGGCGACTTACTGGGCCGTAACTGACGCTGAGGCGCGAAAGCGTGGGGAGCGAAC"
##  [67] "GCAAGCGTTGTCCGGATTTACTGGGTGTAAAGGGCGTGTAGGCGGGCATGCAAGTTGGATGTGAAATGTCACGGCTTAACCGTGGAGCTGCATCCAAAACTGCAAGTCTTGAGTGCCGGAGAGGAAAGCGGAATTCCTAGTGTAGCGGTGAAATGCGTAGATATTAGGAGGAACACCGGTGGCGAAGGCGGCTTTCTGGACGGTAACTGACGCTGAGGCGCGAAAGCGTGGGGAGCAAAC"
##  [68] "GCAAGCGTTATCCGGATTTACTGGGTGTAAAGGGAGCGTAGACGGCGATGCAAGTCTGAAGTGAAATACCCGGGCTCAACCTGGGAACTGCTTTGGAAACTGTATTGCTAGAGTGCTGGAGAGGTAAGCGGAATTCCTAGTGTAGCGGTGAAATGCGTAGATATTAGGAAGAACACCAGTGGCGAAGGCGGCTTACTGGACAGTAACTGACGTTGAGGCTCGAAAGCGTGGGGAGCAAAC"
##  [69] "CCAGGCGTTATCCGGATTTATTGGGTTTAAAGGGAGCGTAGGCCGTTTTTTAAGCGTGTTGTGAAATACTGTCGCTCAACGACAGAGGTGCAGCGCGAACTGGAGGACTTGAGTGCGCGGAATGTAGGCGGAATTCGTCGTGTAGCGGTGAAATGCTTAGATATGACGAAGAACTCCGATTGCGAAGGCAGCTTACAGTAGCGTAACTGACGCTGAAGCTCGAAAGTGCGGGTATCGAAC"
##  [70] "GCAAGCGTTATCCGGATTTACTGGGTGTAAAGGGAGTGTAGGTGGCCAGGCAAGTCAGAAGTGAAAGCCCGGGGCTCAACCCCGGGACTGCTTTTGAAACTGCAGGGCTAGAGTGCAGGAGGGGCAAGTGGAATTCCTAGTGTAGCGGTGAAATGCGTAGATATTAGGAGGAACACCAGTGGCGAAGGCGGCTTGCTGGACTGTAACTGACACTGAGGCTCGAAAGCGTGGGGAGCAAAC"
##  [71] "GCGAGCGTTATCCGGATTTATTGGGTTTAAAGGGAGCGCAGACGGGACTTTAAGTCAGCTGTGAAATTTTCCGGCTCAACCGGGAAACTGCAGTTGATACTGGCGTCCTTGAGTACGGTCGAGGCAGGCGGAATTCGTGGTGTAGCGGTGAAATGCTTAGATATCACGAAGAACCCCGATTGCGAAGGCAGCCTGCCAGACCGCAACTGACGTTCATGCTCGAAAGTGCGGGTATCAAAC"
##  [72] "GCAAGCGTTAATCGGAATCACTGGGCGTAAAGCGCACGTAGGCTGTTATGTAAGTCAGGGGTGAAATCCCACGGCTCAACCGTGGAACTGCCCTTGATACTGCACGACTTGAATCCGGGAGAGGGTGGCGGAATTCCAGGTGTAGGAGTGAAATCCGTAGATATCTGGAGGAACATCAGTGGCGAAGGCGGCCACCTGGACCGGTATTGACGCTGAGGTGCGAAAGCGTGGGGAGCAAAC"
##  [73] "CCAGGCGTTATCCGGATTTATTGGGTTTAAAGGGAGCGTAGGCCGTTTGGTAAGCGTGTTGTGAAATGTCGGGGCTCAACCTGGGCATTGCAGCGCGAACTGCCAGACTTGAGTGCGCAGGAAGTAGGCGGAATTCGTCGTGTAGCGGTGAAATGCTTAGATATGACGAAGAACTCCGATTGCGAAGGCAGCCTGCTGTAGCGCAACTGACGCTGAAGCTCGAAAGCGTGGGTATCGAAC"
##  [74] "GCAAGCGTTGTCCGGAATCATTGGGCGTAAAGAGTTCGTAGGCGGTTTGTTAAGTCTGGTGTTAAAGCCCGAAGCTCAACTTCGGTTCGGCACTGGATACTGGCAGACTAGAATGCGGTAGAGGTAAAGGGAATTCCTGGTGTAGCGGTGAAATGCGTAGATATCAGGAGGAACATCGGTGGCGTAAGCGCTTTACTGGGCCGTAATTGACGCTGAGGAACGAAAGCCAGGGTAGCGAAT"
##  [75] "GCAAGCGTTATCCGGATTTACTGGGTGTAAAGGGAGCGTAGACGGTTAAGCAAGTCAGAAGTGAAAGGCTGGGGCTCAACCCCGGGACTGCTTTTGAAACTGTTTAACTAGAGTGCTGGAGAGGTAAGCGGAATTCCTAGTGTAGCGGTGAAATGCGTAGATATTAGGAGGAACACCAGTGGCGAAGGCGGCTTACTGGACAGTAACTGACGTTGAGGCTCGAAAGCGTGGGGAGCAAAC"
##  [76] "GCAAGCGTTATCCGGATTTACTGGGTGTAAAGGGCGTGTAGGCGGGAAAGCAAGTCAGATGTGAAAACTGTGGGCTCAACCCACAGCCTGCATTTGAAACTGTTTTTCTTGAGTACTGGAGAGGCAGATGGAATTCCTAGTGTAGCGGTGAAATGCGTAGATATTAGGAGGAACACCAGTGGCGAAGGCGATCTGCTGGACAGCAACTGACGCTGAGGCGCGAAAGCGTGGGGAGCAAAC"
##  [77] "GCAAGCGTTGTCCGGAATTACTGGGTGTAAAGGGAGCGCAGGCGGGCATGCAAGTTGGAAGTGAAAACTATGGGCTCAACCCATAGCCTGCTTTCAAAACTGCGTGTCTTGAGTAGTGCAGAGGTAGGCGGAATTCCCGGTGTAGCGGTGGAATGCGTAGATATCGGGAGGAACACCAGTGGCGAAGGCGGCCTACTGGGCACCAACTGACGCTGAGGCTCGAAAGCATGGGTAGCAAAC"
##  [78] "GCGAGCGTTATCCGGAATTACTGGGTGTAAAGGGTGTGTAGGCGGGAAGGCAAGTCAGATGTGAAAACCAAAGGCTCAACCTTTGGCTTGCATTTGAAACTGTTTTTCTTGAGAGTGGGAGAGGTAAACGGAATTCCTAGTGTAGTAGTGAAATGCGTAGATATTAGGAGGAACACCGGTGGCGAAGGCGGTTTACTGGACCACAACTGACGCTGAGACACGAAAGCGTGGGGAGCAAAC"
##  [79] "GCAAGCGTTATCCGGAATTACTGGGTGTAAAGGGAGCGTAGGCGGCATGGTAAGTAAGATGTGAAAGCCCGAGGCTTAACCTCGAGGATTGCATTTTAAACTATCAAGCTAGAGTACAGGAGAGGAAAGCGGAATTCCTAGTGTAGCGGTGAAATGCGTAGATATTAGGAAGAACACCAGTGGCGAAGGCGGCTTTCTGGACTGAAACTGACGCTGAGGCTCGAAAGCGTGGGGAGCGAA"
##  [80] "GCAAGCGTTATCCGGATTTACTGGGTGTAAAGGGAGCGTAGACGGTCAAGCAAGTCAGAAGTGAAAGGCTGGGGCTCAACCCCGGGACTGCTTTTGAAACTGTTTGACTGGAGTGCTGGAGAGGTAAGCGGAATTCCTAGTGTAGCGGTGAAATGCGTAGATATTAGGAGGAACACCAGTGGCGAAGGCGGCTTACTGGACAGTAACTGACGTTGAGGCTCGAAAGCGTGGGGAGCAAAC"
##  [81] "GCAAGCGTTATCCGGATTTACTGGGTGTAAAGGGAGCGTAGACGGCGACGCAAGTCTGAAGTGAAATACCCGGGCTCAACCTGGGAACTGCTTTGGAAACTGTGTTGCTAGAGTGCTGGAGAGGTAAGCGGAATTCCTAGTGTAGCGGTGAAATGCGTAGATATTAGGAAGAACACCAGTGGCGAAGGCGGCTTACTGGACAGTAACTGACGTTGAGGCTCGAAAGCGTGGGGAGCAAAC"
##  [82] "GCAAGCGTTGTCCGGATTTACTGGGTGTAAAGGGAGCGCAGGCGGGAGAGCAAGTCAGCGGTGAAATACATGGGCTTAACCCATGGGCTGCCGTTGAAACTGTCCTTCTTGAGTGAAGTAGAGGCAAGCGGAATTCCGAGTGTAGCGGTGAAATGCGTAGATATTCGGAGGAACACCAGTGGCGAAGGCGGCTTGCTGGGCTTTTACTGACGCTGAGGCTCGAAAGTGTGGGGAGCAAAC"
##  [83] "GCAAGCGTTATCCGGATTTACTGGGTGTAAAGGGAGCGCAGGCGGTCTGGCAAGTCTGATGTGAAATCCCGGGGCTCAACCCTGGAACTGCATTGGAAACTGTCAGACTAGAGTGCCGGAGAGGTAAGTGGAATTCCTAGTGTAGCGGTGAAATGCGTAGATATTAGGAGGAACACCAGTGGCGAAGGCGGCTTACTGGACGGTAACTGACGCTGAGGCTCGAAAGCGTGGGGAGCAAAC"
##  [84] "GCAAGCGTTATCCGGATTTACTGGGTGTAAAGGGAGCGTAGACGGCTAAGCAAGTCAGAAGTGAAAGGCTGGGGCTCAACCCCGGGACTGCTTTTGAAACTGTTTGGCTAGAGTGCTGGAGAGGTAAGCGGAATTCCTAGTGTAGCGGTGAAATGCGTAGATATTAGGAGGAACACCAGTGGCGAAGGCGGCTTACTGGACAGTAACTGACGTTGAGGCTCGAAAGCGTGGGGAGCAAAC"
##  [85] "GCAAGCGTTACTCGGAATTACTAGGCGTAAAGCGCGCGTAGGCGGAATGTTAAGTCTGTTGTGTAATCTCTGGGCTCAACCCAGAAACTGCAACAGAAACTGGCGTTCTTGAGTGAGGCAGAGGAAATCGGAATTCCTAGTGTAGCAGTGAAATGCGTAGATATTAGGAGGAACACCGGTGGCGAAGGCGGATTTCTGGGCCTTTACTGACGCTAAAGTGCGAAAGCTAGGGGAGCAAAC"
##  [86] "GCGAGCGTTATCCGGATTCATTGGGCGTAAAGCGCGCGTAGGCGGATGCCTAAGCGGGACCTCTAACCCGGGGGCTCAACCCCCGGCCGGGTCCCGAACTGGGCGTCTCGAGTGCGGTAGGGGCAGGTGGAATTCCATGTGTAGCGGTGGAATGCGCAGATATATGGAAGAACACCGACGGCGAAGGCAGCCTGCTGGGCCGACACTGACGCTGAGGTGCGAAAGCGCGGGGAGCGAACA"
##  [87] "GCAAGCGTTATCCGGATTTACTGGGTGTAAAGGGAGCGTAGACGGTAGTGCAAGTCTGATGTGAAAGCCCGGGGCTCAACCCCGGGACTGCATTGGAAACTGTATAACTAGAGTGTCGGAGAGGTAAGCGGAATTCCTAGTGTAGCGGTGAAATGCGTAGATATTAGGAGGAACACCAGTGGCGAAGGCGGCTTACTGGACGATGACTGACGTTGAGGCTCGAAAGCGTGGGGAGCAAAC"
##  [88] "GCAAGCGTTGTCCGGAATTACTGGGTGTAAAGGGAGCGTAGGCGGGAAGATAAGTTGGACGTCTAATCTATCGGCTCAACCGATAGTCGCGTTCAAAACTGTTTTTCTTGAGTGAAGTAGAGGTAAGCGGAATTCCTAGTGTAGCGGTGAAATGCGTAAATATTAGGAGGAACACCAGTGGCGAAGGCGGCTTACTGGGCTTTAACTGACGCTGAGGCTCGAAAGCGTGGGTAGCAAACA"
##  [89] "GCGAGCGTTATCCGGATTTATTGGGTGTAAAGGGTGCGTAGACGGGAAATTAAGTTAGTTGTGAAATCCCTCGGCTCAACTGAGGAACTGCAACTAAAACTGATTTTCTTGAGTACTGGAGAGGAAAGTGGAATTCCTAGTGTAGCGGTGAAATGCGTAGATATTAGGAGGAACACCAGTGGCGAAGGCGACTTTCTGGACAGAAACTGACGTTGAGGCACGAAAGTGTGGGGAGCAAAC"
##  [90] "GCGAGCGTTGTCCGGAATTATTGGGCGTAAAGAGCTTGTAGGCGGTTTGTCGCGTCTGCTGTGAAAGGCCGGGGCTTAACCCCGTGTATTGCAGTGGGTACGGGCAGACTAGAGTGCAGTAGGGGAGACTGGAATTCCTGGTGTAGCGGTGGAATGCGCAGATATCAGGAGGAACACCGATGGCGAAGGCAGGTCTCTGGGCTGTAACTGACGCTGAGAAGCGAAAGCATGGGGAGCGAA"
##  [91] "GCAAGCGTTATCCGGATTTATTGGGTGTAAAGGGTGCGTAGACGGGAATACAAGTTAGTTGTGAAATCCCTCGGCTTAACTGAGGAACTGCAACTAAAACTATATTTCTTGAGTGCTGGAGAGGAAAGTGGAATTCCTAGTGTAGCGGTGAAATGCGTAGATATTAGGAGGAACACCAGTGGCGAAGGCGACTTTCTGGACAGTAACTGACGTTGAGGCACGAAAGTGTGGGGAGCAAAC"
##  [92] "CCGAGCGTTATCCGGATTTATTGGGTTTAAAGGGAGCGTAGGTGGATTGTTAAGTCAGTTGTGAAAGTTTGCGGCTCAACCGTAAAATTGCAGTTGAAACTGGCAGTCTTGAGTACAGTAGAGGTGGGCGGAATTCGTGGTGTAGCGGTGAAATGCTTAGATATCACGAAGAACTCCGATTGCGAAGGCAGCTCACTAGACTGCAACTGACACTGATGCTCGAAAGTGTGGGTATCAAAC"
##  [93] "GCAAGCGTTATCCGGATTTACTGGGTGTAAAGGGAGCGTAGGCGGTATGGCAAGTCTGATGTGAAAGGCCGGGGCTCAACCCCGGGACTGCATTGGAAACTGTCACACTTGAGTGTCGGAGAGGTAAGTGGAATTCCTAGTGTAGCGGTGAAATGCGTAGATATTAGGAGGAACACCAGTGGCGAAGGCGGCTTACTGGACGACAACTGACGCTGAGGCTCGAAAGCGTGGGGAGCAAAC"
##  [94] "GCAAGCGTTATCCGGATTTATTGGGTGTAAAGGGTGCGTAGACGGGAAATTAAGTTAGTTGTGAAATCCCTCGGCTCAACTGAGGAACTGCAACTAAAACTGGTTTTCTTGAGTGCAGGAGAGGTAAGTGGAATTCCTAGTGTAGCGGTGAAATGCGTAGATATTAGGAGGAACACCAGTGGCGAAGGCGACTTACTGGACTGTAACTGACGTTGAGGCACGAAAGTGTGGGGAGCAAAC"
##  [95] "GCAAGCGTTGTCCGGAATTACTGGGTGTAAAGGGAGCGCAGGCGGGAAGACAAGTTGGAAGTGAAAACCATGGGCTCAACCCATGAATTGCTTTCAAAACTGCTGGCCTTGAGTAGTGCAGAGGTAGGTGGAATTCCCGGTGTAGCGGTGGAATGCGTAGATATCGGGAGGAACACCAGTGGCGAAGGCGGTCTACTGGGCACCAACTGACGCTGAGGCTCGAAAGCATGGGTAGCAAAC"
##  [96] "GCAAGCGTTATCCGGATTTACTGGGTGTAAAGGGAGCGTAGACGGCTGTGTAAGTCTGAAGTGAAAGCCCGGGGCTCAACCGCGGGACTGCTTTGGAAACTATGCAGCTAGAGTGTCGGAGAGGTAAGTGGAATTCCCAGTGTAGCGGTGAAATGCGTAGATATTGGGAGGAACACCAGTGGCGAAGGCGGCTTACTGGACGATGACTGACGTTGAGGCTCGAAAGCGTGGGGAGCAAAC"
##  [97] "GCGAGCGTTATCCGGATTCATTGGGCGTAAAGCGCGCGTAGGCGGCCGCTCGAGCGGGACCTCTAACCCGGGGGCTCAACCTCCGGCCGGGTCCCGGACCGTGCGGCTCGGGTGCGGTAGGGGCAGGCGGAACTCCAAGTGTAGCGGTGAAATGCGCAGATATTTGGAGGAACACCGATGGCGAAGGCAGCCTGCTGGGCCGCCACCGACGCTGAGGCGCGAAAGCCGGGGGAGCGAACA"
##  [98] "GCAAGCGTTATCCGGAATTACTGGGTGTAAAGGGTGCGTAGGTGGTATGGCAAGTCAGAAGTGAAAACCCAGGGCTTAACTCTGGGACTGCTTTTGAAACTGTCAGACTAGAGTGCAGGAGAGGTAAGCGGAATTCCTAGTGTAGCGGTGAAATGCGTAGATATTAGGAGGAACATCAGTGGCGAAGGCGGCTTACTGGACTGAAACTGACACTGAGGCACGAAAGCGTGGGGAGCAAAC"
##  [99] "GCAAGCGTTATCCGGATTTACTGGGTGTAAAGGGAGCGTAGACGGTGTGGCAAGTCTGATGTGAAAGGCATGGGCTCAACCTGTGGACTGCATTGGAAACTGTCATACTTGAGTGCCGGAGGGGTAAGCGGAATTCCTAGTGTAGCGGTGAAATGCGTAGATATTAGGAGGAACACCAGTGGCGAAGGCGGCTTACTGGACGGTAACTGACGTTGAGGCTCGAAAGCGTGGGGAGCAAAC"
## [100] "GCAAGCGTTATCCGGAATTATTGGGCGTAAAGGGCTCGTAGGCGGTTCGTCGCGTCCGGTGTGAAAGTCCATCGCTTAACGGTGGATCTGCGCCGGGTACGGGCGGGCTGGAGTGCGGTAGGGGAGACTGGAATTCCCGGTGTAACGGTGGAATGTGTAGATATCGGGAAGAACACCGACGGCGAAGGCAGCTCTCTGGGCCGAAACTGACGCTGAGGCGCGAAAGCTGGGGGAGCGAAC"
## [101] "CCAAGCGTTATCCGGATTTATTGGGCGTAAAGCGAGCGCAGACGGTTATTTAAGTCTGAAGTGAAAGCCCTCAGCTCAACTGAGGAATTGCTTTGGAAACTGGATGACTTGAGTGCAGTAGAGGAAAGTGGAACTCCATGTGTAGCGGTGAAATGCGTAGATATATGGAAGAACACCAGTGGCGAAGGCGGCTTTCTGGACTGTAACTGACGTTGAGGCTCGAAAGTGTGGGTAGCAAAC"
## [102] "GCAAGCGTTATCCGGAATTATTGGGCGTAAAGAGTACGTAGGTGGTTACCTAAGCACGAGGTATAAGGCAATGGCTTAACCATTGTTCGCCTTGTGAACTGGGCTACTTGAGTGCAGGAGAGGAAAGCGGAATTCCTAGTGTAGCGGTGAAATGCGTAGATATTAGGAGGAACACCAGTGGCGAAGGCGGCTTTCTGGACTGTAACTGACACTGAGGTACGAAAGCGTGGGGAGCAAACA"
## [103] "GCAAGCGTTATCCGGATTTACTGGGTGTAAAGGGAGCGTAGACGGCACGGCAAGCCAGATGTGAAAGCCCGGGGCTCAACCCCGGGACTGCATTTGGAACTGCTGAGCTAGAGTGTCGGAGAGGCAAGTGGAATTCCTAGTGTAGCGGTGAAATGCGTAGATATTAGGAGGAACACCAGTGGCGAAGGCGGCTTGCTGGACGATGACTGACGTTGAGGCTCGAAAGCGTGGGGAGCAAAC"
## [104] "GCAAGCGTTATCCGGAATTATTGGGCGTAAAGAGTACGTAGGTGGTTTTCTAAGCACGGGGTTTAAGGCAATGGCTTAACCATTGTTCGCCTTGTGAACTGGAAGACTTGAGTGCAGGAGAGGAAAGCGGAATTCCTAGTGTAGCGGTGAAATGCGTAGATATTAGGAGGAACACCAGTGGCGAAGGCGGCTTTCTGGACTGTAACTGACACTGAGGTACGAAAGCGTGGGGAGCAAACA"
## [105] "GCAAGCGTTATCCGGATTTACTGGGTGTAAAGGGAGCGTAGACGGCTTTGCAAGTCTGATGTGAAAGGCGGGGGCTCAACCCCTGGACTGCATTGGAAACTGTGAGGCTTGAGTGCCGGAGAGGTAAGCGGAATTCCTAGTGTAGCGGTGAAATGCGTAGATATTAGGAGGAACACCAGTGGCGAAGGCGGCTTACTGGACGGTAACTGACGTTGAGGCTCGAAAGCGTGGGGAGCAAAC"
## [106] "GCAAGCGTTGTCCGGAATTACTGGGTGTAAAGGGAGCGCAGGCGGACCGGCAAGTTGGAAGTGAAAACTATGGGCTCAACCCATAAATTGCTTTCAAAACTGCTGGCCTTGAGTAGTGCAGAGGTAGGTGGAATTCCCGGTGTAGCGGTGGAATGCGTAGATATCGGGAGGAACACCAGTGGCGAAGGCGACCTACTGGGCACCAACTGACGCTGAGGCTCGAAAGCATGGGTAGCAAAC"
## [107] "GCAAGCGTTGTCCGGAATTACTGGGTGTAAAGGGAGCGCAGGCGGACCGGCAAGTTGGAAGTGAAAACCATGGGCTCAACCCGTGAATTGCTTTCAAAACTGCTGGCCTTGAGTAGTGCAGAGGTAGGTGGAATTCCCGGTGTAGCGGTGGAATGCGTAGATATCGGGAGGAACACCAGTGGCGAAGGCGACCTACTGGGCACCAACTGACGCTGAGGCTCGAAAGCATGGGTAGCAAAC"
## [108] "GCGAGCGTTATCCGGAATCATTGGGCGTAAAGCGCGCGCAGGCGGGCTTTCAAGCGGCGGCGTCGAAGCCGGGGGCTCAACCCCCGGAAGCGCCCCGAACTGGAAGCCTCGGATGCGGCAGGGGGAGGCGGAATTCCCGGTGTAGCGGTGAAATGCGCAGATATCGGGAAGAACACCGACGGCGAAGGCAGCCTCCTGGGCCGGCATCGACGCTGAGGCGCGAAAGCTGGGGGAGCGAAC"
## [109] "GCGAGCGTTATCCGGATTCATTGGGCGTAAAGCGCGCGTAGGCGGAGCGCTAAGCGGGACCTCTAACCCGAGGGCTCAACCCCCGGCCGGGTCCCGAACTGGCGCTCTCGAGTGCGGTAGGGGAGAGCGGAATTCCCGGTGTAGCGGTGGAATGCGCAGATATCGGGAAGAACACCGACGGCGAAGGCAGCTCTCTGGGCCGAAACTGACGCTGAGGCGCGAAAGCTGGGGGAGCGAACA"
## [110] "GCTAGCGTTATCCGGATTTACTGGGCGTAAAGGGTGCGTAGGCGGTCTTTTAAGTCAGGAGTGAAAGGCTACGGCTCAACCGTAGTAAGCTCTTGAAACTGGAGGACTTGAGTGCAGGAGAGGAGAGTGGAATTCCTAGTGTAGCGGTGAAATGCGTAGATATTAGGAGGAACACCAGTAGCGAAGGCGGCTCTCTGGACTGTAACTGACGCTGAGGCACGAAAGCGTGGGGAGCAAACA"
## [111] "GCAAGCGTTATCCGGAATTACTGGGTGTAAAGGGTGCGTAGGTGGTATGGCAAGTCAGAAGTGAAAACCCAGGGCTTAACTCTGGGACTGCTTTTGAAACTGTCAGACTGGAGTGCAGGAGAGGTAAGCGGAATTCCTAGTGTAGCGGTGAAATGCGTAGATATTAGGAGGAACATCAGTGGCGAAGGCGGCTTACTGGACTGAAACTGACACTGAGGCACGAAAGCGTGGGGAGCAAAC"
## [112] "GCGAGCGTTATCCGGAATGATTGGGCGTAAAGCGCGCGCAGGCGGCCGCTCAAGCGGGACCTCTAACCCCGGGGCTCAACCCCGGGCCGGGTCCCGAACTGGGCGGCTCGAGTGCGGTAGGGGAGAGCGGAATTCCAAGTGTAGCGGTGAAATGCGCAGATATTTGGAAGAACACCGATGGCGAAGGCAGCTCTCTGGGCCGTCACTGACGCTGAGGCGCGAAAGCCGGGGGAGCGAACA"
## [113] "GCGAGCGTTGTCCGGAATTATTGGGCGTAAAGGGCTTGTAGGCGGTTGGTCGCGTCTGCCGTGAAATCCTCTGGCTTAACTGGGGGCGTGCGGTGGGTACGGGCTGACTTGAGTGCGGTAGGGGAGACTGGAACTCCTGGTGTAGCGGTGGAATGCGCAGATATCAGGAAGAACACCGGTGGCGAAGGCGGGTCTCTGGGCCGTTACTGACGCTGAGGAGCGAAAGCGTGGGGAGCGAAC"
## [114] "GCAAGCGTTATCCGGATTTACTGGGTGTAAAGGGAGCGTAGACGGAATGGCAAGTCTGATGTGAAAGGCCGGGGCTCAACCCCGGGACTGCATTGGAAACTGTCAATCTAGAGTACCGGAGGGGTAAGTGGAATTCCTAGTGTAGCGGTGAAATGCGTAGATATTAGGAGGAACACCAGTGGCGAAGGCGGCTTACTGGACGGTAACTGACGTTGAGGCTCGAAAGCGTGGGGAGCAAAC"
## [115] "TCTAGTGGTAGCAGTTTTTATTGGGCCTAAAGCGTCCGTAGCCGGTTTAATAAGTCTCTGGTGAAATCCTGCAGCTTAACTGTGGGAATTGCTGGAGATACTATTAGACTTGAGATCGGGAGAGGTTAGAGGTACTCCCAGGGTAGAGGTGAAATTCTGTAATCCTGGGAGGACCGCCTGTTGCGAAGGCGTCTGACTGGAACGATTCTGACGGTGAGGGACGAAAGCTAGGGGCGCGAA"
## [116] "GCAAGCGTTATCCGGATTTACTGGGTGTAAAGGGAGCGTAGACGGTATGGCAAGTCTGATGTGAAAGGCCAGGGCTCAACCCTGGGACTGCATTGGAAACTGTCGAACTAGAGTGTCGGAGAGGCAAGTGGAATTCCTAGTGTAGCGGTGAAATGCGTAGATATTAGGAGGAACACCAGTGGCGAAGGCGGCTTGCTGGACGATGACTGACGTTGAGGCTCGAAAGCGTGGGGAGCAAAC"
## [117] "GCGAGCGTTATCCGGATTCATTGGGCGTAAAGCGCGCGTAGGCGGAGCGCTAAGCGGGACCTCTAACCCGAGGGCTCAACCCCCGGCCGGGTCCCGAACTGGCGCTCTCGAGTGCGGTAGGGGAGAGCGGAATTCCCGGTGTAGCGGTGGAATGCGCAGATATCGGGAGGAACACCGACGGCGAAGGCAGCTCTCTGGGCCGAAACTGACGCTGAGGCGCGAAAGCTGGGGGAGCGAACA"
## [118] "GCAAGCGTTATCCGGATTTACTGGGTGTAAAGGGAGCGTAGACGGCTGTGCAAGTCTGAAGTGAAAGGCATGGGCTCAACCTGTGGACTGCTTTGGAAACTGTGCAGCTAGAGTGTCGGAGAGGTAAGTGGAATTCCTAGTGTAGCGGTGAAATGCGTAGATATTAGGAGGAACACCAGTGGCGAAGGCGGCTTACTGGACGATGACTGACGTTGAGGCTCGAAAGCGTGGGGAGCAAAC"
## [119] "GCTAGCGTTATCCGGAATTACTGGGCGTAAAGGGTGCGTAGGTGGTTTCTTAAGTCAGAGGTGAAAGGCTACGGCTCAACCGTAGTAAGCCTTTGAAACTGGGAAACTTGAGTGCAGGAGAGGAGAGTGGAATTCCTAGTGTAGCGGTGAAATGCGTAGATATTAGGAGGAACACCAGTTGCGAAGGCGGCTCTCTGGACTGTAACTGACACTGAGGCACGAAAGCGTGGGGAGCAAACA"
## [120] "GCGAGCGTTATCCGGATTCATTGGGCGTAAAGCGCGCGTAGGCGGCCCGTCAAGCGGGGTTTCAAATCCAGGGGCTCAACCTCTGGCCGGACCCCGAACTGGCGGGCTCGAGTGCGGTAGAGGAAGGTGGAATTCCCAGTGTAGCGGTGAAATGCGCAGATATTGGGAAGAACACCGATGGCGAAGGCAGCCTTCTGGGCCGCCACTGACGCTGAGGCGCGAAAGCTAGGGGAGCGAACA"
## [121] "GCAAGCGTTATCCGGATTTACTGGGTGTAAAGGGAGCGTAGACGGCGCAGCAAGTCTGATGTGAAAGGCAGGGGCTTAACCCCTGGACTGCATTGGAAACTGCTGTGCTTGAGTGCCGGAGGGGTAAGCGGAATTCCTAGTGTAGCGGTGAAATGCGTAGATATTAGGAGGAACACCAGTGGCGAAGGCGGCTTACTGGACGGTAACTGACGTTGAGGCTCGAAAGCGTGGGGAGCAAAC"
## [122] "GCAAGCGTTATCCGGATTTACTGGGTGTAAAGGGAGCGTAGACGGATTAGCAAGTCTGATGTGAAAGGCAGGGGCTCAACCCCTGGACTGCATTGGAAACTGCCAGTCTTGAGTGCCGGAGAGGTAAGCGGAATTCCTAGTGTAGCGGTGAAATGCGTAGATATTAGGAGGAACACCAGTGGCGAAGGCGGCTTACTGGACGGCAACTGACGTTGAGGCTCGAAAGCGTGGGGAGCAAAC"
## [123] "TCAAGCGTTGTTCGGAATCACTGGGCGTAAAGCGTGCGTAGGCTGTTTCGTAAGTCGTGTGTGAAAGGCGCGGGCTCAACCCGCGGACGGCACATGATACTGCGAGACTAGAGTAATGGAGGGGGAACCGGAATTCTCGGTGTAGCAGTGAAATGCGTAGATATCGAGAGGAACACTCGTGGCGAAGGCGGGTTCCTGGACATTAACTGACGCTGAGGCACGAAGGCCAGGGGAGCGAAA"
## [124] "GCGAGCGTTATCCGGATTCATTGGGCGTAAAGCGCGCGTAGGCGGCCCGGCAGGCCGGGGGTCGAAGCGGGGGGCTCAACCCCCCGAAGCCCCCGGAACCTCCGCGGCTTGGGTCCGGTAGGGGAGGGTGGAACACCCGGTGTAGCGGTGGAATGCGCAGATATCGGGTGGAACACCGGTGGCGAAGGCGGCCCTCTGGGCCGAGACCGACGCTGAGGCGCGAAAGCTGGGGGAGCGAAC"
## [125] "GCAAGCGTTGTCCGGAATTACTGGGTGTAAAGGGAGCGCAGGCGGACCGGCAAGTTGGAAGTGAAATCCATGGGCTCAACCCGTGAATTGCTTTCAAAACTGCTGGCCTTGAGTAGTGCAGAGGTAGGTGGAATTCCCGGTGTAGCGGTGGAATGCGTAGATATCGGGAGGAACACCAGTGGCGAAGGCGACCTACTGGGCACCAACTGACGCTGAGGCTCGAAAGCATGGGTAGCAAAC"
## [126] "GCAAGCGTTGTCCGGAATTACTGGGTGTAAAGGGAGCGCAGGCGGGAAGACAAGTTGGAAGTGAAAACCATGGGCTCAACCCATGAATTGCTTTCAAAACTGTTTTTCTTGAGTAGTGCAGAGGTAGATGGAATTCCCGGTGTAGCGGTGGAATGCGTAGATATCGGGAGGAACACCAGTGGCGAAGGCGGTCTACTGGGCACCAACTGACGCTGAGGCTCGAAAGCATGGGTAGCAAAC"
## [127] "GCAAGCGTTATCCGGAATTATTGGGCGTAAAGGGCTCGTAGGCGGTTCGTCGCGTCCGGTGTGAAAGTCCATCGCTTAACGGTGGATCCGCGCCGGGTACGGGCGGGCTTGAGTGCGGTAGGGGAGACTGGAATTCCCGGTGTAACGGTGGAATGTGTAGATATCGGGAAGAACACCAATGGCGAAGGCAGGTCTCTGGGCCGTTACTGACGCTGAGGAGCGAAAGCGTGGGGAGCGAAC"
## [128] "GCAAGCGTTATCCGGAATTATTGGGCGTAAAGGGCTCGTAGGCGGTTCGTCGCGTCCGGTGTGAAAGTCCATCGCTTAACGGTGGATCTGCGCCGGGTACGGGCGGGCTGGAGTGCGGTAGGGGAGACTGGAATTCCCGGTGTAACGGTGGAATGTGTAGATATCGGGAAGAACACCAATGGCGAAGGCAGGTCTCTGGGCCGTTACTGACGCTGAGGAGCGAAAGCGTGGGGAGCGAAC"
## 
## $x_only
##  [1] "GCAAGCGTTATCCGGATTTACTGGGTGTAAAGGGAGCGTAGACGGCATGGCAAGTCTGAAGTGAAATGCGGGGGCTCAACCCCTGAACTGCTTTGGAAACTGTCAGGCTGGAGTGCAGGAGAGGTAAGTGGAATTCCTAGTGTAGCGGTGAAATGCGTAGATATTAGGAGGAACACCAGTGGCGAAGGCGGCTTACTGGACTGTAACTGACGTTGAGGCTCGAAAGCGTGGGGAGCAAAC"
##  [2] "CCGAGCGTTATCCGGATTTATTGGGTTTAAAGGGTGCGTAGGCTGTTTTTTAAGTTAGAGGTGAAAGCTCGACGCTCAACGTCGAAATTGCCTCTGATACTGAGAGACTAGAGTGTAGTTGCGGAAGGCGGAATGTGTGGTGTAGCGGTGAAATGCTTAGATATCACACAGAACACCGATTGCGAAGGCAGCTTTCCAAGCTATTACTGACGCTGAGGCACGAAAGCGTGGGGAGCGAAC"
##  [3] "ACAAGCGTTGTCCGGAATTACTGGGTGTAAAGGGAGCGCAGGCGGGAAGACAAGTTGGAAGTGAAATCTATGGGCTCAACCCATAAACTGCTTTCAAAACTGCTGGCCTTGAGTAGTGCAGAGGTAGGTGGAATTCCCGGTGTAGCGGTGGAATGCGTAGATATCGGGAGGAACACCAGTGGCGAAGGCGGCCTACTGGGCACCAACTGACGCTGAGGCTCGAAAGTGTGGGTAGCAAAC"
##  [4] "GCGAGCGTTGTCCGGAATTACTGGGTGTAAAGGGAGTGTAGGCGGGAAGGCAAGTCAGAAGTGAAAATTATGGGCTTAACCCATAACCTGCTTTTGAAACTGTTTTTCTTGAGTGAGGCAGAGGCAAGCGGAATTCCTAGTGTAGCGGTGAAATGCGTAGATATTAGGAGGAACACCAGTGGCGAAGGCGGCTTGCTGGGCCTTTACTGACGCTGAGGCTCGAAAGCGTGGGGAGCAAAC"
##  [5] "GCAAGCGTTATCCGGATTTACTGGGTGTAAAGGGAGCGTAGGCGGTCCTGCAAGTCTGATGTGAAAACCCGGGGCTCAACCCCGGGACTGCATTGGAAACTGTAGGACTAGAGTGTCGGAGGGGTAAGTGGAATTCCTAGTGTAGCGGTGAAATGCGTAGATATTAGGAGGAACACCAGTGGCGAAGGCGGCTTACTGGACGACCACTGACGCTGAGGCTCGAAAGCGTGGGGAGCAAAC"
##  [6] "GCAAGCGTTGTCCGGAATTACTGGGTGTAAAGGGAGCGTAGGCGGGATGCCAAGTCAGCTGTGAAAACTATGGGCTTAACCTGTAGACTGCAGTTGAAACTGGTATTCTTGAGTGAAGTAGAGGTTGGCGGAATTCCGAGTGTAGCGGTGAAATGCGTAGATATTCGGAGGAACACCGGTGGCGAAGGCGGCCAACTGGGCTTTAACTGACGCTGAGGCTCGAAAGTGTGGGGAGCAAAC"
##  [7] "ACAAGCGTTGTCCGGATTTACTGGGTGTAAAGGGCGCGTAGGCGGACTGTCAAGTCAGTCGTGAAATACCGGGGCTTAACCCCGGGGCTGCGATTGAAACTGACAGCCTTGAGTATCGGAGAGGAAAGCGGAATTCCTAGTGTAGCGGTGAAATGCGTAGATATTAGGAGGAACACCAGTGGCGAAGGCGGCTTACTGGACGGTAACTGACGTTGAGGCTCGAAAGCGTGGGGAGCAAAC"
##  [8] "ACAAGCGTTGTCCGGAATTACTGGGTGTAAAGGGAGCGCAGGCGGGAAGACAAGTTGGAAGTGAAATCTATGGGCTCAACCCATAAACTGCTTTCAAAACTGTTTTTCTTGAGTAGTGCAGAGGTAGGCGGAATTCCCGGTGTAGCGGTGGAATGCGTAGATATCGGGAGGAACACCAGTGGCGAAGGCGGCCTACTGGGCACCAACTGACGCTGAGGCTCGGAAGTGTGGGTAGCAAAC"
##  [9] "ACAAGCGTTGTCCGGAACTACTGGGTGTAAAGGGAGCGCAGGCGGGAAGACAAGTTGGAAGTGAAATCTATGGGCTCAACCCATAAACTGCTTTCAAAACTGTTTTTCTTGAGTAGTGCAGAGGTAGGCGGAATTCCCGGTGTAGCGGTGGAATGCGTAGATATCGGGAGGAACACCAGTGGCGAAGGCGGCCTACTGGGCACCAACTGACGCTGAGGCTCGAAAGTGTGGGTAGCAAAC"
## [10] "GCGAGCGTTATCCGGATTTATTGGGTTTAAAGGGTGCGTAGGCGGCACGCCAAGTCAGCGGTGAAATTTTCGGGCTCAACCCGGACTGTGCCGTTGAAACTGGCGAGCTAGAGTGCACAAGAGGCAGGCGGAATGCGTGGTGTAGCGGTGAAATGCATAGATATCACGCAGAACCCCGATTGCGAAGGCAGCCTGCTAGGGTGCGACAGACGCTGAGGCACGAAAGCGTGGGTATCGAAC"
## [11] "GCGAGCGTTATCCGGATTTATTGGGTTTAAAGGGTGCGTAGGCGGAAGAATAAGTCAGCGGTGAAATGCTTCAGCTCAACTGGAGAATTGCCGATGAAACTGTTTTTCTAGAGTATAAAAGAGGTATGCGGAATGCGTGGTGTAGCGGTGAAATGCATAGATATCACGCAGAACCCCGATTGCGAAGGCAGCATACTGGGCTATAACTGACGCTGAAGCACGAAAGCGTGGGTATCGAAC"
## [12] "ACAAGCGTTGTCCGGAATTACTGGGTGTAAAGGGAGCGCAGGCGGGCGATCAAGTTGGAAGTGAAATCCATGGGCTCAACCCATGAACTGCTTTCAAAACTGATTGTCTTGAGTAGTGCAGAGGTAGGCGGAATTCCCGGTGTAGCGGTGGAATGCGTAGATATCGGGAGGAACACCAGTGGCGAAGGCGGCCTACTGGGCACCAACTGACGCTGAGGCTCGAAAGTGTGGGTAGCAAAC"
## [13] "GCAAGCGTTGTCCGGAATTATTGGGCGTAAAGCGCGCGCAGGCGGCTTCTTAAGTCCATCTTAAAAGTGCGGGGCTTAACCCCGTGATGGGATGGAAACTGGGAGGCTGGAGTATCGGAGAGGAAAGTGGAATTCCTAGTGTAGCGGTGAAATGCGTAGAGATTAGGAAGAACACCGGTGGCGAAGGCGACTTTCTGGACGACAACTGACGCTGAGGCGCGAAAGCGTGGGGAGCAAACA"
## [14] "GCAAGCGTTATCCGGATTTACTGGGTGTAAAGGGAGCGCAGGCGGCATGATAAGTCTGATGTGAAAACCCAAGGCTCAACCATGGGACTGCATTGGAAACTGTCGTGCTGGAGTGTCGGAGAGGTAAGCGGAATTCCTAGTGTAGCGGTGAAATGCGTAGATATTAGGAGGAACACCAGTGGCGAAGGCGGCTTACTGGACGGTAACTGACGTTGAGGCTCGAAAGCGTGGGGAGCAAAC"
## [15] "GCGAGCGTTATCCGGAATTACTGGGTGTAAAGGGTGTGTAGGCGGGGTGTCAAGTCAGATGTGAAAACTGTGGGCTCAACCCACAAACTGCATTTGAAACTGATACTCTTGAGAGTGGGAGAGGTAAACGGAATTCCTGGTGTAGTAGTGAAATGCGTAGATATCAGGAGGAACACCGGTGGCGAAGGCGGTTTACTGGACCACAACTGACGCTGAGACACGAAAGCGTGGGGAGCAAAC"
## [16] "GCAAGCGTTATCCGGATTTACTGGGTGTAAAGGGAGCGTAGACGGCATGGCAAGCCAGATGTGAAAGCCCGGGGCTCAACCCCGGGACTGCATTTGGAACTGTCAGGCTAGAGTGTCGGAGAGGAAAGCGGAATTCCTAGTGTAGCGGTGAAATGCGTAGATATTAGGAGGAACACCAGTGGCGAAGGCGGCTTACTGGACGGTAACTGACGTTGAGGCTCGAAAGCGTGGGGAGCAAAC"
## [17] "ACAAGCGTTGTCCGGAATTACTGGGTGTAAAGGGAGCGCAGGCGGGAAGACAAGTTGGGAGTGAAATCTATGGGCTCAACCCATAAACTGCTTTCAAAACTGTTTTTCTTGAGTAGTGCAGAGGTAGGCGGAATTCCCGGTGTAGCGGTGGAATGCGTAGATATCGGGAGGAACACCAGTGGCGAAGGCGGCCTACTGGGCACCAACTGACGCTGAGGCTCGAAAGTGTGGGTAGCAAAC"
## [18] "GCGAGCGTTATCCGGATTTATTGGGTTTAAAGGGTGCGTAGGCGGGTGATTAAGTCAGCGGTGAAATGCGTCAGCTTAACTGGCGAACTGCCATTGAAACTGGTTACCTTGAGTGTAGCGGAAGTATGCGGAATGCGTGGTGTAGCGGTGAAATGCATAGATATCACGCAGAACTCCGATTGCGAAGGCAGCATACCATACTATAACTGACGCTGAAGCACGAAAGCGTGGGTATCGAAC"
## [19] "GCAAGCGTTGTCCGGATTTATTGGGCGTAAAGCGAGCGCAGGCGGAAGAATAAGTCTGATGTGAAAGCCCTCGGCTTAACCGAGGAACTGCATCGGAAACTGTTTTTCTTGAGTGCAGAAGAGGAGAGTGGAACTCCATGTGTAGCGGTGGAATGCGTAGATATATGGAAGAACACCAGTGGCGAAGGCGGCTCTCTGGTCTGCAACTGACGCTGAGGCTCGAAAGCATGGGTAGCGAAC"
## [20] "GCAAGCGTTATCCGGATTTACTGGGTGTAAAGGGTGCGTAGGTGGCAGTGCAAGTCAGATGTGAAAGGCCGGGGCTCAACCCCGGAGCTGCATTTGAAACTGCGCGGCTAGAGTACAGGAGAGGCAGGCGGAATTCCTAGTGTAGCGGTGAAATGCGTAGATATTAGGAGGAACACCAGTGGCGAAGGCGGCCTGCTGGACTGTTACTGACACTGAGGCACGAAAGCGTGGGGAGCAAAC"
## [21] "GCGAGCGTTGTCCGGAATTATTGGGCGTAAAGAGTACGTAGGCGGTTTGCTAAGCGCAAGGTGAAAGGCAGTGGCTTAACCATTGTAAGCCTTGCGAACTGGCAGACTTGAGTGCAGGAGAGGAAAGCGGAATTCCTAGTGTAGCGGTGAAATGCGTAGATATTAGGAGGAACACCGGTGGCGAAGGCGGCTTTCTGGACTGTAACTGACGCTGAGGTACGAAAGCGTGGGGAGCAAACA"
## [22] "GCAAGCGTTGTCCGGAATCACTGGGCGTAAAGGGCGCGTAGGCGGCAATATAAGTCAGATGTGAAAGGTGAGGGCTCAACCCTTAGACTGCATCTGATACTGTATAGCTTGAGTGTGAGAGAGGAAAGCGGAATTCCTAGTGTAGCGGTGAAATGCGTAGATATTAGGAGGAACACCAGTGGCGAAGGCGGCTTTCTGGCTCATAACTGACGCTGAGGCGCGAAAGCGTGGGGAGCAAAC"
## [23] "GCAAGCGTTATCCGGATTTACTGGGTGTAAAGGGAGCGTAGGCGGCGATGCAAGTCAGAAGTGAAAGCCCAGGGCTTAACCGTGGGACTGCTTTTGAAACTGTGTTGCTGGATTGCCGGAGAGGTAAGTGGAATTCCTAGTGTAGCGGTGAAATGCGTAGATATTAGGAGGAACACCAGTGGCGAAGGCGGCTTACTGGACGGTGAATGACGCTGAGGCTCGAAAGCGTGGGGAGCAAAC"
## [24] "GCGAGCGTTGTCCGGAATTACTGGGCGTAAAGGGAGCGTAGGCGGTCTGATAAGTTGGATGTGAAATACCCGGGCTTAACTTGGGGGGTGCATCCAATACTGTTGGACTAGAGTACAGGAGAGGAAAGCGGAATTCCTAGTGTAGCGGTGAAATGCATAGATATTAGGAGGAACATCGGTGGCGAAGGCGGCTTTCTGGACTGCAACTGACGCTGAGGCTCGAAAGCGTGGGGAGCAAAC"
## [25] "GCAAGCGTTATCCGGATTTATTGGGTGTAAAGGGTGCGTAGACGGGAAGGTAAGTTAGTTGTGAAATCCCTCGGCTCAACTGAGGAACTGCGACTAAAACTGCTTTTCTTGAGTGCTGGAGAGGAAAGTGGAATTCCTAGTGTAGCGGTGAAATGCGTAGATATTAGGAGGAACACCAGTGGCGAAGGCGACTTTCTGGACAGCAACTGACGTTGAGGCACGAAAGTGTGGGGAGCAAAC"
## [26] "CCGAGCGTTATCCGGATTTATTGGGTTTAAAGGGAGCGTAGGTGGACAGTTAAGTCAGTTGTGAAAGTTTGCGGCTCAACCGTAAAATTGCAGTTGATACTGGCTGTCTTGAGTACAGTAGAGGTGGGCGGAATTCGTGGTGTAGCGGTGAAATGCTTAGATATCACGAAGAACTCCGATTGCGAAGGCAGCTCACTGGAGCGCAACTGACGCTGAAGCTCGAAAGTGCGGGTATCGAAC"
## [27] "GCAAGCGTTATCCGGATTTACTGGGTGTAAAGGGTGCGTAGGTGGTGAGACAAGTCTGAAGTGAAAATCCGGGGCTTAACCCCGGAACTGCTTTGGAAACTGCCTGACTAGAGTACAGGAGAGGTAAGTGGAATTCCTAGTGTAGCGGTGAAATGCGTAGATATTAGGAGGAACACCAGTGGCGAAGGCGACTTACTGGACTGCTACTGACACTGAGGCACGAAAGCGTGGGGAGCAAAC"
## [28] "GCAAGCGTTATCCGGATTTACTGGGTGTAAAGGGAGCGTAGGTGGCAAGGCAAGCCAGAAGTGAAAACCCGGGGCTCAACCGCGGGATTGCTTTTGGAACTGTCATGCTAGAGTGCAGGAGGGGTGAGCGGAATTCCTAGTGTAGCGGTGAAATGCGTAGATATTAGGAGGAACACCAGTGGCGAAGGCGGCTTACTGGACGATAACTGACGCTGAGGCTCGAAAGCGTGGGGAGCAAAC"
## [29] "GCAAGCGTTGTCCGGATTTACTGGGTGTAAAGGGCGTGCAGCCGGGCCGGCAAGTCAGATGTGAAATCCACGGGCTTAACCCGTGAACTGCATTTGAAACTGTTGGTCTTGAGTATCGGAGAGGTAATCGGAATTCCTTGTGTAGCGGTGAAATGCGTAGATATAAGGAAGAACACCAGTGGCGAAGGCGGATTACTGGACGACAACTGACGGTGAGGCGCGAAAGCGTGGGGAGCAAAC"
## [30] "CCGAGCGTTATCCGGATTTATTGGGTTTAAAGGGAGCGTAGGCGGATTATTAAGTCAGTTGTGAAAGTTTGCGGCTCAACCGTAAAATTGCAGTTGATACTGGTAGTCTTGAGTGCAGCAGAGGTAGGCGGAATTCGTGGTGTAGCGGTGAAATGCTTAGATATCACGAAGAACTCCGATTGCGAAGGCAGCTTACTGGACTGTAACTGACGCTGATGCTCGAAAGTGTGGGTATCAAAC"
## [31] "GCAAGCGTTATCCGGAATTATTGGGCGTAAAGGGCTCGTAGGCGGTTCGTCGCGTCCGGTGTGAAAGTTCATCGCTTAACGGTGGATCCGCGCCGGGTACGGGCGGGCTTGAGTGCGGTAGGGGAGACTGGAATTCCCGGTGTAACGGTGGAATGTGTAGATATCGGGAAGAACACCAATGGCGAAGGCAGGTCTCTGGGCCGTTACTGACGCTGAGGAGCGAAAGCGTGGGGAGCGAAC"
## [32] "GCAAGCGTTGTCCGGAATTACTGGGTGTAAAGGGAGCGTAGGCGGGATGCCAAGTCAGCTGTGAAAACTATGGGCTTAACTTGTAGACTGCAGTTGAAACTGGTATTCTTGAGTGAAGTAGAGGTTGGCGGAATTCCGAGTGTAGCGGTGAAATGCGTAGATATTCGGAGGAACACCGGTGGCGAAGGCGGCCAACTGGGCTTTAACTGACGCTGAGGCTCGAAAGTGTGGGGAGCAAAC"
## [33] "GCAAGCGTTATCCGGATCTACTGGGTGTAAAGGGAGCGTAGACGGATGGACAAGTCTGATGTGAAAGGCTGGGGCTCAACCCCGGGACTGCATTGGAAACTGCCCGTCTTGAGTGCCGGAGAGGTAAGCGGAATTCCTAGTGTAGCGGTGAAATGCGTAGATATTAGGAGGAACACCAGTGGCGAAGGCGGCTTACTGGACGGTAACTGACGTTGAGGCTCGAAAGCGTGGGGAGCAAAC"
## [34] "GCAAGCGTTATCCGGATTTACTGGGTGTAAAGGGAGCGTAGACGGTATGGCAAGTCTGATGTGAAAGGCCAGGGCTCAACCCTGGGACTGCATTGGAAACTGTCGAACTAGAGTGTCGGAGAGGCAAGTGGAATTCCTAGTGTAGCGGTGAAATGCGTAGATATTAGGAGGAACACCAGTGGCGAAGGCGGCTTACTGGACGGTAACTGACGTTGAGGCTCGAAAGCGTGGGGAGCAAAC"
## [35] "GCAAGCGTTGTCCGGAATGATTGGGCGTAAAGGGCGCGTAGGCGGCCAACTAAGTCTGGAGTGAAAGTCCTGCTTTTAAGGTGGGAATTGCTTTGGAAACTGGATGGCTTGAGTGCAGGAGAGGTAAGCGGAATTCCCGGTGTAGCGGTGAAATGCGTAGAGATCGGGAGGAACACCAGTGGCGAAGGCGGCTTACTGGACTGTAACTGACGCTGAGGCGCGAAAGTGTGGGGAGCAAAC"
## [36] "GCAAGCGTTATCCGGATTTACTGGGTGTAAAGGGAGCGTAGACGGATGGACAAGTCTGATGTGAAAGGCTGGGGCTCAACCCCGGGACTGCATTGGAAACTGCCCGTCTTGAGTGCCGGAGAGGTAAGCGGAATTCCTAGTGTAGCGGTGAAATGCGTAGATATACGGAGGAACACCAGTGGCGAAGGCGGCCTGCTGGACATTAACTGACGCTGAGGCGCGAAAGCGTGGGGAGCAAAC"
## [37] "GCAAGCGTTGTCCGGAATTATTGGGCGTAAAGAGTACGTAGGCGGTCTGGTAAGCGCAAGGTGAAAGGCATAGGCTCAACCAATGTCAGCCTTGCGAACTGTCAGACTTGAGTGCAGGAGGGGAAAGTGGAATTCCTAGTGTAGCGGTGAAATGCGTAGATATTAGGAGGAACACCAGTGGCGAAGGCGACTTTCTGGACTGTAACTGACGCTGAGGTACGAAAGCGTGGGGAGCAAACA"
## [38] "GCGAGCGTTGTCCGGAATTACTGGGTGTAAAGGGAGCGTAGGCGGGACAGCAAGTTGAATGTGAAATCTATGGGCTCAACCCATAAACTGCGTTCAAAACTGTTGTTCTTGAGTGAAGTAGAGGTAGGCGGAATTCCTAGTGTAGCGGTGAAATGCGTAGATATTAGGAGGAACACCAGTGGCGAAGGCGGCCTACTGGGCTTTAACTGACGCTGAGGCTCGAAAGCGTGGGTAGCAAAC"
## [39] "GCAAGCGTTATCCGGATTTACTGGGTGTAAAGGGAGCGTAGGCGGTCCTGCAAGTCTGATGTGAAAGGCCGGGGCTCAACCCCGGGACTGCATTGGAAACTGTAGGACTAGAGTGTCGGAGGGGTAAGTGGAATTCCTAGTGTAGCGGTGAAATGCGTAGATATTAGGAGGAACACCAGTGGCGAAGGCGGCTTACTGGACGGTAACTGACGTTGAGGCTCGAAAGCGTGGGGAGCAAAC"
## [40] "GCAAGCGTTGTCCGGAATTACTGGGTGTAAAGGGAGCGTAGGCGGGGAGACAAGTTGAATGTTTAAACTATCGGCTCAACTGATAGTCGCGTTCAAAACTATCACTCTTGAGTGCAGTAGAGGTAGGCGGAATTCCTAGTGTAGCGGTGAAATGCGTAGATATTAGGAGGAACACCAGTGGCGAAGGCGGCCTACTGGGCTGTAACTGACGCTGAGGCTCGAAAGCGTGGGTAGCAAACA"
## [41] "GCGAGCGTTAATCGGAATTACTGGGCGTAAAGCGCACGTAGGCTGTATGTCAAGTCAAGGGTGAAATCCCACGGCTCAACCGTGGAACTGCCTTTGAAACTGGCAAACTGGAGTATGTGAGAGGGCGGCGGAATTCCTGGTGTAGGAGTGAAATCCGTAGATATCAGGAGGAACATCAGTGGCGAAGGCGGCCGCCTGGCACAAAACTGACGCTGAGGTGCGAAAGCGTGGGTAGCAAAC"
## [42] "GCAAGCGTTATCCGGATTTACTGGGTGTAAAGGGCGTGTAGGCGGGACTGCAAGTCAGGTGTGAAAACCAGGGGCTCAACCTCTGGCCTGCATTTGAAACTGTAGTTCTTGAGTGCTGGAGAGGCAATCGGAATTCCGTGTGTAGCGGTGAAATGCGTAGATATACGGAGGAACACCAGTGGCGAAGGCGGATTGCTGGACAGTAACTGACGCTGAGGCGCGAAAGCGTGGGGAGCAAAC"
## [43] "GCAAGCGTTATCCGGATTTATTGGGTGTAAAGGGTGTGTAGGCGGGACTGCAAGTCAGACGTGAAAATCATGGGCTCAACCCATGACTTGCGTTTGAAACTGCGGTTCTTGAGAGTGGGAGAGGTAAACGGAATTCCTGGTGTAGCGGTGAAATGCGTAGATATCAGGAGGAACACCGGTGGCGAAGGCGGTTTACTGGACCACAACTGACGCTGAGACACGAAAGCGTGGGGAGCAAAC"
## [44] "CCGAGCGTTATCCGGATTTATTGGGTTTAAAGGGAGCGTAGATGGATGTTTAAGTCAGTTGTGAAAGTTTGCGGCTCAACCGTAAAATTGCAGTTGATACTGGCAGTCTTGAGTGCAGTAGAGGTGGGCGGAATTCGTGGTGTAGCGGTGAAATGCTTAGATATCACGAAGAACTCCGATTGCGAAGGCAGCCTGCTAAGCTGCAACTGACATTGAGGCTCGAAAGTGTGGGTATCAAAC"
## [45] "GCGAGCGTTATCCGGATTTATTGGGTTTAAAGGGTGCGTAGGCGGCCCTATAAGTCAGCGGTGAAATGTTCCGGCTCAACCGGGAAACTGCCGTTGAAACTGTAGAGCTAGAGTCCACAAGAGGTATGCGGAATGCGTGGTGTAGCGGTGAAATGCATAGATATCACGCAGAACCCCGATTGCGAAGGCAGCATACTGGGGTGAAACAGACGCTGAAGCACGAAAGCGTGGGTATCGAAC"
## [46] "GCAAGCGTTGTCCGGAATTACTGGGTGTAAAGGGCGTGTAGGCGGAGCTGCAAGTCAGATGTGAAATCCCGGGGCTCAACCCCGGAACTGCATTTGAAACTGTAGCCCTTGAGTATCGGAGAGGCAAGCGGAATTCCTAGTGTAGCGGTGAAATGCGTAGATATTAGGAGGAACACCAGTGGCGAAGGCGGCTTGCTGGACGACAACTGACGCTGAGGCGCGAAAGCGTGGGGAGCAAAC"
## [47] "GCGAGCGTTGTCCGGAATTACTGGGCGTAAAGGGCGCGTAGGCGGCCAATTAAGTTAGATGTGAAATCCCCGGGCTTAACCTGGGTGTTGCATTTAAAACTGATAGGCTTGAGTGCAGGAGAGGGAAGCGGAATTCCTAGTGTAGCGGTGGAATGCGTAGATATTAGGAGGAACACCAGTGGCGAAGGCGGCTTTCTGGACTGTAACTGACGCTGAGGCGCGAGAGCGTGGGGAGCAAAC"
## [48] "GCAAGCGTTATCCGGAATTACTGGGTGTAAAGGGTGAGTAGGCGGCACGGCAAGTAAGATGTGAAAGCCCGAGGCTTAACCTCGGGATTGCATTTTAAACTGCTGAGCTAGAGTACAGGAGAGGAAAGCGGAATTCCTAGTGTAGCGGTGAAATGCGTAGATATTAGGAAGAACACCAGTGGCGAAGGCGGCTTTCTGGACTGAAACTGACGCTGAGGCACGAAAGCGTGGGGAGCGAAC"
## [49] "GCAAGCGTTGTCCGGATTTACTGGGTGTAAAGGGCGTGTAGGCGGAGCAGCAAGTCAGAAGTGAAATCTCTGGGCTCAACCCAGAAACTGCTTTTGAAACTGTTGCCCTTGAGTATCGGAGAGGCAGGCGGAATTCCTAGTGTAGCGGTGAAATGCGTAGATATTAGGAGGAACACCAGTGGCGAAGGCGGCCTGCTGGACGACAACTGACGCTGAGGCGCGAAAGCGTGGGGAGCAAAC"
## [50] "GCGAGCGTTGTCCGGATTTACTGGGTGTAAAGGGTGCGTAGGCGGCTAGACAAGTCAGGTGTGAAATACCGCAGCTCAACTGCGGGGCTGCACTTGAAACTGTAGAGCTTGAGTGATGGAGAGGTAAGCGGAATTCCTAGTGTAGCGGTAAAATGCGTAGATATTAGGAGGAACACCAGTGGCGAAGGCGGCTTACTGGACATTAACTGACGCTGAGGCACGAAAGCGTGGGGAGCAAAC"
## 
## $y_only
##  [1] "GCGAGCGTTGTCCGGATTTACTGGGCGTAAAGGGAGCGTAGGCGGATTTTTAAGTGAGATGTGAAATACTCGGGCTTAACCTGAGTGCTGCATTTCAAACTGGAAGTCTAGAGTGCAGGAGAGGAGAAGGGAATTCCTAGTGTAGCGGTGAAATGCGTAGAGATTAGGAAGAACACCAGTGGCGAAGGCGCTTCTCTGGACTGTAACTGACGCTGAGGCTCGAAAGCGTGGGGAGCAAAC"
##  [2] "GCAAGCGTTGTCCGGATTTACTGGGTGTAAAGGGCGTGCAGCCGGGCATGCAAGTCAGATGTGAAATCTCAGGGCTTAACCCTGAAACTGCATTTGAAACTGTATGTCTTGAGTGCCGGAGAGGTAATCGGAATTCCTTGTGTAGCGGTGAAATGCGTAGATATAAGGAAGAACACCAGTGGCGAAGGCGGATTACTGGACGGTAACTGACGGTGAGGCGCGAAAGCGTGGGGAGCGAAC"
##  [3] "GCGAGCGTTGTCCGGAATTACTGGGTGTAAAGGGAGCGTAGGCGGGATGGCAAGTCAGATGTGAAAACTATGGGCTCAACCCATAGACTGCATTTGAAACTGTTGTTCTTGAGTGAGGTAGAGGTAAGCGGAATTCCTGGTGTAGCGGTGAAATGCGTAGAGATCAGGAGGAACATCGGTGGCGAAGGCGGCTTACTGGGCCTTTACTGACGCTGAGGCTCGAAAGCGTGGGGAGCAAAC"
##  [4] "GCAAGCGTTGTCCGGATTTACTGGGTGTAAAGGGCGTGCAGCCGGGTCTGCAAGTCAGATGTGAAATCCATGGGCTCAACCCATGAACTGCATTTGAAACTGTAGATCTTGAGTGTCGGAGGGGCAATCGGAATTCCTAGTGTAGCGGTGAAATGCGTAGATATTAGGAGGAACACCAGTGGCGAAGGCGGATTGCTGGACGATAACTGACGGTGAGGCGCGAAAGTGTGGGGAGCAAAC"
##  [5] "GCAAGCGTTGTCCGGATTTACTGGGTGTAAAGGGCGTGCAGCCGGGAATGCAAGTCAGATGTGAAATCCATGGGCTTAACCCATGAACTGCATTTGAAACTGTATTTCTTGAGTACTGGAGAGGCAATCGGAATTCCTAGTGTAGCGGTGAAATGCGTAGATATTAGGAGGAACACCAGTGGCGAAGGCGGATTGCTGGACAGCAACTGACGGTGAGGCGCGAAAGTGTGGGGAGCAAAC"
##  [6] "GCAAGCGTTGTCCGGATTTACTGGGTGTAAAGGGCGTGTAGGCGGAGATGCAAGTTAGGAGTGAAATCTATGGGCTCAACCCATAAACTGCTTCTAAAACTGTATCCCTTGAGTATCGGAGAGGCAAGCGGAATTCCTAGTGTAGCGGTGAAATGCGTAGATATTAGGAGGAACACCAGTGGCGAAGGCGGCTTGCTGGACGACAACTGACGCTGAGGCGCGAAAGCGTGGGGAGCAAAC"
##  [7] "GCGAGCGTTGTCCGGAATTACTGGGTGTAAAGGGAGCGTAGGCGGGATTGCAAGTCAGATGTGAAAACTATGGGCTTAACCCATAGACTGCATTTGAAACTGTAGTTCTTGAGTGAAGTAGAGGTAAGCGGAATTCCTAGTGTAGCGGTGAAATGCGTAGATATTAGGAGGAACATCGGTGGCGAAGGCGGCTTACTGGGCTTTTACTGACGCTGAGGCTCGAAAGCGTGGGGAGCAAAC"
##  [8] "GCGAGCGTTGTCCGGAATTACTGGGTGTAAAGGGAGCGTAGGCGGGATCTTAAGTCAGGTGTGAAAACTATGGGCTCAACCCATAGACTGCACTTGAAACTGAGGTTCTTGAGTGAAGTAGAGGCAGGCGGAATTCCTAGTGTAGCGGTGAAATGCGTAGATATTAGGAGGAACATCAGTGGCGAAGGCGGCCTGCTGGGCTTTTACTGACGCTGAGGCTCGAAAGCGTGGGGAGCAAAC"
##  [9] "GCAAGCGTTATCCGGAATTACTGGGTGTAAAGGGTGCGTAGGCGGTATGGCAAGTTTGATGTGAAACCCACAGGCTTAACCTGTGGCTTGCATCGAAAACTACTGAACTAGAGTGCAGGAGAGGAAAGCGGAATTCCTAGTGTAGCGGTGAAATGCGTAGATATTAGGAAGAACACCAGTGGCGAAGGCGGCTTTCTGGACTGCAACTGACGCTGAGGCACGAAAGCGTGGGGAGCAAAC"
## [10] "GCGAGCGTTGTTCGGAATTACTGGGCGTAAAGCGCACGCAGGCGGTTCGTTAAGTCTGCTGTCAAAGGCTGAGGCTCAACCTCAGTTCTACAACAGATACTGGCGGACTAGAGTATGTGAGAGGGAAGTGGAATTCCCGGTGTAGCGGTGAAATGCGTAGATATCGGGAGGAACACCAGTGGCGAAGGCGGCTTCCTGGCACACTACTGACGCTCATGTGCGAAAGCCAGGGCAGCGAAC"
## [11] "GCAAGCGTTATCCGGATTTACTGGGTGTAAAGGGAGCGTAGACGGCGAAGCAAGTCTGAAGTGAAAACCCAGGGCTCAACCCTGGGACTGCTTTGGAAACTGTTTTGCTAGAGTGTCGGAGAGGTAAGTGGAATTCCTAGTGTAGCGGTGAAATGCGTAGATATTAGGAGGAACACCAGTGGCGAAGGCGGCTTACTGGACGATAACTGACGTTGAGGCTCGAAAGCGTGGGGAGCAAAC"
## [12] "GCAAGCGTTGTCCGGATTTACTGGGTGTAAAGGGCGTGCAGCCGGGCCGGCAAGTCAGATGTGAAATCTGGAGGCTTAACCTCCAAACTGCATTTGAAACTGTAGGTCTTGAGTACCGGAGAGGTTATCGGAATTCCTTGTGTAGCGGTGAAATGCGTAGATATAAGGAAGAACACCAGTGGCGAAGGCGGATAACTGGACGGCAACTGACGGTGAGGCGCGAAAGCGTGGGGAGCAAAC"
## [13] "GCAAGCGTTGTCCGGAATTATTGGGCGTAAAGGGCGCGCAGGCGGCGTCGTAAGTCGGTCTTAAAAGTGCGGGGCTTAACCCCGTGAGGGGACCGAAACTGCGATGCTAGAGTATCGGAGAGGAAAGCGGAATTCCTAGTGTAGCGGTGAAATGCGTAGATATTAGGAGGAACACCAGTGGCGAAAGCGGCTTTCTGGACGACAACTGACGCTGAGGCGCGAAAGCCAGGGGAGCAAACG"
## [14] "GCAAGCGTTGTCCGGAATTATTGGGCGTAAAGGGAGCGCAGGCGGGAAACTAAGCGGATCTTAAAAGTGCGGGGCTCAACCCCGTGATGGGGTCCGAACTGGTTTTCTTGAGTGCAGGAGAGGAAAGCGGAATTCCCAGTGTAGCGGTGAAATGCGTAGATATTGGGAAGAACACCAGTGGCGAAGGCGGCTTTCTGGACTGTAACTGACGCTGAGGCTCGAAAGCTAGGGTAGCGAACG"
## 
## $z_only
##   [1] "CCGGGCGTTATCCGGATTTATTGGGTTTAAAGGGAGCGTAGGCCGGAGATTAAGCGTGTTGTGAAATGTAGATGCTCAACATCTGCACTGCAGCGCGAACTGGTTTCCTTGAGTACGCACAAAGTGGGCGGAATTCGTGGTGTAGCGGTGAAATGCTTAGATATCACGAAGAACTCCGATTGCGAAGGCAGCTCACTGGAGCGCAACTGACGCTGAAGCTCGAAAGTGCGGGTATCGAAC"
##   [2] "CCGGGCGTTATCCGGATTTATTGGGTTTAAAGGGAGCGTAGGCCGGAGATTAAGCGTGTTGTGAAATGTAGACGCTCAACGTCTGCACTGCAGCGCGAACTGGTTTCCTTGAGTACGCACAAAGTGGGCGGAATTCGTGGTGTAGCGGTGAAATGCTTAGATATCACGAAGAACTCCGATTGCGAAGGCAGCTCACTGGAGCGCAACTGACGCTGAAGCTCGAAAGTGCGGGTATCGAAC"
##   [3] "CCGGGCGTTATCCGGATTTATTGGGTTTAAAGGGAGCGTAGGCCGCAGGTTAAGCGTGTTGTGAAATGTAGGGGCTCAACCTCTGCACTGCAGCGCGAACTGGCTTGCTTGAGTACGCACAACGTGGGCGGAATTCGTGGTGTAGCGGTGAAATGCTTAGATATCACGAAGAACTCCGATTGCGAAGGCAGCTCACGGGAGCGCAACTGACGCTGAAGCTCGAAAGTGCGGGTATCGAAC"
##   [4] "CCGGGCGTTATCCGGATTTATTGGGTTTAAAGGGAGCGTAGGCCGGAGATTAAGCGTGTTGTGAAATGTAGAGGCTCAACCTCTGCACTGCAGCGCGAACTGGTCTTCTTGAGTACGCACAACGTGGGCGGAATTCGTGGTGTAGCGGTGAAATGCTTAGATATCACGAAGAACTCCGATTGCGAAGGCAGCTCACGGGAGCGCAACTGACGCTGAAGCTCGAAAGTGCGGGTATCGAAC"
##   [5] "CCGGGCGTTATCCGGATTTATTGGGTTTAAAGGGAGCGTAGGCCGGAGATTAAGCGTGTTGTGAAATGTAGACGCTCAACGTCTGCACTGCAGCGCGAACTGGTTTCCTTGAGTACGCACAAAGTGGGCGGAATTCGTGGTGTAGCGGTGAAATGCTTAGATATCACGAAGAACTCCGATTGCGAAGGCAGCTCACGGGAGCGCAACTGACGCTGAAGCTCGAAAGTGCGGGTATCGAAC"
##   [6] "CCGGGCGTTATCCGGATTTATTGGGTTTAAAGGGAGCGTAGGCCGGAGATTAAGCGTGTTGTGAAATGTAGATGCTCAACATCTGCACTGCAGCGCGAACTGGTTTCCTTGAGTACGCATAAAGTGGGCGGAATTCGTGGTGTAGCGGTGAAATGCTTAGATATCACGAAGAACTCCGATTGCGAAGGCAGCTCACTGGGGCGCAACTGACGCTGAAGCTCGAAAGCGCGGGTATCGAAC"
##   [7] "CCGGGCGTTATCCGGATTTATTGGGTTTAAAGGGAGCGTAGGCCGGAGATTAAGCGTGTTGTGAAATGTAGAGGCTCAACCTCTGCACTGCAGCGCGAACTGGTCTTCTTGAGTACGCACAACGTGGGCGGAATTCGTGGTGTAGCGGTGAAATGCTTAGATATCACGAAGAACTCCGATTGCGAAGGCAGCTCACTGGAGCGCAACTGACGCTGAAGCTCGAAAGTGCGGGTATCGAAC"
##   [8] "GCGAGCGTTATCCGGATTTATTGGGTTTAAAGGGTGCGCAGGCGGCGCGCCAAGTCAGCGGTCAAAGTTCCGGGCTCAACCCGGTGTCGCCGTTGAAACTGGCGTGCTCGAGTGCGTGCGAGGAAGGCGGAATGCGTTGTGTAGCGGTGAAATGCATAGATATGACGCAGAACTCCGATTGCGAAGGCAGCTTTCCAGCGCGCTACTGACGCTGAGGCACGAAAGCGTGGGGATCGAACA"
##   [9] "CCGGGCGTTATCCGGATTTATTGGGTTTAAAGGGAGCGTAGGCCGCCCCTTAAGCGTGTTGTGAAATGCCGCGGCTCAACCGTGGCACTGCAGCGCGAACTGGGGGGCTTGAGTGCACGCAACGCAGGCGGAATTCGTGGTGTAGCGGTGAAATGCTTAGATATCACGAAGAACTCCGATTGCGAAGGCAGCTTGCGGGAGTGCGACTGACGCTGAAGCTCGAAGGTGCGGGTATCGAAC"
##  [10] "GCAAGCGTTGTCCGGAATTATTGGGCGTAAAGCGCGCGCAGGCGGCTTCTTAAGTCCATCTTAAAAGTGCGGGGCTTAACCCCGTGATGGGATGGAAACTGAGAGGCTGGAGTATCGGAGAGGAAAGTGGAATTCCTAGTGTAGCGGTGAAATGCGTAGAGATTAGGAAGAACACCGGTGGCGAAGGCGACTTTCTGGACGACAACTGACGCTGAGGCGCGAAAGCGTGGGGAGCAAACA"
##  [11] "CCGGGCGTTATCCGGATTTATTGGGTTTAAAGGGAGCGTAGGCCGGAGATTAAGCGTGTTGTGAAATGTAGACGCTCAACGTCTGCACTGCAGCGCGAACTGGTTTCCTTGAGTACGCACAAAGTGGGCGGAATTCGTGGTGTAGCGGTGAAATGCTTAGATATCACGAAGAACTCCGATTGCGAAGGCAGCTCACTGGGGCGCAACTGACGCTGAAGCTCGAAAGCGCGGGTATCGAAC"
##  [12] "CCGGGCGTTATCCGGATTTATTGGGTTTAAAGGGAGCGTAGGCCGTGAGGTAAGCGTGTTGTGAAATGTAGGCGCCCAACGTCTGCACTGCAGCGCGAACTGCCCCACTTGAGTGCGCGCAACGCCGGCGGAACTCGTCGTGTAGCGGTGAAATGCTTAGATATGACGAAGAACCCCGATTGCGAAGGCAGCTGGCGGGAGCGTAACTGACGCTGAAGCTCGAAAGCGCGGGTATCGAAC"
##  [13] "CCGGGCGTTATCCGGATTTATTGGGTTTAAAGGGAGCGTAGGCCGGAGATTAAGCGTGTTGTGAAATGTAGTGGCTCAACCTCTGCACTGCAGCGCGAACTGGTCTTCTTGAGTACGCACAACGTGGGCGGAATTCGTGGTGTAGCGGTGAAATGCTTAGATATCACGAAGAACTCCGATTGCGAAGGCAGCTCACGGGAGCGCAACTGACGCTGAAGCTCGAAAGTGCGGGTATCGAAC"
##  [14] "CCGGGCGTTATCCGGATTTATTGGGTTTAAAGGGAGCGTAGGCCGTCTTTTAAGCGTGTTGTGAAATACTGTCGCTCAACGACAGAGGTGCAGCGCGAACTGGGAGACTTGAGTGCGCGGAATGCAGGCGGAATTCGTCGTGTAGCGGTGAAATGCTTAGATATGACGAAGAACTCCGATTGCGAAGGCAGCTTGCAGTAGCGTAACTGACGCTGAAGCTCGAAAGTGCGGGTATCGAAC"
##  [15] "CCGGGCGTTATCCGGATTTATTGGGTTTAAAGGGAGCGTAGGCCGTCTTATAAGCGTGTTGTGAAATGTCGGGGCTCAACCTGGGCATTGCAGCGCGAACTGTGAGACTTGAGTGCGCAGGAAGTAGGCGGAATTCGTCGTGTAGCGGTGAAATGCTTAGATATGACGAAGAACTCCGATTGCGAAGGCAGCCTGCTGTAGCGCAACTGACGCTGAAGCTCGAAAGCGTGGGTATCGAAC"
##  [16] "GCGAGCGTTGTTCGGAATCATTGGGCGTAAAGGGCGTGTAGGCGGCCCTGCAAGCCTGGCGTGAAATCCCGGGGCCCAACCCCGGAACCGCGCTGGGAACTGCTGGGCTTGAGCCGCTGTGGCGCAGCCGGAATTCCAGGTGTAGGGGTGAAATCTGTAGATATCTGGAAGAACACCGATGGCGAAGGCAGGCTGCGAGCGGACGGCTGACGCTGAGGCGCGAAGGCGCGGGGAGCGAAC"
##  [17] "ACAAGCGTTGTCCGGAATTACTGGGTGTAAAGGGAGCGCAGGCGGGAAAGCAAGTTGGAAGTGAAATCCATGGGCTCAACCCATGAACTGCTTTCAAAACTGTTTTTCTTGAGTAGTGCAGAGGTAGGCGGAATTCCCGGTGTAGCGGTGGAATGCGTAGATATCGGGAGGAACACCAGTGGCGAAGGCGGCCTACTGGGCACCAACTGACGCTGAGGCTCGAAAGTGTGGGTAGCAAAC"
##  [18] "CCGGGCGTTATCCGGATTTATTGGGTTTAAAGGGAGCGTAGGCCGCAGGTTAAGCGTGTTGTGAAATGTAGGGGCTCAACCTCTGCACTGCAGCGCGAACTGGCTTGCTTGAGTACGCACAACGTGGGCGGAATTCGTGGTGTAGCGGTGAAATGCTTAGATATCACGAAGAACTCCGATTGCGAAGGCAGCTCACTGGAGCGCAACTGACGCTGAAGCTCGAAAGTGCGGGTATCGAAC"
##  [19] "GCGAGCGTTGTCCGGAATTATTGGGCGTAAAGAGCATGTAGGCGGTTTTTTAAGTCTGGAGTGAAAATGCGGGGCTCAACCCCGTATGGCTCTGGATACTGGAAGACTTGAGTGCAGGAGAGGAAAGGGGAATTCCCAGTGTAGCGGTGAAATGCGTAGATATTGGGAGGAACACCAGTGGCGAAGGCGCCTTTCTGGACTGTGTCTGACGCTGAGATGCGAAAGCCAGGGTAGCGAACG"
##  [20] "CCGGGCGTTATCCGGATTTATTGGGTTTAAAGGGAGCGTAGGCCGGAGATTAAGCGTGTTGTGAAATGTAGATGCTCAACATCTGCACTGCAGCGCGAACTGGTTTCCTTGAGTACGCATAAAGTGGGCGGAATTCGTGGTGTAGCGGTGAAATGCTTAGATATCACGAAGAACTCCGATTGCGAAGGCAGCTCACTGGAGCGCAACTGACGCTGAAGCTCGAAAGTGCGGGTATCGAAC"
##  [21] "CCGGGCGTTATCCGGATTTATTGGGTTTAAAGGGAGCGTAGGCCGGAGATTAAGCGTGTTGTGAAATGTAGATGCTCAACATCTGAACTGCAGCGCGAACTGGTTTCCTTGAGTACGCACAAAGTGGGCGGAATTCGTGGTGTAGCGGTGAAATGCTTAGATATCACGAAGAACTCCGATTGCGAAGGCAGCTCACTGGAGCGCAACTGACGCTGAAGCTCGAAAGTGCGGGTATCGAAC"
##  [22] "CCGGGCGTTATCCGGATTTATTGGGTTTAAAGGGAGCGTAGGCCGGAGATTAAGCGTGTTGTGAAATGTAGTGGCTCAACCTCTGCACTGCAGCGCGAACTGGTCTTCTTGAGTACGCACAACGTGGGCGGAATTCGTGGTGTAGCGGTGAAATGCTTAGATATCACGAAGAACTCCGATTGCGAAGGCAGCTCACTGGAGCGCAACTGACGCTGAAGCTCGAAAGTGCGGGTATCGAAC"
##  [23] "CCGGGCGTTATCCGGATTTATTGGGTTTAAAGGGAGCGCAGGCCGTGGGCTAAGCGTGCCGTGAAATTCTGTCGCTCAACGGCAGACGTGCGGCGCGAACTGGTCCACTTGAGTACGCGGGACGTTGGCGGAATTCGTGGTGTAGCGGTGAAATGCTTAGATATCACGAAGAACTCCGATTGCGAAGGCAGCTGACGGTAGCGCAACTGACGCTGAGGCTCGAAAGCGCGGGTATCGAAC"
##  [24] "CCAGGCGTTATCCGGATTCATTGGGTTTAAAGGGAGCGTAGGCCGCCTTTTAAGCGTGTTGTGAAATGTAGGGGCTCAACCCCTGCACTGCAGCGCGAACTGGAGGGCTTGAGTACACACAAAGTAGGCGGAATTCATGGTGTAGCGGTGAAATGCTTAGATATCATGAAGAACCCCGATTGCGAAGGCAGCTTACTGGAGTGTAACTGACGCTGAAGCTCGAAAGCGCGGGTATCGAAC"
##  [25] "GCGAGCGTTGTTCGGAATCATTGGGCGTAAAGGGCATGTAGGCGGTTACGCAAGCCTGGTGTGAAAGCCCGAGGCTCAACCTCGGGATGCGCCGGGAACTGTGCAACTAGAGTAGCTGAGGGGCAGCCGGAATTCCAGGTGTAGGGGTGAAATCTGTAGATATCTGGAAGAACACCGATGGCGAAGGCAGGCTGCCAGCAGATTACTGACGCTGAGGTGCGAAGGTGCGGGGAGCGAACA"
##  [26] "CCAGGCGTTATCCGGATTTATTGGGTTTAAAGGGAGCGCAGGCGGACCTTTAAGTCAGCTGTGAAATACGGCGGCTCAACCGTCGAACTGCAGTTGATACTGGAGGTCTTGAGTGCACACAGGGATACTGGAATTCATGGTGTAGCGGTGAAATGCTCAGATATCATGAAGAACTCCAATCGCGAAGGCAGGTATCCGGGGTGCAACTGACGCTGAGGCTCGAAAGTGCGGGTATCAAAC"
##  [27] "ACAAGCGTTGTCCGGAATTACTGGGTGTAAAGGGAGCGCAGGCGGGAAGGCAAGTTGGAAGTGAAATCCATGGGCTCAACCCATGAACTGCTTTCAAAACTGTTTTTCTTGAGTAGTGCAGAGGTAGGCGGAATTCCCGGTGTAGCGGTGGAATGCGTAGATATCGGGAGGAACACCAGTGGCGAAGGCGGCCTACTGGGCACCAACTGACGCTGAGGCTCGAAAGTGTGGGTAGCAAAC"
##  [28] "CCAGGCGTTATCCGGATTTATTGGGTTTAAAGGGAGCGTAGGCCGCCCCTTAAGCGTGTTGTGAAATGCCGCGGCTCAACCGTGGCACTGCAGCGCGAACTGGGGGGCTTGAGTGCACGCAACGCAGGCGGAATTCGTGGTGTAGCGGTGAAATGCTTAGATATCACGAAGAACTCCGATTGCGAAGGCAGCTTGCGGGAGTGCGACTGACGCTGAAGCTCGAAGGTGCGGGTATCGAAC"
##  [29] "CCGGGCGTTATCCGGATTTATTGGGTTTAAAGGGAGCGTAGGCCGGAGATTAAGCGTGTTGTGAAATGTAGATGCTCAACATCTGCACTGCAGCGCGAACTGGTCTTCTTGAGTACGCACAACGTGGGCGGAATTCGTGGTGTAGCGGTGAAATGCTTAGATATCACGAAGAACTCCGATTGCGAAGGCAGCTCACGGGAGCGCAACTGACGCTGAAGCTCGAAAGTGCGGGTATCGAAC"
##  [30] "GCGAGCGTTGTCCGGAATGACTGGGTGTAAAGGGAGCGTAGGCGGGATGGCAAGTCAGATGTGAAACCTGAGGGCTCAACCTTCAGACTGCATTTGAAACTGCTGTTCTTGAGTGAAGTAGAGGTAAGCGGAATTCCTGGTGTAGCGGTGAAATGCGTAGAGATCAGGAGGAACATCGGTGGCGAAGGCGGCTTACTGGGCTTTTACTGACGCTGAGGCTCGAAAGCGTGGGGAGCAAAC"
##  [31] "CCGGGCGTTATCCGGATTTATTGGGTTTAAAGGGAGCGTAGGCCGGAGATTAAGCGTGTTGTGAAATGTAGACGCTCAACGTCTGCACTGCAGCGCGAACTGGTTTCCTTGAGTACGCACAAAGTGGGCGGAATTCGTGGTGTAGCGGTGAAATGCTTAGATATCACGAAGAACTCCGATTGCGAAGGCAGCTTGCGGGAGTGCGACTGACGCTGAAGCTCGAAGGTGCGGGTATCGAAC"
##  [32] "CCGGGCGTTATCCGGATTTATTGGGTTTAAAGGGAGCGCAGGCCGTGGGTTAAGCGTGTCGTGAAATTCCGTCGCTCAACGGCGGACGTGCGGCGCGAACTGGTCCACTTGAGTACGCGGGACGTTGGCGGAATTCGTGGTGTAGCGGTGAAATGCTTAGATATCACGAAGAACTCCGATTGCGAAGGCAGCTGACGGTAGCGCAACTGACGCTGAGGCTCGAAAGTGCGGGTATCGAAC"
##  [33] "CCGGGCGTTATCCGGATTTATTGGGTTTAAAGGGAGCGTAGGCCGTTTGGTAAGCGTGTTGTGAAATGTCGGGGCTCAACCTGGGCATTGCAGCGCGAACTGCCAGACTTGAGTGCGCAGGAAGTAGGCGGAATTCGTCGTGTAGCGGTGAAATGCTTAGATATGACGAAGAACTCCGATTGCGAAGGCAGCCTGCTGTAGCGCAACTGACGCTGAAGCTCGAAAGCGTGGGTATCGAAC"
##  [34] "CCGGGCGTTATCCGGATTTATTGGGTTTAAAGGGAGCGTAGGCCGTCTGGTAAGCGTGTTGTGAAATGTCGGGGCTCAACCTGGGCATTGCAGCGCGAACTGTCAGACTTGAGTGCGCGGGAAGTAGGCGGAATTCGTCGTGTAGCGGTGAAATGCTTAGATATGACGAAGAACTCCGATTGCGAAGGCAGCCTGCTGTAGCGCAACTGACGCTGAAGCTCGAAAGCGTGGGTATCGAAC"
##  [35] "CCGGGCGTTATCCGGATTTATTGGGTTTAAAGGGAGCGTAGGCCGCCCCTTAAGCGTGTTGTGAAATGCCGCGGCTCAACCGTGGCACTGCAGCGCGAACTGGGGGGCTTGAGTGCACGCAACGCAGGCGGAATTCGTGGTGTAGCGGTGAAATGCTTAGATATCACGAAGAACTCCGATTGCGAAGGCAGCTCACTGGAGCGCAACTGACGCTGAAGCTCGAAAGTGCGGGTATCGAAC"
##  [36] "GCAAGCGTTATCCGGATTTACTGGGTGTAAAGGGAGTGTAGGTGGCCATGCAAGTCAGAAGTGAAAATCCGGGGCTCAACCCCGGAACTGCTTTTGAAACTGTAAGGCTGGAGTGCAGGAGGGGTGAGTGGAATTCCTAGTGTAGCGGTGAAATGCGTAGATATTAGGAGGAACACCAGTGGCGAAGGCGGCTCACTGGACTGTAACTGACACTGAGGCTCGAAAGCGTGGGGAGCAAAC"
##  [37] "CCGGGCGTTATCCGGATTTATTGGGTTTAAAGGGAGCGTAGGCCGGAGATTAAGCGTGTTGTGAAATGTAGTTGCTCAACATCTGCACTGCAGCGCGAACTGGTTTCCTTGAGTACGCACAAAGTGGGCGGAATTCGTGGTGTAGCGGTGAAATGCTTAGATATCACGAAGAACTCCGATTGCGAAGGCAGCTCACTGGAGCGCAACTGACGCTGAAGCTCGAAAGTGCGGGTATCGAAC"
##  [38] "CCGGGCGTTATCCGGATTTATTGGGTTTAAAGGGAGCGTAGGCTGTCCTTTAAGCGTGTTGTGAAATGTAGGCGCTCAACGCCTGCACTGCAGCGCGAACTGAAGGACTTGAGTGTGCACGACGCTGGCGGAATTCGTGGTGTAGCGGTGAAATGCTTAGATATCACGAAGAACTCCGATTGCGAAGGCAGCTGGCGAGAGCACAACTGACGCTGAAGCTCGAAAGTGCGGGTATCGAAC"
##  [39] "ACAAGCGTTGTCCGGAATTACTGGGTGTAAAGGGAGCGCAGGCGGGAGAACAAGTTGGAAGTGAAATCCATGGGCTCAACCCATGAACTGCTTTCAAAACTGTTTTTCTTGAGTAGTGCAGAGGTAGGCGGAATTCCCGGTGTAGCGGTGGAATGCGTAGATATCGGGAGGAACACCAGTGGCGAAGGCGGCCTACTGGGCACCAACTGACGCTGAGGCTCGAAAGTGTGGGTAGCAAAC"
##  [40] "GCAAGCGTTATCCGGATTTACTGGGCGTAAAGGGAGCGTAGGCGGATATTTAAGTGGGATGTGAAATACCCGAGCTTAACTTGGGAGCTGCATTCCAAACTGGATATCTAGAGTGCAGGAGAGGAGAATGGAATTCCTAGTGTAGCGGTGAAATGCGTAGAGATTAGGAAGAACACCAGTGGCGAAGGCGATTCTCTGGACTGTAACTGACGCTGAGGCTCGAAAGCGTGGGGAGCAAAC"
##  [41] "CCGGGCGTTATCCGGATTTATTGGGTTTAAAGGGAGCGCAGGCCGTGGGCTAAGCGTGCCGTGAAATTCTGTCGCTCAACGGCAGACGTGCGGCGCGAACTGGTCCACTTGAGTACGCGGGACGTTGGCGGAATTCGTGGTGTAGCGGTGAAATGCTTAGATATCACGAAGAACTCCGATTGCGAAGGCAGCTCACTGGAGCGCAACTGACGCTGAAGCTCGAAAGTGCGGGTATCGAAC"
##  [42] "GCAAGCGTTATCCGGATTTACTGGGTGTAAAGGGCGCGCAGGCGGGCCGGCAAGTTGGAAGTGAAATCCGGGGGCTTAACCCCCGAACTGCTTTCAAAACTGCTGGTCTTGAGTGATGGAGAGGCAGGCGGAATTCCGTGTGTAGCGGTGAAATGCGTAGATATACGGAGGAACACCAGTGGCGAAGGCGGCCTGCTGGACATTAACTGACGCTGAGGCGCGAAAGCGTGGGGAGCAAAC"
##  [43] "CCGGGCGTTATCCGGATTTATTGGGTTTAAAGGGAGCGTAGGCCGGAGATTAAGCGTGTTGTGAAATGTAGACGCTCAACGTCTGCACTGCAGCGCGAACTGGTTTCCTTGAGTACGCATAAAGTGGGCGGAATTCGTGGTGTAGCGGTGAAATGCTTAGATATCACGAAGAACTCCGATTGCGAAGGCAGCTCACTGGAGCGCAACTGACGCTGAAGCTCGAAAGTGCGGGTATCGAAC"
##  [44] "CCGGGCGTTATCCGGATTTATTGGGTTTAAAGGGAGCGTAGGCCGGAGATTAAGCGTGTTGTGAAATGTAGACGCTCAACGTCTGCACTGCAGCGCGAACTGGTTTCCTTGAGTACGCACAAAGTGGGCGGAATTCGTGGTGTAGCGGTGAAATGCTTAGATATCACGAAGAACTCCGATTGCGAAGGCAGCTGACGGTAGCGCAACTGACGCTGAGGCTCGAAAGCGCGGGTATCGAAC"
##  [45] "CCGGGCGTTATCCGGATTTATTGGGTTTAAAGGGAGCGTAGGCCGTCTTATAAGCGTGTTGTGAAATGTCGGGGCTCAACCTGGGCATTGCAGCGCGAACTGTGAGACTTGAGTGCGCAGGAAGTAGGCGGAATTCGTCGTGTAGCGGTGAAATGCTTAGATATCACGAAGAACTCCGATTGCGAAGGCAGCTCACTGGAGCGCAACTGACGCTGAAGCTCGAAAGTGCGGGTATCGAAC"
##  [46] "CCGGGCGTTATCCGGATTTATTGGGTTTAAAGGGAGCGTAGGCCGTCTGTTAAGCGTGTTGTGAAATGTCGGGGCTCAACCTGGGCATTGCAGCGCGAACTGGCAGACTTGAGTGCGCAGGAAGTAGGCGGAATTCGTCGTGTAGCGGTGAAATGCTTAGATATGACGAAGAACTCCGATTGCGAAGGCAGCCTGCTGTAGCGTAACTGACGCTGAAGCTCGAAAGCGTGGGTATCGAAC"
##  [47] "CCGGGCGTTATCCGGATTTATTGGGTTTAAAGGGAGCGTAGGCCGGAGATTAAGCGTGTTGTGAAATGTAGATGCTCAACATCTGAACTGCAGCGCGAACTGGTTTCCTTGAGTACGCATAAAGTGGGCGGAATTCGTGGTGTAGCGGTGAAATGCTTAGATATCACGAAGAACTCCGATTGCGAAGGCAGCTCACTGGAGCGCAACTGACGCTGAAGCTCGAAAGTGCGGGTATCGAAC"
##  [48] "CCAGGCGTTATCCGGATTTATTGGGTTTAAAGGGAGCGTAGGCCGCCCCTTAAGCGTGTTGTGAAATGCCGCGGCTCAACCGTGGCACTGCAGCGCGAACTGGGGGGCTTGAGTGCACGCAACGCAGGCGGAATTCGTGGTGTAGCGGTGAAATGCTTAGATATCACGAAGAACTCCGATTGCGAAGGCAGCTCACTGGAGCGCAACTGACGCTGAAGCTCGAAAGTGCGGGTATCGAAC"
##  [49] "GCAAGCGTTATCCGGATTTACTGGGTGTAAAGGGCGAGTAGGCGGATTGGCAAGTTGGGAGTGAAATGTCGGGGCTTAACCCCGGAACTGCTTCCAAAACTGTTGATCTTGAGTGATGGAGAGGCAGGCGGAATTCCCAGTGTAGCGGTGAAATGCGTAGATATTGGGAGGAACACCAGTGGCGAAGGCGGCCTGCTGGACATTAACTGACGCTGAGGAGCGAAAGCGTGGGGAGCAAAC"
##  [50] "GCAAGCGTTAATCGGAATAACTGGGCGTAAAGCGCACGTAGGTGGTTCGACAAGTCAGATGTGAAAGCCCCGGGCTTAACCTGGGATGTGCATTTGAAACTGTTGGACTCGAGTACTGTAGAGGGAGGTAGAATTCCAGGTGTAGCGGTGAAATGCGTAGATATCTGGAGGAATACCAGTGGCGAAGGCGGCCTCCTGGACAGACACTGACACTGAGGTGCGAAAGCGTGGGGAGCAAAC"
##  [51] "CCGGGCGTTATCCGGATTTATTGGGTTTAAAGGGAGCGTAGGCTGTCCTTTAAGCGTGTTGTGAAATGTAGGCGCTCAACGCCTGCACTGCAGCGCGAACTGAAGGACTTGAGTGTGCACGACGCTGGCGGAATTCGTGGTGTAGCGGTGAAATGCTTAGATATCACGAAGAACTCCGATTGCGAAGGCAGCTCACTGGAGCGCAACTGACGCTGAAGCTCGAAAGTGCGGGTATCGAAC"
##  [52] "CCGGGCGTTATCCGGATTTATTGGGTTTAAAGGGAGCGCAGGCCGTGGGTTAAGCGTGTCGTGAAATTCCGTCGCTCAACGGCGGACGTGCGGCGCGAACTGGTCCACTTGAGTACGCGGGACGTTGGCGGAATTCGTGGTGTAGCGGTGAAATGCTTAGATATCACGAAGAACTCCGATTGCGAAGGCAGCTCACTGGAGCGCAACTGACGCTGAAGCTCGAAAGTGCGGGTATCGAAC"
##  [53] "GCAAGCGTTATCCGGATTTACTGGGTGTAAAGGGAGCGTAGACGGCGAGACAAGTCTGAAGTGAAAGCCCGGGGCTCAACCCCGGGACTGCTTTGGAAACTGCCTTGCTAGAGTGCTGGAGAGGTAAGTGGAATTCCTAGTGTAGCGGTGAAATGCGTAGATATTAGGAGGAACACCAGTGGCGAAGGCGGCTTACTGGACAGTAACTGACGTTGAGGCTCGAAAGCGTGGGGAGCAAAC"
##  [54] "GCGAGCGTTATCCGGATTTATTGGGTTTAAAGGGTGCGTAGGTCGCCGATTAAGTCAGCGGTGAAATCCAGTGGCTCAACCATCGGACTGCCGTTGAAACTGGCCGGCTTGAGTATGATTGAGGCAGGCGGAATGCGTGGTGTAGCGGTGAAATGCATAGATATCACGCAGAACCCCGATTGCGAAGGCAGCTTGCCAAGCCATGACTGACACTGAAGCACGAAAGCGTGGGTATCAAAC"
##  [55] "GCAAGCGTTATCCGGATTTACTGGGTGTAAAGGGAGCGTAGACGGCACAGCAAGTCTGATGTGAAAGCCCGGGGCCCAACCCCGGAACTGCATTGGAAACTGCTGGGCTTGAGTGCAGGAGAGGTAAGCGGAATTCCTAGTGTAGCGGTGAAATGCGTAGATATTAGGAGGAACACCAGTGGCGAAGGCGGCTTACTGGACTGTAACTGACGTTGAGGCTCGAAAGCGTGGGGAGCAAAC"
##  [56] "GCAAGCGTTATCCGGATTTACTGGGTGTAAAGGGAGCGTAGACGGCATGGCAAGTCTGATGTGAAAATCCCGGGCTCAACCCGGGAACTGCATTGGAAACTGTTAAGCTAGAGTGCAGGAGAGGTAAGTGGAATTCCTAGTGTAGCGGTGAAATGCGTAGATATTAGGAGGAACACCAGTGGCGAAGGCGGCTTACTGGACTGTAACTGACGTTGAGGCTCGAAAGCGTGGGGAGCAAAC"
##  [57] "GCAAGCGTTATCCGGATTTACTGGGTGTAAAGGGAGCGTAGACGGTAAAGCAAGTCTGAAGTGAAAGCCCGGGGCTCAACCGCGGGACTGCTTTGGAAACTGTTTAACTAGAGTGCTGGAGAGGTAAGCGGAATTCCTAGTGTAGCGGTGAAATGCGTAGATATTAGGAGGAACACCAGTGGCGAAGGCGGCTTACTGGACAGTAACTGACGTTGAGGCTCGAAAGCGTGGGGAGCAAAC"
##  [58] "CCGGGCGTTATCCGGATTTATTGGGTTTAAAGGGAGCGTAGGCCGTCTGTTAAGCGTGTTGTGAAATGTCGTGGCTCAACCGGGGCACTGCAGCGCGAACTGGCAGACTTGAGTGCACGGTAGGAAGGCGGAATTCGTCGTGTAGCGGTGAAATGCTTAGATATGACGAAGAACTCCGATTGCGAAGGCAGCTTTCCGTAGTGTAACTGACGCTGAAGCTCGAAAGCGTGGGTATCGAAC"
##  [59] "GCGAGCGTTGTCCGGAATGACTGGGCGTAAAGGGCGTGTAGGCGGCAGTATAAGTCCGGAGTGAAAGTCCTGCTTTCAAGGTGGGAATTGCTTTGGAGACTGTACAGCTTGAGTGCGGAAGAGGTAAGTGGAATTCCCAGTGTAGCGGTGAAATGCGTAGAGATTGGGAGGAACACCAGTGGCGAAGGCGACTTACTGGGCCGTAACTGACGCTGAGGCGCGAAAGCGTGGGGAGCGAAC"
##  [60] "GCAAGCGTTGTCCGGATTTACTGGGTGTAAAGGGCGTGTAGGCGGGCATGCAAGTTGGATGTGAAATGTCACGGCTTAACCGTGGAGCTGCATCCAAAACTGCAAGTCTTGAGTGCCGGAGAGGAAAGCGGAATTCCTAGTGTAGCGGTGAAATGCGTAGATATTAGGAGGAACACCGGTGGCGAAGGCGGCTTTCTGGACGGTAACTGACGCTGAGGCGCGAAAGCGTGGGGAGCAAAC"
##  [61] "GCAAGCGTTATCCGGATTTACTGGGTGTAAAGGGAGCGTAGACGGCGATGCAAGTCTGAAGTGAAATACCCGGGCTCAACCTGGGAACTGCTTTGGAAACTGTATTGCTAGAGTGCTGGAGAGGTAAGCGGAATTCCTAGTGTAGCGGTGAAATGCGTAGATATTAGGAAGAACACCAGTGGCGAAGGCGGCTTACTGGACAGTAACTGACGTTGAGGCTCGAAAGCGTGGGGAGCAAAC"
##  [62] "CCAGGCGTTATCCGGATTTATTGGGTTTAAAGGGAGCGTAGGCCGTTTTTTAAGCGTGTTGTGAAATACTGTCGCTCAACGACAGAGGTGCAGCGCGAACTGGAGGACTTGAGTGCGCGGAATGTAGGCGGAATTCGTCGTGTAGCGGTGAAATGCTTAGATATGACGAAGAACTCCGATTGCGAAGGCAGCTTACAGTAGCGTAACTGACGCTGAAGCTCGAAAGTGCGGGTATCGAAC"
##  [63] "GCAAGCGTTATCCGGATTTACTGGGTGTAAAGGGAGTGTAGGTGGCCAGGCAAGTCAGAAGTGAAAGCCCGGGGCTCAACCCCGGGACTGCTTTTGAAACTGCAGGGCTAGAGTGCAGGAGGGGCAAGTGGAATTCCTAGTGTAGCGGTGAAATGCGTAGATATTAGGAGGAACACCAGTGGCGAAGGCGGCTTGCTGGACTGTAACTGACACTGAGGCTCGAAAGCGTGGGGAGCAAAC"
##  [64] "GCAAGCGTTAATCGGAATCACTGGGCGTAAAGCGCACGTAGGCTGTTATGTAAGTCAGGGGTGAAATCCCACGGCTCAACCGTGGAACTGCCCTTGATACTGCACGACTTGAATCCGGGAGAGGGTGGCGGAATTCCAGGTGTAGGAGTGAAATCCGTAGATATCTGGAGGAACATCAGTGGCGAAGGCGGCCACCTGGACCGGTATTGACGCTGAGGTGCGAAAGCGTGGGGAGCAAAC"
##  [65] "CCAGGCGTTATCCGGATTTATTGGGTTTAAAGGGAGCGTAGGCCGTTTGGTAAGCGTGTTGTGAAATGTCGGGGCTCAACCTGGGCATTGCAGCGCGAACTGCCAGACTTGAGTGCGCAGGAAGTAGGCGGAATTCGTCGTGTAGCGGTGAAATGCTTAGATATGACGAAGAACTCCGATTGCGAAGGCAGCCTGCTGTAGCGCAACTGACGCTGAAGCTCGAAAGCGTGGGTATCGAAC"
##  [66] "GCAAGCGTTGTCCGGAATCATTGGGCGTAAAGAGTTCGTAGGCGGTTTGTTAAGTCTGGTGTTAAAGCCCGAAGCTCAACTTCGGTTCGGCACTGGATACTGGCAGACTAGAATGCGGTAGAGGTAAAGGGAATTCCTGGTGTAGCGGTGAAATGCGTAGATATCAGGAGGAACATCGGTGGCGTAAGCGCTTTACTGGGCCGTAATTGACGCTGAGGAACGAAAGCCAGGGTAGCGAAT"
##  [67] "GCAAGCGTTATCCGGATTTACTGGGTGTAAAGGGAGCGTAGACGGTTAAGCAAGTCAGAAGTGAAAGGCTGGGGCTCAACCCCGGGACTGCTTTTGAAACTGTTTAACTAGAGTGCTGGAGAGGTAAGCGGAATTCCTAGTGTAGCGGTGAAATGCGTAGATATTAGGAGGAACACCAGTGGCGAAGGCGGCTTACTGGACAGTAACTGACGTTGAGGCTCGAAAGCGTGGGGAGCAAAC"
##  [68] "GCAAGCGTTGTCCGGAATTACTGGGTGTAAAGGGAGCGCAGGCGGGCATGCAAGTTGGAAGTGAAAACTATGGGCTCAACCCATAGCCTGCTTTCAAAACTGCGTGTCTTGAGTAGTGCAGAGGTAGGCGGAATTCCCGGTGTAGCGGTGGAATGCGTAGATATCGGGAGGAACACCAGTGGCGAAGGCGGCCTACTGGGCACCAACTGACGCTGAGGCTCGAAAGCATGGGTAGCAAAC"
##  [69] "GCGAGCGTTATCCGGAATTACTGGGTGTAAAGGGTGTGTAGGCGGGAAGGCAAGTCAGATGTGAAAACCAAAGGCTCAACCTTTGGCTTGCATTTGAAACTGTTTTTCTTGAGAGTGGGAGAGGTAAACGGAATTCCTAGTGTAGTAGTGAAATGCGTAGATATTAGGAGGAACACCGGTGGCGAAGGCGGTTTACTGGACCACAACTGACGCTGAGACACGAAAGCGTGGGGAGCAAAC"
##  [70] "GCAAGCGTTATCCGGAATTACTGGGTGTAAAGGGAGCGTAGGCGGCATGGTAAGTAAGATGTGAAAGCCCGAGGCTTAACCTCGAGGATTGCATTTTAAACTATCAAGCTAGAGTACAGGAGAGGAAAGCGGAATTCCTAGTGTAGCGGTGAAATGCGTAGATATTAGGAAGAACACCAGTGGCGAAGGCGGCTTTCTGGACTGAAACTGACGCTGAGGCTCGAAAGCGTGGGGAGCGAA"
##  [71] "GCAAGCGTTATCCGGATTTACTGGGTGTAAAGGGAGCGTAGACGGTCAAGCAAGTCAGAAGTGAAAGGCTGGGGCTCAACCCCGGGACTGCTTTTGAAACTGTTTGACTGGAGTGCTGGAGAGGTAAGCGGAATTCCTAGTGTAGCGGTGAAATGCGTAGATATTAGGAGGAACACCAGTGGCGAAGGCGGCTTACTGGACAGTAACTGACGTTGAGGCTCGAAAGCGTGGGGAGCAAAC"
##  [72] "GCAAGCGTTATCCGGATTTACTGGGTGTAAAGGGAGCGTAGACGGCGACGCAAGTCTGAAGTGAAATACCCGGGCTCAACCTGGGAACTGCTTTGGAAACTGTGTTGCTAGAGTGCTGGAGAGGTAAGCGGAATTCCTAGTGTAGCGGTGAAATGCGTAGATATTAGGAAGAACACCAGTGGCGAAGGCGGCTTACTGGACAGTAACTGACGTTGAGGCTCGAAAGCGTGGGGAGCAAAC"
##  [73] "GCAAGCGTTGTCCGGATTTACTGGGTGTAAAGGGAGCGCAGGCGGGAGAGCAAGTCAGCGGTGAAATACATGGGCTTAACCCATGGGCTGCCGTTGAAACTGTCCTTCTTGAGTGAAGTAGAGGCAAGCGGAATTCCGAGTGTAGCGGTGAAATGCGTAGATATTCGGAGGAACACCAGTGGCGAAGGCGGCTTGCTGGGCTTTTACTGACGCTGAGGCTCGAAAGTGTGGGGAGCAAAC"
##  [74] "GCAAGCGTTATCCGGATTTACTGGGTGTAAAGGGAGCGCAGGCGGTCTGGCAAGTCTGATGTGAAATCCCGGGGCTCAACCCTGGAACTGCATTGGAAACTGTCAGACTAGAGTGCCGGAGAGGTAAGTGGAATTCCTAGTGTAGCGGTGAAATGCGTAGATATTAGGAGGAACACCAGTGGCGAAGGCGGCTTACTGGACGGTAACTGACGCTGAGGCTCGAAAGCGTGGGGAGCAAAC"
##  [75] "GCAAGCGTTATCCGGATTTACTGGGTGTAAAGGGAGCGTAGACGGCTAAGCAAGTCAGAAGTGAAAGGCTGGGGCTCAACCCCGGGACTGCTTTTGAAACTGTTTGGCTAGAGTGCTGGAGAGGTAAGCGGAATTCCTAGTGTAGCGGTGAAATGCGTAGATATTAGGAGGAACACCAGTGGCGAAGGCGGCTTACTGGACAGTAACTGACGTTGAGGCTCGAAAGCGTGGGGAGCAAAC"
##  [76] "GCAAGCGTTACTCGGAATTACTAGGCGTAAAGCGCGCGTAGGCGGAATGTTAAGTCTGTTGTGTAATCTCTGGGCTCAACCCAGAAACTGCAACAGAAACTGGCGTTCTTGAGTGAGGCAGAGGAAATCGGAATTCCTAGTGTAGCAGTGAAATGCGTAGATATTAGGAGGAACACCGGTGGCGAAGGCGGATTTCTGGGCCTTTACTGACGCTAAAGTGCGAAAGCTAGGGGAGCAAAC"
##  [77] "GCGAGCGTTATCCGGATTCATTGGGCGTAAAGCGCGCGTAGGCGGATGCCTAAGCGGGACCTCTAACCCGGGGGCTCAACCCCCGGCCGGGTCCCGAACTGGGCGTCTCGAGTGCGGTAGGGGCAGGTGGAATTCCATGTGTAGCGGTGGAATGCGCAGATATATGGAAGAACACCGACGGCGAAGGCAGCCTGCTGGGCCGACACTGACGCTGAGGTGCGAAAGCGCGGGGAGCGAACA"
##  [78] "GCAAGCGTTATCCGGATTTACTGGGTGTAAAGGGAGCGTAGACGGTAGTGCAAGTCTGATGTGAAAGCCCGGGGCTCAACCCCGGGACTGCATTGGAAACTGTATAACTAGAGTGTCGGAGAGGTAAGCGGAATTCCTAGTGTAGCGGTGAAATGCGTAGATATTAGGAGGAACACCAGTGGCGAAGGCGGCTTACTGGACGATGACTGACGTTGAGGCTCGAAAGCGTGGGGAGCAAAC"
##  [79] "GCAAGCGTTGTCCGGAATTACTGGGTGTAAAGGGAGCGTAGGCGGGAAGATAAGTTGGACGTCTAATCTATCGGCTCAACCGATAGTCGCGTTCAAAACTGTTTTTCTTGAGTGAAGTAGAGGTAAGCGGAATTCCTAGTGTAGCGGTGAAATGCGTAAATATTAGGAGGAACACCAGTGGCGAAGGCGGCTTACTGGGCTTTAACTGACGCTGAGGCTCGAAAGCGTGGGTAGCAAACA"
##  [80] "GCGAGCGTTATCCGGATTTATTGGGTGTAAAGGGTGCGTAGACGGGAAATTAAGTTAGTTGTGAAATCCCTCGGCTCAACTGAGGAACTGCAACTAAAACTGATTTTCTTGAGTACTGGAGAGGAAAGTGGAATTCCTAGTGTAGCGGTGAAATGCGTAGATATTAGGAGGAACACCAGTGGCGAAGGCGACTTTCTGGACAGAAACTGACGTTGAGGCACGAAAGTGTGGGGAGCAAAC"
##  [81] "GCGAGCGTTGTCCGGAATTATTGGGCGTAAAGAGCTTGTAGGCGGTTTGTCGCGTCTGCTGTGAAAGGCCGGGGCTTAACCCCGTGTATTGCAGTGGGTACGGGCAGACTAGAGTGCAGTAGGGGAGACTGGAATTCCTGGTGTAGCGGTGGAATGCGCAGATATCAGGAGGAACACCGATGGCGAAGGCAGGTCTCTGGGCTGTAACTGACGCTGAGAAGCGAAAGCATGGGGAGCGAA"
##  [82] "GCAAGCGTTATCCGGATTTATTGGGTGTAAAGGGTGCGTAGACGGGAATACAAGTTAGTTGTGAAATCCCTCGGCTTAACTGAGGAACTGCAACTAAAACTATATTTCTTGAGTGCTGGAGAGGAAAGTGGAATTCCTAGTGTAGCGGTGAAATGCGTAGATATTAGGAGGAACACCAGTGGCGAAGGCGACTTTCTGGACAGTAACTGACGTTGAGGCACGAAAGTGTGGGGAGCAAAC"
##  [83] "CCGAGCGTTATCCGGATTTATTGGGTTTAAAGGGAGCGTAGGTGGATTGTTAAGTCAGTTGTGAAAGTTTGCGGCTCAACCGTAAAATTGCAGTTGAAACTGGCAGTCTTGAGTACAGTAGAGGTGGGCGGAATTCGTGGTGTAGCGGTGAAATGCTTAGATATCACGAAGAACTCCGATTGCGAAGGCAGCTCACTAGACTGCAACTGACACTGATGCTCGAAAGTGTGGGTATCAAAC"
##  [84] "GCAAGCGTTATCCGGATTTACTGGGTGTAAAGGGAGCGTAGGCGGTATGGCAAGTCTGATGTGAAAGGCCGGGGCTCAACCCCGGGACTGCATTGGAAACTGTCACACTTGAGTGTCGGAGAGGTAAGTGGAATTCCTAGTGTAGCGGTGAAATGCGTAGATATTAGGAGGAACACCAGTGGCGAAGGCGGCTTACTGGACGACAACTGACGCTGAGGCTCGAAAGCGTGGGGAGCAAAC"
##  [85] "GCAAGCGTTATCCGGATTTATTGGGTGTAAAGGGTGCGTAGACGGGAAATTAAGTTAGTTGTGAAATCCCTCGGCTCAACTGAGGAACTGCAACTAAAACTGGTTTTCTTGAGTGCAGGAGAGGTAAGTGGAATTCCTAGTGTAGCGGTGAAATGCGTAGATATTAGGAGGAACACCAGTGGCGAAGGCGACTTACTGGACTGTAACTGACGTTGAGGCACGAAAGTGTGGGGAGCAAAC"
##  [86] "GCAAGCGTTGTCCGGAATTACTGGGTGTAAAGGGAGCGCAGGCGGGAAGACAAGTTGGAAGTGAAAACCATGGGCTCAACCCATGAATTGCTTTCAAAACTGCTGGCCTTGAGTAGTGCAGAGGTAGGTGGAATTCCCGGTGTAGCGGTGGAATGCGTAGATATCGGGAGGAACACCAGTGGCGAAGGCGGTCTACTGGGCACCAACTGACGCTGAGGCTCGAAAGCATGGGTAGCAAAC"
##  [87] "GCAAGCGTTATCCGGATTTACTGGGTGTAAAGGGAGCGTAGACGGCTGTGTAAGTCTGAAGTGAAAGCCCGGGGCTCAACCGCGGGACTGCTTTGGAAACTATGCAGCTAGAGTGTCGGAGAGGTAAGTGGAATTCCCAGTGTAGCGGTGAAATGCGTAGATATTGGGAGGAACACCAGTGGCGAAGGCGGCTTACTGGACGATGACTGACGTTGAGGCTCGAAAGCGTGGGGAGCAAAC"
##  [88] "GCGAGCGTTATCCGGATTCATTGGGCGTAAAGCGCGCGTAGGCGGCCGCTCGAGCGGGACCTCTAACCCGGGGGCTCAACCTCCGGCCGGGTCCCGGACCGTGCGGCTCGGGTGCGGTAGGGGCAGGCGGAACTCCAAGTGTAGCGGTGAAATGCGCAGATATTTGGAGGAACACCGATGGCGAAGGCAGCCTGCTGGGCCGCCACCGACGCTGAGGCGCGAAAGCCGGGGGAGCGAACA"
##  [89] "GCAAGCGTTATCCGGAATTACTGGGTGTAAAGGGTGCGTAGGTGGTATGGCAAGTCAGAAGTGAAAACCCAGGGCTTAACTCTGGGACTGCTTTTGAAACTGTCAGACTAGAGTGCAGGAGAGGTAAGCGGAATTCCTAGTGTAGCGGTGAAATGCGTAGATATTAGGAGGAACATCAGTGGCGAAGGCGGCTTACTGGACTGAAACTGACACTGAGGCACGAAAGCGTGGGGAGCAAAC"
##  [90] "GCAAGCGTTATCCGGATTTACTGGGTGTAAAGGGAGCGTAGACGGTGTGGCAAGTCTGATGTGAAAGGCATGGGCTCAACCTGTGGACTGCATTGGAAACTGTCATACTTGAGTGCCGGAGGGGTAAGCGGAATTCCTAGTGTAGCGGTGAAATGCGTAGATATTAGGAGGAACACCAGTGGCGAAGGCGGCTTACTGGACGGTAACTGACGTTGAGGCTCGAAAGCGTGGGGAGCAAAC"
##  [91] "GCAAGCGTTATCCGGAATTATTGGGCGTAAAGGGCTCGTAGGCGGTTCGTCGCGTCCGGTGTGAAAGTCCATCGCTTAACGGTGGATCTGCGCCGGGTACGGGCGGGCTGGAGTGCGGTAGGGGAGACTGGAATTCCCGGTGTAACGGTGGAATGTGTAGATATCGGGAAGAACACCGACGGCGAAGGCAGCTCTCTGGGCCGAAACTGACGCTGAGGCGCGAAAGCTGGGGGAGCGAAC"
##  [92] "CCAAGCGTTATCCGGATTTATTGGGCGTAAAGCGAGCGCAGACGGTTATTTAAGTCTGAAGTGAAAGCCCTCAGCTCAACTGAGGAATTGCTTTGGAAACTGGATGACTTGAGTGCAGTAGAGGAAAGTGGAACTCCATGTGTAGCGGTGAAATGCGTAGATATATGGAAGAACACCAGTGGCGAAGGCGGCTTTCTGGACTGTAACTGACGTTGAGGCTCGAAAGTGTGGGTAGCAAAC"
##  [93] "GCAAGCGTTATCCGGAATTATTGGGCGTAAAGAGTACGTAGGTGGTTACCTAAGCACGAGGTATAAGGCAATGGCTTAACCATTGTTCGCCTTGTGAACTGGGCTACTTGAGTGCAGGAGAGGAAAGCGGAATTCCTAGTGTAGCGGTGAAATGCGTAGATATTAGGAGGAACACCAGTGGCGAAGGCGGCTTTCTGGACTGTAACTGACACTGAGGTACGAAAGCGTGGGGAGCAAACA"
##  [94] "GCAAGCGTTATCCGGATTTACTGGGTGTAAAGGGAGCGTAGACGGCACGGCAAGCCAGATGTGAAAGCCCGGGGCTCAACCCCGGGACTGCATTTGGAACTGCTGAGCTAGAGTGTCGGAGAGGCAAGTGGAATTCCTAGTGTAGCGGTGAAATGCGTAGATATTAGGAGGAACACCAGTGGCGAAGGCGGCTTGCTGGACGATGACTGACGTTGAGGCTCGAAAGCGTGGGGAGCAAAC"
##  [95] "GCAAGCGTTATCCGGAATTATTGGGCGTAAAGAGTACGTAGGTGGTTTTCTAAGCACGGGGTTTAAGGCAATGGCTTAACCATTGTTCGCCTTGTGAACTGGAAGACTTGAGTGCAGGAGAGGAAAGCGGAATTCCTAGTGTAGCGGTGAAATGCGTAGATATTAGGAGGAACACCAGTGGCGAAGGCGGCTTTCTGGACTGTAACTGACACTGAGGTACGAAAGCGTGGGGAGCAAACA"
##  [96] "GCAAGCGTTATCCGGATTTACTGGGTGTAAAGGGAGCGTAGACGGCTTTGCAAGTCTGATGTGAAAGGCGGGGGCTCAACCCCTGGACTGCATTGGAAACTGTGAGGCTTGAGTGCCGGAGAGGTAAGCGGAATTCCTAGTGTAGCGGTGAAATGCGTAGATATTAGGAGGAACACCAGTGGCGAAGGCGGCTTACTGGACGGTAACTGACGTTGAGGCTCGAAAGCGTGGGGAGCAAAC"
##  [97] "GCAAGCGTTGTCCGGAATTACTGGGTGTAAAGGGAGCGCAGGCGGACCGGCAAGTTGGAAGTGAAAACTATGGGCTCAACCCATAAATTGCTTTCAAAACTGCTGGCCTTGAGTAGTGCAGAGGTAGGTGGAATTCCCGGTGTAGCGGTGGAATGCGTAGATATCGGGAGGAACACCAGTGGCGAAGGCGACCTACTGGGCACCAACTGACGCTGAGGCTCGAAAGCATGGGTAGCAAAC"
##  [98] "GCAAGCGTTGTCCGGAATTACTGGGTGTAAAGGGAGCGCAGGCGGACCGGCAAGTTGGAAGTGAAAACCATGGGCTCAACCCGTGAATTGCTTTCAAAACTGCTGGCCTTGAGTAGTGCAGAGGTAGGTGGAATTCCCGGTGTAGCGGTGGAATGCGTAGATATCGGGAGGAACACCAGTGGCGAAGGCGACCTACTGGGCACCAACTGACGCTGAGGCTCGAAAGCATGGGTAGCAAAC"
##  [99] "GCGAGCGTTATCCGGAATCATTGGGCGTAAAGCGCGCGCAGGCGGGCTTTCAAGCGGCGGCGTCGAAGCCGGGGGCTCAACCCCCGGAAGCGCCCCGAACTGGAAGCCTCGGATGCGGCAGGGGGAGGCGGAATTCCCGGTGTAGCGGTGAAATGCGCAGATATCGGGAAGAACACCGACGGCGAAGGCAGCCTCCTGGGCCGGCATCGACGCTGAGGCGCGAAAGCTGGGGGAGCGAAC"
## [100] "GCGAGCGTTATCCGGATTCATTGGGCGTAAAGCGCGCGTAGGCGGAGCGCTAAGCGGGACCTCTAACCCGAGGGCTCAACCCCCGGCCGGGTCCCGAACTGGCGCTCTCGAGTGCGGTAGGGGAGAGCGGAATTCCCGGTGTAGCGGTGGAATGCGCAGATATCGGGAAGAACACCGACGGCGAAGGCAGCTCTCTGGGCCGAAACTGACGCTGAGGCGCGAAAGCTGGGGGAGCGAACA"
## [101] "GCTAGCGTTATCCGGATTTACTGGGCGTAAAGGGTGCGTAGGCGGTCTTTTAAGTCAGGAGTGAAAGGCTACGGCTCAACCGTAGTAAGCTCTTGAAACTGGAGGACTTGAGTGCAGGAGAGGAGAGTGGAATTCCTAGTGTAGCGGTGAAATGCGTAGATATTAGGAGGAACACCAGTAGCGAAGGCGGCTCTCTGGACTGTAACTGACGCTGAGGCACGAAAGCGTGGGGAGCAAACA"
## [102] "GCAAGCGTTATCCGGAATTACTGGGTGTAAAGGGTGCGTAGGTGGTATGGCAAGTCAGAAGTGAAAACCCAGGGCTTAACTCTGGGACTGCTTTTGAAACTGTCAGACTGGAGTGCAGGAGAGGTAAGCGGAATTCCTAGTGTAGCGGTGAAATGCGTAGATATTAGGAGGAACATCAGTGGCGAAGGCGGCTTACTGGACTGAAACTGACACTGAGGCACGAAAGCGTGGGGAGCAAAC"
## [103] "GCGAGCGTTATCCGGAATGATTGGGCGTAAAGCGCGCGCAGGCGGCCGCTCAAGCGGGACCTCTAACCCCGGGGCTCAACCCCGGGCCGGGTCCCGAACTGGGCGGCTCGAGTGCGGTAGGGGAGAGCGGAATTCCAAGTGTAGCGGTGAAATGCGCAGATATTTGGAAGAACACCGATGGCGAAGGCAGCTCTCTGGGCCGTCACTGACGCTGAGGCGCGAAAGCCGGGGGAGCGAACA"
## [104] "GCGAGCGTTGTCCGGAATTATTGGGCGTAAAGGGCTTGTAGGCGGTTGGTCGCGTCTGCCGTGAAATCCTCTGGCTTAACTGGGGGCGTGCGGTGGGTACGGGCTGACTTGAGTGCGGTAGGGGAGACTGGAACTCCTGGTGTAGCGGTGGAATGCGCAGATATCAGGAAGAACACCGGTGGCGAAGGCGGGTCTCTGGGCCGTTACTGACGCTGAGGAGCGAAAGCGTGGGGAGCGAAC"
## [105] "GCAAGCGTTATCCGGATTTACTGGGTGTAAAGGGAGCGTAGACGGAATGGCAAGTCTGATGTGAAAGGCCGGGGCTCAACCCCGGGACTGCATTGGAAACTGTCAATCTAGAGTACCGGAGGGGTAAGTGGAATTCCTAGTGTAGCGGTGAAATGCGTAGATATTAGGAGGAACACCAGTGGCGAAGGCGGCTTACTGGACGGTAACTGACGTTGAGGCTCGAAAGCGTGGGGAGCAAAC"
## [106] "TCTAGTGGTAGCAGTTTTTATTGGGCCTAAAGCGTCCGTAGCCGGTTTAATAAGTCTCTGGTGAAATCCTGCAGCTTAACTGTGGGAATTGCTGGAGATACTATTAGACTTGAGATCGGGAGAGGTTAGAGGTACTCCCAGGGTAGAGGTGAAATTCTGTAATCCTGGGAGGACCGCCTGTTGCGAAGGCGTCTGACTGGAACGATTCTGACGGTGAGGGACGAAAGCTAGGGGCGCGAA"
## [107] "GCAAGCGTTATCCGGATTTACTGGGTGTAAAGGGAGCGTAGACGGTATGGCAAGTCTGATGTGAAAGGCCAGGGCTCAACCCTGGGACTGCATTGGAAACTGTCGAACTAGAGTGTCGGAGAGGCAAGTGGAATTCCTAGTGTAGCGGTGAAATGCGTAGATATTAGGAGGAACACCAGTGGCGAAGGCGGCTTGCTGGACGATGACTGACGTTGAGGCTCGAAAGCGTGGGGAGCAAAC"
## [108] "GCGAGCGTTATCCGGATTCATTGGGCGTAAAGCGCGCGTAGGCGGAGCGCTAAGCGGGACCTCTAACCCGAGGGCTCAACCCCCGGCCGGGTCCCGAACTGGCGCTCTCGAGTGCGGTAGGGGAGAGCGGAATTCCCGGTGTAGCGGTGGAATGCGCAGATATCGGGAGGAACACCGACGGCGAAGGCAGCTCTCTGGGCCGAAACTGACGCTGAGGCGCGAAAGCTGGGGGAGCGAACA"
## [109] "GCAAGCGTTATCCGGATTTACTGGGTGTAAAGGGAGCGTAGACGGCTGTGCAAGTCTGAAGTGAAAGGCATGGGCTCAACCTGTGGACTGCTTTGGAAACTGTGCAGCTAGAGTGTCGGAGAGGTAAGTGGAATTCCTAGTGTAGCGGTGAAATGCGTAGATATTAGGAGGAACACCAGTGGCGAAGGCGGCTTACTGGACGATGACTGACGTTGAGGCTCGAAAGCGTGGGGAGCAAAC"
## [110] "GCTAGCGTTATCCGGAATTACTGGGCGTAAAGGGTGCGTAGGTGGTTTCTTAAGTCAGAGGTGAAAGGCTACGGCTCAACCGTAGTAAGCCTTTGAAACTGGGAAACTTGAGTGCAGGAGAGGAGAGTGGAATTCCTAGTGTAGCGGTGAAATGCGTAGATATTAGGAGGAACACCAGTTGCGAAGGCGGCTCTCTGGACTGTAACTGACACTGAGGCACGAAAGCGTGGGGAGCAAACA"
## [111] "GCGAGCGTTATCCGGATTCATTGGGCGTAAAGCGCGCGTAGGCGGCCCGTCAAGCGGGGTTTCAAATCCAGGGGCTCAACCTCTGGCCGGACCCCGAACTGGCGGGCTCGAGTGCGGTAGAGGAAGGTGGAATTCCCAGTGTAGCGGTGAAATGCGCAGATATTGGGAAGAACACCGATGGCGAAGGCAGCCTTCTGGGCCGCCACTGACGCTGAGGCGCGAAAGCTAGGGGAGCGAACA"
## [112] "GCAAGCGTTATCCGGATTTACTGGGTGTAAAGGGAGCGTAGACGGCGCAGCAAGTCTGATGTGAAAGGCAGGGGCTTAACCCCTGGACTGCATTGGAAACTGCTGTGCTTGAGTGCCGGAGGGGTAAGCGGAATTCCTAGTGTAGCGGTGAAATGCGTAGATATTAGGAGGAACACCAGTGGCGAAGGCGGCTTACTGGACGGTAACTGACGTTGAGGCTCGAAAGCGTGGGGAGCAAAC"
## [113] "GCAAGCGTTATCCGGATTTACTGGGTGTAAAGGGAGCGTAGACGGATTAGCAAGTCTGATGTGAAAGGCAGGGGCTCAACCCCTGGACTGCATTGGAAACTGCCAGTCTTGAGTGCCGGAGAGGTAAGCGGAATTCCTAGTGTAGCGGTGAAATGCGTAGATATTAGGAGGAACACCAGTGGCGAAGGCGGCTTACTGGACGGCAACTGACGTTGAGGCTCGAAAGCGTGGGGAGCAAAC"
## [114] "TCAAGCGTTGTTCGGAATCACTGGGCGTAAAGCGTGCGTAGGCTGTTTCGTAAGTCGTGTGTGAAAGGCGCGGGCTCAACCCGCGGACGGCACATGATACTGCGAGACTAGAGTAATGGAGGGGGAACCGGAATTCTCGGTGTAGCAGTGAAATGCGTAGATATCGAGAGGAACACTCGTGGCGAAGGCGGGTTCCTGGACATTAACTGACGCTGAGGCACGAAGGCCAGGGGAGCGAAA"
## [115] "GCGAGCGTTATCCGGATTCATTGGGCGTAAAGCGCGCGTAGGCGGCCCGGCAGGCCGGGGGTCGAAGCGGGGGGCTCAACCCCCCGAAGCCCCCGGAACCTCCGCGGCTTGGGTCCGGTAGGGGAGGGTGGAACACCCGGTGTAGCGGTGGAATGCGCAGATATCGGGTGGAACACCGGTGGCGAAGGCGGCCCTCTGGGCCGAGACCGACGCTGAGGCGCGAAAGCTGGGGGAGCGAAC"
## [116] "GCAAGCGTTGTCCGGAATTACTGGGTGTAAAGGGAGCGCAGGCGGACCGGCAAGTTGGAAGTGAAATCCATGGGCTCAACCCGTGAATTGCTTTCAAAACTGCTGGCCTTGAGTAGTGCAGAGGTAGGTGGAATTCCCGGTGTAGCGGTGGAATGCGTAGATATCGGGAGGAACACCAGTGGCGAAGGCGACCTACTGGGCACCAACTGACGCTGAGGCTCGAAAGCATGGGTAGCAAAC"
## [117] "GCAAGCGTTGTCCGGAATTACTGGGTGTAAAGGGAGCGCAGGCGGGAAGACAAGTTGGAAGTGAAAACCATGGGCTCAACCCATGAATTGCTTTCAAAACTGTTTTTCTTGAGTAGTGCAGAGGTAGATGGAATTCCCGGTGTAGCGGTGGAATGCGTAGATATCGGGAGGAACACCAGTGGCGAAGGCGGTCTACTGGGCACCAACTGACGCTGAGGCTCGAAAGCATGGGTAGCAAAC"
## [118] "GCAAGCGTTATCCGGAATTATTGGGCGTAAAGGGCTCGTAGGCGGTTCGTCGCGTCCGGTGTGAAAGTCCATCGCTTAACGGTGGATCCGCGCCGGGTACGGGCGGGCTTGAGTGCGGTAGGGGAGACTGGAATTCCCGGTGTAACGGTGGAATGTGTAGATATCGGGAAGAACACCAATGGCGAAGGCAGGTCTCTGGGCCGTTACTGACGCTGAGGAGCGAAAGCGTGGGGAGCGAAC"
## [119] "GCAAGCGTTATCCGGAATTATTGGGCGTAAAGGGCTCGTAGGCGGTTCGTCGCGTCCGGTGTGAAAGTCCATCGCTTAACGGTGGATCTGCGCCGGGTACGGGCGGGCTGGAGTGCGGTAGGGGAGACTGGAATTCCCGGTGTAACGGTGGAATGTGTAGATATCGGGAAGAACACCAATGGCGAAGGCAGGTCTCTGGGCCGTTACTGACGCTGAGGAGCGAAAGCGTGGGGAGCGAAC"
## 
## $xy
## [1] "GCAAGCGTTATCCGGATTTACTGGGTGTAAAGGGAGCGTAGACGGCCGTGCAAGTCTGATGTGAAAGGCTGGGGCTCAACCCCGGGACTGCATTGGAAACTGTATGGCTGGAGTGCCGGAGAGGTAAGCGGAATTCCTAGTGTAGCGGTGAAATGCGTAGATATTAGGAGGAACACCAGTGGCGAAGGCGGCTTACTGGACGGTAACTGACGTTGAGGCTCGAAAGCGTGGGGAGCAAAC"
## [2] "GCAAGCGTTATCCGGATTTACTGGGTGTAAAGGGAGCGCAGACGGCACTGCAAGTCTGAAGTGAAAGCCCGGGGCTCAACCCCGGGACTGCTTTGGAAACTGTAGAGCTAGAGTGCTGGAGAGGCAAGCGGAATTCCTAGTGTAGCGGTGAAATGCGTAGATATTAGGAGGAACACCAGTGGCGAAGGCGGCTTACTGGACGGTAACTGACGTTGAGGCTCGAAAGCGTGGGGAGCAAAC"
## [3] "GCAAGCGTTATCCGGATTTACTGGGTGTAAAGGGCGCGTAGGCGGGGATGCAAGTCAGATGTGAAATCTATGGGCTTAACCCATAAACTGCATTTGAAACTGTATCTCTTGAGTGCTGGAGAGGTAGACGGAATTCCTTGTGTAGCGGTGAAATGCGTAGATATAAGGAAGAACACCAGTGGCGAAGGCGGTCTACTGGACAGTAACTGACGCTGAGGCGCGAGAGCGTGGGGAGCAAAC"
## 
## $xz
## character(0)
## 
## $yz
## [1] "ACAAGCGTTGTCCGGAATTACTGGGTGTAAAGGGAGCGCAGGCGGGCGATCAAGTTGGAAGTGAAATCCATGGGCTCAACCCATGAACTGCTTTCAAAACTGGTCGTCTTGAGTAGTGCAGAGGTAGGCGGAATTCCCGGTGTAGCGGTGGAATGCGTAGATATCGGGAGGAACACCAGTGGCGAAGGCGGCCTACTGGGCACCAACTGACGCTGAGGCTCGAAAGTGTGGGTAGCAAAC"
## [2] "ACAAGCGTTGTCCGGAATTACTGGGTGTAAAGGGAGCGCAGGCGGGAAGACAAGTTGGAAGTGAAATCCATGGGCTCAACCCATGAACTGCTTTCAAAACTGTTTTTCTTGAGTAGTGCAGAGGTAGGCGGAATTCCCGGTGTAGCGGTGGAATGCGTAGATATCGGGAGGAACACCAGTGGCGAAGGCGGCCTACTGGGCACCAACTGACGCTGAGGCTCGAAAGTGTGGGTAGCAAAC"
## [3] "GCGAGCGTTGTCCGGATTTACTGGGTGTAAAGGGCGTGTAGGCGGAGATGCAAGTTGGGAGTGAAATCCATGGGCTCAACCCATGAACTGCTTCCAAAACTGTATCCCTTGAGTATCGGAGAGGCAAGCGGAATTCCTAGTGTAGCGGTGAAATGCGTAGATATTAGGAGGAACACCAGTGGCGAAGGCGGCTTGCTGGACGACAACTGACGCTGAGGCGCGAAAGCGTGGGGAGCAAAC"
## [4] "GCAAGCGTTGTCCGGATTTACTGGGTGTAAAGGGCGTGTAGGCGGAGATGCAAGTCGGGAGTGAAATCCATGGGCTCAACCCATGAACTGCTTTCGAAACTGTATCCCTTGAGTATCGGAGAGGCAAGCGGAATTCCTAGTGTAGCGGTGAAATGCGTAGATATTAGGAGGAACACCAGTGGCGAAGGCGGCTTGCTGGACGACAACTGACGCTGAGGCGCGAAAGCGTGGGGAGCAAAC"
## [5] "GCAAGCGTTGTCCGGAATTACTGGGTGTAAAGGGAGCGCAGGCGGAAGGACAAGTTGGAAGTGAAACCCACGGGCTCAACCCGTGAACTGCTTTCAAAACTGTTTTTCTTGAGTGGTGTAGAGGTAGGCGGAATTCCCGGTGTAGCGGTGGAATGCGTAGATATCGGGAGGAACACCAGTGGCGAAGGCGGCCTACTGGGCACTAACTGACGCTGAGGCTCGAAAGCATGGGTAGCAAAC"
## [6] "GCGAGCGTTATCCGGATTTATTGGGTTTAAAGGGAGCGCAGACGGGACTTTAAGTCAGCTGTGAAATTTTCCGGCTCAACCGGGAAACTGCAGTTGATACTGGCGTCCTTGAGTACGGTCGAGGCAGGCGGAATTCGTGGTGTAGCGGTGAAATGCTTAGATATCACGAAGAACCCCGATTGCGAAGGCAGCCTGCCAGACCGCAACTGACGTTCATGCTCGAAAGTGCGGGTATCAAAC"
## [7] "GCGAGCGTTATCCGGATTTATTGGGTTTAAAGGGAGCGTAGGCGGGCTGTTAAGTCAGCGGTCAAATGTCAGGGCCCAACCTTGGCATGCCGTTGATACTGGCGGCCTTGAGTTCACACAAGGAAGGTGGAATTCGTCGTGTAGCGGTGAAATGCTTAGATATGACGAAGAACTCCGATTGCGAAGGCAGCCTTCTGGGGTGTTACTGACGCTGAGGCTCGAAAGTGCGGGAATCAAACA"
## [8] "GCAAGCGTTATCCGGATTTACTGGGTGTAAAGGGCGTGTAGGCGGGAAAGCAAGTCAGATGTGAAAACTGTGGGCTCAACCCACAGCCTGCATTTGAAACTGTTTTTCTTGAGTACTGGAGAGGCAGATGGAATTCCTAGTGTAGCGGTGAAATGCGTAGATATTAGGAGGAACACCAGTGGCGAAGGCGATCTGCTGGACAGCAACTGACGCTGAGGCGCGAAAGCGTGGGGAGCAAAC"
## [9] "GCAAGCGTTAATCGGAATAACTGGGCGTAAAGGGCATGCAGGCGGTTCATCAAGTAGGATGTGAAATCCCCGGGCTCAACCTGGGAACAGCATACTAAACTGGTGGACTAGAGTATTGCAGGGGGAGACGGAATTCCAGGTGTAGCGGTGGAATGCGTAGATATCTGGAAGAACACCAAAGGCGAAGGCAGTCTCCTGGGCAAATACTGACGCTCATATGCGAAAGCGTGGGTAGCAAAC"
## 
## $xy_only
## [1] "GCAAGCGTTATCCGGATTTACTGGGTGTAAAGGGAGCGTAGACGGCCGTGCAAGTCTGATGTGAAAGGCTGGGGCTCAACCCCGGGACTGCATTGGAAACTGTATGGCTGGAGTGCCGGAGAGGTAAGCGGAATTCCTAGTGTAGCGGTGAAATGCGTAGATATTAGGAGGAACACCAGTGGCGAAGGCGGCTTACTGGACGGTAACTGACGTTGAGGCTCGAAAGCGTGGGGAGCAAAC"
## [2] "GCAAGCGTTATCCGGATTTACTGGGTGTAAAGGGAGCGCAGACGGCACTGCAAGTCTGAAGTGAAAGCCCGGGGCTCAACCCCGGGACTGCTTTGGAAACTGTAGAGCTAGAGTGCTGGAGAGGCAAGCGGAATTCCTAGTGTAGCGGTGAAATGCGTAGATATTAGGAGGAACACCAGTGGCGAAGGCGGCTTACTGGACGGTAACTGACGTTGAGGCTCGAAAGCGTGGGGAGCAAAC"
## [3] "GCAAGCGTTATCCGGATTTACTGGGTGTAAAGGGCGCGTAGGCGGGGATGCAAGTCAGATGTGAAATCTATGGGCTTAACCCATAAACTGCATTTGAAACTGTATCTCTTGAGTGCTGGAGAGGTAGACGGAATTCCTTGTGTAGCGGTGAAATGCGTAGATATAAGGAAGAACACCAGTGGCGAAGGCGGTCTACTGGACAGTAACTGACGCTGAGGCGCGAGAGCGTGGGGAGCAAAC"
## 
## $xz_only
## character(0)
## 
## $yz_only
## [1] "ACAAGCGTTGTCCGGAATTACTGGGTGTAAAGGGAGCGCAGGCGGGCGATCAAGTTGGAAGTGAAATCCATGGGCTCAACCCATGAACTGCTTTCAAAACTGGTCGTCTTGAGTAGTGCAGAGGTAGGCGGAATTCCCGGTGTAGCGGTGGAATGCGTAGATATCGGGAGGAACACCAGTGGCGAAGGCGGCCTACTGGGCACCAACTGACGCTGAGGCTCGAAAGTGTGGGTAGCAAAC"
## [2] "ACAAGCGTTGTCCGGAATTACTGGGTGTAAAGGGAGCGCAGGCGGGAAGACAAGTTGGAAGTGAAATCCATGGGCTCAACCCATGAACTGCTTTCAAAACTGTTTTTCTTGAGTAGTGCAGAGGTAGGCGGAATTCCCGGTGTAGCGGTGGAATGCGTAGATATCGGGAGGAACACCAGTGGCGAAGGCGGCCTACTGGGCACCAACTGACGCTGAGGCTCGAAAGTGTGGGTAGCAAAC"
## [3] "GCGAGCGTTGTCCGGATTTACTGGGTGTAAAGGGCGTGTAGGCGGAGATGCAAGTTGGGAGTGAAATCCATGGGCTCAACCCATGAACTGCTTCCAAAACTGTATCCCTTGAGTATCGGAGAGGCAAGCGGAATTCCTAGTGTAGCGGTGAAATGCGTAGATATTAGGAGGAACACCAGTGGCGAAGGCGGCTTGCTGGACGACAACTGACGCTGAGGCGCGAAAGCGTGGGGAGCAAAC"
## [4] "GCAAGCGTTGTCCGGATTTACTGGGTGTAAAGGGCGTGTAGGCGGAGATGCAAGTCGGGAGTGAAATCCATGGGCTCAACCCATGAACTGCTTTCGAAACTGTATCCCTTGAGTATCGGAGAGGCAAGCGGAATTCCTAGTGTAGCGGTGAAATGCGTAGATATTAGGAGGAACACCAGTGGCGAAGGCGGCTTGCTGGACGACAACTGACGCTGAGGCGCGAAAGCGTGGGGAGCAAAC"
## [5] "GCAAGCGTTGTCCGGAATTACTGGGTGTAAAGGGAGCGCAGGCGGAAGGACAAGTTGGAAGTGAAACCCACGGGCTCAACCCGTGAACTGCTTTCAAAACTGTTTTTCTTGAGTGGTGTAGAGGTAGGCGGAATTCCCGGTGTAGCGGTGGAATGCGTAGATATCGGGAGGAACACCAGTGGCGAAGGCGGCCTACTGGGCACTAACTGACGCTGAGGCTCGAAAGCATGGGTAGCAAAC"
## [6] "GCGAGCGTTATCCGGATTTATTGGGTTTAAAGGGAGCGCAGACGGGACTTTAAGTCAGCTGTGAAATTTTCCGGCTCAACCGGGAAACTGCAGTTGATACTGGCGTCCTTGAGTACGGTCGAGGCAGGCGGAATTCGTGGTGTAGCGGTGAAATGCTTAGATATCACGAAGAACCCCGATTGCGAAGGCAGCCTGCCAGACCGCAACTGACGTTCATGCTCGAAAGTGCGGGTATCAAAC"
## [7] "GCGAGCGTTATCCGGATTTATTGGGTTTAAAGGGAGCGTAGGCGGGCTGTTAAGTCAGCGGTCAAATGTCAGGGCCCAACCTTGGCATGCCGTTGATACTGGCGGCCTTGAGTTCACACAAGGAAGGTGGAATTCGTCGTGTAGCGGTGAAATGCTTAGATATGACGAAGAACTCCGATTGCGAAGGCAGCCTTCTGGGGTGTTACTGACGCTGAGGCTCGAAAGTGCGGGAATCAAACA"
## [8] "GCAAGCGTTATCCGGATTTACTGGGTGTAAAGGGCGTGTAGGCGGGAAAGCAAGTCAGATGTGAAAACTGTGGGCTCAACCCACAGCCTGCATTTGAAACTGTTTTTCTTGAGTACTGGAGAGGCAGATGGAATTCCTAGTGTAGCGGTGAAATGCGTAGATATTAGGAGGAACACCAGTGGCGAAGGCGATCTGCTGGACAGCAACTGACGCTGAGGCGCGAAAGCGTGGGGAGCAAAC"
## [9] "GCAAGCGTTAATCGGAATAACTGGGCGTAAAGGGCATGCAGGCGGTTCATCAAGTAGGATGTGAAATCCCCGGGCTCAACCTGGGAACAGCATACTAAACTGGTGGACTAGAGTATTGCAGGGGGAGACGGAATTCCAGGTGTAGCGGTGGAATGCGTAGATATCTGGAAGAACACCAAAGGCGAAGGCAGTCTCCTGGGCAAATACTGACGCTCATATGCGAAAGCGTGGGTAGCAAAC"
## 
## $xyz
## character(0)
```

```
#--------------------------------------------------------------------------------------------------------------


#FIGURE 2B, C, D: ANCOM NEG-UNTREATED 
#--------------------------------------------------------------------------------------------------------------
#Transform count data in the phyloseq object
ps_gg_fp_f_prevalence_filter_2019_05_26_proportion<-transform_sample_counts(ps_gg_fp_f_prevalence_filter_2019_05_26, function(x)(x/sum(x)))
```

```
## Found more than one class "phylo" in cache; using the first, from namespace 'phyloseq'
## Also defined by 'tidytree'
```

```
## Found more than one class "phylo" in cache; using the first, from namespace 'phyloseq'
```

```
## Also defined by 'tidytree'
```

```
## Found more than one class "phylo" in cache; using the first, from namespace 'phyloseq'
```

```
## Also defined by 'tidytree'
```

```
## Found more than one class "phylo" in cache; using the first, from namespace 'phyloseq'
```

```
## Also defined by 'tidytree'
```

```
###RUN ANCOM###

#US 
dataset<-ps_gg_fp_f_prevalence_filter_2019_05_26
metadata<-as.data.frame(sample_data(ps_gg_fp_f_prevalence_filter_2019_05_26))
metadata<-metadata[metadata$hiv_phenotype %in% c("1_hiv_negative", "4_unsuppressed"), , drop=F]
metadata<-as.data.frame(as.matrix(metadata[metadata$sexual_orientation != "MSM" | is.na(metadata$sexual_orientation), , drop=F]))
metadata_boston<-metadata[metadata$sample_cohort == "boston", , drop=F]
sample_data(dataset)<-metadata_boston
```

```
## Found more than one class "phylo" in cache; using the first, from namespace 'phyloseq'
## Also defined by 'tidytree'
```

```
## Found more than one class "phylo" in cache; using the first, from namespace 'phyloseq'
```

```
## Also defined by 'tidytree'
```

```
## Found more than one class "phylo" in cache; using the first, from namespace 'phyloseq'
```

```
## Also defined by 'tidytree'
```

```
## Found more than one class "phylo" in cache; using the first, from namespace 'phyloseq'
```

```
## Also defined by 'tidytree'
```

```
ps.taxa.sub <- phyloseq::prune_taxa(taxa_sums(dataset) > 0, dataset)
```

```
## Found more than one class "phylo" in cache; using the first, from namespace 'phyloseq'
## Also defined by 'tidytree'
```

```
out <- ANCOMBC::ancombc(data = ps.taxa.sub, formula = "hiv_phenotype", tax_level = NULL,
                        p_adj_method = "BH", prv_cut = 0.05, lib_cut = 1000, 
                        group = "hiv_phenotype", struc_zero = TRUE, neg_lb = FALSE, tol = 1e-5, 
                        max_iter = 100, conserve = FALSE, alpha = 0.05, global = FALSE, n_cl = 6)
```

```
## 'ancombc' is deprecated 
## Use 'ancombc2' instead
```

```
## Found more than one class "phylo" in cache; using the first, from namespace 'phyloseq'
```

```
## Also defined by 'tidytree'
```

```
## Found more than one class "phylo" in cache; using the first, from namespace 'phyloseq'
```

```
## Also defined by 'tidytree'
```

```
## `tax_level` is not speficified 
## No agglomeration will be performed
## Otherwise, please speficy `tax_level` by one of the following: 
## Kingdom, Phylum, Class, Order, Family, Genus, Species
```

```
## Found more than one class "phylo" in cache; using the first, from namespace 'phyloseq'
```

```
## Also defined by 'tidytree'
```

```
## Found more than one class "phylo" in cache; using the first, from namespace 'phyloseq'
```

```
## Also defined by 'tidytree'
```

```
## Found more than one class "phylo" in cache; using the first, from namespace 'phyloseq'
```

```
## Also defined by 'tidytree'
```

```
## Found more than one class "phylo" in cache; using the first, from namespace 'phyloseq'
```

```
## Also defined by 'tidytree'
```

```
## Warning: The group variable has < 3 categories 
## The multi-group comparisons (global/pairwise/dunnet/trend) will be deactivated
```

```
## Found more than one class "phylo" in cache; using the first, from namespace 'phyloseq'
## Also defined by 'tidytree'
```

```
## Found more than one class "phylo" in cache; using the first, from namespace 'phyloseq'
```

```
## Also defined by 'tidytree'
```

```
res <- out$res
res_rn <- purrr::imap(res, function(x, y) dplyr::rename(x, !!y := hiv_phenotype4_unsuppressed))
res_df <- purrr::reduce(res_rn, dplyr::left_join, by = "taxon")
res_df <- dplyr::select(res_df, !starts_with("(Int"))
res_df_taxa <- dplyr::left_join(res_df, tibble::rownames_to_column(as.data.frame(phyloseq::tax_table(ps.taxa.sub))), by = c("taxon" = "rowname"))
res_df_taxa[["index_num"]] <- 1:nrow(res_df_taxa)
res_df_taxa[["cohort"]] <- "boston"
res_df_taxa[["method"]] <- "ancom"
res_df_taxa <- tidyr::unite(res_df_taxa, col =  "Genus_Species", Genus, Species, index_num, remove = FALSE)
alpha = 0.05
taxa_sig <- dplyr::filter(res_df_taxa, q_val < 0.05)
taxa_sig$Genus_Species <- forcats::fct_reorder(taxa_sig$Genus_Species, taxa_sig$lfc, min)
taxa_sig$taxon_short <- stringr::str_sub(taxa_sig$taxon, 1, 4)
ps.taxa.rel.sig <- phyloseq::prune_taxa(taxa_sig[["taxon"]], ps_gg_fp_f_prevalence_filter_2019_05_26_proportion)
```

```
## Found more than one class "phylo" in cache; using the first, from namespace 'phyloseq'
## Also defined by 'tidytree'
```

```
# Only keep filtered samples 
ps.taxa.rel.sig <- phyloseq::prune_samples(rownames(phyloseq::otu_table(ps.taxa.sub)), ps.taxa.rel.sig)
sigtab_dataset_us <- taxa_sig
write.csv(sigtab_dataset_us, "ANCOM_US_NEG_UNTREAT_Filtered.csv")


#BOTSWANA
dataset<-ps_gg_fp_f_prevalence_filter_2019_05_26
metadata<-as.data.frame(sample_data(ps_gg_fp_f_prevalence_filter_2019_05_26))
metadata<-metadata[metadata$hiv_phenotype %in% c("1_hiv_negative", "4_unsuppressed"), , drop=F]
metadata<-as.data.frame(as.matrix(metadata[metadata$sexual_orientation != "MSM" | is.na(metadata$sexual_orientation), , drop=F]))
metadata_botswana<-metadata[metadata$sample_cohort == "botswana", , drop=F]
sample_data(dataset)<-metadata_botswana
```

```
## Found more than one class "phylo" in cache; using the first, from namespace 'phyloseq'
## Also defined by 'tidytree'
```

```
## Found more than one class "phylo" in cache; using the first, from namespace 'phyloseq'
```

```
## Also defined by 'tidytree'
```

```
## Found more than one class "phylo" in cache; using the first, from namespace 'phyloseq'
```

```
## Also defined by 'tidytree'
```

```
## Found more than one class "phylo" in cache; using the first, from namespace 'phyloseq'
```

```
## Also defined by 'tidytree'
```

```
ps.taxa.sub <- phyloseq::prune_taxa(taxa_sums(dataset) > 0, dataset)
```

```
## Found more than one class "phylo" in cache; using the first, from namespace 'phyloseq'
## Also defined by 'tidytree'
```

```
out <- ANCOMBC::ancombc(data = ps.taxa.sub, formula = "hiv_phenotype", tax_level = NULL,
                        p_adj_method = "BH", prv_cut = 0.05, lib_cut = 1000, 
                        group = "hiv_phenotype", struc_zero = TRUE, neg_lb = FALSE, tol = 1e-5, 
                        max_iter = 100, conserve = FALSE, alpha = 0.05, global = FALSE, n_cl = 6)
```

```
## 'ancombc' is deprecated 
## Use 'ancombc2' instead
```

```
## Found more than one class "phylo" in cache; using the first, from namespace 'phyloseq'
```

```
## Also defined by 'tidytree'
```

```
## Found more than one class "phylo" in cache; using the first, from namespace 'phyloseq'
```

```
## Also defined by 'tidytree'
```

```
## `tax_level` is not speficified 
## No agglomeration will be performed
## Otherwise, please speficy `tax_level` by one of the following: 
## Kingdom, Phylum, Class, Order, Family, Genus, Species
```

```
## Found more than one class "phylo" in cache; using the first, from namespace 'phyloseq'
```

```
## Also defined by 'tidytree'
```

```
## Found more than one class "phylo" in cache; using the first, from namespace 'phyloseq'
```

```
## Also defined by 'tidytree'
```

```
## Found more than one class "phylo" in cache; using the first, from namespace 'phyloseq'
```

```
## Also defined by 'tidytree'
```

```
## Found more than one class "phylo" in cache; using the first, from namespace 'phyloseq'
```

```
## Also defined by 'tidytree'
```

```
## Warning: The group variable has < 3 categories 
## The multi-group comparisons (global/pairwise/dunnet/trend) will be deactivated
```

```
## Found more than one class "phylo" in cache; using the first, from namespace 'phyloseq'
## Also defined by 'tidytree'
```

```
## Found more than one class "phylo" in cache; using the first, from namespace 'phyloseq'
```

```
## Also defined by 'tidytree'
```

```
res <- out$res
res_rn <- purrr::imap(res, function(x, y) dplyr::rename(x, !!y := hiv_phenotype4_unsuppressed))
res_df <- purrr::reduce(res_rn, dplyr::left_join, by = "taxon")
res_df <- dplyr::select(res_df, !starts_with("(Int"))
res_df_taxa <- dplyr::left_join(res_df, tibble::rownames_to_column(as.data.frame(phyloseq::tax_table(ps.taxa.sub))), by = c("taxon" = "rowname"))
res_df_taxa[["index_num"]] <- 1:nrow(res_df_taxa)
res_df_taxa[["cohort"]] <- "botswana"
res_df_taxa[["method"]] <- "ancom"
res_df_taxa <- tidyr::unite(res_df_taxa, col =  "Genus_Species", Genus, Species, index_num, remove = FALSE)
alpha = 0.05
taxa_sig <- dplyr::filter(res_df_taxa, q_val < 0.05)
taxa_sig$Genus_Species <- forcats::fct_reorder(taxa_sig$Genus_Species, taxa_sig$lfc, min)
taxa_sig$taxon_short <- stringr::str_sub(taxa_sig$taxon, 1, 4)
ps.taxa.rel.sig <- phyloseq::prune_taxa(taxa_sig[["taxon"]], ps_gg_fp_f_prevalence_filter_2019_05_26_proportion)
```

```
## Found more than one class "phylo" in cache; using the first, from namespace 'phyloseq'
## Also defined by 'tidytree'
```

```
# Only keep filtered samples 
ps.taxa.rel.sig <- phyloseq::prune_samples(rownames(phyloseq::otu_table(ps.taxa.sub)), ps.taxa.rel.sig)
sigtab_dataset_botswana <- taxa_sig
write.csv(sigtab_dataset_botswana, "ANCOM_BOTS_NEG_UNTREAT_Filtered.csv")


# Save plots of individual ANCOM fold-change
ggsave("Figure2BCD_NEG_UNTREAT_ANCOM_US_v4.pdf", ggplot(data = sigtab_dataset_us, aes(x = Genus_Species, y = lfc)) + theme_bw() + coord_flip() + geom_bar(stat="identity", aes(fill = Genus)) + ggtitle("NEG-UNTREAT US") + theme(legend.position = "bottom"), width = 10, height = 10, units = "in", dpi = 300)

ggsave("Figure2BCD_NEG_UNTREAT_ANCOM_BOTS_v4.pdf", ggplot(data = sigtab_dataset_botswana, aes(x = Genus_Species, y = lfc)) + theme_bw() + coord_flip() + geom_bar(stat="identity", aes(fill = Genus)) + ggtitle("NEG-UNTREAT BOTSWANA") + theme(legend.position = "bottom"), width = 10, height = 10, units = "in", dpi = 300)


#Merge results from both sample cohorts:
sigtab_dataset_us_filter_prev_005 <- dplyr::filter(sigtab_dataset_us, abs(lfc) >= 0.05)
sigtab_dataset<-rbind(sigtab_dataset_us_filter_prev_005, sigtab_dataset_botswana)
sigtab_dataset[["index_num_all"]] <- 1:nrow(sigtab_dataset)
sigtab_dataset <- tidyr::unite(sigtab_dataset, col =  "Genus_Species_all", Genus, Species, index_num_all, sep = "_", remove = FALSE)
sigtab_dataset <- tidyr::unite(sigtab_dataset, col =  "Genus_Species_all_for_color", Genus, Species, sep = " ", remove = FALSE)

# Remove extra brackets around taxonomic name for labels
sigtab_dataset$Genus_label <- stringr::str_replace(sigtab_dataset$Genus, "^\\[([^\\]]+)\\]", "\\1")
sigtab_dataset$Species_label <- stringr::str_replace(sigtab_dataset$Species, "^\\[([^\\]]+)\\]", "\\1")
sigtab_dataset %>% dplyr::mutate(Genus_species_label =
                                   dplyr::case_when(is.na(stringr::str_extract(Genus_label, "\\[")) & is.na(stringr::str_extract(Species_label, "\\[")) ~ paste(Genus_label,Species_label),
                                                    TRUE ~ paste(Genus_label,"sp"))) -> sigtab_dataset
sigtab_dataset$Genus_species_label <- stringr::str_replace(sigtab_dataset$Genus_species_label, "\\[([^\\]]+)\\]", "")
sigtab_dataset$Genus_species_label <- stringr::str_replace(sigtab_dataset$Genus_species_label, "\\ +", " ")
sigtab_dataset <- dplyr::arrange(sigtab_dataset, cohort, lfc)
sigtab_dataset$Genus_Species_all<-factor(sigtab_dataset$Genus_Species_all, levels = c(sigtab_dataset$Genus_Species_all))

# Make lookup table for Genus_species_label
Genus_species_label_lookup <- dplyr::distinct(sigtab_dataset, Genus_Species_all, .keep_all = TRUE)$Genus_species_label
names(Genus_species_label_lookup) <- dplyr::distinct(sigtab_dataset, Genus_Species_all, .keep_all = TRUE)$Genus_Species_all

#Load color dictionary and construct figure:
dictionary_unique <- read.csv("COLOR_DICTIONARY3.csv", sep=";")
sigtab_dataset$Genus_species_label <- factor(sigtab_dataset$Genus_species_label, levels = unique(sigtab_dataset$Genus_species_label))
dictionary_plot <- dictionary_unique[dictionary_unique$Genus_species_label%in%sigtab_dataset$Genus_species_label,,drop=F]
rownames(dictionary_plot)<-dictionary_plot$Genus_species_label
dictionary_plot<-dictionary_plot[as.character(unique(sigtab_dataset$Genus_species_label)),,drop=F]
sigtab_dataset$cohort <- fct_relevel(as.factor(sigtab_dataset$cohort), "boston", "botswana")
header_namer <- as_labeller(c(`boston` = "U.S.\nHIV-uninfected     HIV+ Untreated", `botswana` = "Botswana\nHIV-uninfected     HIV+ Untreated"))


ggsave("Figure2BCD_NEG_UNTREAT_ANCOM_ALL_v7.pdf", 
       ggplot(data = sigtab_dataset, aes(x = Genus_Species_all, y = lfc)) +
         geom_bar(stat = "identity", aes(fill = Genus_species_label)) +
         scale_fill_manual(values = as.character(dictionary_plot$Color)) +
         coord_flip() + scale_x_discrete(label = as_labeller(Genus_species_label_lookup)) + theme_bw() +
         theme(legend.position = "bottom", axis.text.x = element_text(size = 16), axis.text.y = element_text(size = 6, face = "plain"), axis.ticks.y = element_blank(), 
               panel.border = element_rect(linetype = "solid", fill = NA, linewidth = 1), axis.title.y = element_blank(),
               strip.background = element_rect(colour = "white", fill = "white"), strip.text = element_text(colour = "black", face = "bold", size = rel(1.2))) + 
         geom_hline(yintercept = 0) + ylab("log2FoldChange") +
         facet_wrap(vars(cohort), scales = "fixed", ncol = 3, labeller = labeller(cohort = header_namer)), width = 15, height = 20, units = "in", dpi = 300)

#--------------------------------------------------------------------------------------------------------------
```
