# Supplementary material for: HIV-associated gut microbial alterations are dependent on host and geographic context
Source: Nat Commun. 2024 Feb 5;15:1055. doi: 10.1038/s41467-023-44566-4 (PMC10844288; doi:10.1038/s41467-023-44566-4)
Supplement: Supplementary file 10 — Figure3ANCOM_Rocafort-Gootenberg_2023_03_02 [file 41467_2023_44566_MOESM10_ESM.html]

Rocafort-Gootenberg\_Figure3ANCOM


# Rocafort-Gootenberg\_Figure3ANCOM

#Load needed R packages

```
library("phyloseq")
library("tidyverse")
```

```
## ── Attaching packages ─────────────────────────────────────── tidyverse 1.3.2 ──
## ✔ ggplot2 3.4.1     ✔ purrr   1.0.1
## ✔ tibble  3.1.8     ✔ dplyr   1.1.0
## ✔ tidyr   1.3.0     ✔ stringr 1.5.0
## ✔ readr   2.1.4     ✔ forcats 1.0.0
## ── Conflicts ────────────────────────────────────────── tidyverse_conflicts() ──
## ✖ dplyr::filter() masks stats::filter()
## ✖ dplyr::lag()    masks stats::lag()
```

```
library("ggplot2")
library("gridExtra")
```

```
## 
## Attaching package: 'gridExtra'
## 
## The following object is masked from 'package:dplyr':
## 
##     combine
```

```
library("dplyr")
library("vegan")
```

```
## Loading required package: permute
## Loading required package: lattice
## This is vegan 2.6-4
```

```
library("knitr") 
library("reshape")
```

```
## 
## Attaching package: 'reshape'
## 
## The following object is masked from 'package:dplyr':
## 
##     rename
## 
## The following objects are masked from 'package:tidyr':
## 
##     expand, smiths
```

```
library("DESeq2")
```

```
## Loading required package: S4Vectors
## Loading required package: stats4
## Loading required package: BiocGenerics
## 
## Attaching package: 'BiocGenerics'
## 
## The following object is masked from 'package:gridExtra':
## 
##     combine
## 
## The following objects are masked from 'package:dplyr':
## 
##     combine, intersect, setdiff, union
## 
## The following objects are masked from 'package:stats':
## 
##     IQR, mad, sd, var, xtabs
## 
## The following objects are masked from 'package:base':
## 
##     anyDuplicated, aperm, append, as.data.frame, basename, cbind,
##     colnames, dirname, do.call, duplicated, eval, evalq, Filter, Find,
##     get, grep, grepl, intersect, is.unsorted, lapply, Map, mapply,
##     match, mget, order, paste, pmax, pmax.int, pmin, pmin.int,
##     Position, rank, rbind, Reduce, rownames, sapply, setdiff, sort,
##     table, tapply, union, unique, unsplit, which.max, which.min
## 
## 
## Attaching package: 'S4Vectors'
## 
## The following objects are masked from 'package:reshape':
## 
##     expand, rename
## 
## The following objects are masked from 'package:dplyr':
## 
##     first, rename
## 
## The following object is masked from 'package:tidyr':
## 
##     expand
## 
## The following objects are masked from 'package:base':
## 
##     expand.grid, I, unname
## 
## Loading required package: IRanges
## 
## Attaching package: 'IRanges'
## 
## The following objects are masked from 'package:dplyr':
## 
##     collapse, desc, slice
## 
## The following object is masked from 'package:purrr':
## 
##     reduce
## 
## The following object is masked from 'package:phyloseq':
## 
##     distance
## 
## Loading required package: GenomicRanges
## Loading required package: GenomeInfoDb
## Loading required package: SummarizedExperiment
## Loading required package: MatrixGenerics
## Loading required package: matrixStats
## 
## Attaching package: 'matrixStats'
## 
## The following object is masked from 'package:dplyr':
## 
##     count
## 
## 
## Attaching package: 'MatrixGenerics'
## 
## The following objects are masked from 'package:matrixStats':
## 
##     colAlls, colAnyNAs, colAnys, colAvgsPerRowSet, colCollapse,
##     colCounts, colCummaxs, colCummins, colCumprods, colCumsums,
##     colDiffs, colIQRDiffs, colIQRs, colLogSumExps, colMadDiffs,
##     colMads, colMaxs, colMeans2, colMedians, colMins, colOrderStats,
##     colProds, colQuantiles, colRanges, colRanks, colSdDiffs, colSds,
##     colSums2, colTabulates, colVarDiffs, colVars, colWeightedMads,
##     colWeightedMeans, colWeightedMedians, colWeightedSds,
##     colWeightedVars, rowAlls, rowAnyNAs, rowAnys, rowAvgsPerColSet,
##     rowCollapse, rowCounts, rowCummaxs, rowCummins, rowCumprods,
##     rowCumsums, rowDiffs, rowIQRDiffs, rowIQRs, rowLogSumExps,
##     rowMadDiffs, rowMads, rowMaxs, rowMeans2, rowMedians, rowMins,
##     rowOrderStats, rowProds, rowQuantiles, rowRanges, rowRanks,
##     rowSdDiffs, rowSds, rowSums2, rowTabulates, rowVarDiffs, rowVars,
##     rowWeightedMads, rowWeightedMeans, rowWeightedMedians,
##     rowWeightedSds, rowWeightedVars
## 
## Loading required package: Biobase
## Welcome to Bioconductor
## 
##     Vignettes contain introductory material; view with
##     'browseVignettes()'. To cite Bioconductor, see
##     'citation("Biobase")', and for packages 'citation("pkgname")'.
## 
## 
## Attaching package: 'Biobase'
## 
## The following object is masked from 'package:MatrixGenerics':
## 
##     rowMedians
## 
## The following objects are masked from 'package:matrixStats':
## 
##     anyMissing, rowMedians
## 
## The following object is masked from 'package:phyloseq':
## 
##     sampleNames
```

```
library("ANCOMBC")
library("BiodiversityR")
```

```
## Loading required package: tcltk
## BiodiversityR 2.15-1: Use command BiodiversityRGUI() to launch the Graphical User Interface; 
## to see changes use BiodiversityRGUI(changeLog=TRUE, backward.compatibility.messages=TRUE)
```

```
library("usedist") 
library("gplots")
```

```
## Registered S3 method overwritten by 'gplots':
##   method         from     
##   reorder.factor DescTools
## 
## Attaching package: 'gplots'
## 
## The following object is masked from 'package:IRanges':
## 
##     space
## 
## The following object is masked from 'package:S4Vectors':
## 
##     space
## 
## The following object is masked from 'package:stats':
## 
##     lowess
```

#Load original phyloseq object output from DADA2 pipeline and pull in
new metadata

```
ps_gg_fp_f_prevalence_filter_2019_05_26<-readRDS("ps_gg_fp_f_prevalence_filter_2019_05_26")
readr::read_csv(
  "Metadata_formatted_nat_comm_add_2021_10_24.csv",
  col_names = TRUE,
  col_types = NULL,
  col_select = NULL,
  id = NULL,
  locale = default_locale(),
  na = c("", "NA", "empty", "EMPTY"),
  quote = "\"",
  comment = "",
  trim_ws = TRUE,
  skip = 0,
  name_repair = "unique",
  num_threads = readr_threads(),
  progress = show_progress(),
  show_col_types = should_show_types(),
  skip_empty_rows = TRUE,
  lazy = TRUE
) -> new_metadata
```

```
## Rows: 597 Columns: 88
## ── Column specification ────────────────────────────────────────────────────────
## Delimiter: ","
## chr (26): X, SampleID, subject_id, Race, Ethnicity, unique_id, sequencing_da...
## dbl (62): primer_used, read_count, age, height_cm, height_in, weight_kg, wei...
## 
## ℹ Use `spec()` to retrieve the full column specification for this data.
## ℹ Specify the column types or set `show_col_types = FALSE` to quiet this message.
```

```
### add {SampleID} as rownames
new_metadata_as_sample_data <- phyloseq::sample_data(new_metadata)
phyloseq::sample_names(new_metadata_as_sample_data) <- dplyr::pull(new_metadata, 1)
phyloseq::sample_data(ps_gg_fp_f_prevalence_filter_2019_05_26) <- new_metadata_as_sample_data
```

```
## Found more than one class "phylo" in cache; using the first, from namespace 'phyloseq'
## Also defined by 'tidytree'
## Found more than one class "phylo" in cache; using the first, from namespace 'phyloseq'
## Also defined by 'tidytree'
## Found more than one class "phylo" in cache; using the first, from namespace 'phyloseq'
## Also defined by 'tidytree'
## Found more than one class "phylo" in cache; using the first, from namespace 'phyloseq'
## Also defined by 'tidytree'
## Found more than one class "phylo" in cache; using the first, from namespace 'phyloseq'
## Also defined by 'tidytree'
## Found more than one class "phylo" in cache; using the first, from namespace 'phyloseq'
## Also defined by 'tidytree'
## Found more than one class "phylo" in cache; using the first, from namespace 'phyloseq'
## Also defined by 'tidytree'
```

```
#Fix randomness
set.seed(1)
```

#Figure 3A

```
#FIGURE 3A
#--------------------------------------------------------------------------------------------------------------
#COMPARISON non-MSM
#Comparison NEG-ART
#Transform count data in the phyloseq object
ps_gg_fp_f_prevalence_filter_2019_05_26_proportion<-transform_sample_counts(ps_gg_fp_f_prevalence_filter_2019_05_26, function(x)(x/sum(x)))
```

```
## Found more than one class "phylo" in cache; using the first, from namespace 'phyloseq'
```

```
## Also defined by 'tidytree'
```

```
## Found more than one class "phylo" in cache; using the first, from namespace 'phyloseq'
```

```
## Also defined by 'tidytree'
```

```
## Found more than one class "phylo" in cache; using the first, from namespace 'phyloseq'
```

```
## Also defined by 'tidytree'
```

```
## Found more than one class "phylo" in cache; using the first, from namespace 'phyloseq'
```

```
## Also defined by 'tidytree'
```

```
#Select samples of interest and update phyloseq object 
metadata<-sample_data(ps_gg_fp_f_prevalence_filter_2019_05_26)
metadata<-metadata[metadata$sample_cohort=="boston",,drop=F]
metadata<-metadata[metadata$hiv_phenotype%in%c("1_hiv_negative","2_suppressed"),,drop=F]
metadata<-as.data.frame(as.matrix(metadata[metadata$sexual_orientation!="MSM",,drop=F]))
sample_data(ps_gg_fp_f_prevalence_filter_2019_05_26_proportion)<-metadata
```

```
## Found more than one class "phylo" in cache; using the first, from namespace 'phyloseq'
## Also defined by 'tidytree'
```

```
## Found more than one class "phylo" in cache; using the first, from namespace 'phyloseq'
```

```
## Also defined by 'tidytree'
```

```
## Found more than one class "phylo" in cache; using the first, from namespace 'phyloseq'
```

```
## Also defined by 'tidytree'
```

```
## Found more than one class "phylo" in cache; using the first, from namespace 'phyloseq'
```

```
## Also defined by 'tidytree'
```

```
#Run PCoA on the phyloseq object
ordination<-ordinate(ps_gg_fp_f_prevalence_filter_2019_05_26_proportion, "PCoA", "unifrac")
```

```
## Warning in matrix(tree$edge[order(tree$edge[, 1]), ][, 2], byrow = TRUE, : data
## length [8987] is not a sub-multiple or multiple of the number of rows [4494]
```

```
ordination$values[1:2,]
```

```
##   Eigenvalues Relative_eig Broken_stick  Cumul_eig Cumul_br_stick
## 1    2.442768   0.06544973   0.05064846 0.06544973     0.05064846
## 2    1.813608   0.04859246   0.04093972 0.11404219     0.09158818
```

```
metadata_ordered<-metadata[row.names(ordination$vectors),,drop=FALSE]

all.equal(row.names(metadata_ordered), row.names(ordination$vectors))
```

```
## [1] TRUE
```

```
metadata_ordered$Unifrac1<-ordination$vectors[,1]
metadata_ordered$Unifrac2<-ordination$vectors[,2]

#Let's plot the data
plot_noMSM_NEG_ART<-ggplot(data=metadata_ordered, aes(x=Unifrac1, y=Unifrac2))+geom_point(color="royalblue4", aes(alpha=hiv_phenotype), size=2, shape=16)+
  theme_bw()+stat_ellipse(color="royalblue4", aes(alpha=hiv_phenotype), size=1)+scale_alpha_manual(values=c(1,0.6))+ylim(c(-0.55, 0.55))+xlim(c(-0.55,0.55))+
  ggtitle("neg-art noMSM")+geom_point(data=metadata_ordered %>% group_by(hiv_phenotype) %>% summarise_at(vars(matches("UniFrac")), mean),size=5, color="royalblue4", aes(alpha=hiv_phenotype))
```

```
## Warning: Using `size` aesthetic for lines was deprecated in ggplot2 3.4.0.
## ℹ Please use `linewidth` instead.
```

```
#Adonis (n = 104)
ASV_table<-as.data.frame(phyloseq::otu_table(ps_gg_fp_f_prevalence_filter_2019_05_26_proportion))
all.equal(row.names(ASV_table), row.names(metadata_ordered))
```

```
## [1] TRUE
```

```
unifrac.distance<-unname(phyloseq::UniFrac(ps_gg_fp_f_prevalence_filter_2019_05_26_proportion, weighted = FALSE)) ### unname fixes error introduced by Desctools see https://github.com/joey711/phyloseq/issues/1457
```

```
## Warning in matrix(tree$edge[order(tree$edge[, 1]), ][, 2], byrow = TRUE, : data
## length [8987] is not a sub-multiple or multiple of the number of rows [4494]
```

```
attributes(unifrac.distance)$Labels <- phyloseq::sample_names(ps_gg_fp_f_prevalence_filter_2019_05_26_proportion)
print(vegan::adonis2(unifrac.distance~metadata_ordered$hiv_phenotype, data=ASV_table, permutations=1000)) -> adon_hiv_phenotype_nonmsm_neg_art
```

```
## Permutation test for adonis under reduced model
## Terms added sequentially (first to last)
## Permutation: free
## Number of permutations: 1000
## 
## vegan::adonis2(formula = unifrac.distance ~ metadata_ordered$hiv_phenotype, data = ASV_table, permutations = 1000)
##                                 Df SumOfSqs      R2     F Pr(>F)
## metadata_ordered$hiv_phenotype   1    0.385 0.01032 1.064 0.2637
## Residual                       102   36.938 0.98968             
## Total                          103   37.323 1.00000
```

```
###*** r2 = 0.01032 p = 0.2637

# vegan::adonis2(formula = unifrac.distance ~ metadata_ordered$hiv_phenotype, data = ASV_table, permutations = 1000)
#                                 Df SumOfSqs      R2     F Pr(>F)
# metadata_ordered$hiv_phenotype   1    0.385 0.01032 1.064 0.2637
# Residual                       102   36.938 0.98968             
# Total                          103   37.323 1.00000      

#Comparison NEG-UNSUPPRESSED
#Transform count data in the phyloseq object
ps_gg_fp_f_prevalence_filter_2019_05_26_proportion<-transform_sample_counts(ps_gg_fp_f_prevalence_filter_2019_05_26, function(x)(x/sum(x)))
```

```
## Found more than one class "phylo" in cache; using the first, from namespace 'phyloseq'
## Also defined by 'tidytree'
```

```
## Found more than one class "phylo" in cache; using the first, from namespace 'phyloseq'
```

```
## Also defined by 'tidytree'
```

```
## Found more than one class "phylo" in cache; using the first, from namespace 'phyloseq'
```

```
## Also defined by 'tidytree'
```

```
## Found more than one class "phylo" in cache; using the first, from namespace 'phyloseq'
```

```
## Also defined by 'tidytree'
```

```
#Select samples of interest and update phyloseq object 
metadata<-sample_data(ps_gg_fp_f_prevalence_filter_2019_05_26)
metadata<-metadata[metadata$sample_cohort=="boston",,drop=F]
metadata<-metadata[metadata$hiv_phenotype%in%c("1_hiv_negative","4_unsuppressed"),,drop=F]
metadata<-as.data.frame(as.matrix(metadata[metadata$sexual_orientation!="MSM",,drop=F]))
sample_data(ps_gg_fp_f_prevalence_filter_2019_05_26_proportion)<-metadata
```

```
## Found more than one class "phylo" in cache; using the first, from namespace 'phyloseq'
## Also defined by 'tidytree'
```

```
## Found more than one class "phylo" in cache; using the first, from namespace 'phyloseq'
```

```
## Also defined by 'tidytree'
```

```
## Found more than one class "phylo" in cache; using the first, from namespace 'phyloseq'
```

```
## Also defined by 'tidytree'
```

```
## Found more than one class "phylo" in cache; using the first, from namespace 'phyloseq'
```

```
## Also defined by 'tidytree'
```

```
#Run PCoA on the phyloseq object
ordination<-ordinate(ps_gg_fp_f_prevalence_filter_2019_05_26_proportion, "PCoA", "unifrac")
```

```
## Warning in matrix(tree$edge[order(tree$edge[, 1]), ][, 2], byrow = TRUE, : data
## length [8987] is not a sub-multiple or multiple of the number of rows [4494]
```

```
ordination$values[1:2,]
```

```
##   Eigenvalues Relative_eig Broken_stick  Cumul_eig Cumul_br_stick
## 1    2.399651   0.06943654   0.05406681 0.06943654     0.05406681
## 2    1.759546   0.05091440   0.04354049 0.12035094     0.09760729
```

```
metadata_ordered<-metadata[row.names(ordination$vectors),,drop=FALSE]

all.equal(row.names(metadata_ordered), row.names(ordination$vectors))
```

```
## [1] TRUE
```

```
metadata_ordered$Unifrac1<-ordination$vectors[,1]
metadata_ordered$Unifrac2<-ordination$vectors[,2]

#Let's plot the data
plot_noMSM_NEG_UNSUP<-ggplot(data=metadata_ordered, aes(x=Unifrac1, y=Unifrac2))+geom_point(color="royalblue4", aes(alpha=hiv_phenotype), size=2, shape=16)+
  theme_bw()+stat_ellipse(color="royalblue4", aes(alpha=hiv_phenotype), size=1)+scale_alpha_manual(values=c(1,0.3))+ylim(c(-0.55, 0.55))+xlim(c(-0.55,0.55))+
  ggtitle("neg-unsuppressed noMSM")+geom_point(data=metadata_ordered %>% group_by(hiv_phenotype) %>% summarise_at(vars(matches("UniFrac")), mean),size=5, color="royalblue4", aes(alpha=hiv_phenotype))

#Adonis
ASV_table<-as.data.frame(phyloseq::otu_table(ps_gg_fp_f_prevalence_filter_2019_05_26_proportion))
all.equal(row.names(ASV_table), row.names(metadata_ordered))
```

```
## [1] TRUE
```

```
unifrac.distance<-unname(phyloseq::UniFrac(ps_gg_fp_f_prevalence_filter_2019_05_26_proportion, weighted = FALSE)) ### unname fixes error introduced by Desctools see https://github.com/joey711/phyloseq/issues/1457
```

```
## Warning in matrix(tree$edge[order(tree$edge[, 1]), ][, 2], byrow = TRUE, : data
## length [8987] is not a sub-multiple or multiple of the number of rows [4494]
```

```
attributes(unifrac.distance)$Labels <- phyloseq::sample_names(ps_gg_fp_f_prevalence_filter_2019_05_26_proportion)
print(vegan::adonis2(unifrac.distance~metadata_ordered$hiv_phenotype, data=ASV_table, permutations=1000)) -> adon_hiv_phenotype_nonmsm_neg_unsup
```

```
## Permutation test for adonis under reduced model
## Terms added sequentially (first to last)
## Permutation: free
## Number of permutations: 1000
## 
## vegan::adonis2(formula = unifrac.distance ~ metadata_ordered$hiv_phenotype, data = ASV_table, permutations = 1000)
##                                Df SumOfSqs      R2      F Pr(>F)
## metadata_ordered$hiv_phenotype  1    0.393 0.01138 1.0819 0.2468
## Residual                       94   34.166 0.98862              
## Total                          95   34.559 1.00000
```

```
###*** r2 = 0.01138 p = 0.2468

# vegan::adonis2(formula = unifrac.distance ~ metadata_ordered$hiv_phenotype, data = ASV_table, permutations = 1000)
#                                Df SumOfSqs      R2      F Pr(>F)
# metadata_ordered$hiv_phenotype  1    0.393 0.01138 1.0819 0.2468
# Residual                       94   34.166 0.98862              
# Total                          95   34.559 1.00000              

#COMPARISON MSM
#Comparison NEG-ART
#Transform count data in the phyloseq object
ps_gg_fp_f_prevalence_filter_2019_05_26_proportion<-transform_sample_counts(ps_gg_fp_f_prevalence_filter_2019_05_26, function(x)(x/sum(x)))
```

```
## Found more than one class "phylo" in cache; using the first, from namespace 'phyloseq'
## Also defined by 'tidytree'
```

```
## Found more than one class "phylo" in cache; using the first, from namespace 'phyloseq'
```

```
## Also defined by 'tidytree'
```

```
## Found more than one class "phylo" in cache; using the first, from namespace 'phyloseq'
```

```
## Also defined by 'tidytree'
```

```
## Found more than one class "phylo" in cache; using the first, from namespace 'phyloseq'
```

```
## Also defined by 'tidytree'
```

```
#Select samples of interest and update phyloseq object 
metadata<-phyloseq::sample_data(ps_gg_fp_f_prevalence_filter_2019_05_26)
metadata<-metadata[metadata$sample_cohort=="boston",,drop=F]
metadata<-metadata[metadata$hiv_phenotype%in%c("1_hiv_negative","2_suppressed"),,drop=F]
metadata<-as.data.frame(as.matrix(metadata[metadata$sexual_orientation=="MSM",,drop=F]))
phyloseq::sample_data(ps_gg_fp_f_prevalence_filter_2019_05_26_proportion)<-metadata
```

```
## Found more than one class "phylo" in cache; using the first, from namespace 'phyloseq'
## Also defined by 'tidytree'
```

```
## Found more than one class "phylo" in cache; using the first, from namespace 'phyloseq'
```

```
## Also defined by 'tidytree'
```

```
## Found more than one class "phylo" in cache; using the first, from namespace 'phyloseq'
```

```
## Also defined by 'tidytree'
```

```
## Found more than one class "phylo" in cache; using the first, from namespace 'phyloseq'
```

```
## Also defined by 'tidytree'
```

```
#Run PCoA on the phyloseq object
ordination<-phyloseq::ordinate(ps_gg_fp_f_prevalence_filter_2019_05_26_proportion, "PCoA", "unifrac")
```

```
## Warning in matrix(tree$edge[order(tree$edge[, 1]), ][, 2], byrow = TRUE, : data
## length [8987] is not a sub-multiple or multiple of the number of rows [4494]
```

```
ordination$values[1:2,]
```

```
##   Eigenvalues Relative_eig Broken_stick Cumul_eig Cumul_br_stick
## 1    3.021341   0.11143795   0.06677409 0.1114380     0.06677409
## 2    1.840634   0.06788921   0.05307546 0.1793272     0.11984956
```

```
metadata_ordered<-metadata[row.names(ordination$vectors),,drop=FALSE]

all.equal(row.names(metadata_ordered), row.names(ordination$vectors))
```

```
## [1] TRUE
```

```
metadata_ordered$Unifrac1<-ordination$vectors[,1]
metadata_ordered$Unifrac2<-ordination$vectors[,2]

#Let's plot the data
plot_MSM_NEG_ART<-ggplot(data=metadata_ordered, aes(x=Unifrac1, y=Unifrac2))+geom_point(color="royalblue4", aes(alpha=hiv_phenotype), size=2, shape=16)+
  theme_bw()+stat_ellipse(color="royalblue4", aes(alpha=hiv_phenotype), size=1)+scale_alpha_manual(values=c(1,0.6))+ylim(c(-0.55, 0.55))+xlim(c(-0.55,0.55))+
  ggtitle("neg-art MSM")+geom_point(data=metadata_ordered %>% group_by(hiv_phenotype) %>% summarise_at(vars(matches("UniFrac")), mean),size=5, color="royalblue4", aes(alpha=hiv_phenotype))

#Adonis (n=74)
ASV_table<-as.data.frame(phyloseq::otu_table(ps_gg_fp_f_prevalence_filter_2019_05_26_proportion))
all.equal(row.names(ASV_table), row.names(metadata_ordered))
```

```
## [1] TRUE
```

```
unifrac.distance<-unname(phyloseq::UniFrac(ps_gg_fp_f_prevalence_filter_2019_05_26_proportion, weighted = FALSE)) ### unname fixes error introduced by Desctools see https://github.com/joey711/phyloseq/issues/1457
```

```
## Warning in matrix(tree$edge[order(tree$edge[, 1]), ][, 2], byrow = TRUE, : data
## length [8987] is not a sub-multiple or multiple of the number of rows [4494]
```

```
attributes(unifrac.distance)$Labels <- phyloseq::sample_names(ps_gg_fp_f_prevalence_filter_2019_05_26_proportion)
print(vegan::adonis2(unifrac.distance~metadata_ordered$hiv_phenotype, data=ASV_table, permutations=1000)) -> adon_hiv_phenotype_msm_neg_art
```

```
## Permutation test for adonis under reduced model
## Terms added sequentially (first to last)
## Permutation: free
## Number of permutations: 1000
## 
## vegan::adonis2(formula = unifrac.distance ~ metadata_ordered$hiv_phenotype, data = ASV_table, permutations = 1000)
##                                Df SumOfSqs      R2      F   Pr(>F)    
## metadata_ordered$hiv_phenotype  1   1.6479 0.06078 4.6594 0.000999 ***
## Residual                       72  25.4644 0.93922                    
## Total                          73  27.1123 1.00000                    
## ---
## Signif. codes:  0 '***' 0.001 '**' 0.01 '*' 0.05 '.' 0.1 ' ' 1
```

```
###*** r2 = 0.06078 p = 0.000999

# vegan::adonis2(formula = unifrac.distance ~ metadata_ordered$hiv_phenotype, data = ASV_table, permutations = 1000)
#                                Df SumOfSqs      R2      F   Pr(>F)    
# metadata_ordered$hiv_phenotype  1   1.6479 0.06078 4.6594 0.000999 ***
# Residual                       72  25.4644 0.93922                    
# Total                          73  27.1123 1.00000         

### COMPARISON MSM NEG-ART: Adonis: controlling for additional metadata
metadata_ordered$age <- as.numeric(metadata_ordered$age)
metadata_ordered$BMI <- as.numeric(metadata_ordered$BMI)
metadata_ordered$days_on_art <- as.numeric(metadata_ordered$days_on_art)

### control for metadata with full n
covars_full_n <- c("Ethnicity", "age", "Race", "hiv_phenotype")
print(vegan::adonis2(as.formula(paste("unifrac.distance~metadata_ordered$", paste(covars_full_n, collapse = "+metadata_ordered$"), sep = "")), data=ASV_table, permutations=1000)) -> adon_hiv_phenotype_msm_neg_art_full_n
```

```
## Permutation test for adonis under reduced model
## Terms added sequentially (first to last)
## Permutation: free
## Number of permutations: 1000
## 
## vegan::adonis2(formula = as.formula(paste("unifrac.distance~metadata_ordered$", paste(covars_full_n, collapse = "+metadata_ordered$"), sep = "")), data = ASV_table, permutations = 1000)
##                                Df SumOfSqs      R2      F   Pr(>F)    
## metadata_ordered$Ethnicity      1   0.3770 0.01390 1.0761 0.259740    
## metadata_ordered$age            1   0.3670 0.01354 1.0476 0.311688    
## metadata_ordered$Race           4   1.9088 0.07040 1.3621 0.000999 ***
## metadata_ordered$hiv_phenotype  1   1.3367 0.04930 3.8155 0.000999 ***
## Residual                       66  23.1228 0.85285                    
## Total                          73  27.1123 1.00000                    
## ---
## Signif. codes:  0 '***' 0.001 '**' 0.01 '*' 0.05 '.' 0.1 ' ' 1
```

```
###*** hiv_phenotype r2 = 0.04930 p = 0.000999

#                                Df SumOfSqs      R2      F   Pr(>F)    
# metadata_ordered$Ethnicity      1   0.3770 0.01390 1.0761 0.259740    
# metadata_ordered$age            1   0.3670 0.01354 1.0476 0.311688    
# metadata_ordered$Race           4   1.9088 0.07040 1.3621 0.000999 ***
# metadata_ordered$hiv_phenotype  1   1.3367 0.04930 3.8155 0.000999 ***
# Residual                       66  23.1228 0.85285                    
# Total                          73  27.1123 1.00000                    

### COMPARISON MSM NEG-ART: Adonis: controlling for additional metadata - removing samples w/o values - BMI/comorbidities/days_on_art/ART regimen
### remove samples with BMI/comorbidities/days_on_art/ART regimen is NA (n=74 -> n=62)
ps_gg_fp_f_prevalence_filter_2019_05_26_proportion_bcad <- ps_gg_fp_f_prevalence_filter_2019_05_26_proportion
metadata_ordered_bcad <- metadata_ordered[!is.na(metadata_ordered$hld_hx) & !is.na(metadata_ordered$current_art_class_consolid2) & !is.na(metadata_ordered$BMI) & !is.na(metadata_ordered$days_on_art),,drop=FALSE]
sample_data(ps_gg_fp_f_prevalence_filter_2019_05_26_proportion_bcad) <- metadata_ordered_bcad
```

```
## Found more than one class "phylo" in cache; using the first, from namespace 'phyloseq'
## Also defined by 'tidytree'
```

```
## Found more than one class "phylo" in cache; using the first, from namespace 'phyloseq'
```

```
## Also defined by 'tidytree'
```

```
## Found more than one class "phylo" in cache; using the first, from namespace 'phyloseq'
```

```
## Also defined by 'tidytree'
```

```
## Found more than one class "phylo" in cache; using the first, from namespace 'phyloseq'
```

```
## Also defined by 'tidytree'
```

```
### subset ASV table and distance matrix
ASV_table_bcad<-as.data.frame(otu_table(ps_gg_fp_f_prevalence_filter_2019_05_26_proportion_bcad))
all.equal(row.names(ASV_table_bcad), row.names(metadata_ordered_bcad))
```

```
## [1] TRUE
```

```
unifrac.distance_bcad <- unname(usedist::dist_subset(unifrac.distance, sample_names(ps_gg_fp_f_prevalence_filter_2019_05_26_proportion_bcad))) ### unname fixes error introduced by Desctools see https://github.com/joey711/phyloseq/issues/1457

### run adonis
covars_bcad <- c("BMI", "hld_hx", "htn_hx", "cvd_hx", "dm2hx_dx", "hldhx_dx", "cvdhx_dx", "days_on_art", covars_full_n, "current_art_class_consolid2")
print(vegan::adonis2(as.formula(paste("unifrac.distance_bcad~metadata_ordered_bcad$", paste(covars_bcad, collapse = "+metadata_ordered_bcad$"), sep = "")), data=ASV_table_bcad, permutations=1000)) -> adon_hiv_phenotype_msm_neg_art_full_n_bcad
```

```
## Permutation test for adonis under reduced model
## Terms added sequentially (first to last)
## Permutation: free
## Number of permutations: 1000
## 
## vegan::adonis2(formula = as.formula(paste("unifrac.distance_bcad~metadata_ordered_bcad$", paste(covars_bcad, collapse = "+metadata_ordered_bcad$"), sep = "")), data = ASV_table_bcad, permutations = 1000)
##                                                   Df SumOfSqs      R2      F
## metadata_ordered_bcad$BMI                          1   0.4333 0.01909 1.2523
## metadata_ordered_bcad$hld_hx                       1   0.4858 0.02141 1.4041
## metadata_ordered_bcad$htn_hx                       1   0.4440 0.01957 1.2833
## metadata_ordered_bcad$cvd_hx                       1   0.3829 0.01687 1.1066
## metadata_ordered_bcad$dm2hx_dx                     1   0.4034 0.01777 1.1657
## metadata_ordered_bcad$days_on_art                  1   0.9015 0.03972 2.6053
## metadata_ordered_bcad$Ethnicity                    1   0.3157 0.01391 0.9124
## metadata_ordered_bcad$age                          1   0.3921 0.01728 1.1332
## metadata_ordered_bcad$Race                         4   1.5540 0.06847 1.1228
## metadata_ordered_bcad$hiv_phenotype                1   0.7449 0.03282 2.1529
## metadata_ordered_bcad$current_art_class_consolid2  2   0.7212 0.03178 1.0421
## Residual                                          46  15.9165 0.70131       
## Total                                             61  22.6952 1.00000       
##                                                     Pr(>F)    
## metadata_ordered_bcad$BMI                         0.081918 .  
## metadata_ordered_bcad$hld_hx                      0.040959 *  
## metadata_ordered_bcad$htn_hx                      0.074925 .  
## metadata_ordered_bcad$cvd_hx                      0.238761    
## metadata_ordered_bcad$dm2hx_dx                    0.161838    
## metadata_ordered_bcad$days_on_art                 0.000999 ***
## metadata_ordered_bcad$Ethnicity                   0.609391    
## metadata_ordered_bcad$age                         0.195804    
## metadata_ordered_bcad$Race                        0.093906 .  
## metadata_ordered_bcad$hiv_phenotype               0.001998 ** 
## metadata_ordered_bcad$current_art_class_consolid2 0.338661    
## Residual                                                      
## Total                                                         
## ---
## Signif. codes:  0 '***' 0.001 '**' 0.01 '*' 0.05 '.' 0.1 ' ' 1
```

```
###*** hiv_phenotype r2 = 0.03282 p = 0.001998

#                                                   Df SumOfSqs      R2      F   Pr(>F)    
# metadata_ordered_bcad$BMI                          1   0.4333 0.01909 1.2523 0.106893    
# metadata_ordered_bcad$hld_hx                       1   0.4858 0.02141 1.4041 0.041958 *  
# metadata_ordered_bcad$htn_hx                       1   0.4440 0.01957 1.2833 0.086913 .  
# metadata_ordered_bcad$cvd_hx                       1   0.3829 0.01687 1.1066 0.224775    
# metadata_ordered_bcad$dm2hx_dx                     1   0.4034 0.01777 1.1657 0.176823    
# metadata_ordered_bcad$days_on_art                  1   0.9015 0.03972 2.6053 0.000999 ***
# metadata_ordered_bcad$Ethnicity                    1   0.3157 0.01391 0.9124 0.632368    
# metadata_ordered_bcad$age                          1   0.3921 0.01728 1.1332 0.186813    
# metadata_ordered_bcad$Race                         4   1.5540 0.06847 1.1228 0.097902 .  
# metadata_ordered_bcad$hiv_phenotype                1   0.7449 0.03282 2.1529 0.000999 ***
# metadata_ordered_bcad$current_art_class_consolid2  2   0.7212 0.03178 1.0421 0.313686    
# Residual                                          46  15.9165 0.70131                    
# Total                                             61  22.6952 1.00000                   


#COMPARISON MSM
#Comparison NEG-UNSUPPRESSED
#Transform count data in the phyloseq object
ps_gg_fp_f_prevalence_filter_2019_05_26_proportion<-transform_sample_counts(ps_gg_fp_f_prevalence_filter_2019_05_26, function(x)(x/sum(x)))
```

```
## Found more than one class "phylo" in cache; using the first, from namespace 'phyloseq'
## Also defined by 'tidytree'
```

```
## Found more than one class "phylo" in cache; using the first, from namespace 'phyloseq'
```

```
## Also defined by 'tidytree'
```

```
## Found more than one class "phylo" in cache; using the first, from namespace 'phyloseq'
```

```
## Also defined by 'tidytree'
```

```
## Found more than one class "phylo" in cache; using the first, from namespace 'phyloseq'
```

```
## Also defined by 'tidytree'
```

```
#Select samples of interest and update phyloseq object 
metadata<-phyloseq::sample_data(ps_gg_fp_f_prevalence_filter_2019_05_26)
metadata<-metadata[metadata$sample_cohort=="boston",,drop=F]
metadata<-metadata[metadata$hiv_phenotype%in%c("1_hiv_negative","4_unsuppressed"),,drop=F]
metadata<-as.data.frame(as.matrix(metadata[metadata$sexual_orientation=="MSM",,drop=F]))
phyloseq::sample_data(ps_gg_fp_f_prevalence_filter_2019_05_26_proportion)<-metadata
```

```
## Found more than one class "phylo" in cache; using the first, from namespace 'phyloseq'
## Also defined by 'tidytree'
```

```
## Found more than one class "phylo" in cache; using the first, from namespace 'phyloseq'
```

```
## Also defined by 'tidytree'
```

```
## Found more than one class "phylo" in cache; using the first, from namespace 'phyloseq'
```

```
## Also defined by 'tidytree'
```

```
## Found more than one class "phylo" in cache; using the first, from namespace 'phyloseq'
```

```
## Also defined by 'tidytree'
```

```
#Run PCoA on the phyloseq object
ordination<-phyloseq::ordinate(ps_gg_fp_f_prevalence_filter_2019_05_26_proportion, "PCoA", "unifrac")
```

```
## Warning in matrix(tree$edge[order(tree$edge[, 1]), ][, 2], byrow = TRUE, : data
## length [8987] is not a sub-multiple or multiple of the number of rows [4494]
```

```
ordination$values[1:2,]
```

```
##   Eigenvalues Relative_eig Broken_stick  Cumul_eig Cumul_br_stick
## 1    2.776310   0.09857079   0.06535141 0.09857079     0.06535141
## 2    2.131222   0.07566743   0.05201808 0.17423822     0.11736948
```

```
metadata_ordered<-metadata[row.names(ordination$vectors),,drop=FALSE]

all.equal(row.names(metadata_ordered), row.names(ordination$vectors))
```

```
## [1] TRUE
```

```
metadata_ordered$Unifrac1<-ordination$vectors[,1]
metadata_ordered$Unifrac2<-ordination$vectors[,2]

#Let's plot the data
plot_MSM_NEG_UNSUP<-ggplot2::ggplot(data=metadata_ordered, aes(x=Unifrac1, y=Unifrac2))+geom_point(color="royalblue4", aes(alpha=hiv_phenotype), size=2, shape=16)+
  theme_bw()+stat_ellipse(color="royalblue4", aes(alpha=hiv_phenotype), size=1)+scale_alpha_manual(values=c(1,0.3))+ylim(c(-0.55, 0.55))+xlim(c(-0.55,0.55))+
  ggtitle("neg-unsuppressed MSM")+geom_point(data=metadata_ordered %>% group_by(hiv_phenotype) %>% summarise_at(vars(matches("UniFrac")), mean),size=5, color="royalblue4", aes(alpha=hiv_phenotype))

#Adonis (n=76)
ASV_table<-as.data.frame(phyloseq::otu_table(ps_gg_fp_f_prevalence_filter_2019_05_26_proportion))
all.equal(row.names(ASV_table), row.names(metadata_ordered))
```

```
## [1] TRUE
```

```
unifrac.distance<-unname(phyloseq::UniFrac(ps_gg_fp_f_prevalence_filter_2019_05_26_proportion, weighted = FALSE)) ### unname fixes error introduced by Desctools see https://github.com/joey711/phyloseq/issues/1457
```

```
## Warning in matrix(tree$edge[order(tree$edge[, 1]), ][, 2], byrow = TRUE, : data
## length [8987] is not a sub-multiple or multiple of the number of rows [4494]
```

```
attributes(unifrac.distance)$Labels <- phyloseq::sample_names(ps_gg_fp_f_prevalence_filter_2019_05_26_proportion)
print(vegan::adonis2(unifrac.distance~metadata_ordered$hiv_phenotype, data=ASV_table, permutations=1000)) -> adon_hiv_phenotype_msm_neg_unsup
```

```
## Permutation test for adonis under reduced model
## Terms added sequentially (first to last)
## Permutation: free
## Number of permutations: 1000
## 
## vegan::adonis2(formula = unifrac.distance ~ metadata_ordered$hiv_phenotype, data = ASV_table, permutations = 1000)
##                                Df SumOfSqs      R2      F   Pr(>F)    
## metadata_ordered$hiv_phenotype  1   1.3672 0.04854 3.7753 0.000999 ***
## Residual                       74  26.7985 0.95146                    
## Total                          75  28.1657 1.00000                    
## ---
## Signif. codes:  0 '***' 0.001 '**' 0.01 '*' 0.05 '.' 0.1 ' ' 1
```

```
###*** r2 = 0.04854 p = 0.000999

# vegan::adonis2(formula = unifrac.distance ~ metadata_ordered$hiv_phenotype, data = ASV_table, permutations = 1000)
#                                Df SumOfSqs      R2      F   Pr(>F)    
# metadata_ordered$hiv_phenotype  1   1.3672 0.04854 3.7753 0.000999 ***
# Residual                       74  26.7985 0.95146                    
# Total                          75  28.1657 1.00000              

### COMPARISON MSM NEG-UNSUPPRESSED: Adonis: controlling for additional metadata
### Extra metadata that have full n: Race, Ethnicity, age, sex, tmp_smx_active
metadata_ordered$age <- as.numeric(metadata_ordered$age)
metadata_ordered$BMI <- as.numeric(metadata_ordered$BMI)

### control for metadata with full n
covars_full_n <- c("Ethnicity", "age", "Race", "tmp_smx_active", "hiv_phenotype")
print(vegan::adonis2(as.formula(paste("unifrac.distance~metadata_ordered$", paste(covars_full_n, collapse = "+metadata_ordered$"), sep = "")), data=ASV_table, permutations=1000)) -> adon_hiv_phenotype_msm_neg_unsup_full_n
```

```
## Permutation test for adonis under reduced model
## Terms added sequentially (first to last)
## Permutation: free
## Number of permutations: 1000
## 
## vegan::adonis2(formula = as.formula(paste("unifrac.distance~metadata_ordered$", paste(covars_full_n, collapse = "+metadata_ordered$"), sep = "")), data = ASV_table, permutations = 1000)
##                                 Df SumOfSqs      R2      F   Pr(>F)    
## metadata_ordered$Ethnicity       1   0.3394 0.01205 0.9447 0.567433    
## metadata_ordered$age             1   0.4764 0.01691 1.3260 0.064935 .  
## metadata_ordered$Race            4   1.7162 0.06093 1.1942 0.025974 *  
## metadata_ordered$tmp_smx_active  1   0.3408 0.01210 0.9487 0.574426    
## metadata_ordered$hiv_phenotype   1   1.2212 0.04336 3.3990 0.000999 ***
## Residual                        67  24.0716 0.85465                    
## Total                           75  28.1657 1.00000                    
## ---
## Signif. codes:  0 '***' 0.001 '**' 0.01 '*' 0.05 '.' 0.1 ' ' 1
```

```
###*** hiv_phenotype r2 = 0.04336 p = 0.000999 

#                                 Df SumOfSqs      R2      F   Pr(>F)    
# metadata_ordered$Ethnicity       1   0.3394 0.01205 0.9447 0.567433    
# metadata_ordered$age             1   0.4764 0.01691 1.3260 0.064935 .  
# metadata_ordered$Race            4   1.7162 0.06093 1.1942 0.025974 *  
# metadata_ordered$tmp_smx_active  1   0.3408 0.01210 0.9487 0.574426    
# metadata_ordered$hiv_phenotype   1   1.2212 0.04336 3.3990 0.000999 ***
# Residual                        67  24.0716 0.85465                    
# Total                           75  28.1657 1.00000                   

### COMPARISON MSM NEG-UNSUPPRESSED: Adonis: controlling for additional metadata - removing samples w/o values - BMI/comorbidities
### remove samples with BMI/comorbidities is NA (n=76 -> n=65)
ps_gg_fp_f_prevalence_filter_2019_05_26_proportion_bc <- ps_gg_fp_f_prevalence_filter_2019_05_26_proportion
metadata_ordered_bc <- metadata_ordered[!is.na(metadata_ordered$hld_hx) &!is.na(metadata_ordered$BMI),,drop=FALSE]
sample_data(ps_gg_fp_f_prevalence_filter_2019_05_26_proportion_bc) <- metadata_ordered_bc
```

```
## Found more than one class "phylo" in cache; using the first, from namespace 'phyloseq'
## Also defined by 'tidytree'
```

```
## Found more than one class "phylo" in cache; using the first, from namespace 'phyloseq'
```

```
## Also defined by 'tidytree'
```

```
## Found more than one class "phylo" in cache; using the first, from namespace 'phyloseq'
```

```
## Also defined by 'tidytree'
```

```
## Found more than one class "phylo" in cache; using the first, from namespace 'phyloseq'
```

```
## Also defined by 'tidytree'
```

```
### subset ASV table and distance matrix
ASV_table_bc<-as.data.frame(otu_table(ps_gg_fp_f_prevalence_filter_2019_05_26_proportion_bc))
all.equal(row.names(ASV_table_bc), row.names(metadata_ordered_bc))
```

```
## [1] TRUE
```

```
unifrac.distance_bc <- unname(usedist::dist_subset(unifrac.distance, sample_names(ps_gg_fp_f_prevalence_filter_2019_05_26_proportion_bc))) ### unname fixes error introduced by Desctools see https://github.com/joey711/phyloseq/issues/1457

### run adonis
covars_bc <- c("BMI", "hld_hx", "htn_hx", "cvd_hx", "dm2hx_dx", "hldhx_dx", "cvdhx_dx", covars_full_n)
print(vegan::adonis2(as.formula(paste("unifrac.distance_bc~metadata_ordered_bc$", paste(covars_bc, collapse = "+metadata_ordered_bc$"), sep = "")), data=ASV_table_bc, permutations=1000)) -> adon_hiv_phenotype_msm_neg_unsup_full_n_bc
```

```
## Permutation test for adonis under reduced model
## Terms added sequentially (first to last)
## Permutation: free
## Number of permutations: 1000
## 
## vegan::adonis2(formula = as.formula(paste("unifrac.distance_bc~metadata_ordered_bc$", paste(covars_bc, collapse = "+metadata_ordered_bc$"), sep = "")), data = ASV_table_bc, permutations = 1000)
##                                    Df SumOfSqs      R2      F   Pr(>F)    
## metadata_ordered_bc$BMI             1   0.4485 0.01880 1.2756 0.085914 .  
## metadata_ordered_bc$hld_hx          1   0.4089 0.01714 1.1629 0.165834    
## metadata_ordered_bc$htn_hx          1   0.3636 0.01524 1.0341 0.347652    
## metadata_ordered_bc$cvd_hx          1   0.4794 0.02009 1.3634 0.041958 *  
## metadata_ordered_bc$dm2hx_dx        1   0.3553 0.01489 1.0107 0.405594    
## metadata_ordered_bc$Ethnicity       1   0.2665 0.01117 0.7579 0.960040    
## metadata_ordered_bc$age             1   0.4674 0.01959 1.3294 0.043956 *  
## metadata_ordered_bc$Race            4   1.7139 0.07183 1.2187 0.023976 *  
## metadata_ordered_bc$tmp_smx_active  1   0.3446 0.01444 0.9801 0.507493    
## metadata_ordered_bc$hiv_phenotype   1   1.0810 0.04531 3.0748 0.000999 ***
## Residual                           51  17.9309 0.75151                    
## Total                              64  23.8600 1.00000                    
## ---
## Signif. codes:  0 '***' 0.001 '**' 0.01 '*' 0.05 '.' 0.1 ' ' 1
```

```
###*** hiv_phenotype r2 = 0.04531 p = 0.000999


#                                    Df SumOfSqs      R2      F   Pr(>F)    
# metadata_ordered_bc$BMI             1   0.4485 0.01880 1.2756 0.067932 .  
# metadata_ordered_bc$hld_hx          1   0.4089 0.01714 1.1629 0.150849    
# metadata_ordered_bc$htn_hx          1   0.3636 0.01524 1.0341 0.368631    
# metadata_ordered_bc$cvd_hx          1   0.4794 0.02009 1.3634 0.036963 *  
# metadata_ordered_bc$dm2hx_dx        1   0.3553 0.01489 1.0107 0.371628    
# metadata_ordered_bc$Ethnicity       1   0.2665 0.01117 0.7579 0.967033    
# metadata_ordered_bc$age             1   0.4674 0.01959 1.3294 0.045954 *  
# metadata_ordered_bc$Race            4   1.7139 0.07183 1.2187 0.007992 ** 
# metadata_ordered_bc$tmp_smx_active  1   0.3446 0.01444 0.9801 0.477522    
# metadata_ordered_bc$hiv_phenotype   1   1.0810 0.04531 3.0748 0.000999 ***
# Residual                           51  17.9309 0.75151                    
# Total                              64  23.8600 1.00000                    

#Let's plot all 4 PCoA
ggsave("Figure3A_v1.pdf", grid.arrange(plot_noMSM_NEG_ART, plot_noMSM_NEG_UNSUP, plot_MSM_NEG_ART, plot_MSM_NEG_UNSUP,ncol=2, nrow=2), 
       width=15, height=10)
```

```
#--------------------------------------------------------------------------------------------------------------
```

#Figure 3C: Figure 3B uses agricolae which conflicts with ancom, so
Figure 3C is run first

```
#FIGURE 3C: Figure 3B uses agricolae which conflicts with ancom, so Figure 3C is run first
#--------------------------------------------------------------------------------------------------------------
###RUN ANCOM MSM NEG vs UNTREATED###
#Transform count data in the phyloseq object
ps_gg_fp_f_prevalence_filter_2019_05_26_proportion <- phyloseq::transform_sample_counts(ps_gg_fp_f_prevalence_filter_2019_05_26, function(x)(x/sum(x)))
```

```
## Found more than one class "phylo" in cache; using the first, from namespace 'phyloseq'
```

```
## Also defined by 'tidytree'
```

```
## Found more than one class "phylo" in cache; using the first, from namespace 'phyloseq'
```

```
## Also defined by 'tidytree'
```

```
## Found more than one class "phylo" in cache; using the first, from namespace 'phyloseq'
```

```
## Also defined by 'tidytree'
```

```
## Found more than one class "phylo" in cache; using the first, from namespace 'phyloseq'
```

```
## Also defined by 'tidytree'
```

```
#Select samples of interest and update phyloseq object
dataset<-ps_gg_fp_f_prevalence_filter_2019_05_26
metadata<-as.data.frame(sample_data(ps_gg_fp_f_prevalence_filter_2019_05_26))
metadata<-metadata[metadata$hiv_phenotype %in% c("1_hiv_negative", "4_unsuppressed"), , drop=F]
metadata<-as.data.frame(as.matrix(metadata[metadata$sexual_orientation == "MSM" | is.na(metadata$sexual_orientation), , drop=F]))
metadata_boston<-metadata[metadata$sample_cohort == "boston", , drop=F]
sample_data(dataset)<-metadata_boston
```

```
## Found more than one class "phylo" in cache; using the first, from namespace 'phyloseq'
## Also defined by 'tidytree'
```

```
## Found more than one class "phylo" in cache; using the first, from namespace 'phyloseq'
```

```
## Also defined by 'tidytree'
```

```
## Found more than one class "phylo" in cache; using the first, from namespace 'phyloseq'
```

```
## Also defined by 'tidytree'
```

```
## Found more than one class "phylo" in cache; using the first, from namespace 'phyloseq'
```

```
## Also defined by 'tidytree'
```

```
ps.taxa.sub <- phyloseq::prune_taxa(taxa_sums(dataset) > 0, dataset)
```

```
## Found more than one class "phylo" in cache; using the first, from namespace 'phyloseq'
## Also defined by 'tidytree'
```

```
out <- ANCOMBC::ancombc2(data = ps.taxa.sub, assay_name = "counts", tax_level = NULL, fix_formula = "hiv_phenotype", 
              p_adj_method = "BH", pseudo = 0, pseudo_sens = FALSE, prv_cut = 0.05, lib_cut = 1000, 
              group = "hiv_phenotype", struc_zero = TRUE, neg_lb = FALSE, alpha = 0.05, n_cl = 6, global = TRUE,
              em_control = list(tol = 1e-05, max_iter = 100), mdfdr_control = list(fwer_ctrl_method = "holm", B = 100))
```

```
## Found more than one class "phylo" in cache; using the first, from namespace 'phyloseq'
## Also defined by 'tidytree'
```

```
## Found more than one class "phylo" in cache; using the first, from namespace 'phyloseq'
```

```
## Also defined by 'tidytree'
```

```
## `tax_level` is not speficified 
## No agglomeration will be performed
## Otherwise, please speficy `tax_level` by one of the following: 
## Kingdom, Phylum, Class, Order, Family, Genus, Species
```

```
## Found more than one class "phylo" in cache; using the first, from namespace 'phyloseq'
```

```
## Also defined by 'tidytree'
```

```
## Found more than one class "phylo" in cache; using the first, from namespace 'phyloseq'
```

```
## Also defined by 'tidytree'
```

```
## Found more than one class "phylo" in cache; using the first, from namespace 'phyloseq'
```

```
## Also defined by 'tidytree'
```

```
## Found more than one class "phylo" in cache; using the first, from namespace 'phyloseq'
```

```
## Also defined by 'tidytree'
```

```
## Found more than one class "phylo" in cache; using the first, from namespace 'phyloseq'
```

```
## Also defined by 'tidytree'
```

```
## Found more than one class "phylo" in cache; using the first, from namespace 'phyloseq'
```

```
## Also defined by 'tidytree'
```

```
## Warning: The group variable has < 3 categories 
## The multi-group comparisons (global/pairwise/dunnet/trend) will be deactivated
```

```
res_df <- out$res
res_df <- dplyr::rename(res_df, rowname = taxon)
colnames(res_df) <- stringr::str_replace(colnames(res_df), "hiv_phenotype.+", "hiv_phenotype")
res_df_taxa <- dplyr::left_join(res_df, tibble::rownames_to_column(as.data.frame(phyloseq::tax_table(ps.taxa.sub))), by = "rowname")
res_df_taxa[["index_num"]] <- 1:nrow(res_df_taxa)
res_df_taxa[["cohort"]] <- "boston"
res_df_taxa[["cohort_2"]] <- "boston_msm_neg_untreat"
res_df_taxa[["method"]] <- "ancom"
res_df_taxa <- tidyr::unite(res_df_taxa, col =  "Genus_Species", Genus, Species, index_num, remove = FALSE)
alpha = 0.05
taxa_sig <- dplyr::filter(res_df_taxa, q_hiv_phenotype < 0.05)
taxa_sig$Genus_Species <- forcats::fct_reorder(taxa_sig$Genus_Species, taxa_sig$lfc_hiv_phenotype, min)
taxa_sig$rowname_short <- stringr::str_sub(taxa_sig$rowname, 1, 4)
ps.taxa.rel.sig <- phyloseq::prune_taxa(taxa_sig[["rowname"]], ps_gg_fp_f_prevalence_filter_2019_05_26_proportion)
```

```
## Found more than one class "phylo" in cache; using the first, from namespace 'phyloseq'
## Also defined by 'tidytree'
```

```
# Only keep filtered samples 
ps.taxa.rel.sig <- phyloseq::prune_samples(rownames(phyloseq::otu_table(ps.taxa.sub)), ps.taxa.rel.sig)
sigtab_dataset_neg_untreat <- taxa_sig
write.csv(sigtab_dataset_neg_untreat, "ANCOM_MSM_NEG_UNTREATED.csv")
ggsave("Figure3C_NEG_UNTREATANCOM_v5.pdf", ggplot(data = sigtab_dataset_neg_untreat, aes(x = Genus_Species, y = lfc_hiv_phenotype)) + theme_bw() + coord_flip() + geom_bar(stat="identity", aes(fill = Genus)) + ggtitle("MSM NEG-UNTREAT") + theme(legend.position = "bottom"), width = 10, height = 10, units = "in", dpi = 300)


###RUN ANCOM MSM NEG vs ART###
#Transform count data in the phyloseq object
ps_gg_fp_f_prevalence_filter_2019_05_26_proportion<-transform_sample_counts(ps_gg_fp_f_prevalence_filter_2019_05_26, function(x)(x/sum(x)))
```

```
## Found more than one class "phylo" in cache; using the first, from namespace 'phyloseq'
## Also defined by 'tidytree'
```

```
## Found more than one class "phylo" in cache; using the first, from namespace 'phyloseq'
```

```
## Also defined by 'tidytree'
```

```
## Found more than one class "phylo" in cache; using the first, from namespace 'phyloseq'
```

```
## Also defined by 'tidytree'
```

```
## Found more than one class "phylo" in cache; using the first, from namespace 'phyloseq'
```

```
## Also defined by 'tidytree'
```

```
#Select samples of interest and update phyloseq object
dataset<-ps_gg_fp_f_prevalence_filter_2019_05_26
metadata<-as.data.frame(sample_data(ps_gg_fp_f_prevalence_filter_2019_05_26))
metadata<-metadata[metadata$hiv_phenotype %in% c("1_hiv_negative", "2_suppressed"), , drop=F]
metadata<-as.data.frame(as.matrix(metadata[metadata$sexual_orientation == "MSM" | is.na(metadata$sexual_orientation), , drop=F]))
metadata_boston<-metadata[metadata$sample_cohort=="boston", , drop=F]
sample_data(dataset)<-metadata_boston
```

```
## Found more than one class "phylo" in cache; using the first, from namespace 'phyloseq'
## Also defined by 'tidytree'
```

```
## Found more than one class "phylo" in cache; using the first, from namespace 'phyloseq'
```

```
## Also defined by 'tidytree'
```

```
## Found more than one class "phylo" in cache; using the first, from namespace 'phyloseq'
```

```
## Also defined by 'tidytree'
```

```
## Found more than one class "phylo" in cache; using the first, from namespace 'phyloseq'
```

```
## Also defined by 'tidytree'
```

```
ps.taxa.sub <- phyloseq::prune_taxa(taxa_sums(dataset) > 0, dataset)
```

```
## Found more than one class "phylo" in cache; using the first, from namespace 'phyloseq'
## Also defined by 'tidytree'
```

```
out <- ANCOMBC::ancombc2(data = ps.taxa.sub, assay_name = "counts", tax_level = NULL, fix_formula = "hiv_phenotype", 
              p_adj_method = "BH", pseudo = 0, pseudo_sens = FALSE, prv_cut = 0.05, lib_cut = 1000, 
              group = "hiv_phenotype", struc_zero = TRUE, neg_lb = FALSE, alpha = 0.05, n_cl = 6, global = TRUE,
              em_control = list(tol = 1e-05, max_iter = 100), mdfdr_control = list(fwer_ctrl_method = "holm", B = 100))
```

```
## Found more than one class "phylo" in cache; using the first, from namespace 'phyloseq'
## Also defined by 'tidytree'
```

```
## Found more than one class "phylo" in cache; using the first, from namespace 'phyloseq'
```

```
## Also defined by 'tidytree'
```

```
## `tax_level` is not speficified 
## No agglomeration will be performed
## Otherwise, please speficy `tax_level` by one of the following: 
## Kingdom, Phylum, Class, Order, Family, Genus, Species
```

```
## Found more than one class "phylo" in cache; using the first, from namespace 'phyloseq'
```

```
## Also defined by 'tidytree'
```

```
## Found more than one class "phylo" in cache; using the first, from namespace 'phyloseq'
```

```
## Also defined by 'tidytree'
```

```
## Found more than one class "phylo" in cache; using the first, from namespace 'phyloseq'
```

```
## Also defined by 'tidytree'
```

```
## Found more than one class "phylo" in cache; using the first, from namespace 'phyloseq'
```

```
## Also defined by 'tidytree'
```

```
## Found more than one class "phylo" in cache; using the first, from namespace 'phyloseq'
```

```
## Also defined by 'tidytree'
```

```
## Found more than one class "phylo" in cache; using the first, from namespace 'phyloseq'
```

```
## Also defined by 'tidytree'
```

```
## Warning: The group variable has < 3 categories 
## The multi-group comparisons (global/pairwise/dunnet/trend) will be deactivated
```

```
res_df <- out$res
res_df <- dplyr::rename(res_df, rowname = taxon)
colnames(res_df) <- stringr::str_replace(colnames(res_df), "hiv_phenotype.+", "hiv_phenotype")
res_df_taxa <- dplyr::left_join(res_df, tibble::rownames_to_column(as.data.frame(phyloseq::tax_table(ps.taxa.sub))), by = "rowname")
res_df_taxa[["index_num"]] <- 1:nrow(res_df_taxa)
res_df_taxa[["cohort"]] <- "boston"
res_df_taxa[["cohort_2"]] <- "boston_msm_neg_art"
res_df_taxa[["method"]] <- "ancom"
res_df_taxa <- tidyr::unite(res_df_taxa, col =  "Genus_Species", Genus, Species, index_num, remove = FALSE)
alpha = 0.05
taxa_sig <- dplyr::filter(res_df_taxa, q_hiv_phenotype < 0.05)
taxa_sig$Genus_Species <- forcats::fct_reorder(taxa_sig$Genus_Species, taxa_sig$lfc_hiv_phenotype, min)
taxa_sig$rowname_short <- stringr::str_sub(taxa_sig$rowname, 1, 4)
ps.taxa.rel.sig <- phyloseq::prune_taxa(taxa_sig[["rowname"]], ps_gg_fp_f_prevalence_filter_2019_05_26_proportion)
```

```
## Found more than one class "phylo" in cache; using the first, from namespace 'phyloseq'
## Also defined by 'tidytree'
```

```
# Only keep filtered samples 
ps.taxa.rel.sig <- phyloseq::prune_samples(rownames(phyloseq::otu_table(ps.taxa.sub)), ps.taxa.rel.sig)
sigtab_dataset_neg_art <- taxa_sig
write.csv(sigtab_dataset_neg_art, "ANCOM_MSM_NEG_ART.csv")
ggsave("Figure3C_NEG_ARTANCOM_v5.pdf", ggplot(data = sigtab_dataset_neg_art, aes(x = Genus_Species, y = lfc_hiv_phenotype)) + theme_bw() + coord_flip() + geom_bar(stat="identity", aes(fill = Genus)) + ggtitle("MSM NEG-ART") + theme(legend.position = "bottom"), width = 10, height = 10, units = "in", dpi = 300)


#Merge results from both sample cohorts:
sigtab_dataset<-rbind(sigtab_dataset_neg_art, sigtab_dataset_neg_untreat)
sigtab_dataset[["index_num_all"]] <- 1:nrow(sigtab_dataset)
sigtab_dataset <- tidyr::unite(sigtab_dataset, col =  "Genus_Species_all", Genus, Species, index_num_all, sep = "_", remove = FALSE)
sigtab_dataset <- tidyr::unite(sigtab_dataset, col =  "Genus_Species_all_for_color", Genus, Species, sep = " ", remove = FALSE)

# Remove extra brackets around taxonomic name for labels
sigtab_dataset$Genus_label <- stringr::str_replace(sigtab_dataset$Genus, "^\\[([^\\]]+)\\]", "\\1")
sigtab_dataset$Species_label <- stringr::str_replace(sigtab_dataset$Species, "^\\[([^\\]]+)\\]", "\\1")
sigtab_dataset %>% dplyr::mutate(Genus_species_label =
                dplyr::case_when(is.na(stringr::str_extract(Genus_label, "\\[")) & is.na(stringr::str_extract(Species_label, "\\[")) ~ paste(Genus_label,Species_label),
                                 TRUE ~ paste(Genus_label,"sp"))) -> sigtab_dataset
sigtab_dataset$Genus_species_label <- stringr::str_replace(sigtab_dataset$Genus_species_label, "\\[([^\\]]+)\\]", "")
sigtab_dataset$Genus_species_label <- stringr::str_replace(sigtab_dataset$Genus_species_label, "\\ +", " ")
sigtab_dataset<-sigtab_dataset[order(sigtab_dataset$lfc_hiv_phenotype, decreasing = FALSE),,drop=FALSE]
sigtab_dataset$Genus_Species_all<-factor(sigtab_dataset$Genus_Species_all, levels = c(sigtab_dataset$Genus_Species_all))

# Make lookup table for Genus_species_label
Genus_species_label_lookup <- dplyr::distinct(sigtab_dataset, Genus_Species_all, .keep_all = TRUE)$Genus_species_label
names(Genus_species_label_lookup) <- dplyr::distinct(sigtab_dataset, Genus_Species_all, .keep_all = TRUE)$Genus_Species_all

#Load color dictionary and construct figure:
dictionary_unique <- read.csv("COLOR_DICTIONARY3.csv", sep=";")
sigtab_dataset$Genus_species_label <- factor(sigtab_dataset$Genus_species_label, levels = unique(sigtab_dataset$Genus_species_label))
dictionary_plot <- dictionary_unique[dictionary_unique$Genus_species_label%in%sigtab_dataset$Genus_species_label,,drop=F]
rownames(dictionary_plot)<-dictionary_plot$Genus_species_label
dictionary_plot<-dictionary_plot[as.character(unique(sigtab_dataset$Genus_species_label)),,drop=F]
sigtab_dataset$cohort_2 <- fct_relevel(as.factor(sigtab_dataset$cohort_2), "boston_msm_neg_untreat", "boston_msm_neg_art")
header_namer <- as_labeller(c(`boston_msm_neg_art` = "HIV-uninfected    HIV+ ART-treated", `boston_msm_neg_untreat` = "HIV-uninfected   HIV+ untreated"))

ggsave("Figure3C_v10.pdf", 
       ggplot(data = sigtab_dataset, aes(x = Genus_Species_all, y = lfc_hiv_phenotype)) +
         geom_bar(stat = "identity", aes(fill = Genus_species_label)) +
         scale_fill_manual(values = as.character(dictionary_plot$Color)) +
         coord_flip() + scale_x_discrete(label = as_labeller(Genus_species_label_lookup)) + theme_bw() +
         theme(legend.position = "bottom", axis.text.x = element_text(size = 14), axis.text.y = element_text(size = 10, face = "plain"), axis.ticks.y = element_blank(), 
               panel.border = element_rect(linetype = "solid", fill = NA, linewidth = 1), 
               strip.background = element_rect(colour = "white", fill = "white"), strip.text = element_text(colour = "black", face = "bold", size = rel(1.2))) + 
         geom_hline(yintercept = 0) + ggtitle("MSM") + ylab("log2FoldChange") +
         facet_wrap(vars(cohort_2), scales = "free", ncol = 3, labeller = labeller(cohort_2 = header_namer)), width = 15, height = 20, units = "in", dpi = 300)

#--------------------------------------------------------------------------------------------------------------
```

#Figure 3B

```
#--------------------------------------------------------------------------------------------------------------
require("agricolae")
```

```
## Loading required package: agricolae
```

```
## Registered S3 methods overwritten by 'klaR':
##   method      from 
##   predict.rda vegan
##   print.rda   vegan
##   plot.rda    vegan
```

```
#Prepare OTU table and taxonomy files out from the phyloseq object:
OTU_table<-as.data.frame(otu_table(ps_gg_fp_f_prevalence_filter_2019_05_26))
Taxonomy<-as.data.frame(tax_table(ps_gg_fp_f_prevalence_filter_2019_05_26))
metadata<-as.data.frame(sample_data(ps_gg_fp_f_prevalence_filter_2019_05_26))

#Find the sample with the smallest number of reads. It will be the value used to normalize all data so all samples will be comparable among them:
rowSums(OTU_table)
```

```
## 105574.boston1.0139.2014.12.08 108777.boston1.0140.2014.12.08 
##                          72609                          64328 
## 112993.boston1.0141.2014.12.08 123656.boston1.0005.2014.12.08 
##                          79920                          36479 
## 143200.boston1.0006.2014.12.08 153724.boston1.0007.2014.12.08 
##                          81427                          85980 
## 165642.boston1.0008.2014.12.08 194317.boston1.0010.2014.12.08 
##                          98119                          53263 
## 196203.boston1.0011.2014.12.08 205120.boston1.0013.2014.12.08 
##                          36651                          59443 
## 211774.boston1.0014.2014.12.08 228437.boston1.0017.2014.12.08 
##                          57586                          52936 
## 229969.boston1.0018.2014.12.08 237983.boston1.0019.2014.12.08 
##                          62357                          37574 
## 258085.boston1.0142.2014.12.08 273479.boston1.0143.2014.12.08 
##                          59728                         102691 
## 315504.boston1.0028.2014.12.08 330183.boston1.0144.2014.12.08 
##                          21399                          68760 
## 337016.boston1.0030.2014.12.08 365685.boston1.0032.2014.12.08 
##                          43655                          41183 
## 386576.boston1.0035.2014.12.08 389876.boston1.0036.2014.12.08 
##                          36454                          66739 
## 410644.boston1.0125.2014.12.08 410932.boston1.0039.2014.12.08 
##                          73269                          27289 
## 413736.boston1.0126.2014.12.08 427838.boston1.0127.2014.12.08 
##                          39912                          66847 
## 453548.boston1.0045.2014.12.08 460380.boston1.0046.2014.12.08 
##                          34382                          31329 
## 473516.boston1.0047.2014.12.08 479693.boston1.0048.2014.12.08 
##                          46039                          18765 
## 485548.boston1.0049.2014.12.08 498553.boston1.0050.2014.12.08 
##                          35582                          56405 
## 505402.boston1.0051.2014.12.08 516980.boston1.0130.2014.12.08 
##                          59815                          14678 
## 522458.boston1.0132.2014.12.08 526318.boston1.0133.2014.12.08 
##                          68609                          27811 
## 527968.boston1.0057.2014.12.08 529516.boston1.0136.2014.12.08 
##                          26204                          20681 
## 533586.boston1.0059.2014.12.08 534694.boston1.0060.2014.12.08 
##                          57983                          51661 
## 604772.boston1.0071.2014.12.08 614225.boston1.0073.2014.12.08 
##                          52199                          47445 
## 615167.boston1.0074.2014.12.08 616147.boston1.0075.2014.12.08 
##                          37085                          50711 
## 653425.boston1.0077.2014.12.08 666207.boston1.0079.2014.12.08 
##                          42115                          70102 
## 694413.boston1.0080.2014.12.08 708968.boston1.0083.2014.12.08 
##                          44825                          28721 
## 734962.boston1.0085.2014.12.08 745577.boston1.0086.2014.12.08 
##                          43153                          75694 
## 758572.boston1.0088.2014.12.08 775609.boston1.0091.2014.12.08 
##                          63237                          64948 
## 813341.boston1.0095.2014.12.08 819622.boston1.0096.2014.12.08 
##                          74295                          34279 
## 842279.boston1.0097.2014.12.08 847041.boston1.0098.2014.12.08 
##                          61932                          59051 
## 862898.boston1.0103.2014.12.08 874612.boston1.0105.2014.12.08 
##                          38913                          21417 
## 880160.boston1.0106.2014.12.08 899025.boston1.0107.2014.12.08 
##                          61338                          51550 
## 900158.boston1.0108.2014.12.08 911594.boston1.0109.2014.12.08 
##                          18175                          54168 
## 923358.boston1.0111.2014.12.08 950965.boston1.0116.2014.12.08 
##                          34979                          66238 
## 953586.boston1.0117.2014.12.08 958793.boston1.0118.2014.12.08 
##                          54430                          66440 
## 966971.boston1.0120.2014.12.08 970489.boston1.0121.2014.12.08 
##                          44109                          48095 
## 995725.boston1.0123.2014.12.08  529863.boston.0165.2017.04.06 
##                          58954                          42388 
##  608647.boston.0072.2017.04.06    686039.0040.0323.2017.04.06 
##                          31028                         374932 
##   WT24922.0093.0468.2017.04.06    102438.0086.0363.2017.03.15 
##                         107819                          25113 
##    106085.0054.0333.2017.03.15    122897.0017.0307.2017.03.15 
##                          19135                          80414 
##    129226.0089.0367.2017.03.15    136376.0013.0302.2017.03.15 
##                          33236                          27232 
##    148342.0027.0314.2017.03.15    157072.0043.0244.2017.03.15 
##                          45438                          22883 
##    175067.0033.0231.2017.03.15    181090.0091.0369.2017.03.15 
##                          15936                          22823 
##    189326.0070.0343.2017.03.15    191447.0008.0296.2017.03.15 
##                          61451                          62556 
##    207295.0010.0298.2017.03.15    211578.0032.0230.2017.03.15 
##                          30235                          23193 
##    228516.0076.0350.2017.03.15    236532.0078.0379.2017.03.15 
##                          39086                          66657 
##    238426.0046.0247.2017.03.15    249768.0083.0359.2017.03.15 
##                          15578                          65769 
##    251073.0025.0221.2017.03.15    285803.0064.0338.2017.03.15 
##                          27143                          63788 
##    293340.0035.0233.2017.03.15    305385.0051.0330.2017.03.15 
##                          24885                          20588 
##    310817.0006.0293.2017.03.15    331904.0098.0430.2017.03.15 
##                          32927                          35529 
##    347964.0061.0336.2017.03.15    350103.0012.0301.2017.03.15 
##                          66932                          34897 
##    380272.0044.0245.2017.03.15    387879.0030.0228.2017.03.15 
##                          55368                          51686 
##    400609.0058.0262.2017.03.15    408044.0052.0331.2017.03.15 
##                          24415                          57931 
##    419034.0081.0382.2017.03.15    432158.0045.0246.2017.03.15 
##                          57230                          37797 
##    442916.0037.0319.2017.03.15    444991.0047.0327.2017.03.15 
##                          24253                          17042 
##    460929.0041.0324.2017.03.15    466105.0018.0377.2017.03.15 
##                          18796                          48350 
##    470588.0066.0340.2017.03.15  481066.boston.0168.2017.03.15 
##                          52619                          14233 
##    487268.0057.0261.2017.03.15    498229.0036.0318.2017.03.15 
##                          17799                          38925 
##    498554.0062.0337.2017.03.15    502743.0038.0320.2017.03.15 
##                          47910                          29154 
##    515591.0056.0334.2017.03.15    516035.0020.0310.2017.03.15 
##                          16026                          41843 
##    521471.0067.0341.2017.03.15    524541.0024.0313.2017.03.15 
##                          37753                          34986 
##    560575.0080.0381.2017.03.15    564855.0053.0332.2017.03.15 
##                          76936                          32030 
##    565723.0005.0292.2017.03.15    588311.0072.0346.2017.03.15 
##                          33565                          61200 
##    596527.0095.0374.2017.03.15    614631.0065.0339.2017.03.15 
##                          49031                          23279 
##    629358.0009.0297.2017.03.15    637837.0021.0312.2017.03.15 
##                          63706                          33770 
##    651433.0099.0431.2017.03.15    658217.0055.0258.2017.03.15 
##                          27060                          34249 
##    711750.0082.0383.2017.03.15    721154.0060.0335.2017.03.15 
##                          83786                          16367 
##    721729.0088.0366.2017.03.15    722188.0063.0268.2017.03.15 
##                          23928                          26981 
##    725896.0022.0218.2017.03.15    732425.0039.0378.2017.03.15 
##                          18124                          48694 
##    735345.0071.0345.2017.03.15    768392.0096.0375.2017.03.15 
##                          46984                          41304 
##    805457.0094.0373.2017.03.15    805641.0015.0305.2017.03.15 
##                          65857                          38997 
##    839338.0011.0300.2017.03.15    848334.0077.0351.2017.03.15 
##                          70395                          45711 
##    885614.0048.0328.2017.03.15    888751.0019.0309.2017.03.15 
##                          18203                          47832 
##    893231.0074.0348.2017.03.15    902901.0029.0226.2017.03.15 
##                          28255                          18740 
##    905350.0085.0362.2017.03.15    908782.0092.0371.2017.03.15 
##                          42388                          28907 
##  909824.boston.0174.2017.03.15    910641.0031.0316.2017.03.15 
##                          12820                          38990 
##    916034.0034.0232.2017.03.15    919901.0026.0222.2017.03.15 
##                          21405                          19714 
##    940622.0075.0349.2017.03.15    959714.0004.0291.2017.03.15 
##                          42914                          30169 
##    959734.0090.0368.2017.03.15    968359.0087.0365.2017.03.15 
##                          37955                          33934 
##    968902.0073.0347.2017.03.15    972684.0028.0224.2017.03.15 
##                          34542                          22469 
##    975240.0016.0306.2017.03.15    976183.0050.0329.2017.03.15 
##                          30867                          31908 
##    979196.0003.0290.2017.03.15    989517.0068.0274.2017.03.15 
##                          34682                          45375 
##   WT09782.0159.0121.2017.02.01   WT15101.0174.0106.2017.02.01 
##                          52918                          17604 
##   WT42336.0160.0122.2017.02.01   WT44778.0158.0120.2017.02.01 
##                          17747                          80361 
##   XE17833.0092.0100.2017.02.01   XE22903.0043.0125.2017.02.01 
##                          10751                          92243 
##   XE28163.0194.0127.2017.02.01   WM26348.0100.0113.2017.01.11 
##                          77437                          52751 
##   WM26354.0139.0315.2017.01.11   WQ64001.0145.0143.2017.01.11 
##                          45678                          39753 
##   WS20813.0155.0153.2017.01.11   WS21384.0094.0102.2017.01.11 
##                          29351                          12873 
##   WS21401.0047.0283.2017.01.11   WS21556.0141.0317.2017.01.11 
##                          65243                          57521 
##   WS21562.0055.0438.2017.01.11   WS21578.0125.0134.2017.01.11 
##                          13616                          46331 
##   WS21584.0066.0321.2017.01.11   WS22205.0097.0110.2017.01.11 
##                          28249                          65967 
##   WS74808.0165.0347.2017.01.11   WS74858.0098.0111.2017.01.11 
##                          32886                          18675 
##   WS75418.0032.0279.2017.01.11   WS76117.0102.0115.2017.01.11 
##                          66488                          78753 
##   WS76840.0117.0126.2017.01.11   WS77050.0026.0276.2017.01.11 
##                          39459                          15102 
##   WT02695.0166.0104.2017.01.11   WT02712.0078.0323.2017.01.11 
##                          20141                          38952 
##   WT05558.0190.0378.2017.01.11   WT05564.0116.0125.2017.01.11 
##                          17973                          33463 
##   WT06263.0127.0135.2017.01.11   WT07439.0058.0440.2017.01.11 
##                          35147                          43263 
##   WT08061.0090.0098.2017.01.11   WT09760.0053.0437.2017.01.11 
##                          28736                          34666 
##   WT09798.0057.0285.2017.01.11   WT10373.0080.0325.2017.01.11 
##                          78499                          37600 
##   WT10389.0036.0270.2017.01.11   WT10395.0035.0269.2017.01.11 
##                          29095                          43853 
##   WT12565.0189.0377.2017.01.11   WT14129.0153.0151.2017.01.11 
##                          26219                          58444 
##   WT14135.0123.0132.2017.01.11   WT14818.0111.0120.2017.01.11 
##                          42345                          33448 
##   WT15084.0046.0282.2017.01.11   WT15117.0157.0155.2017.01.11 
##                         122971                          41809 
##   WT15123.0164.0346.2017.01.11   WT23223.0144.0320.2017.01.11 
##                          37517                          39399 
##   WT23273.0101.0114.2017.01.11   WT24900.0152.0150.2017.01.11 
##                          32170                          25802 
##   WT24916.0044.0281.2017.01.11   WT24944.0124.0159.2017.01.11 
##                          74046                          36504 
##   WT24950.0033.0267.2017.01.11   WT24966.0142.0318.2017.01.11 
##                          54801                          37339 
##   WT27396.0121.0130.2017.01.11   WT27407.0156.0154.2017.01.11 
##                          30349                          27423 
##   WT27441.0129.0137.2017.01.11   WT30335.0134.0140.2017.01.11 
##                          25366                          76057 
##   WT30357.0049.0274.2017.01.11   WT30818.0107.0118.2017.01.11 
##                          35457                          26163 
##   WT30824.0147.0145.2017.01.11   WT30830.0052.0275.2017.01.11 
##                          45066                          41492 
##   WT34353.0120.0129.2017.01.11   WT40033.0133.0139.2017.01.11 
##                          40414                          22514 
##   WT40049.0148.0146.2017.01.11   WT40083.0038.0271.2017.01.11 
##                          60368                          23791 
##   WT40516.0122.0131.2017.01.11   WT41865.0089.0097.2017.01.11 
##                          69537                          24316 
##   WT41910.0095.0108.2017.01.11   WT42069.0154.0152.2017.01.11 
##                          43202                          25172 
##   WT43352.0087.0447.2017.01.11   WT43368.0118.0127.2017.01.11 
##                          15820                          50208 
##   WT43374.0048.0273.2017.01.11   WT44245.0081.0337.2017.01.11 
##                          57836                          54390 
##   WT44601.0082.0445.2017.01.11   WT44762.0130.0138.2017.01.11 
##                          41145                         118478 
##   WT47297.0173.0105.2017.01.11   WT48435.0099.0112.2017.01.11 
##                          30740                          39063 
##   WT48441.0112.0121.2017.01.11   WT48457.0060.0441.2017.01.11 
##                          31526                          41539 
##   WT48491.0146.0144.2017.01.11   WT48502.0106.0117.2017.01.11 
##                          28055                          63937 
##   WT50842.0187.0376.2017.01.11   WT50858.0128.0136.2017.01.11 
##                          20957                          36154 
##   WY74094.0079.0324.2017.01.11   WY74105.0151.0149.2017.01.11 
##                          55072                          67120 
##   WY74777.0073.0444.2017.01.11   WY74799.0176.0369.2017.01.11 
##                          45932                          27435 
##   WY75915.0114.0123.2017.01.11   WY75959.0191.0379.2017.01.11 
##                          27184                          28579 
##   WY75971.0062.0443.2017.01.11   WY76486.0162.0344.2017.01.11 
##                          49071                          52240 
##   WY78078.0161.0343.2017.01.11   WY78084.0061.0442.2017.01.11 
##                          29088                          15433 
##   WY78567.0119.0128.2017.01.11   WY79216.0040.0272.2017.01.11 
##                          21515                          42024 
##   WY79222.0056.0439.2017.01.11   WY79266.0113.0157.2017.01.11 
##                          27261                          55014 
##   WY79272.0177.0370.2017.01.11   WY80257.0027.0277.2017.01.11 
##                          31091                          65700 
##   WY80324.0137.0287.2017.01.11   WY81156.0104.0116.2017.01.11 
##                          37886                          41525 
##   WY81162.0169.0362.2017.01.11   WY81184.0045.0436.2017.01.11 
##                          65619                          23675 
##   XE13926.0025.0214.2017.01.11   XE13948.0192.0380.2017.01.11 
##                          25666                          43145 
##   XE13960.0150.0148.2017.01.11   XE15596.0149.0147.2017.01.11 
##                          34682                          51049 
##   XE17300.0083.0339.2017.01.11   XE21060.0015.0266.2017.01.11 
##                          60567                          39820 
##   XE21076.0182.0372.2017.01.11   XE21082.0186.0375.2017.01.11 
##                          37296                          20236 
##   XE21098.0115.0158.2017.01.11   XE22892.0171.0364.2017.01.11 
##                          42113                          50713 
##   XE22953.0096.0109.2017.01.11   XE27236.0054.0284.2017.01.11 
##                          52070                         135285 
##   XE27414.0059.0286.2017.01.11   XE28202.0028.0278.2017.01.11 
##                          51840                          44456 
##   XE29167.0135.0141.2017.01.11   XE30225.0020.0212.2017.01.11 
##                          64259                          24576 
##   XE31552.0011.0211.2017.01.11   XE33411.0143.0319.2017.01.11 
##                          20444                          38078 
##   XE36257.0175.0156.2017.01.11   XE36952.0091.0099.2017.01.11 
##                          48972                          11715 
##   XE36996.0009.0210.2017.01.11   XE38944.0167.0360.2017.01.11 
##                          38916                          26375 
##   XE38950.0140.0316.2017.01.11   XE38966.0034.0268.2017.01.11 
##                          38755                          55702 
##   XE38972.0178.0371.2017.01.11   XE38988.0110.0119.2017.01.11 
##                          35384                          32939 
##   XE38994.0185.0374.2017.01.11   XE40684.0024.0213.2017.01.11 
##                          21707                          25565 
##   XE40690.0172.0365.2017.01.11   XE40701.0163.0103.2017.01.11 
##                          46970                          46782 
##   XE40717.0010.0265.2017.01.11   XE40745.0006.0264.2017.01.11 
##                          44631                          59807 
##   XE40751.0088.0096.2017.01.11   XE41305.0193.0381.2017.01.11 
##                          17438                          27692 
##   XE41311.0138.0314.2017.01.11   XE41327.0085.0446.2017.01.11 
##                          63493                          13408 
##   XE41333.0136.0142.2017.01.11   XE41349.0168.0361.2017.01.11 
##                          44701                          26835 
##   XE41561.0037.0280.2017.01.11   XE41577.0170.0363.2017.01.11 
##                          44727                          31898 
##   XE41583.0183.0373.2017.01.11   WS19294.0064.0471.2016.11.13 
##                          26328                          58457 
##   WS20829.0126.0349.2016.11.13   WS20835.0007.0390.2016.11.13 
##                         136145                          35250 
##   WS21390.0076.0299.2016.11.13   WS21540.0031.0414.2016.11.13 
##                         144074                          52735 
##   WT02689.0077.0300.2016.11.13   WT02728.0109.0332.2016.11.13 
##                          70581                          83182 
##   WT04693.0016.0246.2016.11.13   WT04704.0071.0294.2016.11.13 
##                          60837                         133384 
##   WT07417.0184.0222.2016.11.13   WT08055.0075.0298.2016.11.13 
##                         122883                         116845 
##   WT09332.0070.0293.2016.11.13   WT09776.0074.0297.2016.11.13 
##                         159041                         156778 
##   WT10406.0003.0386.2016.11.13   WT11111.0023.0406.2016.11.13 
##                          24724                         196412 
##   WT12559.0072.0295.2016.11.13   WT14141.0002.0385.2016.11.13 
##                         192888                          57645 
##   WT15090.0180.0218.2016.11.13   WT23295.0022.0251.2016.11.13 
##                         106854                          68044 
##   WT27518.0050.0457.2016.11.13   WT27607.0039.0422.2016.11.13 
##                          32576                          90830 
##   WT30341.0012.0243.2016.11.13   WT30868.0042.0449.2016.11.13 
##                          55241                          74533 
##   WT30880.0069.0292.2016.11.13   WT34347.0181.0219.2016.11.13 
##                          81481                         103299 
##   WT37711.0068.0291.2016.11.13   WT42192.0131.0354.2016.11.13 
##                         146323                          52000 
##   WT47308.0004.0387.2016.11.13   WY76492.0067.0290.2016.11.13 
##                          54624                         132789 
##   WY78062.0019.0248.2016.11.13   WY79238.0108.0331.2016.11.13 
##                          67007                          37816 
##   WY79244.0105.0328.2016.11.13   WY80318.0103.0326.2016.11.13 
##                          16417                          53131 
##   WY82116.0188.0226.2016.11.13   XE15574.0065.0288.2016.11.13 
##                          79589                         203487 
##   XE17922.0179.0217.2016.11.13   XE17938.0014.0245.2016.11.13 
##                          93388                          67436 
##   XE18326.0084.0307.2016.11.13   XE21109.0018.0247.2016.11.13 
##                          81864                         105606 
##   XE28157.0013.0244.2016.11.13   XE28274.0051.0458.2016.11.13 
##                          94532                          24318 
##   XE29173.0008.0240.2016.11.13   XE29812.0132.0355.2016.11.13 
##                          82688                         143895 
##   XE33372.0021.0427.2016.11.13   XE36174.0063.0470.2016.11.13 
##                          74927                          92285 
##   XE37001.0030.0413.2016.11.13   XE39009.0029.0412.2016.11.13 
##                          80217                          48882 
##   XE39532.0017.0400.2016.11.13   XE39554.0041.0448.2016.11.13 
##                         205766                          65169 
##   XE40723.0001.0384.2016.11.13   XE40739.0086.0309.2016.11.13 
##                          61118                          34672 
##   XE41599.0005.0388.2016.11.13   MBA4060.0077.0266.2016.03.20 
##                          56531                          11517 
##   MBA1007.0092.0281.2016.03.11   MBA1037.0129.0437.2016.03.11 
##                          16176                          14385 
##   MBA1083.0088.0367.2016.03.11   MBA1141.0108.0297.2016.03.11 
##                          11423                          11454 
##   MBA1166.0047.0236.2016.03.11   MBA1172.0017.0444.2016.03.11 
##                          11848                          11809 
##   MBA1261.0125.0433.2016.03.11   MBA1327.0122.0368.2016.03.11 
##                          13925                          20343 
##   MBA1447.0019.0469.2016.03.11   MBA4044.0024.0470.2016.03.11 
##                          45329                          42889 
##   MBA4049.0043.0416.2016.03.11   MBA4051.0001.0446.2016.03.11 
##                          13037                          12351 
##   MBA4056.0119.0308.2016.03.11   MBA4065.0152.0341.2016.03.11 
##                          12105                          12002 
##   MBA4077.0040.0378.2016.03.11   MBA4085.0075.0264.2016.03.11 
##                          27897                          14923 
##   MBA4088.0082.0271.2016.03.11   MBA4091.0085.0429.2016.03.11 
##                           9947                          14136 
##   MBA4120.0076.0265.2016.03.11   MBA4121.0046.0235.2016.03.11 
##                          13922                          12858 
##   MBA4129.0087.0468.2016.03.11   MBA4130.0148.0337.2016.03.11 
##                          40274                          16263 
##   MBA4134.0120.0363.2016.03.11   MBA4139.0164.0353.2016.03.11 
##                          25631                          11278 
##  233202.Boston.0164.2016.02.14  930024.Boston.0114.2016.02.14 
##                          24841                          27023 
##   MBA1003.0131.0320.2016.02.14   MBA1021.0035.0460.2016.02.14 
##                          49506                          21557 
##   MBA1030.0014.0205.2016.02.14   MBA1033.0055.0244.2016.02.14 
##                          60325                          23191 
##   MBA1035.0102.0426.2016.02.14   MBA1041.0053.0242.2016.02.14 
##                          40213                          14230 
##   MBA1052.0009.0451.2016.02.14   MBA1071.0011.0202.2016.02.14 
##                          14859                          82865 
##   MBA1074.0058.0247.2016.02.14   MBA1082.0023.0463.2016.02.14 
##                          13002                          28556 
##   MBA1084.0114.0303.2016.02.14   MBA1090.0003.0194.2016.02.14 
##                          11543                         133259 
##   MBA1095.0090.0279.2016.02.14   MBA1096.0060.0249.2016.02.14 
##                          23430                          19672 
##   MBA1099.0066.0255.2016.02.14   MBA1100.0027.0386.2016.02.14 
##                          19574                          26058 
##   MBA1101.0149.0338.2016.02.14   MBA1103.0026.0385.2016.02.14 
##                          19728                          24858 
##   MBA1111.0139.0328.2016.02.14   MBA1133.0041.0453.2016.02.14 
##                          17707                          23140 
##   MBA1135.0140.0329.2016.02.14   MBA1139.0018.0445.2016.02.14 
##                           9599                          13882 
##   MBA1143.0105.0294.2016.02.14   MBA1151.0107.0296.2016.02.14 
##                          19881                          13159 
##   MBA1159.0165.0354.2016.02.14   MBA1163.0167.0356.2016.02.14 
##                          30857                          33492 
##   MBA1181.0126.0315.2016.02.14   MBA1187.0062.0251.2016.02.14 
##                          43998                          15695 
##   MBA1190.0048.0237.2016.02.14   MBA1193.0132.0321.2016.02.14 
##                          53414                          15292 
##   MBA1199.0069.0258.2016.02.14   MBA1202.0094.0456.2016.02.14 
##                          21096                          14588 
##   MBA1209.0101.0290.2016.02.14   MBA1211.0136.0325.2016.02.14 
##                          10734                          20052 
##   MBA1218.0030.0425.2016.02.14   MBA1230.0093.0282.2016.02.14 
##                          19204                          19109 
##   MBA1237.0170.0359.2016.02.14   MBA1241.0022.0419.2016.02.14 
##                          25519                          30836 
##   MBA1248.0070.0259.2016.02.14   MBA1251.0033.0467.2016.02.14 
##                          16468                          26599 
##   MBA1260.0158.0347.2016.02.14   MBA1267.0038.0227.2016.02.14 
##                          39840                          17977 
##   MBA1270.0042.0231.2016.02.14   MBA1271.0116.0462.2016.02.14 
##                          16729                          22420 
##   MBA1279.0162.0461.2016.02.14   MBA1307.0029.0372.2016.02.14 
##                          48196                          13686 
##   MBA1312.0074.0263.2016.02.14   MBA1317.0067.0256.2016.02.14 
##                          16407                          16233 
##   MBA1330.0050.0239.2016.02.14   MBA1341.0160.0349.2016.02.14 
##                          12379                          21517 
##   MBA1344.0169.0358.2016.02.14   MBA1355.0037.0458.2016.02.14 
##                          38110                          38263 
##   MBA1363.0123.0312.2016.02.14   MBA1365.0171.0360.2016.02.14 
##                          11750                          19399 
##   MBA1370.0034.0465.2016.02.14   MBA1375.0061.0250.2016.02.14 
##                          32525                          24576 
##   MBA1382.0159.0348.2016.02.14   MBA1385.0124.0313.2016.02.14 
##                          23025                          37460 
##   MBA1392.0134.0323.2016.02.14   MBA1399.0147.0336.2016.02.14 
##                          41620                          26657 
##   MBA1410.0130.0379.2016.02.14   MBA1437.0063.0252.2016.02.14 
##                          19712                          13941 
##   MBA1460.0106.0295.2016.02.14   MBA1480.0166.0355.2016.02.14 
##                           9819                          34300 
##   MBA1486.0115.0304.2016.02.14   MBA1488.0072.0452.2016.02.14 
##                          22751                          13626 
##   MBA1511.0163.0352.2016.02.14   MBA1515.0153.0342.2016.02.14 
##                          21490                          24838 
##   MBA4041.0044.0449.2016.02.14   MBA4043.0059.0248.2016.02.14 
##                          10254                          13060 
##   MBA4045.0157.0346.2016.02.14   MBA4047.0012.0203.2016.02.14 
##                          33741                          48792 
##   MBA4050.0028.0434.2016.02.14   MBA4052.0118.0457.2016.02.14 
##                          14519                          35830 
##   MBA4053.0057.0246.2016.02.14   MBA4057.0133.0322.2016.02.14 
##                          25496                           9954 
##   MBA4061.0010.0201.2016.02.14   MBA4062.0079.0393.2016.02.14 
##                         104643                          11876 
##   MBA4063.0100.0289.2016.02.14   MBA4066.0142.0430.2016.02.14 
##                          16644                          12023 
##   MBA4067.0104.0293.2016.02.14   MBA4068.0007.0198.2016.02.14 
##                          22257                          49188 
##   MBA4069.0004.0195.2016.02.14   MBA4070.0086.0478.2016.02.14 
##                          90587                          41192 
##   MBA4072.0161.0350.2016.02.14   MBA4074.0078.0267.2016.02.14 
##                          27345                          13679 
##   MBA4076.0016.0207.2016.02.14   MBA4078.0151.0340.2016.02.14 
##                          47724                          35591 
##   MBA4080.0065.0254.2016.02.14   MBA4081.0052.0241.2016.02.14 
##                          23350                          20586 
##   MBA4082.0032.0221.2016.02.14   MBA4086.0110.0299.2016.02.14 
##                          15687                          18709 
##   MBA4087.0155.0344.2016.02.14   MBA4089.0141.0330.2016.02.14 
##                          35300                          21650 
##   MBA4092.0008.0199.2016.02.14   MBA4095.0002.0193.2016.02.14 
##                          69046                         138863 
##   MBA4096.0145.0334.2016.02.14   MBA4102.0154.0343.2016.02.14 
##                          18861                          23941 
##   MBA4103.0128.0317.2016.02.14   MBA4106.0168.0357.2016.02.14 
##                          13480                          33202 
##   MBA4107.0138.0327.2016.02.14   MBA4108.0099.0288.2016.02.14 
##                          12705                          14653 
##   MBA4109.0073.0262.2016.02.14   MBA4111.0036.0459.2016.02.14 
##                          10720                          38086 
##   MBA4112.0005.0196.2016.02.14   MBA4113.0137.0396.2016.02.14 
##                          59252                          13144 
##   MBA4114.0064.0253.2016.02.14   MBA4115.0143.0332.2016.02.14 
##                          13795                           9242 
##   MBA4118.0031.0466.2016.02.14   MBA4119.0127.0316.2016.02.14 
##                          42048                          13338 
##   MBA4122.0112.0301.2016.02.14   MBA4123.0056.0245.2016.02.14 
##                          28583                          21135 
##   MBA4126.0150.0339.2016.02.14   MBA4127.0021.0417.2016.02.14 
##                           8596                          18287 
##   MBA4131.0006.0197.2016.02.14   MBA4132.0051.0240.2016.02.14 
##                         121717                          12687 
##   MBA4133.0135.0324.2016.02.14   MBA4136.0020.0464.2016.02.14 
##                          18548                          26125 
##   MBA4138.0068.0257.2016.02.14   MBA4140.0015.0206.2016.02.14 
##                          21282                          32717 
##  226855.boston.0016.2015.11.25  229075.boston.0178.2015.11.25 
##                          12316                          21754 
##  447537.boston.0170.2015.11.25  503564.boston.0169.2015.11.25 
##                          11055                          16113 
##  561130.boston.0065.2015.11.25  588800.boston.0068.2015.11.25 
##                          64533                          54160 
##  629356.boston.0076.2015.11.25  765828.boston.0089.2015.11.25 
##                          17564                          18760 
##  772512.boston.0090.2015.11.25  826391.boston.0177.2015.11.25 
##                          28599                          29717 
##  849016.boston.0176.2015.11.25  872569.boston.0179.2015.11.25 
##                          27270                          22343 
##     136109.048.0334.2018.12.15     137787.006.0293.2018.12.15 
##                         142042                         110949 
##     186400.027.0314.2018.12.15     207722.029.0316.2018.12.15 
##                         130465                         140162 
##     221355.060.0345.2018.12.15     225794.001.0288.2018.12.15 
##                         131800                         169725 
##     235185.033.0320.2018.12.15     236512.015.0302.2018.12.15 
##                         146986                         136583 
##     240884.013.0300.2018.12.15     241367.042.0329.2018.12.15 
##                         134087                         130533 
##     243736.036.0323.2018.12.15     276693.051.0337.2018.12.15 
##                         125215                         143542 
##     282036.026.0313.2018.12.15     298053.032.0319.2018.12.15 
##                         140377                         139377 
##     319820.035.0322.2018.12.15     354109.012.0299.2018.12.15 
##                         156031                         130651 
##     356403.053.0339.2018.12.15     364485.002.0289.2018.12.15 
##                         163928                         138715 
##     373167.021.0308.2018.12.15     395379.010.0297.2018.12.15 
##                          91803                         125471 
##     402235.052.0338.2018.12.15     411794.028.0315.2018.12.15 
##                         103407                         125418 
##     416151.055.0340.2018.12.15     420641.004.0291.2018.12.15 
##                         147234                         137640 
##     423443.005.0292.2018.12.15     445474.025.0312.2018.12.15 
##                         153787                         162503 
##     453551.040.0327.2018.12.15     459452.044.0331.2018.12.15 
##                         149435                         105418 
##     467375.007.0294.2018.12.15     471179.003.0290.2018.12.15 
##                         148513                         139922 
##     492275.058.0343.2018.12.15     570298.041.0328.2018.12.15 
##                         165014                         135343 
##     574541.045.0332.2018.12.15     576662.030.0317.2018.12.15 
##                         124317                         151416 
##     578598.017.0304.2018.12.15     580176.038.0325.2018.12.15 
##                         121232                         141927 
##     588443.031.0318.2018.12.15     588873.064.0349.2018.12.15 
##                          94483                         109394 
##     612872.019.0306.2018.12.15     667045.043.0330.2018.12.15 
##                         139365                         116480 
##     675294.009.0296.2018.12.15     684908.056.0341.2018.12.15 
##                         138860                         122542 
##     714983.057.0342.2018.12.15     720750.034.0321.2018.12.15 
##                         145724                         121774 
##     774051.062.0347.2018.12.15     821034.039.0326.2018.12.15 
##                         147680                         119747 
##     822655.037.0324.2018.12.15     823946.047.0333.2018.12.15 
##                          93713                         121833 
##     850240.022.0309.2018.12.15     851204.014.0301.2018.12.15 
##                         107960                         145594 
##     853781.063.0348.2018.12.15     857829.018.0305.2018.12.15 
##                         130937                         136894 
##     883092.049.0335.2018.12.15     909074.024.0311.2018.12.15 
##                         126602                          83194 
##     918320.061.0346.2018.12.15     938150.059.0344.2018.12.15 
##                         150659                         159642 
##     945496.008.0295.2018.12.15     948983.050.0336.2018.12.15 
##                         130406                         160584 
##     982757.020.0307.2018.12.15     985170.011.0298.2018.12.15 
##                         135822                         132462 
##    MBA1008.062.0371.2018.12.15    MBA1073.103.0380.2018.12.15 
##                          97776                         121454 
##    MBA1144.144.0244.2018.12.15    MBA1215.004.0359.2018.12.15 
##                          58436                         141749 
##    MBA1226.010.0360.2018.12.15    MBA1240.156.0246.2018.12.15 
##                         118025                         123306 
##    MBA1284.025.0362.2018.12.15    MBA1302.089.0375.2018.12.15 
##                         106064                         122557 
##    MBA1315.035.0365.2018.12.15    MBA1398.080.0374.2018.12.15 
##                         122382                         124340 
##    MBA1491.070.0372.2018.12.15    MBA1509.039.0366.2018.12.15 
##                         141649                         123099 
##    MBA1519.096.0377.2018.12.15    MBA4042.011.0361.2018.12.15 
##                         105745                         135959 
##    MBA4046.032.0363.2018.12.15    MBA4048.146.0245.2018.12.15 
##                         121271                         104141 
##    MBA4058.049.0368.2018.12.15    MBA4059.113.0242.2018.12.15 
##                         163357                         154949 
##    MBA4064.117.0243.2018.12.15    MBA4071.098.0379.2018.12.15 
##                          99265                         173599 
##    MBA4083.097.0378.2018.12.15    MBA4093.033.0364.2018.12.15 
##                         131209                         131778 
##    MBA4099.045.0367.2018.12.15    MBA4104.091.0376.2018.12.15 
##                         140969                         128881 
##    MBA4128.059.0369.2018.12.15 
##                         142304
```

```
min(rowSums(OTU_table))
```

```
## [1] 8596
```

```
Total_counts<-as.data.frame(rowSums(OTU_table))
colnames(Total_counts)<-c("Counts")
ggplot(Total_counts, aes(x=Counts))+geom_histogram(binwidth=100)+geom_vline(aes(xintercept=mean(Counts, na.rm=T)), color="red", linetype="dashed", size=1)+theme_bw()+
  ggtitle("Total count per sample distribution")+theme(plot.title=element_text(lineheight=10, size=15))+
  xlab("Counts")+ylab("Number of samples")+theme(axis.text=element_text(size=15), axis.title=element_text(size=15))
```

```
#Remove all those samples that do not reach a minimum threshold of number of reads:
counts<-as.data.frame(rowSums(OTU_table))
colnames(counts)<-"counts"
counts$Sample<-row.names(counts)
subset_8000<-counts[counts$counts>=8000,]
subset_8000$Sample<-NULL
OTU_table<-OTU_table[row.names(OTU_table)%in%as.vector(row.names(subset_8000)),]

#Rarefy to same sequencing depth:
set.seed(1)
OTU_table_8000<-rrarefy(OTU_table, 8000)
```

```
## Warning in rrarefy(OTU_table, 8000): function should be used for observed
## counts, but smallest count is 2
```

```
#Estimate richness:
richness_8000<-estimateR(OTU_table_8000)
richness_8000<-t(richness_8000)
richness_8000<-as.data.frame(richness_8000)

#Estimate evenness:
shannon<-diversityresult(x=OTU_table_8000, method="each site", index="Shannon")
diversity_8000<-cbind(shannon)

#Join data from richness and evenness calculations:
ecology_8000<-cbind(richness_8000[1], diversity_8000)
colnames(ecology_8000)<-c("Observed", "Shannon")

#Subset metadata
metadata<-metadata[row.names(metadata)%in%row.names(ecology_8000),,drop=FALSE]

all.equal(row.names(metadata),row.names(ecology_8000))
```

```
## [1] TRUE
```

```
ecology_8000$SampleID<-metadata$SampleID
ecology_8000$Cohort<-metadata$sample_cohort
ecology_8000$hiv_phenotype<-metadata$hiv_phenotype
ecology_8000$sexual_orientation<-metadata$sexual_orientation

#Subset indivudals for this comparison
ecology_8000_nomsm<-ecology_8000[ecology_8000$sexual_orientation!="MSM" & ecology_8000$Cohort=="boston",,drop=FALSE]
ecology_8000_nomsm_melt<-melt(ecology_8000_nomsm)
```

```
## Using SampleID, Cohort, hiv_phenotype, sexual_orientation as id variables
```

```
ecology_8000_msm<-ecology_8000[ecology_8000$sexual_orientation=="MSM" & ecology_8000$Cohort=="boston",,drop=FALSE]
ecology_8000_msm_melt<-melt(ecology_8000_msm)
```

```
## Using SampleID, Cohort, hiv_phenotype, sexual_orientation as id variables
```

```
plot_noMSM<-ggplot(data=ecology_8000_nomsm_melt, aes(x=hiv_phenotype,y=value))+geom_boxplot(aes(alpha=hiv_phenotype), outlier.color="white", fill="royalblue4")+theme_bw()+
  geom_point(aes(alpha=hiv_phenotype), color="royalblue4", position=position_jitterdodge(jitter.width=0.25), size=1)+
  facet_wrap(~variable, scales="free_y", nrow=1)+scale_alpha_manual(values=c(0.9, 0.6, 0.3))+
  theme(axis.text.x = element_text(angle=90))+ggtitle("noMSM")

plot_MSM<-ggplot(data=ecology_8000_msm_melt, aes(x=hiv_phenotype,y=value))+geom_boxplot(aes(alpha=hiv_phenotype), outlier.color="white", fill="royalblue4")+theme_bw()+
  geom_point(aes(alpha=hiv_phenotype), color="royalblue4", position=position_jitterdodge(jitter.width=0.25), size=1)+
  facet_wrap(~variable, scales="free_y", nrow=1)+scale_alpha_manual(values=c(0.9, 0.6, 0.3))+
  theme(axis.text.x = element_text(angle=90))+ggtitle("MSM")

ggsave("Figure3B.pdf", grid.arrange(plot_noMSM, plot_MSM, ncol=2, nrow=2), width=15, height=10)
```

```
#Statistical test
for (i in colnames(ecology_8000_nomsm)[1:2]){
  print(i)
  print(kruskal(ecology_8000_nomsm[i], ecology_8000_nomsm["hiv_phenotype"],group=F,p.adj = "bonferroni"))
}
```

```
## [1] "Observed"
## $statistics
##        Chisq Df    p.chisq
##   0.51695815  2 0.77222519
## 
## $parameters
##             test  p.ajusted                              name.t ntr alpha
##   Kruskal-Wallis bonferroni ecology_8000_nomsm["hiv_phenotype"]   3  0.05
## 
## $means
##                ecology_8000_nomsm.i.      rank       std  r Min Max   Q25 Q50
## 1_hiv_negative             138.62353 56.711765 48.321093 85  61 249  96.0 137
## 2_suppressed               146.84211 60.842105 52.775482 19  79 300 112.0 130
## 4_unsuppressed             154.72727 63.045455 76.950752 11  46 333 116.5 146
##                  Q75
## 1_hiv_negative 175.0
## 2_suppressed   177.0
## 4_unsuppressed 186.5
## 
## $comparison
##                                 Difference pvalue Signif.        LCL       UCL
## 1_hiv_negative - 2_suppressed   -4.1303406      1         -24.827767 16.567086
## 1_hiv_negative - 4_unsuppressed -6.3336898      1         -32.468284 19.800904
## 2_suppressed - 4_unsuppressed   -2.2033493      1         -33.104413 28.697715
## 
## $groups
## NULL
## 
## attr(,"class")
## [1] "group"
## [1] "Shannon"
## $statistics
##        Chisq Df    p.chisq
##   0.28118102  2 0.86884502
## 
## $parameters
##             test  p.ajusted                              name.t ntr alpha
##   Kruskal-Wallis bonferroni ecology_8000_nomsm["hiv_phenotype"]   3  0.05
## 
## $means
##                ecology_8000_nomsm.i.      rank        std  r       Min
## 1_hiv_negative             3.4220815 57.035294 0.68648328 85 1.4674836
## 2_suppressed               3.5331089 61.157895 0.62121210 19 1.7319634
## 4_unsuppressed             3.4551810 60.000000 0.78369881 11 1.5978440
##                      Max       Q25       Q50       Q75
## 1_hiv_negative 4.4513155 3.0708710 3.5533268 3.9279747
## 2_suppressed   4.5236775 3.1442770 3.7108917 3.9567380
## 4_unsuppressed 4.3767472 3.2269193 3.5800493 3.9414740
## 
## $comparison
##                                 Difference pvalue Signif.        LCL       UCL
## 1_hiv_negative - 2_suppressed   -4.1226006      1         -24.843806 16.598605
## 1_hiv_negative - 4_unsuppressed -2.9647059      1         -29.129326 23.199914
## 2_suppressed - 4_unsuppressed    1.1578947      1         -29.778671 32.094461
## 
## $groups
## NULL
## 
## attr(,"class")
## [1] "group"
```

```
for (i in colnames(ecology_8000_msm)[1:2]){
  print(i)
  print(kruskal(ecology_8000_msm[i], ecology_8000_msm["hiv_phenotype"],group=F,p.adj = "bonferroni"))
}
```

```
## [1] "Observed"
## $statistics
##       Chisq Df       p.chisq
##   24.227218  2 5.4843659e-06
## 
## $parameters
##             test  p.ajusted                            name.t ntr alpha
##   Kruskal-Wallis bonferroni ecology_8000_msm["hiv_phenotype"]   3  0.05
## 
## $means
##                ecology_8000_msm.i.      rank       std  r Min Max    Q25   Q50
## 1_hiv_negative           209.87500 84.500000 73.519254 32  73 332 143.25 223.5
## 2_suppressed             127.97619 46.880952 41.464936 42  56 241 100.25 123.5
## 4_unsuppressed           140.13636 53.363636 52.645630 44  38 268 101.25 131.0
##                   Q75
## 1_hiv_negative 260.75
## 2_suppressed   149.50
## 4_unsuppressed 175.75
## 
## $comparison
##                                 Difference pvalue Signif.        LCL        UCL
## 1_hiv_negative - 2_suppressed    37.619048 0.0000     ***  20.105221 55.1328740
## 1_hiv_negative - 4_unsuppressed  31.136364 0.0001     ***  13.795518 48.4772097
## 2_suppressed - 4_unsuppressed    -6.482684 0.9901         -22.584062  9.6186944
## 
## $groups
## NULL
## 
## attr(,"class")
## [1] "group"
## [1] "Shannon"
## $statistics
##       Chisq Df       p.chisq
##   17.723352  2 0.00014171734
## 
## $parameters
##             test  p.ajusted                            name.t ntr alpha
##   Kruskal-Wallis bonferroni ecology_8000_msm["hiv_phenotype"]   3  0.05
## 
## $means
##                ecology_8000_msm.i.      rank        std  r       Min       Max
## 1_hiv_negative           3.8995527 79.156250 0.48983812 32 2.7533384 4.7212715
## 2_suppressed             3.2014241 45.404762 0.73980469 42 1.7331647 4.4821202
## 4_unsuppressed           3.4962105 58.659091 0.64920663 44 1.8739013 4.7411970
##                      Q25       Q50       Q75
## 1_hiv_negative 3.4553813 4.0302095 4.3146287
## 2_suppressed   2.7331945 3.2746910 3.6359910
## 4_unsuppressed 3.1059814 3.6027204 3.8471969
## 
## $comparison
##                                 Difference pvalue Signif.         LCL
## 1_hiv_negative - 2_suppressed    33.751488 0.0000     ***  15.6325655
## 1_hiv_negative - 4_unsuppressed  20.497159 0.0193       *   2.5571932
## 2_suppressed - 4_unsuppressed   -13.254329 0.1670         -29.9120041
##                                        UCL
## 1_hiv_negative - 2_suppressed   51.8704107
## 1_hiv_negative - 4_unsuppressed 38.4371250
## 2_suppressed - 4_unsuppressed    3.4033461
## 
## $groups
## NULL
## 
## attr(,"class")
## [1] "group"
```

```
#--------------------------------------------------------------------------------------------------------------
```
