# Supplementary material for: HIV-associated gut microbial alterations are dependent on host and geographic context
Source: Nat Commun. 2024 Feb 5;15:1055. doi: 10.1038/s41467-023-44566-4 (PMC10844288; doi:10.1038/s41467-023-44566-4)
Supplement: Supplementary file 11 — Figure4ANCOM_Rocafort-Gootenberg_2023_04_05 [file 41467_2023_44566_MOESM11_ESM.html]

Rocafort-Gootenberg\_Figure4ANCOM


# Rocafort-Gootenberg\_Figure4ANCOM

#Load needed R packages

```
library("phyloseq")
library("tidyverse")
```

```
## ── Attaching packages ─────────────────────────────────────── tidyverse 1.3.2 ──
## ✔ ggplot2 3.4.1     ✔ purrr   1.0.1
## ✔ tibble  3.1.8     ✔ dplyr   1.1.0
## ✔ tidyr   1.3.0     ✔ stringr 1.5.0
## ✔ readr   2.1.4     ✔ forcats 1.0.0
## ── Conflicts ────────────────────────────────────────── tidyverse_conflicts() ──
## ✖ dplyr::filter() masks stats::filter()
## ✖ dplyr::lag()    masks stats::lag()
```

```
library("ggplot2")
library("gridExtra")
```

```
## 
## Attaching package: 'gridExtra'
## 
## The following object is masked from 'package:dplyr':
## 
##     combine
```

```
library("dplyr")
library("vegan")
```

```
## Loading required package: permute
## Loading required package: lattice
## This is vegan 2.6-4
```

```
library("knitr") 
library("reshape")
```

```
## 
## Attaching package: 'reshape'
## 
## The following object is masked from 'package:dplyr':
## 
##     rename
## 
## The following objects are masked from 'package:tidyr':
## 
##     expand, smiths
```

```
library("DESeq2")
```

```
## Loading required package: S4Vectors
## Loading required package: stats4
## Loading required package: BiocGenerics
## 
## Attaching package: 'BiocGenerics'
## 
## The following object is masked from 'package:gridExtra':
## 
##     combine
## 
## The following objects are masked from 'package:dplyr':
## 
##     combine, intersect, setdiff, union
## 
## The following objects are masked from 'package:stats':
## 
##     IQR, mad, sd, var, xtabs
## 
## The following objects are masked from 'package:base':
## 
##     anyDuplicated, aperm, append, as.data.frame, basename, cbind,
##     colnames, dirname, do.call, duplicated, eval, evalq, Filter, Find,
##     get, grep, grepl, intersect, is.unsorted, lapply, Map, mapply,
##     match, mget, order, paste, pmax, pmax.int, pmin, pmin.int,
##     Position, rank, rbind, Reduce, rownames, sapply, setdiff, sort,
##     table, tapply, union, unique, unsplit, which.max, which.min
## 
## 
## Attaching package: 'S4Vectors'
## 
## The following objects are masked from 'package:reshape':
## 
##     expand, rename
## 
## The following objects are masked from 'package:dplyr':
## 
##     first, rename
## 
## The following object is masked from 'package:tidyr':
## 
##     expand
## 
## The following objects are masked from 'package:base':
## 
##     expand.grid, I, unname
## 
## Loading required package: IRanges
## 
## Attaching package: 'IRanges'
## 
## The following objects are masked from 'package:dplyr':
## 
##     collapse, desc, slice
## 
## The following object is masked from 'package:purrr':
## 
##     reduce
## 
## The following object is masked from 'package:phyloseq':
## 
##     distance
## 
## Loading required package: GenomicRanges
## Loading required package: GenomeInfoDb
## Loading required package: SummarizedExperiment
## Loading required package: MatrixGenerics
## Loading required package: matrixStats
## 
## Attaching package: 'matrixStats'
## 
## The following object is masked from 'package:dplyr':
## 
##     count
## 
## 
## Attaching package: 'MatrixGenerics'
## 
## The following objects are masked from 'package:matrixStats':
## 
##     colAlls, colAnyNAs, colAnys, colAvgsPerRowSet, colCollapse,
##     colCounts, colCummaxs, colCummins, colCumprods, colCumsums,
##     colDiffs, colIQRDiffs, colIQRs, colLogSumExps, colMadDiffs,
##     colMads, colMaxs, colMeans2, colMedians, colMins, colOrderStats,
##     colProds, colQuantiles, colRanges, colRanks, colSdDiffs, colSds,
##     colSums2, colTabulates, colVarDiffs, colVars, colWeightedMads,
##     colWeightedMeans, colWeightedMedians, colWeightedSds,
##     colWeightedVars, rowAlls, rowAnyNAs, rowAnys, rowAvgsPerColSet,
##     rowCollapse, rowCounts, rowCummaxs, rowCummins, rowCumprods,
##     rowCumsums, rowDiffs, rowIQRDiffs, rowIQRs, rowLogSumExps,
##     rowMadDiffs, rowMads, rowMaxs, rowMeans2, rowMedians, rowMins,
##     rowOrderStats, rowProds, rowQuantiles, rowRanges, rowRanks,
##     rowSdDiffs, rowSds, rowSums2, rowTabulates, rowVarDiffs, rowVars,
##     rowWeightedMads, rowWeightedMeans, rowWeightedMedians,
##     rowWeightedSds, rowWeightedVars
## 
## Loading required package: Biobase
## Welcome to Bioconductor
## 
##     Vignettes contain introductory material; view with
##     'browseVignettes()'. To cite Bioconductor, see
##     'citation("Biobase")', and for packages 'citation("pkgname")'.
## 
## 
## Attaching package: 'Biobase'
## 
## The following object is masked from 'package:MatrixGenerics':
## 
##     rowMedians
## 
## The following objects are masked from 'package:matrixStats':
## 
##     anyMissing, rowMedians
## 
## The following object is masked from 'package:phyloseq':
## 
##     sampleNames
```

```
library("BiodiversityR")
```

```
## Loading required package: tcltk
## BiodiversityR 2.15-1: Use command BiodiversityRGUI() to launch the Graphical User Interface; 
## to see changes use BiodiversityRGUI(changeLog=TRUE, backward.compatibility.messages=TRUE)
```

```
library("usedist") 
library("gplots")
```

```
## 
## Attaching package: 'gplots'
## 
## The following object is masked from 'package:IRanges':
## 
##     space
## 
## The following object is masked from 'package:S4Vectors':
## 
##     space
## 
## The following object is masked from 'package:stats':
## 
##     lowess
```

```
library("agricolae")
```

```
## Registered S3 methods overwritten by 'klaR':
##   method      from 
##   predict.rda vegan
##   print.rda   vegan
##   plot.rda    vegan
```

```
library("BioVenn")
```

#Load original phyloseq oject output from DADA2 pipeline

```
ps_gg_fp_f_prevalence_filter_2019_05_26 <- readRDS("ps_gg_fp_f_prevalence_filter_2019_05_26")
readr::read_csv(
  "Metadata_formatted_nat_comm_add_2021_10_24.csv",
  col_names = TRUE,
  col_types = NULL,
  col_select = NULL,
  id = NULL,
  locale = default_locale(),
  na = c("", "NA", "empty", "EMPTY"),
  quote = "\"",
  comment = "",
  trim_ws = TRUE,
  skip = 0,
  name_repair = "unique",
  num_threads = readr_threads(),
  progress = show_progress(),
  show_col_types = should_show_types(),
  skip_empty_rows = TRUE,
  lazy = TRUE
) -> new_metadata
```

```
## Rows: 597 Columns: 88
## ── Column specification ────────────────────────────────────────────────────────
## Delimiter: ","
## chr (26): X, SampleID, subject_id, Race, Ethnicity, unique_id, sequencing_da...
## dbl (62): primer_used, read_count, age, height_cm, height_in, weight_kg, wei...
## 
## ℹ Use `spec()` to retrieve the full column specification for this data.
## ℹ Specify the column types or set `show_col_types = FALSE` to quiet this message.
```

```
### add {SampleID} as rownames
new_metadata_as_sample_data <- phyloseq::sample_data(new_metadata)
phyloseq::sample_names(new_metadata_as_sample_data) <- dplyr::pull(new_metadata, 1)
phyloseq::sample_data(ps_gg_fp_f_prevalence_filter_2019_05_26) <- new_metadata_as_sample_data

#Fix randomness
set.seed(1)
```

#Figure 4A

```
#FIGURE 4A
#--------------------------------------------------------------------------------------------------------------
metadata<-as.data.frame(as.matrix(sample_data(ps_gg_fp_f_prevalence_filter_2019_05_26)))
table(metadata$sample_cohort)
```

```
## 
##   boston botswana uganda_2 
##      233      194      170
```

```
metadata<-metadata[metadata$sexual_orientation != "MSM" | is.na(metadata$sexual_orientation), , drop=F]
metadata<-metadata[,colnames(metadata)%in%c("hiv_phenotype", "sample_cohort", "SampleID", "scd14_ng_ml_uvm", "ifabp_pg_ml_uvm"),drop=F]
metadata$scd14_ng_ml_uvm<-as.numeric(as.character(metadata$scd14_ng_ml_uvm))
metadata$ifabp_pg_ml_uvm<-as.numeric(as.character(metadata$ifabp_pg_ml_uvm))
table(metadata$sample_cohort)
```

```
## 
##   boston botswana uganda_2 
##      115      194      170
```

```
#Let's prepare a dataframe per sample_cohort
us<-metadata[metadata$sample_cohort == "boston",,drop=F]
botswana<-metadata[metadata$sample_cohort == "botswana",,drop=F]
uganda<-metadata[metadata$sample_cohort == "uganda_2",,drop=F]

#Let's prepare for plotting
us_melt<-melt(us)
```

```
## Using SampleID, hiv_phenotype, sample_cohort as id variables
```

```
plot_us<-ggplot(data=us_melt, aes(x=hiv_phenotype, y=log10(value)))+geom_boxplot(aes(alpha=hiv_phenotype), fill="royalblue4", outlier.color="white")+
  facet_wrap(~variable,nrow=3)+theme_bw()+scale_alpha_manual(values=c(0.9, 0.6, 0.3))+
  geom_point(aes(alpha=hiv_phenotype), color="royalblue4", position=position_jitterdodge(jitter.width=0.25), size=1)+
  theme(axis.text.x=element_blank())

botswana_melt<-melt(botswana)
```

```
## Using SampleID, hiv_phenotype, sample_cohort as id variables
```

```
plot_botswana<-ggplot(data=botswana_melt, aes(x=hiv_phenotype, y=log10(value)))+geom_boxplot(aes(alpha=hiv_phenotype), fill="darkorange", outlier.color="white")+
  facet_wrap(~variable,nrow=3)+theme_bw()+scale_alpha_manual(values=c(0.9, 0.6, 0.3))+
  geom_point(aes(alpha=hiv_phenotype), color="darkorange", position=position_jitterdodge(jitter.width=0.25), size=1)+
  theme(axis.text.x=element_blank())

uganda_melt<-melt(uganda)
```

```
## Using SampleID, hiv_phenotype, sample_cohort as id variables
```

```
plot_uganda<-ggplot(data=uganda_melt, aes(x=hiv_phenotype, y=log10(value)))+geom_boxplot(aes(alpha=hiv_phenotype), fill="darkgreen", outlier.color="white")+
  facet_wrap(~variable,nrow=3)+theme_bw()+scale_alpha_manual(values=c(0.9, 0.6, 0.3))+
  geom_point(aes(alpha=hiv_phenotype), color="darkgreen", position=position_jitterdodge(jitter.width=0.25), size=1)+
  theme(axis.text.x=element_blank())

ggsave("Figure4A_ANCOM_v1.pdf", grid.arrange(plot_us, plot_botswana, plot_uganda, ncol=3), width=15, height=10)
```

```
## Warning: Removed 4 rows containing non-finite values (`stat_boxplot()`).
```

```
## Warning: Removed 4 rows containing missing values (`geom_point()`).
```

```
## Warning: Removed 64 rows containing non-finite values (`stat_boxplot()`).
```

```
## Warning: Removed 64 rows containing missing values (`geom_point()`).
```

```
## Warning: Removed 18 rows containing non-finite values (`stat_boxplot()`).
```

```
## Warning: Removed 18 rows containing missing values (`geom_point()`).
```

```
#Let's do the statistics:
print("US")
```

```
## [1] "US"
```

```
for (i in c("scd14_ng_ml_uvm","ifabp_pg_ml_uvm")){
  print(i)
  print(kruskal(us[[i]],us[["hiv_phenotype"]],group=F))
}
```

```
## [1] "scd14_ng_ml_uvm"
## $statistics
##      Chisq Df      p.chisq
##   14.36612  2 0.0007593418
## 
## $parameters
##             test p.ajusted                name.t ntr alpha
##   Kruskal-Wallis      none us[["hiv_phenotype"]]   3  0.05
## 
## $means
##                 us..i..     rank      std  r      Min      Max      Q25
## 1_hiv_negative 1194.483 50.18072 310.4782 83  639.424 1899.575 1009.565
## 2_suppressed   1401.172 71.73684 306.8136 19  901.777 2013.367 1155.404
## 4_unsuppressed 1497.183 83.00000 255.2225 11 1151.088 1991.145 1327.372
##                     Q50      Q75
## 1_hiv_negative 1120.874 1363.760
## 2_suppressed   1392.933 1578.613
## 4_unsuppressed 1468.130 1634.402
## 
## $comparison
##                                 Difference pvalue Signif.       LCL        UCL
## 1_hiv_negative - 2_suppressed    -21.55612 0.0071      ** -37.11368  -5.998562
## 1_hiv_negative - 4_unsuppressed  -32.81928 0.0012      ** -52.44771 -13.190840
## 2_suppressed - 4_unsuppressed    -11.26316 0.3376         -34.43949  11.913172
## 
## $groups
## NULL
## 
## attr(,"class")
## [1] "group"
## [1] "ifabp_pg_ml_uvm"
## $statistics
##      Chisq Df      p.chisq
##   21.40706  2 2.246546e-05
## 
## $parameters
##             test p.ajusted                name.t ntr alpha
##   Kruskal-Wallis      none us[["hiv_phenotype"]]   3  0.05
## 
## $means
##                 us..i..     rank       std  r     Min     Max      Q25      Q50
## 1_hiv_negative 1156.096 48.65060  792.3421 83 375.677 5748.72  695.867  929.182
## 2_suppressed   1715.127 75.31579  791.7103 19 695.313 3058.39 1039.075 1538.134
## 4_unsuppressed 3135.097 88.36364 2662.8295 11 958.458 8481.13 1420.111 1991.881
##                     Q75
## 1_hiv_negative 1392.254
## 2_suppressed   2396.276
## 4_unsuppressed 3867.836
## 
## $comparison
##                                 Difference pvalue Signif.       LCL        UCL
## 1_hiv_negative - 2_suppressed    -26.66519 0.0006     *** -41.65128 -11.679098
## 1_hiv_negative - 4_unsuppressed  -39.71303 0.0001     *** -58.62047 -20.805599
## 2_suppressed - 4_unsuppressed    -13.04785 0.2493         -35.37285   9.277157
## 
## $groups
## NULL
## 
## attr(,"class")
## [1] "group"
```

```
print("Botswana")
```

```
## [1] "Botswana"
```

```
for (i in c("scd14_ng_ml_uvm","ifabp_pg_ml_uvm")){
  print(i)
  print(kruskal(botswana[[i]],botswana[["hiv_phenotype"]],group=F))
}
```

```
## [1] "scd14_ng_ml_uvm"
## $statistics
##      Chisq Df      p.chisq
##   52.45356  2 4.072409e-12
## 
## $parameters
##             test p.ajusted                      name.t ntr alpha
##   Kruskal-Wallis      none botswana[["hiv_phenotype"]]   3  0.05
## 
## $means
##                botswana..i..     rank      std  r     Min      Max       Q25
## 1_hiv_negative      1174.199  46.7250 400.6804 60 564.048 3200.000  957.3195
## 2_suppressed        1625.941 102.9254 407.9902 67 582.338 2857.865 1373.8330
## 4_unsuppressed      1643.809 100.1000 506.1090 35 925.374 3200.000 1372.2420
##                     Q50      Q75
## 1_hiv_negative 1112.646 1305.509
## 2_suppressed   1582.146 1813.456
## 4_unsuppressed 1571.896 1675.753
## 
## $comparison
##                                 Difference pvalue Signif.       LCL       UCL
## 1_hiv_negative - 2_suppressed   -56.200373 0.0000     *** -69.80613 -42.59462
## 1_hiv_negative - 4_unsuppressed -53.375000 0.0000     *** -69.65618 -37.09382
## 2_suppressed - 4_unsuppressed     2.825373 0.7272         -13.13939  18.79013
## 
## $groups
## NULL
## 
## attr(,"class")
## [1] "group"
## [1] "ifabp_pg_ml_uvm"
## $statistics
##      Chisq Df     p.chisq
##   35.22398  2 2.24497e-08
## 
## $parameters
##             test p.ajusted                      name.t ntr alpha
##   Kruskal-Wallis      none botswana[["hiv_phenotype"]]   3  0.05
## 
## $means
##                botswana..i..     rank       std  r     Min      Max      Q25
## 1_hiv_negative      1334.551  55.1000  793.4213 60 270.216 3855.908  791.647
## 2_suppressed        3109.232 104.5672 2313.7557 67 560.614 9466.078 1444.849
## 4_unsuppressed      1966.431  82.6000 1261.6810 35 441.693 6299.814 1270.408
##                     Q50      Q75
## 1_hiv_negative 1102.502 1575.731
## 2_suppressed   2336.995 3823.148
## 4_unsuppressed 1555.080 2527.746
## 
## $comparison
##                                 Difference pvalue Signif.        LCL        UCL
## 1_hiv_negative - 2_suppressed    -49.46716 0.0000     *** -64.113057 -34.821271
## 1_hiv_negative - 4_unsuppressed  -27.50000 0.0023      ** -45.025853  -9.974147
## 2_suppressed - 4_unsuppressed     21.96716 0.0126       *   4.781921  39.152407
## 
## $groups
## NULL
## 
## attr(,"class")
## [1] "group"
```

```
print("Uganda")
```

```
## [1] "Uganda"
```

```
for (i in c("scd14_ng_ml_uvm","ifabp_pg_ml_uvm")){
  print(i)
  print(wilcox.test(uganda[[i]]~uganda[["hiv_phenotype"]]))
}
```

```
## [1] "scd14_ng_ml_uvm"
## 
##  Wilcoxon rank sum test with continuity correction
## 
## data:  uganda[[i]] by uganda[["hiv_phenotype"]]
## W = 2040, p-value = 5.967e-05
## alternative hypothesis: true location shift is not equal to 0
## 
## [1] "ifabp_pg_ml_uvm"
## 
##  Wilcoxon rank sum test with continuity correction
## 
## data:  uganda[[i]] by uganda[["hiv_phenotype"]]
## W = 2758, p-value = 0.1139
## alternative hypothesis: true location shift is not equal to 0
```

```
#--------------------------------------------------------------------------------------------------------------
```

#Figure 4B

```
#FIGURE 4B
#--------------------------------------------------------------------------------------------------------------
us <- read.csv("ANCOM_US_NEG_ART_Filtered.csv", header = TRUE)
us <- dplyr::rename(us, log2FoldChange = lfc)
us <- us[order(us$log2FoldChange),,drop = F]
botswana <- read.csv("ANCOM_BOTS_NEG_ART_Filtered.csv", header = TRUE)
botswana <- dplyr::rename(botswana, log2FoldChange = lfc)
botswana <- botswana[order(botswana$log2FoldChange), , drop = F]
uganda <- read.csv("ANCOM_UGANDA_NEG_ART_Filtered.csv", header = TRUE)
uganda <- dplyr::rename(uganda, log2FoldChange = lfc)
uganda <- uganda[order(uganda$log2FoldChange), , drop = F]

sigtab_dataset <- rbind(us, botswana, uganda)

#Calculate ASV's relative abundance
ps_gg_fp_f_prevalence_filter_2019_05_26_proportion <- transform_sample_counts(ps_gg_fp_f_prevalence_filter_2019_05_26, function(x)(x/sum(x)))

ASV_table <- as.data.frame(otu_table(ps_gg_fp_f_prevalence_filter_2019_05_26_proportion))
TAX_table <- as.data.frame(tax_table(ps_gg_fp_f_prevalence_filter_2019_05_26_proportion))
metadata <- as.data.frame(sample_data(ps_gg_fp_f_prevalence_filter_2019_05_26_proportion))

#US
metadata_us <- metadata[metadata$sample_cohort == "boston" & metadata$hiv_phenotype %in% c("1_hiv_negative", "2_suppressed"), , drop = F]
metadata_us <- metadata_us[metadata_us$sexual_orientation != "MSM", , drop = F]
us_asv_table <- ASV_table[row.names(ASV_table) %in% row.names(metadata_us), , drop = F]
hiv_us <- us_asv_table[,colnames(us_asv_table) %in% us[us$log2FoldChange>0, , drop = F]$taxon,drop = F]
neg_us <- us_asv_table[,colnames(us_asv_table) %in% us[us$log2FoldChange<0, , drop = F]$taxon,drop = F]

hiv_us <- as.data.frame(rowSums(hiv_us))
neg_us <- as.data.frame(rowSums(neg_us))

metadata_us <- metadata[row.names(metadata) %in% row.names(hiv_us), , drop = F]
all.equal(row.names(metadata_us), row.names(hiv_us))
```

```
## [1] TRUE
```

```
all.equal(row.names(metadata_us), row.names(neg_us))
```

```
## [1] TRUE
```

```
us_data <- cbind(hiv_us, neg_us, metadata_us)

# Create each individual plot

standard_error_bool = TRUE

cor.test(us_data$`rowSums(neg_us)`, us_data$scd14_ng_ml_uvm, method = "pearson") -> cor_neg_us_scd14
paste("Rho = ", signif(cor_neg_us_scd14$estimate, digits = 2), "\np = ", signif(cor_neg_us_scd14$p.value, digits = 2), sep = "") -> cor_neg_us_scd14_annot

# data:  us_data$`rowSums(neg_us)` and us_data$scd14_ng_ml_uvm
# t = -2.06954, df = 100, p-value = 0.041074
# alternative hypothesis: true correlation is not equal to 0
# 95 percent confidence interval:
#  -0.3820760411 -0.0085205193
# sample estimates:
#        cor 
# -0.2026596 

plot_us_neg_scd14 <- ggplot(data = us_data, aes(x = `rowSums(neg_us)`, y = scd14_ng_ml_uvm))+geom_point(aes(alpha = hiv_phenotype),color = "royalblue4", size = 2)+
  theme_bw()+geom_smooth(method = "lm", color = "black", se = standard_error_bool)+scale_alpha_manual(values = c(1, 0.6))+ggtitle("Decreased in HIV")+theme(legend.position = "none", plot.title = element_text(hjust = 0.5), axis.title = element_blank()) + annotate("text", x = Inf, y = Inf, label = cor_neg_us_scd14_annot, hjust = "inward", vjust = "inward", fontface = "bold")

cor.test(us_data$`rowSums(hiv_us)`, us_data$scd14_ng_ml_uvm, method = "pearson") -> cor_hiv_us_scd14
```

```
## Warning in cor(x, y): the standard deviation is zero
```

```
paste("Rho = ", signif(cor_hiv_us_scd14$estimate, digits = 2), "\np = ", signif(cor_hiv_us_scd14$p.value, digits = 2), sep = "") -> cor_hiv_us_scd14_annot

plot_us_pos_scd14 <- ggplot(data = us_data, aes(x = `rowSums(hiv_us)`, y = scd14_ng_ml_uvm))+geom_point(aes(alpha = hiv_phenotype),color = "royalblue4", size = 2)+
  theme_bw()+geom_smooth(method = "lm", color = "black", se = standard_error_bool)+scale_alpha_manual(values = c(1, 0.6))+ggtitle("Increased in HIV")+theme(legend.position = "none", plot.title = element_text(hjust = 0.5), axis.title = element_blank()) + annotate("text", x = Inf, y = Inf, label = cor_hiv_us_scd14_annot, hjust = "inward", vjust = "inward")

cor.test(us_data$`rowSums(neg_us)`, us_data$ifabp_pg_ml_uvm, method = "pearson") -> cor_neg_us_ifabp
paste("Rho = ", signif(cor_neg_us_ifabp$estimate, digits = 2), "\np = ", signif(cor_neg_us_ifabp$p.value, digits = 2), sep = "") -> cor_neg_us_ifabp_annot

# data:  us_data$`rowSums(neg_us)` and us_data$ifabp_pg_ml_uvm
# t = -0.448099, df = 100, p-value = 0.65505
# alternative hypothesis: true correlation is not equal to 0
# 95 percent confidence interval:
#  -0.23717511  0.15102469
# sample estimates:
#          cor 
# -0.044764978 

plot_us_neg_ifabp <- ggplot(data = us_data, aes(x = `rowSums(neg_us)`, y = ifabp_pg_ml_uvm))+geom_point(aes(alpha = hiv_phenotype),color = "royalblue4", size = 2)+
  theme_bw()+geom_smooth(method = "lm", color = "black", se = standard_error_bool)+scale_alpha_manual(values = c(1, 0.6))+ggtitle("neg us")+theme(legend.position = "none", plot.title = element_blank(), axis.title = element_blank()) + annotate("text", x = Inf, y = Inf, label = cor_neg_us_ifabp_annot, hjust = "inward", vjust = "inward")

cor.test(us_data$`rowSums(hiv_us)`, us_data$ifabp_pg_ml_uvm, method = "pearson") -> cor_hiv_us_ifabp
```

```
## Warning in cor(x, y): the standard deviation is zero
```

```
paste("Rho = ", signif(cor_hiv_us_ifabp$estimate, digits = 2), "\np = ", signif(cor_hiv_us_ifabp$p.value, digits = 2), sep = "") -> cor_hiv_us_ifabp_annot

plot_us_pos_ifabp <- ggplot(data = us_data, aes(x = `rowSums(hiv_us)`, y = ifabp_pg_ml_uvm))+geom_point(aes(alpha = hiv_phenotype), color = "royalblue4",size = 2)+
  theme_bw()+geom_smooth(method = "lm", color = "black", se = standard_error_bool)+scale_alpha_manual(values = c(1, 0.6))+ggtitle("hiv us")+theme(legend.position = "none", plot.title = element_blank(), axis.title = element_blank()) + annotate("text", x = Inf, y = Inf, label = cor_hiv_us_ifabp_annot, hjust = "inward", vjust = "inward")

# Test within each HIV phenotype

metadata_us <-  metadata[metadata$sample_cohort == "boston" & metadata$sexual_orientation != "MSM", , drop = F]
us_test_data <- tibble::tibble(corr = c("cor_neg_us_scd14", "cor_hiv_us_scd14", "cor_neg_us_ifabp", "cor_hiv_us_ifabp"), display = c(cor_neg_us_scd14_annot, cor_hiv_us_scd14_annot, cor_neg_us_ifabp_annot, cor_hiv_us_ifabp_annot))

for(test_phenotype in c("1_hiv_negative", "2_suppressed", "4_unsuppressed")){
  
  metadata_us_test <- metadata_us[metadata_us$hiv_phenotype == test_phenotype, , drop = F]
  us_asv_table <- ASV_table[row.names(ASV_table) %in% row.names(metadata_us_test), , drop = F]
  hiv_us <- us_asv_table[,colnames(us_asv_table) %in% us[us$log2FoldChange>0, , drop = F]$taxon,drop = F]
  neg_us <- us_asv_table[,colnames(us_asv_table) %in% us[us$log2FoldChange<0, , drop = F]$taxon,drop = F]
  
  hiv_us <- as.data.frame(rowSums(hiv_us))
  neg_us <- as.data.frame(rowSums(neg_us))
  
  metadata_us_test <- metadata[row.names(metadata) %in% row.names(hiv_us), , drop = F]
  all.equal(row.names(metadata_us_test), row.names(hiv_us))
  all.equal(row.names(metadata_us_test), row.names(neg_us))
  
  us_data_test <- cbind(hiv_us, neg_us, metadata_us_test)
  
  cor.test(us_data_test$`rowSums(neg_us)`, us_data_test$scd14_ng_ml_uvm, method = "pearson") -> cor_neg_us_scd14
  cor.test(us_data_test$`rowSums(hiv_us)`, us_data_test$scd14_ng_ml_uvm, method = "pearson") -> cor_hiv_us_scd14
  cor.test(us_data_test$`rowSums(neg_us)`, us_data_test$ifabp_pg_ml_uvm, method = "pearson") -> cor_neg_us_ifabp
  cor.test(us_data_test$`rowSums(hiv_us)`, us_data_test$ifabp_pg_ml_uvm, method = "pearson") -> cor_hiv_us_ifabp
  
  us_test_data[[paste(test_phenotype, "Rho")]] <- c(signif(cor_neg_us_scd14$estimate, digits = 2), signif(cor_hiv_us_scd14$estimate, digits = 2), signif(cor_neg_us_ifabp$estimate, digits = 2), signif(cor_hiv_us_ifabp$estimate, digits = 2))
  us_test_data[[paste(test_phenotype, "p")]] <- c(signif(cor_neg_us_scd14$p.value, digits = 2), signif(cor_hiv_us_scd14$p.value, digits = 2), signif(cor_neg_us_ifabp$p.value, digits = 2), signif(cor_hiv_us_ifabp$p.value, digits = 2))
} # end
```

```
## Warning in cor(x, y): the standard deviation is zero

## Warning in cor(x, y): the standard deviation is zero

## Warning in cor(x, y): the standard deviation is zero

## Warning in cor(x, y): the standard deviation is zero

## Warning in cor(x, y): the standard deviation is zero

## Warning in cor(x, y): the standard deviation is zero

## Warning in cor(x, y): the standard deviation is zero

## Warning in cor(x, y): the standard deviation is zero
```

```
print(us_test_data)
```

```
## # A tibble: 4 × 8
##   corr             display       1_hiv…¹ 1_hiv…² 2_sup…³ 2_sup…⁴ 4_uns…⁵ 4_uns…⁶
##   <chr>            <chr>           <dbl>   <dbl>   <dbl>   <dbl>   <dbl>   <dbl>
## 1 cor_neg_us_scd14 "Rho = -0.2\…   -0.15    0.17      NA      NA -0.0046    0.99
## 2 cor_hiv_us_scd14 "Rho = NA\np…   NA      NA         NA      NA NA        NA   
## 3 cor_neg_us_ifabp "Rho = -0.04…    0.04    0.72      NA      NA -0.48      0.13
## 4 cor_hiv_us_ifabp "Rho = NA\np…   NA      NA         NA      NA NA        NA   
## # … with abbreviated variable names ¹​`1_hiv_negative Rho`, ²​`1_hiv_negative p`,
## #   ³​`2_suppressed Rho`, ⁴​`2_suppressed p`, ⁵​`4_unsuppressed Rho`,
## #   ⁶​`4_unsuppressed p`
```

```
#BOTSWANA
metadata_botswana <- metadata[metadata$sample_cohort == "botswana" & metadata$hiv_phenotype %in% c("1_hiv_negative", "2_suppressed"), , drop = F]
botswana_asv_table <- ASV_table[row.names(ASV_table) %in% row.names(metadata_botswana), , drop = F]
hiv_botswana <- botswana_asv_table[,colnames(botswana_asv_table) %in% botswana[botswana$log2FoldChange>0, , drop = F]$taxon,drop = F]
neg_botswana <- botswana_asv_table[,colnames(botswana_asv_table) %in% botswana[botswana$log2FoldChange<0, , drop = F]$taxon,drop = F]

hiv_botswana <- as.data.frame(rowSums(hiv_botswana))
neg_botswana <- as.data.frame(rowSums(neg_botswana))

metadata_botswana <- metadata[row.names(metadata) %in% row.names(hiv_botswana), , drop = F]
all.equal(row.names(metadata_botswana), row.names(hiv_botswana))
```

```
## [1] TRUE
```

```
all.equal(row.names(metadata_botswana), row.names(neg_botswana))
```

```
## [1] TRUE
```

```
botswana_data <- cbind(hiv_botswana, neg_botswana, metadata_botswana)

# Create each individual plot

cor.test(botswana_data$`rowSums(neg_botswana`, botswana_data$scd14_ng_ml_uvm, method = "pearson") -> cor_neg_botswana_scd14
paste("Rho = ", signif(cor_neg_botswana_scd14$estimate, digits = 2), "\np = ", signif(cor_neg_botswana_scd14$p.value, digits = 2), sep = "") -> cor_neg_botswana_scd14_annot

# data:  botswana_data$`rowSums(neg_botswana` and botswana_data$scd14_ng_ml_uvm
# t = -1.36529, df = 125, p-value = 0.17461
# alternative hypothesis: true correlation is not equal to 0
# 95 percent confidence interval:
#  -0.289319682  0.054143286
# sample estimates:
#         cor 
# -0.12121472 

plot_botswana_neg_scd14 <- ggplot(data = botswana_data, aes(x = `rowSums(neg_botswana)`, y = scd14_ng_ml_uvm))+geom_point(aes(alpha = hiv_phenotype),color = "darkorange", size = 2)+
  theme_bw()+geom_smooth(method = "lm", color = "black", se = standard_error_bool)+scale_alpha_manual(values = c(1, 0.6))+ggtitle("Decreased in HIV")+theme(legend.position = "none", plot.title = element_text(hjust = 0.5), axis.title = element_blank()) + annotate("text", x = Inf, y = Inf, label = cor_neg_botswana_scd14_annot, hjust = "inward", vjust = "inward")

cor.test(botswana_data$`rowSums(hiv_botswana)`, botswana_data$scd14_ng_ml_uvm, method = "pearson") -> cor_hiv_botswana_scd14
paste("Rho = ", signif(cor_hiv_botswana_scd14$estimate, digits = 2), "\np = ", signif(cor_hiv_botswana_scd14$p.value, digits = 2), sep = "") -> cor_hiv_botswana_scd14_annot

# data:  botswana_data$`rowSums(hiv_botswana)` and botswana_data$scd14_ng_ml_uvm
# t = 1.8925, df = 125, p-value = 0.060737
# alternative hypothesis: true correlation is not equal to 0
# 95 percent confidence interval:
#  -0.0075376786  0.3314729384
# sample estimates:
#        cor 
# 0.16689612 

plot_botswana_pos_scd14 <- ggplot(data = botswana_data, aes(x = `rowSums(hiv_botswana)`, y = scd14_ng_ml_uvm))+geom_point(aes(alpha = hiv_phenotype),color = "darkorange", size = 2)+
  theme_bw()+geom_smooth(method = "lm", color = "black", se = standard_error_bool)+scale_alpha_manual(values = c(1, 0.6))+ggtitle("Increased in HIV")+theme(legend.position = "none", plot.title = element_text(hjust = 0.5), axis.title = element_blank()) + annotate("text", x = Inf, y = Inf, label = cor_hiv_botswana_scd14_annot, hjust = "inward", vjust = "inward")

cor.test(botswana_data$`rowSums(neg_botswana)`, botswana_data$ifabp_pg_ml_uvm, method = "pearson") -> cor_neg_botswana_ifabp
paste("Rho = ", signif(cor_neg_botswana_ifabp$estimate, digits = 2), "\np = ", signif(cor_neg_botswana_ifabp$p.value, digits = 2), sep = "") -> cor_neg_botswana_ifabp_annot

# data:  botswana_data$`rowSums(neg_botswana)` and botswana_data$ifabp_pg_ml_uvm
# t = -0.687037, df = 125, p-value = 0.49333
# alternative hypothesis: true correlation is not equal to 0
# 95 percent confidence interval:
#  -0.23305908  0.11409906
# sample estimates:
#          cor 
# -0.061334779 

plot_botswana_neg_ifabp <- ggplot(data = botswana_data, aes(x = `rowSums(neg_botswana)`, y = ifabp_pg_ml_uvm))+geom_point(aes(alpha = hiv_phenotype),color = "darkorange", size = 2)+
  theme_bw()+geom_smooth(method = "lm", color = "black", se = standard_error_bool)+scale_alpha_manual(values = c(1, 0.6))+ggtitle("neg botswana")+theme(legend.position = "none", plot.title =  element_blank(), axis.title = element_blank()) + annotate("text", x = Inf, y = Inf, label = cor_neg_botswana_ifabp_annot, hjust = "inward", vjust = "inward")

cor.test(botswana_data$`rowSums(hiv_botswana)`, botswana_data$ifabp_pg_ml_uvm, method = "pearson") -> cor_hiv_botswana_ifabp
paste("Rho = ", signif(cor_hiv_botswana_ifabp$estimate, digits = 2), "\np = ", signif(cor_hiv_botswana_ifabp$p.value, digits = 2), sep = "") -> cor_hiv_botswana_ifabp_annot

# data:  botswana_data$`rowSums(hiv_botswana)` and botswana_data$ifabp_pg_ml_uvm
# t = 2.61702, df = 125, p-value = 0.0099643
# alternative hypothesis: true correlation is not equal to 0
# 95 percent confidence interval:
#  0.055918694 0.386770642
# sample estimates:
#        cor 
# 0.22791304 

plot_botswana_pos_ifabp <- ggplot(data = botswana_data, aes(x = `rowSums(hiv_botswana)`, y = ifabp_pg_ml_uvm))+geom_point(aes(alpha = hiv_phenotype), color = "darkorange",size = 2)+
  theme_bw()+geom_smooth(method = "lm", color = "black", se = standard_error_bool)+scale_alpha_manual(values = c(1, 0.6))+ggtitle("hiv botswana")+theme(legend.position = "none", plot.title =  element_blank(), axis.title = element_blank()) + annotate("text", x = Inf, y = Inf, label = cor_hiv_botswana_ifabp_annot, hjust = "inward", vjust = "inward", fontface = "bold")

# Test within each HIV phenotype

metadata_botswana <-  metadata[metadata$sample_cohort == "botswana" & metadata$sexual_orientation != "MSM", , drop = F]
botswana_test_data <- tibble::tibble(corr = c("cor_neg_botswana_scd14", "cor_hiv_botswana_scd14", "cor_neg_botswana_ifabp", "cor_hiv_botswana_ifabp"), display = c(cor_neg_botswana_scd14_annot, cor_hiv_botswana_scd14_annot, cor_neg_botswana_ifabp_annot, cor_hiv_botswana_ifabp_annot))

for(test_phenotype in c("1_hiv_negative", "2_suppressed", "4_unsuppressed")){
  
  metadata_botswana_test <- metadata_botswana[metadata_botswana$hiv_phenotype == test_phenotype, , drop = F]
  botswana_asv_table <- ASV_table[row.names(ASV_table) %in% row.names(metadata_botswana_test), , drop = F]
  hiv_botswana <- botswana_asv_table[,colnames(botswana_asv_table) %in% botswana[botswana$log2FoldChange>0, , drop = F]$taxon,drop = F]
  neg_botswana <- botswana_asv_table[,colnames(botswana_asv_table) %in% botswana[botswana$log2FoldChange<0, , drop = F]$taxon,drop = F]
  
  hiv_botswana <- as.data.frame(rowSums(hiv_botswana))
  neg_botswana <- as.data.frame(rowSums(neg_botswana))
  
  metadata_botswana_test <- metadata[row.names(metadata) %in% row.names(hiv_botswana), , drop = F]
  all.equal(row.names(metadata_botswana_test), row.names(hiv_botswana))
  all.equal(row.names(metadata_botswana_test), row.names(neg_botswana))
  
  botswana_data_test <- cbind(hiv_botswana, neg_botswana, metadata_botswana_test)
  
  cor.test(botswana_data_test$`rowSums(neg_botswana)`, botswana_data_test$scd14_ng_ml_uvm, method = "pearson") -> cor_neg_botswana_scd14
  cor.test(botswana_data_test$`rowSums(hiv_botswana)`, botswana_data_test$scd14_ng_ml_uvm, method = "pearson") -> cor_hiv_botswana_scd14
  cor.test(botswana_data_test$`rowSums(neg_botswana)`, botswana_data_test$ifabp_pg_ml_uvm, method = "pearson") -> cor_neg_botswana_ifabp
  cor.test(botswana_data_test$`rowSums(hiv_botswana)`, botswana_data_test$ifabp_pg_ml_uvm, method = "pearson") -> cor_hiv_botswana_ifabp
  
  botswana_test_data[[paste(test_phenotype, "Rho")]] <- c(signif(cor_neg_botswana_scd14$estimate, digits = 2), signif(cor_hiv_botswana_scd14$estimate, digits = 2), signif(cor_neg_botswana_ifabp$estimate, digits = 2), signif(cor_hiv_botswana_ifabp$estimate, digits = 2))
  botswana_test_data[[paste(test_phenotype, "p")]] <- c(signif(cor_neg_botswana_scd14$p.value, digits = 2), signif(cor_hiv_botswana_scd14$p.value, digits = 2), signif(cor_neg_botswana_ifabp$p.value, digits = 2), signif(cor_hiv_botswana_ifabp$p.value, digits = 2))
} # end 

print(botswana_test_data)
```

```
## # A tibble: 4 × 8
##   corr                   display 1_hiv…¹ 1_hiv…² 2_sup…³ 2_sup…⁴ 4_uns…⁵ 4_uns…⁶
##   <chr>                  <chr>     <dbl>   <dbl>   <dbl>   <dbl>   <dbl>   <dbl>
## 1 cor_neg_botswana_scd14 "Rho =…  -0.13     0.33   0.13     0.29  -0.02    0.91 
## 2 cor_hiv_botswana_scd14 "Rho =…   0.11     0.41   0.027    0.83   0.32    0.057
## 3 cor_neg_botswana_ifabp "Rho =…  -0.025    0.85   0.099    0.43  -0.066   0.71 
## 4 cor_hiv_botswana_ifabp "Rho =…   0.2      0.13   0.11     0.37  -0.036   0.84 
## # … with abbreviated variable names ¹​`1_hiv_negative Rho`, ²​`1_hiv_negative p`,
## #   ³​`2_suppressed Rho`, ⁴​`2_suppressed p`, ⁵​`4_unsuppressed Rho`,
## #   ⁶​`4_unsuppressed p`
```

```
#UGANDA
metadata_uganda <- metadata[metadata$sample_cohort == "uganda_2" & metadata$hiv_phenotype %in% c("1_hiv_negative", "2_suppressed"), , drop = F]
uganda_asv_table <- ASV_table[row.names(ASV_table) %in% row.names(metadata_uganda), , drop = F]
hiv_uganda <- uganda_asv_table[,colnames(uganda_asv_table) %in% uganda[uganda$log2FoldChange>0, , drop = F]$taxon,drop = F]
neg_uganda <- uganda_asv_table[,colnames(uganda_asv_table) %in% uganda[uganda$log2FoldChange<0, , drop = F]$taxon,drop = F]

hiv_uganda <- as.data.frame(rowSums(hiv_uganda))
neg_uganda <- as.data.frame(rowSums(neg_uganda))

metadata_uganda <- metadata[row.names(metadata) %in% row.names(hiv_uganda), , drop = F]
all.equal(row.names(metadata_uganda), row.names(hiv_uganda))
```

```
## [1] TRUE
```

```
all.equal(row.names(metadata_uganda), row.names(neg_uganda))
```

```
## [1] TRUE
```

```
uganda_data <- cbind(hiv_uganda, neg_uganda, metadata_uganda)

# Create each individual plot

cor.test(uganda_data$`rowSums(neg_uganda`, uganda_data$scd14_ng_ml_uvm, method = "pearson") -> cor_neg_uganda_scd14
paste("Rho = ", signif(cor_neg_uganda_scd14$estimate, digits = 2), "\np = ", signif(cor_neg_uganda_scd14$p.value, digits = 2), sep = "") -> cor_neg_uganda_scd14_annot

# data:  uganda_data$`rowSums(neg_uganda` and uganda_data$scd14_ng_ml_uvm
# t = -3.74324, df = 159, p-value = 0.00025342
# alternative hypothesis: true correlation is not equal to 0
# 95 percent confidence interval:
#  -0.42073857 -0.13589055
# sample estimates:
#         cor 
# -0.28458377 

plot_uganda_neg_scd14 <- ggplot(data = uganda_data, aes(x = `rowSums(neg_uganda)`, y = scd14_ng_ml_uvm))+geom_point(aes(alpha = hiv_phenotype),color = "darkgreen", size = 2)+
  theme_bw()+geom_smooth(method = "lm", color = "black", se = standard_error_bool)+scale_alpha_manual(values = c(1, 0.6))+ggtitle("Decreased in HIV")+theme(legend.position = "none", plot.title = element_text(hjust = 0.5), axis.title = element_blank()) + annotate("text", x = Inf, y = Inf, label = cor_neg_uganda_scd14_annot, hjust = "inward", vjust = "inward", fontface = "bold")

cor.test(uganda_data$`rowSums(hiv_uganda)`, uganda_data$scd14_ng_ml_uvm, method = "pearson") -> cor_hiv_uganda_scd14
paste("Rho = ", signif(cor_hiv_uganda_scd14$estimate, digits = 2), "\np = ", signif(cor_hiv_uganda_scd14$p.value, digits = 2), sep = "") -> cor_hiv_uganda_scd14_annot

# data:  uganda_data$`rowSums(hiv_uganda)` and uganda_data$scd14_ng_ml_uvm
# t = 2.88903, df = 159, p-value = 0.0044037
# alternative hypothesis: true correlation is not equal to 0
# 95 percent confidence interval:
#  0.071109591 0.365381479
# sample estimates:
#        cor 
# 0.22332809 

plot_uganda_pos_scd14 <- ggplot(data = uganda_data, aes(x = `rowSums(hiv_uganda)`, y = scd14_ng_ml_uvm))+geom_point(aes(alpha = hiv_phenotype),color = "darkgreen", size = 2)+
  theme_bw()+geom_smooth(method = "lm", color = "black", se = standard_error_bool)+scale_alpha_manual(values = c(1, 0.6))+ggtitle("Increased in HIV")+theme(legend.position = "none", plot.title = element_text(hjust = 0.5), axis.title = element_blank()) + annotate("text", x = Inf, y = Inf, label = cor_hiv_uganda_scd14_annot, hjust = "inward", vjust = "inward", fontface = "bold")

cor.test(uganda_data$`rowSums(neg_uganda)`, uganda_data$ifabp_pg_ml_uvm, method = "pearson") -> cor_neg_uganda_ifabp
paste("Rho = ", signif(cor_neg_uganda_ifabp$estimate, digits = 2), "\np = ", signif(cor_neg_uganda_ifabp$p.value, digits = 2), sep = "") -> cor_neg_uganda_ifabp_annot

# data:  uganda_data$`rowSums(neg_uganda)` and uganda_data$ifabp_pg_ml_uvm
# t = -0.891309, df = 159, p-value = 0.37411
# alternative hypothesis: true correlation is not equal to 0
# 95 percent confidence interval:
#  -0.222754953  0.085093408
# sample estimates:
#          cor 
# -0.070509476 

plot_uganda_neg_ifabp <- ggplot(data = uganda_data, aes(x = `rowSums(neg_uganda)`, y = ifabp_pg_ml_uvm))+geom_point(aes(alpha = hiv_phenotype),color = "darkgreen", size = 2)+
  theme_bw()+geom_smooth(method = "lm", color = "black", se = standard_error_bool)+scale_alpha_manual(values = c(1, 0.6))+ggtitle("neg uganda")+theme(legend.position = "none", plot.title =  element_blank(), axis.title = element_blank()) + annotate("text", x = Inf, y = Inf, label = cor_neg_uganda_ifabp_annot, hjust = "inward", vjust = "inward")

cor.test(uganda_data$`rowSums(hiv_uganda)`, uganda_data$ifabp_pg_ml_uvm, method = "pearson") -> cor_hiv_uganda_ifabp
paste("Rho = ", signif(cor_hiv_uganda_ifabp$estimate, digits = 2), "\np = ", signif(cor_hiv_uganda_ifabp$p.value, digits = 2), sep = "") -> cor_hiv_uganda_ifabp_annot

# data:  uganda_data$`rowSums(hiv_uganda)` and uganda_data$ifabp_pg_ml_uvm
# t = 1.99129, df = 159, p-value = 0.048162
# alternative hypothesis: true correlation is not equal to 0
# 95 percent confidence interval:
#  0.0013438605 0.3033423516
# sample estimates:
#        cor 
# 0.15598629 

plot_uganda_pos_ifabp <- ggplot(data = uganda_data, aes(x = `rowSums(hiv_uganda)`, y = ifabp_pg_ml_uvm))+geom_point(aes(alpha = hiv_phenotype), color = "darkgreen",size = 2)+
  theme_bw()+geom_smooth(method = "lm", color = "black", se = standard_error_bool)+scale_alpha_manual(values = c(1, 0.6))+ggtitle("hiv uganda")+theme(legend.position = "none", plot.title =  element_blank(), axis.title = element_blank()) + annotate("text", x = Inf, y = Inf, label = cor_hiv_uganda_ifabp_annot, hjust = "inward", vjust = "inward", fontface = "bold")

# Test within each HIV phenotype

metadata_uganda <-  metadata[metadata$sample_cohort == "uganda_2" & metadata$sexual_orientation != "MSM", , drop = F]
uganda_test_data <- tibble::tibble(corr = c("cor_neg_uganda_scd14", "cor_hiv_uganda_scd14", "cor_neg_uganda_ifabp", "cor_hiv_uganda_ifabp"), display = c(cor_neg_uganda_scd14_annot, cor_hiv_uganda_scd14_annot, cor_neg_uganda_ifabp_annot, cor_hiv_uganda_ifabp_annot))

for(test_phenotype in c("1_hiv_negative", "2_suppressed")){
  
  metadata_uganda_test <- metadata_uganda[metadata_uganda$hiv_phenotype == test_phenotype, , drop = F]
  uganda_asv_table <- ASV_table[row.names(ASV_table) %in% row.names(metadata_uganda_test), , drop = F]
  hiv_uganda <- uganda_asv_table[,colnames(uganda_asv_table) %in% uganda[uganda$log2FoldChange>0, , drop = F]$taxon,drop = F]
  neg_uganda <- uganda_asv_table[,colnames(uganda_asv_table) %in% uganda[uganda$log2FoldChange<0, , drop = F]$taxon,drop = F]
  
  hiv_uganda <- as.data.frame(rowSums(hiv_uganda))
  neg_uganda <- as.data.frame(rowSums(neg_uganda))
  
  metadata_uganda_test <- metadata[row.names(metadata) %in% row.names(hiv_uganda), , drop = F]
  all.equal(row.names(metadata_uganda_test), row.names(hiv_uganda))
  all.equal(row.names(metadata_uganda_test), row.names(neg_uganda))
  
  uganda_data_test <- cbind(hiv_uganda, neg_uganda, metadata_uganda_test)
  
  cor.test(uganda_data_test$`rowSums(neg_uganda)`, uganda_data_test$scd14_ng_ml_uvm, method = "pearson") -> cor_neg_uganda_scd14
  cor.test(uganda_data_test$`rowSums(hiv_uganda)`, uganda_data_test$scd14_ng_ml_uvm, method = "pearson") -> cor_hiv_uganda_scd14
  cor.test(uganda_data_test$`rowSums(neg_uganda)`, uganda_data_test$ifabp_pg_ml_uvm, method = "pearson") -> cor_neg_uganda_ifabp
  cor.test(uganda_data_test$`rowSums(hiv_uganda)`, uganda_data_test$ifabp_pg_ml_uvm, method = "pearson") -> cor_hiv_uganda_ifabp
  
  uganda_test_data[[paste(test_phenotype, "Rho")]] <- c(signif(cor_neg_uganda_scd14$estimate, digits = 2), signif(cor_hiv_uganda_scd14$estimate, digits = 2), signif(cor_neg_uganda_ifabp$estimate, digits = 2), signif(cor_hiv_uganda_ifabp$estimate, digits = 2))
  uganda_test_data[[paste(test_phenotype, "p")]] <- c(signif(cor_neg_uganda_scd14$p.value, digits = 2), signif(cor_hiv_uganda_scd14$p.value, digits = 2), signif(cor_neg_uganda_ifabp$p.value, digits = 2), signif(cor_hiv_uganda_ifabp$p.value, digits = 2))
} # end 

print(uganda_test_data)
```

```
## # A tibble: 4 × 6
##   corr                 display                   1_hiv…¹ 1_hiv…² 2_sup…³ 2_sup…⁴
##   <chr>                <chr>                       <dbl>   <dbl>   <dbl>   <dbl>
## 1 cor_neg_uganda_scd14 "Rho = -0.28\np = 0.0002…  -0.084    0.47  -0.2      0.06
## 2 cor_hiv_uganda_scd14 "Rho = 0.22\np = 0.0044"    0.13     0.28   0.033    0.76
## 3 cor_neg_uganda_ifabp "Rho = -0.071\np = 0.37"    0.021    0.86  -0.014    0.9 
## 4 cor_hiv_uganda_ifabp "Rho = 0.16\np = 0.048"     0.044    0.71   0.14     0.21
## # … with abbreviated variable names ¹​`1_hiv_negative Rho`, ²​`1_hiv_negative p`,
## #   ³​`2_suppressed Rho`, ⁴​`2_suppressed p`
```

```
# Combine plots for final figure

us_test_data
```

```
## # A tibble: 4 × 8
##   corr             display       1_hiv…¹ 1_hiv…² 2_sup…³ 2_sup…⁴ 4_uns…⁵ 4_uns…⁶
##   <chr>            <chr>           <dbl>   <dbl>   <dbl>   <dbl>   <dbl>   <dbl>
## 1 cor_neg_us_scd14 "Rho = -0.2\…   -0.15    0.17      NA      NA -0.0046    0.99
## 2 cor_hiv_us_scd14 "Rho = NA\np…   NA      NA         NA      NA NA        NA   
## 3 cor_neg_us_ifabp "Rho = -0.04…    0.04    0.72      NA      NA -0.48      0.13
## 4 cor_hiv_us_ifabp "Rho = NA\np…   NA      NA         NA      NA NA        NA   
## # … with abbreviated variable names ¹​`1_hiv_negative Rho`, ²​`1_hiv_negative p`,
## #   ³​`2_suppressed Rho`, ⁴​`2_suppressed p`, ⁵​`4_unsuppressed Rho`,
## #   ⁶​`4_unsuppressed p`
```

```
botswana_test_data
```

```
## # A tibble: 4 × 8
##   corr                   display 1_hiv…¹ 1_hiv…² 2_sup…³ 2_sup…⁴ 4_uns…⁵ 4_uns…⁶
##   <chr>                  <chr>     <dbl>   <dbl>   <dbl>   <dbl>   <dbl>   <dbl>
## 1 cor_neg_botswana_scd14 "Rho =…  -0.13     0.33   0.13     0.29  -0.02    0.91 
## 2 cor_hiv_botswana_scd14 "Rho =…   0.11     0.41   0.027    0.83   0.32    0.057
## 3 cor_neg_botswana_ifabp "Rho =…  -0.025    0.85   0.099    0.43  -0.066   0.71 
## 4 cor_hiv_botswana_ifabp "Rho =…   0.2      0.13   0.11     0.37  -0.036   0.84 
## # … with abbreviated variable names ¹​`1_hiv_negative Rho`, ²​`1_hiv_negative p`,
## #   ³​`2_suppressed Rho`, ⁴​`2_suppressed p`, ⁵​`4_unsuppressed Rho`,
## #   ⁶​`4_unsuppressed p`
```

```
uganda_test_data
```

```
## # A tibble: 4 × 6
##   corr                 display                   1_hiv…¹ 1_hiv…² 2_sup…³ 2_sup…⁴
##   <chr>                <chr>                       <dbl>   <dbl>   <dbl>   <dbl>
## 1 cor_neg_uganda_scd14 "Rho = -0.28\np = 0.0002…  -0.084    0.47  -0.2      0.06
## 2 cor_hiv_uganda_scd14 "Rho = 0.22\np = 0.0044"    0.13     0.28   0.033    0.76
## 3 cor_neg_uganda_ifabp "Rho = -0.071\np = 0.37"    0.021    0.86  -0.014    0.9 
## 4 cor_hiv_uganda_ifabp "Rho = 0.16\np = 0.048"     0.044    0.71   0.14     0.21
## # … with abbreviated variable names ¹​`1_hiv_negative Rho`, ²​`1_hiv_negative p`,
## #   ³​`2_suppressed Rho`, ⁴​`2_suppressed p`
```

```
ggsave("Figure4B_ANCOM_v18.pdf", grid.arrange(plot_us_neg_scd14, plot_botswana_neg_scd14, plot_botswana_pos_scd14, plot_uganda_neg_scd14, plot_uganda_pos_scd14, plot_us_neg_ifabp, plot_botswana_neg_ifabp, plot_botswana_pos_ifabp, plot_uganda_neg_ifabp, plot_uganda_pos_ifabp, ncol = 5, bottom = "Relative abundance", 
                                             top = "US                             Botswana                             Uganda", 
                                             left = "iFABP (pg/ml)                                      sCD14 (ng/ml)"), width = 15, height = 8)
```

```
## `geom_smooth()` using formula = 'y ~ x'
```

```
## Warning: Removed 2 rows containing non-finite values (`stat_smooth()`).
```

```
## Warning: Removed 2 rows containing missing values (`geom_point()`).
```

```
## `geom_smooth()` using formula = 'y ~ x'
```

```
## Warning: Removed 26 rows containing non-finite values (`stat_smooth()`).
```

```
## Warning: Removed 26 rows containing missing values (`geom_point()`).
```

```
## `geom_smooth()` using formula = 'y ~ x'
```

```
## Warning: Removed 26 rows containing non-finite values (`stat_smooth()`).
## Removed 26 rows containing missing values (`geom_point()`).
```

```
## `geom_smooth()` using formula = 'y ~ x'
```

```
## Warning: Removed 9 rows containing non-finite values (`stat_smooth()`).
```

```
## Warning: Removed 9 rows containing missing values (`geom_point()`).
```

```
## `geom_smooth()` using formula = 'y ~ x'
```

```
## Warning: Removed 9 rows containing non-finite values (`stat_smooth()`).
## Removed 9 rows containing missing values (`geom_point()`).
```

```
## `geom_smooth()` using formula = 'y ~ x'
```

```
## Warning: Removed 2 rows containing non-finite values (`stat_smooth()`).
```

```
## Warning: Removed 2 rows containing missing values (`geom_point()`).
```

```
## `geom_smooth()` using formula = 'y ~ x'
```

```
## Warning: Removed 26 rows containing non-finite values (`stat_smooth()`).
```

```
## Warning: Removed 26 rows containing missing values (`geom_point()`).
```

```
## `geom_smooth()` using formula = 'y ~ x'
```

```
## Warning: Removed 26 rows containing non-finite values (`stat_smooth()`).
## Removed 26 rows containing missing values (`geom_point()`).
```

```
## `geom_smooth()` using formula = 'y ~ x'
```

```
## Warning: Removed 9 rows containing non-finite values (`stat_smooth()`).
```

```
## Warning: Removed 9 rows containing missing values (`geom_point()`).
```

```
## `geom_smooth()` using formula = 'y ~ x'
```

```
## Warning: Removed 9 rows containing non-finite values (`stat_smooth()`).
## Removed 9 rows containing missing values (`geom_point()`).
```

```
ggsave("Figure4B_ANCOM_logscale_v2.pdf", grid.arrange(plot_us_neg_scd14 + scale_x_log10(), plot_botswana_neg_scd14 + scale_x_log10(), plot_botswana_pos_scd14 + scale_x_log10(), plot_uganda_neg_scd14 + scale_x_log10(), plot_uganda_pos_scd14 + scale_x_log10(), plot_us_neg_ifabp + scale_x_log10(), plot_botswana_neg_ifabp + scale_x_log10(), plot_botswana_pos_ifabp + scale_x_log10(), plot_uganda_neg_ifabp + scale_x_log10(), plot_uganda_pos_ifabp + scale_x_log10(), ncol = 5, bottom = "Relative abundance", 
                                             top = "US                             Botswana                             Uganda", 
                                             left = "iFABP (pg/ml)                                      sCD14 (ng/ml)"), width = 15, height = 8)
```

```
## Warning: Transformation introduced infinite values in continuous x-axis
```

```
## Warning: Transformation introduced infinite values in continuous x-axis
```

```
## `geom_smooth()` using formula = 'y ~ x'
```

```
## Warning: Removed 22 rows containing non-finite values (`stat_smooth()`).
```

```
## Warning: Removed 2 rows containing missing values (`geom_point()`).
```

```
## Warning: Transformation introduced infinite values in continuous x-axis
## Transformation introduced infinite values in continuous x-axis
```

```
## `geom_smooth()` using formula = 'y ~ x'
```

```
## Warning: Removed 35 rows containing non-finite values (`stat_smooth()`).
```

```
## Warning: Removed 26 rows containing missing values (`geom_point()`).
```

```
## Warning: Transformation introduced infinite values in continuous x-axis
## Transformation introduced infinite values in continuous x-axis
```

```
## `geom_smooth()` using formula = 'y ~ x'
```

```
## Warning: Removed 66 rows containing non-finite values (`stat_smooth()`).
```

```
## Warning: Removed 26 rows containing missing values (`geom_point()`).
```

```
## Warning: Transformation introduced infinite values in continuous x-axis
## Transformation introduced infinite values in continuous x-axis
```

```
## `geom_smooth()` using formula = 'y ~ x'
```

```
## Warning: Removed 14 rows containing non-finite values (`stat_smooth()`).
```

```
## Warning: Removed 9 rows containing missing values (`geom_point()`).
```

```
## `geom_smooth()` using formula = 'y ~ x'
```

```
## Warning: Removed 9 rows containing non-finite values (`stat_smooth()`).
## Removed 9 rows containing missing values (`geom_point()`).
```

```
## Warning: Transformation introduced infinite values in continuous x-axis
## Transformation introduced infinite values in continuous x-axis
```

```
## `geom_smooth()` using formula = 'y ~ x'
```

```
## Warning: Removed 22 rows containing non-finite values (`stat_smooth()`).
```

```
## Warning: Removed 2 rows containing missing values (`geom_point()`).
```

```
## Warning: Transformation introduced infinite values in continuous x-axis
## Transformation introduced infinite values in continuous x-axis
```

```
## `geom_smooth()` using formula = 'y ~ x'
```

```
## Warning: Removed 35 rows containing non-finite values (`stat_smooth()`).
```

```
## Warning: Removed 26 rows containing missing values (`geom_point()`).
```

```
## Warning: Transformation introduced infinite values in continuous x-axis
## Transformation introduced infinite values in continuous x-axis
```

```
## `geom_smooth()` using formula = 'y ~ x'
```

```
## Warning: Removed 66 rows containing non-finite values (`stat_smooth()`).
```

```
## Warning: Removed 26 rows containing missing values (`geom_point()`).
```

```
## Warning: Transformation introduced infinite values in continuous x-axis
## Transformation introduced infinite values in continuous x-axis
```

```
## `geom_smooth()` using formula = 'y ~ x'
```

```
## Warning: Removed 14 rows containing non-finite values (`stat_smooth()`).
```

```
## Warning: Removed 9 rows containing missing values (`geom_point()`).
```

```
## `geom_smooth()` using formula = 'y ~ x'
```

```
## Warning: Removed 9 rows containing non-finite values (`stat_smooth()`).
## Removed 9 rows containing missing values (`geom_point()`).
```

```
#--------------------------------------------------------------------------------------------------------------
```

#Figure 4C

```
#FIGURE 4C
#--------------------------------------------------------------------------------------------------------------
#Transform count data in the phyloseq object
ps_gg_fp_f_prevalence_filter_2019_05_26_proportion <- phyloseq::transform_sample_counts(ps_gg_fp_f_prevalence_filter_2019_05_26, function(x)(x/sum(x)))

# Get ASVs significant by ANCOM
us <- read.csv("ANCOM_US_NEG_ART_Filtered.csv", header = TRUE)
us <- dplyr::rename(us, log2FoldChange = lfc)
us <- us[order(us$log2FoldChange), , drop = F]
botswana <- read.csv("ANCOM_BOTS_NEG_ART_Filtered.csv", header = TRUE)
botswana <- dplyr::rename(botswana, log2FoldChange = lfc)
botswana <- botswana[order(botswana$log2FoldChange), , drop = F]
uganda <- read.csv("ANCOM_UGANDA_NEG_ART_Filtered.csv", header = TRUE)
uganda <- dplyr::rename(uganda, log2FoldChange = lfc)
uganda <- uganda[order(uganda$log2FoldChange), , drop = F]

sigtab_dataset <- rbind(us, botswana, uganda)

# Extract ASV and TAX tables
ASV_table <- as.data.frame(otu_table(ps_gg_fp_f_prevalence_filter_2019_05_26_proportion))
TAX_table <- as.data.frame(tax_table(ps_gg_fp_f_prevalence_filter_2019_05_26_proportion))

# Prune metadata to non-MSM and (HIV-uninfected or HIV-infected, ART-suppressed) and confirm inflammatory markers are numeric
metadata <- phyloseq::sample_data(ps_gg_fp_f_prevalence_filter_2019_05_26)
metadata <- metadata[metadata$hiv_phenotype %in% c("1_hiv_negative", "2_suppressed"), , drop = F]
metadata <- as.data.frame(as.matrix(metadata[metadata$sexual_orientation != "MSM", , drop = FALSE])) ### swap to dropping "MSM" 
metadata$scd14_ng_ml_uvm <- as.numeric(as.character(metadata$scd14_ng_ml_uvm))
metadata$ifabp_pg_ml_uvm <- as.numeric(as.character(metadata$ifabp_pg_ml_uvm))


#US
metadata_us <- metadata[metadata$sample_cohort == "boston", , drop = F]
TAX_table_us <- TAX_table[row.names(TAX_table) %in% us$taxon, , drop = F]
ASV_table_us <- ASV_table[, colnames(ASV_table) %in% us$taxon, drop = F]

cor_matrix_us <- c()
for (i in colnames(ASV_table_us)){
  for (j in c("scd14_ng_ml_uvm", "ifabp_pg_ml_uvm")){
    data_valid <- metadata_us[!is.na(metadata_us[[j]]), , drop = F]
    asv_valid <- ASV_table_us[row.names(ASV_table_us) %in% row.names(data_valid), , drop = F]
    result <- cor.test(asv_valid[[i]], data_valid[[j]], method = "spearman")
    result2 <- c(i,j,result$p.value, result$estimate)
    cor_matrix_us <- rbind(cor_matrix_us, result2)
  }
}
```

```
## Warning in cor.test.default(asv_valid[[i]], data_valid[[j]], method =
## "spearman"): Cannot compute exact p-value with ties

## Warning in cor.test.default(asv_valid[[i]], data_valid[[j]], method =
## "spearman"): Cannot compute exact p-value with ties

## Warning in cor.test.default(asv_valid[[i]], data_valid[[j]], method =
## "spearman"): Cannot compute exact p-value with ties

## Warning in cor.test.default(asv_valid[[i]], data_valid[[j]], method =
## "spearman"): Cannot compute exact p-value with ties

## Warning in cor.test.default(asv_valid[[i]], data_valid[[j]], method =
## "spearman"): Cannot compute exact p-value with ties

## Warning in cor.test.default(asv_valid[[i]], data_valid[[j]], method =
## "spearman"): Cannot compute exact p-value with ties

## Warning in cor.test.default(asv_valid[[i]], data_valid[[j]], method =
## "spearman"): Cannot compute exact p-value with ties

## Warning in cor.test.default(asv_valid[[i]], data_valid[[j]], method =
## "spearman"): Cannot compute exact p-value with ties

## Warning in cor.test.default(asv_valid[[i]], data_valid[[j]], method =
## "spearman"): Cannot compute exact p-value with ties

## Warning in cor.test.default(asv_valid[[i]], data_valid[[j]], method =
## "spearman"): Cannot compute exact p-value with ties

## Warning in cor.test.default(asv_valid[[i]], data_valid[[j]], method =
## "spearman"): Cannot compute exact p-value with ties

## Warning in cor.test.default(asv_valid[[i]], data_valid[[j]], method =
## "spearman"): Cannot compute exact p-value with ties

## Warning in cor.test.default(asv_valid[[i]], data_valid[[j]], method =
## "spearman"): Cannot compute exact p-value with ties

## Warning in cor.test.default(asv_valid[[i]], data_valid[[j]], method =
## "spearman"): Cannot compute exact p-value with ties

## Warning in cor.test.default(asv_valid[[i]], data_valid[[j]], method =
## "spearman"): Cannot compute exact p-value with ties

## Warning in cor.test.default(asv_valid[[i]], data_valid[[j]], method =
## "spearman"): Cannot compute exact p-value with ties

## Warning in cor.test.default(asv_valid[[i]], data_valid[[j]], method =
## "spearman"): Cannot compute exact p-value with ties

## Warning in cor.test.default(asv_valid[[i]], data_valid[[j]], method =
## "spearman"): Cannot compute exact p-value with ties

## Warning in cor.test.default(asv_valid[[i]], data_valid[[j]], method =
## "spearman"): Cannot compute exact p-value with ties

## Warning in cor.test.default(asv_valid[[i]], data_valid[[j]], method =
## "spearman"): Cannot compute exact p-value with ties

## Warning in cor.test.default(asv_valid[[i]], data_valid[[j]], method =
## "spearman"): Cannot compute exact p-value with ties

## Warning in cor.test.default(asv_valid[[i]], data_valid[[j]], method =
## "spearman"): Cannot compute exact p-value with ties

## Warning in cor.test.default(asv_valid[[i]], data_valid[[j]], method =
## "spearman"): Cannot compute exact p-value with ties

## Warning in cor.test.default(asv_valid[[i]], data_valid[[j]], method =
## "spearman"): Cannot compute exact p-value with ties

## Warning in cor.test.default(asv_valid[[i]], data_valid[[j]], method =
## "spearman"): Cannot compute exact p-value with ties

## Warning in cor.test.default(asv_valid[[i]], data_valid[[j]], method =
## "spearman"): Cannot compute exact p-value with ties

## Warning in cor.test.default(asv_valid[[i]], data_valid[[j]], method =
## "spearman"): Cannot compute exact p-value with ties

## Warning in cor.test.default(asv_valid[[i]], data_valid[[j]], method =
## "spearman"): Cannot compute exact p-value with ties

## Warning in cor.test.default(asv_valid[[i]], data_valid[[j]], method =
## "spearman"): Cannot compute exact p-value with ties

## Warning in cor.test.default(asv_valid[[i]], data_valid[[j]], method =
## "spearman"): Cannot compute exact p-value with ties

## Warning in cor.test.default(asv_valid[[i]], data_valid[[j]], method =
## "spearman"): Cannot compute exact p-value with ties

## Warning in cor.test.default(asv_valid[[i]], data_valid[[j]], method =
## "spearman"): Cannot compute exact p-value with ties

## Warning in cor.test.default(asv_valid[[i]], data_valid[[j]], method =
## "spearman"): Cannot compute exact p-value with ties

## Warning in cor.test.default(asv_valid[[i]], data_valid[[j]], method =
## "spearman"): Cannot compute exact p-value with ties

## Warning in cor.test.default(asv_valid[[i]], data_valid[[j]], method =
## "spearman"): Cannot compute exact p-value with ties

## Warning in cor.test.default(asv_valid[[i]], data_valid[[j]], method =
## "spearman"): Cannot compute exact p-value with ties

## Warning in cor.test.default(asv_valid[[i]], data_valid[[j]], method =
## "spearman"): Cannot compute exact p-value with ties

## Warning in cor.test.default(asv_valid[[i]], data_valid[[j]], method =
## "spearman"): Cannot compute exact p-value with ties

## Warning in cor.test.default(asv_valid[[i]], data_valid[[j]], method =
## "spearman"): Cannot compute exact p-value with ties

## Warning in cor.test.default(asv_valid[[i]], data_valid[[j]], method =
## "spearman"): Cannot compute exact p-value with ties

## Warning in cor.test.default(asv_valid[[i]], data_valid[[j]], method =
## "spearman"): Cannot compute exact p-value with ties

## Warning in cor.test.default(asv_valid[[i]], data_valid[[j]], method =
## "spearman"): Cannot compute exact p-value with ties

## Warning in cor.test.default(asv_valid[[i]], data_valid[[j]], method =
## "spearman"): Cannot compute exact p-value with ties

## Warning in cor.test.default(asv_valid[[i]], data_valid[[j]], method =
## "spearman"): Cannot compute exact p-value with ties

## Warning in cor.test.default(asv_valid[[i]], data_valid[[j]], method =
## "spearman"): Cannot compute exact p-value with ties

## Warning in cor.test.default(asv_valid[[i]], data_valid[[j]], method =
## "spearman"): Cannot compute exact p-value with ties

## Warning in cor.test.default(asv_valid[[i]], data_valid[[j]], method =
## "spearman"): Cannot compute exact p-value with ties

## Warning in cor.test.default(asv_valid[[i]], data_valid[[j]], method =
## "spearman"): Cannot compute exact p-value with ties

## Warning in cor.test.default(asv_valid[[i]], data_valid[[j]], method =
## "spearman"): Cannot compute exact p-value with ties

## Warning in cor.test.default(asv_valid[[i]], data_valid[[j]], method =
## "spearman"): Cannot compute exact p-value with ties

## Warning in cor.test.default(asv_valid[[i]], data_valid[[j]], method =
## "spearman"): Cannot compute exact p-value with ties

## Warning in cor.test.default(asv_valid[[i]], data_valid[[j]], method =
## "spearman"): Cannot compute exact p-value with ties

## Warning in cor.test.default(asv_valid[[i]], data_valid[[j]], method =
## "spearman"): Cannot compute exact p-value with ties

## Warning in cor.test.default(asv_valid[[i]], data_valid[[j]], method =
## "spearman"): Cannot compute exact p-value with ties

## Warning in cor.test.default(asv_valid[[i]], data_valid[[j]], method =
## "spearman"): Cannot compute exact p-value with ties

## Warning in cor.test.default(asv_valid[[i]], data_valid[[j]], method =
## "spearman"): Cannot compute exact p-value with ties

## Warning in cor.test.default(asv_valid[[i]], data_valid[[j]], method =
## "spearman"): Cannot compute exact p-value with ties

## Warning in cor.test.default(asv_valid[[i]], data_valid[[j]], method =
## "spearman"): Cannot compute exact p-value with ties

## Warning in cor.test.default(asv_valid[[i]], data_valid[[j]], method =
## "spearman"): Cannot compute exact p-value with ties

## Warning in cor.test.default(asv_valid[[i]], data_valid[[j]], method =
## "spearman"): Cannot compute exact p-value with ties

## Warning in cor.test.default(asv_valid[[i]], data_valid[[j]], method =
## "spearman"): Cannot compute exact p-value with ties

## Warning in cor.test.default(asv_valid[[i]], data_valid[[j]], method =
## "spearman"): Cannot compute exact p-value with ties

## Warning in cor.test.default(asv_valid[[i]], data_valid[[j]], method =
## "spearman"): Cannot compute exact p-value with ties

## Warning in cor.test.default(asv_valid[[i]], data_valid[[j]], method =
## "spearman"): Cannot compute exact p-value with ties

## Warning in cor.test.default(asv_valid[[i]], data_valid[[j]], method =
## "spearman"): Cannot compute exact p-value with ties

## Warning in cor.test.default(asv_valid[[i]], data_valid[[j]], method =
## "spearman"): Cannot compute exact p-value with ties

## Warning in cor.test.default(asv_valid[[i]], data_valid[[j]], method =
## "spearman"): Cannot compute exact p-value with ties

## Warning in cor.test.default(asv_valid[[i]], data_valid[[j]], method =
## "spearman"): Cannot compute exact p-value with ties

## Warning in cor.test.default(asv_valid[[i]], data_valid[[j]], method =
## "spearman"): Cannot compute exact p-value with ties

## Warning in cor.test.default(asv_valid[[i]], data_valid[[j]], method =
## "spearman"): Cannot compute exact p-value with ties

## Warning in cor.test.default(asv_valid[[i]], data_valid[[j]], method =
## "spearman"): Cannot compute exact p-value with ties

## Warning in cor.test.default(asv_valid[[i]], data_valid[[j]], method =
## "spearman"): Cannot compute exact p-value with ties

## Warning in cor.test.default(asv_valid[[i]], data_valid[[j]], method =
## "spearman"): Cannot compute exact p-value with ties

## Warning in cor.test.default(asv_valid[[i]], data_valid[[j]], method =
## "spearman"): Cannot compute exact p-value with ties

## Warning in cor.test.default(asv_valid[[i]], data_valid[[j]], method =
## "spearman"): Cannot compute exact p-value with ties

## Warning in cor.test.default(asv_valid[[i]], data_valid[[j]], method =
## "spearman"): Cannot compute exact p-value with ties

## Warning in cor.test.default(asv_valid[[i]], data_valid[[j]], method =
## "spearman"): Cannot compute exact p-value with ties

## Warning in cor.test.default(asv_valid[[i]], data_valid[[j]], method =
## "spearman"): Cannot compute exact p-value with ties

## Warning in cor.test.default(asv_valid[[i]], data_valid[[j]], method =
## "spearman"): Cannot compute exact p-value with ties

## Warning in cor.test.default(asv_valid[[i]], data_valid[[j]], method =
## "spearman"): Cannot compute exact p-value with ties

## Warning in cor.test.default(asv_valid[[i]], data_valid[[j]], method =
## "spearman"): Cannot compute exact p-value with ties

## Warning in cor.test.default(asv_valid[[i]], data_valid[[j]], method =
## "spearman"): Cannot compute exact p-value with ties

## Warning in cor.test.default(asv_valid[[i]], data_valid[[j]], method =
## "spearman"): Cannot compute exact p-value with ties

## Warning in cor.test.default(asv_valid[[i]], data_valid[[j]], method =
## "spearman"): Cannot compute exact p-value with ties

## Warning in cor.test.default(asv_valid[[i]], data_valid[[j]], method =
## "spearman"): Cannot compute exact p-value with ties

## Warning in cor.test.default(asv_valid[[i]], data_valid[[j]], method =
## "spearman"): Cannot compute exact p-value with ties

## Warning in cor.test.default(asv_valid[[i]], data_valid[[j]], method =
## "spearman"): Cannot compute exact p-value with ties

## Warning in cor.test.default(asv_valid[[i]], data_valid[[j]], method =
## "spearman"): Cannot compute exact p-value with ties

## Warning in cor.test.default(asv_valid[[i]], data_valid[[j]], method =
## "spearman"): Cannot compute exact p-value with ties

## Warning in cor.test.default(asv_valid[[i]], data_valid[[j]], method =
## "spearman"): Cannot compute exact p-value with ties

## Warning in cor.test.default(asv_valid[[i]], data_valid[[j]], method =
## "spearman"): Cannot compute exact p-value with ties

## Warning in cor.test.default(asv_valid[[i]], data_valid[[j]], method =
## "spearman"): Cannot compute exact p-value with ties

## Warning in cor.test.default(asv_valid[[i]], data_valid[[j]], method =
## "spearman"): Cannot compute exact p-value with ties

## Warning in cor.test.default(asv_valid[[i]], data_valid[[j]], method =
## "spearman"): Cannot compute exact p-value with ties

## Warning in cor.test.default(asv_valid[[i]], data_valid[[j]], method =
## "spearman"): Cannot compute exact p-value with ties

## Warning in cor.test.default(asv_valid[[i]], data_valid[[j]], method =
## "spearman"): Cannot compute exact p-value with ties

## Warning in cor.test.default(asv_valid[[i]], data_valid[[j]], method =
## "spearman"): Cannot compute exact p-value with ties

## Warning in cor.test.default(asv_valid[[i]], data_valid[[j]], method =
## "spearman"): Cannot compute exact p-value with ties

## Warning in cor.test.default(asv_valid[[i]], data_valid[[j]], method =
## "spearman"): Cannot compute exact p-value with ties

## Warning in cor.test.default(asv_valid[[i]], data_valid[[j]], method =
## "spearman"): Cannot compute exact p-value with ties

## Warning in cor.test.default(asv_valid[[i]], data_valid[[j]], method =
## "spearman"): Cannot compute exact p-value with ties

## Warning in cor.test.default(asv_valid[[i]], data_valid[[j]], method =
## "spearman"): Cannot compute exact p-value with ties

## Warning in cor.test.default(asv_valid[[i]], data_valid[[j]], method =
## "spearman"): Cannot compute exact p-value with ties

## Warning in cor.test.default(asv_valid[[i]], data_valid[[j]], method =
## "spearman"): Cannot compute exact p-value with ties

## Warning in cor.test.default(asv_valid[[i]], data_valid[[j]], method =
## "spearman"): Cannot compute exact p-value with ties

## Warning in cor.test.default(asv_valid[[i]], data_valid[[j]], method =
## "spearman"): Cannot compute exact p-value with ties
```

```
cor_matrix_us <- as.data.frame(cor_matrix_us)
colnames(cor_matrix_us) <- c("ASV", "Marker", "Pvalue", "Rho")
cor_matrix_us$Pvalue <- as.numeric(as.character(cor_matrix_us$Pvalue))
cor_matrix_us$Rho <- as.numeric(as.character(cor_matrix_us$Rho))
cor_matrix_us_valid <- cor_matrix_us[!is.na(cor_matrix_us$Pvalue), , drop = F]
cor_matrix_us_valid$Marker <- factor(cor_matrix_us_valid$Marker, levels = c("scd14_ng_ml_uvm", "ifabp_pg_ml_uvm"))
cor_matrix_us_valid$Cohort <- "us"
cor_matrix_us_valid[cor_matrix_us_valid$Marker == "scd14_ng_ml_uvm", "Padj"] <- p.adjust(cor_matrix_us_valid[cor_matrix_us_valid$Marker == "scd14_ng_ml_uvm", "Pvalue"], method = "fdr")
cor_matrix_us_valid[cor_matrix_us_valid$Marker == "ifabp_pg_ml_uvm", "Padj"] <- p.adjust(cor_matrix_us_valid[cor_matrix_us_valid$Marker == "ifabp_pg_ml_uvm", "Pvalue"], method = "fdr")
cor_matrix_us_valid$Sig <- cor_matrix_us_valid$Padj
cor_matrix_us_valid$Sig[cor_matrix_us_valid$Sig <= 0.05] <- "Yes"
cor_matrix_us_valid$Sig[cor_matrix_us_valid$Sig != "Yes"] <- "No"


#BOTSWANA
metadata_botswana <- metadata[metadata$sample_cohort == "botswana", , drop = F]
TAX_table_botswana <- TAX_table[row.names(TAX_table) %in% botswana$taxon, , drop = F]
ASV_table_botswana <- ASV_table[, colnames(ASV_table) %in% botswana$taxon, drop = F]

cor_matrix_botswana <- c()
for (i in colnames(ASV_table_botswana)){
  for (j in c("scd14_ng_ml_uvm", "ifabp_pg_ml_uvm")){
    data_valid <- metadata_botswana[!is.na(metadata_botswana[[j]]), , drop = F]
    asv_valid <- ASV_table_botswana[row.names(ASV_table_botswana) %in% row.names(data_valid), , drop = F]
    result <- cor.test(asv_valid[[i]], data_valid[[j]], method = "spearman")
    result2 <- c(i,j,result$p.value, result$estimate)
    cor_matrix_botswana <- rbind(cor_matrix_botswana, result2)
  }
}
```

```
## Warning in cor.test.default(asv_valid[[i]], data_valid[[j]], method =
## "spearman"): Cannot compute exact p-value with ties

## Warning in cor.test.default(asv_valid[[i]], data_valid[[j]], method =
## "spearman"): Cannot compute exact p-value with ties

## Warning in cor.test.default(asv_valid[[i]], data_valid[[j]], method =
## "spearman"): Cannot compute exact p-value with ties

## Warning in cor.test.default(asv_valid[[i]], data_valid[[j]], method =
## "spearman"): Cannot compute exact p-value with ties

## Warning in cor.test.default(asv_valid[[i]], data_valid[[j]], method =
## "spearman"): Cannot compute exact p-value with ties

## Warning in cor.test.default(asv_valid[[i]], data_valid[[j]], method =
## "spearman"): Cannot compute exact p-value with ties

## Warning in cor.test.default(asv_valid[[i]], data_valid[[j]], method =
## "spearman"): Cannot compute exact p-value with ties

## Warning in cor.test.default(asv_valid[[i]], data_valid[[j]], method =
## "spearman"): Cannot compute exact p-value with ties

## Warning in cor.test.default(asv_valid[[i]], data_valid[[j]], method =
## "spearman"): Cannot compute exact p-value with ties

## Warning in cor.test.default(asv_valid[[i]], data_valid[[j]], method =
## "spearman"): Cannot compute exact p-value with ties

## Warning in cor.test.default(asv_valid[[i]], data_valid[[j]], method =
## "spearman"): Cannot compute exact p-value with ties

## Warning in cor.test.default(asv_valid[[i]], data_valid[[j]], method =
## "spearman"): Cannot compute exact p-value with ties

## Warning in cor.test.default(asv_valid[[i]], data_valid[[j]], method =
## "spearman"): Cannot compute exact p-value with ties

## Warning in cor.test.default(asv_valid[[i]], data_valid[[j]], method =
## "spearman"): Cannot compute exact p-value with ties

## Warning in cor.test.default(asv_valid[[i]], data_valid[[j]], method =
## "spearman"): Cannot compute exact p-value with ties

## Warning in cor.test.default(asv_valid[[i]], data_valid[[j]], method =
## "spearman"): Cannot compute exact p-value with ties

## Warning in cor.test.default(asv_valid[[i]], data_valid[[j]], method =
## "spearman"): Cannot compute exact p-value with ties

## Warning in cor.test.default(asv_valid[[i]], data_valid[[j]], method =
## "spearman"): Cannot compute exact p-value with ties

## Warning in cor.test.default(asv_valid[[i]], data_valid[[j]], method =
## "spearman"): Cannot compute exact p-value with ties

## Warning in cor.test.default(asv_valid[[i]], data_valid[[j]], method =
## "spearman"): Cannot compute exact p-value with ties

## Warning in cor.test.default(asv_valid[[i]], data_valid[[j]], method =
## "spearman"): Cannot compute exact p-value with ties

## Warning in cor.test.default(asv_valid[[i]], data_valid[[j]], method =
## "spearman"): Cannot compute exact p-value with ties

## Warning in cor.test.default(asv_valid[[i]], data_valid[[j]], method =
## "spearman"): Cannot compute exact p-value with ties

## Warning in cor.test.default(asv_valid[[i]], data_valid[[j]], method =
## "spearman"): Cannot compute exact p-value with ties

## Warning in cor.test.default(asv_valid[[i]], data_valid[[j]], method =
## "spearman"): Cannot compute exact p-value with ties

## Warning in cor.test.default(asv_valid[[i]], data_valid[[j]], method =
## "spearman"): Cannot compute exact p-value with ties

## Warning in cor.test.default(asv_valid[[i]], data_valid[[j]], method =
## "spearman"): Cannot compute exact p-value with ties

## Warning in cor.test.default(asv_valid[[i]], data_valid[[j]], method =
## "spearman"): Cannot compute exact p-value with ties

## Warning in cor.test.default(asv_valid[[i]], data_valid[[j]], method =
## "spearman"): Cannot compute exact p-value with ties

## Warning in cor.test.default(asv_valid[[i]], data_valid[[j]], method =
## "spearman"): Cannot compute exact p-value with ties

## Warning in cor.test.default(asv_valid[[i]], data_valid[[j]], method =
## "spearman"): Cannot compute exact p-value with ties

## Warning in cor.test.default(asv_valid[[i]], data_valid[[j]], method =
## "spearman"): Cannot compute exact p-value with ties

## Warning in cor.test.default(asv_valid[[i]], data_valid[[j]], method =
## "spearman"): Cannot compute exact p-value with ties

## Warning in cor.test.default(asv_valid[[i]], data_valid[[j]], method =
## "spearman"): Cannot compute exact p-value with ties

## Warning in cor.test.default(asv_valid[[i]], data_valid[[j]], method =
## "spearman"): Cannot compute exact p-value with ties

## Warning in cor.test.default(asv_valid[[i]], data_valid[[j]], method =
## "spearman"): Cannot compute exact p-value with ties

## Warning in cor.test.default(asv_valid[[i]], data_valid[[j]], method =
## "spearman"): Cannot compute exact p-value with ties

## Warning in cor.test.default(asv_valid[[i]], data_valid[[j]], method =
## "spearman"): Cannot compute exact p-value with ties

## Warning in cor.test.default(asv_valid[[i]], data_valid[[j]], method =
## "spearman"): Cannot compute exact p-value with ties

## Warning in cor.test.default(asv_valid[[i]], data_valid[[j]], method =
## "spearman"): Cannot compute exact p-value with ties

## Warning in cor.test.default(asv_valid[[i]], data_valid[[j]], method =
## "spearman"): Cannot compute exact p-value with ties

## Warning in cor.test.default(asv_valid[[i]], data_valid[[j]], method =
## "spearman"): Cannot compute exact p-value with ties

## Warning in cor.test.default(asv_valid[[i]], data_valid[[j]], method =
## "spearman"): Cannot compute exact p-value with ties

## Warning in cor.test.default(asv_valid[[i]], data_valid[[j]], method =
## "spearman"): Cannot compute exact p-value with ties

## Warning in cor.test.default(asv_valid[[i]], data_valid[[j]], method =
## "spearman"): Cannot compute exact p-value with ties

## Warning in cor.test.default(asv_valid[[i]], data_valid[[j]], method =
## "spearman"): Cannot compute exact p-value with ties

## Warning in cor.test.default(asv_valid[[i]], data_valid[[j]], method =
## "spearman"): Cannot compute exact p-value with ties

## Warning in cor.test.default(asv_valid[[i]], data_valid[[j]], method =
## "spearman"): Cannot compute exact p-value with ties

## Warning in cor.test.default(asv_valid[[i]], data_valid[[j]], method =
## "spearman"): Cannot compute exact p-value with ties

## Warning in cor.test.default(asv_valid[[i]], data_valid[[j]], method =
## "spearman"): Cannot compute exact p-value with ties

## Warning in cor.test.default(asv_valid[[i]], data_valid[[j]], method =
## "spearman"): Cannot compute exact p-value with ties

## Warning in cor.test.default(asv_valid[[i]], data_valid[[j]], method =
## "spearman"): Cannot compute exact p-value with ties
```

```
cor_matrix_botswana <- as.data.frame(cor_matrix_botswana)
colnames(cor_matrix_botswana) <- c("ASV", "Marker", "Pvalue", "Rho")
cor_matrix_botswana$Pvalue <- as.numeric(as.character(cor_matrix_botswana$Pvalue))
cor_matrix_botswana$Rho <- as.numeric(as.character(cor_matrix_botswana$Rho))
cor_matrix_botswana_valid <- cor_matrix_botswana[!is.na(cor_matrix_botswana$Pvalue), , drop = F]
cor_matrix_botswana_valid$Marker <- factor(cor_matrix_botswana_valid$Marker, levels = c("scd14_ng_ml_uvm","ifabp_pg_ml_uvm"))
cor_matrix_botswana_valid$Cohort <- "botswana"
cor_matrix_botswana_valid[cor_matrix_botswana_valid$Marker == "scd14_ng_ml_uvm", "Padj"] <- p.adjust(cor_matrix_botswana_valid[cor_matrix_botswana_valid$Marker == "scd14_ng_ml_uvm", "Pvalue"], method = "fdr")
cor_matrix_botswana_valid[cor_matrix_botswana_valid$Marker == "ifabp_pg_ml_uvm", "Padj"] <- p.adjust(cor_matrix_botswana_valid[cor_matrix_botswana_valid$Marker == "ifabp_pg_ml_uvm", "Pvalue"], method = "fdr")
cor_matrix_botswana_valid$Sig <- cor_matrix_botswana_valid$Padj
cor_matrix_botswana_valid$Sig[cor_matrix_botswana_valid$Sig <= 0.05] <- "Yes"
cor_matrix_botswana_valid$Sig[cor_matrix_botswana_valid$Sig != "Yes"] <- "No"


#UGANDA
metadata_uganda <- metadata[metadata$sample_cohort == "uganda_2", , drop = F]
TAX_table_uganda <- TAX_table[row.names(TAX_table) %in% uganda$taxon, , drop = F]
ASV_table_uganda <- ASV_table[, colnames(ASV_table) %in% uganda$taxon, drop = F]

cor_matrix_uganda <- c()
for (i in colnames(ASV_table_uganda)){
  for (j in c("scd14_ng_ml_uvm", "ifabp_pg_ml_uvm")){
    data_valid <- metadata_uganda[!is.na(metadata_uganda[[j]]), , drop = F]
    asv_valid <- ASV_table_uganda[row.names(ASV_table_uganda) %in% row.names(data_valid), , drop = F]
    result <- cor.test(asv_valid[[i]], data_valid[[j]], method = "spearman")
    result2 <- c(i,j,result$p.value, result$estimate)
    cor_matrix_uganda <- rbind(cor_matrix_uganda, result2)
  }
}
```

```
## Warning in cor.test.default(asv_valid[[i]], data_valid[[j]], method =
## "spearman"): Cannot compute exact p-value with ties

## Warning in cor.test.default(asv_valid[[i]], data_valid[[j]], method =
## "spearman"): Cannot compute exact p-value with ties

## Warning in cor.test.default(asv_valid[[i]], data_valid[[j]], method =
## "spearman"): Cannot compute exact p-value with ties

## Warning in cor.test.default(asv_valid[[i]], data_valid[[j]], method =
## "spearman"): Cannot compute exact p-value with ties

## Warning in cor.test.default(asv_valid[[i]], data_valid[[j]], method =
## "spearman"): Cannot compute exact p-value with ties

## Warning in cor.test.default(asv_valid[[i]], data_valid[[j]], method =
## "spearman"): Cannot compute exact p-value with ties

## Warning in cor.test.default(asv_valid[[i]], data_valid[[j]], method =
## "spearman"): Cannot compute exact p-value with ties

## Warning in cor.test.default(asv_valid[[i]], data_valid[[j]], method =
## "spearman"): Cannot compute exact p-value with ties

## Warning in cor.test.default(asv_valid[[i]], data_valid[[j]], method =
## "spearman"): Cannot compute exact p-value with ties

## Warning in cor.test.default(asv_valid[[i]], data_valid[[j]], method =
## "spearman"): Cannot compute exact p-value with ties

## Warning in cor.test.default(asv_valid[[i]], data_valid[[j]], method =
## "spearman"): Cannot compute exact p-value with ties

## Warning in cor.test.default(asv_valid[[i]], data_valid[[j]], method =
## "spearman"): Cannot compute exact p-value with ties

## Warning in cor.test.default(asv_valid[[i]], data_valid[[j]], method =
## "spearman"): Cannot compute exact p-value with ties

## Warning in cor.test.default(asv_valid[[i]], data_valid[[j]], method =
## "spearman"): Cannot compute exact p-value with ties

## Warning in cor.test.default(asv_valid[[i]], data_valid[[j]], method =
## "spearman"): Cannot compute exact p-value with ties

## Warning in cor.test.default(asv_valid[[i]], data_valid[[j]], method =
## "spearman"): Cannot compute exact p-value with ties

## Warning in cor.test.default(asv_valid[[i]], data_valid[[j]], method =
## "spearman"): Cannot compute exact p-value with ties

## Warning in cor.test.default(asv_valid[[i]], data_valid[[j]], method =
## "spearman"): Cannot compute exact p-value with ties

## Warning in cor.test.default(asv_valid[[i]], data_valid[[j]], method =
## "spearman"): Cannot compute exact p-value with ties

## Warning in cor.test.default(asv_valid[[i]], data_valid[[j]], method =
## "spearman"): Cannot compute exact p-value with ties

## Warning in cor.test.default(asv_valid[[i]], data_valid[[j]], method =
## "spearman"): Cannot compute exact p-value with ties

## Warning in cor.test.default(asv_valid[[i]], data_valid[[j]], method =
## "spearman"): Cannot compute exact p-value with ties

## Warning in cor.test.default(asv_valid[[i]], data_valid[[j]], method =
## "spearman"): Cannot compute exact p-value with ties

## Warning in cor.test.default(asv_valid[[i]], data_valid[[j]], method =
## "spearman"): Cannot compute exact p-value with ties

## Warning in cor.test.default(asv_valid[[i]], data_valid[[j]], method =
## "spearman"): Cannot compute exact p-value with ties

## Warning in cor.test.default(asv_valid[[i]], data_valid[[j]], method =
## "spearman"): Cannot compute exact p-value with ties

## Warning in cor.test.default(asv_valid[[i]], data_valid[[j]], method =
## "spearman"): Cannot compute exact p-value with ties

## Warning in cor.test.default(asv_valid[[i]], data_valid[[j]], method =
## "spearman"): Cannot compute exact p-value with ties

## Warning in cor.test.default(asv_valid[[i]], data_valid[[j]], method =
## "spearman"): Cannot compute exact p-value with ties

## Warning in cor.test.default(asv_valid[[i]], data_valid[[j]], method =
## "spearman"): Cannot compute exact p-value with ties

## Warning in cor.test.default(asv_valid[[i]], data_valid[[j]], method =
## "spearman"): Cannot compute exact p-value with ties

## Warning in cor.test.default(asv_valid[[i]], data_valid[[j]], method =
## "spearman"): Cannot compute exact p-value with ties

## Warning in cor.test.default(asv_valid[[i]], data_valid[[j]], method =
## "spearman"): Cannot compute exact p-value with ties

## Warning in cor.test.default(asv_valid[[i]], data_valid[[j]], method =
## "spearman"): Cannot compute exact p-value with ties

## Warning in cor.test.default(asv_valid[[i]], data_valid[[j]], method =
## "spearman"): Cannot compute exact p-value with ties

## Warning in cor.test.default(asv_valid[[i]], data_valid[[j]], method =
## "spearman"): Cannot compute exact p-value with ties

## Warning in cor.test.default(asv_valid[[i]], data_valid[[j]], method =
## "spearman"): Cannot compute exact p-value with ties

## Warning in cor.test.default(asv_valid[[i]], data_valid[[j]], method =
## "spearman"): Cannot compute exact p-value with ties

## Warning in cor.test.default(asv_valid[[i]], data_valid[[j]], method =
## "spearman"): Cannot compute exact p-value with ties

## Warning in cor.test.default(asv_valid[[i]], data_valid[[j]], method =
## "spearman"): Cannot compute exact p-value with ties

## Warning in cor.test.default(asv_valid[[i]], data_valid[[j]], method =
## "spearman"): Cannot compute exact p-value with ties

## Warning in cor.test.default(asv_valid[[i]], data_valid[[j]], method =
## "spearman"): Cannot compute exact p-value with ties

## Warning in cor.test.default(asv_valid[[i]], data_valid[[j]], method =
## "spearman"): Cannot compute exact p-value with ties

## Warning in cor.test.default(asv_valid[[i]], data_valid[[j]], method =
## "spearman"): Cannot compute exact p-value with ties

## Warning in cor.test.default(asv_valid[[i]], data_valid[[j]], method =
## "spearman"): Cannot compute exact p-value with ties

## Warning in cor.test.default(asv_valid[[i]], data_valid[[j]], method =
## "spearman"): Cannot compute exact p-value with ties

## Warning in cor.test.default(asv_valid[[i]], data_valid[[j]], method =
## "spearman"): Cannot compute exact p-value with ties

## Warning in cor.test.default(asv_valid[[i]], data_valid[[j]], method =
## "spearman"): Cannot compute exact p-value with ties

## Warning in cor.test.default(asv_valid[[i]], data_valid[[j]], method =
## "spearman"): Cannot compute exact p-value with ties

## Warning in cor.test.default(asv_valid[[i]], data_valid[[j]], method =
## "spearman"): Cannot compute exact p-value with ties

## Warning in cor.test.default(asv_valid[[i]], data_valid[[j]], method =
## "spearman"): Cannot compute exact p-value with ties

## Warning in cor.test.default(asv_valid[[i]], data_valid[[j]], method =
## "spearman"): Cannot compute exact p-value with ties

## Warning in cor.test.default(asv_valid[[i]], data_valid[[j]], method =
## "spearman"): Cannot compute exact p-value with ties

## Warning in cor.test.default(asv_valid[[i]], data_valid[[j]], method =
## "spearman"): Cannot compute exact p-value with ties

## Warning in cor.test.default(asv_valid[[i]], data_valid[[j]], method =
## "spearman"): Cannot compute exact p-value with ties

## Warning in cor.test.default(asv_valid[[i]], data_valid[[j]], method =
## "spearman"): Cannot compute exact p-value with ties

## Warning in cor.test.default(asv_valid[[i]], data_valid[[j]], method =
## "spearman"): Cannot compute exact p-value with ties

## Warning in cor.test.default(asv_valid[[i]], data_valid[[j]], method =
## "spearman"): Cannot compute exact p-value with ties

## Warning in cor.test.default(asv_valid[[i]], data_valid[[j]], method =
## "spearman"): Cannot compute exact p-value with ties

## Warning in cor.test.default(asv_valid[[i]], data_valid[[j]], method =
## "spearman"): Cannot compute exact p-value with ties

## Warning in cor.test.default(asv_valid[[i]], data_valid[[j]], method =
## "spearman"): Cannot compute exact p-value with ties

## Warning in cor.test.default(asv_valid[[i]], data_valid[[j]], method =
## "spearman"): Cannot compute exact p-value with ties

## Warning in cor.test.default(asv_valid[[i]], data_valid[[j]], method =
## "spearman"): Cannot compute exact p-value with ties

## Warning in cor.test.default(asv_valid[[i]], data_valid[[j]], method =
## "spearman"): Cannot compute exact p-value with ties

## Warning in cor.test.default(asv_valid[[i]], data_valid[[j]], method =
## "spearman"): Cannot compute exact p-value with ties

## Warning in cor.test.default(asv_valid[[i]], data_valid[[j]], method =
## "spearman"): Cannot compute exact p-value with ties

## Warning in cor.test.default(asv_valid[[i]], data_valid[[j]], method =
## "spearman"): Cannot compute exact p-value with ties

## Warning in cor.test.default(asv_valid[[i]], data_valid[[j]], method =
## "spearman"): Cannot compute exact p-value with ties

## Warning in cor.test.default(asv_valid[[i]], data_valid[[j]], method =
## "spearman"): Cannot compute exact p-value with ties

## Warning in cor.test.default(asv_valid[[i]], data_valid[[j]], method =
## "spearman"): Cannot compute exact p-value with ties

## Warning in cor.test.default(asv_valid[[i]], data_valid[[j]], method =
## "spearman"): Cannot compute exact p-value with ties

## Warning in cor.test.default(asv_valid[[i]], data_valid[[j]], method =
## "spearman"): Cannot compute exact p-value with ties

## Warning in cor.test.default(asv_valid[[i]], data_valid[[j]], method =
## "spearman"): Cannot compute exact p-value with ties

## Warning in cor.test.default(asv_valid[[i]], data_valid[[j]], method =
## "spearman"): Cannot compute exact p-value with ties

## Warning in cor.test.default(asv_valid[[i]], data_valid[[j]], method =
## "spearman"): Cannot compute exact p-value with ties

## Warning in cor.test.default(asv_valid[[i]], data_valid[[j]], method =
## "spearman"): Cannot compute exact p-value with ties

## Warning in cor.test.default(asv_valid[[i]], data_valid[[j]], method =
## "spearman"): Cannot compute exact p-value with ties

## Warning in cor.test.default(asv_valid[[i]], data_valid[[j]], method =
## "spearman"): Cannot compute exact p-value with ties

## Warning in cor.test.default(asv_valid[[i]], data_valid[[j]], method =
## "spearman"): Cannot compute exact p-value with ties

## Warning in cor.test.default(asv_valid[[i]], data_valid[[j]], method =
## "spearman"): Cannot compute exact p-value with ties

## Warning in cor.test.default(asv_valid[[i]], data_valid[[j]], method =
## "spearman"): Cannot compute exact p-value with ties

## Warning in cor.test.default(asv_valid[[i]], data_valid[[j]], method =
## "spearman"): Cannot compute exact p-value with ties

## Warning in cor.test.default(asv_valid[[i]], data_valid[[j]], method =
## "spearman"): Cannot compute exact p-value with ties

## Warning in cor.test.default(asv_valid[[i]], data_valid[[j]], method =
## "spearman"): Cannot compute exact p-value with ties

## Warning in cor.test.default(asv_valid[[i]], data_valid[[j]], method =
## "spearman"): Cannot compute exact p-value with ties

## Warning in cor.test.default(asv_valid[[i]], data_valid[[j]], method =
## "spearman"): Cannot compute exact p-value with ties

## Warning in cor.test.default(asv_valid[[i]], data_valid[[j]], method =
## "spearman"): Cannot compute exact p-value with ties

## Warning in cor.test.default(asv_valid[[i]], data_valid[[j]], method =
## "spearman"): Cannot compute exact p-value with ties

## Warning in cor.test.default(asv_valid[[i]], data_valid[[j]], method =
## "spearman"): Cannot compute exact p-value with ties

## Warning in cor.test.default(asv_valid[[i]], data_valid[[j]], method =
## "spearman"): Cannot compute exact p-value with ties

## Warning in cor.test.default(asv_valid[[i]], data_valid[[j]], method =
## "spearman"): Cannot compute exact p-value with ties

## Warning in cor.test.default(asv_valid[[i]], data_valid[[j]], method =
## "spearman"): Cannot compute exact p-value with ties

## Warning in cor.test.default(asv_valid[[i]], data_valid[[j]], method =
## "spearman"): Cannot compute exact p-value with ties

## Warning in cor.test.default(asv_valid[[i]], data_valid[[j]], method =
## "spearman"): Cannot compute exact p-value with ties

## Warning in cor.test.default(asv_valid[[i]], data_valid[[j]], method =
## "spearman"): Cannot compute exact p-value with ties

## Warning in cor.test.default(asv_valid[[i]], data_valid[[j]], method =
## "spearman"): Cannot compute exact p-value with ties

## Warning in cor.test.default(asv_valid[[i]], data_valid[[j]], method =
## "spearman"): Cannot compute exact p-value with ties

## Warning in cor.test.default(asv_valid[[i]], data_valid[[j]], method =
## "spearman"): Cannot compute exact p-value with ties

## Warning in cor.test.default(asv_valid[[i]], data_valid[[j]], method =
## "spearman"): Cannot compute exact p-value with ties

## Warning in cor.test.default(asv_valid[[i]], data_valid[[j]], method =
## "spearman"): Cannot compute exact p-value with ties

## Warning in cor.test.default(asv_valid[[i]], data_valid[[j]], method =
## "spearman"): Cannot compute exact p-value with ties

## Warning in cor.test.default(asv_valid[[i]], data_valid[[j]], method =
## "spearman"): Cannot compute exact p-value with ties

## Warning in cor.test.default(asv_valid[[i]], data_valid[[j]], method =
## "spearman"): Cannot compute exact p-value with ties

## Warning in cor.test.default(asv_valid[[i]], data_valid[[j]], method =
## "spearman"): Cannot compute exact p-value with ties

## Warning in cor.test.default(asv_valid[[i]], data_valid[[j]], method =
## "spearman"): Cannot compute exact p-value with ties

## Warning in cor.test.default(asv_valid[[i]], data_valid[[j]], method =
## "spearman"): Cannot compute exact p-value with ties

## Warning in cor.test.default(asv_valid[[i]], data_valid[[j]], method =
## "spearman"): Cannot compute exact p-value with ties

## Warning in cor.test.default(asv_valid[[i]], data_valid[[j]], method =
## "spearman"): Cannot compute exact p-value with ties

## Warning in cor.test.default(asv_valid[[i]], data_valid[[j]], method =
## "spearman"): Cannot compute exact p-value with ties

## Warning in cor.test.default(asv_valid[[i]], data_valid[[j]], method =
## "spearman"): Cannot compute exact p-value with ties

## Warning in cor.test.default(asv_valid[[i]], data_valid[[j]], method =
## "spearman"): Cannot compute exact p-value with ties

## Warning in cor.test.default(asv_valid[[i]], data_valid[[j]], method =
## "spearman"): Cannot compute exact p-value with ties

## Warning in cor.test.default(asv_valid[[i]], data_valid[[j]], method =
## "spearman"): Cannot compute exact p-value with ties

## Warning in cor.test.default(asv_valid[[i]], data_valid[[j]], method =
## "spearman"): Cannot compute exact p-value with ties

## Warning in cor.test.default(asv_valid[[i]], data_valid[[j]], method =
## "spearman"): Cannot compute exact p-value with ties

## Warning in cor.test.default(asv_valid[[i]], data_valid[[j]], method =
## "spearman"): Cannot compute exact p-value with ties

## Warning in cor.test.default(asv_valid[[i]], data_valid[[j]], method =
## "spearman"): Cannot compute exact p-value with ties

## Warning in cor.test.default(asv_valid[[i]], data_valid[[j]], method =
## "spearman"): Cannot compute exact p-value with ties

## Warning in cor.test.default(asv_valid[[i]], data_valid[[j]], method =
## "spearman"): Cannot compute exact p-value with ties

## Warning in cor.test.default(asv_valid[[i]], data_valid[[j]], method =
## "spearman"): Cannot compute exact p-value with ties

## Warning in cor.test.default(asv_valid[[i]], data_valid[[j]], method =
## "spearman"): Cannot compute exact p-value with ties

## Warning in cor.test.default(asv_valid[[i]], data_valid[[j]], method =
## "spearman"): Cannot compute exact p-value with ties

## Warning in cor.test.default(asv_valid[[i]], data_valid[[j]], method =
## "spearman"): Cannot compute exact p-value with ties

## Warning in cor.test.default(asv_valid[[i]], data_valid[[j]], method =
## "spearman"): Cannot compute exact p-value with ties

## Warning in cor.test.default(asv_valid[[i]], data_valid[[j]], method =
## "spearman"): Cannot compute exact p-value with ties

## Warning in cor.test.default(asv_valid[[i]], data_valid[[j]], method =
## "spearman"): Cannot compute exact p-value with ties

## Warning in cor.test.default(asv_valid[[i]], data_valid[[j]], method =
## "spearman"): Cannot compute exact p-value with ties

## Warning in cor.test.default(asv_valid[[i]], data_valid[[j]], method =
## "spearman"): Cannot compute exact p-value with ties

## Warning in cor.test.default(asv_valid[[i]], data_valid[[j]], method =
## "spearman"): Cannot compute exact p-value with ties

## Warning in cor.test.default(asv_valid[[i]], data_valid[[j]], method =
## "spearman"): Cannot compute exact p-value with ties

## Warning in cor.test.default(asv_valid[[i]], data_valid[[j]], method =
## "spearman"): Cannot compute exact p-value with ties

## Warning in cor.test.default(asv_valid[[i]], data_valid[[j]], method =
## "spearman"): Cannot compute exact p-value with ties

## Warning in cor.test.default(asv_valid[[i]], data_valid[[j]], method =
## "spearman"): Cannot compute exact p-value with ties

## Warning in cor.test.default(asv_valid[[i]], data_valid[[j]], method =
## "spearman"): Cannot compute exact p-value with ties

## Warning in cor.test.default(asv_valid[[i]], data_valid[[j]], method =
## "spearman"): Cannot compute exact p-value with ties

## Warning in cor.test.default(asv_valid[[i]], data_valid[[j]], method =
## "spearman"): Cannot compute exact p-value with ties

## Warning in cor.test.default(asv_valid[[i]], data_valid[[j]], method =
## "spearman"): Cannot compute exact p-value with ties

## Warning in cor.test.default(asv_valid[[i]], data_valid[[j]], method =
## "spearman"): Cannot compute exact p-value with ties

## Warning in cor.test.default(asv_valid[[i]], data_valid[[j]], method =
## "spearman"): Cannot compute exact p-value with ties

## Warning in cor.test.default(asv_valid[[i]], data_valid[[j]], method =
## "spearman"): Cannot compute exact p-value with ties

## Warning in cor.test.default(asv_valid[[i]], data_valid[[j]], method =
## "spearman"): Cannot compute exact p-value with ties

## Warning in cor.test.default(asv_valid[[i]], data_valid[[j]], method =
## "spearman"): Cannot compute exact p-value with ties

## Warning in cor.test.default(asv_valid[[i]], data_valid[[j]], method =
## "spearman"): Cannot compute exact p-value with ties

## Warning in cor.test.default(asv_valid[[i]], data_valid[[j]], method =
## "spearman"): Cannot compute exact p-value with ties

## Warning in cor.test.default(asv_valid[[i]], data_valid[[j]], method =
## "spearman"): Cannot compute exact p-value with ties

## Warning in cor.test.default(asv_valid[[i]], data_valid[[j]], method =
## "spearman"): Cannot compute exact p-value with ties

## Warning in cor.test.default(asv_valid[[i]], data_valid[[j]], method =
## "spearman"): Cannot compute exact p-value with ties

## Warning in cor.test.default(asv_valid[[i]], data_valid[[j]], method =
## "spearman"): Cannot compute exact p-value with ties

## Warning in cor.test.default(asv_valid[[i]], data_valid[[j]], method =
## "spearman"): Cannot compute exact p-value with ties

## Warning in cor.test.default(asv_valid[[i]], data_valid[[j]], method =
## "spearman"): Cannot compute exact p-value with ties

## Warning in cor.test.default(asv_valid[[i]], data_valid[[j]], method =
## "spearman"): Cannot compute exact p-value with ties

## Warning in cor.test.default(asv_valid[[i]], data_valid[[j]], method =
## "spearman"): Cannot compute exact p-value with ties

## Warning in cor.test.default(asv_valid[[i]], data_valid[[j]], method =
## "spearman"): Cannot compute exact p-value with ties

## Warning in cor.test.default(asv_valid[[i]], data_valid[[j]], method =
## "spearman"): Cannot compute exact p-value with ties

## Warning in cor.test.default(asv_valid[[i]], data_valid[[j]], method =
## "spearman"): Cannot compute exact p-value with ties

## Warning in cor.test.default(asv_valid[[i]], data_valid[[j]], method =
## "spearman"): Cannot compute exact p-value with ties

## Warning in cor.test.default(asv_valid[[i]], data_valid[[j]], method =
## "spearman"): Cannot compute exact p-value with ties

## Warning in cor.test.default(asv_valid[[i]], data_valid[[j]], method =
## "spearman"): Cannot compute exact p-value with ties

## Warning in cor.test.default(asv_valid[[i]], data_valid[[j]], method =
## "spearman"): Cannot compute exact p-value with ties

## Warning in cor.test.default(asv_valid[[i]], data_valid[[j]], method =
## "spearman"): Cannot compute exact p-value with ties

## Warning in cor.test.default(asv_valid[[i]], data_valid[[j]], method =
## "spearman"): Cannot compute exact p-value with ties

## Warning in cor.test.default(asv_valid[[i]], data_valid[[j]], method =
## "spearman"): Cannot compute exact p-value with ties

## Warning in cor.test.default(asv_valid[[i]], data_valid[[j]], method =
## "spearman"): Cannot compute exact p-value with ties

## Warning in cor.test.default(asv_valid[[i]], data_valid[[j]], method =
## "spearman"): Cannot compute exact p-value with ties

## Warning in cor.test.default(asv_valid[[i]], data_valid[[j]], method =
## "spearman"): Cannot compute exact p-value with ties

## Warning in cor.test.default(asv_valid[[i]], data_valid[[j]], method =
## "spearman"): Cannot compute exact p-value with ties

## Warning in cor.test.default(asv_valid[[i]], data_valid[[j]], method =
## "spearman"): Cannot compute exact p-value with ties

## Warning in cor.test.default(asv_valid[[i]], data_valid[[j]], method =
## "spearman"): Cannot compute exact p-value with ties

## Warning in cor.test.default(asv_valid[[i]], data_valid[[j]], method =
## "spearman"): Cannot compute exact p-value with ties

## Warning in cor.test.default(asv_valid[[i]], data_valid[[j]], method =
## "spearman"): Cannot compute exact p-value with ties

## Warning in cor.test.default(asv_valid[[i]], data_valid[[j]], method =
## "spearman"): Cannot compute exact p-value with ties

## Warning in cor.test.default(asv_valid[[i]], data_valid[[j]], method =
## "spearman"): Cannot compute exact p-value with ties

## Warning in cor.test.default(asv_valid[[i]], data_valid[[j]], method =
## "spearman"): Cannot compute exact p-value with ties

## Warning in cor.test.default(asv_valid[[i]], data_valid[[j]], method =
## "spearman"): Cannot compute exact p-value with ties

## Warning in cor.test.default(asv_valid[[i]], data_valid[[j]], method =
## "spearman"): Cannot compute exact p-value with ties

## Warning in cor.test.default(asv_valid[[i]], data_valid[[j]], method =
## "spearman"): Cannot compute exact p-value with ties

## Warning in cor.test.default(asv_valid[[i]], data_valid[[j]], method =
## "spearman"): Cannot compute exact p-value with ties

## Warning in cor.test.default(asv_valid[[i]], data_valid[[j]], method =
## "spearman"): Cannot compute exact p-value with ties

## Warning in cor.test.default(asv_valid[[i]], data_valid[[j]], method =
## "spearman"): Cannot compute exact p-value with ties

## Warning in cor.test.default(asv_valid[[i]], data_valid[[j]], method =
## "spearman"): Cannot compute exact p-value with ties

## Warning in cor.test.default(asv_valid[[i]], data_valid[[j]], method =
## "spearman"): Cannot compute exact p-value with ties

## Warning in cor.test.default(asv_valid[[i]], data_valid[[j]], method =
## "spearman"): Cannot compute exact p-value with ties

## Warning in cor.test.default(asv_valid[[i]], data_valid[[j]], method =
## "spearman"): Cannot compute exact p-value with ties

## Warning in cor.test.default(asv_valid[[i]], data_valid[[j]], method =
## "spearman"): Cannot compute exact p-value with ties

## Warning in cor.test.default(asv_valid[[i]], data_valid[[j]], method =
## "spearman"): Cannot compute exact p-value with ties

## Warning in cor.test.default(asv_valid[[i]], data_valid[[j]], method =
## "spearman"): Cannot compute exact p-value with ties

## Warning in cor.test.default(asv_valid[[i]], data_valid[[j]], method =
## "spearman"): Cannot compute exact p-value with ties

## Warning in cor.test.default(asv_valid[[i]], data_valid[[j]], method =
## "spearman"): Cannot compute exact p-value with ties

## Warning in cor.test.default(asv_valid[[i]], data_valid[[j]], method =
## "spearman"): Cannot compute exact p-value with ties

## Warning in cor.test.default(asv_valid[[i]], data_valid[[j]], method =
## "spearman"): Cannot compute exact p-value with ties

## Warning in cor.test.default(asv_valid[[i]], data_valid[[j]], method =
## "spearman"): Cannot compute exact p-value with ties

## Warning in cor.test.default(asv_valid[[i]], data_valid[[j]], method =
## "spearman"): Cannot compute exact p-value with ties

## Warning in cor.test.default(asv_valid[[i]], data_valid[[j]], method =
## "spearman"): Cannot compute exact p-value with ties

## Warning in cor.test.default(asv_valid[[i]], data_valid[[j]], method =
## "spearman"): Cannot compute exact p-value with ties

## Warning in cor.test.default(asv_valid[[i]], data_valid[[j]], method =
## "spearman"): Cannot compute exact p-value with ties

## Warning in cor.test.default(asv_valid[[i]], data_valid[[j]], method =
## "spearman"): Cannot compute exact p-value with ties

## Warning in cor.test.default(asv_valid[[i]], data_valid[[j]], method =
## "spearman"): Cannot compute exact p-value with ties

## Warning in cor.test.default(asv_valid[[i]], data_valid[[j]], method =
## "spearman"): Cannot compute exact p-value with ties

## Warning in cor.test.default(asv_valid[[i]], data_valid[[j]], method =
## "spearman"): Cannot compute exact p-value with ties

## Warning in cor.test.default(asv_valid[[i]], data_valid[[j]], method =
## "spearman"): Cannot compute exact p-value with ties

## Warning in cor.test.default(asv_valid[[i]], data_valid[[j]], method =
## "spearman"): Cannot compute exact p-value with ties

## Warning in cor.test.default(asv_valid[[i]], data_valid[[j]], method =
## "spearman"): Cannot compute exact p-value with ties

## Warning in cor.test.default(asv_valid[[i]], data_valid[[j]], method =
## "spearman"): Cannot compute exact p-value with ties

## Warning in cor.test.default(asv_valid[[i]], data_valid[[j]], method =
## "spearman"): Cannot compute exact p-value with ties

## Warning in cor.test.default(asv_valid[[i]], data_valid[[j]], method =
## "spearman"): Cannot compute exact p-value with ties

## Warning in cor.test.default(asv_valid[[i]], data_valid[[j]], method =
## "spearman"): Cannot compute exact p-value with ties

## Warning in cor.test.default(asv_valid[[i]], data_valid[[j]], method =
## "spearman"): Cannot compute exact p-value with ties

## Warning in cor.test.default(asv_valid[[i]], data_valid[[j]], method =
## "spearman"): Cannot compute exact p-value with ties

## Warning in cor.test.default(asv_valid[[i]], data_valid[[j]], method =
## "spearman"): Cannot compute exact p-value with ties

## Warning in cor.test.default(asv_valid[[i]], data_valid[[j]], method =
## "spearman"): Cannot compute exact p-value with ties

## Warning in cor.test.default(asv_valid[[i]], data_valid[[j]], method =
## "spearman"): Cannot compute exact p-value with ties

## Warning in cor.test.default(asv_valid[[i]], data_valid[[j]], method =
## "spearman"): Cannot compute exact p-value with ties

## Warning in cor.test.default(asv_valid[[i]], data_valid[[j]], method =
## "spearman"): Cannot compute exact p-value with ties

## Warning in cor.test.default(asv_valid[[i]], data_valid[[j]], method =
## "spearman"): Cannot compute exact p-value with ties

## Warning in cor.test.default(asv_valid[[i]], data_valid[[j]], method =
## "spearman"): Cannot compute exact p-value with ties

## Warning in cor.test.default(asv_valid[[i]], data_valid[[j]], method =
## "spearman"): Cannot compute exact p-value with ties

## Warning in cor.test.default(asv_valid[[i]], data_valid[[j]], method =
## "spearman"): Cannot compute exact p-value with ties

## Warning in cor.test.default(asv_valid[[i]], data_valid[[j]], method =
## "spearman"): Cannot compute exact p-value with ties

## Warning in cor.test.default(asv_valid[[i]], data_valid[[j]], method =
## "spearman"): Cannot compute exact p-value with ties

## Warning in cor.test.default(asv_valid[[i]], data_valid[[j]], method =
## "spearman"): Cannot compute exact p-value with ties

## Warning in cor.test.default(asv_valid[[i]], data_valid[[j]], method =
## "spearman"): Cannot compute exact p-value with ties

## Warning in cor.test.default(asv_valid[[i]], data_valid[[j]], method =
## "spearman"): Cannot compute exact p-value with ties

## Warning in cor.test.default(asv_valid[[i]], data_valid[[j]], method =
## "spearman"): Cannot compute exact p-value with ties

## Warning in cor.test.default(asv_valid[[i]], data_valid[[j]], method =
## "spearman"): Cannot compute exact p-value with ties

## Warning in cor.test.default(asv_valid[[i]], data_valid[[j]], method =
## "spearman"): Cannot compute exact p-value with ties

## Warning in cor.test.default(asv_valid[[i]], data_valid[[j]], method =
## "spearman"): Cannot compute exact p-value with ties

## Warning in cor.test.default(asv_valid[[i]], data_valid[[j]], method =
## "spearman"): Cannot compute exact p-value with ties

## Warning in cor.test.default(asv_valid[[i]], data_valid[[j]], method =
## "spearman"): Cannot compute exact p-value with ties

## Warning in cor.test.default(asv_valid[[i]], data_valid[[j]], method =
## "spearman"): Cannot compute exact p-value with ties

## Warning in cor.test.default(asv_valid[[i]], data_valid[[j]], method =
## "spearman"): Cannot compute exact p-value with ties

## Warning in cor.test.default(asv_valid[[i]], data_valid[[j]], method =
## "spearman"): Cannot compute exact p-value with ties

## Warning in cor.test.default(asv_valid[[i]], data_valid[[j]], method =
## "spearman"): Cannot compute exact p-value with ties

## Warning in cor.test.default(asv_valid[[i]], data_valid[[j]], method =
## "spearman"): Cannot compute exact p-value with ties

## Warning in cor.test.default(asv_valid[[i]], data_valid[[j]], method =
## "spearman"): Cannot compute exact p-value with ties

## Warning in cor.test.default(asv_valid[[i]], data_valid[[j]], method =
## "spearman"): Cannot compute exact p-value with ties

## Warning in cor.test.default(asv_valid[[i]], data_valid[[j]], method =
## "spearman"): Cannot compute exact p-value with ties

## Warning in cor.test.default(asv_valid[[i]], data_valid[[j]], method =
## "spearman"): Cannot compute exact p-value with ties

## Warning in cor.test.default(asv_valid[[i]], data_valid[[j]], method =
## "spearman"): Cannot compute exact p-value with ties

## Warning in cor.test.default(asv_valid[[i]], data_valid[[j]], method =
## "spearman"): Cannot compute exact p-value with ties

## Warning in cor.test.default(asv_valid[[i]], data_valid[[j]], method =
## "spearman"): Cannot compute exact p-value with ties

## Warning in cor.test.default(asv_valid[[i]], data_valid[[j]], method =
## "spearman"): Cannot compute exact p-value with ties

## Warning in cor.test.default(asv_valid[[i]], data_valid[[j]], method =
## "spearman"): Cannot compute exact p-value with ties

## Warning in cor.test.default(asv_valid[[i]], data_valid[[j]], method =
## "spearman"): Cannot compute exact p-value with ties

## Warning in cor.test.default(asv_valid[[i]], data_valid[[j]], method =
## "spearman"): Cannot compute exact p-value with ties

## Warning in cor.test.default(asv_valid[[i]], data_valid[[j]], method =
## "spearman"): Cannot compute exact p-value with ties

## Warning in cor.test.default(asv_valid[[i]], data_valid[[j]], method =
## "spearman"): Cannot compute exact p-value with ties

## Warning in cor.test.default(asv_valid[[i]], data_valid[[j]], method =
## "spearman"): Cannot compute exact p-value with ties

## Warning in cor.test.default(asv_valid[[i]], data_valid[[j]], method =
## "spearman"): Cannot compute exact p-value with ties

## Warning in cor.test.default(asv_valid[[i]], data_valid[[j]], method =
## "spearman"): Cannot compute exact p-value with ties

## Warning in cor.test.default(asv_valid[[i]], data_valid[[j]], method =
## "spearman"): Cannot compute exact p-value with ties

## Warning in cor.test.default(asv_valid[[i]], data_valid[[j]], method =
## "spearman"): Cannot compute exact p-value with ties

## Warning in cor.test.default(asv_valid[[i]], data_valid[[j]], method =
## "spearman"): Cannot compute exact p-value with ties

## Warning in cor.test.default(asv_valid[[i]], data_valid[[j]], method =
## "spearman"): Cannot compute exact p-value with ties

## Warning in cor.test.default(asv_valid[[i]], data_valid[[j]], method =
## "spearman"): Cannot compute exact p-value with ties

## Warning in cor.test.default(asv_valid[[i]], data_valid[[j]], method =
## "spearman"): Cannot compute exact p-value with ties

## Warning in cor.test.default(asv_valid[[i]], data_valid[[j]], method =
## "spearman"): Cannot compute exact p-value with ties
```

```
cor_matrix_uganda <- as.data.frame(cor_matrix_uganda)
colnames(cor_matrix_uganda) <- c("ASV", "Marker", "Pvalue", "Rho")
cor_matrix_uganda$Pvalue <- as.numeric(as.character(cor_matrix_uganda$Pvalue))
cor_matrix_uganda$Rho <- as.numeric(as.character(cor_matrix_uganda$Rho))
cor_matrix_uganda_valid <- cor_matrix_uganda[!is.na(cor_matrix_uganda$Pvalue), , drop = F]
cor_matrix_uganda_valid$Marker <- factor(cor_matrix_uganda_valid$Marker, levels = c("scd14_ng_ml_uvm", "ifabp_pg_ml_uvm"))
cor_matrix_uganda_valid$Cohort <- "uganda"
cor_matrix_uganda_valid[cor_matrix_uganda_valid$Marker == "scd14_ng_ml_uvm", "Padj"] <- p.adjust(cor_matrix_uganda_valid[cor_matrix_uganda_valid$Marker == "scd14_ng_ml_uvm", "Pvalue"], method = "fdr")
cor_matrix_uganda_valid[cor_matrix_uganda_valid$Marker == "ifabp_pg_ml_uvm", "Padj"] <- p.adjust(cor_matrix_uganda_valid[cor_matrix_uganda_valid$Marker == "ifabp_pg_ml_uvm", "Pvalue"], method = "fdr")
cor_matrix_uganda_valid$Sig <- cor_matrix_uganda_valid$Padj
cor_matrix_uganda_valid$Sig[cor_matrix_uganda_valid$Sig <= 0.05] <- "Yes"
cor_matrix_uganda_valid$Sig[cor_matrix_uganda_valid$Sig != "Yes"] <- "No"

#Merge the results from the three cohorts:
all <- rbind(cor_matrix_us_valid, cor_matrix_botswana_valid, cor_matrix_uganda_valid)

us_all <- all[all$Cohort == "us", , drop = F]
us_all <- dplyr::left_join(us_all, dplyr::select(us, tidyselect::any_of(c("taxon", "Genus_Species", "Genus", "Species", "log2FoldChange"))), by = c("ASV" = "taxon"))
us_all$ASV <- factor(us_all$ASV, levels = us$taxon)
us_all %>% dplyr::mutate(Log2FC =
                           dplyr::case_when(log2FoldChange > 0 ~ "HIV",
                                            log2FoldChange < 0 ~ "NEG", )) -> us_all

botswana_all <- all[all$Cohort == "botswana", , drop = F]
botswana_all <- dplyr::left_join(botswana_all, dplyr::select(botswana, tidyselect::any_of(c("taxon", "Genus_Species", "Genus", "Species", "log2FoldChange"))), by = c("ASV" = "taxon"))
botswana_all$ASV <- factor(botswana_all$ASV, levels = botswana$taxon)
botswana_all %>% dplyr::mutate(Log2FC =
                                  dplyr::case_when(log2FoldChange > 0 ~ "HIV",
                                                   log2FoldChange < 0 ~ "NEG", )) -> botswana_all

uganda_all <- all[all$Cohort == "uganda", , drop = F]
uganda_all <- dplyr::left_join(uganda_all, dplyr::select(uganda, tidyselect::any_of(c("taxon", "Genus_Species", "Genus", "Species", "log2FoldChange"))), by = c("ASV" = "taxon"))
uganda_all$ASV <- factor(uganda_all$ASV, levels = uganda$taxon)
uganda_all %>% dplyr::mutate(Log2FC =
                               dplyr::case_when(log2FoldChange > 0 ~ "HIV",
                                                log2FoldChange < 0 ~ "NEG", ))  -> uganda_all

all <- rbind(us_all, botswana_all, uganda_all)
all$ASV <- fct_reorder(all$ASV, all$log2FoldChange)
all$Cohort <- fct_relevel(all$Cohort, "us")
all$Log2FC <- fct_rev(all$Log2FC)

# Remove extra brackets around taxonomic name
all$Genus <- stringr::str_replace(all$Genus, "^\\[([^\\]]+)\\]", "\\1")
all$Species <- stringr::str_replace(all$Species, "^\\[([^\\]]+)\\]", "\\1")

all %>% dplyr::mutate(Genus_species_label =
                dplyr::case_when(is.na(stringr::str_extract(Genus, "\\[")) & is.na(stringr::str_extract(Species, "\\[")) ~ paste(Genus,Species),
                                 TRUE ~ paste(Genus,"sp"))) -> all

all$Genus_species_label <- stringr::str_replace(all$Genus_species_label, "\\[([^\\]]+)\\]", "")
all$Genus_species_label <- stringr::str_replace(all$Genus_species_label, "\\ +", " ")
Genus_species_label_lookup <- dplyr::distinct(all, ASV, .keep_all = TRUE)$Genus_species_label
names(Genus_species_label_lookup) <- dplyr::distinct(all, ASV, .keep_all = TRUE)$ASV

# Make plots

hiv_header_namer <- as_labeller(c(`HIV` = "Increased in\nHIV infection", `NEG` = "Decreased in\nHIV infection"))
marker_namer <- c(`scd14_ng_ml_uvm` = "sCD14", `ifabp_pg_ml_uvm` = "iFABP")

plot_all <- ggplot(data = all, aes(x = Marker, y = reorder(ASV, log2FoldChange))) + geom_tile(aes(fill = Rho, color = Sig), linewidth = 0.5, width = 0.9, height = 0.9) +
  scale_fill_gradientn(colors = c("darkred","white","darkblue"), limits = c(-0.4,0.4)) + scale_color_manual(values = c("white", "black")) +
  scale_y_discrete(label = as_labeller(Genus_species_label_lookup)) + scale_x_discrete(label = as_labeller(marker_namer)) + theme_bw() +
  theme(axis.text.x = element_text(angle = 0, size = 14, colour = "black", face = "bold"), axis.text.y = element_text(size = 8), axis.ticks = element_blank(), axis.title = element_blank(), panel.grid.major.x = element_blank(), panel.grid.minor.x = element_blank(), strip.background = element_rect(colour = "white", fill = "white"), strip.text = element_text(colour = "black", face = "bold", size = rel(1.2))) +
  facet_grid(vars(Cohort), vars(Log2FC), scales = "free", space = "free", switch = "y", labeller = labeller(Cohort = label_value, Log2FC = hiv_header_namer))

ggsave("Figure4C_ANCOM_v20.pdf", plot_all, width = 6, height = 30)
#--------------------------------------------------------------------------------------------------------------
```

#Figure 4D

```
#FIGURE 4D
#--------------------------------------------------------------------------------------------------------------
shared_ASVs <- as.character(dplyr::filter(fct_count(all$ASV), n > 2)$f)
all_intersect <- all[all$ASV %in% shared_ASVs, , drop = F]
all_intersect$ASV <- fct_reorder2(fct_drop(all_intersect$ASV), all_intersect$log2FoldChange, all_intersect$Cohort)

marker_namer <- c(`scd14_ng_ml_uvm` = "sCD14", `ifabp_pg_ml_uvm` = "iFABP")
cohort_namer <- as_labeller(c(`us` = "U.S.", `botswana` = "Botswana", `uganda` = "Uganda"))

Genus_species_label_lookup <- dplyr::distinct(all, ASV, .keep_all = TRUE)$Genus_species_label
names(Genus_species_label_lookup) <- dplyr::distinct(all, ASV, .keep_all = TRUE)$ASV

ggsave("Figure4D_ANCOM_v14.pdf", 
       ggplot(data = all_intersect, aes(x = Marker, y = ASV)) + geom_tile(aes(fill = Rho, color = Sig), size = 0.9, width = 0.90, height = 0.90) +
         scale_fill_gradientn(colors = c("darkred","white","darkblue"), limits = c(-0.4,0.4)) + scale_color_manual(values = c("white", "black")) +
         coord_fixed(ratio = 1.0) +
         scale_y_discrete(label = as_labeller(Genus_species_label_lookup)) + scale_x_discrete(label = as_labeller(marker_namer)) +
         geom_hline(yintercept = seq(1.5, length(unique(all_intersect$ASV)), by = 1), size = 0.06, alpha = 0.2, linetype = "dashed") +
         theme_bw() + 
         theme(axis.text.x = element_text(angle = 90, size = 22, colour = "black", face = "bold", vjust = 0.5, hjust = 1.0), axis.text.y = element_text(size = 16),
               axis.ticks = element_blank(), axis.title = element_blank(), panel.grid.major = element_blank(), panel.grid.minor = element_blank(),
               strip.background = element_rect(colour = "white", fill = "white"), strip.text = element_text(colour = "black", face = "bold", size = rel(1.2))) + 
         facet_wrap(vars(Cohort), labeller = labeller(Cohort = cohort_namer)), width = 8, height = 10)
```

```
## Warning: Using `size` aesthetic for lines was deprecated in ggplot2 3.4.0.
## ℹ Please use `linewidth` instead.
```

```
#--------------------------------------------------------------------------------------------------------------

#FIGURE 2E
#--------------------------------------------------------------------------------------------------------------
all -> all_test #remove


# HEATMAPS

shared_ASVs <- as.character(dplyr::filter(fct_count(all$ASV), n > 2)$f)
all <- dplyr::mutate(.data = all, ASV = as.character(ASV))
all_intersect <- all[all$ASV %in% shared_ASVs, , drop = F]

marker_namer <- c(`scd14_ng_ml_uvm` = "sCD14", `ifabp_pg_ml_uvm` = "iFABP")
cohort_namer <- as_labeller(c(`us` = "U.S.", `botswana` = "Botswana", `uganda` = "Uganda"))

us <- dplyr::filter(.data = all, Cohort == "us")
botswana <- dplyr::filter(.data = all, Cohort == "botswana")
uganda <- dplyr::filter(.data = all, Cohort == "uganda")

length(intersect(dplyr::filter(.data = all_intersect, Cohort %in% c("us"))$ASV, dplyr::filter(.data = all_intersect, Cohort %in% c("botswana"))$ASV)) -> us_botswana_overlap #3
length(intersect(dplyr::filter(.data = all_intersect, Cohort %in% c("us"))$ASV, dplyr::filter(.data = all_intersect, Cohort %in% c("uganda"))$ASV))  -> us_uganda_overlap #0
length(intersect(dplyr::filter(.data = all_intersect, Cohort %in% c("botswana"))$ASV, dplyr::filter(.data = all_intersect, Cohort %in% c("uganda"))$ASV)) -> botswana_uganda_overlap #9

# US-BOTSWANA Overlap
common_us_bots <- c(intersect(dplyr::filter(.data = all_intersect, Cohort %in% c("us"))$ASV, dplyr::filter(.data = all_intersect, Cohort %in% c("botswana"))$ASV))
botswana_common <- botswana[botswana$ASV %in% common_us_bots, , drop = F]
us_common <- us[us$ASV %in% common_us_bots, , drop = F]
all.equal(botswana_common$ASV, us_common$ASV)
```

```
## [1] TRUE
```

```
botswana_us_common <- dplyr::bind_rows(botswana_common, us_common)
botswana_us_common$ASV <- fct_reorder2(fct_drop(botswana_us_common$ASV), botswana_us_common$log2FoldChange, botswana_us_common$Cohort)

ggsave("Figure4D_heat_US_BOTSWANA_v1.pdf", 
       ggplot(data = botswana_us_common, aes(x = Marker, y = ASV)) + geom_tile(aes(fill = Rho, color = Sig), size = 0.9, width = 0.90, height = 0.90) +
         scale_fill_gradientn(colors = c("darkred","white","darkblue"), limits = c(-0.4,0.4)) + scale_color_manual(values = c("white", "black")) +
         coord_fixed(ratio = 1.0) +
         scale_y_discrete(label = as_labeller(Genus_species_label_lookup)) + scale_x_discrete(label = as_labeller(marker_namer)) +
         geom_hline(yintercept = seq(1.5, length(unique(botswana_us_common$ASV)), by = 1), size = 0.06, alpha = 0.2, linetype = "dashed") +
         theme_bw() + 
         theme(axis.text.x = element_text(angle = 90, size = 22, colour = "black", face = "bold", vjust = 0.5, hjust = 1.0), axis.text.y = element_text(size = 16),
               axis.ticks = element_blank(), axis.title = element_blank(), panel.grid.major = element_blank(), panel.grid.minor = element_blank(),
               strip.background = element_rect(colour = "white", fill = "white"), strip.text = element_text(colour = "black", face = "bold", size = rel(1.2))) + 
         facet_wrap(vars(Cohort), labeller = labeller(Cohort = cohort_namer)), width = 8, height = 10)


# UGANDA-BOTSWANA Overlap
common_uganda_bots <- c(intersect(dplyr::filter(.data = all_intersect, Cohort %in% c("uganda"))$ASV, dplyr::filter(.data = all_intersect, Cohort %in% c("botswana"))$ASV))
botswana_common <- botswana[botswana$ASV %in% common_uganda_bots, , drop = F]
uganda_common <- uganda[uganda$ASV %in% common_uganda_bots, , drop = F]
all.equal(botswana_common$ASV, uganda_common$ASV)
```

```
## [1] TRUE
```

```
botswana_uganda_common <- dplyr::bind_rows(botswana_common, uganda_common)
botswana_uganda_common$ASV <- fct_reorder2(fct_drop(botswana_uganda_common$ASV), botswana_uganda_common$Cohort, botswana_uganda_common$log2FoldChange)

ggsave("Figure4D_heat_UGANDA_BOTSWANA_v1.pdf", 
       ggplot(data = botswana_uganda_common, aes(x = Marker, y = ASV)) + geom_tile(aes(fill = Rho, color = Sig), size = 0.9, width = 0.90, height = 0.90) +
         scale_fill_gradientn(colors = c("darkred","white","darkblue"), limits = c(-0.4,0.4)) + scale_color_manual(values = c("white", "black")) +
         coord_fixed(ratio = 1.0) +
         scale_y_discrete(label = as_labeller(Genus_species_label_lookup)) + scale_x_discrete(label = as_labeller(marker_namer)) +
         geom_hline(yintercept = seq(1.5, length(unique(botswana_uganda_common$ASV)), by = 1), size = 0.06, alpha = 0.2, linetype = "dashed") +
         theme_bw() + 
         theme(axis.text.x = element_text(angle = 90, size = 22, colour = "black", face = "bold", vjust = 0.5, hjust = 1.0), axis.text.y = element_text(size = 16),
               axis.ticks = element_blank(), axis.title = element_blank(), panel.grid.major = element_blank(), panel.grid.minor = element_blank(),
               strip.background = element_rect(colour = "white", fill = "white"), strip.text = element_text(colour = "black", face = "bold", size = rel(1.2))) + 
         facet_wrap(vars(Cohort), labeller = labeller(Cohort = cohort_namer)), width = 8, height = 10)

#VENN-EULER DIAGRAMS
BioVenn::draw.venn(us$ASV, botswana$ASV, uganda$ASV, title = NULL, subtitle = NULL, 
                   xtitle = "U.S.", x_c = "royalblue4",
                   ytitle = "Botswana", y_c = "darkorange",
                   ztitle = "Uganda", z_c = "forestgreen",
                   nrtype = "abs", 
                   output = "pdf", filename = "Figure4D_venn_v1.pdf")
```

```
## [1] "x total: 53"
## [1] "y total: 26"
## [1] "z total: 128"
## [1] "x only: 50"
## [1] "y only: 14"
## [1] "z only: 119"
## [1] "x-y total overlap: 3"
## [1] "x-z total overlap: 0"
## [1] "y-z total overlap: 9"
## [1] "x-y only overlap: 3"
## [1] "x-z only overlap: 0"
## [1] "y-z only overlap: 9"
## [1] "x-y-z overlap: 0"
```

```
## $x
##  [1] "GCAAGCGTTATCCGGATTTACTGGGTGTAAAGGGAGCGTAGGCGGCGATGCAAGTCAGAAGTGAAAGCCCAGGGCTTAACCGTGGGACTGCTTTTGAAACTGTGTTGCTGGATTGCCGGAGAGGTAAGTGGAATTCCTAGTGTAGCGGTGAAATGCGTAGATATTAGGAGGAACACCAGTGGCGAAGGCGGCTTACTGGACGGTGAATGACGCTGAGGCTCGAAAGCGTGGGGAGCAAAC"
##  [2] "GCAAGCGTTATCCGGATTTACTGGGTGTAAAGGGTGCGTAGGTGGCAGTGCAAGTCAGATGTGAAAGGCCGGGGCTCAACCCCGGAGCTGCATTTGAAACTGCGCGGCTAGAGTACAGGAGAGGCAGGCGGAATTCCTAGTGTAGCGGTGAAATGCGTAGATATTAGGAGGAACACCAGTGGCGAAGGCGGCCTGCTGGACTGTTACTGACACTGAGGCACGAAAGCGTGGGGAGCAAAC"
##  [3] "GCAAGCGTTATCCGGATTTATTGGGTGTAAAGGGTGCGTAGACGGGAAGGTAAGTTAGTTGTGAAATCCCTCGGCTCAACTGAGGAACTGCGACTAAAACTGCTTTTCTTGAGTGCTGGAGAGGAAAGTGGAATTCCTAGTGTAGCGGTGAAATGCGTAGATATTAGGAGGAACACCAGTGGCGAAGGCGACTTTCTGGACAGCAACTGACGTTGAGGCACGAAAGTGTGGGGAGCAAAC"
##  [4] "ACAAGCGTTGTCCGGATTTACTGGGTGTAAAGGGCGCGTAGGCGGACTGTCAAGTCAGTCGTGAAATACCGGGGCTTAACCCCGGGGCTGCGATTGAAACTGACAGCCTTGAGTATCGGAGAGGAAAGCGGAATTCCTAGTGTAGCGGTGAAATGCGTAGATATTAGGAGGAACACCAGTGGCGAAGGCGGCTTACTGGACGGTAACTGACGTTGAGGCTCGAAAGCGTGGGGAGCAAAC"
##  [5] "GCGAGCGTTGTCCGGAATTACTGGGTGTAAAGGGAGTGTAGGCGGGAAGGCAAGTCAGAAGTGAAAATTATGGGCTTAACCCATAACCTGCTTTTGAAACTGTTTTTCTTGAGTGAGGCAGAGGCAAGCGGAATTCCTAGTGTAGCGGTGAAATGCGTAGATATTAGGAGGAACACCAGTGGCGAAGGCGGCTTGCTGGGCCTTTACTGACGCTGAGGCTCGAAAGCGTGGGGAGCAAAC"
##  [6] "GCAAGCGTTGTCCGGAATTACTGGGTGTAAAGGGAGCGTAGGCGGGATGCCAAGTCAGCTGTGAAAACTATGGGCTTAACTTGTAGACTGCAGTTGAAACTGGTATTCTTGAGTGAAGTAGAGGTTGGCGGAATTCCGAGTGTAGCGGTGAAATGCGTAGATATTCGGAGGAACACCGGTGGCGAAGGCGGCCAACTGGGCTTTAACTGACGCTGAGGCTCGAAAGTGTGGGGAGCAAAC"
##  [7] "GCAAGCGTTGTCCGGAATTACTGGGTGTAAAGGGAGCGTAGGCGGGATGCCAAGTCAGCTGTGAAAACTATGGGCTTAACCTGTAGACTGCAGTTGAAACTGGTATTCTTGAGTGAAGTAGAGGTTGGCGGAATTCCGAGTGTAGCGGTGAAATGCGTAGATATTCGGAGGAACACCGGTGGCGAAGGCGGCCAACTGGGCTTTAACTGACGCTGAGGCTCGAAAGTGTGGGGAGCAAAC"
##  [8] "GCGAGCGTTGTCCGGAATTACTGGGTGTAAAGGGAGCGTAGGCGGGACAGCAAGTTGAATGTGAAATCTATGGGCTCAACCCATAAACTGCGTTCAAAACTGTTGTTCTTGAGTGAAGTAGAGGTAGGCGGAATTCCTAGTGTAGCGGTGAAATGCGTAGATATTAGGAGGAACACCAGTGGCGAAGGCGGCCTACTGGGCTTTAACTGACGCTGAGGCTCGAAAGCGTGGGTAGCAAAC"
##  [9] "GCAAGCGTTGTCCGGAATTACTGGGTGTAAAGGGAGCGTAGGCGGGGAGACAAGTTGAATGTTTAAACTATCGGCTCAACTGATAGTCGCGTTCAAAACTATCACTCTTGAGTGCAGTAGAGGTAGGCGGAATTCCTAGTGTAGCGGTGAAATGCGTAGATATTAGGAGGAACACCAGTGGCGAAGGCGGCCTACTGGGCTGTAACTGACGCTGAGGCTCGAAAGCGTGGGTAGCAAACA"
## [10] "ACAAGCGTTGTCCGGAATTACTGGGTGTAAAGGGAGCGCAGGCGGGCGATCAAGTTGGAAGTGAAATCCATGGGCTCAACCCATGAACTGCTTTCAAAACTGATTGTCTTGAGTAGTGCAGAGGTAGGCGGAATTCCCGGTGTAGCGGTGGAATGCGTAGATATCGGGAGGAACACCAGTGGCGAAGGCGGCCTACTGGGCACCAACTGACGCTGAGGCTCGAAAGTGTGGGTAGCAAAC"
## [11] "ACAAGCGTTGTCCGGAATTACTGGGTGTAAAGGGAGCGCAGGCGGGAAGACAAGTTGGAAGTGAAATCTATGGGCTCAACCCATAAACTGCTTTCAAAACTGCTGGCCTTGAGTAGTGCAGAGGTAGGTGGAATTCCCGGTGTAGCGGTGGAATGCGTAGATATCGGGAGGAACACCAGTGGCGAAGGCGGCCTACTGGGCACCAACTGACGCTGAGGCTCGAAAGTGTGGGTAGCAAAC"
## [12] "ACAAGCGTTGTCCGGAATTACTGGGTGTAAAGGGAGCGCAGGCGGGAAGACAAGTTGGGAGTGAAATCTATGGGCTCAACCCATAAACTGCTTTCAAAACTGTTTTTCTTGAGTAGTGCAGAGGTAGGCGGAATTCCCGGTGTAGCGGTGGAATGCGTAGATATCGGGAGGAACACCAGTGGCGAAGGCGGCCTACTGGGCACCAACTGACGCTGAGGCTCGAAAGTGTGGGTAGCAAAC"
## [13] "ACAAGCGTTGTCCGGAATTACTGGGTGTAAAGGGAGCGCAGGCGGGAAGACAAGTTGGAAGTGAAATCTATGGGCTCAACCCATAAACTGCTTTCAAAACTGTTTTTCTTGAGTAGTGCAGAGGTAGGCGGAATTCCCGGTGTAGCGGTGGAATGCGTAGATATCGGGAGGAACACCAGTGGCGAAGGCGGCCTACTGGGCACCAACTGACGCTGAGGCTCGGAAGTGTGGGTAGCAAAC"
## [14] "ACAAGCGTTGTCCGGAACTACTGGGTGTAAAGGGAGCGCAGGCGGGAAGACAAGTTGGAAGTGAAATCTATGGGCTCAACCCATAAACTGCTTTCAAAACTGTTTTTCTTGAGTAGTGCAGAGGTAGGCGGAATTCCCGGTGTAGCGGTGGAATGCGTAGATATCGGGAGGAACACCAGTGGCGAAGGCGGCCTACTGGGCACCAACTGACGCTGAGGCTCGAAAGTGTGGGTAGCAAAC"
## [15] "GCAAGCGTTGTCCGGAATTACTGGGTGTAAAGGGCGTGTAGGCGGAGCTGCAAGTCAGATGTGAAATCCCGGGGCTCAACCCCGGAACTGCATTTGAAACTGTAGCCCTTGAGTATCGGAGAGGCAAGCGGAATTCCTAGTGTAGCGGTGAAATGCGTAGATATTAGGAGGAACACCAGTGGCGAAGGCGGCTTGCTGGACGACAACTGACGCTGAGGCGCGAAAGCGTGGGGAGCAAAC"
## [16] "GCAAGCGTTGTCCGGATTTACTGGGTGTAAAGGGCGTGTAGGCGGAGCAGCAAGTCAGAAGTGAAATCTCTGGGCTCAACCCAGAAACTGCTTTTGAAACTGTTGCCCTTGAGTATCGGAGAGGCAGGCGGAATTCCTAGTGTAGCGGTGAAATGCGTAGATATTAGGAGGAACACCAGTGGCGAAGGCGGCCTGCTGGACGACAACTGACGCTGAGGCGCGAAAGCGTGGGGAGCAAAC"
## [17] "GCAAGCGTTGTCCGGATTTACTGGGTGTAAAGGGCGTGCAGCCGGGCCGGCAAGTCAGATGTGAAATCCACGGGCTTAACCCGTGAACTGCATTTGAAACTGTTGGTCTTGAGTATCGGAGAGGTAATCGGAATTCCTTGTGTAGCGGTGAAATGCGTAGATATAAGGAAGAACACCAGTGGCGAAGGCGGATTACTGGACGACAACTGACGGTGAGGCGCGAAAGCGTGGGGAGCAAAC"
## [18] "GCAAGCGTTATCCGGATTTACTGGGTGTAAAGGGCGCGTAGGCGGGGATGCAAGTCAGATGTGAAATCTATGGGCTTAACCCATAAACTGCATTTGAAACTGTATCTCTTGAGTGCTGGAGAGGTAGACGGAATTCCTTGTGTAGCGGTGAAATGCGTAGATATAAGGAAGAACACCAGTGGCGAAGGCGGTCTACTGGACAGTAACTGACGCTGAGGCGCGAGAGCGTGGGGAGCAAAC"
## [19] "GCAAGCGTTATCCGGATTTATTGGGTGTAAAGGGTGTGTAGGCGGGACTGCAAGTCAGACGTGAAAATCATGGGCTCAACCCATGACTTGCGTTTGAAACTGCGGTTCTTGAGAGTGGGAGAGGTAAACGGAATTCCTGGTGTAGCGGTGAAATGCGTAGATATCAGGAGGAACACCGGTGGCGAAGGCGGTTTACTGGACCACAACTGACGCTGAGACACGAAAGCGTGGGGAGCAAAC"
## [20] "GCGAGCGTTATCCGGAATTACTGGGTGTAAAGGGTGTGTAGGCGGGGTGTCAAGTCAGATGTGAAAACTGTGGGCTCAACCCACAAACTGCATTTGAAACTGATACTCTTGAGAGTGGGAGAGGTAAACGGAATTCCTGGTGTAGTAGTGAAATGCGTAGATATCAGGAGGAACACCGGTGGCGAAGGCGGTTTACTGGACCACAACTGACGCTGAGACACGAAAGCGTGGGGAGCAAAC"
## [21] "GCAAGCGTTATCCGGATTTACTGGGTGTAAAGGGCGTGTAGGCGGGACTGCAAGTCAGGTGTGAAAACCAGGGGCTCAACCTCTGGCCTGCATTTGAAACTGTAGTTCTTGAGTGCTGGAGAGGCAATCGGAATTCCGTGTGTAGCGGTGAAATGCGTAGATATACGGAGGAACACCAGTGGCGAAGGCGGATTGCTGGACAGTAACTGACGCTGAGGCGCGAAAGCGTGGGGAGCAAAC"
## [22] "GCGAGCGTTGTCCGGAATTACTGGGCGTAAAGGGCGCGTAGGCGGCCAATTAAGTTAGATGTGAAATCCCCGGGCTTAACCTGGGTGTTGCATTTAAAACTGATAGGCTTGAGTGCAGGAGAGGGAAGCGGAATTCCTAGTGTAGCGGTGGAATGCGTAGATATTAGGAGGAACACCAGTGGCGAAGGCGGCTTTCTGGACTGTAACTGACGCTGAGGCGCGAGAGCGTGGGGAGCAAAC"
## [23] "GCGAGCGTTAATCGGAATTACTGGGCGTAAAGCGCACGTAGGCTGTATGTCAAGTCAAGGGTGAAATCCCACGGCTCAACCGTGGAACTGCCTTTGAAACTGGCAAACTGGAGTATGTGAGAGGGCGGCGGAATTCCTGGTGTAGGAGTGAAATCCGTAGATATCAGGAGGAACATCAGTGGCGAAGGCGGCCGCCTGGCACAAAACTGACGCTGAGGTGCGAAAGCGTGGGTAGCAAAC"
## [24] "GCAAGCGTTATCCGGAATTATTGGGCGTAAAGGGCTCGTAGGCGGTTCGTCGCGTCCGGTGTGAAAGTTCATCGCTTAACGGTGGATCCGCGCCGGGTACGGGCGGGCTTGAGTGCGGTAGGGGAGACTGGAATTCCCGGTGTAACGGTGGAATGTGTAGATATCGGGAAGAACACCAATGGCGAAGGCAGGTCTCTGGGCCGTTACTGACGCTGAGGAGCGAAAGCGTGGGGAGCGAAC"
## [25] "CCGAGCGTTATCCGGATTTATTGGGTTTAAAGGGAGCGTAGATGGATGTTTAAGTCAGTTGTGAAAGTTTGCGGCTCAACCGTAAAATTGCAGTTGATACTGGCAGTCTTGAGTGCAGTAGAGGTGGGCGGAATTCGTGGTGTAGCGGTGAAATGCTTAGATATCACGAAGAACTCCGATTGCGAAGGCAGCCTGCTAAGCTGCAACTGACATTGAGGCTCGAAAGTGTGGGTATCAAAC"
## [26] "CCGAGCGTTATCCGGATTTATTGGGTTTAAAGGGAGCGTAGGTGGACAGTTAAGTCAGTTGTGAAAGTTTGCGGCTCAACCGTAAAATTGCAGTTGATACTGGCTGTCTTGAGTACAGTAGAGGTGGGCGGAATTCGTGGTGTAGCGGTGAAATGCTTAGATATCACGAAGAACTCCGATTGCGAAGGCAGCTCACTGGAGCGCAACTGACGCTGAAGCTCGAAAGTGCGGGTATCGAAC"
## [27] "CCGAGCGTTATCCGGATTTATTGGGTTTAAAGGGAGCGTAGGCGGATTATTAAGTCAGTTGTGAAAGTTTGCGGCTCAACCGTAAAATTGCAGTTGATACTGGTAGTCTTGAGTGCAGCAGAGGTAGGCGGAATTCGTGGTGTAGCGGTGAAATGCTTAGATATCACGAAGAACTCCGATTGCGAAGGCAGCTTACTGGACTGTAACTGACGCTGATGCTCGAAAGTGTGGGTATCAAAC"
## [28] "GCGAGCGTTATCCGGATTTATTGGGTTTAAAGGGTGCGTAGGCGGCCCTATAAGTCAGCGGTGAAATGTTCCGGCTCAACCGGGAAACTGCCGTTGAAACTGTAGAGCTAGAGTCCACAAGAGGTATGCGGAATGCGTGGTGTAGCGGTGAAATGCATAGATATCACGCAGAACCCCGATTGCGAAGGCAGCATACTGGGGTGAAACAGACGCTGAAGCACGAAAGCGTGGGTATCGAAC"
## [29] "GCGAGCGTTATCCGGATTTATTGGGTTTAAAGGGTGCGTAGGCGGAAGAATAAGTCAGCGGTGAAATGCTTCAGCTCAACTGGAGAATTGCCGATGAAACTGTTTTTCTAGAGTATAAAAGAGGTATGCGGAATGCGTGGTGTAGCGGTGAAATGCATAGATATCACGCAGAACCCCGATTGCGAAGGCAGCATACTGGGCTATAACTGACGCTGAAGCACGAAAGCGTGGGTATCGAAC"
## [30] "CCGAGCGTTATCCGGATTTATTGGGTTTAAAGGGTGCGTAGGCTGTTTTTTAAGTTAGAGGTGAAAGCTCGACGCTCAACGTCGAAATTGCCTCTGATACTGAGAGACTAGAGTGTAGTTGCGGAAGGCGGAATGTGTGGTGTAGCGGTGAAATGCTTAGATATCACACAGAACACCGATTGCGAAGGCAGCTTTCCAAGCTATTACTGACGCTGAGGCACGAAAGCGTGGGGAGCGAAC"
## [31] "GCGAGCGTTATCCGGATTTATTGGGTTTAAAGGGTGCGTAGGCGGGTGATTAAGTCAGCGGTGAAATGCGTCAGCTTAACTGGCGAACTGCCATTGAAACTGGTTACCTTGAGTGTAGCGGAAGTATGCGGAATGCGTGGTGTAGCGGTGAAATGCATAGATATCACGCAGAACTCCGATTGCGAAGGCAGCATACCATACTATAACTGACGCTGAAGCACGAAAGCGTGGGTATCGAAC"
## [32] "GCGAGCGTTATCCGGATTTATTGGGTTTAAAGGGTGCGTAGGCGGCACGCCAAGTCAGCGGTGAAATTTTCGGGCTCAACCCGGACTGTGCCGTTGAAACTGGCGAGCTAGAGTGCACAAGAGGCAGGCGGAATGCGTGGTGTAGCGGTGAAATGCATAGATATCACGCAGAACCCCGATTGCGAAGGCAGCCTGCTAGGGTGCGACAGACGCTGAGGCACGAAAGCGTGGGTATCGAAC"
## [33] "GCAAGCGTTGTCCGGATTTATTGGGCGTAAAGCGAGCGCAGGCGGAAGAATAAGTCTGATGTGAAAGCCCTCGGCTTAACCGAGGAACTGCATCGGAAACTGTTTTTCTTGAGTGCAGAAGAGGAGAGTGGAACTCCATGTGTAGCGGTGGAATGCGTAGATATATGGAAGAACACCAGTGGCGAAGGCGGCTCTCTGGTCTGCAACTGACGCTGAGGCTCGAAAGCATGGGTAGCGAAC"
## [34] "GCAAGCGTTGTCCGGAATTATTGGGCGTAAAGCGCGCGCAGGCGGCTTCTTAAGTCCATCTTAAAAGTGCGGGGCTTAACCCCGTGATGGGATGGAAACTGGGAGGCTGGAGTATCGGAGAGGAAAGTGGAATTCCTAGTGTAGCGGTGAAATGCGTAGAGATTAGGAAGAACACCGGTGGCGAAGGCGACTTTCTGGACGACAACTGACGCTGAGGCGCGAAAGCGTGGGGAGCAAACA"
## [35] "GCGAGCGTTGTCCGGAATTATTGGGCGTAAAGAGTACGTAGGCGGTTTGCTAAGCGCAAGGTGAAAGGCAGTGGCTTAACCATTGTAAGCCTTGCGAACTGGCAGACTTGAGTGCAGGAGAGGAAAGCGGAATTCCTAGTGTAGCGGTGAAATGCGTAGATATTAGGAGGAACACCGGTGGCGAAGGCGGCTTTCTGGACTGTAACTGACGCTGAGGTACGAAAGCGTGGGGAGCAAACA"
## [36] "GCAAGCGTTGTCCGGAATTATTGGGCGTAAAGAGTACGTAGGCGGTCTGGTAAGCGCAAGGTGAAAGGCATAGGCTCAACCAATGTCAGCCTTGCGAACTGTCAGACTTGAGTGCAGGAGGGGAAAGTGGAATTCCTAGTGTAGCGGTGAAATGCGTAGATATTAGGAGGAACACCAGTGGCGAAGGCGACTTTCTGGACTGTAACTGACGCTGAGGTACGAAAGCGTGGGGAGCAAACA"
## [37] "GCAAGCGTTGTCCGGAATGATTGGGCGTAAAGGGCGCGTAGGCGGCCAACTAAGTCTGGAGTGAAAGTCCTGCTTTTAAGGTGGGAATTGCTTTGGAAACTGGATGGCTTGAGTGCAGGAGAGGTAAGCGGAATTCCCGGTGTAGCGGTGAAATGCGTAGAGATCGGGAGGAACACCAGTGGCGAAGGCGGCTTACTGGACTGTAACTGACGCTGAGGCGCGAAAGTGTGGGGAGCAAAC"
## [38] "GCGAGCGTTGTCCGGAATTACTGGGCGTAAAGGGAGCGTAGGCGGTCTGATAAGTTGGATGTGAAATACCCGGGCTTAACTTGGGGGGTGCATCCAATACTGTTGGACTAGAGTACAGGAGAGGAAAGCGGAATTCCTAGTGTAGCGGTGAAATGCATAGATATTAGGAGGAACATCGGTGGCGAAGGCGGCTTTCTGGACTGCAACTGACGCTGAGGCTCGAAAGCGTGGGGAGCAAAC"
## [39] "GCAAGCGTTGTCCGGAATCACTGGGCGTAAAGGGCGCGTAGGCGGCAATATAAGTCAGATGTGAAAGGTGAGGGCTCAACCCTTAGACTGCATCTGATACTGTATAGCTTGAGTGTGAGAGAGGAAAGCGGAATTCCTAGTGTAGCGGTGAAATGCGTAGATATTAGGAGGAACACCAGTGGCGAAGGCGGCTTTCTGGCTCATAACTGACGCTGAGGCGCGAAAGCGTGGGGAGCAAAC"
## [40] "GCGAGCGTTGTCCGGATTTACTGGGTGTAAAGGGTGCGTAGGCGGCTAGACAAGTCAGGTGTGAAATACCGCAGCTCAACTGCGGGGCTGCACTTGAAACTGTAGAGCTTGAGTGATGGAGAGGTAAGCGGAATTCCTAGTGTAGCGGTAAAATGCGTAGATATTAGGAGGAACACCAGTGGCGAAGGCGGCTTACTGGACATTAACTGACGCTGAGGCACGAAAGCGTGGGGAGCAAAC"
## [41] "GCAAGCGTTATCCGGATTTACTGGGTGTAAAGGGAGCGTAGACGGCCGTGCAAGTCTGATGTGAAAGGCTGGGGCTCAACCCCGGGACTGCATTGGAAACTGTATGGCTGGAGTGCCGGAGAGGTAAGCGGAATTCCTAGTGTAGCGGTGAAATGCGTAGATATTAGGAGGAACACCAGTGGCGAAGGCGGCTTACTGGACGGTAACTGACGTTGAGGCTCGAAAGCGTGGGGAGCAAAC"
## [42] "GCAAGCGTTATCCGGATCTACTGGGTGTAAAGGGAGCGTAGACGGATGGACAAGTCTGATGTGAAAGGCTGGGGCTCAACCCCGGGACTGCATTGGAAACTGCCCGTCTTGAGTGCCGGAGAGGTAAGCGGAATTCCTAGTGTAGCGGTGAAATGCGTAGATATTAGGAGGAACACCAGTGGCGAAGGCGGCTTACTGGACGGTAACTGACGTTGAGGCTCGAAAGCGTGGGGAGCAAAC"
## [43] "GCAAGCGTTATCCGGATTTACTGGGTGTAAAGGGAGCGTAGACGGATGGACAAGTCTGATGTGAAAGGCTGGGGCTCAACCCCGGGACTGCATTGGAAACTGCCCGTCTTGAGTGCCGGAGAGGTAAGCGGAATTCCTAGTGTAGCGGTGAAATGCGTAGATATACGGAGGAACACCAGTGGCGAAGGCGGCCTGCTGGACATTAACTGACGCTGAGGCGCGAAAGCGTGGGGAGCAAAC"
## [44] "GCAAGCGTTATCCGGATTTACTGGGTGTAAAGGGAGCGCAGGCGGCATGATAAGTCTGATGTGAAAACCCAAGGCTCAACCATGGGACTGCATTGGAAACTGTCGTGCTGGAGTGTCGGAGAGGTAAGCGGAATTCCTAGTGTAGCGGTGAAATGCGTAGATATTAGGAGGAACACCAGTGGCGAAGGCGGCTTACTGGACGGTAACTGACGTTGAGGCTCGAAAGCGTGGGGAGCAAAC"
## [45] "GCAAGCGTTATCCGGATTTACTGGGTGTAAAGGGAGCGTAGGCGGTCCTGCAAGTCTGATGTGAAAGGCCGGGGCTCAACCCCGGGACTGCATTGGAAACTGTAGGACTAGAGTGTCGGAGGGGTAAGTGGAATTCCTAGTGTAGCGGTGAAATGCGTAGATATTAGGAGGAACACCAGTGGCGAAGGCGGCTTACTGGACGGTAACTGACGTTGAGGCTCGAAAGCGTGGGGAGCAAAC"
## [46] "GCAAGCGTTATCCGGATTTACTGGGTGTAAAGGGAGCGTAGGCGGTCCTGCAAGTCTGATGTGAAAACCCGGGGCTCAACCCCGGGACTGCATTGGAAACTGTAGGACTAGAGTGTCGGAGGGGTAAGTGGAATTCCTAGTGTAGCGGTGAAATGCGTAGATATTAGGAGGAACACCAGTGGCGAAGGCGGCTTACTGGACGACCACTGACGCTGAGGCTCGAAAGCGTGGGGAGCAAAC"
## [47] "GCAAGCGTTATCCGGATTTACTGGGTGTAAAGGGAGCGTAGACGGTATGGCAAGTCTGATGTGAAAGGCCAGGGCTCAACCCTGGGACTGCATTGGAAACTGTCGAACTAGAGTGTCGGAGAGGCAAGTGGAATTCCTAGTGTAGCGGTGAAATGCGTAGATATTAGGAGGAACACCAGTGGCGAAGGCGGCTTACTGGACGGTAACTGACGTTGAGGCTCGAAAGCGTGGGGAGCAAAC"
## [48] "GCAAGCGTTATCCGGATTTACTGGGTGTAAAGGGAGCGCAGACGGCACTGCAAGTCTGAAGTGAAAGCCCGGGGCTCAACCCCGGGACTGCTTTGGAAACTGTAGAGCTAGAGTGCTGGAGAGGCAAGCGGAATTCCTAGTGTAGCGGTGAAATGCGTAGATATTAGGAGGAACACCAGTGGCGAAGGCGGCTTACTGGACGGTAACTGACGTTGAGGCTCGAAAGCGTGGGGAGCAAAC"
## [49] "GCAAGCGTTATCCGGATTTACTGGGTGTAAAGGGAGCGTAGACGGCATGGCAAGTCTGAAGTGAAATGCGGGGGCTCAACCCCTGAACTGCTTTGGAAACTGTCAGGCTGGAGTGCAGGAGAGGTAAGTGGAATTCCTAGTGTAGCGGTGAAATGCGTAGATATTAGGAGGAACACCAGTGGCGAAGGCGGCTTACTGGACTGTAACTGACGTTGAGGCTCGAAAGCGTGGGGAGCAAAC"
## [50] "GCAAGCGTTATCCGGATTTACTGGGTGTAAAGGGTGCGTAGGTGGTGAGACAAGTCTGAAGTGAAAATCCGGGGCTTAACCCCGGAACTGCTTTGGAAACTGCCTGACTAGAGTACAGGAGAGGTAAGTGGAATTCCTAGTGTAGCGGTGAAATGCGTAGATATTAGGAGGAACACCAGTGGCGAAGGCGACTTACTGGACTGCTACTGACACTGAGGCACGAAAGCGTGGGGAGCAAAC"
## [51] "GCAAGCGTTATCCGGATTTACTGGGTGTAAAGGGAGCGTAGACGGCATGGCAAGCCAGATGTGAAAGCCCGGGGCTCAACCCCGGGACTGCATTTGGAACTGTCAGGCTAGAGTGTCGGAGAGGAAAGCGGAATTCCTAGTGTAGCGGTGAAATGCGTAGATATTAGGAGGAACACCAGTGGCGAAGGCGGCTTACTGGACGGTAACTGACGTTGAGGCTCGAAAGCGTGGGGAGCAAAC"
## [52] "GCAAGCGTTATCCGGAATTACTGGGTGTAAAGGGTGAGTAGGCGGCACGGCAAGTAAGATGTGAAAGCCCGAGGCTTAACCTCGGGATTGCATTTTAAACTGCTGAGCTAGAGTACAGGAGAGGAAAGCGGAATTCCTAGTGTAGCGGTGAAATGCGTAGATATTAGGAAGAACACCAGTGGCGAAGGCGGCTTTCTGGACTGAAACTGACGCTGAGGCACGAAAGCGTGGGGAGCGAAC"
## [53] "GCAAGCGTTATCCGGATTTACTGGGTGTAAAGGGAGCGTAGGTGGCAAGGCAAGCCAGAAGTGAAAACCCGGGGCTCAACCGCGGGATTGCTTTTGGAACTGTCATGCTAGAGTGCAGGAGGGGTGAGCGGAATTCCTAGTGTAGCGGTGAAATGCGTAGATATTAGGAGGAACACCAGTGGCGAAGGCGGCTTACTGGACGATAACTGACGCTGAGGCTCGAAAGCGTGGGGAGCAAAC"
## 
## $y
##  [1] "GCAAGCGTTGTCCGGATTTACTGGGTGTAAAGGGCGTGCAGCCGGGCATGCAAGTCAGATGTGAAATCTCAGGGCTTAACCCTGAAACTGCATTTGAAACTGTATGTCTTGAGTGCCGGAGAGGTAATCGGAATTCCTTGTGTAGCGGTGAAATGCGTAGATATAAGGAAGAACACCAGTGGCGAAGGCGGATTACTGGACGGTAACTGACGGTGAGGCGCGAAAGCGTGGGGAGCGAAC"
##  [2] "GCGAGCGTTGTCCGGAATTACTGGGTGTAAAGGGAGCGTAGGCGGGATCTTAAGTCAGGTGTGAAAACTATGGGCTCAACCCATAGACTGCACTTGAAACTGAGGTTCTTGAGTGAAGTAGAGGCAGGCGGAATTCCTAGTGTAGCGGTGAAATGCGTAGATATTAGGAGGAACATCAGTGGCGAAGGCGGCCTGCTGGGCTTTTACTGACGCTGAGGCTCGAAAGCGTGGGGAGCAAAC"
##  [3] "GCGAGCGTTGTCCGGAATTACTGGGTGTAAAGGGAGCGTAGGCGGGATGGCAAGTCAGATGTGAAAACTATGGGCTCAACCCATAGACTGCATTTGAAACTGTTGTTCTTGAGTGAGGTAGAGGTAAGCGGAATTCCTGGTGTAGCGGTGAAATGCGTAGAGATCAGGAGGAACATCGGTGGCGAAGGCGGCTTACTGGGCCTTTACTGACGCTGAGGCTCGAAAGCGTGGGGAGCAAAC"
##  [4] "GCGAGCGTTGTCCGGAATTACTGGGTGTAAAGGGAGCGTAGGCGGGATTGCAAGTCAGATGTGAAAACTATGGGCTTAACCCATAGACTGCATTTGAAACTGTAGTTCTTGAGTGAAGTAGAGGTAAGCGGAATTCCTAGTGTAGCGGTGAAATGCGTAGATATTAGGAGGAACATCGGTGGCGAAGGCGGCTTACTGGGCTTTTACTGACGCTGAGGCTCGAAAGCGTGGGGAGCAAAC"
##  [5] "ACAAGCGTTGTCCGGAATTACTGGGTGTAAAGGGAGCGCAGGCGGGCGATCAAGTTGGAAGTGAAATCCATGGGCTCAACCCATGAACTGCTTTCAAAACTGGTCGTCTTGAGTAGTGCAGAGGTAGGCGGAATTCCCGGTGTAGCGGTGGAATGCGTAGATATCGGGAGGAACACCAGTGGCGAAGGCGGCCTACTGGGCACCAACTGACGCTGAGGCTCGAAAGTGTGGGTAGCAAAC"
##  [6] "GCAAGCGTTGTCCGGAATTACTGGGTGTAAAGGGAGCGCAGGCGGAAGGACAAGTTGGAAGTGAAACCCACGGGCTCAACCCGTGAACTGCTTTCAAAACTGTTTTTCTTGAGTGGTGTAGAGGTAGGCGGAATTCCCGGTGTAGCGGTGGAATGCGTAGATATCGGGAGGAACACCAGTGGCGAAGGCGGCCTACTGGGCACTAACTGACGCTGAGGCTCGAAAGCATGGGTAGCAAAC"
##  [7] "ACAAGCGTTGTCCGGAATTACTGGGTGTAAAGGGAGCGCAGGCGGGAAGACAAGTTGGAAGTGAAATCCATGGGCTCAACCCATGAACTGCTTTCAAAACTGTTTTTCTTGAGTAGTGCAGAGGTAGGCGGAATTCCCGGTGTAGCGGTGGAATGCGTAGATATCGGGAGGAACACCAGTGGCGAAGGCGGCCTACTGGGCACCAACTGACGCTGAGGCTCGAAAGTGTGGGTAGCAAAC"
##  [8] "GCAAGCGTTGTCCGGATTTACTGGGTGTAAAGGGCGTGTAGGCGGAGATGCAAGTCGGGAGTGAAATCCATGGGCTCAACCCATGAACTGCTTTCGAAACTGTATCCCTTGAGTATCGGAGAGGCAAGCGGAATTCCTAGTGTAGCGGTGAAATGCGTAGATATTAGGAGGAACACCAGTGGCGAAGGCGGCTTGCTGGACGACAACTGACGCTGAGGCGCGAAAGCGTGGGGAGCAAAC"
##  [9] "GCGAGCGTTGTCCGGATTTACTGGGTGTAAAGGGCGTGTAGGCGGAGATGCAAGTTGGGAGTGAAATCCATGGGCTCAACCCATGAACTGCTTCCAAAACTGTATCCCTTGAGTATCGGAGAGGCAAGCGGAATTCCTAGTGTAGCGGTGAAATGCGTAGATATTAGGAGGAACACCAGTGGCGAAGGCGGCTTGCTGGACGACAACTGACGCTGAGGCGCGAAAGCGTGGGGAGCAAAC"
## [10] "GCAAGCGTTGTCCGGATTTACTGGGTGTAAAGGGCGTGTAGGCGGAGATGCAAGTTAGGAGTGAAATCTATGGGCTCAACCCATAAACTGCTTCTAAAACTGTATCCCTTGAGTATCGGAGAGGCAAGCGGAATTCCTAGTGTAGCGGTGAAATGCGTAGATATTAGGAGGAACACCAGTGGCGAAGGCGGCTTGCTGGACGACAACTGACGCTGAGGCGCGAAAGCGTGGGGAGCAAAC"
## [11] "GCAAGCGTTGTCCGGATTTACTGGGTGTAAAGGGCGTGCAGCCGGGCCGGCAAGTCAGATGTGAAATCTGGAGGCTTAACCTCCAAACTGCATTTGAAACTGTAGGTCTTGAGTACCGGAGAGGTTATCGGAATTCCTTGTGTAGCGGTGAAATGCGTAGATATAAGGAAGAACACCAGTGGCGAAGGCGGATAACTGGACGGCAACTGACGGTGAGGCGCGAAAGCGTGGGGAGCAAAC"
## [12] "GCAAGCGTTGTCCGGATTTACTGGGTGTAAAGGGCGTGCAGCCGGGTCTGCAAGTCAGATGTGAAATCCATGGGCTCAACCCATGAACTGCATTTGAAACTGTAGATCTTGAGTGTCGGAGGGGCAATCGGAATTCCTAGTGTAGCGGTGAAATGCGTAGATATTAGGAGGAACACCAGTGGCGAAGGCGGATTGCTGGACGATAACTGACGGTGAGGCGCGAAAGTGTGGGGAGCAAAC"
## [13] "GCAAGCGTTGTCCGGATTTACTGGGTGTAAAGGGCGTGCAGCCGGGAATGCAAGTCAGATGTGAAATCCATGGGCTTAACCCATGAACTGCATTTGAAACTGTATTTCTTGAGTACTGGAGAGGCAATCGGAATTCCTAGTGTAGCGGTGAAATGCGTAGATATTAGGAGGAACACCAGTGGCGAAGGCGGATTGCTGGACAGCAACTGACGGTGAGGCGCGAAAGTGTGGGGAGCAAAC"
## [14] "GCAAGCGTTATCCGGATTTACTGGGTGTAAAGGGCGCGTAGGCGGGGATGCAAGTCAGATGTGAAATCTATGGGCTTAACCCATAAACTGCATTTGAAACTGTATCTCTTGAGTGCTGGAGAGGTAGACGGAATTCCTTGTGTAGCGGTGAAATGCGTAGATATAAGGAAGAACACCAGTGGCGAAGGCGGTCTACTGGACAGTAACTGACGCTGAGGCGCGAGAGCGTGGGGAGCAAAC"
## [15] "GCAAGCGTTATCCGGATTTACTGGGTGTAAAGGGCGTGTAGGCGGGAAAGCAAGTCAGATGTGAAAACTGTGGGCTCAACCCACAGCCTGCATTTGAAACTGTTTTTCTTGAGTACTGGAGAGGCAGATGGAATTCCTAGTGTAGCGGTGAAATGCGTAGATATTAGGAGGAACACCAGTGGCGAAGGCGATCTGCTGGACAGCAACTGACGCTGAGGCGCGAAAGCGTGGGGAGCAAAC"
## [16] "GCAAGCGTTAATCGGAATAACTGGGCGTAAAGGGCATGCAGGCGGTTCATCAAGTAGGATGTGAAATCCCCGGGCTCAACCTGGGAACAGCATACTAAACTGGTGGACTAGAGTATTGCAGGGGGAGACGGAATTCCAGGTGTAGCGGTGGAATGCGTAGATATCTGGAAGAACACCAAAGGCGAAGGCAGTCTCCTGGGCAAATACTGACGCTCATATGCGAAAGCGTGGGTAGCAAAC"
## [17] "GCGAGCGTTGTTCGGAATTACTGGGCGTAAAGCGCACGCAGGCGGTTCGTTAAGTCTGCTGTCAAAGGCTGAGGCTCAACCTCAGTTCTACAACAGATACTGGCGGACTAGAGTATGTGAGAGGGAAGTGGAATTCCCGGTGTAGCGGTGAAATGCGTAGATATCGGGAGGAACACCAGTGGCGAAGGCGGCTTCCTGGCACACTACTGACGCTCATGTGCGAAAGCCAGGGCAGCGAAC"
## [18] "GCGAGCGTTATCCGGATTTATTGGGTTTAAAGGGAGCGCAGACGGGACTTTAAGTCAGCTGTGAAATTTTCCGGCTCAACCGGGAAACTGCAGTTGATACTGGCGTCCTTGAGTACGGTCGAGGCAGGCGGAATTCGTGGTGTAGCGGTGAAATGCTTAGATATCACGAAGAACCCCGATTGCGAAGGCAGCCTGCCAGACCGCAACTGACGTTCATGCTCGAAAGTGCGGGTATCAAAC"
## [19] "GCGAGCGTTATCCGGATTTATTGGGTTTAAAGGGAGCGTAGGCGGGCTGTTAAGTCAGCGGTCAAATGTCAGGGCCCAACCTTGGCATGCCGTTGATACTGGCGGCCTTGAGTTCACACAAGGAAGGTGGAATTCGTCGTGTAGCGGTGAAATGCTTAGATATGACGAAGAACTCCGATTGCGAAGGCAGCCTTCTGGGGTGTTACTGACGCTGAGGCTCGAAAGTGCGGGAATCAAACA"
## [20] "GCAAGCGTTGTCCGGAATTATTGGGCGTAAAGGGCGCGCAGGCGGCGTCGTAAGTCGGTCTTAAAAGTGCGGGGCTTAACCCCGTGAGGGGACCGAAACTGCGATGCTAGAGTATCGGAGAGGAAAGCGGAATTCCTAGTGTAGCGGTGAAATGCGTAGATATTAGGAGGAACACCAGTGGCGAAAGCGGCTTTCTGGACGACAACTGACGCTGAGGCGCGAAAGCCAGGGGAGCAAACG"
## [21] "GCAAGCGTTGTCCGGAATTATTGGGCGTAAAGGGAGCGCAGGCGGGAAACTAAGCGGATCTTAAAAGTGCGGGGCTCAACCCCGTGATGGGGTCCGAACTGGTTTTCTTGAGTGCAGGAGAGGAAAGCGGAATTCCCAGTGTAGCGGTGAAATGCGTAGATATTGGGAAGAACACCAGTGGCGAAGGCGGCTTTCTGGACTGTAACTGACGCTGAGGCTCGAAAGCTAGGGTAGCGAACG"
## [22] "GCGAGCGTTGTCCGGATTTACTGGGCGTAAAGGGAGCGTAGGCGGATTTTTAAGTGAGATGTGAAATACTCGGGCTTAACCTGAGTGCTGCATTTCAAACTGGAAGTCTAGAGTGCAGGAGAGGAGAAGGGAATTCCTAGTGTAGCGGTGAAATGCGTAGAGATTAGGAAGAACACCAGTGGCGAAGGCGCTTCTCTGGACTGTAACTGACGCTGAGGCTCGAAAGCGTGGGGAGCAAAC"
## [23] "GCAAGCGTTATCCGGATTTACTGGGTGTAAAGGGAGCGTAGACGGCCGTGCAAGTCTGATGTGAAAGGCTGGGGCTCAACCCCGGGACTGCATTGGAAACTGTATGGCTGGAGTGCCGGAGAGGTAAGCGGAATTCCTAGTGTAGCGGTGAAATGCGTAGATATTAGGAGGAACACCAGTGGCGAAGGCGGCTTACTGGACGGTAACTGACGTTGAGGCTCGAAAGCGTGGGGAGCAAAC"
## [24] "GCAAGCGTTATCCGGATTTACTGGGTGTAAAGGGAGCGTAGACGGCGAAGCAAGTCTGAAGTGAAAACCCAGGGCTCAACCCTGGGACTGCTTTGGAAACTGTTTTGCTAGAGTGTCGGAGAGGTAAGTGGAATTCCTAGTGTAGCGGTGAAATGCGTAGATATTAGGAGGAACACCAGTGGCGAAGGCGGCTTACTGGACGATAACTGACGTTGAGGCTCGAAAGCGTGGGGAGCAAAC"
## [25] "GCAAGCGTTATCCGGATTTACTGGGTGTAAAGGGAGCGCAGACGGCACTGCAAGTCTGAAGTGAAAGCCCGGGGCTCAACCCCGGGACTGCTTTGGAAACTGTAGAGCTAGAGTGCTGGAGAGGCAAGCGGAATTCCTAGTGTAGCGGTGAAATGCGTAGATATTAGGAGGAACACCAGTGGCGAAGGCGGCTTACTGGACGGTAACTGACGTTGAGGCTCGAAAGCGTGGGGAGCAAAC"
## [26] "GCAAGCGTTATCCGGAATTACTGGGTGTAAAGGGTGCGTAGGCGGTATGGCAAGTTTGATGTGAAACCCACAGGCTTAACCTGTGGCTTGCATCGAAAACTACTGAACTAGAGTGCAGGAGAGGAAAGCGGAATTCCTAGTGTAGCGGTGAAATGCGTAGATATTAGGAAGAACACCAGTGGCGAAGGCGGCTTTCTGGACTGCAACTGACGCTGAGGCACGAAAGCGTGGGGAGCAAAC"
## 
## $z
##   [1] "GCAAGCGTTATCCGGATTTATTGGGTGTAAAGGGTGCGTAGACGGGAATACAAGTTAGTTGTGAAATCCCTCGGCTTAACTGAGGAACTGCAACTAAAACTATATTTCTTGAGTGCTGGAGAGGAAAGTGGAATTCCTAGTGTAGCGGTGAAATGCGTAGATATTAGGAGGAACACCAGTGGCGAAGGCGACTTTCTGGACAGTAACTGACGTTGAGGCACGAAAGTGTGGGGAGCAAAC"
##   [2] "GCGAGCGTTATCCGGATTTATTGGGTGTAAAGGGTGCGTAGACGGGAAATTAAGTTAGTTGTGAAATCCCTCGGCTCAACTGAGGAACTGCAACTAAAACTGATTTTCTTGAGTACTGGAGAGGAAAGTGGAATTCCTAGTGTAGCGGTGAAATGCGTAGATATTAGGAGGAACACCAGTGGCGAAGGCGACTTTCTGGACAGAAACTGACGTTGAGGCACGAAAGTGTGGGGAGCAAAC"
##   [3] "GCAAGCGTTATCCGGATTTATTGGGTGTAAAGGGTGCGTAGACGGGAAATTAAGTTAGTTGTGAAATCCCTCGGCTCAACTGAGGAACTGCAACTAAAACTGGTTTTCTTGAGTGCAGGAGAGGTAAGTGGAATTCCTAGTGTAGCGGTGAAATGCGTAGATATTAGGAGGAACACCAGTGGCGAAGGCGACTTACTGGACTGTAACTGACGTTGAGGCACGAAAGTGTGGGGAGCAAAC"
##   [4] "GCAAGCGTTGTCCGGATTTACTGGGTGTAAAGGGCGTGTAGGCGGGCATGCAAGTTGGATGTGAAATGTCACGGCTTAACCGTGGAGCTGCATCCAAAACTGCAAGTCTTGAGTGCCGGAGAGGAAAGCGGAATTCCTAGTGTAGCGGTGAAATGCGTAGATATTAGGAGGAACACCGGTGGCGAAGGCGGCTTTCTGGACGGTAACTGACGCTGAGGCGCGAAAGCGTGGGGAGCAAAC"
##   [5] "GCGAGCGTTGTCCGGAATGACTGGGTGTAAAGGGAGCGTAGGCGGGATGGCAAGTCAGATGTGAAACCTGAGGGCTCAACCTTCAGACTGCATTTGAAACTGCTGTTCTTGAGTGAAGTAGAGGTAAGCGGAATTCCTGGTGTAGCGGTGAAATGCGTAGAGATCAGGAGGAACATCGGTGGCGAAGGCGGCTTACTGGGCTTTTACTGACGCTGAGGCTCGAAAGCGTGGGGAGCAAAC"
##   [6] "GCAAGCGTTGTCCGGATTTACTGGGTGTAAAGGGAGCGCAGGCGGGAGAGCAAGTCAGCGGTGAAATACATGGGCTTAACCCATGGGCTGCCGTTGAAACTGTCCTTCTTGAGTGAAGTAGAGGCAAGCGGAATTCCGAGTGTAGCGGTGAAATGCGTAGATATTCGGAGGAACACCAGTGGCGAAGGCGGCTTGCTGGGCTTTTACTGACGCTGAGGCTCGAAAGTGTGGGGAGCAAAC"
##   [7] "GCAAGCGTTGTCCGGAATTACTGGGTGTAAAGGGAGCGTAGGCGGGAAGATAAGTTGGACGTCTAATCTATCGGCTCAACCGATAGTCGCGTTCAAAACTGTTTTTCTTGAGTGAAGTAGAGGTAAGCGGAATTCCTAGTGTAGCGGTGAAATGCGTAAATATTAGGAGGAACACCAGTGGCGAAGGCGGCTTACTGGGCTTTAACTGACGCTGAGGCTCGAAAGCGTGGGTAGCAAACA"
##   [8] "ACAAGCGTTGTCCGGAATTACTGGGTGTAAAGGGAGCGCAGGCGGGAGAACAAGTTGGAAGTGAAATCCATGGGCTCAACCCATGAACTGCTTTCAAAACTGTTTTTCTTGAGTAGTGCAGAGGTAGGCGGAATTCCCGGTGTAGCGGTGGAATGCGTAGATATCGGGAGGAACACCAGTGGCGAAGGCGGCCTACTGGGCACCAACTGACGCTGAGGCTCGAAAGTGTGGGTAGCAAAC"
##   [9] "ACAAGCGTTGTCCGGAATTACTGGGTGTAAAGGGAGCGCAGGCGGGAAGGCAAGTTGGAAGTGAAATCCATGGGCTCAACCCATGAACTGCTTTCAAAACTGTTTTTCTTGAGTAGTGCAGAGGTAGGCGGAATTCCCGGTGTAGCGGTGGAATGCGTAGATATCGGGAGGAACACCAGTGGCGAAGGCGGCCTACTGGGCACCAACTGACGCTGAGGCTCGAAAGTGTGGGTAGCAAAC"
##  [10] "ACAAGCGTTGTCCGGAATTACTGGGTGTAAAGGGAGCGCAGGCGGGAAAGCAAGTTGGAAGTGAAATCCATGGGCTCAACCCATGAACTGCTTTCAAAACTGTTTTTCTTGAGTAGTGCAGAGGTAGGCGGAATTCCCGGTGTAGCGGTGGAATGCGTAGATATCGGGAGGAACACCAGTGGCGAAGGCGGCCTACTGGGCACCAACTGACGCTGAGGCTCGAAAGTGTGGGTAGCAAAC"
##  [11] "ACAAGCGTTGTCCGGAATTACTGGGTGTAAAGGGAGCGCAGGCGGGCGATCAAGTTGGAAGTGAAATCCATGGGCTCAACCCATGAACTGCTTTCAAAACTGGTCGTCTTGAGTAGTGCAGAGGTAGGCGGAATTCCCGGTGTAGCGGTGGAATGCGTAGATATCGGGAGGAACACCAGTGGCGAAGGCGGCCTACTGGGCACCAACTGACGCTGAGGCTCGAAAGTGTGGGTAGCAAAC"
##  [12] "GCAAGCGTTGTCCGGAATTACTGGGTGTAAAGGGAGCGCAGGCGGGAAGACAAGTTGGAAGTGAAAACCATGGGCTCAACCCATGAATTGCTTTCAAAACTGCTGGCCTTGAGTAGTGCAGAGGTAGGTGGAATTCCCGGTGTAGCGGTGGAATGCGTAGATATCGGGAGGAACACCAGTGGCGAAGGCGGTCTACTGGGCACCAACTGACGCTGAGGCTCGAAAGCATGGGTAGCAAAC"
##  [13] "GCAAGCGTTGTCCGGAATTACTGGGTGTAAAGGGAGCGCAGGCGGGAAGACAAGTTGGAAGTGAAAACCATGGGCTCAACCCATGAATTGCTTTCAAAACTGTTTTTCTTGAGTAGTGCAGAGGTAGATGGAATTCCCGGTGTAGCGGTGGAATGCGTAGATATCGGGAGGAACACCAGTGGCGAAGGCGGTCTACTGGGCACCAACTGACGCTGAGGCTCGAAAGCATGGGTAGCAAAC"
##  [14] "GCAAGCGTTGTCCGGAATTACTGGGTGTAAAGGGAGCGCAGGCGGACCGGCAAGTTGGAAGTGAAAACTATGGGCTCAACCCATAAATTGCTTTCAAAACTGCTGGCCTTGAGTAGTGCAGAGGTAGGTGGAATTCCCGGTGTAGCGGTGGAATGCGTAGATATCGGGAGGAACACCAGTGGCGAAGGCGACCTACTGGGCACCAACTGACGCTGAGGCTCGAAAGCATGGGTAGCAAAC"
##  [15] "GCAAGCGTTGTCCGGAATTACTGGGTGTAAAGGGAGCGCAGGCGGACCGGCAAGTTGGAAGTGAAATCCATGGGCTCAACCCGTGAATTGCTTTCAAAACTGCTGGCCTTGAGTAGTGCAGAGGTAGGTGGAATTCCCGGTGTAGCGGTGGAATGCGTAGATATCGGGAGGAACACCAGTGGCGAAGGCGACCTACTGGGCACCAACTGACGCTGAGGCTCGAAAGCATGGGTAGCAAAC"
##  [16] "GCAAGCGTTGTCCGGAATTACTGGGTGTAAAGGGAGCGCAGGCGGACCGGCAAGTTGGAAGTGAAAACCATGGGCTCAACCCGTGAATTGCTTTCAAAACTGCTGGCCTTGAGTAGTGCAGAGGTAGGTGGAATTCCCGGTGTAGCGGTGGAATGCGTAGATATCGGGAGGAACACCAGTGGCGAAGGCGACCTACTGGGCACCAACTGACGCTGAGGCTCGAAAGCATGGGTAGCAAAC"
##  [17] "GCAAGCGTTGTCCGGAATTACTGGGTGTAAAGGGAGCGCAGGCGGAAGGACAAGTTGGAAGTGAAACCCACGGGCTCAACCCGTGAACTGCTTTCAAAACTGTTTTTCTTGAGTGGTGTAGAGGTAGGCGGAATTCCCGGTGTAGCGGTGGAATGCGTAGATATCGGGAGGAACACCAGTGGCGAAGGCGGCCTACTGGGCACTAACTGACGCTGAGGCTCGAAAGCATGGGTAGCAAAC"
##  [18] "ACAAGCGTTGTCCGGAATTACTGGGTGTAAAGGGAGCGCAGGCGGGAAGACAAGTTGGAAGTGAAATCCATGGGCTCAACCCATGAACTGCTTTCAAAACTGTTTTTCTTGAGTAGTGCAGAGGTAGGCGGAATTCCCGGTGTAGCGGTGGAATGCGTAGATATCGGGAGGAACACCAGTGGCGAAGGCGGCCTACTGGGCACCAACTGACGCTGAGGCTCGAAAGTGTGGGTAGCAAAC"
##  [19] "GCAAGCGTTGTCCGGAATTACTGGGTGTAAAGGGAGCGCAGGCGGGCATGCAAGTTGGAAGTGAAAACTATGGGCTCAACCCATAGCCTGCTTTCAAAACTGCGTGTCTTGAGTAGTGCAGAGGTAGGCGGAATTCCCGGTGTAGCGGTGGAATGCGTAGATATCGGGAGGAACACCAGTGGCGAAGGCGGCCTACTGGGCACCAACTGACGCTGAGGCTCGAAAGCATGGGTAGCAAAC"
##  [20] "GCAAGCGTTGTCCGGATTTACTGGGTGTAAAGGGCGTGTAGGCGGAGATGCAAGTCGGGAGTGAAATCCATGGGCTCAACCCATGAACTGCTTTCGAAACTGTATCCCTTGAGTATCGGAGAGGCAAGCGGAATTCCTAGTGTAGCGGTGAAATGCGTAGATATTAGGAGGAACACCAGTGGCGAAGGCGGCTTGCTGGACGACAACTGACGCTGAGGCGCGAAAGCGTGGGGAGCAAAC"
##  [21] "GCGAGCGTTGTCCGGATTTACTGGGTGTAAAGGGCGTGTAGGCGGAGATGCAAGTTGGGAGTGAAATCCATGGGCTCAACCCATGAACTGCTTCCAAAACTGTATCCCTTGAGTATCGGAGAGGCAAGCGGAATTCCTAGTGTAGCGGTGAAATGCGTAGATATTAGGAGGAACACCAGTGGCGAAGGCGGCTTGCTGGACGACAACTGACGCTGAGGCGCGAAAGCGTGGGGAGCAAAC"
##  [22] "GCAAGCGTTATCCGGATTTACTGGGTGTAAAGGGCGTGTAGGCGGGAAAGCAAGTCAGATGTGAAAACTGTGGGCTCAACCCACAGCCTGCATTTGAAACTGTTTTTCTTGAGTACTGGAGAGGCAGATGGAATTCCTAGTGTAGCGGTGAAATGCGTAGATATTAGGAGGAACACCAGTGGCGAAGGCGATCTGCTGGACAGCAACTGACGCTGAGGCGCGAAAGCGTGGGGAGCAAAC"
##  [23] "GCGAGCGTTATCCGGAATTACTGGGTGTAAAGGGTGTGTAGGCGGGAAGGCAAGTCAGATGTGAAAACCAAAGGCTCAACCTTTGGCTTGCATTTGAAACTGTTTTTCTTGAGAGTGGGAGAGGTAAACGGAATTCCTAGTGTAGTAGTGAAATGCGTAGATATTAGGAGGAACACCGGTGGCGAAGGCGGTTTACTGGACCACAACTGACGCTGAGACACGAAAGCGTGGGGAGCAAAC"
##  [24] "TCTAGTGGTAGCAGTTTTTATTGGGCCTAAAGCGTCCGTAGCCGGTTTAATAAGTCTCTGGTGAAATCCTGCAGCTTAACTGTGGGAATTGCTGGAGATACTATTAGACTTGAGATCGGGAGAGGTTAGAGGTACTCCCAGGGTAGAGGTGAAATTCTGTAATCCTGGGAGGACCGCCTGTTGCGAAGGCGTCTGACTGGAACGATTCTGACGGTGAGGGACGAAAGCTAGGGGCGCGAA"
##  [25] "GCAAGCGTTATCCGGATTTACTGGGTGTAAAGGGCGAGTAGGCGGATTGGCAAGTTGGGAGTGAAATGTCGGGGCTTAACCCCGGAACTGCTTCCAAAACTGTTGATCTTGAGTGATGGAGAGGCAGGCGGAATTCCCAGTGTAGCGGTGAAATGCGTAGATATTGGGAGGAACACCAGTGGCGAAGGCGGCCTGCTGGACATTAACTGACGCTGAGGAGCGAAAGCGTGGGGAGCAAAC"
##  [26] "GCAAGCGTTATCCGGATTTACTGGGTGTAAAGGGCGCGCAGGCGGGCCGGCAAGTTGGAAGTGAAATCCGGGGGCTTAACCCCCGAACTGCTTTCAAAACTGCTGGTCTTGAGTGATGGAGAGGCAGGCGGAATTCCGTGTGTAGCGGTGAAATGCGTAGATATACGGAGGAACACCAGTGGCGAAGGCGGCCTGCTGGACATTAACTGACGCTGAGGCGCGAAAGCGTGGGGAGCAAAC"
##  [27] "GCAAGCGTTAATCGGAATAACTGGGCGTAAAGCGCACGTAGGTGGTTCGACAAGTCAGATGTGAAAGCCCCGGGCTTAACCTGGGATGTGCATTTGAAACTGTTGGACTCGAGTACTGTAGAGGGAGGTAGAATTCCAGGTGTAGCGGTGAAATGCGTAGATATCTGGAGGAATACCAGTGGCGAAGGCGGCCTCCTGGACAGACACTGACACTGAGGTGCGAAAGCGTGGGGAGCAAAC"
##  [28] "GCAAGCGTTAATCGGAATAACTGGGCGTAAAGGGCATGCAGGCGGTTCATCAAGTAGGATGTGAAATCCCCGGGCTCAACCTGGGAACAGCATACTAAACTGGTGGACTAGAGTATTGCAGGGGGAGACGGAATTCCAGGTGTAGCGGTGGAATGCGTAGATATCTGGAAGAACACCAAAGGCGAAGGCAGTCTCCTGGGCAAATACTGACGCTCATATGCGAAAGCGTGGGTAGCAAAC"
##  [29] "GCAAGCGTTAATCGGAATCACTGGGCGTAAAGCGCACGTAGGCTGTTATGTAAGTCAGGGGTGAAATCCCACGGCTCAACCGTGGAACTGCCCTTGATACTGCACGACTTGAATCCGGGAGAGGGTGGCGGAATTCCAGGTGTAGGAGTGAAATCCGTAGATATCTGGAGGAACATCAGTGGCGAAGGCGGCCACCTGGACCGGTATTGACGCTGAGGTGCGAAAGCGTGGGGAGCAAAC"
##  [30] "GCGAGCGTTGTCCGGAATTATTGGGCGTAAAGAGCTTGTAGGCGGTTTGTCGCGTCTGCTGTGAAAGGCCGGGGCTTAACCCCGTGTATTGCAGTGGGTACGGGCAGACTAGAGTGCAGTAGGGGAGACTGGAATTCCTGGTGTAGCGGTGGAATGCGCAGATATCAGGAGGAACACCGATGGCGAAGGCAGGTCTCTGGGCTGTAACTGACGCTGAGAAGCGAAAGCATGGGGAGCGAA"
##  [31] "GCAAGCGTTATCCGGAATTATTGGGCGTAAAGGGCTCGTAGGCGGTTCGTCGCGTCCGGTGTGAAAGTCCATCGCTTAACGGTGGATCCGCGCCGGGTACGGGCGGGCTTGAGTGCGGTAGGGGAGACTGGAATTCCCGGTGTAACGGTGGAATGTGTAGATATCGGGAAGAACACCAATGGCGAAGGCAGGTCTCTGGGCCGTTACTGACGCTGAGGAGCGAAAGCGTGGGGAGCGAAC"
##  [32] "GCAAGCGTTATCCGGAATTATTGGGCGTAAAGGGCTCGTAGGCGGTTCGTCGCGTCCGGTGTGAAAGTCCATCGCTTAACGGTGGATCTGCGCCGGGTACGGGCGGGCTGGAGTGCGGTAGGGGAGACTGGAATTCCCGGTGTAACGGTGGAATGTGTAGATATCGGGAAGAACACCGACGGCGAAGGCAGCTCTCTGGGCCGAAACTGACGCTGAGGCGCGAAAGCTGGGGGAGCGAAC"
##  [33] "GCAAGCGTTATCCGGAATTATTGGGCGTAAAGGGCTCGTAGGCGGTTCGTCGCGTCCGGTGTGAAAGTCCATCGCTTAACGGTGGATCTGCGCCGGGTACGGGCGGGCTGGAGTGCGGTAGGGGAGACTGGAATTCCCGGTGTAACGGTGGAATGTGTAGATATCGGGAAGAACACCAATGGCGAAGGCAGGTCTCTGGGCCGTTACTGACGCTGAGGAGCGAAAGCGTGGGGAGCGAAC"
##  [34] "GCGAGCGTTGTCCGGAATTATTGGGCGTAAAGGGCTTGTAGGCGGTTGGTCGCGTCTGCCGTGAAATCCTCTGGCTTAACTGGGGGCGTGCGGTGGGTACGGGCTGACTTGAGTGCGGTAGGGGAGACTGGAACTCCTGGTGTAGCGGTGGAATGCGCAGATATCAGGAAGAACACCGGTGGCGAAGGCGGGTCTCTGGGCCGTTACTGACGCTGAGGAGCGAAAGCGTGGGGAGCGAAC"
##  [35] "GCAAGCGTTGTCCGGAATCATTGGGCGTAAAGAGTTCGTAGGCGGTTTGTTAAGTCTGGTGTTAAAGCCCGAAGCTCAACTTCGGTTCGGCACTGGATACTGGCAGACTAGAATGCGGTAGAGGTAAAGGGAATTCCTGGTGTAGCGGTGAAATGCGTAGATATCAGGAGGAACATCGGTGGCGTAAGCGCTTTACTGGGCCGTAATTGACGCTGAGGAACGAAAGCCAGGGTAGCGAAT"
##  [36] "TCAAGCGTTGTTCGGAATCACTGGGCGTAAAGCGTGCGTAGGCTGTTTCGTAAGTCGTGTGTGAAAGGCGCGGGCTCAACCCGCGGACGGCACATGATACTGCGAGACTAGAGTAATGGAGGGGGAACCGGAATTCTCGGTGTAGCAGTGAAATGCGTAGATATCGAGAGGAACACTCGTGGCGAAGGCGGGTTCCTGGACATTAACTGACGCTGAGGCACGAAGGCCAGGGGAGCGAAA"
##  [37] "GCGAGCGTTGTTCGGAATCATTGGGCGTAAAGGGCATGTAGGCGGTTACGCAAGCCTGGTGTGAAAGCCCGAGGCTCAACCTCGGGATGCGCCGGGAACTGTGCAACTAGAGTAGCTGAGGGGCAGCCGGAATTCCAGGTGTAGGGGTGAAATCTGTAGATATCTGGAAGAACACCGATGGCGAAGGCAGGCTGCCAGCAGATTACTGACGCTGAGGTGCGAAGGTGCGGGGAGCGAACA"
##  [38] "GCGAGCGTTGTTCGGAATCATTGGGCGTAAAGGGCGTGTAGGCGGCCCTGCAAGCCTGGCGTGAAATCCCGGGGCCCAACCCCGGAACCGCGCTGGGAACTGCTGGGCTTGAGCCGCTGTGGCGCAGCCGGAATTCCAGGTGTAGGGGTGAAATCTGTAGATATCTGGAAGAACACCGATGGCGAAGGCAGGCTGCGAGCGGACGGCTGACGCTGAGGCGCGAAGGCGCGGGGAGCGAAC"
##  [39] "CCGAGCGTTATCCGGATTTATTGGGTTTAAAGGGAGCGTAGGTGGATTGTTAAGTCAGTTGTGAAAGTTTGCGGCTCAACCGTAAAATTGCAGTTGAAACTGGCAGTCTTGAGTACAGTAGAGGTGGGCGGAATTCGTGGTGTAGCGGTGAAATGCTTAGATATCACGAAGAACTCCGATTGCGAAGGCAGCTCACTAGACTGCAACTGACACTGATGCTCGAAAGTGTGGGTATCAAAC"
##  [40] "GCGAGCGTTATCCGGATTTATTGGGTTTAAAGGGAGCGCAGACGGGACTTTAAGTCAGCTGTGAAATTTTCCGGCTCAACCGGGAAACTGCAGTTGATACTGGCGTCCTTGAGTACGGTCGAGGCAGGCGGAATTCGTGGTGTAGCGGTGAAATGCTTAGATATCACGAAGAACCCCGATTGCGAAGGCAGCCTGCCAGACCGCAACTGACGTTCATGCTCGAAAGTGCGGGTATCAAAC"
##  [41] "GCGAGCGTTATCCGGATTTATTGGGTTTAAAGGGAGCGTAGGCGGGCTGTTAAGTCAGCGGTCAAATGTCAGGGCCCAACCTTGGCATGCCGTTGATACTGGCGGCCTTGAGTTCACACAAGGAAGGTGGAATTCGTCGTGTAGCGGTGAAATGCTTAGATATGACGAAGAACTCCGATTGCGAAGGCAGCCTTCTGGGGTGTTACTGACGCTGAGGCTCGAAAGTGCGGGAATCAAACA"
##  [42] "CCAGGCGTTATCCGGATTTATTGGGTTTAAAGGGAGCGCAGGCGGACCTTTAAGTCAGCTGTGAAATACGGCGGCTCAACCGTCGAACTGCAGTTGATACTGGAGGTCTTGAGTGCACACAGGGATACTGGAATTCATGGTGTAGCGGTGAAATGCTCAGATATCATGAAGAACTCCAATCGCGAAGGCAGGTATCCGGGGTGCAACTGACGCTGAGGCTCGAAAGTGCGGGTATCAAAC"
##  [43] "CCAGGCGTTATCCGGATTCATTGGGTTTAAAGGGAGCGTAGGCCGCCTTTTAAGCGTGTTGTGAAATGTAGGGGCTCAACCCCTGCACTGCAGCGCGAACTGGAGGGCTTGAGTACACACAAAGTAGGCGGAATTCATGGTGTAGCGGTGAAATGCTTAGATATCATGAAGAACCCCGATTGCGAAGGCAGCTTACTGGAGTGTAACTGACGCTGAAGCTCGAAAGCGCGGGTATCGAAC"
##  [44] "CCGGGCGTTATCCGGATTTATTGGGTTTAAAGGGAGCGTAGGCTGTCCTTTAAGCGTGTTGTGAAATGTAGGCGCTCAACGCCTGCACTGCAGCGCGAACTGAAGGACTTGAGTGTGCACGACGCTGGCGGAATTCGTGGTGTAGCGGTGAAATGCTTAGATATCACGAAGAACTCCGATTGCGAAGGCAGCTCACTGGAGCGCAACTGACGCTGAAGCTCGAAAGTGCGGGTATCGAAC"
##  [45] "CCGGGCGTTATCCGGATTTATTGGGTTTAAAGGGAGCGTAGGCTGTCCTTTAAGCGTGTTGTGAAATGTAGGCGCTCAACGCCTGCACTGCAGCGCGAACTGAAGGACTTGAGTGTGCACGACGCTGGCGGAATTCGTGGTGTAGCGGTGAAATGCTTAGATATCACGAAGAACTCCGATTGCGAAGGCAGCTGGCGAGAGCACAACTGACGCTGAAGCTCGAAAGTGCGGGTATCGAAC"
##  [46] "CCGGGCGTTATCCGGATTTATTGGGTTTAAAGGGAGCGTAGGCCGGAGATTAAGCGTGTTGTGAAATGTAGATGCTCAACATCTGAACTGCAGCGCGAACTGGTTTCCTTGAGTACGCACAAAGTGGGCGGAATTCGTGGTGTAGCGGTGAAATGCTTAGATATCACGAAGAACTCCGATTGCGAAGGCAGCTCACTGGAGCGCAACTGACGCTGAAGCTCGAAAGTGCGGGTATCGAAC"
##  [47] "CCGGGCGTTATCCGGATTTATTGGGTTTAAAGGGAGCGTAGGCCGGAGATTAAGCGTGTTGTGAAATGTAGATGCTCAACATCTGCACTGCAGCGCGAACTGGTTTCCTTGAGTACGCACAAAGTGGGCGGAATTCGTGGTGTAGCGGTGAAATGCTTAGATATCACGAAGAACTCCGATTGCGAAGGCAGCTCACTGGAGCGCAACTGACGCTGAAGCTCGAAAGTGCGGGTATCGAAC"
##  [48] "CCGGGCGTTATCCGGATTTATTGGGTTTAAAGGGAGCGTAGGCCGGAGATTAAGCGTGTTGTGAAATGTAGAGGCTCAACCTCTGCACTGCAGCGCGAACTGGTCTTCTTGAGTACGCACAACGTGGGCGGAATTCGTGGTGTAGCGGTGAAATGCTTAGATATCACGAAGAACTCCGATTGCGAAGGCAGCTCACTGGAGCGCAACTGACGCTGAAGCTCGAAAGTGCGGGTATCGAAC"
##  [49] "CCGGGCGTTATCCGGATTTATTGGGTTTAAAGGGAGCGTAGGCCGGAGATTAAGCGTGTTGTGAAATGTAGTGGCTCAACCTCTGCACTGCAGCGCGAACTGGTCTTCTTGAGTACGCACAACGTGGGCGGAATTCGTGGTGTAGCGGTGAAATGCTTAGATATCACGAAGAACTCCGATTGCGAAGGCAGCTCACTGGAGCGCAACTGACGCTGAAGCTCGAAAGTGCGGGTATCGAAC"
##  [50] "CCGGGCGTTATCCGGATTTATTGGGTTTAAAGGGAGCGTAGGCCGGAGATTAAGCGTGTTGTGAAATGTAGTGGCTCAACCTCTGCACTGCAGCGCGAACTGGTCTTCTTGAGTACGCACAACGTGGGCGGAATTCGTGGTGTAGCGGTGAAATGCTTAGATATCACGAAGAACTCCGATTGCGAAGGCAGCTCACGGGAGCGCAACTGACGCTGAAGCTCGAAAGTGCGGGTATCGAAC"
##  [51] "CCGGGCGTTATCCGGATTTATTGGGTTTAAAGGGAGCGTAGGCCGGAGATTAAGCGTGTTGTGAAATGTAGAGGCTCAACCTCTGCACTGCAGCGCGAACTGGTCTTCTTGAGTACGCACAACGTGGGCGGAATTCGTGGTGTAGCGGTGAAATGCTTAGATATCACGAAGAACTCCGATTGCGAAGGCAGCTCACGGGAGCGCAACTGACGCTGAAGCTCGAAAGTGCGGGTATCGAAC"
##  [52] "CCGGGCGTTATCCGGATTTATTGGGTTTAAAGGGAGCGTAGGCCGGAGATTAAGCGTGTTGTGAAATGTAGATGCTCAACATCTGCACTGCAGCGCGAACTGGTCTTCTTGAGTACGCACAACGTGGGCGGAATTCGTGGTGTAGCGGTGAAATGCTTAGATATCACGAAGAACTCCGATTGCGAAGGCAGCTCACGGGAGCGCAACTGACGCTGAAGCTCGAAAGTGCGGGTATCGAAC"
##  [53] "CCGGGCGTTATCCGGATTTATTGGGTTTAAAGGGAGCGTAGGCCGGAGATTAAGCGTGTTGTGAAATGTAGACGCTCAACGTCTGCACTGCAGCGCGAACTGGTTTCCTTGAGTACGCACAAAGTGGGCGGAATTCGTGGTGTAGCGGTGAAATGCTTAGATATCACGAAGAACTCCGATTGCGAAGGCAGCTCACTGGAGCGCAACTGACGCTGAAGCTCGAAAGTGCGGGTATCGAAC"
##  [54] "CCGGGCGTTATCCGGATTTATTGGGTTTAAAGGGAGCGTAGGCCGGAGATTAAGCGTGTTGTGAAATGTAGACGCTCAACGTCTGCACTGCAGCGCGAACTGGTTTCCTTGAGTACGCACAAAGTGGGCGGAATTCGTGGTGTAGCGGTGAAATGCTTAGATATCACGAAGAACTCCGATTGCGAAGGCAGCTCACTGGGGCGCAACTGACGCTGAAGCTCGAAAGCGCGGGTATCGAAC"
##  [55] "CCGGGCGTTATCCGGATTTATTGGGTTTAAAGGGAGCGTAGGCCGTGAGGTAAGCGTGTTGTGAAATGTAGGCGCCCAACGTCTGCACTGCAGCGCGAACTGCCCCACTTGAGTGCGCGCAACGCCGGCGGAACTCGTCGTGTAGCGGTGAAATGCTTAGATATGACGAAGAACCCCGATTGCGAAGGCAGCTGGCGGGAGCGTAACTGACGCTGAAGCTCGAAAGCGCGGGTATCGAAC"
##  [56] "CCGGGCGTTATCCGGATTTATTGGGTTTAAAGGGAGCGTAGGCCGGAGATTAAGCGTGTTGTGAAATGTAGACGCTCAACGTCTGCACTGCAGCGCGAACTGGTTTCCTTGAGTACGCACAAAGTGGGCGGAATTCGTGGTGTAGCGGTGAAATGCTTAGATATCACGAAGAACTCCGATTGCGAAGGCAGCTTGCGGGAGTGCGACTGACGCTGAAGCTCGAAGGTGCGGGTATCGAAC"
##  [57] "CCGGGCGTTATCCGGATTTATTGGGTTTAAAGGGAGCGTAGGCCGGAGATTAAGCGTGTTGTGAAATGTAGACGCTCAACGTCTGCACTGCAGCGCGAACTGGTTTCCTTGAGTACGCACAAAGTGGGCGGAATTCGTGGTGTAGCGGTGAAATGCTTAGATATCACGAAGAACTCCGATTGCGAAGGCAGCTCACGGGAGCGCAACTGACGCTGAAGCTCGAAAGTGCGGGTATCGAAC"
##  [58] "CCGGGCGTTATCCGGATTTATTGGGTTTAAAGGGAGCGTAGGCCGGAGATTAAGCGTGTTGTGAAATGTAGACGCTCAACGTCTGCACTGCAGCGCGAACTGGTTTCCTTGAGTACGCACAAAGTGGGCGGAATTCGTGGTGTAGCGGTGAAATGCTTAGATATCACGAAGAACTCCGATTGCGAAGGCAGCTGACGGTAGCGCAACTGACGCTGAGGCTCGAAAGCGCGGGTATCGAAC"
##  [59] "CCGGGCGTTATCCGGATTTATTGGGTTTAAAGGGAGCGTAGGCCGGAGATTAAGCGTGTTGTGAAATGTAGACGCTCAACGTCTGCACTGCAGCGCGAACTGGTTTCCTTGAGTACGCATAAAGTGGGCGGAATTCGTGGTGTAGCGGTGAAATGCTTAGATATCACGAAGAACTCCGATTGCGAAGGCAGCTCACTGGAGCGCAACTGACGCTGAAGCTCGAAAGTGCGGGTATCGAAC"
##  [60] "CCGGGCGTTATCCGGATTTATTGGGTTTAAAGGGAGCGTAGGCCGGAGATTAAGCGTGTTGTGAAATGTAGATGCTCAACATCTGCACTGCAGCGCGAACTGGTTTCCTTGAGTACGCATAAAGTGGGCGGAATTCGTGGTGTAGCGGTGAAATGCTTAGATATCACGAAGAACTCCGATTGCGAAGGCAGCTCACTGGGGCGCAACTGACGCTGAAGCTCGAAAGCGCGGGTATCGAAC"
##  [61] "CCGGGCGTTATCCGGATTTATTGGGTTTAAAGGGAGCGTAGGCCGGAGATTAAGCGTGTTGTGAAATGTAGATGCTCAACATCTGAACTGCAGCGCGAACTGGTTTCCTTGAGTACGCATAAAGTGGGCGGAATTCGTGGTGTAGCGGTGAAATGCTTAGATATCACGAAGAACTCCGATTGCGAAGGCAGCTCACTGGAGCGCAACTGACGCTGAAGCTCGAAAGTGCGGGTATCGAAC"
##  [62] "CCGGGCGTTATCCGGATTTATTGGGTTTAAAGGGAGCGTAGGCCGGAGATTAAGCGTGTTGTGAAATGTAGATGCTCAACATCTGCACTGCAGCGCGAACTGGTTTCCTTGAGTACGCATAAAGTGGGCGGAATTCGTGGTGTAGCGGTGAAATGCTTAGATATCACGAAGAACTCCGATTGCGAAGGCAGCTCACTGGAGCGCAACTGACGCTGAAGCTCGAAAGTGCGGGTATCGAAC"
##  [63] "CCGGGCGTTATCCGGATTTATTGGGTTTAAAGGGAGCGTAGGCCGGAGATTAAGCGTGTTGTGAAATGTAGTTGCTCAACATCTGCACTGCAGCGCGAACTGGTTTCCTTGAGTACGCACAAAGTGGGCGGAATTCGTGGTGTAGCGGTGAAATGCTTAGATATCACGAAGAACTCCGATTGCGAAGGCAGCTCACTGGAGCGCAACTGACGCTGAAGCTCGAAAGTGCGGGTATCGAAC"
##  [64] "CCGGGCGTTATCCGGATTTATTGGGTTTAAAGGGAGCGTAGGCCGCAGGTTAAGCGTGTTGTGAAATGTAGGGGCTCAACCTCTGCACTGCAGCGCGAACTGGCTTGCTTGAGTACGCACAACGTGGGCGGAATTCGTGGTGTAGCGGTGAAATGCTTAGATATCACGAAGAACTCCGATTGCGAAGGCAGCTCACGGGAGCGCAACTGACGCTGAAGCTCGAAAGTGCGGGTATCGAAC"
##  [65] "CCGGGCGTTATCCGGATTTATTGGGTTTAAAGGGAGCGTAGGCCGCAGGTTAAGCGTGTTGTGAAATGTAGGGGCTCAACCTCTGCACTGCAGCGCGAACTGGCTTGCTTGAGTACGCACAACGTGGGCGGAATTCGTGGTGTAGCGGTGAAATGCTTAGATATCACGAAGAACTCCGATTGCGAAGGCAGCTCACTGGAGCGCAACTGACGCTGAAGCTCGAAAGTGCGGGTATCGAAC"
##  [66] "CCAGGCGTTATCCGGATTTATTGGGTTTAAAGGGAGCGTAGGCCGCCCCTTAAGCGTGTTGTGAAATGCCGCGGCTCAACCGTGGCACTGCAGCGCGAACTGGGGGGCTTGAGTGCACGCAACGCAGGCGGAATTCGTGGTGTAGCGGTGAAATGCTTAGATATCACGAAGAACTCCGATTGCGAAGGCAGCTCACTGGAGCGCAACTGACGCTGAAGCTCGAAAGTGCGGGTATCGAAC"
##  [67] "CCGGGCGTTATCCGGATTTATTGGGTTTAAAGGGAGCGTAGGCCGCCCCTTAAGCGTGTTGTGAAATGCCGCGGCTCAACCGTGGCACTGCAGCGCGAACTGGGGGGCTTGAGTGCACGCAACGCAGGCGGAATTCGTGGTGTAGCGGTGAAATGCTTAGATATCACGAAGAACTCCGATTGCGAAGGCAGCTTGCGGGAGTGCGACTGACGCTGAAGCTCGAAGGTGCGGGTATCGAAC"
##  [68] "CCAGGCGTTATCCGGATTTATTGGGTTTAAAGGGAGCGTAGGCCGCCCCTTAAGCGTGTTGTGAAATGCCGCGGCTCAACCGTGGCACTGCAGCGCGAACTGGGGGGCTTGAGTGCACGCAACGCAGGCGGAATTCGTGGTGTAGCGGTGAAATGCTTAGATATCACGAAGAACTCCGATTGCGAAGGCAGCTTGCGGGAGTGCGACTGACGCTGAAGCTCGAAGGTGCGGGTATCGAAC"
##  [69] "CCGGGCGTTATCCGGATTTATTGGGTTTAAAGGGAGCGTAGGCCGCCCCTTAAGCGTGTTGTGAAATGCCGCGGCTCAACCGTGGCACTGCAGCGCGAACTGGGGGGCTTGAGTGCACGCAACGCAGGCGGAATTCGTGGTGTAGCGGTGAAATGCTTAGATATCACGAAGAACTCCGATTGCGAAGGCAGCTCACTGGAGCGCAACTGACGCTGAAGCTCGAAAGTGCGGGTATCGAAC"
##  [70] "CCGGGCGTTATCCGGATTTATTGGGTTTAAAGGGAGCGTAGGCCGTTTGGTAAGCGTGTTGTGAAATGTCGGGGCTCAACCTGGGCATTGCAGCGCGAACTGCCAGACTTGAGTGCGCAGGAAGTAGGCGGAATTCGTCGTGTAGCGGTGAAATGCTTAGATATGACGAAGAACTCCGATTGCGAAGGCAGCCTGCTGTAGCGCAACTGACGCTGAAGCTCGAAAGCGTGGGTATCGAAC"
##  [71] "CCAGGCGTTATCCGGATTTATTGGGTTTAAAGGGAGCGTAGGCCGTTTGGTAAGCGTGTTGTGAAATGTCGGGGCTCAACCTGGGCATTGCAGCGCGAACTGCCAGACTTGAGTGCGCAGGAAGTAGGCGGAATTCGTCGTGTAGCGGTGAAATGCTTAGATATGACGAAGAACTCCGATTGCGAAGGCAGCCTGCTGTAGCGCAACTGACGCTGAAGCTCGAAAGCGTGGGTATCGAAC"
##  [72] "CCGGGCGTTATCCGGATTTATTGGGTTTAAAGGGAGCGTAGGCCGTCTGTTAAGCGTGTTGTGAAATGTCGTGGCTCAACCGGGGCACTGCAGCGCGAACTGGCAGACTTGAGTGCACGGTAGGAAGGCGGAATTCGTCGTGTAGCGGTGAAATGCTTAGATATGACGAAGAACTCCGATTGCGAAGGCAGCTTTCCGTAGTGTAACTGACGCTGAAGCTCGAAAGCGTGGGTATCGAAC"
##  [73] "CCGGGCGTTATCCGGATTTATTGGGTTTAAAGGGAGCGTAGGCCGTCTGTTAAGCGTGTTGTGAAATGTCGGGGCTCAACCTGGGCATTGCAGCGCGAACTGGCAGACTTGAGTGCGCAGGAAGTAGGCGGAATTCGTCGTGTAGCGGTGAAATGCTTAGATATGACGAAGAACTCCGATTGCGAAGGCAGCCTGCTGTAGCGTAACTGACGCTGAAGCTCGAAAGCGTGGGTATCGAAC"
##  [74] "CCGGGCGTTATCCGGATTTATTGGGTTTAAAGGGAGCGTAGGCCGTCTGGTAAGCGTGTTGTGAAATGTCGGGGCTCAACCTGGGCATTGCAGCGCGAACTGTCAGACTTGAGTGCGCGGGAAGTAGGCGGAATTCGTCGTGTAGCGGTGAAATGCTTAGATATGACGAAGAACTCCGATTGCGAAGGCAGCCTGCTGTAGCGCAACTGACGCTGAAGCTCGAAAGCGTGGGTATCGAAC"
##  [75] "CCGGGCGTTATCCGGATTTATTGGGTTTAAAGGGAGCGTAGGCCGTCTTATAAGCGTGTTGTGAAATGTCGGGGCTCAACCTGGGCATTGCAGCGCGAACTGTGAGACTTGAGTGCGCAGGAAGTAGGCGGAATTCGTCGTGTAGCGGTGAAATGCTTAGATATGACGAAGAACTCCGATTGCGAAGGCAGCCTGCTGTAGCGCAACTGACGCTGAAGCTCGAAAGCGTGGGTATCGAAC"
##  [76] "CCGGGCGTTATCCGGATTTATTGGGTTTAAAGGGAGCGTAGGCCGTCTTATAAGCGTGTTGTGAAATGTCGGGGCTCAACCTGGGCATTGCAGCGCGAACTGTGAGACTTGAGTGCGCAGGAAGTAGGCGGAATTCGTCGTGTAGCGGTGAAATGCTTAGATATCACGAAGAACTCCGATTGCGAAGGCAGCTCACTGGAGCGCAACTGACGCTGAAGCTCGAAAGTGCGGGTATCGAAC"
##  [77] "CCAGGCGTTATCCGGATTTATTGGGTTTAAAGGGAGCGTAGGCCGTTTTTTAAGCGTGTTGTGAAATACTGTCGCTCAACGACAGAGGTGCAGCGCGAACTGGAGGACTTGAGTGCGCGGAATGTAGGCGGAATTCGTCGTGTAGCGGTGAAATGCTTAGATATGACGAAGAACTCCGATTGCGAAGGCAGCTTACAGTAGCGTAACTGACGCTGAAGCTCGAAAGTGCGGGTATCGAAC"
##  [78] "CCGGGCGTTATCCGGATTTATTGGGTTTAAAGGGAGCGTAGGCCGTCTTTTAAGCGTGTTGTGAAATACTGTCGCTCAACGACAGAGGTGCAGCGCGAACTGGGAGACTTGAGTGCGCGGAATGCAGGCGGAATTCGTCGTGTAGCGGTGAAATGCTTAGATATGACGAAGAACTCCGATTGCGAAGGCAGCTTGCAGTAGCGTAACTGACGCTGAAGCTCGAAAGTGCGGGTATCGAAC"
##  [79] "CCGGGCGTTATCCGGATTTATTGGGTTTAAAGGGAGCGCAGGCCGTGGGCTAAGCGTGCCGTGAAATTCTGTCGCTCAACGGCAGACGTGCGGCGCGAACTGGTCCACTTGAGTACGCGGGACGTTGGCGGAATTCGTGGTGTAGCGGTGAAATGCTTAGATATCACGAAGAACTCCGATTGCGAAGGCAGCTCACTGGAGCGCAACTGACGCTGAAGCTCGAAAGTGCGGGTATCGAAC"
##  [80] "CCGGGCGTTATCCGGATTTATTGGGTTTAAAGGGAGCGCAGGCCGTGGGTTAAGCGTGTCGTGAAATTCCGTCGCTCAACGGCGGACGTGCGGCGCGAACTGGTCCACTTGAGTACGCGGGACGTTGGCGGAATTCGTGGTGTAGCGGTGAAATGCTTAGATATCACGAAGAACTCCGATTGCGAAGGCAGCTGACGGTAGCGCAACTGACGCTGAGGCTCGAAAGTGCGGGTATCGAAC"
##  [81] "CCGGGCGTTATCCGGATTTATTGGGTTTAAAGGGAGCGCAGGCCGTGGGCTAAGCGTGCCGTGAAATTCTGTCGCTCAACGGCAGACGTGCGGCGCGAACTGGTCCACTTGAGTACGCGGGACGTTGGCGGAATTCGTGGTGTAGCGGTGAAATGCTTAGATATCACGAAGAACTCCGATTGCGAAGGCAGCTGACGGTAGCGCAACTGACGCTGAGGCTCGAAAGCGCGGGTATCGAAC"
##  [82] "CCGGGCGTTATCCGGATTTATTGGGTTTAAAGGGAGCGCAGGCCGTGGGTTAAGCGTGTCGTGAAATTCCGTCGCTCAACGGCGGACGTGCGGCGCGAACTGGTCCACTTGAGTACGCGGGACGTTGGCGGAATTCGTGGTGTAGCGGTGAAATGCTTAGATATCACGAAGAACTCCGATTGCGAAGGCAGCTCACTGGAGCGCAACTGACGCTGAAGCTCGAAAGTGCGGGTATCGAAC"
##  [83] "GCGAGCGTTATCCGGATTTATTGGGTTTAAAGGGTGCGTAGGTCGCCGATTAAGTCAGCGGTGAAATCCAGTGGCTCAACCATCGGACTGCCGTTGAAACTGGCCGGCTTGAGTATGATTGAGGCAGGCGGAATGCGTGGTGTAGCGGTGAAATGCATAGATATCACGCAGAACCCCGATTGCGAAGGCAGCTTGCCAAGCCATGACTGACACTGAAGCACGAAAGCGTGGGTATCAAAC"
##  [84] "GCGAGCGTTATCCGGATTTATTGGGTTTAAAGGGTGCGCAGGCGGCGCGCCAAGTCAGCGGTCAAAGTTCCGGGCTCAACCCGGTGTCGCCGTTGAAACTGGCGTGCTCGAGTGCGTGCGAGGAAGGCGGAATGCGTTGTGTAGCGGTGAAATGCATAGATATGACGCAGAACTCCGATTGCGAAGGCAGCTTTCCAGCGCGCTACTGACGCTGAGGCACGAAAGCGTGGGGATCGAACA"
##  [85] "CCAAGCGTTATCCGGATTTATTGGGCGTAAAGCGAGCGCAGACGGTTATTTAAGTCTGAAGTGAAAGCCCTCAGCTCAACTGAGGAATTGCTTTGGAAACTGGATGACTTGAGTGCAGTAGAGGAAAGTGGAACTCCATGTGTAGCGGTGAAATGCGTAGATATATGGAAGAACACCAGTGGCGAAGGCGGCTTTCTGGACTGTAACTGACGTTGAGGCTCGAAAGTGTGGGTAGCAAAC"
##  [86] "GCGAGCGTTGTCCGGAATTATTGGGCGTAAAGAGCATGTAGGCGGTTTTTTAAGTCTGGAGTGAAAATGCGGGGCTCAACCCCGTATGGCTCTGGATACTGGAAGACTTGAGTGCAGGAGAGGAAAGGGGAATTCCCAGTGTAGCGGTGAAATGCGTAGATATTGGGAGGAACACCAGTGGCGAAGGCGCCTTTCTGGACTGTGTCTGACGCTGAGATGCGAAAGCCAGGGTAGCGAACG"
##  [87] "GCAAGCGTTGTCCGGAATTATTGGGCGTAAAGCGCGCGCAGGCGGCTTCTTAAGTCCATCTTAAAAGTGCGGGGCTTAACCCCGTGATGGGATGGAAACTGAGAGGCTGGAGTATCGGAGAGGAAAGTGGAATTCCTAGTGTAGCGGTGAAATGCGTAGAGATTAGGAAGAACACCGGTGGCGAAGGCGACTTTCTGGACGACAACTGACGCTGAGGCGCGAAAGCGTGGGGAGCAAACA"
##  [88] "GCGAGCGTTATCCGGATTCATTGGGCGTAAAGCGCGCGTAGGCGGCCCGTCAAGCGGGGTTTCAAATCCAGGGGCTCAACCTCTGGCCGGACCCCGAACTGGCGGGCTCGAGTGCGGTAGAGGAAGGTGGAATTCCCAGTGTAGCGGTGAAATGCGCAGATATTGGGAAGAACACCGATGGCGAAGGCAGCCTTCTGGGCCGCCACTGACGCTGAGGCGCGAAAGCTAGGGGAGCGAACA"
##  [89] "GCGAGCGTTATCCGGATTCATTGGGCGTAAAGCGCGCGTAGGCGGCCCGGCAGGCCGGGGGTCGAAGCGGGGGGCTCAACCCCCCGAAGCCCCCGGAACCTCCGCGGCTTGGGTCCGGTAGGGGAGGGTGGAACACCCGGTGTAGCGGTGGAATGCGCAGATATCGGGTGGAACACCGGTGGCGAAGGCGGCCCTCTGGGCCGAGACCGACGCTGAGGCGCGAAAGCTGGGGGAGCGAAC"
##  [90] "GCGAGCGTTATCCGGAATCATTGGGCGTAAAGCGCGCGCAGGCGGGCTTTCAAGCGGCGGCGTCGAAGCCGGGGGCTCAACCCCCGGAAGCGCCCCGAACTGGAAGCCTCGGATGCGGCAGGGGGAGGCGGAATTCCCGGTGTAGCGGTGAAATGCGCAGATATCGGGAAGAACACCGACGGCGAAGGCAGCCTCCTGGGCCGGCATCGACGCTGAGGCGCGAAAGCTGGGGGAGCGAAC"
##  [91] "GCGAGCGTTATCCGGAATGATTGGGCGTAAAGCGCGCGCAGGCGGCCGCTCAAGCGGGACCTCTAACCCCGGGGCTCAACCCCGGGCCGGGTCCCGAACTGGGCGGCTCGAGTGCGGTAGGGGAGAGCGGAATTCCAAGTGTAGCGGTGAAATGCGCAGATATTTGGAAGAACACCGATGGCGAAGGCAGCTCTCTGGGCCGTCACTGACGCTGAGGCGCGAAAGCCGGGGGAGCGAACA"
##  [92] "GCGAGCGTTATCCGGATTCATTGGGCGTAAAGCGCGCGTAGGCGGAGCGCTAAGCGGGACCTCTAACCCGAGGGCTCAACCCCCGGCCGGGTCCCGAACTGGCGCTCTCGAGTGCGGTAGGGGAGAGCGGAATTCCCGGTGTAGCGGTGGAATGCGCAGATATCGGGAAGAACACCGACGGCGAAGGCAGCTCTCTGGGCCGAAACTGACGCTGAGGCGCGAAAGCTGGGGGAGCGAACA"
##  [93] "GCGAGCGTTATCCGGATTCATTGGGCGTAAAGCGCGCGTAGGCGGAGCGCTAAGCGGGACCTCTAACCCGAGGGCTCAACCCCCGGCCGGGTCCCGAACTGGCGCTCTCGAGTGCGGTAGGGGAGAGCGGAATTCCCGGTGTAGCGGTGGAATGCGCAGATATCGGGAGGAACACCGACGGCGAAGGCAGCTCTCTGGGCCGAAACTGACGCTGAGGCGCGAAAGCTGGGGGAGCGAACA"
##  [94] "GCGAGCGTTATCCGGATTCATTGGGCGTAAAGCGCGCGTAGGCGGATGCCTAAGCGGGACCTCTAACCCGGGGGCTCAACCCCCGGCCGGGTCCCGAACTGGGCGTCTCGAGTGCGGTAGGGGCAGGTGGAATTCCATGTGTAGCGGTGGAATGCGCAGATATATGGAAGAACACCGACGGCGAAGGCAGCCTGCTGGGCCGACACTGACGCTGAGGTGCGAAAGCGCGGGGAGCGAACA"
##  [95] "GCGAGCGTTATCCGGATTCATTGGGCGTAAAGCGCGCGTAGGCGGCCGCTCGAGCGGGACCTCTAACCCGGGGGCTCAACCTCCGGCCGGGTCCCGGACCGTGCGGCTCGGGTGCGGTAGGGGCAGGCGGAACTCCAAGTGTAGCGGTGAAATGCGCAGATATTTGGAGGAACACCGATGGCGAAGGCAGCCTGCTGGGCCGCCACCGACGCTGAGGCGCGAAAGCCGGGGGAGCGAACA"
##  [96] "GCTAGCGTTATCCGGAATTACTGGGCGTAAAGGGTGCGTAGGTGGTTTCTTAAGTCAGAGGTGAAAGGCTACGGCTCAACCGTAGTAAGCCTTTGAAACTGGGAAACTTGAGTGCAGGAGAGGAGAGTGGAATTCCTAGTGTAGCGGTGAAATGCGTAGATATTAGGAGGAACACCAGTTGCGAAGGCGGCTCTCTGGACTGTAACTGACACTGAGGCACGAAAGCGTGGGGAGCAAACA"
##  [97] "GCTAGCGTTATCCGGATTTACTGGGCGTAAAGGGTGCGTAGGCGGTCTTTTAAGTCAGGAGTGAAAGGCTACGGCTCAACCGTAGTAAGCTCTTGAAACTGGAGGACTTGAGTGCAGGAGAGGAGAGTGGAATTCCTAGTGTAGCGGTGAAATGCGTAGATATTAGGAGGAACACCAGTAGCGAAGGCGGCTCTCTGGACTGTAACTGACGCTGAGGCACGAAAGCGTGGGGAGCAAACA"
##  [98] "GCAAGCGTTATCCGGAATTATTGGGCGTAAAGAGTACGTAGGTGGTTTTCTAAGCACGGGGTTTAAGGCAATGGCTTAACCATTGTTCGCCTTGTGAACTGGAAGACTTGAGTGCAGGAGAGGAAAGCGGAATTCCTAGTGTAGCGGTGAAATGCGTAGATATTAGGAGGAACACCAGTGGCGAAGGCGGCTTTCTGGACTGTAACTGACACTGAGGTACGAAAGCGTGGGGAGCAAACA"
##  [99] "GCAAGCGTTATCCGGAATTATTGGGCGTAAAGAGTACGTAGGTGGTTACCTAAGCACGAGGTATAAGGCAATGGCTTAACCATTGTTCGCCTTGTGAACTGGGCTACTTGAGTGCAGGAGAGGAAAGCGGAATTCCTAGTGTAGCGGTGAAATGCGTAGATATTAGGAGGAACACCAGTGGCGAAGGCGGCTTTCTGGACTGTAACTGACACTGAGGTACGAAAGCGTGGGGAGCAAACA"
## [100] "GCAAGCGTTACTCGGAATTACTAGGCGTAAAGCGCGCGTAGGCGGAATGTTAAGTCTGTTGTGTAATCTCTGGGCTCAACCCAGAAACTGCAACAGAAACTGGCGTTCTTGAGTGAGGCAGAGGAAATCGGAATTCCTAGTGTAGCAGTGAAATGCGTAGATATTAGGAGGAACACCGGTGGCGAAGGCGGATTTCTGGGCCTTTACTGACGCTAAAGTGCGAAAGCTAGGGGAGCAAAC"
## [101] "GCGAGCGTTGTCCGGAATGACTGGGCGTAAAGGGCGTGTAGGCGGCAGTATAAGTCCGGAGTGAAAGTCCTGCTTTCAAGGTGGGAATTGCTTTGGAGACTGTACAGCTTGAGTGCGGAAGAGGTAAGTGGAATTCCCAGTGTAGCGGTGAAATGCGTAGAGATTGGGAGGAACACCAGTGGCGAAGGCGACTTACTGGGCCGTAACTGACGCTGAGGCGCGAAAGCGTGGGGAGCGAAC"
## [102] "GCAAGCGTTATCCGGATTTACTGGGCGTAAAGGGAGCGTAGGCGGATATTTAAGTGGGATGTGAAATACCCGAGCTTAACTTGGGAGCTGCATTCCAAACTGGATATCTAGAGTGCAGGAGAGGAGAATGGAATTCCTAGTGTAGCGGTGAAATGCGTAGAGATTAGGAAGAACACCAGTGGCGAAGGCGATTCTCTGGACTGTAACTGACGCTGAGGCTCGAAAGCGTGGGGAGCAAAC"
## [103] "GCAAGCGTTATCCGGATTTACTGGGTGTAAAGGGAGCGTAGACGGTGTGGCAAGTCTGATGTGAAAGGCATGGGCTCAACCTGTGGACTGCATTGGAAACTGTCATACTTGAGTGCCGGAGGGGTAAGCGGAATTCCTAGTGTAGCGGTGAAATGCGTAGATATTAGGAGGAACACCAGTGGCGAAGGCGGCTTACTGGACGGTAACTGACGTTGAGGCTCGAAAGCGTGGGGAGCAAAC"
## [104] "GCAAGCGTTATCCGGATTTACTGGGTGTAAAGGGAGCGTAGACGGCGCAGCAAGTCTGATGTGAAAGGCAGGGGCTTAACCCCTGGACTGCATTGGAAACTGCTGTGCTTGAGTGCCGGAGGGGTAAGCGGAATTCCTAGTGTAGCGGTGAAATGCGTAGATATTAGGAGGAACACCAGTGGCGAAGGCGGCTTACTGGACGGTAACTGACGTTGAGGCTCGAAAGCGTGGGGAGCAAAC"
## [105] "GCAAGCGTTATCCGGATTTACTGGGTGTAAAGGGAGCGTAGACGGATTAGCAAGTCTGATGTGAAAGGCAGGGGCTCAACCCCTGGACTGCATTGGAAACTGCCAGTCTTGAGTGCCGGAGAGGTAAGCGGAATTCCTAGTGTAGCGGTGAAATGCGTAGATATTAGGAGGAACACCAGTGGCGAAGGCGGCTTACTGGACGGCAACTGACGTTGAGGCTCGAAAGCGTGGGGAGCAAAC"
## [106] "GCAAGCGTTATCCGGATTTACTGGGTGTAAAGGGAGCGTAGACGGCTTTGCAAGTCTGATGTGAAAGGCGGGGGCTCAACCCCTGGACTGCATTGGAAACTGTGAGGCTTGAGTGCCGGAGAGGTAAGCGGAATTCCTAGTGTAGCGGTGAAATGCGTAGATATTAGGAGGAACACCAGTGGCGAAGGCGGCTTACTGGACGGTAACTGACGTTGAGGCTCGAAAGCGTGGGGAGCAAAC"
## [107] "GCAAGCGTTATCCGGATTTACTGGGTGTAAAGGGAGCGTAGGCGGTATGGCAAGTCTGATGTGAAAGGCCGGGGCTCAACCCCGGGACTGCATTGGAAACTGTCACACTTGAGTGTCGGAGAGGTAAGTGGAATTCCTAGTGTAGCGGTGAAATGCGTAGATATTAGGAGGAACACCAGTGGCGAAGGCGGCTTACTGGACGACAACTGACGCTGAGGCTCGAAAGCGTGGGGAGCAAAC"
## [108] "GCAAGCGTTATCCGGATTTACTGGGTGTAAAGGGAGCGCAGGCGGTCTGGCAAGTCTGATGTGAAATCCCGGGGCTCAACCCTGGAACTGCATTGGAAACTGTCAGACTAGAGTGCCGGAGAGGTAAGTGGAATTCCTAGTGTAGCGGTGAAATGCGTAGATATTAGGAGGAACACCAGTGGCGAAGGCGGCTTACTGGACGGTAACTGACGCTGAGGCTCGAAAGCGTGGGGAGCAAAC"
## [109] "GCAAGCGTTATCCGGATTTACTGGGTGTAAAGGGAGCGTAGACGGTATGGCAAGTCTGATGTGAAAGGCCAGGGCTCAACCCTGGGACTGCATTGGAAACTGTCGAACTAGAGTGTCGGAGAGGCAAGTGGAATTCCTAGTGTAGCGGTGAAATGCGTAGATATTAGGAGGAACACCAGTGGCGAAGGCGGCTTGCTGGACGATGACTGACGTTGAGGCTCGAAAGCGTGGGGAGCAAAC"
## [110] "GCAAGCGTTATCCGGATTTACTGGGTGTAAAGGGAGCGTAGACGGAATGGCAAGTCTGATGTGAAAGGCCGGGGCTCAACCCCGGGACTGCATTGGAAACTGTCAATCTAGAGTACCGGAGGGGTAAGTGGAATTCCTAGTGTAGCGGTGAAATGCGTAGATATTAGGAGGAACACCAGTGGCGAAGGCGGCTTACTGGACGGTAACTGACGTTGAGGCTCGAAAGCGTGGGGAGCAAAC"
## [111] "GCAAGCGTTATCCGGATTTACTGGGTGTAAAGGGAGCGTAGACGGCACAGCAAGTCTGATGTGAAAGCCCGGGGCCCAACCCCGGAACTGCATTGGAAACTGCTGGGCTTGAGTGCAGGAGAGGTAAGCGGAATTCCTAGTGTAGCGGTGAAATGCGTAGATATTAGGAGGAACACCAGTGGCGAAGGCGGCTTACTGGACTGTAACTGACGTTGAGGCTCGAAAGCGTGGGGAGCAAAC"
## [112] "GCAAGCGTTATCCGGATTTACTGGGTGTAAAGGGAGCGTAGACGGCGAGACAAGTCTGAAGTGAAAGCCCGGGGCTCAACCCCGGGACTGCTTTGGAAACTGCCTTGCTAGAGTGCTGGAGAGGTAAGTGGAATTCCTAGTGTAGCGGTGAAATGCGTAGATATTAGGAGGAACACCAGTGGCGAAGGCGGCTTACTGGACAGTAACTGACGTTGAGGCTCGAAAGCGTGGGGAGCAAAC"
## [113] "GCAAGCGTTATCCGGATTTACTGGGTGTAAAGGGAGCGTAGACGGTCAAGCAAGTCAGAAGTGAAAGGCTGGGGCTCAACCCCGGGACTGCTTTTGAAACTGTTTGACTGGAGTGCTGGAGAGGTAAGCGGAATTCCTAGTGTAGCGGTGAAATGCGTAGATATTAGGAGGAACACCAGTGGCGAAGGCGGCTTACTGGACAGTAACTGACGTTGAGGCTCGAAAGCGTGGGGAGCAAAC"
## [114] "GCAAGCGTTATCCGGATTTACTGGGTGTAAAGGGAGCGTAGACGGCTAAGCAAGTCAGAAGTGAAAGGCTGGGGCTCAACCCCGGGACTGCTTTTGAAACTGTTTGGCTAGAGTGCTGGAGAGGTAAGCGGAATTCCTAGTGTAGCGGTGAAATGCGTAGATATTAGGAGGAACACCAGTGGCGAAGGCGGCTTACTGGACAGTAACTGACGTTGAGGCTCGAAAGCGTGGGGAGCAAAC"
## [115] "GCAAGCGTTATCCGGATTTACTGGGTGTAAAGGGAGCGTAGACGGTTAAGCAAGTCAGAAGTGAAAGGCTGGGGCTCAACCCCGGGACTGCTTTTGAAACTGTTTAACTAGAGTGCTGGAGAGGTAAGCGGAATTCCTAGTGTAGCGGTGAAATGCGTAGATATTAGGAGGAACACCAGTGGCGAAGGCGGCTTACTGGACAGTAACTGACGTTGAGGCTCGAAAGCGTGGGGAGCAAAC"
## [116] "GCAAGCGTTATCCGGATTTACTGGGTGTAAAGGGAGCGTAGACGGTAAAGCAAGTCTGAAGTGAAAGCCCGGGGCTCAACCGCGGGACTGCTTTGGAAACTGTTTAACTAGAGTGCTGGAGAGGTAAGCGGAATTCCTAGTGTAGCGGTGAAATGCGTAGATATTAGGAGGAACACCAGTGGCGAAGGCGGCTTACTGGACAGTAACTGACGTTGAGGCTCGAAAGCGTGGGGAGCAAAC"
## [117] "GCAAGCGTTATCCGGATTTACTGGGTGTAAAGGGAGCGTAGACGGCGATGCAAGTCTGAAGTGAAATACCCGGGCTCAACCTGGGAACTGCTTTGGAAACTGTATTGCTAGAGTGCTGGAGAGGTAAGCGGAATTCCTAGTGTAGCGGTGAAATGCGTAGATATTAGGAAGAACACCAGTGGCGAAGGCGGCTTACTGGACAGTAACTGACGTTGAGGCTCGAAAGCGTGGGGAGCAAAC"
## [118] "GCAAGCGTTATCCGGATTTACTGGGTGTAAAGGGAGCGTAGACGGCGACGCAAGTCTGAAGTGAAATACCCGGGCTCAACCTGGGAACTGCTTTGGAAACTGTGTTGCTAGAGTGCTGGAGAGGTAAGCGGAATTCCTAGTGTAGCGGTGAAATGCGTAGATATTAGGAAGAACACCAGTGGCGAAGGCGGCTTACTGGACAGTAACTGACGTTGAGGCTCGAAAGCGTGGGGAGCAAAC"
## [119] "GCAAGCGTTATCCGGATTTACTGGGTGTAAAGGGAGCGTAGACGGTAGTGCAAGTCTGATGTGAAAGCCCGGGGCTCAACCCCGGGACTGCATTGGAAACTGTATAACTAGAGTGTCGGAGAGGTAAGCGGAATTCCTAGTGTAGCGGTGAAATGCGTAGATATTAGGAGGAACACCAGTGGCGAAGGCGGCTTACTGGACGATGACTGACGTTGAGGCTCGAAAGCGTGGGGAGCAAAC"
## [120] "GCAAGCGTTATCCGGATTTACTGGGTGTAAAGGGAGCGTAGACGGCTGTGTAAGTCTGAAGTGAAAGCCCGGGGCTCAACCGCGGGACTGCTTTGGAAACTATGCAGCTAGAGTGTCGGAGAGGTAAGTGGAATTCCCAGTGTAGCGGTGAAATGCGTAGATATTGGGAGGAACACCAGTGGCGAAGGCGGCTTACTGGACGATGACTGACGTTGAGGCTCGAAAGCGTGGGGAGCAAAC"
## [121] "GCAAGCGTTATCCGGATTTACTGGGTGTAAAGGGAGCGTAGACGGCTGTGCAAGTCTGAAGTGAAAGGCATGGGCTCAACCTGTGGACTGCTTTGGAAACTGTGCAGCTAGAGTGTCGGAGAGGTAAGTGGAATTCCTAGTGTAGCGGTGAAATGCGTAGATATTAGGAGGAACACCAGTGGCGAAGGCGGCTTACTGGACGATGACTGACGTTGAGGCTCGAAAGCGTGGGGAGCAAAC"
## [122] "GCAAGCGTTATCCGGATTTACTGGGTGTAAAGGGAGCGTAGACGGCATGGCAAGTCTGATGTGAAAATCCCGGGCTCAACCCGGGAACTGCATTGGAAACTGTTAAGCTAGAGTGCAGGAGAGGTAAGTGGAATTCCTAGTGTAGCGGTGAAATGCGTAGATATTAGGAGGAACACCAGTGGCGAAGGCGGCTTACTGGACTGTAACTGACGTTGAGGCTCGAAAGCGTGGGGAGCAAAC"
## [123] "GCAAGCGTTATCCGGATTTACTGGGTGTAAAGGGAGCGTAGACGGCACGGCAAGCCAGATGTGAAAGCCCGGGGCTCAACCCCGGGACTGCATTTGGAACTGCTGAGCTAGAGTGTCGGAGAGGCAAGTGGAATTCCTAGTGTAGCGGTGAAATGCGTAGATATTAGGAGGAACACCAGTGGCGAAGGCGGCTTGCTGGACGATGACTGACGTTGAGGCTCGAAAGCGTGGGGAGCAAAC"
## [124] "GCAAGCGTTATCCGGAATTACTGGGTGTAAAGGGAGCGTAGGCGGCATGGTAAGTAAGATGTGAAAGCCCGAGGCTTAACCTCGAGGATTGCATTTTAAACTATCAAGCTAGAGTACAGGAGAGGAAAGCGGAATTCCTAGTGTAGCGGTGAAATGCGTAGATATTAGGAAGAACACCAGTGGCGAAGGCGGCTTTCTGGACTGAAACTGACGCTGAGGCTCGAAAGCGTGGGGAGCGAA"
## [125] "GCAAGCGTTATCCGGAATTACTGGGTGTAAAGGGTGCGTAGGTGGTATGGCAAGTCAGAAGTGAAAACCCAGGGCTTAACTCTGGGACTGCTTTTGAAACTGTCAGACTGGAGTGCAGGAGAGGTAAGCGGAATTCCTAGTGTAGCGGTGAAATGCGTAGATATTAGGAGGAACATCAGTGGCGAAGGCGGCTTACTGGACTGAAACTGACACTGAGGCACGAAAGCGTGGGGAGCAAAC"
## [126] "GCAAGCGTTATCCGGAATTACTGGGTGTAAAGGGTGCGTAGGTGGTATGGCAAGTCAGAAGTGAAAACCCAGGGCTTAACTCTGGGACTGCTTTTGAAACTGTCAGACTAGAGTGCAGGAGAGGTAAGCGGAATTCCTAGTGTAGCGGTGAAATGCGTAGATATTAGGAGGAACATCAGTGGCGAAGGCGGCTTACTGGACTGAAACTGACACTGAGGCACGAAAGCGTGGGGAGCAAAC"
## [127] "GCAAGCGTTATCCGGATTTACTGGGTGTAAAGGGAGTGTAGGTGGCCATGCAAGTCAGAAGTGAAAATCCGGGGCTCAACCCCGGAACTGCTTTTGAAACTGTAAGGCTGGAGTGCAGGAGGGGTGAGTGGAATTCCTAGTGTAGCGGTGAAATGCGTAGATATTAGGAGGAACACCAGTGGCGAAGGCGGCTCACTGGACTGTAACTGACACTGAGGCTCGAAAGCGTGGGGAGCAAAC"
## [128] "GCAAGCGTTATCCGGATTTACTGGGTGTAAAGGGAGTGTAGGTGGCCAGGCAAGTCAGAAGTGAAAGCCCGGGGCTCAACCCCGGGACTGCTTTTGAAACTGCAGGGCTAGAGTGCAGGAGGGGCAAGTGGAATTCCTAGTGTAGCGGTGAAATGCGTAGATATTAGGAGGAACACCAGTGGCGAAGGCGGCTTGCTGGACTGTAACTGACACTGAGGCTCGAAAGCGTGGGGAGCAAAC"
## 
## $x_only
##  [1] "GCAAGCGTTATCCGGATTTACTGGGTGTAAAGGGAGCGTAGGCGGCGATGCAAGTCAGAAGTGAAAGCCCAGGGCTTAACCGTGGGACTGCTTTTGAAACTGTGTTGCTGGATTGCCGGAGAGGTAAGTGGAATTCCTAGTGTAGCGGTGAAATGCGTAGATATTAGGAGGAACACCAGTGGCGAAGGCGGCTTACTGGACGGTGAATGACGCTGAGGCTCGAAAGCGTGGGGAGCAAAC"
##  [2] "GCAAGCGTTATCCGGATTTACTGGGTGTAAAGGGTGCGTAGGTGGCAGTGCAAGTCAGATGTGAAAGGCCGGGGCTCAACCCCGGAGCTGCATTTGAAACTGCGCGGCTAGAGTACAGGAGAGGCAGGCGGAATTCCTAGTGTAGCGGTGAAATGCGTAGATATTAGGAGGAACACCAGTGGCGAAGGCGGCCTGCTGGACTGTTACTGACACTGAGGCACGAAAGCGTGGGGAGCAAAC"
##  [3] "GCAAGCGTTATCCGGATTTATTGGGTGTAAAGGGTGCGTAGACGGGAAGGTAAGTTAGTTGTGAAATCCCTCGGCTCAACTGAGGAACTGCGACTAAAACTGCTTTTCTTGAGTGCTGGAGAGGAAAGTGGAATTCCTAGTGTAGCGGTGAAATGCGTAGATATTAGGAGGAACACCAGTGGCGAAGGCGACTTTCTGGACAGCAACTGACGTTGAGGCACGAAAGTGTGGGGAGCAAAC"
##  [4] "ACAAGCGTTGTCCGGATTTACTGGGTGTAAAGGGCGCGTAGGCGGACTGTCAAGTCAGTCGTGAAATACCGGGGCTTAACCCCGGGGCTGCGATTGAAACTGACAGCCTTGAGTATCGGAGAGGAAAGCGGAATTCCTAGTGTAGCGGTGAAATGCGTAGATATTAGGAGGAACACCAGTGGCGAAGGCGGCTTACTGGACGGTAACTGACGTTGAGGCTCGAAAGCGTGGGGAGCAAAC"
##  [5] "GCGAGCGTTGTCCGGAATTACTGGGTGTAAAGGGAGTGTAGGCGGGAAGGCAAGTCAGAAGTGAAAATTATGGGCTTAACCCATAACCTGCTTTTGAAACTGTTTTTCTTGAGTGAGGCAGAGGCAAGCGGAATTCCTAGTGTAGCGGTGAAATGCGTAGATATTAGGAGGAACACCAGTGGCGAAGGCGGCTTGCTGGGCCTTTACTGACGCTGAGGCTCGAAAGCGTGGGGAGCAAAC"
##  [6] "GCAAGCGTTGTCCGGAATTACTGGGTGTAAAGGGAGCGTAGGCGGGATGCCAAGTCAGCTGTGAAAACTATGGGCTTAACTTGTAGACTGCAGTTGAAACTGGTATTCTTGAGTGAAGTAGAGGTTGGCGGAATTCCGAGTGTAGCGGTGAAATGCGTAGATATTCGGAGGAACACCGGTGGCGAAGGCGGCCAACTGGGCTTTAACTGACGCTGAGGCTCGAAAGTGTGGGGAGCAAAC"
##  [7] "GCAAGCGTTGTCCGGAATTACTGGGTGTAAAGGGAGCGTAGGCGGGATGCCAAGTCAGCTGTGAAAACTATGGGCTTAACCTGTAGACTGCAGTTGAAACTGGTATTCTTGAGTGAAGTAGAGGTTGGCGGAATTCCGAGTGTAGCGGTGAAATGCGTAGATATTCGGAGGAACACCGGTGGCGAAGGCGGCCAACTGGGCTTTAACTGACGCTGAGGCTCGAAAGTGTGGGGAGCAAAC"
##  [8] "GCGAGCGTTGTCCGGAATTACTGGGTGTAAAGGGAGCGTAGGCGGGACAGCAAGTTGAATGTGAAATCTATGGGCTCAACCCATAAACTGCGTTCAAAACTGTTGTTCTTGAGTGAAGTAGAGGTAGGCGGAATTCCTAGTGTAGCGGTGAAATGCGTAGATATTAGGAGGAACACCAGTGGCGAAGGCGGCCTACTGGGCTTTAACTGACGCTGAGGCTCGAAAGCGTGGGTAGCAAAC"
##  [9] "GCAAGCGTTGTCCGGAATTACTGGGTGTAAAGGGAGCGTAGGCGGGGAGACAAGTTGAATGTTTAAACTATCGGCTCAACTGATAGTCGCGTTCAAAACTATCACTCTTGAGTGCAGTAGAGGTAGGCGGAATTCCTAGTGTAGCGGTGAAATGCGTAGATATTAGGAGGAACACCAGTGGCGAAGGCGGCCTACTGGGCTGTAACTGACGCTGAGGCTCGAAAGCGTGGGTAGCAAACA"
## [10] "ACAAGCGTTGTCCGGAATTACTGGGTGTAAAGGGAGCGCAGGCGGGCGATCAAGTTGGAAGTGAAATCCATGGGCTCAACCCATGAACTGCTTTCAAAACTGATTGTCTTGAGTAGTGCAGAGGTAGGCGGAATTCCCGGTGTAGCGGTGGAATGCGTAGATATCGGGAGGAACACCAGTGGCGAAGGCGGCCTACTGGGCACCAACTGACGCTGAGGCTCGAAAGTGTGGGTAGCAAAC"
## [11] "ACAAGCGTTGTCCGGAATTACTGGGTGTAAAGGGAGCGCAGGCGGGAAGACAAGTTGGAAGTGAAATCTATGGGCTCAACCCATAAACTGCTTTCAAAACTGCTGGCCTTGAGTAGTGCAGAGGTAGGTGGAATTCCCGGTGTAGCGGTGGAATGCGTAGATATCGGGAGGAACACCAGTGGCGAAGGCGGCCTACTGGGCACCAACTGACGCTGAGGCTCGAAAGTGTGGGTAGCAAAC"
## [12] "ACAAGCGTTGTCCGGAATTACTGGGTGTAAAGGGAGCGCAGGCGGGAAGACAAGTTGGGAGTGAAATCTATGGGCTCAACCCATAAACTGCTTTCAAAACTGTTTTTCTTGAGTAGTGCAGAGGTAGGCGGAATTCCCGGTGTAGCGGTGGAATGCGTAGATATCGGGAGGAACACCAGTGGCGAAGGCGGCCTACTGGGCACCAACTGACGCTGAGGCTCGAAAGTGTGGGTAGCAAAC"
## [13] "ACAAGCGTTGTCCGGAATTACTGGGTGTAAAGGGAGCGCAGGCGGGAAGACAAGTTGGAAGTGAAATCTATGGGCTCAACCCATAAACTGCTTTCAAAACTGTTTTTCTTGAGTAGTGCAGAGGTAGGCGGAATTCCCGGTGTAGCGGTGGAATGCGTAGATATCGGGAGGAACACCAGTGGCGAAGGCGGCCTACTGGGCACCAACTGACGCTGAGGCTCGGAAGTGTGGGTAGCAAAC"
## [14] "ACAAGCGTTGTCCGGAACTACTGGGTGTAAAGGGAGCGCAGGCGGGAAGACAAGTTGGAAGTGAAATCTATGGGCTCAACCCATAAACTGCTTTCAAAACTGTTTTTCTTGAGTAGTGCAGAGGTAGGCGGAATTCCCGGTGTAGCGGTGGAATGCGTAGATATCGGGAGGAACACCAGTGGCGAAGGCGGCCTACTGGGCACCAACTGACGCTGAGGCTCGAAAGTGTGGGTAGCAAAC"
## [15] "GCAAGCGTTGTCCGGAATTACTGGGTGTAAAGGGCGTGTAGGCGGAGCTGCAAGTCAGATGTGAAATCCCGGGGCTCAACCCCGGAACTGCATTTGAAACTGTAGCCCTTGAGTATCGGAGAGGCAAGCGGAATTCCTAGTGTAGCGGTGAAATGCGTAGATATTAGGAGGAACACCAGTGGCGAAGGCGGCTTGCTGGACGACAACTGACGCTGAGGCGCGAAAGCGTGGGGAGCAAAC"
## [16] "GCAAGCGTTGTCCGGATTTACTGGGTGTAAAGGGCGTGTAGGCGGAGCAGCAAGTCAGAAGTGAAATCTCTGGGCTCAACCCAGAAACTGCTTTTGAAACTGTTGCCCTTGAGTATCGGAGAGGCAGGCGGAATTCCTAGTGTAGCGGTGAAATGCGTAGATATTAGGAGGAACACCAGTGGCGAAGGCGGCCTGCTGGACGACAACTGACGCTGAGGCGCGAAAGCGTGGGGAGCAAAC"
## [17] "GCAAGCGTTGTCCGGATTTACTGGGTGTAAAGGGCGTGCAGCCGGGCCGGCAAGTCAGATGTGAAATCCACGGGCTTAACCCGTGAACTGCATTTGAAACTGTTGGTCTTGAGTATCGGAGAGGTAATCGGAATTCCTTGTGTAGCGGTGAAATGCGTAGATATAAGGAAGAACACCAGTGGCGAAGGCGGATTACTGGACGACAACTGACGGTGAGGCGCGAAAGCGTGGGGAGCAAAC"
## [18] "GCAAGCGTTATCCGGATTTATTGGGTGTAAAGGGTGTGTAGGCGGGACTGCAAGTCAGACGTGAAAATCATGGGCTCAACCCATGACTTGCGTTTGAAACTGCGGTTCTTGAGAGTGGGAGAGGTAAACGGAATTCCTGGTGTAGCGGTGAAATGCGTAGATATCAGGAGGAACACCGGTGGCGAAGGCGGTTTACTGGACCACAACTGACGCTGAGACACGAAAGCGTGGGGAGCAAAC"
## [19] "GCGAGCGTTATCCGGAATTACTGGGTGTAAAGGGTGTGTAGGCGGGGTGTCAAGTCAGATGTGAAAACTGTGGGCTCAACCCACAAACTGCATTTGAAACTGATACTCTTGAGAGTGGGAGAGGTAAACGGAATTCCTGGTGTAGTAGTGAAATGCGTAGATATCAGGAGGAACACCGGTGGCGAAGGCGGTTTACTGGACCACAACTGACGCTGAGACACGAAAGCGTGGGGAGCAAAC"
## [20] "GCAAGCGTTATCCGGATTTACTGGGTGTAAAGGGCGTGTAGGCGGGACTGCAAGTCAGGTGTGAAAACCAGGGGCTCAACCTCTGGCCTGCATTTGAAACTGTAGTTCTTGAGTGCTGGAGAGGCAATCGGAATTCCGTGTGTAGCGGTGAAATGCGTAGATATACGGAGGAACACCAGTGGCGAAGGCGGATTGCTGGACAGTAACTGACGCTGAGGCGCGAAAGCGTGGGGAGCAAAC"
## [21] "GCGAGCGTTGTCCGGAATTACTGGGCGTAAAGGGCGCGTAGGCGGCCAATTAAGTTAGATGTGAAATCCCCGGGCTTAACCTGGGTGTTGCATTTAAAACTGATAGGCTTGAGTGCAGGAGAGGGAAGCGGAATTCCTAGTGTAGCGGTGGAATGCGTAGATATTAGGAGGAACACCAGTGGCGAAGGCGGCTTTCTGGACTGTAACTGACGCTGAGGCGCGAGAGCGTGGGGAGCAAAC"
## [22] "GCGAGCGTTAATCGGAATTACTGGGCGTAAAGCGCACGTAGGCTGTATGTCAAGTCAAGGGTGAAATCCCACGGCTCAACCGTGGAACTGCCTTTGAAACTGGCAAACTGGAGTATGTGAGAGGGCGGCGGAATTCCTGGTGTAGGAGTGAAATCCGTAGATATCAGGAGGAACATCAGTGGCGAAGGCGGCCGCCTGGCACAAAACTGACGCTGAGGTGCGAAAGCGTGGGTAGCAAAC"
## [23] "GCAAGCGTTATCCGGAATTATTGGGCGTAAAGGGCTCGTAGGCGGTTCGTCGCGTCCGGTGTGAAAGTTCATCGCTTAACGGTGGATCCGCGCCGGGTACGGGCGGGCTTGAGTGCGGTAGGGGAGACTGGAATTCCCGGTGTAACGGTGGAATGTGTAGATATCGGGAAGAACACCAATGGCGAAGGCAGGTCTCTGGGCCGTTACTGACGCTGAGGAGCGAAAGCGTGGGGAGCGAAC"
## [24] "CCGAGCGTTATCCGGATTTATTGGGTTTAAAGGGAGCGTAGATGGATGTTTAAGTCAGTTGTGAAAGTTTGCGGCTCAACCGTAAAATTGCAGTTGATACTGGCAGTCTTGAGTGCAGTAGAGGTGGGCGGAATTCGTGGTGTAGCGGTGAAATGCTTAGATATCACGAAGAACTCCGATTGCGAAGGCAGCCTGCTAAGCTGCAACTGACATTGAGGCTCGAAAGTGTGGGTATCAAAC"
## [25] "CCGAGCGTTATCCGGATTTATTGGGTTTAAAGGGAGCGTAGGTGGACAGTTAAGTCAGTTGTGAAAGTTTGCGGCTCAACCGTAAAATTGCAGTTGATACTGGCTGTCTTGAGTACAGTAGAGGTGGGCGGAATTCGTGGTGTAGCGGTGAAATGCTTAGATATCACGAAGAACTCCGATTGCGAAGGCAGCTCACTGGAGCGCAACTGACGCTGAAGCTCGAAAGTGCGGGTATCGAAC"
## [26] "CCGAGCGTTATCCGGATTTATTGGGTTTAAAGGGAGCGTAGGCGGATTATTAAGTCAGTTGTGAAAGTTTGCGGCTCAACCGTAAAATTGCAGTTGATACTGGTAGTCTTGAGTGCAGCAGAGGTAGGCGGAATTCGTGGTGTAGCGGTGAAATGCTTAGATATCACGAAGAACTCCGATTGCGAAGGCAGCTTACTGGACTGTAACTGACGCTGATGCTCGAAAGTGTGGGTATCAAAC"
## [27] "GCGAGCGTTATCCGGATTTATTGGGTTTAAAGGGTGCGTAGGCGGCCCTATAAGTCAGCGGTGAAATGTTCCGGCTCAACCGGGAAACTGCCGTTGAAACTGTAGAGCTAGAGTCCACAAGAGGTATGCGGAATGCGTGGTGTAGCGGTGAAATGCATAGATATCACGCAGAACCCCGATTGCGAAGGCAGCATACTGGGGTGAAACAGACGCTGAAGCACGAAAGCGTGGGTATCGAAC"
## [28] "GCGAGCGTTATCCGGATTTATTGGGTTTAAAGGGTGCGTAGGCGGAAGAATAAGTCAGCGGTGAAATGCTTCAGCTCAACTGGAGAATTGCCGATGAAACTGTTTTTCTAGAGTATAAAAGAGGTATGCGGAATGCGTGGTGTAGCGGTGAAATGCATAGATATCACGCAGAACCCCGATTGCGAAGGCAGCATACTGGGCTATAACTGACGCTGAAGCACGAAAGCGTGGGTATCGAAC"
## [29] "CCGAGCGTTATCCGGATTTATTGGGTTTAAAGGGTGCGTAGGCTGTTTTTTAAGTTAGAGGTGAAAGCTCGACGCTCAACGTCGAAATTGCCTCTGATACTGAGAGACTAGAGTGTAGTTGCGGAAGGCGGAATGTGTGGTGTAGCGGTGAAATGCTTAGATATCACACAGAACACCGATTGCGAAGGCAGCTTTCCAAGCTATTACTGACGCTGAGGCACGAAAGCGTGGGGAGCGAAC"
## [30] "GCGAGCGTTATCCGGATTTATTGGGTTTAAAGGGTGCGTAGGCGGGTGATTAAGTCAGCGGTGAAATGCGTCAGCTTAACTGGCGAACTGCCATTGAAACTGGTTACCTTGAGTGTAGCGGAAGTATGCGGAATGCGTGGTGTAGCGGTGAAATGCATAGATATCACGCAGAACTCCGATTGCGAAGGCAGCATACCATACTATAACTGACGCTGAAGCACGAAAGCGTGGGTATCGAAC"
## [31] "GCGAGCGTTATCCGGATTTATTGGGTTTAAAGGGTGCGTAGGCGGCACGCCAAGTCAGCGGTGAAATTTTCGGGCTCAACCCGGACTGTGCCGTTGAAACTGGCGAGCTAGAGTGCACAAGAGGCAGGCGGAATGCGTGGTGTAGCGGTGAAATGCATAGATATCACGCAGAACCCCGATTGCGAAGGCAGCCTGCTAGGGTGCGACAGACGCTGAGGCACGAAAGCGTGGGTATCGAAC"
## [32] "GCAAGCGTTGTCCGGATTTATTGGGCGTAAAGCGAGCGCAGGCGGAAGAATAAGTCTGATGTGAAAGCCCTCGGCTTAACCGAGGAACTGCATCGGAAACTGTTTTTCTTGAGTGCAGAAGAGGAGAGTGGAACTCCATGTGTAGCGGTGGAATGCGTAGATATATGGAAGAACACCAGTGGCGAAGGCGGCTCTCTGGTCTGCAACTGACGCTGAGGCTCGAAAGCATGGGTAGCGAAC"
## [33] "GCAAGCGTTGTCCGGAATTATTGGGCGTAAAGCGCGCGCAGGCGGCTTCTTAAGTCCATCTTAAAAGTGCGGGGCTTAACCCCGTGATGGGATGGAAACTGGGAGGCTGGAGTATCGGAGAGGAAAGTGGAATTCCTAGTGTAGCGGTGAAATGCGTAGAGATTAGGAAGAACACCGGTGGCGAAGGCGACTTTCTGGACGACAACTGACGCTGAGGCGCGAAAGCGTGGGGAGCAAACA"
## [34] "GCGAGCGTTGTCCGGAATTATTGGGCGTAAAGAGTACGTAGGCGGTTTGCTAAGCGCAAGGTGAAAGGCAGTGGCTTAACCATTGTAAGCCTTGCGAACTGGCAGACTTGAGTGCAGGAGAGGAAAGCGGAATTCCTAGTGTAGCGGTGAAATGCGTAGATATTAGGAGGAACACCGGTGGCGAAGGCGGCTTTCTGGACTGTAACTGACGCTGAGGTACGAAAGCGTGGGGAGCAAACA"
## [35] "GCAAGCGTTGTCCGGAATTATTGGGCGTAAAGAGTACGTAGGCGGTCTGGTAAGCGCAAGGTGAAAGGCATAGGCTCAACCAATGTCAGCCTTGCGAACTGTCAGACTTGAGTGCAGGAGGGGAAAGTGGAATTCCTAGTGTAGCGGTGAAATGCGTAGATATTAGGAGGAACACCAGTGGCGAAGGCGACTTTCTGGACTGTAACTGACGCTGAGGTACGAAAGCGTGGGGAGCAAACA"
## [36] "GCAAGCGTTGTCCGGAATGATTGGGCGTAAAGGGCGCGTAGGCGGCCAACTAAGTCTGGAGTGAAAGTCCTGCTTTTAAGGTGGGAATTGCTTTGGAAACTGGATGGCTTGAGTGCAGGAGAGGTAAGCGGAATTCCCGGTGTAGCGGTGAAATGCGTAGAGATCGGGAGGAACACCAGTGGCGAAGGCGGCTTACTGGACTGTAACTGACGCTGAGGCGCGAAAGTGTGGGGAGCAAAC"
## [37] "GCGAGCGTTGTCCGGAATTACTGGGCGTAAAGGGAGCGTAGGCGGTCTGATAAGTTGGATGTGAAATACCCGGGCTTAACTTGGGGGGTGCATCCAATACTGTTGGACTAGAGTACAGGAGAGGAAAGCGGAATTCCTAGTGTAGCGGTGAAATGCATAGATATTAGGAGGAACATCGGTGGCGAAGGCGGCTTTCTGGACTGCAACTGACGCTGAGGCTCGAAAGCGTGGGGAGCAAAC"
## [38] "GCAAGCGTTGTCCGGAATCACTGGGCGTAAAGGGCGCGTAGGCGGCAATATAAGTCAGATGTGAAAGGTGAGGGCTCAACCCTTAGACTGCATCTGATACTGTATAGCTTGAGTGTGAGAGAGGAAAGCGGAATTCCTAGTGTAGCGGTGAAATGCGTAGATATTAGGAGGAACACCAGTGGCGAAGGCGGCTTTCTGGCTCATAACTGACGCTGAGGCGCGAAAGCGTGGGGAGCAAAC"
## [39] "GCGAGCGTTGTCCGGATTTACTGGGTGTAAAGGGTGCGTAGGCGGCTAGACAAGTCAGGTGTGAAATACCGCAGCTCAACTGCGGGGCTGCACTTGAAACTGTAGAGCTTGAGTGATGGAGAGGTAAGCGGAATTCCTAGTGTAGCGGTAAAATGCGTAGATATTAGGAGGAACACCAGTGGCGAAGGCGGCTTACTGGACATTAACTGACGCTGAGGCACGAAAGCGTGGGGAGCAAAC"
## [40] "GCAAGCGTTATCCGGATCTACTGGGTGTAAAGGGAGCGTAGACGGATGGACAAGTCTGATGTGAAAGGCTGGGGCTCAACCCCGGGACTGCATTGGAAACTGCCCGTCTTGAGTGCCGGAGAGGTAAGCGGAATTCCTAGTGTAGCGGTGAAATGCGTAGATATTAGGAGGAACACCAGTGGCGAAGGCGGCTTACTGGACGGTAACTGACGTTGAGGCTCGAAAGCGTGGGGAGCAAAC"
## [41] "GCAAGCGTTATCCGGATTTACTGGGTGTAAAGGGAGCGTAGACGGATGGACAAGTCTGATGTGAAAGGCTGGGGCTCAACCCCGGGACTGCATTGGAAACTGCCCGTCTTGAGTGCCGGAGAGGTAAGCGGAATTCCTAGTGTAGCGGTGAAATGCGTAGATATACGGAGGAACACCAGTGGCGAAGGCGGCCTGCTGGACATTAACTGACGCTGAGGCGCGAAAGCGTGGGGAGCAAAC"
## [42] "GCAAGCGTTATCCGGATTTACTGGGTGTAAAGGGAGCGCAGGCGGCATGATAAGTCTGATGTGAAAACCCAAGGCTCAACCATGGGACTGCATTGGAAACTGTCGTGCTGGAGTGTCGGAGAGGTAAGCGGAATTCCTAGTGTAGCGGTGAAATGCGTAGATATTAGGAGGAACACCAGTGGCGAAGGCGGCTTACTGGACGGTAACTGACGTTGAGGCTCGAAAGCGTGGGGAGCAAAC"
## [43] "GCAAGCGTTATCCGGATTTACTGGGTGTAAAGGGAGCGTAGGCGGTCCTGCAAGTCTGATGTGAAAGGCCGGGGCTCAACCCCGGGACTGCATTGGAAACTGTAGGACTAGAGTGTCGGAGGGGTAAGTGGAATTCCTAGTGTAGCGGTGAAATGCGTAGATATTAGGAGGAACACCAGTGGCGAAGGCGGCTTACTGGACGGTAACTGACGTTGAGGCTCGAAAGCGTGGGGAGCAAAC"
## [44] "GCAAGCGTTATCCGGATTTACTGGGTGTAAAGGGAGCGTAGGCGGTCCTGCAAGTCTGATGTGAAAACCCGGGGCTCAACCCCGGGACTGCATTGGAAACTGTAGGACTAGAGTGTCGGAGGGGTAAGTGGAATTCCTAGTGTAGCGGTGAAATGCGTAGATATTAGGAGGAACACCAGTGGCGAAGGCGGCTTACTGGACGACCACTGACGCTGAGGCTCGAAAGCGTGGGGAGCAAAC"
## [45] "GCAAGCGTTATCCGGATTTACTGGGTGTAAAGGGAGCGTAGACGGTATGGCAAGTCTGATGTGAAAGGCCAGGGCTCAACCCTGGGACTGCATTGGAAACTGTCGAACTAGAGTGTCGGAGAGGCAAGTGGAATTCCTAGTGTAGCGGTGAAATGCGTAGATATTAGGAGGAACACCAGTGGCGAAGGCGGCTTACTGGACGGTAACTGACGTTGAGGCTCGAAAGCGTGGGGAGCAAAC"
## [46] "GCAAGCGTTATCCGGATTTACTGGGTGTAAAGGGAGCGTAGACGGCATGGCAAGTCTGAAGTGAAATGCGGGGGCTCAACCCCTGAACTGCTTTGGAAACTGTCAGGCTGGAGTGCAGGAGAGGTAAGTGGAATTCCTAGTGTAGCGGTGAAATGCGTAGATATTAGGAGGAACACCAGTGGCGAAGGCGGCTTACTGGACTGTAACTGACGTTGAGGCTCGAAAGCGTGGGGAGCAAAC"
## [47] "GCAAGCGTTATCCGGATTTACTGGGTGTAAAGGGTGCGTAGGTGGTGAGACAAGTCTGAAGTGAAAATCCGGGGCTTAACCCCGGAACTGCTTTGGAAACTGCCTGACTAGAGTACAGGAGAGGTAAGTGGAATTCCTAGTGTAGCGGTGAAATGCGTAGATATTAGGAGGAACACCAGTGGCGAAGGCGACTTACTGGACTGCTACTGACACTGAGGCACGAAAGCGTGGGGAGCAAAC"
## [48] "GCAAGCGTTATCCGGATTTACTGGGTGTAAAGGGAGCGTAGACGGCATGGCAAGCCAGATGTGAAAGCCCGGGGCTCAACCCCGGGACTGCATTTGGAACTGTCAGGCTAGAGTGTCGGAGAGGAAAGCGGAATTCCTAGTGTAGCGGTGAAATGCGTAGATATTAGGAGGAACACCAGTGGCGAAGGCGGCTTACTGGACGGTAACTGACGTTGAGGCTCGAAAGCGTGGGGAGCAAAC"
## [49] "GCAAGCGTTATCCGGAATTACTGGGTGTAAAGGGTGAGTAGGCGGCACGGCAAGTAAGATGTGAAAGCCCGAGGCTTAACCTCGGGATTGCATTTTAAACTGCTGAGCTAGAGTACAGGAGAGGAAAGCGGAATTCCTAGTGTAGCGGTGAAATGCGTAGATATTAGGAAGAACACCAGTGGCGAAGGCGGCTTTCTGGACTGAAACTGACGCTGAGGCACGAAAGCGTGGGGAGCGAAC"
## [50] "GCAAGCGTTATCCGGATTTACTGGGTGTAAAGGGAGCGTAGGTGGCAAGGCAAGCCAGAAGTGAAAACCCGGGGCTCAACCGCGGGATTGCTTTTGGAACTGTCATGCTAGAGTGCAGGAGGGGTGAGCGGAATTCCTAGTGTAGCGGTGAAATGCGTAGATATTAGGAGGAACACCAGTGGCGAAGGCGGCTTACTGGACGATAACTGACGCTGAGGCTCGAAAGCGTGGGGAGCAAAC"
## 
## $y_only
##  [1] "GCAAGCGTTGTCCGGATTTACTGGGTGTAAAGGGCGTGCAGCCGGGCATGCAAGTCAGATGTGAAATCTCAGGGCTTAACCCTGAAACTGCATTTGAAACTGTATGTCTTGAGTGCCGGAGAGGTAATCGGAATTCCTTGTGTAGCGGTGAAATGCGTAGATATAAGGAAGAACACCAGTGGCGAAGGCGGATTACTGGACGGTAACTGACGGTGAGGCGCGAAAGCGTGGGGAGCGAAC"
##  [2] "GCGAGCGTTGTCCGGAATTACTGGGTGTAAAGGGAGCGTAGGCGGGATCTTAAGTCAGGTGTGAAAACTATGGGCTCAACCCATAGACTGCACTTGAAACTGAGGTTCTTGAGTGAAGTAGAGGCAGGCGGAATTCCTAGTGTAGCGGTGAAATGCGTAGATATTAGGAGGAACATCAGTGGCGAAGGCGGCCTGCTGGGCTTTTACTGACGCTGAGGCTCGAAAGCGTGGGGAGCAAAC"
##  [3] "GCGAGCGTTGTCCGGAATTACTGGGTGTAAAGGGAGCGTAGGCGGGATGGCAAGTCAGATGTGAAAACTATGGGCTCAACCCATAGACTGCATTTGAAACTGTTGTTCTTGAGTGAGGTAGAGGTAAGCGGAATTCCTGGTGTAGCGGTGAAATGCGTAGAGATCAGGAGGAACATCGGTGGCGAAGGCGGCTTACTGGGCCTTTACTGACGCTGAGGCTCGAAAGCGTGGGGAGCAAAC"
##  [4] "GCGAGCGTTGTCCGGAATTACTGGGTGTAAAGGGAGCGTAGGCGGGATTGCAAGTCAGATGTGAAAACTATGGGCTTAACCCATAGACTGCATTTGAAACTGTAGTTCTTGAGTGAAGTAGAGGTAAGCGGAATTCCTAGTGTAGCGGTGAAATGCGTAGATATTAGGAGGAACATCGGTGGCGAAGGCGGCTTACTGGGCTTTTACTGACGCTGAGGCTCGAAAGCGTGGGGAGCAAAC"
##  [5] "GCAAGCGTTGTCCGGATTTACTGGGTGTAAAGGGCGTGTAGGCGGAGATGCAAGTTAGGAGTGAAATCTATGGGCTCAACCCATAAACTGCTTCTAAAACTGTATCCCTTGAGTATCGGAGAGGCAAGCGGAATTCCTAGTGTAGCGGTGAAATGCGTAGATATTAGGAGGAACACCAGTGGCGAAGGCGGCTTGCTGGACGACAACTGACGCTGAGGCGCGAAAGCGTGGGGAGCAAAC"
##  [6] "GCAAGCGTTGTCCGGATTTACTGGGTGTAAAGGGCGTGCAGCCGGGCCGGCAAGTCAGATGTGAAATCTGGAGGCTTAACCTCCAAACTGCATTTGAAACTGTAGGTCTTGAGTACCGGAGAGGTTATCGGAATTCCTTGTGTAGCGGTGAAATGCGTAGATATAAGGAAGAACACCAGTGGCGAAGGCGGATAACTGGACGGCAACTGACGGTGAGGCGCGAAAGCGTGGGGAGCAAAC"
##  [7] "GCAAGCGTTGTCCGGATTTACTGGGTGTAAAGGGCGTGCAGCCGGGTCTGCAAGTCAGATGTGAAATCCATGGGCTCAACCCATGAACTGCATTTGAAACTGTAGATCTTGAGTGTCGGAGGGGCAATCGGAATTCCTAGTGTAGCGGTGAAATGCGTAGATATTAGGAGGAACACCAGTGGCGAAGGCGGATTGCTGGACGATAACTGACGGTGAGGCGCGAAAGTGTGGGGAGCAAAC"
##  [8] "GCAAGCGTTGTCCGGATTTACTGGGTGTAAAGGGCGTGCAGCCGGGAATGCAAGTCAGATGTGAAATCCATGGGCTTAACCCATGAACTGCATTTGAAACTGTATTTCTTGAGTACTGGAGAGGCAATCGGAATTCCTAGTGTAGCGGTGAAATGCGTAGATATTAGGAGGAACACCAGTGGCGAAGGCGGATTGCTGGACAGCAACTGACGGTGAGGCGCGAAAGTGTGGGGAGCAAAC"
##  [9] "GCGAGCGTTGTTCGGAATTACTGGGCGTAAAGCGCACGCAGGCGGTTCGTTAAGTCTGCTGTCAAAGGCTGAGGCTCAACCTCAGTTCTACAACAGATACTGGCGGACTAGAGTATGTGAGAGGGAAGTGGAATTCCCGGTGTAGCGGTGAAATGCGTAGATATCGGGAGGAACACCAGTGGCGAAGGCGGCTTCCTGGCACACTACTGACGCTCATGTGCGAAAGCCAGGGCAGCGAAC"
## [10] "GCAAGCGTTGTCCGGAATTATTGGGCGTAAAGGGCGCGCAGGCGGCGTCGTAAGTCGGTCTTAAAAGTGCGGGGCTTAACCCCGTGAGGGGACCGAAACTGCGATGCTAGAGTATCGGAGAGGAAAGCGGAATTCCTAGTGTAGCGGTGAAATGCGTAGATATTAGGAGGAACACCAGTGGCGAAAGCGGCTTTCTGGACGACAACTGACGCTGAGGCGCGAAAGCCAGGGGAGCAAACG"
## [11] "GCAAGCGTTGTCCGGAATTATTGGGCGTAAAGGGAGCGCAGGCGGGAAACTAAGCGGATCTTAAAAGTGCGGGGCTCAACCCCGTGATGGGGTCCGAACTGGTTTTCTTGAGTGCAGGAGAGGAAAGCGGAATTCCCAGTGTAGCGGTGAAATGCGTAGATATTGGGAAGAACACCAGTGGCGAAGGCGGCTTTCTGGACTGTAACTGACGCTGAGGCTCGAAAGCTAGGGTAGCGAACG"
## [12] "GCGAGCGTTGTCCGGATTTACTGGGCGTAAAGGGAGCGTAGGCGGATTTTTAAGTGAGATGTGAAATACTCGGGCTTAACCTGAGTGCTGCATTTCAAACTGGAAGTCTAGAGTGCAGGAGAGGAGAAGGGAATTCCTAGTGTAGCGGTGAAATGCGTAGAGATTAGGAAGAACACCAGTGGCGAAGGCGCTTCTCTGGACTGTAACTGACGCTGAGGCTCGAAAGCGTGGGGAGCAAAC"
## [13] "GCAAGCGTTATCCGGATTTACTGGGTGTAAAGGGAGCGTAGACGGCGAAGCAAGTCTGAAGTGAAAACCCAGGGCTCAACCCTGGGACTGCTTTGGAAACTGTTTTGCTAGAGTGTCGGAGAGGTAAGTGGAATTCCTAGTGTAGCGGTGAAATGCGTAGATATTAGGAGGAACACCAGTGGCGAAGGCGGCTTACTGGACGATAACTGACGTTGAGGCTCGAAAGCGTGGGGAGCAAAC"
## [14] "GCAAGCGTTATCCGGAATTACTGGGTGTAAAGGGTGCGTAGGCGGTATGGCAAGTTTGATGTGAAACCCACAGGCTTAACCTGTGGCTTGCATCGAAAACTACTGAACTAGAGTGCAGGAGAGGAAAGCGGAATTCCTAGTGTAGCGGTGAAATGCGTAGATATTAGGAAGAACACCAGTGGCGAAGGCGGCTTTCTGGACTGCAACTGACGCTGAGGCACGAAAGCGTGGGGAGCAAAC"
## 
## $z_only
##   [1] "GCAAGCGTTATCCGGATTTATTGGGTGTAAAGGGTGCGTAGACGGGAATACAAGTTAGTTGTGAAATCCCTCGGCTTAACTGAGGAACTGCAACTAAAACTATATTTCTTGAGTGCTGGAGAGGAAAGTGGAATTCCTAGTGTAGCGGTGAAATGCGTAGATATTAGGAGGAACACCAGTGGCGAAGGCGACTTTCTGGACAGTAACTGACGTTGAGGCACGAAAGTGTGGGGAGCAAAC"
##   [2] "GCGAGCGTTATCCGGATTTATTGGGTGTAAAGGGTGCGTAGACGGGAAATTAAGTTAGTTGTGAAATCCCTCGGCTCAACTGAGGAACTGCAACTAAAACTGATTTTCTTGAGTACTGGAGAGGAAAGTGGAATTCCTAGTGTAGCGGTGAAATGCGTAGATATTAGGAGGAACACCAGTGGCGAAGGCGACTTTCTGGACAGAAACTGACGTTGAGGCACGAAAGTGTGGGGAGCAAAC"
##   [3] "GCAAGCGTTATCCGGATTTATTGGGTGTAAAGGGTGCGTAGACGGGAAATTAAGTTAGTTGTGAAATCCCTCGGCTCAACTGAGGAACTGCAACTAAAACTGGTTTTCTTGAGTGCAGGAGAGGTAAGTGGAATTCCTAGTGTAGCGGTGAAATGCGTAGATATTAGGAGGAACACCAGTGGCGAAGGCGACTTACTGGACTGTAACTGACGTTGAGGCACGAAAGTGTGGGGAGCAAAC"
##   [4] "GCAAGCGTTGTCCGGATTTACTGGGTGTAAAGGGCGTGTAGGCGGGCATGCAAGTTGGATGTGAAATGTCACGGCTTAACCGTGGAGCTGCATCCAAAACTGCAAGTCTTGAGTGCCGGAGAGGAAAGCGGAATTCCTAGTGTAGCGGTGAAATGCGTAGATATTAGGAGGAACACCGGTGGCGAAGGCGGCTTTCTGGACGGTAACTGACGCTGAGGCGCGAAAGCGTGGGGAGCAAAC"
##   [5] "GCGAGCGTTGTCCGGAATGACTGGGTGTAAAGGGAGCGTAGGCGGGATGGCAAGTCAGATGTGAAACCTGAGGGCTCAACCTTCAGACTGCATTTGAAACTGCTGTTCTTGAGTGAAGTAGAGGTAAGCGGAATTCCTGGTGTAGCGGTGAAATGCGTAGAGATCAGGAGGAACATCGGTGGCGAAGGCGGCTTACTGGGCTTTTACTGACGCTGAGGCTCGAAAGCGTGGGGAGCAAAC"
##   [6] "GCAAGCGTTGTCCGGATTTACTGGGTGTAAAGGGAGCGCAGGCGGGAGAGCAAGTCAGCGGTGAAATACATGGGCTTAACCCATGGGCTGCCGTTGAAACTGTCCTTCTTGAGTGAAGTAGAGGCAAGCGGAATTCCGAGTGTAGCGGTGAAATGCGTAGATATTCGGAGGAACACCAGTGGCGAAGGCGGCTTGCTGGGCTTTTACTGACGCTGAGGCTCGAAAGTGTGGGGAGCAAAC"
##   [7] "GCAAGCGTTGTCCGGAATTACTGGGTGTAAAGGGAGCGTAGGCGGGAAGATAAGTTGGACGTCTAATCTATCGGCTCAACCGATAGTCGCGTTCAAAACTGTTTTTCTTGAGTGAAGTAGAGGTAAGCGGAATTCCTAGTGTAGCGGTGAAATGCGTAAATATTAGGAGGAACACCAGTGGCGAAGGCGGCTTACTGGGCTTTAACTGACGCTGAGGCTCGAAAGCGTGGGTAGCAAACA"
##   [8] "ACAAGCGTTGTCCGGAATTACTGGGTGTAAAGGGAGCGCAGGCGGGAGAACAAGTTGGAAGTGAAATCCATGGGCTCAACCCATGAACTGCTTTCAAAACTGTTTTTCTTGAGTAGTGCAGAGGTAGGCGGAATTCCCGGTGTAGCGGTGGAATGCGTAGATATCGGGAGGAACACCAGTGGCGAAGGCGGCCTACTGGGCACCAACTGACGCTGAGGCTCGAAAGTGTGGGTAGCAAAC"
##   [9] "ACAAGCGTTGTCCGGAATTACTGGGTGTAAAGGGAGCGCAGGCGGGAAGGCAAGTTGGAAGTGAAATCCATGGGCTCAACCCATGAACTGCTTTCAAAACTGTTTTTCTTGAGTAGTGCAGAGGTAGGCGGAATTCCCGGTGTAGCGGTGGAATGCGTAGATATCGGGAGGAACACCAGTGGCGAAGGCGGCCTACTGGGCACCAACTGACGCTGAGGCTCGAAAGTGTGGGTAGCAAAC"
##  [10] "ACAAGCGTTGTCCGGAATTACTGGGTGTAAAGGGAGCGCAGGCGGGAAAGCAAGTTGGAAGTGAAATCCATGGGCTCAACCCATGAACTGCTTTCAAAACTGTTTTTCTTGAGTAGTGCAGAGGTAGGCGGAATTCCCGGTGTAGCGGTGGAATGCGTAGATATCGGGAGGAACACCAGTGGCGAAGGCGGCCTACTGGGCACCAACTGACGCTGAGGCTCGAAAGTGTGGGTAGCAAAC"
##  [11] "GCAAGCGTTGTCCGGAATTACTGGGTGTAAAGGGAGCGCAGGCGGGAAGACAAGTTGGAAGTGAAAACCATGGGCTCAACCCATGAATTGCTTTCAAAACTGCTGGCCTTGAGTAGTGCAGAGGTAGGTGGAATTCCCGGTGTAGCGGTGGAATGCGTAGATATCGGGAGGAACACCAGTGGCGAAGGCGGTCTACTGGGCACCAACTGACGCTGAGGCTCGAAAGCATGGGTAGCAAAC"
##  [12] "GCAAGCGTTGTCCGGAATTACTGGGTGTAAAGGGAGCGCAGGCGGGAAGACAAGTTGGAAGTGAAAACCATGGGCTCAACCCATGAATTGCTTTCAAAACTGTTTTTCTTGAGTAGTGCAGAGGTAGATGGAATTCCCGGTGTAGCGGTGGAATGCGTAGATATCGGGAGGAACACCAGTGGCGAAGGCGGTCTACTGGGCACCAACTGACGCTGAGGCTCGAAAGCATGGGTAGCAAAC"
##  [13] "GCAAGCGTTGTCCGGAATTACTGGGTGTAAAGGGAGCGCAGGCGGACCGGCAAGTTGGAAGTGAAAACTATGGGCTCAACCCATAAATTGCTTTCAAAACTGCTGGCCTTGAGTAGTGCAGAGGTAGGTGGAATTCCCGGTGTAGCGGTGGAATGCGTAGATATCGGGAGGAACACCAGTGGCGAAGGCGACCTACTGGGCACCAACTGACGCTGAGGCTCGAAAGCATGGGTAGCAAAC"
##  [14] "GCAAGCGTTGTCCGGAATTACTGGGTGTAAAGGGAGCGCAGGCGGACCGGCAAGTTGGAAGTGAAATCCATGGGCTCAACCCGTGAATTGCTTTCAAAACTGCTGGCCTTGAGTAGTGCAGAGGTAGGTGGAATTCCCGGTGTAGCGGTGGAATGCGTAGATATCGGGAGGAACACCAGTGGCGAAGGCGACCTACTGGGCACCAACTGACGCTGAGGCTCGAAAGCATGGGTAGCAAAC"
##  [15] "GCAAGCGTTGTCCGGAATTACTGGGTGTAAAGGGAGCGCAGGCGGACCGGCAAGTTGGAAGTGAAAACCATGGGCTCAACCCGTGAATTGCTTTCAAAACTGCTGGCCTTGAGTAGTGCAGAGGTAGGTGGAATTCCCGGTGTAGCGGTGGAATGCGTAGATATCGGGAGGAACACCAGTGGCGAAGGCGACCTACTGGGCACCAACTGACGCTGAGGCTCGAAAGCATGGGTAGCAAAC"
##  [16] "GCAAGCGTTGTCCGGAATTACTGGGTGTAAAGGGAGCGCAGGCGGGCATGCAAGTTGGAAGTGAAAACTATGGGCTCAACCCATAGCCTGCTTTCAAAACTGCGTGTCTTGAGTAGTGCAGAGGTAGGCGGAATTCCCGGTGTAGCGGTGGAATGCGTAGATATCGGGAGGAACACCAGTGGCGAAGGCGGCCTACTGGGCACCAACTGACGCTGAGGCTCGAAAGCATGGGTAGCAAAC"
##  [17] "GCGAGCGTTATCCGGAATTACTGGGTGTAAAGGGTGTGTAGGCGGGAAGGCAAGTCAGATGTGAAAACCAAAGGCTCAACCTTTGGCTTGCATTTGAAACTGTTTTTCTTGAGAGTGGGAGAGGTAAACGGAATTCCTAGTGTAGTAGTGAAATGCGTAGATATTAGGAGGAACACCGGTGGCGAAGGCGGTTTACTGGACCACAACTGACGCTGAGACACGAAAGCGTGGGGAGCAAAC"
##  [18] "TCTAGTGGTAGCAGTTTTTATTGGGCCTAAAGCGTCCGTAGCCGGTTTAATAAGTCTCTGGTGAAATCCTGCAGCTTAACTGTGGGAATTGCTGGAGATACTATTAGACTTGAGATCGGGAGAGGTTAGAGGTACTCCCAGGGTAGAGGTGAAATTCTGTAATCCTGGGAGGACCGCCTGTTGCGAAGGCGTCTGACTGGAACGATTCTGACGGTGAGGGACGAAAGCTAGGGGCGCGAA"
##  [19] "GCAAGCGTTATCCGGATTTACTGGGTGTAAAGGGCGAGTAGGCGGATTGGCAAGTTGGGAGTGAAATGTCGGGGCTTAACCCCGGAACTGCTTCCAAAACTGTTGATCTTGAGTGATGGAGAGGCAGGCGGAATTCCCAGTGTAGCGGTGAAATGCGTAGATATTGGGAGGAACACCAGTGGCGAAGGCGGCCTGCTGGACATTAACTGACGCTGAGGAGCGAAAGCGTGGGGAGCAAAC"
##  [20] "GCAAGCGTTATCCGGATTTACTGGGTGTAAAGGGCGCGCAGGCGGGCCGGCAAGTTGGAAGTGAAATCCGGGGGCTTAACCCCCGAACTGCTTTCAAAACTGCTGGTCTTGAGTGATGGAGAGGCAGGCGGAATTCCGTGTGTAGCGGTGAAATGCGTAGATATACGGAGGAACACCAGTGGCGAAGGCGGCCTGCTGGACATTAACTGACGCTGAGGCGCGAAAGCGTGGGGAGCAAAC"
##  [21] "GCAAGCGTTAATCGGAATAACTGGGCGTAAAGCGCACGTAGGTGGTTCGACAAGTCAGATGTGAAAGCCCCGGGCTTAACCTGGGATGTGCATTTGAAACTGTTGGACTCGAGTACTGTAGAGGGAGGTAGAATTCCAGGTGTAGCGGTGAAATGCGTAGATATCTGGAGGAATACCAGTGGCGAAGGCGGCCTCCTGGACAGACACTGACACTGAGGTGCGAAAGCGTGGGGAGCAAAC"
##  [22] "GCAAGCGTTAATCGGAATCACTGGGCGTAAAGCGCACGTAGGCTGTTATGTAAGTCAGGGGTGAAATCCCACGGCTCAACCGTGGAACTGCCCTTGATACTGCACGACTTGAATCCGGGAGAGGGTGGCGGAATTCCAGGTGTAGGAGTGAAATCCGTAGATATCTGGAGGAACATCAGTGGCGAAGGCGGCCACCTGGACCGGTATTGACGCTGAGGTGCGAAAGCGTGGGGAGCAAAC"
##  [23] "GCGAGCGTTGTCCGGAATTATTGGGCGTAAAGAGCTTGTAGGCGGTTTGTCGCGTCTGCTGTGAAAGGCCGGGGCTTAACCCCGTGTATTGCAGTGGGTACGGGCAGACTAGAGTGCAGTAGGGGAGACTGGAATTCCTGGTGTAGCGGTGGAATGCGCAGATATCAGGAGGAACACCGATGGCGAAGGCAGGTCTCTGGGCTGTAACTGACGCTGAGAAGCGAAAGCATGGGGAGCGAA"
##  [24] "GCAAGCGTTATCCGGAATTATTGGGCGTAAAGGGCTCGTAGGCGGTTCGTCGCGTCCGGTGTGAAAGTCCATCGCTTAACGGTGGATCCGCGCCGGGTACGGGCGGGCTTGAGTGCGGTAGGGGAGACTGGAATTCCCGGTGTAACGGTGGAATGTGTAGATATCGGGAAGAACACCAATGGCGAAGGCAGGTCTCTGGGCCGTTACTGACGCTGAGGAGCGAAAGCGTGGGGAGCGAAC"
##  [25] "GCAAGCGTTATCCGGAATTATTGGGCGTAAAGGGCTCGTAGGCGGTTCGTCGCGTCCGGTGTGAAAGTCCATCGCTTAACGGTGGATCTGCGCCGGGTACGGGCGGGCTGGAGTGCGGTAGGGGAGACTGGAATTCCCGGTGTAACGGTGGAATGTGTAGATATCGGGAAGAACACCGACGGCGAAGGCAGCTCTCTGGGCCGAAACTGACGCTGAGGCGCGAAAGCTGGGGGAGCGAAC"
##  [26] "GCAAGCGTTATCCGGAATTATTGGGCGTAAAGGGCTCGTAGGCGGTTCGTCGCGTCCGGTGTGAAAGTCCATCGCTTAACGGTGGATCTGCGCCGGGTACGGGCGGGCTGGAGTGCGGTAGGGGAGACTGGAATTCCCGGTGTAACGGTGGAATGTGTAGATATCGGGAAGAACACCAATGGCGAAGGCAGGTCTCTGGGCCGTTACTGACGCTGAGGAGCGAAAGCGTGGGGAGCGAAC"
##  [27] "GCGAGCGTTGTCCGGAATTATTGGGCGTAAAGGGCTTGTAGGCGGTTGGTCGCGTCTGCCGTGAAATCCTCTGGCTTAACTGGGGGCGTGCGGTGGGTACGGGCTGACTTGAGTGCGGTAGGGGAGACTGGAACTCCTGGTGTAGCGGTGGAATGCGCAGATATCAGGAAGAACACCGGTGGCGAAGGCGGGTCTCTGGGCCGTTACTGACGCTGAGGAGCGAAAGCGTGGGGAGCGAAC"
##  [28] "GCAAGCGTTGTCCGGAATCATTGGGCGTAAAGAGTTCGTAGGCGGTTTGTTAAGTCTGGTGTTAAAGCCCGAAGCTCAACTTCGGTTCGGCACTGGATACTGGCAGACTAGAATGCGGTAGAGGTAAAGGGAATTCCTGGTGTAGCGGTGAAATGCGTAGATATCAGGAGGAACATCGGTGGCGTAAGCGCTTTACTGGGCCGTAATTGACGCTGAGGAACGAAAGCCAGGGTAGCGAAT"
##  [29] "TCAAGCGTTGTTCGGAATCACTGGGCGTAAAGCGTGCGTAGGCTGTTTCGTAAGTCGTGTGTGAAAGGCGCGGGCTCAACCCGCGGACGGCACATGATACTGCGAGACTAGAGTAATGGAGGGGGAACCGGAATTCTCGGTGTAGCAGTGAAATGCGTAGATATCGAGAGGAACACTCGTGGCGAAGGCGGGTTCCTGGACATTAACTGACGCTGAGGCACGAAGGCCAGGGGAGCGAAA"
##  [30] "GCGAGCGTTGTTCGGAATCATTGGGCGTAAAGGGCATGTAGGCGGTTACGCAAGCCTGGTGTGAAAGCCCGAGGCTCAACCTCGGGATGCGCCGGGAACTGTGCAACTAGAGTAGCTGAGGGGCAGCCGGAATTCCAGGTGTAGGGGTGAAATCTGTAGATATCTGGAAGAACACCGATGGCGAAGGCAGGCTGCCAGCAGATTACTGACGCTGAGGTGCGAAGGTGCGGGGAGCGAACA"
##  [31] "GCGAGCGTTGTTCGGAATCATTGGGCGTAAAGGGCGTGTAGGCGGCCCTGCAAGCCTGGCGTGAAATCCCGGGGCCCAACCCCGGAACCGCGCTGGGAACTGCTGGGCTTGAGCCGCTGTGGCGCAGCCGGAATTCCAGGTGTAGGGGTGAAATCTGTAGATATCTGGAAGAACACCGATGGCGAAGGCAGGCTGCGAGCGGACGGCTGACGCTGAGGCGCGAAGGCGCGGGGAGCGAAC"
##  [32] "CCGAGCGTTATCCGGATTTATTGGGTTTAAAGGGAGCGTAGGTGGATTGTTAAGTCAGTTGTGAAAGTTTGCGGCTCAACCGTAAAATTGCAGTTGAAACTGGCAGTCTTGAGTACAGTAGAGGTGGGCGGAATTCGTGGTGTAGCGGTGAAATGCTTAGATATCACGAAGAACTCCGATTGCGAAGGCAGCTCACTAGACTGCAACTGACACTGATGCTCGAAAGTGTGGGTATCAAAC"
##  [33] "CCAGGCGTTATCCGGATTTATTGGGTTTAAAGGGAGCGCAGGCGGACCTTTAAGTCAGCTGTGAAATACGGCGGCTCAACCGTCGAACTGCAGTTGATACTGGAGGTCTTGAGTGCACACAGGGATACTGGAATTCATGGTGTAGCGGTGAAATGCTCAGATATCATGAAGAACTCCAATCGCGAAGGCAGGTATCCGGGGTGCAACTGACGCTGAGGCTCGAAAGTGCGGGTATCAAAC"
##  [34] "CCAGGCGTTATCCGGATTCATTGGGTTTAAAGGGAGCGTAGGCCGCCTTTTAAGCGTGTTGTGAAATGTAGGGGCTCAACCCCTGCACTGCAGCGCGAACTGGAGGGCTTGAGTACACACAAAGTAGGCGGAATTCATGGTGTAGCGGTGAAATGCTTAGATATCATGAAGAACCCCGATTGCGAAGGCAGCTTACTGGAGTGTAACTGACGCTGAAGCTCGAAAGCGCGGGTATCGAAC"
##  [35] "CCGGGCGTTATCCGGATTTATTGGGTTTAAAGGGAGCGTAGGCTGTCCTTTAAGCGTGTTGTGAAATGTAGGCGCTCAACGCCTGCACTGCAGCGCGAACTGAAGGACTTGAGTGTGCACGACGCTGGCGGAATTCGTGGTGTAGCGGTGAAATGCTTAGATATCACGAAGAACTCCGATTGCGAAGGCAGCTCACTGGAGCGCAACTGACGCTGAAGCTCGAAAGTGCGGGTATCGAAC"
##  [36] "CCGGGCGTTATCCGGATTTATTGGGTTTAAAGGGAGCGTAGGCTGTCCTTTAAGCGTGTTGTGAAATGTAGGCGCTCAACGCCTGCACTGCAGCGCGAACTGAAGGACTTGAGTGTGCACGACGCTGGCGGAATTCGTGGTGTAGCGGTGAAATGCTTAGATATCACGAAGAACTCCGATTGCGAAGGCAGCTGGCGAGAGCACAACTGACGCTGAAGCTCGAAAGTGCGGGTATCGAAC"
##  [37] "CCGGGCGTTATCCGGATTTATTGGGTTTAAAGGGAGCGTAGGCCGGAGATTAAGCGTGTTGTGAAATGTAGATGCTCAACATCTGAACTGCAGCGCGAACTGGTTTCCTTGAGTACGCACAAAGTGGGCGGAATTCGTGGTGTAGCGGTGAAATGCTTAGATATCACGAAGAACTCCGATTGCGAAGGCAGCTCACTGGAGCGCAACTGACGCTGAAGCTCGAAAGTGCGGGTATCGAAC"
##  [38] "CCGGGCGTTATCCGGATTTATTGGGTTTAAAGGGAGCGTAGGCCGGAGATTAAGCGTGTTGTGAAATGTAGATGCTCAACATCTGCACTGCAGCGCGAACTGGTTTCCTTGAGTACGCACAAAGTGGGCGGAATTCGTGGTGTAGCGGTGAAATGCTTAGATATCACGAAGAACTCCGATTGCGAAGGCAGCTCACTGGAGCGCAACTGACGCTGAAGCTCGAAAGTGCGGGTATCGAAC"
##  [39] "CCGGGCGTTATCCGGATTTATTGGGTTTAAAGGGAGCGTAGGCCGGAGATTAAGCGTGTTGTGAAATGTAGAGGCTCAACCTCTGCACTGCAGCGCGAACTGGTCTTCTTGAGTACGCACAACGTGGGCGGAATTCGTGGTGTAGCGGTGAAATGCTTAGATATCACGAAGAACTCCGATTGCGAAGGCAGCTCACTGGAGCGCAACTGACGCTGAAGCTCGAAAGTGCGGGTATCGAAC"
##  [40] "CCGGGCGTTATCCGGATTTATTGGGTTTAAAGGGAGCGTAGGCCGGAGATTAAGCGTGTTGTGAAATGTAGTGGCTCAACCTCTGCACTGCAGCGCGAACTGGTCTTCTTGAGTACGCACAACGTGGGCGGAATTCGTGGTGTAGCGGTGAAATGCTTAGATATCACGAAGAACTCCGATTGCGAAGGCAGCTCACTGGAGCGCAACTGACGCTGAAGCTCGAAAGTGCGGGTATCGAAC"
##  [41] "CCGGGCGTTATCCGGATTTATTGGGTTTAAAGGGAGCGTAGGCCGGAGATTAAGCGTGTTGTGAAATGTAGTGGCTCAACCTCTGCACTGCAGCGCGAACTGGTCTTCTTGAGTACGCACAACGTGGGCGGAATTCGTGGTGTAGCGGTGAAATGCTTAGATATCACGAAGAACTCCGATTGCGAAGGCAGCTCACGGGAGCGCAACTGACGCTGAAGCTCGAAAGTGCGGGTATCGAAC"
##  [42] "CCGGGCGTTATCCGGATTTATTGGGTTTAAAGGGAGCGTAGGCCGGAGATTAAGCGTGTTGTGAAATGTAGAGGCTCAACCTCTGCACTGCAGCGCGAACTGGTCTTCTTGAGTACGCACAACGTGGGCGGAATTCGTGGTGTAGCGGTGAAATGCTTAGATATCACGAAGAACTCCGATTGCGAAGGCAGCTCACGGGAGCGCAACTGACGCTGAAGCTCGAAAGTGCGGGTATCGAAC"
##  [43] "CCGGGCGTTATCCGGATTTATTGGGTTTAAAGGGAGCGTAGGCCGGAGATTAAGCGTGTTGTGAAATGTAGATGCTCAACATCTGCACTGCAGCGCGAACTGGTCTTCTTGAGTACGCACAACGTGGGCGGAATTCGTGGTGTAGCGGTGAAATGCTTAGATATCACGAAGAACTCCGATTGCGAAGGCAGCTCACGGGAGCGCAACTGACGCTGAAGCTCGAAAGTGCGGGTATCGAAC"
##  [44] "CCGGGCGTTATCCGGATTTATTGGGTTTAAAGGGAGCGTAGGCCGGAGATTAAGCGTGTTGTGAAATGTAGACGCTCAACGTCTGCACTGCAGCGCGAACTGGTTTCCTTGAGTACGCACAAAGTGGGCGGAATTCGTGGTGTAGCGGTGAAATGCTTAGATATCACGAAGAACTCCGATTGCGAAGGCAGCTCACTGGAGCGCAACTGACGCTGAAGCTCGAAAGTGCGGGTATCGAAC"
##  [45] "CCGGGCGTTATCCGGATTTATTGGGTTTAAAGGGAGCGTAGGCCGGAGATTAAGCGTGTTGTGAAATGTAGACGCTCAACGTCTGCACTGCAGCGCGAACTGGTTTCCTTGAGTACGCACAAAGTGGGCGGAATTCGTGGTGTAGCGGTGAAATGCTTAGATATCACGAAGAACTCCGATTGCGAAGGCAGCTCACTGGGGCGCAACTGACGCTGAAGCTCGAAAGCGCGGGTATCGAAC"
##  [46] "CCGGGCGTTATCCGGATTTATTGGGTTTAAAGGGAGCGTAGGCCGTGAGGTAAGCGTGTTGTGAAATGTAGGCGCCCAACGTCTGCACTGCAGCGCGAACTGCCCCACTTGAGTGCGCGCAACGCCGGCGGAACTCGTCGTGTAGCGGTGAAATGCTTAGATATGACGAAGAACCCCGATTGCGAAGGCAGCTGGCGGGAGCGTAACTGACGCTGAAGCTCGAAAGCGCGGGTATCGAAC"
##  [47] "CCGGGCGTTATCCGGATTTATTGGGTTTAAAGGGAGCGTAGGCCGGAGATTAAGCGTGTTGTGAAATGTAGACGCTCAACGTCTGCACTGCAGCGCGAACTGGTTTCCTTGAGTACGCACAAAGTGGGCGGAATTCGTGGTGTAGCGGTGAAATGCTTAGATATCACGAAGAACTCCGATTGCGAAGGCAGCTTGCGGGAGTGCGACTGACGCTGAAGCTCGAAGGTGCGGGTATCGAAC"
##  [48] "CCGGGCGTTATCCGGATTTATTGGGTTTAAAGGGAGCGTAGGCCGGAGATTAAGCGTGTTGTGAAATGTAGACGCTCAACGTCTGCACTGCAGCGCGAACTGGTTTCCTTGAGTACGCACAAAGTGGGCGGAATTCGTGGTGTAGCGGTGAAATGCTTAGATATCACGAAGAACTCCGATTGCGAAGGCAGCTCACGGGAGCGCAACTGACGCTGAAGCTCGAAAGTGCGGGTATCGAAC"
##  [49] "CCGGGCGTTATCCGGATTTATTGGGTTTAAAGGGAGCGTAGGCCGGAGATTAAGCGTGTTGTGAAATGTAGACGCTCAACGTCTGCACTGCAGCGCGAACTGGTTTCCTTGAGTACGCACAAAGTGGGCGGAATTCGTGGTGTAGCGGTGAAATGCTTAGATATCACGAAGAACTCCGATTGCGAAGGCAGCTGACGGTAGCGCAACTGACGCTGAGGCTCGAAAGCGCGGGTATCGAAC"
##  [50] "CCGGGCGTTATCCGGATTTATTGGGTTTAAAGGGAGCGTAGGCCGGAGATTAAGCGTGTTGTGAAATGTAGACGCTCAACGTCTGCACTGCAGCGCGAACTGGTTTCCTTGAGTACGCATAAAGTGGGCGGAATTCGTGGTGTAGCGGTGAAATGCTTAGATATCACGAAGAACTCCGATTGCGAAGGCAGCTCACTGGAGCGCAACTGACGCTGAAGCTCGAAAGTGCGGGTATCGAAC"
##  [51] "CCGGGCGTTATCCGGATTTATTGGGTTTAAAGGGAGCGTAGGCCGGAGATTAAGCGTGTTGTGAAATGTAGATGCTCAACATCTGCACTGCAGCGCGAACTGGTTTCCTTGAGTACGCATAAAGTGGGCGGAATTCGTGGTGTAGCGGTGAAATGCTTAGATATCACGAAGAACTCCGATTGCGAAGGCAGCTCACTGGGGCGCAACTGACGCTGAAGCTCGAAAGCGCGGGTATCGAAC"
##  [52] "CCGGGCGTTATCCGGATTTATTGGGTTTAAAGGGAGCGTAGGCCGGAGATTAAGCGTGTTGTGAAATGTAGATGCTCAACATCTGAACTGCAGCGCGAACTGGTTTCCTTGAGTACGCATAAAGTGGGCGGAATTCGTGGTGTAGCGGTGAAATGCTTAGATATCACGAAGAACTCCGATTGCGAAGGCAGCTCACTGGAGCGCAACTGACGCTGAAGCTCGAAAGTGCGGGTATCGAAC"
##  [53] "CCGGGCGTTATCCGGATTTATTGGGTTTAAAGGGAGCGTAGGCCGGAGATTAAGCGTGTTGTGAAATGTAGATGCTCAACATCTGCACTGCAGCGCGAACTGGTTTCCTTGAGTACGCATAAAGTGGGCGGAATTCGTGGTGTAGCGGTGAAATGCTTAGATATCACGAAGAACTCCGATTGCGAAGGCAGCTCACTGGAGCGCAACTGACGCTGAAGCTCGAAAGTGCGGGTATCGAAC"
##  [54] "CCGGGCGTTATCCGGATTTATTGGGTTTAAAGGGAGCGTAGGCCGGAGATTAAGCGTGTTGTGAAATGTAGTTGCTCAACATCTGCACTGCAGCGCGAACTGGTTTCCTTGAGTACGCACAAAGTGGGCGGAATTCGTGGTGTAGCGGTGAAATGCTTAGATATCACGAAGAACTCCGATTGCGAAGGCAGCTCACTGGAGCGCAACTGACGCTGAAGCTCGAAAGTGCGGGTATCGAAC"
##  [55] "CCGGGCGTTATCCGGATTTATTGGGTTTAAAGGGAGCGTAGGCCGCAGGTTAAGCGTGTTGTGAAATGTAGGGGCTCAACCTCTGCACTGCAGCGCGAACTGGCTTGCTTGAGTACGCACAACGTGGGCGGAATTCGTGGTGTAGCGGTGAAATGCTTAGATATCACGAAGAACTCCGATTGCGAAGGCAGCTCACGGGAGCGCAACTGACGCTGAAGCTCGAAAGTGCGGGTATCGAAC"
##  [56] "CCGGGCGTTATCCGGATTTATTGGGTTTAAAGGGAGCGTAGGCCGCAGGTTAAGCGTGTTGTGAAATGTAGGGGCTCAACCTCTGCACTGCAGCGCGAACTGGCTTGCTTGAGTACGCACAACGTGGGCGGAATTCGTGGTGTAGCGGTGAAATGCTTAGATATCACGAAGAACTCCGATTGCGAAGGCAGCTCACTGGAGCGCAACTGACGCTGAAGCTCGAAAGTGCGGGTATCGAAC"
##  [57] "CCAGGCGTTATCCGGATTTATTGGGTTTAAAGGGAGCGTAGGCCGCCCCTTAAGCGTGTTGTGAAATGCCGCGGCTCAACCGTGGCACTGCAGCGCGAACTGGGGGGCTTGAGTGCACGCAACGCAGGCGGAATTCGTGGTGTAGCGGTGAAATGCTTAGATATCACGAAGAACTCCGATTGCGAAGGCAGCTCACTGGAGCGCAACTGACGCTGAAGCTCGAAAGTGCGGGTATCGAAC"
##  [58] "CCGGGCGTTATCCGGATTTATTGGGTTTAAAGGGAGCGTAGGCCGCCCCTTAAGCGTGTTGTGAAATGCCGCGGCTCAACCGTGGCACTGCAGCGCGAACTGGGGGGCTTGAGTGCACGCAACGCAGGCGGAATTCGTGGTGTAGCGGTGAAATGCTTAGATATCACGAAGAACTCCGATTGCGAAGGCAGCTTGCGGGAGTGCGACTGACGCTGAAGCTCGAAGGTGCGGGTATCGAAC"
##  [59] "CCAGGCGTTATCCGGATTTATTGGGTTTAAAGGGAGCGTAGGCCGCCCCTTAAGCGTGTTGTGAAATGCCGCGGCTCAACCGTGGCACTGCAGCGCGAACTGGGGGGCTTGAGTGCACGCAACGCAGGCGGAATTCGTGGTGTAGCGGTGAAATGCTTAGATATCACGAAGAACTCCGATTGCGAAGGCAGCTTGCGGGAGTGCGACTGACGCTGAAGCTCGAAGGTGCGGGTATCGAAC"
##  [60] "CCGGGCGTTATCCGGATTTATTGGGTTTAAAGGGAGCGTAGGCCGCCCCTTAAGCGTGTTGTGAAATGCCGCGGCTCAACCGTGGCACTGCAGCGCGAACTGGGGGGCTTGAGTGCACGCAACGCAGGCGGAATTCGTGGTGTAGCGGTGAAATGCTTAGATATCACGAAGAACTCCGATTGCGAAGGCAGCTCACTGGAGCGCAACTGACGCTGAAGCTCGAAAGTGCGGGTATCGAAC"
##  [61] "CCGGGCGTTATCCGGATTTATTGGGTTTAAAGGGAGCGTAGGCCGTTTGGTAAGCGTGTTGTGAAATGTCGGGGCTCAACCTGGGCATTGCAGCGCGAACTGCCAGACTTGAGTGCGCAGGAAGTAGGCGGAATTCGTCGTGTAGCGGTGAAATGCTTAGATATGACGAAGAACTCCGATTGCGAAGGCAGCCTGCTGTAGCGCAACTGACGCTGAAGCTCGAAAGCGTGGGTATCGAAC"
##  [62] "CCAGGCGTTATCCGGATTTATTGGGTTTAAAGGGAGCGTAGGCCGTTTGGTAAGCGTGTTGTGAAATGTCGGGGCTCAACCTGGGCATTGCAGCGCGAACTGCCAGACTTGAGTGCGCAGGAAGTAGGCGGAATTCGTCGTGTAGCGGTGAAATGCTTAGATATGACGAAGAACTCCGATTGCGAAGGCAGCCTGCTGTAGCGCAACTGACGCTGAAGCTCGAAAGCGTGGGTATCGAAC"
##  [63] "CCGGGCGTTATCCGGATTTATTGGGTTTAAAGGGAGCGTAGGCCGTCTGTTAAGCGTGTTGTGAAATGTCGTGGCTCAACCGGGGCACTGCAGCGCGAACTGGCAGACTTGAGTGCACGGTAGGAAGGCGGAATTCGTCGTGTAGCGGTGAAATGCTTAGATATGACGAAGAACTCCGATTGCGAAGGCAGCTTTCCGTAGTGTAACTGACGCTGAAGCTCGAAAGCGTGGGTATCGAAC"
##  [64] "CCGGGCGTTATCCGGATTTATTGGGTTTAAAGGGAGCGTAGGCCGTCTGTTAAGCGTGTTGTGAAATGTCGGGGCTCAACCTGGGCATTGCAGCGCGAACTGGCAGACTTGAGTGCGCAGGAAGTAGGCGGAATTCGTCGTGTAGCGGTGAAATGCTTAGATATGACGAAGAACTCCGATTGCGAAGGCAGCCTGCTGTAGCGTAACTGACGCTGAAGCTCGAAAGCGTGGGTATCGAAC"
##  [65] "CCGGGCGTTATCCGGATTTATTGGGTTTAAAGGGAGCGTAGGCCGTCTGGTAAGCGTGTTGTGAAATGTCGGGGCTCAACCTGGGCATTGCAGCGCGAACTGTCAGACTTGAGTGCGCGGGAAGTAGGCGGAATTCGTCGTGTAGCGGTGAAATGCTTAGATATGACGAAGAACTCCGATTGCGAAGGCAGCCTGCTGTAGCGCAACTGACGCTGAAGCTCGAAAGCGTGGGTATCGAAC"
##  [66] "CCGGGCGTTATCCGGATTTATTGGGTTTAAAGGGAGCGTAGGCCGTCTTATAAGCGTGTTGTGAAATGTCGGGGCTCAACCTGGGCATTGCAGCGCGAACTGTGAGACTTGAGTGCGCAGGAAGTAGGCGGAATTCGTCGTGTAGCGGTGAAATGCTTAGATATGACGAAGAACTCCGATTGCGAAGGCAGCCTGCTGTAGCGCAACTGACGCTGAAGCTCGAAAGCGTGGGTATCGAAC"
##  [67] "CCGGGCGTTATCCGGATTTATTGGGTTTAAAGGGAGCGTAGGCCGTCTTATAAGCGTGTTGTGAAATGTCGGGGCTCAACCTGGGCATTGCAGCGCGAACTGTGAGACTTGAGTGCGCAGGAAGTAGGCGGAATTCGTCGTGTAGCGGTGAAATGCTTAGATATCACGAAGAACTCCGATTGCGAAGGCAGCTCACTGGAGCGCAACTGACGCTGAAGCTCGAAAGTGCGGGTATCGAAC"
##  [68] "CCAGGCGTTATCCGGATTTATTGGGTTTAAAGGGAGCGTAGGCCGTTTTTTAAGCGTGTTGTGAAATACTGTCGCTCAACGACAGAGGTGCAGCGCGAACTGGAGGACTTGAGTGCGCGGAATGTAGGCGGAATTCGTCGTGTAGCGGTGAAATGCTTAGATATGACGAAGAACTCCGATTGCGAAGGCAGCTTACAGTAGCGTAACTGACGCTGAAGCTCGAAAGTGCGGGTATCGAAC"
##  [69] "CCGGGCGTTATCCGGATTTATTGGGTTTAAAGGGAGCGTAGGCCGTCTTTTAAGCGTGTTGTGAAATACTGTCGCTCAACGACAGAGGTGCAGCGCGAACTGGGAGACTTGAGTGCGCGGAATGCAGGCGGAATTCGTCGTGTAGCGGTGAAATGCTTAGATATGACGAAGAACTCCGATTGCGAAGGCAGCTTGCAGTAGCGTAACTGACGCTGAAGCTCGAAAGTGCGGGTATCGAAC"
##  [70] "CCGGGCGTTATCCGGATTTATTGGGTTTAAAGGGAGCGCAGGCCGTGGGCTAAGCGTGCCGTGAAATTCTGTCGCTCAACGGCAGACGTGCGGCGCGAACTGGTCCACTTGAGTACGCGGGACGTTGGCGGAATTCGTGGTGTAGCGGTGAAATGCTTAGATATCACGAAGAACTCCGATTGCGAAGGCAGCTCACTGGAGCGCAACTGACGCTGAAGCTCGAAAGTGCGGGTATCGAAC"
##  [71] "CCGGGCGTTATCCGGATTTATTGGGTTTAAAGGGAGCGCAGGCCGTGGGTTAAGCGTGTCGTGAAATTCCGTCGCTCAACGGCGGACGTGCGGCGCGAACTGGTCCACTTGAGTACGCGGGACGTTGGCGGAATTCGTGGTGTAGCGGTGAAATGCTTAGATATCACGAAGAACTCCGATTGCGAAGGCAGCTGACGGTAGCGCAACTGACGCTGAGGCTCGAAAGTGCGGGTATCGAAC"
##  [72] "CCGGGCGTTATCCGGATTTATTGGGTTTAAAGGGAGCGCAGGCCGTGGGCTAAGCGTGCCGTGAAATTCTGTCGCTCAACGGCAGACGTGCGGCGCGAACTGGTCCACTTGAGTACGCGGGACGTTGGCGGAATTCGTGGTGTAGCGGTGAAATGCTTAGATATCACGAAGAACTCCGATTGCGAAGGCAGCTGACGGTAGCGCAACTGACGCTGAGGCTCGAAAGCGCGGGTATCGAAC"
##  [73] "CCGGGCGTTATCCGGATTTATTGGGTTTAAAGGGAGCGCAGGCCGTGGGTTAAGCGTGTCGTGAAATTCCGTCGCTCAACGGCGGACGTGCGGCGCGAACTGGTCCACTTGAGTACGCGGGACGTTGGCGGAATTCGTGGTGTAGCGGTGAAATGCTTAGATATCACGAAGAACTCCGATTGCGAAGGCAGCTCACTGGAGCGCAACTGACGCTGAAGCTCGAAAGTGCGGGTATCGAAC"
##  [74] "GCGAGCGTTATCCGGATTTATTGGGTTTAAAGGGTGCGTAGGTCGCCGATTAAGTCAGCGGTGAAATCCAGTGGCTCAACCATCGGACTGCCGTTGAAACTGGCCGGCTTGAGTATGATTGAGGCAGGCGGAATGCGTGGTGTAGCGGTGAAATGCATAGATATCACGCAGAACCCCGATTGCGAAGGCAGCTTGCCAAGCCATGACTGACACTGAAGCACGAAAGCGTGGGTATCAAAC"
##  [75] "GCGAGCGTTATCCGGATTTATTGGGTTTAAAGGGTGCGCAGGCGGCGCGCCAAGTCAGCGGTCAAAGTTCCGGGCTCAACCCGGTGTCGCCGTTGAAACTGGCGTGCTCGAGTGCGTGCGAGGAAGGCGGAATGCGTTGTGTAGCGGTGAAATGCATAGATATGACGCAGAACTCCGATTGCGAAGGCAGCTTTCCAGCGCGCTACTGACGCTGAGGCACGAAAGCGTGGGGATCGAACA"
##  [76] "CCAAGCGTTATCCGGATTTATTGGGCGTAAAGCGAGCGCAGACGGTTATTTAAGTCTGAAGTGAAAGCCCTCAGCTCAACTGAGGAATTGCTTTGGAAACTGGATGACTTGAGTGCAGTAGAGGAAAGTGGAACTCCATGTGTAGCGGTGAAATGCGTAGATATATGGAAGAACACCAGTGGCGAAGGCGGCTTTCTGGACTGTAACTGACGTTGAGGCTCGAAAGTGTGGGTAGCAAAC"
##  [77] "GCGAGCGTTGTCCGGAATTATTGGGCGTAAAGAGCATGTAGGCGGTTTTTTAAGTCTGGAGTGAAAATGCGGGGCTCAACCCCGTATGGCTCTGGATACTGGAAGACTTGAGTGCAGGAGAGGAAAGGGGAATTCCCAGTGTAGCGGTGAAATGCGTAGATATTGGGAGGAACACCAGTGGCGAAGGCGCCTTTCTGGACTGTGTCTGACGCTGAGATGCGAAAGCCAGGGTAGCGAACG"
##  [78] "GCAAGCGTTGTCCGGAATTATTGGGCGTAAAGCGCGCGCAGGCGGCTTCTTAAGTCCATCTTAAAAGTGCGGGGCTTAACCCCGTGATGGGATGGAAACTGAGAGGCTGGAGTATCGGAGAGGAAAGTGGAATTCCTAGTGTAGCGGTGAAATGCGTAGAGATTAGGAAGAACACCGGTGGCGAAGGCGACTTTCTGGACGACAACTGACGCTGAGGCGCGAAAGCGTGGGGAGCAAACA"
##  [79] "GCGAGCGTTATCCGGATTCATTGGGCGTAAAGCGCGCGTAGGCGGCCCGTCAAGCGGGGTTTCAAATCCAGGGGCTCAACCTCTGGCCGGACCCCGAACTGGCGGGCTCGAGTGCGGTAGAGGAAGGTGGAATTCCCAGTGTAGCGGTGAAATGCGCAGATATTGGGAAGAACACCGATGGCGAAGGCAGCCTTCTGGGCCGCCACTGACGCTGAGGCGCGAAAGCTAGGGGAGCGAACA"
##  [80] "GCGAGCGTTATCCGGATTCATTGGGCGTAAAGCGCGCGTAGGCGGCCCGGCAGGCCGGGGGTCGAAGCGGGGGGCTCAACCCCCCGAAGCCCCCGGAACCTCCGCGGCTTGGGTCCGGTAGGGGAGGGTGGAACACCCGGTGTAGCGGTGGAATGCGCAGATATCGGGTGGAACACCGGTGGCGAAGGCGGCCCTCTGGGCCGAGACCGACGCTGAGGCGCGAAAGCTGGGGGAGCGAAC"
##  [81] "GCGAGCGTTATCCGGAATCATTGGGCGTAAAGCGCGCGCAGGCGGGCTTTCAAGCGGCGGCGTCGAAGCCGGGGGCTCAACCCCCGGAAGCGCCCCGAACTGGAAGCCTCGGATGCGGCAGGGGGAGGCGGAATTCCCGGTGTAGCGGTGAAATGCGCAGATATCGGGAAGAACACCGACGGCGAAGGCAGCCTCCTGGGCCGGCATCGACGCTGAGGCGCGAAAGCTGGGGGAGCGAAC"
##  [82] "GCGAGCGTTATCCGGAATGATTGGGCGTAAAGCGCGCGCAGGCGGCCGCTCAAGCGGGACCTCTAACCCCGGGGCTCAACCCCGGGCCGGGTCCCGAACTGGGCGGCTCGAGTGCGGTAGGGGAGAGCGGAATTCCAAGTGTAGCGGTGAAATGCGCAGATATTTGGAAGAACACCGATGGCGAAGGCAGCTCTCTGGGCCGTCACTGACGCTGAGGCGCGAAAGCCGGGGGAGCGAACA"
##  [83] "GCGAGCGTTATCCGGATTCATTGGGCGTAAAGCGCGCGTAGGCGGAGCGCTAAGCGGGACCTCTAACCCGAGGGCTCAACCCCCGGCCGGGTCCCGAACTGGCGCTCTCGAGTGCGGTAGGGGAGAGCGGAATTCCCGGTGTAGCGGTGGAATGCGCAGATATCGGGAAGAACACCGACGGCGAAGGCAGCTCTCTGGGCCGAAACTGACGCTGAGGCGCGAAAGCTGGGGGAGCGAACA"
##  [84] "GCGAGCGTTATCCGGATTCATTGGGCGTAAAGCGCGCGTAGGCGGAGCGCTAAGCGGGACCTCTAACCCGAGGGCTCAACCCCCGGCCGGGTCCCGAACTGGCGCTCTCGAGTGCGGTAGGGGAGAGCGGAATTCCCGGTGTAGCGGTGGAATGCGCAGATATCGGGAGGAACACCGACGGCGAAGGCAGCTCTCTGGGCCGAAACTGACGCTGAGGCGCGAAAGCTGGGGGAGCGAACA"
##  [85] "GCGAGCGTTATCCGGATTCATTGGGCGTAAAGCGCGCGTAGGCGGATGCCTAAGCGGGACCTCTAACCCGGGGGCTCAACCCCCGGCCGGGTCCCGAACTGGGCGTCTCGAGTGCGGTAGGGGCAGGTGGAATTCCATGTGTAGCGGTGGAATGCGCAGATATATGGAAGAACACCGACGGCGAAGGCAGCCTGCTGGGCCGACACTGACGCTGAGGTGCGAAAGCGCGGGGAGCGAACA"
##  [86] "GCGAGCGTTATCCGGATTCATTGGGCGTAAAGCGCGCGTAGGCGGCCGCTCGAGCGGGACCTCTAACCCGGGGGCTCAACCTCCGGCCGGGTCCCGGACCGTGCGGCTCGGGTGCGGTAGGGGCAGGCGGAACTCCAAGTGTAGCGGTGAAATGCGCAGATATTTGGAGGAACACCGATGGCGAAGGCAGCCTGCTGGGCCGCCACCGACGCTGAGGCGCGAAAGCCGGGGGAGCGAACA"
##  [87] "GCTAGCGTTATCCGGAATTACTGGGCGTAAAGGGTGCGTAGGTGGTTTCTTAAGTCAGAGGTGAAAGGCTACGGCTCAACCGTAGTAAGCCTTTGAAACTGGGAAACTTGAGTGCAGGAGAGGAGAGTGGAATTCCTAGTGTAGCGGTGAAATGCGTAGATATTAGGAGGAACACCAGTTGCGAAGGCGGCTCTCTGGACTGTAACTGACACTGAGGCACGAAAGCGTGGGGAGCAAACA"
##  [88] "GCTAGCGTTATCCGGATTTACTGGGCGTAAAGGGTGCGTAGGCGGTCTTTTAAGTCAGGAGTGAAAGGCTACGGCTCAACCGTAGTAAGCTCTTGAAACTGGAGGACTTGAGTGCAGGAGAGGAGAGTGGAATTCCTAGTGTAGCGGTGAAATGCGTAGATATTAGGAGGAACACCAGTAGCGAAGGCGGCTCTCTGGACTGTAACTGACGCTGAGGCACGAAAGCGTGGGGAGCAAACA"
##  [89] "GCAAGCGTTATCCGGAATTATTGGGCGTAAAGAGTACGTAGGTGGTTTTCTAAGCACGGGGTTTAAGGCAATGGCTTAACCATTGTTCGCCTTGTGAACTGGAAGACTTGAGTGCAGGAGAGGAAAGCGGAATTCCTAGTGTAGCGGTGAAATGCGTAGATATTAGGAGGAACACCAGTGGCGAAGGCGGCTTTCTGGACTGTAACTGACACTGAGGTACGAAAGCGTGGGGAGCAAACA"
##  [90] "GCAAGCGTTATCCGGAATTATTGGGCGTAAAGAGTACGTAGGTGGTTACCTAAGCACGAGGTATAAGGCAATGGCTTAACCATTGTTCGCCTTGTGAACTGGGCTACTTGAGTGCAGGAGAGGAAAGCGGAATTCCTAGTGTAGCGGTGAAATGCGTAGATATTAGGAGGAACACCAGTGGCGAAGGCGGCTTTCTGGACTGTAACTGACACTGAGGTACGAAAGCGTGGGGAGCAAACA"
##  [91] "GCAAGCGTTACTCGGAATTACTAGGCGTAAAGCGCGCGTAGGCGGAATGTTAAGTCTGTTGTGTAATCTCTGGGCTCAACCCAGAAACTGCAACAGAAACTGGCGTTCTTGAGTGAGGCAGAGGAAATCGGAATTCCTAGTGTAGCAGTGAAATGCGTAGATATTAGGAGGAACACCGGTGGCGAAGGCGGATTTCTGGGCCTTTACTGACGCTAAAGTGCGAAAGCTAGGGGAGCAAAC"
##  [92] "GCGAGCGTTGTCCGGAATGACTGGGCGTAAAGGGCGTGTAGGCGGCAGTATAAGTCCGGAGTGAAAGTCCTGCTTTCAAGGTGGGAATTGCTTTGGAGACTGTACAGCTTGAGTGCGGAAGAGGTAAGTGGAATTCCCAGTGTAGCGGTGAAATGCGTAGAGATTGGGAGGAACACCAGTGGCGAAGGCGACTTACTGGGCCGTAACTGACGCTGAGGCGCGAAAGCGTGGGGAGCGAAC"
##  [93] "GCAAGCGTTATCCGGATTTACTGGGCGTAAAGGGAGCGTAGGCGGATATTTAAGTGGGATGTGAAATACCCGAGCTTAACTTGGGAGCTGCATTCCAAACTGGATATCTAGAGTGCAGGAGAGGAGAATGGAATTCCTAGTGTAGCGGTGAAATGCGTAGAGATTAGGAAGAACACCAGTGGCGAAGGCGATTCTCTGGACTGTAACTGACGCTGAGGCTCGAAAGCGTGGGGAGCAAAC"
##  [94] "GCAAGCGTTATCCGGATTTACTGGGTGTAAAGGGAGCGTAGACGGTGTGGCAAGTCTGATGTGAAAGGCATGGGCTCAACCTGTGGACTGCATTGGAAACTGTCATACTTGAGTGCCGGAGGGGTAAGCGGAATTCCTAGTGTAGCGGTGAAATGCGTAGATATTAGGAGGAACACCAGTGGCGAAGGCGGCTTACTGGACGGTAACTGACGTTGAGGCTCGAAAGCGTGGGGAGCAAAC"
##  [95] "GCAAGCGTTATCCGGATTTACTGGGTGTAAAGGGAGCGTAGACGGCGCAGCAAGTCTGATGTGAAAGGCAGGGGCTTAACCCCTGGACTGCATTGGAAACTGCTGTGCTTGAGTGCCGGAGGGGTAAGCGGAATTCCTAGTGTAGCGGTGAAATGCGTAGATATTAGGAGGAACACCAGTGGCGAAGGCGGCTTACTGGACGGTAACTGACGTTGAGGCTCGAAAGCGTGGGGAGCAAAC"
##  [96] "GCAAGCGTTATCCGGATTTACTGGGTGTAAAGGGAGCGTAGACGGATTAGCAAGTCTGATGTGAAAGGCAGGGGCTCAACCCCTGGACTGCATTGGAAACTGCCAGTCTTGAGTGCCGGAGAGGTAAGCGGAATTCCTAGTGTAGCGGTGAAATGCGTAGATATTAGGAGGAACACCAGTGGCGAAGGCGGCTTACTGGACGGCAACTGACGTTGAGGCTCGAAAGCGTGGGGAGCAAAC"
##  [97] "GCAAGCGTTATCCGGATTTACTGGGTGTAAAGGGAGCGTAGACGGCTTTGCAAGTCTGATGTGAAAGGCGGGGGCTCAACCCCTGGACTGCATTGGAAACTGTGAGGCTTGAGTGCCGGAGAGGTAAGCGGAATTCCTAGTGTAGCGGTGAAATGCGTAGATATTAGGAGGAACACCAGTGGCGAAGGCGGCTTACTGGACGGTAACTGACGTTGAGGCTCGAAAGCGTGGGGAGCAAAC"
##  [98] "GCAAGCGTTATCCGGATTTACTGGGTGTAAAGGGAGCGTAGGCGGTATGGCAAGTCTGATGTGAAAGGCCGGGGCTCAACCCCGGGACTGCATTGGAAACTGTCACACTTGAGTGTCGGAGAGGTAAGTGGAATTCCTAGTGTAGCGGTGAAATGCGTAGATATTAGGAGGAACACCAGTGGCGAAGGCGGCTTACTGGACGACAACTGACGCTGAGGCTCGAAAGCGTGGGGAGCAAAC"
##  [99] "GCAAGCGTTATCCGGATTTACTGGGTGTAAAGGGAGCGCAGGCGGTCTGGCAAGTCTGATGTGAAATCCCGGGGCTCAACCCTGGAACTGCATTGGAAACTGTCAGACTAGAGTGCCGGAGAGGTAAGTGGAATTCCTAGTGTAGCGGTGAAATGCGTAGATATTAGGAGGAACACCAGTGGCGAAGGCGGCTTACTGGACGGTAACTGACGCTGAGGCTCGAAAGCGTGGGGAGCAAAC"
## [100] "GCAAGCGTTATCCGGATTTACTGGGTGTAAAGGGAGCGTAGACGGTATGGCAAGTCTGATGTGAAAGGCCAGGGCTCAACCCTGGGACTGCATTGGAAACTGTCGAACTAGAGTGTCGGAGAGGCAAGTGGAATTCCTAGTGTAGCGGTGAAATGCGTAGATATTAGGAGGAACACCAGTGGCGAAGGCGGCTTGCTGGACGATGACTGACGTTGAGGCTCGAAAGCGTGGGGAGCAAAC"
## [101] "GCAAGCGTTATCCGGATTTACTGGGTGTAAAGGGAGCGTAGACGGAATGGCAAGTCTGATGTGAAAGGCCGGGGCTCAACCCCGGGACTGCATTGGAAACTGTCAATCTAGAGTACCGGAGGGGTAAGTGGAATTCCTAGTGTAGCGGTGAAATGCGTAGATATTAGGAGGAACACCAGTGGCGAAGGCGGCTTACTGGACGGTAACTGACGTTGAGGCTCGAAAGCGTGGGGAGCAAAC"
## [102] "GCAAGCGTTATCCGGATTTACTGGGTGTAAAGGGAGCGTAGACGGCACAGCAAGTCTGATGTGAAAGCCCGGGGCCCAACCCCGGAACTGCATTGGAAACTGCTGGGCTTGAGTGCAGGAGAGGTAAGCGGAATTCCTAGTGTAGCGGTGAAATGCGTAGATATTAGGAGGAACACCAGTGGCGAAGGCGGCTTACTGGACTGTAACTGACGTTGAGGCTCGAAAGCGTGGGGAGCAAAC"
## [103] "GCAAGCGTTATCCGGATTTACTGGGTGTAAAGGGAGCGTAGACGGCGAGACAAGTCTGAAGTGAAAGCCCGGGGCTCAACCCCGGGACTGCTTTGGAAACTGCCTTGCTAGAGTGCTGGAGAGGTAAGTGGAATTCCTAGTGTAGCGGTGAAATGCGTAGATATTAGGAGGAACACCAGTGGCGAAGGCGGCTTACTGGACAGTAACTGACGTTGAGGCTCGAAAGCGTGGGGAGCAAAC"
## [104] "GCAAGCGTTATCCGGATTTACTGGGTGTAAAGGGAGCGTAGACGGTCAAGCAAGTCAGAAGTGAAAGGCTGGGGCTCAACCCCGGGACTGCTTTTGAAACTGTTTGACTGGAGTGCTGGAGAGGTAAGCGGAATTCCTAGTGTAGCGGTGAAATGCGTAGATATTAGGAGGAACACCAGTGGCGAAGGCGGCTTACTGGACAGTAACTGACGTTGAGGCTCGAAAGCGTGGGGAGCAAAC"
## [105] "GCAAGCGTTATCCGGATTTACTGGGTGTAAAGGGAGCGTAGACGGCTAAGCAAGTCAGAAGTGAAAGGCTGGGGCTCAACCCCGGGACTGCTTTTGAAACTGTTTGGCTAGAGTGCTGGAGAGGTAAGCGGAATTCCTAGTGTAGCGGTGAAATGCGTAGATATTAGGAGGAACACCAGTGGCGAAGGCGGCTTACTGGACAGTAACTGACGTTGAGGCTCGAAAGCGTGGGGAGCAAAC"
## [106] "GCAAGCGTTATCCGGATTTACTGGGTGTAAAGGGAGCGTAGACGGTTAAGCAAGTCAGAAGTGAAAGGCTGGGGCTCAACCCCGGGACTGCTTTTGAAACTGTTTAACTAGAGTGCTGGAGAGGTAAGCGGAATTCCTAGTGTAGCGGTGAAATGCGTAGATATTAGGAGGAACACCAGTGGCGAAGGCGGCTTACTGGACAGTAACTGACGTTGAGGCTCGAAAGCGTGGGGAGCAAAC"
## [107] "GCAAGCGTTATCCGGATTTACTGGGTGTAAAGGGAGCGTAGACGGTAAAGCAAGTCTGAAGTGAAAGCCCGGGGCTCAACCGCGGGACTGCTTTGGAAACTGTTTAACTAGAGTGCTGGAGAGGTAAGCGGAATTCCTAGTGTAGCGGTGAAATGCGTAGATATTAGGAGGAACACCAGTGGCGAAGGCGGCTTACTGGACAGTAACTGACGTTGAGGCTCGAAAGCGTGGGGAGCAAAC"
## [108] "GCAAGCGTTATCCGGATTTACTGGGTGTAAAGGGAGCGTAGACGGCGATGCAAGTCTGAAGTGAAATACCCGGGCTCAACCTGGGAACTGCTTTGGAAACTGTATTGCTAGAGTGCTGGAGAGGTAAGCGGAATTCCTAGTGTAGCGGTGAAATGCGTAGATATTAGGAAGAACACCAGTGGCGAAGGCGGCTTACTGGACAGTAACTGACGTTGAGGCTCGAAAGCGTGGGGAGCAAAC"
## [109] "GCAAGCGTTATCCGGATTTACTGGGTGTAAAGGGAGCGTAGACGGCGACGCAAGTCTGAAGTGAAATACCCGGGCTCAACCTGGGAACTGCTTTGGAAACTGTGTTGCTAGAGTGCTGGAGAGGTAAGCGGAATTCCTAGTGTAGCGGTGAAATGCGTAGATATTAGGAAGAACACCAGTGGCGAAGGCGGCTTACTGGACAGTAACTGACGTTGAGGCTCGAAAGCGTGGGGAGCAAAC"
## [110] "GCAAGCGTTATCCGGATTTACTGGGTGTAAAGGGAGCGTAGACGGTAGTGCAAGTCTGATGTGAAAGCCCGGGGCTCAACCCCGGGACTGCATTGGAAACTGTATAACTAGAGTGTCGGAGAGGTAAGCGGAATTCCTAGTGTAGCGGTGAAATGCGTAGATATTAGGAGGAACACCAGTGGCGAAGGCGGCTTACTGGACGATGACTGACGTTGAGGCTCGAAAGCGTGGGGAGCAAAC"
## [111] "GCAAGCGTTATCCGGATTTACTGGGTGTAAAGGGAGCGTAGACGGCTGTGTAAGTCTGAAGTGAAAGCCCGGGGCTCAACCGCGGGACTGCTTTGGAAACTATGCAGCTAGAGTGTCGGAGAGGTAAGTGGAATTCCCAGTGTAGCGGTGAAATGCGTAGATATTGGGAGGAACACCAGTGGCGAAGGCGGCTTACTGGACGATGACTGACGTTGAGGCTCGAAAGCGTGGGGAGCAAAC"
## [112] "GCAAGCGTTATCCGGATTTACTGGGTGTAAAGGGAGCGTAGACGGCTGTGCAAGTCTGAAGTGAAAGGCATGGGCTCAACCTGTGGACTGCTTTGGAAACTGTGCAGCTAGAGTGTCGGAGAGGTAAGTGGAATTCCTAGTGTAGCGGTGAAATGCGTAGATATTAGGAGGAACACCAGTGGCGAAGGCGGCTTACTGGACGATGACTGACGTTGAGGCTCGAAAGCGTGGGGAGCAAAC"
## [113] "GCAAGCGTTATCCGGATTTACTGGGTGTAAAGGGAGCGTAGACGGCATGGCAAGTCTGATGTGAAAATCCCGGGCTCAACCCGGGAACTGCATTGGAAACTGTTAAGCTAGAGTGCAGGAGAGGTAAGTGGAATTCCTAGTGTAGCGGTGAAATGCGTAGATATTAGGAGGAACACCAGTGGCGAAGGCGGCTTACTGGACTGTAACTGACGTTGAGGCTCGAAAGCGTGGGGAGCAAAC"
## [114] "GCAAGCGTTATCCGGATTTACTGGGTGTAAAGGGAGCGTAGACGGCACGGCAAGCCAGATGTGAAAGCCCGGGGCTCAACCCCGGGACTGCATTTGGAACTGCTGAGCTAGAGTGTCGGAGAGGCAAGTGGAATTCCTAGTGTAGCGGTGAAATGCGTAGATATTAGGAGGAACACCAGTGGCGAAGGCGGCTTGCTGGACGATGACTGACGTTGAGGCTCGAAAGCGTGGGGAGCAAAC"
## [115] "GCAAGCGTTATCCGGAATTACTGGGTGTAAAGGGAGCGTAGGCGGCATGGTAAGTAAGATGTGAAAGCCCGAGGCTTAACCTCGAGGATTGCATTTTAAACTATCAAGCTAGAGTACAGGAGAGGAAAGCGGAATTCCTAGTGTAGCGGTGAAATGCGTAGATATTAGGAAGAACACCAGTGGCGAAGGCGGCTTTCTGGACTGAAACTGACGCTGAGGCTCGAAAGCGTGGGGAGCGAA"
## [116] "GCAAGCGTTATCCGGAATTACTGGGTGTAAAGGGTGCGTAGGTGGTATGGCAAGTCAGAAGTGAAAACCCAGGGCTTAACTCTGGGACTGCTTTTGAAACTGTCAGACTGGAGTGCAGGAGAGGTAAGCGGAATTCCTAGTGTAGCGGTGAAATGCGTAGATATTAGGAGGAACATCAGTGGCGAAGGCGGCTTACTGGACTGAAACTGACACTGAGGCACGAAAGCGTGGGGAGCAAAC"
## [117] "GCAAGCGTTATCCGGAATTACTGGGTGTAAAGGGTGCGTAGGTGGTATGGCAAGTCAGAAGTGAAAACCCAGGGCTTAACTCTGGGACTGCTTTTGAAACTGTCAGACTAGAGTGCAGGAGAGGTAAGCGGAATTCCTAGTGTAGCGGTGAAATGCGTAGATATTAGGAGGAACATCAGTGGCGAAGGCGGCTTACTGGACTGAAACTGACACTGAGGCACGAAAGCGTGGGGAGCAAAC"
## [118] "GCAAGCGTTATCCGGATTTACTGGGTGTAAAGGGAGTGTAGGTGGCCATGCAAGTCAGAAGTGAAAATCCGGGGCTCAACCCCGGAACTGCTTTTGAAACTGTAAGGCTGGAGTGCAGGAGGGGTGAGTGGAATTCCTAGTGTAGCGGTGAAATGCGTAGATATTAGGAGGAACACCAGTGGCGAAGGCGGCTCACTGGACTGTAACTGACACTGAGGCTCGAAAGCGTGGGGAGCAAAC"
## [119] "GCAAGCGTTATCCGGATTTACTGGGTGTAAAGGGAGTGTAGGTGGCCAGGCAAGTCAGAAGTGAAAGCCCGGGGCTCAACCCCGGGACTGCTTTTGAAACTGCAGGGCTAGAGTGCAGGAGGGGCAAGTGGAATTCCTAGTGTAGCGGTGAAATGCGTAGATATTAGGAGGAACACCAGTGGCGAAGGCGGCTTGCTGGACTGTAACTGACACTGAGGCTCGAAAGCGTGGGGAGCAAAC"
## 
## $xy
## [1] "GCAAGCGTTATCCGGATTTACTGGGTGTAAAGGGCGCGTAGGCGGGGATGCAAGTCAGATGTGAAATCTATGGGCTTAACCCATAAACTGCATTTGAAACTGTATCTCTTGAGTGCTGGAGAGGTAGACGGAATTCCTTGTGTAGCGGTGAAATGCGTAGATATAAGGAAGAACACCAGTGGCGAAGGCGGTCTACTGGACAGTAACTGACGCTGAGGCGCGAGAGCGTGGGGAGCAAAC"
## [2] "GCAAGCGTTATCCGGATTTACTGGGTGTAAAGGGAGCGTAGACGGCCGTGCAAGTCTGATGTGAAAGGCTGGGGCTCAACCCCGGGACTGCATTGGAAACTGTATGGCTGGAGTGCCGGAGAGGTAAGCGGAATTCCTAGTGTAGCGGTGAAATGCGTAGATATTAGGAGGAACACCAGTGGCGAAGGCGGCTTACTGGACGGTAACTGACGTTGAGGCTCGAAAGCGTGGGGAGCAAAC"
## [3] "GCAAGCGTTATCCGGATTTACTGGGTGTAAAGGGAGCGCAGACGGCACTGCAAGTCTGAAGTGAAAGCCCGGGGCTCAACCCCGGGACTGCTTTGGAAACTGTAGAGCTAGAGTGCTGGAGAGGCAAGCGGAATTCCTAGTGTAGCGGTGAAATGCGTAGATATTAGGAGGAACACCAGTGGCGAAGGCGGCTTACTGGACGGTAACTGACGTTGAGGCTCGAAAGCGTGGGGAGCAAAC"
## 
## $xz
## character(0)
## 
## $yz
## [1] "ACAAGCGTTGTCCGGAATTACTGGGTGTAAAGGGAGCGCAGGCGGGCGATCAAGTTGGAAGTGAAATCCATGGGCTCAACCCATGAACTGCTTTCAAAACTGGTCGTCTTGAGTAGTGCAGAGGTAGGCGGAATTCCCGGTGTAGCGGTGGAATGCGTAGATATCGGGAGGAACACCAGTGGCGAAGGCGGCCTACTGGGCACCAACTGACGCTGAGGCTCGAAAGTGTGGGTAGCAAAC"
## [2] "GCAAGCGTTGTCCGGAATTACTGGGTGTAAAGGGAGCGCAGGCGGAAGGACAAGTTGGAAGTGAAACCCACGGGCTCAACCCGTGAACTGCTTTCAAAACTGTTTTTCTTGAGTGGTGTAGAGGTAGGCGGAATTCCCGGTGTAGCGGTGGAATGCGTAGATATCGGGAGGAACACCAGTGGCGAAGGCGGCCTACTGGGCACTAACTGACGCTGAGGCTCGAAAGCATGGGTAGCAAAC"
## [3] "ACAAGCGTTGTCCGGAATTACTGGGTGTAAAGGGAGCGCAGGCGGGAAGACAAGTTGGAAGTGAAATCCATGGGCTCAACCCATGAACTGCTTTCAAAACTGTTTTTCTTGAGTAGTGCAGAGGTAGGCGGAATTCCCGGTGTAGCGGTGGAATGCGTAGATATCGGGAGGAACACCAGTGGCGAAGGCGGCCTACTGGGCACCAACTGACGCTGAGGCTCGAAAGTGTGGGTAGCAAAC"
## [4] "GCAAGCGTTGTCCGGATTTACTGGGTGTAAAGGGCGTGTAGGCGGAGATGCAAGTCGGGAGTGAAATCCATGGGCTCAACCCATGAACTGCTTTCGAAACTGTATCCCTTGAGTATCGGAGAGGCAAGCGGAATTCCTAGTGTAGCGGTGAAATGCGTAGATATTAGGAGGAACACCAGTGGCGAAGGCGGCTTGCTGGACGACAACTGACGCTGAGGCGCGAAAGCGTGGGGAGCAAAC"
## [5] "GCGAGCGTTGTCCGGATTTACTGGGTGTAAAGGGCGTGTAGGCGGAGATGCAAGTTGGGAGTGAAATCCATGGGCTCAACCCATGAACTGCTTCCAAAACTGTATCCCTTGAGTATCGGAGAGGCAAGCGGAATTCCTAGTGTAGCGGTGAAATGCGTAGATATTAGGAGGAACACCAGTGGCGAAGGCGGCTTGCTGGACGACAACTGACGCTGAGGCGCGAAAGCGTGGGGAGCAAAC"
## [6] "GCAAGCGTTATCCGGATTTACTGGGTGTAAAGGGCGTGTAGGCGGGAAAGCAAGTCAGATGTGAAAACTGTGGGCTCAACCCACAGCCTGCATTTGAAACTGTTTTTCTTGAGTACTGGAGAGGCAGATGGAATTCCTAGTGTAGCGGTGAAATGCGTAGATATTAGGAGGAACACCAGTGGCGAAGGCGATCTGCTGGACAGCAACTGACGCTGAGGCGCGAAAGCGTGGGGAGCAAAC"
## [7] "GCAAGCGTTAATCGGAATAACTGGGCGTAAAGGGCATGCAGGCGGTTCATCAAGTAGGATGTGAAATCCCCGGGCTCAACCTGGGAACAGCATACTAAACTGGTGGACTAGAGTATTGCAGGGGGAGACGGAATTCCAGGTGTAGCGGTGGAATGCGTAGATATCTGGAAGAACACCAAAGGCGAAGGCAGTCTCCTGGGCAAATACTGACGCTCATATGCGAAAGCGTGGGTAGCAAAC"
## [8] "GCGAGCGTTATCCGGATTTATTGGGTTTAAAGGGAGCGCAGACGGGACTTTAAGTCAGCTGTGAAATTTTCCGGCTCAACCGGGAAACTGCAGTTGATACTGGCGTCCTTGAGTACGGTCGAGGCAGGCGGAATTCGTGGTGTAGCGGTGAAATGCTTAGATATCACGAAGAACCCCGATTGCGAAGGCAGCCTGCCAGACCGCAACTGACGTTCATGCTCGAAAGTGCGGGTATCAAAC"
## [9] "GCGAGCGTTATCCGGATTTATTGGGTTTAAAGGGAGCGTAGGCGGGCTGTTAAGTCAGCGGTCAAATGTCAGGGCCCAACCTTGGCATGCCGTTGATACTGGCGGCCTTGAGTTCACACAAGGAAGGTGGAATTCGTCGTGTAGCGGTGAAATGCTTAGATATGACGAAGAACTCCGATTGCGAAGGCAGCCTTCTGGGGTGTTACTGACGCTGAGGCTCGAAAGTGCGGGAATCAAACA"
## 
## $xy_only
## [1] "GCAAGCGTTATCCGGATTTACTGGGTGTAAAGGGCGCGTAGGCGGGGATGCAAGTCAGATGTGAAATCTATGGGCTTAACCCATAAACTGCATTTGAAACTGTATCTCTTGAGTGCTGGAGAGGTAGACGGAATTCCTTGTGTAGCGGTGAAATGCGTAGATATAAGGAAGAACACCAGTGGCGAAGGCGGTCTACTGGACAGTAACTGACGCTGAGGCGCGAGAGCGTGGGGAGCAAAC"
## [2] "GCAAGCGTTATCCGGATTTACTGGGTGTAAAGGGAGCGTAGACGGCCGTGCAAGTCTGATGTGAAAGGCTGGGGCTCAACCCCGGGACTGCATTGGAAACTGTATGGCTGGAGTGCCGGAGAGGTAAGCGGAATTCCTAGTGTAGCGGTGAAATGCGTAGATATTAGGAGGAACACCAGTGGCGAAGGCGGCTTACTGGACGGTAACTGACGTTGAGGCTCGAAAGCGTGGGGAGCAAAC"
## [3] "GCAAGCGTTATCCGGATTTACTGGGTGTAAAGGGAGCGCAGACGGCACTGCAAGTCTGAAGTGAAAGCCCGGGGCTCAACCCCGGGACTGCTTTGGAAACTGTAGAGCTAGAGTGCTGGAGAGGCAAGCGGAATTCCTAGTGTAGCGGTGAAATGCGTAGATATTAGGAGGAACACCAGTGGCGAAGGCGGCTTACTGGACGGTAACTGACGTTGAGGCTCGAAAGCGTGGGGAGCAAAC"
## 
## $xz_only
## character(0)
## 
## $yz_only
## [1] "ACAAGCGTTGTCCGGAATTACTGGGTGTAAAGGGAGCGCAGGCGGGCGATCAAGTTGGAAGTGAAATCCATGGGCTCAACCCATGAACTGCTTTCAAAACTGGTCGTCTTGAGTAGTGCAGAGGTAGGCGGAATTCCCGGTGTAGCGGTGGAATGCGTAGATATCGGGAGGAACACCAGTGGCGAAGGCGGCCTACTGGGCACCAACTGACGCTGAGGCTCGAAAGTGTGGGTAGCAAAC"
## [2] "GCAAGCGTTGTCCGGAATTACTGGGTGTAAAGGGAGCGCAGGCGGAAGGACAAGTTGGAAGTGAAACCCACGGGCTCAACCCGTGAACTGCTTTCAAAACTGTTTTTCTTGAGTGGTGTAGAGGTAGGCGGAATTCCCGGTGTAGCGGTGGAATGCGTAGATATCGGGAGGAACACCAGTGGCGAAGGCGGCCTACTGGGCACTAACTGACGCTGAGGCTCGAAAGCATGGGTAGCAAAC"
## [3] "ACAAGCGTTGTCCGGAATTACTGGGTGTAAAGGGAGCGCAGGCGGGAAGACAAGTTGGAAGTGAAATCCATGGGCTCAACCCATGAACTGCTTTCAAAACTGTTTTTCTTGAGTAGTGCAGAGGTAGGCGGAATTCCCGGTGTAGCGGTGGAATGCGTAGATATCGGGAGGAACACCAGTGGCGAAGGCGGCCTACTGGGCACCAACTGACGCTGAGGCTCGAAAGTGTGGGTAGCAAAC"
## [4] "GCAAGCGTTGTCCGGATTTACTGGGTGTAAAGGGCGTGTAGGCGGAGATGCAAGTCGGGAGTGAAATCCATGGGCTCAACCCATGAACTGCTTTCGAAACTGTATCCCTTGAGTATCGGAGAGGCAAGCGGAATTCCTAGTGTAGCGGTGAAATGCGTAGATATTAGGAGGAACACCAGTGGCGAAGGCGGCTTGCTGGACGACAACTGACGCTGAGGCGCGAAAGCGTGGGGAGCAAAC"
## [5] "GCGAGCGTTGTCCGGATTTACTGGGTGTAAAGGGCGTGTAGGCGGAGATGCAAGTTGGGAGTGAAATCCATGGGCTCAACCCATGAACTGCTTCCAAAACTGTATCCCTTGAGTATCGGAGAGGCAAGCGGAATTCCTAGTGTAGCGGTGAAATGCGTAGATATTAGGAGGAACACCAGTGGCGAAGGCGGCTTGCTGGACGACAACTGACGCTGAGGCGCGAAAGCGTGGGGAGCAAAC"
## [6] "GCAAGCGTTATCCGGATTTACTGGGTGTAAAGGGCGTGTAGGCGGGAAAGCAAGTCAGATGTGAAAACTGTGGGCTCAACCCACAGCCTGCATTTGAAACTGTTTTTCTTGAGTACTGGAGAGGCAGATGGAATTCCTAGTGTAGCGGTGAAATGCGTAGATATTAGGAGGAACACCAGTGGCGAAGGCGATCTGCTGGACAGCAACTGACGCTGAGGCGCGAAAGCGTGGGGAGCAAAC"
## [7] "GCAAGCGTTAATCGGAATAACTGGGCGTAAAGGGCATGCAGGCGGTTCATCAAGTAGGATGTGAAATCCCCGGGCTCAACCTGGGAACAGCATACTAAACTGGTGGACTAGAGTATTGCAGGGGGAGACGGAATTCCAGGTGTAGCGGTGGAATGCGTAGATATCTGGAAGAACACCAAAGGCGAAGGCAGTCTCCTGGGCAAATACTGACGCTCATATGCGAAAGCGTGGGTAGCAAAC"
## [8] "GCGAGCGTTATCCGGATTTATTGGGTTTAAAGGGAGCGCAGACGGGACTTTAAGTCAGCTGTGAAATTTTCCGGCTCAACCGGGAAACTGCAGTTGATACTGGCGTCCTTGAGTACGGTCGAGGCAGGCGGAATTCGTGGTGTAGCGGTGAAATGCTTAGATATCACGAAGAACCCCGATTGCGAAGGCAGCCTGCCAGACCGCAACTGACGTTCATGCTCGAAAGTGCGGGTATCAAAC"
## [9] "GCGAGCGTTATCCGGATTTATTGGGTTTAAAGGGAGCGTAGGCGGGCTGTTAAGTCAGCGGTCAAATGTCAGGGCCCAACCTTGGCATGCCGTTGATACTGGCGGCCTTGAGTTCACACAAGGAAGGTGGAATTCGTCGTGTAGCGGTGAAATGCTTAGATATGACGAAGAACTCCGATTGCGAAGGCAGCCTTCTGGGGTGTTACTGACGCTGAGGCTCGAAAGTGCGGGAATCAAACA"
## 
## $xyz
## character(0)
```
