# Supplementary material for: HIV-associated gut microbial alterations are dependent on host and geographic context
Source: Nat Commun. 2024 Feb 5;15:1055. doi: 10.1038/s41467-023-44566-4 (PMC10844288; doi:10.1038/s41467-023-44566-4)
Supplement: Supplementary file 12 — SupplementaryFigure1_Rocafort-Gootenberg_2022_03_20 [file 41467_2023_44566_MOESM12_ESM.html]

Rocafort-Gootenberg\_SupplementaryFigure1


# Rocafort-Gootenberg\_SupplementaryFigure1

#Load needed R packages

```
library("phyloseq")
library("tidyverse")
```

```
## ── Attaching packages ─────────────────────────────────────── tidyverse 1.3.2 ──
## ✔ ggplot2 3.4.1     ✔ purrr   1.0.1
## ✔ tibble  3.1.8     ✔ dplyr   1.1.0
## ✔ tidyr   1.3.0     ✔ stringr 1.5.0
## ✔ readr   2.1.4     ✔ forcats 1.0.0
## ── Conflicts ────────────────────────────────────────── tidyverse_conflicts() ──
## ✖ dplyr::filter() masks stats::filter()
## ✖ dplyr::lag()    masks stats::lag()
```

```
library("stringr")
library("ggplot2")
library("gridExtra")
```

```
## 
## Attaching package: 'gridExtra'
## 
## The following object is masked from 'package:dplyr':
## 
##     combine
```

```
library("dplyr")
library("vegan")
```

```
## Loading required package: permute
## Loading required package: lattice
## This is vegan 2.6-4
```

```
library("agricolae")
```

```
## Registered S3 methods overwritten by 'klaR':
##   method      from 
##   predict.rda vegan
##   print.rda   vegan
##   plot.rda    vegan
```

```
library("knitr") 
library("BiodiversityR")
```

```
## Loading required package: tcltk
## BiodiversityR 2.15-1: Use command BiodiversityRGUI() to launch the Graphical User Interface; 
## to see changes use BiodiversityRGUI(changeLog=TRUE, backward.compatibility.messages=TRUE)
```

```
library("reshape")
```

```
## 
## Attaching package: 'reshape'
## 
## The following object is masked from 'package:dplyr':
## 
##     rename
## 
## The following objects are masked from 'package:tidyr':
## 
##     expand, smiths
```

```
library("usedist")
library("rms")
```

```
## Loading required package: Hmisc
## Loading required package: survival
## Loading required package: Formula
## 
## Attaching package: 'Hmisc'
## 
## The following objects are masked from 'package:dplyr':
## 
##     src, summarize
## 
## The following objects are masked from 'package:base':
## 
##     format.pval, units
## 
## 
## Attaching package: 'rms'
## 
## The following object is masked from 'package:vegan':
## 
##     calibrate
```

```
library("DESeq2")
```

```
## Loading required package: S4Vectors
## Loading required package: stats4
## Loading required package: BiocGenerics
## 
## Attaching package: 'BiocGenerics'
## 
## The following object is masked from 'package:gridExtra':
## 
##     combine
## 
## The following objects are masked from 'package:dplyr':
## 
##     combine, intersect, setdiff, union
## 
## The following objects are masked from 'package:stats':
## 
##     IQR, mad, sd, var, xtabs
## 
## The following objects are masked from 'package:base':
## 
##     anyDuplicated, aperm, append, as.data.frame, basename, cbind,
##     colnames, dirname, do.call, duplicated, eval, evalq, Filter, Find,
##     get, grep, grepl, intersect, is.unsorted, lapply, Map, mapply,
##     match, mget, order, paste, pmax, pmax.int, pmin, pmin.int,
##     Position, rank, rbind, Reduce, rownames, sapply, setdiff, sort,
##     table, tapply, union, unique, unsplit, which.max, which.min
## 
## 
## Attaching package: 'S4Vectors'
## 
## The following objects are masked from 'package:reshape':
## 
##     expand, rename
## 
## The following objects are masked from 'package:dplyr':
## 
##     first, rename
## 
## The following object is masked from 'package:tidyr':
## 
##     expand
## 
## The following objects are masked from 'package:base':
## 
##     expand.grid, I, unname
## 
## Loading required package: IRanges
## 
## Attaching package: 'IRanges'
## 
## The following objects are masked from 'package:dplyr':
## 
##     collapse, desc, slice
## 
## The following object is masked from 'package:purrr':
## 
##     reduce
## 
## The following object is masked from 'package:phyloseq':
## 
##     distance
## 
## Loading required package: GenomicRanges
## Loading required package: GenomeInfoDb
## Loading required package: SummarizedExperiment
## Loading required package: MatrixGenerics
## Loading required package: matrixStats
## 
## Attaching package: 'matrixStats'
## 
## The following object is masked from 'package:dplyr':
## 
##     count
## 
## 
## Attaching package: 'MatrixGenerics'
## 
## The following objects are masked from 'package:matrixStats':
## 
##     colAlls, colAnyNAs, colAnys, colAvgsPerRowSet, colCollapse,
##     colCounts, colCummaxs, colCummins, colCumprods, colCumsums,
##     colDiffs, colIQRDiffs, colIQRs, colLogSumExps, colMadDiffs,
##     colMads, colMaxs, colMeans2, colMedians, colMins, colOrderStats,
##     colProds, colQuantiles, colRanges, colRanks, colSdDiffs, colSds,
##     colSums2, colTabulates, colVarDiffs, colVars, colWeightedMads,
##     colWeightedMeans, colWeightedMedians, colWeightedSds,
##     colWeightedVars, rowAlls, rowAnyNAs, rowAnys, rowAvgsPerColSet,
##     rowCollapse, rowCounts, rowCummaxs, rowCummins, rowCumprods,
##     rowCumsums, rowDiffs, rowIQRDiffs, rowIQRs, rowLogSumExps,
##     rowMadDiffs, rowMads, rowMaxs, rowMeans2, rowMedians, rowMins,
##     rowOrderStats, rowProds, rowQuantiles, rowRanges, rowRanks,
##     rowSdDiffs, rowSds, rowSums2, rowTabulates, rowVarDiffs, rowVars,
##     rowWeightedMads, rowWeightedMeans, rowWeightedMedians,
##     rowWeightedSds, rowWeightedVars
## 
## Loading required package: Biobase
## Welcome to Bioconductor
## 
##     Vignettes contain introductory material; view with
##     'browseVignettes()'. To cite Bioconductor, see
##     'citation("Biobase")', and for packages 'citation("pkgname")'.
## 
## 
## Attaching package: 'Biobase'
## 
## The following object is masked from 'package:MatrixGenerics':
## 
##     rowMedians
## 
## The following objects are masked from 'package:matrixStats':
## 
##     anyMissing, rowMedians
## 
## The following object is masked from 'package:Hmisc':
## 
##     contents
## 
## The following object is masked from 'package:phyloseq':
## 
##     sampleNames
```

```
library("gplots")
```

```
## 
## Attaching package: 'gplots'
## 
## The following object is masked from 'package:IRanges':
## 
##     space
## 
## The following object is masked from 'package:S4Vectors':
## 
##     space
## 
## The following object is masked from 'package:stats':
## 
##     lowess
```

#Load original phyloseq oject output from DADA2 pipeline and put in
new metadata

```
ps_gg_fp_f_prevalence_filter_2019_05_26<-readRDS("ps_gg_fp_f_prevalence_filter_2019_05_26")
readr::read_csv(
  "Metadata_formatted_nat_comm_add_2021_10_24.csv",
  col_names = TRUE,
  col_types = NULL,
  col_select = NULL,
  id = NULL,
  locale = default_locale(),
  na = c("", "NA", "empty", "EMPTY"),
  quote = "\"",
  comment = "",
  trim_ws = TRUE,
  skip = 0,
  name_repair = "unique",
  num_threads = readr_threads(),
  progress = show_progress(),
  show_col_types = should_show_types(),
  skip_empty_rows = TRUE,
  lazy = TRUE
) -> new_metadata
```

```
## Rows: 597 Columns: 88
## ── Column specification ────────────────────────────────────────────────────────
## Delimiter: ","
## chr (26): X, SampleID, subject_id, Race, Ethnicity, unique_id, sequencing_da...
## dbl (62): primer_used, read_count, age, height_cm, height_in, weight_kg, wei...
## 
## ℹ Use `spec()` to retrieve the full column specification for this data.
## ℹ Specify the column types or set `show_col_types = FALSE` to quiet this message.
```

```
### add {SampleID} as rownames
new_metadata_as_sample_data <- phyloseq::sample_data(new_metadata)
phyloseq::sample_names(new_metadata_as_sample_data) <- dplyr::pull(new_metadata, 1)
phyloseq::sample_data(ps_gg_fp_f_prevalence_filter_2019_05_26) <- new_metadata_as_sample_data

#Fix randomness
set.seed(1)
```

#Figure 1

```
#FIGURE A (1A)
#--------------------------------------------------------------------------------------------------------------
#Transform count data in the phyloseq object
ps_gg_fp_f_prevalence_filter_2019_05_26_proportion<-phyloseq::transform_sample_counts(ps_gg_fp_f_prevalence_filter_2019_05_26, function(x)(x/sum(x)))

#Select samples of interest and update phyloseq object
metadata<-as.data.frame(phyloseq::sample_data(ps_gg_fp_f_prevalence_filter_2019_05_26_proportion))
metadata<-metadata[metadata$hiv_phenotype=="1_hiv_negative",,drop=FALSE]
metadata<-as.data.frame(as.matrix(metadata[metadata$sexual_orientation!="MSM",,drop=FALSE]))
metadata<-metadata[metadata$sex=="female",,drop=F]
phyloseq::sample_data(ps_gg_fp_f_prevalence_filter_2019_05_26_proportion)<-metadata

#Run PCoA on the phyloseq object
ordination<-phyloseq::ordinate(ps_gg_fp_f_prevalence_filter_2019_05_26_proportion, "PCoA", "unifrac")
```

```
## Warning in matrix(tree$edge[order(tree$edge[, 1]), ][, 2], byrow = TRUE, : data
## length [8987] is not a sub-multiple or multiple of the number of rows [4494]
```

```
ordination$values[1:2,]
```

```
##   Eigenvalues Relative_eig Broken_stick  Cumul_eig Cumul_br_stick
## 1    3.062138   0.07005927   0.04766886 0.07005927     0.04766886
## 2    2.308448   0.05281543   0.03865985 0.12287470     0.08632871
```

```
metadata<-as.data.frame(phyloseq::sample_data(ps_gg_fp_f_prevalence_filter_2019_05_26_proportion))
metadata<-metadata[row.names(ordination$vectors),,drop=FALSE]
all.equal(row.names(metadata), row.names(ordination$vectors))
```

```
## [1] TRUE
```

```
metadata$Unifrac1<-ordination$vectors[,1]
metadata$Unifrac2<-ordination$vectors[,2]

plot1<-ggplot2::ggplot(data=metadata, aes(x=Unifrac1, y=Unifrac2))+geom_point(aes(color=sample_cohort), size=3)+theme_bw()+stat_ellipse(aes(color=sample_cohort))+
  geom_point(data=metadata %>% group_by(sample_cohort) %>% summarise_at(vars(matches("Unifrac")), mean),size=6, aes(color=sample_cohort))+
  scale_color_manual(values=c("royalblue4","darkorange", "forestgreen"))+ggtitle("Figure 1A - JustFemales")

plot1_axis<-ggplot2::ggplot(data=metadata, aes(x=sample_cohort, y=Unifrac1))+coord_flip()+geom_boxplot(aes(fill=sample_cohort), alpha=0.5, outlier.color="white")+theme_bw()+
  scale_fill_manual(values=c("royalblue4","darkorange","forestgreen"))+geom_point(aes(color=sample_cohort), position=position_jitterdodge(jitter.width=0.25),alpha=0.75, size=2)+
  scale_color_manual(values=c("royalblue4","darkorange","forestgreen"))

ggplot2::ggsave("SupplementaryFigure1A_v1.pdf", grid.arrange(plot1,plot1_axis, ncol=2, nrow=2), width=15, height=10)
```

```
#Run Adonis (n=112) and run orm to compare axis position along Axis 1
ASV_table<-as.data.frame(phyloseq::otu_table(ps_gg_fp_f_prevalence_filter_2019_05_26_proportion))
all.equal(row.names(ASV_table), row.names(metadata))
```

```
## [1] TRUE
```

```
unifrac.distance<-unname(phyloseq::UniFrac(ps_gg_fp_f_prevalence_filter_2019_05_26_proportion, weighted = FALSE)) ### unname fixes error introduced by Desctools see https://github.com/joey711/phyloseq/issues/1457
```

```
## Warning in matrix(tree$edge[order(tree$edge[, 1]), ][, 2], byrow = TRUE, : data
## length [8987] is not a sub-multiple or multiple of the number of rows [4494]
```

```
attributes(unifrac.distance)$Labels <- phyloseq::sample_names(ps_gg_fp_f_prevalence_filter_2019_05_26_proportion)
print(vegan::adonis2(unifrac.distance~metadata$sample_cohort, data=ASV_table, permutations=1000)) -> adon_univar
```

```
## Permutation test for adonis under reduced model
## Terms added sequentially (first to last)
## Permutation: free
## Number of permutations: 1000
## 
## vegan::adonis2(formula = unifrac.distance ~ metadata$sample_cohort, data = ASV_table, permutations = 1000)
##                         Df SumOfSqs      R2      F   Pr(>F)    
## metadata$sample_cohort   2    2.990 0.06841 4.0023 0.000999 ***
## Residual               109   40.718 0.93159                    
## Total                  111   43.708 1.00000                    
## ---
## Signif. codes:  0 '***' 0.001 '**' 0.01 '*' 0.05 '.' 0.1 ' ' 1
```

```
###*** sample_cohort r2 = 0.068413 p = 0.000999

# vegan::adonis2(formula = unifrac.distance ~ metadata$sample_cohort, data = ASV_table, permutations = 1000)
#                         Df SumOfSqs      R2      F   Pr(>F)    
# metadata$sample_cohort   2    2.990 0.06841 4.0023 0.000999 ***
# Residual               109   40.718 0.93159                    
# Total                  111   43.708 1.00000   

# Kruskal and orm to test boxplot panel
print(agricolae::kruskal(metadata$Unifrac1, metadata$sample_cohort, group=F, p.adj = "bonferroni")) -> kruskal_univar
```

```
## $statistics
##      Chisq Df      p.chisq
##   59.74024  2 1.065814e-13
## 
## $parameters
##             test  p.ajusted                 name.t ntr alpha
##   Kruskal-Wallis bonferroni metadata$sample_cohort   3  0.05
## 
## $means
##          metadata.Unifrac1     rank        std  r        Min         Max
## boston         -0.16031579 24.48649 0.07623536 37 -0.2565736 -0.01842416
## botswana        0.03048283 62.69444 0.14694058 36 -0.2136109  0.27919726
## uganda_2        0.12395647 81.15385 0.11625423 39 -0.1135911  0.30695830
##                  Q25         Q50        Q75
## boston   -0.23148286 -0.16902596 -0.1127361
## botswana -0.08281186  0.02679362  0.1492383
## uganda_2  0.02913367  0.10863485  0.2243248
## 
## $comparison
##                     Difference pvalue Signif.       LCL        UCL
## boston - botswana    -38.20796 0.0000     *** -50.88489 -25.531025
## boston - uganda_2    -56.66736 0.0000     *** -69.09471 -44.240010
## botswana - uganda_2  -18.45940 0.0015      ** -30.97501  -5.943793
## 
## $groups
## NULL
## 
## attr(,"class")
## [1] "group"
```

```
rms::orm(formula =  Unifrac1~sample_cohort, data = as_tibble(metadata)) -> orm_univar
print(orm_univar)
```

```
## Logistic (Proportional Odds) Ordinal Regression Model
## 
## rms::orm(formula = Unifrac1 ~ sample_cohort, data = as_tibble(metadata))
## 
##                              Model Likelihood               Discrimination    Rank Discrim.    
##                                    Ratio Test                      Indexes          Indexes    
## Obs                 112    LR chi2      76.63    R2                  0.496    rho     0.719    
## Distinct Y          112    d.f.             2    R2(2,112)           0.486                     
## Median Y    -0.01298409    Pr(> chi2) <0.0001    R2(2,112)           0.486                     
## max |deriv|      0.0002    Score chi2   75.58    |Pr(Y>=median)-0.5| 0.295                     
##                            Pr(> chi2) <0.0001                                                  
## 
##                        Coef   S.E.   Wald Z Pr(>|Z|)
## sample_cohort=botswana 2.8632 0.5006 5.72   <0.0001 
## sample_cohort=uganda_2 4.1151 0.5311 7.75   <0.0001
```

```
###*** botswana-boston p = <0.0001 uganda-boston p = <0.0001 uganda-bostwana p = <0.0001

# Logistic (Proportional Odds) Ordinal Regression Model
# 
# rms::orm(formula = Unifrac1 ~ sample_cohort, data = as_tibble(metadata))
# 
#                              Model Likelihood               Discrimination    Rank Discrim.    
#                                    Ratio Test                      Indexes          Indexes    
# Obs                 112    LR chi2      76.63    R2                  0.496    rho     0.719    
# Distinct Y          112    d.f.             2    R2(2,112)           0.486                     
# Median Y    -0.01298409    Pr(> chi2) <0.0001    R2(2,112)           0.486                     
# max |deriv|      0.0002    Score chi2   75.58    |Pr(Y>=median)-0.5| 0.295                     
#                            Pr(> chi2) <0.0001                                                  
# 
#                        Coef   S.E.   Wald Z Pr(>|Z|)
# sample_cohort=botswana 2.8632 0.5006 5.72   <0.0001 
# sample_cohort=uganda_2 4.1151 0.5311 7.75   <0.0001 

### Extra metadata to add that have full n: Race, Ethnicity, age, sex
### Extra metadata to add that have <n:BMI, comorbidities (dm2_hx, hld_hx, htn_hx, cvd_hx, ckd_hx, dm2hx_dx, hldhx_dx, htnhx_dx, cvdhx_dx)
metadata$age <- as.numeric(metadata$age)
metadata$BMI <- as.numeric(metadata$BMI)

### control for metadata with full n
covars_full_n <- c("Ethnicity", "age", "Race", "sample_cohort")
print(vegan::adonis2(as.formula(paste("unifrac.distance~metadata$", paste(covars_full_n, collapse = "+metadata$"), sep = "")), data=ASV_table, permutations=1000)) -> adon_full_n
```

```
## Permutation test for adonis under reduced model
## Terms added sequentially (first to last)
## Permutation: free
## Number of permutations: 1000
## 
## vegan::adonis2(formula = as.formula(paste("unifrac.distance~metadata$", paste(covars_full_n, collapse = "+metadata$"), sep = "")), data = ASV_table, permutations = 1000)
##                         Df SumOfSqs      R2      F   Pr(>F)    
## metadata$Ethnicity       1    0.425 0.00972 1.1424 0.124875    
## metadata$age             1    0.869 0.01988 2.3354 0.000999 ***
## metadata$Race            3    2.104 0.04815 1.8852 0.000999 ***
## metadata$sample_cohort   2    1.613 0.03690 2.1675 0.000999 ***
## Residual               104   38.697 0.88535                    
## Total                  111   43.708 1.00000                    
## ---
## Signif. codes:  0 '***' 0.001 '**' 0.01 '*' 0.05 '.' 0.1 ' ' 1
```

```
###*** sample_cohort r2 = 0.036903 p = 0.000999
#                         Df SumOfSqs      R2      F   Pr(>F)    
# metadata$Ethnicity       1    0.425 0.00972 1.1424 0.124875    
# metadata$age             1    0.869 0.01988 2.3354 0.000999 ***
# metadata$Race            3    2.104 0.04815 1.8852 0.000999 ***
# metadata$sample_cohort   2    1.613 0.03690 2.1675 0.000999 ***
# Residual               104   38.697 0.88535                    
# Total                  111   43.708 1.00000                   

# orm to test boxplot panel
rms::orm(formula =  as.formula(paste("Unifrac1~", paste(covars_full_n, collapse = "+"), sep = "")), data = as_tibble(metadata)) -> orm_full_n
print(orm_full_n)
```

```
## Logistic (Proportional Odds) Ordinal Regression Model
## 
## rms::orm(formula = as.formula(paste("Unifrac1~", paste(covars_full_n, 
##     collapse = "+"), sep = "")), data = as_tibble(metadata))
## 
##                              Model Likelihood               Discrimination    Rank Discrim.    
##                                    Ratio Test                      Indexes          Indexes    
## Obs                 112    LR chi2      89.12    R2                  0.549    rho     0.737    
## Distinct Y          112    d.f.             7    R2(7,112)           0.520                     
## Median Y    -0.01298409    Pr(> chi2) <0.0001    R2(7,112)           0.520                     
## max |deriv|      0.0008    Score chi2   90.70    |Pr(Y>=median)-0.5| 0.289                     
##                            Pr(> chi2) <0.0001                                                  
## 
##                               Coef    S.E.   Wald Z Pr(>|Z|)
## Ethnicity=Not_Hispanic_Latino -0.8913 0.8544 -1.04  0.2969  
## age                            0.0395 0.0181  2.18  0.0291  
## Race=Black_AA                 -3.4851 1.6230 -2.15  0.0318  
## Race=Unknown                  -0.9494 1.8187 -0.52  0.6017  
## Race=White                    -3.8077 1.5547 -2.45  0.0143  
## sample_cohort=botswana         3.2255 0.7323  4.40  <0.0001 
## sample_cohort=uganda_2         4.0517 0.7544  5.37  <0.0001
```

```
###*** botswana-boston p = <0.0001 uganda-boston p = <0.0001 uganda-bostwana p = <0.0001

# Logistic (Proportional Odds) Ordinal Regression Model
# 
# rms::orm(formula = as.formula(paste("Unifrac1~", paste(covars_full_n, 
#     collapse = "+"), sep = "")), data = as_tibble(metadata))
# 
#                              Model Likelihood               Discrimination    Rank Discrim.    
#                                    Ratio Test                      Indexes          Indexes    
# Obs                 112    LR chi2      89.12    R2                  0.549    rho     0.737    
# Distinct Y          112    d.f.             7    R2(7,112)           0.520                     
# Median Y    -0.01298409    Pr(> chi2) <0.0001    R2(7,112)           0.520                     
# max |deriv|      0.0008    Score chi2   90.70    |Pr(Y>=median)-0.5| 0.289                     
#                            Pr(> chi2) <0.0001                                                  
# 
#                               Coef    S.E.   Wald Z Pr(>|Z|)
# Ethnicity=Not_Hispanic_Latino -0.8913 0.8544 -1.04  0.2969  
# age                            0.0395 0.0181  2.18  0.0291  
# Race=Black_AA                 -3.4851 1.6230 -2.15  0.0318  
# Race=Unknown                  -0.9494 1.8187 -0.52  0.6017  
# Race=White                    -3.8077 1.5547 -2.45  0.0143  
# sample_cohort=botswana         3.2255 0.7323  4.40  <0.0001 
# sample_cohort=uganda_2         4.0517 0.7544  5.37  <0.0001 

### remove samples with BMI/comorbidities is NA (n=112 -> n=103)
ps_gg_fp_f_prevalence_filter_2019_05_26_proportion_bc <- ps_gg_fp_f_prevalence_filter_2019_05_26_proportion
metadata_bc<-metadata[!is.na(metadata$BMI) & !is.na(metadata$dm2_hx),,drop=FALSE]
sample_data(ps_gg_fp_f_prevalence_filter_2019_05_26_proportion_bc) <- metadata_bc

### subset ASV table and distance matrix
ASV_table_bc<-as.data.frame(otu_table(ps_gg_fp_f_prevalence_filter_2019_05_26_proportion_bc))
all.equal(row.names(ASV_table_bc), row.names(metadata_bc))
```

```
## [1] TRUE
```

```
unifrac.distance_bc <- unname(usedist::dist_subset(unifrac.distance, sample_names(ps_gg_fp_f_prevalence_filter_2019_05_26_proportion_bc))) ### unname fixes error introduced by Desctools see https://github.com/joey711/phyloseq/issues/1457

### run adonis
covars_bc <- c("BMI", "dm2_hx", "hld_hx", "htn_hx", "cvd_hx", "dm2hx_dx", "hldhx_dx", "htnhx_dx", "cvdhx_dx", covars_full_n)
print(vegan::adonis2(as.formula(paste("unifrac.distance_bc~metadata_bc$", paste(covars_bc, collapse = "+metadata_bc$"), sep = "")), data=ASV_table_bc, permutations=1000)) -> adon_bc
```

```
## Permutation test for adonis under reduced model
## Terms added sequentially (first to last)
## Permutation: free
## Number of permutations: 1000
## 
## vegan::adonis2(formula = as.formula(paste("unifrac.distance_bc~metadata_bc$", paste(covars_bc, collapse = "+metadata_bc$"), sep = "")), data = ASV_table_bc, permutations = 1000)
##                            Df SumOfSqs      R2      F   Pr(>F)    
## metadata_bc$BMI             1    0.390 0.00978 1.0618 0.294705    
## metadata_bc$dm2_hx          1    0.402 0.01009 1.0951 0.190809    
## metadata_bc$hld_hx          1    0.443 0.01112 1.2071 0.077922 .  
## metadata_bc$htn_hx          1    0.685 0.01719 1.8655 0.000999 ***
## metadata_bc$cvd_hx          1    0.416 0.01043 1.1323 0.156843    
## metadata_bc$dm2hx_dx        1    0.488 0.01223 1.3275 0.022977 *  
## metadata_bc$hldhx_dx        1    0.533 0.01338 1.4522 0.007992 ** 
## metadata_bc$cvdhx_dx        1    0.576 0.01445 1.5681 0.003996 ** 
## metadata_bc$Ethnicity       1    0.437 0.01097 1.1903 0.093906 .  
## metadata_bc$age             1    0.624 0.01566 1.6998 0.001998 ** 
## metadata_bc$Race            3    1.651 0.04140 1.4978 0.000999 ***
## metadata_bc$sample_cohort   2    1.262 0.03165 1.7176 0.000999 ***
## Residual                   87   31.958 0.80163                    
## Total                     102   39.866 1.00000                    
## ---
## Signif. codes:  0 '***' 0.001 '**' 0.01 '*' 0.05 '.' 0.1 ' ' 1
```

```
###*** sample_cohort r2 = 0.031653 p = 0.000999

#                            Df SumOfSqs      R2      F   Pr(>F)    
# metadata_bc$BMI             1    0.390 0.00978 1.0618 0.294705    
# metadata_bc$dm2_hx          1    0.402 0.01009 1.0951 0.190809    
# metadata_bc$hld_hx          1    0.443 0.01112 1.2071 0.077922 .  
# metadata_bc$htn_hx          1    0.685 0.01719 1.8655 0.000999 ***
# metadata_bc$cvd_hx          1    0.416 0.01043 1.1323 0.156843    
# metadata_bc$dm2hx_dx        1    0.488 0.01223 1.3275 0.022977 *  
# metadata_bc$hldhx_dx        1    0.533 0.01338 1.4522 0.007992 ** 
# metadata_bc$cvdhx_dx        1    0.576 0.01445 1.5681 0.003996 ** 
# metadata_bc$Ethnicity       1    0.437 0.01097 1.1903 0.093906 .  
# metadata_bc$age             1    0.624 0.01566 1.6998 0.001998 ** 
# metadata_bc$Race            3    1.651 0.04140 1.4978 0.000999 ***
# metadata_bc$sample_cohort   2    1.262 0.03165 1.7176 0.000999 ***
# Residual                   87   31.958 0.80163                    
# Total                     102   39.866 1.00000                    

# orm to test boxplot panel
rms::orm(formula =  as.formula(paste("Unifrac1~", paste(covars_bc[!covars_bc %in% c("htn_hx")], collapse = "+"), sep = "")), data = as_tibble(metadata)) -> orm_bc
print(orm_bc)
```

```
## Frequencies of Missing Values Due to Each Variable
##      Unifrac1           BMI        dm2_hx        hld_hx        cvd_hx 
##             0             6             9             9             9 
##      dm2hx_dx      hldhx_dx      htnhx_dx      cvdhx_dx     Ethnicity 
##             9             9             9             9             0 
##           age          Race sample_cohort 
##             0             0             0 
## 
## Logistic (Proportional Odds) Ordinal Regression Model
## 
## rms::orm(formula = as.formula(paste("Unifrac1~", paste(covars_bc[!covars_bc %in% 
##     c("htn_hx")], collapse = "+"), sep = "")), data = as_tibble(metadata))
## 
## 
##                            Model Likelihood               Discrimination    Rank Discrim.    
##                                  Ratio Test                      Indexes          Indexes    
## Obs               103    LR chi2      83.57    R2                  0.556    rho     0.746    
## Distinct Y        103    d.f.            15    R2(15,103)          0.486                     
## Median Y    0.0235283    Pr(> chi2) <0.0001    R2(15,103)          0.486                     
## max |deriv|    0.0009    Score chi2   87.21    |Pr(Y>=median)-0.5| 0.279                     
##                          Pr(> chi2) <0.0001                                                  
## 
##                               Coef    S.E.   Wald Z Pr(>|Z|)
## BMI                           -0.1083 0.0405 -2.67  0.0075  
## dm2_hx= 1                      2.5988 1.4813  1.75  0.0794  
## hld_hx= 1                     -1.4645 0.9513 -1.54  0.1237  
## cvd_hx= 1                      0.5765 0.9209  0.63  0.5313  
## dm2hx_dx= 1                   -2.3733 1.1786 -2.01  0.0440  
## hldhx_dx= 1                    1.1069 0.5915  1.87  0.0613  
## htnhx_dx= 1                    0.7383 0.5448  1.36  0.1754  
## cvdhx_dx= 1                   -0.8687 0.8131 -1.07  0.2853  
## Ethnicity=Not_Hispanic_Latino -0.6601 1.2099 -0.55  0.5854  
## age                            0.0358 0.0220  1.62  0.1042  
## Race=Black_AA                 -2.3916 1.8090 -1.32  0.1862  
## Race=Unknown                  -1.4918 2.3370 -0.64  0.5232  
## Race=White                    -3.1924 1.5649 -2.04  0.0414  
## sample_cohort=botswana         2.4163 1.0012  2.41  0.0158  
## sample_cohort=uganda_2         3.5118 0.9633  3.65  0.0003
```

```
###*** botswana-boston p = 0.0158 uganda-boston p = 0.0003 uganda-bostwana p = 0.0452

# Logistic (Proportional Odds) Ordinal Regression Model
# 
# rms::orm(formula = as.formula(paste("Unifrac1~", paste(covars_bc[!covars_bc %in% 
#     c("htn_hx")], collapse = "+"), sep = "")), data = as_tibble(metadata))
# 
# 
#                            Model Likelihood               Discrimination    Rank Discrim.    
#                                  Ratio Test                      Indexes          Indexes    
# Obs               103    LR chi2      83.57    R2                  0.556    rho     0.746    
# Distinct Y        103    d.f.            15    R2(15,103)          0.486                     
# Median Y    0.0235283    Pr(> chi2) <0.0001    R2(15,103)          0.486                     
# max |deriv|    0.0009    Score chi2   87.21    |Pr(Y>=median)-0.5| 0.279                     
#                          Pr(> chi2) <0.0001                                                  
# 
#                               Coef    S.E.   Wald Z Pr(>|Z|)
# BMI                           -0.1083 0.0405 -2.67  0.0075  
# dm2_hx= 1                      2.5988 1.4813  1.75  0.0794  
# hld_hx= 1                     -1.4645 0.9513 -1.54  0.1237  
# cvd_hx= 1                      0.5765 0.9209  0.63  0.5313  
# dm2hx_dx= 1                   -2.3733 1.1786 -2.01  0.0440  
# hldhx_dx= 1                    1.1069 0.5915  1.87  0.0613  
# htnhx_dx= 1                    0.7383 0.5448  1.36  0.1754  
# cvdhx_dx= 1                   -0.8687 0.8131 -1.07  0.2853  
# Ethnicity=Not_Hispanic_Latino -0.6601 1.2099 -0.55  0.5854  
# age                            0.0358 0.0220  1.62  0.1042  
# Race=Black_AA                 -2.3916 1.8090 -1.32  0.1862  
# Race=Unknown                  -1.4918 2.3370 -0.64  0.5232  
# Race=White                    -3.1924 1.5649 -2.04  0.0414  
# sample_cohort=botswana         2.4163 1.0012  2.41  0.0158  
# sample_cohort=uganda_2         3.5118 0.9633  3.65  0.0003  

#--------------------------------------------------------------------------------------------------------------
```

```
#FIGURE B (1B)
#--------------------------------------------------------------------------------------------------------------
#Tax glom at the Family level
ps_gg_fp_f_prevalence_filter_2019_05_26_agglomerate<-phyloseq::tax_glom(ps_gg_fp_f_prevalence_filter_2019_05_26, taxrank="Family")

#Apply filters: min 5000 counts in 50% of the samples
phyloseq::otu_table(ps_gg_fp_f_prevalence_filter_2019_05_26_agglomerate)<-t(phyloseq::otu_table(ps_gg_fp_f_prevalence_filter_2019_05_26_agglomerate))
filter_conditions<-phyloseq::filterfun_sample(function(x) x>=5000)
filtered<-phyloseq::genefilter_sample(phyloseq::otu_table(ps_gg_fp_f_prevalence_filter_2019_05_26_agglomerate), filter_conditions, A=(0.5*nrow(phyloseq::otu_table(ps_gg_fp_f_prevalence_filter_2019_05_26_agglomerate))))
ps_gg_fp_f_prevalence_filter_2019_05_26_agglomerate_filtered<-phyloseq::prune_taxa(filtered, ps_gg_fp_f_prevalence_filter_2019_05_26_agglomerate)

#Select samples of interest (the metadata dataframe has been already been subset before)
phyloseq::sample_data(ps_gg_fp_f_prevalence_filter_2019_05_26_agglomerate_filtered)<-metadata

#Calculate relative abundance
ps_gg_fp_f_prevalence_filter_2019_05_26_agglomerate_filtered_proportion<-phyloseq::transform_sample_counts(ps_gg_fp_f_prevalence_filter_2019_05_26_agglomerate_filtered, function(x)(x/sum(x)))

#Get data out from phyloseq object to plot with ggplot2:
ASV_table<-as.data.frame(t(phyloseq::otu_table(ps_gg_fp_f_prevalence_filter_2019_05_26_agglomerate_filtered_proportion)))
TAX_table<-as.data.frame(as.matrix(ps_gg_fp_f_prevalence_filter_2019_05_26_agglomerate_filtered_proportion@tax_table@.Data))

dim(TAX_table)
```

```
## [1] 7 7
```

```
TAX_table$Name<-paste(TAX_table$Kingdom, TAX_table$Phylum, TAX_table$Class, TAX_table$Order, TAX_table$Family)
all.equal(colnames(ASV_table), row.names(TAX_table))
```

```
## [1] TRUE
```

```
colnames(ASV_table)<- TAX_table$Name

all.equal(row.names(ASV_table), row.names(metadata))
```

```
## [1] TRUE
```

```
ASV_table$sample_cohort<-metadata$sample_cohort
ASV_table$SampleID<-row.names(ASV_table)
ASV_table$hiv_phenotype<-metadata$hiv_phenotype
ASV_table$Unifrac1<-metadata$Unifrac1

#Sort samples by position in Axis1 from Figure 1A
ASV_table<-ASV_table[order(ASV_table$Unifrac1),,drop=FALSE]
ASV_table$SampleID<-factor(ASV_table$SampleID, levels=ASV_table$SampleID)
ASV_table$Unifrac1<-as.factor(ASV_table$Unifrac1)
ASV_table_melt<-reshape::melt(ASV_table)
```

```
## Using sample_cohort, SampleID, hiv_phenotype, Unifrac1 as id variables
```

```
ASV_table_melt$variable<-as.character(ASV_table_melt$variable)
ASV_table_melt$Family<-stringr::str_split_fixed(ASV_table_melt$variable,"_",4)[,1]
ASV_table_melt$Genus<-stringr::str_split_fixed(ASV_table_melt$variable,"_",4)[,2]
ASV_table_melt$Species<-paste(stringr::str_split_fixed(ASV_table_melt$variable,"_",4)[,2],stringr::str_split_fixed(ASV_table_melt$variable,"_",4)[,3])

#Kruskal Wallis test for differential abundance between cohorts
family<-c()
all_sig<-c() #We are interested in those families have all pairwise comparisons statistically significant:
for (i in colnames(ASV_table)[1:(dim(ASV_table)[2]-5)]){
  result<-kruskal.test(ASV_table[[i]],ASV_table[["sample_cohort"]])
  if (result$p.value<=0.01){
    print(i)
    result2<-agricolae::kruskal(ASV_table[[i]],ASV_table[["sample_cohort"]],group=FALSE, p.adj ="BH")
    if (result2$comparison$pvalue[1]<0.1 & result2$comparison$pvalue[2]<0.1 & result2$comparison$pvalue[3]<0.1){
      all_sig<-c(all_sig, i)
    }
    family<-c(family, i)
  }
}
```

```
## [1] "Bacteria Bacteroidetes Bacteroidia Bacteroidales Bacteroidaceae"
## [1] "Bacteria Bacteroidetes Bacteroidia Bacteroidales Prevotellaceae"
```

```
# Add all metadata to the table for multivariate testing of abundance differences
metadata$age <- as.numeric(metadata$age)
metadata$BMI <- as.numeric(metadata$BMI)
dplyr::left_join(ASV_table[colnames(ASV_table) %in% c("SampleID", "Unifrac1") | !colnames(ASV_table) %in% colnames(metadata)], metadata[, !colnames(metadata) %in% c("Unifrac1")], by = "SampleID") -> ASV_table_full_metadata

# Run orm (n=112) and compare family abundances between sample_cohort
rms::orm(formula =  as.formula(paste("ASV_table_full_metadata[[", which(stringr::str_detect(colnames(ASV_table_full_metadata), "Bacteroidaceae")), "]]~", "sample_cohort", sep = "")), data = ASV_table_full_metadata) -> orm_abund_bact
print(orm_abund_bact)
```

```
## Logistic (Proportional Odds) Ordinal Regression Model
## 
## rms::orm(formula = as.formula(paste("ASV_table_full_metadata[[", 
##     which(stringr::str_detect(colnames(ASV_table_full_metadata), 
##         "Bacteroidaceae")), "]]~", "sample_cohort", sep = "")), 
##     data = ASV_table_full_metadata)
## 
##                              Model Likelihood               Discrimination    Rank Discrim.    
##                                    Ratio Test                      Indexes          Indexes    
## Obs                 112    LR chi2      68.29    R2                  0.457    rho     0.703    
## Distinct Y           96    d.f.             2    R2(2,112)           0.447                     
## Median Y    0.007536948    Pr(> chi2) <0.0001    R2(2,111.6)         0.448                     
## max |deriv|       6e-06    Score chi2   67.15    |Pr(Y>=median)-0.5| 0.255                     
##                            Pr(> chi2) <0.0001                                                  
## 
##                        Coef    S.E.   Wald Z Pr(>|Z|)
## sample_cohort=botswana -2.0788 0.4556 -4.56  <0.0001 
## sample_cohort=uganda_2 -3.8592 0.5136 -7.51  <0.0001
```

```
###*** botswana-boston p = <0.0001 uganda-boston p = <0.0001 uganda-bostwana p = <0.0001

# rms::orm(formula = as.formula(paste("ASV_table_full_metadata[[", 
#     which(stringr::str_detect(colnames(ASV_table_full_metadata), 
#         "Bacteroidaceae")), "]]~", "sample_cohort", sep = "")), 
#     data = ASV_table_full_metadata)
# 
#                               Model Likelihood               Discrimination    Rank Discrim.    
#                                     Ratio Test                      Indexes          Indexes    
# Obs                  112    LR chi2      68.29    R2                  0.457    rho     0.703    
# Distinct Y            96    d.f.             2    R2(2,112)           0.447                     
# Median Y    0.0075369476    Pr(> chi2) <0.0001    R2(2,111.6)         0.448                     
# max |deriv|        6e-06    Score chi2   67.15    |Pr(Y>=median)-0.5| 0.255                     
#                             Pr(> chi2) <0.0001                                                  
# 
#                        Coef    S.E.   Wald Z Pr(>|Z|)
# sample_cohort=botswana -2.0788 0.4556 -4.56  <0.0001 
# sample_cohort=uganda_2 -3.8592 0.5136 -7.51  <0.0001 

rms::orm(formula =  as.formula(paste("ASV_table_full_metadata[[", which(stringr::str_detect(colnames(ASV_table_full_metadata), "Prevotellaceae")), "]]~", "sample_cohort", sep = "")), data = ASV_table_full_metadata) -> orm_abund_prev
print(orm_abund_prev)
```

```
## Logistic (Proportional Odds) Ordinal Regression Model
## 
## rms::orm(formula = as.formula(paste("ASV_table_full_metadata[[", 
##     which(stringr::str_detect(colnames(ASV_table_full_metadata), 
##         "Prevotellaceae")), "]]~", "sample_cohort", sep = "")), 
##     data = ASV_table_full_metadata)
## 
##                            Model Likelihood               Discrimination    Rank Discrim.    
##                                  Ratio Test                      Indexes          Indexes    
## Obs               112    LR chi2      31.35    R2                  0.244    rho     0.475    
## Distinct Y        107    d.f.             2    R2(2,112)           0.231                     
## Median Y    0.1297365    Pr(> chi2) <0.0001    R2(2,112)           0.231                     
## max |deriv|     6e-07    Score chi2   30.85    |Pr(Y>=median)-0.5| 0.199                     
##                          Pr(> chi2) <0.0001                                                  
## 
##                        Coef   S.E.   Wald Z Pr(>|Z|)
## sample_cohort=botswana 1.7774 0.4512 3.94   <0.0001 
## sample_cohort=uganda_2 2.4144 0.4601 5.25   <0.0001
```

```
###*** botswana-boston p = <0.0001 uganda-boston p = <0.0001 uganda-bostwana p = <0.0001

# rms::orm(formula = as.formula(paste("ASV_table_full_metadata[[", 
#     which(stringr::str_detect(colnames(ASV_table_full_metadata), 
#         "Prevotellaceae")), "]]~", "sample_cohort", sep = "")), 
#     data = ASV_table_full_metadata)
# 
#                             Model Likelihood               Discrimination    Rank Discrim.    
#                                   Ratio Test                      Indexes          Indexes    
# Obs                112    LR chi2      31.35    R2                  0.244    rho     0.475    
# Distinct Y         107    d.f.             2    R2(2,112)           0.231                     
# Median Y    0.12973647    Pr(> chi2) <0.0001    R2(2,112)           0.231                     
# max |deriv|      6e-07    Score chi2   30.85    |Pr(Y>=median)-0.5| 0.199                     
#                           Pr(> chi2) <0.0001                                                  
# 
#                        Coef   S.E.   Wald Z Pr(>|Z|)
# sample_cohort=botswana 1.7774 0.4512 3.94   <0.0001 
# sample_cohort=uganda_2 2.4144 0.4601 5.25   <0.0001 

### Extra metadata to add that have full n: Race, Ethnicity, age, sex
### Extra metadata to add that have <n:BMI, comorbidities (dm2_hx, hld_hx, htn_hx, cvd_hx, ckd_hx, dm2hx_dx, hldhx_dx, htnhx_dx, cvdhx_dx)
### control for metadata with full n
covars_full_n <- c("Ethnicity", "age", "Race", "sample_cohort")
rms::orm(formula =  as.formula(paste("ASV_table_full_metadata[[", which(stringr::str_detect(colnames(ASV_table_full_metadata), "Bacteroidaceae")), "]]~", paste(covars_full_n, collapse = "+"), sep = "")), data = ASV_table_full_metadata) -> orm_abund_bact_full_n
print(orm_abund_bact_full_n)
```

```
## Logistic (Proportional Odds) Ordinal Regression Model
## 
## rms::orm(formula = as.formula(paste("ASV_table_full_metadata[[", 
##     which(stringr::str_detect(colnames(ASV_table_full_metadata), 
##         "Bacteroidaceae")), "]]~", paste(covars_full_n, collapse = "+"), 
##     sep = "")), data = ASV_table_full_metadata)
## 
##                              Model Likelihood               Discrimination    Rank Discrim.    
##                                    Ratio Test                      Indexes          Indexes    
## Obs                 112    LR chi2      76.62    R2                  0.496    rho     0.696    
## Distinct Y           96    d.f.             7    R2(7,112)           0.463                     
## Median Y    0.007536948    Pr(> chi2) <0.0001    R2(7,111.6)         0.464                     
## max |deriv|      0.0003    Score chi2   78.40    |Pr(Y>=median)-0.5| 0.257                     
##                            Pr(> chi2) <0.0001                                                  
## 
##                               Coef    S.E.   Wald Z Pr(>|Z|)
## Ethnicity=Not_Hispanic_Latino -1.3561 1.0536 -1.29  0.1980  
## age                            0.0075 0.0183  0.41  0.6821  
## Race=Black_AA                  2.3089 1.5961  1.45  0.1480  
## Race=Unknown                  -0.9613 1.9188 -0.50  0.6164  
## Race=White                     1.5411 1.5080  1.02  0.3068  
## sample_cohort=botswana        -2.8756 0.7246 -3.97  <0.0001 
## sample_cohort=uganda_2        -4.7742 0.8058 -5.93  <0.0001
```

```
###*** botswana-boston p = <0.0001 uganda-boston p = <0.0001 uganda-bostwana p = <0.0001
rms::orm(formula =  as.formula(paste("ASV_table_full_metadata[[", which(stringr::str_detect(colnames(ASV_table_full_metadata), "Prevotellaceae")), "]]~", paste(covars_full_n, collapse = "+"), sep = "")), data = ASV_table_full_metadata) -> orm_abund_prev_full_n
print(orm_abund_prev_full_n)
```

```
## Logistic (Proportional Odds) Ordinal Regression Model
## 
## rms::orm(formula = as.formula(paste("ASV_table_full_metadata[[", 
##     which(stringr::str_detect(colnames(ASV_table_full_metadata), 
##         "Prevotellaceae")), "]]~", paste(covars_full_n, collapse = "+"), 
##     sep = "")), data = ASV_table_full_metadata)
## 
##                            Model Likelihood               Discrimination    Rank Discrim.    
##                                  Ratio Test                      Indexes          Indexes    
## Obs               112    LR chi2      40.56    R2                  0.304    rho     0.489    
## Distinct Y        107    d.f.             7    R2(7,112)           0.259                     
## Median Y    0.1297365    Pr(> chi2) <0.0001    R2(7,112)           0.259                     
## max |deriv|     8e-06    Score chi2   41.38    |Pr(Y>=median)-0.5| 0.200                     
##                          Pr(> chi2) <0.0001                                                  
## 
##                               Coef    S.E.   Wald Z Pr(>|Z|)
## Ethnicity=Not_Hispanic_Latino  0.3798 0.9253  0.41  0.6815  
## age                            0.0215 0.0180  1.20  0.2314  
## Race=Black_AA                 -0.9786 1.5789 -0.62  0.5354  
## Race=Unknown                   1.1884 1.9714  0.60  0.5466  
## Race=White                    -2.2808 1.5302 -1.49  0.1361  
## sample_cohort=botswana         1.1215 0.6856  1.64  0.1019  
## sample_cohort=uganda_2         1.5076 0.7283  2.07  0.0385
```

```
###*** botswana-boston p = 0.1019 uganda-boston p = 0.0385 uganda-bostwana p = 0.3978

ASV_table_melt_significant<-ASV_table_melt[ASV_table_melt$variable%in%all_sig,,drop=FALSE]
unique(ASV_table_melt_significant$variable)
```

```
## [1] "Bacteria Bacteroidetes Bacteroidia Bacteroidales Bacteroidaceae"
## [2] "Bacteria Bacteroidetes Bacteroidia Bacteroidales Prevotellaceae"
```

```
ordered_names<-c(names(sort(colSums(ASV_table[1:(dim(ASV_table)[2]-5)]), decreasing=FALSE)))

ASV_table_melt_significant$variable<-factor(ASV_table_melt_significant$variable, levels=ordered_names)
ASV_table_melt_significant$Unifrac1<-as.numeric(as.character(ASV_table_melt_significant$Unifrac1))

plot1<-ggplot(data=ASV_table_melt_significant, aes(x=Unifrac1, y=value))+geom_point(aes(color=sample_cohort), size=2.5)+
         stat_smooth(geom = "area", method = "loess", alpha = 0.5, size = 1,fill="grey60")+theme_bw()+
         facet_wrap(~variable, scales="free_y", ncol=1)+
         theme(legend.position = "left", axis.text.x = element_text(size=3), panel.grid.major.x=element_blank())+
         scale_colour_manual(values=c("royalblue4","darkorange","forestgreen"))
```

```
## Warning: Using `size` aesthetic for lines was deprecated in ggplot2 3.4.0.
## ℹ Please use `linewidth` instead.
```

```
ggsave("SupplementaryFigure1B.pdf", plot1)
```

```
## Saving 7 x 5 in image
```

```
## `geom_smooth()` using formula = 'y ~ x'
```

```
#--------------------------------------------------------------------------------------------------------------
```

```
#FIGURE C (2A)
#--------------------------------------------------------------------------------------------------------------
#COMPARISON 1 NEG vs ART
#Transform count data in the phyloseq object
ps_gg_fp_f_prevalence_filter_2019_05_26_proportion<-phyloseq::transform_sample_counts(ps_gg_fp_f_prevalence_filter_2019_05_26, function(x)(x/sum(x)))

#Select samples of interest and update phyloseq object 
metadata<-phyloseq::sample_data(ps_gg_fp_f_prevalence_filter_2019_05_26)
metadata<-metadata[metadata$hiv_phenotype%in%c("1_hiv_negative","2_suppressed"),,drop=F]
metadata<-as.data.frame(as.matrix(metadata[metadata$sexual_orientation!="MSM",,drop=FALSE]))
metadata<-metadata[metadata$sex=="female",,drop=F]
phyloseq::sample_data(ps_gg_fp_f_prevalence_filter_2019_05_26_proportion)<-metadata

#Run PCoA on the phyloseq object
ordination<-phyloseq::ordinate(ps_gg_fp_f_prevalence_filter_2019_05_26_proportion, "PCoA", "unifrac")
```

```
## Warning in matrix(tree$edge[order(tree$edge[, 1]), ][, 2], byrow = TRUE, : data
## length [8987] is not a sub-multiple or multiple of the number of rows [4494]
```

```
ordination$values[1:2,]
```

```
##   Eigenvalues Relative_eig Broken_stick  Cumul_eig Cumul_br_stick
## 1    4.372220   0.05476327   0.02891071 0.05476327     0.02891071
## 2    4.082333   0.05113237   0.02400875 0.10589564     0.05291946
```

```
metadata_ordered<-metadata[row.names(ordination$vectors),,drop=FALSE]

all.equal(row.names(metadata_ordered), row.names(ordination$vectors))
```

```
## [1] TRUE
```

```
metadata_ordered$Unifrac1<-ordination$vectors[,1]
metadata_ordered$Unifrac2<-ordination$vectors[,2]

comparison1<-metadata_ordered
comparison1$Grouping<-as.factor(paste(comparison1$hiv_phenotype, comparison1$sample_cohort, sep="_"))

#Let's plot the data
us<-comparison1[comparison1$sample_cohort=="boston",,drop=F]
botswana<-comparison1[comparison1$sample_cohort=="botswana",,drop=F]
uganda<-comparison1[comparison1$sample_cohort=="uganda_2",,drop=F]

plot_us_comparison<-ggplot2::ggplot(data=us, aes(x=Unifrac1, y=Unifrac2))+geom_point(color="royalblue4", aes(alpha=hiv_phenotype), size=2, shape=16)+
  theme_bw()+stat_ellipse(color="royalblue4", aes(alpha=hiv_phenotype), size=1)+scale_alpha_manual(values=c(1,0.3))+ylim(c(-0.5, 0.5))+xlim(c(-0.5,0.5))+
  ggtitle("neg-art boston")+geom_point(data=us %>% group_by(hiv_phenotype) %>% summarise_at(vars(matches("UniFrac")), mean),size=5, color="royalblue4", aes(alpha=hiv_phenotype))
plot_botswana_comparison<-ggplot2::ggplot(data=botswana, aes(x=Unifrac1, y=Unifrac2))+geom_point(color="darkorange", aes(alpha=hiv_phenotype), size=2, shape=16)+
  theme_bw()+stat_ellipse(color="darkorange", aes(alpha=hiv_phenotype), size=1)+scale_alpha_manual(values=c(1,0.3))+ylim(c(-0.5, 0.5))+xlim(c(-0.5,0.5))+
  ggtitle("neg-art botswana")+geom_point(data=botswana %>% group_by(hiv_phenotype) %>% summarise_at(vars(matches("UniFrac")), mean),size=5, color="darkorange", aes(alpha=hiv_phenotype))
plot_uganda_comparison<-ggplot2::ggplot(data=uganda, aes(x=Unifrac1, y=Unifrac2))+geom_point(color="forestgreen", aes(alpha=hiv_phenotype), size=2, shape=16)+
  theme_bw()+stat_ellipse(color="forestgreen", aes(alpha=hiv_phenotype), size=1)+scale_alpha_manual(values=c(1,0.3))+ylim(c(-0.5, 0.5))+xlim(c(-0.5,0.5))+
  ggtitle("neg-art uganda")+geom_point(data=uganda %>% group_by(hiv_phenotype) %>% summarise_at(vars(matches("UniFrac")), mean),size=5, color="forestgreen", aes(alpha=hiv_phenotype))

ggsave("SupplementaryFigure1C_1_v1.pdf", grid.arrange(plot_us_comparison,plot_botswana_comparison,plot_uganda_comparison, nrow=3, ncol=3), width=15, height=10)
```

```
#Adonis (n = 205)
ASV_table<-as.data.frame(phyloseq::otu_table(ps_gg_fp_f_prevalence_filter_2019_05_26_proportion))
all.equal(row.names(ASV_table), row.names(metadata_ordered))
```

```
## [1] TRUE
```

```
unifrac.distance<-unname(phyloseq::UniFrac(ps_gg_fp_f_prevalence_filter_2019_05_26_proportion, weighted = FALSE)) ### unname fixes error introduced by Desctools see https://github.com/joey711/phyloseq/issues/1457
```

```
## Warning in matrix(tree$edge[order(tree$edge[, 1]), ][, 2], byrow = TRUE, : data
## length [8987] is not a sub-multiple or multiple of the number of rows [4494]
```

```
attributes(unifrac.distance)$Labels <- phyloseq::sample_names(ps_gg_fp_f_prevalence_filter_2019_05_26_proportion)
print(vegan::adonis2(unifrac.distance~metadata_ordered$hiv_phenotype, data=ASV_table, permutations=1000)) -> adon_hiv_phenotype
```

```
## Permutation test for adonis under reduced model
## Terms added sequentially (first to last)
## Permutation: free
## Number of permutations: 1000
## 
## vegan::adonis2(formula = unifrac.distance ~ metadata_ordered$hiv_phenotype, data = ASV_table, permutations = 1000)
##                                 Df SumOfSqs      R2     F   Pr(>F)    
## metadata_ordered$hiv_phenotype   1    0.716 0.00897 1.837 0.000999 ***
## Residual                       203   79.123 0.99103                   
## Total                          204   79.839 1.00000                   
## ---
## Signif. codes:  0 '***' 0.001 '**' 0.01 '*' 0.05 '.' 0.1 ' ' 1
```

```
###*** hiv_phenotype r2 = 0.008968 p = 0.000999

# vegan::adonis2(formula = unifrac.distance ~ metadata_ordered$hiv_phenotype, data = ASV_table, permutations = 1000)
#                                 Df SumOfSqs       R2     F   Pr(>F)    
# metadata_ordered$hiv_phenotype   1   0.7160 0.008968 1.837 0.000999 ***
# Residual                       203  79.1225 0.991032                   
# Total                          204  79.8385 1.000000               

print(vegan::adonis2(unifrac.distance~metadata_ordered$sample_cohort, data=ASV_table, permutations=1000)) -> adon_cohort
```

```
## Permutation test for adonis under reduced model
## Terms added sequentially (first to last)
## Permutation: free
## Number of permutations: 1000
## 
## vegan::adonis2(formula = unifrac.distance ~ metadata_ordered$sample_cohort, data = ASV_table, permutations = 1000)
##                                 Df SumOfSqs      R2      F   Pr(>F)    
## metadata_ordered$sample_cohort   2    4.160 0.05211 5.5522 0.000999 ***
## Residual                       202   75.678 0.94789                    
## Total                          204   79.839 1.00000                    
## ---
## Signif. codes:  0 '***' 0.001 '**' 0.01 '*' 0.05 '.' 0.1 ' ' 1
```

```
###*** sample_cohort r2 = 0.052108 p = 0.000999

# vegan::adonis2(formula = unifrac.distance ~ metadata_ordered$sample_cohort, data = ASV_table, permutations = 1000)
#                                 Df SumOfSqs       R2      F   Pr(>F)    
# metadata_ordered$sample_cohort   2   4.1602 0.052108 5.5522 0.000999 ***
# Residual                       202  75.6783 0.947892                    
# Total                          204  79.8385 1.000000                          

print(vegan::adonis2(unifrac.distance~metadata_ordered$hiv_phenotype+metadata_ordered$sample_cohort, data=ASV_table, permutations=1000)) -> adon_cohort_phenotype
```

```
## Permutation test for adonis under reduced model
## Terms added sequentially (first to last)
## Permutation: free
## Number of permutations: 1000
## 
## vegan::adonis2(formula = unifrac.distance ~ metadata_ordered$hiv_phenotype + metadata_ordered$sample_cohort, data = ASV_table, permutations = 1000)
##                                 Df SumOfSqs      R2      F   Pr(>F)    
## metadata_ordered$hiv_phenotype   1    0.716 0.00897 1.9204 0.000999 ***
## metadata_ordered$sample_cohort   2    4.181 0.05237 5.6069 0.000999 ***
## Residual                       201   74.942 0.93866                    
## Total                          204   79.839 1.00000                    
## ---
## Signif. codes:  0 '***' 0.001 '**' 0.01 '*' 0.05 '.' 0.1 ' ' 1
```

```
###*** sample_cohort r2 = 0.052368 p = 0.000999, hiv_phenotype r2 = 0.008968 p = 0.000999

# vegan::adonis2(formula = unifrac.distance ~ metadata_ordered$hiv_phenotype + metadata_ordered$sample_cohort, data = ASV_table, permutations = 1000)
#                                 Df SumOfSqs       R2       F   Pr(>F)    
# metadata_ordered$hiv_phenotype   1   0.7160 0.008968 1.92038 0.000999 ***
# metadata_ordered$sample_cohort   2   4.1810 0.052368 5.60693 0.000999 ***
# Residual                       201  74.9415 0.938663                     
# Total                          204  79.8385 1.000000                     


### Extra metadata that have full n: Race, Ethnicity, age, sex, current_art_class_consolid2, tmp_smx_active
### Extra metadata that have <n: BMI, comorbidities (dm2_hx, hld_hx, htn_hx, cvd_hx, ckd_hx, cvd_dx [missing boston], dm2hx_dx, hldhx_dx, htnhx_dx, cvdhx_dx, ever_smoke, current_smoke, smoke_cat), school_level [uganda2 only], monthly_income[uganda2 only], current_art_class_consolid2, tmp_smx_active, days_on_art, sexual_orientation
### additional: smoking_years, fram_10yr_risk_lab, fram_10yr_risk_nonlab, mean_imt, total_plaques, any_plaques
metadata_ordered$age <- as.numeric(metadata_ordered$age)
metadata_ordered$BMI <- as.numeric(metadata_ordered$BMI)
metadata_ordered$days_on_art <- as.numeric(metadata_ordered$days_on_art)

### control for metadata with full n
covars_full_n <- c("Ethnicity", "age", "Race", "hiv_phenotype", "tmp_smx_active", "current_art_class_consolid2", "sample_cohort")
print(vegan::adonis2(as.formula(paste("unifrac.distance~metadata_ordered$", paste(covars_full_n, collapse = "+metadata_ordered$"), sep = "")), data=ASV_table, permutations=1000)) -> adon_full_n
```

```
## Permutation test for adonis under reduced model
## Terms added sequentially (first to last)
## Permutation: free
## Number of permutations: 1000
## 
## vegan::adonis2(formula = as.formula(paste("unifrac.distance~metadata_ordered$", paste(covars_full_n, collapse = "+metadata_ordered$"), sep = "")), data = ASV_table, permutations = 1000)
##                                               Df SumOfSqs      R2      F
## metadata_ordered$Ethnicity                     1    0.472 0.00591 1.2681
## metadata_ordered$age                           1    0.992 0.01242 2.6632
## metadata_ordered$Race                          5    3.028 0.03793 1.6265
## metadata_ordered$hiv_phenotype                 1    0.690 0.00864 1.8535
## metadata_ordered$tmp_smx_active                1    0.924 0.01157 2.4804
## metadata_ordered$current_art_class_consolid2   3    1.155 0.01446 1.0336
## metadata_ordered$sample_cohort                 2    1.832 0.02294 2.4599
## Residual                                     190   70.746 0.88612       
## Total                                        204   79.839 1.00000       
##                                                Pr(>F)    
## metadata_ordered$Ethnicity                   0.035964 *  
## metadata_ordered$age                         0.000999 ***
## metadata_ordered$Race                        0.000999 ***
## metadata_ordered$hiv_phenotype               0.000999 ***
## metadata_ordered$tmp_smx_active              0.000999 ***
## metadata_ordered$current_art_class_consolid2 0.306693    
## metadata_ordered$sample_cohort               0.000999 ***
## Residual                                                 
## Total                                                    
## ---
## Signif. codes:  0 '***' 0.001 '**' 0.01 '*' 0.05 '.' 0.1 ' ' 1
```

```
###*** sample_cohort r2 = 0.02294 p = 0.000999, hiv_phenotype r2 = 0.00864 p = 0.000999

#                                               Df SumOfSqs      R2      F   Pr(>F)    
# metadata_ordered$Ethnicity                     1    0.472 0.00591 1.2681 0.033966 *  
# metadata_ordered$age                           1    0.992 0.01242 2.6632 0.000999 ***
# metadata_ordered$Race                          5    3.028 0.03793 1.6265 0.000999 ***
# metadata_ordered$hiv_phenotype                 1    0.690 0.00864 1.8535 0.000999 ***
# metadata_ordered$tmp_smx_active                1    0.924 0.01157 2.4804 0.000999 ***
# metadata_ordered$current_art_class_consolid2   3    1.155 0.01446 1.0336 0.310689    
# metadata_ordered$sample_cohort                 2    1.832 0.02294 2.4599 0.000999 ***
# Residual                                     190   70.746 0.88612                    
# Total                                        204   79.839 1.00000                    

### remove samples with BMI/comorbidities/days_on_art is NA (n=205 -> n=190)
ps_gg_fp_f_prevalence_filter_2019_05_26_proportion_bcd <- ps_gg_fp_f_prevalence_filter_2019_05_26_proportion
metadata_ordered_bcd <- metadata_ordered[!is.na(metadata_ordered$dm2_hx) & !is.na(metadata_ordered$days_on_art) & !is.na(metadata_ordered$BMI),,drop=FALSE]
sample_data(ps_gg_fp_f_prevalence_filter_2019_05_26_proportion_bcd) <- metadata_ordered_bcd

### subset ASV table and distance matrix
ASV_table_bcd<-as.data.frame(otu_table(ps_gg_fp_f_prevalence_filter_2019_05_26_proportion_bcd))
all.equal(row.names(ASV_table_bcd), row.names(metadata_ordered_bcd))
```

```
## [1] TRUE
```

```
unifrac.distance_bcd <- unname(usedist::dist_subset(unifrac.distance, sample_names(ps_gg_fp_f_prevalence_filter_2019_05_26_proportion_bcd))) ### unname fixes error introduced by Desctools see https://github.com/joey711/phyloseq/issues/1457

### run adonis
covars_bcd <- c("BMI", "dm2_hx", "hld_hx", "htn_hx", "cvd_hx", "dm2hx_dx", "hldhx_dx", "cvdhx_dx", "days_on_art", covars_full_n)
print(vegan::adonis2(as.formula(paste("unifrac.distance_bcd~metadata_ordered_bcd$", paste(covars_bcd, collapse = "+metadata_ordered_bcd$"), sep = "")), data=ASV_table_bcd, permutations=1000)) -> adon_full_n_bcd
```

```
## Permutation test for adonis under reduced model
## Terms added sequentially (first to last)
## Permutation: free
## Number of permutations: 1000
## 
## vegan::adonis2(formula = as.formula(paste("unifrac.distance_bcd~metadata_ordered_bcd$", paste(covars_bcd, collapse = "+metadata_ordered_bcd$"), sep = "")), data = ASV_table_bcd, permutations = 1000)
##                                                   Df SumOfSqs      R2      F
## metadata_ordered_bcd$BMI                           1    0.448 0.00611 1.2177
## metadata_ordered_bcd$dm2_hx                        1    0.402 0.00547 1.0905
## metadata_ordered_bcd$hld_hx                        1    0.495 0.00675 1.3444
## metadata_ordered_bcd$htn_hx                        1    0.600 0.00817 1.6288
## metadata_ordered_bcd$cvd_hx                        1    0.474 0.00645 1.2859
## metadata_ordered_bcd$dm2hx_dx                      1    0.459 0.00625 1.2462
## metadata_ordered_bcd$hldhx_dx                      1    0.569 0.00776 1.5462
## metadata_ordered_bcd$cvdhx_dx                      1    0.610 0.00830 1.6552
## metadata_ordered_bcd$days_on_art                   1    0.617 0.00841 1.6762
## metadata_ordered_bcd$Ethnicity                     1    0.446 0.00607 1.2107
## metadata_ordered_bcd$age                           1    0.873 0.01190 2.3715
## metadata_ordered_bcd$Race                          4    2.179 0.02969 1.4794
## metadata_ordered_bcd$hiv_phenotype                 1    0.644 0.00877 1.7479
## metadata_ordered_bcd$tmp_smx_active                1    0.616 0.00839 1.6721
## metadata_ordered_bcd$current_art_class_consolid2   3    1.074 0.01464 0.9726
## metadata_ordered_bcd$sample_cohort                 2    1.391 0.01896 1.8891
## Residual                                         167   61.499 0.83790       
## Total                                            189   73.397 1.00000       
##                                                    Pr(>F)    
## metadata_ordered_bcd$BMI                         0.070929 .  
## metadata_ordered_bcd$dm2_hx                      0.203796    
## metadata_ordered_bcd$hld_hx                      0.021978 *  
## metadata_ordered_bcd$htn_hx                      0.000999 ***
## metadata_ordered_bcd$cvd_hx                      0.029970 *  
## metadata_ordered_bcd$dm2hx_dx                    0.049950 *  
## metadata_ordered_bcd$hldhx_dx                    0.004995 ** 
## metadata_ordered_bcd$cvdhx_dx                    0.000999 ***
## metadata_ordered_bcd$days_on_art                 0.001998 ** 
## metadata_ordered_bcd$Ethnicity                   0.061938 .  
## metadata_ordered_bcd$age                         0.000999 ***
## metadata_ordered_bcd$Race                        0.000999 ***
## metadata_ordered_bcd$hiv_phenotype               0.001998 ** 
## metadata_ordered_bcd$tmp_smx_active              0.001998 ** 
## metadata_ordered_bcd$current_art_class_consolid2 0.630370    
## metadata_ordered_bcd$sample_cohort               0.000999 ***
## Residual                                                     
## Total                                                        
## ---
## Signif. codes:  0 '***' 0.001 '**' 0.01 '*' 0.05 '.' 0.1 ' ' 1
```

```
###*** sample_cohort r2 = 0.018956 p = 0.000999, hiv_phenotype r2 = 0.008770 p = 0.000999

#                                                   Df SumOfSqs      R2      F   Pr(>F)    
# metadata_ordered_bcd$BMI                           1    0.448 0.00611 1.2177 0.054945 .  
# metadata_ordered_bcd$dm2_hx                        1    0.402 0.00547 1.0905 0.233766    
# metadata_ordered_bcd$hld_hx                        1    0.495 0.00675 1.3444 0.025974 *  
# metadata_ordered_bcd$htn_hx                        1    0.600 0.00817 1.6288 0.002997 ** 
# metadata_ordered_bcd$cvd_hx                        1    0.474 0.00645 1.2859 0.033966 *  
# metadata_ordered_bcd$dm2hx_dx                      1    0.459 0.00625 1.2462 0.046953 *  
# metadata_ordered_bcd$hldhx_dx                      1    0.569 0.00776 1.5462 0.001998 ** 
# metadata_ordered_bcd$cvdhx_dx                      1    0.610 0.00830 1.6552 0.000999 ***
# metadata_ordered_bcd$days_on_art                   1    0.617 0.00841 1.6762 0.001998 ** 
# metadata_ordered_bcd$Ethnicity                     1    0.446 0.00607 1.2107 0.067932 .  
# metadata_ordered_bcd$age                           1    0.873 0.01190 2.3715 0.000999 ***
# metadata_ordered_bcd$Race                          4    2.179 0.02969 1.4794 0.000999 ***
# metadata_ordered_bcd$hiv_phenotype                 1    0.644 0.00877 1.7479 0.000999 ***
# metadata_ordered_bcd$tmp_smx_active                1    0.616 0.00839 1.6721 0.001998 ** 
# metadata_ordered_bcd$current_art_class_consolid2   3    1.074 0.01464 0.9726 0.623377    
# metadata_ordered_bcd$sample_cohort                 2    1.391 0.01896 1.8891 0.000999 ***
# Residual                                         167   61.499 0.83790                    
# Total                                            189   73.397 1.00000                  


#COMPARISON 2 NEG vs UNTREAT
#Transform count data in the phyloseq object
ps_gg_fp_f_prevalence_filter_2019_05_26_proportion<-phyloseq::transform_sample_counts(ps_gg_fp_f_prevalence_filter_2019_05_26, function(x)(x/sum(x)))

#Select samples of interest and update phyloseq object 
metadata<-phyloseq::sample_data(ps_gg_fp_f_prevalence_filter_2019_05_26)
metadata<-metadata[metadata$hiv_phenotype%in%c("1_hiv_negative","4_unsuppressed"),,drop=F]
metadata<-as.data.frame(as.matrix(metadata[metadata$sexual_orientation!="MSM",,drop=F]))
metadata<-metadata[metadata$sample_cohort%in%c("botswana", "boston"),,drop=F]
metadata<-metadata[metadata$sex=="female",,drop=F]
phyloseq::sample_data(ps_gg_fp_f_prevalence_filter_2019_05_26_proportion)<-metadata

#Run PCoA on the phyloseq object
ordination<-phyloseq::ordinate(ps_gg_fp_f_prevalence_filter_2019_05_26_proportion, "PCoA", "unifrac")
```

```
## Warning in matrix(tree$edge[order(tree$edge[, 1]), ][, 2], byrow = TRUE, : data
## length [8987] is not a sub-multiple or multiple of the number of rows [4494]
```

```
ordination$values[1:2,]
```

```
##   Eigenvalues Relative_eig Broken_stick  Cumul_eig Cumul_br_stick
## 1    2.709784   0.06988037   0.05145820 0.06988037     0.05145820
## 2    2.090828   0.05391863   0.04155721 0.12379900     0.09301542
```

```
metadata_ordered<-metadata[row.names(ordination$vectors),,drop=FALSE]

all.equal(row.names(metadata_ordered), row.names(ordination$vectors))
```

```
## [1] TRUE
```

```
metadata_ordered$Unifrac1<-ordination$vectors[,1]
metadata_ordered$Unifrac2<-ordination$vectors[,2]

comparison2<-metadata_ordered
comparison2$Grouping<-as.factor(paste(comparison2$hiv_phenotype, comparison2$sample_cohort, sep="_"))

#Let's plot the data
us<-comparison2[comparison2$sample_cohort=="boston",,drop=F]
botswana<-comparison2[comparison2$sample_cohort=="botswana",,drop=F]

plot_us_comparison<-ggplot2::ggplot(data=us, aes(x=Unifrac1, y=Unifrac2))+geom_point(color="royalblue4", aes(alpha=hiv_phenotype), size=2, shape=16)+
  theme_bw()+stat_ellipse(color="royalblue4", aes(alpha=hiv_phenotype), size=1)+scale_alpha_manual(values=c(1,0.3))+ylim(c(-0.6, 0.6))+xlim(c(-0.5,0.5))+
  ggtitle("neg-naive us")+geom_point(data=us %>% group_by(hiv_phenotype) %>% summarise_at(vars(matches("UniFrac")), mean),size=5, color="royalblue4", aes(alpha=hiv_phenotype))
plot_botswana_comparison<-ggplot(data=botswana, aes(x=Unifrac1, y=Unifrac2))+geom_point(color="darkorange", aes(alpha=hiv_phenotype), size=2, shape=16)+
  theme_bw()+stat_ellipse(color="darkorange", aes(alpha=hiv_phenotype), size=1)+scale_alpha_manual(values=c(1,0.3))+ylim(c(-0.6, 0.6))+xlim(c(-0.5,0.5))+
  ggtitle("neg-naive botswana")+geom_point(data=botswana %>% group_by(hiv_phenotype) %>% summarise_at(vars(matches("UniFrac")), mean),size=5, color="darkorange", aes(alpha=hiv_phenotype))

ggsave("SupplementaryFigure1C_2_v1.pdf", grid.arrange(plot_us_comparison,plot_botswana_comparison, nrow=3, ncol=3), width=15, height=10)
```

```
#Adonis (n=102)
ASV_table<-as.data.frame(phyloseq::otu_table(ps_gg_fp_f_prevalence_filter_2019_05_26_proportion))
all.equal(row.names(ASV_table), row.names(metadata_ordered))
```

```
## [1] TRUE
```

```
unifrac.distance<-unname(phyloseq::UniFrac(ps_gg_fp_f_prevalence_filter_2019_05_26_proportion, weighted = FALSE)) ### unname fixes error introduced by Desctools see https://github.com/joey711/phyloseq/issues/1457
```

```
## Warning in matrix(tree$edge[order(tree$edge[, 1]), ][, 2], byrow = TRUE, : data
## length [8987] is not a sub-multiple or multiple of the number of rows [4494]
```

```
attributes(unifrac.distance)$Labels <- phyloseq::sample_names(ps_gg_fp_f_prevalence_filter_2019_05_26_proportion)
print(vegan::adonis2(unifrac.distance~metadata_ordered$hiv_phenotype, data=ASV_table, permutations=1000)) -> adon_hiv_phenotype
```

```
## Permutation test for adonis under reduced model
## Terms added sequentially (first to last)
## Permutation: free
## Number of permutations: 1000
## 
## vegan::adonis2(formula = unifrac.distance ~ metadata_ordered$hiv_phenotype, data = ASV_table, permutations = 1000)
##                                 Df SumOfSqs      R2      F Pr(>F)
## metadata_ordered$hiv_phenotype   1    0.407 0.01049 1.0606 0.2757
## Residual                       100   38.371 0.98951              
## Total                          101   38.777 1.00000
```

```
###*** r2 = 0.010495 p = 0.24276

# vegan::adonis2(formula = unifrac.distance ~ metadata_ordered$hiv_phenotype, data = ASV_table, permutations = 1000)
#                                 Df SumOfSqs       R2       F  Pr(>F)
# metadata_ordered$hiv_phenotype   1   0.4070 0.010495 1.06059 0.27173
# Residual                       100  38.3705 0.989505                
# Total                          101  38.7775 1.000000                

print(vegan::adonis2(unifrac.distance~metadata_ordered$sample_cohort, data=ASV_table, permutations=1000)) -> adon_cohort
```

```
## Permutation test for adonis under reduced model
## Terms added sequentially (first to last)
## Permutation: free
## Number of permutations: 1000
## 
## vegan::adonis2(formula = unifrac.distance ~ metadata_ordered$sample_cohort, data = ASV_table, permutations = 1000)
##                                 Df SumOfSqs      R2      F   Pr(>F)    
## metadata_ordered$sample_cohort   1    1.560 0.04022 4.1908 0.000999 ***
## Residual                       100   37.218 0.95978                    
## Total                          101   38.777 1.00000                    
## ---
## Signif. codes:  0 '***' 0.001 '**' 0.01 '*' 0.05 '.' 0.1 ' ' 1
```

```
###*** r2 = 0.040223 p = 0.000999

# vegan::adonis2(formula = unifrac.distance ~ metadata_ordered$sample_cohort, data = ASV_table, permutations = 1000)
#                                 Df SumOfSqs       R2       F   Pr(>F)    
# metadata_ordered$sample_cohort   1   1.5597 0.040223 4.19084 0.000999 ***
# Residual                       100  37.2177 0.959777                     
# Total                          101  38.7775 1.000000             

print(vegan::adonis2(unifrac.distance~metadata_ordered$hiv_phenotype+metadata_ordered$sample_cohort, data=ASV_table, permutations=1000)) -> adon_cohort_phenotype
```

```
## Permutation test for adonis under reduced model
## Terms added sequentially (first to last)
## Permutation: free
## Number of permutations: 1000
## 
## vegan::adonis2(formula = unifrac.distance ~ metadata_ordered$hiv_phenotype + metadata_ordered$sample_cohort, data = ASV_table, permutations = 1000)
##                                 Df SumOfSqs      R2      F   Pr(>F)    
## metadata_ordered$hiv_phenotype   1    0.407 0.01049 1.0948 0.197802    
## metadata_ordered$sample_cohort   1    1.572 0.04055 4.2300 0.000999 ***
## Residual                        99   36.798 0.94896                    
## Total                          101   38.777 1.00000                    
## ---
## Signif. codes:  0 '***' 0.001 '**' 0.01 '*' 0.05 '.' 0.1 ' ' 1
```

```
###*** sample_cohort r2 = 0.040547 p = 0.000999, hiv_phenotype r2 = 0.010495 p = 0.187812

# vegan::adonis2(formula = unifrac.distance ~ metadata_ordered$hiv_phenotype + metadata_ordered$sample_cohort, data = ASV_table, permutations = 1000)
#                                 Df SumOfSqs       R2       F   Pr(>F)    
# metadata_ordered$hiv_phenotype   1   0.4070 0.010495 1.09485 0.238761    
# metadata_ordered$sample_cohort   1   1.5723 0.040547 4.23002 0.000999 ***
# Residual                        99  36.7982 0.948959                     
# Total                          101  38.7775 1.000000          

### Extra metadata to add: (in addition to sample_cohort, hiv_phenotype)
### that have full n: Race, Ethnicity, age, sex
### reviewer requested: BMI, comorbidities (dm2_hx, hld_hx, htn_hx, cvd_hx, ckd_hx, cvd_dx [missing boston], dm2hx_dx, hldhx_dx, htnhx_dx, cvdhx_dx, ever_smoke, current_smoke, smoke_cat), school_level [uganda2 only], monthly_income[uganda2 only], sexual_orientation
### additional: smoking_years, fram_10yr_risk_lab, fram_10yr_risk_nonlab, mean_imt, total_plaques, any_plaques
metadata_ordered$age <- as.numeric(metadata_ordered$age)
metadata_ordered$BMI <- as.numeric(metadata_ordered$BMI)

### control for metadata with full n
covars_full_n <- c("Ethnicity", "age", "Race", "hiv_phenotype", "sample_cohort")
print(vegan::adonis2(as.formula(paste("unifrac.distance~metadata_ordered$", paste(covars_full_n, collapse = "+metadata_ordered$"), sep = "")), data=ASV_table, permutations=1000)) -> adon_full_n
```

```
## Permutation test for adonis under reduced model
## Terms added sequentially (first to last)
## Permutation: free
## Number of permutations: 1000
## 
## vegan::adonis2(formula = as.formula(paste("unifrac.distance~metadata_ordered$", paste(covars_full_n, collapse = "+metadata_ordered$"), sep = "")), data = ASV_table, permutations = 1000)
##                                 Df SumOfSqs      R2      F   Pr(>F)    
## metadata_ordered$Ethnicity       1    0.380 0.00980 1.0251 0.348651    
## metadata_ordered$age             1    0.528 0.01361 1.4246 0.014985 *  
## metadata_ordered$Race            4    2.241 0.05780 1.5121 0.000999 ***
## metadata_ordered$hiv_phenotype   1    0.376 0.00971 1.0156 0.413586    
## metadata_ordered$sample_cohort   1    0.788 0.02032 2.1262 0.000999 ***
## Residual                        93   34.464 0.88876                    
## Total                          101   38.777 1.00000                    
## ---
## Signif. codes:  0 '***' 0.001 '**' 0.01 '*' 0.05 '.' 0.1 ' ' 1
```

```
###*** sample_cohort r2 = 0.020320 p = 0.000999, hiv_phenotype r2 = 0.00971 p = 0.389610

#                                 Df SumOfSqs      R2      F   Pr(>F)    
# metadata_ordered$Ethnicity       1    0.380 0.00980 1.0251 0.367632    
# metadata_ordered$age             1    0.528 0.01361 1.4246 0.012987 *  
# metadata_ordered$Race            4    2.241 0.05780 1.5121 0.000999 ***
# metadata_ordered$hiv_phenotype   1    0.376 0.00971 1.0156 0.389610    
# metadata_ordered$sample_cohort   1    0.788 0.02032 2.1262 0.000999 ***
# Residual                        93   34.464 0.88876                    
# Total                          101   38.777 1.00000                    

### remove samples with BMI/comorbidities is NA (n=102 -> n=91)
ps_gg_fp_f_prevalence_filter_2019_05_26_proportion_bc <- ps_gg_fp_f_prevalence_filter_2019_05_26_proportion
metadata_ordered_bc <- metadata_ordered[!is.na(metadata_ordered$dm2_hx) & !is.na(metadata_ordered$BMI),,drop=FALSE]
sample_data(ps_gg_fp_f_prevalence_filter_2019_05_26_proportion_bc) <- metadata_ordered_bc

### subset ASV table and distance matrix
ASV_table_bc<-as.data.frame(phyloseq::otu_table(ps_gg_fp_f_prevalence_filter_2019_05_26_proportion_bc))
all.equal(row.names(ASV_table_bc), row.names(metadata_ordered_bc))
```

```
## [1] TRUE
```

```
unifrac.distance_bc <- unname(usedist::dist_subset(unifrac.distance, sample_names(ps_gg_fp_f_prevalence_filter_2019_05_26_proportion_bc))) ### unname fixes error introduced by Desctools see https://github.com/joey711/phyloseq/issues/1457

### run adonis
covars_bc <- c("BMI", "dm2_hx", "hld_hx", "htn_hx", "cvd_hx", "dm2hx_dx", "hldhx_dx", "cvdhx_dx", covars_full_n)
print(vegan::adonis2(as.formula(paste("unifrac.distance_bc~metadata_ordered_bc$", paste(covars_bc, collapse = "+metadata_ordered_bc$"), sep = "")), data=ASV_table_bc, permutations=1000)) -> adon_full_n_bc
```

```
## Permutation test for adonis under reduced model
## Terms added sequentially (first to last)
## Permutation: free
## Number of permutations: 1000
## 
## vegan::adonis2(formula = as.formula(paste("unifrac.distance_bc~metadata_ordered_bc$", paste(covars_bc, collapse = "+metadata_ordered_bc$"), sep = "")), data = ASV_table_bc, permutations = 1000)
##                                   Df SumOfSqs      R2      F   Pr(>F)    
## metadata_ordered_bc$BMI            1    0.334 0.00978 0.9144 0.706294    
## metadata_ordered_bc$dm2_hx         1    0.423 0.01238 1.1583 0.124875    
## metadata_ordered_bc$hld_hx         1    0.351 0.01028 0.9611 0.544456    
## metadata_ordered_bc$htn_hx         1    0.409 0.01198 1.1203 0.164835    
## metadata_ordered_bc$cvd_hx         1    0.516 0.01510 1.4127 0.011988 *  
## metadata_ordered_bc$dm2hx_dx       1    0.430 0.01259 1.1772 0.107892    
## metadata_ordered_bc$hldhx_dx       1    0.567 0.01660 1.5525 0.004995 ** 
## metadata_ordered_bc$cvdhx_dx       1    0.372 0.01088 1.0174 0.380619    
## metadata_ordered_bc$Ethnicity      1    0.354 0.01037 0.9701 0.510490    
## metadata_ordered_bc$age            1    0.462 0.01352 1.2649 0.059940 .  
## metadata_ordered_bc$Race           4    2.001 0.05856 1.3693 0.000999 ***
## metadata_ordered_bc$hiv_phenotype  1    0.357 0.01044 0.9761 0.493506    
## metadata_ordered_bc$sample_cohort  1    0.560 0.01638 1.5316 0.007992 ** 
## Residual                          74   27.037 0.79116                    
## Total                             90   34.174 1.00000                    
## ---
## Signif. codes:  0 '***' 0.001 '**' 0.01 '*' 0.05 '.' 0.1 ' ' 1
```

```
###*** sample_cohort r2 = 0.016375 p = 0.004995, hiv_phenotype r2 = 0.010436 p = 0.513487

#                                   Df SumOfSqs      R2      F   Pr(>F)    
# metadata_ordered_bc$BMI            1    0.334 0.00978 0.9144 0.718282    
# metadata_ordered_bc$dm2_hx         1    0.423 0.01238 1.1583 0.115884    
# metadata_ordered_bc$hld_hx         1    0.351 0.01028 0.9611 0.521479    
# metadata_ordered_bc$htn_hx         1    0.409 0.01198 1.1203 0.166833    
# metadata_ordered_bc$cvd_hx         1    0.516 0.01510 1.4127 0.014985 *  
# metadata_ordered_bc$dm2hx_dx       1    0.430 0.01259 1.1772 0.091908 .  
# metadata_ordered_bc$hldhx_dx       1    0.567 0.01660 1.5525 0.000999 ***
# metadata_ordered_bc$cvdhx_dx       1    0.372 0.01088 1.0174 0.374625    
# metadata_ordered_bc$Ethnicity      1    0.354 0.01037 0.9701 0.519481    
# metadata_ordered_bc$age            1    0.462 0.01352 1.2649 0.048951 *  
# metadata_ordered_bc$Race           4    2.001 0.05856 1.3693 0.000999 ***
# metadata_ordered_bc$hiv_phenotype  1    0.357 0.01044 0.9761 0.513487    
# metadata_ordered_bc$sample_cohort  1    0.560 0.01638 1.5316 0.004995 ** 
# Residual                          74   27.037 0.79116                    
# Total                             90   34.174 1.00000                   

#--------------------------------------------------------------------------------------------------------------
```

```
#FIGURE D (Supplemental Figure 3B)
#--------------------------------------------------------------------------------------------------------------
#Prepare OTU table and taxonomy files out from the phyloseq object:
OTU_table<-as.data.frame(phyloseq::otu_table(ps_gg_fp_f_prevalence_filter_2019_05_26))
Taxonomy<-as.data.frame(phyloseq::tax_table(ps_gg_fp_f_prevalence_filter_2019_05_26))
metadata<-as.data.frame(phyloseq::sample_data(ps_gg_fp_f_prevalence_filter_2019_05_26))

#Find the sample with the smallest number of reads. It will be the value used to normalize all data so all samples will be comparable among them:
rowSums(OTU_table)
```

```
## 105574.boston1.0139.2014.12.08 108777.boston1.0140.2014.12.08 
##                          72609                          64328 
## 112993.boston1.0141.2014.12.08 123656.boston1.0005.2014.12.08 
##                          79920                          36479 
## 143200.boston1.0006.2014.12.08 153724.boston1.0007.2014.12.08 
##                          81427                          85980 
## 165642.boston1.0008.2014.12.08 194317.boston1.0010.2014.12.08 
##                          98119                          53263 
## 196203.boston1.0011.2014.12.08 205120.boston1.0013.2014.12.08 
##                          36651                          59443 
## 211774.boston1.0014.2014.12.08 228437.boston1.0017.2014.12.08 
##                          57586                          52936 
## 229969.boston1.0018.2014.12.08 237983.boston1.0019.2014.12.08 
##                          62357                          37574 
## 258085.boston1.0142.2014.12.08 273479.boston1.0143.2014.12.08 
##                          59728                         102691 
## 315504.boston1.0028.2014.12.08 330183.boston1.0144.2014.12.08 
##                          21399                          68760 
## 337016.boston1.0030.2014.12.08 365685.boston1.0032.2014.12.08 
##                          43655                          41183 
## 386576.boston1.0035.2014.12.08 389876.boston1.0036.2014.12.08 
##                          36454                          66739 
## 410644.boston1.0125.2014.12.08 410932.boston1.0039.2014.12.08 
##                          73269                          27289 
## 413736.boston1.0126.2014.12.08 427838.boston1.0127.2014.12.08 
##                          39912                          66847 
## 453548.boston1.0045.2014.12.08 460380.boston1.0046.2014.12.08 
##                          34382                          31329 
## 473516.boston1.0047.2014.12.08 479693.boston1.0048.2014.12.08 
##                          46039                          18765 
## 485548.boston1.0049.2014.12.08 498553.boston1.0050.2014.12.08 
##                          35582                          56405 
## 505402.boston1.0051.2014.12.08 516980.boston1.0130.2014.12.08 
##                          59815                          14678 
## 522458.boston1.0132.2014.12.08 526318.boston1.0133.2014.12.08 
##                          68609                          27811 
## 527968.boston1.0057.2014.12.08 529516.boston1.0136.2014.12.08 
##                          26204                          20681 
## 533586.boston1.0059.2014.12.08 534694.boston1.0060.2014.12.08 
##                          57983                          51661 
## 604772.boston1.0071.2014.12.08 614225.boston1.0073.2014.12.08 
##                          52199                          47445 
## 615167.boston1.0074.2014.12.08 616147.boston1.0075.2014.12.08 
##                          37085                          50711 
## 653425.boston1.0077.2014.12.08 666207.boston1.0079.2014.12.08 
##                          42115                          70102 
## 694413.boston1.0080.2014.12.08 708968.boston1.0083.2014.12.08 
##                          44825                          28721 
## 734962.boston1.0085.2014.12.08 745577.boston1.0086.2014.12.08 
##                          43153                          75694 
## 758572.boston1.0088.2014.12.08 775609.boston1.0091.2014.12.08 
##                          63237                          64948 
## 813341.boston1.0095.2014.12.08 819622.boston1.0096.2014.12.08 
##                          74295                          34279 
## 842279.boston1.0097.2014.12.08 847041.boston1.0098.2014.12.08 
##                          61932                          59051 
## 862898.boston1.0103.2014.12.08 874612.boston1.0105.2014.12.08 
##                          38913                          21417 
## 880160.boston1.0106.2014.12.08 899025.boston1.0107.2014.12.08 
##                          61338                          51550 
## 900158.boston1.0108.2014.12.08 911594.boston1.0109.2014.12.08 
##                          18175                          54168 
## 923358.boston1.0111.2014.12.08 950965.boston1.0116.2014.12.08 
##                          34979                          66238 
## 953586.boston1.0117.2014.12.08 958793.boston1.0118.2014.12.08 
##                          54430                          66440 
## 966971.boston1.0120.2014.12.08 970489.boston1.0121.2014.12.08 
##                          44109                          48095 
## 995725.boston1.0123.2014.12.08  529863.boston.0165.2017.04.06 
##                          58954                          42388 
##  608647.boston.0072.2017.04.06    686039.0040.0323.2017.04.06 
##                          31028                         374932 
##   WT24922.0093.0468.2017.04.06    102438.0086.0363.2017.03.15 
##                         107819                          25113 
##    106085.0054.0333.2017.03.15    122897.0017.0307.2017.03.15 
##                          19135                          80414 
##    129226.0089.0367.2017.03.15    136376.0013.0302.2017.03.15 
##                          33236                          27232 
##    148342.0027.0314.2017.03.15    157072.0043.0244.2017.03.15 
##                          45438                          22883 
##    175067.0033.0231.2017.03.15    181090.0091.0369.2017.03.15 
##                          15936                          22823 
##    189326.0070.0343.2017.03.15    191447.0008.0296.2017.03.15 
##                          61451                          62556 
##    207295.0010.0298.2017.03.15    211578.0032.0230.2017.03.15 
##                          30235                          23193 
##    228516.0076.0350.2017.03.15    236532.0078.0379.2017.03.15 
##                          39086                          66657 
##    238426.0046.0247.2017.03.15    249768.0083.0359.2017.03.15 
##                          15578                          65769 
##    251073.0025.0221.2017.03.15    285803.0064.0338.2017.03.15 
##                          27143                          63788 
##    293340.0035.0233.2017.03.15    305385.0051.0330.2017.03.15 
##                          24885                          20588 
##    310817.0006.0293.2017.03.15    331904.0098.0430.2017.03.15 
##                          32927                          35529 
##    347964.0061.0336.2017.03.15    350103.0012.0301.2017.03.15 
##                          66932                          34897 
##    380272.0044.0245.2017.03.15    387879.0030.0228.2017.03.15 
##                          55368                          51686 
##    400609.0058.0262.2017.03.15    408044.0052.0331.2017.03.15 
##                          24415                          57931 
##    419034.0081.0382.2017.03.15    432158.0045.0246.2017.03.15 
##                          57230                          37797 
##    442916.0037.0319.2017.03.15    444991.0047.0327.2017.03.15 
##                          24253                          17042 
##    460929.0041.0324.2017.03.15    466105.0018.0377.2017.03.15 
##                          18796                          48350 
##    470588.0066.0340.2017.03.15  481066.boston.0168.2017.03.15 
##                          52619                          14233 
##    487268.0057.0261.2017.03.15    498229.0036.0318.2017.03.15 
##                          17799                          38925 
##    498554.0062.0337.2017.03.15    502743.0038.0320.2017.03.15 
##                          47910                          29154 
##    515591.0056.0334.2017.03.15    516035.0020.0310.2017.03.15 
##                          16026                          41843 
##    521471.0067.0341.2017.03.15    524541.0024.0313.2017.03.15 
##                          37753                          34986 
##    560575.0080.0381.2017.03.15    564855.0053.0332.2017.03.15 
##                          76936                          32030 
##    565723.0005.0292.2017.03.15    588311.0072.0346.2017.03.15 
##                          33565                          61200 
##    596527.0095.0374.2017.03.15    614631.0065.0339.2017.03.15 
##                          49031                          23279 
##    629358.0009.0297.2017.03.15    637837.0021.0312.2017.03.15 
##                          63706                          33770 
##    651433.0099.0431.2017.03.15    658217.0055.0258.2017.03.15 
##                          27060                          34249 
##    711750.0082.0383.2017.03.15    721154.0060.0335.2017.03.15 
##                          83786                          16367 
##    721729.0088.0366.2017.03.15    722188.0063.0268.2017.03.15 
##                          23928                          26981 
##    725896.0022.0218.2017.03.15    732425.0039.0378.2017.03.15 
##                          18124                          48694 
##    735345.0071.0345.2017.03.15    768392.0096.0375.2017.03.15 
##                          46984                          41304 
##    805457.0094.0373.2017.03.15    805641.0015.0305.2017.03.15 
##                          65857                          38997 
##    839338.0011.0300.2017.03.15    848334.0077.0351.2017.03.15 
##                          70395                          45711 
##    885614.0048.0328.2017.03.15    888751.0019.0309.2017.03.15 
##                          18203                          47832 
##    893231.0074.0348.2017.03.15    902901.0029.0226.2017.03.15 
##                          28255                          18740 
##    905350.0085.0362.2017.03.15    908782.0092.0371.2017.03.15 
##                          42388                          28907 
##  909824.boston.0174.2017.03.15    910641.0031.0316.2017.03.15 
##                          12820                          38990 
##    916034.0034.0232.2017.03.15    919901.0026.0222.2017.03.15 
##                          21405                          19714 
##    940622.0075.0349.2017.03.15    959714.0004.0291.2017.03.15 
##                          42914                          30169 
##    959734.0090.0368.2017.03.15    968359.0087.0365.2017.03.15 
##                          37955                          33934 
##    968902.0073.0347.2017.03.15    972684.0028.0224.2017.03.15 
##                          34542                          22469 
##    975240.0016.0306.2017.03.15    976183.0050.0329.2017.03.15 
##                          30867                          31908 
##    979196.0003.0290.2017.03.15    989517.0068.0274.2017.03.15 
##                          34682                          45375 
##   WT09782.0159.0121.2017.02.01   WT15101.0174.0106.2017.02.01 
##                          52918                          17604 
##   WT42336.0160.0122.2017.02.01   WT44778.0158.0120.2017.02.01 
##                          17747                          80361 
##   XE17833.0092.0100.2017.02.01   XE22903.0043.0125.2017.02.01 
##                          10751                          92243 
##   XE28163.0194.0127.2017.02.01   WM26348.0100.0113.2017.01.11 
##                          77437                          52751 
##   WM26354.0139.0315.2017.01.11   WQ64001.0145.0143.2017.01.11 
##                          45678                          39753 
##   WS20813.0155.0153.2017.01.11   WS21384.0094.0102.2017.01.11 
##                          29351                          12873 
##   WS21401.0047.0283.2017.01.11   WS21556.0141.0317.2017.01.11 
##                          65243                          57521 
##   WS21562.0055.0438.2017.01.11   WS21578.0125.0134.2017.01.11 
##                          13616                          46331 
##   WS21584.0066.0321.2017.01.11   WS22205.0097.0110.2017.01.11 
##                          28249                          65967 
##   WS74808.0165.0347.2017.01.11   WS74858.0098.0111.2017.01.11 
##                          32886                          18675 
##   WS75418.0032.0279.2017.01.11   WS76117.0102.0115.2017.01.11 
##                          66488                          78753 
##   WS76840.0117.0126.2017.01.11   WS77050.0026.0276.2017.01.11 
##                          39459                          15102 
##   WT02695.0166.0104.2017.01.11   WT02712.0078.0323.2017.01.11 
##                          20141                          38952 
##   WT05558.0190.0378.2017.01.11   WT05564.0116.0125.2017.01.11 
##                          17973                          33463 
##   WT06263.0127.0135.2017.01.11   WT07439.0058.0440.2017.01.11 
##                          35147                          43263 
##   WT08061.0090.0098.2017.01.11   WT09760.0053.0437.2017.01.11 
##                          28736                          34666 
##   WT09798.0057.0285.2017.01.11   WT10373.0080.0325.2017.01.11 
##                          78499                          37600 
##   WT10389.0036.0270.2017.01.11   WT10395.0035.0269.2017.01.11 
##                          29095                          43853 
##   WT12565.0189.0377.2017.01.11   WT14129.0153.0151.2017.01.11 
##                          26219                          58444 
##   WT14135.0123.0132.2017.01.11   WT14818.0111.0120.2017.01.11 
##                          42345                          33448 
##   WT15084.0046.0282.2017.01.11   WT15117.0157.0155.2017.01.11 
##                         122971                          41809 
##   WT15123.0164.0346.2017.01.11   WT23223.0144.0320.2017.01.11 
##                          37517                          39399 
##   WT23273.0101.0114.2017.01.11   WT24900.0152.0150.2017.01.11 
##                          32170                          25802 
##   WT24916.0044.0281.2017.01.11   WT24944.0124.0159.2017.01.11 
##                          74046                          36504 
##   WT24950.0033.0267.2017.01.11   WT24966.0142.0318.2017.01.11 
##                          54801                          37339 
##   WT27396.0121.0130.2017.01.11   WT27407.0156.0154.2017.01.11 
##                          30349                          27423 
##   WT27441.0129.0137.2017.01.11   WT30335.0134.0140.2017.01.11 
##                          25366                          76057 
##   WT30357.0049.0274.2017.01.11   WT30818.0107.0118.2017.01.11 
##                          35457                          26163 
##   WT30824.0147.0145.2017.01.11   WT30830.0052.0275.2017.01.11 
##                          45066                          41492 
##   WT34353.0120.0129.2017.01.11   WT40033.0133.0139.2017.01.11 
##                          40414                          22514 
##   WT40049.0148.0146.2017.01.11   WT40083.0038.0271.2017.01.11 
##                          60368                          23791 
##   WT40516.0122.0131.2017.01.11   WT41865.0089.0097.2017.01.11 
##                          69537                          24316 
##   WT41910.0095.0108.2017.01.11   WT42069.0154.0152.2017.01.11 
##                          43202                          25172 
##   WT43352.0087.0447.2017.01.11   WT43368.0118.0127.2017.01.11 
##                          15820                          50208 
##   WT43374.0048.0273.2017.01.11   WT44245.0081.0337.2017.01.11 
##                          57836                          54390 
##   WT44601.0082.0445.2017.01.11   WT44762.0130.0138.2017.01.11 
##                          41145                         118478 
##   WT47297.0173.0105.2017.01.11   WT48435.0099.0112.2017.01.11 
##                          30740                          39063 
##   WT48441.0112.0121.2017.01.11   WT48457.0060.0441.2017.01.11 
##                          31526                          41539 
##   WT48491.0146.0144.2017.01.11   WT48502.0106.0117.2017.01.11 
##                          28055                          63937 
##   WT50842.0187.0376.2017.01.11   WT50858.0128.0136.2017.01.11 
##                          20957                          36154 
##   WY74094.0079.0324.2017.01.11   WY74105.0151.0149.2017.01.11 
##                          55072                          67120 
##   WY74777.0073.0444.2017.01.11   WY74799.0176.0369.2017.01.11 
##                          45932                          27435 
##   WY75915.0114.0123.2017.01.11   WY75959.0191.0379.2017.01.11 
##                          27184                          28579 
##   WY75971.0062.0443.2017.01.11   WY76486.0162.0344.2017.01.11 
##                          49071                          52240 
##   WY78078.0161.0343.2017.01.11   WY78084.0061.0442.2017.01.11 
##                          29088                          15433 
##   WY78567.0119.0128.2017.01.11   WY79216.0040.0272.2017.01.11 
##                          21515                          42024 
##   WY79222.0056.0439.2017.01.11   WY79266.0113.0157.2017.01.11 
##                          27261                          55014 
##   WY79272.0177.0370.2017.01.11   WY80257.0027.0277.2017.01.11 
##                          31091                          65700 
##   WY80324.0137.0287.2017.01.11   WY81156.0104.0116.2017.01.11 
##                          37886                          41525 
##   WY81162.0169.0362.2017.01.11   WY81184.0045.0436.2017.01.11 
##                          65619                          23675 
##   XE13926.0025.0214.2017.01.11   XE13948.0192.0380.2017.01.11 
##                          25666                          43145 
##   XE13960.0150.0148.2017.01.11   XE15596.0149.0147.2017.01.11 
##                          34682                          51049 
##   XE17300.0083.0339.2017.01.11   XE21060.0015.0266.2017.01.11 
##                          60567                          39820 
##   XE21076.0182.0372.2017.01.11   XE21082.0186.0375.2017.01.11 
##                          37296                          20236 
##   XE21098.0115.0158.2017.01.11   XE22892.0171.0364.2017.01.11 
##                          42113                          50713 
##   XE22953.0096.0109.2017.01.11   XE27236.0054.0284.2017.01.11 
##                          52070                         135285 
##   XE27414.0059.0286.2017.01.11   XE28202.0028.0278.2017.01.11 
##                          51840                          44456 
##   XE29167.0135.0141.2017.01.11   XE30225.0020.0212.2017.01.11 
##                          64259                          24576 
##   XE31552.0011.0211.2017.01.11   XE33411.0143.0319.2017.01.11 
##                          20444                          38078 
##   XE36257.0175.0156.2017.01.11   XE36952.0091.0099.2017.01.11 
##                          48972                          11715 
##   XE36996.0009.0210.2017.01.11   XE38944.0167.0360.2017.01.11 
##                          38916                          26375 
##   XE38950.0140.0316.2017.01.11   XE38966.0034.0268.2017.01.11 
##                          38755                          55702 
##   XE38972.0178.0371.2017.01.11   XE38988.0110.0119.2017.01.11 
##                          35384                          32939 
##   XE38994.0185.0374.2017.01.11   XE40684.0024.0213.2017.01.11 
##                          21707                          25565 
##   XE40690.0172.0365.2017.01.11   XE40701.0163.0103.2017.01.11 
##                          46970                          46782 
##   XE40717.0010.0265.2017.01.11   XE40745.0006.0264.2017.01.11 
##                          44631                          59807 
##   XE40751.0088.0096.2017.01.11   XE41305.0193.0381.2017.01.11 
##                          17438                          27692 
##   XE41311.0138.0314.2017.01.11   XE41327.0085.0446.2017.01.11 
##                          63493                          13408 
##   XE41333.0136.0142.2017.01.11   XE41349.0168.0361.2017.01.11 
##                          44701                          26835 
##   XE41561.0037.0280.2017.01.11   XE41577.0170.0363.2017.01.11 
##                          44727                          31898 
##   XE41583.0183.0373.2017.01.11   WS19294.0064.0471.2016.11.13 
##                          26328                          58457 
##   WS20829.0126.0349.2016.11.13   WS20835.0007.0390.2016.11.13 
##                         136145                          35250 
##   WS21390.0076.0299.2016.11.13   WS21540.0031.0414.2016.11.13 
##                         144074                          52735 
##   WT02689.0077.0300.2016.11.13   WT02728.0109.0332.2016.11.13 
##                          70581                          83182 
##   WT04693.0016.0246.2016.11.13   WT04704.0071.0294.2016.11.13 
##                          60837                         133384 
##   WT07417.0184.0222.2016.11.13   WT08055.0075.0298.2016.11.13 
##                         122883                         116845 
##   WT09332.0070.0293.2016.11.13   WT09776.0074.0297.2016.11.13 
##                         159041                         156778 
##   WT10406.0003.0386.2016.11.13   WT11111.0023.0406.2016.11.13 
##                          24724                         196412 
##   WT12559.0072.0295.2016.11.13   WT14141.0002.0385.2016.11.13 
##                         192888                          57645 
##   WT15090.0180.0218.2016.11.13   WT23295.0022.0251.2016.11.13 
##                         106854                          68044 
##   WT27518.0050.0457.2016.11.13   WT27607.0039.0422.2016.11.13 
##                          32576                          90830 
##   WT30341.0012.0243.2016.11.13   WT30868.0042.0449.2016.11.13 
##                          55241                          74533 
##   WT30880.0069.0292.2016.11.13   WT34347.0181.0219.2016.11.13 
##                          81481                         103299 
##   WT37711.0068.0291.2016.11.13   WT42192.0131.0354.2016.11.13 
##                         146323                          52000 
##   WT47308.0004.0387.2016.11.13   WY76492.0067.0290.2016.11.13 
##                          54624                         132789 
##   WY78062.0019.0248.2016.11.13   WY79238.0108.0331.2016.11.13 
##                          67007                          37816 
##   WY79244.0105.0328.2016.11.13   WY80318.0103.0326.2016.11.13 
##                          16417                          53131 
##   WY82116.0188.0226.2016.11.13   XE15574.0065.0288.2016.11.13 
##                          79589                         203487 
##   XE17922.0179.0217.2016.11.13   XE17938.0014.0245.2016.11.13 
##                          93388                          67436 
##   XE18326.0084.0307.2016.11.13   XE21109.0018.0247.2016.11.13 
##                          81864                         105606 
##   XE28157.0013.0244.2016.11.13   XE28274.0051.0458.2016.11.13 
##                          94532                          24318 
##   XE29173.0008.0240.2016.11.13   XE29812.0132.0355.2016.11.13 
##                          82688                         143895 
##   XE33372.0021.0427.2016.11.13   XE36174.0063.0470.2016.11.13 
##                          74927                          92285 
##   XE37001.0030.0413.2016.11.13   XE39009.0029.0412.2016.11.13 
##                          80217                          48882 
##   XE39532.0017.0400.2016.11.13   XE39554.0041.0448.2016.11.13 
##                         205766                          65169 
##   XE40723.0001.0384.2016.11.13   XE40739.0086.0309.2016.11.13 
##                          61118                          34672 
##   XE41599.0005.0388.2016.11.13   MBA4060.0077.0266.2016.03.20 
##                          56531                          11517 
##   MBA1007.0092.0281.2016.03.11   MBA1037.0129.0437.2016.03.11 
##                          16176                          14385 
##   MBA1083.0088.0367.2016.03.11   MBA1141.0108.0297.2016.03.11 
##                          11423                          11454 
##   MBA1166.0047.0236.2016.03.11   MBA1172.0017.0444.2016.03.11 
##                          11848                          11809 
##   MBA1261.0125.0433.2016.03.11   MBA1327.0122.0368.2016.03.11 
##                          13925                          20343 
##   MBA1447.0019.0469.2016.03.11   MBA4044.0024.0470.2016.03.11 
##                          45329                          42889 
##   MBA4049.0043.0416.2016.03.11   MBA4051.0001.0446.2016.03.11 
##                          13037                          12351 
##   MBA4056.0119.0308.2016.03.11   MBA4065.0152.0341.2016.03.11 
##                          12105                          12002 
##   MBA4077.0040.0378.2016.03.11   MBA4085.0075.0264.2016.03.11 
##                          27897                          14923 
##   MBA4088.0082.0271.2016.03.11   MBA4091.0085.0429.2016.03.11 
##                           9947                          14136 
##   MBA4120.0076.0265.2016.03.11   MBA4121.0046.0235.2016.03.11 
##                          13922                          12858 
##   MBA4129.0087.0468.2016.03.11   MBA4130.0148.0337.2016.03.11 
##                          40274                          16263 
##   MBA4134.0120.0363.2016.03.11   MBA4139.0164.0353.2016.03.11 
##                          25631                          11278 
##  233202.Boston.0164.2016.02.14  930024.Boston.0114.2016.02.14 
##                          24841                          27023 
##   MBA1003.0131.0320.2016.02.14   MBA1021.0035.0460.2016.02.14 
##                          49506                          21557 
##   MBA1030.0014.0205.2016.02.14   MBA1033.0055.0244.2016.02.14 
##                          60325                          23191 
##   MBA1035.0102.0426.2016.02.14   MBA1041.0053.0242.2016.02.14 
##                          40213                          14230 
##   MBA1052.0009.0451.2016.02.14   MBA1071.0011.0202.2016.02.14 
##                          14859                          82865 
##   MBA1074.0058.0247.2016.02.14   MBA1082.0023.0463.2016.02.14 
##                          13002                          28556 
##   MBA1084.0114.0303.2016.02.14   MBA1090.0003.0194.2016.02.14 
##                          11543                         133259 
##   MBA1095.0090.0279.2016.02.14   MBA1096.0060.0249.2016.02.14 
##                          23430                          19672 
##   MBA1099.0066.0255.2016.02.14   MBA1100.0027.0386.2016.02.14 
##                          19574                          26058 
##   MBA1101.0149.0338.2016.02.14   MBA1103.0026.0385.2016.02.14 
##                          19728                          24858 
##   MBA1111.0139.0328.2016.02.14   MBA1133.0041.0453.2016.02.14 
##                          17707                          23140 
##   MBA1135.0140.0329.2016.02.14   MBA1139.0018.0445.2016.02.14 
##                           9599                          13882 
##   MBA1143.0105.0294.2016.02.14   MBA1151.0107.0296.2016.02.14 
##                          19881                          13159 
##   MBA1159.0165.0354.2016.02.14   MBA1163.0167.0356.2016.02.14 
##                          30857                          33492 
##   MBA1181.0126.0315.2016.02.14   MBA1187.0062.0251.2016.02.14 
##                          43998                          15695 
##   MBA1190.0048.0237.2016.02.14   MBA1193.0132.0321.2016.02.14 
##                          53414                          15292 
##   MBA1199.0069.0258.2016.02.14   MBA1202.0094.0456.2016.02.14 
##                          21096                          14588 
##   MBA1209.0101.0290.2016.02.14   MBA1211.0136.0325.2016.02.14 
##                          10734                          20052 
##   MBA1218.0030.0425.2016.02.14   MBA1230.0093.0282.2016.02.14 
##                          19204                          19109 
##   MBA1237.0170.0359.2016.02.14   MBA1241.0022.0419.2016.02.14 
##                          25519                          30836 
##   MBA1248.0070.0259.2016.02.14   MBA1251.0033.0467.2016.02.14 
##                          16468                          26599 
##   MBA1260.0158.0347.2016.02.14   MBA1267.0038.0227.2016.02.14 
##                          39840                          17977 
##   MBA1270.0042.0231.2016.02.14   MBA1271.0116.0462.2016.02.14 
##                          16729                          22420 
##   MBA1279.0162.0461.2016.02.14   MBA1307.0029.0372.2016.02.14 
##                          48196                          13686 
##   MBA1312.0074.0263.2016.02.14   MBA1317.0067.0256.2016.02.14 
##                          16407                          16233 
##   MBA1330.0050.0239.2016.02.14   MBA1341.0160.0349.2016.02.14 
##                          12379                          21517 
##   MBA1344.0169.0358.2016.02.14   MBA1355.0037.0458.2016.02.14 
##                          38110                          38263 
##   MBA1363.0123.0312.2016.02.14   MBA1365.0171.0360.2016.02.14 
##                          11750                          19399 
##   MBA1370.0034.0465.2016.02.14   MBA1375.0061.0250.2016.02.14 
##                          32525                          24576 
##   MBA1382.0159.0348.2016.02.14   MBA1385.0124.0313.2016.02.14 
##                          23025                          37460 
##   MBA1392.0134.0323.2016.02.14   MBA1399.0147.0336.2016.02.14 
##                          41620                          26657 
##   MBA1410.0130.0379.2016.02.14   MBA1437.0063.0252.2016.02.14 
##                          19712                          13941 
##   MBA1460.0106.0295.2016.02.14   MBA1480.0166.0355.2016.02.14 
##                           9819                          34300 
##   MBA1486.0115.0304.2016.02.14   MBA1488.0072.0452.2016.02.14 
##                          22751                          13626 
##   MBA1511.0163.0352.2016.02.14   MBA1515.0153.0342.2016.02.14 
##                          21490                          24838 
##   MBA4041.0044.0449.2016.02.14   MBA4043.0059.0248.2016.02.14 
##                          10254                          13060 
##   MBA4045.0157.0346.2016.02.14   MBA4047.0012.0203.2016.02.14 
##                          33741                          48792 
##   MBA4050.0028.0434.2016.02.14   MBA4052.0118.0457.2016.02.14 
##                          14519                          35830 
##   MBA4053.0057.0246.2016.02.14   MBA4057.0133.0322.2016.02.14 
##                          25496                           9954 
##   MBA4061.0010.0201.2016.02.14   MBA4062.0079.0393.2016.02.14 
##                         104643                          11876 
##   MBA4063.0100.0289.2016.02.14   MBA4066.0142.0430.2016.02.14 
##                          16644                          12023 
##   MBA4067.0104.0293.2016.02.14   MBA4068.0007.0198.2016.02.14 
##                          22257                          49188 
##   MBA4069.0004.0195.2016.02.14   MBA4070.0086.0478.2016.02.14 
##                          90587                          41192 
##   MBA4072.0161.0350.2016.02.14   MBA4074.0078.0267.2016.02.14 
##                          27345                          13679 
##   MBA4076.0016.0207.2016.02.14   MBA4078.0151.0340.2016.02.14 
##                          47724                          35591 
##   MBA4080.0065.0254.2016.02.14   MBA4081.0052.0241.2016.02.14 
##                          23350                          20586 
##   MBA4082.0032.0221.2016.02.14   MBA4086.0110.0299.2016.02.14 
##                          15687                          18709 
##   MBA4087.0155.0344.2016.02.14   MBA4089.0141.0330.2016.02.14 
##                          35300                          21650 
##   MBA4092.0008.0199.2016.02.14   MBA4095.0002.0193.2016.02.14 
##                          69046                         138863 
##   MBA4096.0145.0334.2016.02.14   MBA4102.0154.0343.2016.02.14 
##                          18861                          23941 
##   MBA4103.0128.0317.2016.02.14   MBA4106.0168.0357.2016.02.14 
##                          13480                          33202 
##   MBA4107.0138.0327.2016.02.14   MBA4108.0099.0288.2016.02.14 
##                          12705                          14653 
##   MBA4109.0073.0262.2016.02.14   MBA4111.0036.0459.2016.02.14 
##                          10720                          38086 
##   MBA4112.0005.0196.2016.02.14   MBA4113.0137.0396.2016.02.14 
##                          59252                          13144 
##   MBA4114.0064.0253.2016.02.14   MBA4115.0143.0332.2016.02.14 
##                          13795                           9242 
##   MBA4118.0031.0466.2016.02.14   MBA4119.0127.0316.2016.02.14 
##                          42048                          13338 
##   MBA4122.0112.0301.2016.02.14   MBA4123.0056.0245.2016.02.14 
##                          28583                          21135 
##   MBA4126.0150.0339.2016.02.14   MBA4127.0021.0417.2016.02.14 
##                           8596                          18287 
##   MBA4131.0006.0197.2016.02.14   MBA4132.0051.0240.2016.02.14 
##                         121717                          12687 
##   MBA4133.0135.0324.2016.02.14   MBA4136.0020.0464.2016.02.14 
##                          18548                          26125 
##   MBA4138.0068.0257.2016.02.14   MBA4140.0015.0206.2016.02.14 
##                          21282                          32717 
##  226855.boston.0016.2015.11.25  229075.boston.0178.2015.11.25 
##                          12316                          21754 
##  447537.boston.0170.2015.11.25  503564.boston.0169.2015.11.25 
##                          11055                          16113 
##  561130.boston.0065.2015.11.25  588800.boston.0068.2015.11.25 
##                          64533                          54160 
##  629356.boston.0076.2015.11.25  765828.boston.0089.2015.11.25 
##                          17564                          18760 
##  772512.boston.0090.2015.11.25  826391.boston.0177.2015.11.25 
##                          28599                          29717 
##  849016.boston.0176.2015.11.25  872569.boston.0179.2015.11.25 
##                          27270                          22343 
##     136109.048.0334.2018.12.15     137787.006.0293.2018.12.15 
##                         142042                         110949 
##     186400.027.0314.2018.12.15     207722.029.0316.2018.12.15 
##                         130465                         140162 
##     221355.060.0345.2018.12.15     225794.001.0288.2018.12.15 
##                         131800                         169725 
##     235185.033.0320.2018.12.15     236512.015.0302.2018.12.15 
##                         146986                         136583 
##     240884.013.0300.2018.12.15     241367.042.0329.2018.12.15 
##                         134087                         130533 
##     243736.036.0323.2018.12.15     276693.051.0337.2018.12.15 
##                         125215                         143542 
##     282036.026.0313.2018.12.15     298053.032.0319.2018.12.15 
##                         140377                         139377 
##     319820.035.0322.2018.12.15     354109.012.0299.2018.12.15 
##                         156031                         130651 
##     356403.053.0339.2018.12.15     364485.002.0289.2018.12.15 
##                         163928                         138715 
##     373167.021.0308.2018.12.15     395379.010.0297.2018.12.15 
##                          91803                         125471 
##     402235.052.0338.2018.12.15     411794.028.0315.2018.12.15 
##                         103407                         125418 
##     416151.055.0340.2018.12.15     420641.004.0291.2018.12.15 
##                         147234                         137640 
##     423443.005.0292.2018.12.15     445474.025.0312.2018.12.15 
##                         153787                         162503 
##     453551.040.0327.2018.12.15     459452.044.0331.2018.12.15 
##                         149435                         105418 
##     467375.007.0294.2018.12.15     471179.003.0290.2018.12.15 
##                         148513                         139922 
##     492275.058.0343.2018.12.15     570298.041.0328.2018.12.15 
##                         165014                         135343 
##     574541.045.0332.2018.12.15     576662.030.0317.2018.12.15 
##                         124317                         151416 
##     578598.017.0304.2018.12.15     580176.038.0325.2018.12.15 
##                         121232                         141927 
##     588443.031.0318.2018.12.15     588873.064.0349.2018.12.15 
##                          94483                         109394 
##     612872.019.0306.2018.12.15     667045.043.0330.2018.12.15 
##                         139365                         116480 
##     675294.009.0296.2018.12.15     684908.056.0341.2018.12.15 
##                         138860                         122542 
##     714983.057.0342.2018.12.15     720750.034.0321.2018.12.15 
##                         145724                         121774 
##     774051.062.0347.2018.12.15     821034.039.0326.2018.12.15 
##                         147680                         119747 
##     822655.037.0324.2018.12.15     823946.047.0333.2018.12.15 
##                          93713                         121833 
##     850240.022.0309.2018.12.15     851204.014.0301.2018.12.15 
##                         107960                         145594 
##     853781.063.0348.2018.12.15     857829.018.0305.2018.12.15 
##                         130937                         136894 
##     883092.049.0335.2018.12.15     909074.024.0311.2018.12.15 
##                         126602                          83194 
##     918320.061.0346.2018.12.15     938150.059.0344.2018.12.15 
##                         150659                         159642 
##     945496.008.0295.2018.12.15     948983.050.0336.2018.12.15 
##                         130406                         160584 
##     982757.020.0307.2018.12.15     985170.011.0298.2018.12.15 
##                         135822                         132462 
##    MBA1008.062.0371.2018.12.15    MBA1073.103.0380.2018.12.15 
##                          97776                         121454 
##    MBA1144.144.0244.2018.12.15    MBA1215.004.0359.2018.12.15 
##                          58436                         141749 
##    MBA1226.010.0360.2018.12.15    MBA1240.156.0246.2018.12.15 
##                         118025                         123306 
##    MBA1284.025.0362.2018.12.15    MBA1302.089.0375.2018.12.15 
##                         106064                         122557 
##    MBA1315.035.0365.2018.12.15    MBA1398.080.0374.2018.12.15 
##                         122382                         124340 
##    MBA1491.070.0372.2018.12.15    MBA1509.039.0366.2018.12.15 
##                         141649                         123099 
##    MBA1519.096.0377.2018.12.15    MBA4042.011.0361.2018.12.15 
##                         105745                         135959 
##    MBA4046.032.0363.2018.12.15    MBA4048.146.0245.2018.12.15 
##                         121271                         104141 
##    MBA4058.049.0368.2018.12.15    MBA4059.113.0242.2018.12.15 
##                         163357                         154949 
##    MBA4064.117.0243.2018.12.15    MBA4071.098.0379.2018.12.15 
##                          99265                         173599 
##    MBA4083.097.0378.2018.12.15    MBA4093.033.0364.2018.12.15 
##                         131209                         131778 
##    MBA4099.045.0367.2018.12.15    MBA4104.091.0376.2018.12.15 
##                         140969                         128881 
##    MBA4128.059.0369.2018.12.15 
##                         142304
```

```
min(rowSums(OTU_table))
```

```
## [1] 8596
```

```
Total_counts<-as.data.frame(rowSums(OTU_table))
colnames(Total_counts)<-c("Counts")
ggplot2::ggplot(Total_counts, aes(x=Counts))+geom_histogram(binwidth=100)+geom_vline(aes(xintercept=mean(Counts, na.rm=T)), color="red", linetype="dashed", size=1)+theme_bw()+
  ggtitle("Total count per sample distribution")+theme(plot.title=element_text(lineheight=10, size=15))+
  xlab("Counts")+ylab("Number of samples")+theme(axis.text=element_text(size=15), axis.title=element_text(size=15))
```

```
#Remove all those samples that do not reach a minimum threshold of number of reads:
counts<-as.data.frame(rowSums(OTU_table))
colnames(counts)<-"counts"
counts$Sample<-row.names(counts)
subset_8000<-counts[counts$counts>=8000,]
subset_8000$Sample<-NULL
OTU_table<-OTU_table[row.names(OTU_table)%in%as.vector(row.names(subset_8000)),]

#Rarefy to same sequencing depth:
set.seed(1)
OTU_table_8000<-vegan::rrarefy(OTU_table, 8000)
```

```
## Warning in vegan::rrarefy(OTU_table, 8000): function should be used for
## observed counts, but smallest count is 2
```

```
#Estimate richness:
richness_8000<-vegan::estimateR(OTU_table_8000)
richness_8000<-t(richness_8000)
richness_8000<-as.data.frame(richness_8000)
richness_8000$se.chao1<-NULL
richness_8000$se.ACE<-NULL

#Estimate evenness:
shannon<-BiodiversityR::diversityresult(x=OTU_table_8000, method="each site", index="Shannon")
diversity_8000<-cbind(shannon)

#Join data from richness and evenness calculations:
ecology_8000<-cbind(richness_8000, diversity_8000)
colnames(ecology_8000)<-c("Observed", "Chao1", "ACE", "Shannon")

#Subset metadata
metadata<-metadata[row.names(metadata)%in%row.names(ecology_8000),,drop=FALSE]

all.equal(row.names(metadata),row.names(ecology_8000))
```

```
## [1] TRUE
```

```
ecology_8000$SampleID<-metadata$SampleID
ecology_8000$Cohort<-metadata$sample_cohort
ecology_8000$hiv_phenotype<-metadata$hiv_phenotype
ecology_8000$sexual_orientation<-metadata$sexual_orientation
ecology_8000$sex<-metadata$sex

#Subset indivudals for this comparison
ecology_8000<-ecology_8000[ecology_8000$sex=="female",,drop=FALSE]
ecology_8000_melt<-reshape2::melt(ecology_8000)
```

```
## Using SampleID, Cohort, hiv_phenotype, sexual_orientation, sex as id variables
```

```
ecology_8000_melt_boston<-ecology_8000_melt[ecology_8000_melt$Cohort=="boston",,drop=F]
ecology_8000_melt_botswana<-ecology_8000_melt[ecology_8000_melt$Cohort=="botswana",,drop=F]
ecology_8000_melt_uganda<-ecology_8000_melt[ecology_8000_melt$Cohort=="uganda_2",,drop=F]

plot_boston<-ggplot2::ggplot(data=ecology_8000_melt_boston, aes(x=hiv_phenotype,y=value))+geom_boxplot(aes(alpha=hiv_phenotype), outlier.color="white", fill="royalblue4")+theme_bw()+
  geom_point(aes(alpha=hiv_phenotype), color="royalblue4", position=position_jitterdodge(jitter.width=0.25), size=1)+
  facet_wrap(~variable, scales="free_y", nrow=1)+scale_alpha_manual(values=c(0.9, 0.6, 0.3))+
  theme(axis.text.x = element_text(angle=90))+ggtitle("Just Female - Boston")

plot_botswana<-ggplot2::ggplot(data=ecology_8000_melt_botswana, aes(x=hiv_phenotype,y=value))+geom_boxplot(aes(alpha=hiv_phenotype), outlier.color="white", fill="darkorange")+theme_bw()+
  geom_point(aes(alpha=hiv_phenotype), color="darkorange", position=position_jitterdodge(jitter.width=0.25), size=1)+
  facet_wrap(~variable, scales="free_y", nrow=1)+scale_alpha_manual(values=c(0.9, 0.6, 0.3))+
  theme(axis.text.x = element_text(angle=90))+ggtitle("Just Female - Botswana")

plot_uganda<-ggplot2::ggplot(data=ecology_8000_melt_uganda, aes(x=hiv_phenotype,y=value))+geom_boxplot(aes(alpha=hiv_phenotype), outlier.color="white", fill="forestgreen")+theme_bw()+
  geom_point(aes(alpha=hiv_phenotype), color="forestgreen", position=position_jitterdodge(jitter.width=0.25), size=1)+
  facet_wrap(~variable, scales="free_y", nrow=1)+scale_alpha_manual(values=c(0.9, 0.6, 0.3))+
  theme(axis.text.x = element_text(angle=90))+ggtitle("Just Female - Uganda")

ggplot2::ggsave("SupplementaryFigure1D.pdf", grid.arrange(plot_boston, plot_botswana,plot_uganda, ncol=2, nrow=3), width=15, height=10)
```

```
#Statistical test
ecology_8000_boston<-ecology_8000[ecology_8000$Cohort=="boston",,drop=F]
ecology_8000_botswana<-ecology_8000[ecology_8000$Cohort=="botswana",,drop=F]
ecology_8000_uganda<-ecology_8000[ecology_8000$Cohort=="uganda_2",,drop=F]
# Add all metadata to the table for multivariate testing of abundance differences
metadata$age <- as.numeric(metadata$age)
metadata$BMI <- as.numeric(metadata$BMI)
### metadata not collected in Boston:
metadata_ordered$monthly_income <- as.numeric(metadata_ordered$monthly_income)
metadata_ordered$smoking_years <- as.numeric(metadata_ordered$smoking_years)
metadata_ordered$fram_10yr_risk_lab <- as.numeric(metadata_ordered$fram_10yr_risk_lab)
metadata_ordered$fram_10yr_risk_nonlab <- as.numeric(metadata_ordered$fram_10yr_risk_nonlab)
metadata_ordered$mean_imt <- as.numeric(metadata_ordered$mean_imt)
metadata_ordered$total_plaques <- as.numeric(metadata_ordered$total_plaques)
metadata_ordered$any_plaques <- as.numeric(metadata_ordered$any_plaques)
dplyr::left_join(ecology_8000_boston[colnames(ecology_8000_boston) %in% c("SampleID") | !colnames(ecology_8000_boston) %in% colnames(metadata)], metadata, by = "SampleID") -> us_full_metadata
dplyr::left_join(ecology_8000_botswana[colnames(ecology_8000_botswana) %in% c("SampleID") | !colnames(ecology_8000_botswana) %in% colnames(metadata)], metadata, by = "SampleID") -> botswana_full_metadata
dplyr::left_join(ecology_8000_uganda[colnames(ecology_8000_uganda) %in% c("SampleID") | !colnames(ecology_8000_uganda) %in% colnames(metadata)], metadata, by = "SampleID") -> uganda_full_metadata

# Kruskal and wilcox testing
#US
for (i in c("Observed", "Shannon")){
  print(i)
  print(kruskal(ecology_8000_boston[[i]], ecology_8000_boston[["hiv_phenotype"]],group=F,p.adj = "bonferroni"))
}
```

```
## [1] "Observed"
## $statistics
##        Chisq Df    p.chisq
##   0.70437301  2 0.70314897
## 
## $parameters
##             test  p.ajusted                                 name.t ntr alpha
##   Kruskal-Wallis bonferroni ecology_8000_boston[["hiv_phenotype"]]   3  0.05
## 
## $means
##                ecology_8000_boston..i..      rank       std  r Min Max Q25 Q50
## 1_hiv_negative                129.02703 25.878378 41.407518 37  67 220  96 128
## 2_suppressed                  134.11111 28.777778 35.547308  9  79 185 103 138
## 4_unsuppressed                139.42857 30.642857 52.357289  7  46 216 124 146
##                Q75
## 1_hiv_negative 148
## 2_suppressed   159
## 4_unsuppressed 160
## 
## $comparison
##                                 Difference pvalue Signif.        LCL       UCL
## 1_hiv_negative - 2_suppressed   -2.8993994      1         -17.296558 11.497759
## 1_hiv_negative - 4_unsuppressed -4.7644788      1         -20.730491 11.201533
## 2_suppressed - 4_unsuppressed   -1.8650794      1         -21.386423 17.656264
## 
## $groups
## NULL
## 
## attr(,"class")
## [1] "group"
## [1] "Shannon"
## $statistics
##        Chisq Df    p.chisq
##   0.81960127  2 0.66378257
## 
## $parameters
##             test  p.ajusted                                 name.t ntr alpha
##   Kruskal-Wallis bonferroni ecology_8000_boston[["hiv_phenotype"]]   3  0.05
## 
## $means
##                ecology_8000_boston..i..      rank        std  r       Min
## 1_hiv_negative                3.3661959 25.756757 0.68182782 37 1.4674836
## 2_suppressed                  3.4574302 29.333333 0.74233344  9 1.7319634
## 4_unsuppressed                3.4537007 30.571429 0.88170851  7 1.5978440
##                      Max       Q25       Q50       Q75
## 1_hiv_negative 4.3075641 3.1224702 3.5533268 3.8161932
## 2_suppressed   4.0715204 3.1530909 3.7108917 3.9640645
## 4_unsuppressed 4.2118247 3.4097806 3.6637269 3.9414740
## 
## $comparison
##                                 Difference pvalue Signif.        LCL       UCL
## 1_hiv_negative - 2_suppressed   -3.5765766      1         -17.962196 10.809043
## 1_hiv_negative - 4_unsuppressed -4.8146718      1         -20.767887 11.138544
## 2_suppressed - 4_unsuppressed   -1.2380952      1         -20.743793 18.267602
## 
## $groups
## NULL
## 
## attr(,"class")
## [1] "group"
```

```
#Botswana
for (i in c("Observed", "Shannon")){
  print(i)
  print(kruskal(ecology_8000_botswana[[i]], ecology_8000_botswana[["hiv_phenotype"]],group=F,p.adj = "bonferroni"))
}
```

```
## [1] "Observed"
## $statistics
##        Chisq Df    p.chisq
##   0.21741699  2 0.89699186
## 
## $parameters
##             test  p.ajusted                                   name.t ntr alpha
##   Kruskal-Wallis bonferroni ecology_8000_botswana[["hiv_phenotype"]]   3  0.05
## 
## $means
##                ecology_8000_botswana..i..      rank       std  r Min Max    Q25
## 1_hiv_negative                  140.16667 48.277778 63.032645 36  32 268  93.25
## 2_suppressed                    137.07895 47.368421 54.384210 38  48 294  91.25
## 4_unsuppressed                  141.45455 50.818182 57.853450 22  26 256 112.00
##                  Q50    Q75
## 1_hiv_negative 131.0 172.50
## 2_suppressed   131.5 167.00
## 4_unsuppressed 134.0 174.75
## 
## $comparison
##                                  Difference pvalue Signif.        LCL       UCL
## 1_hiv_negative - 2_suppressed    0.90935673      1         -15.034818 16.853531
## 1_hiv_negative - 4_unsuppressed -2.54040404      1         -21.091973 16.011165
## 2_suppressed - 4_unsuppressed   -3.44976077      1         -21.815217 14.915695
## 
## $groups
## NULL
## 
## attr(,"class")
## [1] "group"
## [1] "Shannon"
## $statistics
##        Chisq Df    p.chisq
##   0.25412837  2 0.88067714
## 
## $parameters
##             test  p.ajusted                                   name.t ntr alpha
##   Kruskal-Wallis bonferroni ecology_8000_botswana[["hiv_phenotype"]]   3  0.05
## 
## $means
##                ecology_8000_botswana..i..      rank        std  r       Min
## 1_hiv_negative                  3.1865789 50.277778 0.87535038 36 1.1161733
## 2_suppressed                    3.1420968 47.815789 0.74484672 38 1.2704241
## 4_unsuppressed                  3.1040233 46.772727 0.87779570 22 1.5072167
##                      Max       Q25       Q50       Q75
## 1_hiv_negative 4.5147266 2.4664050 3.2917045 3.8719558
## 2_suppressed   4.5307380 2.7925236 3.2256206 3.6305207
## 4_unsuppressed 4.5031901 2.3758476 3.0924713 3.8279199
## 
## $comparison
##                                 Difference pvalue Signif.        LCL       UCL
## 1_hiv_negative - 2_suppressed    2.4619883      1         -13.481586 18.405562
## 1_hiv_negative - 4_unsuppressed  3.5050505      1         -15.045819 22.055920
## 2_suppressed - 4_unsuppressed    1.0430622      1         -17.321702 19.407826
## 
## $groups
## NULL
## 
## attr(,"class")
## [1] "group"
```

```
#Uganda
for (i in c("Observed", "Shannon")){
  print(i)
  print(wilcox.test(ecology_8000_uganda[[i]]~ecology_8000_uganda[["hiv_phenotype"]]))
}
```

```
## [1] "Observed"
```

```
## Warning in wilcox.test.default(x = DATA[[1L]], y = DATA[[2L]], ...): cannot
## compute exact p-value with ties
```

```
## 
##  Wilcoxon rank sum test with continuity correction
## 
## data:  ecology_8000_uganda[[i]] by ecology_8000_uganda[["hiv_phenotype"]]
## W = 1148, p-value = 0.027147
## alternative hypothesis: true location shift is not equal to 0
## 
## [1] "Shannon"
## 
##  Wilcoxon rank sum exact test
## 
## data:  ecology_8000_uganda[[i]] by ecology_8000_uganda[["hiv_phenotype"]]
## W = 1203, p-value = 0.0066101
## alternative hypothesis: true location shift is not equal to 0
```

```
### Run orm (n=479) and compare alpha diversity by hiv_phenotype
### Extra metadata that have full n: Race, Ethnicity, age, sex, current_art_class_consolid2, tmp_smx_active
### Extra metadata that have <n: BMI, comorbidities (dm2_hx, hld_hx, htn_hx, cvd_hx, ckd_hx, cvd_dx [missing boston], dm2hx_dx, hldhx_dx, htnhx_dx, cvdhx_dx, ever_smoke, current_smoke, smoke_cat), school_level [uganda2 only], monthly_income[uganda2 only], current_art_class_consolid2, tmp_smx_active, days_on_art, sexual_orientation
### additional: smoking_years, fram_10yr_risk_lab, fram_10yr_risk_nonlab, mean_imt, total_plaques, any_plaques
covars_full_n <- c("Ethnicity", "age", "Race", "current_art_class_consolid2", "tmp_smx_active", "hiv_phenotype")

#US
for (i in c("Observed", "Shannon")){
  print(i)
  print(rms::orm(formula =  as.formula(paste("us_full_metadata[[",which(stringr::str_detect(colnames(us_full_metadata), i)), "]]~", paste(covars_full_n, collapse = "+"), sep = "")), data = us_full_metadata))
}
```

```
## [1] "Observed"
```

```
## Warning in .local(x, ...): singularity problem
```

```
## Warning in .local(x, ...): singularity problem

## Warning in .local(x, ...): singularity problem

## Warning in .local(x, ...): singularity problem

## Warning in .local(x, ...): singularity problem

## Warning in .local(x, ...): singularity problem

## Warning in .local(x, ...): singularity problem
```

```
## Logistic (Proportional Odds) Ordinal Regression Model
## 
## rms::orm(formula = as.formula(paste("us_full_metadata[[", which(stringr::str_detect(colnames(us_full_metadata), 
##     i)), "]]~", paste(covars_full_n, collapse = "+"), sep = "")), 
##     data = us_full_metadata)
## 
##                       Model Likelihood               Discrimination    Rank Discrim.    
##                             Ratio Test                      Indexes          Indexes    
## Obs            53    LR chi2     11.29    R2                  0.192    rho     0.408    
## Distinct Y     41    d.f.           14    R2(14,53)           0.000                     
## Median Y      131    Pr(> chi2) 0.6634    R2(14,52.9)         0.000                     
## max |deriv| 3e-07    Score chi2  13.92    |Pr(Y>=median)-0.5| 0.138                     
##                      Pr(> chi2) 0.4557                                                  
## 
##                                             Coef    S.E.   Wald Z Pr(>|Z|)
## Ethnicity=Not_Hispanic_Latino                0.1280 0.8177  0.16  0.8756  
## age                                          0.0288 0.0226  1.27  0.2033  
## Race=Asian                                   1.1766 2.6354  0.45  0.6553  
## Race=Black_AA                               -1.2481 2.0657 -0.60  0.5457  
## Race=Unknown                                 0.6294 2.4956  0.25  0.8009  
## Race=Varied                                 -0.5575 2.6094 -0.21  0.8308  
## Race=White                                  -0.3616 2.1417 -0.17  0.8659  
## current_art_class_consolid2=none            -0.7700 1.5172 -0.51  0.6118  
## current_art_class_consolid2=NRTI_NRTI_ISTI  -1.1225 1.7434 -0.64  0.5197  
## current_art_class_consolid2=NRTI_NRTI_NNRTI -3.2945 2.1708 -1.52  0.1291  
## current_art_class_consolid2=NRTI_NRTI_PI     1.2130 1.7763  0.68  0.4947  
## tmp_smx_active                               0.0000 0.0000                
## hiv_phenotype=2_suppressed                  -0.3072 0.0000  -Inf  <0.0001 
## hiv_phenotype=4_unsuppressed                 0.5066 0.8789  0.58  0.5644  
## 
## [1] "Shannon"
```

```
## Warning in .local(x, ...): singularity problem

## Warning in .local(x, ...): singularity problem

## Warning in .local(x, ...): singularity problem

## Warning in .local(x, ...): singularity problem

## Warning in .local(x, ...): singularity problem

## Warning in .local(x, ...): singularity problem

## Warning in .local(x, ...): singularity problem
```

```
## Logistic (Proportional Odds) Ordinal Regression Model
## 
## rms::orm(formula = as.formula(paste("us_full_metadata[[", which(stringr::str_detect(colnames(us_full_metadata), 
##     i)), "]]~", paste(covars_full_n, collapse = "+"), sep = "")), 
##     data = us_full_metadata)
## 
##                           Model Likelihood               Discrimination    Rank Discrim.    
##                                 Ratio Test                      Indexes          Indexes    
## Obs                53    LR chi2      7.59    R2                  0.134    rho     0.342    
## Distinct Y         53    d.f.           14    R2(14,53)           0.000                     
## Median Y    3.5800493    Pr(> chi2) 0.9094    R2(14,53)           0.000                     
## max |deriv|     3e-07    Score chi2   9.65    |Pr(Y>=median)-0.5| 0.115                     
##                          Pr(> chi2) 0.7874                                                  
## 
##                                             Coef    S.E.         Wald Z
## Ethnicity=Not_Hispanic_Latino                0.0918       0.7872  0.12 
## age                                         -0.0227       0.0221 -1.03 
## Race=Asian                                  -2.0851       2.6818 -0.78 
## Race=Black_AA                               -2.4580       2.1043 -1.17 
## Race=Unknown                                -3.5350       2.5599 -1.38 
## Race=Varied                                 -3.0222       2.6792 -1.13 
## Race=White                                  -2.4851       2.1746 -1.14 
## current_art_class_consolid2=none             0.1477 1924623.2038  0.00 
## current_art_class_consolid2=NRTI_NRTI_ISTI  -1.4163       1.8272 -0.78 
## current_art_class_consolid2=NRTI_NRTI_NNRTI -2.0384       2.1297 -0.96 
## current_art_class_consolid2=NRTI_NRTI_PI     0.7435       1.8321  0.41 
## tmp_smx_active                               0.0000       0.0000       
## hiv_phenotype=2_suppressed                   0.9997 1924623.2038  0.00 
## hiv_phenotype=4_unsuppressed                 0.9477       0.9122  1.04 
##                                             Pr(>|Z|)
## Ethnicity=Not_Hispanic_Latino               0.9071  
## age                                         0.3050  
## Race=Asian                                  0.4369  
## Race=Black_AA                               0.2428  
## Race=Unknown                                0.1673  
## Race=Varied                                 0.2593  
## Race=White                                  0.2531  
## current_art_class_consolid2=none            1.0000  
## current_art_class_consolid2=NRTI_NRTI_ISTI  0.4383  
## current_art_class_consolid2=NRTI_NRTI_NNRTI 0.3385  
## current_art_class_consolid2=NRTI_NRTI_PI    0.6849  
## tmp_smx_active                                      
## hiv_phenotype=2_suppressed                  1.0000  
## hiv_phenotype=4_unsuppressed                0.2989
```

```
#Botswana
for (i in c("Observed", "Shannon")){
  print(i)
  print(rms::orm(formula =  as.formula(paste("botswana_full_metadata[[",which(stringr::str_detect(colnames(botswana_full_metadata), i)), "]]~", paste(covars_full_n[!covars_full_n %in% c("Ethnicity", "Race")], collapse = "+"), sep = "")), data = botswana_full_metadata))
}
```

```
## [1] "Observed"
```

```
## Warning in .local(x, ...): singularity problem

## Warning in .local(x, ...): singularity problem

## Warning in .local(x, ...): singularity problem

## Warning in .local(x, ...): singularity problem
```

```
## Logistic (Proportional Odds) Ordinal Regression Model
## 
## rms::orm(formula = as.formula(paste("botswana_full_metadata[[", 
##     which(stringr::str_detect(colnames(botswana_full_metadata), 
##         i)), "]]~", paste(covars_full_n[!covars_full_n %in% c("Ethnicity", 
##         "Race")], collapse = "+"), sep = "")), data = botswana_full_metadata)
## 
##                       Model Likelihood               Discrimination    Rank Discrim.    
##                             Ratio Test                      Indexes          Indexes    
## Obs            96    LR chi2      3.13    R2                  0.032    rho     0.179    
## Distinct Y     70    d.f.            6    R2(6,96)            0.000                     
## Median Y      131    Pr(> chi2) 0.7929    R2(6,96)            0.000                     
## max |deriv| 4e-08    Score chi2   3.14    |Pr(Y>=median)-0.5| 0.064                     
##                      Pr(> chi2) 0.7908                                                  
## 
##                                             Coef     S.E.   Wald Z Pr(>|Z|)
## age                                           0.0479 0.0354  1.35  0.1759  
## current_art_class_consolid2=NRTI_NRTI_NNRTI  27.0231 0.4589 58.88  <0.0001 
## current_art_class_consolid2=NRTI_NRTI_PI     26.3989 0.5333 49.50  <0.0001 
## tmp_smx_active                                0.0000 0.0000                
## hiv_phenotype=2_suppressed                  -26.7654 0.0000  -Inf  <0.0001 
## hiv_phenotype=4_unsuppressed                  0.2414 0.4836  0.50  0.6177  
## 
## [1] "Shannon"
```

```
## Warning in .local(x, ...): singularity problem

## Warning in .local(x, ...): singularity problem

## Warning in .local(x, ...): singularity problem

## Warning in .local(x, ...): singularity problem
```

```
## Logistic (Proportional Odds) Ordinal Regression Model
## 
## rms::orm(formula = as.formula(paste("botswana_full_metadata[[", 
##     which(stringr::str_detect(colnames(botswana_full_metadata), 
##         i)), "]]~", paste(covars_full_n[!covars_full_n %in% c("Ethnicity", 
##         "Race")], collapse = "+"), sep = "")), data = botswana_full_metadata)
## 
##                           Model Likelihood               Discrimination    Rank Discrim.    
##                                 Ratio Test                      Indexes          Indexes    
## Obs                96    LR chi2      6.10    R2                  0.062    rho     0.211    
## Distinct Y         96    d.f.            6    R2(6,96)            0.001                     
## Median Y    3.2296705    Pr(> chi2) 0.4120    R2(6,96)            0.001                     
## max |deriv|    0.0001    Score chi2   6.32    |Pr(Y>=median)-0.5| 0.091                     
##                          Pr(> chi2) 0.3886                                                  
## 
##                                             Coef    S.E.   Wald Z Pr(>|Z|)
## age                                          0.0894 0.0371  2.41  0.0159  
## current_art_class_consolid2=NRTI_NRTI_NNRTI  0.1237 0.4488  0.28  0.7828  
## current_art_class_consolid2=NRTI_NRTI_PI    -0.1251 0.5303 -0.24  0.8135  
## tmp_smx_active                               0.0000 0.0000                
## hiv_phenotype=2_suppressed                  -0.1436 0.0000  -Inf  <0.0001 
## hiv_phenotype=4_unsuppressed                -0.1488 0.4948 -0.30  0.7637
```

```
#Uganda
for (i in c("Observed", "Shannon")){
  print(i)
  print(rms::orm(formula =  as.formula(paste("uganda_full_metadata[[",which(stringr::str_detect(colnames(uganda_full_metadata), i)), "]]~", paste(covars_full_n[!covars_full_n %in% c("Ethnicity", "Race")], collapse = "+"), sep = "")), data = uganda_full_metadata))
} ###*** Observed hiv_phenotype p = 1.0000 Shannon hiv_phenotype p = 1.0000
```

```
## [1] "Observed"
```

```
## Warning in .local(x, ...): singularity problem

## Warning in .local(x, ...): singularity problem
```

```
## Logistic (Proportional Odds) Ordinal Regression Model
## 
## rms::orm(formula = as.formula(paste("uganda_full_metadata[[", 
##     which(stringr::str_detect(colnames(uganda_full_metadata), 
##         i)), "]]~", paste(covars_full_n[!covars_full_n %in% c("Ethnicity", 
##         "Race")], collapse = "+"), sep = "")), data = uganda_full_metadata)
## 
##                       Model Likelihood               Discrimination    Rank Discrim.    
##                             Ratio Test                      Indexes          Indexes    
## Obs            85    LR chi2     11.64    R2                  0.128    rho     0.252    
## Distinct Y     72    d.f.            5    R2(5,85)            0.075                     
## Median Y      109    Pr(> chi2) 0.0400    R2(5,85)            0.075                     
## max |deriv| 0.001    Score chi2  16.00    |Pr(Y>=median)-0.5| 0.101                     
##                      Pr(> chi2) 0.0068                                                  
## 
##                                             Coef    S.E.         Wald Z
## age                                         -0.0046       0.0337 -0.14 
## current_art_class_consolid2=NRTI_NRTI_NNRTI -5.0701 8390977.5322  0.00 
## current_art_class_consolid2=NRTI_NRTI_PI    -5.0391 8390977.5322  0.00 
## tmp_smx_active                               2.8636       1.3130  2.18 
## hiv_phenotype=2_suppressed                   1.4733 8390977.5322  0.00 
##                                             Pr(>|Z|)
## age                                         0.8910  
## current_art_class_consolid2=NRTI_NRTI_NNRTI 1.0000  
## current_art_class_consolid2=NRTI_NRTI_PI    1.0000  
## tmp_smx_active                              0.0292  
## hiv_phenotype=2_suppressed                  1.0000  
## 
## [1] "Shannon"
```

```
## Warning in .local(x, ...): singularity problem

## Warning in .local(x, ...): singularity problem

## Warning in .local(x, ...): singularity problem
```

```
## Logistic (Proportional Odds) Ordinal Regression Model
## 
## rms::orm(formula = as.formula(paste("uganda_full_metadata[[", 
##     which(stringr::str_detect(colnames(uganda_full_metadata), 
##         i)), "]]~", paste(covars_full_n[!covars_full_n %in% c("Ethnicity", 
##         "Race")], collapse = "+"), sep = "")), data = uganda_full_metadata)
## 
##                           Model Likelihood               Discrimination    Rank Discrim.    
##                                 Ratio Test                      Indexes          Indexes    
## Obs                85    LR chi2     17.91    R2                  0.190    rho     0.284    
## Distinct Y         85    d.f.            5    R2(5,85)            0.141                     
## Median Y    3.4195344    Pr(> chi2) 0.0031    R2(5,85)            0.141                     
## max |deriv|    0.0006    Score chi2  23.60    |Pr(Y>=median)-0.5| 0.125                     
##                          Pr(> chi2) 0.0003                                                  
## 
##                                             Coef     S.E.   Wald Z Pr(>|Z|)
## age                                           0.0027 0.0336  0.08  0.9362  
## current_art_class_consolid2=NRTI_NRTI_NNRTI  -9.1266 1.4691 -6.21  <0.0001 
## current_art_class_consolid2=NRTI_NRTI_PI    -10.1710 0.0000  -Inf  <0.0001 
## tmp_smx_active                                4.1564 1.6363  2.54  0.0111  
## hiv_phenotype=2_suppressed                    4.0728 1.9696  2.07  0.0387
```

```
#--------------------------------------------------------------------------------------------------------------
```
