# Supplementary material for: HIV-associated gut microbial alterations are dependent on host and geographic context
Source: Nat Commun. 2024 Feb 5;15:1055. doi: 10.1038/s41467-023-44566-4 (PMC10844288; doi:10.1038/s41467-023-44566-4)
Supplement: Supplementary file 13 — SupplementaryFigure2_Rocafort-Gootenberg_2022_03_20 [file 41467_2023_44566_MOESM13_ESM.html]

Rocafort-Gootenberg\_SupplementaryFigure2


# Rocafort-Gootenberg\_SupplementaryFigure2

#Load needed R packages

```
library("phyloseq")
library("tidyverse")
```

```
## ── Attaching packages ─────────────────────────────────────── tidyverse 1.3.2 ──
## ✔ ggplot2 3.4.1     ✔ purrr   1.0.1
## ✔ tibble  3.1.8     ✔ dplyr   1.1.0
## ✔ tidyr   1.3.0     ✔ stringr 1.5.0
## ✔ readr   2.1.4     ✔ forcats 1.0.0
## ── Conflicts ────────────────────────────────────────── tidyverse_conflicts() ──
## ✖ dplyr::filter() masks stats::filter()
## ✖ dplyr::lag()    masks stats::lag()
```

```
library("stringr")
library("ggplot2")
library("gridExtra")
```

```
## 
## Attaching package: 'gridExtra'
## 
## The following object is masked from 'package:dplyr':
## 
##     combine
```

```
library("dplyr")
library("vegan")
```

```
## Loading required package: permute
## Loading required package: lattice
## This is vegan 2.6-4
```

```
library("agricolae")
```

```
## Registered S3 methods overwritten by 'klaR':
##   method      from 
##   predict.rda vegan
##   print.rda   vegan
##   plot.rda    vegan
```

```
library("knitr") 
library("BiodiversityR")
```

```
## Loading required package: tcltk
## BiodiversityR 2.15-1: Use command BiodiversityRGUI() to launch the Graphical User Interface; 
## to see changes use BiodiversityRGUI(changeLog=TRUE, backward.compatibility.messages=TRUE)
```

```
library("reshape")
```

```
## 
## Attaching package: 'reshape'
## 
## The following object is masked from 'package:dplyr':
## 
##     rename
## 
## The following objects are masked from 'package:tidyr':
## 
##     expand, smiths
```

```
library("usedist")
library("rms")
```

```
## Loading required package: Hmisc
## Loading required package: survival
## Loading required package: Formula
## 
## Attaching package: 'Hmisc'
## 
## The following objects are masked from 'package:dplyr':
## 
##     src, summarize
## 
## The following objects are masked from 'package:base':
## 
##     format.pval, units
## 
## 
## Attaching package: 'rms'
## 
## The following object is masked from 'package:vegan':
## 
##     calibrate
```

```
library("DESeq2")
```

```
## Loading required package: S4Vectors
## Loading required package: stats4
## Loading required package: BiocGenerics
## 
## Attaching package: 'BiocGenerics'
## 
## The following object is masked from 'package:gridExtra':
## 
##     combine
## 
## The following objects are masked from 'package:dplyr':
## 
##     combine, intersect, setdiff, union
## 
## The following objects are masked from 'package:stats':
## 
##     IQR, mad, sd, var, xtabs
## 
## The following objects are masked from 'package:base':
## 
##     anyDuplicated, aperm, append, as.data.frame, basename, cbind,
##     colnames, dirname, do.call, duplicated, eval, evalq, Filter, Find,
##     get, grep, grepl, intersect, is.unsorted, lapply, Map, mapply,
##     match, mget, order, paste, pmax, pmax.int, pmin, pmin.int,
##     Position, rank, rbind, Reduce, rownames, sapply, setdiff, sort,
##     table, tapply, union, unique, unsplit, which.max, which.min
## 
## 
## Attaching package: 'S4Vectors'
## 
## The following objects are masked from 'package:reshape':
## 
##     expand, rename
## 
## The following objects are masked from 'package:dplyr':
## 
##     first, rename
## 
## The following object is masked from 'package:tidyr':
## 
##     expand
## 
## The following objects are masked from 'package:base':
## 
##     expand.grid, I, unname
## 
## Loading required package: IRanges
## 
## Attaching package: 'IRanges'
## 
## The following objects are masked from 'package:dplyr':
## 
##     collapse, desc, slice
## 
## The following object is masked from 'package:purrr':
## 
##     reduce
## 
## The following object is masked from 'package:phyloseq':
## 
##     distance
## 
## Loading required package: GenomicRanges
## Loading required package: GenomeInfoDb
## Loading required package: SummarizedExperiment
## Loading required package: MatrixGenerics
## Loading required package: matrixStats
## 
## Attaching package: 'matrixStats'
## 
## The following object is masked from 'package:dplyr':
## 
##     count
## 
## 
## Attaching package: 'MatrixGenerics'
## 
## The following objects are masked from 'package:matrixStats':
## 
##     colAlls, colAnyNAs, colAnys, colAvgsPerRowSet, colCollapse,
##     colCounts, colCummaxs, colCummins, colCumprods, colCumsums,
##     colDiffs, colIQRDiffs, colIQRs, colLogSumExps, colMadDiffs,
##     colMads, colMaxs, colMeans2, colMedians, colMins, colOrderStats,
##     colProds, colQuantiles, colRanges, colRanks, colSdDiffs, colSds,
##     colSums2, colTabulates, colVarDiffs, colVars, colWeightedMads,
##     colWeightedMeans, colWeightedMedians, colWeightedSds,
##     colWeightedVars, rowAlls, rowAnyNAs, rowAnys, rowAvgsPerColSet,
##     rowCollapse, rowCounts, rowCummaxs, rowCummins, rowCumprods,
##     rowCumsums, rowDiffs, rowIQRDiffs, rowIQRs, rowLogSumExps,
##     rowMadDiffs, rowMads, rowMaxs, rowMeans2, rowMedians, rowMins,
##     rowOrderStats, rowProds, rowQuantiles, rowRanges, rowRanks,
##     rowSdDiffs, rowSds, rowSums2, rowTabulates, rowVarDiffs, rowVars,
##     rowWeightedMads, rowWeightedMeans, rowWeightedMedians,
##     rowWeightedSds, rowWeightedVars
## 
## Loading required package: Biobase
## Welcome to Bioconductor
## 
##     Vignettes contain introductory material; view with
##     'browseVignettes()'. To cite Bioconductor, see
##     'citation("Biobase")', and for packages 'citation("pkgname")'.
## 
## 
## Attaching package: 'Biobase'
## 
## The following object is masked from 'package:MatrixGenerics':
## 
##     rowMedians
## 
## The following objects are masked from 'package:matrixStats':
## 
##     anyMissing, rowMedians
## 
## The following object is masked from 'package:Hmisc':
## 
##     contents
## 
## The following object is masked from 'package:phyloseq':
## 
##     sampleNames
```

```
library("gplots")
```

```
## 
## Attaching package: 'gplots'
## 
## The following object is masked from 'package:IRanges':
## 
##     space
## 
## The following object is masked from 'package:S4Vectors':
## 
##     space
## 
## The following object is masked from 'package:stats':
## 
##     lowess
```

#Load original phyloseq oject output from DADA2 pipeline and put in
new metadata

```
ps_gg_fp_f_prevalence_filter_2019_05_26<-readRDS("ps_gg_fp_f_prevalence_filter_2019_05_26")
readr::read_csv(
  "Metadata_formatted_nat_comm_add_2021_10_24.csv",
  col_names = TRUE,
  col_types = NULL,
  col_select = NULL,
  id = NULL,
  locale = default_locale(),
  na = c("", "NA", "empty", "EMPTY"),
  quote = "\"",
  comment = "",
  trim_ws = TRUE,
  skip = 0,
  name_repair = "unique",
  num_threads = readr_threads(),
  progress = show_progress(),
  show_col_types = should_show_types(),
  skip_empty_rows = TRUE,
  lazy = TRUE
) -> new_metadata
```

```
## Rows: 597 Columns: 88
## ── Column specification ────────────────────────────────────────────────────────
## Delimiter: ","
## chr (26): X, SampleID, subject_id, Race, Ethnicity, unique_id, sequencing_da...
## dbl (62): primer_used, read_count, age, height_cm, height_in, weight_kg, wei...
## 
## ℹ Use `spec()` to retrieve the full column specification for this data.
## ℹ Specify the column types or set `show_col_types = FALSE` to quiet this message.
```

```
### add {SampleID} as rownames
new_metadata_as_sample_data <- phyloseq::sample_data(new_metadata)
phyloseq::sample_names(new_metadata_as_sample_data) <- dplyr::pull(new_metadata, 1)
phyloseq::sample_data(ps_gg_fp_f_prevalence_filter_2019_05_26) <- new_metadata_as_sample_data
#Fix randomness
set.seed(1)
```

#Supplemental Figure 2

```
#FIGURE A (1A)
#--------------------------------------------------------------------------------------------------------------
#Transform count data in the phyloseq object
ps_gg_fp_f_prevalence_filter_2019_05_26_proportion<-phyloseq::transform_sample_counts(ps_gg_fp_f_prevalence_filter_2019_05_26, function(x)(x/sum(x)))

#Select samples of interest and update phyloseq object
metadata<-as.data.frame(phyloseq::sample_data(ps_gg_fp_f_prevalence_filter_2019_05_26_proportion))
metadata<-metadata[metadata$hiv_phenotype=="1_hiv_negative",,drop=FALSE]
metadata<-as.data.frame(as.matrix(metadata[metadata$sexual_orientation!="MSM",,drop=FALSE]))
metadata<-metadata[metadata$sex=="male",,drop=F]
phyloseq::sample_data(ps_gg_fp_f_prevalence_filter_2019_05_26_proportion)<-metadata

#Run PCoA on the phyloseq object
ordination<-phyloseq::ordinate(ps_gg_fp_f_prevalence_filter_2019_05_26_proportion, "PCoA", "unifrac")
```

```
## Warning in matrix(tree$edge[order(tree$edge[, 1]), ][, 2], byrow = TRUE, : data
## length [8987] is not a sub-multiple or multiple of the number of rows [4494]
```

```
ordination$values[1:2,]
```

```
##   Eigenvalues Relative_eig Broken_stick  Cumul_eig Cumul_br_stick
## 1    3.477893   0.06752945   0.04139243 0.06752945     0.04139243
## 2    2.700878   0.05244232   0.03381667 0.11997178     0.07520910
```

```
metadata<-as.data.frame(phyloseq::sample_data(ps_gg_fp_f_prevalence_filter_2019_05_26_proportion))
metadata<-metadata[row.names(ordination$vectors),,drop=FALSE]
all.equal(row.names(metadata), row.names(ordination$vectors))
```

```
## [1] TRUE
```

```
metadata$Unifrac1<-ordination$vectors[,1]
metadata$Unifrac2<-ordination$vectors[,2]

plot1<-ggplot(data=metadata, aes(x=Unifrac1, y=Unifrac2))+geom_point(aes(color=sample_cohort), size=3)+theme_bw()+stat_ellipse(aes(color=sample_cohort))+
  geom_point(data=metadata %>% group_by(sample_cohort) %>% summarise_at(vars(matches("Unifrac")), mean),size=6, aes(color=sample_cohort))+
  scale_color_manual(values=c("royalblue4","darkorange", "forestgreen"))+ggtitle("Figure 1A - JustMales")

plot1_axis<-ggplot(data=metadata, aes(x=sample_cohort, y=Unifrac1))+coord_flip()+geom_boxplot(aes(fill=sample_cohort), alpha=0.5, outlier.color="white")+theme_bw()+
  scale_fill_manual(values=c("royalblue4","darkorange","forestgreen"))+geom_point(aes(color=sample_cohort), position=position_jitterdodge(jitter.width=0.25),alpha=0.75, size=2)+
  scale_color_manual(values=c("royalblue4","darkorange","forestgreen"))

ggsave("SupplementaryFigure2A_v1.pdf", grid.arrange(plot1,plot1_axis, ncol=2, nrow=2), width=15, height=10)
```

```
#Run Adonis (n=133) and run orm to compare axis position along Axis 1
ASV_table<-as.data.frame(phyloseq::otu_table(ps_gg_fp_f_prevalence_filter_2019_05_26_proportion))
all.equal(row.names(ASV_table), row.names(metadata))
```

```
## [1] TRUE
```

```
unifrac.distance<-unname(phyloseq::UniFrac(ps_gg_fp_f_prevalence_filter_2019_05_26_proportion, weighted = FALSE)) ### unname fixes error introduced by Desctools see https://github.com/joey711/phyloseq/issues/1457
```

```
## Warning in matrix(tree$edge[order(tree$edge[, 1]), ][, 2], byrow = TRUE, : data
## length [8987] is not a sub-multiple or multiple of the number of rows [4494]
```

```
attributes(unifrac.distance)$Labels <- phyloseq::sample_names(ps_gg_fp_f_prevalence_filter_2019_05_26_proportion)
print(vegan::adonis2(unifrac.distance~metadata$sample_cohort, data=ASV_table, permutations=1000)) -> adon_univar
```

```
## Permutation test for adonis under reduced model
## Terms added sequentially (first to last)
## Permutation: free
## Number of permutations: 1000
## 
## vegan::adonis2(formula = unifrac.distance ~ metadata$sample_cohort, data = ASV_table, permutations = 1000)
##                         Df SumOfSqs      R2      F   Pr(>F)    
## metadata$sample_cohort   2    3.488 0.06773 4.7224 0.000999 ***
## Residual               130   48.014 0.93227                    
## Total                  132   51.502 1.00000                    
## ---
## Signif. codes:  0 '***' 0.001 '**' 0.01 '*' 0.05 '.' 0.1 ' ' 1
```

```
###*** sample_cohort r2 = 0.067732 p = 0.000999

# vegan::adonis2(formula = unifrac.distance ~ metadata$sample_cohort, data = ASV_table, permutations = 1000)
#                         Df SumOfSqs      R2      F   Pr(>F)    
# metadata$sample_cohort   2    3.488 0.06773 4.7224 0.000999 ***
# Residual               130   48.014 0.93227                    
# Total                  132   51.502 1.00000                    

# Kruskal and orm to test boxplot panel
print(agricolae::kruskal(metadata$Unifrac1, metadata$sample_cohort, group=F, p.adj = "bonferroni")) -> kruskal_univar
```

```
## $statistics
##      Chisq Df p.chisq
##   89.12321  2       0
## 
## $parameters
##             test  p.ajusted                 name.t ntr alpha
##   Kruskal-Wallis bonferroni metadata$sample_cohort   3  0.05
## 
## $means
##          metadata.Unifrac1      rank        std  r         Min       Max
## boston          -0.1575291  29.68750 0.10411729 48 -0.33358096 0.1175571
## botswana         0.0192830  70.61364 0.09538961 44 -0.17247186 0.2436780
## uganda_2         0.1637304 106.80488 0.08223672 41 -0.02764645 0.3140904
##                 Q25         Q50         Q75
## boston   -0.2374715 -0.16582904 -0.08815772
## botswana -0.0434202  0.01918731  0.08430027
## uganda_2  0.1236396  0.18081445  0.22026042
## 
## $comparison
##                     Difference pvalue Signif.       LCL       UCL
## boston - botswana    -40.92614      0     *** -52.12952 -29.72275
## boston - uganda_2    -77.11738      0     *** -88.53261 -65.70215
## botswana - uganda_2  -36.19124      0     *** -47.84305 -24.53943
## 
## $groups
## NULL
## 
## attr(,"class")
## [1] "group"
```

```
rms::orm(formula =  Unifrac1~sample_cohort, data = as_tibble(metadata)) -> orm_univar
print(orm_univar)
```

```
## Logistic (Proportional Odds) Ordinal Regression Model
## 
## rms::orm(formula = Unifrac1 ~ sample_cohort, data = as_tibble(metadata))
## 
##                             Model Likelihood               Discrimination    Rank Discrim.    
##                                   Ratio Test                      Indexes          Indexes    
## Obs                133    LR chi2     134.17    R2                  0.635    rho     0.822    
## Distinct Y         133    d.f.             2    R2(2,133)           0.630                     
## Median Y    0.01232073    Pr(> chi2) <0.0001    R2(2,133)           0.630                     
## max |deriv|      3e-06    Score chi2  127.74    |Pr(Y>=median)-0.5| 0.319                     
##                           Pr(> chi2) <0.0001                                                  
## 
##                        Coef   S.E.   Wald Z Pr(>|Z|)
## sample_cohort=botswana 3.0921 0.4790 6.46   <0.0001 
## sample_cohort=uganda_2 5.8432 0.6030 9.69   <0.0001
```

```
###*** botswana-boston p = <0.0001 uganda-boston p = <0.0001 uganda-bostwana p = <0.0001

# Logistic (Proportional Odds) Ordinal Regression Model
# 
# rms::orm(formula = Unifrac1 ~ sample_cohort, data = as_tibble(metadata))
# 
#                             Model Likelihood               Discrimination    Rank Discrim.    
#                                   Ratio Test                      Indexes          Indexes    
# Obs                133    LR chi2     134.17    R2                  0.635    rho     0.822    
# Distinct Y         133    d.f.             2    R2(2,133)           0.630                     
# Median Y    0.01232073    Pr(> chi2) <0.0001    R2(2,133)           0.630                     
# max |deriv|      3e-06    Score chi2  127.74    |Pr(Y>=median)-0.5| 0.319                     
#                           Pr(> chi2) <0.0001                                                  
# 
#                        Coef   S.E.   Wald Z Pr(>|Z|)
# sample_cohort=botswana 3.0921 0.4790 6.46   <0.0001 
# sample_cohort=uganda_2 5.8432 0.6030 9.69   <0.0001 

### Extra metadata to add that have full n: Race, Ethnicity, age, sex
### Extra metadata to add that have <n:BMI, comorbidities (dm2_hx, hld_hx, htn_hx, cvd_hx, ckd_hx, dm2hx_dx, hldhx_dx, htnhx_dx, cvdhx_dx)
metadata$age <- as.numeric(metadata$age)
metadata$BMI <- as.numeric(metadata$BMI)

### control for metadata with full n
covars_full_n <- c("Ethnicity", "age", "Race", "sample_cohort")
print(vegan::adonis2(as.formula(paste("unifrac.distance~metadata$", paste(covars_full_n, collapse = "+metadata$"), sep = "")), data=ASV_table, permutations=1000)) -> adon_full_n
```

```
## Permutation test for adonis under reduced model
## Terms added sequentially (first to last)
## Permutation: free
## Number of permutations: 1000
## 
## vegan::adonis2(formula = as.formula(paste("unifrac.distance~metadata$", paste(covars_full_n, collapse = "+metadata$"), sep = "")), data = ASV_table, permutations = 1000)
##                         Df SumOfSqs      R2      F   Pr(>F)    
## metadata$Ethnicity       1    0.410 0.00796 1.1158 0.180819    
## metadata$age             1    0.668 0.01297 1.8178 0.000999 ***
## metadata$Race            3    2.902 0.05634 2.6320 0.000999 ***
## metadata$sample_cohort   2    1.589 0.03085 2.1617 0.000999 ***
## Residual               125   45.934 0.89188                    
## Total                  132   51.502 1.00000                    
## ---
## Signif. codes:  0 '***' 0.001 '**' 0.01 '*' 0.05 '.' 0.1 ' ' 1
```

```
###*** sample_cohort r2 = 0.030847 p = 0.000999

#                        Df SumOfSqs      R2      F   Pr(>F)    
# metadata$Ethnicity       1    0.410 0.00796 1.1158 0.180819    
# metadata$age             1    0.668 0.01297 1.8178 0.000999 ***
# metadata$Race            3    2.902 0.05634 2.6320 0.000999 ***
# metadata$sample_cohort   2    1.589 0.03085 2.1617 0.000999 ***
# Residual               125   45.934 0.89188                    
# Total                  132   51.502 1.00000                   

# orm to test boxplot panel
rms::orm(formula =  as.formula(paste("Unifrac1~", paste(covars_full_n, collapse = "+"), sep = "")), data = as_tibble(metadata)) -> orm_full_n
print(orm_full_n)
```

```
## Logistic (Proportional Odds) Ordinal Regression Model
## 
## rms::orm(formula = as.formula(paste("Unifrac1~", paste(covars_full_n, 
##     collapse = "+"), sep = "")), data = as_tibble(metadata))
## 
##                             Model Likelihood               Discrimination    Rank Discrim.    
##                                   Ratio Test                      Indexes          Indexes    
## Obs                133    LR chi2     145.71    R2                  0.666    rho     0.811    
## Distinct Y         133    d.f.             7    R2(7,133)           0.648                     
## Median Y    0.01232073    Pr(> chi2) <0.0001    R2(7,133)           0.648                     
## max |deriv|      3e-05    Score chi2  139.44    |Pr(Y>=median)-0.5| 0.318                     
##                           Pr(> chi2) <0.0001                                                  
## 
##                               Coef    S.E.   Wald Z Pr(>|Z|)
## Ethnicity=Not_Hispanic_Latino -2.9015 1.2774 -2.27  0.0231  
## age                           -0.0291 0.0176 -1.66  0.0979  
## Race=Black_AA                  1.2523 1.2543  1.00  0.3181  
## Race=Varied                    0.4495 1.5148  0.30  0.7666  
## Race=White                    -0.1786 0.9634 -0.19  0.8529  
## sample_cohort=botswana         1.9945 0.9436  2.11  0.0345  
## sample_cohort=uganda_2         5.1854 0.9855  5.26  <0.0001
```

```
###*** botswana-boston p = 0.0345 uganda-boston p = <0.0001 uganda-bostwana p = <0.0001

# Logistic (Proportional Odds) Ordinal Regression Model
# 
# rms::orm(formula = as.formula(paste("Unifrac1~", paste(covars_full_n, 
#     collapse = "+"), sep = "")), data = as_tibble(metadata))
# 
#                             Model Likelihood               Discrimination    Rank Discrim.    
#                                   Ratio Test                      Indexes          Indexes    
# Obs                133    LR chi2     145.71    R2                  0.666    rho     0.811    
# Distinct Y         133    d.f.             7    R2(7,133)           0.648                     
# Median Y    0.01232073    Pr(> chi2) <0.0001    R2(7,133)           0.648                     
# max |deriv|      3e-05    Score chi2  139.44    |Pr(Y>=median)-0.5| 0.318                     
#                           Pr(> chi2) <0.0001                                                  
# 
#                               Coef    S.E.   Wald Z Pr(>|Z|)
# Ethnicity=Not_Hispanic_Latino -2.9015 1.2774 -2.27  0.0231  
# age                           -0.0291 0.0176 -1.66  0.0979  
# Race=Black_AA                  1.2523 1.2543  1.00  0.3181  
# Race=Varied                    0.4495 1.5148  0.30  0.7666  
# Race=White                    -0.1786 0.9634 -0.19  0.8529  
# sample_cohort=botswana         1.9945 0.9436  2.11  0.0345  
# sample_cohort=uganda_2         5.1854 0.9855  5.26  <0.0001 

### remove samples with BMI/comorbidities is NA (n=133 -> n=128)
ps_gg_fp_f_prevalence_filter_2019_05_26_proportion_bc <- ps_gg_fp_f_prevalence_filter_2019_05_26_proportion
metadata_bc<-metadata[!is.na(metadata$BMI) & !is.na(metadata$dm2_hx),,drop=FALSE]
sample_data(ps_gg_fp_f_prevalence_filter_2019_05_26_proportion_bc) <- metadata_bc

### subset ASV table and distance matrix
ASV_table_bc<-as.data.frame(phyloseq::otu_table(ps_gg_fp_f_prevalence_filter_2019_05_26_proportion_bc))
all.equal(row.names(ASV_table_bc), row.names(metadata_bc))
```

```
## [1] TRUE
```

```
unifrac.distance_bc <- unname(usedist::dist_subset(unifrac.distance, sample_names(ps_gg_fp_f_prevalence_filter_2019_05_26_proportion_bc))) ### unname fixes error introduced by Desctools see https://github.com/joey711/phyloseq/issues/1457

### run adonis
covars_bc <- c("BMI", "dm2_hx", "hld_hx", "htn_hx", "cvd_hx", "dm2hx_dx", "hldhx_dx", "htnhx_dx", "cvdhx_dx", covars_full_n)
print(vegan::adonis2(as.formula(paste("unifrac.distance_bc~metadata_bc$", paste(covars_bc, collapse = "+metadata_bc$"), sep = "")), data=ASV_table_bc, permutations=1000)) -> adon_bc
```

```
## Permutation test for adonis under reduced model
## Terms added sequentially (first to last)
## Permutation: free
## Number of permutations: 1000
## 
## vegan::adonis2(formula = as.formula(paste("unifrac.distance_bc~metadata_bc$", paste(covars_bc, collapse = "+metadata_bc$"), sep = "")), data = ASV_table_bc, permutations = 1000)
##                            Df SumOfSqs      R2      F   Pr(>F)    
## metadata_bc$BMI             1    1.090 0.02200 2.9827 0.000999 ***
## metadata_bc$dm2_hx          1    0.413 0.00834 1.1300 0.146853    
## metadata_bc$hld_hx          1    0.364 0.00736 0.9973 0.451548    
## metadata_bc$htn_hx          1    0.395 0.00798 1.0814 0.242757    
## metadata_bc$cvd_hx          1    0.403 0.00814 1.1033 0.194805    
## metadata_bc$dm2hx_dx        1    0.422 0.00852 1.1547 0.113886    
## metadata_bc$hldhx_dx        1    0.470 0.00950 1.2876 0.034965 *  
## metadata_bc$cvdhx_dx        1    0.643 0.01297 1.7586 0.001998 ** 
## metadata_bc$Ethnicity       1    0.368 0.00743 1.0070 0.400599    
## metadata_bc$age             1    0.658 0.01329 1.8020 0.000999 ***
## metadata_bc$Race            3    1.932 0.03900 1.7625 0.000999 ***
## metadata_bc$sample_cohort   2    1.451 0.02930 1.9857 0.000999 ***
## Residual                  112   40.925 0.82618                    
## Total                     127   49.535 1.00000                    
## ---
## Signif. codes:  0 '***' 0.001 '**' 0.01 '*' 0.05 '.' 0.1 ' ' 1
```

```
###*** sample_cohort r2 = 0.029295 p = 0.000999

#                            Df SumOfSqs      R2      F   Pr(>F)    
# metadata_bc$BMI             1    1.090 0.02200 2.9827 0.000999 ***
# metadata_bc$dm2_hx          1    0.413 0.00834 1.1300 0.146853    
# metadata_bc$hld_hx          1    0.364 0.00736 0.9973 0.451548    
# metadata_bc$htn_hx          1    0.395 0.00798 1.0814 0.242757    
# metadata_bc$cvd_hx          1    0.403 0.00814 1.1033 0.194805    
# metadata_bc$dm2hx_dx        1    0.422 0.00852 1.1547 0.113886    
# metadata_bc$hldhx_dx        1    0.470 0.00950 1.2876 0.034965 *  
# metadata_bc$cvdhx_dx        1    0.643 0.01297 1.7586 0.001998 ** 
# metadata_bc$Ethnicity       1    0.368 0.00743 1.0070 0.400599    
# metadata_bc$age             1    0.658 0.01329 1.8020 0.000999 ***
# metadata_bc$Race            3    1.932 0.03900 1.7625 0.000999 ***
# metadata_bc$sample_cohort   2    1.451 0.02930 1.9857 0.000999 ***
# Residual                  112   40.925 0.82618                    
# Total                     127   49.535 1.00000    

# orm to test boxplot panel
rms::orm(formula =  as.formula(paste("Unifrac1~", paste(covars_bc[!covars_bc %in% c("htn_hx")], collapse = "+"), sep = "")), data = as_tibble(metadata)) -> orm_bc
print(orm_bc)
```

```
## Frequencies of Missing Values Due to Each Variable
##      Unifrac1           BMI        dm2_hx        hld_hx        cvd_hx 
##             0             5             5             5             5 
##      dm2hx_dx      hldhx_dx      htnhx_dx      cvdhx_dx     Ethnicity 
##             5             5             5             5             0 
##           age          Race sample_cohort 
##             0             0             0 
## 
## Logistic (Proportional Odds) Ordinal Regression Model
## 
## rms::orm(formula = as.formula(paste("Unifrac1~", paste(covars_bc[!covars_bc %in% 
##     c("htn_hx")], collapse = "+"), sep = "")), data = as_tibble(metadata))
## 
## 
##                             Model Likelihood               Discrimination    Rank Discrim.    
##                                   Ratio Test                      Indexes          Indexes    
## Obs                128    LR chi2     149.10    R2                  0.688    rho     0.830    
## Distinct Y         128    d.f.            15    R2(15,128)          0.649                     
## Median Y    0.01742838    Pr(> chi2) <0.0001    R2(15,128)          0.649                     
## max |deriv|      3e-05    Score chi2  144.64    |Pr(Y>=median)-0.5| 0.317                     
##                           Pr(> chi2) <0.0001                                                  
## 
##                               Coef    S.E.   Wald Z Pr(>|Z|)
## BMI                           -0.0136 0.0471 -0.29  0.7732  
## dm2_hx= 1                      0.6016 1.4678  0.41  0.6819  
## hld_hx= 1                      0.1441 0.9830  0.15  0.8835  
## cvd_hx= 1                     -0.3936 1.3399 -0.29  0.7690  
## dm2hx_dx= 1                   -0.0995 1.0747 -0.09  0.9263  
## hldhx_dx= 1                    0.6688 0.6498  1.03  0.3034  
## htnhx_dx= 1                   -0.0552 0.6250 -0.09  0.9297  
## cvdhx_dx= 1                   -0.4705 0.6150 -0.76  0.4443  
## Ethnicity=Not_Hispanic_Latino -4.0350 1.5527 -2.60  0.0094  
## age                           -0.0363 0.0205 -1.77  0.0768  
## Race=Black_AA                  1.4030 1.2829  1.09  0.2741  
## Race=Varied                    1.0305 1.7439  0.59  0.5546  
## Race=White                    -0.4805 1.0092 -0.48  0.6340  
## sample_cohort=botswana         1.8509 0.9820  1.88  0.0594  
## sample_cohort=uganda_2         5.2919 1.0461  5.06  <0.0001
```

```
###*** botswana-boston p = 0.0594 uganda-boston p = <0.0001 uganda-bostwana p = <0.0001

# Logistic (Proportional Odds) Ordinal Regression Model
# 
# rms::orm(formula = as.formula(paste("Unifrac1~", paste(covars_bc[!covars_bc %in% 
#     c("htn_hx")], collapse = "+"), sep = "")), data = as_tibble(metadata))
# 
# 
#                             Model Likelihood               Discrimination    Rank Discrim.    
#                                   Ratio Test                      Indexes          Indexes    
# Obs                128    LR chi2     149.10    R2                  0.688    rho     0.830    
# Distinct Y         128    d.f.            15    R2(15,128)          0.649                     
# Median Y    0.01742838    Pr(> chi2) <0.0001    R2(15,128)          0.649                     
# max |deriv|      3e-05    Score chi2  144.64    |Pr(Y>=median)-0.5| 0.317                     
#                           Pr(> chi2) <0.0001                                                  
# 
#                               Coef    S.E.   Wald Z Pr(>|Z|)
# BMI                           -0.0136 0.0471 -0.29  0.7732  
# dm2_hx= 1                      0.6016 1.4678  0.41  0.6819  
# hld_hx= 1                      0.1441 0.9830  0.15  0.8835  
# cvd_hx= 1                     -0.3936 1.3399 -0.29  0.7690  
# dm2hx_dx= 1                   -0.0995 1.0747 -0.09  0.9263  
# hldhx_dx= 1                    0.6688 0.6498  1.03  0.3034  
# htnhx_dx= 1                   -0.0552 0.6250 -0.09  0.9297  
# cvdhx_dx= 1                   -0.4705 0.6150 -0.76  0.4443  
# Ethnicity=Not_Hispanic_Latino -4.0350 1.5527 -2.60  0.0094  
# age                           -0.0363 0.0205 -1.77  0.0768  
# Race=Black_AA                  1.4030 1.2829  1.09  0.2741  
# Race=Varied                    1.0305 1.7439  0.59  0.5546  
# Race=White                    -0.4805 1.0092 -0.48  0.6340  
# sample_cohort=botswana         1.8509 0.9820  1.88  0.0594  
# sample_cohort=uganda_2         5.2919 1.0461  5.06  <0.0001 

#--------------------------------------------------------------------------------------------------------------
```

```
#FIGURE B (1B)
#--------------------------------------------------------------------------------------------------------------
#Tax glom at the Family level
ps_gg_fp_f_prevalence_filter_2019_05_26_agglomerate<-phyloseq::tax_glom(ps_gg_fp_f_prevalence_filter_2019_05_26, taxrank="Family")

#Apply filters: min 5000 counts in 50% of the samples
phyloseq::otu_table(ps_gg_fp_f_prevalence_filter_2019_05_26_agglomerate)<-t(phyloseq::otu_table(ps_gg_fp_f_prevalence_filter_2019_05_26_agglomerate))
filter_conditions<-phyloseq::filterfun_sample(function(x) x>=5000)
filtered<-phyloseq::genefilter_sample(phyloseq::otu_table(ps_gg_fp_f_prevalence_filter_2019_05_26_agglomerate), filter_conditions, A=(0.5*nrow(phyloseq::otu_table(ps_gg_fp_f_prevalence_filter_2019_05_26_agglomerate))))
ps_gg_fp_f_prevalence_filter_2019_05_26_agglomerate_filtered<-phyloseq::prune_taxa(filtered, ps_gg_fp_f_prevalence_filter_2019_05_26_agglomerate)

#Select samples of interest (the metadata dataframe has been already been subset before)
phyloseq::sample_data(ps_gg_fp_f_prevalence_filter_2019_05_26_agglomerate_filtered)<-metadata

#Calculate relative abundance
ps_gg_fp_f_prevalence_filter_2019_05_26_agglomerate_filtered_proportion<-phyloseq::transform_sample_counts(ps_gg_fp_f_prevalence_filter_2019_05_26_agglomerate_filtered, function(x)(x/sum(x)))

#Get data out from phyloseq object to plot with ggplot2:
ASV_table<-as.data.frame(t(phyloseq::otu_table(ps_gg_fp_f_prevalence_filter_2019_05_26_agglomerate_filtered_proportion)))
TAX_table<-as.data.frame(as.matrix(ps_gg_fp_f_prevalence_filter_2019_05_26_agglomerate_filtered_proportion@tax_table@.Data))

dim(TAX_table)
```

```
## [1] 7 7
```

```
TAX_table$Name<-paste(TAX_table$Kingdom, TAX_table$Phylum, TAX_table$Class, TAX_table$Order, TAX_table$Family)
all.equal(colnames(ASV_table), row.names(TAX_table))
```

```
## [1] TRUE
```

```
colnames(ASV_table)<- TAX_table$Name

all.equal(row.names(ASV_table), row.names(metadata))
```

```
## [1] TRUE
```

```
ASV_table$sample_cohort<-metadata$sample_cohort
ASV_table$SampleID<-row.names(ASV_table)
ASV_table$hiv_phenotype<-metadata$hiv_phenotype
ASV_table$Unifrac1<-metadata$Unifrac1

#Sort samples by position in Axis1 from Figure 1A
ASV_table<-ASV_table[order(ASV_table$Unifrac1),,drop=FALSE]
ASV_table$SampleID<-factor(ASV_table$SampleID, levels=ASV_table$SampleID)
ASV_table$Unifrac1<-as.factor(ASV_table$Unifrac1)
ASV_table_melt<-reshape::melt(ASV_table)
```

```
## Using sample_cohort, SampleID, hiv_phenotype, Unifrac1 as id variables
```

```
ASV_table_melt$variable<-as.character(ASV_table_melt$variable)
ASV_table_melt$Family<-stringr::str_split_fixed(ASV_table_melt$variable,"_",4)[,1]
ASV_table_melt$Genus<-stringr::str_split_fixed(ASV_table_melt$variable,"_",4)[,2]
ASV_table_melt$Species<-paste(stringr::str_split_fixed(ASV_table_melt$variable,"_",4)[,2],stringr::str_split_fixed(ASV_table_melt$variable,"_",4)[,3])

#Kruskal Wallis test for differential abundance between cohorts
family<-c()
all_sig<-c() #We are interested in those families have all pairwise comparisons statistically significant:
for (i in colnames(ASV_table)[1:(dim(ASV_table)[2]-4)]){
  result<-kruskal.test(ASV_table[[i]],ASV_table[["sample_cohort"]])
  if (result$p.value<=0.01){
    print(i)
    result2<-agricolae::kruskal(ASV_table[[i]],ASV_table[["sample_cohort"]],group=FALSE, p.adj ="BH")
    if (result2$comparison$pvalue[1]<0.1 & result2$comparison$pvalue[2]<0.1 & result2$comparison$pvalue[3]<0.1){
      all_sig<-c(all_sig, i)
    }
    family<-c(family, i)
  }
}
```

```
## [1] "Bacteria Actinobacteria Actinobacteria Bifidobacteriales Bifidobacteriaceae"
## [1] "Bacteria Verrucomicrobia Verrucomicrobiae Verrucomicrobiales Verrucomicrobiaceae"
## [1] "Bacteria Bacteroidetes Bacteroidia Bacteroidales Bacteroidaceae"
## [1] "Bacteria Bacteroidetes Bacteroidia Bacteroidales Prevotellaceae"
## [1] "Bacteria Firmicutes Clostridia Clostridiales Veillonellaceae"
## [1] "Bacteria Firmicutes Clostridia Clostridiales Lachnospiraceae"
```

```
# Add all metadata to the table for multivariate testing of abundance differences
metadata$age <- as.numeric(metadata$age)
metadata$BMI <- as.numeric(metadata$BMI)
dplyr::left_join(ASV_table[colnames(ASV_table) %in% c("SampleID", "Unifrac1") | !colnames(ASV_table) %in% colnames(metadata)], metadata[, !colnames(metadata) %in% c("Unifrac1")], by = "SampleID") -> ASV_table_full_metadata

# Run orm (n=133) and compare family abundances between sample_cohort
rms::orm(formula =  as.formula(paste("ASV_table_full_metadata[[", which(stringr::str_detect(colnames(ASV_table_full_metadata), "Bacteroidaceae")), "]]~", "sample_cohort", sep = "")), data = ASV_table_full_metadata) -> orm_abund_bact
print(orm_abund_bact)
```

```
## Logistic (Proportional Odds) Ordinal Regression Model
## 
## rms::orm(formula = as.formula(paste("ASV_table_full_metadata[[", 
##     which(stringr::str_detect(colnames(ASV_table_full_metadata), 
##         "Bacteroidaceae")), "]]~", "sample_cohort", sep = "")), 
##     data = ASV_table_full_metadata)
## 
##                             Model Likelihood               Discrimination    Rank Discrim.    
##                                   Ratio Test                      Indexes          Indexes    
## Obs                133    LR chi2     101.71    R2                  0.535    rho     0.758    
## Distinct Y         109    d.f.             2    R2(2,133)           0.527                     
## Median Y    0.01153122    Pr(> chi2) <0.0001    R2(2,132.1)         0.530                     
## max |deriv|     0.0002    Score chi2   99.06    |Pr(Y>=median)-0.5| 0.287                     
##                           Pr(> chi2) <0.0001                                                  
## 
##                        Coef    S.E.   Wald Z Pr(>|Z|)
## sample_cohort=botswana -2.2249 0.4152 -5.36  <0.0001 
## sample_cohort=uganda_2 -4.6457 0.5227 -8.89  <0.0001
```

```
###*** botswana-boston p = <0.0001 uganda-boston p = <0.0001 uganda-bostwana p = <0.0001
rms::orm(formula =  as.formula(paste("ASV_table_full_metadata[[", which(stringr::str_detect(colnames(ASV_table_full_metadata), "Prevotellaceae")), "]]~", "sample_cohort", sep = "")), data = ASV_table_full_metadata) -> orm_abund_prev
print(orm_abund_prev)
```

```
## Logistic (Proportional Odds) Ordinal Regression Model
## 
## rms::orm(formula = as.formula(paste("ASV_table_full_metadata[[", 
##     which(stringr::str_detect(colnames(ASV_table_full_metadata), 
##         "Prevotellaceae")), "]]~", "sample_cohort", sep = "")), 
##     data = ASV_table_full_metadata)
## 
##                           Model Likelihood               Discrimination    Rank Discrim.    
##                                 Ratio Test                      Indexes          Indexes    
## Obs              133    LR chi2      30.58    R2                  0.205    rho     0.457    
## Distinct Y       124    d.f.             2    R2(2,133)           0.193                     
## Median Y    0.246429    Pr(> chi2) <0.0001    R2(2,132.9)         0.193                     
## max |deriv|   0.0001    Score chi2   30.42    |Pr(Y>=median)-0.5| 0.173                     
##                         Pr(> chi2) <0.0001                                                  
## 
##                        Coef   S.E.   Wald Z Pr(>|Z|)
## sample_cohort=botswana 0.9486 0.3769 2.52   0.0118  
## sample_cohort=uganda_2 2.1627 0.4053 5.34   <0.0001
```

```
###*** botswana-boston p = 0.0118 uganda-boston p = <0.0001 uganda-bostwana p = 0.0015

### Extra metadata to add that have full n: Race, Ethnicity, age, sex
### Extra metadata to add that have <n:BMI, comorbidities (dm2_hx, hld_hx, htn_hx, cvd_hx, ckd_hx, dm2hx_dx, hldhx_dx, htnhx_dx, cvdhx_dx)
### control for metadata with full n
covars_full_n <- c("Ethnicity", "age", "Race", "sample_cohort")
rms::orm(formula =  as.formula(paste("ASV_table_full_metadata[[", which(stringr::str_detect(colnames(ASV_table_full_metadata), "Bacteroidaceae")), "]]~", paste(covars_full_n, collapse = "+"), sep = "")), data = ASV_table_full_metadata) -> orm_abund_bact_full_n
print(orm_abund_bact_full_n)
```

```
## Logistic (Proportional Odds) Ordinal Regression Model
## 
## rms::orm(formula = as.formula(paste("ASV_table_full_metadata[[", 
##     which(stringr::str_detect(colnames(ASV_table_full_metadata), 
##         "Bacteroidaceae")), "]]~", paste(covars_full_n, collapse = "+"), 
##     sep = "")), data = ASV_table_full_metadata)
## 
##                             Model Likelihood               Discrimination    Rank Discrim.    
##                                   Ratio Test                      Indexes          Indexes    
## Obs                133    LR chi2     106.89    R2                  0.552    rho     0.738    
## Distinct Y         109    d.f.             7    R2(7,133)           0.528                     
## Median Y    0.01153122    Pr(> chi2) <0.0001    R2(7,132.1)         0.531                     
## max |deriv|     0.0002    Score chi2  104.34    |Pr(Y>=median)-0.5| 0.288                     
##                           Pr(> chi2) <0.0001                                                  
## 
##                               Coef    S.E.   Wald Z Pr(>|Z|)
## Ethnicity=Not_Hispanic_Latino  0.9628 1.3026  0.74  0.4598  
## age                            0.0112 0.0181  0.62  0.5365  
## Race=Black_AA                 -2.3078 1.4426 -1.60  0.1097  
## Race=Varied                   -1.4493 1.7956 -0.81  0.4196  
## Race=White                    -0.8961 1.2564 -0.71  0.4757  
## sample_cohort=botswana        -0.9934 0.8067 -1.23  0.2181  
## sample_cohort=uganda_2        -3.5703 0.8230 -4.34  <0.0001
```

```
###*** botswana-boston p = 0.2181 uganda-boston p = <0.0001 uganda-bostwana p = <0.0001
rms::orm(formula =  as.formula(paste("ASV_table_full_metadata[[", which(stringr::str_detect(colnames(ASV_table_full_metadata), "Prevotellaceae")), "]]~", paste(covars_full_n, collapse = "+"), sep = "")), data = ASV_table_full_metadata) -> orm_abund_prev_full_n
print(orm_abund_prev_full_n)
```

```
## Logistic (Proportional Odds) Ordinal Regression Model
## 
## rms::orm(formula = as.formula(paste("ASV_table_full_metadata[[", 
##     which(stringr::str_detect(colnames(ASV_table_full_metadata), 
##         "Prevotellaceae")), "]]~", paste(covars_full_n, collapse = "+"), 
##     sep = "")), data = ASV_table_full_metadata)
## 
##                           Model Likelihood               Discrimination    Rank Discrim.    
##                                 Ratio Test                      Indexes          Indexes    
## Obs              133    LR chi2      36.82    R2                  0.242    rho     0.502    
## Distinct Y       124    d.f.             7    R2(7,133)           0.201                     
## Median Y    0.246429    Pr(> chi2) <0.0001    R2(7,132.9)         0.201                     
## max |deriv|    0.001    Score chi2   36.71    |Pr(Y>=median)-0.5| 0.182                     
##                         Pr(> chi2) <0.0001                                                  
## 
##                               Coef    S.E.   Wald Z Pr(>|Z|)
## Ethnicity=Not_Hispanic_Latino -1.8477 1.5784 -1.17  0.2418  
## age                           -0.0175 0.0182 -0.96  0.3355  
## Race=Black_AA                  2.5460 1.4559  1.75  0.0803  
## Race=Varied                   -0.3881 1.9732 -0.20  0.8441  
## Race=White                     0.6528 1.2251  0.53  0.5942  
## sample_cohort=botswana        -0.9465 0.8815 -1.07  0.2829  
## sample_cohort=uganda_2         0.5524 0.8319  0.66  0.5067
```

```
###*** botswana-boston p = 0.2829 uganda-boston p = 0.5067 uganda-bostwana p = 0.0014

ASV_table_melt_significant<-ASV_table_melt[ASV_table_melt$variable%in%all_sig,,drop=FALSE]
unique(ASV_table_melt_significant$variable)
```

```
## [1] "Bacteria Bacteroidetes Bacteroidia Bacteroidales Bacteroidaceae"
## [2] "Bacteria Bacteroidetes Bacteroidia Bacteroidales Prevotellaceae"
```

```
ordered_names<-c(names(sort(colSums(ASV_table[1:(dim(ASV_table)[2]-5)]), decreasing=FALSE)))

ASV_table_melt_significant$variable<-factor(ASV_table_melt_significant$variable, levels=ordered_names)
ASV_table_melt_significant$Unifrac1<-as.numeric(as.character(ASV_table_melt_significant$Unifrac1))

plot1<-ggplot(data=ASV_table_melt_significant, aes(x=Unifrac1, y=value))+geom_point(aes(color=sample_cohort), size=2.5)+
         stat_smooth(geom = "area", method = "loess", alpha = 0.5, size = 1,fill="grey60")+theme_bw()+
         facet_wrap(~variable, scales="free_y", ncol=1)+
         theme(legend.position = "left", axis.text.x = element_text(size=3), panel.grid.major.x=element_blank())+
         scale_colour_manual(values=c("royalblue4","darkorange","forestgreen"))
```

```
## Warning: Using `size` aesthetic for lines was deprecated in ggplot2 3.4.0.
## ℹ Please use `linewidth` instead.
```

```
ggsave("SupplementaryFigure2B.pdf", plot1)
```

```
## Saving 7 x 5 in image
```

```
## `geom_smooth()` using formula = 'y ~ x'
```

```
#--------------------------------------------------------------------------------------------------------------
```

```
#FIGURE C (2A)
#--------------------------------------------------------------------------------------------------------------
#COMPARISON 1 NEG vs ART
#Transform count data in the phyloseq object
ps_gg_fp_f_prevalence_filter_2019_05_26_proportion<-phyloseq::transform_sample_counts(ps_gg_fp_f_prevalence_filter_2019_05_26, function(x)(x/sum(x)))

#Select samples of interest and update phyloseq object 
metadata<-phyloseq::sample_data(ps_gg_fp_f_prevalence_filter_2019_05_26)
metadata<-metadata[metadata$hiv_phenotype%in%c("1_hiv_negative","2_suppressed"),,drop=F]
metadata<-as.data.frame(as.matrix(metadata[metadata$sexual_orientation!="MSM",,drop=FALSE]))
metadata<-metadata[metadata$sex=="male",,drop=F]
phyloseq::sample_data(ps_gg_fp_f_prevalence_filter_2019_05_26_proportion)<-metadata

#Run PCoA on the phyloseq object
ordination<-phyloseq::ordinate(ps_gg_fp_f_prevalence_filter_2019_05_26_proportion, "PCoA", "unifrac")
```

```
## Warning in matrix(tree$edge[order(tree$edge[, 1]), ][, 2], byrow = TRUE, : data
## length [8987] is not a sub-multiple or multiple of the number of rows [4494]
```

```
ordination$values[1:2,]
```

```
##   Eigenvalues Relative_eig Broken_stick  Cumul_eig Cumul_br_stick
## 1    5.073451   0.05841664   0.02704814 0.05841664     0.02704814
## 2    4.606459   0.05303961   0.02252325 0.11145626     0.04957139
```

```
metadata_ordered<-metadata[row.names(ordination$vectors),,drop=FALSE]

all.equal(row.names(metadata_ordered), row.names(ordination$vectors))
```

```
## [1] TRUE
```

```
metadata_ordered$Unifrac1<-ordination$vectors[,1]
metadata_ordered$Unifrac2<-ordination$vectors[,2]

comparison1<-metadata_ordered
comparison1$Grouping<-as.factor(paste(comparison1$hiv_phenotype, comparison1$sample_cohort, sep="_"))

#Let's plot the data
us<-comparison1[comparison1$sample_cohort=="boston",,drop=F]
botswana<-comparison1[comparison1$sample_cohort=="botswana",,drop=F]
uganda<-comparison1[comparison1$sample_cohort=="uganda_2",,drop=F]

plot_us_comparison<-ggplot(data=us, aes(x=Unifrac1, y=Unifrac2))+geom_point(color="royalblue4", aes(alpha=hiv_phenotype), size=2, shape=16)+
  theme_bw()+stat_ellipse(color="royalblue4", aes(alpha=hiv_phenotype), size=1)+scale_alpha_manual(values=c(1,0.3))+ylim(c(-0.5, 0.5))+xlim(c(-0.5,0.5))+
  ggtitle("neg-art boston")+geom_point(data=us %>% group_by(hiv_phenotype) %>% summarise_at(vars(matches("UniFrac")), mean),size=5, color="royalblue4", aes(alpha=hiv_phenotype))
plot_botswana_comparison<-ggplot(data=botswana, aes(x=Unifrac1, y=Unifrac2))+geom_point(color="darkorange", aes(alpha=hiv_phenotype), size=2, shape=16)+
  theme_bw()+stat_ellipse(color="darkorange", aes(alpha=hiv_phenotype), size=1)+scale_alpha_manual(values=c(1,0.3))+ylim(c(-0.5, 0.5))+xlim(c(-0.5,0.5))+
  ggtitle("neg-art botswana")+geom_point(data=botswana %>% group_by(hiv_phenotype) %>% summarise_at(vars(matches("UniFrac")), mean),size=5, color="darkorange", aes(alpha=hiv_phenotype))
plot_uganda_comparison<-ggplot(data=uganda, aes(x=Unifrac1, y=Unifrac2))+geom_point(color="forestgreen", aes(alpha=hiv_phenotype), size=2, shape=16)+
  theme_bw()+stat_ellipse(color="forestgreen", aes(alpha=hiv_phenotype), size=1)+scale_alpha_manual(values=c(1,0.3))+ylim(c(-0.5, 0.5))+xlim(c(-0.5,0.5))+
  ggtitle("neg-art uganda")+geom_point(data=uganda %>% group_by(hiv_phenotype) %>% summarise_at(vars(matches("UniFrac")), mean),size=5, color="forestgreen", aes(alpha=hiv_phenotype))

ggsave("SupplementaryFigure2C_1.pdf", grid.arrange(plot_us_comparison,plot_botswana_comparison,plot_uganda_comparison, nrow=3, ncol=3), width=15, height=10)
```

```
#Adonis (n = 222)
ASV_table<-as.data.frame(phyloseq::otu_table(ps_gg_fp_f_prevalence_filter_2019_05_26_proportion))
all.equal(row.names(ASV_table), row.names(metadata_ordered))
```

```
## [1] TRUE
```

```
unifrac.distance<-unname(phyloseq::UniFrac(ps_gg_fp_f_prevalence_filter_2019_05_26_proportion, weighted = FALSE)) ### unname fixes error introduced by Desctools see https://github.com/joey711/phyloseq/issues/1457
```

```
## Warning in matrix(tree$edge[order(tree$edge[, 1]), ][, 2], byrow = TRUE, : data
## length [8987] is not a sub-multiple or multiple of the number of rows [4494]
```

```
attributes(unifrac.distance)$Labels <- phyloseq::sample_names(ps_gg_fp_f_prevalence_filter_2019_05_26_proportion)
print(vegan::adonis2(unifrac.distance~metadata_ordered$hiv_phenotype, data=ASV_table, permutations=1000)) -> adon_hiv_phenotype
```

```
## Permutation test for adonis under reduced model
## Terms added sequentially (first to last)
## Permutation: free
## Number of permutations: 1000
## 
## vegan::adonis2(formula = unifrac.distance ~ metadata_ordered$hiv_phenotype, data = ASV_table, permutations = 1000)
##                                 Df SumOfSqs     R2      F   Pr(>F)    
## metadata_ordered$hiv_phenotype   1    0.860 0.0099 2.1995 0.000999 ***
## Residual                       220   85.990 0.9901                    
## Total                          221   86.849 1.0000                    
## ---
## Signif. codes:  0 '***' 0.001 '**' 0.01 '*' 0.05 '.' 0.1 ' ' 1
```

```
###*** hiv_phenotype r2 = 0.009899 p = 0.000999

# vegan::adonis2(formula = unifrac.distance ~ metadata_ordered$hiv_phenotype, data = ASV_table, permutations = 1000)
#                                 Df SumOfSqs       R2       F   Pr(>F)    
# metadata_ordered$hiv_phenotype   1   0.8597 0.009899 2.19946 0.000999 ***
# Residual                       220  85.9897 0.990101                     
# Total                          221  86.8494 1.000000                     

print(vegan::adonis2(unifrac.distance~metadata_ordered$sample_cohort, data=ASV_table, permutations=1000)) -> adon_cohort
```

```
## Permutation test for adonis under reduced model
## Terms added sequentially (first to last)
## Permutation: free
## Number of permutations: 1000
## 
## vegan::adonis2(formula = unifrac.distance ~ metadata_ordered$sample_cohort, data = ASV_table, permutations = 1000)
##                                 Df SumOfSqs     R2      F   Pr(>F)    
## metadata_ordered$sample_cohort   2    4.829 0.0556 6.4472 0.000999 ***
## Residual                       219   82.020 0.9444                    
## Total                          221   86.849 1.0000                    
## ---
## Signif. codes:  0 '***' 0.001 '**' 0.01 '*' 0.05 '.' 0.1 ' ' 1
```

```
###*** sample_cohort r2 = 0.055605 p = 0.000999

# vegan::adonis2(formula = unifrac.distance ~ metadata_ordered$sample_cohort, data = ASV_table, permutations = 1000)
#                                 Df SumOfSqs       R2       F   Pr(>F)    
# metadata_ordered$sample_cohort   2   4.8292 0.055605 6.44721 0.000999 ***
# Residual                       219  82.0202 0.944395                     
# Total                          221  86.8494 1.000000                 

print(vegan::adonis2(unifrac.distance~metadata_ordered$hiv_phenotype+metadata_ordered$sample_cohort, data=ASV_table, permutations=1000)) -> adon_cohort_phenotype
```

```
## Permutation test for adonis under reduced model
## Terms added sequentially (first to last)
## Permutation: free
## Number of permutations: 1000
## 
## vegan::adonis2(formula = unifrac.distance ~ metadata_ordered$hiv_phenotype + metadata_ordered$sample_cohort, data = ASV_table, permutations = 1000)
##                                 Df SumOfSqs      R2      F   Pr(>F)    
## metadata_ordered$hiv_phenotype   1    0.860 0.00990 2.3065 0.000999 ***
## metadata_ordered$sample_cohort   2    4.737 0.05454 6.3540 0.000999 ***
## Residual                       218   81.253 0.93556                    
## Total                          221   86.849 1.00000                    
## ---
## Signif. codes:  0 '***' 0.001 '**' 0.01 '*' 0.05 '.' 0.1 ' ' 1
```

```
###*** sample_cohort r2 = 0.054537 p = 0.000999, hiv_phenotype r2 = 0.009899 p = 0.000999

# vegan::adonis2(formula = unifrac.distance ~ metadata_ordered$hiv_phenotype + metadata_ordered$sample_cohort, data = ASV_table, permutations = 1000)
#                                 Df SumOfSqs       R2       F   Pr(>F)    
# metadata_ordered$hiv_phenotype   1   0.8597 0.009899 2.30651 0.000999 ***
# metadata_ordered$sample_cohort   2   4.7365 0.054537 6.35396 0.000999 ***
# Residual                       218  81.2532 0.935564                     
# Total                          221  86.8494 1.000000              

### Extra metadata that have full n: Race, Ethnicity, age, sex, current_art_class_consolid2, tmp_smx_active
### Extra metadata that have <n: BMI, comorbidities (dm2_hx, hld_hx, htn_hx, cvd_hx, ckd_hx, cvd_dx [missing boston], dm2hx_dx, hldhx_dx, htnhx_dx, cvdhx_dx, ever_smoke, current_smoke, smoke_cat), school_level [uganda2 only], monthly_income[uganda2 only], current_art_class_consolid2, tmp_smx_active, days_on_art, sexual_orientation
### additional: smoking_years, fram_10yr_risk_lab, fram_10yr_risk_nonlab, mean_imt, total_plaques, any_plaques
metadata_ordered$age <- as.numeric(metadata_ordered$age)
metadata_ordered$BMI <- as.numeric(metadata_ordered$BMI)
metadata_ordered$days_on_art <- as.numeric(metadata_ordered$days_on_art)

### control for metadata with full n
covars_full_n <- c("Ethnicity", "age", "Race", "hiv_phenotype", "tmp_smx_active", "current_art_class_consolid2", "sample_cohort")
print(vegan::adonis2(as.formula(paste("unifrac.distance~metadata_ordered$", paste(covars_full_n, collapse = "+metadata_ordered$"), sep = "")), data=ASV_table, permutations=1000)) -> adon_full_n
```

```
## Permutation test for adonis under reduced model
## Terms added sequentially (first to last)
## Permutation: free
## Number of permutations: 1000
## 
## vegan::adonis2(formula = as.formula(paste("unifrac.distance~metadata_ordered$", paste(covars_full_n, collapse = "+metadata_ordered$"), sep = "")), data = ASV_table, permutations = 1000)
##                                               Df SumOfSqs      R2      F
## metadata_ordered$Ethnicity                     1    0.402 0.00463 1.0860
## metadata_ordered$age                           1    0.951 0.01095 2.5668
## metadata_ordered$Race                          3    3.096 0.03564 2.7846
## metadata_ordered$hiv_phenotype                 1    0.786 0.00906 2.1223
## metadata_ordered$tmp_smx_active                1    1.007 0.01159 2.7172
## metadata_ordered$current_art_class_consolid2   3    1.415 0.01629 1.2727
## metadata_ordered$sample_cohort                 2    1.740 0.02004 2.3479
## Residual                                     209   77.452 0.89179       
## Total                                        221   86.849 1.00000       
##                                                Pr(>F)    
## metadata_ordered$Ethnicity                   0.250749    
## metadata_ordered$age                         0.000999 ***
## metadata_ordered$Race                        0.000999 ***
## metadata_ordered$hiv_phenotype               0.000999 ***
## metadata_ordered$tmp_smx_active              0.000999 ***
## metadata_ordered$current_art_class_consolid2 0.002997 ** 
## metadata_ordered$sample_cohort               0.000999 ***
## Residual                                                 
## Total                                                    
## ---
## Signif. codes:  0 '***' 0.001 '**' 0.01 '*' 0.05 '.' 0.1 ' ' 1
```

```
###*** sample_cohort r2 = 0.020036 p = 0.000999, hiv_phenotype r2 = 0.009056 p = 0.000999

#                                               Df SumOfSqs      R2      F   Pr(>F)    
# metadata_ordered$Ethnicity                     1    0.402 0.00463 1.0860 0.250749    
# metadata_ordered$age                           1    0.951 0.01095 2.5668 0.000999 ***
# metadata_ordered$Race                          3    3.096 0.03564 2.7846 0.000999 ***
# metadata_ordered$hiv_phenotype                 1    0.786 0.00906 2.1223 0.000999 ***
# metadata_ordered$tmp_smx_active                1    1.007 0.01159 2.7172 0.000999 ***
# metadata_ordered$current_art_class_consolid2   3    1.415 0.01629 1.2727 0.002997 ** 
# metadata_ordered$sample_cohort                 2    1.740 0.02004 2.3479 0.000999 ***
# Residual                                     209   77.452 0.89179                    
# Total                                        221   86.849 1.00000                    

### remove samples with BMI/comorbidities/days_on_art is NA (n=222 -> n=215)
ps_gg_fp_f_prevalence_filter_2019_05_26_proportion_bcd <- ps_gg_fp_f_prevalence_filter_2019_05_26_proportion
metadata_ordered_bcd <- metadata_ordered[!is.na(metadata_ordered$dm2_hx) & !is.na(metadata_ordered$days_on_art) & !is.na(metadata_ordered$BMI),,drop=FALSE]
sample_data(ps_gg_fp_f_prevalence_filter_2019_05_26_proportion_bcd) <- metadata_ordered_bcd

### subset ASV table and distance matrix
ASV_table_bcd<-as.data.frame(otu_table(ps_gg_fp_f_prevalence_filter_2019_05_26_proportion_bcd))
all.equal(row.names(ASV_table_bcd), row.names(metadata_ordered_bcd))
```

```
## [1] TRUE
```

```
unifrac.distance_bcd <- unname(usedist::dist_subset(unifrac.distance, sample_names(ps_gg_fp_f_prevalence_filter_2019_05_26_proportion_bcd))) ### unname fixes error introduced by Desctools see https://github.com/joey711/phyloseq/issues/1457

### run adonis
covars_bcd <- c("BMI", "dm2_hx", "hld_hx", "htn_hx", "cvd_hx", "dm2hx_dx", "hldhx_dx", "cvdhx_dx", "days_on_art", covars_full_n)
print(vegan::adonis2(as.formula(paste("unifrac.distance_bcd~metadata_ordered_bcd$", paste(covars_bcd, collapse = "+metadata_ordered_bcd$"), sep = "")), data=ASV_table_bcd, permutations=1000)) -> adon_full_n_bcd
```

```
## Permutation test for adonis under reduced model
## Terms added sequentially (first to last)
## Permutation: free
## Number of permutations: 1000
## 
## vegan::adonis2(formula = as.formula(paste("unifrac.distance_bcd~metadata_ordered_bcd$", paste(covars_bcd, collapse = "+metadata_ordered_bcd$"), sep = "")), data = ASV_table_bcd, permutations = 1000)
##                                                   Df SumOfSqs      R2      F
## metadata_ordered_bcd$BMI                           1    1.324 0.01574 3.5871
## metadata_ordered_bcd$dm2_hx                        1    0.400 0.00475 1.0827
## metadata_ordered_bcd$hld_hx                        1    0.381 0.00452 1.0309
## metadata_ordered_bcd$htn_hx                        1    0.357 0.00424 0.9658
## metadata_ordered_bcd$cvd_hx                        1    0.471 0.00560 1.2751
## metadata_ordered_bcd$dm2hx_dx                      1    0.423 0.00502 1.1450
## metadata_ordered_bcd$hldhx_dx                      1    0.530 0.00630 1.4347
## metadata_ordered_bcd$cvdhx_dx                      1    0.712 0.00847 1.9296
## metadata_ordered_bcd$days_on_art                   1    0.734 0.00872 1.9872
## metadata_ordered_bcd$Ethnicity                     1    0.363 0.00431 0.9831
## metadata_ordered_bcd$age                           1    0.784 0.00932 2.1229
## metadata_ordered_bcd$Race                          3    2.160 0.02567 1.9497
## metadata_ordered_bcd$hiv_phenotype                 1    0.471 0.00560 1.2755
## metadata_ordered_bcd$tmp_smx_active                1    0.754 0.00896 2.0426
## metadata_ordered_bcd$current_art_class_consolid2   3    1.427 0.01696 1.2882
## metadata_ordered_bcd$sample_cohort                 2    1.593 0.01894 2.1577
## Residual                                         193   71.259 0.84690       
## Total                                            214   84.141 1.00000       
##                                                    Pr(>F)    
## metadata_ordered_bcd$BMI                         0.000999 ***
## metadata_ordered_bcd$dm2_hx                      0.228771    
## metadata_ordered_bcd$hld_hx                      0.333666    
## metadata_ordered_bcd$htn_hx                      0.518482    
## metadata_ordered_bcd$cvd_hx                      0.033966 *  
## metadata_ordered_bcd$dm2hx_dx                    0.140859    
## metadata_ordered_bcd$hldhx_dx                    0.008991 ** 
## metadata_ordered_bcd$cvdhx_dx                    0.000999 ***
## metadata_ordered_bcd$days_on_art                 0.000999 ***
## metadata_ordered_bcd$Ethnicity                   0.496503    
## metadata_ordered_bcd$age                         0.000999 ***
## metadata_ordered_bcd$Race                        0.000999 ***
## metadata_ordered_bcd$hiv_phenotype               0.030969 *  
## metadata_ordered_bcd$tmp_smx_active              0.000999 ***
## metadata_ordered_bcd$current_art_class_consolid2 0.000999 ***
## metadata_ordered_bcd$sample_cohort               0.000999 ***
## Residual                                                     
## Total                                                        
## ---
## Signif. codes:  0 '***' 0.001 '**' 0.01 '*' 0.05 '.' 0.1 ' ' 1
```

```
###*** sample_cohort r2 = 0.018936 p = 0.000999, hiv_phenotype r2 = 0.005597 p = 0.045954

#                                                  Df SumOfSqs      R2      F   Pr(>F)    
# metadata_ordered_bcd$BMI                           1    1.324 0.01574 3.5871 0.000999 ***
# metadata_ordered_bcd$dm2_hx                        1    0.400 0.00475 1.0827 0.228771    
# metadata_ordered_bcd$hld_hx                        1    0.381 0.00452 1.0309 0.333666    
# metadata_ordered_bcd$htn_hx                        1    0.357 0.00424 0.9658 0.518482    
# metadata_ordered_bcd$cvd_hx                        1    0.471 0.00560 1.2751 0.033966 *  
# metadata_ordered_bcd$dm2hx_dx                      1    0.423 0.00502 1.1450 0.140859    
# metadata_ordered_bcd$hldhx_dx                      1    0.530 0.00630 1.4347 0.008991 ** 
# metadata_ordered_bcd$cvdhx_dx                      1    0.712 0.00847 1.9296 0.000999 ***
# metadata_ordered_bcd$days_on_art                   1    0.734 0.00872 1.9872 0.000999 ***
# metadata_ordered_bcd$Ethnicity                     1    0.363 0.00431 0.9831 0.496503    
# metadata_ordered_bcd$age                           1    0.784 0.00932 2.1229 0.000999 ***
# metadata_ordered_bcd$Race                          3    2.160 0.02567 1.9497 0.000999 ***
# metadata_ordered_bcd$hiv_phenotype                 1    0.471 0.00560 1.2755 0.030969 *  
# metadata_ordered_bcd$tmp_smx_active                1    0.754 0.00896 2.0426 0.000999 ***
# metadata_ordered_bcd$current_art_class_consolid2   3    1.427 0.01696 1.2882 0.000999 ***
# metadata_ordered_bcd$sample_cohort                 2    1.593 0.01894 2.1577 0.000999 ***
# Residual                                         193   71.259 0.84690                    
# Total                                            214   84.141 1.00000                 

#COMPARISON 2 NEG vs UNTREAT
#Transform count data in the phyloseq object
ps_gg_fp_f_prevalence_filter_2019_05_26_proportion<-phyloseq::transform_sample_counts(ps_gg_fp_f_prevalence_filter_2019_05_26, function(x)(x/sum(x)))

#Select samples of interest and update phyloseq object 
metadata<-phyloseq::sample_data(ps_gg_fp_f_prevalence_filter_2019_05_26)
metadata<-metadata[metadata$hiv_phenotype%in%c("1_hiv_negative","4_unsuppressed"),,drop=F]
metadata<-as.data.frame(as.matrix(metadata[metadata$sexual_orientation!="MSM",,drop=F]))
metadata<-metadata[metadata$sample_cohort%in%c("botswana", "boston"),,drop=F]
metadata<-metadata[metadata$sex=="male",,drop=F]
phyloseq::sample_data(ps_gg_fp_f_prevalence_filter_2019_05_26_proportion)<-metadata

#Run PCoA on the phyloseq object
ordination<-phyloseq::ordinate(ps_gg_fp_f_prevalence_filter_2019_05_26_proportion, "PCoA", "unifrac")
```

```
## Warning in matrix(tree$edge[order(tree$edge[, 1]), ][, 2], byrow = TRUE, : data
## length [8987] is not a sub-multiple or multiple of the number of rows [4494]
```

```
ordination$values[1:2,]
```

```
##   Eigenvalues Relative_eig Broken_stick Cumul_eig Cumul_br_stick
## 1    2.936624   0.06781010   0.04664731 0.0678101     0.04664731
## 2    2.460202   0.05680895   0.03787538 0.1246190     0.08452270
```

```
metadata_ordered<-metadata[row.names(ordination$vectors),,drop=FALSE]

all.equal(row.names(metadata_ordered), row.names(ordination$vectors))
```

```
## [1] TRUE
```

```
metadata_ordered$Unifrac1<-ordination$vectors[,1]
metadata_ordered$Unifrac2<-ordination$vectors[,2]

comparison2<-metadata_ordered
comparison2$Grouping<-as.factor(paste(comparison2$hiv_phenotype, comparison2$sample_cohort, sep="_"))

#Let's plot the data
us<-comparison2[comparison2$sample_cohort=="boston",,drop=F]
botswana<-comparison2[comparison2$sample_cohort=="botswana",,drop=F]

plot_us_comparison<-ggplot(data=us, aes(x=Unifrac1, y=Unifrac2))+geom_point(color="royalblue4", aes(alpha=hiv_phenotype), size=2, shape=16)+
  theme_bw()+stat_ellipse(color="royalblue4", aes(alpha=hiv_phenotype), size=1)+scale_alpha_manual(values=c(1,0.3))+ylim(c(-0.9, 0.9))+xlim(c(-0.9,0.9))+
  ggtitle("neg-naive us")+geom_point(data=us %>% group_by(hiv_phenotype) %>% summarise_at(vars(matches("UniFrac")), mean),size=5, color="royalblue4", aes(alpha=hiv_phenotype))
plot_botswana_comparison<-ggplot(data=botswana, aes(x=Unifrac1, y=Unifrac2))+geom_point(color="darkorange", aes(alpha=hiv_phenotype), size=2, shape=16)+
  theme_bw()+stat_ellipse(color="darkorange", aes(alpha=hiv_phenotype), size=1)+scale_alpha_manual(values=c(1,0.3))+ylim(c(-0.9, 0.9))+xlim(c(-0.9,0.9))+
  ggtitle("neg-naive botswana")+geom_point(data=botswana %>% group_by(hiv_phenotype) %>% summarise_at(vars(matches("UniFrac")), mean),size=5, color="darkorange", aes(alpha=hiv_phenotype))

ggsave("SupplementaryFigure2C_2.pdf", grid.arrange(plot_us_comparison,plot_botswana_comparison, nrow=3, ncol=3), width=15, height=10)
```

```
#Adonis (n=115)
ASV_table<-as.data.frame(phyloseq::otu_table(ps_gg_fp_f_prevalence_filter_2019_05_26_proportion))
all.equal(row.names(ASV_table), row.names(metadata_ordered))
```

```
## [1] TRUE
```

```
unifrac.distance<-unname(phyloseq::UniFrac(ps_gg_fp_f_prevalence_filter_2019_05_26_proportion, weighted = FALSE)) ### unname fixes error introduced by Desctools see https://github.com/joey711/phyloseq/issues/1457
```

```
## Warning in matrix(tree$edge[order(tree$edge[, 1]), ][, 2], byrow = TRUE, : data
## length [8987] is not a sub-multiple or multiple of the number of rows [4494]
```

```
attributes(unifrac.distance)$Labels <- phyloseq::sample_names(ps_gg_fp_f_prevalence_filter_2019_05_26_proportion)
print(vegan::adonis2(unifrac.distance~metadata_ordered$hiv_phenotype, data=ASV_table, permutations=1000)) -> adon_hiv_phenotype
```

```
## Permutation test for adonis under reduced model
## Terms added sequentially (first to last)
## Permutation: free
## Number of permutations: 1000
## 
## vegan::adonis2(formula = unifrac.distance ~ metadata_ordered$hiv_phenotype, data = ASV_table, permutations = 1000)
##                                 Df SumOfSqs      R2      F  Pr(>F)  
## metadata_ordered$hiv_phenotype   1    0.538 0.01243 1.4218 0.01998 *
## Residual                       113   42.768 0.98757                 
## Total                          114   43.307 1.00000                 
## ---
## Signif. codes:  0 '***' 0.001 '**' 0.01 '*' 0.05 '.' 0.1 ' ' 1
```

```
###*** r2 = 0.012426 p = 0.020979

# vegan::adonis2(formula = unifrac.distance ~ metadata_ordered$hiv_phenotype, data = ASV_table, permutations = 1000)
#                                 Df SumOfSqs       R2       F   Pr(>F)  
# metadata_ordered$hiv_phenotype   1   0.5381 0.012426 1.42183 0.017982 *
# Residual                       113  42.7685 0.987574                   
# Total                          114  43.3066 1.000000                

print(vegan::adonis2(unifrac.distance~metadata_ordered$sample_cohort, data=ASV_table, permutations=1000)) -> adon_cohort
```

```
## Permutation test for adonis under reduced model
## Terms added sequentially (first to last)
## Permutation: free
## Number of permutations: 1000
## 
## vegan::adonis2(formula = unifrac.distance ~ metadata_ordered$sample_cohort, data = ASV_table, permutations = 1000)
##                                 Df SumOfSqs      R2      F   Pr(>F)    
## metadata_ordered$sample_cohort   1    1.638 0.03782 4.4413 0.000999 ***
## Residual                       113   41.669 0.96218                    
## Total                          114   43.307 1.00000                    
## ---
## Signif. codes:  0 '***' 0.001 '**' 0.01 '*' 0.05 '.' 0.1 ' ' 1
```

```
###*** r2 = 0.037817 p = 0.000999

# vegan::adonis2(formula = unifrac.distance ~ metadata_ordered$sample_cohort, data = ASV_table, permutations = 1000)
#                                 Df SumOfSqs       R2       F   Pr(>F)    
# metadata_ordered$sample_cohort   1   1.6377 0.037817 4.44125 0.000999 ***
# Residual                       113  41.6689 0.962183                     
# Total                          114  43.3066 1.000000                    

print(vegan::adonis2(unifrac.distance~metadata_ordered$hiv_phenotype+metadata_ordered$sample_cohort, data=ASV_table, permutations=1000)) -> adon_cohort_phenotype
```

```
## Permutation test for adonis under reduced model
## Terms added sequentially (first to last)
## Permutation: free
## Number of permutations: 1000
## 
## vegan::adonis2(formula = unifrac.distance ~ metadata_ordered$hiv_phenotype + metadata_ordered$sample_cohort, data = ASV_table, permutations = 1000)
##                                 Df SumOfSqs      R2      F   Pr(>F)    
## metadata_ordered$hiv_phenotype   1    0.538 0.01243 1.4620 0.008991 ** 
## metadata_ordered$sample_cohort   1    1.542 0.03562 4.1903 0.000999 ***
## Residual                       112   41.226 0.95196                    
## Total                          114   43.307 1.00000                    
## ---
## Signif. codes:  0 '***' 0.001 '**' 0.01 '*' 0.05 '.' 0.1 ' ' 1
```

```
###*** sample_cohort r2 = 0.035616 p = 0.000999, hiv_phenotype r2 = 0.012426 p = 0.008991

# vegan::adonis2(formula = unifrac.distance ~ metadata_ordered$hiv_phenotype + metadata_ordered$sample_cohort, data = ASV_table, permutations = 1000)
#                                 Df SumOfSqs       R2       F   Pr(>F)    
# metadata_ordered$hiv_phenotype   1   0.5381 0.012426 1.46197 0.007992 ** 
# metadata_ordered$sample_cohort   1   1.5424 0.035616 4.19034 0.000999 ***
# Residual                       112  41.2260 0.951958                     
# Total                          114  43.3066 1.000000        

### Extra metadata to add: (in addition to sample_cohort, hiv_phenotype)
### that have full n: Race, Ethnicity, age, sex
metadata_ordered$age <- as.numeric(metadata_ordered$age)
metadata_ordered$BMI <- as.numeric(metadata_ordered$BMI)

### control for metadata with full n
covars_full_n <- c("Ethnicity", "age", "Race", "hiv_phenotype", "sample_cohort")
print(vegan::adonis2(as.formula(paste("unifrac.distance~metadata_ordered$", paste(covars_full_n, collapse = "+metadata_ordered$"), sep = "")), data=ASV_table, permutations=1000)) -> adon_full_n
```

```
## Permutation test for adonis under reduced model
## Terms added sequentially (first to last)
## Permutation: free
## Number of permutations: 1000
## 
## vegan::adonis2(formula = as.formula(paste("unifrac.distance~metadata_ordered$", paste(covars_full_n, collapse = "+metadata_ordered$"), sep = "")), data = ASV_table, permutations = 1000)
##                                 Df SumOfSqs      R2      F   Pr(>F)    
## metadata_ordered$Ethnicity       1    0.414 0.00956 1.1343 0.186813    
## metadata_ordered$age             1    0.870 0.02008 2.3834 0.000999 ***
## metadata_ordered$Race            3    2.036 0.04701 1.8597 0.000999 ***
## metadata_ordered$hiv_phenotype   1    0.453 0.01046 1.2415 0.057942 .  
## metadata_ordered$sample_cohort   1    0.485 0.01121 1.3297 0.036963 *  
## Residual                       107   39.048 0.90167                    
## Total                          114   43.307 1.00000                    
## ---
## Signif. codes:  0 '***' 0.001 '**' 0.01 '*' 0.05 '.' 0.1 ' ' 1
```

```
###*** sample_cohort r2 = 0.011205 p = 0.020979, hiv_phenotype r2 = 0.010462 p = 0.054945

#                                 Df SumOfSqs      R2      F   Pr(>F)    
# metadata_ordered$Ethnicity       1    0.414 0.00956 1.1343 0.186813    
# metadata_ordered$age             1    0.870 0.02008 2.3834 0.000999 ***
# metadata_ordered$Race            3    2.036 0.04701 1.8597 0.000999 ***
# metadata_ordered$hiv_phenotype   1    0.453 0.01046 1.2415 0.057942 .  
# metadata_ordered$sample_cohort   1    0.485 0.01121 1.3297 0.036963 *  
# Residual                       107   39.048 0.90167                    
# Total                          114   43.307 1.00000                   

### remove samples with BMI/comorbidities is NA (n=115 -> n=110)
ps_gg_fp_f_prevalence_filter_2019_05_26_proportion_bc <- ps_gg_fp_f_prevalence_filter_2019_05_26_proportion
metadata_ordered_bc <- metadata_ordered[!is.na(metadata_ordered$dm2_hx) & !is.na(metadata_ordered$BMI),,drop=FALSE]
sample_data(ps_gg_fp_f_prevalence_filter_2019_05_26_proportion_bc) <- metadata_ordered_bc

### subset ASV table and distance matrix
ASV_table_bc<-as.data.frame(phyloseq::otu_table(ps_gg_fp_f_prevalence_filter_2019_05_26_proportion_bc))
all.equal(row.names(ASV_table_bc), row.names(metadata_ordered_bc))
```

```
## [1] TRUE
```

```
unifrac.distance_bc <- unname(usedist::dist_subset(unifrac.distance, sample_names(ps_gg_fp_f_prevalence_filter_2019_05_26_proportion_bc))) ### unname fixes error introduced by Desctools see https://github.com/joey711/phyloseq/issues/1457

### run adonis
covars_bc <- c("BMI", "dm2_hx", "hld_hx", "htn_hx", "cvd_hx", "dm2hx_dx", "hldhx_dx", "cvdhx_dx", covars_full_n)
print(vegan::adonis2(as.formula(paste("unifrac.distance_bc~metadata_ordered_bc$", paste(covars_bc, collapse = "+metadata_ordered_bc$"), sep = "")), data=ASV_table_bc, permutations=1000)) -> adon_full_n_bc
```

```
## Permutation test for adonis under reduced model
## Terms added sequentially (first to last)
## Permutation: free
## Number of permutations: 1000
## 
## vegan::adonis2(formula = as.formula(paste("unifrac.distance_bc~metadata_ordered_bc$", paste(covars_bc, collapse = "+metadata_ordered_bc$"), sep = "")), data = ASV_table_bc, permutations = 1000)
##                                    Df SumOfSqs      R2      F   Pr(>F)    
## metadata_ordered_bc$BMI             1    0.639 0.01544 1.7643 0.001998 ** 
## metadata_ordered_bc$dm2_hx          1    0.463 0.01118 1.2779 0.047952 *  
## metadata_ordered_bc$hld_hx          1    0.347 0.00840 0.9593 0.563437    
## metadata_ordered_bc$htn_hx          1    0.328 0.00793 0.9060 0.732268    
## metadata_ordered_bc$cvd_hx          1    0.440 0.01063 1.2142 0.067932 .  
## metadata_ordered_bc$dm2hx_dx        1    0.453 0.01094 1.2502 0.064935 .  
## metadata_ordered_bc$hldhx_dx        1    0.496 0.01200 1.3713 0.023976 *  
## metadata_ordered_bc$cvdhx_dx        1    0.426 0.01029 1.1762 0.106893    
## metadata_ordered_bc$Ethnicity       1    0.374 0.00904 1.0333 0.374625    
## metadata_ordered_bc$age             1    0.650 0.01571 1.7951 0.001998 ** 
## metadata_ordered_bc$Race            3    1.812 0.04381 1.6684 0.000999 ***
## metadata_ordered_bc$hiv_phenotype   1    0.425 0.01027 1.1737 0.109890    
## metadata_ordered_bc$sample_cohort   1    0.480 0.01160 1.3253 0.029970 *  
## Residual                           94   34.029 0.82274                    
## Total                             109   41.361 1.00000                    
## ---
## Signif. codes:  0 '***' 0.001 '**' 0.01 '*' 0.05 '.' 0.1 ' ' 1
```

```
###*** sample_cohort r2 = 0.011600 p = 0.025974, hiv_phenotype r2 = 0.010273 p = 0.104895

#                                    Df SumOfSqs      R2      F   Pr(>F)    
# metadata_ordered_bc$BMI             1    0.639 0.01544 1.7643 0.001998 ** 
# metadata_ordered_bc$dm2_hx          1    0.463 0.01118 1.2779 0.047952 *  
# metadata_ordered_bc$hld_hx          1    0.347 0.00840 0.9593 0.563437    
# metadata_ordered_bc$htn_hx          1    0.328 0.00793 0.9060 0.732268    
# metadata_ordered_bc$cvd_hx          1    0.440 0.01063 1.2142 0.067932 .  
# metadata_ordered_bc$dm2hx_dx        1    0.453 0.01094 1.2502 0.064935 .  
# metadata_ordered_bc$hldhx_dx        1    0.496 0.01200 1.3713 0.023976 *  
# metadata_ordered_bc$cvdhx_dx        1    0.426 0.01029 1.1762 0.106893    
# metadata_ordered_bc$Ethnicity       1    0.374 0.00904 1.0333 0.374625    
# metadata_ordered_bc$age             1    0.650 0.01571 1.7951 0.001998 ** 
# metadata_ordered_bc$Race            3    1.812 0.04381 1.6684 0.000999 ***
# metadata_ordered_bc$hiv_phenotype   1    0.425 0.01027 1.1737 0.109890    
# metadata_ordered_bc$sample_cohort   1    0.480 0.01160 1.3253 0.029970 *  
# Residual                           94   34.029 0.82274                    
# Total                             109   41.361 1.00000                 

#--------------------------------------------------------------------------------------------------------------
```

```
#FIGURE D (Supplemental Figure 4A)
#--------------------------------------------------------------------------------------------------------------
#Prepare OTU table and taxonomy files out from the phyloseq object:
OTU_table<-as.data.frame(phyloseq::otu_table(ps_gg_fp_f_prevalence_filter_2019_05_26))
Taxonomy<-as.data.frame(phyloseq::tax_table(ps_gg_fp_f_prevalence_filter_2019_05_26))
metadata<-as.data.frame(phyloseq::sample_data(ps_gg_fp_f_prevalence_filter_2019_05_26))

#Find the sample with the smallest number of reads. It will be the value used to normalize all data so all samples will be comparable among them:
rowSums(OTU_table)
```

```
## 105574.boston1.0139.2014.12.08 108777.boston1.0140.2014.12.08 
##                          72609                          64328 
## 112993.boston1.0141.2014.12.08 123656.boston1.0005.2014.12.08 
##                          79920                          36479 
## 143200.boston1.0006.2014.12.08 153724.boston1.0007.2014.12.08 
##                          81427                          85980 
## 165642.boston1.0008.2014.12.08 194317.boston1.0010.2014.12.08 
##                          98119                          53263 
## 196203.boston1.0011.2014.12.08 205120.boston1.0013.2014.12.08 
##                          36651                          59443 
## 211774.boston1.0014.2014.12.08 228437.boston1.0017.2014.12.08 
##                          57586                          52936 
## 229969.boston1.0018.2014.12.08 237983.boston1.0019.2014.12.08 
##                          62357                          37574 
## 258085.boston1.0142.2014.12.08 273479.boston1.0143.2014.12.08 
##                          59728                         102691 
## 315504.boston1.0028.2014.12.08 330183.boston1.0144.2014.12.08 
##                          21399                          68760 
## 337016.boston1.0030.2014.12.08 365685.boston1.0032.2014.12.08 
##                          43655                          41183 
## 386576.boston1.0035.2014.12.08 389876.boston1.0036.2014.12.08 
##                          36454                          66739 
## 410644.boston1.0125.2014.12.08 410932.boston1.0039.2014.12.08 
##                          73269                          27289 
## 413736.boston1.0126.2014.12.08 427838.boston1.0127.2014.12.08 
##                          39912                          66847 
## 453548.boston1.0045.2014.12.08 460380.boston1.0046.2014.12.08 
##                          34382                          31329 
## 473516.boston1.0047.2014.12.08 479693.boston1.0048.2014.12.08 
##                          46039                          18765 
## 485548.boston1.0049.2014.12.08 498553.boston1.0050.2014.12.08 
##                          35582                          56405 
## 505402.boston1.0051.2014.12.08 516980.boston1.0130.2014.12.08 
##                          59815                          14678 
## 522458.boston1.0132.2014.12.08 526318.boston1.0133.2014.12.08 
##                          68609                          27811 
## 527968.boston1.0057.2014.12.08 529516.boston1.0136.2014.12.08 
##                          26204                          20681 
## 533586.boston1.0059.2014.12.08 534694.boston1.0060.2014.12.08 
##                          57983                          51661 
## 604772.boston1.0071.2014.12.08 614225.boston1.0073.2014.12.08 
##                          52199                          47445 
## 615167.boston1.0074.2014.12.08 616147.boston1.0075.2014.12.08 
##                          37085                          50711 
## 653425.boston1.0077.2014.12.08 666207.boston1.0079.2014.12.08 
##                          42115                          70102 
## 694413.boston1.0080.2014.12.08 708968.boston1.0083.2014.12.08 
##                          44825                          28721 
## 734962.boston1.0085.2014.12.08 745577.boston1.0086.2014.12.08 
##                          43153                          75694 
## 758572.boston1.0088.2014.12.08 775609.boston1.0091.2014.12.08 
##                          63237                          64948 
## 813341.boston1.0095.2014.12.08 819622.boston1.0096.2014.12.08 
##                          74295                          34279 
## 842279.boston1.0097.2014.12.08 847041.boston1.0098.2014.12.08 
##                          61932                          59051 
## 862898.boston1.0103.2014.12.08 874612.boston1.0105.2014.12.08 
##                          38913                          21417 
## 880160.boston1.0106.2014.12.08 899025.boston1.0107.2014.12.08 
##                          61338                          51550 
## 900158.boston1.0108.2014.12.08 911594.boston1.0109.2014.12.08 
##                          18175                          54168 
## 923358.boston1.0111.2014.12.08 950965.boston1.0116.2014.12.08 
##                          34979                          66238 
## 953586.boston1.0117.2014.12.08 958793.boston1.0118.2014.12.08 
##                          54430                          66440 
## 966971.boston1.0120.2014.12.08 970489.boston1.0121.2014.12.08 
##                          44109                          48095 
## 995725.boston1.0123.2014.12.08  529863.boston.0165.2017.04.06 
##                          58954                          42388 
##  608647.boston.0072.2017.04.06    686039.0040.0323.2017.04.06 
##                          31028                         374932 
##   WT24922.0093.0468.2017.04.06    102438.0086.0363.2017.03.15 
##                         107819                          25113 
##    106085.0054.0333.2017.03.15    122897.0017.0307.2017.03.15 
##                          19135                          80414 
##    129226.0089.0367.2017.03.15    136376.0013.0302.2017.03.15 
##                          33236                          27232 
##    148342.0027.0314.2017.03.15    157072.0043.0244.2017.03.15 
##                          45438                          22883 
##    175067.0033.0231.2017.03.15    181090.0091.0369.2017.03.15 
##                          15936                          22823 
##    189326.0070.0343.2017.03.15    191447.0008.0296.2017.03.15 
##                          61451                          62556 
##    207295.0010.0298.2017.03.15    211578.0032.0230.2017.03.15 
##                          30235                          23193 
##    228516.0076.0350.2017.03.15    236532.0078.0379.2017.03.15 
##                          39086                          66657 
##    238426.0046.0247.2017.03.15    249768.0083.0359.2017.03.15 
##                          15578                          65769 
##    251073.0025.0221.2017.03.15    285803.0064.0338.2017.03.15 
##                          27143                          63788 
##    293340.0035.0233.2017.03.15    305385.0051.0330.2017.03.15 
##                          24885                          20588 
##    310817.0006.0293.2017.03.15    331904.0098.0430.2017.03.15 
##                          32927                          35529 
##    347964.0061.0336.2017.03.15    350103.0012.0301.2017.03.15 
##                          66932                          34897 
##    380272.0044.0245.2017.03.15    387879.0030.0228.2017.03.15 
##                          55368                          51686 
##    400609.0058.0262.2017.03.15    408044.0052.0331.2017.03.15 
##                          24415                          57931 
##    419034.0081.0382.2017.03.15    432158.0045.0246.2017.03.15 
##                          57230                          37797 
##    442916.0037.0319.2017.03.15    444991.0047.0327.2017.03.15 
##                          24253                          17042 
##    460929.0041.0324.2017.03.15    466105.0018.0377.2017.03.15 
##                          18796                          48350 
##    470588.0066.0340.2017.03.15  481066.boston.0168.2017.03.15 
##                          52619                          14233 
##    487268.0057.0261.2017.03.15    498229.0036.0318.2017.03.15 
##                          17799                          38925 
##    498554.0062.0337.2017.03.15    502743.0038.0320.2017.03.15 
##                          47910                          29154 
##    515591.0056.0334.2017.03.15    516035.0020.0310.2017.03.15 
##                          16026                          41843 
##    521471.0067.0341.2017.03.15    524541.0024.0313.2017.03.15 
##                          37753                          34986 
##    560575.0080.0381.2017.03.15    564855.0053.0332.2017.03.15 
##                          76936                          32030 
##    565723.0005.0292.2017.03.15    588311.0072.0346.2017.03.15 
##                          33565                          61200 
##    596527.0095.0374.2017.03.15    614631.0065.0339.2017.03.15 
##                          49031                          23279 
##    629358.0009.0297.2017.03.15    637837.0021.0312.2017.03.15 
##                          63706                          33770 
##    651433.0099.0431.2017.03.15    658217.0055.0258.2017.03.15 
##                          27060                          34249 
##    711750.0082.0383.2017.03.15    721154.0060.0335.2017.03.15 
##                          83786                          16367 
##    721729.0088.0366.2017.03.15    722188.0063.0268.2017.03.15 
##                          23928                          26981 
##    725896.0022.0218.2017.03.15    732425.0039.0378.2017.03.15 
##                          18124                          48694 
##    735345.0071.0345.2017.03.15    768392.0096.0375.2017.03.15 
##                          46984                          41304 
##    805457.0094.0373.2017.03.15    805641.0015.0305.2017.03.15 
##                          65857                          38997 
##    839338.0011.0300.2017.03.15    848334.0077.0351.2017.03.15 
##                          70395                          45711 
##    885614.0048.0328.2017.03.15    888751.0019.0309.2017.03.15 
##                          18203                          47832 
##    893231.0074.0348.2017.03.15    902901.0029.0226.2017.03.15 
##                          28255                          18740 
##    905350.0085.0362.2017.03.15    908782.0092.0371.2017.03.15 
##                          42388                          28907 
##  909824.boston.0174.2017.03.15    910641.0031.0316.2017.03.15 
##                          12820                          38990 
##    916034.0034.0232.2017.03.15    919901.0026.0222.2017.03.15 
##                          21405                          19714 
##    940622.0075.0349.2017.03.15    959714.0004.0291.2017.03.15 
##                          42914                          30169 
##    959734.0090.0368.2017.03.15    968359.0087.0365.2017.03.15 
##                          37955                          33934 
##    968902.0073.0347.2017.03.15    972684.0028.0224.2017.03.15 
##                          34542                          22469 
##    975240.0016.0306.2017.03.15    976183.0050.0329.2017.03.15 
##                          30867                          31908 
##    979196.0003.0290.2017.03.15    989517.0068.0274.2017.03.15 
##                          34682                          45375 
##   WT09782.0159.0121.2017.02.01   WT15101.0174.0106.2017.02.01 
##                          52918                          17604 
##   WT42336.0160.0122.2017.02.01   WT44778.0158.0120.2017.02.01 
##                          17747                          80361 
##   XE17833.0092.0100.2017.02.01   XE22903.0043.0125.2017.02.01 
##                          10751                          92243 
##   XE28163.0194.0127.2017.02.01   WM26348.0100.0113.2017.01.11 
##                          77437                          52751 
##   WM26354.0139.0315.2017.01.11   WQ64001.0145.0143.2017.01.11 
##                          45678                          39753 
##   WS20813.0155.0153.2017.01.11   WS21384.0094.0102.2017.01.11 
##                          29351                          12873 
##   WS21401.0047.0283.2017.01.11   WS21556.0141.0317.2017.01.11 
##                          65243                          57521 
##   WS21562.0055.0438.2017.01.11   WS21578.0125.0134.2017.01.11 
##                          13616                          46331 
##   WS21584.0066.0321.2017.01.11   WS22205.0097.0110.2017.01.11 
##                          28249                          65967 
##   WS74808.0165.0347.2017.01.11   WS74858.0098.0111.2017.01.11 
##                          32886                          18675 
##   WS75418.0032.0279.2017.01.11   WS76117.0102.0115.2017.01.11 
##                          66488                          78753 
##   WS76840.0117.0126.2017.01.11   WS77050.0026.0276.2017.01.11 
##                          39459                          15102 
##   WT02695.0166.0104.2017.01.11   WT02712.0078.0323.2017.01.11 
##                          20141                          38952 
##   WT05558.0190.0378.2017.01.11   WT05564.0116.0125.2017.01.11 
##                          17973                          33463 
##   WT06263.0127.0135.2017.01.11   WT07439.0058.0440.2017.01.11 
##                          35147                          43263 
##   WT08061.0090.0098.2017.01.11   WT09760.0053.0437.2017.01.11 
##                          28736                          34666 
##   WT09798.0057.0285.2017.01.11   WT10373.0080.0325.2017.01.11 
##                          78499                          37600 
##   WT10389.0036.0270.2017.01.11   WT10395.0035.0269.2017.01.11 
##                          29095                          43853 
##   WT12565.0189.0377.2017.01.11   WT14129.0153.0151.2017.01.11 
##                          26219                          58444 
##   WT14135.0123.0132.2017.01.11   WT14818.0111.0120.2017.01.11 
##                          42345                          33448 
##   WT15084.0046.0282.2017.01.11   WT15117.0157.0155.2017.01.11 
##                         122971                          41809 
##   WT15123.0164.0346.2017.01.11   WT23223.0144.0320.2017.01.11 
##                          37517                          39399 
##   WT23273.0101.0114.2017.01.11   WT24900.0152.0150.2017.01.11 
##                          32170                          25802 
##   WT24916.0044.0281.2017.01.11   WT24944.0124.0159.2017.01.11 
##                          74046                          36504 
##   WT24950.0033.0267.2017.01.11   WT24966.0142.0318.2017.01.11 
##                          54801                          37339 
##   WT27396.0121.0130.2017.01.11   WT27407.0156.0154.2017.01.11 
##                          30349                          27423 
##   WT27441.0129.0137.2017.01.11   WT30335.0134.0140.2017.01.11 
##                          25366                          76057 
##   WT30357.0049.0274.2017.01.11   WT30818.0107.0118.2017.01.11 
##                          35457                          26163 
##   WT30824.0147.0145.2017.01.11   WT30830.0052.0275.2017.01.11 
##                          45066                          41492 
##   WT34353.0120.0129.2017.01.11   WT40033.0133.0139.2017.01.11 
##                          40414                          22514 
##   WT40049.0148.0146.2017.01.11   WT40083.0038.0271.2017.01.11 
##                          60368                          23791 
##   WT40516.0122.0131.2017.01.11   WT41865.0089.0097.2017.01.11 
##                          69537                          24316 
##   WT41910.0095.0108.2017.01.11   WT42069.0154.0152.2017.01.11 
##                          43202                          25172 
##   WT43352.0087.0447.2017.01.11   WT43368.0118.0127.2017.01.11 
##                          15820                          50208 
##   WT43374.0048.0273.2017.01.11   WT44245.0081.0337.2017.01.11 
##                          57836                          54390 
##   WT44601.0082.0445.2017.01.11   WT44762.0130.0138.2017.01.11 
##                          41145                         118478 
##   WT47297.0173.0105.2017.01.11   WT48435.0099.0112.2017.01.11 
##                          30740                          39063 
##   WT48441.0112.0121.2017.01.11   WT48457.0060.0441.2017.01.11 
##                          31526                          41539 
##   WT48491.0146.0144.2017.01.11   WT48502.0106.0117.2017.01.11 
##                          28055                          63937 
##   WT50842.0187.0376.2017.01.11   WT50858.0128.0136.2017.01.11 
##                          20957                          36154 
##   WY74094.0079.0324.2017.01.11   WY74105.0151.0149.2017.01.11 
##                          55072                          67120 
##   WY74777.0073.0444.2017.01.11   WY74799.0176.0369.2017.01.11 
##                          45932                          27435 
##   WY75915.0114.0123.2017.01.11   WY75959.0191.0379.2017.01.11 
##                          27184                          28579 
##   WY75971.0062.0443.2017.01.11   WY76486.0162.0344.2017.01.11 
##                          49071                          52240 
##   WY78078.0161.0343.2017.01.11   WY78084.0061.0442.2017.01.11 
##                          29088                          15433 
##   WY78567.0119.0128.2017.01.11   WY79216.0040.0272.2017.01.11 
##                          21515                          42024 
##   WY79222.0056.0439.2017.01.11   WY79266.0113.0157.2017.01.11 
##                          27261                          55014 
##   WY79272.0177.0370.2017.01.11   WY80257.0027.0277.2017.01.11 
##                          31091                          65700 
##   WY80324.0137.0287.2017.01.11   WY81156.0104.0116.2017.01.11 
##                          37886                          41525 
##   WY81162.0169.0362.2017.01.11   WY81184.0045.0436.2017.01.11 
##                          65619                          23675 
##   XE13926.0025.0214.2017.01.11   XE13948.0192.0380.2017.01.11 
##                          25666                          43145 
##   XE13960.0150.0148.2017.01.11   XE15596.0149.0147.2017.01.11 
##                          34682                          51049 
##   XE17300.0083.0339.2017.01.11   XE21060.0015.0266.2017.01.11 
##                          60567                          39820 
##   XE21076.0182.0372.2017.01.11   XE21082.0186.0375.2017.01.11 
##                          37296                          20236 
##   XE21098.0115.0158.2017.01.11   XE22892.0171.0364.2017.01.11 
##                          42113                          50713 
##   XE22953.0096.0109.2017.01.11   XE27236.0054.0284.2017.01.11 
##                          52070                         135285 
##   XE27414.0059.0286.2017.01.11   XE28202.0028.0278.2017.01.11 
##                          51840                          44456 
##   XE29167.0135.0141.2017.01.11   XE30225.0020.0212.2017.01.11 
##                          64259                          24576 
##   XE31552.0011.0211.2017.01.11   XE33411.0143.0319.2017.01.11 
##                          20444                          38078 
##   XE36257.0175.0156.2017.01.11   XE36952.0091.0099.2017.01.11 
##                          48972                          11715 
##   XE36996.0009.0210.2017.01.11   XE38944.0167.0360.2017.01.11 
##                          38916                          26375 
##   XE38950.0140.0316.2017.01.11   XE38966.0034.0268.2017.01.11 
##                          38755                          55702 
##   XE38972.0178.0371.2017.01.11   XE38988.0110.0119.2017.01.11 
##                          35384                          32939 
##   XE38994.0185.0374.2017.01.11   XE40684.0024.0213.2017.01.11 
##                          21707                          25565 
##   XE40690.0172.0365.2017.01.11   XE40701.0163.0103.2017.01.11 
##                          46970                          46782 
##   XE40717.0010.0265.2017.01.11   XE40745.0006.0264.2017.01.11 
##                          44631                          59807 
##   XE40751.0088.0096.2017.01.11   XE41305.0193.0381.2017.01.11 
##                          17438                          27692 
##   XE41311.0138.0314.2017.01.11   XE41327.0085.0446.2017.01.11 
##                          63493                          13408 
##   XE41333.0136.0142.2017.01.11   XE41349.0168.0361.2017.01.11 
##                          44701                          26835 
##   XE41561.0037.0280.2017.01.11   XE41577.0170.0363.2017.01.11 
##                          44727                          31898 
##   XE41583.0183.0373.2017.01.11   WS19294.0064.0471.2016.11.13 
##                          26328                          58457 
##   WS20829.0126.0349.2016.11.13   WS20835.0007.0390.2016.11.13 
##                         136145                          35250 
##   WS21390.0076.0299.2016.11.13   WS21540.0031.0414.2016.11.13 
##                         144074                          52735 
##   WT02689.0077.0300.2016.11.13   WT02728.0109.0332.2016.11.13 
##                          70581                          83182 
##   WT04693.0016.0246.2016.11.13   WT04704.0071.0294.2016.11.13 
##                          60837                         133384 
##   WT07417.0184.0222.2016.11.13   WT08055.0075.0298.2016.11.13 
##                         122883                         116845 
##   WT09332.0070.0293.2016.11.13   WT09776.0074.0297.2016.11.13 
##                         159041                         156778 
##   WT10406.0003.0386.2016.11.13   WT11111.0023.0406.2016.11.13 
##                          24724                         196412 
##   WT12559.0072.0295.2016.11.13   WT14141.0002.0385.2016.11.13 
##                         192888                          57645 
##   WT15090.0180.0218.2016.11.13   WT23295.0022.0251.2016.11.13 
##                         106854                          68044 
##   WT27518.0050.0457.2016.11.13   WT27607.0039.0422.2016.11.13 
##                          32576                          90830 
##   WT30341.0012.0243.2016.11.13   WT30868.0042.0449.2016.11.13 
##                          55241                          74533 
##   WT30880.0069.0292.2016.11.13   WT34347.0181.0219.2016.11.13 
##                          81481                         103299 
##   WT37711.0068.0291.2016.11.13   WT42192.0131.0354.2016.11.13 
##                         146323                          52000 
##   WT47308.0004.0387.2016.11.13   WY76492.0067.0290.2016.11.13 
##                          54624                         132789 
##   WY78062.0019.0248.2016.11.13   WY79238.0108.0331.2016.11.13 
##                          67007                          37816 
##   WY79244.0105.0328.2016.11.13   WY80318.0103.0326.2016.11.13 
##                          16417                          53131 
##   WY82116.0188.0226.2016.11.13   XE15574.0065.0288.2016.11.13 
##                          79589                         203487 
##   XE17922.0179.0217.2016.11.13   XE17938.0014.0245.2016.11.13 
##                          93388                          67436 
##   XE18326.0084.0307.2016.11.13   XE21109.0018.0247.2016.11.13 
##                          81864                         105606 
##   XE28157.0013.0244.2016.11.13   XE28274.0051.0458.2016.11.13 
##                          94532                          24318 
##   XE29173.0008.0240.2016.11.13   XE29812.0132.0355.2016.11.13 
##                          82688                         143895 
##   XE33372.0021.0427.2016.11.13   XE36174.0063.0470.2016.11.13 
##                          74927                          92285 
##   XE37001.0030.0413.2016.11.13   XE39009.0029.0412.2016.11.13 
##                          80217                          48882 
##   XE39532.0017.0400.2016.11.13   XE39554.0041.0448.2016.11.13 
##                         205766                          65169 
##   XE40723.0001.0384.2016.11.13   XE40739.0086.0309.2016.11.13 
##                          61118                          34672 
##   XE41599.0005.0388.2016.11.13   MBA4060.0077.0266.2016.03.20 
##                          56531                          11517 
##   MBA1007.0092.0281.2016.03.11   MBA1037.0129.0437.2016.03.11 
##                          16176                          14385 
##   MBA1083.0088.0367.2016.03.11   MBA1141.0108.0297.2016.03.11 
##                          11423                          11454 
##   MBA1166.0047.0236.2016.03.11   MBA1172.0017.0444.2016.03.11 
##                          11848                          11809 
##   MBA1261.0125.0433.2016.03.11   MBA1327.0122.0368.2016.03.11 
##                          13925                          20343 
##   MBA1447.0019.0469.2016.03.11   MBA4044.0024.0470.2016.03.11 
##                          45329                          42889 
##   MBA4049.0043.0416.2016.03.11   MBA4051.0001.0446.2016.03.11 
##                          13037                          12351 
##   MBA4056.0119.0308.2016.03.11   MBA4065.0152.0341.2016.03.11 
##                          12105                          12002 
##   MBA4077.0040.0378.2016.03.11   MBA4085.0075.0264.2016.03.11 
##                          27897                          14923 
##   MBA4088.0082.0271.2016.03.11   MBA4091.0085.0429.2016.03.11 
##                           9947                          14136 
##   MBA4120.0076.0265.2016.03.11   MBA4121.0046.0235.2016.03.11 
##                          13922                          12858 
##   MBA4129.0087.0468.2016.03.11   MBA4130.0148.0337.2016.03.11 
##                          40274                          16263 
##   MBA4134.0120.0363.2016.03.11   MBA4139.0164.0353.2016.03.11 
##                          25631                          11278 
##  233202.Boston.0164.2016.02.14  930024.Boston.0114.2016.02.14 
##                          24841                          27023 
##   MBA1003.0131.0320.2016.02.14   MBA1021.0035.0460.2016.02.14 
##                          49506                          21557 
##   MBA1030.0014.0205.2016.02.14   MBA1033.0055.0244.2016.02.14 
##                          60325                          23191 
##   MBA1035.0102.0426.2016.02.14   MBA1041.0053.0242.2016.02.14 
##                          40213                          14230 
##   MBA1052.0009.0451.2016.02.14   MBA1071.0011.0202.2016.02.14 
##                          14859                          82865 
##   MBA1074.0058.0247.2016.02.14   MBA1082.0023.0463.2016.02.14 
##                          13002                          28556 
##   MBA1084.0114.0303.2016.02.14   MBA1090.0003.0194.2016.02.14 
##                          11543                         133259 
##   MBA1095.0090.0279.2016.02.14   MBA1096.0060.0249.2016.02.14 
##                          23430                          19672 
##   MBA1099.0066.0255.2016.02.14   MBA1100.0027.0386.2016.02.14 
##                          19574                          26058 
##   MBA1101.0149.0338.2016.02.14   MBA1103.0026.0385.2016.02.14 
##                          19728                          24858 
##   MBA1111.0139.0328.2016.02.14   MBA1133.0041.0453.2016.02.14 
##                          17707                          23140 
##   MBA1135.0140.0329.2016.02.14   MBA1139.0018.0445.2016.02.14 
##                           9599                          13882 
##   MBA1143.0105.0294.2016.02.14   MBA1151.0107.0296.2016.02.14 
##                          19881                          13159 
##   MBA1159.0165.0354.2016.02.14   MBA1163.0167.0356.2016.02.14 
##                          30857                          33492 
##   MBA1181.0126.0315.2016.02.14   MBA1187.0062.0251.2016.02.14 
##                          43998                          15695 
##   MBA1190.0048.0237.2016.02.14   MBA1193.0132.0321.2016.02.14 
##                          53414                          15292 
##   MBA1199.0069.0258.2016.02.14   MBA1202.0094.0456.2016.02.14 
##                          21096                          14588 
##   MBA1209.0101.0290.2016.02.14   MBA1211.0136.0325.2016.02.14 
##                          10734                          20052 
##   MBA1218.0030.0425.2016.02.14   MBA1230.0093.0282.2016.02.14 
##                          19204                          19109 
##   MBA1237.0170.0359.2016.02.14   MBA1241.0022.0419.2016.02.14 
##                          25519                          30836 
##   MBA1248.0070.0259.2016.02.14   MBA1251.0033.0467.2016.02.14 
##                          16468                          26599 
##   MBA1260.0158.0347.2016.02.14   MBA1267.0038.0227.2016.02.14 
##                          39840                          17977 
##   MBA1270.0042.0231.2016.02.14   MBA1271.0116.0462.2016.02.14 
##                          16729                          22420 
##   MBA1279.0162.0461.2016.02.14   MBA1307.0029.0372.2016.02.14 
##                          48196                          13686 
##   MBA1312.0074.0263.2016.02.14   MBA1317.0067.0256.2016.02.14 
##                          16407                          16233 
##   MBA1330.0050.0239.2016.02.14   MBA1341.0160.0349.2016.02.14 
##                          12379                          21517 
##   MBA1344.0169.0358.2016.02.14   MBA1355.0037.0458.2016.02.14 
##                          38110                          38263 
##   MBA1363.0123.0312.2016.02.14   MBA1365.0171.0360.2016.02.14 
##                          11750                          19399 
##   MBA1370.0034.0465.2016.02.14   MBA1375.0061.0250.2016.02.14 
##                          32525                          24576 
##   MBA1382.0159.0348.2016.02.14   MBA1385.0124.0313.2016.02.14 
##                          23025                          37460 
##   MBA1392.0134.0323.2016.02.14   MBA1399.0147.0336.2016.02.14 
##                          41620                          26657 
##   MBA1410.0130.0379.2016.02.14   MBA1437.0063.0252.2016.02.14 
##                          19712                          13941 
##   MBA1460.0106.0295.2016.02.14   MBA1480.0166.0355.2016.02.14 
##                           9819                          34300 
##   MBA1486.0115.0304.2016.02.14   MBA1488.0072.0452.2016.02.14 
##                          22751                          13626 
##   MBA1511.0163.0352.2016.02.14   MBA1515.0153.0342.2016.02.14 
##                          21490                          24838 
##   MBA4041.0044.0449.2016.02.14   MBA4043.0059.0248.2016.02.14 
##                          10254                          13060 
##   MBA4045.0157.0346.2016.02.14   MBA4047.0012.0203.2016.02.14 
##                          33741                          48792 
##   MBA4050.0028.0434.2016.02.14   MBA4052.0118.0457.2016.02.14 
##                          14519                          35830 
##   MBA4053.0057.0246.2016.02.14   MBA4057.0133.0322.2016.02.14 
##                          25496                           9954 
##   MBA4061.0010.0201.2016.02.14   MBA4062.0079.0393.2016.02.14 
##                         104643                          11876 
##   MBA4063.0100.0289.2016.02.14   MBA4066.0142.0430.2016.02.14 
##                          16644                          12023 
##   MBA4067.0104.0293.2016.02.14   MBA4068.0007.0198.2016.02.14 
##                          22257                          49188 
##   MBA4069.0004.0195.2016.02.14   MBA4070.0086.0478.2016.02.14 
##                          90587                          41192 
##   MBA4072.0161.0350.2016.02.14   MBA4074.0078.0267.2016.02.14 
##                          27345                          13679 
##   MBA4076.0016.0207.2016.02.14   MBA4078.0151.0340.2016.02.14 
##                          47724                          35591 
##   MBA4080.0065.0254.2016.02.14   MBA4081.0052.0241.2016.02.14 
##                          23350                          20586 
##   MBA4082.0032.0221.2016.02.14   MBA4086.0110.0299.2016.02.14 
##                          15687                          18709 
##   MBA4087.0155.0344.2016.02.14   MBA4089.0141.0330.2016.02.14 
##                          35300                          21650 
##   MBA4092.0008.0199.2016.02.14   MBA4095.0002.0193.2016.02.14 
##                          69046                         138863 
##   MBA4096.0145.0334.2016.02.14   MBA4102.0154.0343.2016.02.14 
##                          18861                          23941 
##   MBA4103.0128.0317.2016.02.14   MBA4106.0168.0357.2016.02.14 
##                          13480                          33202 
##   MBA4107.0138.0327.2016.02.14   MBA4108.0099.0288.2016.02.14 
##                          12705                          14653 
##   MBA4109.0073.0262.2016.02.14   MBA4111.0036.0459.2016.02.14 
##                          10720                          38086 
##   MBA4112.0005.0196.2016.02.14   MBA4113.0137.0396.2016.02.14 
##                          59252                          13144 
##   MBA4114.0064.0253.2016.02.14   MBA4115.0143.0332.2016.02.14 
##                          13795                           9242 
##   MBA4118.0031.0466.2016.02.14   MBA4119.0127.0316.2016.02.14 
##                          42048                          13338 
##   MBA4122.0112.0301.2016.02.14   MBA4123.0056.0245.2016.02.14 
##                          28583                          21135 
##   MBA4126.0150.0339.2016.02.14   MBA4127.0021.0417.2016.02.14 
##                           8596                          18287 
##   MBA4131.0006.0197.2016.02.14   MBA4132.0051.0240.2016.02.14 
##                         121717                          12687 
##   MBA4133.0135.0324.2016.02.14   MBA4136.0020.0464.2016.02.14 
##                          18548                          26125 
##   MBA4138.0068.0257.2016.02.14   MBA4140.0015.0206.2016.02.14 
##                          21282                          32717 
##  226855.boston.0016.2015.11.25  229075.boston.0178.2015.11.25 
##                          12316                          21754 
##  447537.boston.0170.2015.11.25  503564.boston.0169.2015.11.25 
##                          11055                          16113 
##  561130.boston.0065.2015.11.25  588800.boston.0068.2015.11.25 
##                          64533                          54160 
##  629356.boston.0076.2015.11.25  765828.boston.0089.2015.11.25 
##                          17564                          18760 
##  772512.boston.0090.2015.11.25  826391.boston.0177.2015.11.25 
##                          28599                          29717 
##  849016.boston.0176.2015.11.25  872569.boston.0179.2015.11.25 
##                          27270                          22343 
##     136109.048.0334.2018.12.15     137787.006.0293.2018.12.15 
##                         142042                         110949 
##     186400.027.0314.2018.12.15     207722.029.0316.2018.12.15 
##                         130465                         140162 
##     221355.060.0345.2018.12.15     225794.001.0288.2018.12.15 
##                         131800                         169725 
##     235185.033.0320.2018.12.15     236512.015.0302.2018.12.15 
##                         146986                         136583 
##     240884.013.0300.2018.12.15     241367.042.0329.2018.12.15 
##                         134087                         130533 
##     243736.036.0323.2018.12.15     276693.051.0337.2018.12.15 
##                         125215                         143542 
##     282036.026.0313.2018.12.15     298053.032.0319.2018.12.15 
##                         140377                         139377 
##     319820.035.0322.2018.12.15     354109.012.0299.2018.12.15 
##                         156031                         130651 
##     356403.053.0339.2018.12.15     364485.002.0289.2018.12.15 
##                         163928                         138715 
##     373167.021.0308.2018.12.15     395379.010.0297.2018.12.15 
##                          91803                         125471 
##     402235.052.0338.2018.12.15     411794.028.0315.2018.12.15 
##                         103407                         125418 
##     416151.055.0340.2018.12.15     420641.004.0291.2018.12.15 
##                         147234                         137640 
##     423443.005.0292.2018.12.15     445474.025.0312.2018.12.15 
##                         153787                         162503 
##     453551.040.0327.2018.12.15     459452.044.0331.2018.12.15 
##                         149435                         105418 
##     467375.007.0294.2018.12.15     471179.003.0290.2018.12.15 
##                         148513                         139922 
##     492275.058.0343.2018.12.15     570298.041.0328.2018.12.15 
##                         165014                         135343 
##     574541.045.0332.2018.12.15     576662.030.0317.2018.12.15 
##                         124317                         151416 
##     578598.017.0304.2018.12.15     580176.038.0325.2018.12.15 
##                         121232                         141927 
##     588443.031.0318.2018.12.15     588873.064.0349.2018.12.15 
##                          94483                         109394 
##     612872.019.0306.2018.12.15     667045.043.0330.2018.12.15 
##                         139365                         116480 
##     675294.009.0296.2018.12.15     684908.056.0341.2018.12.15 
##                         138860                         122542 
##     714983.057.0342.2018.12.15     720750.034.0321.2018.12.15 
##                         145724                         121774 
##     774051.062.0347.2018.12.15     821034.039.0326.2018.12.15 
##                         147680                         119747 
##     822655.037.0324.2018.12.15     823946.047.0333.2018.12.15 
##                          93713                         121833 
##     850240.022.0309.2018.12.15     851204.014.0301.2018.12.15 
##                         107960                         145594 
##     853781.063.0348.2018.12.15     857829.018.0305.2018.12.15 
##                         130937                         136894 
##     883092.049.0335.2018.12.15     909074.024.0311.2018.12.15 
##                         126602                          83194 
##     918320.061.0346.2018.12.15     938150.059.0344.2018.12.15 
##                         150659                         159642 
##     945496.008.0295.2018.12.15     948983.050.0336.2018.12.15 
##                         130406                         160584 
##     982757.020.0307.2018.12.15     985170.011.0298.2018.12.15 
##                         135822                         132462 
##    MBA1008.062.0371.2018.12.15    MBA1073.103.0380.2018.12.15 
##                          97776                         121454 
##    MBA1144.144.0244.2018.12.15    MBA1215.004.0359.2018.12.15 
##                          58436                         141749 
##    MBA1226.010.0360.2018.12.15    MBA1240.156.0246.2018.12.15 
##                         118025                         123306 
##    MBA1284.025.0362.2018.12.15    MBA1302.089.0375.2018.12.15 
##                         106064                         122557 
##    MBA1315.035.0365.2018.12.15    MBA1398.080.0374.2018.12.15 
##                         122382                         124340 
##    MBA1491.070.0372.2018.12.15    MBA1509.039.0366.2018.12.15 
##                         141649                         123099 
##    MBA1519.096.0377.2018.12.15    MBA4042.011.0361.2018.12.15 
##                         105745                         135959 
##    MBA4046.032.0363.2018.12.15    MBA4048.146.0245.2018.12.15 
##                         121271                         104141 
##    MBA4058.049.0368.2018.12.15    MBA4059.113.0242.2018.12.15 
##                         163357                         154949 
##    MBA4064.117.0243.2018.12.15    MBA4071.098.0379.2018.12.15 
##                          99265                         173599 
##    MBA4083.097.0378.2018.12.15    MBA4093.033.0364.2018.12.15 
##                         131209                         131778 
##    MBA4099.045.0367.2018.12.15    MBA4104.091.0376.2018.12.15 
##                         140969                         128881 
##    MBA4128.059.0369.2018.12.15 
##                         142304
```

```
min(rowSums(OTU_table))
```

```
## [1] 8596
```

```
Total_counts<-as.data.frame(rowSums(OTU_table))
colnames(Total_counts)<-c("Counts")
ggplot2::ggplot(Total_counts, aes(x=Counts))+geom_histogram(binwidth=100)+geom_vline(aes(xintercept=mean(Counts, na.rm=T)), color="red", linetype="dashed", size=1)+theme_bw()+
  ggtitle("Total count per sample distribution")+theme(plot.title=element_text(lineheight=10, size=15))+
  xlab("Counts")+ylab("Number of samples")+theme(axis.text=element_text(size=15), axis.title=element_text(size=15))
```

```
#Remove all those samples that do not reach a minimum threshold of number of reads:
counts<-as.data.frame(rowSums(OTU_table))
colnames(counts)<-"counts"
counts$Sample<-row.names(counts)
subset_8000<-counts[counts$counts>=8000,]
subset_8000$Sample<-NULL
OTU_table<-OTU_table[row.names(OTU_table)%in%as.vector(row.names(subset_8000)),]

#Rarefy to same sequencing depth:
set.seed(1)
OTU_table_8000<-vegan::rrarefy(OTU_table, 8000)
```

```
## Warning in vegan::rrarefy(OTU_table, 8000): function should be used for
## observed counts, but smallest count is 2
```

```
#Estimate richness:
richness_8000<-vegan::estimateR(OTU_table_8000)
richness_8000<-t(richness_8000)
richness_8000<-as.data.frame(richness_8000)
richness_8000$se.chao1<-NULL
richness_8000$se.ACE<-NULL

#Estimate evenness:
shannon<-BiodiversityR::diversityresult(x=OTU_table_8000, method="each site", index="Shannon")
diversity_8000<-cbind(shannon)

#Join data from richness and evenness calculations:
ecology_8000<-cbind(richness_8000, diversity_8000)
colnames(ecology_8000)<-c("Observed", "Chao1", "ACE", "Shannon")

#Subset metadata
metadata<-metadata[row.names(metadata)%in%row.names(ecology_8000),,drop=FALSE]

all.equal(row.names(metadata),row.names(ecology_8000))
```

```
## [1] TRUE
```

```
ecology_8000$SampleID<-metadata$SampleID
ecology_8000$Cohort<-metadata$sample_cohort
ecology_8000$hiv_phenotype<-metadata$hiv_phenotype
ecology_8000$sexual_orientation<-metadata$sexual_orientation
ecology_8000$sex<-metadata$sex

#Subset indivudals for this comparison
ecology_8000<-ecology_8000[ecology_8000$sex=="male",,drop=FALSE]
ecology_8000<-ecology_8000[ecology_8000$sexual_orientation!="MSM",,drop=FALSE]

ecology_8000_melt<-reshape2::melt(ecology_8000)
```

```
## Using SampleID, Cohort, hiv_phenotype, sexual_orientation, sex as id variables
```

```
ecology_8000_melt_boston<-ecology_8000_melt[ecology_8000_melt$Cohort=="boston",,drop=F]
ecology_8000_melt_botswana<-ecology_8000_melt[ecology_8000_melt$Cohort=="botswana",,drop=F]
ecology_8000_melt_uganda<-ecology_8000_melt[ecology_8000_melt$Cohort=="uganda_2",,drop=F]

plot_boston<-ggplot(data=ecology_8000_melt_boston, aes(x=hiv_phenotype,y=value))+geom_boxplot(aes(alpha=hiv_phenotype), outlier.color="white", fill="royalblue4")+theme_bw()+
  geom_point(aes(alpha=hiv_phenotype), color="royalblue4", position=position_jitterdodge(jitter.width=0.25), size=1)+
  facet_wrap(~variable, scales="free_y", nrow=1)+scale_alpha_manual(values=c(0.9, 0.6, 0.3))+
  theme(axis.text.x = element_text(angle=90))+ggtitle("Just Male - Boston")

plot_botswana<-ggplot(data=ecology_8000_melt_botswana, aes(x=hiv_phenotype,y=value))+geom_boxplot(aes(alpha=hiv_phenotype), outlier.color="white", fill="darkorange")+theme_bw()+
  geom_point(aes(alpha=hiv_phenotype), color="darkorange", position=position_jitterdodge(jitter.width=0.25), size=1)+
  facet_wrap(~variable, scales="free_y", nrow=1)+scale_alpha_manual(values=c(0.9, 0.6, 0.3))+
  theme(axis.text.x = element_text(angle=90))+ggtitle("Just Male - Botswana")

plot_uganda<-ggplot(data=ecology_8000_melt_uganda, aes(x=hiv_phenotype,y=value))+geom_boxplot(aes(alpha=hiv_phenotype), outlier.color="white", fill="forestgreen")+theme_bw()+
  geom_point(aes(alpha=hiv_phenotype), color="forestgreen", position=position_jitterdodge(jitter.width=0.25), size=1)+
  facet_wrap(~variable, scales="free_y", nrow=1)+scale_alpha_manual(values=c(0.9, 0.6, 0.3))+
  theme(axis.text.x = element_text(angle=90))+ggtitle("Just Male - Uganda")

ggsave("SupplementaryFigure2D.pdf", grid.arrange(plot_boston, plot_botswana,plot_uganda, ncol=2, nrow=3), width=15, height=10)
```

```
#Statistical test
ecology_8000_boston<-ecology_8000[ecology_8000$Cohort=="boston",,drop=F]
ecology_8000_botswana<-ecology_8000[ecology_8000$Cohort=="botswana",,drop=F]
ecology_8000_uganda<-ecology_8000[ecology_8000$Cohort=="uganda_2",,drop=F]
# Add all metadata to the table for multivariate testing of abundance differences
metadata$age <- as.numeric(metadata$age)
metadata$BMI <- as.numeric(metadata$BMI)
### metadata not collected in Boston:
metadata_ordered$monthly_income <- as.numeric(metadata_ordered$monthly_income)
metadata_ordered$smoking_years <- as.numeric(metadata_ordered$smoking_years)
metadata_ordered$fram_10yr_risk_lab <- as.numeric(metadata_ordered$fram_10yr_risk_lab)
metadata_ordered$fram_10yr_risk_nonlab <- as.numeric(metadata_ordered$fram_10yr_risk_nonlab)
metadata_ordered$mean_imt <- as.numeric(metadata_ordered$mean_imt)
metadata_ordered$total_plaques <- as.numeric(metadata_ordered$total_plaques)
metadata_ordered$any_plaques <- as.numeric(metadata_ordered$any_plaques)
dplyr::left_join(ecology_8000_boston[colnames(ecology_8000_boston) %in% c("SampleID") | !colnames(ecology_8000_boston) %in% colnames(metadata)], metadata, by = "SampleID") -> us_full_metadata
dplyr::left_join(ecology_8000_botswana[colnames(ecology_8000_botswana) %in% c("SampleID") | !colnames(ecology_8000_botswana) %in% colnames(metadata)], metadata, by = "SampleID") -> botswana_full_metadata
dplyr::left_join(ecology_8000_uganda[colnames(ecology_8000_uganda) %in% c("SampleID") | !colnames(ecology_8000_uganda) %in% colnames(metadata)], metadata, by = "SampleID") -> uganda_full_metadata

# Kruskal and wilcox testing
#US
for (i in c("Observed", "Shannon")){
  print(i)
  print(kruskal(ecology_8000_boston[[i]], ecology_8000_boston[["hiv_phenotype"]],group=F,p.adj = "bonferroni"))
}
```

```
## [1] "Observed"
## $statistics
##        Chisq Df    p.chisq
##   0.31430406  2 0.85457413
## 
## $parameters
##             test  p.ajusted                                 name.t ntr alpha
##   Kruskal-Wallis bonferroni ecology_8000_boston[["hiv_phenotype"]]   3  0.05
## 
## $means
##                ecology_8000_boston..i..      rank        std  r Min Max    Q25
## 1_hiv_negative                146.02083 30.822917  52.262630 48  61 249  98.25
## 2_suppressed                  158.30000 33.450000  64.337737 10 107 300 116.25
## 4_unsuppressed                181.50000 34.750000 112.932133  4  78 333 106.50
##                  Q50    Q75
## 1_hiv_negative 142.0 184.25
## 2_suppressed   119.0 192.25
## 4_unsuppressed 157.5 232.50
## 
## $comparison
##                                 Difference pvalue Signif.        LCL       UCL
## 1_hiv_negative - 2_suppressed   -2.6270833      1         -18.298176 13.044009
## 1_hiv_negative - 4_unsuppressed -3.9270833      1         -27.388647 19.534480
## 2_suppressed - 4_unsuppressed   -1.3000000      1         -27.971038 25.371038
## 
## $groups
## NULL
## 
## attr(,"class")
## [1] "group"
## [1] "Shannon"
## $statistics
##        Chisq Df    p.chisq
##   0.10559396  2 0.94857257
## 
## $parameters
##             test  p.ajusted                                 name.t ntr alpha
##   Kruskal-Wallis bonferroni ecology_8000_boston[["hiv_phenotype"]]   3  0.05
## 
## $means
##                ecology_8000_boston..i..      rank        std  r       Min
## 1_hiv_negative                3.4651600 31.270833 0.69412635 48 1.8486823
## 2_suppressed                  3.6012197 33.100000 0.52066974 10 2.8723326
## 4_unsuppressed                3.4577715 30.250000 0.70174463  4 2.7044438
##                      Max       Q25       Q50       Q75
## 1_hiv_negative 4.4513155 3.0092617 3.5788184 4.0109915
## 2_suppressed   4.5236775 3.1900293 3.6674493 3.9149916
## 4_unsuppressed 4.3767472 3.0868560 3.3749475 3.7458629
## 
## $comparison
##                                 Difference pvalue Signif.        LCL       UCL
## 1_hiv_negative - 2_suppressed   -1.8291667      1         -17.529161 13.870828
## 1_hiv_negative - 4_unsuppressed  1.0208333      1         -22.484000 24.525666
## 2_suppressed - 4_unsuppressed    2.8500000      1         -23.870227 29.570227
## 
## $groups
## NULL
## 
## attr(,"class")
## [1] "group"
```

```
#Botswana
for (i in c("Observed", "Shannon")){
  print(i)
  print(kruskal(ecology_8000_botswana[[i]], ecology_8000_botswana[["hiv_phenotype"]],group=F,p.adj = "bonferroni"))
}
```

```
## [1] "Observed"
## $statistics
##       Chisq Df     p.chisq
##   6.2008023  2 0.045031135
## 
## $parameters
##             test  p.ajusted                                   name.t ntr alpha
##   Kruskal-Wallis bonferroni ecology_8000_botswana[["hiv_phenotype"]]   3  0.05
## 
## $means
##                ecology_8000_botswana..i..      rank       std  r Min Max Q25
## 1_hiv_negative                  133.34091 53.954545 49.073719 44  50 260  93
## 2_suppressed                    109.17143 40.000000 46.913240 35  35 235  80
## 4_unsuppressed                  140.89474 56.684211 66.087396 19  13 270  89
##                  Q50    Q75
## 1_hiv_negative 131.5 162.75
## 2_suppressed    98.0 132.50
## 4_unsuppressed 144.0 188.50
## 
## $comparison
##                                  Difference pvalue Signif.         LCL
## 1_hiv_negative - 2_suppressed    13.9545455 0.0871       .  -1.3884523
## 1_hiv_negative - 4_unsuppressed  -2.7296651 1.0000         -21.3258635
## 2_suppressed - 4_unsuppressed   -16.6842105 0.1134         -35.9880231
##                                        UCL
## 1_hiv_negative - 2_suppressed   29.2975432
## 1_hiv_negative - 4_unsuppressed 15.8665334
## 2_suppressed - 4_unsuppressed    2.6196021
## 
## $groups
## NULL
## 
## attr(,"class")
## [1] "group"
## [1] "Shannon"
## $statistics
##       Chisq Df     p.chisq
##   4.9192591  2 0.085466606
## 
## $parameters
##             test  p.ajusted                                   name.t ntr alpha
##   Kruskal-Wallis bonferroni ecology_8000_botswana[["hiv_phenotype"]]   3  0.05
## 
## $means
##                ecology_8000_botswana..i..      rank        std  r       Min
## 1_hiv_negative                  3.1636213 50.454545 0.76439368 44 1.3520706
## 2_suppressed                    2.9238934 42.457143 0.72137885 35 1.1496331
## 4_unsuppressed                  3.4304824 60.263158 0.90653308 19 1.7459509
##                      Max       Q25       Q50       Q75
## 1_hiv_negative 4.4841180 2.6421683 3.1379851 3.6924835
## 2_suppressed   4.1755434 2.4813163 3.0602279 3.4520488
## 4_unsuppressed 4.8490515 2.7517017 3.5386393 4.2169656
## 
## $comparison
##                                  Difference pvalue Signif.         LCL
## 1_hiv_negative - 2_suppressed     7.9974026 0.6308          -7.4548216
## 1_hiv_negative - 4_unsuppressed  -9.8086124 0.6148         -28.5371968
## 2_suppressed - 4_unsuppressed   -17.8060150 0.0839       . -37.2472511
##                                        UCL
## 1_hiv_negative - 2_suppressed   23.4496268
## 1_hiv_negative - 4_unsuppressed  8.9199719
## 2_suppressed - 4_unsuppressed    1.6352210
## 
## $groups
## NULL
## 
## attr(,"class")
## [1] "group"
```

```
#Uganda
for (i in c("Observed", "Shannon")){
  print(i)
  print(wilcox.test(ecology_8000_uganda[[i]]~ecology_8000_uganda[["hiv_phenotype"]]))
}
```

```
## [1] "Observed"
```

```
## Warning in wilcox.test.default(x = DATA[[1L]], y = DATA[[2L]], ...): cannot
## compute exact p-value with ties
```

```
## 
##  Wilcoxon rank sum test with continuity correction
## 
## data:  ecology_8000_uganda[[i]] by ecology_8000_uganda[["hiv_phenotype"]]
## W = 901.5, p-value = 1
## alternative hypothesis: true location shift is not equal to 0
## 
## [1] "Shannon"
## 
##  Wilcoxon rank sum exact test
## 
## data:  ecology_8000_uganda[[i]] by ecology_8000_uganda[["hiv_phenotype"]]
## W = 927, p-value = 0.83028
## alternative hypothesis: true location shift is not equal to 0
```

```
### Run orm () and compare alpha diversity by hiv_phenotype
### Extra metadata that have full n: Race, Ethnicity, age, sex, current_art_class_consolid2, tmp_smx_active
### Extra metadata that have <n: BMI, comorbidities (dm2_hx, hld_hx, htn_hx, cvd_hx, ckd_hx, cvd_dx [missing boston], dm2hx_dx, hldhx_dx, htnhx_dx, cvdhx_dx, ever_smoke, current_smoke, smoke_cat), school_level [uganda2 only], monthly_income[uganda2 only], current_art_class_consolid2, tmp_smx_active, days_on_art, sexual_orientation
### additional: smoking_years, fram_10yr_risk_lab, fram_10yr_risk_nonlab, mean_imt, total_plaques, any_plaques
covars_full_n <- c("Ethnicity", "age", "Race", "current_art_class_consolid2", "tmp_smx_active", "hiv_phenotype")

#US
for (i in c("Observed", "Shannon")){
  print(i)
  print(rms::orm(formula =  as.formula(paste("us_full_metadata[[",which(stringr::str_detect(colnames(us_full_metadata), i)), "]]~", paste(covars_full_n, collapse = "+"), sep = "")), data = us_full_metadata))
}
```

```
## [1] "Observed"
```

```
## Warning in .local(x, ...): singularity problem
```

```
## Warning in .local(x, ...): singularity problem

## Warning in .local(x, ...): singularity problem

## Warning in .local(x, ...): singularity problem

## Warning in .local(x, ...): singularity problem
```

```
## Logistic (Proportional Odds) Ordinal Regression Model
## 
## rms::orm(formula = as.formula(paste("us_full_metadata[[", which(stringr::str_detect(colnames(us_full_metadata), 
##     i)), "]]~", paste(covars_full_n, collapse = "+"), sep = "")), 
##     data = us_full_metadata)
## 
##                       Model Likelihood               Discrimination    Rank Discrim.    
##                             Ratio Test                      Indexes          Indexes    
## Obs            62    LR chi2      6.37    R2                  0.098    rho     0.353    
## Distinct Y     56    d.f.           12    R2(12,62)           0.000                     
## Median Y      138    Pr(> chi2) 0.8964    R2(12,62)           0.000                     
## max |deriv| 1e-06    Score chi2   7.81    |Pr(Y>=median)-0.5| 0.081                     
##                      Pr(> chi2) 0.7997                                                  
## 
##                                             Coef    S.E.   Wald Z Pr(>|Z|)
## Ethnicity=Not_Hispanic_Latino                1.0703 1.2142  0.88  0.3781  
## age                                          0.0094 0.0205  0.46  0.6488  
## Race=Black_AA                                0.2673 1.2586  0.21  0.8318  
## Race=Varied                                 -1.4977 1.5243 -0.98  0.3258  
## Race=White                                   0.0396 1.0145  0.04  0.9688  
## current_art_class_consolid2=none            -2.5256 1.4599 -1.73  0.0836  
## current_art_class_consolid2=NRTI_NRTI_ISTI  -0.9119 1.8525 -0.49  0.6225  
## current_art_class_consolid2=NRTI_NRTI_NNRTI -1.8269 1.8057 -1.01  0.3117  
## current_art_class_consolid2=NRTI_NRTI_PI    -0.5793 1.7966 -0.32  0.7471  
## tmp_smx_active                               0.0000 0.0000                
## hiv_phenotype=2_suppressed                  -1.6513 0.0000  -Inf  <0.0001 
## hiv_phenotype=4_unsuppressed                 0.5049 1.1445  0.44  0.6591  
## 
## [1] "Shannon"
```

```
## Warning in .local(x, ...): singularity problem

## Warning in .local(x, ...): singularity problem

## Warning in .local(x, ...): singularity problem

## Warning in .local(x, ...): singularity problem

## Warning in .local(x, ...): singularity problem

## Warning in .local(x, ...): singularity problem
```

```
## Logistic (Proportional Odds) Ordinal Regression Model
## 
## rms::orm(formula = as.formula(paste("us_full_metadata[[", which(stringr::str_detect(colnames(us_full_metadata), 
##     i)), "]]~", paste(covars_full_n, collapse = "+"), sep = "")), 
##     data = us_full_metadata)
## 
##                           Model Likelihood               Discrimination    Rank Discrim.    
##                                 Ratio Test                      Indexes          Indexes    
## Obs                62    LR chi2      8.05    R2                  0.122    rho     0.277    
## Distinct Y         62    d.f.           12    R2(12,62)           0.000                     
## Median Y    3.5355682    Pr(> chi2) 0.7814    R2(12,62)           0.000                     
## max |deriv|     4e-06    Score chi2  10.45    |Pr(Y>=median)-0.5| 0.098                     
##                          Pr(> chi2) 0.5762                                                  
## 
##                                             Coef    S.E.         Wald Z
## Ethnicity=Not_Hispanic_Latino                2.5552       1.2298  2.08 
## age                                         -0.0121       0.0205 -0.59 
## Race=Black_AA                                0.3959       1.1337  0.35 
## Race=Varied                                  0.7525       1.3126  0.57 
## Race=White                                   0.7989       0.9238  0.86 
## current_art_class_consolid2=none             0.6066 1761840.7685  0.00 
## current_art_class_consolid2=NRTI_NRTI_ISTI   0.1830       1.7759  0.10 
## current_art_class_consolid2=NRTI_NRTI_NNRTI -1.6562       1.8069 -0.92 
## current_art_class_consolid2=NRTI_NRTI_PI    -1.7793       1.7753 -1.00 
## tmp_smx_active                               0.0000       0.0000       
## hiv_phenotype=2_suppressed                   1.5453 1761840.7685  0.00 
## hiv_phenotype=4_unsuppressed                -0.1676       0.9844 -0.17 
##                                             Pr(>|Z|)
## Ethnicity=Not_Hispanic_Latino               0.0377  
## age                                         0.5543  
## Race=Black_AA                               0.7269  
## Race=Varied                                 0.5665  
## Race=White                                  0.3871  
## current_art_class_consolid2=none            1.0000  
## current_art_class_consolid2=NRTI_NRTI_ISTI  0.9179  
## current_art_class_consolid2=NRTI_NRTI_NNRTI 0.3593  
## current_art_class_consolid2=NRTI_NRTI_PI    0.3162  
## tmp_smx_active                                      
## hiv_phenotype=2_suppressed                  1.0000  
## hiv_phenotype=4_unsuppressed                0.8648
```

```
#Botswana
for (i in c("Observed", "Shannon")){
  print(i)
  print(rms::orm(formula =  as.formula(paste("botswana_full_metadata[[",which(stringr::str_detect(colnames(botswana_full_metadata), i)), "]]~", paste(covars_full_n[!covars_full_n %in% c("Ethnicity", "Race")], collapse = "+"), sep = "")), data = botswana_full_metadata))
}
```

```
## [1] "Observed"
```

```
## Warning in .local(x, ...): singularity problem

## Warning in .local(x, ...): singularity problem

## Warning in .local(x, ...): singularity problem

## Warning in .local(x, ...): singularity problem
```

```
## Logistic (Proportional Odds) Ordinal Regression Model
## 
## rms::orm(formula = as.formula(paste("botswana_full_metadata[[", 
##     which(stringr::str_detect(colnames(botswana_full_metadata), 
##         i)), "]]~", paste(covars_full_n[!covars_full_n %in% c("Ethnicity", 
##         "Race")], collapse = "+"), sep = "")), data = botswana_full_metadata)
## 
##                       Model Likelihood               Discrimination    Rank Discrim.    
##                             Ratio Test                      Indexes          Indexes    
## Obs            98    LR chi2      8.69    R2                  0.085    rho     0.241    
## Distinct Y     77    d.f.            7    R2(7,98)            0.017                     
## Median Y      121    Pr(> chi2) 0.2754    R2(7,98)            0.017                     
## max |deriv| 0.004    Score chi2   9.38    |Pr(Y>=median)-0.5| 0.109                     
##                      Pr(> chi2) 0.2266                                                  
## 
##                                             Coef    S.E.         Wald Z
## age                                          0.0017       0.0355 0.05  
## current_art_class_consolid2=none             0.1754 3675663.4091 0.00  
## current_art_class_consolid2=NRTI_NRTI_NNRTI  1.5279       1.0755 1.42  
## current_art_class_consolid2=NRTI_NRTI_PI     1.4509       1.3336 1.09  
## tmp_smx_active                               0.0000       0.0000       
## hiv_phenotype=2_suppressed                  -2.0805 3675663.4091 0.00  
## hiv_phenotype=4_unsuppressed                 0.2825       0.5072 0.56  
##                                             Pr(>|Z|)
## age                                         0.9619  
## current_art_class_consolid2=none            1.0000  
## current_art_class_consolid2=NRTI_NRTI_NNRTI 0.1554  
## current_art_class_consolid2=NRTI_NRTI_PI    0.2766  
## tmp_smx_active                                      
## hiv_phenotype=2_suppressed                  1.0000  
## hiv_phenotype=4_unsuppressed                0.5775  
## 
## [1] "Shannon"
```

```
## Warning in .local(x, ...): singularity problem

## Warning in .local(x, ...): singularity problem

## Warning in .local(x, ...): singularity problem

## Warning in .local(x, ...): singularity problem
```

```
## Logistic (Proportional Odds) Ordinal Regression Model
## 
## rms::orm(formula = as.formula(paste("botswana_full_metadata[[", 
##     which(stringr::str_detect(colnames(botswana_full_metadata), 
##         i)), "]]~", paste(covars_full_n[!covars_full_n %in% c("Ethnicity", 
##         "Race")], collapse = "+"), sep = "")), data = botswana_full_metadata)
## 
##                           Model Likelihood               Discrimination    Rank Discrim.    
##                                 Ratio Test                      Indexes          Indexes    
## Obs                98    LR chi2      7.36    R2                  0.072    rho     0.266    
## Distinct Y         98    d.f.            7    R2(7,98)            0.004                     
## Median Y    3.1108357    Pr(> chi2) 0.3919    R2(7,98)            0.004                     
## max |deriv|     1e-05    Score chi2   7.36    |Pr(Y>=median)-0.5| 0.096                     
##                          Pr(> chi2) 0.3920                                                  
## 
##                                             Coef    S.E.   Wald Z Pr(>|Z|)
## age                                          0.0150 0.0371  0.40  0.6859  
## current_art_class_consolid2=none            -0.4192 1.1605 -0.36  0.7179  
## current_art_class_consolid2=NRTI_NRTI_NNRTI  0.7378 1.1661  0.63  0.5269  
## current_art_class_consolid2=NRTI_NRTI_PI     1.6699 1.3583  1.23  0.2189  
## tmp_smx_active                               0.0000 0.0000                
## hiv_phenotype=2_suppressed                  -1.7411 0.0000  -Inf  <0.0001 
## hiv_phenotype=4_unsuppressed                 0.7590 0.5179  1.47  0.1428
```

```
#Uganda
for (i in c("Observed", "Shannon")){
  print(i)
  print(rms::orm(formula =  as.formula(paste("uganda_full_metadata[[",which(stringr::str_detect(colnames(uganda_full_metadata), i)), "]]~", paste(covars_full_n[!covars_full_n %in% c("Ethnicity", "Race")], collapse = "+"), sep = "")), data = uganda_full_metadata))
} ###*** Observed hiv_phenotype p = 1.0000 Shannon hiv_phenotype p = 1.0000
```

```
## [1] "Observed"
```

```
## Warning in .local(x, ...): singularity problem

## Warning in .local(x, ...): singularity problem

## Warning in .local(x, ...): singularity problem
```

```
## Logistic (Proportional Odds) Ordinal Regression Model
## 
## rms::orm(formula = as.formula(paste("uganda_full_metadata[[", 
##     which(stringr::str_detect(colnames(uganda_full_metadata), 
##         i)), "]]~", paste(covars_full_n[!covars_full_n %in% c("Ethnicity", 
##         "Race")], collapse = "+"), sep = "")), data = uganda_full_metadata)
## 
##                       Model Likelihood               Discrimination    Rank Discrim.    
##                             Ratio Test                      Indexes          Indexes    
## Obs            85    LR chi2      4.25    R2                  0.049    rho     0.228    
## Distinct Y     67    d.f.            5    R2(5,85)            0.000                     
## Median Y       98    Pr(> chi2) 0.5140    R2(5,85)            0.000                     
## max |deriv| 6e-07    Score chi2   4.47    |Pr(Y>=median)-0.5| 0.063                     
##                      Pr(> chi2) 0.4842                                                  
## 
##                                             Coef    S.E.   Wald Z Pr(>|Z|)
## age                                          0.0428 0.0238  1.80  0.0726  
## current_art_class_consolid2=NRTI_NRTI_NNRTI  0.3504 0.7040  0.50  0.6187  
## current_art_class_consolid2=NRTI_NRTI_PI    -0.1076 0.0000  -Inf  <0.0001 
## tmp_smx_active                              -0.3636 0.7580 -0.48  0.6314  
## hiv_phenotype=2_suppressed                   0.1103 0.9216  0.12  0.9047  
## 
## [1] "Shannon"
```

```
## Warning in .local(x, ...): singularity problem

## Warning in .local(x, ...): singularity problem
```

```
## Logistic (Proportional Odds) Ordinal Regression Model
## 
## rms::orm(formula = as.formula(paste("uganda_full_metadata[[", 
##     which(stringr::str_detect(colnames(uganda_full_metadata), 
##         i)), "]]~", paste(covars_full_n[!covars_full_n %in% c("Ethnicity", 
##         "Race")], collapse = "+"), sep = "")), data = uganda_full_metadata)
## 
##                         Model Likelihood               Discrimination    Rank Discrim.    
##                               Ratio Test                      Indexes          Indexes    
## Obs              85    LR chi2      6.83    R2                  0.077    rho     0.278    
## Distinct Y       85    d.f.            5    R2(5,85)            0.021                     
## Median Y    3.32637    Pr(> chi2) 0.2334    R2(5,85)            0.021                     
## max |deriv|   2e-05    Score chi2   6.94    |Pr(Y>=median)-0.5| 0.086                     
##                        Pr(> chi2) 0.2252                                                  
## 
##                                             Coef    S.E.   Wald Z Pr(>|Z|)
## age                                          0.0430 0.0240  1.79  0.0728  
## current_art_class_consolid2=NRTI_NRTI_NNRTI  1.0578 0.7990  1.32  0.1856  
## current_art_class_consolid2=NRTI_NRTI_PI     0.7446 0.0000   Inf  <0.0001 
## tmp_smx_active                              -1.4185 0.7658 -1.85  0.0640  
## hiv_phenotype=2_suppressed                   0.1418 0.9758  0.15  0.8844
```

```
#--------------------------------------------------------------------------------------------------------------
```
