# Supplementary material for: HIV-associated gut microbial alterations are dependent on host and geographic context
Source: Nat Commun. 2024 Feb 5;15:1055. doi: 10.1038/s41467-023-44566-4 (PMC10844288; doi:10.1038/s41467-023-44566-4)
Supplement: Supplementary file 14 — SupplementaryFigure3_Rocafort-Gootenberg_2023_03_20 [file 41467_2023_44566_MOESM14_ESM.html]

Rocafort-Gootenberg\_SupplementaryFigure3


# Rocafort-Gootenberg\_SupplementaryFigure3

#Load needed R packages

```
library("phyloseq")
library("tidyverse")
```

```
## ── Attaching packages ─────────────────────────────────────── tidyverse 1.3.2 ──
## ✔ ggplot2 3.4.1     ✔ purrr   1.0.1
## ✔ tibble  3.1.8     ✔ dplyr   1.1.0
## ✔ tidyr   1.3.0     ✔ stringr 1.5.0
## ✔ readr   2.1.4     ✔ forcats 1.0.0
## ── Conflicts ────────────────────────────────────────── tidyverse_conflicts() ──
## ✖ dplyr::filter() masks stats::filter()
## ✖ dplyr::lag()    masks stats::lag()
```

```
library("ggplot2")
library("gridExtra")
```

```
## 
## Attaching package: 'gridExtra'
## 
## The following object is masked from 'package:dplyr':
## 
##     combine
```

```
library("BiodiversityR")
```

```
## Loading required package: tcltk
## Loading required package: vegan
## Loading required package: permute
## Loading required package: lattice
## This is vegan 2.6-4
## BiodiversityR 2.15-1: Use command BiodiversityRGUI() to launch the Graphical User Interface; 
## to see changes use BiodiversityRGUI(changeLog=TRUE, backward.compatibility.messages=TRUE)
```

```
library("dplyr")
library("vegan")
library("knitr") 
library("agricolae")
```

```
## Registered S3 methods overwritten by 'klaR':
##   method      from 
##   predict.rda vegan
##   print.rda   vegan
##   plot.rda    vegan
```

```
library("reshape")
```

```
## 
## Attaching package: 'reshape'
## 
## The following object is masked from 'package:dplyr':
## 
##     rename
## 
## The following objects are masked from 'package:tidyr':
## 
##     expand, smiths
```

```
library("phyloseq")
library("usedist")
library("gplots")
```

```
## 
## Attaching package: 'gplots'
## 
## The following object is masked from 'package:stats':
## 
##     lowess
```

#Load original phyloseq oject output from DADA2 pipeline and add in
new metadata

```
ps_gg_fp_f_prevalence_filter_2019_05_26<-readRDS("ps_gg_fp_f_prevalence_filter_2019_05_26")
readr::read_csv(
  "Metadata_formatted_nat_comm_add_2021_10_24.csv",
  col_names = TRUE,
  col_types = NULL,
  col_select = NULL,
  id = NULL,
  locale = default_locale(),
  na = c("", "NA", "empty", "EMPTY"),
  quote = "\"",
  comment = "",
  trim_ws = TRUE,
  skip = 0,
  name_repair = "unique",
  num_threads = readr_threads(),
  progress = show_progress(),
  show_col_types = should_show_types(),
  skip_empty_rows = TRUE,
  lazy = TRUE
) -> new_metadata
```

```
## Rows: 597 Columns: 88
## ── Column specification ────────────────────────────────────────────────────────
## Delimiter: ","
## chr (26): X, SampleID, subject_id, Race, Ethnicity, unique_id, sequencing_da...
## dbl (62): primer_used, read_count, age, height_cm, height_in, weight_kg, wei...
## 
## ℹ Use `spec()` to retrieve the full column specification for this data.
## ℹ Specify the column types or set `show_col_types = FALSE` to quiet this message.
```

```
### add {SampleID} as rownames
new_metadata_as_sample_data <- phyloseq::sample_data(new_metadata)
phyloseq::sample_names(new_metadata_as_sample_data) <- dplyr::pull(new_metadata, 1)
phyloseq::sample_data(ps_gg_fp_f_prevalence_filter_2019_05_26) <- new_metadata_as_sample_data

#Fix randomness
set.seed(1)
```

#Supplementary Figure 3

```
#SUPPLEMENTARY FIGURE 3A
#--------------------------------------------------------------------------------------------------------------
#Negative - ART comparison
#US
#Transform count data in the phyloseq object
ps_gg_fp_f_prevalence_filter_2019_05_26_proportion<-transform_sample_counts(ps_gg_fp_f_prevalence_filter_2019_05_26, function(x)(x/sum(x)))

#Select samples of interest and update phyloseq object 
metadata<-phyloseq::sample_data(ps_gg_fp_f_prevalence_filter_2019_05_26)
metadata<-metadata[metadata$hiv_phenotype%in%c("1_hiv_negative","2_suppressed"),,drop=F]
metadata<-metadata[metadata$sample_cohort%in%c("boston"),,drop=F]
metadata<-as.data.frame(as.matrix(metadata[metadata$sexual_orientation!="MSM",,drop=F]))
phyloseq::sample_data(ps_gg_fp_f_prevalence_filter_2019_05_26_proportion)<-metadata

#Run PCoA on the phyloseq object
ordination<-phyloseq::ordinate(ps_gg_fp_f_prevalence_filter_2019_05_26_proportion, "PCoA", "unifrac")
```

```
## Warning in matrix(tree$edge[order(tree$edge[, 1]), ][, 2], byrow = TRUE, : data
## length [8987] is not a sub-multiple or multiple of the number of rows [4494]
```

```
ordination$values[1:2,]
```

```
##   Eigenvalues Relative_eig Broken_stick  Cumul_eig Cumul_br_stick
## 1    2.442768   0.06544973   0.05064846 0.06544973     0.05064846
## 2    1.813608   0.04859246   0.04093972 0.11404219     0.09158818
```

```
metadata_ordered<-metadata[row.names(ordination$vectors),,drop=FALSE]

all.equal(row.names(metadata_ordered), row.names(ordination$vectors))
```

```
## [1] TRUE
```

```
metadata_ordered$Unifrac1<-ordination$vectors[,1]
metadata_ordered$Unifrac2<-ordination$vectors[,2]

plot_us_neg_art<-ggplot2::ggplot(data=metadata_ordered, aes(x=Unifrac1, y=Unifrac2))+geom_point(color="royalblue4", aes(alpha=hiv_phenotype), size=2, shape=16)+
  theme_bw()+stat_ellipse(color="royalblue4", aes(alpha=hiv_phenotype), size=1)+scale_alpha_manual(values=c(1,0.6))+
  ggtitle("neg-art us")+geom_point(data=metadata_ordered %>% group_by(hiv_phenotype) %>% summarise_at(vars(matches("UniFrac")), mean),size=5, color="royalblue4", aes(alpha=hiv_phenotype))
```

```
## Warning: Using `size` aesthetic for lines was deprecated in ggplot2 3.4.0.
## ℹ Please use `linewidth` instead.
```

```
#Adonis (n=104)
ASV_table<-as.data.frame(phyloseq::otu_table(ps_gg_fp_f_prevalence_filter_2019_05_26_proportion))
all.equal(row.names(ASV_table), row.names(metadata_ordered))
```

```
## [1] TRUE
```

```
unifrac.distance<-unname(phyloseq::UniFrac(ps_gg_fp_f_prevalence_filter_2019_05_26_proportion, weighted = FALSE)) ### unname fixes error introduced by Desctools see https://github.com/joey711/phyloseq/issues/1457
```

```
## Warning in matrix(tree$edge[order(tree$edge[, 1]), ][, 2], byrow = TRUE, : data
## length [8987] is not a sub-multiple or multiple of the number of rows [4494]
```

```
attributes(unifrac.distance)$Labels <- phyloseq::sample_names(ps_gg_fp_f_prevalence_filter_2019_05_26_proportion)
print(vegan::adonis2(unifrac.distance~metadata_ordered$hiv_phenotype, data=ASV_table, permutations=1000)) -> adon_hiv_phenotype
```

```
## Permutation test for adonis under reduced model
## Terms added sequentially (first to last)
## Permutation: free
## Number of permutations: 1000
## 
## vegan::adonis2(formula = unifrac.distance ~ metadata_ordered$hiv_phenotype, data = ASV_table, permutations = 1000)
##                                 Df SumOfSqs      R2     F Pr(>F)
## metadata_ordered$hiv_phenotype   1    0.385 0.01032 1.064 0.2637
## Residual                       102   36.938 0.98968             
## Total                          103   37.323 1.00000
```

```
###*** hiv_phenotype r2 = 0.01032 p = 0.2717

# vegan::adonis2(formula = unifrac.distance ~ metadata_ordered$hiv_phenotype, data = ASV_table, permutations = 1000)
#                                 Df SumOfSqs       R2       F  Pr(>F)
# metadata_ordered$hiv_phenotype   1   0.3853 0.010324 1.06401 0.26374
# Residual                       102  36.9375 0.989676                
# Total                          103  37.3228 1.000000       

### #Negative - ART comparison: #USA: Adonis: controlling for additional metadata
### Extra metadata that have full n: race, ethnicity, age, sex, current_art_class_consolid2, (tmp_smx_active is all 0)
### Extra metadata that have <n: BMI, smoking_years, fram_10yr_risk_lab, fram_10yr_risk_nonlab
metadata_ordered$age <- as.numeric(metadata_ordered$age)
metadata_ordered$BMI <- as.numeric(metadata_ordered$BMI)
metadata_ordered$days_on_art <- as.numeric(metadata_ordered$days_on_art)

### control for metadata with full n
covars_full_n <- c("Ethnicity", "age", "Race", "sex", "hiv_phenotype", "current_art_class_consolid2")
print(vegan::adonis2(as.formula(paste("unifrac.distance~metadata_ordered$", paste(covars_full_n, collapse = "+metadata_ordered$"), sep = "")), data=ASV_table, permutations=1000)) -> adon_full_n
```

```
## Permutation test for adonis under reduced model
## Terms added sequentially (first to last)
## Permutation: free
## Number of permutations: 1000
## 
## vegan::adonis2(formula = as.formula(paste("unifrac.distance~metadata_ordered$", paste(covars_full_n, collapse = "+metadata_ordered$"), sep = "")), data = ASV_table, permutations = 1000)
##                                               Df SumOfSqs      R2      F
## metadata_ordered$Ethnicity                     1    0.422 0.01131 1.1869
## metadata_ordered$age                           1    0.731 0.01957 2.0540
## metadata_ordered$Race                          5    1.825 0.04891 1.0264
## metadata_ordered$sex                           1    0.447 0.01198 1.2575
## metadata_ordered$hiv_phenotype                 1    0.319 0.00855 0.8974
## metadata_ordered$current_art_class_consolid2   3    1.212 0.03247 1.1356
## Residual                                      91   32.367 0.86721       
## Total                                        103   37.323 1.00000       
##                                                Pr(>F)    
## metadata_ordered$Ethnicity                   0.074925 .  
## metadata_ordered$age                         0.000999 ***
## metadata_ordered$Race                        0.323676    
## metadata_ordered$sex                         0.042957 *  
## metadata_ordered$hiv_phenotype               0.791209    
## metadata_ordered$current_art_class_consolid2 0.057942 .  
## Residual                                                 
## Total                                                    
## ---
## Signif. codes:  0 '***' 0.001 '**' 0.01 '*' 0.05 '.' 0.1 ' ' 1
```

```
###*** hiv_phenotype r2 = 0.00855 p = 0.791209

#                                               Df SumOfSqs      R2      F   Pr(>F)    
# metadata_ordered$Ethnicity                     1    0.422 0.01131 1.1869 0.074925 .  
# metadata_ordered$age                           1    0.731 0.01957 2.0540 0.000999 ***
# metadata_ordered$Race                          5    1.825 0.04891 1.0264 0.323676    
# metadata_ordered$sex                           1    0.447 0.01198 1.2575 0.042957 *  
# metadata_ordered$hiv_phenotype                 1    0.319 0.00855 0.8974 0.791209    
# metadata_ordered$current_art_class_consolid2   3    1.212 0.03247 1.1356 0.057942 .  
# Residual                                      91   32.367 0.86721                    
# Total                                        103   37.323 1.00000                 

#BOTSWANA
#Transform count data in the phyloseq object
ps_gg_fp_f_prevalence_filter_2019_05_26_proportion<-transform_sample_counts(ps_gg_fp_f_prevalence_filter_2019_05_26, function(x)(x/sum(x)))

#Select samples of interest and update phyloseq object 
metadata<-phyloseq::sample_data(ps_gg_fp_f_prevalence_filter_2019_05_26)
metadata<-metadata[metadata$hiv_phenotype%in%c("1_hiv_negative","2_suppressed"),,drop=F]
metadata<-metadata[metadata$sample_cohort%in%c("botswana"),,drop=F]
metadata<-as.data.frame(as.matrix(metadata[metadata$sexual_orientation!="MSM",,drop=F]))
phyloseq::sample_data(ps_gg_fp_f_prevalence_filter_2019_05_26_proportion)<-metadata

#Run PCoA on the phyloseq object
ordination<-phyloseq::ordinate(ps_gg_fp_f_prevalence_filter_2019_05_26_proportion, "PCoA", "unifrac")
```

```
## Warning in matrix(tree$edge[order(tree$edge[, 1]), ][, 2], byrow = TRUE, : data
## length [8987] is not a sub-multiple or multiple of the number of rows [4494]
```

```
ordination$values[1:2,]
```

```
##   Eigenvalues Relative_eig Broken_stick  Cumul_eig Cumul_br_stick
## 1    4.119933   0.07158472   0.03687093 0.07158472     0.03687093
## 2    2.073390   0.03602561   0.03029199 0.10761033     0.06716292
```

```
metadata_ordered<-metadata[row.names(ordination$vectors),,drop=FALSE]

all.equal(row.names(metadata_ordered), row.names(ordination$vectors))
```

```
## [1] TRUE
```

```
metadata_ordered$Unifrac1<-ordination$vectors[,1]
metadata_ordered$Unifrac2<-ordination$vectors[,2]

plot_botswana_neg_art<-ggplot2::ggplot(data=metadata_ordered, aes(x=Unifrac1, y=Unifrac2))+geom_point(color="darkorange", aes(alpha=hiv_phenotype), size=2, shape=16)+
  theme_bw()+stat_ellipse(color="darkorange", aes(alpha=hiv_phenotype), size=1)+scale_alpha_manual(values=c(1,0.6))+
  ggtitle("neg-art botswana")+geom_point(data=metadata_ordered %>% group_by(hiv_phenotype) %>% summarise_at(vars(matches("UniFrac")), mean),size=5, color="darkorange", aes(alpha=hiv_phenotype))

#Adonis (n=153)
ASV_table<-as.data.frame(phyloseq::otu_table(ps_gg_fp_f_prevalence_filter_2019_05_26_proportion))
all.equal(row.names(ASV_table), row.names(metadata_ordered))
```

```
## [1] TRUE
```

```
unifrac.distance<-unname(phyloseq::UniFrac(ps_gg_fp_f_prevalence_filter_2019_05_26_proportion, weighted = FALSE)) ### unname fixes error introduced by Desctools see https://github.com/joey711/phyloseq/issues/1457
```

```
## Warning in matrix(tree$edge[order(tree$edge[, 1]), ][, 2], byrow = TRUE, : data
## length [8987] is not a sub-multiple or multiple of the number of rows [4494]
```

```
attributes(unifrac.distance)$Labels <- phyloseq::sample_names(ps_gg_fp_f_prevalence_filter_2019_05_26_proportion)
print(vegan::adonis2(unifrac.distance~metadata_ordered$hiv_phenotype, data=ASV_table, permutations=1000)) -> adon_hiv_phenotype
```

```
## Permutation test for adonis under reduced model
## Terms added sequentially (first to last)
## Permutation: free
## Number of permutations: 1000
## 
## vegan::adonis2(formula = unifrac.distance ~ metadata_ordered$hiv_phenotype, data = ASV_table, permutations = 1000)
##                                 Df SumOfSqs      R2      F   Pr(>F)    
## metadata_ordered$hiv_phenotype   1    0.648 0.01125 1.7186 0.000999 ***
## Residual                       151   56.906 0.98875                    
## Total                          152   57.553 1.00000                    
## ---
## Signif. codes:  0 '***' 0.001 '**' 0.01 '*' 0.05 '.' 0.1 ' ' 1
```

```
###*** hiv_phenotype r2 = 0.01125 p = 0.000999

# vegan::adonis2(formula = unifrac.distance ~ metadata_ordered$hiv_phenotype, data = ASV_table, permutations = 1000)
#                                 Df SumOfSqs       R2       F   Pr(>F)    
# metadata_ordered$hiv_phenotype   1   0.6477 0.011253 1.71858 0.000999 ***
# Residual                       151  56.9056 0.988747                     
# Total                          152  57.5532 1.000000                     

### #Negative - ART comparison: #BOTSWANA: Adonis: controlling for additional metadata
### Extra metadata that have full n: age, sex, current_art_class_consolid2, days_on_art, comorbidities (dm2_hx, hld_hx, htn_hx, cvd_hx, ckd_hx, cvd_dx [missing boston], dm2hx_dx, hldhx_dx, htnhx_dx, cvdhx_dx, smoke_cat), mean_imt, total_plaques, any_plaques
### Extra metadata that have <n: BMI, smoking_years, fram_10yr_risk_lab, fram_10yr_risk_nonlab
metadata_ordered$age <- as.numeric(metadata_ordered$age)
metadata_ordered$BMI <- as.numeric(metadata_ordered$BMI)
metadata_ordered$days_on_art <- as.numeric(metadata_ordered$days_on_art)
### metadata not collected in Boston:
metadata_ordered$smoking_years <- as.numeric(metadata_ordered$smoking_years)
metadata_ordered$fram_10yr_risk_lab <- as.numeric(metadata_ordered$fram_10yr_risk_lab)
metadata_ordered$fram_10yr_risk_nonlab <- as.numeric(metadata_ordered$fram_10yr_risk_nonlab)
metadata_ordered$mean_imt <- as.numeric(metadata_ordered$mean_imt)
metadata_ordered$total_plaques <- as.numeric(metadata_ordered$total_plaques)
metadata_ordered$any_plaques <- as.logical(as.numeric(metadata_ordered$any_plaques))

### control for metadata with full n
covars_full_n <- c("age", "sex", "dm2_hx", "hld_hx", "htn_hx", "ckd_hx", "cvd_dx", "dm2hx_dx", "hldhx_dx", "htnhx_dx", "cvdhx_dx", "smoke_cat", "mean_imt", "total_plaques", "any_plaques", "hiv_phenotype", "current_art_class_consolid2", "days_on_art")
print(vegan::adonis2(as.formula(paste("unifrac.distance~metadata_ordered$", paste(covars_full_n, collapse = "+metadata_ordered$"), sep = "")), data=ASV_table, permutations=1000)) -> adon_full_n
```

```
## Permutation test for adonis under reduced model
## Terms added sequentially (first to last)
## Permutation: free
## Number of permutations: 1000
## 
## vegan::adonis2(formula = as.formula(paste("unifrac.distance~metadata_ordered$", paste(covars_full_n, collapse = "+metadata_ordered$"), sep = "")), data = ASV_table, permutations = 1000)
##                                               Df SumOfSqs      R2      F
## metadata_ordered$age                           1    0.476 0.00828 1.2723
## metadata_ordered$sex                           1    0.579 0.01006 1.5461
## metadata_ordered$dm2_hx                        1    0.347 0.00603 0.9271
## metadata_ordered$hld_hx                        1    0.416 0.00723 1.1107
## metadata_ordered$htn_hx                        1    0.359 0.00623 0.9580
## metadata_ordered$ckd_hx                        1    0.369 0.00641 0.9846
## metadata_ordered$cvd_dx                        1    0.418 0.00726 1.1154
## metadata_ordered$dm2hx_dx                      1    0.331 0.00575 0.8845
## metadata_ordered$hldhx_dx                      1    0.348 0.00605 0.9306
## metadata_ordered$smoke_cat                     2    0.774 0.01346 1.0343
## metadata_ordered$mean_imt                      1    0.333 0.00579 0.8893
## metadata_ordered$total_plaques                 1    0.344 0.00597 0.9179
## metadata_ordered$any_plaques                   1    0.344 0.00598 0.9196
## metadata_ordered$hiv_phenotype                 1    0.676 0.01174 1.8045
## metadata_ordered$current_art_class_consolid2   2    0.896 0.01558 1.1972
## metadata_ordered$days_on_art                   1    0.371 0.00644 0.9900
## Residual                                     134   50.172 0.87175       
## Total                                        152   57.553 1.00000       
##                                                Pr(>F)   
## metadata_ordered$age                         0.045954 * 
## metadata_ordered$sex                         0.003996 **
## metadata_ordered$dm2_hx                      0.673327   
## metadata_ordered$hld_hx                      0.171828   
## metadata_ordered$htn_hx                      0.602398   
## metadata_ordered$ckd_hx                      0.511489   
## metadata_ordered$cvd_dx                      0.179820   
## metadata_ordered$dm2hx_dx                    0.810190   
## metadata_ordered$hldhx_dx                    0.681319   
## metadata_ordered$smoke_cat                   0.312687   
## metadata_ordered$mean_imt                    0.799201   
## metadata_ordered$total_plaques               0.704296   
## metadata_ordered$any_plaques                 0.712288   
## metadata_ordered$hiv_phenotype               0.001998 **
## metadata_ordered$current_art_class_consolid2 0.039960 * 
## metadata_ordered$days_on_art                 0.511489   
## Residual                                                
## Total                                                   
## ---
## Signif. codes:  0 '***' 0.001 '**' 0.01 '*' 0.05 '.' 0.1 ' ' 1
```

```
###*** hiv_phenotype r2 = 0.01174 p = 0.001998

#                                               Df SumOfSqs      R2      F   Pr(>F)   
# metadata_ordered$age                           1    0.476 0.00828 1.2723 0.045954 * 
# metadata_ordered$sex                           1    0.579 0.01006 1.5461 0.003996 **
# metadata_ordered$dm2_hx                        1    0.347 0.00603 0.9271 0.673327   
# metadata_ordered$hld_hx                        1    0.416 0.00723 1.1107 0.171828   
# metadata_ordered$htn_hx                        1    0.359 0.00623 0.9580 0.602398   
# metadata_ordered$ckd_hx                        1    0.369 0.00641 0.9846 0.511489   
# metadata_ordered$cvd_dx                        1    0.418 0.00726 1.1154 0.179820   
# metadata_ordered$dm2hx_dx                      1    0.331 0.00575 0.8845 0.810190   
# metadata_ordered$hldhx_dx                      1    0.348 0.00605 0.9306 0.681319   
# metadata_ordered$smoke_cat                     2    0.774 0.01346 1.0343 0.312687   
# metadata_ordered$mean_imt                      1    0.333 0.00579 0.8893 0.799201   
# metadata_ordered$total_plaques                 1    0.344 0.00597 0.9179 0.704296   
# metadata_ordered$any_plaques                   1    0.344 0.00598 0.9196 0.712288   
# metadata_ordered$hiv_phenotype                 1    0.676 0.01174 1.8045 0.001998 **
# metadata_ordered$current_art_class_consolid2   2    0.896 0.01558 1.1972 0.039960 * 
# metadata_ordered$days_on_art                   1    0.371 0.00644 0.9900 0.511489   
# Residual                                     134   50.172 0.87175                   
# Total                                        152   57.553 1.00000               

### remove samples with BMI/smoking_years/framingham_10year_risk is NA or hiv_risk_unknown = 1 (n=153 -> n=140)
ps_gg_fp_f_prevalence_filter_2019_05_26_proportion_bsfh <- ps_gg_fp_f_prevalence_filter_2019_05_26_proportion
m_bsfh <- metadata_ordered[!is.na(metadata_ordered$BMI) & !is.na(metadata_ordered$smoking_years) & !is.na(metadata_ordered$fram_10yr_risk_lab) & metadata_ordered$hiv_risk_unknown=="0",,drop=FALSE]
sample_data(ps_gg_fp_f_prevalence_filter_2019_05_26_proportion_bsfh) <- m_bsfh

### subset ASV table and distance matrix
ASV_table_bsfh<-as.data.frame(phyloseq::otu_table(ps_gg_fp_f_prevalence_filter_2019_05_26_proportion_bsfh))
all.equal(row.names(ASV_table_bsfh), row.names(m_bsfh))
```

```
## [1] TRUE
```

```
unifrac.distance <- unname(usedist::dist_subset(unifrac.distance, sample_names(ps_gg_fp_f_prevalence_filter_2019_05_26_proportion_bsfh))) ### unname fixes error introduced by Desctools see https://github.com/joey711/phyloseq/issues/1457

### run adonis
c_bsfh <- c("BMI", "smoking_years", "fram_10yr_risk_lab", "fram_10yr_risk_nonlab", covars_full_n)
print(vegan::adonis2(as.formula(paste("unifrac.distance~m_bsfh$", paste(c_bsfh, collapse = "+m_bsfh$"), sep = "")), data=ASV_table_bsfh, permutations=1000)) -> adon_full_n_bsfh
```

```
## Permutation test for adonis under reduced model
## Terms added sequentially (first to last)
## Permutation: free
## Number of permutations: 1000
## 
## vegan::adonis2(formula = as.formula(paste("unifrac.distance~m_bsfh$", paste(c_bsfh, collapse = "+m_bsfh$"), sep = "")), data = ASV_table_bsfh, permutations = 1000)
##                                     Df SumOfSqs      R2      F   Pr(>F)    
## m_bsfh$BMI                           1    0.409 0.00775 1.0909 0.203796    
## m_bsfh$smoking_years                 1    0.522 0.00988 1.3909 0.014985 *  
## m_bsfh$fram_10yr_risk_lab            1    0.358 0.00677 0.9535 0.605395    
## m_bsfh$fram_10yr_risk_nonlab         1    0.390 0.00739 1.0409 0.325674    
## m_bsfh$age                           1    0.476 0.00902 1.2690 0.037962 *  
## m_bsfh$sex                           1    0.476 0.00901 1.2681 0.053946 .  
## m_bsfh$dm2_hx                        1    0.345 0.00654 0.9208 0.706294    
## m_bsfh$hld_hx                        1    0.370 0.00700 0.9853 0.522478    
## m_bsfh$htn_hx                        1    0.339 0.00643 0.9049 0.766234    
## m_bsfh$ckd_hx                        1    0.362 0.00686 0.9661 0.572428    
## m_bsfh$cvd_dx                        1    0.445 0.00843 1.1865 0.068931 .  
## m_bsfh$dm2hx_dx                      1    0.346 0.00655 0.9217 0.696304    
## m_bsfh$hldhx_dx                      1    0.308 0.00583 0.8209 0.962038    
## m_bsfh$smoke_cat                     2    0.703 0.01332 0.9377 0.732268    
## m_bsfh$mean_imt                      1    0.363 0.00687 0.9667 0.531469    
## m_bsfh$total_plaques                 1    0.343 0.00650 0.9146 0.748252    
## m_bsfh$any_plaques                   1    0.343 0.00649 0.9142 0.723277    
## m_bsfh$hiv_phenotype                 1    0.740 0.01401 1.9718 0.000999 ***
## m_bsfh$current_art_class_consolid2   2    0.901 0.01707 1.2013 0.027972 *  
## m_bsfh$days_on_art                   1    0.377 0.00714 1.0044 0.429570    
## Residual                           117   43.885 0.83115                    
## Total                              139   52.800 1.00000                    
## ---
## Signif. codes:  0 '***' 0.001 '**' 0.01 '*' 0.05 '.' 0.1 ' ' 1
```

```
###*** hiv_phenotype r2 = 0.01401 p = 0.000999

#                                     Df SumOfSqs      R2      F   Pr(>F)    
# m_bsfh$BMI                           1    0.409 0.00775 1.0909 0.175824    
# m_bsfh$smoking_years                 1    0.522 0.00988 1.3909 0.009990 ** 
# m_bsfh$fram_10yr_risk_lab            1    0.358 0.00677 0.9535 0.595405    
# m_bsfh$fram_10yr_risk_nonlab         1    0.390 0.00739 1.0409 0.326673    
# m_bsfh$age                           1    0.476 0.00902 1.2690 0.042957 *  
# m_bsfh$sex                           1    0.476 0.00901 1.2681 0.037962 *  
# m_bsfh$dm2_hx                        1    0.345 0.00654 0.9208 0.697303    
# m_bsfh$hld_hx                        1    0.370 0.00700 0.9853 0.504496    
# m_bsfh$htn_hx                        1    0.339 0.00643 0.9049 0.745255    
# m_bsfh$ckd_hx                        1    0.362 0.00686 0.9661 0.561439    
# m_bsfh$cvd_dx                        1    0.445 0.00843 1.1865 0.085914 .  
# m_bsfh$dm2hx_dx                      1    0.346 0.00655 0.9217 0.722278    
# m_bsfh$hldhx_dx                      1    0.308 0.00583 0.8209 0.954046    
# m_bsfh$smoke_cat                     2    0.703 0.01332 0.9377 0.751249    
# m_bsfh$mean_imt                      1    0.363 0.00687 0.9667 0.580420    
# m_bsfh$total_plaques                 1    0.343 0.00650 0.9146 0.731269    
# m_bsfh$any_plaques                   1    0.343 0.00649 0.9142 0.746254    
# m_bsfh$hiv_phenotype                 1    0.740 0.01401 1.9718 0.000999 ***
# m_bsfh$current_art_class_consolid2   2    0.901 0.01707 1.2013 0.026973 *  
# m_bsfh$days_on_art                   1    0.377 0.00714 1.0044 0.435564    
# Residual                           117   43.885 0.83115                    
# Total                              139   52.800 1.00000      

#UGANDA
#Transform count data in the phyloseq object
ps_gg_fp_f_prevalence_filter_2019_05_26_proportion<-transform_sample_counts(ps_gg_fp_f_prevalence_filter_2019_05_26, function(x)(x/sum(x)))

#Select samples of interest and update phyloseq object 
metadata<-phyloseq::sample_data(ps_gg_fp_f_prevalence_filter_2019_05_26)
metadata<-metadata[metadata$hiv_phenotype%in%c("1_hiv_negative","2_suppressed"),,drop=F]
metadata<-metadata[metadata$sample_cohort%in%c("uganda_2"),,drop=F]
metadata<-as.data.frame(as.matrix(metadata[metadata$sexual_orientation!="MSM",,drop=F]))
phyloseq::sample_data(ps_gg_fp_f_prevalence_filter_2019_05_26_proportion)<-metadata

#Run PCoA on the phyloseq object
ordination<-phyloseq::ordinate(ps_gg_fp_f_prevalence_filter_2019_05_26_proportion, "PCoA", "unifrac")
```

```
## Warning in matrix(tree$edge[order(tree$edge[, 1]), ][, 2], byrow = TRUE, : data
## length [8987] is not a sub-multiple or multiple of the number of rows [4494]
```

```
ordination$values[1:2,]
```

```
##   Eigenvalues Relative_eig Broken_stick  Cumul_eig Cumul_br_stick
## 1    4.928983   0.07658144   0.03378740 0.07658144     0.03378740
## 2    3.272090   0.05083836   0.02787024 0.12741980     0.06165763
```

```
metadata_ordered<-metadata[row.names(ordination$vectors),,drop=FALSE]

all.equal(row.names(metadata_ordered), row.names(ordination$vectors))
```

```
## [1] TRUE
```

```
metadata_ordered$Unifrac1<-ordination$vectors[,1]
metadata_ordered$Unifrac2<-ordination$vectors[,2]

plot_uganda_neg_art<-ggplot2::ggplot(data=metadata_ordered, aes(x=Unifrac1, y=Unifrac2))+geom_point(color="darkgreen", aes(alpha=hiv_phenotype), size=2, shape=16)+
  theme_bw()+stat_ellipse(color="darkgreen", aes(alpha=hiv_phenotype), size=1)+scale_alpha_manual(values=c(1,0.6))+
  ggtitle("neg-art uganda")+geom_point(data=metadata_ordered %>% group_by(hiv_phenotype) %>% summarise_at(vars(matches("UniFrac")), mean),size=5, color="darkgreen", aes(alpha=hiv_phenotype))

#Adonis (n=170)
ASV_table<-as.data.frame(phyloseq::otu_table(ps_gg_fp_f_prevalence_filter_2019_05_26_proportion))
all.equal(row.names(ASV_table), row.names(metadata_ordered))
```

```
## [1] TRUE
```

```
unifrac.distance<-unname(phyloseq::UniFrac(ps_gg_fp_f_prevalence_filter_2019_05_26_proportion, weighted = FALSE)) ### unname fixes error introduced by Desctools see https://github.com/joey711/phyloseq/issues/1457
```

```
## Warning in matrix(tree$edge[order(tree$edge[, 1]), ][, 2], byrow = TRUE, : data
## length [8987] is not a sub-multiple or multiple of the number of rows [4494]
```

```
attributes(unifrac.distance)$Labels <- phyloseq::sample_names(ps_gg_fp_f_prevalence_filter_2019_05_26_proportion)
print(vegan::adonis2(unifrac.distance~metadata_ordered$hiv_phenotype, data=ASV_table, permutations=1000)) -> adon_hiv_phenotype
```

```
## Permutation test for adonis under reduced model
## Terms added sequentially (first to last)
## Permutation: free
## Number of permutations: 1000
## 
## vegan::adonis2(formula = unifrac.distance ~ metadata_ordered$hiv_phenotype, data = ASV_table, permutations = 1000)
##                                 Df SumOfSqs      R2      F   Pr(>F)    
## metadata_ordered$hiv_phenotype   1    1.380 0.02144 3.6812 0.000999 ***
## Residual                       168   62.983 0.97856                    
## Total                          169   64.363 1.00000                    
## ---
## Signif. codes:  0 '***' 0.001 '**' 0.01 '*' 0.05 '.' 0.1 ' ' 1
```

```
###*** r2 = 0.02144 p = 0.000999

# vegan::adonis2(formula = unifrac.distance ~ metadata_ordered$hiv_phenotype, data = ASV_table, permutations = 1000)
#                                 Df SumOfSqs       R2       F   Pr(>F)    
# metadata_ordered$hiv_phenotype   1   1.3801 0.021442 3.68119 0.000999 ***
# Residual                       168  62.9826 0.978558                     
# Total                          169  64.3626 1.000000                     

### #Negative - ART comparison: #UGANDA: Adonis: controlling for additional metadata
### Extra metadata that have full n: age, sex, BMI, current_art_class_consolid2, days_on_art, comorbidities (dm2_hx, hld_hx, htn_hx, cvd_hx, ckd_hx, cvd_dx [missing boston], dm2hx_dx, hldhx_dx, htnhx_dx, cvdhx_dx, smoke_cat), mean_imt, total_plaques, any_plaques, smoking_years, tmp_smx_active
### Extra metadata that have <n: fram_10yr_risk_lab, fram_10yr_risk_nonlab, school_level, monthly_income, #hungry_frequency, electricity, toilet, walls_material, floor_material
metadata_ordered$age <- as.numeric(metadata_ordered$age)
metadata_ordered$BMI <- as.numeric(metadata_ordered$BMI)
metadata_ordered$days_on_art <- as.numeric(metadata_ordered$days_on_art)
metadata_ordered$monthly_income <- as.numeric(metadata_ordered$monthly_income)
metadata_ordered$smoking_years <- as.numeric(metadata_ordered$smoking_years)
metadata_ordered$fram_10yr_risk_lab <- as.numeric(metadata_ordered$fram_10yr_risk_lab)
metadata_ordered$fram_10yr_risk_nonlab <- as.numeric(metadata_ordered$fram_10yr_risk_nonlab)
metadata_ordered$mean_imt <- as.numeric(metadata_ordered$mean_imt)
metadata_ordered$total_plaques <- as.numeric(metadata_ordered$total_plaques)
metadata_ordered$any_plaques <- as.logical(as.numeric(metadata_ordered$any_plaques))

m_o <- metadata_ordered

### control for metadata with full n
covars_full_n <- c("age", "sex", "BMI", "dm2_hx", "hld_hx", "ckd_hx", "cvd_dx", "dm2hx_dx", "hldhx_dx", "htnhx_dx", "cvdhx_dx", "smoke_cat", "mean_imt", "total_plaques", "any_plaques", "smoking_years", "hiv_phenotype", "tmp_smx_active", "current_art_class_consolid2", "days_on_art")
print(vegan::adonis2(as.formula(paste("unifrac.distance~m_o$", paste(covars_full_n, collapse = "+m_o$"), sep = "")), data=ASV_table, permutations=1000)) -> adon_full_n
```

```
## Permutation test for adonis under reduced model
## Terms added sequentially (first to last)
## Permutation: free
## Number of permutations: 1000
## 
## vegan::adonis2(formula = as.formula(paste("unifrac.distance~m_o$", paste(covars_full_n, collapse = "+m_o$"), sep = "")), data = ASV_table, permutations = 1000)
##                                  Df SumOfSqs      R2      F   Pr(>F)    
## m_o$age                           1    0.455 0.00707 1.2213 0.085914 .  
## m_o$sex                           1    0.497 0.00773 1.3346 0.033966 *  
## m_o$BMI                           1    0.397 0.00617 1.0652 0.285714    
## m_o$dm2_hx                        1    0.341 0.00530 0.9157 0.686314    
## m_o$hld_hx                        1    0.490 0.00761 1.3136 0.038961 *  
## m_o$ckd_hx                        1    0.335 0.00521 0.8994 0.740260    
## m_o$cvd_dx                        1    0.314 0.00489 0.8438 0.875125    
## m_o$dm2hx_dx                      1    0.488 0.00758 1.3085 0.037962 *  
## m_o$hldhx_dx                      1    0.339 0.00527 0.9102 0.713287    
## m_o$htnhx_dx                      1    0.349 0.00542 0.9362 0.624376    
## m_o$cvdhx_dx                      1    0.360 0.00559 0.9661 0.535465    
## m_o$smoke_cat                     2    0.742 0.01153 0.9956 0.455544    
## m_o$mean_imt                      1    0.322 0.00501 0.8645 0.826174    
## m_o$total_plaques                 1    0.444 0.00690 1.1911 0.093906 .  
## m_o$any_plaques                   1    0.352 0.00547 0.9444 0.611389    
## m_o$smoking_years                 1    0.373 0.00580 1.0015 0.455544    
## m_o$hiv_phenotype                 1    1.371 0.02131 3.6796 0.000999 ***
## m_o$tmp_smx_active                1    0.302 0.00470 0.8113 0.937063    
## m_o$current_art_class_consolid2   1    0.498 0.00774 1.3367 0.033966 *  
## m_o$days_on_art                   1    0.437 0.00679 1.1719 0.109890    
## Residual                        148   55.155 0.85694                    
## Total                           169   64.363 1.00000                    
## ---
## Signif. codes:  0 '***' 0.001 '**' 0.01 '*' 0.05 '.' 0.1 ' ' 1
```

```
###*** hiv_phenotype r2 = 0.02131 p = 0.000999

#                                 Df SumOfSqs      R2      F   Pr(>F)    
# m_o$age                           1    0.455 0.00707 1.2213 0.076923 .  
# m_o$sex                           1    0.497 0.00773 1.3346 0.038961 *  
# m_o$BMI                           1    0.397 0.00617 1.0652 0.280719    
# m_o$dm2_hx                        1    0.341 0.00530 0.9157 0.663337    
# m_o$hld_hx                        1    0.490 0.00761 1.3136 0.039960 *  
# m_o$ckd_hx                        1    0.335 0.00521 0.8994 0.773227    
# m_o$cvd_dx                        1    0.314 0.00489 0.8438 0.872128    
# m_o$dm2hx_dx                      1    0.488 0.00758 1.3085 0.035964 *  
# m_o$hldhx_dx                      1    0.339 0.00527 0.9102 0.692308    
# m_o$htnhx_dx                      1    0.349 0.00542 0.9362 0.611389    
# m_o$cvdhx_dx                      1    0.360 0.00559 0.9661 0.503497    
# m_o$smoke_cat                     2    0.742 0.01153 0.9956 0.470529    
# m_o$mean_imt                      1    0.322 0.00501 0.8645 0.821179    
# m_o$total_plaques                 1    0.444 0.00690 1.1911 0.108891    
# m_o$any_plaques                   1    0.352 0.00547 0.9444 0.587413    
# m_o$smoking_years                 1    0.373 0.00580 1.0015 0.435564    
# m_o$hiv_phenotype                 1    1.371 0.02131 3.6796 0.000999 ***
# m_o$tmp_smx_active                1    0.302 0.00470 0.8113 0.934066    
# m_o$current_art_class_consolid2   1    0.498 0.00774 1.3367 0.043956 *  
# m_o$days_on_art                   1    0.437 0.00679 1.1719 0.127872    
# Residual                        148   55.155 0.85694                    
# Total                           169   64.363 1.00000                 

### remove samples with school_level/monthly_income/socioeconomic/framingham_10year_risk is NA (n=170 -> n=150)
ps_gg_fp_f_prevalence_filter_2019_05_26_proportion_smf <- ps_gg_fp_f_prevalence_filter_2019_05_26_proportion
m_o <- metadata_ordered[!is.na(metadata_ordered$school_level) & !is.na(metadata_ordered$monthly_income) & !is.na(metadata_ordered$fram_10yr_risk_lab),,drop=FALSE]
sample_data(ps_gg_fp_f_prevalence_filter_2019_05_26_proportion_smf) <- m_o

### subset ASV table and distance matrix
ASV_table_smf<-as.data.frame(otu_table(ps_gg_fp_f_prevalence_filter_2019_05_26_proportion_smf))
all.equal(row.names(ASV_table_smf), row.names(m_o))
```

```
## [1] TRUE
```

```
uf.dist <- unname(usedist::dist_subset(unifrac.distance, sample_names(ps_gg_fp_f_prevalence_filter_2019_05_26_proportion_smf))) ### unname fixes error introduced by Desctools see https://github.com/joey711/phyloseq/issues/1457
covars_smf <- c("school_level", "monthly_income", "hungry_frequency", "electricity", "toilet", "walls_material", "floor_material", "fram_10yr_risk_lab", "fram_10yr_risk_nonlab", covars_full_n)
print(vegan::adonis2(as.formula(paste("uf.dist~m_o$", paste(covars_smf, collapse = "+m_o$"), sep = "")), data = ASV_table_smf, permutations = 1000)) -> adon_full_n_smf
```

```
## Permutation test for adonis under reduced model
## Terms added sequentially (first to last)
## Permutation: free
## Number of permutations: 1000
## 
## vegan::adonis2(formula = as.formula(paste("uf.dist~m_o$", paste(covars_smf, collapse = "+m_o$"), sep = "")), data = ASV_table_smf, permutations = 1000)
##                                  Df SumOfSqs      R2      F   Pr(>F)    
## m_o$school_level                  6    2.385 0.04197 1.0662 0.139860    
## m_o$monthly_income                1    0.448 0.00788 1.2005 0.082917 .  
## m_o$hungry_frequency              4    1.418 0.02495 0.9509 0.743257    
## m_o$electricity                   1    0.443 0.00780 1.1881 0.100899    
## m_o$toilet                        4    1.537 0.02704 1.0305 0.315684    
## m_o$walls_material                3    1.234 0.02171 1.1030 0.141858    
## m_o$floor_material                3    1.225 0.02155 1.0951 0.152847    
## m_o$fram_10yr_risk_lab            1    0.388 0.00683 1.0416 0.347652    
## m_o$fram_10yr_risk_nonlab         1    0.420 0.00739 1.1256 0.176823    
## m_o$age                           1    0.343 0.00604 0.9204 0.673327    
## m_o$sex                           1    0.447 0.00787 1.1991 0.099900 .  
## m_o$BMI                           1    0.438 0.00771 1.1752 0.097902 .  
## m_o$dm2_hx                        1    0.353 0.00620 0.9454 0.628372    
## m_o$hld_hx                        1    0.443 0.00780 1.1894 0.092907 .  
## m_o$ckd_hx                        1    0.342 0.00602 0.9178 0.685315    
## m_o$cvd_dx                        1    0.303 0.00533 0.8127 0.923077    
## m_o$dm2hx_dx                      1    0.543 0.00955 1.4551 0.008991 ** 
## m_o$hldhx_dx                      1    0.351 0.00618 0.9412 0.607393    
## m_o$htnhx_dx                      1    0.381 0.00670 1.0217 0.392607    
## m_o$cvdhx_dx                      1    0.445 0.00783 1.1927 0.091908 .  
## m_o$smoke_cat                     2    0.730 0.01285 0.9796 0.563437    
## m_o$mean_imt                      1    0.287 0.00505 0.7698 0.967033    
## m_o$total_plaques                 1    0.325 0.00571 0.8709 0.799201    
## m_o$any_plaques                   1    0.374 0.00658 1.0023 0.434565    
## m_o$smoking_years                 1    0.297 0.00523 0.7970 0.943057    
## m_o$hiv_phenotype                 1    0.881 0.01550 2.3617 0.000999 ***
## m_o$tmp_smx_active                1    0.293 0.00515 0.7850 0.960040    
## m_o$current_art_class_consolid2   1    0.564 0.00993 1.5133 0.008991 ** 
## m_o$days_on_art                   1    0.415 0.00730 1.1120 0.205794    
## Residual                        104   38.776 0.68235                    
## Total                           149   56.827 1.00000                    
## ---
## Signif. codes:  0 '***' 0.001 '**' 0.01 '*' 0.05 '.' 0.1 ' ' 1
```

```
###*** hiv_phenotype r2 = 0.00753 p = 0.153846

#                                  Df SumOfSqs      R2      F   Pr(>F)    
# m_o$school_level                  6    2.385 0.04197 1.0662 0.133866    
# m_o$monthly_income                1    0.448 0.00788 1.2005 0.087912 .  
# m_o$hungry_frequency              4    1.418 0.02495 0.9509 0.735265    
# m_o$electricity                   1    0.443 0.00780 1.1881 0.110889    
# m_o$toilet                        4    1.537 0.02704 1.0305 0.322677    
# m_o$walls_material                3    1.234 0.02171 1.1030 0.118881    
# m_o$floor_material                3    1.225 0.02155 1.0951 0.138861    
# m_o$fram_10yr_risk_lab            1    0.388 0.00683 1.0416 0.351648    
# m_o$fram_10yr_risk_nonlab         1    0.420 0.00739 1.1256 0.194805    
# m_o$age                           1    0.343 0.00604 0.9204 0.674326    
# m_o$sex                           1    0.447 0.00787 1.1991 0.096903 .  
# m_o$BMI                           1    0.438 0.00771 1.1752 0.109890    
# m_o$dm2_hx                        1    0.353 0.00620 0.9454 0.599401    
# m_o$hld_hx                        1    0.443 0.00780 1.1894 0.115884    
# m_o$ckd_hx                        1    0.342 0.00602 0.9178 0.690310    
# m_o$cvd_dx                        1    0.303 0.00533 0.8127 0.931069    
# m_o$dm2hx_dx                      1    0.543 0.00955 1.4551 0.012987 *  
# m_o$hldhx_dx                      1    0.351 0.00618 0.9412 0.626374    
# m_o$htnhx_dx                      1    0.381 0.00670 1.0217 0.388611    
# m_o$cvdhx_dx                      1    0.445 0.00783 1.1927 0.088911 .  
# m_o$smoke_cat                     2    0.730 0.01285 0.9796 0.547453    
# m_o$mean_imt                      1    0.287 0.00505 0.7698 0.972028    
# m_o$total_plaques                 1    0.325 0.00571 0.8709 0.805195    
# m_o$any_plaques                   1    0.374 0.00658 1.0023 0.434565    
# m_o$smoking_years                 1    0.297 0.00523 0.7970 0.951049    
# m_o$hiv_phenotype                 1    0.881 0.01550 2.3617 0.000999 ***
# m_o$tmp_smx_active                1    0.293 0.00515 0.7850 0.963037    
# m_o$current_art_class_consolid2   1    0.564 0.00993 1.5133 0.005994 ** 
# m_o$days_on_art                   1    0.415 0.00730 1.1120 0.194805    
# Residual                        104   38.776 0.68235                    
# Total                           149   56.827 1.00000                    

#Negative - Unsuppressed comparison
#US
#Transform count data in the phyloseq object
ps_gg_fp_f_prevalence_filter_2019_05_26_proportion<-transform_sample_counts(ps_gg_fp_f_prevalence_filter_2019_05_26, function(x)(x/sum(x)))

#Select samples of interest and update phyloseq object 
metadata<-phyloseq::sample_data(ps_gg_fp_f_prevalence_filter_2019_05_26)
metadata<-metadata[metadata$hiv_phenotype%in%c("1_hiv_negative","4_unsuppressed"),,drop=F]
metadata<-metadata[metadata$sample_cohort%in%c("boston"),,drop=F]
metadata<-as.data.frame(as.matrix(metadata[metadata$sexual_orientation!="MSM",,drop=F]))
phyloseq::sample_data(ps_gg_fp_f_prevalence_filter_2019_05_26_proportion)<-metadata

#Run PCoA on the phyloseq object
ordination<-phyloseq::ordinate(ps_gg_fp_f_prevalence_filter_2019_05_26_proportion, "PCoA", "unifrac")
```

```
## Warning in matrix(tree$edge[order(tree$edge[, 1]), ][, 2], byrow = TRUE, : data
## length [8987] is not a sub-multiple or multiple of the number of rows [4494]
```

```
ordination$values[1:2,]
```

```
##   Eigenvalues Relative_eig Broken_stick  Cumul_eig Cumul_br_stick
## 1    2.399651   0.06943654   0.05406681 0.06943654     0.05406681
## 2    1.759546   0.05091440   0.04354049 0.12035094     0.09760729
```

```
metadata_ordered<-metadata[row.names(ordination$vectors),,drop=FALSE]

all.equal(row.names(metadata_ordered), row.names(ordination$vectors))
```

```
## [1] TRUE
```

```
metadata_ordered$Unifrac1<-ordination$vectors[,1]
metadata_ordered$Unifrac2<-ordination$vectors[,2]

plot_us_neg_unsup<-ggplot2::ggplot(data=metadata_ordered, aes(x=Unifrac1, y=Unifrac2))+geom_point(color="royalblue4", aes(alpha=hiv_phenotype), size=2, shape=16)+
  theme_bw()+stat_ellipse(color="royalblue4", aes(alpha=hiv_phenotype), size=1)+scale_alpha_manual(values=c(1,0.3))+
  ggtitle("neg-unsup us")+geom_point(data=metadata_ordered %>% group_by(hiv_phenotype) %>% summarise_at(vars(matches("UniFrac")), mean),size=5, color="royalblue4", aes(alpha=hiv_phenotype))

#Adonis (n=96)
ASV_table<-as.data.frame(phyloseq::otu_table(ps_gg_fp_f_prevalence_filter_2019_05_26_proportion))
all.equal(row.names(ASV_table), row.names(metadata_ordered))
```

```
## [1] TRUE
```

```
unifrac.distance<-unname(phyloseq::UniFrac(ps_gg_fp_f_prevalence_filter_2019_05_26_proportion, weighted = FALSE)) ### unname fixes error introduced by Desctools see https://github.com/joey711/phyloseq/issues/1457
```

```
## Warning in matrix(tree$edge[order(tree$edge[, 1]), ][, 2], byrow = TRUE, : data
## length [8987] is not a sub-multiple or multiple of the number of rows [4494]
```

```
attributes(unifrac.distance)$Labels <- phyloseq::sample_names(ps_gg_fp_f_prevalence_filter_2019_05_26_proportion)
print(vegan::adonis2(unifrac.distance~metadata_ordered$hiv_phenotype, data=ASV_table, permutations=1000)) -> adon_hiv_phenotype
```

```
## Permutation test for adonis under reduced model
## Terms added sequentially (first to last)
## Permutation: free
## Number of permutations: 1000
## 
## vegan::adonis2(formula = unifrac.distance ~ metadata_ordered$hiv_phenotype, data = ASV_table, permutations = 1000)
##                                Df SumOfSqs      R2      F Pr(>F)
## metadata_ordered$hiv_phenotype  1    0.393 0.01138 1.0819 0.2318
## Residual                       94   34.166 0.98862              
## Total                          95   34.559 1.00000
```

```
###*** r2 = 0.01138 p = 0.2398

# vegan::adonis2(formula = unifrac.distance ~ metadata_ordered$hiv_phenotype, data = ASV_table, permutations = 1000)
#                                Df SumOfSqs       R2       F  Pr(>F)
# metadata_ordered$hiv_phenotype  1   0.3932 0.011379 1.08189 0.23676
# Residual                       94  34.1657 0.988621                
# Total                          95  34.5589 1.000000       

### #Negative - unsuppressed comparison: #USA: Adonis: controlling for additional metadata
### Extra metadata that have full n: race, ethnicity, age, sex, current_art_class_consolid2, (tmp_smx_active is all 0)
### Extra metadata that have <n: BMI, smoking_years, fram_10yr_risk_lab, fram_10yr_risk_nonlab
metadata_ordered$age <- as.numeric(metadata_ordered$age)
metadata_ordered$BMI <- as.numeric(metadata_ordered$BMI)
metadata_ordered$days_on_art <- as.numeric(metadata_ordered$days_on_art)

### control for metadata with full n
covars_full_n <- c("Ethnicity", "age", "Race", "sex", "hiv_phenotype")
print(vegan::adonis2(as.formula(paste("unifrac.distance~metadata_ordered$", paste(covars_full_n, collapse = "+metadata_ordered$"), sep = "")), data=ASV_table, permutations=1000)) -> adon_full_n
```

```
## Permutation test for adonis under reduced model
## Terms added sequentially (first to last)
## Permutation: free
## Number of permutations: 1000
## 
## vegan::adonis2(formula = as.formula(paste("unifrac.distance~metadata_ordered$", paste(covars_full_n, collapse = "+metadata_ordered$"), sep = "")), data = ASV_table, permutations = 1000)
##                                Df SumOfSqs      R2      F   Pr(>F)    
## metadata_ordered$Ethnicity      1    0.361 0.01046 1.0119 0.410589    
## metadata_ordered$age            1    0.664 0.01921 1.8588 0.000999 ***
## metadata_ordered$Race           4    1.687 0.04882 1.1808 0.010989 *  
## metadata_ordered$sex            1    0.434 0.01254 1.2138 0.089910 .  
## metadata_ordered$hiv_phenotype  1    0.339 0.00980 0.9487 0.638362    
## Residual                       87   31.074 0.89917                    
## Total                          95   34.559 1.00000                    
## ---
## Signif. codes:  0 '***' 0.001 '**' 0.01 '*' 0.05 '.' 0.1 ' ' 1
```

```
###*** hiv_phenotype r2 = 0.00980 p = 0.610390

#                                Df SumOfSqs      R2      F   Pr(>F)    
# metadata_ordered$Ethnicity      1    0.361 0.01046 1.0119 0.388611    
# metadata_ordered$age            1    0.664 0.01921 1.8588 0.000999 ***
# metadata_ordered$Race           4    1.687 0.04882 1.1808 0.018981 *  
# metadata_ordered$sex            1    0.434 0.01254 1.2138 0.083916 .  
# metadata_ordered$hiv_phenotype  1    0.339 0.00980 0.9487 0.610390    
# Residual                       87   31.074 0.89917                    
# Total                          95   34.559 1.00000              

#BOTSWANA
#Transform count data in the phyloseq object
ps_gg_fp_f_prevalence_filter_2019_05_26_proportion<-transform_sample_counts(ps_gg_fp_f_prevalence_filter_2019_05_26, function(x)(x/sum(x)))

#Select samples of interest and update phyloseq object 
metadata<-phyloseq::sample_data(ps_gg_fp_f_prevalence_filter_2019_05_26)
metadata<-metadata[metadata$hiv_phenotype%in%c("1_hiv_negative","4_unsuppressed"),,drop=F]
metadata<-metadata[metadata$sample_cohort%in%c("botswana"),,drop=F]
metadata<-as.data.frame(as.matrix(metadata[metadata$sexual_orientation!="MSM",,drop=F]))
phyloseq::sample_data(ps_gg_fp_f_prevalence_filter_2019_05_26_proportion)<-metadata

#Run PCoA on the phyloseq object
ordination<-phyloseq::ordinate(ps_gg_fp_f_prevalence_filter_2019_05_26_proportion, "PCoA", "unifrac")
```

```
## Warning in matrix(tree$edge[order(tree$edge[, 1]), ][, 2], byrow = TRUE, : data
## length [8987] is not a sub-multiple or multiple of the number of rows [4494]
```

```
ordination$values[1:2,]
```

```
##   Eigenvalues Relative_eig Broken_stick  Cumul_eig Cumul_br_stick
## 1    3.648158   0.08075091   0.04474057 0.08075091     0.04474057
## 2    1.851241   0.04097668   0.03640724 0.12172759     0.08114780
```

```
metadata_ordered<-metadata[row.names(ordination$vectors),,drop=FALSE]

all.equal(row.names(metadata_ordered), row.names(ordination$vectors))
```

```
## [1] TRUE
```

```
metadata_ordered$Unifrac1<-ordination$vectors[,1]
metadata_ordered$Unifrac2<-ordination$vectors[,2]

plot_botswana_neg_unsup<-ggplot2::ggplot(data=metadata_ordered, aes(x=Unifrac1, y=Unifrac2))+geom_point(color="darkorange", aes(alpha=hiv_phenotype), size=2, shape=16)+
  theme_bw()+stat_ellipse(color="darkorange", aes(alpha=hiv_phenotype), size=1)+scale_alpha_manual(values=c(1,0.3))+
  ggtitle("neg-unsup botswana")+geom_point(data=metadata_ordered %>% group_by(hiv_phenotype) %>% summarise_at(vars(matches("UniFrac")), mean),size=5, color="darkorange", aes(alpha=hiv_phenotype))

#Adonis (n=121)
ASV_table<-as.data.frame(phyloseq::otu_table(ps_gg_fp_f_prevalence_filter_2019_05_26_proportion))
all.equal(row.names(ASV_table), row.names(metadata_ordered))
```

```
## [1] TRUE
```

```
unifrac.distance<-unname(phyloseq::UniFrac(ps_gg_fp_f_prevalence_filter_2019_05_26_proportion, weighted = FALSE)) ### unname fixes error introduced by Desctools see https://github.com/joey711/phyloseq/issues/1457
```

```
## Warning in matrix(tree$edge[order(tree$edge[, 1]), ][, 2], byrow = TRUE, : data
## length [8987] is not a sub-multiple or multiple of the number of rows [4494]
```

```
attributes(unifrac.distance)$Labels <- phyloseq::sample_names(ps_gg_fp_f_prevalence_filter_2019_05_26_proportion)
print(vegan::adonis2(unifrac.distance~metadata_ordered$hiv_phenotype, data=ASV_table, permutations=1000)) -> adon_hiv_phenotype
```

```
## Permutation test for adonis under reduced model
## Terms added sequentially (first to last)
## Permutation: free
## Number of permutations: 1000
## 
## vegan::adonis2(formula = unifrac.distance ~ metadata_ordered$hiv_phenotype, data = ASV_table, permutations = 1000)
##                                 Df SumOfSqs      R2      F  Pr(>F)  
## metadata_ordered$hiv_phenotype   1    0.455 0.01006 1.2094 0.06394 .
## Residual                       119   44.723 0.98994                 
## Total                          120   45.178 1.00000                 
## ---
## Signif. codes:  0 '***' 0.001 '**' 0.01 '*' 0.05 '.' 0.1 ' ' 1
```

```
###*** r2 = 0.01006 p = 0.06893

# vegan::adonis2(formula = unifrac.distance ~ metadata_ordered$hiv_phenotype, data = ASV_table, permutations = 1000)
#                                 Df SumOfSqs       R2       F  Pr(>F)  
# metadata_ordered$hiv_phenotype   1   0.4545 0.010061 1.20942 0.07992 .
# Residual                       119  44.7234 0.989939                  
# Total                          120  45.1779 1.000000         

### #Negative - ART comparison: #BOTSWANA: Adonis: controlling for additional metadata
### Extra metadata that have full n: age, sex, current_art_class_consolid2, days_on_art, comorbidities (dm2_hx, hld_hx, htn_hx, cvd_hx, ckd_hx, cvd_dx [missing boston], dm2hx_dx, hldhx_dx, htnhx_dx, cvdhx_dx, smoke_cat), mean_imt, total_plaques, any_plaques
### Extra metadata that have <n: BMI, smoking_years, fram_10yr_risk_lab, fram_10yr_risk_nonlab
metadata_ordered$age <- as.numeric(metadata_ordered$age)
metadata_ordered$BMI <- as.numeric(metadata_ordered$BMI)
metadata_ordered$days_on_art <- as.numeric(metadata_ordered$days_on_art)
### metadata not collected in Boston:
metadata_ordered$smoking_years <- as.numeric(metadata_ordered$smoking_years)
metadata_ordered$fram_10yr_risk_lab <- as.numeric(metadata_ordered$fram_10yr_risk_lab)
metadata_ordered$fram_10yr_risk_nonlab <- as.numeric(metadata_ordered$fram_10yr_risk_nonlab)
metadata_ordered$mean_imt <- as.numeric(metadata_ordered$mean_imt)
metadata_ordered$total_plaques <- as.numeric(metadata_ordered$total_plaques)
metadata_ordered$any_plaques <- as.logical(as.numeric(metadata_ordered$any_plaques))

### control for metadata with full n
covars_full_n <- c("age", "sex", "BMI", "dm2_hx", "hld_hx", "htn_hx", "cvd_dx", "dm2hx_dx", "hldhx_dx", "htnhx_dx", "cvdhx_dx", "smoke_cat", "mean_imt", "total_plaques", "any_plaques", "hiv_phenotype")
print(vegan::adonis2(as.formula(paste("unifrac.distance~metadata_ordered$", paste(covars_full_n, collapse = "+metadata_ordered$"), sep = "")), data=ASV_table, permutations=1000)) -> adon_full_n
```

```
## Permutation test for adonis under reduced model
## Terms added sequentially (first to last)
## Permutation: free
## Number of permutations: 1000
## 
## vegan::adonis2(formula = as.formula(paste("unifrac.distance~metadata_ordered$", paste(covars_full_n, collapse = "+metadata_ordered$"), sep = "")), data = ASV_table, permutations = 1000)
##                                 Df SumOfSqs      R2      F  Pr(>F)  
## metadata_ordered$age             1    0.480 0.01061 1.2803 0.04196 *
## metadata_ordered$sex             1    0.432 0.00957 1.1544 0.11688  
## metadata_ordered$BMI             1    0.412 0.00911 1.0990 0.21079  
## metadata_ordered$dm2_hx          1    0.371 0.00822 0.9909 0.41958  
## metadata_ordered$hld_hx          1    0.313 0.00694 0.8369 0.88711  
## metadata_ordered$htn_hx          1    0.342 0.00757 0.9133 0.69630  
## metadata_ordered$cvd_dx          1    0.400 0.00886 1.0691 0.25375  
## metadata_ordered$dm2hx_dx        1    0.350 0.00775 0.9347 0.63736  
## metadata_ordered$hldhx_dx        1    0.336 0.00743 0.8961 0.78621  
## metadata_ordered$smoke_cat       2    0.956 0.02117 1.2764 0.01099 *
## metadata_ordered$mean_imt        1    0.349 0.00773 0.9329 0.66234  
## metadata_ordered$total_plaques   1    0.320 0.00709 0.8552 0.85714  
## metadata_ordered$any_plaques     1    0.346 0.00766 0.9234 0.67433  
## metadata_ordered$hiv_phenotype   1    0.440 0.00975 1.1759 0.11489  
## Residual                       105   39.329 0.87054                 
## Total                          120   45.178 1.00000                 
## ---
## Signif. codes:  0 '***' 0.001 '**' 0.01 '*' 0.05 '.' 0.1 ' ' 1
```

```
###*** hiv_phenotype r2 = 0.00977 p = 0.10090

#                                 Df SumOfSqs      R2      F   Pr(>F)   
# metadata_ordered$age             1    0.480 0.01061 1.2803 0.039960 * 
# metadata_ordered$sex             1    0.432 0.00957 1.1544 0.119880   
# metadata_ordered$BMI             1    0.412 0.00911 1.0990 0.214785   
# metadata_ordered$dm2_hx          1    0.371 0.00822 0.9909 0.407592   
# metadata_ordered$hld_hx          1    0.313 0.00694 0.8369 0.896104   
# metadata_ordered$htn_hx          1    0.342 0.00757 0.9133 0.723277   
# metadata_ordered$cvd_dx          1    0.400 0.00886 1.0691 0.248751   
# metadata_ordered$dm2hx_dx        1    0.350 0.00775 0.9347 0.665335   
# metadata_ordered$hldhx_dx        1    0.336 0.00743 0.8961 0.769231   
# metadata_ordered$smoke_cat       2    0.956 0.02117 1.2764 0.008991 **
# metadata_ordered$mean_imt        1    0.349 0.00773 0.9329 0.655345   
# metadata_ordered$total_plaques   1    0.320 0.00709 0.8552 0.866134   
# metadata_ordered$any_plaques     1    0.346 0.00766 0.9234 0.670330   
# metadata_ordered$hiv_phenotype   1    0.440 0.00975 1.1759 0.109890   
# Residual                       105   39.329 0.87054                   
# Total                          120   45.178 1.00000                 

### remove samples with smoking_years/framingham_10year_risk is NA or hiv_risk_unknown = 1 (n=121 -> n=140)
ps_gg_fp_f_prevalence_filter_2019_05_26_proportion_bsfh <- ps_gg_fp_f_prevalence_filter_2019_05_26_proportion
m_bsfh <- metadata_ordered[!is.na(metadata_ordered$smoking_years) & !is.na(metadata_ordered$fram_10yr_risk_lab) & metadata_ordered$hiv_risk_unknown=="0",,drop=FALSE]
sample_data(ps_gg_fp_f_prevalence_filter_2019_05_26_proportion_bsfh) <- m_bsfh

### subset ASV table and distance matrix
ASV_table_bsfh<-as.data.frame(phyloseq::otu_table(ps_gg_fp_f_prevalence_filter_2019_05_26_proportion_bsfh))
all.equal(row.names(ASV_table_bsfh), row.names(m_bsfh))
```

```
## [1] TRUE
```

```
unifrac.distance <- unname(usedist::dist_subset(unifrac.distance, sample_names(ps_gg_fp_f_prevalence_filter_2019_05_26_proportion_bsfh))) ### unname fixes error introduced by Desctools see https://github.com/joey711/phyloseq/issues/1457

### run adonis
c_bsfh <- c("smoking_years", "fram_10yr_risk_lab", "fram_10yr_risk_nonlab", covars_full_n)
print(vegan::adonis2(as.formula(paste("unifrac.distance~m_bsfh$", paste(c_bsfh, collapse = "+m_bsfh$"), sep = "")), data=ASV_table_bsfh, permutations=1000)) -> adon_full_n_bsfh
```

```
## Permutation test for adonis under reduced model
## Terms added sequentially (first to last)
## Permutation: free
## Number of permutations: 1000
## 
## vegan::adonis2(formula = as.formula(paste("unifrac.distance~m_bsfh$", paste(c_bsfh, collapse = "+m_bsfh$"), sep = "")), data = ASV_table_bsfh, permutations = 1000)
##                               Df SumOfSqs      R2      F   Pr(>F)   
## m_bsfh$smoking_years           1    0.600 0.01362 1.6048 0.006993 **
## m_bsfh$fram_10yr_risk_lab      1    0.371 0.00842 0.9920 0.464535   
## m_bsfh$fram_10yr_risk_nonlab   1    0.364 0.00827 0.9747 0.501499   
## m_bsfh$age                     1    0.596 0.01352 1.5932 0.003996 **
## m_bsfh$sex                     1    0.350 0.00794 0.9357 0.647353   
## m_bsfh$BMI                     1    0.386 0.00876 1.0318 0.356643   
## m_bsfh$dm2_hx                  1    0.374 0.00848 0.9995 0.397602   
## m_bsfh$hld_hx                  1    0.330 0.00749 0.8824 0.779221   
## m_bsfh$htn_hx                  1    0.329 0.00747 0.8796 0.808192   
## m_bsfh$cvd_dx                  1    0.334 0.00758 0.8936 0.746254   
## m_bsfh$dm2hx_dx                1    0.389 0.00883 1.0403 0.319680   
## m_bsfh$hldhx_dx                1    0.354 0.00803 0.9454 0.632368   
## m_bsfh$smoke_cat               2    0.734 0.01666 0.9815 0.518482   
## m_bsfh$mean_imt                1    0.393 0.00893 1.0519 0.307692   
## m_bsfh$total_plaques           1    0.326 0.00741 0.8725 0.839161   
## m_bsfh$any_plaques             1    0.367 0.00834 0.9826 0.483516   
## m_bsfh$hiv_phenotype           1    0.437 0.00993 1.1697 0.121878   
## Residual                      99   37.018 0.84032                   
## Total                        117   44.052 1.00000                   
## ---
## Signif. codes:  0 '***' 0.001 '**' 0.01 '*' 0.05 '.' 0.1 ' ' 1
```

```
###*** hiv_phenotype r2 = 0.00993 p = 0.128871

#                               Df SumOfSqs      R2      F   Pr(>F)   
# m_bsfh$smoking_years           1    0.600 0.01362 1.6048 0.002997 **
# m_bsfh$fram_10yr_risk_lab      1    0.371 0.00842 0.9920 0.490509   
# m_bsfh$fram_10yr_risk_nonlab   1    0.364 0.00827 0.9747 0.503497   
# m_bsfh$age                     1    0.596 0.01352 1.5932 0.003996 **
# m_bsfh$sex                     1    0.350 0.00794 0.9357 0.626374   
# m_bsfh$BMI                     1    0.386 0.00876 1.0318 0.345654   
# m_bsfh$dm2_hx                  1    0.374 0.00848 0.9995 0.384615   
# m_bsfh$hld_hx                  1    0.330 0.00749 0.8824 0.765235   
# m_bsfh$htn_hx                  1    0.329 0.00747 0.8796 0.802198   
# m_bsfh$cvd_dx                  1    0.334 0.00758 0.8936 0.760240   
# m_bsfh$dm2hx_dx                1    0.389 0.00883 1.0403 0.338661   
# m_bsfh$hldhx_dx                1    0.354 0.00803 0.9454 0.589411   
# m_bsfh$smoke_cat               2    0.734 0.01666 0.9815 0.503497   
# m_bsfh$mean_imt                1    0.393 0.00893 1.0519 0.273726   
# m_bsfh$total_plaques           1    0.326 0.00741 0.8725 0.816184   
# m_bsfh$any_plaques             1    0.367 0.00834 0.9826 0.533467   
# m_bsfh$hiv_phenotype           1    0.437 0.00993 1.1697 0.128871   
# Residual                      99   37.018 0.84032                   
# Total                        117   44.052 1.00000              

#Let's plot the data
ggsave("SupplementaryFigure4A.pdf", grid.arrange(plot_us_neg_art, plot_us_neg_unsup, plot_botswana_neg_art,
             plot_botswana_neg_unsup, plot_uganda_neg_art), width=15, height=10)
```

```
#--------------------------------------------------------------------------------------------------------------
```

```
#SUPPLEMENTARY FIGURE 3B
#--------------------------------------------------------------------------------------------------------------
#Prepare OTU table and taxonomy files out from the phyloseq object:
OTU_table<-as.data.frame(phyloseq::otu_table(ps_gg_fp_f_prevalence_filter_2019_05_26))
Taxonomy<-as.data.frame(phyloseq::tax_table(ps_gg_fp_f_prevalence_filter_2019_05_26))
metadata<-as.data.frame(phyloseq::sample_data(ps_gg_fp_f_prevalence_filter_2019_05_26))

#Find the sample with the smallest number of reads. It will be the value used to normalize all data so all samples will be comparable among them:
rowSums(OTU_table)
```

```
## 105574.boston1.0139.2014.12.08 108777.boston1.0140.2014.12.08 
##                          72609                          64328 
## 112993.boston1.0141.2014.12.08 123656.boston1.0005.2014.12.08 
##                          79920                          36479 
## 143200.boston1.0006.2014.12.08 153724.boston1.0007.2014.12.08 
##                          81427                          85980 
## 165642.boston1.0008.2014.12.08 194317.boston1.0010.2014.12.08 
##                          98119                          53263 
## 196203.boston1.0011.2014.12.08 205120.boston1.0013.2014.12.08 
##                          36651                          59443 
## 211774.boston1.0014.2014.12.08 228437.boston1.0017.2014.12.08 
##                          57586                          52936 
## 229969.boston1.0018.2014.12.08 237983.boston1.0019.2014.12.08 
##                          62357                          37574 
## 258085.boston1.0142.2014.12.08 273479.boston1.0143.2014.12.08 
##                          59728                         102691 
## 315504.boston1.0028.2014.12.08 330183.boston1.0144.2014.12.08 
##                          21399                          68760 
## 337016.boston1.0030.2014.12.08 365685.boston1.0032.2014.12.08 
##                          43655                          41183 
## 386576.boston1.0035.2014.12.08 389876.boston1.0036.2014.12.08 
##                          36454                          66739 
## 410644.boston1.0125.2014.12.08 410932.boston1.0039.2014.12.08 
##                          73269                          27289 
## 413736.boston1.0126.2014.12.08 427838.boston1.0127.2014.12.08 
##                          39912                          66847 
## 453548.boston1.0045.2014.12.08 460380.boston1.0046.2014.12.08 
##                          34382                          31329 
## 473516.boston1.0047.2014.12.08 479693.boston1.0048.2014.12.08 
##                          46039                          18765 
## 485548.boston1.0049.2014.12.08 498553.boston1.0050.2014.12.08 
##                          35582                          56405 
## 505402.boston1.0051.2014.12.08 516980.boston1.0130.2014.12.08 
##                          59815                          14678 
## 522458.boston1.0132.2014.12.08 526318.boston1.0133.2014.12.08 
##                          68609                          27811 
## 527968.boston1.0057.2014.12.08 529516.boston1.0136.2014.12.08 
##                          26204                          20681 
## 533586.boston1.0059.2014.12.08 534694.boston1.0060.2014.12.08 
##                          57983                          51661 
## 604772.boston1.0071.2014.12.08 614225.boston1.0073.2014.12.08 
##                          52199                          47445 
## 615167.boston1.0074.2014.12.08 616147.boston1.0075.2014.12.08 
##                          37085                          50711 
## 653425.boston1.0077.2014.12.08 666207.boston1.0079.2014.12.08 
##                          42115                          70102 
## 694413.boston1.0080.2014.12.08 708968.boston1.0083.2014.12.08 
##                          44825                          28721 
## 734962.boston1.0085.2014.12.08 745577.boston1.0086.2014.12.08 
##                          43153                          75694 
## 758572.boston1.0088.2014.12.08 775609.boston1.0091.2014.12.08 
##                          63237                          64948 
## 813341.boston1.0095.2014.12.08 819622.boston1.0096.2014.12.08 
##                          74295                          34279 
## 842279.boston1.0097.2014.12.08 847041.boston1.0098.2014.12.08 
##                          61932                          59051 
## 862898.boston1.0103.2014.12.08 874612.boston1.0105.2014.12.08 
##                          38913                          21417 
## 880160.boston1.0106.2014.12.08 899025.boston1.0107.2014.12.08 
##                          61338                          51550 
## 900158.boston1.0108.2014.12.08 911594.boston1.0109.2014.12.08 
##                          18175                          54168 
## 923358.boston1.0111.2014.12.08 950965.boston1.0116.2014.12.08 
##                          34979                          66238 
## 953586.boston1.0117.2014.12.08 958793.boston1.0118.2014.12.08 
##                          54430                          66440 
## 966971.boston1.0120.2014.12.08 970489.boston1.0121.2014.12.08 
##                          44109                          48095 
## 995725.boston1.0123.2014.12.08  529863.boston.0165.2017.04.06 
##                          58954                          42388 
##  608647.boston.0072.2017.04.06    686039.0040.0323.2017.04.06 
##                          31028                         374932 
##   WT24922.0093.0468.2017.04.06    102438.0086.0363.2017.03.15 
##                         107819                          25113 
##    106085.0054.0333.2017.03.15    122897.0017.0307.2017.03.15 
##                          19135                          80414 
##    129226.0089.0367.2017.03.15    136376.0013.0302.2017.03.15 
##                          33236                          27232 
##    148342.0027.0314.2017.03.15    157072.0043.0244.2017.03.15 
##                          45438                          22883 
##    175067.0033.0231.2017.03.15    181090.0091.0369.2017.03.15 
##                          15936                          22823 
##    189326.0070.0343.2017.03.15    191447.0008.0296.2017.03.15 
##                          61451                          62556 
##    207295.0010.0298.2017.03.15    211578.0032.0230.2017.03.15 
##                          30235                          23193 
##    228516.0076.0350.2017.03.15    236532.0078.0379.2017.03.15 
##                          39086                          66657 
##    238426.0046.0247.2017.03.15    249768.0083.0359.2017.03.15 
##                          15578                          65769 
##    251073.0025.0221.2017.03.15    285803.0064.0338.2017.03.15 
##                          27143                          63788 
##    293340.0035.0233.2017.03.15    305385.0051.0330.2017.03.15 
##                          24885                          20588 
##    310817.0006.0293.2017.03.15    331904.0098.0430.2017.03.15 
##                          32927                          35529 
##    347964.0061.0336.2017.03.15    350103.0012.0301.2017.03.15 
##                          66932                          34897 
##    380272.0044.0245.2017.03.15    387879.0030.0228.2017.03.15 
##                          55368                          51686 
##    400609.0058.0262.2017.03.15    408044.0052.0331.2017.03.15 
##                          24415                          57931 
##    419034.0081.0382.2017.03.15    432158.0045.0246.2017.03.15 
##                          57230                          37797 
##    442916.0037.0319.2017.03.15    444991.0047.0327.2017.03.15 
##                          24253                          17042 
##    460929.0041.0324.2017.03.15    466105.0018.0377.2017.03.15 
##                          18796                          48350 
##    470588.0066.0340.2017.03.15  481066.boston.0168.2017.03.15 
##                          52619                          14233 
##    487268.0057.0261.2017.03.15    498229.0036.0318.2017.03.15 
##                          17799                          38925 
##    498554.0062.0337.2017.03.15    502743.0038.0320.2017.03.15 
##                          47910                          29154 
##    515591.0056.0334.2017.03.15    516035.0020.0310.2017.03.15 
##                          16026                          41843 
##    521471.0067.0341.2017.03.15    524541.0024.0313.2017.03.15 
##                          37753                          34986 
##    560575.0080.0381.2017.03.15    564855.0053.0332.2017.03.15 
##                          76936                          32030 
##    565723.0005.0292.2017.03.15    588311.0072.0346.2017.03.15 
##                          33565                          61200 
##    596527.0095.0374.2017.03.15    614631.0065.0339.2017.03.15 
##                          49031                          23279 
##    629358.0009.0297.2017.03.15    637837.0021.0312.2017.03.15 
##                          63706                          33770 
##    651433.0099.0431.2017.03.15    658217.0055.0258.2017.03.15 
##                          27060                          34249 
##    711750.0082.0383.2017.03.15    721154.0060.0335.2017.03.15 
##                          83786                          16367 
##    721729.0088.0366.2017.03.15    722188.0063.0268.2017.03.15 
##                          23928                          26981 
##    725896.0022.0218.2017.03.15    732425.0039.0378.2017.03.15 
##                          18124                          48694 
##    735345.0071.0345.2017.03.15    768392.0096.0375.2017.03.15 
##                          46984                          41304 
##    805457.0094.0373.2017.03.15    805641.0015.0305.2017.03.15 
##                          65857                          38997 
##    839338.0011.0300.2017.03.15    848334.0077.0351.2017.03.15 
##                          70395                          45711 
##    885614.0048.0328.2017.03.15    888751.0019.0309.2017.03.15 
##                          18203                          47832 
##    893231.0074.0348.2017.03.15    902901.0029.0226.2017.03.15 
##                          28255                          18740 
##    905350.0085.0362.2017.03.15    908782.0092.0371.2017.03.15 
##                          42388                          28907 
##  909824.boston.0174.2017.03.15    910641.0031.0316.2017.03.15 
##                          12820                          38990 
##    916034.0034.0232.2017.03.15    919901.0026.0222.2017.03.15 
##                          21405                          19714 
##    940622.0075.0349.2017.03.15    959714.0004.0291.2017.03.15 
##                          42914                          30169 
##    959734.0090.0368.2017.03.15    968359.0087.0365.2017.03.15 
##                          37955                          33934 
##    968902.0073.0347.2017.03.15    972684.0028.0224.2017.03.15 
##                          34542                          22469 
##    975240.0016.0306.2017.03.15    976183.0050.0329.2017.03.15 
##                          30867                          31908 
##    979196.0003.0290.2017.03.15    989517.0068.0274.2017.03.15 
##                          34682                          45375 
##   WT09782.0159.0121.2017.02.01   WT15101.0174.0106.2017.02.01 
##                          52918                          17604 
##   WT42336.0160.0122.2017.02.01   WT44778.0158.0120.2017.02.01 
##                          17747                          80361 
##   XE17833.0092.0100.2017.02.01   XE22903.0043.0125.2017.02.01 
##                          10751                          92243 
##   XE28163.0194.0127.2017.02.01   WM26348.0100.0113.2017.01.11 
##                          77437                          52751 
##   WM26354.0139.0315.2017.01.11   WQ64001.0145.0143.2017.01.11 
##                          45678                          39753 
##   WS20813.0155.0153.2017.01.11   WS21384.0094.0102.2017.01.11 
##                          29351                          12873 
##   WS21401.0047.0283.2017.01.11   WS21556.0141.0317.2017.01.11 
##                          65243                          57521 
##   WS21562.0055.0438.2017.01.11   WS21578.0125.0134.2017.01.11 
##                          13616                          46331 
##   WS21584.0066.0321.2017.01.11   WS22205.0097.0110.2017.01.11 
##                          28249                          65967 
##   WS74808.0165.0347.2017.01.11   WS74858.0098.0111.2017.01.11 
##                          32886                          18675 
##   WS75418.0032.0279.2017.01.11   WS76117.0102.0115.2017.01.11 
##                          66488                          78753 
##   WS76840.0117.0126.2017.01.11   WS77050.0026.0276.2017.01.11 
##                          39459                          15102 
##   WT02695.0166.0104.2017.01.11   WT02712.0078.0323.2017.01.11 
##                          20141                          38952 
##   WT05558.0190.0378.2017.01.11   WT05564.0116.0125.2017.01.11 
##                          17973                          33463 
##   WT06263.0127.0135.2017.01.11   WT07439.0058.0440.2017.01.11 
##                          35147                          43263 
##   WT08061.0090.0098.2017.01.11   WT09760.0053.0437.2017.01.11 
##                          28736                          34666 
##   WT09798.0057.0285.2017.01.11   WT10373.0080.0325.2017.01.11 
##                          78499                          37600 
##   WT10389.0036.0270.2017.01.11   WT10395.0035.0269.2017.01.11 
##                          29095                          43853 
##   WT12565.0189.0377.2017.01.11   WT14129.0153.0151.2017.01.11 
##                          26219                          58444 
##   WT14135.0123.0132.2017.01.11   WT14818.0111.0120.2017.01.11 
##                          42345                          33448 
##   WT15084.0046.0282.2017.01.11   WT15117.0157.0155.2017.01.11 
##                         122971                          41809 
##   WT15123.0164.0346.2017.01.11   WT23223.0144.0320.2017.01.11 
##                          37517                          39399 
##   WT23273.0101.0114.2017.01.11   WT24900.0152.0150.2017.01.11 
##                          32170                          25802 
##   WT24916.0044.0281.2017.01.11   WT24944.0124.0159.2017.01.11 
##                          74046                          36504 
##   WT24950.0033.0267.2017.01.11   WT24966.0142.0318.2017.01.11 
##                          54801                          37339 
##   WT27396.0121.0130.2017.01.11   WT27407.0156.0154.2017.01.11 
##                          30349                          27423 
##   WT27441.0129.0137.2017.01.11   WT30335.0134.0140.2017.01.11 
##                          25366                          76057 
##   WT30357.0049.0274.2017.01.11   WT30818.0107.0118.2017.01.11 
##                          35457                          26163 
##   WT30824.0147.0145.2017.01.11   WT30830.0052.0275.2017.01.11 
##                          45066                          41492 
##   WT34353.0120.0129.2017.01.11   WT40033.0133.0139.2017.01.11 
##                          40414                          22514 
##   WT40049.0148.0146.2017.01.11   WT40083.0038.0271.2017.01.11 
##                          60368                          23791 
##   WT40516.0122.0131.2017.01.11   WT41865.0089.0097.2017.01.11 
##                          69537                          24316 
##   WT41910.0095.0108.2017.01.11   WT42069.0154.0152.2017.01.11 
##                          43202                          25172 
##   WT43352.0087.0447.2017.01.11   WT43368.0118.0127.2017.01.11 
##                          15820                          50208 
##   WT43374.0048.0273.2017.01.11   WT44245.0081.0337.2017.01.11 
##                          57836                          54390 
##   WT44601.0082.0445.2017.01.11   WT44762.0130.0138.2017.01.11 
##                          41145                         118478 
##   WT47297.0173.0105.2017.01.11   WT48435.0099.0112.2017.01.11 
##                          30740                          39063 
##   WT48441.0112.0121.2017.01.11   WT48457.0060.0441.2017.01.11 
##                          31526                          41539 
##   WT48491.0146.0144.2017.01.11   WT48502.0106.0117.2017.01.11 
##                          28055                          63937 
##   WT50842.0187.0376.2017.01.11   WT50858.0128.0136.2017.01.11 
##                          20957                          36154 
##   WY74094.0079.0324.2017.01.11   WY74105.0151.0149.2017.01.11 
##                          55072                          67120 
##   WY74777.0073.0444.2017.01.11   WY74799.0176.0369.2017.01.11 
##                          45932                          27435 
##   WY75915.0114.0123.2017.01.11   WY75959.0191.0379.2017.01.11 
##                          27184                          28579 
##   WY75971.0062.0443.2017.01.11   WY76486.0162.0344.2017.01.11 
##                          49071                          52240 
##   WY78078.0161.0343.2017.01.11   WY78084.0061.0442.2017.01.11 
##                          29088                          15433 
##   WY78567.0119.0128.2017.01.11   WY79216.0040.0272.2017.01.11 
##                          21515                          42024 
##   WY79222.0056.0439.2017.01.11   WY79266.0113.0157.2017.01.11 
##                          27261                          55014 
##   WY79272.0177.0370.2017.01.11   WY80257.0027.0277.2017.01.11 
##                          31091                          65700 
##   WY80324.0137.0287.2017.01.11   WY81156.0104.0116.2017.01.11 
##                          37886                          41525 
##   WY81162.0169.0362.2017.01.11   WY81184.0045.0436.2017.01.11 
##                          65619                          23675 
##   XE13926.0025.0214.2017.01.11   XE13948.0192.0380.2017.01.11 
##                          25666                          43145 
##   XE13960.0150.0148.2017.01.11   XE15596.0149.0147.2017.01.11 
##                          34682                          51049 
##   XE17300.0083.0339.2017.01.11   XE21060.0015.0266.2017.01.11 
##                          60567                          39820 
##   XE21076.0182.0372.2017.01.11   XE21082.0186.0375.2017.01.11 
##                          37296                          20236 
##   XE21098.0115.0158.2017.01.11   XE22892.0171.0364.2017.01.11 
##                          42113                          50713 
##   XE22953.0096.0109.2017.01.11   XE27236.0054.0284.2017.01.11 
##                          52070                         135285 
##   XE27414.0059.0286.2017.01.11   XE28202.0028.0278.2017.01.11 
##                          51840                          44456 
##   XE29167.0135.0141.2017.01.11   XE30225.0020.0212.2017.01.11 
##                          64259                          24576 
##   XE31552.0011.0211.2017.01.11   XE33411.0143.0319.2017.01.11 
##                          20444                          38078 
##   XE36257.0175.0156.2017.01.11   XE36952.0091.0099.2017.01.11 
##                          48972                          11715 
##   XE36996.0009.0210.2017.01.11   XE38944.0167.0360.2017.01.11 
##                          38916                          26375 
##   XE38950.0140.0316.2017.01.11   XE38966.0034.0268.2017.01.11 
##                          38755                          55702 
##   XE38972.0178.0371.2017.01.11   XE38988.0110.0119.2017.01.11 
##                          35384                          32939 
##   XE38994.0185.0374.2017.01.11   XE40684.0024.0213.2017.01.11 
##                          21707                          25565 
##   XE40690.0172.0365.2017.01.11   XE40701.0163.0103.2017.01.11 
##                          46970                          46782 
##   XE40717.0010.0265.2017.01.11   XE40745.0006.0264.2017.01.11 
##                          44631                          59807 
##   XE40751.0088.0096.2017.01.11   XE41305.0193.0381.2017.01.11 
##                          17438                          27692 
##   XE41311.0138.0314.2017.01.11   XE41327.0085.0446.2017.01.11 
##                          63493                          13408 
##   XE41333.0136.0142.2017.01.11   XE41349.0168.0361.2017.01.11 
##                          44701                          26835 
##   XE41561.0037.0280.2017.01.11   XE41577.0170.0363.2017.01.11 
##                          44727                          31898 
##   XE41583.0183.0373.2017.01.11   WS19294.0064.0471.2016.11.13 
##                          26328                          58457 
##   WS20829.0126.0349.2016.11.13   WS20835.0007.0390.2016.11.13 
##                         136145                          35250 
##   WS21390.0076.0299.2016.11.13   WS21540.0031.0414.2016.11.13 
##                         144074                          52735 
##   WT02689.0077.0300.2016.11.13   WT02728.0109.0332.2016.11.13 
##                          70581                          83182 
##   WT04693.0016.0246.2016.11.13   WT04704.0071.0294.2016.11.13 
##                          60837                         133384 
##   WT07417.0184.0222.2016.11.13   WT08055.0075.0298.2016.11.13 
##                         122883                         116845 
##   WT09332.0070.0293.2016.11.13   WT09776.0074.0297.2016.11.13 
##                         159041                         156778 
##   WT10406.0003.0386.2016.11.13   WT11111.0023.0406.2016.11.13 
##                          24724                         196412 
##   WT12559.0072.0295.2016.11.13   WT14141.0002.0385.2016.11.13 
##                         192888                          57645 
##   WT15090.0180.0218.2016.11.13   WT23295.0022.0251.2016.11.13 
##                         106854                          68044 
##   WT27518.0050.0457.2016.11.13   WT27607.0039.0422.2016.11.13 
##                          32576                          90830 
##   WT30341.0012.0243.2016.11.13   WT30868.0042.0449.2016.11.13 
##                          55241                          74533 
##   WT30880.0069.0292.2016.11.13   WT34347.0181.0219.2016.11.13 
##                          81481                         103299 
##   WT37711.0068.0291.2016.11.13   WT42192.0131.0354.2016.11.13 
##                         146323                          52000 
##   WT47308.0004.0387.2016.11.13   WY76492.0067.0290.2016.11.13 
##                          54624                         132789 
##   WY78062.0019.0248.2016.11.13   WY79238.0108.0331.2016.11.13 
##                          67007                          37816 
##   WY79244.0105.0328.2016.11.13   WY80318.0103.0326.2016.11.13 
##                          16417                          53131 
##   WY82116.0188.0226.2016.11.13   XE15574.0065.0288.2016.11.13 
##                          79589                         203487 
##   XE17922.0179.0217.2016.11.13   XE17938.0014.0245.2016.11.13 
##                          93388                          67436 
##   XE18326.0084.0307.2016.11.13   XE21109.0018.0247.2016.11.13 
##                          81864                         105606 
##   XE28157.0013.0244.2016.11.13   XE28274.0051.0458.2016.11.13 
##                          94532                          24318 
##   XE29173.0008.0240.2016.11.13   XE29812.0132.0355.2016.11.13 
##                          82688                         143895 
##   XE33372.0021.0427.2016.11.13   XE36174.0063.0470.2016.11.13 
##                          74927                          92285 
##   XE37001.0030.0413.2016.11.13   XE39009.0029.0412.2016.11.13 
##                          80217                          48882 
##   XE39532.0017.0400.2016.11.13   XE39554.0041.0448.2016.11.13 
##                         205766                          65169 
##   XE40723.0001.0384.2016.11.13   XE40739.0086.0309.2016.11.13 
##                          61118                          34672 
##   XE41599.0005.0388.2016.11.13   MBA4060.0077.0266.2016.03.20 
##                          56531                          11517 
##   MBA1007.0092.0281.2016.03.11   MBA1037.0129.0437.2016.03.11 
##                          16176                          14385 
##   MBA1083.0088.0367.2016.03.11   MBA1141.0108.0297.2016.03.11 
##                          11423                          11454 
##   MBA1166.0047.0236.2016.03.11   MBA1172.0017.0444.2016.03.11 
##                          11848                          11809 
##   MBA1261.0125.0433.2016.03.11   MBA1327.0122.0368.2016.03.11 
##                          13925                          20343 
##   MBA1447.0019.0469.2016.03.11   MBA4044.0024.0470.2016.03.11 
##                          45329                          42889 
##   MBA4049.0043.0416.2016.03.11   MBA4051.0001.0446.2016.03.11 
##                          13037                          12351 
##   MBA4056.0119.0308.2016.03.11   MBA4065.0152.0341.2016.03.11 
##                          12105                          12002 
##   MBA4077.0040.0378.2016.03.11   MBA4085.0075.0264.2016.03.11 
##                          27897                          14923 
##   MBA4088.0082.0271.2016.03.11   MBA4091.0085.0429.2016.03.11 
##                           9947                          14136 
##   MBA4120.0076.0265.2016.03.11   MBA4121.0046.0235.2016.03.11 
##                          13922                          12858 
##   MBA4129.0087.0468.2016.03.11   MBA4130.0148.0337.2016.03.11 
##                          40274                          16263 
##   MBA4134.0120.0363.2016.03.11   MBA4139.0164.0353.2016.03.11 
##                          25631                          11278 
##  233202.Boston.0164.2016.02.14  930024.Boston.0114.2016.02.14 
##                          24841                          27023 
##   MBA1003.0131.0320.2016.02.14   MBA1021.0035.0460.2016.02.14 
##                          49506                          21557 
##   MBA1030.0014.0205.2016.02.14   MBA1033.0055.0244.2016.02.14 
##                          60325                          23191 
##   MBA1035.0102.0426.2016.02.14   MBA1041.0053.0242.2016.02.14 
##                          40213                          14230 
##   MBA1052.0009.0451.2016.02.14   MBA1071.0011.0202.2016.02.14 
##                          14859                          82865 
##   MBA1074.0058.0247.2016.02.14   MBA1082.0023.0463.2016.02.14 
##                          13002                          28556 
##   MBA1084.0114.0303.2016.02.14   MBA1090.0003.0194.2016.02.14 
##                          11543                         133259 
##   MBA1095.0090.0279.2016.02.14   MBA1096.0060.0249.2016.02.14 
##                          23430                          19672 
##   MBA1099.0066.0255.2016.02.14   MBA1100.0027.0386.2016.02.14 
##                          19574                          26058 
##   MBA1101.0149.0338.2016.02.14   MBA1103.0026.0385.2016.02.14 
##                          19728                          24858 
##   MBA1111.0139.0328.2016.02.14   MBA1133.0041.0453.2016.02.14 
##                          17707                          23140 
##   MBA1135.0140.0329.2016.02.14   MBA1139.0018.0445.2016.02.14 
##                           9599                          13882 
##   MBA1143.0105.0294.2016.02.14   MBA1151.0107.0296.2016.02.14 
##                          19881                          13159 
##   MBA1159.0165.0354.2016.02.14   MBA1163.0167.0356.2016.02.14 
##                          30857                          33492 
##   MBA1181.0126.0315.2016.02.14   MBA1187.0062.0251.2016.02.14 
##                          43998                          15695 
##   MBA1190.0048.0237.2016.02.14   MBA1193.0132.0321.2016.02.14 
##                          53414                          15292 
##   MBA1199.0069.0258.2016.02.14   MBA1202.0094.0456.2016.02.14 
##                          21096                          14588 
##   MBA1209.0101.0290.2016.02.14   MBA1211.0136.0325.2016.02.14 
##                          10734                          20052 
##   MBA1218.0030.0425.2016.02.14   MBA1230.0093.0282.2016.02.14 
##                          19204                          19109 
##   MBA1237.0170.0359.2016.02.14   MBA1241.0022.0419.2016.02.14 
##                          25519                          30836 
##   MBA1248.0070.0259.2016.02.14   MBA1251.0033.0467.2016.02.14 
##                          16468                          26599 
##   MBA1260.0158.0347.2016.02.14   MBA1267.0038.0227.2016.02.14 
##                          39840                          17977 
##   MBA1270.0042.0231.2016.02.14   MBA1271.0116.0462.2016.02.14 
##                          16729                          22420 
##   MBA1279.0162.0461.2016.02.14   MBA1307.0029.0372.2016.02.14 
##                          48196                          13686 
##   MBA1312.0074.0263.2016.02.14   MBA1317.0067.0256.2016.02.14 
##                          16407                          16233 
##   MBA1330.0050.0239.2016.02.14   MBA1341.0160.0349.2016.02.14 
##                          12379                          21517 
##   MBA1344.0169.0358.2016.02.14   MBA1355.0037.0458.2016.02.14 
##                          38110                          38263 
##   MBA1363.0123.0312.2016.02.14   MBA1365.0171.0360.2016.02.14 
##                          11750                          19399 
##   MBA1370.0034.0465.2016.02.14   MBA1375.0061.0250.2016.02.14 
##                          32525                          24576 
##   MBA1382.0159.0348.2016.02.14   MBA1385.0124.0313.2016.02.14 
##                          23025                          37460 
##   MBA1392.0134.0323.2016.02.14   MBA1399.0147.0336.2016.02.14 
##                          41620                          26657 
##   MBA1410.0130.0379.2016.02.14   MBA1437.0063.0252.2016.02.14 
##                          19712                          13941 
##   MBA1460.0106.0295.2016.02.14   MBA1480.0166.0355.2016.02.14 
##                           9819                          34300 
##   MBA1486.0115.0304.2016.02.14   MBA1488.0072.0452.2016.02.14 
##                          22751                          13626 
##   MBA1511.0163.0352.2016.02.14   MBA1515.0153.0342.2016.02.14 
##                          21490                          24838 
##   MBA4041.0044.0449.2016.02.14   MBA4043.0059.0248.2016.02.14 
##                          10254                          13060 
##   MBA4045.0157.0346.2016.02.14   MBA4047.0012.0203.2016.02.14 
##                          33741                          48792 
##   MBA4050.0028.0434.2016.02.14   MBA4052.0118.0457.2016.02.14 
##                          14519                          35830 
##   MBA4053.0057.0246.2016.02.14   MBA4057.0133.0322.2016.02.14 
##                          25496                           9954 
##   MBA4061.0010.0201.2016.02.14   MBA4062.0079.0393.2016.02.14 
##                         104643                          11876 
##   MBA4063.0100.0289.2016.02.14   MBA4066.0142.0430.2016.02.14 
##                          16644                          12023 
##   MBA4067.0104.0293.2016.02.14   MBA4068.0007.0198.2016.02.14 
##                          22257                          49188 
##   MBA4069.0004.0195.2016.02.14   MBA4070.0086.0478.2016.02.14 
##                          90587                          41192 
##   MBA4072.0161.0350.2016.02.14   MBA4074.0078.0267.2016.02.14 
##                          27345                          13679 
##   MBA4076.0016.0207.2016.02.14   MBA4078.0151.0340.2016.02.14 
##                          47724                          35591 
##   MBA4080.0065.0254.2016.02.14   MBA4081.0052.0241.2016.02.14 
##                          23350                          20586 
##   MBA4082.0032.0221.2016.02.14   MBA4086.0110.0299.2016.02.14 
##                          15687                          18709 
##   MBA4087.0155.0344.2016.02.14   MBA4089.0141.0330.2016.02.14 
##                          35300                          21650 
##   MBA4092.0008.0199.2016.02.14   MBA4095.0002.0193.2016.02.14 
##                          69046                         138863 
##   MBA4096.0145.0334.2016.02.14   MBA4102.0154.0343.2016.02.14 
##                          18861                          23941 
##   MBA4103.0128.0317.2016.02.14   MBA4106.0168.0357.2016.02.14 
##                          13480                          33202 
##   MBA4107.0138.0327.2016.02.14   MBA4108.0099.0288.2016.02.14 
##                          12705                          14653 
##   MBA4109.0073.0262.2016.02.14   MBA4111.0036.0459.2016.02.14 
##                          10720                          38086 
##   MBA4112.0005.0196.2016.02.14   MBA4113.0137.0396.2016.02.14 
##                          59252                          13144 
##   MBA4114.0064.0253.2016.02.14   MBA4115.0143.0332.2016.02.14 
##                          13795                           9242 
##   MBA4118.0031.0466.2016.02.14   MBA4119.0127.0316.2016.02.14 
##                          42048                          13338 
##   MBA4122.0112.0301.2016.02.14   MBA4123.0056.0245.2016.02.14 
##                          28583                          21135 
##   MBA4126.0150.0339.2016.02.14   MBA4127.0021.0417.2016.02.14 
##                           8596                          18287 
##   MBA4131.0006.0197.2016.02.14   MBA4132.0051.0240.2016.02.14 
##                         121717                          12687 
##   MBA4133.0135.0324.2016.02.14   MBA4136.0020.0464.2016.02.14 
##                          18548                          26125 
##   MBA4138.0068.0257.2016.02.14   MBA4140.0015.0206.2016.02.14 
##                          21282                          32717 
##  226855.boston.0016.2015.11.25  229075.boston.0178.2015.11.25 
##                          12316                          21754 
##  447537.boston.0170.2015.11.25  503564.boston.0169.2015.11.25 
##                          11055                          16113 
##  561130.boston.0065.2015.11.25  588800.boston.0068.2015.11.25 
##                          64533                          54160 
##  629356.boston.0076.2015.11.25  765828.boston.0089.2015.11.25 
##                          17564                          18760 
##  772512.boston.0090.2015.11.25  826391.boston.0177.2015.11.25 
##                          28599                          29717 
##  849016.boston.0176.2015.11.25  872569.boston.0179.2015.11.25 
##                          27270                          22343 
##     136109.048.0334.2018.12.15     137787.006.0293.2018.12.15 
##                         142042                         110949 
##     186400.027.0314.2018.12.15     207722.029.0316.2018.12.15 
##                         130465                         140162 
##     221355.060.0345.2018.12.15     225794.001.0288.2018.12.15 
##                         131800                         169725 
##     235185.033.0320.2018.12.15     236512.015.0302.2018.12.15 
##                         146986                         136583 
##     240884.013.0300.2018.12.15     241367.042.0329.2018.12.15 
##                         134087                         130533 
##     243736.036.0323.2018.12.15     276693.051.0337.2018.12.15 
##                         125215                         143542 
##     282036.026.0313.2018.12.15     298053.032.0319.2018.12.15 
##                         140377                         139377 
##     319820.035.0322.2018.12.15     354109.012.0299.2018.12.15 
##                         156031                         130651 
##     356403.053.0339.2018.12.15     364485.002.0289.2018.12.15 
##                         163928                         138715 
##     373167.021.0308.2018.12.15     395379.010.0297.2018.12.15 
##                          91803                         125471 
##     402235.052.0338.2018.12.15     411794.028.0315.2018.12.15 
##                         103407                         125418 
##     416151.055.0340.2018.12.15     420641.004.0291.2018.12.15 
##                         147234                         137640 
##     423443.005.0292.2018.12.15     445474.025.0312.2018.12.15 
##                         153787                         162503 
##     453551.040.0327.2018.12.15     459452.044.0331.2018.12.15 
##                         149435                         105418 
##     467375.007.0294.2018.12.15     471179.003.0290.2018.12.15 
##                         148513                         139922 
##     492275.058.0343.2018.12.15     570298.041.0328.2018.12.15 
##                         165014                         135343 
##     574541.045.0332.2018.12.15     576662.030.0317.2018.12.15 
##                         124317                         151416 
##     578598.017.0304.2018.12.15     580176.038.0325.2018.12.15 
##                         121232                         141927 
##     588443.031.0318.2018.12.15     588873.064.0349.2018.12.15 
##                          94483                         109394 
##     612872.019.0306.2018.12.15     667045.043.0330.2018.12.15 
##                         139365                         116480 
##     675294.009.0296.2018.12.15     684908.056.0341.2018.12.15 
##                         138860                         122542 
##     714983.057.0342.2018.12.15     720750.034.0321.2018.12.15 
##                         145724                         121774 
##     774051.062.0347.2018.12.15     821034.039.0326.2018.12.15 
##                         147680                         119747 
##     822655.037.0324.2018.12.15     823946.047.0333.2018.12.15 
##                          93713                         121833 
##     850240.022.0309.2018.12.15     851204.014.0301.2018.12.15 
##                         107960                         145594 
##     853781.063.0348.2018.12.15     857829.018.0305.2018.12.15 
##                         130937                         136894 
##     883092.049.0335.2018.12.15     909074.024.0311.2018.12.15 
##                         126602                          83194 
##     918320.061.0346.2018.12.15     938150.059.0344.2018.12.15 
##                         150659                         159642 
##     945496.008.0295.2018.12.15     948983.050.0336.2018.12.15 
##                         130406                         160584 
##     982757.020.0307.2018.12.15     985170.011.0298.2018.12.15 
##                         135822                         132462 
##    MBA1008.062.0371.2018.12.15    MBA1073.103.0380.2018.12.15 
##                          97776                         121454 
##    MBA1144.144.0244.2018.12.15    MBA1215.004.0359.2018.12.15 
##                          58436                         141749 
##    MBA1226.010.0360.2018.12.15    MBA1240.156.0246.2018.12.15 
##                         118025                         123306 
##    MBA1284.025.0362.2018.12.15    MBA1302.089.0375.2018.12.15 
##                         106064                         122557 
##    MBA1315.035.0365.2018.12.15    MBA1398.080.0374.2018.12.15 
##                         122382                         124340 
##    MBA1491.070.0372.2018.12.15    MBA1509.039.0366.2018.12.15 
##                         141649                         123099 
##    MBA1519.096.0377.2018.12.15    MBA4042.011.0361.2018.12.15 
##                         105745                         135959 
##    MBA4046.032.0363.2018.12.15    MBA4048.146.0245.2018.12.15 
##                         121271                         104141 
##    MBA4058.049.0368.2018.12.15    MBA4059.113.0242.2018.12.15 
##                         163357                         154949 
##    MBA4064.117.0243.2018.12.15    MBA4071.098.0379.2018.12.15 
##                          99265                         173599 
##    MBA4083.097.0378.2018.12.15    MBA4093.033.0364.2018.12.15 
##                         131209                         131778 
##    MBA4099.045.0367.2018.12.15    MBA4104.091.0376.2018.12.15 
##                         140969                         128881 
##    MBA4128.059.0369.2018.12.15 
##                         142304
```

```
min(rowSums(OTU_table))
```

```
## [1] 8596
```

```
Total_counts<-as.data.frame(rowSums(OTU_table))
colnames(Total_counts)<-c("Counts")
ggplot(Total_counts, aes(x=Counts))+geom_histogram(binwidth=100)+geom_vline(aes(xintercept=mean(Counts, na.rm=T)), color="red", linetype="dashed", size=1)+theme_bw()+
  ggtitle("Total count per sample distribution")+theme(plot.title=element_text(lineheight=10, size=15))+
  xlab("Counts")+ylab("Number of samples")+theme(axis.text=element_text(size=15), axis.title=element_text(size=15))
```

```
#Remove all those samples that do not reach a minimum threshold of number of reads:
counts<-as.data.frame(rowSums(OTU_table))
colnames(counts)<-"counts"
counts$Sample<-row.names(counts)
subset_8000<-counts[counts$counts>=8000,]
subset_8000$Sample<-NULL
OTU_table<-OTU_table[row.names(OTU_table)%in%as.vector(row.names(subset_8000)),]

#Rarefy to same sequencing depth:
set.seed(1)
OTU_table_8000<-vegan::rrarefy(OTU_table, 8000)
```

```
## Warning in vegan::rrarefy(OTU_table, 8000): function should be used for
## observed counts, but smallest count is 2
```

```
#Estimate richness:
richness_8000<-vegan::estimateR(OTU_table_8000)
richness_8000<-t(richness_8000)
richness_8000<-as.data.frame(richness_8000)
richness_8000$se.chao1<-NULL
richness_8000$se.ACE<-NULL

#Estimate evenness:
shannon<-BiodiversityR::diversityresult(x=OTU_table_8000, method="each site", index="Shannon")
diversity_8000<-cbind(shannon)

#Join data from richness and evenness calculations:
ecology_8000<-cbind(richness_8000, diversity_8000)
colnames(ecology_8000)<-c("Observed", "Chao1", "ACE", "Shannon")

#Subset metadata
metadata<-metadata[row.names(metadata)%in%row.names(ecology_8000),,drop=FALSE]

all.equal(row.names(metadata),row.names(ecology_8000))
```

```
## [1] TRUE
```

```
ecology_8000$SampleID<-metadata$SampleID
ecology_8000$Cohort<-metadata$sample_cohort
ecology_8000$hiv_phenotype<-metadata$hiv_phenotype
ecology_8000$sexual_orientation<-metadata$sexual_orientation

#Subset indivudals for this comparison
ecology_8000_nomsm<-ecology_8000[ecology_8000$sexual_orientation!="MSM",,drop=FALSE]
ecology_8000_nomsm_melt<-reshape2::melt(ecology_8000_nomsm)
```

```
## Using SampleID, Cohort, hiv_phenotype, sexual_orientation as id variables
```

```
ecology_8000_nomsm_melt_Observed<-ecology_8000_nomsm_melt[ecology_8000_nomsm_melt$variable=="Observed",,drop=F]
ecology_8000_nomsm_melt_Shannon<-ecology_8000_nomsm_melt[ecology_8000_nomsm_melt$variable=="Shannon",,drop=F]

plot_Observed<-ggplot(data=ecology_8000_nomsm_melt_Observed, aes(x=hiv_phenotype,y=value))+
  geom_boxplot(aes(fill=Cohort, alpha=hiv_phenotype), outlier.color="white")+theme_bw()+
  scale_fill_manual(values=c("royalblue4","darkorange","darkgreen"))+
  geom_point(aes(color=Cohort, alpha=hiv_phenotype), position=position_jitterdodge(jitter.width=0.25), size=1)+
  facet_wrap(~Cohort, scales="free_x", nrow=1)+
  scale_alpha_manual(values=c(0.9, 0.6, 0.3))+
  scale_color_manual(values=c("royalblue4","darkorange","darkgreen"))+
  theme(axis.text.x = element_text(angle=90))

plot_Shannon<-ggplot(data=ecology_8000_nomsm_melt_Shannon, aes(x=hiv_phenotype,y=value))+
  geom_boxplot(aes(fill=Cohort, alpha=hiv_phenotype), outlier.color="white")+theme_bw()+
  scale_fill_manual(values=c("royalblue4","darkorange","darkgreen"))+
  geom_point(aes(color=Cohort, alpha=hiv_phenotype), position=position_jitterdodge(jitter.width=0.25), size=1)+
  facet_wrap(~Cohort, scales="free_x", nrow=1)+
  scale_alpha_manual(values=c(0.9, 0.6, 0.3))+
  scale_color_manual(values=c("royalblue4","darkorange","darkgreen"))+
  theme(axis.text.x = element_text(angle=90))

ggsave("SupplementaryFigure4B.pdf", grid.arrange(plot_Observed, plot_Shannon, nrow=2), width=15, height=10)
```

```
#Statistical testing:
us<-ecology_8000_nomsm[ecology_8000_nomsm$Cohort=="boston",,drop=F]
botswana<-ecology_8000_nomsm[ecology_8000_nomsm$Cohort=="botswana",,drop=F]
uganda<-ecology_8000_nomsm[ecology_8000_nomsm$Cohort=="uganda_2",,drop=F]
# Add all metadata to the table for multivariate testing of abundance differences
metadata$age <- as.numeric(metadata$age)
metadata$BMI <- as.numeric(metadata$BMI)
### metadata not collected in Boston:
metadata_ordered$monthly_income <- as.numeric(metadata_ordered$monthly_income)
metadata_ordered$smoking_years <- as.numeric(metadata_ordered$smoking_years)
metadata_ordered$fram_10yr_risk_lab <- as.numeric(metadata_ordered$fram_10yr_risk_lab)
metadata_ordered$fram_10yr_risk_nonlab <- as.numeric(metadata_ordered$fram_10yr_risk_nonlab)
metadata_ordered$mean_imt <- as.numeric(metadata_ordered$mean_imt)
metadata_ordered$total_plaques <- as.numeric(metadata_ordered$total_plaques)
metadata_ordered$any_plaques <- as.numeric(metadata_ordered$any_plaques)
dplyr::left_join(us[colnames(us) %in% c("SampleID") | !colnames(us) %in% colnames(metadata)], metadata, by = "SampleID") -> us_full_metadata
dplyr::left_join(botswana[colnames(botswana) %in% c("SampleID") | !colnames(botswana) %in% colnames(metadata)], metadata, by = "SampleID") -> botswana_full_metadata
dplyr::left_join(uganda[colnames(uganda) %in% c("SampleID") | !colnames(uganda) %in% colnames(metadata)], metadata, by = "SampleID") -> uganda_full_metadata

# Kruskal and wilcox testing
#US
for (i in c("Observed", "Shannon")){
  print(i)
  print(kruskal(us[[i]], us[["hiv_phenotype"]],group=F,p.adj = "bonferroni"))
}
```

```
## [1] "Observed"
## $statistics
##        Chisq Df    p.chisq
##   0.51695815  2 0.77222519
## 
## $parameters
##             test  p.ajusted                name.t ntr alpha
##   Kruskal-Wallis bonferroni us[["hiv_phenotype"]]   3  0.05
## 
## $means
##                  us..i..      rank       std  r Min Max   Q25 Q50   Q75
## 1_hiv_negative 138.62353 56.711765 48.321093 85  61 249  96.0 137 175.0
## 2_suppressed   146.84211 60.842105 52.775482 19  79 300 112.0 130 177.0
## 4_unsuppressed 154.72727 63.045455 76.950752 11  46 333 116.5 146 186.5
## 
## $comparison
##                                 Difference pvalue Signif.        LCL       UCL
## 1_hiv_negative - 2_suppressed   -4.1303406      1         -24.827767 16.567086
## 1_hiv_negative - 4_unsuppressed -6.3336898      1         -32.468284 19.800904
## 2_suppressed - 4_unsuppressed   -2.2033493      1         -33.104413 28.697715
## 
## $groups
## NULL
## 
## attr(,"class")
## [1] "group"
## [1] "Shannon"
## $statistics
##        Chisq Df    p.chisq
##   0.28118102  2 0.86884502
## 
## $parameters
##             test  p.ajusted                name.t ntr alpha
##   Kruskal-Wallis bonferroni us[["hiv_phenotype"]]   3  0.05
## 
## $means
##                  us..i..      rank        std  r       Min       Max       Q25
## 1_hiv_negative 3.4220815 57.035294 0.68648328 85 1.4674836 4.4513155 3.0708710
## 2_suppressed   3.5331089 61.157895 0.62121210 19 1.7319634 4.5236775 3.1442770
## 4_unsuppressed 3.4551810 60.000000 0.78369881 11 1.5978440 4.3767472 3.2269193
##                      Q50       Q75
## 1_hiv_negative 3.5533268 3.9279747
## 2_suppressed   3.7108917 3.9567380
## 4_unsuppressed 3.5800493 3.9414740
## 
## $comparison
##                                 Difference pvalue Signif.        LCL       UCL
## 1_hiv_negative - 2_suppressed   -4.1226006      1         -24.843806 16.598605
## 1_hiv_negative - 4_unsuppressed -2.9647059      1         -29.129326 23.199914
## 2_suppressed - 4_unsuppressed    1.1578947      1         -29.778671 32.094461
## 
## $groups
## NULL
## 
## attr(,"class")
## [1] "group"
```

```
#Botswana
for (i in c("Observed", "Shannon")){
  print(i)
  print(kruskal(botswana[[i]], botswana[["hiv_phenotype"]],group=F,p.adj = "bonferroni"))
}
```

```
## [1] "Observed"
## $statistics
##       Chisq Df    p.chisq
##   3.5969225  2 0.16555344
## 
## $parameters
##             test  p.ajusted                      name.t ntr alpha
##   Kruskal-Wallis bonferroni botswana[["hiv_phenotype"]]   3  0.05
## 
## $means
##                botswana..i..       rank       std  r Min Max Q25 Q50    Q75
## 1_hiv_negative     136.41250 100.975000 55.522306 80  32 268  93 131 171.25
## 2_suppressed       123.69863  88.150685 52.500181 73  35 294  81 119 163.00
## 4_unsuppressed     141.19512 107.365854 61.013613 41  13 270 101 137 179.00
## 
## $comparison
##                                  Difference pvalue Signif.         LCL
## 1_hiv_negative - 2_suppressed    12.8243151 0.4742          -9.0319041
## 1_hiv_negative - 4_unsuppressed  -6.3908537 1.0000         -32.3261676
## 2_suppressed - 4_unsuppressed   -19.2151687 0.2395         -45.5684570
##                                        UCL
## 1_hiv_negative - 2_suppressed   34.6805343
## 1_hiv_negative - 4_unsuppressed 19.5444603
## 2_suppressed - 4_unsuppressed    7.1381196
## 
## $groups
## NULL
## 
## attr(,"class")
## [1] "group"
## [1] "Shannon"
## $statistics
##       Chisq Df    p.chisq
##   2.1165807  2 0.34704863
## 
## $parameters
##             test  p.ajusted                      name.t ntr alpha
##   Kruskal-Wallis bonferroni botswana[["hiv_phenotype"]]   3  0.05
## 
## $means
##                botswana..i..       rank        std  r       Min       Max
## 1_hiv_negative     3.1739522  99.912500 0.81095019 80 1.1161733 4.5147266
## 2_suppressed       3.0374787  90.424658 0.73681101 73 1.1496331 4.5307380
## 4_unsuppressed     3.2553092 105.390244 0.89526564 41 1.5072167 4.8490515
##                      Q25       Q50       Q75
## 1_hiv_negative 2.5829754 3.1852682 3.7863951
## 2_suppressed   2.6008279 3.1411895 3.5201833
## 4_unsuppressed 2.6495564 3.4153120 3.9651143
## 
## $comparison
##                                  Difference pvalue Signif.        LCL       UCL
## 1_hiv_negative - 2_suppressed     9.4878425 0.8930         -12.455174 31.430858
## 1_hiv_negative - 4_unsuppressed  -5.4777439 1.0000         -31.516054 20.560566
## 2_suppressed - 4_unsuppressed   -14.9655864 0.5205         -41.423531 11.492358
## 
## $groups
## NULL
## 
## attr(,"class")
## [1] "group"
```

```
#Uganda
for (i in c("Observed", "Shannon")){
  print(i)
  print(wilcox.test(uganda[[i]]~uganda[["hiv_phenotype"]]))
}
```

```
## [1] "Observed"
## 
##  Wilcoxon rank sum test with continuity correction
## 
## data:  uganda[[i]] by uganda[["hiv_phenotype"]]
## W = 4082, p-value = 0.13275
## alternative hypothesis: true location shift is not equal to 0
## 
## [1] "Shannon"
## 
##  Wilcoxon rank sum test with continuity correction
## 
## data:  uganda[[i]] by uganda[["hiv_phenotype"]]
## W = 4263, p-value = 0.038612
## alternative hypothesis: true location shift is not equal to 0
```

```
### Run orm (n=479) and compare alpha diversity by hiv_phenotype
### Extra metadata that have full n: Race, Ethnicity, age, sex, current_art_class_consolid2, tmp_smx_active
### Extra metadata that have <n: BMI, comorbidities (dm2_hx, hld_hx, htn_hx, cvd_hx, ckd_hx, cvd_dx [missing boston], dm2hx_dx, hldhx_dx, htnhx_dx, cvdhx_dx, ever_smoke, current_smoke, smoke_cat), school_level [uganda2 only], monthly_income[uganda2 only], current_art_class_consolid2, tmp_smx_active, days_on_art, sexual_orientation
### additional: smoking_years, fram_10yr_risk_lab, fram_10yr_risk_nonlab, mean_imt, total_plaques, any_plaques
covars_full_n <- c("Ethnicity", "age", "Race", "sex", "current_art_class_consolid2", "tmp_smx_active", "hiv_phenotype")
orm_us_full_n <- list()
orm_botswana_full_n <- list()
orm_uganda_full_n <- list()

#US
for (i in c("Observed", "Shannon")){
  print(i)
  print(rms::orm(formula =  as.formula(paste("us_full_metadata[[",which(stringr::str_detect(colnames(us_full_metadata), i)), "]]~", paste(covars_full_n, collapse = "+"), sep = "")), data = us_full_metadata))
}
```

```
## [1] "Observed"
```

```
## Warning in .local(x, ...): singularity problem
```

```
## Warning in .local(x, ...): singularity problem

## Warning in .local(x, ...): singularity problem

## Warning in .local(x, ...): singularity problem
```

```
## Logistic (Proportional Odds) Ordinal Regression Model
## 
## rms::orm(formula = as.formula(paste("us_full_metadata[[", which(stringr::str_detect(colnames(us_full_metadata), 
##     i)), "]]~", paste(covars_full_n, collapse = "+"), sep = "")), 
##     data = us_full_metadata)
## 
##                       Model Likelihood               Discrimination    Rank Discrim.    
##                             Ratio Test                      Indexes          Indexes    
## Obs           115    LR chi2     12.57    R2                  0.104    rho     0.304    
## Distinct Y     84    d.f.           15    R2(15,115)          0.000                     
## Median Y      137    Pr(> chi2) 0.6352    R2(15,115)          0.000                     
## max |deriv| 8e-06    Score chi2  13.17    |Pr(Y>=median)-0.5| 0.108                     
##                      Pr(> chi2) 0.5894                                                  
## 
##                                             Coef    S.E.   Wald Z Pr(>|Z|)
## Ethnicity=Not_Hispanic_Latino                0.2721 0.6897  0.39  0.6931  
## age                                          0.0150 0.0145  1.03  0.3014  
## Race=Asian                                   0.3337 2.0111  0.17  0.8682  
## Race=Black_AA                               -0.4620 1.7792 -0.26  0.7951  
## Race=Unknown                                 0.7919 2.1310  0.37  0.7102  
## Race=Varied                                 -1.2391 2.0451 -0.61  0.5446  
## Race=White                                  -0.1114 1.7907 -0.06  0.9504  
## sex=male                                     0.5333 0.3685  1.45  0.1479  
## current_art_class_consolid2=none            -0.7553 1.0522 -0.72  0.4729  
## current_art_class_consolid2=NRTI_NRTI_ISTI  -0.7813 1.2011 -0.65  0.5154  
## current_art_class_consolid2=NRTI_NRTI_NNRTI -2.0581 1.3443 -1.53  0.1258  
## current_art_class_consolid2=NRTI_NRTI_PI     0.3942 1.2576  0.31  0.7539  
## tmp_smx_active                               0.0000 0.0000                
## hiv_phenotype=2_suppressed                   0.0000 0.0000   Inf  <0.0001 
## hiv_phenotype=4_unsuppressed                 0.6795 0.6435  1.06  0.2910  
## 
## [1] "Shannon"
```

```
## Warning in .local(x, ...): singularity problem

## Warning in .local(x, ...): singularity problem

## Warning in .local(x, ...): singularity problem

## Warning in .local(x, ...): singularity problem

## Warning in .local(x, ...): singularity problem
```

```
## Logistic (Proportional Odds) Ordinal Regression Model
## 
## rms::orm(formula = as.formula(paste("us_full_metadata[[", which(stringr::str_detect(colnames(us_full_metadata), 
##     i)), "]]~", paste(covars_full_n, collapse = "+"), sep = "")), 
##     data = us_full_metadata)
## 
##                          Model Likelihood               Discrimination    Rank Discrim.    
##                                Ratio Test                      Indexes          Indexes    
## Obs              115    LR chi2      7.14    R2                  0.060    rho     0.211    
## Distinct Y       115    d.f.           15    R2(15,115)          0.000                     
## Median Y    3.572198    Pr(> chi2) 0.9536    R2(15,115)          0.000                     
## max |deriv|   0.0002    Score chi2   8.18    |Pr(Y>=median)-0.5| 0.070                     
##                         Pr(> chi2) 0.9162                                                  
## 
##                                             Coef    S.E.         Wald Z
## Ethnicity=Not_Hispanic_Latino                0.5303       0.6804  0.78 
## age                                         -0.0193       0.0143 -1.34 
## Race=Asian                                  -2.1920       1.9948 -1.10 
## Race=Black_AA                               -1.5671       1.7907 -0.88 
## Race=Unknown                                -2.3322       2.1447 -1.09 
## Race=Varied                                 -2.0524       2.0240 -1.01 
## Race=White                                  -1.6893       1.8001 -0.94 
## sex=male                                     0.3597       0.3707  0.97 
## current_art_class_consolid2=none            -2.3442 1086969.3823  0.00 
## current_art_class_consolid2=NRTI_NRTI_ISTI  -0.5918       1.2113 -0.49 
## current_art_class_consolid2=NRTI_NRTI_NNRTI -1.7697       1.3239 -1.34 
## current_art_class_consolid2=NRTI_NRTI_PI    -0.5492       1.2814 -0.43 
## tmp_smx_active                               0.0000       0.0000       
## hiv_phenotype=2_suppressed                  -1.4446 1086969.3823  0.00 
## hiv_phenotype=4_unsuppressed                 0.4065       0.6552  0.62 
##                                             Pr(>|Z|)
## Ethnicity=Not_Hispanic_Latino               0.4357  
## age                                         0.1790  
## Race=Asian                                  0.2718  
## Race=Black_AA                               0.3815  
## Race=Unknown                                0.2769  
## Race=Varied                                 0.3106  
## Race=White                                  0.3480  
## sex=male                                    0.3318  
## current_art_class_consolid2=none            1.0000  
## current_art_class_consolid2=NRTI_NRTI_ISTI  0.6251  
## current_art_class_consolid2=NRTI_NRTI_NNRTI 0.1813  
## current_art_class_consolid2=NRTI_NRTI_PI    0.6682  
## tmp_smx_active                                      
## hiv_phenotype=2_suppressed                  1.0000  
## hiv_phenotype=4_unsuppressed                0.5350
```

```
#Botswana
for (i in c("Observed", "Shannon")){
  print(i)
  print(rms::orm(formula =  as.formula(paste("botswana_full_metadata[[",which(stringr::str_detect(colnames(botswana_full_metadata), i)), "]]~", paste(covars_full_n[!covars_full_n %in% c("Ethnicity", "Race")], collapse = "+"), sep = "")), data = botswana_full_metadata))
}
```

```
## [1] "Observed"
```

```
## Warning in .local(x, ...): singularity problem

## Warning in .local(x, ...): singularity problem

## Warning in .local(x, ...): singularity problem

## Warning in .local(x, ...): singularity problem
```

```
## Logistic (Proportional Odds) Ordinal Regression Model
## 
## rms::orm(formula = as.formula(paste("botswana_full_metadata[[", 
##     which(stringr::str_detect(colnames(botswana_full_metadata), 
##         i)), "]]~", paste(covars_full_n[!covars_full_n %in% c("Ethnicity", 
##         "Race")], collapse = "+"), sep = "")), data = botswana_full_metadata)
## 
##                       Model Likelihood               Discrimination    Rank Discrim.    
##                             Ratio Test                      Indexes          Indexes    
## Obs           194    LR chi2      9.65    R2                  0.049    rho     0.208    
## Distinct Y    124    d.f.            8    R2(8,194)           0.008                     
## Median Y      129    Pr(> chi2) 0.2907    R2(8,194)           0.008                     
## max |deriv| 0.007    Score chi2  10.65    |Pr(Y>=median)-0.5| 0.064                     
##                      Pr(> chi2) 0.2221                                                  
## 
##                                             Coef    S.E.   Wald Z Pr(>|Z|)
## age                                          0.0168 0.0245  0.68  0.4934  
## sex=male                                    -0.3686 0.2590 -1.42  0.1547  
## current_art_class_consolid2=none             2.0381 1.0193  2.00  0.0455  
## current_art_class_consolid2=NRTI_NRTI_NNRTI  1.7309 1.0248  1.69  0.0912  
## current_art_class_consolid2=NRTI_NRTI_PI     1.4525 1.0847  1.34  0.1805  
## tmp_smx_active                               0.0000 0.0000                
## hiv_phenotype=2_suppressed                   0.0000 0.0000   Inf  <0.0001 
## hiv_phenotype=4_unsuppressed                 0.1679 0.3434  0.49  0.6248  
## 
## [1] "Shannon"
```

```
## Warning in .local(x, ...): singularity problem

## Warning in .local(x, ...): singularity problem

## Warning in .local(x, ...): singularity problem

## Warning in .local(x, ...): singularity problem
```

```
## Logistic (Proportional Odds) Ordinal Regression Model
## 
## rms::orm(formula = as.formula(paste("botswana_full_metadata[[", 
##     which(stringr::str_detect(colnames(botswana_full_metadata), 
##         i)), "]]~", paste(covars_full_n[!covars_full_n %in% c("Ethnicity", 
##         "Race")], collapse = "+"), sep = "")), data = botswana_full_metadata)
## 
##                           Model Likelihood               Discrimination    Rank Discrim.    
##                                 Ratio Test                      Indexes          Indexes    
## Obs               194    LR chi2      6.33    R2                  0.032    rho     0.161    
## Distinct Y        194    d.f.            8    R2(8,194)           0.000                     
## Median Y    3.1947071    Pr(> chi2) 0.6104    R2(8,194)           0.000                     
## max |deriv|     4e-06    Score chi2   6.44    |Pr(Y>=median)-0.5| 0.062                     
##                          Pr(> chi2) 0.5985                                                  
## 
##                                             Coef    S.E.        Wald Z Pr(>|Z|)
## age                                          0.0457      0.0259  1.77  0.0768  
## sex=male                                    -0.0177      0.2565 -0.07  0.9450  
## current_art_class_consolid2=none            -1.6504 900318.0211  0.00  1.0000  
## current_art_class_consolid2=NRTI_NRTI_NNRTI  0.9288      1.0963  0.85  0.3969  
## current_art_class_consolid2=NRTI_NRTI_PI     1.1836      1.1477  1.03  0.3024  
## tmp_smx_active                               0.0000      0.0000                
## hiv_phenotype=2_suppressed                  -2.9390 900318.0211  0.00  1.0000  
## hiv_phenotype=4_unsuppressed                 0.2235      0.3553  0.63  0.5293
```

```
#Uganda
for (i in c("Observed", "Shannon")){
  print(i)
  print(rms::orm(formula =  as.formula(paste("uganda_full_metadata[[",which(stringr::str_detect(colnames(uganda_full_metadata), i)), "]]~", paste(covars_full_n[!covars_full_n %in% c("Ethnicity", "Race")], collapse = "+"), sep = "")), data = uganda_full_metadata))
}
```

```
## [1] "Observed"
```

```
## Warning in .local(x, ...): singularity problem

## Warning in .local(x, ...): singularity problem

## Warning in .local(x, ...): singularity problem

## Warning in .local(x, ...): singularity problem
```

```
## Logistic (Proportional Odds) Ordinal Regression Model
## 
## rms::orm(formula = as.formula(paste("uganda_full_metadata[[", 
##     which(stringr::str_detect(colnames(uganda_full_metadata), 
##         i)), "]]~", paste(covars_full_n[!covars_full_n %in% c("Ethnicity", 
##         "Race")], collapse = "+"), sep = "")), data = uganda_full_metadata)
## 
##                       Model Likelihood               Discrimination    Rank Discrim.    
##                             Ratio Test                      Indexes          Indexes    
## Obs           170    LR chi2      8.32    R2                  0.048    rho     0.239    
## Distinct Y    112    d.f.            6    R2(6,170)           0.014                     
## Median Y      104    Pr(> chi2) 0.2156    R2(6,170)           0.014                     
## max |deriv| 2e-06    Score chi2   8.35    |Pr(Y>=median)-0.5| 0.072                     
##                      Pr(> chi2) 0.2136                                                  
## 
##                                             Coef    S.E.   Wald Z Pr(>|Z|)
## age                                          0.0262 0.0193  1.36  0.1744  
## sex=male                                    -0.4011 0.2721 -1.47  0.1404  
## current_art_class_consolid2=NRTI_NRTI_NNRTI  0.5012 0.6077  0.82  0.4096  
## current_art_class_consolid2=NRTI_NRTI_PI     0.0000 0.0000  -Inf  <0.0001 
## tmp_smx_active                               0.3743 0.6369  0.59  0.5568  
## hiv_phenotype=2_suppressed                  -1.1740 0.7862 -1.49  0.1354  
## 
## [1] "Shannon"
```

```
## Warning in .local(x, ...): singularity problem

## Warning in .local(x, ...): singularity problem
```

```
## Logistic (Proportional Odds) Ordinal Regression Model
## 
## rms::orm(formula = as.formula(paste("uganda_full_metadata[[", 
##     which(stringr::str_detect(colnames(uganda_full_metadata), 
##         i)), "]]~", paste(covars_full_n[!covars_full_n %in% c("Ethnicity", 
##         "Race")], collapse = "+"), sep = "")), data = uganda_full_metadata)
## 
##                           Model Likelihood               Discrimination    Rank Discrim.    
##                                 Ratio Test                      Indexes          Indexes    
## Obs               170    LR chi2      7.92    R2                  0.046    rho     0.215    
## Distinct Y        170    d.f.            6    R2(6,170)           0.011                     
## Median Y    3.3660425    Pr(> chi2) 0.2438    R2(6,170)           0.011                     
## max |deriv|     7e-07    Score chi2   7.93    |Pr(Y>=median)-0.5| 0.078                     
##                          Pr(> chi2) 0.2432                                                  
## 
##                                             Coef    S.E.         Wald Z
## age                                          0.0291       0.0192  1.51 
## sex=male                                    -0.1940       0.2720 -0.71 
## current_art_class_consolid2=NRTI_NRTI_NNRTI  0.5693 9895937.6933  0.00 
## current_art_class_consolid2=NRTI_NRTI_PI     0.1058 9895937.6933  0.00 
## tmp_smx_active                              -0.3310       0.7030 -0.47 
## hiv_phenotype=2_suppressed                  -0.7765 9895937.6933  0.00 
##                                             Pr(>|Z|)
## age                                         0.1298  
## sex=male                                    0.4756  
## current_art_class_consolid2=NRTI_NRTI_NNRTI 1.0000  
## current_art_class_consolid2=NRTI_NRTI_PI    1.0000  
## tmp_smx_active                              0.6377  
## hiv_phenotype=2_suppressed                  1.0000
```

```
###*** Observed hiv_phenotype p = 1 Shannon hiv_phenotype p = 0.4395
#--------------------------------------------------------------------------------------------------------------
```
