# Supplementary material for: HIV-associated gut microbial alterations are dependent on host and geographic context
Source: Nat Commun. 2024 Feb 5;15:1055. doi: 10.1038/s41467-023-44566-4 (PMC10844288; doi:10.1038/s41467-023-44566-4)
Supplement: Supplementary file 15 — SupplementaryFigure5_Rocafort-Gootenberg_2023_02_07 [file 41467_2023_44566_MOESM15_ESM.html]

Rocafort-Gootenberg\_SupplementaryFigure5


# Rocafort-Gootenberg\_SupplementaryFigure5

#Load needed R packages

```
library("phyloseq")
library("ggplot2")
library("gridExtra")
library("ANCOMBC")
library("dplyr")
```

```
## 
## Attaching package: 'dplyr'
```

```
## The following object is masked from 'package:gridExtra':
## 
##     combine
```

```
## The following objects are masked from 'package:stats':
## 
##     filter, lag
```

```
## The following objects are masked from 'package:base':
## 
##     intersect, setdiff, setequal, union
```

```
library("knitr") 
library("reshape")
```

```
## 
## Attaching package: 'reshape'
```

```
## The following object is masked from 'package:dplyr':
## 
##     rename
```

```
library("forcats")
```

#Load original phyloseq oject output from DADA2 pipeline

```
ps_gg_fp_f_prevalence_filter_2019_05_26<-readRDS("ps_gg_fp_f_prevalence_filter_2019_05_26")
#Fix randomness
set.seed(1)
```

#Supplementary Figure 5AC

```
#SUPPLEMENTARY FIGURE 5A & 5C
#--------------------------------------------------------------------------------------------------------------
#US
#Load results from ANCOM comparisons in Figure 2 for the U.S. cohort
comp1<-read.csv("ANCOM_US_NEG_ART_Filtered.csv", header=TRUE)
comp2<-read.csv("ANCOM_US_NEG_UNTREAT_Filtered.csv", header=TRUE)

dim(comp1)
```

```
## [1] 53 20
```

```
dim(comp2)
```

```
## [1] 117  20
```

```
#Find shared ASVs
common<-intersect(comp1$taxon, comp2$taxon)

comp1<-comp1[comp1$taxon%in%common,,drop=F]
comp2<-comp2[comp2$taxon%in%common,,drop=F]

comp1$Group<-"ART"
comp2$Group<-"UNTREATED"
all.equal(as.character(comp1$taxon), as.character(comp2$taxon))
```

```
## [1] TRUE
```

```
#Combine shared ASVs datasets
sigtab_dataset<-rbind(comp1, comp2)
sigtab_dataset <- dplyr::rename(sigtab_dataset, Genus_Species_all = Genus_Species)
sigtab_dataset <- tidyr::unite(sigtab_dataset, col =  "Genus_Species_all_for_color", Genus, Species, sep = " ", remove = FALSE)

# Remove extra brackets around taxonomic name for labels
sigtab_dataset$Genus_label <- stringr::str_replace(sigtab_dataset$Genus, "^\\[([^\\]]+)\\]", "\\1")
sigtab_dataset$Species_label <- stringr::str_replace(sigtab_dataset$Species, "^\\[([^\\]]+)\\]", "\\1")
sigtab_dataset %>% dplyr::mutate(Genus_species_label =
                dplyr::case_when(is.na(stringr::str_extract(Genus_label, "\\[")) & is.na(stringr::str_extract(Species_label, "\\[")) ~ paste(Genus_label,Species_label),
                                 TRUE ~ paste(Genus_label,"sp"))) -> sigtab_dataset
sigtab_dataset$Genus_species_label <- stringr::str_replace(sigtab_dataset$Genus_species_label, "\\[([^\\]]+)\\]", "")
sigtab_dataset$Genus_species_label <- stringr::str_replace(sigtab_dataset$Genus_species_label, "\\ +", " ")
sigtab_dataset <- dplyr::arrange(sigtab_dataset, lfc)
sigtab_dataset$taxon<-factor(sigtab_dataset$taxon, levels = dplyr::distinct(sigtab_dataset, taxon, .keep_all = TRUE)$taxon)


# Make lookup table for Genus_species_label
Genus_species_label_lookup <- dplyr::distinct(sigtab_dataset, taxon, .keep_all = TRUE)$Genus_species_label
names(Genus_species_label_lookup) <- dplyr::distinct(sigtab_dataset, taxon, .keep_all = TRUE)$taxon

#Load color dictionary:
dictionary_unique <- read.csv("COLOR_DICTIONARY3.csv", sep=";")
sigtab_dataset$Genus_species_label <- factor(sigtab_dataset$Genus_species_label, levels = unique(sigtab_dataset$Genus_species_label))
dictionary_plot <- dictionary_unique[dictionary_unique$Genus_species_label%in%sigtab_dataset$Genus_species_label,,drop=F]
rownames(dictionary_plot) <- dictionary_plot$Genus_species_label
dictionary_plot <- dictionary_plot[as.character(unique(sigtab_dataset$Genus_species_label)),,drop=F]

#Let's plot the data for U.S:
plot_comp_us <- 
  ggplot(data = sigtab_dataset, aes(x = taxon, y = lfc)) +
  geom_bar(stat = "identity", width = 0.9, position = "dodge", aes(group = Group, fill = Genus_species_label, alpha = Group)) +
  scale_fill_manual(values = as.character(dictionary_plot$Color)) +
  scale_alpha_manual(values = c(1 , 0.5)) + coord_flip() +
  scale_x_discrete(label = as_labeller(Genus_species_label_lookup)) + theme_bw() +
  theme(legend.position = "bottom", axis.text.x = element_text(size = 16), axis.text.y = element_text(size = 10, face = "plain"), axis.ticks.y = element_blank(), 
        panel.border = element_rect(linetype = "solid", fill = NA, linewidth = 1), axis.title.y = element_blank()) + 
  geom_hline(yintercept = 0) + ylab("log2FoldChange") + ggtitle("U.S.")

#Final plot:
ggsave("SupplementaryFigure5AC_ANCOM_v9.pdf", grid.arrange(plot_comp_us), width = 15, height = 10, units = "in", dpi = 300)
```

```
#--------------------------------------------------------------------------------------------------------------
```

#Supplementary Figure 5BD

```
#SUPPLEMENTARY FIGURE 5B & 5D
#--------------------------------------------------------------------------------------------------------------
###RUN ANCOM US ART vs UNTREATED###
dataset<-ps_gg_fp_f_prevalence_filter_2019_05_26
metadata<-as.data.frame(sample_data(ps_gg_fp_f_prevalence_filter_2019_05_26))
metadata<-metadata[metadata$hiv_phenotype %in% c("2_suppressed", "4_unsuppressed"), , drop=F]
metadata<-as.data.frame(as.matrix(metadata[metadata$sexual_orientation != "MSM" | is.na(metadata$sexual_orientation), , drop=F]))
metadata_boston<-metadata[metadata$sample_cohort == "boston", , drop=F]
sample_data(dataset)<-metadata_boston
```

```
## Found more than one class "phylo" in cache; using the first, from namespace 'phyloseq'
```

```
## Also defined by 'tidytree'
```

```
## Found more than one class "phylo" in cache; using the first, from namespace 'phyloseq'
```

```
## Also defined by 'tidytree'
```

```
## Found more than one class "phylo" in cache; using the first, from namespace 'phyloseq'
```

```
## Also defined by 'tidytree'
```

```
## Found more than one class "phylo" in cache; using the first, from namespace 'phyloseq'
```

```
## Also defined by 'tidytree'
```

```
## Found more than one class "phylo" in cache; using the first, from namespace 'phyloseq'
```

```
## Also defined by 'tidytree'
```

```
## Found more than one class "phylo" in cache; using the first, from namespace 'phyloseq'
```

```
## Also defined by 'tidytree'
```

```
## Found more than one class "phylo" in cache; using the first, from namespace 'phyloseq'
```

```
## Also defined by 'tidytree'
```

```
ps.taxa.sub <- phyloseq::prune_taxa(taxa_sums(dataset) > 0, dataset)
```

```
## Found more than one class "phylo" in cache; using the first, from namespace 'phyloseq'
## Also defined by 'tidytree'
```

```
out <- ANCOMBC::ancombc(data = ps.taxa.sub, formula = "hiv_phenotype", tax_level = NULL,
                        p_adj_method = "BH", prv_cut = 0.05, lib_cut = 1000, 
                        group = "hiv_phenotype", struc_zero = TRUE, neg_lb = FALSE, tol = 1e-5, 
                        max_iter = 100, conserve = FALSE, alpha = 0.05, global = FALSE, n_cl = 6)
```

```
## 'ancombc' is deprecated 
## Use 'ancombc2' instead
```

```
## Found more than one class "phylo" in cache; using the first, from namespace 'phyloseq'
```

```
## Also defined by 'tidytree'
```

```
## Found more than one class "phylo" in cache; using the first, from namespace 'phyloseq'
```

```
## Also defined by 'tidytree'
```

```
## `tax_level` is not speficified 
## No agglomeration will be performed
## Otherwise, please speficy `tax_level` by one of the following: 
## Kingdom, Phylum, Class, Order, Family, Genus, Species
```

```
## Found more than one class "phylo" in cache; using the first, from namespace 'phyloseq'
```

```
## Also defined by 'tidytree'
```

```
## Found more than one class "phylo" in cache; using the first, from namespace 'phyloseq'
```

```
## Also defined by 'tidytree'
```

```
## Found more than one class "phylo" in cache; using the first, from namespace 'phyloseq'
```

```
## Also defined by 'tidytree'
```

```
## Found more than one class "phylo" in cache; using the first, from namespace 'phyloseq'
```

```
## Also defined by 'tidytree'
```

```
## Warning: The group variable has < 3 categories 
## The multi-group comparisons (global/pairwise/dunnet/trend) will be deactivated
```

```
## Found more than one class "phylo" in cache; using the first, from namespace 'phyloseq'
## Also defined by 'tidytree'
```

```
## Found more than one class "phylo" in cache; using the first, from namespace 'phyloseq'
```

```
## Also defined by 'tidytree'
```

```
res <- out$res
res_rn <- purrr::imap(res, function(x, y) dplyr::rename(x, !!y := hiv_phenotype4_unsuppressed))
res_df <- purrr::reduce(res_rn, dplyr::left_join, by = "taxon")
res_df <- dplyr::select(res_df, !starts_with("(Int"))
res_df_taxa <- dplyr::left_join(res_df, tibble::rownames_to_column(as.data.frame(phyloseq::tax_table(ps.taxa.sub))), by = c("taxon" = "rowname"))
res_df_taxa[["index_num"]] <- 1:nrow(res_df_taxa)
res_df_taxa[["cohort"]] <- "boston"
res_df_taxa[["method"]] <- "ancom"
res_df_taxa <- tidyr::unite(res_df_taxa, col =  "Genus_Species", Genus, Species, index_num, remove = FALSE)
alpha = 0.05
taxa_sig <- dplyr::filter(res_df_taxa, q_val < 0.05)
```

```
## Warning: Using one column matrices in `filter()` was deprecated in dplyr 1.1.0.
## ℹ Please use one dimensional logical vectors instead.
## ℹ The deprecated feature was likely used in the dplyr package.
##   Please report the issue at <]8;;https://github.com/tidyverse/dplyr/issueshttps://github.com/tidyverse/dplyr/issues]8;;>.
```

```
taxa_sig$Genus_Species <- forcats::fct_reorder(taxa_sig$Genus_Species, taxa_sig$lfc, min)
taxa_sig$taxon_short <- stringr::str_sub(taxa_sig$taxon, 1, 4)
ps.taxa.rel.sig <- phyloseq::prune_taxa(taxa_sig[["taxon"]], ps.taxa.sub)
```

```
## Found more than one class "phylo" in cache; using the first, from namespace 'phyloseq'
## Also defined by 'tidytree'
```

```
# Only keep filtered samples 
ps.taxa.rel.sig <- phyloseq::prune_samples(rownames(phyloseq::otu_table(ps.taxa.sub)), ps.taxa.rel.sig)
sigtab_dataset_us <- taxa_sig
write.csv(sigtab_dataset_us, "ANCOM_US_ART_UNSUPPRESSED.csv")


###RUN ANCOM BOTSWANA ART vs UNTREATED###
dataset<-ps_gg_fp_f_prevalence_filter_2019_05_26
metadata<-as.data.frame(sample_data(ps_gg_fp_f_prevalence_filter_2019_05_26))
metadata<-metadata[metadata$hiv_phenotype %in% c("2_suppressed", "4_unsuppressed"), , drop=F]
metadata<-as.data.frame(as.matrix(metadata[metadata$sexual_orientation != "MSM" | is.na(metadata$sexual_orientation), , drop=F]))
metadata_botswana<-metadata[metadata$sample_cohort == "botswana", , drop=F]
sample_data(dataset)<-metadata_botswana
```

```
## Found more than one class "phylo" in cache; using the first, from namespace 'phyloseq'
## Also defined by 'tidytree'
```

```
## Found more than one class "phylo" in cache; using the first, from namespace 'phyloseq'
```

```
## Also defined by 'tidytree'
```

```
## Found more than one class "phylo" in cache; using the first, from namespace 'phyloseq'
```

```
## Also defined by 'tidytree'
```

```
## Found more than one class "phylo" in cache; using the first, from namespace 'phyloseq'
```

```
## Also defined by 'tidytree'
```

```
ps.taxa.sub <- phyloseq::prune_taxa(taxa_sums(dataset) > 0, dataset)
```

```
## Found more than one class "phylo" in cache; using the first, from namespace 'phyloseq'
## Also defined by 'tidytree'
```

```
out <- ANCOMBC::ancombc(data = ps.taxa.sub, formula = "hiv_phenotype", tax_level = NULL,
                        p_adj_method = "BH", prv_cut = 0.05, lib_cut = 1000, 
                        group = "hiv_phenotype", struc_zero = TRUE, neg_lb = FALSE, tol = 1e-5, 
                        max_iter = 100, conserve = FALSE, alpha = 0.05, global = FALSE, n_cl = 6)
```

```
## 'ancombc' is deprecated 
## Use 'ancombc2' instead
```

```
## Found more than one class "phylo" in cache; using the first, from namespace 'phyloseq'
```

```
## Also defined by 'tidytree'
```

```
## Found more than one class "phylo" in cache; using the first, from namespace 'phyloseq'
```

```
## Also defined by 'tidytree'
```

```
## `tax_level` is not speficified 
## No agglomeration will be performed
## Otherwise, please speficy `tax_level` by one of the following: 
## Kingdom, Phylum, Class, Order, Family, Genus, Species
```

```
## Found more than one class "phylo" in cache; using the first, from namespace 'phyloseq'
```

```
## Also defined by 'tidytree'
```

```
## Found more than one class "phylo" in cache; using the first, from namespace 'phyloseq'
```

```
## Also defined by 'tidytree'
```

```
## Found more than one class "phylo" in cache; using the first, from namespace 'phyloseq'
```

```
## Also defined by 'tidytree'
```

```
## Found more than one class "phylo" in cache; using the first, from namespace 'phyloseq'
```

```
## Also defined by 'tidytree'
```

```
## Warning: The group variable has < 3 categories 
## The multi-group comparisons (global/pairwise/dunnet/trend) will be deactivated
```

```
## Found more than one class "phylo" in cache; using the first, from namespace 'phyloseq'
## Also defined by 'tidytree'
```

```
## Found more than one class "phylo" in cache; using the first, from namespace 'phyloseq'
```

```
## Also defined by 'tidytree'
```

```
res <- out$res
res_rn <- purrr::imap(res, function(x, y) dplyr::rename(x, !!y := hiv_phenotype4_unsuppressed))
res_df <- purrr::reduce(res_rn, dplyr::left_join, by = "taxon")
res_df <- dplyr::select(res_df, !starts_with("(Int"))
res_df_taxa <- dplyr::left_join(res_df, tibble::rownames_to_column(as.data.frame(phyloseq::tax_table(ps.taxa.sub))), by = c("taxon" = "rowname"))
res_df_taxa[["index_num"]] <- 1:nrow(res_df_taxa)
res_df_taxa[["cohort"]] <- "botswana"
res_df_taxa[["method"]] <- "ancom"
res_df_taxa <- tidyr::unite(res_df_taxa, col =  "Genus_Species", Genus, Species, index_num, remove = FALSE)
alpha = 0.05
taxa_sig <- dplyr::filter(res_df_taxa, q_val < 0.05)
taxa_sig$Genus_Species <- forcats::fct_reorder(taxa_sig$Genus_Species, taxa_sig$lfc, min)
taxa_sig$taxon_short <- stringr::str_sub(taxa_sig$taxon, 1, 4)
ps.taxa.rel.sig <- phyloseq::prune_taxa(taxa_sig[["taxon"]], ps.taxa.sub)
```

```
## Found more than one class "phylo" in cache; using the first, from namespace 'phyloseq'
## Also defined by 'tidytree'
```

```
# Only keep filtered samples 
ps.taxa.rel.sig <- phyloseq::prune_samples(rownames(phyloseq::otu_table(ps.taxa.sub)), ps.taxa.rel.sig)
sigtab_dataset_botswana <- taxa_sig
write.csv(sigtab_dataset_botswana, "ANCOM_BOTSWANA_ART_UNSUPPRESSED.csv")

#Merge results from both sample cohorts:
sigtab_dataset<-rbind(sigtab_dataset_us, sigtab_dataset_botswana)
sigtab_dataset[["index_num_all"]] <- 1:nrow(sigtab_dataset)
sigtab_dataset <- tidyr::unite(sigtab_dataset, col =  "Genus_Species_all", Genus, Species, index_num_all, sep = "_", remove = FALSE)
sigtab_dataset <- tidyr::unite(sigtab_dataset, col =  "Genus_Species_all_for_color", Genus, Species, sep = " ", remove = FALSE)

# Remove extra brackets around taxonomic name for labels
sigtab_dataset$Genus_label <- stringr::str_replace(sigtab_dataset$Genus, "^\\[([^\\]]+)\\]", "\\1")
sigtab_dataset$Species_label <- stringr::str_replace(sigtab_dataset$Species, "^\\[([^\\]]+)\\]", "\\1")
sigtab_dataset %>% dplyr::mutate(Genus_species_label =
                                   dplyr::case_when(is.na(stringr::str_extract(Genus_label, "\\[")) & is.na(stringr::str_extract(Species_label, "\\[")) ~ paste(Genus_label,Species_label),
                                                    TRUE ~ paste(Genus_label,"sp"))) -> sigtab_dataset
sigtab_dataset$Genus_species_label <- stringr::str_replace(sigtab_dataset$Genus_species_label, "\\[([^\\]]+)\\]", "")
sigtab_dataset$Genus_species_label <- stringr::str_replace(sigtab_dataset$Genus_species_label, "\\ +", " ")
sigtab_dataset <- dplyr::arrange(sigtab_dataset, cohort, lfc)
sigtab_dataset$Genus_Species_all<-factor(sigtab_dataset$Genus_Species_all, levels = c(sigtab_dataset$Genus_Species_all))

# Make lookup table for Genus_species_label
Genus_species_label_lookup <- dplyr::distinct(sigtab_dataset, Genus_Species_all, .keep_all = TRUE)$Genus_species_label
names(Genus_species_label_lookup) <- dplyr::distinct(sigtab_dataset, Genus_Species_all, .keep_all = TRUE)$Genus_Species_all

#Load color dictionary and construct figure:
dictionary_unique <- read.csv("COLOR_DICTIONARY3.csv", sep=";")
sigtab_dataset$Genus_species_label <- factor(sigtab_dataset$Genus_species_label, levels = unique(sigtab_dataset$Genus_species_label))
dictionary_plot <- dictionary_unique[dictionary_unique$Genus_species_label%in%sigtab_dataset$Genus_species_label,,drop=F]
rownames(dictionary_plot)<-dictionary_plot$Genus_species_label
dictionary_plot<-dictionary_plot[as.character(unique(sigtab_dataset$Genus_species_label)),,drop=F]
sigtab_dataset$cohort <- forcats::fct_relevel(as.factor(sigtab_dataset$cohort), "boston", "botswana")
header_namer <- as_labeller(c(`boston` = "U.S.\nHIV+ ART-treated     HIV+ Untreated", `botswana` = "Botswana\nHIV+ ART-treated    HIV+ Untreated"))

ggsave("SupplementaryFigure5BD_ANCOM_v3.pdf", 
       ggplot(data = sigtab_dataset, aes(x = Genus_Species_all, y = lfc)) +
         geom_bar(stat = "identity", aes(fill = Genus_species_label)) +
         scale_fill_manual(values = as.character(dictionary_plot$Color)) +
         coord_flip() + scale_x_discrete(label = as_labeller(Genus_species_label_lookup)) + theme_bw() +
         theme(legend.position = "bottom", axis.text.x = element_text(size = 16), axis.text.y = element_text(size = 6, face = "plain"), axis.ticks.y = element_blank(), 
               panel.border = element_rect(linetype = "solid", fill = NA, linewidth = 1), axis.title.y = element_blank(),
               strip.background = element_rect(colour = "white", fill = "white"), strip.text = element_text(colour = "black", face = "bold", size = rel(1.2))) + 
         geom_hline(yintercept = 0) + ylab("log2FoldChange") +
         facet_wrap(vars(cohort), scales = "fixed", ncol = 2, labeller = labeller(cohort = header_namer)), width = 15, height = 20, units = "in", dpi = 300)

#--------------------------------------------------------------------------------------------------------------
```
