# Supplementary material for: HIV-associated gut microbial alterations are dependent on host and geographic context
Source: Nat Commun. 2024 Feb 5;15:1055. doi: 10.1038/s41467-023-44566-4 (PMC10844288; doi:10.1038/s41467-023-44566-4)
Supplement: Supplementary file 16 — SupplementaryFigure6_Rocafort-Gootenberg_2023_03_01 [file 41467_2023_44566_MOESM16_ESM.html]

Rocafort-Gootenberg\_SupplementaryFigure6


# Rocafort-Gootenberg\_SupplementaryFigure6

#Load needed R packages

```
library("phyloseq")
library("ggplot2")
library("gridExtra")
library("knitr") 
library("reshape")
library("DESeq2")
```

```
## Loading required package: S4Vectors
```

```
## Loading required package: stats4
```

```
## Loading required package: BiocGenerics
```

```
## 
## Attaching package: 'BiocGenerics'
```

```
## The following object is masked from 'package:gridExtra':
## 
##     combine
```

```
## The following objects are masked from 'package:stats':
## 
##     IQR, mad, sd, var, xtabs
```

```
## The following objects are masked from 'package:base':
## 
##     anyDuplicated, aperm, append, as.data.frame, basename, cbind,
##     colnames, dirname, do.call, duplicated, eval, evalq, Filter, Find,
##     get, grep, grepl, intersect, is.unsorted, lapply, Map, mapply,
##     match, mget, order, paste, pmax, pmax.int, pmin, pmin.int,
##     Position, rank, rbind, Reduce, rownames, sapply, setdiff, sort,
##     table, tapply, union, unique, unsplit, which.max, which.min
```

```
## 
## Attaching package: 'S4Vectors'
```

```
## The following objects are masked from 'package:reshape':
## 
##     expand, rename
```

```
## The following objects are masked from 'package:base':
## 
##     expand.grid, I, unname
```

```
## Loading required package: IRanges
```

```
## 
## Attaching package: 'IRanges'
```

```
## The following object is masked from 'package:phyloseq':
## 
##     distance
```

```
## Loading required package: GenomicRanges
```

```
## Loading required package: GenomeInfoDb
```

```
## Loading required package: SummarizedExperiment
```

```
## Loading required package: MatrixGenerics
```

```
## Loading required package: matrixStats
```

```
## 
## Attaching package: 'MatrixGenerics'
```

```
## The following objects are masked from 'package:matrixStats':
## 
##     colAlls, colAnyNAs, colAnys, colAvgsPerRowSet, colCollapse,
##     colCounts, colCummaxs, colCummins, colCumprods, colCumsums,
##     colDiffs, colIQRDiffs, colIQRs, colLogSumExps, colMadDiffs,
##     colMads, colMaxs, colMeans2, colMedians, colMins, colOrderStats,
##     colProds, colQuantiles, colRanges, colRanks, colSdDiffs, colSds,
##     colSums2, colTabulates, colVarDiffs, colVars, colWeightedMads,
##     colWeightedMeans, colWeightedMedians, colWeightedSds,
##     colWeightedVars, rowAlls, rowAnyNAs, rowAnys, rowAvgsPerColSet,
##     rowCollapse, rowCounts, rowCummaxs, rowCummins, rowCumprods,
##     rowCumsums, rowDiffs, rowIQRDiffs, rowIQRs, rowLogSumExps,
##     rowMadDiffs, rowMads, rowMaxs, rowMeans2, rowMedians, rowMins,
##     rowOrderStats, rowProds, rowQuantiles, rowRanges, rowRanks,
##     rowSdDiffs, rowSds, rowSums2, rowTabulates, rowVarDiffs, rowVars,
##     rowWeightedMads, rowWeightedMeans, rowWeightedMedians,
##     rowWeightedSds, rowWeightedVars
```

```
## Loading required package: Biobase
```

```
## Welcome to Bioconductor
## 
##     Vignettes contain introductory material; view with
##     'browseVignettes()'. To cite Bioconductor, see
##     'citation("Biobase")', and for packages 'citation("pkgname")'.
```

```
## 
## Attaching package: 'Biobase'
```

```
## The following object is masked from 'package:MatrixGenerics':
## 
##     rowMedians
```

```
## The following objects are masked from 'package:matrixStats':
## 
##     anyMissing, rowMedians
```

```
## The following object is masked from 'package:phyloseq':
## 
##     sampleNames
```

```
library("magrittr")
```

```
## 
## Attaching package: 'magrittr'
```

```
## The following object is masked from 'package:GenomicRanges':
## 
##     subtract
```

#Load original phyloseq oject output from DADA2 pipeline

```
ps_gg_fp_f_prevalence_filter_2019_05_26<-readRDS("ps_gg_fp_f_prevalence_filter_2019_05_26")
#Fix randomness
set.seed(1)
```

#Supplementary Figure 6A

```
#SUPPLEMENTARY FIGURE 6 A - GENUS
#--------------------------------------------------------------------------------------------------------------
###RUN ANCOM NEG vs ART at Genus###
#Agglomerate phyloseq object at Genus level
ps_gg_fp_f_prevalence_filter_2019_05_26_genus <- tax_glom(ps_gg_fp_f_prevalence_filter_2019_05_26, "Genus")
#US 
dataset<-ps_gg_fp_f_prevalence_filter_2019_05_26_genus
metadata<-as.data.frame(sample_data(ps_gg_fp_f_prevalence_filter_2019_05_26_genus))
metadata<-metadata[metadata$hiv_phenotype %in% c("1_hiv_negative", "2_suppressed"), , drop=F]
metadata<-as.data.frame(as.matrix(metadata[metadata$sexual_orientation != "MSM" | is.na(metadata$sexual_orientation), , drop=F]))
metadata_boston<-metadata[metadata$sample_cohort == "boston", , drop=F]
sample_data(dataset)<-metadata_boston
ps.taxa.sub <- phyloseq::prune_taxa(taxa_sums(dataset) > 0, dataset)
out <- ANCOMBC::ancombc(data = ps.taxa.sub, formula = "hiv_phenotype", tax_level = NULL,
              p_adj_method = "BH", prv_cut = 0.05, lib_cut = 1000, 
              group = "hiv_phenotype", struc_zero = TRUE, neg_lb = FALSE, tol = 1e-5, 
              max_iter = 100, conserve = FALSE, alpha = 0.05, global = FALSE, n_cl = 6)
```

```
## 'ancombc' is deprecated 
## Use 'ancombc2' instead
```

```
## Found more than one class "phylo" in cache; using the first, from namespace 'phyloseq'
```

```
## Also defined by 'tidytree'
```

```
## Found more than one class "phylo" in cache; using the first, from namespace 'phyloseq'
```

```
## Also defined by 'tidytree'
```

```
## `tax_level` is not speficified 
## No agglomeration will be performed
## Otherwise, please speficy `tax_level` by one of the following: 
## Kingdom, Phylum, Class, Order, Family, Genus, Species
```

```
## Found more than one class "phylo" in cache; using the first, from namespace 'phyloseq'
```

```
## Also defined by 'tidytree'
```

```
## Found more than one class "phylo" in cache; using the first, from namespace 'phyloseq'
```

```
## Also defined by 'tidytree'
```

```
## Found more than one class "phylo" in cache; using the first, from namespace 'phyloseq'
```

```
## Also defined by 'tidytree'
```

```
## Found more than one class "phylo" in cache; using the first, from namespace 'phyloseq'
```

```
## Also defined by 'tidytree'
```

```
## Warning: The group variable has < 3 categories 
## The multi-group comparisons (global/pairwise/dunnet/trend) will be deactivated
```

```
## Found more than one class "phylo" in cache; using the first, from namespace 'phyloseq'
## Also defined by 'tidytree'
```

```
## Found more than one class "phylo" in cache; using the first, from namespace 'phyloseq'
```

```
## Also defined by 'tidytree'
```

```
res <- out$res
res_rn <- purrr::imap(res, function(x, y) dplyr::rename(x, !!y := hiv_phenotype2_suppressed))
res_df <- purrr::reduce(res_rn, dplyr::left_join, by = "taxon")
res_df <- dplyr::select(res_df, !starts_with("(Int"))
res_df_taxa <- dplyr::left_join(res_df, tibble::rownames_to_column(as.data.frame(phyloseq::tax_table(ps.taxa.sub))), by = c("taxon" = "rowname"))
res_df_taxa[["index_num"]] <- 1:nrow(res_df_taxa)
res_df_taxa[["cohort"]] <- "boston"
res_df_taxa[["method"]] <- "ancom"
res_df_taxa <- tidyr::unite(res_df_taxa, col =  "Genus_Species", Genus, Species, index_num, remove = FALSE)
alpha = 0.05
taxa_sig <- dplyr::filter(res_df_taxa, q_val < 0.05)
```

```
## Warning: Using one column matrices in `filter()` was deprecated in dplyr 1.1.0.
## ℹ Please use one dimensional logical vectors instead.
## ℹ The deprecated feature was likely used in the dplyr package.
##   Please report the issue at <]8;;https://github.com/tidyverse/dplyr/issueshttps://github.com/tidyverse/dplyr/issues]8;;>.
```

```
taxa_sig$Genus_Species <- forcats::fct_reorder(taxa_sig$Genus_Species, taxa_sig$lfc, min)
taxa_sig$taxon_short <- stringr::str_sub(taxa_sig$taxon, 1, 4)
ps.taxa.rel.sig <- phyloseq::prune_taxa(taxa_sig[["taxon"]], ps.taxa.sub)
```

```
## Found more than one class "phylo" in cache; using the first, from namespace 'phyloseq'
## Also defined by 'tidytree'
```

```
# Only keep filtered samples 
ps.taxa.rel.sig <- phyloseq::prune_samples(rownames(phyloseq::otu_table(ps.taxa.sub)), ps.taxa.rel.sig)
sigtab_dataset_us <- taxa_sig
write.csv(sigtab_dataset_us, "ANCOM_US_Genus_NEG_ART_Filtered.csv")


#BOTSWANA
dataset<-ps_gg_fp_f_prevalence_filter_2019_05_26_genus
metadata<-as.data.frame(sample_data(ps_gg_fp_f_prevalence_filter_2019_05_26_genus))
metadata<-metadata[metadata$hiv_phenotype %in% c("1_hiv_negative", "2_suppressed"), , drop=F]
metadata<-as.data.frame(as.matrix(metadata[metadata$sexual_orientation != "MSM" | is.na(metadata$sexual_orientation), , drop=F]))
metadata_botswana<-metadata[metadata$sample_cohort == "botswana", , drop=F]
sample_data(dataset)<-metadata_botswana
```

```
## Found more than one class "phylo" in cache; using the first, from namespace 'phyloseq'
## Also defined by 'tidytree'
```

```
## Found more than one class "phylo" in cache; using the first, from namespace 'phyloseq'
```

```
## Also defined by 'tidytree'
```

```
## Found more than one class "phylo" in cache; using the first, from namespace 'phyloseq'
```

```
## Also defined by 'tidytree'
```

```
## Found more than one class "phylo" in cache; using the first, from namespace 'phyloseq'
```

```
## Also defined by 'tidytree'
```

```
ps.taxa.sub <- phyloseq::prune_taxa(taxa_sums(dataset) > 0, dataset)
```

```
## Found more than one class "phylo" in cache; using the first, from namespace 'phyloseq'
## Also defined by 'tidytree'
```

```
out <- ANCOMBC::ancombc(data = ps.taxa.sub, formula = "hiv_phenotype", tax_level = NULL,
              p_adj_method = "BH", prv_cut = 0.05, lib_cut = 1000, 
              group = "hiv_phenotype", struc_zero = TRUE, neg_lb = FALSE, tol = 1e-5, 
              max_iter = 100, conserve = FALSE, alpha = 0.05, global = FALSE, n_cl = 6)
```

```
## 'ancombc' is deprecated 
## Use 'ancombc2' instead
```

```
## Found more than one class "phylo" in cache; using the first, from namespace 'phyloseq'
```

```
## Also defined by 'tidytree'
```

```
## Found more than one class "phylo" in cache; using the first, from namespace 'phyloseq'
```

```
## Also defined by 'tidytree'
```

```
## `tax_level` is not speficified 
## No agglomeration will be performed
## Otherwise, please speficy `tax_level` by one of the following: 
## Kingdom, Phylum, Class, Order, Family, Genus, Species
```

```
## Found more than one class "phylo" in cache; using the first, from namespace 'phyloseq'
```

```
## Also defined by 'tidytree'
```

```
## Found more than one class "phylo" in cache; using the first, from namespace 'phyloseq'
```

```
## Also defined by 'tidytree'
```

```
## Found more than one class "phylo" in cache; using the first, from namespace 'phyloseq'
```

```
## Also defined by 'tidytree'
```

```
## Found more than one class "phylo" in cache; using the first, from namespace 'phyloseq'
```

```
## Also defined by 'tidytree'
```

```
## Warning: The group variable has < 3 categories 
## The multi-group comparisons (global/pairwise/dunnet/trend) will be deactivated
```

```
## Found more than one class "phylo" in cache; using the first, from namespace 'phyloseq'
## Also defined by 'tidytree'
```

```
## Found more than one class "phylo" in cache; using the first, from namespace 'phyloseq'
```

```
## Also defined by 'tidytree'
```

```
res <- out$res
res_rn <- purrr::imap(res, function(x, y) dplyr::rename(x, !!y := hiv_phenotype2_suppressed))
res_df <- purrr::reduce(res_rn, dplyr::left_join, by = "taxon")
res_df <- dplyr::select(res_df, !starts_with("(Int"))
res_df_taxa <- dplyr::left_join(res_df, tibble::rownames_to_column(as.data.frame(phyloseq::tax_table(ps.taxa.sub))), by = c("taxon" = "rowname"))
res_df_taxa[["index_num"]] <- 1:nrow(res_df_taxa)
res_df_taxa[["cohort"]] <- "botswana"
res_df_taxa[["method"]] <- "ancom"
res_df_taxa <- tidyr::unite(res_df_taxa, col =  "Genus_Species", Genus, Species, index_num, remove = FALSE)
alpha = 0.05
taxa_sig <- dplyr::filter(res_df_taxa, q_val < 0.05)
taxa_sig$Genus_Species <- forcats::fct_reorder(taxa_sig$Genus_Species, taxa_sig$lfc, min)
taxa_sig$taxon_short <- stringr::str_sub(taxa_sig$taxon, 1, 4)
ps.taxa.rel.sig <- phyloseq::prune_taxa(taxa_sig[["taxon"]], ps.taxa.sub)
```

```
## Found more than one class "phylo" in cache; using the first, from namespace 'phyloseq'
## Also defined by 'tidytree'
```

```
# Only keep filtered samples 
ps.taxa.rel.sig <- phyloseq::prune_samples(rownames(phyloseq::otu_table(ps.taxa.sub)), ps.taxa.rel.sig)
sigtab_dataset_botswana <- taxa_sig
write.csv(sigtab_dataset_botswana, "ANCOM_BOTS_Genus_NEG_ART_Filtered.csv")


#UGANDA
dataset<-ps_gg_fp_f_prevalence_filter_2019_05_26_genus
metadata<-as.data.frame(sample_data(ps_gg_fp_f_prevalence_filter_2019_05_26_genus))
metadata<-metadata[metadata$hiv_phenotype %in% c("1_hiv_negative", "2_suppressed"), , drop=F]
metadata<-as.data.frame(as.matrix(metadata[metadata$sexual_orientation != "MSM" | is.na(metadata$sexual_orientation), , drop=F]))
metadata_uganda<-metadata[metadata$sample_cohort == "uganda_2", , drop=F]
sample_data(dataset)<-metadata_uganda
```

```
## Found more than one class "phylo" in cache; using the first, from namespace 'phyloseq'
## Also defined by 'tidytree'
```

```
## Found more than one class "phylo" in cache; using the first, from namespace 'phyloseq'
```

```
## Also defined by 'tidytree'
```

```
## Found more than one class "phylo" in cache; using the first, from namespace 'phyloseq'
```

```
## Also defined by 'tidytree'
```

```
## Found more than one class "phylo" in cache; using the first, from namespace 'phyloseq'
```

```
## Also defined by 'tidytree'
```

```
ps.taxa.sub <- phyloseq::prune_taxa(taxa_sums(dataset) > 0, dataset)
```

```
## Found more than one class "phylo" in cache; using the first, from namespace 'phyloseq'
## Also defined by 'tidytree'
```

```
out <- ANCOMBC::ancombc(data = ps.taxa.sub, formula = "hiv_phenotype", tax_level = NULL,
              p_adj_method = "BH", prv_cut = 0.05, lib_cut = 1000, 
              group = "hiv_phenotype", struc_zero = TRUE, neg_lb = FALSE, tol = 1e-5, 
              max_iter = 100, conserve = FALSE, alpha = 0.05, global = FALSE, n_cl = 6)
```

```
## 'ancombc' is deprecated 
## Use 'ancombc2' instead
```

```
## Found more than one class "phylo" in cache; using the first, from namespace 'phyloseq'
```

```
## Also defined by 'tidytree'
```

```
## Found more than one class "phylo" in cache; using the first, from namespace 'phyloseq'
```

```
## Also defined by 'tidytree'
```

```
## `tax_level` is not speficified 
## No agglomeration will be performed
## Otherwise, please speficy `tax_level` by one of the following: 
## Kingdom, Phylum, Class, Order, Family, Genus, Species
```

```
## Found more than one class "phylo" in cache; using the first, from namespace 'phyloseq'
```

```
## Also defined by 'tidytree'
```

```
## Found more than one class "phylo" in cache; using the first, from namespace 'phyloseq'
```

```
## Also defined by 'tidytree'
```

```
## Found more than one class "phylo" in cache; using the first, from namespace 'phyloseq'
```

```
## Also defined by 'tidytree'
```

```
## Found more than one class "phylo" in cache; using the first, from namespace 'phyloseq'
```

```
## Also defined by 'tidytree'
```

```
## Warning: The group variable has < 3 categories 
## The multi-group comparisons (global/pairwise/dunnet/trend) will be deactivated
```

```
## Found more than one class "phylo" in cache; using the first, from namespace 'phyloseq'
## Also defined by 'tidytree'
```

```
## Found more than one class "phylo" in cache; using the first, from namespace 'phyloseq'
```

```
## Also defined by 'tidytree'
```

```
res <- out$res
res_rn <- purrr::imap(res, function(x, y) dplyr::rename(x, !!y := hiv_phenotype2_suppressed))
res_df <- purrr::reduce(res_rn, dplyr::left_join, by = "taxon")
res_df <- dplyr::select(res_df, !starts_with("(Int"))
res_df_taxa <- dplyr::left_join(res_df, tibble::rownames_to_column(as.data.frame(phyloseq::tax_table(ps.taxa.sub))), by = c("taxon" = "rowname"))
res_df_taxa[["index_num"]] <- 1:nrow(res_df_taxa)
res_df_taxa[["cohort"]] <- "uganda_2"
res_df_taxa[["method"]] <- "ancom"
res_df_taxa <- tidyr::unite(res_df_taxa, col =  "Genus_Species", Genus, Species, index_num, remove = FALSE)
alpha = 0.05
taxa_sig <- dplyr::filter(res_df_taxa, q_val < 0.05)
taxa_sig$Genus_Species <- forcats::fct_reorder(taxa_sig$Genus_Species, taxa_sig$lfc, min)
taxa_sig$taxon_short <- stringr::str_sub(taxa_sig$taxon, 1, 4)
ps.taxa.rel.sig <- phyloseq::prune_taxa(taxa_sig[["taxon"]], ps.taxa.sub)
```

```
## Found more than one class "phylo" in cache; using the first, from namespace 'phyloseq'
## Also defined by 'tidytree'
```

```
# Only keep filtered samples 
ps.taxa.rel.sig <- phyloseq::prune_samples(rownames(phyloseq::otu_table(ps.taxa.sub)), ps.taxa.rel.sig)
sigtab_dataset_uganda <- taxa_sig
write.csv(sigtab_dataset_uganda, "ANCOM_UGANDA_Genus_NEG_ART_Filtered.csv")

#Merge results from all three sample cohorts:
sigtab_dataset<-rbind(sigtab_dataset_us, sigtab_dataset_botswana, sigtab_dataset_uganda)
all <- sigtab_dataset[, colnames(sigtab_dataset) %in% c("lfc", "Genus", "cohort"), drop=F]
all <- dplyr::arrange(all, lfc)
# all <- all[order(all$lfc),,drop=F]
all$Genus <- factor(all$Genus, levels = unique(all$Genus))
all_melt <- melt(all)
```

```
## Using Genus, cohort as id variables
```

```
all_melt$cohort <- factor(all_melt$cohort, levels = c("boston", "botswana", "uganda_2"))

ggsave("SupplementaryFigure6A_ANCOM_v4.pdf", ggplot(data = all_melt, aes(x = Genus, y = cohort)) + geom_tile(aes(fill = value)) +
         coord_flip() + theme_bw() + scale_y_discrete(position = "right") +
         scale_fill_gradient2(low = "brown", mid = "white", high = "darkblue") +
         theme(axis.text.y = element_text(size = 10), axis.ticks = element_blank(), axis.title = element_blank(),
               panel.grid = element_line(linewidth = 0.1), panel.background = element_rect(fill = "white")) + 
         geom_hline(yintercept = c(1.5, 2.5), size = 0.2, color = "grey90")+ ggtitle("GENUS"), width = 10, height = 15, units = "in", dpi = 300)
```

```
## Warning: Using `size` aesthetic for lines was deprecated in ggplot2 3.4.0.
## ℹ Please use `linewidth` instead.
```

```
#--------------------------------------------------------------------------------------------------------------
```

#Supplementary Figure 6B

```
#SUPPLEMENTARY FIGURE 6 B - SPECIES
#--------------------------------------------------------------------------------------------------------------
###RUN ANCOM NEG vs ART at Species###
#Agglomerate phyloseq object at Species level
ps_gg_fp_f_prevalence_filter_2019_05_26_species <- tax_glom(ps_gg_fp_f_prevalence_filter_2019_05_26, "Species")
```

```
## Found more than one class "phylo" in cache; using the first, from namespace 'phyloseq'
```

```
## Also defined by 'tidytree'
```

```
## Found more than one class "phylo" in cache; using the first, from namespace 'phyloseq'
```

```
## Also defined by 'tidytree'
```

```
## Found more than one class "phylo" in cache; using the first, from namespace 'phyloseq'
```

```
## Also defined by 'tidytree'
```

```
## Found more than one class "phylo" in cache; using the first, from namespace 'phyloseq'
```

```
## Also defined by 'tidytree'
```

```
## Found more than one class "phylo" in cache; using the first, from namespace 'phyloseq'
```

```
## Also defined by 'tidytree'
```

```
## Found more than one class "phylo" in cache; using the first, from namespace 'phyloseq'
```

```
## Also defined by 'tidytree'
```

```
## Found more than one class "phylo" in cache; using the first, from namespace 'phyloseq'
```

```
## Also defined by 'tidytree'
```

```
## Found more than one class "phylo" in cache; using the first, from namespace 'phyloseq'
```

```
## Also defined by 'tidytree'
```

```
## Found more than one class "phylo" in cache; using the first, from namespace 'phyloseq'
```

```
## Also defined by 'tidytree'
```

```
## Found more than one class "phylo" in cache; using the first, from namespace 'phyloseq'
```

```
## Also defined by 'tidytree'
```

```
## Found more than one class "phylo" in cache; using the first, from namespace 'phyloseq'
```

```
## Also defined by 'tidytree'
```

```
## Found more than one class "phylo" in cache; using the first, from namespace 'phyloseq'
```

```
## Also defined by 'tidytree'
```

```
## Found more than one class "phylo" in cache; using the first, from namespace 'phyloseq'
```

```
## Also defined by 'tidytree'
```

```
## Found more than one class "phylo" in cache; using the first, from namespace 'phyloseq'
```

```
## Also defined by 'tidytree'
```

```
## Found more than one class "phylo" in cache; using the first, from namespace 'phyloseq'
```

```
## Also defined by 'tidytree'
```

```
## Found more than one class "phylo" in cache; using the first, from namespace 'phyloseq'
```

```
## Also defined by 'tidytree'
```

```
## Found more than one class "phylo" in cache; using the first, from namespace 'phyloseq'
```

```
## Also defined by 'tidytree'
```

```
## Found more than one class "phylo" in cache; using the first, from namespace 'phyloseq'
```

```
## Also defined by 'tidytree'
```

```
## Found more than one class "phylo" in cache; using the first, from namespace 'phyloseq'
```

```
## Also defined by 'tidytree'
```

```
## Found more than one class "phylo" in cache; using the first, from namespace 'phyloseq'
```

```
## Also defined by 'tidytree'
```

```
## Found more than one class "phylo" in cache; using the first, from namespace 'phyloseq'
```

```
## Also defined by 'tidytree'
```

```
## Found more than one class "phylo" in cache; using the first, from namespace 'phyloseq'
```

```
## Also defined by 'tidytree'
```

```
## Found more than one class "phylo" in cache; using the first, from namespace 'phyloseq'
```

```
## Also defined by 'tidytree'
```

```
## Found more than one class "phylo" in cache; using the first, from namespace 'phyloseq'
```

```
## Also defined by 'tidytree'
```

```
## Found more than one class "phylo" in cache; using the first, from namespace 'phyloseq'
```

```
## Also defined by 'tidytree'
```

```
## Found more than one class "phylo" in cache; using the first, from namespace 'phyloseq'
```

```
## Also defined by 'tidytree'
```

```
## Found more than one class "phylo" in cache; using the first, from namespace 'phyloseq'
```

```
## Also defined by 'tidytree'
```

```
## Found more than one class "phylo" in cache; using the first, from namespace 'phyloseq'
```

```
## Also defined by 'tidytree'
```

```
## Found more than one class "phylo" in cache; using the first, from namespace 'phyloseq'
```

```
## Also defined by 'tidytree'
```

```
## Found more than one class "phylo" in cache; using the first, from namespace 'phyloseq'
```

```
## Also defined by 'tidytree'
```

```
## Found more than one class "phylo" in cache; using the first, from namespace 'phyloseq'
```

```
## Also defined by 'tidytree'
```

```
## Found more than one class "phylo" in cache; using the first, from namespace 'phyloseq'
```

```
## Also defined by 'tidytree'
```

```
## Found more than one class "phylo" in cache; using the first, from namespace 'phyloseq'
```

```
## Also defined by 'tidytree'
```

```
## Found more than one class "phylo" in cache; using the first, from namespace 'phyloseq'
```

```
## Also defined by 'tidytree'
```

```
## Found more than one class "phylo" in cache; using the first, from namespace 'phyloseq'
```

```
## Also defined by 'tidytree'
```

```
## Found more than one class "phylo" in cache; using the first, from namespace 'phyloseq'
```

```
## Also defined by 'tidytree'
```

```
## Found more than one class "phylo" in cache; using the first, from namespace 'phyloseq'
```

```
## Also defined by 'tidytree'
```

```
## Found more than one class "phylo" in cache; using the first, from namespace 'phyloseq'
```

```
## Also defined by 'tidytree'
```

```
## Found more than one class "phylo" in cache; using the first, from namespace 'phyloseq'
```

```
## Also defined by 'tidytree'
```

```
## Found more than one class "phylo" in cache; using the first, from namespace 'phyloseq'
```

```
## Also defined by 'tidytree'
```

```
## Found more than one class "phylo" in cache; using the first, from namespace 'phyloseq'
```

```
## Also defined by 'tidytree'
```

```
## Found more than one class "phylo" in cache; using the first, from namespace 'phyloseq'
```

```
## Also defined by 'tidytree'
```

```
## Found more than one class "phylo" in cache; using the first, from namespace 'phyloseq'
```

```
## Also defined by 'tidytree'
```

```
## Found more than one class "phylo" in cache; using the first, from namespace 'phyloseq'
```

```
## Also defined by 'tidytree'
```

```
## Found more than one class "phylo" in cache; using the first, from namespace 'phyloseq'
```

```
## Also defined by 'tidytree'
```

```
## Found more than one class "phylo" in cache; using the first, from namespace 'phyloseq'
```

```
## Also defined by 'tidytree'
```

```
## Found more than one class "phylo" in cache; using the first, from namespace 'phyloseq'
```

```
## Also defined by 'tidytree'
```

```
## Found more than one class "phylo" in cache; using the first, from namespace 'phyloseq'
```

```
## Also defined by 'tidytree'
```

```
## Found more than one class "phylo" in cache; using the first, from namespace 'phyloseq'
```

```
## Also defined by 'tidytree'
```

```
## Found more than one class "phylo" in cache; using the first, from namespace 'phyloseq'
```

```
## Also defined by 'tidytree'
```

```
## Found more than one class "phylo" in cache; using the first, from namespace 'phyloseq'
```

```
## Also defined by 'tidytree'
```

```
## Found more than one class "phylo" in cache; using the first, from namespace 'phyloseq'
```

```
## Also defined by 'tidytree'
```

```
## Found more than one class "phylo" in cache; using the first, from namespace 'phyloseq'
```

```
## Also defined by 'tidytree'
```

```
## Found more than one class "phylo" in cache; using the first, from namespace 'phyloseq'
```

```
## Also defined by 'tidytree'
```

```
## Found more than one class "phylo" in cache; using the first, from namespace 'phyloseq'
```

```
## Also defined by 'tidytree'
```

```
## Found more than one class "phylo" in cache; using the first, from namespace 'phyloseq'
```

```
## Also defined by 'tidytree'
```

```
## Found more than one class "phylo" in cache; using the first, from namespace 'phyloseq'
```

```
## Also defined by 'tidytree'
```

```
## Found more than one class "phylo" in cache; using the first, from namespace 'phyloseq'
```

```
## Also defined by 'tidytree'
```

```
## Found more than one class "phylo" in cache; using the first, from namespace 'phyloseq'
```

```
## Also defined by 'tidytree'
```

```
## Found more than one class "phylo" in cache; using the first, from namespace 'phyloseq'
```

```
## Also defined by 'tidytree'
```

```
## Found more than one class "phylo" in cache; using the first, from namespace 'phyloseq'
```

```
## Also defined by 'tidytree'
```

```
## Found more than one class "phylo" in cache; using the first, from namespace 'phyloseq'
```

```
## Also defined by 'tidytree'
```

```
## Found more than one class "phylo" in cache; using the first, from namespace 'phyloseq'
```

```
## Also defined by 'tidytree'
```

```
## Found more than one class "phylo" in cache; using the first, from namespace 'phyloseq'
```

```
## Also defined by 'tidytree'
```

```
## Found more than one class "phylo" in cache; using the first, from namespace 'phyloseq'
```

```
## Also defined by 'tidytree'
```

```
## Found more than one class "phylo" in cache; using the first, from namespace 'phyloseq'
```

```
## Also defined by 'tidytree'
```

```
## Found more than one class "phylo" in cache; using the first, from namespace 'phyloseq'
```

```
## Also defined by 'tidytree'
```

```
## Found more than one class "phylo" in cache; using the first, from namespace 'phyloseq'
```

```
## Also defined by 'tidytree'
```

```
## Found more than one class "phylo" in cache; using the first, from namespace 'phyloseq'
```

```
## Also defined by 'tidytree'
```

```
## Found more than one class "phylo" in cache; using the first, from namespace 'phyloseq'
```

```
## Also defined by 'tidytree'
```

```
## Found more than one class "phylo" in cache; using the first, from namespace 'phyloseq'
```

```
## Also defined by 'tidytree'
```

```
## Found more than one class "phylo" in cache; using the first, from namespace 'phyloseq'
```

```
## Also defined by 'tidytree'
```

```
## Found more than one class "phylo" in cache; using the first, from namespace 'phyloseq'
```

```
## Also defined by 'tidytree'
```

```
## Found more than one class "phylo" in cache; using the first, from namespace 'phyloseq'
```

```
## Also defined by 'tidytree'
```

```
## Found more than one class "phylo" in cache; using the first, from namespace 'phyloseq'
```

```
## Also defined by 'tidytree'
```

```
## Found more than one class "phylo" in cache; using the first, from namespace 'phyloseq'
```

```
## Also defined by 'tidytree'
```

```
## Found more than one class "phylo" in cache; using the first, from namespace 'phyloseq'
```

```
## Also defined by 'tidytree'
```

```
## Found more than one class "phylo" in cache; using the first, from namespace 'phyloseq'
```

```
## Also defined by 'tidytree'
```

```
## Found more than one class "phylo" in cache; using the first, from namespace 'phyloseq'
```

```
## Also defined by 'tidytree'
```

```
## Found more than one class "phylo" in cache; using the first, from namespace 'phyloseq'
```

```
## Also defined by 'tidytree'
```

```
## Found more than one class "phylo" in cache; using the first, from namespace 'phyloseq'
```

```
## Also defined by 'tidytree'
```

```
## Found more than one class "phylo" in cache; using the first, from namespace 'phyloseq'
```

```
## Also defined by 'tidytree'
```

```
## Found more than one class "phylo" in cache; using the first, from namespace 'phyloseq'
```

```
## Also defined by 'tidytree'
```

```
## Found more than one class "phylo" in cache; using the first, from namespace 'phyloseq'
```

```
## Also defined by 'tidytree'
```

```
## Found more than one class "phylo" in cache; using the first, from namespace 'phyloseq'
```

```
## Also defined by 'tidytree'
```

```
## Found more than one class "phylo" in cache; using the first, from namespace 'phyloseq'
```

```
## Also defined by 'tidytree'
```

```
## Found more than one class "phylo" in cache; using the first, from namespace 'phyloseq'
```

```
## Also defined by 'tidytree'
```

```
## Found more than one class "phylo" in cache; using the first, from namespace 'phyloseq'
```

```
## Also defined by 'tidytree'
```

```
## Found more than one class "phylo" in cache; using the first, from namespace 'phyloseq'
```

```
## Also defined by 'tidytree'
```

```
## Found more than one class "phylo" in cache; using the first, from namespace 'phyloseq'
```

```
## Also defined by 'tidytree'
```

```
## Found more than one class "phylo" in cache; using the first, from namespace 'phyloseq'
```

```
## Also defined by 'tidytree'
```

```
## Found more than one class "phylo" in cache; using the first, from namespace 'phyloseq'
```

```
## Also defined by 'tidytree'
```

```
## Found more than one class "phylo" in cache; using the first, from namespace 'phyloseq'
```

```
## Also defined by 'tidytree'
```

```
## Found more than one class "phylo" in cache; using the first, from namespace 'phyloseq'
```

```
## Also defined by 'tidytree'
```

```
## Found more than one class "phylo" in cache; using the first, from namespace 'phyloseq'
```

```
## Also defined by 'tidytree'
```

```
## Found more than one class "phylo" in cache; using the first, from namespace 'phyloseq'
```

```
## Also defined by 'tidytree'
```

```
## Found more than one class "phylo" in cache; using the first, from namespace 'phyloseq'
```

```
## Also defined by 'tidytree'
```

```
## Found more than one class "phylo" in cache; using the first, from namespace 'phyloseq'
```

```
## Also defined by 'tidytree'
```

```
## Found more than one class "phylo" in cache; using the first, from namespace 'phyloseq'
```

```
## Also defined by 'tidytree'
```

```
## Found more than one class "phylo" in cache; using the first, from namespace 'phyloseq'
```

```
## Also defined by 'tidytree'
```

```
## Found more than one class "phylo" in cache; using the first, from namespace 'phyloseq'
```

```
## Also defined by 'tidytree'
```

```
## Found more than one class "phylo" in cache; using the first, from namespace 'phyloseq'
```

```
## Also defined by 'tidytree'
```

```
## Found more than one class "phylo" in cache; using the first, from namespace 'phyloseq'
```

```
## Also defined by 'tidytree'
```

```
## Found more than one class "phylo" in cache; using the first, from namespace 'phyloseq'
```

```
## Also defined by 'tidytree'
```

```
## Found more than one class "phylo" in cache; using the first, from namespace 'phyloseq'
```

```
## Also defined by 'tidytree'
```

```
## Found more than one class "phylo" in cache; using the first, from namespace 'phyloseq'
```

```
## Also defined by 'tidytree'
```

```
## Found more than one class "phylo" in cache; using the first, from namespace 'phyloseq'
```

```
## Also defined by 'tidytree'
```

```
## Found more than one class "phylo" in cache; using the first, from namespace 'phyloseq'
```

```
## Also defined by 'tidytree'
```

```
## Found more than one class "phylo" in cache; using the first, from namespace 'phyloseq'
```

```
## Also defined by 'tidytree'
```

```
## Found more than one class "phylo" in cache; using the first, from namespace 'phyloseq'
```

```
## Also defined by 'tidytree'
```

```
## Found more than one class "phylo" in cache; using the first, from namespace 'phyloseq'
```

```
## Also defined by 'tidytree'
```

```
## Found more than one class "phylo" in cache; using the first, from namespace 'phyloseq'
```

```
## Also defined by 'tidytree'
```

```
## Found more than one class "phylo" in cache; using the first, from namespace 'phyloseq'
```

```
## Also defined by 'tidytree'
```

```
## Found more than one class "phylo" in cache; using the first, from namespace 'phyloseq'
```

```
## Also defined by 'tidytree'
```

```
## Found more than one class "phylo" in cache; using the first, from namespace 'phyloseq'
```

```
## Also defined by 'tidytree'
```

```
## Found more than one class "phylo" in cache; using the first, from namespace 'phyloseq'
```

```
## Also defined by 'tidytree'
```

```
## Found more than one class "phylo" in cache; using the first, from namespace 'phyloseq'
```

```
## Also defined by 'tidytree'
```

```
## Found more than one class "phylo" in cache; using the first, from namespace 'phyloseq'
```

```
## Also defined by 'tidytree'
```

```
## Found more than one class "phylo" in cache; using the first, from namespace 'phyloseq'
```

```
## Also defined by 'tidytree'
```

```
## Found more than one class "phylo" in cache; using the first, from namespace 'phyloseq'
```

```
## Also defined by 'tidytree'
```

```
## Found more than one class "phylo" in cache; using the first, from namespace 'phyloseq'
```

```
## Also defined by 'tidytree'
```

```
## Found more than one class "phylo" in cache; using the first, from namespace 'phyloseq'
```

```
## Also defined by 'tidytree'
```

```
## Found more than one class "phylo" in cache; using the first, from namespace 'phyloseq'
```

```
## Also defined by 'tidytree'
```

```
## Found more than one class "phylo" in cache; using the first, from namespace 'phyloseq'
```

```
## Also defined by 'tidytree'
```

```
## Found more than one class "phylo" in cache; using the first, from namespace 'phyloseq'
```

```
## Also defined by 'tidytree'
```

```
## Found more than one class "phylo" in cache; using the first, from namespace 'phyloseq'
```

```
## Also defined by 'tidytree'
```

```
## Found more than one class "phylo" in cache; using the first, from namespace 'phyloseq'
```

```
## Also defined by 'tidytree'
```

```
## Found more than one class "phylo" in cache; using the first, from namespace 'phyloseq'
```

```
## Also defined by 'tidytree'
```

```
## Found more than one class "phylo" in cache; using the first, from namespace 'phyloseq'
```

```
## Also defined by 'tidytree'
```

```
## Found more than one class "phylo" in cache; using the first, from namespace 'phyloseq'
```

```
## Also defined by 'tidytree'
```

```
## Found more than one class "phylo" in cache; using the first, from namespace 'phyloseq'
```

```
## Also defined by 'tidytree'
```

```
## Found more than one class "phylo" in cache; using the first, from namespace 'phyloseq'
```

```
## Also defined by 'tidytree'
```

```
## Found more than one class "phylo" in cache; using the first, from namespace 'phyloseq'
```

```
## Also defined by 'tidytree'
```

```
## Found more than one class "phylo" in cache; using the first, from namespace 'phyloseq'
```

```
## Also defined by 'tidytree'
```

```
## Found more than one class "phylo" in cache; using the first, from namespace 'phyloseq'
```

```
## Also defined by 'tidytree'
```

```
## Found more than one class "phylo" in cache; using the first, from namespace 'phyloseq'
```

```
## Also defined by 'tidytree'
```

```
## Found more than one class "phylo" in cache; using the first, from namespace 'phyloseq'
```

```
## Also defined by 'tidytree'
```

```
## Found more than one class "phylo" in cache; using the first, from namespace 'phyloseq'
```

```
## Also defined by 'tidytree'
```

```
## Found more than one class "phylo" in cache; using the first, from namespace 'phyloseq'
```

```
## Also defined by 'tidytree'
```

```
## Found more than one class "phylo" in cache; using the first, from namespace 'phyloseq'
```

```
## Also defined by 'tidytree'
```

```
## Found more than one class "phylo" in cache; using the first, from namespace 'phyloseq'
```

```
## Also defined by 'tidytree'
```

```
## Found more than one class "phylo" in cache; using the first, from namespace 'phyloseq'
```

```
## Also defined by 'tidytree'
```

```
## Found more than one class "phylo" in cache; using the first, from namespace 'phyloseq'
```

```
## Also defined by 'tidytree'
```

```
## Found more than one class "phylo" in cache; using the first, from namespace 'phyloseq'
```

```
## Also defined by 'tidytree'
```

```
## Found more than one class "phylo" in cache; using the first, from namespace 'phyloseq'
```

```
## Also defined by 'tidytree'
```

```
## Found more than one class "phylo" in cache; using the first, from namespace 'phyloseq'
```

```
## Also defined by 'tidytree'
```

```
## Found more than one class "phylo" in cache; using the first, from namespace 'phyloseq'
```

```
## Also defined by 'tidytree'
```

```
## Found more than one class "phylo" in cache; using the first, from namespace 'phyloseq'
```

```
## Also defined by 'tidytree'
```

```
## Found more than one class "phylo" in cache; using the first, from namespace 'phyloseq'
```

```
## Also defined by 'tidytree'
```

```
## Found more than one class "phylo" in cache; using the first, from namespace 'phyloseq'
```

```
## Also defined by 'tidytree'
```

```
## Found more than one class "phylo" in cache; using the first, from namespace 'phyloseq'
```

```
## Also defined by 'tidytree'
```

```
## Found more than one class "phylo" in cache; using the first, from namespace 'phyloseq'
```

```
## Also defined by 'tidytree'
```

```
## Found more than one class "phylo" in cache; using the first, from namespace 'phyloseq'
```

```
## Also defined by 'tidytree'
```

```
## Found more than one class "phylo" in cache; using the first, from namespace 'phyloseq'
```

```
## Also defined by 'tidytree'
```

```
## Found more than one class "phylo" in cache; using the first, from namespace 'phyloseq'
```

```
## Also defined by 'tidytree'
```

```
## Found more than one class "phylo" in cache; using the first, from namespace 'phyloseq'
```

```
## Also defined by 'tidytree'
```

```
## Found more than one class "phylo" in cache; using the first, from namespace 'phyloseq'
```

```
## Also defined by 'tidytree'
```

```
## Found more than one class "phylo" in cache; using the first, from namespace 'phyloseq'
```

```
## Also defined by 'tidytree'
```

```
## Found more than one class "phylo" in cache; using the first, from namespace 'phyloseq'
```

```
## Also defined by 'tidytree'
```

```
## Found more than one class "phylo" in cache; using the first, from namespace 'phyloseq'
```

```
## Also defined by 'tidytree'
```

```
## Found more than one class "phylo" in cache; using the first, from namespace 'phyloseq'
```

```
## Also defined by 'tidytree'
```

```
## Found more than one class "phylo" in cache; using the first, from namespace 'phyloseq'
```

```
## Also defined by 'tidytree'
```

```
## Found more than one class "phylo" in cache; using the first, from namespace 'phyloseq'
```

```
## Also defined by 'tidytree'
```

```
## Found more than one class "phylo" in cache; using the first, from namespace 'phyloseq'
```

```
## Also defined by 'tidytree'
```

```
## Found more than one class "phylo" in cache; using the first, from namespace 'phyloseq'
```

```
## Also defined by 'tidytree'
```

```
## Found more than one class "phylo" in cache; using the first, from namespace 'phyloseq'
```

```
## Also defined by 'tidytree'
```

```
## Found more than one class "phylo" in cache; using the first, from namespace 'phyloseq'
```

```
## Also defined by 'tidytree'
```

```
## Found more than one class "phylo" in cache; using the first, from namespace 'phyloseq'
```

```
## Also defined by 'tidytree'
```

```
## Found more than one class "phylo" in cache; using the first, from namespace 'phyloseq'
```

```
## Also defined by 'tidytree'
```

```
## Found more than one class "phylo" in cache; using the first, from namespace 'phyloseq'
```

```
## Also defined by 'tidytree'
```

```
## Found more than one class "phylo" in cache; using the first, from namespace 'phyloseq'
```

```
## Also defined by 'tidytree'
```

```
## Found more than one class "phylo" in cache; using the first, from namespace 'phyloseq'
```

```
## Also defined by 'tidytree'
```

```
## Found more than one class "phylo" in cache; using the first, from namespace 'phyloseq'
```

```
## Also defined by 'tidytree'
```

```
## Found more than one class "phylo" in cache; using the first, from namespace 'phyloseq'
```

```
## Also defined by 'tidytree'
```

```
## Found more than one class "phylo" in cache; using the first, from namespace 'phyloseq'
```

```
## Also defined by 'tidytree'
```

```
## Found more than one class "phylo" in cache; using the first, from namespace 'phyloseq'
```

```
## Also defined by 'tidytree'
```

```
## Found more than one class "phylo" in cache; using the first, from namespace 'phyloseq'
```

```
## Also defined by 'tidytree'
```

```
## Found more than one class "phylo" in cache; using the first, from namespace 'phyloseq'
```

```
## Also defined by 'tidytree'
```

```
## Found more than one class "phylo" in cache; using the first, from namespace 'phyloseq'
```

```
## Also defined by 'tidytree'
```

```
## Found more than one class "phylo" in cache; using the first, from namespace 'phyloseq'
```

```
## Also defined by 'tidytree'
```

```
## Found more than one class "phylo" in cache; using the first, from namespace 'phyloseq'
```

```
## Also defined by 'tidytree'
```

```
## Found more than one class "phylo" in cache; using the first, from namespace 'phyloseq'
```

```
## Also defined by 'tidytree'
```

```
## Found more than one class "phylo" in cache; using the first, from namespace 'phyloseq'
```

```
## Also defined by 'tidytree'
```

```
## Found more than one class "phylo" in cache; using the first, from namespace 'phyloseq'
```

```
## Also defined by 'tidytree'
```

```
## Found more than one class "phylo" in cache; using the first, from namespace 'phyloseq'
```

```
## Also defined by 'tidytree'
```

```
## Found more than one class "phylo" in cache; using the first, from namespace 'phyloseq'
```

```
## Also defined by 'tidytree'
```

```
## Found more than one class "phylo" in cache; using the first, from namespace 'phyloseq'
```

```
## Also defined by 'tidytree'
```

```
## Found more than one class "phylo" in cache; using the first, from namespace 'phyloseq'
```

```
## Also defined by 'tidytree'
```

```
## Found more than one class "phylo" in cache; using the first, from namespace 'phyloseq'
```

```
## Also defined by 'tidytree'
```

```
## Found more than one class "phylo" in cache; using the first, from namespace 'phyloseq'
```

```
## Also defined by 'tidytree'
```

```
## Found more than one class "phylo" in cache; using the first, from namespace 'phyloseq'
```

```
## Also defined by 'tidytree'
```

```
## Found more than one class "phylo" in cache; using the first, from namespace 'phyloseq'
```

```
## Also defined by 'tidytree'
```

```
## Found more than one class "phylo" in cache; using the first, from namespace 'phyloseq'
```

```
## Also defined by 'tidytree'
```

```
## Found more than one class "phylo" in cache; using the first, from namespace 'phyloseq'
```

```
## Also defined by 'tidytree'
```

```
## Found more than one class "phylo" in cache; using the first, from namespace 'phyloseq'
```

```
## Also defined by 'tidytree'
```

```
## Found more than one class "phylo" in cache; using the first, from namespace 'phyloseq'
```

```
## Also defined by 'tidytree'
```

```
## Found more than one class "phylo" in cache; using the first, from namespace 'phyloseq'
```

```
## Also defined by 'tidytree'
```

```
## Found more than one class "phylo" in cache; using the first, from namespace 'phyloseq'
```

```
## Also defined by 'tidytree'
```

```
## Found more than one class "phylo" in cache; using the first, from namespace 'phyloseq'
```

```
## Also defined by 'tidytree'
```

```
## Found more than one class "phylo" in cache; using the first, from namespace 'phyloseq'
```

```
## Also defined by 'tidytree'
```

```
## Found more than one class "phylo" in cache; using the first, from namespace 'phyloseq'
```

```
## Also defined by 'tidytree'
```

```
## Found more than one class "phylo" in cache; using the first, from namespace 'phyloseq'
```

```
## Also defined by 'tidytree'
```

```
## Found more than one class "phylo" in cache; using the first, from namespace 'phyloseq'
```

```
## Also defined by 'tidytree'
```

```
## Found more than one class "phylo" in cache; using the first, from namespace 'phyloseq'
```

```
## Also defined by 'tidytree'
```

```
## Found more than one class "phylo" in cache; using the first, from namespace 'phyloseq'
```

```
## Also defined by 'tidytree'
```

```
## Found more than one class "phylo" in cache; using the first, from namespace 'phyloseq'
```

```
## Also defined by 'tidytree'
```

```
## Found more than one class "phylo" in cache; using the first, from namespace 'phyloseq'
```

```
## Also defined by 'tidytree'
```

```
## Found more than one class "phylo" in cache; using the first, from namespace 'phyloseq'
```

```
## Also defined by 'tidytree'
```

```
## Found more than one class "phylo" in cache; using the first, from namespace 'phyloseq'
```

```
## Also defined by 'tidytree'
```

```
## Found more than one class "phylo" in cache; using the first, from namespace 'phyloseq'
```

```
## Also defined by 'tidytree'
```

```
## Found more than one class "phylo" in cache; using the first, from namespace 'phyloseq'
```

```
## Also defined by 'tidytree'
```

```
## Found more than one class "phylo" in cache; using the first, from namespace 'phyloseq'
```

```
## Also defined by 'tidytree'
```

```
## Found more than one class "phylo" in cache; using the first, from namespace 'phyloseq'
```

```
## Also defined by 'tidytree'
```

```
## Found more than one class "phylo" in cache; using the first, from namespace 'phyloseq'
```

```
## Also defined by 'tidytree'
```

```
## Found more than one class "phylo" in cache; using the first, from namespace 'phyloseq'
```

```
## Also defined by 'tidytree'
```

```
## Found more than one class "phylo" in cache; using the first, from namespace 'phyloseq'
```

```
## Also defined by 'tidytree'
```

```
## Found more than one class "phylo" in cache; using the first, from namespace 'phyloseq'
```

```
## Also defined by 'tidytree'
```

```
## Found more than one class "phylo" in cache; using the first, from namespace 'phyloseq'
```

```
## Also defined by 'tidytree'
```

```
## Found more than one class "phylo" in cache; using the first, from namespace 'phyloseq'
```

```
## Also defined by 'tidytree'
```

```
## Found more than one class "phylo" in cache; using the first, from namespace 'phyloseq'
```

```
## Also defined by 'tidytree'
```

```
## Found more than one class "phylo" in cache; using the first, from namespace 'phyloseq'
```

```
## Also defined by 'tidytree'
```

```
## Found more than one class "phylo" in cache; using the first, from namespace 'phyloseq'
```

```
## Also defined by 'tidytree'
```

```
## Found more than one class "phylo" in cache; using the first, from namespace 'phyloseq'
```

```
## Also defined by 'tidytree'
```

```
## Found more than one class "phylo" in cache; using the first, from namespace 'phyloseq'
```

```
## Also defined by 'tidytree'
```

```
## Found more than one class "phylo" in cache; using the first, from namespace 'phyloseq'
```

```
## Also defined by 'tidytree'
```

```
## Found more than one class "phylo" in cache; using the first, from namespace 'phyloseq'
```

```
## Also defined by 'tidytree'
```

```
## Found more than one class "phylo" in cache; using the first, from namespace 'phyloseq'
```

```
## Also defined by 'tidytree'
```

```
## Found more than one class "phylo" in cache; using the first, from namespace 'phyloseq'
```

```
## Also defined by 'tidytree'
```

```
## Found more than one class "phylo" in cache; using the first, from namespace 'phyloseq'
```

```
## Also defined by 'tidytree'
```

```
## Found more than one class "phylo" in cache; using the first, from namespace 'phyloseq'
```

```
## Also defined by 'tidytree'
```

```
## Found more than one class "phylo" in cache; using the first, from namespace 'phyloseq'
```

```
## Also defined by 'tidytree'
```

```
## Found more than one class "phylo" in cache; using the first, from namespace 'phyloseq'
```

```
## Also defined by 'tidytree'
```

```
## Found more than one class "phylo" in cache; using the first, from namespace 'phyloseq'
```

```
## Also defined by 'tidytree'
```

```
## Found more than one class "phylo" in cache; using the first, from namespace 'phyloseq'
```

```
## Also defined by 'tidytree'
```

```
## Found more than one class "phylo" in cache; using the first, from namespace 'phyloseq'
```

```
## Also defined by 'tidytree'
```

```
## Found more than one class "phylo" in cache; using the first, from namespace 'phyloseq'
```

```
## Also defined by 'tidytree'
```

```
## Found more than one class "phylo" in cache; using the first, from namespace 'phyloseq'
```

```
## Also defined by 'tidytree'
```

```
## Found more than one class "phylo" in cache; using the first, from namespace 'phyloseq'
```

```
## Also defined by 'tidytree'
```

```
## Found more than one class "phylo" in cache; using the first, from namespace 'phyloseq'
```

```
## Also defined by 'tidytree'
```

```
## Found more than one class "phylo" in cache; using the first, from namespace 'phyloseq'
```

```
## Also defined by 'tidytree'
```

```
## Found more than one class "phylo" in cache; using the first, from namespace 'phyloseq'
```

```
## Also defined by 'tidytree'
```

```
## Found more than one class "phylo" in cache; using the first, from namespace 'phyloseq'
```

```
## Also defined by 'tidytree'
```

```
## Found more than one class "phylo" in cache; using the first, from namespace 'phyloseq'
```

```
## Also defined by 'tidytree'
```

```
## Found more than one class "phylo" in cache; using the first, from namespace 'phyloseq'
```

```
## Also defined by 'tidytree'
```

```
## Found more than one class "phylo" in cache; using the first, from namespace 'phyloseq'
```

```
## Also defined by 'tidytree'
```

```
## Found more than one class "phylo" in cache; using the first, from namespace 'phyloseq'
```

```
## Also defined by 'tidytree'
```

```
## Found more than one class "phylo" in cache; using the first, from namespace 'phyloseq'
```

```
## Also defined by 'tidytree'
```

```
## Found more than one class "phylo" in cache; using the first, from namespace 'phyloseq'
```

```
## Also defined by 'tidytree'
```

```
## Found more than one class "phylo" in cache; using the first, from namespace 'phyloseq'
```

```
## Also defined by 'tidytree'
```

```
## Found more than one class "phylo" in cache; using the first, from namespace 'phyloseq'
```

```
## Also defined by 'tidytree'
```

```
## Found more than one class "phylo" in cache; using the first, from namespace 'phyloseq'
```

```
## Also defined by 'tidytree'
```

```
## Found more than one class "phylo" in cache; using the first, from namespace 'phyloseq'
```

```
## Also defined by 'tidytree'
```

```
## Found more than one class "phylo" in cache; using the first, from namespace 'phyloseq'
```

```
## Also defined by 'tidytree'
```

```
## Found more than one class "phylo" in cache; using the first, from namespace 'phyloseq'
```

```
## Also defined by 'tidytree'
```

```
## Found more than one class "phylo" in cache; using the first, from namespace 'phyloseq'
```

```
## Also defined by 'tidytree'
```

```
## Found more than one class "phylo" in cache; using the first, from namespace 'phyloseq'
```

```
## Also defined by 'tidytree'
```

```
## Found more than one class "phylo" in cache; using the first, from namespace 'phyloseq'
```

```
## Also defined by 'tidytree'
```

```
## Found more than one class "phylo" in cache; using the first, from namespace 'phyloseq'
```

```
## Also defined by 'tidytree'
```

```
## Found more than one class "phylo" in cache; using the first, from namespace 'phyloseq'
```

```
## Also defined by 'tidytree'
```

```
## Found more than one class "phylo" in cache; using the first, from namespace 'phyloseq'
```

```
## Also defined by 'tidytree'
```

```
## Found more than one class "phylo" in cache; using the first, from namespace 'phyloseq'
```

```
## Also defined by 'tidytree'
```

```
## Found more than one class "phylo" in cache; using the first, from namespace 'phyloseq'
```

```
## Also defined by 'tidytree'
```

```
## Found more than one class "phylo" in cache; using the first, from namespace 'phyloseq'
```

```
## Also defined by 'tidytree'
```

```
## Found more than one class "phylo" in cache; using the first, from namespace 'phyloseq'
```

```
## Also defined by 'tidytree'
```

```
## Found more than one class "phylo" in cache; using the first, from namespace 'phyloseq'
```

```
## Also defined by 'tidytree'
```

```
## Found more than one class "phylo" in cache; using the first, from namespace 'phyloseq'
```

```
## Also defined by 'tidytree'
```

```
## Found more than one class "phylo" in cache; using the first, from namespace 'phyloseq'
```

```
## Also defined by 'tidytree'
```

```
## Found more than one class "phylo" in cache; using the first, from namespace 'phyloseq'
```

```
## Also defined by 'tidytree'
```

```
## Found more than one class "phylo" in cache; using the first, from namespace 'phyloseq'
```

```
## Also defined by 'tidytree'
```

```
## Found more than one class "phylo" in cache; using the first, from namespace 'phyloseq'
```

```
## Also defined by 'tidytree'
```

```
## Found more than one class "phylo" in cache; using the first, from namespace 'phyloseq'
```

```
## Also defined by 'tidytree'
```

```
## Found more than one class "phylo" in cache; using the first, from namespace 'phyloseq'
```

```
## Also defined by 'tidytree'
```

```
## Found more than one class "phylo" in cache; using the first, from namespace 'phyloseq'
```

```
## Also defined by 'tidytree'
```

```
## Found more than one class "phylo" in cache; using the first, from namespace 'phyloseq'
```

```
## Also defined by 'tidytree'
```

```
## Found more than one class "phylo" in cache; using the first, from namespace 'phyloseq'
```

```
## Also defined by 'tidytree'
```

```
## Found more than one class "phylo" in cache; using the first, from namespace 'phyloseq'
```

```
## Also defined by 'tidytree'
```

```
## Found more than one class "phylo" in cache; using the first, from namespace 'phyloseq'
```

```
## Also defined by 'tidytree'
```

```
## Found more than one class "phylo" in cache; using the first, from namespace 'phyloseq'
```

```
## Also defined by 'tidytree'
```

```
## Found more than one class "phylo" in cache; using the first, from namespace 'phyloseq'
```

```
## Also defined by 'tidytree'
```

```
## Found more than one class "phylo" in cache; using the first, from namespace 'phyloseq'
```

```
## Also defined by 'tidytree'
```

```
## Found more than one class "phylo" in cache; using the first, from namespace 'phyloseq'
```

```
## Also defined by 'tidytree'
```

```
## Found more than one class "phylo" in cache; using the first, from namespace 'phyloseq'
```

```
## Also defined by 'tidytree'
```

```
## Found more than one class "phylo" in cache; using the first, from namespace 'phyloseq'
```

```
## Also defined by 'tidytree'
```

```
## Found more than one class "phylo" in cache; using the first, from namespace 'phyloseq'
```

```
## Also defined by 'tidytree'
```

```
## Found more than one class "phylo" in cache; using the first, from namespace 'phyloseq'
```

```
## Also defined by 'tidytree'
```

```
## Found more than one class "phylo" in cache; using the first, from namespace 'phyloseq'
```

```
## Also defined by 'tidytree'
```

```
## Found more than one class "phylo" in cache; using the first, from namespace 'phyloseq'
```

```
## Also defined by 'tidytree'
```

```
## Found more than one class "phylo" in cache; using the first, from namespace 'phyloseq'
```

```
## Also defined by 'tidytree'
```

```
## Found more than one class "phylo" in cache; using the first, from namespace 'phyloseq'
```

```
## Also defined by 'tidytree'
```

```
## Found more than one class "phylo" in cache; using the first, from namespace 'phyloseq'
```

```
## Also defined by 'tidytree'
```

```
## Found more than one class "phylo" in cache; using the first, from namespace 'phyloseq'
```

```
## Also defined by 'tidytree'
```

```
## Found more than one class "phylo" in cache; using the first, from namespace 'phyloseq'
```

```
## Also defined by 'tidytree'
```

```
## Found more than one class "phylo" in cache; using the first, from namespace 'phyloseq'
```

```
## Also defined by 'tidytree'
```

```
## Found more than one class "phylo" in cache; using the first, from namespace 'phyloseq'
```

```
## Also defined by 'tidytree'
```

```
## Found more than one class "phylo" in cache; using the first, from namespace 'phyloseq'
```

```
## Also defined by 'tidytree'
```

```
## Found more than one class "phylo" in cache; using the first, from namespace 'phyloseq'
```

```
## Also defined by 'tidytree'
```

```
## Found more than one class "phylo" in cache; using the first, from namespace 'phyloseq'
```

```
## Also defined by 'tidytree'
```

```
## Found more than one class "phylo" in cache; using the first, from namespace 'phyloseq'
```

```
## Also defined by 'tidytree'
```

```
## Found more than one class "phylo" in cache; using the first, from namespace 'phyloseq'
```

```
## Also defined by 'tidytree'
```

```
## Found more than one class "phylo" in cache; using the first, from namespace 'phyloseq'
```

```
## Also defined by 'tidytree'
```

```
## Found more than one class "phylo" in cache; using the first, from namespace 'phyloseq'
```

```
## Also defined by 'tidytree'
```

```
## Found more than one class "phylo" in cache; using the first, from namespace 'phyloseq'
```

```
## Also defined by 'tidytree'
```

```
## Found more than one class "phylo" in cache; using the first, from namespace 'phyloseq'
```

```
## Also defined by 'tidytree'
```

```
## Found more than one class "phylo" in cache; using the first, from namespace 'phyloseq'
```

```
## Also defined by 'tidytree'
```

```
## Found more than one class "phylo" in cache; using the first, from namespace 'phyloseq'
```

```
## Also defined by 'tidytree'
```

```
## Found more than one class "phylo" in cache; using the first, from namespace 'phyloseq'
```

```
## Also defined by 'tidytree'
```

```
## Found more than one class "phylo" in cache; using the first, from namespace 'phyloseq'
```

```
## Also defined by 'tidytree'
```

```
## Found more than one class "phylo" in cache; using the first, from namespace 'phyloseq'
```

```
## Also defined by 'tidytree'
```

```
## Found more than one class "phylo" in cache; using the first, from namespace 'phyloseq'
```

```
## Also defined by 'tidytree'
```

```
## Found more than one class "phylo" in cache; using the first, from namespace 'phyloseq'
```

```
## Also defined by 'tidytree'
```

```
## Found more than one class "phylo" in cache; using the first, from namespace 'phyloseq'
```

```
## Also defined by 'tidytree'
```

```
## Found more than one class "phylo" in cache; using the first, from namespace 'phyloseq'
```

```
## Also defined by 'tidytree'
```

```
## Found more than one class "phylo" in cache; using the first, from namespace 'phyloseq'
```

```
## Also defined by 'tidytree'
```

```
## Found more than one class "phylo" in cache; using the first, from namespace 'phyloseq'
```

```
## Also defined by 'tidytree'
```

```
## Found more than one class "phylo" in cache; using the first, from namespace 'phyloseq'
```

```
## Also defined by 'tidytree'
```

```
## Found more than one class "phylo" in cache; using the first, from namespace 'phyloseq'
```

```
## Also defined by 'tidytree'
```

```
## Found more than one class "phylo" in cache; using the first, from namespace 'phyloseq'
```

```
## Also defined by 'tidytree'
```

```
## Found more than one class "phylo" in cache; using the first, from namespace 'phyloseq'
```

```
## Also defined by 'tidytree'
```

```
## Found more than one class "phylo" in cache; using the first, from namespace 'phyloseq'
```

```
## Also defined by 'tidytree'
```

```
## Found more than one class "phylo" in cache; using the first, from namespace 'phyloseq'
```

```
## Also defined by 'tidytree'
```

```
## Found more than one class "phylo" in cache; using the first, from namespace 'phyloseq'
```

```
## Also defined by 'tidytree'
```

```
## Found more than one class "phylo" in cache; using the first, from namespace 'phyloseq'
```

```
## Also defined by 'tidytree'
```

```
## Found more than one class "phylo" in cache; using the first, from namespace 'phyloseq'
```

```
## Also defined by 'tidytree'
```

```
## Found more than one class "phylo" in cache; using the first, from namespace 'phyloseq'
```

```
## Also defined by 'tidytree'
```

```
## Found more than one class "phylo" in cache; using the first, from namespace 'phyloseq'
```

```
## Also defined by 'tidytree'
```

```
## Found more than one class "phylo" in cache; using the first, from namespace 'phyloseq'
```

```
## Also defined by 'tidytree'
```

```
## Found more than one class "phylo" in cache; using the first, from namespace 'phyloseq'
```

```
## Also defined by 'tidytree'
```

```
## Found more than one class "phylo" in cache; using the first, from namespace 'phyloseq'
```

```
## Also defined by 'tidytree'
```

```
## Found more than one class "phylo" in cache; using the first, from namespace 'phyloseq'
```

```
## Also defined by 'tidytree'
```

```
## Found more than one class "phylo" in cache; using the first, from namespace 'phyloseq'
```

```
## Also defined by 'tidytree'
```

```
## Found more than one class "phylo" in cache; using the first, from namespace 'phyloseq'
```

```
## Also defined by 'tidytree'
```

```
## Found more than one class "phylo" in cache; using the first, from namespace 'phyloseq'
```

```
## Also defined by 'tidytree'
```

```
## Found more than one class "phylo" in cache; using the first, from namespace 'phyloseq'
```

```
## Also defined by 'tidytree'
```

```
## Found more than one class "phylo" in cache; using the first, from namespace 'phyloseq'
```

```
## Also defined by 'tidytree'
```

```
## Found more than one class "phylo" in cache; using the first, from namespace 'phyloseq'
```

```
## Also defined by 'tidytree'
```

```
## Found more than one class "phylo" in cache; using the first, from namespace 'phyloseq'
```

```
## Also defined by 'tidytree'
```

```
## Found more than one class "phylo" in cache; using the first, from namespace 'phyloseq'
```

```
## Also defined by 'tidytree'
```

```
## Found more than one class "phylo" in cache; using the first, from namespace 'phyloseq'
```

```
## Also defined by 'tidytree'
```

```
## Found more than one class "phylo" in cache; using the first, from namespace 'phyloseq'
```

```
## Also defined by 'tidytree'
```

```
## Found more than one class "phylo" in cache; using the first, from namespace 'phyloseq'
```

```
## Also defined by 'tidytree'
```

```
## Found more than one class "phylo" in cache; using the first, from namespace 'phyloseq'
```

```
## Also defined by 'tidytree'
```

```
## Found more than one class "phylo" in cache; using the first, from namespace 'phyloseq'
```

```
## Also defined by 'tidytree'
```

```
## Found more than one class "phylo" in cache; using the first, from namespace 'phyloseq'
```

```
## Also defined by 'tidytree'
```

```
## Found more than one class "phylo" in cache; using the first, from namespace 'phyloseq'
```

```
## Also defined by 'tidytree'
```

```
## Found more than one class "phylo" in cache; using the first, from namespace 'phyloseq'
```

```
## Also defined by 'tidytree'
```

```
## Found more than one class "phylo" in cache; using the first, from namespace 'phyloseq'
```

```
## Also defined by 'tidytree'
```

```
## Found more than one class "phylo" in cache; using the first, from namespace 'phyloseq'
```

```
## Also defined by 'tidytree'
```

```
## Found more than one class "phylo" in cache; using the first, from namespace 'phyloseq'
```

```
## Also defined by 'tidytree'
```

```
## Found more than one class "phylo" in cache; using the first, from namespace 'phyloseq'
```

```
## Also defined by 'tidytree'
```

```
## Found more than one class "phylo" in cache; using the first, from namespace 'phyloseq'
```

```
## Also defined by 'tidytree'
```

```
## Found more than one class "phylo" in cache; using the first, from namespace 'phyloseq'
```

```
## Also defined by 'tidytree'
```

```
## Found more than one class "phylo" in cache; using the first, from namespace 'phyloseq'
```

```
## Also defined by 'tidytree'
```

```
## Found more than one class "phylo" in cache; using the first, from namespace 'phyloseq'
```

```
## Also defined by 'tidytree'
```

```
## Found more than one class "phylo" in cache; using the first, from namespace 'phyloseq'
```

```
## Also defined by 'tidytree'
```

```
## Found more than one class "phylo" in cache; using the first, from namespace 'phyloseq'
```

```
## Also defined by 'tidytree'
```

```
## Found more than one class "phylo" in cache; using the first, from namespace 'phyloseq'
```

```
## Also defined by 'tidytree'
```

```
## Found more than one class "phylo" in cache; using the first, from namespace 'phyloseq'
```

```
## Also defined by 'tidytree'
```

```
## Found more than one class "phylo" in cache; using the first, from namespace 'phyloseq'
```

```
## Also defined by 'tidytree'
```

```
## Found more than one class "phylo" in cache; using the first, from namespace 'phyloseq'
```

```
## Also defined by 'tidytree'
```

```
## Found more than one class "phylo" in cache; using the first, from namespace 'phyloseq'
```

```
## Also defined by 'tidytree'
```

```
## Found more than one class "phylo" in cache; using the first, from namespace 'phyloseq'
```

```
## Also defined by 'tidytree'
```

```
## Found more than one class "phylo" in cache; using the first, from namespace 'phyloseq'
```

```
## Also defined by 'tidytree'
```

```
## Found more than one class "phylo" in cache; using the first, from namespace 'phyloseq'
```

```
## Also defined by 'tidytree'
```

```
## Found more than one class "phylo" in cache; using the first, from namespace 'phyloseq'
```

```
## Also defined by 'tidytree'
```

```
## Found more than one class "phylo" in cache; using the first, from namespace 'phyloseq'
```

```
## Also defined by 'tidytree'
```

```
## Found more than one class "phylo" in cache; using the first, from namespace 'phyloseq'
```

```
## Also defined by 'tidytree'
```

```
## Found more than one class "phylo" in cache; using the first, from namespace 'phyloseq'
```

```
## Also defined by 'tidytree'
```

```
## Found more than one class "phylo" in cache; using the first, from namespace 'phyloseq'
```

```
## Also defined by 'tidytree'
```

```
## Found more than one class "phylo" in cache; using the first, from namespace 'phyloseq'
```

```
## Also defined by 'tidytree'
```

```
## Found more than one class "phylo" in cache; using the first, from namespace 'phyloseq'
```

```
## Also defined by 'tidytree'
```

```
## Found more than one class "phylo" in cache; using the first, from namespace 'phyloseq'
```

```
## Also defined by 'tidytree'
```

```
## Found more than one class "phylo" in cache; using the first, from namespace 'phyloseq'
```

```
## Also defined by 'tidytree'
```

```
## Found more than one class "phylo" in cache; using the first, from namespace 'phyloseq'
```

```
## Also defined by 'tidytree'
```

```
## Found more than one class "phylo" in cache; using the first, from namespace 'phyloseq'
```

```
## Also defined by 'tidytree'
```

```
## Found more than one class "phylo" in cache; using the first, from namespace 'phyloseq'
```

```
## Also defined by 'tidytree'
```

```
## Found more than one class "phylo" in cache; using the first, from namespace 'phyloseq'
```

```
## Also defined by 'tidytree'
```

```
## Found more than one class "phylo" in cache; using the first, from namespace 'phyloseq'
```

```
## Also defined by 'tidytree'
```

```
## Found more than one class "phylo" in cache; using the first, from namespace 'phyloseq'
```

```
## Also defined by 'tidytree'
```

```
## Found more than one class "phylo" in cache; using the first, from namespace 'phyloseq'
```

```
## Also defined by 'tidytree'
```

```
## Found more than one class "phylo" in cache; using the first, from namespace 'phyloseq'
```

```
## Also defined by 'tidytree'
```

```
## Found more than one class "phylo" in cache; using the first, from namespace 'phyloseq'
```

```
## Also defined by 'tidytree'
```

```
## Found more than one class "phylo" in cache; using the first, from namespace 'phyloseq'
```

```
## Also defined by 'tidytree'
```

```
## Found more than one class "phylo" in cache; using the first, from namespace 'phyloseq'
```

```
## Also defined by 'tidytree'
```

```
## Found more than one class "phylo" in cache; using the first, from namespace 'phyloseq'
```

```
## Also defined by 'tidytree'
```

```
## Found more than one class "phylo" in cache; using the first, from namespace 'phyloseq'
```

```
## Also defined by 'tidytree'
```

```
## Found more than one class "phylo" in cache; using the first, from namespace 'phyloseq'
```

```
## Also defined by 'tidytree'
```

```
## Found more than one class "phylo" in cache; using the first, from namespace 'phyloseq'
```

```
## Also defined by 'tidytree'
```

```
## Found more than one class "phylo" in cache; using the first, from namespace 'phyloseq'
```

```
## Also defined by 'tidytree'
```

```
## Found more than one class "phylo" in cache; using the first, from namespace 'phyloseq'
```

```
## Also defined by 'tidytree'
```

```
## Found more than one class "phylo" in cache; using the first, from namespace 'phyloseq'
```

```
## Also defined by 'tidytree'
```

```
## Found more than one class "phylo" in cache; using the first, from namespace 'phyloseq'
```

```
## Also defined by 'tidytree'
```

```
## Found more than one class "phylo" in cache; using the first, from namespace 'phyloseq'
```

```
## Also defined by 'tidytree'
```

```
## Found more than one class "phylo" in cache; using the first, from namespace 'phyloseq'
```

```
## Also defined by 'tidytree'
```

```
## Found more than one class "phylo" in cache; using the first, from namespace 'phyloseq'
```

```
## Also defined by 'tidytree'
```

```
## Found more than one class "phylo" in cache; using the first, from namespace 'phyloseq'
```

```
## Also defined by 'tidytree'
```

```
## Found more than one class "phylo" in cache; using the first, from namespace 'phyloseq'
```

```
## Also defined by 'tidytree'
```

```
## Found more than one class "phylo" in cache; using the first, from namespace 'phyloseq'
```

```
## Also defined by 'tidytree'
```

```
## Found more than one class "phylo" in cache; using the first, from namespace 'phyloseq'
```

```
## Also defined by 'tidytree'
```

```
## Found more than one class "phylo" in cache; using the first, from namespace 'phyloseq'
```

```
## Also defined by 'tidytree'
```

```
## Found more than one class "phylo" in cache; using the first, from namespace 'phyloseq'
```

```
## Also defined by 'tidytree'
```

```
## Found more than one class "phylo" in cache; using the first, from namespace 'phyloseq'
```

```
## Also defined by 'tidytree'
```

```
## Found more than one class "phylo" in cache; using the first, from namespace 'phyloseq'
```

```
## Also defined by 'tidytree'
```

```
## Found more than one class "phylo" in cache; using the first, from namespace 'phyloseq'
```

```
## Also defined by 'tidytree'
```

```
## Found more than one class "phylo" in cache; using the first, from namespace 'phyloseq'
```

```
## Also defined by 'tidytree'
```

```
## Found more than one class "phylo" in cache; using the first, from namespace 'phyloseq'
```

```
## Also defined by 'tidytree'
```

```
## Found more than one class "phylo" in cache; using the first, from namespace 'phyloseq'
```

```
## Also defined by 'tidytree'
```

```
## Found more than one class "phylo" in cache; using the first, from namespace 'phyloseq'
```

```
## Also defined by 'tidytree'
```

```
## Found more than one class "phylo" in cache; using the first, from namespace 'phyloseq'
```

```
## Also defined by 'tidytree'
```

```
## Found more than one class "phylo" in cache; using the first, from namespace 'phyloseq'
```

```
## Also defined by 'tidytree'
```

```
## Found more than one class "phylo" in cache; using the first, from namespace 'phyloseq'
```

```
## Also defined by 'tidytree'
```

```
## Found more than one class "phylo" in cache; using the first, from namespace 'phyloseq'
```

```
## Also defined by 'tidytree'
```

```
## Found more than one class "phylo" in cache; using the first, from namespace 'phyloseq'
```

```
## Also defined by 'tidytree'
```

```
## Found more than one class "phylo" in cache; using the first, from namespace 'phyloseq'
```

```
## Also defined by 'tidytree'
```

```
## Found more than one class "phylo" in cache; using the first, from namespace 'phyloseq'
```

```
## Also defined by 'tidytree'
```

```
## Found more than one class "phylo" in cache; using the first, from namespace 'phyloseq'
```

```
## Also defined by 'tidytree'
```

```
## Found more than one class "phylo" in cache; using the first, from namespace 'phyloseq'
```

```
## Also defined by 'tidytree'
```

```
## Found more than one class "phylo" in cache; using the first, from namespace 'phyloseq'
```

```
## Also defined by 'tidytree'
```

```
## Found more than one class "phylo" in cache; using the first, from namespace 'phyloseq'
```

```
## Also defined by 'tidytree'
```

```
## Found more than one class "phylo" in cache; using the first, from namespace 'phyloseq'
```

```
## Also defined by 'tidytree'
```

```
## Found more than one class "phylo" in cache; using the first, from namespace 'phyloseq'
```

```
## Also defined by 'tidytree'
```

```
## Found more than one class "phylo" in cache; using the first, from namespace 'phyloseq'
```

```
## Also defined by 'tidytree'
```

```
## Found more than one class "phylo" in cache; using the first, from namespace 'phyloseq'
```

```
## Also defined by 'tidytree'
```

```
## Found more than one class "phylo" in cache; using the first, from namespace 'phyloseq'
```

```
## Also defined by 'tidytree'
```

```
## Found more than one class "phylo" in cache; using the first, from namespace 'phyloseq'
```

```
## Also defined by 'tidytree'
```

```
## Found more than one class "phylo" in cache; using the first, from namespace 'phyloseq'
```

```
## Also defined by 'tidytree'
```

```
## Found more than one class "phylo" in cache; using the first, from namespace 'phyloseq'
```

```
## Also defined by 'tidytree'
```

```
## Found more than one class "phylo" in cache; using the first, from namespace 'phyloseq'
```

```
## Also defined by 'tidytree'
```

```
## Found more than one class "phylo" in cache; using the first, from namespace 'phyloseq'
```

```
## Also defined by 'tidytree'
```

```
## Found more than one class "phylo" in cache; using the first, from namespace 'phyloseq'
```

```
## Also defined by 'tidytree'
```

```
## Found more than one class "phylo" in cache; using the first, from namespace 'phyloseq'
```

```
## Also defined by 'tidytree'
```

```
## Found more than one class "phylo" in cache; using the first, from namespace 'phyloseq'
```

```
## Also defined by 'tidytree'
```

```
## Found more than one class "phylo" in cache; using the first, from namespace 'phyloseq'
```

```
## Also defined by 'tidytree'
```

```
## Found more than one class "phylo" in cache; using the first, from namespace 'phyloseq'
```

```
## Also defined by 'tidytree'
```

```
## Found more than one class "phylo" in cache; using the first, from namespace 'phyloseq'
```

```
## Also defined by 'tidytree'
```

```
## Found more than one class "phylo" in cache; using the first, from namespace 'phyloseq'
```

```
## Also defined by 'tidytree'
```

```
## Found more than one class "phylo" in cache; using the first, from namespace 'phyloseq'
```

```
## Also defined by 'tidytree'
```

```
## Found more than one class "phylo" in cache; using the first, from namespace 'phyloseq'
```

```
## Also defined by 'tidytree'
```

```
## Found more than one class "phylo" in cache; using the first, from namespace 'phyloseq'
```

```
## Also defined by 'tidytree'
```

```
## Found more than one class "phylo" in cache; using the first, from namespace 'phyloseq'
```

```
## Also defined by 'tidytree'
```

```
## Found more than one class "phylo" in cache; using the first, from namespace 'phyloseq'
```

```
## Also defined by 'tidytree'
```

```
## Found more than one class "phylo" in cache; using the first, from namespace 'phyloseq'
```

```
## Also defined by 'tidytree'
```

```
## Found more than one class "phylo" in cache; using the first, from namespace 'phyloseq'
```

```
## Also defined by 'tidytree'
```

```
## Found more than one class "phylo" in cache; using the first, from namespace 'phyloseq'
```

```
## Also defined by 'tidytree'
```

```
## Found more than one class "phylo" in cache; using the first, from namespace 'phyloseq'
```

```
## Also defined by 'tidytree'
```

```
## Found more than one class "phylo" in cache; using the first, from namespace 'phyloseq'
```

```
## Also defined by 'tidytree'
```

```
## Found more than one class "phylo" in cache; using the first, from namespace 'phyloseq'
```

```
## Also defined by 'tidytree'
```

```
## Found more than one class "phylo" in cache; using the first, from namespace 'phyloseq'
```

```
## Also defined by 'tidytree'
```

```
## Found more than one class "phylo" in cache; using the first, from namespace 'phyloseq'
```

```
## Also defined by 'tidytree'
```

```
## Found more than one class "phylo" in cache; using the first, from namespace 'phyloseq'
```

```
## Also defined by 'tidytree'
```

```
## Found more than one class "phylo" in cache; using the first, from namespace 'phyloseq'
```

```
## Also defined by 'tidytree'
```

```
## Found more than one class "phylo" in cache; using the first, from namespace 'phyloseq'
```

```
## Also defined by 'tidytree'
```

```
## Found more than one class "phylo" in cache; using the first, from namespace 'phyloseq'
```

```
## Also defined by 'tidytree'
```

```
## Found more than one class "phylo" in cache; using the first, from namespace 'phyloseq'
```

```
## Also defined by 'tidytree'
```

```
## Found more than one class "phylo" in cache; using the first, from namespace 'phyloseq'
```

```
## Also defined by 'tidytree'
```

```
## Found more than one class "phylo" in cache; using the first, from namespace 'phyloseq'
```

```
## Also defined by 'tidytree'
```

```
## Found more than one class "phylo" in cache; using the first, from namespace 'phyloseq'
```

```
## Also defined by 'tidytree'
```

```
## Found more than one class "phylo" in cache; using the first, from namespace 'phyloseq'
```

```
## Also defined by 'tidytree'
```

```
## Found more than one class "phylo" in cache; using the first, from namespace 'phyloseq'
```

```
## Also defined by 'tidytree'
```

```
## Found more than one class "phylo" in cache; using the first, from namespace 'phyloseq'
```

```
## Also defined by 'tidytree'
```

```
## Found more than one class "phylo" in cache; using the first, from namespace 'phyloseq'
```

```
## Also defined by 'tidytree'
```

```
## Found more than one class "phylo" in cache; using the first, from namespace 'phyloseq'
```

```
## Also defined by 'tidytree'
```

```
## Found more than one class "phylo" in cache; using the first, from namespace 'phyloseq'
```

```
## Also defined by 'tidytree'
```

```
## Found more than one class "phylo" in cache; using the first, from namespace 'phyloseq'
```

```
## Also defined by 'tidytree'
```

```
## Found more than one class "phylo" in cache; using the first, from namespace 'phyloseq'
```

```
## Also defined by 'tidytree'
```

```
## Found more than one class "phylo" in cache; using the first, from namespace 'phyloseq'
```

```
## Also defined by 'tidytree'
```

```
## Found more than one class "phylo" in cache; using the first, from namespace 'phyloseq'
```

```
## Also defined by 'tidytree'
```

```
## Found more than one class "phylo" in cache; using the first, from namespace 'phyloseq'
```

```
## Also defined by 'tidytree'
```

```
## Found more than one class "phylo" in cache; using the first, from namespace 'phyloseq'
```

```
## Also defined by 'tidytree'
```

```
## Found more than one class "phylo" in cache; using the first, from namespace 'phyloseq'
```

```
## Also defined by 'tidytree'
```

```
## Found more than one class "phylo" in cache; using the first, from namespace 'phyloseq'
```

```
## Also defined by 'tidytree'
```

```
## Found more than one class "phylo" in cache; using the first, from namespace 'phyloseq'
```

```
## Also defined by 'tidytree'
```

```
## Found more than one class "phylo" in cache; using the first, from namespace 'phyloseq'
```

```
## Also defined by 'tidytree'
```

```
## Found more than one class "phylo" in cache; using the first, from namespace 'phyloseq'
```

```
## Also defined by 'tidytree'
```

```
## Found more than one class "phylo" in cache; using the first, from namespace 'phyloseq'
```

```
## Also defined by 'tidytree'
```

```
## Found more than one class "phylo" in cache; using the first, from namespace 'phyloseq'
```

```
## Also defined by 'tidytree'
```

```
## Found more than one class "phylo" in cache; using the first, from namespace 'phyloseq'
```

```
## Also defined by 'tidytree'
```

```
## Found more than one class "phylo" in cache; using the first, from namespace 'phyloseq'
```

```
## Also defined by 'tidytree'
```

```
## Found more than one class "phylo" in cache; using the first, from namespace 'phyloseq'
```

```
## Also defined by 'tidytree'
```

```
## Found more than one class "phylo" in cache; using the first, from namespace 'phyloseq'
```

```
## Also defined by 'tidytree'
```

```
## Found more than one class "phylo" in cache; using the first, from namespace 'phyloseq'
```

```
## Also defined by 'tidytree'
```

```
## Found more than one class "phylo" in cache; using the first, from namespace 'phyloseq'
```

```
## Also defined by 'tidytree'
```

```
## Found more than one class "phylo" in cache; using the first, from namespace 'phyloseq'
```

```
## Also defined by 'tidytree'
```

```
## Found more than one class "phylo" in cache; using the first, from namespace 'phyloseq'
```

```
## Also defined by 'tidytree'
```

```
## Found more than one class "phylo" in cache; using the first, from namespace 'phyloseq'
```

```
## Also defined by 'tidytree'
```

```
## Found more than one class "phylo" in cache; using the first, from namespace 'phyloseq'
```

```
## Also defined by 'tidytree'
```

```
## Found more than one class "phylo" in cache; using the first, from namespace 'phyloseq'
```

```
## Also defined by 'tidytree'
```

```
## Found more than one class "phylo" in cache; using the first, from namespace 'phyloseq'
```

```
## Also defined by 'tidytree'
```

```
## Found more than one class "phylo" in cache; using the first, from namespace 'phyloseq'
```

```
## Also defined by 'tidytree'
```

```
## Found more than one class "phylo" in cache; using the first, from namespace 'phyloseq'
```

```
## Also defined by 'tidytree'
```

```
## Found more than one class "phylo" in cache; using the first, from namespace 'phyloseq'
```

```
## Also defined by 'tidytree'
```

```
## Found more than one class "phylo" in cache; using the first, from namespace 'phyloseq'
```

```
## Also defined by 'tidytree'
```

```
## Found more than one class "phylo" in cache; using the first, from namespace 'phyloseq'
```

```
## Also defined by 'tidytree'
```

```
## Found more than one class "phylo" in cache; using the first, from namespace 'phyloseq'
```

```
## Also defined by 'tidytree'
```

```
## Found more than one class "phylo" in cache; using the first, from namespace 'phyloseq'
```

```
## Also defined by 'tidytree'
```

```
## Found more than one class "phylo" in cache; using the first, from namespace 'phyloseq'
```

```
## Also defined by 'tidytree'
```

```
## Found more than one class "phylo" in cache; using the first, from namespace 'phyloseq'
```

```
## Also defined by 'tidytree'
```

```
## Found more than one class "phylo" in cache; using the first, from namespace 'phyloseq'
```

```
## Also defined by 'tidytree'
```

```
## Found more than one class "phylo" in cache; using the first, from namespace 'phyloseq'
```

```
## Also defined by 'tidytree'
```

```
## Found more than one class "phylo" in cache; using the first, from namespace 'phyloseq'
```

```
## Also defined by 'tidytree'
```

```
## Found more than one class "phylo" in cache; using the first, from namespace 'phyloseq'
```

```
## Also defined by 'tidytree'
```

```
## Found more than one class "phylo" in cache; using the first, from namespace 'phyloseq'
```

```
## Also defined by 'tidytree'
```

```
## Found more than one class "phylo" in cache; using the first, from namespace 'phyloseq'
```

```
## Also defined by 'tidytree'
```

```
## Found more than one class "phylo" in cache; using the first, from namespace 'phyloseq'
```

```
## Also defined by 'tidytree'
```

```
## Found more than one class "phylo" in cache; using the first, from namespace 'phyloseq'
```

```
## Also defined by 'tidytree'
```

```
## Found more than one class "phylo" in cache; using the first, from namespace 'phyloseq'
```

```
## Also defined by 'tidytree'
```

```
## Found more than one class "phylo" in cache; using the first, from namespace 'phyloseq'
```

```
## Also defined by 'tidytree'
```

```
## Found more than one class "phylo" in cache; using the first, from namespace 'phyloseq'
```

```
## Also defined by 'tidytree'
```

```
## Found more than one class "phylo" in cache; using the first, from namespace 'phyloseq'
```

```
## Also defined by 'tidytree'
```

```
## Found more than one class "phylo" in cache; using the first, from namespace 'phyloseq'
```

```
## Also defined by 'tidytree'
```

```
## Found more than one class "phylo" in cache; using the first, from namespace 'phyloseq'
```

```
## Also defined by 'tidytree'
```

```
## Found more than one class "phylo" in cache; using the first, from namespace 'phyloseq'
```

```
## Also defined by 'tidytree'
```

```
## Found more than one class "phylo" in cache; using the first, from namespace 'phyloseq'
```

```
## Also defined by 'tidytree'
```

```
## Found more than one class "phylo" in cache; using the first, from namespace 'phyloseq'
```

```
## Also defined by 'tidytree'
```

```
## Found more than one class "phylo" in cache; using the first, from namespace 'phyloseq'
```

```
## Also defined by 'tidytree'
```

```
## Found more than one class "phylo" in cache; using the first, from namespace 'phyloseq'
```

```
## Also defined by 'tidytree'
```

```
## Found more than one class "phylo" in cache; using the first, from namespace 'phyloseq'
```

```
## Also defined by 'tidytree'
```

```
## Found more than one class "phylo" in cache; using the first, from namespace 'phyloseq'
```

```
## Also defined by 'tidytree'
```

```
## Found more than one class "phylo" in cache; using the first, from namespace 'phyloseq'
```

```
## Also defined by 'tidytree'
```

```
## Found more than one class "phylo" in cache; using the first, from namespace 'phyloseq'
```

```
## Also defined by 'tidytree'
```

```
## Found more than one class "phylo" in cache; using the first, from namespace 'phyloseq'
```

```
## Also defined by 'tidytree'
```

```
## Found more than one class "phylo" in cache; using the first, from namespace 'phyloseq'
```

```
## Also defined by 'tidytree'
```

```
## Found more than one class "phylo" in cache; using the first, from namespace 'phyloseq'
```

```
## Also defined by 'tidytree'
```

```
## Found more than one class "phylo" in cache; using the first, from namespace 'phyloseq'
```

```
## Also defined by 'tidytree'
```

```
## Found more than one class "phylo" in cache; using the first, from namespace 'phyloseq'
```

```
## Also defined by 'tidytree'
```

```
## Found more than one class "phylo" in cache; using the first, from namespace 'phyloseq'
```

```
## Also defined by 'tidytree'
```

```
## Found more than one class "phylo" in cache; using the first, from namespace 'phyloseq'
```

```
## Also defined by 'tidytree'
```

```
## Found more than one class "phylo" in cache; using the first, from namespace 'phyloseq'
```

```
## Also defined by 'tidytree'
```

```
## Found more than one class "phylo" in cache; using the first, from namespace 'phyloseq'
```

```
## Also defined by 'tidytree'
```

```
## Found more than one class "phylo" in cache; using the first, from namespace 'phyloseq'
```

```
## Also defined by 'tidytree'
```

```
## Found more than one class "phylo" in cache; using the first, from namespace 'phyloseq'
```

```
## Also defined by 'tidytree'
```

```
## Found more than one class "phylo" in cache; using the first, from namespace 'phyloseq'
```

```
## Also defined by 'tidytree'
```

```
## Found more than one class "phylo" in cache; using the first, from namespace 'phyloseq'
```

```
## Also defined by 'tidytree'
```

```
## Found more than one class "phylo" in cache; using the first, from namespace 'phyloseq'
```

```
## Also defined by 'tidytree'
```

```
## Found more than one class "phylo" in cache; using the first, from namespace 'phyloseq'
```

```
## Also defined by 'tidytree'
```

```
## Found more than one class "phylo" in cache; using the first, from namespace 'phyloseq'
```

```
## Also defined by 'tidytree'
```

```
## Found more than one class "phylo" in cache; using the first, from namespace 'phyloseq'
```

```
## Also defined by 'tidytree'
```

```
## Found more than one class "phylo" in cache; using the first, from namespace 'phyloseq'
```

```
## Also defined by 'tidytree'
```

```
## Found more than one class "phylo" in cache; using the first, from namespace 'phyloseq'
```

```
## Also defined by 'tidytree'
```

```
## Found more than one class "phylo" in cache; using the first, from namespace 'phyloseq'
```

```
## Also defined by 'tidytree'
```

```
## Found more than one class "phylo" in cache; using the first, from namespace 'phyloseq'
```

```
## Also defined by 'tidytree'
```

```
## Found more than one class "phylo" in cache; using the first, from namespace 'phyloseq'
```

```
## Also defined by 'tidytree'
```

```
## Found more than one class "phylo" in cache; using the first, from namespace 'phyloseq'
```

```
## Also defined by 'tidytree'
```

```
## Found more than one class "phylo" in cache; using the first, from namespace 'phyloseq'
```

```
## Also defined by 'tidytree'
```

```
## Found more than one class "phylo" in cache; using the first, from namespace 'phyloseq'
```

```
## Also defined by 'tidytree'
```

```
## Found more than one class "phylo" in cache; using the first, from namespace 'phyloseq'
```

```
## Also defined by 'tidytree'
```

```
## Found more than one class "phylo" in cache; using the first, from namespace 'phyloseq'
```

```
## Also defined by 'tidytree'
```

```
## Found more than one class "phylo" in cache; using the first, from namespace 'phyloseq'
```

```
## Also defined by 'tidytree'
```

```
## Found more than one class "phylo" in cache; using the first, from namespace 'phyloseq'
```

```
## Also defined by 'tidytree'
```

```
## Found more than one class "phylo" in cache; using the first, from namespace 'phyloseq'
```

```
## Also defined by 'tidytree'
```

```
## Found more than one class "phylo" in cache; using the first, from namespace 'phyloseq'
```

```
## Also defined by 'tidytree'
```

```
## Found more than one class "phylo" in cache; using the first, from namespace 'phyloseq'
```

```
## Also defined by 'tidytree'
```

```
## Found more than one class "phylo" in cache; using the first, from namespace 'phyloseq'
```

```
## Also defined by 'tidytree'
```

```
## Found more than one class "phylo" in cache; using the first, from namespace 'phyloseq'
```

```
## Also defined by 'tidytree'
```

```
## Found more than one class "phylo" in cache; using the first, from namespace 'phyloseq'
```

```
## Also defined by 'tidytree'
```

```
## Found more than one class "phylo" in cache; using the first, from namespace 'phyloseq'
```

```
## Also defined by 'tidytree'
```

```
## Found more than one class "phylo" in cache; using the first, from namespace 'phyloseq'
```

```
## Also defined by 'tidytree'
```

```
## Found more than one class "phylo" in cache; using the first, from namespace 'phyloseq'
```

```
## Also defined by 'tidytree'
```

```
## Found more than one class "phylo" in cache; using the first, from namespace 'phyloseq'
```

```
## Also defined by 'tidytree'
```

```
## Found more than one class "phylo" in cache; using the first, from namespace 'phyloseq'
```

```
## Also defined by 'tidytree'
```

```
## Found more than one class "phylo" in cache; using the first, from namespace 'phyloseq'
```

```
## Also defined by 'tidytree'
```

```
## Found more than one class "phylo" in cache; using the first, from namespace 'phyloseq'
```

```
## Also defined by 'tidytree'
```

```
## Found more than one class "phylo" in cache; using the first, from namespace 'phyloseq'
```

```
## Also defined by 'tidytree'
```

```
## Found more than one class "phylo" in cache; using the first, from namespace 'phyloseq'
```

```
## Also defined by 'tidytree'
```

```
## Found more than one class "phylo" in cache; using the first, from namespace 'phyloseq'
```

```
## Also defined by 'tidytree'
```

```
## Found more than one class "phylo" in cache; using the first, from namespace 'phyloseq'
```

```
## Also defined by 'tidytree'
```

```
## Found more than one class "phylo" in cache; using the first, from namespace 'phyloseq'
```

```
## Also defined by 'tidytree'
```

```
## Found more than one class "phylo" in cache; using the first, from namespace 'phyloseq'
```

```
## Also defined by 'tidytree'
```

```
## Found more than one class "phylo" in cache; using the first, from namespace 'phyloseq'
```

```
## Also defined by 'tidytree'
```

```
## Found more than one class "phylo" in cache; using the first, from namespace 'phyloseq'
```

```
## Also defined by 'tidytree'
```

```
## Found more than one class "phylo" in cache; using the first, from namespace 'phyloseq'
```

```
## Also defined by 'tidytree'
```

```
## Found more than one class "phylo" in cache; using the first, from namespace 'phyloseq'
```

```
## Also defined by 'tidytree'
```

```
## Found more than one class "phylo" in cache; using the first, from namespace 'phyloseq'
```

```
## Also defined by 'tidytree'
```

```
## Found more than one class "phylo" in cache; using the first, from namespace 'phyloseq'
```

```
## Also defined by 'tidytree'
```

```
## Found more than one class "phylo" in cache; using the first, from namespace 'phyloseq'
```

```
## Also defined by 'tidytree'
```

```
## Found more than one class "phylo" in cache; using the first, from namespace 'phyloseq'
```

```
## Also defined by 'tidytree'
```

```
## Found more than one class "phylo" in cache; using the first, from namespace 'phyloseq'
```

```
## Also defined by 'tidytree'
```

```
## Found more than one class "phylo" in cache; using the first, from namespace 'phyloseq'
```

```
## Also defined by 'tidytree'
```

```
## Found more than one class "phylo" in cache; using the first, from namespace 'phyloseq'
```

```
## Also defined by 'tidytree'
```

```
## Found more than one class "phylo" in cache; using the first, from namespace 'phyloseq'
```

```
## Also defined by 'tidytree'
```

```
## Found more than one class "phylo" in cache; using the first, from namespace 'phyloseq'
```

```
## Also defined by 'tidytree'
```

```
## Found more than one class "phylo" in cache; using the first, from namespace 'phyloseq'
```

```
## Also defined by 'tidytree'
```

```
## Found more than one class "phylo" in cache; using the first, from namespace 'phyloseq'
```

```
## Also defined by 'tidytree'
```

```
## Found more than one class "phylo" in cache; using the first, from namespace 'phyloseq'
```

```
## Also defined by 'tidytree'
```

```
## Found more than one class "phylo" in cache; using the first, from namespace 'phyloseq'
```

```
## Also defined by 'tidytree'
```

```
## Found more than one class "phylo" in cache; using the first, from namespace 'phyloseq'
```

```
## Also defined by 'tidytree'
```

```
## Found more than one class "phylo" in cache; using the first, from namespace 'phyloseq'
```

```
## Also defined by 'tidytree'
```

```
## Found more than one class "phylo" in cache; using the first, from namespace 'phyloseq'
```

```
## Also defined by 'tidytree'
```

```
## Found more than one class "phylo" in cache; using the first, from namespace 'phyloseq'
```

```
## Also defined by 'tidytree'
```

```
## Found more than one class "phylo" in cache; using the first, from namespace 'phyloseq'
```

```
## Also defined by 'tidytree'
```

```
## Found more than one class "phylo" in cache; using the first, from namespace 'phyloseq'
```

```
## Also defined by 'tidytree'
```

```
## Found more than one class "phylo" in cache; using the first, from namespace 'phyloseq'
```

```
## Also defined by 'tidytree'
```

```
## Found more than one class "phylo" in cache; using the first, from namespace 'phyloseq'
```

```
## Also defined by 'tidytree'
```

```
## Found more than one class "phylo" in cache; using the first, from namespace 'phyloseq'
```

```
## Also defined by 'tidytree'
```

```
## Found more than one class "phylo" in cache; using the first, from namespace 'phyloseq'
```

```
## Also defined by 'tidytree'
```

```
## Found more than one class "phylo" in cache; using the first, from namespace 'phyloseq'
```

```
## Also defined by 'tidytree'
```

```
## Found more than one class "phylo" in cache; using the first, from namespace 'phyloseq'
```

```
## Also defined by 'tidytree'
```

```
## Found more than one class "phylo" in cache; using the first, from namespace 'phyloseq'
```

```
## Also defined by 'tidytree'
```

```
## Found more than one class "phylo" in cache; using the first, from namespace 'phyloseq'
```

```
## Also defined by 'tidytree'
```

```
## Found more than one class "phylo" in cache; using the first, from namespace 'phyloseq'
```

```
## Also defined by 'tidytree'
```

```
## Found more than one class "phylo" in cache; using the first, from namespace 'phyloseq'
```

```
## Also defined by 'tidytree'
```

```
## Found more than one class "phylo" in cache; using the first, from namespace 'phyloseq'
```

```
## Also defined by 'tidytree'
```

```
## Found more than one class "phylo" in cache; using the first, from namespace 'phyloseq'
```

```
## Also defined by 'tidytree'
```

```
## Found more than one class "phylo" in cache; using the first, from namespace 'phyloseq'
```

```
## Also defined by 'tidytree'
```

```
## Found more than one class "phylo" in cache; using the first, from namespace 'phyloseq'
```

```
## Also defined by 'tidytree'
```

```
## Found more than one class "phylo" in cache; using the first, from namespace 'phyloseq'
```

```
## Also defined by 'tidytree'
```

```
## Found more than one class "phylo" in cache; using the first, from namespace 'phyloseq'
```

```
## Also defined by 'tidytree'
```

```
## Found more than one class "phylo" in cache; using the first, from namespace 'phyloseq'
```

```
## Also defined by 'tidytree'
```

```
## Found more than one class "phylo" in cache; using the first, from namespace 'phyloseq'
```

```
## Also defined by 'tidytree'
```

```
## Found more than one class "phylo" in cache; using the first, from namespace 'phyloseq'
```

```
## Also defined by 'tidytree'
```

```
## Found more than one class "phylo" in cache; using the first, from namespace 'phyloseq'
```

```
## Also defined by 'tidytree'
```

```
## Found more than one class "phylo" in cache; using the first, from namespace 'phyloseq'
```

```
## Also defined by 'tidytree'
```

```
## Found more than one class "phylo" in cache; using the first, from namespace 'phyloseq'
```

```
## Also defined by 'tidytree'
```

```
## Found more than one class "phylo" in cache; using the first, from namespace 'phyloseq'
```

```
## Also defined by 'tidytree'
```

```
## Found more than one class "phylo" in cache; using the first, from namespace 'phyloseq'
```

```
## Also defined by 'tidytree'
```

```
## Found more than one class "phylo" in cache; using the first, from namespace 'phyloseq'
```

```
## Also defined by 'tidytree'
```

```
## Found more than one class "phylo" in cache; using the first, from namespace 'phyloseq'
```

```
## Also defined by 'tidytree'
```

```
## Found more than one class "phylo" in cache; using the first, from namespace 'phyloseq'
```

```
## Also defined by 'tidytree'
```

```
## Found more than one class "phylo" in cache; using the first, from namespace 'phyloseq'
```

```
## Also defined by 'tidytree'
```

```
## Found more than one class "phylo" in cache; using the first, from namespace 'phyloseq'
```

```
## Also defined by 'tidytree'
```

```
## Found more than one class "phylo" in cache; using the first, from namespace 'phyloseq'
```

```
## Also defined by 'tidytree'
```

```
## Found more than one class "phylo" in cache; using the first, from namespace 'phyloseq'
```

```
## Also defined by 'tidytree'
```

```
## Found more than one class "phylo" in cache; using the first, from namespace 'phyloseq'
```

```
## Also defined by 'tidytree'
```

```
## Found more than one class "phylo" in cache; using the first, from namespace 'phyloseq'
```

```
## Also defined by 'tidytree'
```

```
## Found more than one class "phylo" in cache; using the first, from namespace 'phyloseq'
```

```
## Also defined by 'tidytree'
```

```
## Found more than one class "phylo" in cache; using the first, from namespace 'phyloseq'
```

```
## Also defined by 'tidytree'
```

```
## Found more than one class "phylo" in cache; using the first, from namespace 'phyloseq'
```

```
## Also defined by 'tidytree'
```

```
## Found more than one class "phylo" in cache; using the first, from namespace 'phyloseq'
```

```
## Also defined by 'tidytree'
```

```
## Found more than one class "phylo" in cache; using the first, from namespace 'phyloseq'
```

```
## Also defined by 'tidytree'
```

```
## Found more than one class "phylo" in cache; using the first, from namespace 'phyloseq'
```

```
## Also defined by 'tidytree'
```

```
## Found more than one class "phylo" in cache; using the first, from namespace 'phyloseq'
```

```
## Also defined by 'tidytree'
```

```
## Found more than one class "phylo" in cache; using the first, from namespace 'phyloseq'
```

```
## Also defined by 'tidytree'
```

```
## Found more than one class "phylo" in cache; using the first, from namespace 'phyloseq'
```

```
## Also defined by 'tidytree'
```

```
## Found more than one class "phylo" in cache; using the first, from namespace 'phyloseq'
```

```
## Also defined by 'tidytree'
```

```
## Found more than one class "phylo" in cache; using the first, from namespace 'phyloseq'
```

```
## Also defined by 'tidytree'
```

```
## Found more than one class "phylo" in cache; using the first, from namespace 'phyloseq'
```

```
## Also defined by 'tidytree'
```

```
## Found more than one class "phylo" in cache; using the first, from namespace 'phyloseq'
```

```
## Also defined by 'tidytree'
```

```
## Found more than one class "phylo" in cache; using the first, from namespace 'phyloseq'
```

```
## Also defined by 'tidytree'
```

```
## Found more than one class "phylo" in cache; using the first, from namespace 'phyloseq'
```

```
## Also defined by 'tidytree'
```

```
## Found more than one class "phylo" in cache; using the first, from namespace 'phyloseq'
```

```
## Also defined by 'tidytree'
```

```
## Found more than one class "phylo" in cache; using the first, from namespace 'phyloseq'
```

```
## Also defined by 'tidytree'
```

```
## Found more than one class "phylo" in cache; using the first, from namespace 'phyloseq'
```

```
## Also defined by 'tidytree'
```

```
## Found more than one class "phylo" in cache; using the first, from namespace 'phyloseq'
```

```
## Also defined by 'tidytree'
```

```
## Found more than one class "phylo" in cache; using the first, from namespace 'phyloseq'
```

```
## Also defined by 'tidytree'
```

```
## Found more than one class "phylo" in cache; using the first, from namespace 'phyloseq'
```

```
## Also defined by 'tidytree'
```

```
## Found more than one class "phylo" in cache; using the first, from namespace 'phyloseq'
```

```
## Also defined by 'tidytree'
```

```
## Found more than one class "phylo" in cache; using the first, from namespace 'phyloseq'
```

```
## Also defined by 'tidytree'
```

```
## Found more than one class "phylo" in cache; using the first, from namespace 'phyloseq'
```

```
## Also defined by 'tidytree'
```

```
## Found more than one class "phylo" in cache; using the first, from namespace 'phyloseq'
```

```
## Also defined by 'tidytree'
```

```
## Found more than one class "phylo" in cache; using the first, from namespace 'phyloseq'
```

```
## Also defined by 'tidytree'
```

```
## Found more than one class "phylo" in cache; using the first, from namespace 'phyloseq'
```

```
## Also defined by 'tidytree'
```

```
## Found more than one class "phylo" in cache; using the first, from namespace 'phyloseq'
```

```
## Also defined by 'tidytree'
```

```
## Found more than one class "phylo" in cache; using the first, from namespace 'phyloseq'
```

```
## Also defined by 'tidytree'
```

```
## Found more than one class "phylo" in cache; using the first, from namespace 'phyloseq'
```

```
## Also defined by 'tidytree'
```

```
## Found more than one class "phylo" in cache; using the first, from namespace 'phyloseq'
```

```
## Also defined by 'tidytree'
```

```
## Found more than one class "phylo" in cache; using the first, from namespace 'phyloseq'
```

```
## Also defined by 'tidytree'
```

```
## Found more than one class "phylo" in cache; using the first, from namespace 'phyloseq'
```

```
## Also defined by 'tidytree'
```

```
## Found more than one class "phylo" in cache; using the first, from namespace 'phyloseq'
```

```
## Also defined by 'tidytree'
```

```
## Found more than one class "phylo" in cache; using the first, from namespace 'phyloseq'
```

```
## Also defined by 'tidytree'
```

```
## Found more than one class "phylo" in cache; using the first, from namespace 'phyloseq'
```

```
## Also defined by 'tidytree'
```

```
## Found more than one class "phylo" in cache; using the first, from namespace 'phyloseq'
```

```
## Also defined by 'tidytree'
```

```
## Found more than one class "phylo" in cache; using the first, from namespace 'phyloseq'
```

```
## Also defined by 'tidytree'
```

```
## Found more than one class "phylo" in cache; using the first, from namespace 'phyloseq'
```

```
## Also defined by 'tidytree'
```

```
## Found more than one class "phylo" in cache; using the first, from namespace 'phyloseq'
```

```
## Also defined by 'tidytree'
```

```
## Found more than one class "phylo" in cache; using the first, from namespace 'phyloseq'
```

```
## Also defined by 'tidytree'
```

```
## Found more than one class "phylo" in cache; using the first, from namespace 'phyloseq'
```

```
## Also defined by 'tidytree'
```

```
## Found more than one class "phylo" in cache; using the first, from namespace 'phyloseq'
```

```
## Also defined by 'tidytree'
```

```
## Found more than one class "phylo" in cache; using the first, from namespace 'phyloseq'
```

```
## Also defined by 'tidytree'
```

```
## Found more than one class "phylo" in cache; using the first, from namespace 'phyloseq'
```

```
## Also defined by 'tidytree'
```

```
## Found more than one class "phylo" in cache; using the first, from namespace 'phyloseq'
```

```
## Also defined by 'tidytree'
```

```
## Found more than one class "phylo" in cache; using the first, from namespace 'phyloseq'
```

```
## Also defined by 'tidytree'
```

```
## Found more than one class "phylo" in cache; using the first, from namespace 'phyloseq'
```

```
## Also defined by 'tidytree'
```

```
## Found more than one class "phylo" in cache; using the first, from namespace 'phyloseq'
```

```
## Also defined by 'tidytree'
```

```
## Found more than one class "phylo" in cache; using the first, from namespace 'phyloseq'
```

```
## Also defined by 'tidytree'
```

```
## Found more than one class "phylo" in cache; using the first, from namespace 'phyloseq'
```

```
## Also defined by 'tidytree'
```

```
## Found more than one class "phylo" in cache; using the first, from namespace 'phyloseq'
```

```
## Also defined by 'tidytree'
```

```
## Found more than one class "phylo" in cache; using the first, from namespace 'phyloseq'
```

```
## Also defined by 'tidytree'
```

```
## Found more than one class "phylo" in cache; using the first, from namespace 'phyloseq'
```

```
## Also defined by 'tidytree'
```

```
## Found more than one class "phylo" in cache; using the first, from namespace 'phyloseq'
```

```
## Also defined by 'tidytree'
```

```
## Found more than one class "phylo" in cache; using the first, from namespace 'phyloseq'
```

```
## Also defined by 'tidytree'
```

```
## Found more than one class "phylo" in cache; using the first, from namespace 'phyloseq'
```

```
## Also defined by 'tidytree'
```

```
## Found more than one class "phylo" in cache; using the first, from namespace 'phyloseq'
```

```
## Also defined by 'tidytree'
```

```
## Found more than one class "phylo" in cache; using the first, from namespace 'phyloseq'
```

```
## Also defined by 'tidytree'
```

```
## Found more than one class "phylo" in cache; using the first, from namespace 'phyloseq'
```

```
## Also defined by 'tidytree'
```

```
## Found more than one class "phylo" in cache; using the first, from namespace 'phyloseq'
```

```
## Also defined by 'tidytree'
```

```
## Found more than one class "phylo" in cache; using the first, from namespace 'phyloseq'
```

```
## Also defined by 'tidytree'
```

```
## Found more than one class "phylo" in cache; using the first, from namespace 'phyloseq'
```

```
## Also defined by 'tidytree'
```

```
## Found more than one class "phylo" in cache; using the first, from namespace 'phyloseq'
```

```
## Also defined by 'tidytree'
```

```
## Found more than one class "phylo" in cache; using the first, from namespace 'phyloseq'
```

```
## Also defined by 'tidytree'
```

```
## Found more than one class "phylo" in cache; using the first, from namespace 'phyloseq'
```

```
## Also defined by 'tidytree'
```

```
## Found more than one class "phylo" in cache; using the first, from namespace 'phyloseq'
```

```
## Also defined by 'tidytree'
```

```
## Found more than one class "phylo" in cache; using the first, from namespace 'phyloseq'
```

```
## Also defined by 'tidytree'
```

```
## Found more than one class "phylo" in cache; using the first, from namespace 'phyloseq'
```

```
## Also defined by 'tidytree'
```

```
## Found more than one class "phylo" in cache; using the first, from namespace 'phyloseq'
```

```
## Also defined by 'tidytree'
```

```
## Found more than one class "phylo" in cache; using the first, from namespace 'phyloseq'
```

```
## Also defined by 'tidytree'
```

```
## Found more than one class "phylo" in cache; using the first, from namespace 'phyloseq'
```

```
## Also defined by 'tidytree'
```

```
## Found more than one class "phylo" in cache; using the first, from namespace 'phyloseq'
```

```
## Also defined by 'tidytree'
```

```
## Found more than one class "phylo" in cache; using the first, from namespace 'phyloseq'
```

```
## Also defined by 'tidytree'
```

```
## Found more than one class "phylo" in cache; using the first, from namespace 'phyloseq'
```

```
## Also defined by 'tidytree'
```

```
## Found more than one class "phylo" in cache; using the first, from namespace 'phyloseq'
```

```
## Also defined by 'tidytree'
```

```
## Found more than one class "phylo" in cache; using the first, from namespace 'phyloseq'
```

```
## Also defined by 'tidytree'
```

```
## Found more than one class "phylo" in cache; using the first, from namespace 'phyloseq'
```

```
## Also defined by 'tidytree'
```

```
## Found more than one class "phylo" in cache; using the first, from namespace 'phyloseq'
```

```
## Also defined by 'tidytree'
```

```
## Found more than one class "phylo" in cache; using the first, from namespace 'phyloseq'
```

```
## Also defined by 'tidytree'
```

```
## Found more than one class "phylo" in cache; using the first, from namespace 'phyloseq'
```

```
## Also defined by 'tidytree'
```

```
## Found more than one class "phylo" in cache; using the first, from namespace 'phyloseq'
```

```
## Also defined by 'tidytree'
```

```
## Found more than one class "phylo" in cache; using the first, from namespace 'phyloseq'
```

```
## Also defined by 'tidytree'
```

```
## Found more than one class "phylo" in cache; using the first, from namespace 'phyloseq'
```

```
## Also defined by 'tidytree'
```

```
## Found more than one class "phylo" in cache; using the first, from namespace 'phyloseq'
```

```
## Also defined by 'tidytree'
```

```
## Found more than one class "phylo" in cache; using the first, from namespace 'phyloseq'
```

```
## Also defined by 'tidytree'
```

```
## Found more than one class "phylo" in cache; using the first, from namespace 'phyloseq'
```

```
## Also defined by 'tidytree'
```

```
## Found more than one class "phylo" in cache; using the first, from namespace 'phyloseq'
```

```
## Also defined by 'tidytree'
```

```
## Found more than one class "phylo" in cache; using the first, from namespace 'phyloseq'
```

```
## Also defined by 'tidytree'
```

```
## Found more than one class "phylo" in cache; using the first, from namespace 'phyloseq'
```

```
## Also defined by 'tidytree'
```

```
## Found more than one class "phylo" in cache; using the first, from namespace 'phyloseq'
```

```
## Also defined by 'tidytree'
```

```
## Found more than one class "phylo" in cache; using the first, from namespace 'phyloseq'
```

```
## Also defined by 'tidytree'
```

```
## Found more than one class "phylo" in cache; using the first, from namespace 'phyloseq'
```

```
## Also defined by 'tidytree'
```

```
## Found more than one class "phylo" in cache; using the first, from namespace 'phyloseq'
```

```
## Also defined by 'tidytree'
```

```
## Found more than one class "phylo" in cache; using the first, from namespace 'phyloseq'
```

```
## Also defined by 'tidytree'
```

```
## Found more than one class "phylo" in cache; using the first, from namespace 'phyloseq'
```

```
## Also defined by 'tidytree'
```

```
## Found more than one class "phylo" in cache; using the first, from namespace 'phyloseq'
```

```
## Also defined by 'tidytree'
```

```
## Found more than one class "phylo" in cache; using the first, from namespace 'phyloseq'
```

```
## Also defined by 'tidytree'
```

```
## Found more than one class "phylo" in cache; using the first, from namespace 'phyloseq'
```

```
## Also defined by 'tidytree'
```

```
## Found more than one class "phylo" in cache; using the first, from namespace 'phyloseq'
```

```
## Also defined by 'tidytree'
```

```
## Found more than one class "phylo" in cache; using the first, from namespace 'phyloseq'
```

```
## Also defined by 'tidytree'
```

```
## Found more than one class "phylo" in cache; using the first, from namespace 'phyloseq'
```

```
## Also defined by 'tidytree'
```

```
## Found more than one class "phylo" in cache; using the first, from namespace 'phyloseq'
```

```
## Also defined by 'tidytree'
```

```
## Found more than one class "phylo" in cache; using the first, from namespace 'phyloseq'
```

```
## Also defined by 'tidytree'
```

```
## Found more than one class "phylo" in cache; using the first, from namespace 'phyloseq'
```

```
## Also defined by 'tidytree'
```

```
## Found more than one class "phylo" in cache; using the first, from namespace 'phyloseq'
```

```
## Also defined by 'tidytree'
```

```
## Found more than one class "phylo" in cache; using the first, from namespace 'phyloseq'
```

```
## Also defined by 'tidytree'
```

```
## Found more than one class "phylo" in cache; using the first, from namespace 'phyloseq'
```

```
## Also defined by 'tidytree'
```

```
## Found more than one class "phylo" in cache; using the first, from namespace 'phyloseq'
```

```
## Also defined by 'tidytree'
```

```
## Found more than one class "phylo" in cache; using the first, from namespace 'phyloseq'
```

```
## Also defined by 'tidytree'
```

```
## Found more than one class "phylo" in cache; using the first, from namespace 'phyloseq'
```

```
## Also defined by 'tidytree'
```

```
## Found more than one class "phylo" in cache; using the first, from namespace 'phyloseq'
```

```
## Also defined by 'tidytree'
```

```
## Found more than one class "phylo" in cache; using the first, from namespace 'phyloseq'
```

```
## Also defined by 'tidytree'
```

```
## Found more than one class "phylo" in cache; using the first, from namespace 'phyloseq'
```

```
## Also defined by 'tidytree'
```

```
## Found more than one class "phylo" in cache; using the first, from namespace 'phyloseq'
```

```
## Also defined by 'tidytree'
```

```
## Found more than one class "phylo" in cache; using the first, from namespace 'phyloseq'
```

```
## Also defined by 'tidytree'
```

```
## Found more than one class "phylo" in cache; using the first, from namespace 'phyloseq'
```

```
## Also defined by 'tidytree'
```

```
## Found more than one class "phylo" in cache; using the first, from namespace 'phyloseq'
```

```
## Also defined by 'tidytree'
```

```
## Found more than one class "phylo" in cache; using the first, from namespace 'phyloseq'
```

```
## Also defined by 'tidytree'
```

```
## Found more than one class "phylo" in cache; using the first, from namespace 'phyloseq'
```

```
## Also defined by 'tidytree'
```

```
## Found more than one class "phylo" in cache; using the first, from namespace 'phyloseq'
```

```
## Also defined by 'tidytree'
```

```
## Found more than one class "phylo" in cache; using the first, from namespace 'phyloseq'
```

```
## Also defined by 'tidytree'
```

```
## Found more than one class "phylo" in cache; using the first, from namespace 'phyloseq'
```

```
## Also defined by 'tidytree'
```

```
## Found more than one class "phylo" in cache; using the first, from namespace 'phyloseq'
```

```
## Also defined by 'tidytree'
```

```
## Found more than one class "phylo" in cache; using the first, from namespace 'phyloseq'
```

```
## Also defined by 'tidytree'
```

```
## Found more than one class "phylo" in cache; using the first, from namespace 'phyloseq'
```

```
## Also defined by 'tidytree'
```

```
## Found more than one class "phylo" in cache; using the first, from namespace 'phyloseq'
```

```
## Also defined by 'tidytree'
```

```
## Found more than one class "phylo" in cache; using the first, from namespace 'phyloseq'
```

```
## Also defined by 'tidytree'
```

```
## Found more than one class "phylo" in cache; using the first, from namespace 'phyloseq'
```

```
## Also defined by 'tidytree'
```

```
## Found more than one class "phylo" in cache; using the first, from namespace 'phyloseq'
```

```
## Also defined by 'tidytree'
```

```
## Found more than one class "phylo" in cache; using the first, from namespace 'phyloseq'
```

```
## Also defined by 'tidytree'
```

```
## Found more than one class "phylo" in cache; using the first, from namespace 'phyloseq'
```

```
## Also defined by 'tidytree'
```

```
## Found more than one class "phylo" in cache; using the first, from namespace 'phyloseq'
```

```
## Also defined by 'tidytree'
```

```
## Found more than one class "phylo" in cache; using the first, from namespace 'phyloseq'
```

```
## Also defined by 'tidytree'
```

```
## Found more than one class "phylo" in cache; using the first, from namespace 'phyloseq'
```

```
## Also defined by 'tidytree'
```

```
## Found more than one class "phylo" in cache; using the first, from namespace 'phyloseq'
```

```
## Also defined by 'tidytree'
```

```
## Found more than one class "phylo" in cache; using the first, from namespace 'phyloseq'
```

```
## Also defined by 'tidytree'
```

```
## Found more than one class "phylo" in cache; using the first, from namespace 'phyloseq'
```

```
## Also defined by 'tidytree'
```

```
## Found more than one class "phylo" in cache; using the first, from namespace 'phyloseq'
```

```
## Also defined by 'tidytree'
```

```
## Found more than one class "phylo" in cache; using the first, from namespace 'phyloseq'
```

```
## Also defined by 'tidytree'
```

```
## Found more than one class "phylo" in cache; using the first, from namespace 'phyloseq'
```

```
## Also defined by 'tidytree'
```

```
## Found more than one class "phylo" in cache; using the first, from namespace 'phyloseq'
```

```
## Also defined by 'tidytree'
```

```
## Found more than one class "phylo" in cache; using the first, from namespace 'phyloseq'
```

```
## Also defined by 'tidytree'
```

```
## Found more than one class "phylo" in cache; using the first, from namespace 'phyloseq'
```

```
## Also defined by 'tidytree'
```

```
## Found more than one class "phylo" in cache; using the first, from namespace 'phyloseq'
```

```
## Also defined by 'tidytree'
```

```
## Found more than one class "phylo" in cache; using the first, from namespace 'phyloseq'
```

```
## Also defined by 'tidytree'
```

```
## Found more than one class "phylo" in cache; using the first, from namespace 'phyloseq'
```

```
## Also defined by 'tidytree'
```

```
## Found more than one class "phylo" in cache; using the first, from namespace 'phyloseq'
```

```
## Also defined by 'tidytree'
```

```
## Found more than one class "phylo" in cache; using the first, from namespace 'phyloseq'
```

```
## Also defined by 'tidytree'
```

```
## Found more than one class "phylo" in cache; using the first, from namespace 'phyloseq'
```

```
## Also defined by 'tidytree'
```

```
## Found more than one class "phylo" in cache; using the first, from namespace 'phyloseq'
```

```
## Also defined by 'tidytree'
```

```
## Found more than one class "phylo" in cache; using the first, from namespace 'phyloseq'
```

```
## Also defined by 'tidytree'
```

```
## Found more than one class "phylo" in cache; using the first, from namespace 'phyloseq'
```

```
## Also defined by 'tidytree'
```

```
## Found more than one class "phylo" in cache; using the first, from namespace 'phyloseq'
```

```
## Also defined by 'tidytree'
```

```
## Found more than one class "phylo" in cache; using the first, from namespace 'phyloseq'
```

```
## Also defined by 'tidytree'
```

```
## Found more than one class "phylo" in cache; using the first, from namespace 'phyloseq'
```

```
## Also defined by 'tidytree'
```

```
## Found more than one class "phylo" in cache; using the first, from namespace 'phyloseq'
```

```
## Also defined by 'tidytree'
```

```
## Found more than one class "phylo" in cache; using the first, from namespace 'phyloseq'
```

```
## Also defined by 'tidytree'
```

```
## Found more than one class "phylo" in cache; using the first, from namespace 'phyloseq'
```

```
## Also defined by 'tidytree'
```

```
## Found more than one class "phylo" in cache; using the first, from namespace 'phyloseq'
```

```
## Also defined by 'tidytree'
```

```
## Found more than one class "phylo" in cache; using the first, from namespace 'phyloseq'
```

```
## Also defined by 'tidytree'
```

```
## Found more than one class "phylo" in cache; using the first, from namespace 'phyloseq'
```

```
## Also defined by 'tidytree'
```

```
## Found more than one class "phylo" in cache; using the first, from namespace 'phyloseq'
```

```
## Also defined by 'tidytree'
```

```
## Found more than one class "phylo" in cache; using the first, from namespace 'phyloseq'
```

```
## Also defined by 'tidytree'
```

```
## Found more than one class "phylo" in cache; using the first, from namespace 'phyloseq'
```

```
## Also defined by 'tidytree'
```

```
## Found more than one class "phylo" in cache; using the first, from namespace 'phyloseq'
```

```
## Also defined by 'tidytree'
```

```
## Found more than one class "phylo" in cache; using the first, from namespace 'phyloseq'
```

```
## Also defined by 'tidytree'
```

```
## Found more than one class "phylo" in cache; using the first, from namespace 'phyloseq'
```

```
## Also defined by 'tidytree'
```

```
## Found more than one class "phylo" in cache; using the first, from namespace 'phyloseq'
```

```
## Also defined by 'tidytree'
```

```
## Found more than one class "phylo" in cache; using the first, from namespace 'phyloseq'
```

```
## Also defined by 'tidytree'
```

```
## Found more than one class "phylo" in cache; using the first, from namespace 'phyloseq'
```

```
## Also defined by 'tidytree'
```

```
## Found more than one class "phylo" in cache; using the first, from namespace 'phyloseq'
```

```
## Also defined by 'tidytree'
```

```
## Found more than one class "phylo" in cache; using the first, from namespace 'phyloseq'
```

```
## Also defined by 'tidytree'
```

```
## Found more than one class "phylo" in cache; using the first, from namespace 'phyloseq'
```

```
## Also defined by 'tidytree'
```

```
## Found more than one class "phylo" in cache; using the first, from namespace 'phyloseq'
```

```
## Also defined by 'tidytree'
```

```
## Found more than one class "phylo" in cache; using the first, from namespace 'phyloseq'
```

```
## Also defined by 'tidytree'
```

```
## Found more than one class "phylo" in cache; using the first, from namespace 'phyloseq'
```

```
## Also defined by 'tidytree'
```

```
## Found more than one class "phylo" in cache; using the first, from namespace 'phyloseq'
```

```
## Also defined by 'tidytree'
```

```
## Found more than one class "phylo" in cache; using the first, from namespace 'phyloseq'
```

```
## Also defined by 'tidytree'
```

```
## Found more than one class "phylo" in cache; using the first, from namespace 'phyloseq'
```

```
## Also defined by 'tidytree'
```

```
## Found more than one class "phylo" in cache; using the first, from namespace 'phyloseq'
```

```
## Also defined by 'tidytree'
```

```
## Found more than one class "phylo" in cache; using the first, from namespace 'phyloseq'
```

```
## Also defined by 'tidytree'
```

```
## Found more than one class "phylo" in cache; using the first, from namespace 'phyloseq'
```

```
## Also defined by 'tidytree'
```

```
## Found more than one class "phylo" in cache; using the first, from namespace 'phyloseq'
```

```
## Also defined by 'tidytree'
```

```
## Found more than one class "phylo" in cache; using the first, from namespace 'phyloseq'
```

```
## Also defined by 'tidytree'
```

```
## Found more than one class "phylo" in cache; using the first, from namespace 'phyloseq'
```

```
## Also defined by 'tidytree'
```

```
## Found more than one class "phylo" in cache; using the first, from namespace 'phyloseq'
```

```
## Also defined by 'tidytree'
```

```
## Found more than one class "phylo" in cache; using the first, from namespace 'phyloseq'
```

```
## Also defined by 'tidytree'
```

```
## Found more than one class "phylo" in cache; using the first, from namespace 'phyloseq'
```

```
## Also defined by 'tidytree'
```

```
## Found more than one class "phylo" in cache; using the first, from namespace 'phyloseq'
```

```
## Also defined by 'tidytree'
```

```
## Found more than one class "phylo" in cache; using the first, from namespace 'phyloseq'
```

```
## Also defined by 'tidytree'
```

```
## Found more than one class "phylo" in cache; using the first, from namespace 'phyloseq'
```

```
## Also defined by 'tidytree'
```

```
## Found more than one class "phylo" in cache; using the first, from namespace 'phyloseq'
```

```
## Also defined by 'tidytree'
```

```
## Found more than one class "phylo" in cache; using the first, from namespace 'phyloseq'
```

```
## Also defined by 'tidytree'
```

```
## Found more than one class "phylo" in cache; using the first, from namespace 'phyloseq'
```

```
## Also defined by 'tidytree'
```

```
## Found more than one class "phylo" in cache; using the first, from namespace 'phyloseq'
```

```
## Also defined by 'tidytree'
```

```
## Found more than one class "phylo" in cache; using the first, from namespace 'phyloseq'
```

```
## Also defined by 'tidytree'
```

```
## Found more than one class "phylo" in cache; using the first, from namespace 'phyloseq'
```

```
## Also defined by 'tidytree'
```

```
## Found more than one class "phylo" in cache; using the first, from namespace 'phyloseq'
```

```
## Also defined by 'tidytree'
```

```
## Found more than one class "phylo" in cache; using the first, from namespace 'phyloseq'
```

```
## Also defined by 'tidytree'
```

```
## Found more than one class "phylo" in cache; using the first, from namespace 'phyloseq'
```

```
## Also defined by 'tidytree'
```

```
## Found more than one class "phylo" in cache; using the first, from namespace 'phyloseq'
```

```
## Also defined by 'tidytree'
```

```
## Found more than one class "phylo" in cache; using the first, from namespace 'phyloseq'
```

```
## Also defined by 'tidytree'
```

```
## Found more than one class "phylo" in cache; using the first, from namespace 'phyloseq'
```

```
## Also defined by 'tidytree'
```

```
## Found more than one class "phylo" in cache; using the first, from namespace 'phyloseq'
```

```
## Also defined by 'tidytree'
```

```
## Found more than one class "phylo" in cache; using the first, from namespace 'phyloseq'
```

```
## Also defined by 'tidytree'
```

```
## Found more than one class "phylo" in cache; using the first, from namespace 'phyloseq'
```

```
## Also defined by 'tidytree'
```

```
## Found more than one class "phylo" in cache; using the first, from namespace 'phyloseq'
```

```
## Also defined by 'tidytree'
```

```
## Found more than one class "phylo" in cache; using the first, from namespace 'phyloseq'
```

```
## Also defined by 'tidytree'
```

```
## Found more than one class "phylo" in cache; using the first, from namespace 'phyloseq'
```

```
## Also defined by 'tidytree'
```

```
## Found more than one class "phylo" in cache; using the first, from namespace 'phyloseq'
```

```
## Also defined by 'tidytree'
```

```
## Found more than one class "phylo" in cache; using the first, from namespace 'phyloseq'
```

```
## Also defined by 'tidytree'
```

```
## Found more than one class "phylo" in cache; using the first, from namespace 'phyloseq'
```

```
## Also defined by 'tidytree'
```

```
## Found more than one class "phylo" in cache; using the first, from namespace 'phyloseq'
```

```
## Also defined by 'tidytree'
```

```
## Found more than one class "phylo" in cache; using the first, from namespace 'phyloseq'
```

```
## Also defined by 'tidytree'
```

```
## Found more than one class "phylo" in cache; using the first, from namespace 'phyloseq'
```

```
## Also defined by 'tidytree'
```

```
## Found more than one class "phylo" in cache; using the first, from namespace 'phyloseq'
```

```
## Also defined by 'tidytree'
```

```
## Found more than one class "phylo" in cache; using the first, from namespace 'phyloseq'
```

```
## Also defined by 'tidytree'
```

```
## Found more than one class "phylo" in cache; using the first, from namespace 'phyloseq'
```

```
## Also defined by 'tidytree'
```

```
## Found more than one class "phylo" in cache; using the first, from namespace 'phyloseq'
```

```
## Also defined by 'tidytree'
```

```
## Found more than one class "phylo" in cache; using the first, from namespace 'phyloseq'
```

```
## Also defined by 'tidytree'
```

```
## Found more than one class "phylo" in cache; using the first, from namespace 'phyloseq'
```

```
## Also defined by 'tidytree'
```

```
## Found more than one class "phylo" in cache; using the first, from namespace 'phyloseq'
```

```
## Also defined by 'tidytree'
```

```
## Found more than one class "phylo" in cache; using the first, from namespace 'phyloseq'
```

```
## Also defined by 'tidytree'
```

```
## Found more than one class "phylo" in cache; using the first, from namespace 'phyloseq'
```

```
## Also defined by 'tidytree'
```

```
## Found more than one class "phylo" in cache; using the first, from namespace 'phyloseq'
```

```
## Also defined by 'tidytree'
```

```
## Found more than one class "phylo" in cache; using the first, from namespace 'phyloseq'
```

```
## Also defined by 'tidytree'
```

```
## Found more than one class "phylo" in cache; using the first, from namespace 'phyloseq'
```

```
## Also defined by 'tidytree'
```

```
## Found more than one class "phylo" in cache; using the first, from namespace 'phyloseq'
```

```
## Also defined by 'tidytree'
```

```
## Found more than one class "phylo" in cache; using the first, from namespace 'phyloseq'
```

```
## Also defined by 'tidytree'
```

```
## Found more than one class "phylo" in cache; using the first, from namespace 'phyloseq'
```

```
## Also defined by 'tidytree'
```

```
## Found more than one class "phylo" in cache; using the first, from namespace 'phyloseq'
```

```
## Also defined by 'tidytree'
```

```
## Found more than one class "phylo" in cache; using the first, from namespace 'phyloseq'
```

```
## Also defined by 'tidytree'
```

```
## Found more than one class "phylo" in cache; using the first, from namespace 'phyloseq'
```

```
## Also defined by 'tidytree'
```

```
## Found more than one class "phylo" in cache; using the first, from namespace 'phyloseq'
```

```
## Also defined by 'tidytree'
```

```
## Found more than one class "phylo" in cache; using the first, from namespace 'phyloseq'
```

```
## Also defined by 'tidytree'
```

```
## Found more than one class "phylo" in cache; using the first, from namespace 'phyloseq'
```

```
## Also defined by 'tidytree'
```

```
## Found more than one class "phylo" in cache; using the first, from namespace 'phyloseq'
```

```
## Also defined by 'tidytree'
```

```
## Found more than one class "phylo" in cache; using the first, from namespace 'phyloseq'
```

```
## Also defined by 'tidytree'
```

```
## Found more than one class "phylo" in cache; using the first, from namespace 'phyloseq'
```

```
## Also defined by 'tidytree'
```

```
## Found more than one class "phylo" in cache; using the first, from namespace 'phyloseq'
```

```
## Also defined by 'tidytree'
```

```
## Found more than one class "phylo" in cache; using the first, from namespace 'phyloseq'
```

```
## Also defined by 'tidytree'
```

```
## Found more than one class "phylo" in cache; using the first, from namespace 'phyloseq'
```

```
## Also defined by 'tidytree'
```

```
## Found more than one class "phylo" in cache; using the first, from namespace 'phyloseq'
```

```
## Also defined by 'tidytree'
```

```
## Found more than one class "phylo" in cache; using the first, from namespace 'phyloseq'
```

```
## Also defined by 'tidytree'
```

```
## Found more than one class "phylo" in cache; using the first, from namespace 'phyloseq'
```

```
## Also defined by 'tidytree'
```

```
## Found more than one class "phylo" in cache; using the first, from namespace 'phyloseq'
```

```
## Also defined by 'tidytree'
```

```
## Found more than one class "phylo" in cache; using the first, from namespace 'phyloseq'
```

```
## Also defined by 'tidytree'
```

```
## Found more than one class "phylo" in cache; using the first, from namespace 'phyloseq'
```

```
## Also defined by 'tidytree'
```

```
## Found more than one class "phylo" in cache; using the first, from namespace 'phyloseq'
```

```
## Also defined by 'tidytree'
```

```
## Found more than one class "phylo" in cache; using the first, from namespace 'phyloseq'
```

```
## Also defined by 'tidytree'
```

```
## Found more than one class "phylo" in cache; using the first, from namespace 'phyloseq'
```

```
## Also defined by 'tidytree'
```

```
## Found more than one class "phylo" in cache; using the first, from namespace 'phyloseq'
```

```
## Also defined by 'tidytree'
```

```
## Found more than one class "phylo" in cache; using the first, from namespace 'phyloseq'
```

```
## Also defined by 'tidytree'
```

```
## Found more than one class "phylo" in cache; using the first, from namespace 'phyloseq'
```

```
## Also defined by 'tidytree'
```

```
## Found more than one class "phylo" in cache; using the first, from namespace 'phyloseq'
```

```
## Also defined by 'tidytree'
```

```
## Found more than one class "phylo" in cache; using the first, from namespace 'phyloseq'
```

```
## Also defined by 'tidytree'
```

```
## Found more than one class "phylo" in cache; using the first, from namespace 'phyloseq'
```

```
## Also defined by 'tidytree'
```

```
## Found more than one class "phylo" in cache; using the first, from namespace 'phyloseq'
```

```
## Also defined by 'tidytree'
```

```
## Found more than one class "phylo" in cache; using the first, from namespace 'phyloseq'
```

```
## Also defined by 'tidytree'
```

```
## Found more than one class "phylo" in cache; using the first, from namespace 'phyloseq'
```

```
## Also defined by 'tidytree'
```

```
## Found more than one class "phylo" in cache; using the first, from namespace 'phyloseq'
```

```
## Also defined by 'tidytree'
```

```
## Found more than one class "phylo" in cache; using the first, from namespace 'phyloseq'
```

```
## Also defined by 'tidytree'
```

```
## Found more than one class "phylo" in cache; using the first, from namespace 'phyloseq'
```

```
## Also defined by 'tidytree'
```

```
## Found more than one class "phylo" in cache; using the first, from namespace 'phyloseq'
```

```
## Also defined by 'tidytree'
```

```
## Found more than one class "phylo" in cache; using the first, from namespace 'phyloseq'
```

```
## Also defined by 'tidytree'
```

```
## Found more than one class "phylo" in cache; using the first, from namespace 'phyloseq'
```

```
## Also defined by 'tidytree'
```

```
## Found more than one class "phylo" in cache; using the first, from namespace 'phyloseq'
```

```
## Also defined by 'tidytree'
```

```
## Found more than one class "phylo" in cache; using the first, from namespace 'phyloseq'
```

```
## Also defined by 'tidytree'
```

```
## Found more than one class "phylo" in cache; using the first, from namespace 'phyloseq'
```

```
## Also defined by 'tidytree'
```

```
## Found more than one class "phylo" in cache; using the first, from namespace 'phyloseq'
```

```
## Also defined by 'tidytree'
```

```
## Found more than one class "phylo" in cache; using the first, from namespace 'phyloseq'
```

```
## Also defined by 'tidytree'
```

```
## Found more than one class "phylo" in cache; using the first, from namespace 'phyloseq'
```

```
## Also defined by 'tidytree'
```

```
## Found more than one class "phylo" in cache; using the first, from namespace 'phyloseq'
```

```
## Also defined by 'tidytree'
```

```
## Found more than one class "phylo" in cache; using the first, from namespace 'phyloseq'
```

```
## Also defined by 'tidytree'
```

```
## Found more than one class "phylo" in cache; using the first, from namespace 'phyloseq'
```

```
## Also defined by 'tidytree'
```

```
## Found more than one class "phylo" in cache; using the first, from namespace 'phyloseq'
```

```
## Also defined by 'tidytree'
```

```
## Found more than one class "phylo" in cache; using the first, from namespace 'phyloseq'
```

```
## Also defined by 'tidytree'
```

```
## Found more than one class "phylo" in cache; using the first, from namespace 'phyloseq'
```

```
## Also defined by 'tidytree'
```

```
## Found more than one class "phylo" in cache; using the first, from namespace 'phyloseq'
```

```
## Also defined by 'tidytree'
```

```
## Found more than one class "phylo" in cache; using the first, from namespace 'phyloseq'
```

```
## Also defined by 'tidytree'
```

```
## Found more than one class "phylo" in cache; using the first, from namespace 'phyloseq'
```

```
## Also defined by 'tidytree'
```

```
## Found more than one class "phylo" in cache; using the first, from namespace 'phyloseq'
```

```
## Also defined by 'tidytree'
```

```
## Found more than one class "phylo" in cache; using the first, from namespace 'phyloseq'
```

```
## Also defined by 'tidytree'
```

```
## Found more than one class "phylo" in cache; using the first, from namespace 'phyloseq'
```

```
## Also defined by 'tidytree'
```

```
## Found more than one class "phylo" in cache; using the first, from namespace 'phyloseq'
```

```
## Also defined by 'tidytree'
```

```
## Found more than one class "phylo" in cache; using the first, from namespace 'phyloseq'
```

```
## Also defined by 'tidytree'
```

```
## Found more than one class "phylo" in cache; using the first, from namespace 'phyloseq'
```

```
## Also defined by 'tidytree'
```

```
## Found more than one class "phylo" in cache; using the first, from namespace 'phyloseq'
```

```
## Also defined by 'tidytree'
```

```
## Found more than one class "phylo" in cache; using the first, from namespace 'phyloseq'
```

```
## Also defined by 'tidytree'
```

```
## Found more than one class "phylo" in cache; using the first, from namespace 'phyloseq'
```

```
## Also defined by 'tidytree'
```

```
## Found more than one class "phylo" in cache; using the first, from namespace 'phyloseq'
```

```
## Also defined by 'tidytree'
```

```
## Found more than one class "phylo" in cache; using the first, from namespace 'phyloseq'
```

```
## Also defined by 'tidytree'
```

```
## Found more than one class "phylo" in cache; using the first, from namespace 'phyloseq'
```

```
## Also defined by 'tidytree'
```

```
## Found more than one class "phylo" in cache; using the first, from namespace 'phyloseq'
```

```
## Also defined by 'tidytree'
```

```
## Found more than one class "phylo" in cache; using the first, from namespace 'phyloseq'
```

```
## Also defined by 'tidytree'
```

```
## Found more than one class "phylo" in cache; using the first, from namespace 'phyloseq'
```

```
## Also defined by 'tidytree'
```

```
## Found more than one class "phylo" in cache; using the first, from namespace 'phyloseq'
```

```
## Also defined by 'tidytree'
```

```
## Found more than one class "phylo" in cache; using the first, from namespace 'phyloseq'
```

```
## Also defined by 'tidytree'
```

```
## Found more than one class "phylo" in cache; using the first, from namespace 'phyloseq'
```

```
## Also defined by 'tidytree'
```

```
## Found more than one class "phylo" in cache; using the first, from namespace 'phyloseq'
```

```
## Also defined by 'tidytree'
```

```
## Found more than one class "phylo" in cache; using the first, from namespace 'phyloseq'
```

```
## Also defined by 'tidytree'
```

```
## Found more than one class "phylo" in cache; using the first, from namespace 'phyloseq'
```

```
## Also defined by 'tidytree'
```

```
## Found more than one class "phylo" in cache; using the first, from namespace 'phyloseq'
```

```
## Also defined by 'tidytree'
```

```
## Found more than one class "phylo" in cache; using the first, from namespace 'phyloseq'
```

```
## Also defined by 'tidytree'
```

```
## Found more than one class "phylo" in cache; using the first, from namespace 'phyloseq'
```

```
## Also defined by 'tidytree'
```

```
## Found more than one class "phylo" in cache; using the first, from namespace 'phyloseq'
```

```
## Also defined by 'tidytree'
```

```
## Found more than one class "phylo" in cache; using the first, from namespace 'phyloseq'
```

```
## Also defined by 'tidytree'
```

```
## Found more than one class "phylo" in cache; using the first, from namespace 'phyloseq'
```

```
## Also defined by 'tidytree'
```

```
## Found more than one class "phylo" in cache; using the first, from namespace 'phyloseq'
```

```
## Also defined by 'tidytree'
```

```
## Found more than one class "phylo" in cache; using the first, from namespace 'phyloseq'
```

```
## Also defined by 'tidytree'
```

```
## Found more than one class "phylo" in cache; using the first, from namespace 'phyloseq'
```

```
## Also defined by 'tidytree'
```

```
## Found more than one class "phylo" in cache; using the first, from namespace 'phyloseq'
```

```
## Also defined by 'tidytree'
```

```
## Found more than one class "phylo" in cache; using the first, from namespace 'phyloseq'
```

```
## Also defined by 'tidytree'
```

```
## Found more than one class "phylo" in cache; using the first, from namespace 'phyloseq'
```

```
## Also defined by 'tidytree'
```

```
## Found more than one class "phylo" in cache; using the first, from namespace 'phyloseq'
```

```
## Also defined by 'tidytree'
```

```
## Found more than one class "phylo" in cache; using the first, from namespace 'phyloseq'
```

```
## Also defined by 'tidytree'
```

```
## Found more than one class "phylo" in cache; using the first, from namespace 'phyloseq'
```

```
## Also defined by 'tidytree'
```

```
## Found more than one class "phylo" in cache; using the first, from namespace 'phyloseq'
```

```
## Also defined by 'tidytree'
```

```
## Found more than one class "phylo" in cache; using the first, from namespace 'phyloseq'
```

```
## Also defined by 'tidytree'
```

```
## Found more than one class "phylo" in cache; using the first, from namespace 'phyloseq'
```

```
## Also defined by 'tidytree'
```

```
## Found more than one class "phylo" in cache; using the first, from namespace 'phyloseq'
```

```
## Also defined by 'tidytree'
```

```
## Found more than one class "phylo" in cache; using the first, from namespace 'phyloseq'
```

```
## Also defined by 'tidytree'
```

```
## Found more than one class "phylo" in cache; using the first, from namespace 'phyloseq'
```

```
## Also defined by 'tidytree'
```

```
## Found more than one class "phylo" in cache; using the first, from namespace 'phyloseq'
```

```
## Also defined by 'tidytree'
```

```
## Found more than one class "phylo" in cache; using the first, from namespace 'phyloseq'
```

```
## Also defined by 'tidytree'
```

```
## Found more than one class "phylo" in cache; using the first, from namespace 'phyloseq'
```

```
## Also defined by 'tidytree'
```

```
## Found more than one class "phylo" in cache; using the first, from namespace 'phyloseq'
```

```
## Also defined by 'tidytree'
```

```
## Found more than one class "phylo" in cache; using the first, from namespace 'phyloseq'
```

```
## Also defined by 'tidytree'
```

```
## Found more than one class "phylo" in cache; using the first, from namespace 'phyloseq'
```

```
## Also defined by 'tidytree'
```

```
## Found more than one class "phylo" in cache; using the first, from namespace 'phyloseq'
```

```
## Also defined by 'tidytree'
```

```
## Found more than one class "phylo" in cache; using the first, from namespace 'phyloseq'
```

```
## Also defined by 'tidytree'
```

```
## Found more than one class "phylo" in cache; using the first, from namespace 'phyloseq'
```

```
## Also defined by 'tidytree'
```

```
## Found more than one class "phylo" in cache; using the first, from namespace 'phyloseq'
```

```
## Also defined by 'tidytree'
```

```
## Found more than one class "phylo" in cache; using the first, from namespace 'phyloseq'
```

```
## Also defined by 'tidytree'
```

```
## Found more than one class "phylo" in cache; using the first, from namespace 'phyloseq'
```

```
## Also defined by 'tidytree'
```

```
## Found more than one class "phylo" in cache; using the first, from namespace 'phyloseq'
```

```
## Also defined by 'tidytree'
```

```
## Found more than one class "phylo" in cache; using the first, from namespace 'phyloseq'
```

```
## Also defined by 'tidytree'
```

```
## Found more than one class "phylo" in cache; using the first, from namespace 'phyloseq'
```

```
## Also defined by 'tidytree'
```

```
## Found more than one class "phylo" in cache; using the first, from namespace 'phyloseq'
```

```
## Also defined by 'tidytree'
```

```
## Found more than one class "phylo" in cache; using the first, from namespace 'phyloseq'
```

```
## Also defined by 'tidytree'
```

```
## Found more than one class "phylo" in cache; using the first, from namespace 'phyloseq'
```

```
## Also defined by 'tidytree'
```

```
## Found more than one class "phylo" in cache; using the first, from namespace 'phyloseq'
```

```
## Also defined by 'tidytree'
```

```
## Found more than one class "phylo" in cache; using the first, from namespace 'phyloseq'
```

```
## Also defined by 'tidytree'
```

```
## Found more than one class "phylo" in cache; using the first, from namespace 'phyloseq'
```

```
## Also defined by 'tidytree'
```

```
## Found more than one class "phylo" in cache; using the first, from namespace 'phyloseq'
```

```
## Also defined by 'tidytree'
```

```
## Found more than one class "phylo" in cache; using the first, from namespace 'phyloseq'
```

```
## Also defined by 'tidytree'
```

```
## Found more than one class "phylo" in cache; using the first, from namespace 'phyloseq'
```

```
## Also defined by 'tidytree'
```

```
## Found more than one class "phylo" in cache; using the first, from namespace 'phyloseq'
```

```
## Also defined by 'tidytree'
```

```
## Found more than one class "phylo" in cache; using the first, from namespace 'phyloseq'
```

```
## Also defined by 'tidytree'
```

```
## Found more than one class "phylo" in cache; using the first, from namespace 'phyloseq'
```

```
## Also defined by 'tidytree'
```

```
## Found more than one class "phylo" in cache; using the first, from namespace 'phyloseq'
```

```
## Also defined by 'tidytree'
```

```
## Found more than one class "phylo" in cache; using the first, from namespace 'phyloseq'
```

```
## Also defined by 'tidytree'
```

```
## Found more than one class "phylo" in cache; using the first, from namespace 'phyloseq'
```

```
## Also defined by 'tidytree'
```

```
## Found more than one class "phylo" in cache; using the first, from namespace 'phyloseq'
```

```
## Also defined by 'tidytree'
```

```
## Found more than one class "phylo" in cache; using the first, from namespace 'phyloseq'
```

```
## Also defined by 'tidytree'
```

```
## Found more than one class "phylo" in cache; using the first, from namespace 'phyloseq'
```

```
## Also defined by 'tidytree'
```

```
## Found more than one class "phylo" in cache; using the first, from namespace 'phyloseq'
```

```
## Also defined by 'tidytree'
```

```
## Found more than one class "phylo" in cache; using the first, from namespace 'phyloseq'
```

```
## Also defined by 'tidytree'
```

```
## Found more than one class "phylo" in cache; using the first, from namespace 'phyloseq'
```

```
## Also defined by 'tidytree'
```

```
## Found more than one class "phylo" in cache; using the first, from namespace 'phyloseq'
```

```
## Also defined by 'tidytree'
```

```
## Found more than one class "phylo" in cache; using the first, from namespace 'phyloseq'
```

```
## Also defined by 'tidytree'
```

```
## Found more than one class "phylo" in cache; using the first, from namespace 'phyloseq'
```

```
## Also defined by 'tidytree'
```

```
## Found more than one class "phylo" in cache; using the first, from namespace 'phyloseq'
```

```
## Also defined by 'tidytree'
```

```
## Found more than one class "phylo" in cache; using the first, from namespace 'phyloseq'
```

```
## Also defined by 'tidytree'
```

```
## Found more than one class "phylo" in cache; using the first, from namespace 'phyloseq'
```

```
## Also defined by 'tidytree'
```

```
## Found more than one class "phylo" in cache; using the first, from namespace 'phyloseq'
```

```
## Also defined by 'tidytree'
```

```
## Found more than one class "phylo" in cache; using the first, from namespace 'phyloseq'
```

```
## Also defined by 'tidytree'
```

```
## Found more than one class "phylo" in cache; using the first, from namespace 'phyloseq'
```

```
## Also defined by 'tidytree'
```

```
## Found more than one class "phylo" in cache; using the first, from namespace 'phyloseq'
```

```
## Also defined by 'tidytree'
```

```
## Found more than one class "phylo" in cache; using the first, from namespace 'phyloseq'
```

```
## Also defined by 'tidytree'
```

```
## Found more than one class "phylo" in cache; using the first, from namespace 'phyloseq'
```

```
## Also defined by 'tidytree'
```

```
## Found more than one class "phylo" in cache; using the first, from namespace 'phyloseq'
```

```
## Also defined by 'tidytree'
```

```
## Found more than one class "phylo" in cache; using the first, from namespace 'phyloseq'
```

```
## Also defined by 'tidytree'
```

```
## Found more than one class "phylo" in cache; using the first, from namespace 'phyloseq'
```

```
## Also defined by 'tidytree'
```

```
## Found more than one class "phylo" in cache; using the first, from namespace 'phyloseq'
```

```
## Also defined by 'tidytree'
```

```
## Found more than one class "phylo" in cache; using the first, from namespace 'phyloseq'
```

```
## Also defined by 'tidytree'
```

```
## Found more than one class "phylo" in cache; using the first, from namespace 'phyloseq'
```

```
## Also defined by 'tidytree'
```

```
## Found more than one class "phylo" in cache; using the first, from namespace 'phyloseq'
```

```
## Also defined by 'tidytree'
```

```
## Found more than one class "phylo" in cache; using the first, from namespace 'phyloseq'
```

```
## Also defined by 'tidytree'
```

```
## Found more than one class "phylo" in cache; using the first, from namespace 'phyloseq'
```

```
## Also defined by 'tidytree'
```

```
## Found more than one class "phylo" in cache; using the first, from namespace 'phyloseq'
```

```
## Also defined by 'tidytree'
```

```
## Found more than one class "phylo" in cache; using the first, from namespace 'phyloseq'
```

```
## Also defined by 'tidytree'
```

```
## Found more than one class "phylo" in cache; using the first, from namespace 'phyloseq'
```

```
## Also defined by 'tidytree'
```

```
## Found more than one class "phylo" in cache; using the first, from namespace 'phyloseq'
```

```
## Also defined by 'tidytree'
```

```
## Found more than one class "phylo" in cache; using the first, from namespace 'phyloseq'
```

```
## Also defined by 'tidytree'
```

```
## Found more than one class "phylo" in cache; using the first, from namespace 'phyloseq'
```

```
## Also defined by 'tidytree'
```

```
## Found more than one class "phylo" in cache; using the first, from namespace 'phyloseq'
```

```
## Also defined by 'tidytree'
```

```
## Found more than one class "phylo" in cache; using the first, from namespace 'phyloseq'
```

```
## Also defined by 'tidytree'
```

```
## Found more than one class "phylo" in cache; using the first, from namespace 'phyloseq'
```

```
## Also defined by 'tidytree'
```

```
## Found more than one class "phylo" in cache; using the first, from namespace 'phyloseq'
```

```
## Also defined by 'tidytree'
```

```
## Found more than one class "phylo" in cache; using the first, from namespace 'phyloseq'
```

```
## Also defined by 'tidytree'
```

```
## Found more than one class "phylo" in cache; using the first, from namespace 'phyloseq'
```

```
## Also defined by 'tidytree'
```

```
## Found more than one class "phylo" in cache; using the first, from namespace 'phyloseq'
```

```
## Also defined by 'tidytree'
```

```
## Found more than one class "phylo" in cache; using the first, from namespace 'phyloseq'
```

```
## Also defined by 'tidytree'
```

```
## Found more than one class "phylo" in cache; using the first, from namespace 'phyloseq'
```

```
## Also defined by 'tidytree'
```

```
## Found more than one class "phylo" in cache; using the first, from namespace 'phyloseq'
```

```
## Also defined by 'tidytree'
```

```
## Found more than one class "phylo" in cache; using the first, from namespace 'phyloseq'
```

```
## Also defined by 'tidytree'
```

```
## Found more than one class "phylo" in cache; using the first, from namespace 'phyloseq'
```

```
## Also defined by 'tidytree'
```

```
## Found more than one class "phylo" in cache; using the first, from namespace 'phyloseq'
```

```
## Also defined by 'tidytree'
```

```
## Found more than one class "phylo" in cache; using the first, from namespace 'phyloseq'
```

```
## Also defined by 'tidytree'
```

```
## Found more than one class "phylo" in cache; using the first, from namespace 'phyloseq'
```

```
## Also defined by 'tidytree'
```

```
## Found more than one class "phylo" in cache; using the first, from namespace 'phyloseq'
```

```
## Also defined by 'tidytree'
```

```
## Found more than one class "phylo" in cache; using the first, from namespace 'phyloseq'
```

```
## Also defined by 'tidytree'
```

```
## Found more than one class "phylo" in cache; using the first, from namespace 'phyloseq'
```

```
## Also defined by 'tidytree'
```

```
## Found more than one class "phylo" in cache; using the first, from namespace 'phyloseq'
```

```
## Also defined by 'tidytree'
```

```
## Found more than one class "phylo" in cache; using the first, from namespace 'phyloseq'
```

```
## Also defined by 'tidytree'
```

```
## Found more than one class "phylo" in cache; using the first, from namespace 'phyloseq'
```

```
## Also defined by 'tidytree'
```

```
## Found more than one class "phylo" in cache; using the first, from namespace 'phyloseq'
```

```
## Also defined by 'tidytree'
```

```
## Found more than one class "phylo" in cache; using the first, from namespace 'phyloseq'
```

```
## Also defined by 'tidytree'
```

```
## Found more than one class "phylo" in cache; using the first, from namespace 'phyloseq'
```

```
## Also defined by 'tidytree'
```

```
## Found more than one class "phylo" in cache; using the first, from namespace 'phyloseq'
```

```
## Also defined by 'tidytree'
```

```
## Found more than one class "phylo" in cache; using the first, from namespace 'phyloseq'
```

```
## Also defined by 'tidytree'
```

```
## Found more than one class "phylo" in cache; using the first, from namespace 'phyloseq'
```

```
## Also defined by 'tidytree'
```

```
## Found more than one class "phylo" in cache; using the first, from namespace 'phyloseq'
```

```
## Also defined by 'tidytree'
```

```
## Found more than one class "phylo" in cache; using the first, from namespace 'phyloseq'
```

```
## Also defined by 'tidytree'
```

```
## Found more than one class "phylo" in cache; using the first, from namespace 'phyloseq'
```

```
## Also defined by 'tidytree'
```

```
## Found more than one class "phylo" in cache; using the first, from namespace 'phyloseq'
```

```
## Also defined by 'tidytree'
```

```
## Found more than one class "phylo" in cache; using the first, from namespace 'phyloseq'
```

```
## Also defined by 'tidytree'
```

```
## Found more than one class "phylo" in cache; using the first, from namespace 'phyloseq'
```

```
## Also defined by 'tidytree'
```

```
## Found more than one class "phylo" in cache; using the first, from namespace 'phyloseq'
```

```
## Also defined by 'tidytree'
```

```
## Found more than one class "phylo" in cache; using the first, from namespace 'phyloseq'
```

```
## Also defined by 'tidytree'
```

```
## Found more than one class "phylo" in cache; using the first, from namespace 'phyloseq'
```

```
## Also defined by 'tidytree'
```

```
## Found more than one class "phylo" in cache; using the first, from namespace 'phyloseq'
```

```
## Also defined by 'tidytree'
```

```
## Found more than one class "phylo" in cache; using the first, from namespace 'phyloseq'
```

```
## Also defined by 'tidytree'
```

```
## Found more than one class "phylo" in cache; using the first, from namespace 'phyloseq'
```

```
## Also defined by 'tidytree'
```

```
## Found more than one class "phylo" in cache; using the first, from namespace 'phyloseq'
```

```
## Also defined by 'tidytree'
```

```
## Found more than one class "phylo" in cache; using the first, from namespace 'phyloseq'
```

```
## Also defined by 'tidytree'
```

```
## Found more than one class "phylo" in cache; using the first, from namespace 'phyloseq'
```

```
## Also defined by 'tidytree'
```

```
## Found more than one class "phylo" in cache; using the first, from namespace 'phyloseq'
```

```
## Also defined by 'tidytree'
```

```
## Found more than one class "phylo" in cache; using the first, from namespace 'phyloseq'
```

```
## Also defined by 'tidytree'
```

```
## Found more than one class "phylo" in cache; using the first, from namespace 'phyloseq'
```

```
## Also defined by 'tidytree'
```

```
## Found more than one class "phylo" in cache; using the first, from namespace 'phyloseq'
```

```
## Also defined by 'tidytree'
```

```
## Found more than one class "phylo" in cache; using the first, from namespace 'phyloseq'
```

```
## Also defined by 'tidytree'
```

```
## Found more than one class "phylo" in cache; using the first, from namespace 'phyloseq'
```

```
## Also defined by 'tidytree'
```

```
## Found more than one class "phylo" in cache; using the first, from namespace 'phyloseq'
```

```
## Also defined by 'tidytree'
```

```
## Found more than one class "phylo" in cache; using the first, from namespace 'phyloseq'
```

```
## Also defined by 'tidytree'
```

```
## Found more than one class "phylo" in cache; using the first, from namespace 'phyloseq'
```

```
## Also defined by 'tidytree'
```

```
## Found more than one class "phylo" in cache; using the first, from namespace 'phyloseq'
```

```
## Also defined by 'tidytree'
```

```
## Found more than one class "phylo" in cache; using the first, from namespace 'phyloseq'
```

```
## Also defined by 'tidytree'
```

```
## Found more than one class "phylo" in cache; using the first, from namespace 'phyloseq'
```

```
## Also defined by 'tidytree'
```

```
## Found more than one class "phylo" in cache; using the first, from namespace 'phyloseq'
```

```
## Also defined by 'tidytree'
```

```
## Found more than one class "phylo" in cache; using the first, from namespace 'phyloseq'
```

```
## Also defined by 'tidytree'
```

```
## Found more than one class "phylo" in cache; using the first, from namespace 'phyloseq'
```

```
## Also defined by 'tidytree'
```

```
## Found more than one class "phylo" in cache; using the first, from namespace 'phyloseq'
```

```
## Also defined by 'tidytree'
```

```
## Found more than one class "phylo" in cache; using the first, from namespace 'phyloseq'
```

```
## Also defined by 'tidytree'
```

```
## Found more than one class "phylo" in cache; using the first, from namespace 'phyloseq'
```

```
## Also defined by 'tidytree'
```

```
## Found more than one class "phylo" in cache; using the first, from namespace 'phyloseq'
```

```
## Also defined by 'tidytree'
```

```
## Found more than one class "phylo" in cache; using the first, from namespace 'phyloseq'
```

```
## Also defined by 'tidytree'
```

```
## Found more than one class "phylo" in cache; using the first, from namespace 'phyloseq'
```

```
## Also defined by 'tidytree'
```

```
## Found more than one class "phylo" in cache; using the first, from namespace 'phyloseq'
```

```
## Also defined by 'tidytree'
```

```
## Found more than one class "phylo" in cache; using the first, from namespace 'phyloseq'
```

```
## Also defined by 'tidytree'
```

```
## Found more than one class "phylo" in cache; using the first, from namespace 'phyloseq'
```

```
## Also defined by 'tidytree'
```

```
## Found more than one class "phylo" in cache; using the first, from namespace 'phyloseq'
```

```
## Also defined by 'tidytree'
```

```
## Found more than one class "phylo" in cache; using the first, from namespace 'phyloseq'
```

```
## Also defined by 'tidytree'
```

```
## Found more than one class "phylo" in cache; using the first, from namespace 'phyloseq'
```

```
## Also defined by 'tidytree'
```

```
## Found more than one class "phylo" in cache; using the first, from namespace 'phyloseq'
```

```
## Also defined by 'tidytree'
```

```
## Found more than one class "phylo" in cache; using the first, from namespace 'phyloseq'
```

```
## Also defined by 'tidytree'
```

```
## Found more than one class "phylo" in cache; using the first, from namespace 'phyloseq'
```

```
## Also defined by 'tidytree'
```

```
## Found more than one class "phylo" in cache; using the first, from namespace 'phyloseq'
```

```
## Also defined by 'tidytree'
```

```
## Found more than one class "phylo" in cache; using the first, from namespace 'phyloseq'
```

```
## Also defined by 'tidytree'
```

```
## Found more than one class "phylo" in cache; using the first, from namespace 'phyloseq'
```

```
## Also defined by 'tidytree'
```

```
## Found more than one class "phylo" in cache; using the first, from namespace 'phyloseq'
```

```
## Also defined by 'tidytree'
```

```
## Found more than one class "phylo" in cache; using the first, from namespace 'phyloseq'
```

```
## Also defined by 'tidytree'
```

```
## Found more than one class "phylo" in cache; using the first, from namespace 'phyloseq'
```

```
## Also defined by 'tidytree'
```

```
## Found more than one class "phylo" in cache; using the first, from namespace 'phyloseq'
```

```
## Also defined by 'tidytree'
```

```
## Found more than one class "phylo" in cache; using the first, from namespace 'phyloseq'
```

```
## Also defined by 'tidytree'
```

```
## Found more than one class "phylo" in cache; using the first, from namespace 'phyloseq'
```

```
## Also defined by 'tidytree'
```

```
## Found more than one class "phylo" in cache; using the first, from namespace 'phyloseq'
```

```
## Also defined by 'tidytree'
```

```
## Found more than one class "phylo" in cache; using the first, from namespace 'phyloseq'
```

```
## Also defined by 'tidytree'
```

```
## Found more than one class "phylo" in cache; using the first, from namespace 'phyloseq'
```

```
## Also defined by 'tidytree'
```

```
## Found more than one class "phylo" in cache; using the first, from namespace 'phyloseq'
```

```
## Also defined by 'tidytree'
```

```
## Found more than one class "phylo" in cache; using the first, from namespace 'phyloseq'
```

```
## Also defined by 'tidytree'
```

```
## Found more than one class "phylo" in cache; using the first, from namespace 'phyloseq'
```

```
## Also defined by 'tidytree'
```

```
## Found more than one class "phylo" in cache; using the first, from namespace 'phyloseq'
```

```
## Also defined by 'tidytree'
```

```
## Found more than one class "phylo" in cache; using the first, from namespace 'phyloseq'
```

```
## Also defined by 'tidytree'
```

```
## Found more than one class "phylo" in cache; using the first, from namespace 'phyloseq'
```

```
## Also defined by 'tidytree'
```

```
## Found more than one class "phylo" in cache; using the first, from namespace 'phyloseq'
```

```
## Also defined by 'tidytree'
```

```
## Found more than one class "phylo" in cache; using the first, from namespace 'phyloseq'
```

```
## Also defined by 'tidytree'
```

```
## Found more than one class "phylo" in cache; using the first, from namespace 'phyloseq'
```

```
## Also defined by 'tidytree'
```

```
## Found more than one class "phylo" in cache; using the first, from namespace 'phyloseq'
```

```
## Also defined by 'tidytree'
```

```
## Found more than one class "phylo" in cache; using the first, from namespace 'phyloseq'
```

```
## Also defined by 'tidytree'
```

```
## Found more than one class "phylo" in cache; using the first, from namespace 'phyloseq'
```

```
## Also defined by 'tidytree'
```

```
## Found more than one class "phylo" in cache; using the first, from namespace 'phyloseq'
```

```
## Also defined by 'tidytree'
```

```
## Found more than one class "phylo" in cache; using the first, from namespace 'phyloseq'
```

```
## Also defined by 'tidytree'
```

```
## Found more than one class "phylo" in cache; using the first, from namespace 'phyloseq'
```

```
## Also defined by 'tidytree'
```

```
## Found more than one class "phylo" in cache; using the first, from namespace 'phyloseq'
```

```
## Also defined by 'tidytree'
```

```
## Found more than one class "phylo" in cache; using the first, from namespace 'phyloseq'
```

```
## Also defined by 'tidytree'
```

```
## Found more than one class "phylo" in cache; using the first, from namespace 'phyloseq'
```

```
## Also defined by 'tidytree'
```

```
## Found more than one class "phylo" in cache; using the first, from namespace 'phyloseq'
```

```
## Also defined by 'tidytree'
```

```
## Found more than one class "phylo" in cache; using the first, from namespace 'phyloseq'
```

```
## Also defined by 'tidytree'
```

```
## Found more than one class "phylo" in cache; using the first, from namespace 'phyloseq'
```

```
## Also defined by 'tidytree'
```

```
## Found more than one class "phylo" in cache; using the first, from namespace 'phyloseq'
```

```
## Also defined by 'tidytree'
```

```
## Found more than one class "phylo" in cache; using the first, from namespace 'phyloseq'
```

```
## Also defined by 'tidytree'
```

```
## Found more than one class "phylo" in cache; using the first, from namespace 'phyloseq'
```

```
## Also defined by 'tidytree'
```

```
## Found more than one class "phylo" in cache; using the first, from namespace 'phyloseq'
```

```
## Also defined by 'tidytree'
```

```
## Found more than one class "phylo" in cache; using the first, from namespace 'phyloseq'
```

```
## Also defined by 'tidytree'
```

```
## Found more than one class "phylo" in cache; using the first, from namespace 'phyloseq'
```

```
## Also defined by 'tidytree'
```

```
## Found more than one class "phylo" in cache; using the first, from namespace 'phyloseq'
```

```
## Also defined by 'tidytree'
```

```
## Found more than one class "phylo" in cache; using the first, from namespace 'phyloseq'
```

```
## Also defined by 'tidytree'
```

```
## Found more than one class "phylo" in cache; using the first, from namespace 'phyloseq'
```

```
## Also defined by 'tidytree'
```

```
## Found more than one class "phylo" in cache; using the first, from namespace 'phyloseq'
```

```
## Also defined by 'tidytree'
```

```
## Found more than one class "phylo" in cache; using the first, from namespace 'phyloseq'
```

```
## Also defined by 'tidytree'
```

```
## Found more than one class "phylo" in cache; using the first, from namespace 'phyloseq'
```

```
## Also defined by 'tidytree'
```

```
## Found more than one class "phylo" in cache; using the first, from namespace 'phyloseq'
```

```
## Also defined by 'tidytree'
```

```
## Found more than one class "phylo" in cache; using the first, from namespace 'phyloseq'
```

```
## Also defined by 'tidytree'
```

```
## Found more than one class "phylo" in cache; using the first, from namespace 'phyloseq'
```

```
## Also defined by 'tidytree'
```

```
## Found more than one class "phylo" in cache; using the first, from namespace 'phyloseq'
```

```
## Also defined by 'tidytree'
```

```
## Found more than one class "phylo" in cache; using the first, from namespace 'phyloseq'
```

```
## Also defined by 'tidytree'
```

```
## Found more than one class "phylo" in cache; using the first, from namespace 'phyloseq'
```

```
## Also defined by 'tidytree'
```

```
## Found more than one class "phylo" in cache; using the first, from namespace 'phyloseq'
```

```
## Also defined by 'tidytree'
```

```
## Found more than one class "phylo" in cache; using the first, from namespace 'phyloseq'
```

```
## Also defined by 'tidytree'
```

```
## Found more than one class "phylo" in cache; using the first, from namespace 'phyloseq'
```

```
## Also defined by 'tidytree'
```

```
## Found more than one class "phylo" in cache; using the first, from namespace 'phyloseq'
```

```
## Also defined by 'tidytree'
```

```
## Found more than one class "phylo" in cache; using the first, from namespace 'phyloseq'
```

```
## Also defined by 'tidytree'
```

```
## Found more than one class "phylo" in cache; using the first, from namespace 'phyloseq'
```

```
## Also defined by 'tidytree'
```

```
## Found more than one class "phylo" in cache; using the first, from namespace 'phyloseq'
```

```
## Also defined by 'tidytree'
```

```
## Found more than one class "phylo" in cache; using the first, from namespace 'phyloseq'
```

```
## Also defined by 'tidytree'
```

```
## Found more than one class "phylo" in cache; using the first, from namespace 'phyloseq'
```

```
## Also defined by 'tidytree'
```

```
## Found more than one class "phylo" in cache; using the first, from namespace 'phyloseq'
```

```
## Also defined by 'tidytree'
```

```
## Found more than one class "phylo" in cache; using the first, from namespace 'phyloseq'
```

```
## Also defined by 'tidytree'
```

```
## Found more than one class "phylo" in cache; using the first, from namespace 'phyloseq'
```

```
## Also defined by 'tidytree'
```

```
## Found more than one class "phylo" in cache; using the first, from namespace 'phyloseq'
```

```
## Also defined by 'tidytree'
```

```
## Found more than one class "phylo" in cache; using the first, from namespace 'phyloseq'
```

```
## Also defined by 'tidytree'
```

```
## Found more than one class "phylo" in cache; using the first, from namespace 'phyloseq'
```

```
## Also defined by 'tidytree'
```

```
## Found more than one class "phylo" in cache; using the first, from namespace 'phyloseq'
```

```
## Also defined by 'tidytree'
```

```
## Found more than one class "phylo" in cache; using the first, from namespace 'phyloseq'
```

```
## Also defined by 'tidytree'
```

```
## Found more than one class "phylo" in cache; using the first, from namespace 'phyloseq'
```

```
## Also defined by 'tidytree'
```

```
## Found more than one class "phylo" in cache; using the first, from namespace 'phyloseq'
```

```
## Also defined by 'tidytree'
```

```
## Found more than one class "phylo" in cache; using the first, from namespace 'phyloseq'
```

```
## Also defined by 'tidytree'
```

```
## Found more than one class "phylo" in cache; using the first, from namespace 'phyloseq'
```

```
## Also defined by 'tidytree'
```

```
## Found more than one class "phylo" in cache; using the first, from namespace 'phyloseq'
```

```
## Also defined by 'tidytree'
```

```
## Found more than one class "phylo" in cache; using the first, from namespace 'phyloseq'
```

```
## Also defined by 'tidytree'
```

```
## Found more than one class "phylo" in cache; using the first, from namespace 'phyloseq'
```

```
## Also defined by 'tidytree'
```

```
## Found more than one class "phylo" in cache; using the first, from namespace 'phyloseq'
```

```
## Also defined by 'tidytree'
```

```
## Found more than one class "phylo" in cache; using the first, from namespace 'phyloseq'
```

```
## Also defined by 'tidytree'
```

```
## Found more than one class "phylo" in cache; using the first, from namespace 'phyloseq'
```

```
## Also defined by 'tidytree'
```

```
## Found more than one class "phylo" in cache; using the first, from namespace 'phyloseq'
```

```
## Also defined by 'tidytree'
```

```
## Found more than one class "phylo" in cache; using the first, from namespace 'phyloseq'
```

```
## Also defined by 'tidytree'
```

```
## Found more than one class "phylo" in cache; using the first, from namespace 'phyloseq'
```

```
## Also defined by 'tidytree'
```

```
## Found more than one class "phylo" in cache; using the first, from namespace 'phyloseq'
```

```
## Also defined by 'tidytree'
```

```
## Found more than one class "phylo" in cache; using the first, from namespace 'phyloseq'
```

```
## Also defined by 'tidytree'
```

```
## Found more than one class "phylo" in cache; using the first, from namespace 'phyloseq'
```

```
## Also defined by 'tidytree'
```

```
## Found more than one class "phylo" in cache; using the first, from namespace 'phyloseq'
```

```
## Also defined by 'tidytree'
```

```
## Found more than one class "phylo" in cache; using the first, from namespace 'phyloseq'
```

```
## Also defined by 'tidytree'
```

```
## Found more than one class "phylo" in cache; using the first, from namespace 'phyloseq'
```

```
## Also defined by 'tidytree'
```

```
## Found more than one class "phylo" in cache; using the first, from namespace 'phyloseq'
```

```
## Also defined by 'tidytree'
```

```
## Found more than one class "phylo" in cache; using the first, from namespace 'phyloseq'
```

```
## Also defined by 'tidytree'
```

```
## Found more than one class "phylo" in cache; using the first, from namespace 'phyloseq'
```

```
## Also defined by 'tidytree'
```

```
## Found more than one class "phylo" in cache; using the first, from namespace 'phyloseq'
```

```
## Also defined by 'tidytree'
```

```
## Found more than one class "phylo" in cache; using the first, from namespace 'phyloseq'
```

```
## Also defined by 'tidytree'
```

```
## Found more than one class "phylo" in cache; using the first, from namespace 'phyloseq'
```

```
## Also defined by 'tidytree'
```

```
## Found more than one class "phylo" in cache; using the first, from namespace 'phyloseq'
```

```
## Also defined by 'tidytree'
```

```
## Found more than one class "phylo" in cache; using the first, from namespace 'phyloseq'
```

```
## Also defined by 'tidytree'
```

```
## Found more than one class "phylo" in cache; using the first, from namespace 'phyloseq'
```

```
## Also defined by 'tidytree'
```

```
## Found more than one class "phylo" in cache; using the first, from namespace 'phyloseq'
```

```
## Also defined by 'tidytree'
```

```
## Found more than one class "phylo" in cache; using the first, from namespace 'phyloseq'
```

```
## Also defined by 'tidytree'
```

```
## Found more than one class "phylo" in cache; using the first, from namespace 'phyloseq'
```

```
## Also defined by 'tidytree'
```

```
## Found more than one class "phylo" in cache; using the first, from namespace 'phyloseq'
```

```
## Also defined by 'tidytree'
```

```
## Found more than one class "phylo" in cache; using the first, from namespace 'phyloseq'
```

```
## Also defined by 'tidytree'
```

```
## Found more than one class "phylo" in cache; using the first, from namespace 'phyloseq'
```

```
## Also defined by 'tidytree'
```

```
## Found more than one class "phylo" in cache; using the first, from namespace 'phyloseq'
```

```
## Also defined by 'tidytree'
```

```
## Found more than one class "phylo" in cache; using the first, from namespace 'phyloseq'
```

```
## Also defined by 'tidytree'
```

```
## Found more than one class "phylo" in cache; using the first, from namespace 'phyloseq'
```

```
## Also defined by 'tidytree'
```

```
## Found more than one class "phylo" in cache; using the first, from namespace 'phyloseq'
```

```
## Also defined by 'tidytree'
```

```
## Found more than one class "phylo" in cache; using the first, from namespace 'phyloseq'
```

```
## Also defined by 'tidytree'
```

```
## Found more than one class "phylo" in cache; using the first, from namespace 'phyloseq'
```

```
## Also defined by 'tidytree'
```

```
## Found more than one class "phylo" in cache; using the first, from namespace 'phyloseq'
```

```
## Also defined by 'tidytree'
```

```
## Found more than one class "phylo" in cache; using the first, from namespace 'phyloseq'
```

```
## Also defined by 'tidytree'
```

```
## Found more than one class "phylo" in cache; using the first, from namespace 'phyloseq'
```

```
## Also defined by 'tidytree'
```

```
## Found more than one class "phylo" in cache; using the first, from namespace 'phyloseq'
```

```
## Also defined by 'tidytree'
```

```
## Found more than one class "phylo" in cache; using the first, from namespace 'phyloseq'
```

```
## Also defined by 'tidytree'
```

```
## Found more than one class "phylo" in cache; using the first, from namespace 'phyloseq'
```

```
## Also defined by 'tidytree'
```

```
## Found more than one class "phylo" in cache; using the first, from namespace 'phyloseq'
```

```
## Also defined by 'tidytree'
```

```
## Found more than one class "phylo" in cache; using the first, from namespace 'phyloseq'
```

```
## Also defined by 'tidytree'
```

```
## Found more than one class "phylo" in cache; using the first, from namespace 'phyloseq'
```

```
## Also defined by 'tidytree'
```

```
## Found more than one class "phylo" in cache; using the first, from namespace 'phyloseq'
```

```
## Also defined by 'tidytree'
```

```
## Found more than one class "phylo" in cache; using the first, from namespace 'phyloseq'
```

```
## Also defined by 'tidytree'
```

```
## Found more than one class "phylo" in cache; using the first, from namespace 'phyloseq'
```

```
## Also defined by 'tidytree'
```

```
## Found more than one class "phylo" in cache; using the first, from namespace 'phyloseq'
```

```
## Also defined by 'tidytree'
```

```
## Found more than one class "phylo" in cache; using the first, from namespace 'phyloseq'
```

```
## Also defined by 'tidytree'
```

```
## Found more than one class "phylo" in cache; using the first, from namespace 'phyloseq'
```

```
## Also defined by 'tidytree'
```

```
## Found more than one class "phylo" in cache; using the first, from namespace 'phyloseq'
```

```
## Also defined by 'tidytree'
```

```
## Found more than one class "phylo" in cache; using the first, from namespace 'phyloseq'
```

```
## Also defined by 'tidytree'
```

```
## Found more than one class "phylo" in cache; using the first, from namespace 'phyloseq'
```

```
## Also defined by 'tidytree'
```

```
## Found more than one class "phylo" in cache; using the first, from namespace 'phyloseq'
```

```
## Also defined by 'tidytree'
```

```
## Found more than one class "phylo" in cache; using the first, from namespace 'phyloseq'
```

```
## Also defined by 'tidytree'
```

```
## Found more than one class "phylo" in cache; using the first, from namespace 'phyloseq'
```

```
## Also defined by 'tidytree'
```

```
## Found more than one class "phylo" in cache; using the first, from namespace 'phyloseq'
```

```
## Also defined by 'tidytree'
```

```
## Found more than one class "phylo" in cache; using the first, from namespace 'phyloseq'
```

```
## Also defined by 'tidytree'
```

```
## Found more than one class "phylo" in cache; using the first, from namespace 'phyloseq'
```

```
## Also defined by 'tidytree'
```

```
## Found more than one class "phylo" in cache; using the first, from namespace 'phyloseq'
```

```
## Also defined by 'tidytree'
```

```
## Found more than one class "phylo" in cache; using the first, from namespace 'phyloseq'
```

```
## Also defined by 'tidytree'
```

```
## Found more than one class "phylo" in cache; using the first, from namespace 'phyloseq'
```

```
## Also defined by 'tidytree'
```

```
## Found more than one class "phylo" in cache; using the first, from namespace 'phyloseq'
```

```
## Also defined by 'tidytree'
```

```
## Found more than one class "phylo" in cache; using the first, from namespace 'phyloseq'
```

```
## Also defined by 'tidytree'
```

```
## Found more than one class "phylo" in cache; using the first, from namespace 'phyloseq'
```

```
## Also defined by 'tidytree'
```

```
## Found more than one class "phylo" in cache; using the first, from namespace 'phyloseq'
```

```
## Also defined by 'tidytree'
```

```
## Found more than one class "phylo" in cache; using the first, from namespace 'phyloseq'
```

```
## Also defined by 'tidytree'
```

```
## Found more than one class "phylo" in cache; using the first, from namespace 'phyloseq'
```

```
## Also defined by 'tidytree'
```

```
## Found more than one class "phylo" in cache; using the first, from namespace 'phyloseq'
```

```
## Also defined by 'tidytree'
```

```
## Found more than one class "phylo" in cache; using the first, from namespace 'phyloseq'
```

```
## Also defined by 'tidytree'
```

```
## Found more than one class "phylo" in cache; using the first, from namespace 'phyloseq'
```

```
## Also defined by 'tidytree'
```

```
## Found more than one class "phylo" in cache; using the first, from namespace 'phyloseq'
```

```
## Also defined by 'tidytree'
```

```
## Found more than one class "phylo" in cache; using the first, from namespace 'phyloseq'
```

```
## Also defined by 'tidytree'
```

```
## Found more than one class "phylo" in cache; using the first, from namespace 'phyloseq'
```

```
## Also defined by 'tidytree'
```

```
## Found more than one class "phylo" in cache; using the first, from namespace 'phyloseq'
```

```
## Also defined by 'tidytree'
```

```
## Found more than one class "phylo" in cache; using the first, from namespace 'phyloseq'
```

```
## Also defined by 'tidytree'
```

```
## Found more than one class "phylo" in cache; using the first, from namespace 'phyloseq'
```

```
## Also defined by 'tidytree'
```

```
## Found more than one class "phylo" in cache; using the first, from namespace 'phyloseq'
```

```
## Also defined by 'tidytree'
```

```
## Found more than one class "phylo" in cache; using the first, from namespace 'phyloseq'
```

```
## Also defined by 'tidytree'
```

```
## Found more than one class "phylo" in cache; using the first, from namespace 'phyloseq'
```

```
## Also defined by 'tidytree'
```

```
## Found more than one class "phylo" in cache; using the first, from namespace 'phyloseq'
```

```
## Also defined by 'tidytree'
```

```
## Found more than one class "phylo" in cache; using the first, from namespace 'phyloseq'
```

```
## Also defined by 'tidytree'
```

```
## Found more than one class "phylo" in cache; using the first, from namespace 'phyloseq'
```

```
## Also defined by 'tidytree'
```

```
## Found more than one class "phylo" in cache; using the first, from namespace 'phyloseq'
```

```
## Also defined by 'tidytree'
```

```
## Found more than one class "phylo" in cache; using the first, from namespace 'phyloseq'
```

```
## Also defined by 'tidytree'
```

```
## Found more than one class "phylo" in cache; using the first, from namespace 'phyloseq'
```

```
## Also defined by 'tidytree'
```

```
## Found more than one class "phylo" in cache; using the first, from namespace 'phyloseq'
```

```
## Also defined by 'tidytree'
```

```
## Found more than one class "phylo" in cache; using the first, from namespace 'phyloseq'
```

```
## Also defined by 'tidytree'
```

```
## Found more than one class "phylo" in cache; using the first, from namespace 'phyloseq'
```

```
## Also defined by 'tidytree'
```

```
## Found more than one class "phylo" in cache; using the first, from namespace 'phyloseq'
```

```
## Also defined by 'tidytree'
```

```
## Found more than one class "phylo" in cache; using the first, from namespace 'phyloseq'
```

```
## Also defined by 'tidytree'
```

```
## Found more than one class "phylo" in cache; using the first, from namespace 'phyloseq'
```

```
## Also defined by 'tidytree'
```

```
## Found more than one class "phylo" in cache; using the first, from namespace 'phyloseq'
```

```
## Also defined by 'tidytree'
```

```
## Found more than one class "phylo" in cache; using the first, from namespace 'phyloseq'
```

```
## Also defined by 'tidytree'
```

```
## Found more than one class "phylo" in cache; using the first, from namespace 'phyloseq'
```

```
## Also defined by 'tidytree'
```

```
## Found more than one class "phylo" in cache; using the first, from namespace 'phyloseq'
```

```
## Also defined by 'tidytree'
```

```
## Found more than one class "phylo" in cache; using the first, from namespace 'phyloseq'
```

```
## Also defined by 'tidytree'
```

```
## Found more than one class "phylo" in cache; using the first, from namespace 'phyloseq'
```

```
## Also defined by 'tidytree'
```

```
## Found more than one class "phylo" in cache; using the first, from namespace 'phyloseq'
```

```
## Also defined by 'tidytree'
```

```
## Found more than one class "phylo" in cache; using the first, from namespace 'phyloseq'
```

```
## Also defined by 'tidytree'
```

```
## Found more than one class "phylo" in cache; using the first, from namespace 'phyloseq'
```

```
## Also defined by 'tidytree'
```

```
## Found more than one class "phylo" in cache; using the first, from namespace 'phyloseq'
```

```
## Also defined by 'tidytree'
```

```
## Found more than one class "phylo" in cache; using the first, from namespace 'phyloseq'
```

```
## Also defined by 'tidytree'
```

```
## Found more than one class "phylo" in cache; using the first, from namespace 'phyloseq'
```

```
## Also defined by 'tidytree'
```

```
## Found more than one class "phylo" in cache; using the first, from namespace 'phyloseq'
```

```
## Also defined by 'tidytree'
```

```
## Found more than one class "phylo" in cache; using the first, from namespace 'phyloseq'
```

```
## Also defined by 'tidytree'
```

```
## Found more than one class "phylo" in cache; using the first, from namespace 'phyloseq'
```

```
## Also defined by 'tidytree'
```

```
## Found more than one class "phylo" in cache; using the first, from namespace 'phyloseq'
```

```
## Also defined by 'tidytree'
```

```
## Found more than one class "phylo" in cache; using the first, from namespace 'phyloseq'
```

```
## Also defined by 'tidytree'
```

```
## Found more than one class "phylo" in cache; using the first, from namespace 'phyloseq'
```

```
## Also defined by 'tidytree'
```

```
## Found more than one class "phylo" in cache; using the first, from namespace 'phyloseq'
```

```
## Also defined by 'tidytree'
```

```
## Found more than one class "phylo" in cache; using the first, from namespace 'phyloseq'
```

```
## Also defined by 'tidytree'
```

```
## Found more than one class "phylo" in cache; using the first, from namespace 'phyloseq'
```

```
## Also defined by 'tidytree'
```

```
## Found more than one class "phylo" in cache; using the first, from namespace 'phyloseq'
```

```
## Also defined by 'tidytree'
```

```
## Found more than one class "phylo" in cache; using the first, from namespace 'phyloseq'
```

```
## Also defined by 'tidytree'
```

```
## Found more than one class "phylo" in cache; using the first, from namespace 'phyloseq'
```

```
## Also defined by 'tidytree'
```

```
## Found more than one class "phylo" in cache; using the first, from namespace 'phyloseq'
```

```
## Also defined by 'tidytree'
```

```
## Found more than one class "phylo" in cache; using the first, from namespace 'phyloseq'
```

```
## Also defined by 'tidytree'
```

```
## Found more than one class "phylo" in cache; using the first, from namespace 'phyloseq'
```

```
## Also defined by 'tidytree'
```

```
## Found more than one class "phylo" in cache; using the first, from namespace 'phyloseq'
```

```
## Also defined by 'tidytree'
```

```
## Found more than one class "phylo" in cache; using the first, from namespace 'phyloseq'
```

```
## Also defined by 'tidytree'
```

```
## Found more than one class "phylo" in cache; using the first, from namespace 'phyloseq'
```

```
## Also defined by 'tidytree'
```

```
## Found more than one class "phylo" in cache; using the first, from namespace 'phyloseq'
```

```
## Also defined by 'tidytree'
```

```
## Found more than one class "phylo" in cache; using the first, from namespace 'phyloseq'
```

```
## Also defined by 'tidytree'
```

```
## Found more than one class "phylo" in cache; using the first, from namespace 'phyloseq'
```

```
## Also defined by 'tidytree'
```

```
## Found more than one class "phylo" in cache; using the first, from namespace 'phyloseq'
```

```
## Also defined by 'tidytree'
```

```
## Found more than one class "phylo" in cache; using the first, from namespace 'phyloseq'
```

```
## Also defined by 'tidytree'
```

```
## Found more than one class "phylo" in cache; using the first, from namespace 'phyloseq'
```

```
## Also defined by 'tidytree'
```

```
## Found more than one class "phylo" in cache; using the first, from namespace 'phyloseq'
```

```
## Also defined by 'tidytree'
```

```
## Found more than one class "phylo" in cache; using the first, from namespace 'phyloseq'
```

```
## Also defined by 'tidytree'
```

```
## Found more than one class "phylo" in cache; using the first, from namespace 'phyloseq'
```

```
## Also defined by 'tidytree'
```

```
## Found more than one class "phylo" in cache; using the first, from namespace 'phyloseq'
```

```
## Also defined by 'tidytree'
```

```
## Found more than one class "phylo" in cache; using the first, from namespace 'phyloseq'
```

```
## Also defined by 'tidytree'
```

```
## Found more than one class "phylo" in cache; using the first, from namespace 'phyloseq'
```

```
## Also defined by 'tidytree'
```

```
## Found more than one class "phylo" in cache; using the first, from namespace 'phyloseq'
```

```
## Also defined by 'tidytree'
```

```
## Found more than one class "phylo" in cache; using the first, from namespace 'phyloseq'
```

```
## Also defined by 'tidytree'
```

```
## Found more than one class "phylo" in cache; using the first, from namespace 'phyloseq'
```

```
## Also defined by 'tidytree'
```

```
## Found more than one class "phylo" in cache; using the first, from namespace 'phyloseq'
```

```
## Also defined by 'tidytree'
```

```
## Found more than one class "phylo" in cache; using the first, from namespace 'phyloseq'
```

```
## Also defined by 'tidytree'
```

```
## Found more than one class "phylo" in cache; using the first, from namespace 'phyloseq'
```

```
## Also defined by 'tidytree'
```

```
## Found more than one class "phylo" in cache; using the first, from namespace 'phyloseq'
```

```
## Also defined by 'tidytree'
```

```
## Found more than one class "phylo" in cache; using the first, from namespace 'phyloseq'
```

```
## Also defined by 'tidytree'
```

```
## Found more than one class "phylo" in cache; using the first, from namespace 'phyloseq'
```

```
## Also defined by 'tidytree'
```

```
## Found more than one class "phylo" in cache; using the first, from namespace 'phyloseq'
```

```
## Also defined by 'tidytree'
```

```
## Found more than one class "phylo" in cache; using the first, from namespace 'phyloseq'
```

```
## Also defined by 'tidytree'
```

```
## Found more than one class "phylo" in cache; using the first, from namespace 'phyloseq'
```

```
## Also defined by 'tidytree'
```

```
## Found more than one class "phylo" in cache; using the first, from namespace 'phyloseq'
```

```
## Also defined by 'tidytree'
```

```
## Found more than one class "phylo" in cache; using the first, from namespace 'phyloseq'
```

```
## Also defined by 'tidytree'
```

```
## Found more than one class "phylo" in cache; using the first, from namespace 'phyloseq'
```

```
## Also defined by 'tidytree'
```

```
## Found more than one class "phylo" in cache; using the first, from namespace 'phyloseq'
```

```
## Also defined by 'tidytree'
```

```
## Found more than one class "phylo" in cache; using the first, from namespace 'phyloseq'
```

```
## Also defined by 'tidytree'
```

```
## Found more than one class "phylo" in cache; using the first, from namespace 'phyloseq'
```

```
## Also defined by 'tidytree'
```

```
## Found more than one class "phylo" in cache; using the first, from namespace 'phyloseq'
```

```
## Also defined by 'tidytree'
```

```
## Found more than one class "phylo" in cache; using the first, from namespace 'phyloseq'
```

```
## Also defined by 'tidytree'
```

```
## Found more than one class "phylo" in cache; using the first, from namespace 'phyloseq'
```

```
## Also defined by 'tidytree'
```

```
## Found more than one class "phylo" in cache; using the first, from namespace 'phyloseq'
```

```
## Also defined by 'tidytree'
```

```
## Found more than one class "phylo" in cache; using the first, from namespace 'phyloseq'
```

```
## Also defined by 'tidytree'
```

```
## Found more than one class "phylo" in cache; using the first, from namespace 'phyloseq'
```

```
## Also defined by 'tidytree'
```

```
## Found more than one class "phylo" in cache; using the first, from namespace 'phyloseq'
```

```
## Also defined by 'tidytree'
```

```
## Found more than one class "phylo" in cache; using the first, from namespace 'phyloseq'
```

```
## Also defined by 'tidytree'
```

```
## Found more than one class "phylo" in cache; using the first, from namespace 'phyloseq'
```

```
## Also defined by 'tidytree'
```

```
## Found more than one class "phylo" in cache; using the first, from namespace 'phyloseq'
```

```
## Also defined by 'tidytree'
```

```
## Found more than one class "phylo" in cache; using the first, from namespace 'phyloseq'
```

```
## Also defined by 'tidytree'
```

```
## Found more than one class "phylo" in cache; using the first, from namespace 'phyloseq'
```

```
## Also defined by 'tidytree'
```

```
## Found more than one class "phylo" in cache; using the first, from namespace 'phyloseq'
```

```
## Also defined by 'tidytree'
```

```
## Found more than one class "phylo" in cache; using the first, from namespace 'phyloseq'
```

```
## Also defined by 'tidytree'
```

```
## Found more than one class "phylo" in cache; using the first, from namespace 'phyloseq'
```

```
## Also defined by 'tidytree'
```

```
## Found more than one class "phylo" in cache; using the first, from namespace 'phyloseq'
```

```
## Also defined by 'tidytree'
```

```
## Found more than one class "phylo" in cache; using the first, from namespace 'phyloseq'
```

```
## Also defined by 'tidytree'
```

```
## Found more than one class "phylo" in cache; using the first, from namespace 'phyloseq'
```

```
## Also defined by 'tidytree'
```

```
## Found more than one class "phylo" in cache; using the first, from namespace 'phyloseq'
```

```
## Also defined by 'tidytree'
```

```
## Found more than one class "phylo" in cache; using the first, from namespace 'phyloseq'
```

```
## Also defined by 'tidytree'
```

```
## Found more than one class "phylo" in cache; using the first, from namespace 'phyloseq'
```

```
## Also defined by 'tidytree'
```

```
## Found more than one class "phylo" in cache; using the first, from namespace 'phyloseq'
```

```
## Also defined by 'tidytree'
```

```
## Found more than one class "phylo" in cache; using the first, from namespace 'phyloseq'
```

```
## Also defined by 'tidytree'
```

```
## Found more than one class "phylo" in cache; using the first, from namespace 'phyloseq'
```

```
## Also defined by 'tidytree'
```

```
## Found more than one class "phylo" in cache; using the first, from namespace 'phyloseq'
```

```
## Also defined by 'tidytree'
```

```
## Found more than one class "phylo" in cache; using the first, from namespace 'phyloseq'
```

```
## Also defined by 'tidytree'
```

```
## Found more than one class "phylo" in cache; using the first, from namespace 'phyloseq'
```

```
## Also defined by 'tidytree'
```

```
## Found more than one class "phylo" in cache; using the first, from namespace 'phyloseq'
```

```
## Also defined by 'tidytree'
```

```
## Found more than one class "phylo" in cache; using the first, from namespace 'phyloseq'
```

```
## Also defined by 'tidytree'
```

```
## Found more than one class "phylo" in cache; using the first, from namespace 'phyloseq'
```

```
## Also defined by 'tidytree'
```

```
## Found more than one class "phylo" in cache; using the first, from namespace 'phyloseq'
```

```
## Also defined by 'tidytree'
```

```
## Found more than one class "phylo" in cache; using the first, from namespace 'phyloseq'
```

```
## Also defined by 'tidytree'
```

```
## Found more than one class "phylo" in cache; using the first, from namespace 'phyloseq'
```

```
## Also defined by 'tidytree'
```

```
## Found more than one class "phylo" in cache; using the first, from namespace 'phyloseq'
```

```
## Also defined by 'tidytree'
```

```
## Found more than one class "phylo" in cache; using the first, from namespace 'phyloseq'
```

```
## Also defined by 'tidytree'
```

```
## Found more than one class "phylo" in cache; using the first, from namespace 'phyloseq'
```

```
## Also defined by 'tidytree'
```

```
## Found more than one class "phylo" in cache; using the first, from namespace 'phyloseq'
```

```
## Also defined by 'tidytree'
```

```
## Found more than one class "phylo" in cache; using the first, from namespace 'phyloseq'
```

```
## Also defined by 'tidytree'
```

```
## Found more than one class "phylo" in cache; using the first, from namespace 'phyloseq'
```

```
## Also defined by 'tidytree'
```

```
## Found more than one class "phylo" in cache; using the first, from namespace 'phyloseq'
```

```
## Also defined by 'tidytree'
```

```
## Found more than one class "phylo" in cache; using the first, from namespace 'phyloseq'
```

```
## Also defined by 'tidytree'
```

```
## Found more than one class "phylo" in cache; using the first, from namespace 'phyloseq'
```

```
## Also defined by 'tidytree'
```

```
## Found more than one class "phylo" in cache; using the first, from namespace 'phyloseq'
```

```
## Also defined by 'tidytree'
```

```
## Found more than one class "phylo" in cache; using the first, from namespace 'phyloseq'
```

```
## Also defined by 'tidytree'
```

```
## Found more than one class "phylo" in cache; using the first, from namespace 'phyloseq'
```

```
## Also defined by 'tidytree'
```

```
## Found more than one class "phylo" in cache; using the first, from namespace 'phyloseq'
```

```
## Also defined by 'tidytree'
```

```
## Found more than one class "phylo" in cache; using the first, from namespace 'phyloseq'
```

```
## Also defined by 'tidytree'
```

```
## Found more than one class "phylo" in cache; using the first, from namespace 'phyloseq'
```

```
## Also defined by 'tidytree'
```

```
## Found more than one class "phylo" in cache; using the first, from namespace 'phyloseq'
```

```
## Also defined by 'tidytree'
```

```
## Found more than one class "phylo" in cache; using the first, from namespace 'phyloseq'
```

```
## Also defined by 'tidytree'
```

```
## Found more than one class "phylo" in cache; using the first, from namespace 'phyloseq'
```

```
## Also defined by 'tidytree'
```

```
## Found more than one class "phylo" in cache; using the first, from namespace 'phyloseq'
```

```
## Also defined by 'tidytree'
```

```
## Found more than one class "phylo" in cache; using the first, from namespace 'phyloseq'
```

```
## Also defined by 'tidytree'
```

```
## Found more than one class "phylo" in cache; using the first, from namespace 'phyloseq'
```

```
## Also defined by 'tidytree'
```

```
## Found more than one class "phylo" in cache; using the first, from namespace 'phyloseq'
```

```
## Also defined by 'tidytree'
```

```
## Found more than one class "phylo" in cache; using the first, from namespace 'phyloseq'
```

```
## Also defined by 'tidytree'
```

```
## Found more than one class "phylo" in cache; using the first, from namespace 'phyloseq'
```

```
## Also defined by 'tidytree'
```

```
## Found more than one class "phylo" in cache; using the first, from namespace 'phyloseq'
```

```
## Also defined by 'tidytree'
```

```
## Found more than one class "phylo" in cache; using the first, from namespace 'phyloseq'
```

```
## Also defined by 'tidytree'
```

```
## Found more than one class "phylo" in cache; using the first, from namespace 'phyloseq'
```

```
## Also defined by 'tidytree'
```

```
## Found more than one class "phylo" in cache; using the first, from namespace 'phyloseq'
```

```
## Also defined by 'tidytree'
```

```
## Found more than one class "phylo" in cache; using the first, from namespace 'phyloseq'
```

```
## Also defined by 'tidytree'
```

```
## Found more than one class "phylo" in cache; using the first, from namespace 'phyloseq'
```

```
## Also defined by 'tidytree'
```

```
## Found more than one class "phylo" in cache; using the first, from namespace 'phyloseq'
```

```
## Also defined by 'tidytree'
```

```
## Found more than one class "phylo" in cache; using the first, from namespace 'phyloseq'
```

```
## Also defined by 'tidytree'
```

```
## Found more than one class "phylo" in cache; using the first, from namespace 'phyloseq'
```

```
## Also defined by 'tidytree'
```

```
## Found more than one class "phylo" in cache; using the first, from namespace 'phyloseq'
```

```
## Also defined by 'tidytree'
```

```
## Found more than one class "phylo" in cache; using the first, from namespace 'phyloseq'
```

```
## Also defined by 'tidytree'
```

```
## Found more than one class "phylo" in cache; using the first, from namespace 'phyloseq'
```

```
## Also defined by 'tidytree'
```

```
## Found more than one class "phylo" in cache; using the first, from namespace 'phyloseq'
```

```
## Also defined by 'tidytree'
```

```
## Found more than one class "phylo" in cache; using the first, from namespace 'phyloseq'
```

```
## Also defined by 'tidytree'
```

```
## Found more than one class "phylo" in cache; using the first, from namespace 'phyloseq'
```

```
## Also defined by 'tidytree'
```

```
## Found more than one class "phylo" in cache; using the first, from namespace 'phyloseq'
```

```
## Also defined by 'tidytree'
```

```
## Found more than one class "phylo" in cache; using the first, from namespace 'phyloseq'
```

```
## Also defined by 'tidytree'
```

```
## Found more than one class "phylo" in cache; using the first, from namespace 'phyloseq'
```

```
## Also defined by 'tidytree'
```

```
## Found more than one class "phylo" in cache; using the first, from namespace 'phyloseq'
```

```
## Also defined by 'tidytree'
```

```
## Found more than one class "phylo" in cache; using the first, from namespace 'phyloseq'
```

```
## Also defined by 'tidytree'
```

```
## Found more than one class "phylo" in cache; using the first, from namespace 'phyloseq'
```

```
## Also defined by 'tidytree'
```

```
## Found more than one class "phylo" in cache; using the first, from namespace 'phyloseq'
```

```
## Also defined by 'tidytree'
```

```
## Found more than one class "phylo" in cache; using the first, from namespace 'phyloseq'
```

```
## Also defined by 'tidytree'
```

```
## Found more than one class "phylo" in cache; using the first, from namespace 'phyloseq'
```

```
## Also defined by 'tidytree'
```

```
## Found more than one class "phylo" in cache; using the first, from namespace 'phyloseq'
```

```
## Also defined by 'tidytree'
```

```
## Found more than one class "phylo" in cache; using the first, from namespace 'phyloseq'
```

```
## Also defined by 'tidytree'
```

```
## Found more than one class "phylo" in cache; using the first, from namespace 'phyloseq'
```

```
## Also defined by 'tidytree'
```

```
## Found more than one class "phylo" in cache; using the first, from namespace 'phyloseq'
```

```
## Also defined by 'tidytree'
```

```
## Found more than one class "phylo" in cache; using the first, from namespace 'phyloseq'
```

```
## Also defined by 'tidytree'
```

```
## Found more than one class "phylo" in cache; using the first, from namespace 'phyloseq'
```

```
## Also defined by 'tidytree'
```

```
## Found more than one class "phylo" in cache; using the first, from namespace 'phyloseq'
```

```
## Also defined by 'tidytree'
```

```
## Found more than one class "phylo" in cache; using the first, from namespace 'phyloseq'
```

```
## Also defined by 'tidytree'
```

```
## Found more than one class "phylo" in cache; using the first, from namespace 'phyloseq'
```

```
## Also defined by 'tidytree'
```

```
## Found more than one class "phylo" in cache; using the first, from namespace 'phyloseq'
```

```
## Also defined by 'tidytree'
```

```
## Found more than one class "phylo" in cache; using the first, from namespace 'phyloseq'
```

```
## Also defined by 'tidytree'
```

```
## Found more than one class "phylo" in cache; using the first, from namespace 'phyloseq'
```

```
## Also defined by 'tidytree'
```

```
## Found more than one class "phylo" in cache; using the first, from namespace 'phyloseq'
```

```
## Also defined by 'tidytree'
```

```
## Found more than one class "phylo" in cache; using the first, from namespace 'phyloseq'
```

```
## Also defined by 'tidytree'
```

```
## Found more than one class "phylo" in cache; using the first, from namespace 'phyloseq'
```

```
## Also defined by 'tidytree'
```

```
## Found more than one class "phylo" in cache; using the first, from namespace 'phyloseq'
```

```
## Also defined by 'tidytree'
```

```
## Found more than one class "phylo" in cache; using the first, from namespace 'phyloseq'
```

```
## Also defined by 'tidytree'
```

```
## Found more than one class "phylo" in cache; using the first, from namespace 'phyloseq'
```

```
## Also defined by 'tidytree'
```

```
## Found more than one class "phylo" in cache; using the first, from namespace 'phyloseq'
```

```
## Also defined by 'tidytree'
```

```
## Found more than one class "phylo" in cache; using the first, from namespace 'phyloseq'
```

```
## Also defined by 'tidytree'
```

```
## Found more than one class "phylo" in cache; using the first, from namespace 'phyloseq'
```

```
## Also defined by 'tidytree'
```

```
## Found more than one class "phylo" in cache; using the first, from namespace 'phyloseq'
```

```
## Also defined by 'tidytree'
```

```
## Found more than one class "phylo" in cache; using the first, from namespace 'phyloseq'
```

```
## Also defined by 'tidytree'
```

```
## Found more than one class "phylo" in cache; using the first, from namespace 'phyloseq'
```

```
## Also defined by 'tidytree'
```

```
## Found more than one class "phylo" in cache; using the first, from namespace 'phyloseq'
```

```
## Also defined by 'tidytree'
```

```
## Found more than one class "phylo" in cache; using the first, from namespace 'phyloseq'
```

```
## Also defined by 'tidytree'
```

```
## Found more than one class "phylo" in cache; using the first, from namespace 'phyloseq'
```

```
## Also defined by 'tidytree'
```

```
## Found more than one class "phylo" in cache; using the first, from namespace 'phyloseq'
```

```
## Also defined by 'tidytree'
```

```
## Found more than one class "phylo" in cache; using the first, from namespace 'phyloseq'
```

```
## Also defined by 'tidytree'
```

```
## Found more than one class "phylo" in cache; using the first, from namespace 'phyloseq'
```

```
## Also defined by 'tidytree'
```

```
## Found more than one class "phylo" in cache; using the first, from namespace 'phyloseq'
```

```
## Also defined by 'tidytree'
```

```
## Found more than one class "phylo" in cache; using the first, from namespace 'phyloseq'
```

```
## Also defined by 'tidytree'
```

```
## Found more than one class "phylo" in cache; using the first, from namespace 'phyloseq'
```

```
## Also defined by 'tidytree'
```

```
## Found more than one class "phylo" in cache; using the first, from namespace 'phyloseq'
```

```
## Also defined by 'tidytree'
```

```
## Found more than one class "phylo" in cache; using the first, from namespace 'phyloseq'
```

```
## Also defined by 'tidytree'
```

```
## Found more than one class "phylo" in cache; using the first, from namespace 'phyloseq'
```

```
## Also defined by 'tidytree'
```

```
## Found more than one class "phylo" in cache; using the first, from namespace 'phyloseq'
```

```
## Also defined by 'tidytree'
```

```
## Found more than one class "phylo" in cache; using the first, from namespace 'phyloseq'
```

```
## Also defined by 'tidytree'
```

```
## Found more than one class "phylo" in cache; using the first, from namespace 'phyloseq'
```

```
## Also defined by 'tidytree'
```

```
## Found more than one class "phylo" in cache; using the first, from namespace 'phyloseq'
```

```
## Also defined by 'tidytree'
```

```
## Found more than one class "phylo" in cache; using the first, from namespace 'phyloseq'
```

```
## Also defined by 'tidytree'
```

```
## Found more than one class "phylo" in cache; using the first, from namespace 'phyloseq'
```

```
## Also defined by 'tidytree'
```

```
## Found more than one class "phylo" in cache; using the first, from namespace 'phyloseq'
```

```
## Also defined by 'tidytree'
```

```
## Found more than one class "phylo" in cache; using the first, from namespace 'phyloseq'
```

```
## Also defined by 'tidytree'
```

```
## Found more than one class "phylo" in cache; using the first, from namespace 'phyloseq'
```

```
## Also defined by 'tidytree'
```

```
## Found more than one class "phylo" in cache; using the first, from namespace 'phyloseq'
```

```
## Also defined by 'tidytree'
```

```
## Found more than one class "phylo" in cache; using the first, from namespace 'phyloseq'
```

```
## Also defined by 'tidytree'
```

```
## Found more than one class "phylo" in cache; using the first, from namespace 'phyloseq'
```

```
## Also defined by 'tidytree'
```

```
## Found more than one class "phylo" in cache; using the first, from namespace 'phyloseq'
```

```
## Also defined by 'tidytree'
```

```
## Found more than one class "phylo" in cache; using the first, from namespace 'phyloseq'
```

```
## Also defined by 'tidytree'
```

```
## Found more than one class "phylo" in cache; using the first, from namespace 'phyloseq'
```

```
## Also defined by 'tidytree'
```

```
## Found more than one class "phylo" in cache; using the first, from namespace 'phyloseq'
```

```
## Also defined by 'tidytree'
```

```
## Found more than one class "phylo" in cache; using the first, from namespace 'phyloseq'
```

```
## Also defined by 'tidytree'
```

```
## Found more than one class "phylo" in cache; using the first, from namespace 'phyloseq'
```

```
## Also defined by 'tidytree'
```

```
## Found more than one class "phylo" in cache; using the first, from namespace 'phyloseq'
```

```
## Also defined by 'tidytree'
```

```
## Found more than one class "phylo" in cache; using the first, from namespace 'phyloseq'
```

```
## Also defined by 'tidytree'
```

```
## Found more than one class "phylo" in cache; using the first, from namespace 'phyloseq'
```

```
## Also defined by 'tidytree'
```

```
## Found more than one class "phylo" in cache; using the first, from namespace 'phyloseq'
```

```
## Also defined by 'tidytree'
```

```
## Found more than one class "phylo" in cache; using the first, from namespace 'phyloseq'
```

```
## Also defined by 'tidytree'
```

```
## Found more than one class "phylo" in cache; using the first, from namespace 'phyloseq'
```

```
## Also defined by 'tidytree'
```

```
## Found more than one class "phylo" in cache; using the first, from namespace 'phyloseq'
```

```
## Also defined by 'tidytree'
```

```
## Found more than one class "phylo" in cache; using the first, from namespace 'phyloseq'
```

```
## Also defined by 'tidytree'
```

```
## Found more than one class "phylo" in cache; using the first, from namespace 'phyloseq'
```

```
## Also defined by 'tidytree'
```

```
## Found more than one class "phylo" in cache; using the first, from namespace 'phyloseq'
```

```
## Also defined by 'tidytree'
```

```
## Found more than one class "phylo" in cache; using the first, from namespace 'phyloseq'
```

```
## Also defined by 'tidytree'
```

```
## Found more than one class "phylo" in cache; using the first, from namespace 'phyloseq'
```

```
## Also defined by 'tidytree'
```

```
## Found more than one class "phylo" in cache; using the first, from namespace 'phyloseq'
```

```
## Also defined by 'tidytree'
```

```
## Found more than one class "phylo" in cache; using the first, from namespace 'phyloseq'
```

```
## Also defined by 'tidytree'
```

```
## Found more than one class "phylo" in cache; using the first, from namespace 'phyloseq'
```

```
## Also defined by 'tidytree'
```

```
## Found more than one class "phylo" in cache; using the first, from namespace 'phyloseq'
```

```
## Also defined by 'tidytree'
```

```
## Found more than one class "phylo" in cache; using the first, from namespace 'phyloseq'
```

```
## Also defined by 'tidytree'
```

```
## Found more than one class "phylo" in cache; using the first, from namespace 'phyloseq'
```

```
## Also defined by 'tidytree'
```

```
## Found more than one class "phylo" in cache; using the first, from namespace 'phyloseq'
```

```
## Also defined by 'tidytree'
```

```
## Found more than one class "phylo" in cache; using the first, from namespace 'phyloseq'
```

```
## Also defined by 'tidytree'
```

```
## Found more than one class "phylo" in cache; using the first, from namespace 'phyloseq'
```

```
## Also defined by 'tidytree'
```

```
## Found more than one class "phylo" in cache; using the first, from namespace 'phyloseq'
```

```
## Also defined by 'tidytree'
```

```
## Found more than one class "phylo" in cache; using the first, from namespace 'phyloseq'
```

```
## Also defined by 'tidytree'
```

```
## Found more than one class "phylo" in cache; using the first, from namespace 'phyloseq'
```

```
## Also defined by 'tidytree'
```

```
## Found more than one class "phylo" in cache; using the first, from namespace 'phyloseq'
```

```
## Also defined by 'tidytree'
```

```
## Found more than one class "phylo" in cache; using the first, from namespace 'phyloseq'
```

```
## Also defined by 'tidytree'
```

```
## Found more than one class "phylo" in cache; using the first, from namespace 'phyloseq'
```

```
## Also defined by 'tidytree'
```

```
## Found more than one class "phylo" in cache; using the first, from namespace 'phyloseq'
```

```
## Also defined by 'tidytree'
```

```
## Found more than one class "phylo" in cache; using the first, from namespace 'phyloseq'
```

```
## Also defined by 'tidytree'
```

```
## Found more than one class "phylo" in cache; using the first, from namespace 'phyloseq'
```

```
## Also defined by 'tidytree'
```

```
## Found more than one class "phylo" in cache; using the first, from namespace 'phyloseq'
```

```
## Also defined by 'tidytree'
```

```
## Found more than one class "phylo" in cache; using the first, from namespace 'phyloseq'
```

```
## Also defined by 'tidytree'
```

```
## Found more than one class "phylo" in cache; using the first, from namespace 'phyloseq'
```

```
## Also defined by 'tidytree'
```

```
## Found more than one class "phylo" in cache; using the first, from namespace 'phyloseq'
```

```
## Also defined by 'tidytree'
```

```
## Found more than one class "phylo" in cache; using the first, from namespace 'phyloseq'
```

```
## Also defined by 'tidytree'
```

```
## Found more than one class "phylo" in cache; using the first, from namespace 'phyloseq'
```

```
## Also defined by 'tidytree'
```

```
## Found more than one class "phylo" in cache; using the first, from namespace 'phyloseq'
```

```
## Also defined by 'tidytree'
```

```
## Found more than one class "phylo" in cache; using the first, from namespace 'phyloseq'
```

```
## Also defined by 'tidytree'
```

```
## Found more than one class "phylo" in cache; using the first, from namespace 'phyloseq'
```

```
## Also defined by 'tidytree'
```

```
## Found more than one class "phylo" in cache; using the first, from namespace 'phyloseq'
```

```
## Also defined by 'tidytree'
```

```
## Found more than one class "phylo" in cache; using the first, from namespace 'phyloseq'
```

```
## Also defined by 'tidytree'
```

```
## Found more than one class "phylo" in cache; using the first, from namespace 'phyloseq'
```

```
## Also defined by 'tidytree'
```

```
## Found more than one class "phylo" in cache; using the first, from namespace 'phyloseq'
```

```
## Also defined by 'tidytree'
```

```
## Found more than one class "phylo" in cache; using the first, from namespace 'phyloseq'
```

```
## Also defined by 'tidytree'
```

```
## Found more than one class "phylo" in cache; using the first, from namespace 'phyloseq'
```

```
## Also defined by 'tidytree'
```

```
## Found more than one class "phylo" in cache; using the first, from namespace 'phyloseq'
```

```
## Also defined by 'tidytree'
```

```
## Found more than one class "phylo" in cache; using the first, from namespace 'phyloseq'
```

```
## Also defined by 'tidytree'
```

```
## Found more than one class "phylo" in cache; using the first, from namespace 'phyloseq'
```

```
## Also defined by 'tidytree'
```

```
## Found more than one class "phylo" in cache; using the first, from namespace 'phyloseq'
```

```
## Also defined by 'tidytree'
```

```
## Found more than one class "phylo" in cache; using the first, from namespace 'phyloseq'
```

```
## Also defined by 'tidytree'
```

```
## Found more than one class "phylo" in cache; using the first, from namespace 'phyloseq'
```

```
## Also defined by 'tidytree'
```

```
## Found more than one class "phylo" in cache; using the first, from namespace 'phyloseq'
```

```
## Also defined by 'tidytree'
```

```
## Found more than one class "phylo" in cache; using the first, from namespace 'phyloseq'
```

```
## Also defined by 'tidytree'
```

```
## Found more than one class "phylo" in cache; using the first, from namespace 'phyloseq'
```

```
## Also defined by 'tidytree'
```

```
## Found more than one class "phylo" in cache; using the first, from namespace 'phyloseq'
```

```
## Also defined by 'tidytree'
```

```
## Found more than one class "phylo" in cache; using the first, from namespace 'phyloseq'
```

```
## Also defined by 'tidytree'
```

```
## Found more than one class "phylo" in cache; using the first, from namespace 'phyloseq'
```

```
## Also defined by 'tidytree'
```

```
## Found more than one class "phylo" in cache; using the first, from namespace 'phyloseq'
```

```
## Also defined by 'tidytree'
```

```
## Found more than one class "phylo" in cache; using the first, from namespace 'phyloseq'
```

```
## Also defined by 'tidytree'
```

```
## Found more than one class "phylo" in cache; using the first, from namespace 'phyloseq'
```

```
## Also defined by 'tidytree'
```

```
## Found more than one class "phylo" in cache; using the first, from namespace 'phyloseq'
```

```
## Also defined by 'tidytree'
```

```
## Found more than one class "phylo" in cache; using the first, from namespace 'phyloseq'
```

```
## Also defined by 'tidytree'
```

```
## Found more than one class "phylo" in cache; using the first, from namespace 'phyloseq'
```

```
## Also defined by 'tidytree'
```

```
## Found more than one class "phylo" in cache; using the first, from namespace 'phyloseq'
```

```
## Also defined by 'tidytree'
```

```
## Found more than one class "phylo" in cache; using the first, from namespace 'phyloseq'
```

```
## Also defined by 'tidytree'
```

```
## Found more than one class "phylo" in cache; using the first, from namespace 'phyloseq'
```

```
## Also defined by 'tidytree'
```

```
## Found more than one class "phylo" in cache; using the first, from namespace 'phyloseq'
```

```
## Also defined by 'tidytree'
```

```
## Found more than one class "phylo" in cache; using the first, from namespace 'phyloseq'
```

```
## Also defined by 'tidytree'
```

```
## Found more than one class "phylo" in cache; using the first, from namespace 'phyloseq'
```

```
## Also defined by 'tidytree'
```

```
## Found more than one class "phylo" in cache; using the first, from namespace 'phyloseq'
```

```
## Also defined by 'tidytree'
```

```
## Found more than one class "phylo" in cache; using the first, from namespace 'phyloseq'
```

```
## Also defined by 'tidytree'
```

```
## Found more than one class "phylo" in cache; using the first, from namespace 'phyloseq'
```

```
## Also defined by 'tidytree'
```

```
## Found more than one class "phylo" in cache; using the first, from namespace 'phyloseq'
```

```
## Also defined by 'tidytree'
```

```
## Found more than one class "phylo" in cache; using the first, from namespace 'phyloseq'
```

```
## Also defined by 'tidytree'
```

```
## Found more than one class "phylo" in cache; using the first, from namespace 'phyloseq'
```

```
## Also defined by 'tidytree'
```

```
## Found more than one class "phylo" in cache; using the first, from namespace 'phyloseq'
```

```
## Also defined by 'tidytree'
```

```
## Found more than one class "phylo" in cache; using the first, from namespace 'phyloseq'
```

```
## Also defined by 'tidytree'
```

```
## Found more than one class "phylo" in cache; using the first, from namespace 'phyloseq'
```

```
## Also defined by 'tidytree'
```

```
## Found more than one class "phylo" in cache; using the first, from namespace 'phyloseq'
```

```
## Also defined by 'tidytree'
```

```
## Found more than one class "phylo" in cache; using the first, from namespace 'phyloseq'
```

```
## Also defined by 'tidytree'
```

```
## Found more than one class "phylo" in cache; using the first, from namespace 'phyloseq'
```

```
## Also defined by 'tidytree'
```

```
## Found more than one class "phylo" in cache; using the first, from namespace 'phyloseq'
```

```
## Also defined by 'tidytree'
```

```
## Found more than one class "phylo" in cache; using the first, from namespace 'phyloseq'
```

```
## Also defined by 'tidytree'
```

```
## Found more than one class "phylo" in cache; using the first, from namespace 'phyloseq'
```

```
## Also defined by 'tidytree'
```

```
## Found more than one class "phylo" in cache; using the first, from namespace 'phyloseq'
```

```
## Also defined by 'tidytree'
```

```
## Found more than one class "phylo" in cache; using the first, from namespace 'phyloseq'
```

```
## Also defined by 'tidytree'
```

```
## Found more than one class "phylo" in cache; using the first, from namespace 'phyloseq'
```

```
## Also defined by 'tidytree'
```

```
## Found more than one class "phylo" in cache; using the first, from namespace 'phyloseq'
```

```
## Also defined by 'tidytree'
```

```
## Found more than one class "phylo" in cache; using the first, from namespace 'phyloseq'
```

```
## Also defined by 'tidytree'
```

```
## Found more than one class "phylo" in cache; using the first, from namespace 'phyloseq'
```

```
## Also defined by 'tidytree'
```

```
## Found more than one class "phylo" in cache; using the first, from namespace 'phyloseq'
```

```
## Also defined by 'tidytree'
```

```
## Found more than one class "phylo" in cache; using the first, from namespace 'phyloseq'
```

```
## Also defined by 'tidytree'
```

```
## Found more than one class "phylo" in cache; using the first, from namespace 'phyloseq'
```

```
## Also defined by 'tidytree'
```

```
## Found more than one class "phylo" in cache; using the first, from namespace 'phyloseq'
```

```
## Also defined by 'tidytree'
```

```
## Found more than one class "phylo" in cache; using the first, from namespace 'phyloseq'
```

```
## Also defined by 'tidytree'
```

```
## Found more than one class "phylo" in cache; using the first, from namespace 'phyloseq'
```

```
## Also defined by 'tidytree'
```

```
## Found more than one class "phylo" in cache; using the first, from namespace 'phyloseq'
```

```
## Also defined by 'tidytree'
```

```
## Found more than one class "phylo" in cache; using the first, from namespace 'phyloseq'
```

```
## Also defined by 'tidytree'
```

```
## Found more than one class "phylo" in cache; using the first, from namespace 'phyloseq'
```

```
## Also defined by 'tidytree'
```

```
## Found more than one class "phylo" in cache; using the first, from namespace 'phyloseq'
```

```
## Also defined by 'tidytree'
```

```
## Found more than one class "phylo" in cache; using the first, from namespace 'phyloseq'
```

```
## Also defined by 'tidytree'
```

```
## Found more than one class "phylo" in cache; using the first, from namespace 'phyloseq'
```

```
## Also defined by 'tidytree'
```

```
## Found more than one class "phylo" in cache; using the first, from namespace 'phyloseq'
```

```
## Also defined by 'tidytree'
```

```
## Found more than one class "phylo" in cache; using the first, from namespace 'phyloseq'
```

```
## Also defined by 'tidytree'
```

```
## Found more than one class "phylo" in cache; using the first, from namespace 'phyloseq'
```

```
## Also defined by 'tidytree'
```

```
## Found more than one class "phylo" in cache; using the first, from namespace 'phyloseq'
```

```
## Also defined by 'tidytree'
```

```
## Found more than one class "phylo" in cache; using the first, from namespace 'phyloseq'
```

```
## Also defined by 'tidytree'
```

```
## Found more than one class "phylo" in cache; using the first, from namespace 'phyloseq'
```

```
## Also defined by 'tidytree'
```

```
## Found more than one class "phylo" in cache; using the first, from namespace 'phyloseq'
```

```
## Also defined by 'tidytree'
```

```
## Found more than one class "phylo" in cache; using the first, from namespace 'phyloseq'
```

```
## Also defined by 'tidytree'
```

```
## Found more than one class "phylo" in cache; using the first, from namespace 'phyloseq'
```

```
## Also defined by 'tidytree'
```

```
## Found more than one class "phylo" in cache; using the first, from namespace 'phyloseq'
```

```
## Also defined by 'tidytree'
```

```
## Found more than one class "phylo" in cache; using the first, from namespace 'phyloseq'
```

```
## Also defined by 'tidytree'
```

```
## Found more than one class "phylo" in cache; using the first, from namespace 'phyloseq'
```

```
## Also defined by 'tidytree'
```

```
## Found more than one class "phylo" in cache; using the first, from namespace 'phyloseq'
```

```
## Also defined by 'tidytree'
```

```
## Found more than one class "phylo" in cache; using the first, from namespace 'phyloseq'
```

```
## Also defined by 'tidytree'
```

```
## Found more than one class "phylo" in cache; using the first, from namespace 'phyloseq'
```

```
## Also defined by 'tidytree'
```

```
## Found more than one class "phylo" in cache; using the first, from namespace 'phyloseq'
```

```
## Also defined by 'tidytree'
```

```
## Found more than one class "phylo" in cache; using the first, from namespace 'phyloseq'
```

```
## Also defined by 'tidytree'
```

```
## Found more than one class "phylo" in cache; using the first, from namespace 'phyloseq'
```

```
## Also defined by 'tidytree'
```

```
## Found more than one class "phylo" in cache; using the first, from namespace 'phyloseq'
```

```
## Also defined by 'tidytree'
```

```
## Found more than one class "phylo" in cache; using the first, from namespace 'phyloseq'
```

```
## Also defined by 'tidytree'
```

```
## Found more than one class "phylo" in cache; using the first, from namespace 'phyloseq'
```

```
## Also defined by 'tidytree'
```

```
## Found more than one class "phylo" in cache; using the first, from namespace 'phyloseq'
```

```
## Also defined by 'tidytree'
```

```
## Found more than one class "phylo" in cache; using the first, from namespace 'phyloseq'
```

```
## Also defined by 'tidytree'
```

```
## Found more than one class "phylo" in cache; using the first, from namespace 'phyloseq'
```

```
## Also defined by 'tidytree'
```

```
## Found more than one class "phylo" in cache; using the first, from namespace 'phyloseq'
```

```
## Also defined by 'tidytree'
```

```
## Found more than one class "phylo" in cache; using the first, from namespace 'phyloseq'
```

```
## Also defined by 'tidytree'
```

```
## Found more than one class "phylo" in cache; using the first, from namespace 'phyloseq'
```

```
## Also defined by 'tidytree'
```

```
## Found more than one class "phylo" in cache; using the first, from namespace 'phyloseq'
```

```
## Also defined by 'tidytree'
```

```
## Found more than one class "phylo" in cache; using the first, from namespace 'phyloseq'
```

```
## Also defined by 'tidytree'
```

```
## Found more than one class "phylo" in cache; using the first, from namespace 'phyloseq'
```

```
## Also defined by 'tidytree'
```

```
## Found more than one class "phylo" in cache; using the first, from namespace 'phyloseq'
```

```
## Also defined by 'tidytree'
```

```
## Found more than one class "phylo" in cache; using the first, from namespace 'phyloseq'
```

```
## Also defined by 'tidytree'
```

```
## Found more than one class "phylo" in cache; using the first, from namespace 'phyloseq'
```

```
## Also defined by 'tidytree'
```

```
## Found more than one class "phylo" in cache; using the first, from namespace 'phyloseq'
```

```
## Also defined by 'tidytree'
```

```
## Found more than one class "phylo" in cache; using the first, from namespace 'phyloseq'
```

```
## Also defined by 'tidytree'
```

```
## Found more than one class "phylo" in cache; using the first, from namespace 'phyloseq'
```

```
## Also defined by 'tidytree'
```

```
## Found more than one class "phylo" in cache; using the first, from namespace 'phyloseq'
```

```
## Also defined by 'tidytree'
```

```
## Found more than one class "phylo" in cache; using the first, from namespace 'phyloseq'
```

```
## Also defined by 'tidytree'
```

```
## Found more than one class "phylo" in cache; using the first, from namespace 'phyloseq'
```

```
## Also defined by 'tidytree'
```

```
## Found more than one class "phylo" in cache; using the first, from namespace 'phyloseq'
```

```
## Also defined by 'tidytree'
```

```
## Found more than one class "phylo" in cache; using the first, from namespace 'phyloseq'
```

```
## Also defined by 'tidytree'
```

```
## Found more than one class "phylo" in cache; using the first, from namespace 'phyloseq'
```

```
## Also defined by 'tidytree'
```

```
## Found more than one class "phylo" in cache; using the first, from namespace 'phyloseq'
```

```
## Also defined by 'tidytree'
```

```
## Found more than one class "phylo" in cache; using the first, from namespace 'phyloseq'
```

```
## Also defined by 'tidytree'
```

```
## Found more than one class "phylo" in cache; using the first, from namespace 'phyloseq'
```

```
## Also defined by 'tidytree'
```

```
## Found more than one class "phylo" in cache; using the first, from namespace 'phyloseq'
```

```
## Also defined by 'tidytree'
```

```
## Found more than one class "phylo" in cache; using the first, from namespace 'phyloseq'
```

```
## Also defined by 'tidytree'
```

```
## Found more than one class "phylo" in cache; using the first, from namespace 'phyloseq'
```

```
## Also defined by 'tidytree'
```

```
## Found more than one class "phylo" in cache; using the first, from namespace 'phyloseq'
```

```
## Also defined by 'tidytree'
```

```
## Found more than one class "phylo" in cache; using the first, from namespace 'phyloseq'
```

```
## Also defined by 'tidytree'
```

```
## Found more than one class "phylo" in cache; using the first, from namespace 'phyloseq'
```

```
## Also defined by 'tidytree'
```

```
## Found more than one class "phylo" in cache; using the first, from namespace 'phyloseq'
```

```
## Also defined by 'tidytree'
```

```
## Found more than one class "phylo" in cache; using the first, from namespace 'phyloseq'
```

```
## Also defined by 'tidytree'
```

```
## Found more than one class "phylo" in cache; using the first, from namespace 'phyloseq'
```

```
## Also defined by 'tidytree'
```

```
## Found more than one class "phylo" in cache; using the first, from namespace 'phyloseq'
```

```
## Also defined by 'tidytree'
```

```
## Found more than one class "phylo" in cache; using the first, from namespace 'phyloseq'
```

```
## Also defined by 'tidytree'
```

```
## Found more than one class "phylo" in cache; using the first, from namespace 'phyloseq'
```

```
## Also defined by 'tidytree'
```

```
## Found more than one class "phylo" in cache; using the first, from namespace 'phyloseq'
```

```
## Also defined by 'tidytree'
```

```
## Found more than one class "phylo" in cache; using the first, from namespace 'phyloseq'
```

```
## Also defined by 'tidytree'
```

```
## Found more than one class "phylo" in cache; using the first, from namespace 'phyloseq'
```

```
## Also defined by 'tidytree'
```

```
## Found more than one class "phylo" in cache; using the first, from namespace 'phyloseq'
```

```
## Also defined by 'tidytree'
```

```
## Found more than one class "phylo" in cache; using the first, from namespace 'phyloseq'
```

```
## Also defined by 'tidytree'
```

```
## Found more than one class "phylo" in cache; using the first, from namespace 'phyloseq'
```

```
## Also defined by 'tidytree'
```

```
## Found more than one class "phylo" in cache; using the first, from namespace 'phyloseq'
```

```
## Also defined by 'tidytree'
```

```
## Found more than one class "phylo" in cache; using the first, from namespace 'phyloseq'
```

```
## Also defined by 'tidytree'
```

```
## Found more than one class "phylo" in cache; using the first, from namespace 'phyloseq'
```

```
## Also defined by 'tidytree'
```

```
## Found more than one class "phylo" in cache; using the first, from namespace 'phyloseq'
```

```
## Also defined by 'tidytree'
```

```
## Found more than one class "phylo" in cache; using the first, from namespace 'phyloseq'
```

```
## Also defined by 'tidytree'
```

```
## Found more than one class "phylo" in cache; using the first, from namespace 'phyloseq'
```

```
## Also defined by 'tidytree'
```

```
## Found more than one class "phylo" in cache; using the first, from namespace 'phyloseq'
```

```
## Also defined by 'tidytree'
```

```
## Found more than one class "phylo" in cache; using the first, from namespace 'phyloseq'
```

```
## Also defined by 'tidytree'
```

```
## Found more than one class "phylo" in cache; using the first, from namespace 'phyloseq'
```

```
## Also defined by 'tidytree'
```

```
## Found more than one class "phylo" in cache; using the first, from namespace 'phyloseq'
```

```
## Also defined by 'tidytree'
```

```
## Found more than one class "phylo" in cache; using the first, from namespace 'phyloseq'
```

```
## Also defined by 'tidytree'
```

```
## Found more than one class "phylo" in cache; using the first, from namespace 'phyloseq'
```

```
## Also defined by 'tidytree'
```

```
## Found more than one class "phylo" in cache; using the first, from namespace 'phyloseq'
```

```
## Also defined by 'tidytree'
```

```
## Found more than one class "phylo" in cache; using the first, from namespace 'phyloseq'
```

```
## Also defined by 'tidytree'
```

```
## Found more than one class "phylo" in cache; using the first, from namespace 'phyloseq'
```

```
## Also defined by 'tidytree'
```

```
## Found more than one class "phylo" in cache; using the first, from namespace 'phyloseq'
```

```
## Also defined by 'tidytree'
```

```
## Found more than one class "phylo" in cache; using the first, from namespace 'phyloseq'
```

```
## Also defined by 'tidytree'
```

```
## Found more than one class "phylo" in cache; using the first, from namespace 'phyloseq'
```

```
## Also defined by 'tidytree'
```

```
## Found more than one class "phylo" in cache; using the first, from namespace 'phyloseq'
```

```
## Also defined by 'tidytree'
```

```
## Found more than one class "phylo" in cache; using the first, from namespace 'phyloseq'
```

```
## Also defined by 'tidytree'
```

```
## Found more than one class "phylo" in cache; using the first, from namespace 'phyloseq'
```

```
## Also defined by 'tidytree'
```

```
## Found more than one class "phylo" in cache; using the first, from namespace 'phyloseq'
```

```
## Also defined by 'tidytree'
```

```
## Found more than one class "phylo" in cache; using the first, from namespace 'phyloseq'
```

```
## Also defined by 'tidytree'
```

```
## Found more than one class "phylo" in cache; using the first, from namespace 'phyloseq'
```

```
## Also defined by 'tidytree'
```

```
## Found more than one class "phylo" in cache; using the first, from namespace 'phyloseq'
```

```
## Also defined by 'tidytree'
```

```
## Found more than one class "phylo" in cache; using the first, from namespace 'phyloseq'
```

```
## Also defined by 'tidytree'
```

```
## Found more than one class "phylo" in cache; using the first, from namespace 'phyloseq'
```

```
## Also defined by 'tidytree'
```

```
## Found more than one class "phylo" in cache; using the first, from namespace 'phyloseq'
```

```
## Also defined by 'tidytree'
```

```
## Found more than one class "phylo" in cache; using the first, from namespace 'phyloseq'
```

```
## Also defined by 'tidytree'
```

```
## Found more than one class "phylo" in cache; using the first, from namespace 'phyloseq'
```

```
## Also defined by 'tidytree'
```

```
## Found more than one class "phylo" in cache; using the first, from namespace 'phyloseq'
```

```
## Also defined by 'tidytree'
```

```
## Found more than one class "phylo" in cache; using the first, from namespace 'phyloseq'
```

```
## Also defined by 'tidytree'
```

```
## Found more than one class "phylo" in cache; using the first, from namespace 'phyloseq'
```

```
## Also defined by 'tidytree'
```

```
## Found more than one class "phylo" in cache; using the first, from namespace 'phyloseq'
```

```
## Also defined by 'tidytree'
```

```
## Found more than one class "phylo" in cache; using the first, from namespace 'phyloseq'
```

```
## Also defined by 'tidytree'
```

```
## Found more than one class "phylo" in cache; using the first, from namespace 'phyloseq'
```

```
## Also defined by 'tidytree'
```

```
## Found more than one class "phylo" in cache; using the first, from namespace 'phyloseq'
```

```
## Also defined by 'tidytree'
```

```
## Found more than one class "phylo" in cache; using the first, from namespace 'phyloseq'
```

```
## Also defined by 'tidytree'
```

```
## Found more than one class "phylo" in cache; using the first, from namespace 'phyloseq'
```

```
## Also defined by 'tidytree'
```

```
## Found more than one class "phylo" in cache; using the first, from namespace 'phyloseq'
```

```
## Also defined by 'tidytree'
```

```
## Found more than one class "phylo" in cache; using the first, from namespace 'phyloseq'
```

```
## Also defined by 'tidytree'
```

```
## Found more than one class "phylo" in cache; using the first, from namespace 'phyloseq'
```

```
## Also defined by 'tidytree'
```

```
## Found more than one class "phylo" in cache; using the first, from namespace 'phyloseq'
```

```
## Also defined by 'tidytree'
```

```
## Found more than one class "phylo" in cache; using the first, from namespace 'phyloseq'
```

```
## Also defined by 'tidytree'
```

```
## Found more than one class "phylo" in cache; using the first, from namespace 'phyloseq'
```

```
## Also defined by 'tidytree'
```

```
## Found more than one class "phylo" in cache; using the first, from namespace 'phyloseq'
```

```
## Also defined by 'tidytree'
```

```
## Found more than one class "phylo" in cache; using the first, from namespace 'phyloseq'
```

```
## Also defined by 'tidytree'
```

```
## Found more than one class "phylo" in cache; using the first, from namespace 'phyloseq'
```

```
## Also defined by 'tidytree'
```

```
## Found more than one class "phylo" in cache; using the first, from namespace 'phyloseq'
```

```
## Also defined by 'tidytree'
```

```
## Found more than one class "phylo" in cache; using the first, from namespace 'phyloseq'
```

```
## Also defined by 'tidytree'
```

```
## Found more than one class "phylo" in cache; using the first, from namespace 'phyloseq'
```

```
## Also defined by 'tidytree'
```

```
## Found more than one class "phylo" in cache; using the first, from namespace 'phyloseq'
```

```
## Also defined by 'tidytree'
```

```
## Found more than one class "phylo" in cache; using the first, from namespace 'phyloseq'
```

```
## Also defined by 'tidytree'
```

```
## Found more than one class "phylo" in cache; using the first, from namespace 'phyloseq'
```

```
## Also defined by 'tidytree'
```

```
## Found more than one class "phylo" in cache; using the first, from namespace 'phyloseq'
```

```
## Also defined by 'tidytree'
```

```
## Found more than one class "phylo" in cache; using the first, from namespace 'phyloseq'
```

```
## Also defined by 'tidytree'
```

```
## Found more than one class "phylo" in cache; using the first, from namespace 'phyloseq'
```

```
## Also defined by 'tidytree'
```

```
## Found more than one class "phylo" in cache; using the first, from namespace 'phyloseq'
```

```
## Also defined by 'tidytree'
```

```
## Found more than one class "phylo" in cache; using the first, from namespace 'phyloseq'
```

```
## Also defined by 'tidytree'
```

```
## Found more than one class "phylo" in cache; using the first, from namespace 'phyloseq'
```

```
## Also defined by 'tidytree'
```

```
## Found more than one class "phylo" in cache; using the first, from namespace 'phyloseq'
```

```
## Also defined by 'tidytree'
```

```
## Found more than one class "phylo" in cache; using the first, from namespace 'phyloseq'
```

```
## Also defined by 'tidytree'
```

```
## Found more than one class "phylo" in cache; using the first, from namespace 'phyloseq'
```

```
## Also defined by 'tidytree'
```

```
## Found more than one class "phylo" in cache; using the first, from namespace 'phyloseq'
```

```
## Also defined by 'tidytree'
```

```
## Found more than one class "phylo" in cache; using the first, from namespace 'phyloseq'
```

```
## Also defined by 'tidytree'
```

```
## Found more than one class "phylo" in cache; using the first, from namespace 'phyloseq'
```

```
## Also defined by 'tidytree'
```

```
## Found more than one class "phylo" in cache; using the first, from namespace 'phyloseq'
```

```
## Also defined by 'tidytree'
```

```
## Found more than one class "phylo" in cache; using the first, from namespace 'phyloseq'
```

```
## Also defined by 'tidytree'
```

```
## Found more than one class "phylo" in cache; using the first, from namespace 'phyloseq'
```

```
## Also defined by 'tidytree'
```

```
## Found more than one class "phylo" in cache; using the first, from namespace 'phyloseq'
```

```
## Also defined by 'tidytree'
```

```
## Found more than one class "phylo" in cache; using the first, from namespace 'phyloseq'
```

```
## Also defined by 'tidytree'
```

```
## Found more than one class "phylo" in cache; using the first, from namespace 'phyloseq'
```

```
## Also defined by 'tidytree'
```

```
## Found more than one class "phylo" in cache; using the first, from namespace 'phyloseq'
```

```
## Also defined by 'tidytree'
```

```
## Found more than one class "phylo" in cache; using the first, from namespace 'phyloseq'
```

```
## Also defined by 'tidytree'
```

```
## Found more than one class "phylo" in cache; using the first, from namespace 'phyloseq'
```

```
## Also defined by 'tidytree'
```

```
## Found more than one class "phylo" in cache; using the first, from namespace 'phyloseq'
```

```
## Also defined by 'tidytree'
```

```
## Found more than one class "phylo" in cache; using the first, from namespace 'phyloseq'
```

```
## Also defined by 'tidytree'
```

```
## Found more than one class "phylo" in cache; using the first, from namespace 'phyloseq'
```

```
## Also defined by 'tidytree'
```

```
## Found more than one class "phylo" in cache; using the first, from namespace 'phyloseq'
```

```
## Also defined by 'tidytree'
```

```
## Found more than one class "phylo" in cache; using the first, from namespace 'phyloseq'
```

```
## Also defined by 'tidytree'
```

```
## Found more than one class "phylo" in cache; using the first, from namespace 'phyloseq'
```

```
## Also defined by 'tidytree'
```

```
## Found more than one class "phylo" in cache; using the first, from namespace 'phyloseq'
```

```
## Also defined by 'tidytree'
```

```
## Found more than one class "phylo" in cache; using the first, from namespace 'phyloseq'
```

```
## Also defined by 'tidytree'
```

```
## Found more than one class "phylo" in cache; using the first, from namespace 'phyloseq'
```

```
## Also defined by 'tidytree'
```

```
## Found more than one class "phylo" in cache; using the first, from namespace 'phyloseq'
```

```
## Also defined by 'tidytree'
```

```
## Found more than one class "phylo" in cache; using the first, from namespace 'phyloseq'
```

```
## Also defined by 'tidytree'
```

```
## Found more than one class "phylo" in cache; using the first, from namespace 'phyloseq'
```

```
## Also defined by 'tidytree'
```

```
## Found more than one class "phylo" in cache; using the first, from namespace 'phyloseq'
```

```
## Also defined by 'tidytree'
```

```
## Found more than one class "phylo" in cache; using the first, from namespace 'phyloseq'
```

```
## Also defined by 'tidytree'
```

```
## Found more than one class "phylo" in cache; using the first, from namespace 'phyloseq'
```

```
## Also defined by 'tidytree'
```

```
## Found more than one class "phylo" in cache; using the first, from namespace 'phyloseq'
```

```
## Also defined by 'tidytree'
```

```
## Found more than one class "phylo" in cache; using the first, from namespace 'phyloseq'
```

```
## Also defined by 'tidytree'
```

```
## Found more than one class "phylo" in cache; using the first, from namespace 'phyloseq'
```

```
## Also defined by 'tidytree'
```

```
## Found more than one class "phylo" in cache; using the first, from namespace 'phyloseq'
```

```
## Also defined by 'tidytree'
```

```
## Found more than one class "phylo" in cache; using the first, from namespace 'phyloseq'
```

```
## Also defined by 'tidytree'
```

```
## Found more than one class "phylo" in cache; using the first, from namespace 'phyloseq'
```

```
## Also defined by 'tidytree'
```

```
## Found more than one class "phylo" in cache; using the first, from namespace 'phyloseq'
```

```
## Also defined by 'tidytree'
```

```
## Found more than one class "phylo" in cache; using the first, from namespace 'phyloseq'
```

```
## Also defined by 'tidytree'
```

```
## Found more than one class "phylo" in cache; using the first, from namespace 'phyloseq'
```

```
## Also defined by 'tidytree'
```

```
## Found more than one class "phylo" in cache; using the first, from namespace 'phyloseq'
```

```
## Also defined by 'tidytree'
```

```
## Found more than one class "phylo" in cache; using the first, from namespace 'phyloseq'
```

```
## Also defined by 'tidytree'
```

```
## Found more than one class "phylo" in cache; using the first, from namespace 'phyloseq'
```

```
## Also defined by 'tidytree'
```

```
## Found more than one class "phylo" in cache; using the first, from namespace 'phyloseq'
```

```
## Also defined by 'tidytree'
```

```
## Found more than one class "phylo" in cache; using the first, from namespace 'phyloseq'
```

```
## Also defined by 'tidytree'
```

```
## Found more than one class "phylo" in cache; using the first, from namespace 'phyloseq'
```

```
## Also defined by 'tidytree'
```

```
## Found more than one class "phylo" in cache; using the first, from namespace 'phyloseq'
```

```
## Also defined by 'tidytree'
```

```
## Found more than one class "phylo" in cache; using the first, from namespace 'phyloseq'
```

```
## Also defined by 'tidytree'
```

```
## Found more than one class "phylo" in cache; using the first, from namespace 'phyloseq'
```

```
## Also defined by 'tidytree'
```

```
## Found more than one class "phylo" in cache; using the first, from namespace 'phyloseq'
```

```
## Also defined by 'tidytree'
```

```
## Found more than one class "phylo" in cache; using the first, from namespace 'phyloseq'
```

```
## Also defined by 'tidytree'
```

```
## Found more than one class "phylo" in cache; using the first, from namespace 'phyloseq'
```

```
## Also defined by 'tidytree'
```

```
## Found more than one class "phylo" in cache; using the first, from namespace 'phyloseq'
```

```
## Also defined by 'tidytree'
```

```
## Found more than one class "phylo" in cache; using the first, from namespace 'phyloseq'
```

```
## Also defined by 'tidytree'
```

```
## Found more than one class "phylo" in cache; using the first, from namespace 'phyloseq'
```

```
## Also defined by 'tidytree'
```

```
## Found more than one class "phylo" in cache; using the first, from namespace 'phyloseq'
```

```
## Also defined by 'tidytree'
```

```
## Found more than one class "phylo" in cache; using the first, from namespace 'phyloseq'
```

```
## Also defined by 'tidytree'
```

```
## Found more than one class "phylo" in cache; using the first, from namespace 'phyloseq'
```

```
## Also defined by 'tidytree'
```

```
## Found more than one class "phylo" in cache; using the first, from namespace 'phyloseq'
```

```
## Also defined by 'tidytree'
```

```
## Found more than one class "phylo" in cache; using the first, from namespace 'phyloseq'
```

```
## Also defined by 'tidytree'
```

```
## Found more than one class "phylo" in cache; using the first, from namespace 'phyloseq'
```

```
## Also defined by 'tidytree'
```

```
## Found more than one class "phylo" in cache; using the first, from namespace 'phyloseq'
```

```
## Also defined by 'tidytree'
```

```
## Found more than one class "phylo" in cache; using the first, from namespace 'phyloseq'
```

```
## Also defined by 'tidytree'
```

```
## Found more than one class "phylo" in cache; using the first, from namespace 'phyloseq'
```

```
## Also defined by 'tidytree'
```

```
## Found more than one class "phylo" in cache; using the first, from namespace 'phyloseq'
```

```
## Also defined by 'tidytree'
```

```
## Found more than one class "phylo" in cache; using the first, from namespace 'phyloseq'
```

```
## Also defined by 'tidytree'
```

```
## Found more than one class "phylo" in cache; using the first, from namespace 'phyloseq'
```

```
## Also defined by 'tidytree'
```

```
## Found more than one class "phylo" in cache; using the first, from namespace 'phyloseq'
```

```
## Also defined by 'tidytree'
```

```
## Found more than one class "phylo" in cache; using the first, from namespace 'phyloseq'
```

```
## Also defined by 'tidytree'
```

```
## Found more than one class "phylo" in cache; using the first, from namespace 'phyloseq'
```

```
## Also defined by 'tidytree'
```

```
## Found more than one class "phylo" in cache; using the first, from namespace 'phyloseq'
```

```
## Also defined by 'tidytree'
```

```
## Found more than one class "phylo" in cache; using the first, from namespace 'phyloseq'
```

```
## Also defined by 'tidytree'
```

```
## Found more than one class "phylo" in cache; using the first, from namespace 'phyloseq'
```

```
## Also defined by 'tidytree'
```

```
## Found more than one class "phylo" in cache; using the first, from namespace 'phyloseq'
```

```
## Also defined by 'tidytree'
```

```
## Found more than one class "phylo" in cache; using the first, from namespace 'phyloseq'
```

```
## Also defined by 'tidytree'
```

```
## Found more than one class "phylo" in cache; using the first, from namespace 'phyloseq'
```

```
## Also defined by 'tidytree'
```

```
## Found more than one class "phylo" in cache; using the first, from namespace 'phyloseq'
```

```
## Also defined by 'tidytree'
```

```
## Found more than one class "phylo" in cache; using the first, from namespace 'phyloseq'
```

```
## Also defined by 'tidytree'
```

```
## Found more than one class "phylo" in cache; using the first, from namespace 'phyloseq'
```

```
## Also defined by 'tidytree'
```

```
## Found more than one class "phylo" in cache; using the first, from namespace 'phyloseq'
```

```
## Also defined by 'tidytree'
```

```
## Found more than one class "phylo" in cache; using the first, from namespace 'phyloseq'
```

```
## Also defined by 'tidytree'
```

```
## Found more than one class "phylo" in cache; using the first, from namespace 'phyloseq'
```

```
## Also defined by 'tidytree'
```

```
## Found more than one class "phylo" in cache; using the first, from namespace 'phyloseq'
```

```
## Also defined by 'tidytree'
```

```
## Found more than one class "phylo" in cache; using the first, from namespace 'phyloseq'
```

```
## Also defined by 'tidytree'
```

```
## Found more than one class "phylo" in cache; using the first, from namespace 'phyloseq'
```

```
## Also defined by 'tidytree'
```

```
## Found more than one class "phylo" in cache; using the first, from namespace 'phyloseq'
```

```
## Also defined by 'tidytree'
```

```
## Found more than one class "phylo" in cache; using the first, from namespace 'phyloseq'
```

```
## Also defined by 'tidytree'
```

```
## Found more than one class "phylo" in cache; using the first, from namespace 'phyloseq'
```

```
## Also defined by 'tidytree'
```

```
## Found more than one class "phylo" in cache; using the first, from namespace 'phyloseq'
```

```
## Also defined by 'tidytree'
```

```
## Found more than one class "phylo" in cache; using the first, from namespace 'phyloseq'
```

```
## Also defined by 'tidytree'
```

```
## Found more than one class "phylo" in cache; using the first, from namespace 'phyloseq'
```

```
## Also defined by 'tidytree'
```

```
## Found more than one class "phylo" in cache; using the first, from namespace 'phyloseq'
```

```
## Also defined by 'tidytree'
```

```
## Found more than one class "phylo" in cache; using the first, from namespace 'phyloseq'
```

```
## Also defined by 'tidytree'
```

```
## Found more than one class "phylo" in cache; using the first, from namespace 'phyloseq'
```

```
## Also defined by 'tidytree'
```

```
## Found more than one class "phylo" in cache; using the first, from namespace 'phyloseq'
```

```
## Also defined by 'tidytree'
```

```
## Found more than one class "phylo" in cache; using the first, from namespace 'phyloseq'
```

```
## Also defined by 'tidytree'
```

```
## Found more than one class "phylo" in cache; using the first, from namespace 'phyloseq'
```

```
## Also defined by 'tidytree'
```

```
## Found more than one class "phylo" in cache; using the first, from namespace 'phyloseq'
```

```
## Also defined by 'tidytree'
```

```
## Found more than one class "phylo" in cache; using the first, from namespace 'phyloseq'
```

```
## Also defined by 'tidytree'
```

```
## Found more than one class "phylo" in cache; using the first, from namespace 'phyloseq'
```

```
## Also defined by 'tidytree'
```

```
## Found more than one class "phylo" in cache; using the first, from namespace 'phyloseq'
```

```
## Also defined by 'tidytree'
```

```
## Found more than one class "phylo" in cache; using the first, from namespace 'phyloseq'
```

```
## Also defined by 'tidytree'
```

```
## Found more than one class "phylo" in cache; using the first, from namespace 'phyloseq'
```

```
## Also defined by 'tidytree'
```

```
## Found more than one class "phylo" in cache; using the first, from namespace 'phyloseq'
```

```
## Also defined by 'tidytree'
```

```
## Found more than one class "phylo" in cache; using the first, from namespace 'phyloseq'
```

```
## Also defined by 'tidytree'
```

```
## Found more than one class "phylo" in cache; using the first, from namespace 'phyloseq'
```

```
## Also defined by 'tidytree'
```

```
## Found more than one class "phylo" in cache; using the first, from namespace 'phyloseq'
```

```
## Also defined by 'tidytree'
```

```
## Found more than one class "phylo" in cache; using the first, from namespace 'phyloseq'
```

```
## Also defined by 'tidytree'
```

```
## Found more than one class "phylo" in cache; using the first, from namespace 'phyloseq'
```

```
## Also defined by 'tidytree'
```

```
## Found more than one class "phylo" in cache; using the first, from namespace 'phyloseq'
```

```
## Also defined by 'tidytree'
```

```
## Found more than one class "phylo" in cache; using the first, from namespace 'phyloseq'
```

```
## Also defined by 'tidytree'
```

```
## Found more than one class "phylo" in cache; using the first, from namespace 'phyloseq'
```

```
## Also defined by 'tidytree'
```

```
## Found more than one class "phylo" in cache; using the first, from namespace 'phyloseq'
```

```
## Also defined by 'tidytree'
```

```
## Found more than one class "phylo" in cache; using the first, from namespace 'phyloseq'
```

```
## Also defined by 'tidytree'
```

```
## Found more than one class "phylo" in cache; using the first, from namespace 'phyloseq'
```

```
## Also defined by 'tidytree'
```

```
## Found more than one class "phylo" in cache; using the first, from namespace 'phyloseq'
```

```
## Also defined by 'tidytree'
```

```
## Found more than one class "phylo" in cache; using the first, from namespace 'phyloseq'
```

```
## Also defined by 'tidytree'
```

```
## Found more than one class "phylo" in cache; using the first, from namespace 'phyloseq'
```

```
## Also defined by 'tidytree'
```

```
## Found more than one class "phylo" in cache; using the first, from namespace 'phyloseq'
```

```
## Also defined by 'tidytree'
```

```
## Found more than one class "phylo" in cache; using the first, from namespace 'phyloseq'
```

```
## Also defined by 'tidytree'
```

```
## Found more than one class "phylo" in cache; using the first, from namespace 'phyloseq'
```

```
## Also defined by 'tidytree'
```

```
## Found more than one class "phylo" in cache; using the first, from namespace 'phyloseq'
```

```
## Also defined by 'tidytree'
```

```
## Found more than one class "phylo" in cache; using the first, from namespace 'phyloseq'
```

```
## Also defined by 'tidytree'
```

```
## Found more than one class "phylo" in cache; using the first, from namespace 'phyloseq'
```

```
## Also defined by 'tidytree'
```

```
## Found more than one class "phylo" in cache; using the first, from namespace 'phyloseq'
```

```
## Also defined by 'tidytree'
```

```
## Found more than one class "phylo" in cache; using the first, from namespace 'phyloseq'
```

```
## Also defined by 'tidytree'
```

```
## Found more than one class "phylo" in cache; using the first, from namespace 'phyloseq'
```

```
## Also defined by 'tidytree'
```

```
## Found more than one class "phylo" in cache; using the first, from namespace 'phyloseq'
```

```
## Also defined by 'tidytree'
```

```
## Found more than one class "phylo" in cache; using the first, from namespace 'phyloseq'
```

```
## Also defined by 'tidytree'
```

```
## Found more than one class "phylo" in cache; using the first, from namespace 'phyloseq'
```

```
## Also defined by 'tidytree'
```

```
## Found more than one class "phylo" in cache; using the first, from namespace 'phyloseq'
```

```
## Also defined by 'tidytree'
```

```
## Found more than one class "phylo" in cache; using the first, from namespace 'phyloseq'
```

```
## Also defined by 'tidytree'
```

```
## Found more than one class "phylo" in cache; using the first, from namespace 'phyloseq'
```

```
## Also defined by 'tidytree'
```

```
## Found more than one class "phylo" in cache; using the first, from namespace 'phyloseq'
```

```
## Also defined by 'tidytree'
```

```
## Found more than one class "phylo" in cache; using the first, from namespace 'phyloseq'
```

```
## Also defined by 'tidytree'
```

```
## Found more than one class "phylo" in cache; using the first, from namespace 'phyloseq'
```

```
## Also defined by 'tidytree'
```

```
## Found more than one class "phylo" in cache; using the first, from namespace 'phyloseq'
```

```
## Also defined by 'tidytree'
```

```
## Found more than one class "phylo" in cache; using the first, from namespace 'phyloseq'
```

```
## Also defined by 'tidytree'
```

```
## Found more than one class "phylo" in cache; using the first, from namespace 'phyloseq'
```

```
## Also defined by 'tidytree'
```

```
## Found more than one class "phylo" in cache; using the first, from namespace 'phyloseq'
```

```
## Also defined by 'tidytree'
```

```
## Found more than one class "phylo" in cache; using the first, from namespace 'phyloseq'
```

```
## Also defined by 'tidytree'
```

```
## Found more than one class "phylo" in cache; using the first, from namespace 'phyloseq'
```

```
## Also defined by 'tidytree'
```

```
## Found more than one class "phylo" in cache; using the first, from namespace 'phyloseq'
```

```
## Also defined by 'tidytree'
```

```
## Found more than one class "phylo" in cache; using the first, from namespace 'phyloseq'
```

```
## Also defined by 'tidytree'
```

```
## Found more than one class "phylo" in cache; using the first, from namespace 'phyloseq'
```

```
## Also defined by 'tidytree'
```

```
## Found more than one class "phylo" in cache; using the first, from namespace 'phyloseq'
```

```
## Also defined by 'tidytree'
```

```
## Found more than one class "phylo" in cache; using the first, from namespace 'phyloseq'
```

```
## Also defined by 'tidytree'
```

```
## Found more than one class "phylo" in cache; using the first, from namespace 'phyloseq'
```

```
## Also defined by 'tidytree'
```

```
## Found more than one class "phylo" in cache; using the first, from namespace 'phyloseq'
```

```
## Also defined by 'tidytree'
```

```
## Found more than one class "phylo" in cache; using the first, from namespace 'phyloseq'
```

```
## Also defined by 'tidytree'
```

```
## Found more than one class "phylo" in cache; using the first, from namespace 'phyloseq'
```

```
## Also defined by 'tidytree'
```

```
## Found more than one class "phylo" in cache; using the first, from namespace 'phyloseq'
```

```
## Also defined by 'tidytree'
```

```
## Found more than one class "phylo" in cache; using the first, from namespace 'phyloseq'
```

```
## Also defined by 'tidytree'
```

```
## Found more than one class "phylo" in cache; using the first, from namespace 'phyloseq'
```

```
## Also defined by 'tidytree'
```

```
## Found more than one class "phylo" in cache; using the first, from namespace 'phyloseq'
```

```
## Also defined by 'tidytree'
```

```
## Found more than one class "phylo" in cache; using the first, from namespace 'phyloseq'
```

```
## Also defined by 'tidytree'
```

```
## Found more than one class "phylo" in cache; using the first, from namespace 'phyloseq'
```

```
## Also defined by 'tidytree'
```

```
## Found more than one class "phylo" in cache; using the first, from namespace 'phyloseq'
```

```
## Also defined by 'tidytree'
```

```
## Found more than one class "phylo" in cache; using the first, from namespace 'phyloseq'
```

```
## Also defined by 'tidytree'
```

```
## Found more than one class "phylo" in cache; using the first, from namespace 'phyloseq'
```

```
## Also defined by 'tidytree'
```

```
## Found more than one class "phylo" in cache; using the first, from namespace 'phyloseq'
```

```
## Also defined by 'tidytree'
```

```
## Found more than one class "phylo" in cache; using the first, from namespace 'phyloseq'
```

```
## Also defined by 'tidytree'
```

```
## Found more than one class "phylo" in cache; using the first, from namespace 'phyloseq'
```

```
## Also defined by 'tidytree'
```

```
## Found more than one class "phylo" in cache; using the first, from namespace 'phyloseq'
```

```
## Also defined by 'tidytree'
```

```
## Found more than one class "phylo" in cache; using the first, from namespace 'phyloseq'
```

```
## Also defined by 'tidytree'
```

```
## Found more than one class "phylo" in cache; using the first, from namespace 'phyloseq'
```

```
## Also defined by 'tidytree'
```

```
## Found more than one class "phylo" in cache; using the first, from namespace 'phyloseq'
```

```
## Also defined by 'tidytree'
```

```
## Found more than one class "phylo" in cache; using the first, from namespace 'phyloseq'
```

```
## Also defined by 'tidytree'
```

```
## Found more than one class "phylo" in cache; using the first, from namespace 'phyloseq'
```

```
## Also defined by 'tidytree'
```

```
## Found more than one class "phylo" in cache; using the first, from namespace 'phyloseq'
```

```
## Also defined by 'tidytree'
```

```
## Found more than one class "phylo" in cache; using the first, from namespace 'phyloseq'
```

```
## Also defined by 'tidytree'
```

```
#US 
dataset<-ps_gg_fp_f_prevalence_filter_2019_05_26_species
metadata<-as.data.frame(sample_data(ps_gg_fp_f_prevalence_filter_2019_05_26_species))
metadata<-metadata[metadata$hiv_phenotype %in% c("1_hiv_negative", "2_suppressed"), , drop=F]
metadata<-as.data.frame(as.matrix(metadata[metadata$sexual_orientation != "MSM" | is.na(metadata$sexual_orientation), , drop=F]))
metadata_boston<-metadata[metadata$sample_cohort == "boston", , drop=F]
sample_data(dataset)<-metadata_boston
```

```
## Found more than one class "phylo" in cache; using the first, from namespace 'phyloseq'
## Also defined by 'tidytree'
```

```
## Found more than one class "phylo" in cache; using the first, from namespace 'phyloseq'
```

```
## Also defined by 'tidytree'
```

```
## Found more than one class "phylo" in cache; using the first, from namespace 'phyloseq'
```

```
## Also defined by 'tidytree'
```

```
## Found more than one class "phylo" in cache; using the first, from namespace 'phyloseq'
```

```
## Also defined by 'tidytree'
```

```
ps.taxa.sub <- phyloseq::prune_taxa(taxa_sums(dataset) > 0, dataset)
```

```
## Found more than one class "phylo" in cache; using the first, from namespace 'phyloseq'
## Also defined by 'tidytree'
```

```
out <- ANCOMBC::ancombc(data = ps.taxa.sub, formula = "hiv_phenotype", tax_level = NULL,
              p_adj_method = "BH", prv_cut = 0.05, lib_cut = 1000, 
              group = "hiv_phenotype", struc_zero = TRUE, neg_lb = FALSE, tol = 1e-5, 
              max_iter = 100, conserve = FALSE, alpha = 0.05, global = FALSE, n_cl = 6)
```

```
## 'ancombc' is deprecated 
## Use 'ancombc2' instead
```

```
## Found more than one class "phylo" in cache; using the first, from namespace 'phyloseq'
```

```
## Also defined by 'tidytree'
```

```
## Found more than one class "phylo" in cache; using the first, from namespace 'phyloseq'
```

```
## Also defined by 'tidytree'
```

```
## `tax_level` is not speficified 
## No agglomeration will be performed
## Otherwise, please speficy `tax_level` by one of the following: 
## Kingdom, Phylum, Class, Order, Family, Genus, Species
```

```
## Found more than one class "phylo" in cache; using the first, from namespace 'phyloseq'
```

```
## Also defined by 'tidytree'
```

```
## Found more than one class "phylo" in cache; using the first, from namespace 'phyloseq'
```

```
## Also defined by 'tidytree'
```

```
## Found more than one class "phylo" in cache; using the first, from namespace 'phyloseq'
```

```
## Also defined by 'tidytree'
```

```
## Found more than one class "phylo" in cache; using the first, from namespace 'phyloseq'
```

```
## Also defined by 'tidytree'
```

```
## Warning: The group variable has < 3 categories 
## The multi-group comparisons (global/pairwise/dunnet/trend) will be deactivated
```

```
## Found more than one class "phylo" in cache; using the first, from namespace 'phyloseq'
## Also defined by 'tidytree'
```

```
## Found more than one class "phylo" in cache; using the first, from namespace 'phyloseq'
```

```
## Also defined by 'tidytree'
```

```
res <- out$res
res_rn <- purrr::imap(res, function(x, y) dplyr::rename(x, !!y := hiv_phenotype2_suppressed))
res_df <- purrr::reduce(res_rn, dplyr::left_join, by = "taxon")
res_df <- dplyr::select(res_df, !starts_with("(Int"))
res_df_taxa <- dplyr::left_join(res_df, tibble::rownames_to_column(as.data.frame(phyloseq::tax_table(ps.taxa.sub))), by = c("taxon" = "rowname"))
res_df_taxa[["index_num"]] <- 1:nrow(res_df_taxa)
res_df_taxa[["cohort"]] <- "boston"
res_df_taxa[["method"]] <- "ancom"
res_df_taxa <- tidyr::unite(res_df_taxa, col =  "Genus_Species", Genus, Species, index_num, remove = FALSE)
alpha = 0.05
taxa_sig <- dplyr::filter(res_df_taxa, q_val < 0.05)
taxa_sig$Genus_Species <- forcats::fct_reorder(taxa_sig$Genus_Species, taxa_sig$lfc, min)
taxa_sig$taxon_short <- stringr::str_sub(taxa_sig$taxon, 1, 4)
ps.taxa.rel.sig <- phyloseq::prune_taxa(taxa_sig[["taxon"]], ps.taxa.sub)
```

```
## Found more than one class "phylo" in cache; using the first, from namespace 'phyloseq'
## Also defined by 'tidytree'
```

```
# Only keep filtered samples 
ps.taxa.rel.sig <- phyloseq::prune_samples(rownames(phyloseq::otu_table(ps.taxa.sub)), ps.taxa.rel.sig)
sigtab_dataset_us <- taxa_sig
write.csv(sigtab_dataset_us, "ANCOM_US_Species_NEG_ART_Filtered.csv")


#BOTSWANA
dataset<-ps_gg_fp_f_prevalence_filter_2019_05_26_species
metadata<-as.data.frame(sample_data(ps_gg_fp_f_prevalence_filter_2019_05_26_species))
metadata<-metadata[metadata$hiv_phenotype %in% c("1_hiv_negative", "2_suppressed"), , drop=F]
metadata<-as.data.frame(as.matrix(metadata[metadata$sexual_orientation != "MSM" | is.na(metadata$sexual_orientation), , drop=F]))
metadata_botswana<-metadata[metadata$sample_cohort == "botswana", , drop=F]
sample_data(dataset)<-metadata_botswana
```

```
## Found more than one class "phylo" in cache; using the first, from namespace 'phyloseq'
## Also defined by 'tidytree'
```

```
## Found more than one class "phylo" in cache; using the first, from namespace 'phyloseq'
```

```
## Also defined by 'tidytree'
```

```
## Found more than one class "phylo" in cache; using the first, from namespace 'phyloseq'
```

```
## Also defined by 'tidytree'
```

```
## Found more than one class "phylo" in cache; using the first, from namespace 'phyloseq'
```

```
## Also defined by 'tidytree'
```

```
ps.taxa.sub <- phyloseq::prune_taxa(taxa_sums(dataset) > 0, dataset)
```

```
## Found more than one class "phylo" in cache; using the first, from namespace 'phyloseq'
## Also defined by 'tidytree'
```

```
out <- ANCOMBC::ancombc(data = ps.taxa.sub, formula = "hiv_phenotype", tax_level = NULL,
              p_adj_method = "BH", prv_cut = 0.05, lib_cut = 1000, 
              group = "hiv_phenotype", struc_zero = TRUE, neg_lb = FALSE, tol = 1e-5, 
              max_iter = 100, conserve = FALSE, alpha = 0.05, global = FALSE, n_cl = 6)
```

```
## 'ancombc' is deprecated 
## Use 'ancombc2' instead
```

```
## Found more than one class "phylo" in cache; using the first, from namespace 'phyloseq'
```

```
## Also defined by 'tidytree'
```

```
## Found more than one class "phylo" in cache; using the first, from namespace 'phyloseq'
```

```
## Also defined by 'tidytree'
```

```
## `tax_level` is not speficified 
## No agglomeration will be performed
## Otherwise, please speficy `tax_level` by one of the following: 
## Kingdom, Phylum, Class, Order, Family, Genus, Species
```

```
## Found more than one class "phylo" in cache; using the first, from namespace 'phyloseq'
```

```
## Also defined by 'tidytree'
```

```
## Found more than one class "phylo" in cache; using the first, from namespace 'phyloseq'
```

```
## Also defined by 'tidytree'
```

```
## Found more than one class "phylo" in cache; using the first, from namespace 'phyloseq'
```

```
## Also defined by 'tidytree'
```

```
## Found more than one class "phylo" in cache; using the first, from namespace 'phyloseq'
```

```
## Also defined by 'tidytree'
```

```
## Warning: The group variable has < 3 categories 
## The multi-group comparisons (global/pairwise/dunnet/trend) will be deactivated
```

```
## Found more than one class "phylo" in cache; using the first, from namespace 'phyloseq'
## Also defined by 'tidytree'
```

```
## Found more than one class "phylo" in cache; using the first, from namespace 'phyloseq'
```

```
## Also defined by 'tidytree'
```

```
res <- out$res
res_rn <- purrr::imap(res, function(x, y) dplyr::rename(x, !!y := hiv_phenotype2_suppressed))
res_df <- purrr::reduce(res_rn, dplyr::left_join, by = "taxon")
res_df <- dplyr::select(res_df, !starts_with("(Int"))
res_df_taxa <- dplyr::left_join(res_df, tibble::rownames_to_column(as.data.frame(phyloseq::tax_table(ps.taxa.sub))), by = c("taxon" = "rowname"))
res_df_taxa[["index_num"]] <- 1:nrow(res_df_taxa)
res_df_taxa[["cohort"]] <- "botswana"
res_df_taxa[["method"]] <- "ancom"
res_df_taxa <- tidyr::unite(res_df_taxa, col =  "Genus_Species", Genus, Species, index_num, remove = FALSE)
alpha = 0.05
taxa_sig <- dplyr::filter(res_df_taxa, q_val < 0.05)
taxa_sig$Genus_Species <- forcats::fct_reorder(taxa_sig$Genus_Species, taxa_sig$lfc, min)
taxa_sig$taxon_short <- stringr::str_sub(taxa_sig$taxon, 1, 4)
ps.taxa.rel.sig <- phyloseq::prune_taxa(taxa_sig[["taxon"]], ps.taxa.sub)
```

```
## Found more than one class "phylo" in cache; using the first, from namespace 'phyloseq'
## Also defined by 'tidytree'
```

```
# Only keep filtered samples 
ps.taxa.rel.sig <- phyloseq::prune_samples(rownames(phyloseq::otu_table(ps.taxa.sub)), ps.taxa.rel.sig)
sigtab_dataset_botswana <- taxa_sig
write.csv(sigtab_dataset_botswana, "ANCOM_BOTS_Species_NEG_ART_Filtered.csv")


#UGANDA
dataset<-ps_gg_fp_f_prevalence_filter_2019_05_26_species
metadata<-as.data.frame(sample_data(ps_gg_fp_f_prevalence_filter_2019_05_26_species))
metadata<-metadata[metadata$hiv_phenotype %in% c("1_hiv_negative", "2_suppressed"), , drop=F]
metadata<-as.data.frame(as.matrix(metadata[metadata$sexual_orientation != "MSM" | is.na(metadata$sexual_orientation), , drop=F]))
metadata_uganda<-metadata[metadata$sample_cohort == "uganda_2", , drop=F]
sample_data(dataset)<-metadata_uganda
```

```
## Found more than one class "phylo" in cache; using the first, from namespace 'phyloseq'
## Also defined by 'tidytree'
```

```
## Found more than one class "phylo" in cache; using the first, from namespace 'phyloseq'
```

```
## Also defined by 'tidytree'
```

```
## Found more than one class "phylo" in cache; using the first, from namespace 'phyloseq'
```

```
## Also defined by 'tidytree'
```

```
## Found more than one class "phylo" in cache; using the first, from namespace 'phyloseq'
```

```
## Also defined by 'tidytree'
```

```
ps.taxa.sub <- phyloseq::prune_taxa(taxa_sums(dataset) > 0, dataset)
```

```
## Found more than one class "phylo" in cache; using the first, from namespace 'phyloseq'
## Also defined by 'tidytree'
```

```
out <- ANCOMBC::ancombc(data = ps.taxa.sub, formula = "hiv_phenotype", tax_level = NULL,
              p_adj_method = "BH", prv_cut = 0.05, lib_cut = 1000, 
              group = "hiv_phenotype", struc_zero = TRUE, neg_lb = FALSE, tol = 1e-5, 
              max_iter = 100, conserve = FALSE, alpha = 0.05, global = FALSE, n_cl = 6)
```

```
## 'ancombc' is deprecated 
## Use 'ancombc2' instead
```

```
## Found more than one class "phylo" in cache; using the first, from namespace 'phyloseq'
```

```
## Also defined by 'tidytree'
```

```
## Found more than one class "phylo" in cache; using the first, from namespace 'phyloseq'
```

```
## Also defined by 'tidytree'
```

```
## `tax_level` is not speficified 
## No agglomeration will be performed
## Otherwise, please speficy `tax_level` by one of the following: 
## Kingdom, Phylum, Class, Order, Family, Genus, Species
```

```
## Found more than one class "phylo" in cache; using the first, from namespace 'phyloseq'
```

```
## Also defined by 'tidytree'
```

```
## Found more than one class "phylo" in cache; using the first, from namespace 'phyloseq'
```

```
## Also defined by 'tidytree'
```

```
## Found more than one class "phylo" in cache; using the first, from namespace 'phyloseq'
```

```
## Also defined by 'tidytree'
```

```
## Found more than one class "phylo" in cache; using the first, from namespace 'phyloseq'
```

```
## Also defined by 'tidytree'
```

```
## Warning: The group variable has < 3 categories 
## The multi-group comparisons (global/pairwise/dunnet/trend) will be deactivated
```

```
## Found more than one class "phylo" in cache; using the first, from namespace 'phyloseq'
## Also defined by 'tidytree'
```

```
## Found more than one class "phylo" in cache; using the first, from namespace 'phyloseq'
```

```
## Also defined by 'tidytree'
```

```
res <- out$res
res_rn <- purrr::imap(res, function(x, y) dplyr::rename(x, !!y := hiv_phenotype2_suppressed))
res_df <- purrr::reduce(res_rn, dplyr::left_join, by = "taxon")
res_df <- dplyr::select(res_df, !starts_with("(Int"))
res_df_taxa <- dplyr::left_join(res_df, tibble::rownames_to_column(as.data.frame(phyloseq::tax_table(ps.taxa.sub))), by = c("taxon" = "rowname"))
res_df_taxa[["index_num"]] <- 1:nrow(res_df_taxa)
res_df_taxa[["cohort"]] <- "uganda_2"
res_df_taxa[["method"]] <- "ancom"
res_df_taxa <- tidyr::unite(res_df_taxa, col =  "Genus_Species", Genus, Species, index_num, remove = FALSE)
alpha = 0.05
taxa_sig <- dplyr::filter(res_df_taxa, q_val < 0.05)
taxa_sig$Genus_Species <- forcats::fct_reorder(taxa_sig$Genus_Species, taxa_sig$lfc, min)
taxa_sig$taxon_short <- stringr::str_sub(taxa_sig$taxon, 1, 4)
ps.taxa.rel.sig <- phyloseq::prune_taxa(taxa_sig[["taxon"]], ps.taxa.sub)
```

```
## Found more than one class "phylo" in cache; using the first, from namespace 'phyloseq'
## Also defined by 'tidytree'
```

```
# Only keep filtered samples 
ps.taxa.rel.sig <- phyloseq::prune_samples(rownames(phyloseq::otu_table(ps.taxa.sub)), ps.taxa.rel.sig)
sigtab_dataset_uganda <- taxa_sig
write.csv(sigtab_dataset_uganda, "ANCOM_UGANDA_Species_NEG_ART_Filtered.csv")

# Merge results from all three sample cohorts:
sigtab_dataset<-rbind(sigtab_dataset_us, sigtab_dataset_botswana, sigtab_dataset_uganda)

# Remove extra brackets around taxonomic name for labels
sigtab_dataset$Genus_label <- stringr::str_replace(sigtab_dataset$Genus, "^\\[([^\\]]+)\\]", "\\1")
sigtab_dataset$Species_label <- stringr::str_replace(sigtab_dataset$Species, "^\\[([^\\]]+)\\]", "\\1")
sigtab_dataset %>% dplyr::mutate(Genus_species_label =
                dplyr::case_when(is.na(stringr::str_extract(Genus_label, "\\[")) & is.na(stringr::str_extract(Species_label, "\\[")) ~ paste(Genus_label,Species_label),
                                 TRUE ~ paste(Genus_label,"sp"))) -> sigtab_dataset
sigtab_dataset$Genus_species_label <- stringr::str_replace(sigtab_dataset$Genus_species_label, "\\[([^\\]]+)\\]", "")
sigtab_dataset$Genus_species_label <- stringr::str_replace(sigtab_dataset$Genus_species_label, "\\ +", " ")

# Melt data and create figure
all <- sigtab_dataset[, colnames(sigtab_dataset) %in% c("lfc", "Genus_species_label", "cohort"), drop=F]
all <- dplyr::arrange(all, lfc)
all$Genus_species_label <- factor(all$Genus_species_label, levels = unique(all$Genus_species_label))
all_melt <- melt(all)
```

```
## Using cohort, Genus_species_label as id variables
```

```
all_melt$cohort <- factor(all_melt$cohort, levels = c("boston", "botswana", "uganda_2"))

ggsave("SupplementaryFigure6B_ANCOM_v4.pdf", ggplot(data = all_melt, aes(x = Genus_species_label, y = cohort)) + geom_tile(aes(fill = value)) +
         coord_flip() + theme_bw() + scale_y_discrete(position = "right") +
         scale_fill_gradient2(low = "brown", mid = "white", high = "darkblue") +
         theme(axis.text.y = element_text(size = 10), axis.ticks = element_blank(), axis.title = element_blank(),
               panel.grid = element_line(linewidth = 0.1), panel.background = element_rect(fill = "white")) + 
         geom_hline(yintercept = c(1.5, 2.5), size = 0.2, color = "grey90") + ggtitle("SPECIES"), width = 10, height = 15, units = "in", dpi = 300)
#--------------------------------------------------------------------------------------------------------------
```
