# Supplementary material for: HIV-associated gut microbial alterations are dependent on host and geographic context
Source: Nat Commun. 2024 Feb 5;15:1055. doi: 10.1038/s41467-023-44566-4 (PMC10844288; doi:10.1038/s41467-023-44566-4)
Supplement: Supplementary file 17 — SupplementaryFigure7_Rocafort-Gootenberg_2023_04_19 [file 41467_2023_44566_MOESM17_ESM.html]

SupplementalFigure7ANCOM\_Rocafort-Gootenberg\_2023\_04\_14


# SupplementalFigure7ANCOM\_Rocafort-Gootenberg\_2023\_04\_14

#Load needed R packages

```
library("phyloseq")
library("ggplot2")
library("DESeq2")
```

```
## Loading required package: S4Vectors
```

```
## Loading required package: stats4
```

```
## Loading required package: BiocGenerics
```

```
## 
## Attaching package: 'BiocGenerics'
```

```
## The following objects are masked from 'package:stats':
## 
##     IQR, mad, sd, var, xtabs
```

```
## The following objects are masked from 'package:base':
## 
##     anyDuplicated, aperm, append, as.data.frame, basename, cbind,
##     colnames, dirname, do.call, duplicated, eval, evalq, Filter, Find,
##     get, grep, grepl, intersect, is.unsorted, lapply, Map, mapply,
##     match, mget, order, paste, pmax, pmax.int, pmin, pmin.int,
##     Position, rank, rbind, Reduce, rownames, sapply, setdiff, sort,
##     table, tapply, union, unique, unsplit, which.max, which.min
```

```
## 
## Attaching package: 'S4Vectors'
```

```
## The following objects are masked from 'package:base':
## 
##     expand.grid, I, unname
```

```
## Loading required package: IRanges
```

```
## 
## Attaching package: 'IRanges'
```

```
## The following object is masked from 'package:phyloseq':
## 
##     distance
```

```
## Loading required package: GenomicRanges
```

```
## Loading required package: GenomeInfoDb
```

```
## Loading required package: SummarizedExperiment
```

```
## Loading required package: MatrixGenerics
```

```
## Loading required package: matrixStats
```

```
## 
## Attaching package: 'MatrixGenerics'
```

```
## The following objects are masked from 'package:matrixStats':
## 
##     colAlls, colAnyNAs, colAnys, colAvgsPerRowSet, colCollapse,
##     colCounts, colCummaxs, colCummins, colCumprods, colCumsums,
##     colDiffs, colIQRDiffs, colIQRs, colLogSumExps, colMadDiffs,
##     colMads, colMaxs, colMeans2, colMedians, colMins, colOrderStats,
##     colProds, colQuantiles, colRanges, colRanks, colSdDiffs, colSds,
##     colSums2, colTabulates, colVarDiffs, colVars, colWeightedMads,
##     colWeightedMeans, colWeightedMedians, colWeightedSds,
##     colWeightedVars, rowAlls, rowAnyNAs, rowAnys, rowAvgsPerColSet,
##     rowCollapse, rowCounts, rowCummaxs, rowCummins, rowCumprods,
##     rowCumsums, rowDiffs, rowIQRDiffs, rowIQRs, rowLogSumExps,
##     rowMadDiffs, rowMads, rowMaxs, rowMeans2, rowMedians, rowMins,
##     rowOrderStats, rowProds, rowQuantiles, rowRanges, rowRanks,
##     rowSdDiffs, rowSds, rowSums2, rowTabulates, rowVarDiffs, rowVars,
##     rowWeightedMads, rowWeightedMeans, rowWeightedMedians,
##     rowWeightedSds, rowWeightedVars
```

```
## Loading required package: Biobase
```

```
## Welcome to Bioconductor
## 
##     Vignettes contain introductory material; view with
##     'browseVignettes()'. To cite Bioconductor, see
##     'citation("Biobase")', and for packages 'citation("pkgname")'.
```

```
## 
## Attaching package: 'Biobase'
```

```
## The following object is masked from 'package:MatrixGenerics':
## 
##     rowMedians
```

```
## The following objects are masked from 'package:matrixStats':
## 
##     anyMissing, rowMedians
```

```
## The following object is masked from 'package:phyloseq':
## 
##     sampleNames
```

```
library("ANCOMBC")
library("reshape")
```

```
## 
## Attaching package: 'reshape'
```

```
## The following objects are masked from 'package:S4Vectors':
## 
##     expand, rename
```

```
library("readr")
library("gridExtra")
```

```
## 
## Attaching package: 'gridExtra'
```

```
## The following object is masked from 'package:Biobase':
## 
##     combine
```

```
## The following object is masked from 'package:BiocGenerics':
## 
##     combine
```

#Load picrust pathways

```
pathways <- read.delim("Supplemental_Figure_7/path_abun_unstrat_descrip.tsv")
row.names(pathways)<-pathways$pathway
pathways$pathway<-NULL
pathways$description<-NULL
pathways<-t(pathways)
row.names(pathways)<-gsub("X1", "1", row.names(pathways))
row.names(pathways)<-gsub("X2", "2", row.names(pathways))
row.names(pathways)<-gsub("X3", "3", row.names(pathways))
row.names(pathways)<-gsub("X4", "4", row.names(pathways))
row.names(pathways)<-gsub("X5", "5", row.names(pathways))
row.names(pathways)<-gsub("X6", "6", row.names(pathways))
row.names(pathways)<-gsub("X7", "7", row.names(pathways))
row.names(pathways)<-gsub("X8", "8", row.names(pathways))
row.names(pathways)<-gsub("X9", "9", row.names(pathways))
```

#Load original phyloseq object output from DADA2 pipeline and pull in
new metadata

```
ps_gg_fp_f_prevalence_filter_2019_05_26<-readRDS("ps_gg_fp_f_prevalence_filter_2019_05_26")
readr::read_csv(
  "Metadata_formatted_nat_comm_add_2021_10_24.csv",
  col_names = TRUE,
  col_types = NULL,
  col_select = NULL,
  id = NULL,
  locale = default_locale(),
  na = c("", "NA", "empty", "EMPTY"),
  quote = "\"",
  comment = "",
  trim_ws = TRUE,
  skip = 0,
  name_repair = "unique",
  num_threads = readr_threads(),
  progress = show_progress(),
  show_col_types = should_show_types(),
  skip_empty_rows = TRUE,
  lazy = TRUE
) -> new_metadata
```

```
## Rows: 597 Columns: 88
## ── Column specification ────────────────────────────────────────────────────────
## Delimiter: ","
## chr (26): X, SampleID, subject_id, Race, Ethnicity, unique_id, sequencing_da...
## dbl (62): primer_used, read_count, age, height_cm, height_in, weight_kg, wei...
## 
## ℹ Use `spec()` to retrieve the full column specification for this data.
## ℹ Specify the column types or set `show_col_types = FALSE` to quiet this message.
```

```
### add {SampleID} as rownames
new_metadata_as_sample_data <- phyloseq::sample_data(new_metadata)
phyloseq::sample_names(new_metadata_as_sample_data) <- dplyr::pull(new_metadata, 1)
phyloseq::sample_data(ps_gg_fp_f_prevalence_filter_2019_05_26) <- new_metadata_as_sample_data
```

```
## Found more than one class "phylo" in cache; using the first, from namespace 'phyloseq'
## Also defined by 'tidytree'
## Found more than one class "phylo" in cache; using the first, from namespace 'phyloseq'
## Also defined by 'tidytree'
## Found more than one class "phylo" in cache; using the first, from namespace 'phyloseq'
## Also defined by 'tidytree'
## Found more than one class "phylo" in cache; using the first, from namespace 'phyloseq'
## Also defined by 'tidytree'
## Found more than one class "phylo" in cache; using the first, from namespace 'phyloseq'
## Also defined by 'tidytree'
## Found more than one class "phylo" in cache; using the first, from namespace 'phyloseq'
## Also defined by 'tidytree'
## Found more than one class "phylo" in cache; using the first, from namespace 'phyloseq'
## Also defined by 'tidytree'
```

```
dim(new_metadata)
```

```
## [1] 597  88
```

```
all.equal(stringr::str_sort(new_metadata$SampleID), stringr::str_sort(row.names(pathways)))
```

```
## [1] TRUE
```

```
####
#Prepare phyloseq object
OTU = otu_table(t(pathways), taxa_are_rows = TRUE)
samples = phyloseq::sample_data(new_metadata)
phyloseq::sample_names(samples) <- new_metadata$SampleID
pathways_phyloseq <- phyloseq(OTU, samples)


#Fix randomness
set.seed(1)
```

#Categories (and fill blanks with previous ID)

```
#Categories (and fill blanks with previous ID)
All_pathways_of_MetaCyc_ontology_v1 <- read.delim("Supplemental_Figure_7/All_pathways_of_MetaCyc_ontology_v1_formatted.tsv")

table(row.names(otu_table(pathways_phyloseq)) %in% All_pathways_of_MetaCyc_ontology_v1$Object.ID)
```

```
## 
## FALSE  TRUE 
##     1   364
```

```
row.names(All_pathways_of_MetaCyc_ontology_v1) <- All_pathways_of_MetaCyc_ontology_v1$Object.ID
All_pathways_of_MetaCyc_ontology_v1$Object.ID <- NULL
All_pathways_of_MetaCyc_ontology_v1<-All_pathways_of_MetaCyc_ontology_v1[ , c("Level1", "Level2", "Level3", "Level4", "Level5", "Level6", "Pathways"), drop=F]

colnames(All_pathways_of_MetaCyc_ontology_v1) <- c("Category1", "Category2", "Category3", "Category4", "Category5", "Category6", "Category7")
All_pathways_of_MetaCyc_ontology_v1$Category3 <- gsub("Superpathways  ", "Superpathways ", All_pathways_of_MetaCyc_ontology_v1$Category3)

tax_table(pathways_phyloseq) <- as.matrix(All_pathways_of_MetaCyc_ontology_v1)

pathways_phyloseq
```

```
## phyloseq-class experiment-level object
## otu_table()   OTU Table:         [ 364 taxa and 597 samples ]
## sample_data() Sample Data:       [ 597 samples by 88 sample variables ]
## tax_table()   Taxonomy Table:    [ 364 taxa by 7 taxonomic ranks ]
```

```
pathways_phyloseq_group <- tax_glom(pathways_phyloseq, taxrank = "Category5")

pathways_phyloseq_group
```

```
## phyloseq-class experiment-level object
## otu_table()   OTU Table:         [ 92 taxa and 597 samples ]
## sample_data() Sample Data:       [ 597 samples by 88 sample variables ]
## tax_table()   Taxonomy Table:    [ 92 taxa by 7 taxonomic ranks ]
```

#ANCOM and plot

```
#ANCOM functions - NEG - ART
##################
##US Neg - ART
#Select samples of interest and update phyloseq object
metadata<-as.data.frame(sample_data(pathways_phyloseq))
metadata<-metadata[metadata$sample_cohort == "boston", , drop = F]
metadata<-metadata[metadata$hiv_phenotype %in% c("1_hiv_negative", "2_suppressed"), , drop = F]
metadata<-as.data.frame(as.matrix(metadata[metadata$sexual_orientation != "MSM", , drop = F]))

pathways_phyloseq_test <- pathways_phyloseq_group
sample_data(pathways_phyloseq_test) <- metadata

#Run ANCOM
pathways_phyloseq_test_prune <- phyloseq::prune_taxa(taxa_sums(pathways_phyloseq_test) > 0, pathways_phyloseq_test)
out <- ANCOMBC::ancombc2(data = pathways_phyloseq_test_prune, assay_name = "counts", tax_level = NULL, fix_formula = "hiv_phenotype", 
              p_adj_method = "BH", pseudo = 0, pseudo_sens = FALSE, prv_cut = 0.05, lib_cut = 1000, 
              group = "hiv_phenotype", struc_zero = TRUE, neg_lb = FALSE, alpha = 0.05, n_cl = 6, global = TRUE,
              em_control = list(tol = 1e-05, max_iter = 100), mdfdr_control = list(fwer_ctrl_method = "holm", B = 100))
```

```
## `tax_level` is not speficified 
## No agglomeration will be performed
## Otherwise, please speficy `tax_level` by one of the following:
```

```
## Warning: The group variable has < 3 categories 
## The multi-group comparisons (global/pairwise/dunnet/trend) will be deactivated
```

```
res_df <- out$res
res_df <- dplyr::rename(res_df, rowname = taxon)
colnames(res_df) <- stringr::str_replace(colnames(res_df), "hiv_phenotype.+", "hiv_phenotype")
res_df_taxa <- dplyr::left_join(res_df, tibble::rownames_to_column(as.data.frame(phyloseq::tax_table(pathways_phyloseq_test_prune))), by = "rowname")
res_df_taxa[["index_num"]] <- 1:nrow(res_df_taxa)
res_df_taxa[["cohort"]] <- "boston_treated"
res_df_taxa[["method"]] <- "ancom2"
# res_df_taxa <- tidyr::unite(res_df_taxa, col =  "Genus_Species", Genus, Species, index_num, remove = FALSE)
alpha = 0.05
taxa_sig <- dplyr::filter(res_df_taxa, q_hiv_phenotype < 0.05)
sigtab_dataset_us_treated <- taxa_sig
write.csv(sigtab_dataset_us_treated, "ANCOM_US_NEG_ART_Pathway_Filtered.csv")

##BOTSWANA NEG-ART
#Select samples of interest and update phyloseq object
metadata<-as.data.frame(sample_data(pathways_phyloseq))
metadata<-metadata[metadata$sample_cohort == "botswana", , drop = F]
metadata<-metadata[metadata$hiv_phenotype %in% c("1_hiv_negative", "2_suppressed"), , drop = F]
metadata<-as.data.frame(as.matrix(metadata[metadata$sexual_orientation != "MSM", , drop = F]))

pathways_phyloseq_test <- pathways_phyloseq_group
sample_data(pathways_phyloseq_test) <- metadata

#Run ANCOM
pathways_phyloseq_test_prune <- phyloseq::prune_taxa(taxa_sums(pathways_phyloseq_test) > 0, pathways_phyloseq_test)
out <- ANCOMBC::ancombc2(data = pathways_phyloseq_test_prune, assay_name = "counts", tax_level = NULL, fix_formula = "hiv_phenotype", 
              p_adj_method = "BH", pseudo = 0, pseudo_sens = FALSE, prv_cut = 0.05, lib_cut = 1000, 
              group = "hiv_phenotype", struc_zero = TRUE, neg_lb = FALSE, alpha = 0.05, n_cl = 6, global = TRUE,
              em_control = list(tol = 1e-05, max_iter = 100), mdfdr_control = list(fwer_ctrl_method = "holm", B = 100))
```

```
## `tax_level` is not speficified 
## No agglomeration will be performed
## Otherwise, please speficy `tax_level` by one of the following:
```

```
## Warning: The group variable has < 3 categories 
## The multi-group comparisons (global/pairwise/dunnet/trend) will be deactivated
```

```
res_df <- out$res
res_df <- dplyr::rename(res_df, rowname = taxon)
colnames(res_df) <- stringr::str_replace(colnames(res_df), "hiv_phenotype.+", "hiv_phenotype")
res_df_taxa <- dplyr::left_join(res_df, tibble::rownames_to_column(as.data.frame(phyloseq::tax_table(pathways_phyloseq_test_prune))), by = "rowname")
res_df_taxa[["index_num"]] <- 1:nrow(res_df_taxa)
res_df_taxa[["cohort"]] <- "botswana_treated"
res_df_taxa[["method"]] <- "ancom2"
# res_df_taxa <- tidyr::unite(res_df_taxa, col =  "Genus_Species", Genus, Species, index_num, remove = FALSE)
alpha = 0.05
taxa_sig <- dplyr::filter(res_df_taxa, q_hiv_phenotype < 0.05)
sigtab_dataset_botswana_treated <- taxa_sig
write.csv(sigtab_dataset_botswana_treated, "ANCOM_BOTS_NEG_ART_Pathway_Filtered.csv")

##UGANDA NEG-ART
#Select samples of interest and update phyloseq object
metadata<-as.data.frame(sample_data(pathways_phyloseq))
metadata<-metadata[metadata$sample_cohort == "uganda_2", , drop = F]
metadata<-metadata[metadata$hiv_phenotype %in% c("1_hiv_negative", "2_suppressed"), , drop = F]
metadata<-as.data.frame(as.matrix(metadata[metadata$sexual_orientation != "MSM", , drop = F]))

pathways_phyloseq_test <- pathways_phyloseq_group
sample_data(pathways_phyloseq_test) <- metadata

#Run ANCOM
pathways_phyloseq_test_prune <- phyloseq::prune_taxa(taxa_sums(pathways_phyloseq_test) > 0, pathways_phyloseq_test)
out <- ANCOMBC::ancombc2(data = pathways_phyloseq_test_prune, assay_name = "counts", tax_level = NULL, fix_formula = "hiv_phenotype", 
              p_adj_method = "BH", pseudo = 0, pseudo_sens = FALSE, prv_cut = 0.05, lib_cut = 1000, 
              group = "hiv_phenotype", struc_zero = TRUE, neg_lb = FALSE, alpha = 0.05, n_cl = 6, global = TRUE,
              em_control = list(tol = 1e-05, max_iter = 100), mdfdr_control = list(fwer_ctrl_method = "holm", B = 100))
```

```
## `tax_level` is not speficified 
## No agglomeration will be performed
## Otherwise, please speficy `tax_level` by one of the following:
```

```
## Warning: The group variable has < 3 categories 
## The multi-group comparisons (global/pairwise/dunnet/trend) will be deactivated
```

```
res_df <- out$res
res_df <- dplyr::rename(res_df, rowname = taxon)
colnames(res_df) <- stringr::str_replace(colnames(res_df), "hiv_phenotype.+", "hiv_phenotype")
res_df_taxa <- dplyr::left_join(res_df, tibble::rownames_to_column(as.data.frame(phyloseq::tax_table(pathways_phyloseq_test_prune))), by = "rowname")
res_df_taxa[["index_num"]] <- 1:nrow(res_df_taxa)
res_df_taxa[["cohort"]] <- "uganda_treated"
res_df_taxa[["method"]] <- "ancom2"
alpha = 0.05
taxa_sig <- dplyr::filter(res_df_taxa, q_hiv_phenotype < 0.05)
sigtab_dataset_uganda_treated <- taxa_sig
write.csv(sigtab_dataset_uganda_treated, "ANCOM_UGANDA_NEG_ART_Pathway_Filtered.csv")

##US Neg - UNSUP
#Select samples of interest and update phyloseq object
metadata<-as.data.frame(sample_data(pathways_phyloseq))
metadata<-metadata[metadata$sample_cohort == "boston", , drop = F]
metadata<-metadata[metadata$hiv_phenotype %in% c("1_hiv_negative", "4_unsuppressed"), , drop = F]
metadata<-as.data.frame(as.matrix(metadata[metadata$sexual_orientation != "MSM", , drop = F]))

pathways_phyloseq_test <- pathways_phyloseq_group
sample_data(pathways_phyloseq_test) <- metadata

#Run ANCOM
pathways_phyloseq_test_prune <- phyloseq::prune_taxa(taxa_sums(pathways_phyloseq_test) > 0, pathways_phyloseq_test)
out <- ANCOMBC::ancombc2(data = pathways_phyloseq_test_prune, assay_name = "counts", tax_level = NULL, fix_formula = "hiv_phenotype", 
              p_adj_method = "BH", pseudo = 0, pseudo_sens = FALSE, prv_cut = 0.05, lib_cut = 1000, 
              group = "hiv_phenotype", struc_zero = TRUE, neg_lb = FALSE, alpha = 0.05, n_cl = 6, global = TRUE,
              em_control = list(tol = 1e-05, max_iter = 100), mdfdr_control = list(fwer_ctrl_method = "holm", B = 100))
```

```
## `tax_level` is not speficified 
## No agglomeration will be performed
## Otherwise, please speficy `tax_level` by one of the following:
```

```
## Warning: The group variable has < 3 categories 
## The multi-group comparisons (global/pairwise/dunnet/trend) will be deactivated
```

```
res_df <- out$res
res_df <- dplyr::rename(res_df, rowname = taxon)
colnames(res_df) <- stringr::str_replace(colnames(res_df), "hiv_phenotype.+", "hiv_phenotype")
res_df_taxa <- dplyr::left_join(res_df, tibble::rownames_to_column(as.data.frame(phyloseq::tax_table(pathways_phyloseq_test_prune))), by = "rowname")
res_df_taxa[["index_num"]] <- 1:nrow(res_df_taxa)
res_df_taxa[["cohort"]] <- "boston_untreated"
res_df_taxa[["method"]] <- "ancom2"
alpha = 0.05
taxa_sig <- dplyr::filter(res_df_taxa, q_hiv_phenotype < 0.05)
sigtab_dataset_us_untreated <- taxa_sig
write.csv(sigtab_dataset_us_untreated, "ANCOM_US_NEG_UNSUP_Pathway_Filtered.csv")


##BOTSWANA NEG-UNSUP
#Select samples of interest and update phyloseq object
metadata<-as.data.frame(sample_data(pathways_phyloseq))
metadata<-metadata[metadata$sample_cohort == "botswana", , drop = F]
metadata<-metadata[metadata$hiv_phenotype %in% c("1_hiv_negative", "4_unsuppressed"), , drop = F]
metadata<-as.data.frame(as.matrix(metadata[metadata$sexual_orientation != "MSM", , drop = F]))

pathways_phyloseq_test <- pathways_phyloseq_group
sample_data(pathways_phyloseq_test) <- metadata

#Run ANCOM
pathways_phyloseq_test_prune <- phyloseq::prune_taxa(taxa_sums(pathways_phyloseq_test) > 0, pathways_phyloseq_test)
out <- ANCOMBC::ancombc2(data = pathways_phyloseq_test_prune, assay_name = "counts", tax_level = NULL, fix_formula = "hiv_phenotype", 
              p_adj_method = "BH", pseudo = 0, pseudo_sens = FALSE, prv_cut = 0.05, lib_cut = 1000, 
              group = "hiv_phenotype", struc_zero = TRUE, neg_lb = FALSE, alpha = 0.05, n_cl = 6, global = TRUE,
              em_control = list(tol = 1e-05, max_iter = 100), mdfdr_control = list(fwer_ctrl_method = "holm", B = 100))
```

```
## `tax_level` is not speficified 
## No agglomeration will be performed
## Otherwise, please speficy `tax_level` by one of the following:
```

```
## Warning: The group variable has < 3 categories 
## The multi-group comparisons (global/pairwise/dunnet/trend) will be deactivated
```

```
res_df <- out$res
res_df <- dplyr::rename(res_df, rowname = taxon)
colnames(res_df) <- stringr::str_replace(colnames(res_df), "hiv_phenotype.+", "hiv_phenotype")
res_df_taxa <- dplyr::left_join(res_df, tibble::rownames_to_column(as.data.frame(phyloseq::tax_table(pathways_phyloseq_test_prune))), by = "rowname")
res_df_taxa[["index_num"]] <- 1:nrow(res_df_taxa)
res_df_taxa[["cohort"]] <- "botswana_untreated"
res_df_taxa[["method"]] <- "ancom2"
alpha = 0.05
taxa_sig <- dplyr::filter(res_df_taxa, q_hiv_phenotype < 0.05)
sigtab_dataset_botswana_untreated <- taxa_sig
write.csv(sigtab_dataset_botswana_untreated, "ANCOM_BOTS_NEG_UNSUP_Pathway_Filtered.csv")

# Compile and order data for plot
sigtab_dataset <- as.data.frame(rbind(sigtab_dataset_us_treated, sigtab_dataset_botswana_treated, sigtab_dataset_uganda_treated, sigtab_dataset_us_untreated, sigtab_dataset_botswana_untreated))
sigtab_dataset_plot <- sigtab_dataset
sigtab_dataset_plot <- tidyr::unite(sigtab_dataset_plot, col =  "rowname_cohort", rowname, cohort, remove = FALSE)
sigtab_dataset_plot$cohort <- forcats::fct_relevel(as.factor(sigtab_dataset_plot$cohort), "boston_treated", "botswana_treated", "uganda_treated")
sigtab_dataset_plot <- dplyr::arrange(sigtab_dataset_plot, desc(cohort), lfc_hiv_phenotype)
sigtab_dataset_plot$rowname_cohort <- factor(sigtab_dataset_plot$rowname_cohort, levels = c(sigtab_dataset_plot$rowname_cohort))

# Make lookup table for cat5_pathway_label_lookup
cat5_pathway_label_lookup <- dplyr::distinct(sigtab_dataset_plot, rowname_cohort, .keep_all = TRUE)$Category5
names(cat5_pathway_label_lookup) <- dplyr::distinct(sigtab_dataset_plot, rowname_cohort, .keep_all = TRUE)$rowname_cohort

# Create plot color dictionary
Cat3_dictionary<-read.csv("Supplemental_Figure_7/Category3_Dictionary.csv", sep=";")
Cat5_dic <- Cat3_dictionary[Cat3_dictionary$Pathway %in% sigtab_dataset_plot$Category3, , drop = F]
row.names(Cat5_dic) <- Cat5_dic$Pathway
Cat5_dic <- Cat5_dic[unique(as.character(sigtab_dataset_plot$Category3)), , drop = F]
sigtab_dataset_plot$Category3 <- factor(sigtab_dataset_plot$Category3, levels = unique(sigtab_dataset_plot$Category3))
Cat5_dic$Color <- factor(Cat5_dic$Color, levels = Cat5_dic$Color)

# Build plot
cat5_total_plot <- ggplot(data = sigtab_dataset_plot, aes(x = rowname_cohort, y = lfc_hiv_phenotype)) +
         geom_bar(stat = "identity", aes(fill = Category3)) +
         scale_fill_manual(values = as.character(Cat5_dic$Color)) +
         coord_flip() + scale_x_discrete(label = as_labeller(cat5_pathway_label_lookup)) + theme_bw() +
         theme(legend.position = "none", axis.text.x = element_text(size = 16), axis.text.y = element_text(size = 8, face = "plain"), axis.ticks.y = element_blank()) + 
         geom_hline(yintercept = 0) + ylab("log2FoldChange") + ggtitle("Total NEG-ART")

ggsave("SupplementaryFigure7ABC_v3.pdf", cat5_total_plot, width = 15, height = 10)

##################
```
